# Untargeted Multimodal Metabolomics Investigation of the *Haemonchus contortus* Exsheathment Secretome

**DOI:** 10.3390/cells11162525

**Published:** 2022-08-15

**Authors:** Nikola Palevich, Paul H. Maclean, Paul M. Candy, Wendy Taylor, Ivona Mladineo, Mingshu Cao

**Affiliations:** 1 AgResearch Ltd., Grasslands Research Centre, Palmerston North 4442, New Zealand; 2 Laboratory of Functional Helminthology, Institute of Parasitology, Biology Centre of Czech Academy of Science, 37005 Ceske Budejovice, Czech Republic

**Keywords:** *Haemonchus contortus*, metabolomics, lipidomics, helminth, parasite, exsheathment

## Abstract

In nematodes that invade the gastro-intestinal tract of the ruminant, the process of larval exsheathment marks the transition from the free-living to the parasitic stages of these parasites. To investigate the secretome associated with larval exsheathment, a closed in vitro system that effectively reproduces the two basic components of an anaerobic rumen environment (CO_2_ and 39 °C) was developed to trigger exsheathment in one of the most pathogenic and model gastrointestinal parasitic nematodes, *Haemonchus contortus* (barber‘s pole worm). This study reports the use of multimodal untargeted metabolomics and lipidomics methodologies to identify the metabolic signatures and compounds secreted during in vitro larval exsheathment in the *H. contortus* infective third-stage larva (iL3). A combination of statistical and chemoinformatic analyses using three analytical platforms revealed a panel of metabolites detected post exsheathment and associated with amino acids, purines, as well as select organic compounds. The major lipid classes identified by the non-targeted lipidomics method applied were lysophosphatidylglycerols, diglycerides, fatty acyls, glycerophospholipids, and a triglyceride. The identified metabolites may serve as metabolic signatures to improve tractability of parasitic nematodes for characterizing small molecule host–parasite interactions related to pathogenesis, vaccine and drug design, as well as the discovery of metabolic biomarkers.

## 1. Introduction

Parasitic nematodes cause numerous diseases with major health consequences for both humans and animals [1,2]. From an agricultural perspective, *Haemonchus contortus* (barber’s pole worm), is one of the most economically important pathogenic nematodes infecting small ruminants, such as sheep and goats, representing a global animal health issue through the drastic losses in livestock [3]. These blood-feeding strongylid nematodes are orally transmitted via contaminated pasture to the host where they penetrate the fourth stomach (abomasum) mucosa, causing anemia and associated complications, often leading to death [4]. Although many of the worm species can be managed using existing prophylactic drugs (anthelmintics), the research in this space is important owing to the remarkable natural tendency of roundworms to develop resistance induced by the excessive use and consequent diminishing efficacy demonstrated for both old and new chemicals, threatening the global livestock industry [5,6,7].

**Figure 1:**
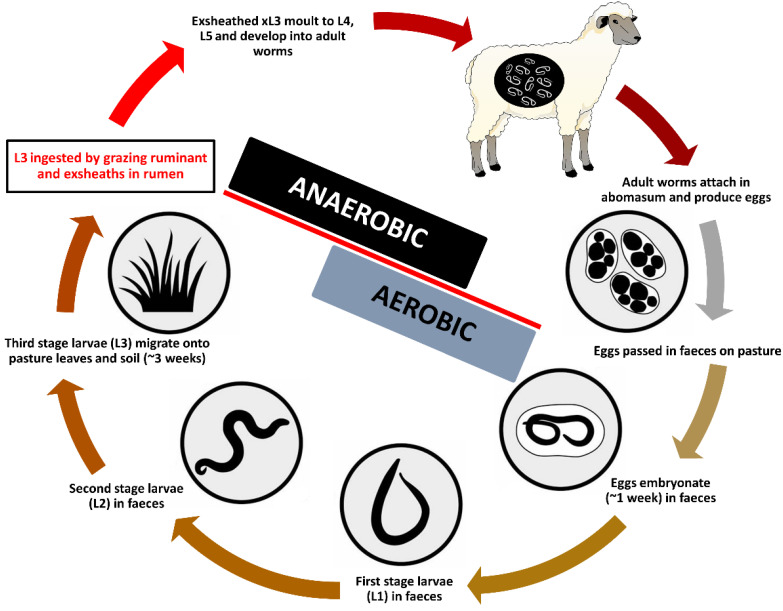
Developmental life cycle of the parasitic nematode *Haemonchus contortus*.

In the nematodes that invade the gastro-intestinal tract of the ruminant, ecdysis (εκδυο, ekduo, “to take or strip off”), is a key developmental process involving the molting of the cuticle (Figure 1). In *H. contortus* and the related nematodes, the post-embryonic stages of the life cycle are marked by four sequential molts, where larval exsheathment does not take place until the infective third-stage larva (L3) enters the rumen [8,9,10,11]. This sheath provides protection from various external (or environmental) stressors, such as low temperatures and desiccation. The L3s emerge from within their sheath (xL3) once they are exposed to the drastic rumen conditions, namely an increase in temperature (39 °C), a strictly anaerobic environment of predominantly carbon dioxide (CO_2_), and the influence of a pH change [12,13,14,15,16,17,18,19]. The process of larval exsheathment is thus of great interest, because it marks the sensitive phase of transition from the free-living to the parasitic stages of these parasites.

Metabolomics is an increasingly recognized research area to semi-quantitatively measure the small molecular metabolites in biological samples, using high-throughput approaches [20,21]. Lipidomics involves the profiling of the lipids within biological samples that are essential for the synthesis of steroid hormones, the molecules involved in the transmission of membrane signals, and for the transport of vitamins [22,23]. While significant ground has been gained in various ‘omics’ fields to facilitate our molecular-level understanding using the free-living model nematode *Caenorhabditis elegans* [24,25,26,27,28], much less has been achieved for the parasitic nematodes of veterinary significance [29,30]. Moreover, although *H. contortus* is currently regarded as a near-model organism, with notable landmark metabolomics studies investigating its various life-cycle stages [31,32,33], further molecular investigations into the key developmental process of larval exsheathment are warranted.

In this study, we present a closed in vitro parasite culture system that effectively mimics the rumen conditions to stimulate exsheathment without chemical interventions that is suitable for all of the currently available molecular and functional experimentation applications (such as genomics, transcriptomics, proteomics, glycomics, lipidomics, and metabolomics), as well as microbiome investigations. Our application of non-targeted multimodal metabolome and lipidome approaches to identify secreted compounds with varying chemical properties (e.g., polarity, hydrophobicity, etc.), is the first step in the multiomic investigation of the distinct secondary metabolites, pheromones, hormones, signaling pathways, and post-transcriptional/post-translational regulations associated with the larval exsheathment in *H. contortus*.

## 2. Materials and Methods

### 2.1. Production and Procurement of H. contortus

All of the experimental procedures used in generating the parasite material for this study were approved by the AgResearch’s Grasslands Animal Ethics Committee under the Animal Welfare Act 1999 in New Zealand (AEC application number 13928). The pure cultures of *H. contortus* third-stage larvae (L3) were maintained by the regular passage through five otherwise parasite-free lambs housed indoors at the AgResearch’s Grasslands campus. The L3 were cultured in fresh fecal material containing eggs collected into fecal bags on infected sheep. The feces were pooled and mixed with vermiculite, then placed in trays, moistened with tap water (at 20 °C), covered and cultured for 10 days at 22–24 °C. A modified Baermann technique [34] was used to clean and separate the larvae from the feces. Briefly, approximately 150 g of feces was enclosed in paper facial tissues and suspended over a large conical measuring flask filled with unchlorinated water. The samples were incubated for 20 h and the larvae washed by their movement through the apparatus. The feces were then removed and the liquid carefully siphoned to a remaining volume of about 20 mL. The resulting L3s and the volume were examined in a counting chamber (Whitlock S.F.E.L.O., Australia; volume 2 mL) at ×100 magnification and stored in tap water at 10 °C until required. A single batch of larvae were used for all of the experiments to account for any batch-related confounding factors with larval viability, and the motility was checked microscopically prior to any further experimentation.

The specificity of the genomic DNA was verified by automated Sanger sequencing of the second internal transcribed spacer (ITS-2) of the nuclear ribosomal DNA, following PCR amplification from genomic DNA [35]. A 100% similarity identity was achieved corresponding to the *H. contortus* NZ_Hco_NP (genome version 1.0, BioProject accession number PRJNA517503), the representative chromosome-level genome of the anthelmintic-susceptible *H. contortus* field strain, isolated from pasture-grazed New Zealand sheep [36,37,38].

### 2.2. In Vitro Larval Exsheathment Culture Assay

Prior to in vitro testing, the pooled L3 cultures were cleaned using autoclaved phosphate-buffered saline (1× PBS) solution (137 mM NaCl, 2.7 mM KCl, 8 mM Na_2_HPO_4_, and 2 mM KH_2_PO_4_, pH 7.4) and acclimated overnight to room temperature by gravity migration filtration through nylon mesh (pore size 20 μm). The larval viability and motility were checked microscopically and quantified using a Petroff–Hausser chamber (Hausser Scientific, Horsham, PA, USA), according to the manufacturer’s instructions.

**Figure 2:**
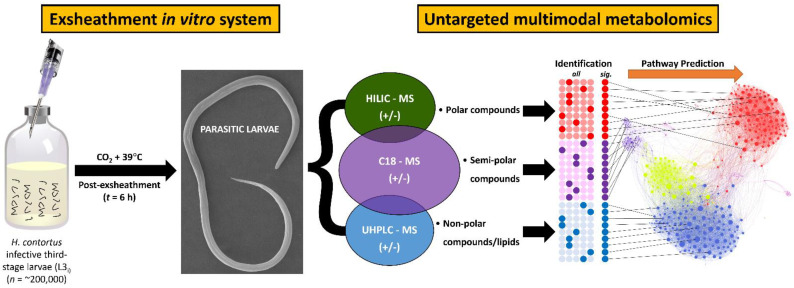
Overview of the experimental procedure and multimodal metabolomics workflow. Schematic diagram of the closed in vitro system that effectively reproduces the two basic components of an anaerobic rumen environment (CO_2_ and 39 °C) was used to trigger exsheathment (xL3) in *H. contortus* third-stage infective larvae (iL3) in O_2_-free CO_2_ saturated saline solution (**left**). Multi-modal metabolomics workflow and statistical analyses used to process data integrated from multiple analytical approaches through to pathway mapping (**right**).

In this study, we report a ‘closed’ and high-throughput system designed to simulate the physiological conditions of the rumen (39 °C and high CO_2_ concentration) for the purposes of culturing rumen microorganisms [39,40,41,42,43]. This closed system was modified from previously described work [9,10,19], to be used for in vitro larval exsheathment assays that are suitable for future parasite–host–microbiome interactions and multiomic studies (Figure 2). Briefly, the anaerobic 1× PBS solution was mixed in boiling dH_2_O and cooled to room temperature under a continuous flow of O_2_-free CO_2_. Once cooled, the PBS solution was transferred to 100 mL serum bottles in 70 mL aliquots and flushed with CO_2_ for 1 h. The bottles were sealed with butyl rubber bungs and aluminum crimp caps before being autoclaved at 121 °C for 20 min.

The larval cultures (*n* = 5 biological replicates) were transferred anaerobically (approximately 200,000 L3/sample), using an O_2_-free CO_2_-flushed 3 mL syringe and a wide-bore hypodermic needle (16 G thickness and length of 1–1/2 inch, BD), into aluminum-wrapped and pre-warmed serum bottles containing 70 mL of autoclaved PBS, and incubated anaerobically at 39 °C with gentle horizontal shaking at 75 rpm for up to 24 h in darkness. As control treatments, distilled water (dH_2_O) was used instead of 1× PBS (*n* = 5), and also in the absence of rumen anaerobic conditions. The exsheathment of the L3 larvae (xL3) was determined by either complete or partial loss of the sheath and was measured by sub-sampling each replicate beginning at *t* = 0 min, then at 5 min intervals up to 2 h, with another seven time points (*t* = 2.5, 3, 4, 5, 6, 12, and 24 h) after incubation at 39 °C and CO_2_ anaerobic conditions. At each time point, the contents were thoroughly mixed before 1 mL was transferred using an O_2_-free CO_2_-flushed 1 mL syringe and wide-bore hypodermic needle to a 24-well plate and exsheathment enumerated. The numbers of xL3s in each subsample was quantified via 200-fold dilution of sample to another 24-well plate containing dH_2_O to yield approximately 200 larvae per replicate. The larvae were killed by the addition of one drop of 3% helminthological iodine solution (Lugol’s solution) and the exsheathment enumerated.

The Student’s *t*-test was applied to investigate the larval exsheathment, resulting in a significant difference between PBS and the dH_2_O (*p* < 0.05) negative control samples. The samples were collected from the *t* = 6 h time point, snap-frozen in liquid nitrogen, transferred to glass vials, and stored at −80 °C until further use for subsequent metabolomics analysis.

### 2.3. Electron Microscopy of H. contortus Larval Exsheathment

The scanning electron microscopy (SEM) imaging was performed (Figure 2), using methodology as previously described [36]. Briefly, the cryopreserved worms were gently spun, washed three times in PBS, and fixed in SEM primary fixative (3% glutaraldehyde, 2% formaldehyde in 0.1 M Phosphate Buffer pH 7.2) for 2 days at room temperature. The samples were dehydrated in a graded ethanol series, that is, 25%, 50%, 75%, and 95% for 10–15 min each and two times in 100% ethanol for 1 h, then Critical Point (CP)-dried using liquid CO_2_ and mounted onto an aluminum specimen support stub using double-sided adhesive tape. The samples were sputter-coated with gold (200 sec) and observed using a FEI Quanta 200 Environmental SEM microscope, with an energy dispersive X-ray spectroscopy (EDAX) module. The electron microscopy was conducted with the assistance of the Manawatū Microscopy and Imaging Centre at Massey University (Palmerston North, New Zealand).

### 2.4. Metabolomics Analysis

#### 2.4.1. Metabolomics Conditions and Analytical Procedures

A combination of multiple metabolomics platforms, or ‘multi-modal’ strategy, was applied in parallel to the same batch of biological samples to facilitate interpretation and provide extensive coverage of the parasite metabolome (Figure 2). To elucidate the metabolites associated with the larval exsheathment we applied: hydrophilic interaction liquid chromatography (HILIC) to separate the polar compounds; ultra-high-performance liquid chromatography (UHPLC) with C18 chromatography to separate the semi-polar compounds; and CSH C18 chromatography to separate the lipids [44,45]. From here on, HILIC, C18, and LIPID terms are used to refer to the above-mentioned metabolomics approaches. The LC–MS studies were conducted in both positive and negative electrospray ionization (ESI) modes.

To evaluate the enriched lipids and metabolites secreted by *H. contortus* xL3, seven aliquots of 1 mL each (five for the analyses and two for quality control) of each sample were transferred into the microcentrifuge tubes. The two QC samples were pooled and solely used for monitoring sample degradation, tracking run-order effects within a batch, and quality control purposes. Briefly, the samples were thawed overnight at 4 °C, centrifuged (4 °C, 11,000× *g*) for 10 min, and 200 µL of supernatant transferred into a 2 mL micro-centrifuge tube. An extraction solvent comprising 800 µL of chloroform: methanol (1:1; *v*/*v*) was added and the samples were vortexed (1 min). The sample was diluted with water (400 µL), again vortexed (1 min), and centrifuged (4 °C, 11,000× *g*) for 15 min.

For the lipid compounds, the lower organic layer (200 µL) was taken separately, evaporated to dryness under a continuous stream of nitrogen (30 °C), and the dried extract was reconstituted in 200 µL of chloroform: methanol (2:1; *v*/*v*), with 16:0 d31–18:1 phosphatidylethanolamine (10 µg/mL) as an internal standard. Finally, samples were vortexed (1 min), and 100 µL was transferred to a glass insert in an auto-sampler vial for the LC–MS analysis. For the polar and semi-polar compounds HILIC and C18 chromatography were applied, respectively (46–49). For these analyses, supernatants (200 µL) were mixed with 800 µL of pre-chilled chloroform: methanol (1:1, v/v) containing 1.6 mg/L of internal standards; d5-L-tryptophan, d4-citric acid, d10-leucine, d2-tyrosine, d35-stearic acid, d5-benzoic acid, 13C2-glucose, and d7-alanine. The upper aqueous layers (200 µL) were taken and evaporated as above, then reconstituted in 200 µL of the extraction solvents comprised of acetonitrile: water, containing 0.1% formic acid (1:1 for HILIC and 1:9 for C18, *v*/*v*).

#### 2.4.2. Chromatography and Mass Spectrometry Spectral Acquisition

The chromatographic gradient and the other conditions were selected to detect metabolites over a wide polarity range for the non-targeted LC–MS and lipid analyses, as previously described [44,46]. For the semi-polar compounds, the C18 conditions were set as described; the extract (2 µL) was injected into a 100 mm × 2.1 mm Thermo Hypersil Gold C18 column with 1.9 µm particle size and eluted over a 16 min gradient with a flow rate of 400 μL/min. The mobile phase was a mixture of water with 0.1% formic acid (solvent A), and acetonitrile with 0.1% formic acid (solvent B). For the polar compounds, the extract (2 µL) was injected into a 100 mm × 2.1 mm ZIC-pHILIC column with 5 µm particle size and eluted over 17 min with a solvent gradient from 97% solvent A (1 min), 97–70% solvent A (1–12 min), 70–10% solvent A (12–14.5 min), to 10% solvent A (14.5–17 min). The mobile phase solvent A was a mixture of acetonitrile with 0.1% formic acid, solvent B was a mixture of water with 16 mM ammonium formate, and the flowrate was 250 µL/min. The chromatographic gradient and other LC–MS conditions were previously described [45,46].

The C18 and HILIC column effluents were connected to a high-resolution mass spectrometer, Exactive Orbitrap™ (ThermoFisher Scientific, Waltham, MA, USA) with electrospray ionization technology, and the lipid analysis was conducted on a Q-Exactive mass spectrometer (ThermoFisher Scientific, Waltham, MA, USA). Both of the full and data-dependent MS^2^ (ddMS^2^) scans were collected in profile data acquisition mode. For the full scan mode, a mass resolution setting of 35,000 was set to record a mass range of *m/z* 200–2000 with a maximum trap fill time of 250 ms. In ddMS^2^, the MS^2^ measurements were activated when a set peak intensity threshold was achieved. For the ddMS^2^ scan mode, the same mass resolution setting was maintained, with a maximum trap fill time of 120 ms. The isolation window of the selected MS^1^ scans was ± 1.5 *m/z* with a normalized collision energy of 30. The samples were run separately in both positive and negative ionization modes. The positive ion mode parameters were as follows: spray voltage, 4.0 kV; capillary temperature, 275 °C; capillary voltage, 90 V; tube lens 120 V. The negative ion mode parameters were as follows: spray voltage, −2.5 kV; capillary temperature, 275 °C; capillary voltage, −90 V; tube lens, -100 V. The nitrogen source gas desolvation settings were the same for all of the modes (arbitrary units): sheath gas, 40; auxiliary gas, 10; sweep gas, 5. The Xcalibur software package provided by the manufacturer was used to create these settings.

The QC comprised a pooled aliquot of the extract of all of the samples. The pooled samples with internal standards were used as the controls and the samples were randomized prior to injection to allow for the investigation of any systematic variations. Blank subtraction was applied after the internal standard correction. To verify and maintain the data quality, the QC sample was injected once every 10 samples. The retention time, signal intensity, and mass error of the internal standard were constantly monitored during the runs. The fragmentation data on approximately four samples in total per ionization mode (positive and negative) were used for the identification of metabolite ions/classes. The amino acid standards (Sigma-Aldrich, St. Louis, MO, USA, A9906) were spiked with the samples within the same analytical run in both C18 and HILIC.

#### 2.4.3. Data Processing, Peak Detection and Statistical Analysis

The MS raw data files (Thermo.raw files) were converted to mzXML files using the MSConvert function of ProteoWizard™ [47]. Peak detection, retention time alignment, grouping, and gap filling [48] were implemented, based on tools from the *xcms* R package [49]. Briefly, the chromatographic peak detection was based on the ‘centWave’ method with parameters of ppm = 15, peakwidth = c(3:15), mzdiff = 0.02, and snthresh = 10. The retention time alignment was based on the ‘orbitrap’ method with the default parameters. The peaks were grouped using ‘group.density’ with bw = two and mzwid = 0.02. The missing peaks were filled by the “chrom” method, which integrates signals in the same region where the peaks were detected from the other samples. The peak tables were then exported for statistical evaluation, where the weak peaks (with mean intensity <1000) were removed and the ^13^C isotopic peaks were filtered out [50]. The broad peaks were investigated and removed with in-house scripts. The same procedures of peak detection and analysis were employed for all of HILIC, C18 and LIPID after the peak shape of some of the known metabolites were monitored.

#### 2.4.4. Metabolite Annotation and Identification

The massive peak features can be collected from LCMS-based metabolomics. The metabolite annotation, based on the peaks features, was a challenging task. Typical practices involve identifying the significant peak features and focusing on the top-ranked peaks with the biological relevance under study. Further independent evidence is often required to elucidate the structures of those unknowns. Here, we are interested in what could be annotated in the exsheathment secretome. We carried out MSI (Metabolomics Standards Initiative) level 2 annotation, supported by orthogonal evidence from both the accurate mass and retention time. The accurate mass must be with 5 ppm accuracy when searching for candidates from the databases, which include HMDB, LIPID MAPS, and WormBase. The presence of the adduct ions depends on the experimental conditions, such as the solvents. We have included [M+H]^+^, [M+Na]^+^, [M+K]^+^, and [M+NH_4_]^+^ for the positive ions and [M−H]^−^, [M+FA−H]^−^, and [M+Cl]^−^ for the negative ions. The isotopic distribution of the potential molecular formula was investigated to check the adduct identity. The match of the retention time (RT) of the identified peaks was based on the external standards and in-house RT library [51]. The QSRR (Quantitative Structure-Retention Relationship) model [51] was also deployed here, to predict the RT of the candidates from HILIC. The validity of this practice is supported by the RTs’ common 21 metabolites from the external standards (this study), and in the recently published work [51] is shown to be linearly correlated (R-squared = 0.994, *p*-value < 2.2 × 10^−16^). However, we have not established QSRR models for the CSH chromatography. The RT information used for annotating the lipids was based on our knowledge and published resources (https://www.waters.com/webassets/cms/library/docs/720004107en.pdf, accessed on 20 April 2022) for the similar chromatographic conditions. Furthermore, the fragmentation spectra (MS^2^) were collected and analyzed [52] to support the lipid annotation.

The metabolic pathway coverage and enzymes (ECs) in a reference pathway were annotated by manual investigation of the metabolomic datasets, using the KEGG (Kyoto Encyclopedia of Genes and Genomes) metabolic pathways database. The MS data (in mzML) and metadata reported in this study were submitted to the MetaboLights database with the study identifier: MTBLS1717.

## 3. Results

### 3.1. In Vitro Culture Technique Effectively Induces Third-Stage Larval Exsheathment in H. contortus

**Figure 3:**
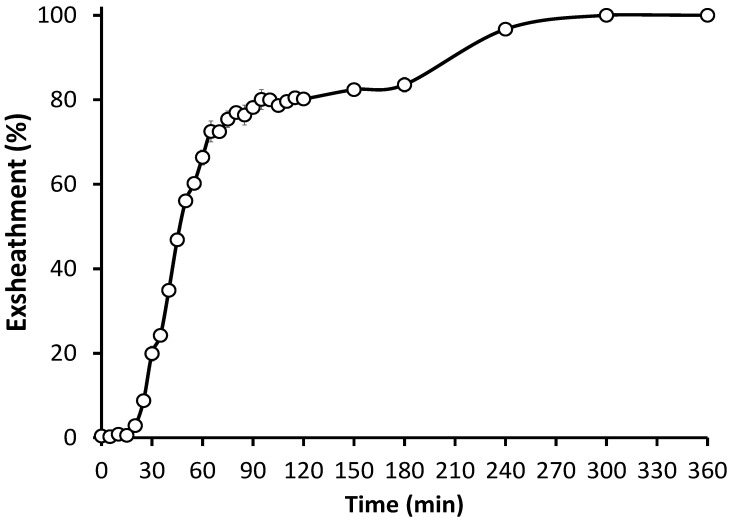
*H. contortus* infective larvae during in vitro exsheathment. Time series analysis of the exsheathment activity of *H. contortus* L3 using the anaerobic in vitro system. Mean of the total exsheathment percentage (±SEM) at each time point across replicates (*n* = 5). Significant (*p* < 0.001) exsheathment was obtained resulting in 75% and 100% of larvae exsheathing after 70 min and 5 h, respectively, post trigger exposure. No exsheathment activity was observed in the absence of anaerobic treatment conditions. Exsheathment up to 6 h post trigger application shown.

Our results showed that a high and reproducible level of larval exsheathment was readily achieved in *H. contortus* (Figure 3). The efficacy of the larval exsheathment was determined by measuring the percentage of larvae that exsheath at 39 °C and in an anaerobic environment (CO_2_), and then in the absence of rumen conditions on freshly cultivated *H. contortus* L3s. At 5 h post incubation, 100% exsheathment was achieved in the *H. contortus* third-stage larvae (L3), with no exsheathment observed in the distilled water negative control or in the absence of rumen conditions. Overall, an incubation period of only 25 min was sufficient to trigger the exsheathment cascade, with a total of 75% exsheathment achieved at the 70 min time point.

**Figure 4:**
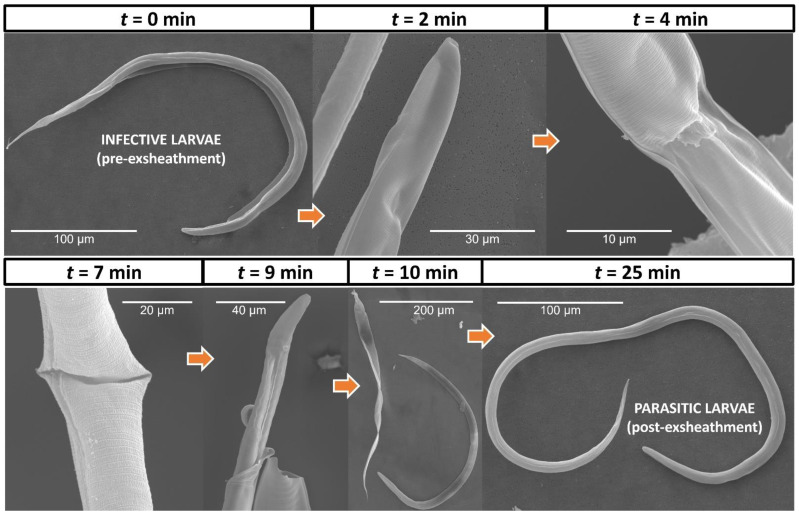
Scanning electron micrographs of the exsheathment process in *H. contortus*. SEMs are shown for seven time points post incubation (*t* = 0, 2, 4, 7, 9, 10 and 25 min). Each image depicts the earliest observations of key morphological changes that occur during the exsheathment process and is representative of the larval population for each biological sample. Scale bars differ and have been adjusted according to magnification of each image.

The SEM imaging was able to capture the physiological changes and provide a detailed insight into the anatomy of the *H. contortus* third-stage larvae that occurs during exsheathment, a crucial step determining infection as the larvae transition from infective to parasitic developmental stages. The first indication of ecdysis and initiation of cuticle digestion occurred after only 2 min incubation, and was characterized by an indentation at the anterior termination of the lateral alae (Figure 4). The cuticle region both anterior and posterior to this point swelled, and then depressed to form the refractile ring region. Along the circumference of the refractile ring region, the indentation gradually deepened until holes began to appear in the cuticle, and the release of the exsheathment fluid could first be observed at the 4 min time point. The cuticle hole increased in size to form a continuous separation along the annulus (*t* = 7 min), and at the 9 min time point the first examples of the cuticular cap being forced off by the head of the L3 was observed. As the L3 escaped through the opening, the posterior portion of the cuticle generally separated along the lateral alae, leaving the cap attached to the remainder of the cuticle. Notably and in agreement with early microscopic descriptions of the ecdysis process in other species of parasitic nematodes, no pores or other openings were visible on the body of the exsheathed xL3 in the region where the refractile ring had initially formed. In prospective, our results call for further investigation of the application of our in vitro system to activate a succession of events that begin with triggering exsheathment and the subsequent development to the fourth stage in additional species of parasitic nematodes infecting a variety of hosts.

### 3.2. Identification of Exsheathment Associated Metabolites

We applied a ‘multi-modal’ strategy to elucidate the metabolites associated with larval exsheathment by investigating the enriched lipids and metabolites that were secreted in the dense populations of 100% exsheathed *H. contortus* xL3 (i.e., 6 h post incubation). The normalized peak tables of metabolites are described in Table S1 (Supplementary Materials). The peak names were denoted with (HP/HN, CP/CN, and LP/LN), where ‘H’, ‘C’, ‘L’ represents peaks derived from HILIC, C18, and LIPID platforms, respectively, and ‘P’ and ‘N’ for positive (HP, CP, LP) and negative (HN, CN, LN) ionization mode peaks, respectively.

To tease out the natural metabolites that constitute the exsheathment fluid secreted by the larvae upon ecdysis (i.e., iL3 to xL3) of *H. contortus*, the fragmentation data were further mined, with a total of 26 compounds identified (Table 1). Overall, the C18 and HILIC top peaks were classed as amino acids (*n* = 10), purines (*n* = 2) and organic compounds (*n* = 2). Among these were a panel of metabolites, including methionine, leucine, alanine, phenylalanine, xanthine, and hypoxanthine, that were significant in two separate metabolomics streams and associated with the amino acids and purine metabolism pathways. Of interest, the lysine (C_6_H_14_N_2_O_2_) was annotated with both the positive and negative HILIC ionization modes from our in-house database.

For the LIPID peaks, we also investigated the available MS^2^ spectra collected for a few precursor *m/z*, using the previously described method [51,52]. Overall, five distinct lipid classes were identified by the non-targeted lipidomics analysis, lysophosphatidylglycerols (LPGs; *n* = 2), diglycerides (DGs; *n* = 5), glycerophospholipids (GPs; *n* = 2), fatty acyls (FAs; *n* = 2), and the triglyceride TG(C16:0/C18:0/C18:0) were identified.

## 4. Discussion

The early work exploring the effects of the fundamental anaerobic (CO_2_) and temperature (39 °C) rumen conditions on the exsheathment of a variety of nematode species [10,11,12,13,14], formed the conceptual basis for this work. The initial biological objective of this study was to validate and determine the efficacy on larval exsheathment using our in vitro exsheathment system that mimics the inoculation of the *H. contortus* L3 larvae into the rumen. To date, in order to obtain xL3s for numerous gastro-intestinal nematode (GIN) parasite species, the common laboratory practice is to use sodium hypochlorite as a desheathment agent [53,54,55]. The developed ‘closed’ in vitro exsheathment system presented in this work provides a straightforward, cost-effective, non-chemical, and efficient alternative method for larval exsheathment. As such, our system maintains a constant and measurable anaerobic environment that does not damage the larvae, enables subsampling without altering the in vitro conditions, and can be tailored to accommodate future ‘omics-oriented studies at high-throughput capacities [56]. Importantly, the natural conditions maintained in this closed system, in contrast to the hypochlorite-stimulated exsheathment, allow for more realistic data interpretation from the downstream ‘omics studies. This is of particular importance to the microbiome and RNA sequencing investigations, in which exposure to oxygen, as with traditional methods and especially with the use of sodium hypochlorite, can affect the transcriptional response in both the worm and its microbiome [57,58,59].

The metabolomic and comparative genomic analyses have underlined the importance of the metabolic networks in the understanding of parasites’ pathogenesis and their metabolic adjustments throughout the life cycle [60]. However, while the rapid developments of the ‘omics field have accelerated our knowledge of nematode biology at the molecular level, much fewer advancements have been achieved for the parasitic nematodes of veterinary importance, and particularly regarding metabolomics. In this study, a set of analytical techniques and methodologies was explored to carry out metabolomic profiling to investigate the larval exsheathment in *H. contortus*.

The discovery of novel or the identification of pathophysiological biomarkers that cause infective L3s to exsheath/molt and develop to become infective, provide much needed opportunities for the development of novel drug or vaccine targets for the mitigation of haemonchosis. To investigate the biological mechanisms at play and identify the potential nematode exsheathment bioactives, we applied a non-targeted multimodal metabolomics and lipidomics approaches. To be noted is that the samples encompassed metabolites that were secreted from the larval body in the *Haemonchus* milieu, but also those potentially carried over from the nematode microbiota as secondary metabolites at the six hours post exsheathment trigger application. The latter offers possibilities to investigate the intimate parasite–microbiome interaction [61,62,63]. It is also possible that the worms acquired the extracellular vesicles (EVs), serving for the delivery of numerous information signals between the parasites and their environment through the enveloped cargo consisting of proteins, glycans, lipids, metabolites, and nucleic acids [64]. Based on our stringent statistical analysis we identified a suite of small molecule-level metabolic changes related to the larval exsheathment in *H. contortus* (Table 1). In order to provide a context for the compounds identified, we investigated their inferred KEGG metabolic pathway annotations from our integrative analysis. For future work, a comprehensive time-series investigation of the exsheathment metabolome would be a valuable resource to differentiate the metabolites associated with the initiation of exsheathment.

In addition to confronting the physiochemical environmental conditions associated with migration into the rumen, such as temperature, pO_2_, pCO_2_, pH, osmotic pressure, and redox potential [65], the parasitic nematodes are able to extract most of their nutritional requirements from the environment to meet the energy requirements of exsheathment [66]. The changes in the amino acids in larval exsheathment were also observed and include methionine, leucine, alanine, and phenylalanine (Table 1). The complex and essential amino acid methionine, which animals cannot synthesize, and the branched-chain amino acids (BCAAs) leucine and isoleucine, were identified in both of the C18 and HILIC streams with high confidence. These amino acids were included in significantly changed KEGG pathways, such as aminoacyl-tRNA biosynthesis (cel00970), biosynthesis, and degradation of BCAAs (cel00290), as well as the cysteine and methionine metabolism (cel00270) in nematodes. The recent metabolomics studies have consistently revealed that BCAAs are positively related to longevity common to dauers in worms [67,68], and proposed to be intricately associated with the mitochondrial biogenesis during exsheathment. Alanine was also identified as being upregulated in the alanine, aspartate, and glutamate metabolism KEGG pathway (cel00260), that is, involved in the protein synthesis and the synthesis of other vital amino acids (glycine, serine, threonine, etc.), phospholipid, and collagen production, as well as in the release of energy, all of which have important roles in rapid cell proliferation. Therefore, the increased levels of particular BCAA profiles, may serve as the potential biomarker candidates of parasitism in the rumen, and possibly other tissue types. The phenylalanine, upregulated within the KEGG pathway Phenylalanine metabolism (cel00360) through tyrosine synthesis, serves for melanin production, which in *C. elegans* cuticle has been designated a protective role [69]. Furthermore, our study validates methionine as a rational candidate for the development of anti-parasitic drugs [70]. A recent study compared the metabolomes and lipidomes of the excretory and secretory products (ESPs) and the somatic extracts (SEs) of the infective L3 of GIN *Nippostrongylus brasiliensis* and *Trichuris* muris, respectively [71]. Some of the top expressed polar metabolites observed herein, such as adenine, xanthine, succinate, hypoxanthine, and methionine, were upregulated in *N. brasiliensis* ESPs, or downregulated (alanine and phenylalanine), implying their unrevealed importance in parasite–host crosstalk. After cataloguing the bioactive properties of these metabolites, it is striking that most of them mediate an anti-inflammatory environment, except xanthine, which strengthens our opinion that these metabolites have a far more complex act in immune modulation than for now meets the eye. For future work, the developed in vitro system should be used to determine the relative abundances of these metabolites produced during *H. contortus* larval exsheathment under basic rumen conditions, to explore their potential as a biomarkers of haemonchosis.

Regarding general energy metabolism, the quiescent *H. contortus* L3 seemingly depend on stored lipid reserves to survive the adverse conditions in the pastures before host infection [72,73], and are secreted in the not yet feeding *H. contortus* xL3 exposed to lack of oxygen in the rumen. Further transcriptomic evidence is required to investigate such hypotheses in the GIN nematodes and shed light on the possible role of alternative metabolic pathways, particularly with regards to dauers and over-winter survival strategies. In this study we applied the first-known untargeted lipidomic approach to describe the secreted lipids of non-chemically induced exsheathment of intact *H. contortus* xL3 larvae, an essential transition from aerobic free-living to anaerobic parasitic stages. A total of 12 lipid species across five classes were identified with high confidence that formed three types of metabolite groupings. In accordance with a recent report on the global lipidome of *H. contortus* [32], long chain (more than 12 carbons) saturated fatty acid levels are high in the transition from the free-living phase. The lipid species represented by GlyceroPhosphoCholine lipids (PCs) associated with the glycerophospholipid metabolism, including the phospholipid biosynthesis (cel00564) KEGG pathways as well as retinol or vitamin A metabolism (cel00830). All of these are related to choline metabolism, but also sarcosine can be generated by alternative pathways via creatine metabolism [74]. Interestingly, the creatine that was clearly detected using both C18 and HILIC streams (Table 1), can be phosphorylated and dephosphorylated by mitochondrial and cytosolic creatine kinase and creatinase to protect against the inhibition of mitochondrial respiration. Multiple copies of both of the creatine metabolism enzymes are present in the *H. contortus* NZ_Hco_NP (v1.0) and may convey an obvious advantage to the ingested L3 as they are able to utilize the host creatine as a source of nitrogen via sarcosine generation from creatine, and the breakdown of sarcosine by sarcosine oxidase to glycine that was also detected. As such, the nematode phosphagens and kinases may serve well for future development as anthelmintic targets and as potential vaccine candidates, due to their strong antigenicity in mammals.

Our detection of the branched fatty acids, palmitic acid (C16:0) and stearic acid (C18:0), are in agreement with the previous studies, particularly *C. elegans* [75,76], that report the adaptation of nematodes to increased levels of saturated fatty acids at higher temperatures, and in response to a change in their environmental temperature. In addition to their roles in helminths’ energy flows, lipid metabolites have been recognized as the key players in the regulation of innate immune responses for over 30 years, especially lysophosphatidylglycerols (palmitic and stearic acids) and fatty acyls, such as arachidonic acid (hydroxy-eicosenoic acid) and 2-oxo-docosanoic acid [77]. While some of them mediate the pro-inflammatory cascade initiating PAMPs (pathogen-associated molecular patterns) recognition via TRL4 (Toll-like receptor 4) [78] or serving as a precursor for prostaglandins and leukotrienes [79], such as palmitic and arachidonic acid, respectively, the others (stearic, α-linolenic acid) have immunosuppressive effects on T cells [80]. GINs’ strategy mediated through the lipid metabolites and employed for the manipulation of the host immune response towards successful propagation and parasitism, opens a wide perspective that should be compounded by the contemporary use of multi-omics approaches.

The robust detection of such metabolites, using our in vitro system coupled with the advent of sequencing technologies and availability of genomic data, warrants a comprehensive transcriptomic investigation to uncover the exsheathment molecular mechanisms. The integration of the metabolic data, identified under basic rumen in vitro conditions with the anthelmintic-susceptible New Zealand *H. contortus* NZ_Hco_NP field strain genome, enables future research to explore the nexus of gene–environment interactions associated with exsheathment to guide the development of precision intervention strategies (i.e., novel drug and vaccine targets) against major parasitic nematodes. A key unanswered question is whether our findings apply to other pathogenic nematodes of ruminants, with future efforts combining genomic and transcriptomic approaches needed to advance this issue. The research in this space is important, owing to the diminished efficacy demonstrated for both old and new anthelmintics in parasitic nematodes and their effect on livestock in NZ and around the world.

## 5. Conclusions

In this study, we validated our ‘closed’ in vitro exsheathment system that provides an effective alternative method for larval exsheathment suitable for many different future ‘omics-oriented applications. We established multivariate analysis models based on numerous statistical models for the identification of the robust metabolic signatures associated with larval exsheathment. In conclusion, this study reports the use of a comprehensive multimodal metabolomics approach, and provides a valuable resource for future research and data mining. The small sample size of our research could serve as a platform study for future functional studies in a systems biology context, investigating larval exsheathment and parasite development.

## Figures and Tables

**Table 1:** Summary of Compounds Identified Across All Metabolomics Streams.

Metabolite Class	Molecular Species	Platform	Isotopic Peak (*m/z*_rt)	Ion Type	Calc Mass	Rt * (min)	*C. elegans* KEGG Pathway ID
**C18 and HILIC**							
**Amino acids**	Lysine	HP	147.1128_15.79	[M+H]^+^	147.1128	15.8	Lysine biosynthesis (cel00300) and degradation (cel00310)
		HN	145.0982_15.78	[M−H]^−^	145.0972	15.8	
	Glycine	HP	76.0396_13.21	[M+H]^+^	76.0398	13.2	cel00260-Glycine, serine, and threonine metabolism
	Threonine	HP	120.0654_12.76	[M+H]^+^	120.0655	12.7	
	Methionine	HP	150.0583_10.57	[M+H]^+^	150.0583	10.5	cel00270-Cysteine and methionine metabolism
		CP	150.0595_1.10	[M+H]^+^	150.0583	1.08	
	Alanine	HP	90.0546_12.54	[M+H]^+^	90.0549	12.5	cel00250-Alanine, aspartate and glutamate metabolism
	Glutamic acid	HN	146.0465_13.2	[M−H]^−^	146.0448	13.3	
	Isoleucine	HP	132.1030_10.12	[M+H]^+^	132.1019	10.2	Valine, leucine, and isoleucine degradation (cel00280) and biosynthesis (cel00290)
		CP	132.1022_1.87	[M+H]^+^	132.1019	1.76	
	Leucine	HP	132.1025_9.84	[M+H]^+^	132.1019	9.8	
		CP	132.1027_1.73	[M+H]^+^	132.1019	1.75	
	Tryptophan	HP	205.0969_10.45	[M+H]^+^	205.0972	10.5	cel00380-Tryptophan metabolism
	Phenylalanine	HP	166.0877_9.80	[M+H]^+^	166.0863	9.8	cel00360-Phenylalanine metabolism
**Purines**	Xanthine	HN	151.0258_8.62	[M−H]^−^	151.0251	8.3 *	cel00230-Purine metabolism
		CP	153.0419_1.39	[M+H]^+^	153.0407		
	Hypoxanthine	HP	137.0470_8.24	[M+H]^+^	137.0458	7.9 *	
		HN	135.0309_8.24	[M−H]^−^	135.0301		
		CP	137.0473_1.2	[M+H]^+^	137.0458	1.2	
**Organic** **compounds**	Piperidine	HP	86.0963_9.84	[M+H]^+^	86.097	10.3 **	cel00310-Lysine degradation
	Carnitine	HP	162.1125_10.46	[M+H]^+^	162.1125	10.6 **	cel01212-Fatty acid metabolism
		CP	162.1131_0.67	[M+H]^+^	162.1125		
**LIPIDS**							
**LPG**	Palmitic acid (16:0)	LN	255.2327_3.5	[M−H]^−^	255.2319	[52]	cel00061-Fatty acid biosynthesis
	Stearic acid (18:0)	LN	283.2641_4.18	[M−H]^−^	283.2632		
**DG**	DG(32:0)	LN	603.4765_7.85	[M+Cl]^−^	603.475		cel00561-Glycerolipid metabolism
	DG(34:0)	LN	631.5075_8.56	[M+Cl]^−^	631.5063		
	DG(36:0)	LN	659.5388_9.23	[M+Cl]^−^	659.5376		
	DG(18:0/18:0) MS2	LP	642.6033_9.98	[M+NH_4_]^+^	642.6031		
	DG(16:0/18:0) MS2	LP	614.5719_8.55	[M+NH_4_]^+^	614.5718		
**GP**	PKHdiA-PS	LN	686.2696_6.46	[M+Cl]^−^	686.2703		cel00564-Glycerophospholipid metabolism
	OHHdiA-PS	LN	714.3014_7.23	[M+Cl]^−^	714.3016		
**FA**	hydroxy-eicosenoic acid	LP	344.3161_2.28	[M+NH_4_]^+^	344.3159		cel00590-Arachidonic acid metabolism
	2-oxo-docosanoic acid	LP	372.3473_2.88	[M+NH_4_]^+^	372.3472		cel01040-Biosynthesis of unsaturated fatty acids
TG *	TG(16:0/18:0/18:0) MS2	LP	880.8331_12.57	[M+NH_4_]^+^	880.8328		cel00561-Glycerolipid metabolism

Metabolomics analysis was performed on samples (approximately 200,000 L3/sample with *n* = 5 biological replicates) containing 100% exsheathed *H. contortus* xL3 at 6 h post incubation. Compounds were identified by matching with a local library of authentic standards, public domain mass spectral databases, and analytical stream. Abbreviations: Rt, retention time in minutes; LPG, lysophosphatidylglycerol; DG, diglyceride; FA, fatty acyls; GP, glycerophospholipids; TG, triglycerides. All metabolite identifications are classed with Level 2 confidence for lipids with 16:0, 18:0, etc., referring to fatty acids with their respective number of carbon atoms and double bonds. rt *, standards as previously described [52]; rt **, QSRR prediction.

## Data Availability

The MS data and associated metadata reported in this study have been submitted to the MetaboLights database with the study identifier: MTBLS1717. Alternatively, the datasets generated from this study are available on request from the corresponding author.

## Supplementary Materials

The following supporting information can be downloaded at: https://www.mdpi.com/article/10.3390/cells11162525/s1, Lists of metabolite peaks identified for the HILIC, C18, LIPID positive and negative ionization streams.
