# Supplementary material for: Untargeted Multimodal Metabolomics Investigation of the Haemonchus contortus Exsheathment Secretome
Source: Cells. 2022 Aug 15;11(16):2525. doi: 10.3390/cells11162525 (PMC9406637; doi:10.3390/cells11162525)
Supplement: Supplementary file 1 [file cells-11-02525-s001.zip › Supplementary Table S1 C18 +ve (CP).pdf]

Treatment	CP362.187	CP489.938	CP102.511	CP103.509	CP114.518	CP122.924	CP155.974	CP169.990	CP175.096
PBS	33632.77	60057.58	4476.29	7246.949	391.3864	26119.06	25990.06	9236.695	8561.192
PBS	32153.81	63248.81	6661.595	11078.95	1125.358	26045.75	22679.04	8097.961	10722.82
PBS	39724.3	57745.36	8322.777	15911.83	2082.053	18807.52	24970.86	9562.77	10904.84
PBS	33512.07	47927.33	8848.969	15597.46	1913.597	19257.87	26190.94	8482.004	12113.21
PBS	31019.48	51739.83	9193.368	11925.49	1358.946	20593.92	24685.68	9907.72	11097.14

CP189.112	CP190.086	CP190.912	CP212.118	CP219.026	CP232.162	CP232.896	CP236.141	CP246.178	CP248.891
24200.06	3074.533	4853.304	40470.36	4023.669	2425.36	124201	12283.19	1914.076	22704.34
23835.72	5716.5	6403.399	47620.56	4189.708	2374.018	122736.9	10218.97	1713.665	20884.04
24762.59	6020.18	5259.109	44906.49	3677.915	2474.125	113406.3	10275.69	2285.991	20860.66
26856.82	6121.23	5582.735	43851.7	3076.671	2481.697	120909.7	11086.97	2273.711	19507.26
29144.6	6929.103	6109.819	46931.74	3520.205	2377.591	122131.1	12544.7	2556.294	21273.67

CP352.876	CP116.017	CP221.033	CP240.839	CP243.929	CP250.868	CP256.899	CP294.897	CP429.240	CP104.509
159130.6	33786.83	17428.99	3363.206	6876.313	1963.279	8829.304	540640.6	47950.87	2106.659
149535.2	21954.21	11534.38	2159.836	6708.654	2265.781	11141.53	567869.8	45180.7	789.3495
156670.3	26886.27	9885.413	2059.668	7680.05	770.0165	12659.86	550021.8	44547.9	3656.093
160565.7	26815.96	8750.915	2040.866	8186.418	2419.918	11452.4	552683.6	43397.16	3810.011
174266.7	23806.88	11540.54	1381.06	7338.171	1367.929	14024.29	771823	46522.11	3641.973

CP115.519	CP117.015	CP238.842	CP266.054	CP104.107	CP135.530	CP150.058	CP170.854	CP186.149	CP193.930
1057.558	7702.756	5529.56	42962.91	612664.8	1682.045	127243	6746.653	4264.878	378321.5
1452.897	4815.399	1848.353	47339.75	561779	4949.063	123525.6	5034.338	3831.691	258843.5
2528.145	6323.288	3355.703	35940.73	507507	9480.57	109199.8	5391.442	3161.538	331886.4
2933.628	6264.681	4152.523	33826.99	499214.9	8675.814	102272.8	6368.993	2820.181	318197.4
2555.317	5299.48	3563.738	39738.27	515592.6	6093.345	112572.9	4443.047	3545.897	300704.7

CP111.055	CP172.851	CP117.041	CP120.014	CP132.102	CP135.065	CP137.528	CP162.112	CP257.936	CP274.839
13176.6	4832.732	2119.888	6677.862	16765.52	14171.83	6035.99	467806.6	22031.83	45193.8
17272.85	3672.138	1989.651	5608.24	18829.88	13590.87	4791.37	435257.4	24462.85	49767.86
13674.52	3587.419	2638.638	6286.716	14269.23	15198.73	7785.622	404564.4	19309.76	48959.13
12560.38	5096.336	2845.147	6484.839	15291.6	15884.4	7136.457	382177	20862.15	46628.15
12663.26	3041.188	1874.874	5164.644	16962.58	16031.27	7465.546	397747	17556.77	47315.39

CP376.858	CP394.874	CP362.253	CP132.101	CP115.075	CP176.936	CP216.073	CP365.105	CP516.676	CP112.895
261.7125	749.6337	38631.12	409029.5	58481.12	7019.487	1753.316	37391.41	42782.16	29913.73
851.6562	671.225	41505.05	389588.2	55925.19	7951.144	1605.313	38496.42	35273.1	25968.05
1526.864	833.8226	29383.71	353911.6	63285.49	8364.534	2717.153	40740.15	35088.71	26036.65
1502.979	1127.009	702144.3	334177.6	63705.6	10181.28	2728.073	31355.03	34567.25	28681.98
2873.189	1078.066	27267.35	349159	70803.71	11310.87	2163.045	35243.52	27957.01	23078.58

CP116.040	CP192.065	CP203.052	CP217.107	CP225.091	CP350.150	CP442.826	CP566.889	CP376.732	CP434.738
14729.37	823.8579	26117.2	15454.28	8534.183	1015.536	10451.62	70777.75	53273.07	76591.01
11204.63	914.3028	20444.95	10386.13	7933.353	1077.92	9520.421	75043.13	37096.15	63532.16
12726.42	1062.408	20631.37	9130.134	8502.86	878.2617	8666.389	85235.59	39049.02	67013.46
12410.09	1248.547	19664.12	9549.011	8781.512	774.5963	7353.936	91233.13	39638.86	62282.03
12840.54	933.178	18309.17	9357.646	8062.5	659.3806	7064.155	103279.7	28754.71	51684.77

CP89.0599	CP150.932	CP176.070	CP190.115	CP212.852	CP228.955	CP311.091	CP378.900	CP430.696	CP490.652
19259.8	122013.1	3450.855	3957.992	4967.602	31652.01	1650.261	25024.45	108712.9	65194.93
13603.2	106929.8	3146.054	6804.917	2156.336	34218.26	2412.133	29516.07	70860.76	42075.76
16202.58	110425.4	4088.905	6492.716	2192.914	35705.15	2861.18	33487.47	80473.88	48080.2
15120.67	111656.8	3662.414	6847.211	2897.251	37104.68	2563.368	37492.63	80423.51	49383.17
17087.18	127527.6	4118.56	6197.307	3929.386	40336.35	2878.777	40119.95	50569.9	30279.11

CP492.649	CP182.902	CP201.112	CP295.942	CP432.693	CP432.741	CP86.0966	CP94.5456	CP112.006	CP194.081
40425.17	10773.56	31533.87	26302.29	102311.4	47235.54	15757.15	25222.56	1162844	9445.306
26242.68	9295.107	30358.33	26579.63	69082.55	46813.26	13253.67	20343.85	1164565	10348.48
29779.36	10089.23	35982.89	27412.88	77943.41	43026.72	14970.1	20971.92	1110875	7479.569
31295.35	9437.578	35698.99	28714.85	77629.14	40238.57	14353.28	21872.44	1037534	8373.031
18801.77	10306.86	39624.99	33065.23	47864.4	37991.1	16371.39	32562.2	1429648	9526.841

CP216.923	CP227.955	CP229.143	CP308.847	CP320.818	CP330.894	CP352.898	CP373.874	CP498.901	CP514.679
1331337	91831.68	13949.38	3763.293	51975.46	26451.65	155238.3	802.2151	96120	52937.17
1235630	107458.7	14847.04	3885.45	46788.22	26191.79	145992.6	1322.413	101386.2	39360.45
1174817	105995.7	16082.65	3998.893	46260.17	23858.96	150684.4	2939.929	117241.9	44269.67
1164395	114238.1	14843.91	3466.03	46041.67	21770.92	155565.3	3404.766	126644.3	39425.05
1195005	125666.2	16233.15	4424.817	44680.03	25341.58	170202.6	2786.192	133456	32875.54

CP130.016	CP130.065	CP140.903	CP153.040	CP217.143	CP259.190	CP272.944	CP284.910	CP297.206	CP340.803
15970.99	10245.59	900339.5	64913.64	7061.463	3421.817	17033.59	220194.2	5806.281	105640.5
6390.979	8569.375	833339.9	103112.9	9765.113	3158.203	19489.51	230073.2	5279.134	87634.47
5584.026	9233.356	790900.1	68718.76	7687.588	3455.334	24244.56	209644.2	5559.466	87000.97
4638.848	9378.146	794190	81819.07	8876.008	3105.09	26327.94	218100.1	5164.818	84171.5
7879.634	10116.41	826286.5	69198.93	8633.848	3941.715	36002.88	239105.9	6653.477	72176.49

CP362.926	CP396.764	CP456.720	CP520.842	CP95.5457	CP123.099	CP129.020	CP151.035	CP152.041	CP198.862
308035.3	62474.48	104496.9	6544.29	1930.015	19469.49	17821.95	173180.9	15501.79	727075.2
351805.6	48194.46	77618.94	8808.221	3046.064	18430.18	14617.41	126680.6	12730.67	618317.3
371131.4	50082.84	85358.79	10579.5	5511.749	21062.85	12278.57	115076.7	12104.7	631241.8
391134.2	45336.68	79372.7	8076.027	3784.211	20936.98	12751.91	130288.5	9974.042	617322.3
442135.9	37093.71	62797.71	8888.253	2422.124	23747.8	18057.86	213984.2	18601	547787.6

CP204.131	CP231.903	CP253.943	CP256.821	CP299.091	CP318.821	CP349.147	CP324.735	CP128.019	CP161.080
8236.886	68599.99	1576.684	622802.2	2159.616	173844.1	313.0033	40505.7	80750.72	9546.761
6227.911	54899.87	2482.747	501418.6	3188.887	179818.8	655.1501	38058.77	69848.87	11072.98
7270.262	87628.37	1951.871	486875.6	2523.903	157711.7	376.4704	35569.9	35455.72	9425.289
7173.518	83798.46	909.1373	489425.7	2707.974	153413.6	581.1915	36759.11	27632.4	10139.72
7859.29	75799.58	969.3007	424323	2516.154	150813.1	636.8143	47500.36	40165.03	11858.29

CP190.05_	CP237.185	CP239.935	CP244.923	CP245.174	CP256.94_	CP258.110	CP280.847	CP94.0453	CP95.044_
11745.43	8600.929	272050.7	23272.14	7695.828	4369.976	27694.32	292728	287866.7	39063.17
9148.209	7878.492	229760.5	22245.48	8539.469	1884.319	30425.85	257331.8	234458.9	29548.78
9798.068	8515.97	281605.3	22210.64	8699.503	443.4345	26767.47	255402.4	201409.1	27658.54
10821.74	8395.541	275280.9	22642.79	8179.865	533.1213	22590.77	237010.7	207792.4	35434.71
11062.46	8226.887	287274.9	24636.22	8810.904	3420.275	26315.39	217874.6	425235.1	45185.49

CP124.922	CP130.086	CP146.496	CP149.940	CP152.035	CP157.105	CP169.097	CP182.950	CP189.888	CP190.05_
30422.52	11489.22	8745.442	243085.6	18263.47	20726.21	13901.22	3544331	76402.22	5353.651
22666.27	9767.557	7792.695	180750.1	14626.94	15691.53	14643.02	3058595	106354.1	4772.556
28470.45	10122.93	4185.172	229945.8	13845.73	22205.84	19346.41	4347790	105799.6	6521.599
27506.81	11163.91	10298.82	231909.7	11334.55	22273.64	21849.1	4386891	106902	6118.224
22664.13	10186.2	15396.59	234893.6	15823.14	22841.03	22179.24	4545115	153315.4	6150.981

CP191.091	CP273.180	CP274.873	CP299.091	CP502.825	CP98.919_	CP296.066	CP177.102	CP154.880	CP284.889
3309.036	1527.442	180271.6	1732.528	9753.912	142471.8	2481.159	3405.555	107042.4	65553.62
6043.75	1244.952	151751.6	1787.88	13222.52	129106.5	4055.868	2926.439	99970.36	75047.81
3280.275	1567.873	121704.2	3239.078	13016.18	127397.2	0	3467.464	97580.89	59531.88
3256.346	1578.58	123752.8	2623.165	11278.53	133343.4	278.1632	3270.321	101774.2	52758.51
10201.53	1688.863	128037.1	3037.489	16736.05	122511	1471.395	3108.057	99851.76	57640.18

CP96.922_	CP86.0966	CP125.071	CP148.075	CP156.877	CP160.968	CP464.722	CP126.989	CP146.117	CP218.878
504025.4	12574.6	26984.4	4892.891	69431.14	24544.23	64548.71	29377.05	95867.04	454270.8
460377.6	10240.25	24866.05	4882.803	64820.58	25634.46	69120.59	29052.81	84986.54	397374.6
444796.4	11002.63	28799.48	5559.877	62286.41	26112.89	58205.45	33244.48	84284.27	363356.8
457400.6	10704.65	28896.39	5777.055	67797.74	27636.68	59637.04	30067.33	76367.74	355610
455192.2	13422.94	29296.98	7087.051	61584.46	30085.78	47798.16	33146.74	82601.05	381142.5

CP137.026	CP229.906	CP378.776	CP174.896	CP147.113	CP222.868	CP262.859	CP268.821	CP274.792	CP172.956
1836.481	248221.9	44839.32	117248.6	46436.74	108582	29305.7	60758.69	44177.94	1412790
3961.619	197009	49481.48	118057.9	47843.86	93419.47	303437.1	93515.18	43760.41	1181125
7046.932	319315.1	45016.26	122990.5	51008.65	93021	32684.58	73190.61	40140.35	1646493
6358.653	296451	44932.82	130284.6	47681.44	92574.14	33093.89	65244.95	43370.75	1636876
7424.577	297362.4	51189	146156.5	51806.7	83909.61	346143.5	114906.3	42066.09	1691768

CP572.638	CP136.061	CP167.089	CP88.1123	CP270.797	CP398.761	CP266.776	CP136.061	CP143.997	CP300.884
36875.34	11568.08	60620.64	188057.2	73175.42	75144.44	41408.59	13293.03	191168.2	186035.2
24245.63	10205.14	56765.62	175145.6	58774.47	57998.95	105997.1	10079.78	184531	181566.3
29591.7	10143.42	42416.68	194714.6	58784.19	62910.93	35835.3	11468.64	186190.2	122796.6
29727.12	10213.05	46035.01	192856.4	61240.14	59220.77	33897.81	10049.51	158327.6	117008.5
19627.18	11111.12	47086.49	203923.3	47899.84	47914.53	53331.52	11696.89	191794.2	184940.7

CP316.836	CP514.875	CP137.045	CP133.972	CP102.535	CP266.849	CP336.802	CP103.031	CP417.847	CP254.836
238787.4	7087.204	198123.1	9265.318	49273.67	132791.3	36562.7	8409.476	6499.033	517460.6
190162.1	7047.866	202251.9	12904.52	51621.95	89825.94	45230.07	8787.822	5639.204	350919.6
188553.2	9077.724	117989.6	5793.655	53774.66	88436.39	50426.45	14965.73	10016.81	382186.7
184745.6	11294.77	117576.5	3364.297	52440.7	84620.02	53534.35	16407.28	7601.647	388536.8
166364.6	11760.97	125211.8	6105.256	73043.46	102224.8	64531.3	9564.33	3630.353	290016.3

CP86.0966	CP102.034	CP188.176	CP312.943	CP131.974	CP122.547	CP376.820	CP316.824	CP324.808	CP326.805
7541.938	693437.6	16563.98	11425.9	97080.43	86721.04	6579.477	183336	107695.5	47062.56
5162.624	703229.8	8459.382	16987.89	103466.3	106858.4	8468.751	177360.5	76532.52	36026.7
7718.002	746134.7	6639.684	23078.08	108481.1	96330.4	4403.146	169129.4	85328.59	35952.03
6894.361	706028.9	8526.042	21887.2	112459.9	114024	6003.602	156515.4	80013.68	36606.37
6210.255	1006172	12900.44	16934.36	116541.6	122003	4916.549	151277.1	65844.14	36769.08

CP141.066	CP142.069	CP142.122	CP147.495	CP171.005	CP191.885	CP322.811	CP209.946	CP91.9802	CP168.016
81223.1	3063.126	4624.311	3791.591	4624.711	56682.22	84918.38	148631.7	49774.78	29892.53
66136.71	1937.964	3778.319	0	2218.731	74981.59	63527.53	154746.7	55264.08	31481.37
99980.65	3215.761	5979.459	10840.85	2305.716	75502.98	66679.58	211316.7	54540.31	32142.02
96239.2	3008.13	5589.474	6630.67	0	70607.91	61866.82	210013.1	57360.9	38273.18
93692.06	2637.357	5604.894	14786.7	2610.825	105423.5	53218.08	193723.6	64568.92	48830.01

CP300.863	CP220.167	CP291.884	CP141.091	CP251.888	CP135.063	CP258.899	CP105.52_	CP331.284	CP89.507_
200532.2	218903.2	19829.56	142286.6	128666.8	7673.532	154685.9	67554.34	119320.4	394638.4
199198.4	189943.7	26240.67	140509.1	158108.4	6653.065	187107	52800.68	118841.1	330549.6
176181.7	211858.8	18715.7	154903.7	177593.1	8440.448	199907.7	98630.66	116961.8	706287.8
168753.6	212255.3	18280.83	159121.4	170097.5	7796.052	192480.3	102615.1	120728.3	507905.4
189918.2	207598.9	23992.39	163135.8	223105.6	8153.142	155122.1	108948.9	105451.9	512833.5

CP214.836	CP154.036	CP173.985	CP244.190	CP127.961	CP181.086	CP110.0202_4.27
77482.72	43824.57	194907.8	45417.57	77464.79	13001.91	3990524
68247.82	42651.38	134160	44271.24	63927.69	14347.41	2663397
61383.7	51969.34	237271.6	47660.29	27120.04	15739.02	2845754
64779.58	51938.21	237566.8	45471.79	20397.66	15807.64	2927590
54467.28	62700.38	246102.6	46545.62	33821.17	15719.51	3472848
