# Supplementary material for: Untargeted Multimodal Metabolomics Investigation of the Haemonchus contortus Exsheathment Secretome
Source: Cells. 2022 Aug 15;11(16):2525. doi: 10.3390/cells11162525 (PMC9406637; doi:10.3390/cells11162525)
Supplement: Supplementary file 1 [file cells-11-02525-s001.zip › Supplementary Table S2 C18 -ve (CN).pdf]

Treatment	CN98.9486	CN100.945	CN149.060	CN165.055	CN201.113	CN202.116	CN229.144	CN230.147	CN187.097
PBS	8193.11	1167.805	0	5971.098	12845.28	503.1235	8772.881	565.3428	49481.42
PBS	9533.267	810.0545	384.358	10068.61	15565.27	341.8457	8130.85	0	50769.55
PBS	10038.57	600.1545	392.5627	12147.68	19492.43	648.1764	9911.283	131.5201	64815.46
PBS	10999.3	1134.629	302.5189	9355.729	19040.31	1135.945	11260.39	163.7893	82404.93
PBS	11242.67	286.4914	743.2662	8762.756	19253.94	515.0542	10832.1	121.9578	72543.12

CN188.100	CN203.056	CN216.132	CN195.810	CN127.016	CN410.820	CN80.9164	CN153.019	CN164.835	CN197.807
4637.684	3319.294	390.1286	395754.7	830.5754	2723.565	4622.508	14153.17	201704.3	488066.3
3866.574	3131.218	274.048	347545.6	680.0252	3166.558	4324.275	9159.258	163958.4	550385.5
4983.882	4877.984	283.0738	381378.5	1765.976	4279.266	4994.241	19653.23	261312	496012.1
7042.788	4978.264	400.5252	346814.3	1253.881	4108.057	5668.366	11731.24	198196.9	454561.2
6349.38	4367.966	369.1629	408761.2	1556.944	4432.946	6189.578	9472.886	248780.6	533488.2

CN260.871	CN317.951	CN162.838	CN174.085	CN215.128	CN257.176	CN309.902	CN78.9184	CN249.963	CN223.061
1687.594	27946.65	903023.6	451.8163	8598.246	0	131.9078	3769.961	34110.33	823.1449
1360.946	29382.81	712859.2	759.3939	7794.245	255.4758	145.805	3861.662	34598.09	187.2923
1343.771	30214.15	706788.9	985.7852	6883.399	222.381	167.9599	4950.146	35475.25	713.077
1925.42	32984.3	892747.6	745.5474	11480.74	100.1958	154.507	4317.999	39278.57	147.0977
1275.503	35498.25	800585	1158.951	9913.563	373.4091	457.3212	4656.055	39682.31	182.0176

CN164.956	CN199.804	CN214.933	CN502.632	CN118.930	CN244.163	CN78.9585	CN120.927	CN253.944	CN330.754
408971.1	271786.7	404941.1	51678.25	24818.45	645.9668	454230.3	5657.373	1845.259	85211.54
579085.7	244706.7	368447.7	43213.07	22243.98	740.493	353832.8	5276.103	5166.645	68777.64
344591.7	307340	361784.2	32123.91	20100.59	175.6732	361633.9	3874.922	5688.866	53943.73
442319.7	240182.9	391927.8	37772.95	25465.8	679.097	371259.9	5438.433	4877.287	57373.61
413350.6	255433.2	394849.4	41195.96	24457.75	766.7324	379243.3	5332.76	4327.884	58014.96

CN330.879	CN80.9744	CN82.9714	CN132.945	CN145.041	CN232.944	CN311.962	CN326.759	CN408.744	CN470.698
311.6341	3207924	1430835	1134070	1266.442	104465.3	1118.72	262448.7	69624.46	63555.43
218.8055	2910142	1292516	1014587	920.1204	108870.3	706.1481	206540.5	58201.47	47637.51
2849.829	3215055	1458624	1331843	1063.981	86461.36	0	156605.1	47157.74	40850.66
215.5849	3763335	1700555	1241325	584.5351	98932.39	1308.001	193956.6	54803	48071.52
2251.33	3678183	1685282	1471610	574.0131	102790.2	541.7574	182145.3	52039.42	45835.13

CN315.087	CN145.050	CN159.066	CN173.081	CN180.930	CN198.917	CN265.144	CN284.916	CN292.827	CN324.762
130.7002	30512.16	24117.9	25360.37	7964.191	77526.93	2044.776	27279.55	84181.59	133100.8
147.0224	22821.95	20076.66	25282.09	10091.5	65146.01	1405.702	23370.08	69343.67	106898.4
158.2306	35346.78	29000.37	30406.47	8065.299	66663.29	2078.963	16325.97	51657.73	78132.49
0	39303.96	27938.3	34251.61	9219.2	64149.39	2139.654	18903.91	63316.76	104792.8
173.1415	36370.68	28916.2	33819.23	10949.83	74954.4	1231.983	19007.63	51816.08	94482

CN328.756	CN348.819	CN243.123	CN214.836	CN237.076	CN94.9246	CN96.9217	CN131.034	CN209.045	CN222.868
203383.9	29870.39	130.643	56479.2	145.0402	4755557	786866.3	22152.46	8260.789	83268.41
165563.5	28207.8	457.0947	52699.78	227.3875	4039819	666301.1	15818.31	8660.98	75662.64
126457.7	20968.63	146.4445	40892.51	0	3265104	546798.8	20594.48	10109.68	52882.84
155523.3	26916.02	424.1478	43329.51	169.0947	4167884	695673.1	26786.47	10341.44	75819.89
145385.5	25724.44	0	44908.26	410.6704	3958188	651111.1	24762.92	10062.02	66676.32

CN243.160	CN272.795	CN342.874	CN410.741	CN295.055	CN128.959	CN154.880	CN159.978	CN209.045	CN213.076
7270.779	139063.6	34006.28	127880.9	0	26783.41	479920.5	5841.565	7615.777	1787.175
8171.298	111078.8	30164.94	113335.6	610.4708	25263.55	401130.9	2087.801	6554.244	450.3133
6968.555	79072.84	24723.91	85915.5	156.2154	29616.32	311558.7	1232.179	8259.354	2248.636
9370.692	107975.4	31549.98	104537.2	1081.691	32721.04	413316.7	3539.57	8348.487	1446.585
10429.7	102833.4	27640.14	94669.73	459.216	26992.38	371916	5286.508	9338.048	1226.039

CN215.056	CN215.092	CN226.965	CN270.845	CN274.839	CN330.801	CN160.975	CN160.841	CN166.860	CN289.920
430.9431	593.0423	15600.45	508206.2	151247.2	345609.8	18606.24	894870.8	48676.26	2103.026
242.5878	337.0689	9633.574	416564.9	126109.5	287551.9	23026.05	460250.5	41300.79	0
476.2091	1157.881	5617.799	326471	97434.85	217967.2	24848.37	723617.4	32072.74	139.5487
1618.816	2205.078	9956.927	447688.2	130366.1	293951.2	25611.95	518798.2	41789.67	528.2464
1206.736	2499.684	11700.99	355710.9	108825.8	237820	25182.49	766434.7	41057.18	182.6404

CN176.935	CN186.085	CN96.9691	CN321.923	CN400.909	CN142.974	CN138.027	CN248.960	CN344.778	CN174.955
132983.9	12726.67	7374661	37682.21	12862.04	588035.6	5629.166	751915.9	44116.38	34957.46
143438.2	17055.92	8593064	46479.12	13354.05	656024.4	4958.237	799349.8	40864.58	38896.25
209245.4	16106.43	7496936	54429.3	15651.22	422133.3	6175.254	804188	32590.89	28163.06
136522.6	22993.63	9760599	41948.01	14913.53	713396.4	5094.305	877007.6	38668.12	32621.11
209785.6	20419.13	8028262	50114.94	17150.25	738291.7	4739.46	922012	39303.41	33790.01

CN238.931	CN240.928	CN137.024	CN224.819	CN117.018	CN172.899	CN232.902	CN261.889	CN244.897	CN360.239
142332.5	42023.19	117564.2	32825.35	372822.6	55152.48	118792.8	1315.226	52887.59	33473.04
144148.7	40561.34	107923.7	26676.65	316417	60607.31	123797.8	1814.164	47173.39	31742.55
170634.5	50943.65	127836.1	30171.15	228181.5	63111.72	99586.79	3800.841	36458.76	27017.36
182537.6	56212.25	106002.9	30495.28	254152.9	69378.6	110993.5	3230.844	37114.1	618099.5
191081.3	56349.74	114261.1	34038.26	275713.8	71010.1	118877.1	3910.675	38514.2	28568.78

CN450.760	CN392.801	CN188.951	CN110.898	CN190.948	CN390.757	CN230.9_0	CN108.901	CN216.881	CN228.860
72716.47	110870.9	110708.4	67658.8	70787.12	127286.7	71946.66	117065.6	91492.97	140338.6
60134.25	103504.1	123062.1	58363.86	82370.54	103127.5	79621.6	100020.4	78453.57	128759.6
51784.84	80068.77	93065.54	44187.52	60888.3	80325.92	73698.54	85622.49	55441.31	114180.9
67221.57	107479.5	117999.9	66605.91	77861.45	108537.1	82502.96	112044.7	75570.82	140080
49189.61	82444.97	118294.7	67696.6	75635.47	86810.83	82765.25	111521.9	60949.8	138824.6

CN312.885	CN284.775	CN180.972	CN248.918	CN265.148	CN112.985	CN392.752	CN217.039	CN113.9886_0.61
48401.24	57135.41	543185.9	88040.27	438548.3	3997017	47417.28	101612.2	83358.11
46606.32	46355.11	603075.8	91356.67	502196.7	4099986	96172.15	97042.18	83563.67
41238.97	28767.36	576213.7	98941.51	419198.3	3607905	31094.35	82571.33	71827.65
44724.71	47666.68	665336.9	105113.6	649022.5	4349816	100867.8	72810.83	92256.57
46191.17	39926.93	700820.2	104125.5	511855.5	4679913	38713.31	104608.9	96802.38
