# Supplementary material for: Untargeted Multimodal Metabolomics Investigation of the Haemonchus contortus Exsheathment Secretome
Source: Cells. 2022 Aug 15;11(16):2525. doi: 10.3390/cells11162525 (PMC9406637; doi:10.3390/cells11162525)
Supplement: Supplementary file 1 [file cells-11-02525-s001.zip › Supplementary Table S3 HILIC +ve (HP).pdf]

Treatment	HP69.0452	HP72.0812	HP79.9901	HP136.061	HP144.101	HP155.081	HP156.101	HP156.101
PBS	31609.38	100934.9	82499.46	40631.45	13850.17	55371.7	40034.71	11261
PBS	32169.4	101284.7	81201.71	38709.28	13871.22	54672.79	40000.91	11001.69
PBS	33348.85	94547.86	83028.61	37262.26	14454.4	53640.1	43998.49	11574.83
PBS	31818.24	97162.58	81840.5	38941.04	13599.39	55952.24	39704.2	10782.67
PBS	30800.68	95046.08	74225.26	36034.78	13752.27	52377.71	38530.12	10518.29

HP166.086	HP166.086	HP191.079	HP268.103	HP87.1002	HP130.086	HP206.138	HP86.0968	HP137.045	HP146.117
65105.88	659205.3	75855.88	12595.43	9996.426	65848.23	1804.81	49962.41	1315592	323796.9
62281.11	572406.1	87033.88	10850.22	10557.41	63135.69	2081.825	46857.66	1235277	303011.9
64674.3	610027.4	78445.83	15544.89	10041.44	67246.78	4858.216	48271.16	1284580	308959.9
62108.52	583850.2	74933.63	9921.812	10281.83	66025.72	1832.383	46508.53	1245669	307816.2
62531.21	563946.3	76259.78	10705.81	10014.18	68385.97	1251.968	46014.35	1193115	293360

HP152.056	HP119.089	HP130.086	HP142.069	HP176.128	HP203.067	HP141.065	HP143.067	HP160.096	HP112.050
13949.19	154154.3	57050.79	10600.12	1506.123	166222.2	79107.19	634936.1	21430.84	43722.97
13625.77	131177.9	58684.86	9740.068	2130.936	163126.2	75589.74	667794.9	18384.14	38489.99
13618.11	141007.6	58523.28	10974.48	1446.277	174268.2	80999.92	622672.3	16599.11	46198.52
13622.01	148221.5	56638.28	10841.63	1267.076	169112.3	83611.68	624116.8	16392.73	42922.83
13634.16	130268	60880.08	11079.63	1469.521	178169.5	86031.13	634018	17403.16	44107.83

HP73.0845	HP123.1_1	HP274.873	HP90.0554	HP157.105	HP189.123	HP95.084_	HP159.041	HP108.949	HP186.112
10153.24	60211.4	34536.81	364824	29087	15635.41	26535.86	146272.7	34044.74	8354.743
10187.29	61375.4	29278.85	233525.3	29215.31	13961.94	21896.29	87166.54	27913.8	5949.844
9896.064	60573.69	40190.29	384100	29582.87	17347.9	26852.36	201834.5	32357.21	10666.1
10236.8	61655.34	37722.6	377590.5	29356.42	17543.45	25862.8	175394.7	30178.78	9483.391
10185.69	60321.64	39508.1	368561.9	30627.84	17529.72	24748.73	188768.3	26924.39	10909.21

HP310.912	HP378.899	HP90.0553	HP150.058	HP186.148	HP340.047	HP102.074	HP140.010	HP91.9804	HP227.954
89319.55	67093.6	94281.59	163812.2	6670.091	124279.1	447612.9	723258.3	366311.6	426156.6
72321.51	67883.12	33759.15	120370.2	6000.897	94083.36	483615.3	400776.5	338219.3	381320.4
85319.16	73390.69	124810.9	149464.9	7069.842	121161.3	511214.7	887300.3	361567.5	408862.2
82658	71892.07	110083.1	147130.2	6530.387	103617.6	485521.2	849253.2	360206.3	396391.1
76222.27	63368.15	112094	140385.8	4896.539	106427.3	493194.8	873427	340666.6	384234.3

HP94.0805	HP228.955	HP171.149	HP105.110	HP258.109	HP259.113	HP159.967	HP185.164	HP230.095	HP104.107
1374970	136215.1	681121.1	131397.7	2097356	163415.4	197876.7	72025.51	80032.25	75741.29
936909.1	124831.4	714719.6	126201.8	571088	44326.33	180786.9	65812.39	29340.91	27886.66
1440955	136061.6	740487.7	134891.7	2827135	223045.3	186839.7	66995.06	99556.24	104771.4
1439235	130751.4	725333.1	130236.4	2600443	198664.9	177709.5	68498.84	93787.06	96578.59
1469085	124519.7	695433.2	127109.3	2614079	207844	178757.8	64891.15	109824.3	101367.7

HP132.065	HP391.284	HP116.107	HP87.0557	HP216.063	HP132.102	HP116.143	HP74.0969	HP141.1133_10.79
283862.8	338321.8	1028418	194886	61886.66	522055.4	675719.4	2039660	243032.3
191937.4	627691.8	1951861	200587	30886.72	511182.6	669300.1	2998908	239641.1
420049.5	336807.5	1868723	193882	92749.03	471198.8	684645.4	3010481	249116
373163.4	320707.5	1916150	197321.1	80855.74	448987.9	680092.7	1387755	245177.4
362610.5	633775	1893238	202625.4	88370.1	432901.9	814440	3410893	242514.4
