# Supplementary material for: Untargeted Multimodal Metabolomics Investigation of the Haemonchus contortus Exsheathment Secretome
Source: Cells. 2022 Aug 15;11(16):2525. doi: 10.3390/cells11162525 (PMC9406637; doi:10.3390/cells11162525)
Supplement: Supplementary file 1 [file cells-11-02525-s001.zip › Supplementary Table S4 HILIC -ve (HN).pdf]

Treatment	HN57.9747	HN129.962	HN134.046	HN163.039	HN167.020	HN212.920	HN223.060	HN229.071	HN233.066
PBS	39736.59	8945.667	12027.37	8571.739	331070.2	8308.28	363.9706	18504.07	812.3506
PBS	38644.02	9023.883	13284.49	8302.6	363208.9	8863.991	227.2641	17973.65	734.041
PBS	38169.9	8392.475	13280.3	7477.957	326880.5	8173	107.8954	17079.79	454.1975
PBS	42244.25	9887.319	11795.93	8705.347	355009.4	9745.863	226.0324	16943.31	523.683
PBS	38171.15	8626.688	11770.26	8651.256	347218.7	7545.504	224.5612	17830.8	1103.575

HN98.9482	HN135.011	HN183.847	HN327.087	HN165.055	HN206.854	HN213.076	HN259.097	HN380.843	HN59.9705
1082739	1885.146	6595.682	725.7523	1952.17	117.3902	21871.01	4591.366	4963.518	10385.98
1191000	2059.482	4661.106	875.7932	2485.102	417.9187	21362.54	4230.368	4686.062	9925.55
1181754	1726.152	7332.383	246.7136	1180.311	112.7975	18691.7	2851.928	3554.84	9973.109
1535205	1699.385	7102.933	1896.061	1443.834	282.1427	20113.89	3120.625	5343.978	9917.558
1401414	1695.078	6798.576	0	1194.255	392.9436	19496.41	3411.229	4602.8	9274.985

HN118.021	HN168.962	HN191.072	HN245.066	HN257.081	HN94.9799	HN185.081	HN217.107	HN248.740	HN250.738
417696.4	955.2641	9914.438	8705.447	6201.627	24879.22	13657.11	5417.295	6701.777	16157.01
415892.3	1512.584	7615.925	7757.327	6501.578	25593.23	13941.34	5573.133	7411.628	18397.31
418845	749.4756	9702.073	7919.794	5489.457	24415.98	12236.23	4348.086	8106.275	18161.38
408413	1433.623	8054.691	7433.078	7217.371	23986.37	13313.87	4296.767	7118.88	17064.98
428551.9	1239.984	7237.679	7233.263	5943.312	24819.75	12026.62	5394.66	8255.082	19252.97

HN252.736	HN203.055	HN253.092	HN164.835	HN152.034	HN214.079	HN232.924	HN61.9874	HN100.945	HN164.071
10859.57	9345.769	4622.217	1666116	110313.4	548.1679	1354.614	275037.9	341492.8	206687
11885.72	9451.541	4088.132	1544792	135945.2	616.0522	4425.024	264190.9	377722.2	208170.7
12112.52	8486.082	3703.525	1801229	126294.8	524.932	2195.617	268952.2	378550.7	201194.2
11445.78	9313.135	4859.668	1681352	125916.4	1108.766	7731.332	271623.9	485704.9	193749.7
12357.41	9126.773	4072.176	1638773	131789.4	585.483	2110.994	274190.8	445761.4	200589.3

HN199.80[illegible]	HN213.91[illegible]	HN214.04[illegible]	HN83.049[illegible]	HN189.03[illegible]	HN280.90[illegible]	HN315.08[illegible]	HN117.05[illegible]	HN153.01[illegible]	HN209.04[illegible]
1063258	77533.97	120088.5	6830.184	5520.998	29567.6	124.8186	34756.23	11820.69	7930.278
1046677	106405.3	128296	5933.883	6041.226	29383.14	1001.242	37030.63	13495.01	10399.29
945030.7	104663.6	105478.1	7314.479	7018.832	27874.55	555.4585	33327.82	11052.87	9253.442
909173.9	82215.96	131893.5	9530.462	6939.194	31154.32	825.3727	34461.31	12033.39	9620.061
1036960	99344.93	121605.2	8340.537	5979.955	28751.91	305.6682	35374.17	11432.84	8755.763

HN215.091	HN90.932	HN130.95	HN161.841	HN195.81	HN197.80	HN285.70	HN89.023	HN98.972	HN215.03
29566.43	156906.2	41776.72	105733.8	1783160	2066776	2553.47	1231471	241416.7	85850.82
31802.32	156812.1	50019.1	107529.8	1708153	2198867	3105.754	1280673	231566.4	79252.73
30257.33	155400.1	120710.3	106723.5	1622504	2017960	2979.333	1351180	229998	67781.54
27444.19	151600.7	49585.71	110685.7	1637444	1991701	3299.951	1698186	234723.3	83614.87
28627.5	149791.4	69982.13	109724.1	1769510	2042783	3858.246	1316518	229550.9	76589.61

HN166.835	HN204.791	HN186.084	HN180.930	HN78.918_	HN80.9159	HN135.030	HN206.788	HN332.921	HN296.686
43342.29	59143.35	35531.66	107824.1	124579.8	124888.9	334978	85058.43	40460.48	95.92649
42344.34	54929.35	38832.7	112721.8	131507.3	132226.4	326506.6	78749.33	43849.04	114.4861
40177.91	61694.85	36025.84	97717.99	125263.6	118555	331751.7	91762.49	46782.31	250.425
43453.4	58805.35	34264.42	129041.8	133581.5	133197.2	312163.4	86209.83	45713.92	225.5328
41451.37	59880.99	36391.93	107435	118502.8	118732.5	319517.2	90759.55	39942.9	0

HN294.68	HN330.87	HN146.04	HN197.80	HN195.81	HN195.04	HN219.03	HN588.89	HN61.987	HN162.83
101.2138	30770.21	177493.1	4621382	3795489	196623.9	176890.6	127163	631531.5	5778670
307.8444	35252.58	188378.6	4680497	3712997	348451.3	180706.4	116839.4	504445.4	5574728
458.5469	20648.26	155593.6	4968468	3916836	367081.7	194393.3	128494.7	458103.1	6069271
249.5833	36303.8	191368.9	4516714	3576850	426419	182683.9	119995.6	517478.3	6220471
243.0363	29252.31	188360.1	4630198	3686414	387951	158587.3	125187.2	467118.7	5734952

HN178.879	HN118.050	HN217.039	HN134.8939_12.57
13050.15	161162.5	575006.1	527997.5
12240.62	164937.4	583119.3	526045.1
12178.44	147376	488017.1	532699.4
12415.47	172848	601940.3	403759.8
12104.18	157856.5	518547.8	442924.2
