# Supplementary material for: Untargeted Multimodal Metabolomics Investigation of the Haemonchus contortus Exsheathment Secretome
Source: Cells. 2022 Aug 15;11(16):2525. doi: 10.3390/cells11162525 (PMC9406637; doi:10.3390/cells11162525)
Supplement: Supplementary file 1 [file cells-11-02525-s001.zip › Supplementary Table S5 LIPID +ve (LP).pdf]

Treatment	LP200.201	LP201.008	LP201.008	LP201.008	LP201.008	LP201.008	LP201.008	LP201.008
PBS	11886.61	27387.22	36947.55	28810.47	28207.21	39273.28	37320.49	36018.21
PBS	12227.42	24562.39	40255.02	31027.6	25052.86	36260.05	42603.38	34485.02
PBS	13139.3	29345.48	38471.07	28952.92	29778.1	39536.68	41133.6	40022.15
PBS	9719.564	25107.17	39542.17	28208.73	28145.27	35686.09	41692.62	37090.66
PBS	17185.59	19740.96	38976.79	27495.49	28481.53	31274.47	34145.84	33906.06

LP201.008	LP201.008	LP201.008	LP201.008	LP201.008	LP201.008	LP201.008	LP201.008	LP201.008	LP201.008
41264.03	30297.02	25243.33	37498.87	39116.76	35928.96	29039.55	33204.19	25282.92	30564.4
38878.08	27572.71	23653.45	39547.75	37767.56	34806.93	29791.69	33107.92	27797.13	33011.47
43988.25	26689.14	24060.21	42418.66	42440.42	40193.98	33374.91	32566.3	30981.5	29867.53
41991.23	27864.42	23616.49	44547.52	38855.45	37397.4	33019.07	30431.64	24679.86	34503.99
33380.91	22453.73	25684.59	35750.01	35124.37	24644.72	27158.9	32874.9	19388.19	23632.29

LP201.008	LP201.008	LP201.008	LP201.008	LP201.008	LP201.008	LP201.008	LP201.008	LP201.008	LP201.008
33381.16	33231.96	36623.99	33247.62	41534.06	37225.61	29154.88	19513.46	25014.11	33008.77
34639.62	30038.44	33556.44	35862.92	42554.21	39428.5	22958.07	18980.95	30004.38	34899.5
36954.7	30485.83	40588.65	37182.01	51979.89	41288.99	28828.66	20749.93	32801.35	38623.9
39668.54	32357.27	37740.39	37466.54	43208.98	38412.7	25686.67	18488.86	27915.16	35047.5
35439.75	29153.22	33166.53	31025.25	42189.52	38770.98	26133.39	16692.67	26151.66	33576.12

LP201.008	LP201.008	LP201.008	LP201.008	LP201.008	LP201.008	LP201.008	LP201.008	LP201.008	LP201.008
48915.78	43671.2	30798.67	39659.83	25426.27	27716.14	27262.64	21289.91	27304.39	29805.8
47294.91	44061.09	28044.27	37785.18	26124.97	27644.52	28062.86	24515.12	27934.89	29499.26
50500.73	47567.8	28850.18	39095.65	27636.34	29804.65	31051.7	22712.59	28700.98	33195.56
49113.24	47482.06	27298.53	35219.78	24938.8	26407.02	25310.76	19427.32	30554.43	27907.38
46846.59	46010.44	28487.96	36276.21	26068.62	25798.29	26375.18	21359.61	29019.5	26517.79

LP201.008	LP202.016	LP202.016	LP202.015	LP202.015	LP202.016	LP202.016	LP203.069	LP203.106	LP204.123
20170.9	38684.89	52584.46	57256.35	29247.11	36648.71	48336.76	10423.42	12951.43	21000.93
20454.11	39319.53	49504.19	59123.76	22238.44	33354.36	43718.27	7969.942	11839.17	22751.68
21450.89	58759.8	71804.19	77675.04	27929.94	44458.64	61103.05	9868.094	11951.29	21756.98
20643.43	59582.87	60745.55	76737.69	29800.34	48632.35	63923.59	8394.191	10512.6	23772.13
19138.58	40013.21	49027.56	57994.89	23328.38	35087.71	43650.41	12084.19	11492.71	22992.66

LP204.123	LP204.123	LP204.123	LP204.123	LP204.123	LP204.123	LP204.123	LP204.123	LP204.123	LP204.123
22628.12	21559.92	22417.34	21614.54	34678.01	32038.44	32464.45	24106.95	23317.15	20101.29
23513.37	25885.22	27775.83	18804.81	33254.18	30123.32	31186.43	24700.94	25768.1	21851.91
26876.43	23950.57	24175.3	18136.95	30541.47	28255.44	31455.9	24299.35	25165.63	20204.87
26539.79	26413.72	27123.82	25954.62	32229.35	30672.19	32731.91	23197.89	23220.9	20913.95
26434.72	22089.83	24333.51	20363.57	32293.92	33383.78	30525.71	26868.81	24217.97	21056.51

LP204.123	LP204.123	LP204.123	LP204.123	LP204.123	LP204.123	LP204.123	LP204.123	LP204.123	LP204.123
33515.68	30392.22	25533.12	21112.8	26518.68	27054.04	22873.07	20882.64	21370.88	22985.58
34359.74	31101.24	22139.9	21202.91	26899.98	26336.21	22053.56	24533.06	20244.66	22763.22
30918.51	28549.49	24712.68	19722.09	28483.6	29280.01	21101.52	23335.81	19592.01	20280.07
33566.42	32468.71	23824.07	21685.7	27490.56	27650.26	23912.82	22618.66	20665.27	23604.55
30534.96	28771.75	23581.51	20615.31	25937.84	28626.13	21454.65	24960.62	18303.06	24025.93

LP204.123	LP204.122	LP204.123	LP205.195	LP205.393	LP206.089	LP206.089	LP206.089	LP206.089	LP206.089
32593.14	28302.36	24667.95	18997.32	28526.89	108953.4	472857	467656.4	547649.5	272299.3
32515.09	28176.5	30851.06	19327.12	31822.92	81627.68	516391.4	501327.7	399075.2	421436.4
33193.32	27507	29633.04	20745.13	38737.37	79786.56	390798.4	737875	464310.6	492991.4
34338.43	28919.65	27101.88	17860.07	22907.41	70959.56	562661.4	636629.4	233596.9	212770.9
34874.24	23973.37	31514.51	22860.77	19624.25	58101.12	369682.2	516963.3	369943.3	494864.7

LP206.089	LP206.089	LP206.089	LP206.089	LP206.089	LP206.089	LP206.089	LP206.089	LP206.089	LP206.089
495512.1	599963.8	334755.9	408316.1	445737.8	571212.9	524196.8	568217.3	404129.5	569878.3
572630.4	777179.1	338238.6	335215.5	460140.3	454346.8	550976.6	388822.8	540107.1	415558.9
570283.5	594132.2	265091.9	345802.7	364257.6	495071.9	571751.8	337338.9	509561.6	380453.5
278330	622806.6	321482.6	294089	415309.6	667669.4	466492.7	284511.3	511268.4	436258.1
539857.2	609770.2	200176.3	413941.8	631708.1	635985.2	420122.7	445823.3	485398.8	479442.6

LP206.089	LP206.089	LP206.089	LP206.089	LP206.089	LP206.089	LP206.089	LP206.089	LP206.089	LP206.089
377457.9	459033.8	549034.7	842988.1	402125.3	459202.9	463464.6	417106.7	297748.2	432614.8
429126.9	396247.7	554460.4	661074.3	508550.2	368652.6	542472.3	337591.8	243727.8	458101.6
476604.2	596886.7	779225.2	660286.5	319584.7	435598	411306	316134.9	254771.3	450176.8
368314.3	441966	565938	898931.7	341529.3	440230	458131.8	243801.6	199404.9	404328.8
342888.1	476254.3	837522.4	645279	292243.6	468480	740687.1	301333.7	312801.9	387443.4

LP206.089[illegible]	LP206.089[illegible]	LP206.089[illegible]	LP206.089[illegible]	LP206.089[illegible]	LP206.089[illegible]	LP206.089[illegible]	LP206.089[illegible]	LP206.089[illegible]	LP206.089[illegible]
463315.1	318453.4	186018	528184.5	394476.1	321513.4	412653.3	401973.9	208882.8	327525.3
482941.1	323414.7	172688.6	437294.9	349682.1	359697.8	387600.1	460545.7	357999.1	398330.1
495628.1	253511.4	195462.8	506397.6	375166.5	406998.3	337267	415457.8	347364.9	300692
382124	295812.4	143403.7	445073.5	317799.7	366951.3	401613.8	582305.8	222802.6	341876.9
459559.9	313881.7	192199.2	483501.8	403698.3	369321.3	386425.6	403563.3	230564.2	319555.5

LP206.089	LP206.089	LP206.089	LP206.089	LP206.089	LP206.089	LP206.089	LP206.089	LP206.089	LP206.089
180691.6	350475.5	348279.9	269048.9	401553.9	262795.7	315173.2	258340.3	207858.2	195990
226390.7	349668.9	372875	265522.4	444961.4	322739.8	316639.2	254267.4	299522.7	240056.5
187535	354778.8	286345	262934.7	402170.7	326731.6	339107.6	254022.3	207264.6	197467
180315.7	397398.6	279534.1	317291.9	392801.9	326231.5	298431.8	273869.2	277029.4	174785
195964.1	434279.9	272570.8	262987.3	388810	313115.8	292849.6	236057.2	238564	171606.2

LP206.089	LP206.089	LP206.089	LP206.089	LP206.089	LP206.089	LP206.089	LP206.089	LP206.089	LP206.089
281889.7	279186.5	463885.1	185494.7	163833.6	142097.6	269551	405068.8	163088.4	216895.1
320481.2	383984.8	259310.7	216056.9	257096.3	150380.7	274723.7	217561.6	164080	172892.8
284177	285629.3	254958.9	195703.4	239476.2	136759.1	206606	268918.3	156338	179866.8
274892.9	285779.3	240564.8	199980.4	255254.4	151876.7	330183.3	290318.8	137182.2	142195.1
256364.3	295589	316091.5	201874.6	217868.5	142430.2	169906.5	278859.2	152981.6	186060.1

LP206.089	LP206.089	LP206.089	LP206.089	LP206.089	LP206.089	LP206.089	LP207.091	LP207.092	LP207.092
202972	174312.4	189777	227706.5	105774.2	158576.9	95987.38	54631.13	37082.92	38789.43
172191	154466.1	147651.3	218501.3	98525.07	172483.2	73031.93	36922.71	32007.28	36244.01
194033.9	157582.1	194569.8	200956.1	92283.25	171230.1	79596.37	52518.04	34812.73	41532.17
263720.4	159288.6	142633.3	216168.8	95208.66	176038.5	75150.59	51845.95	34104.3	42442.43
203569.1	149269.2	134890.2	207022.1	75223.93	168476.6	67448.56	56112.29	36466.24	42186.56

LP207.092	LP207.092	LP207.137	LP207.138	LP207.137	LP207.138	LP207.137	LP207.159	LP207.174	LP207.174
43702	25052.49	40623.79	49708.15	40383.05	36114.76	32449.34	13791.82	25824.24	22353.3
35339.85	22826.56	26624.23	36032.98	39417.68	30129.81	33521.97	13512.81	25915.35	28067.9
43109.51	23417.42	29377.18	38255.46	36835.19	32504.37	33578.83	14865.21	26025.3	24641.56
44154.66	24807.83	29038.87	42124.67	28842.96	34540.61	29784.32	11097.28	26146.62	24441.42
40416.79	23489.61	32310.69	45411.95	34385.85	33402.79	35720.39	13153.16	29727.53	25406.43

LP207.174	LP207.174	LP209.080	LP209.081	LP209.081	LP209.081	LP209.080	LP209.080	LP209.080	LP209.080
23232	16222.5	1391835	562059.6	957275.3	4952660	3386761	2968489	1576469	4901024
22286.84	15858.42	1367111	484306.6	1513692	3784450	2053156	2027902	1988857	4898381
22457.97	19623.41	1355307	521633.5	1815963	4744669	2346030	3379700	1629895	4757065
21746.17	16916.87	1713442	590799.9	1312714	5678167	1992707	2433100	1590789	6397420
24571.11	17777.9	2056573	555216.5	1863595	3854543	2531129	1906729	1967641	3226920

LP209.081	LP209.081	LP209.080	LP209.081	LP209.081	LP209.081	LP209.081	LP209.080	LP209.080	LP209.081
2040499	3341233	2249901	5723159	842968	3899146	2118311	1790445	3138899	3280882
1944331	1984134	2985121	4502510	764645.1	3507968	3004631	1820767	2548092	3564188
2238934	4341235	3021314	6326231	637869.7	3097187	2344721	2759694	1812809	2924755
1929712	1895081	1652575	5083205	679539.8	3838670	2297348	2521430	3431231	2450423
1895890	2573064	2299656	4270926	1260709	2539445	2241817	1540584	2363984	3524294

LP209.081	LP209.081	LP209.081	LP209.080	LP209.081	LP209.081	LP209.080	LP209.081	LP209.081	LP209.081
2431413	3740028	1957619	2180641	347598	5003055	1931895	3116772	3302689	2853394
2828628	3793411	1703709	1640282	510001.5	5536965	2407562	4241222	3349229	2149313
2541983	3439815	1735692	1482901	578292.8	4456411	3061851	5100072	3118179	2268989
2921655	4867376	1843552	1707457	339336.5	4815511	2026734	2964692	4704632	1765081
2564265	3924542	1785313	2280307	562456	4583564	3012039	3607172	2981737	2646275

LP209.081	LP209.081	LP209.080	LP209.081	LP209.080	LP209.081	LP209.081	LP209.081	LP209.081	LP209.081
3406388	2723740	2204928	1167236	3401365	1596941	4147365	1870763	5255031	4915832
3749974	3409332	3071156	1028007	3134781	2291977	2777944	1825539	5559619	3337330
7016685	3134106	1750505	1110576	2649605	1800785	2557847	2270008	6320005	3906839
3635803	2466926	2335709	1212655	2331906	1302430	4126569	1877747	3502715	3948019
2882706	2197024	2199184	2086815	2821942	1478639	2123874	1912172	4756458	2595391

LP209.081	LP209.081	LP209.080	LP209.081	LP209.081	LP209.081	LP209.081	LP209.081	LP209.081	LP209.081
2988167	2025983	2607048	3800880	3239894	1438499	1465788	2171436	2902957	2760468
3281337	1330643	2538848	4700331	3186770	1832262	1483000	1342017	3082959	3700030
4408271	1823999	5465116	4554408	3534219	1300414	1550324	2262755	3733664	2624333
3370442	2019534	3053769	4218167	3366361	1370216	1695478	1404605	3845186	3727133
3138983	2671435	2639023	3772319	3218082	1654829	2161917	1782960	2555330	3133073

LP209.081	LP209.081	LP209.081	LP209.081	LP209.081	LP209.081	LP209.081	LP209.081	LP209.080	LP209.081
4733313	360496.4	2007687	2411614	2394728	1067771	2340735	3413382	2570573	2299233
4840474	474585.2	2125949	2443066	3215304	1229580	3036569	3307011	1481001	2606273
7370062	435267.4	1898369	3421855	2874282	1093699	3318221	3723646	1540218	2137509
4325652	419245.1	3262150	3030358	2874072	962214.3	2685130	3862162	1386909	3203304
4102241	612096.8	2489294	1993843	2524616	1138157	2543439	3244320	1565966	2443384

LP209.081	LP209.081	LP209.080	LP209.081	LP209.081	LP209.080	LP209.081	LP209.081	LP209.081	LP209.081
1527624	1654229	1501773	1803199	3842423	1977379	602125.5	1488497	1867331	2734470
1731797	1716433	1707434	1228454	3649193	2619258	513575.9	1128551	1592457	3375390
2001881	1567330	2038964	2556037	3112071	4285310	447845.4	1346863	1813130	2797164
1153263	1570501	1601774	1940009	2916026	1706061	544147.9	2412094	1421394	2340687
2104472	1732438	1915505	1315072	5419575	2547301	537747.8	1668550	2026283	3093195

LP209.080!	LP209.081	LP209.081	LP209.081	LP209.081	LP209.081	LP209.081	LP209.080!	LP209.081	LP209.081
1513154	362593.9	1469116	1881427	373720.9	2326317	369735.2	1426980	2142522	263315.5
1726206	443913.3	1482639	2775863	447329.6	2597864	521505	1544982	2667695	352656.1
1716726	335544.5	2126140	2497381	395389	1724381	394544.3	1566397	3105472	312606.7
1227811	385942.8	1556324	2065020	425216.1	2628668	365375.4	1513104	2069914	285157.6
1416268	389950.2	1246222	2452045	283560.7	2702078	323528.3	1205131	2152226	252821.2

LP209.081	LP209.081	LP209.081_	LP209.117.	LP209.117	LP209.153	LP209.153	LP209.153	LP209.153	LP209.153
319077.9	215724.2	549560.9	2305039	4976132	68873.5	52678.23	60570.74	81243.47	60936.67
436512.4	181483.4	733154.9	2294842	4965289	60273.35	52743.04	63085.46	75703.35	83381.5
538684.4	222872.1	629952.7	2209882	4878116	54426.83	57631.73	62867.88	74998	67445.76
367530.8	224618.9	626575.1	2065982	4413536	59507.88	52159.98	53260.75	63995.77	47893.57
527820.8	203402.5	596669.8	2053997	4451523	46541.46	52592.17	62210.7	83996.98	67942.95

LP209.153	LP209.153	LP209.153	LP209.153	LP209.153	LP209.153	LP209.153	LP209.153	LP209.154	LP209.154
73096.3	33543.63	42245.04	47300.64	64606.57	34269.51	61565.4	52917.22	55863.11	41107.01
59064.72	55493.43	55079.3	52144.86	72027.96	33495.31	56225.7	58611.22	53233.58	43227.01
62854.3	50513.09	61550.22	53570.4	81330.16	34980.77	63044.53	60974.54	55338.54	42995.89
49751.33	55646.35	54932.56	49379.91	68267.64	37839.58	52898.6	56631.27	55398.1	45645.76
61582.44	52199.72	54091.99	49017.52	79770.37	36138.87	52445.98	61335.21	58852.26	47486.35

LP209.153	LP209.154	LP209.154	LP209.154	LP209.153	LP209.153	LP209.153	LP209.154	LP209.153	LP209.153
61930	61563.25	41763.66	48643	64406.93	50014.01	61759.61	62810.28	49531.06	39929.3
59579.49	54759.15	38403.82	49853.74	53796.64	59876.43	56929.41	61656.58	44942.27	36995.99
67435.73	57741.32	41341.08	48644.38	57764.19	57049.39	56190.59	59566.26	48388.23	40886.15
65628.34	57114.11	40945.7	52149.82	57644.91	58225.55	56113.96	60076.49	41480.1	36238.08
68649.32	59838.97	41506.45	52192.89	61074.6	59822.34	55850.87	61917.71	44519.6	35279.85

LP209.154	LP209.153	LP209.153	LP209.153	LP209.153	LP209.153	LP209.153	LP209.19_	LP209.201	LP209.201
63558.04	39593.81	55147.26	44851.1	63705.46	30238.31	41805.52	79672.71	75249.29	77318.13
61503.88	42726.55	58185.58	36833.64	61490.74	29744.45	35261.71	79395.63	74709.58	89959.9
60460.13	44478.67	53901.82	36718.75	62131	36835.18	45133.21	76844.13	72806.34	92088.66
64122.41	51417.28	55314.03	41742.77	68879.21	25460.13	38118.93	77453.85	74313.83	89927.55
53126.84	37656.12	60434.01	35062.95	62166.42	31840.6	49477.63	78713.15	75421.61	97865.83

LP209.201	LP209.201	LP209.201	LP209.201	LP209.201	LP209.201	LP209.201	LP209.201	LP209.201	LP209.201
94306.07	62631.66	92018.75	67786.9	63840.71	45132.67	49786.7	51872.14	75790.65	54136.84
88594.17	48046.49	94036.93	63443.03	63026.25	53029.45	52645.62	58791.27	69946.91	52351.12
108184.5	58771.26	89506.91	75996.89	61734.24	70989.24	50763.01	55756.87	93308.37	52960.84
82794.92	53041.65	94607.4	62545.78	67631.57	49107.46	57222.05	56868.21	66852.17	52868.86
90903.83	61829.72	81098.15	59208.06	53787.74	45772.16	65524.93	62619.68	75150.92	53171.86

LP209.201	LP209.201	LP209.202	LP209.201	LP209.201	LP209.201	LP209.201	LP209.201	LP209.201	LP209.528
64083.68	57794.89	36515.67	57134.91	41426.34	47338.5	39214.75	65228.08	46759	108318.2
56514.29	59303.09	35573.4	51917.11	46472.3	38774.82	36808.13	64039.64	43082.82	117718.1
67416.85	52726.27	37201.93	53490.24	44679.96	49075.4	39101.93	57873.67	46756.68	94345.82
59914.89	58429.01	37577.69	52529.78	45228.37	47385.83	41405.01	72073.09	41446.57	92654.34
45291.18	74993.41	48617.3	52018.43	45251.24	42494.58	41930.51	63226.4	48352.07	86117.57

LP209.529	LP209.528	LP209.528	LP209.530	LP209.528	LP209.529	LP209.530	LP209.529	LP209.529	LP209.528
65203.95	51512	84315.03	70755.34	65210.6	45955.92	109656.9	74119.24	85930.82	63687.84
64634.97	92776.22	111802.5	55062.47	64836.22	61169.14	81806.48	115754.3	100992.4	85764.3
72746.95	86218.06	89104.18	53744.84	67218.25	42652.24	67467.03	78186.9	68390.17	75214.59
59155	58252.1	80418.58	99157.67	104618.5	58346.24	65147.57	84898.75	77416.88	76365.02
69241.99	89777.1	107852.4	58613.8	67882.11	40567.07	123166.9	102664.4	80716.28	111048.1

LP209.529	LP209.529	LP209.529	LP209.529	LP209.527	LP209.529	LP209.529	LP209.529	LP209.529	LP209.528
45250.53	70761.44	58911.29	55712.36	63276.41	54509.26	66957.83	55020.24	52340.64	86893.86
43320.2	75057.64	57834.61	38105.78	61057.81	112388.1	60335.4	97875.73	52185.65	87959.98
46600.92	54804.54	48967.31	35201.87	60248.6	59758.16	40098.94	51091	49523.86	71439.34
47146.95	62358.56	47897.66	42136.88	75164.85	62724.34	101926.3	68869.62	76857.29	61024.16
50558.96	53633.53	54686	56664.79	89258.7	64407.3	49869.61	69157.11	45084.56	56317.75

LP209.529	LP209.529	LP209.528	LP209.528	LP209.529	LP209.529	LP209.528	LP209.530	LP209.528	LP209.530
70302.92	52328.95	52737.11	63070.06	89143.49	59436.13	54349.06	71837.69	81536.22	52626.21
57322.63	62684.95	55402.46	73003.87	61674.12	54378.25	79453.53	60074.8	62495.87	58603.09
57792.07	50987.44	45705.39	49682.48	92297.88	97659.22	60057.73	56212.27	55476.69	45316.83
58147.19	58781.99	47605.82	53651.74	68502.9	74962.88	61851.35	62729.52	92179.79	51530.1
54642.24	63214.46	46008.55	55785.15	48965.99	52087.27	63536.87	56388.2	53452.48	48815.94

LP209.529	LP209.528	LP209.528	LP209.530	LP209.529	LP209.528	LP209.528	LP209.528	LP209.527	LP209.528
55267.02	61536.16	40996.37	51518.02	61324.14	52938.4	41191.79	39331.64	84144.24	47748.77
54111.42	56552.84	55909.95	77333.34	65898.72	51017.17	51214.13	44943.82	51389.28	49866.7
49968.34	51316.43	42613.98	39046	30046.11	44476.49	46256.46	52136.19	38886.51	43517.14
47294.38	57791.05	72587.55	42175.6	31113.66	49617.86	48945.18	44557.86	44438.86	46087.71
57662.44	58671.78	49088.28	40975.33	31548.39	40240.62	50835.96	52065.77	47003.2	47148.54

LP209.528	LP209.531	LP209.529	LP209.530	LP209.527	LP209.529	LP209.529	LP209.529	LP209.530	LP209.528
50708.52	71643.92	56801.72	62515.06	34750.13	43205.87	31498.98	39118.74	19221.29	29239.15
66371.35	60460.46	53456.87	65877.15	38434.51	39775.99	51805.75	43508.24	26968.81	37492.43
45412.38	48811.47	44515.14	36522.44	37144.66	33664.13	31125.65	40930.62	17217.07	29019.77
50751.42	77461.26	45477.97	43979.99	38646.19	30695.88	31577.25	42217.45	17716.25	29989.21
52140.21	64069.52	48024.33	39671.87	34249.85	31053.87	32199.67	42249.47	17309.07	31859.43

LP209.528	LP209.529	LP209.529	LP209.529	LP209.528	LP209.529	LP209.530	LP209.53_	LP209.529	LP209.529
45203.19	46788.9	20393.75	26635.56	29242.34	27939.77	21078.27	25761.04	15451.86	30933.15
61426.34	59712.27	29676.58	39894.96	31568.77	39124.03	30244.56	38250.69	15869.26	44938.03
42691.96	46794.93	19889.32	27010.67	31038.81	28482.05	21725.07	25453.3	14854.19	32151.89
42934.58	47515.49	19803.51	26285	33723.83	28451.38	21335.94	25749.06	13181.97	32357.35
41122.69	46132.84	19622.46	25917.72	33119.95	27672.61	22547.34	24290.64	14937.96	32560.06

LP209.529	LP209.529	LP209.529	LP209.529	LP209.530	LP209.533	LP209.533	LP210.084	LP210.084	LP210.084
14345.32	16177.93	30491.65	30400.13	17437.2	50772.98	49520.3	63679.65	304292.4	286428
16570.15	18245.41	43165.38	44139.75	15856.14	47215.94	50738.93	53886.32	320568.4	265399
13760.88	14744.7	30880.92	30917.83	15762.49	51166.54	51544.36	55174.31	365596.3	263102.2
12849.66	14754.11	29898.8	29956.92	15567.96	47372.45	67662.94	40027.57	236535.8	224160.4
13587.01	15027.63	28594.9	32127.32	15310.55	67296.02	47992.61	57114.76	369018.2	435930.4

LP210.084	LP210.084	LP210.084	LP210.084	LP210.084	LP210.084	LP210.084	LP210.084	LP210.084	LP210.084
516307.1	396935.4	259880.6	336234.6	465158.2	270076.4	40242.51	312644.3	461181.6	590521
644066.2	259519.3	256031.1	395218	396968.2	292022.9	54223.41	336113.7	508556.3	481757.7
883774.1	285587.4	273173.1	301033.7	475067.1	235753.8	46046.58	379766.7	513936.2	627748.1
623524.9	378494.2	212397	352514.5	368238.9	211457.7	60138.93	295258.3	540230.8	471748.2
484125	388682.4	339031	479636.1	379254.2	301489.3	48771.87	437847.1	473459.7	560004.4

LP210.084	LP210.084	LP210.084	LP210.084	LP210.084	LP210.084	LP210.084	LP210.084	LP210.084	LP210.084
519005.1	402422.4	168204.1	558579.2	74239.24	308814.8	141642.5	197064.6	163922.7	149778.6
637635.5	463112.9	246512	549440.4	89659.59	226061.4	238488.5	238761.3	173534.6	161297.7
523887.1	472262.9	424660.1	451638.7	88534.71	247996.2	178773.9	180625	267609.3	172104.4
451133.6	611236.1	208869.7	370385.3	56599.08	300150.6	183664.3	207986.2	152270.5	170846.8
606552.1	456246.4	290513.8	438014.7	104374.3	202793	285301.9	273728.3	230742.5	202117.6

LP210.084	LP210.084	LP210.084	LP210.084	LP210.084	LP210.084	LP210.084	LP210.084	LP210.084	LP210.084
223636.6	497046.1	76786.62	230325.2	214387.6	345096.4	304089.8	502622.3	284635.9	282741.8
213712.2	688441.9	52277.13	263006	209635.1	276850.1	272670.2	466331	324298.6	223674.1
337926	510029.1	71808.8	196162.9	234951.2	415122.6	370146.2	501631.9	477407.2	325629.8
189647	468828.1	54332.99	200745.7	187830	335000.3	336422.6	575475.8	286147	313684
259873.3	741647	71287.72	203949.6	241830.4	374165.6	384649.1	592405.6	376377.1	249300.1

LP210.084	LP210.084	LP210.084	LP210.084	LP210.084	LP210.084	LP210.084	LP210.084	LP210.084	LP210.084
27029.76	189427.6	471370.1	201527.2	266037.2	209681.4	198811.5	178413.5	208412.5	488578.7
28690.21	228462.8	427120.8	309999.5	202196.8	389684.4	203359.3	229451.8	429176.9	458201.4
36301.12	215688.5	420391.9	270325	220807.5	323011.9	228926.1	247651.1	270356.6	499533.8
22193.94	224618.6	563570.1	197079.9	166873.9	295018.7	154849.3	220988.9	230597.7	340052.7
19035.13	195069.8	608079.2	227619.6	317229.4	273619.2	217209.1	240034.2	300319.6	370418.3

LP210.084	LP210.084	LP210.084	LP210.084	LP210.084	LP210.084	LP210.084	LP210.084	LP210.084	LP210.084
618079	289787.4	514777.2	417517.4	231836.9	312635.3	258335.9	176019.9	243356.4	334909.7
395252.3	243771.2	675894.8	432253.4	246568.8	502202.5	323865.8	210635.9	187662.4	375358.6
391228.9	221395.5	698269.4	421204.6	234322.4	350420.5	201237.8	246256.1	212518.8	515277.8
377517.2	295068.1	400406.8	364976.6	198951.6	289121.3	222515.2	174579.3	208338.4	429337.7
416683.8	289530.7	959311.9	431141.9	297808.5	340040.8	287502.4	255800.1	294072.4	525631.8

LP210.084	LP210.084	LP210.084	LP210.084	LP210.084	LP210.084	LP210.084	LP210.084	LP210.084	LP210.084
250746.6	344857.2	356341.2	305276.8	49752.33	293701.7	164316.9	299272.4	223820.6	65706.75
223978.6	374037.1	359996.6	310501	45908.79	417416.2	213398.2	328320.5	202120.4	116237
302882.6	398818.9	435011.5	336210.5	54931.59	560599.7	340488	334229.8	214324.6	107334.2
209620.8	351957.6	427080.6	224815.2	33191.82	527226.4	199866.6	305697.4	231937.3	70330.29
274733.5	343415.3	519924.8	260903.6	45298.75	325656.2	214021.8	487399.7	320965.1	102699

LP210.084	LP210.084	LP210.084	LP210.084	LP210.084	LP210.084	LP210.084	LP210.084	LP210.084	LP210.084
60534.09	635548.3	189438.6	232217.9	155486	190930.4	417053.3	451603.3	37631.83	202175.7
52511.66	923292.3	139426.7	304374.9	137940.9	167024.8	462122.3	421232.7	41158.33	186841.2
68423.01	631384.2	238575.2	270894.4	135498.9	197238.7	340783.6	364246.6	49716.98	185605.1
62234.62	894538.4	191419.5	203838.8	127612.4	184553.9	401602.8	326403.9	40106.29	277568
53197.94	690906.1	110968.6	351646.2	147501.9	218203.8	445675.6	452238.1	34372.73	213873.4

LP210.084	LP210.084	LP210.084	LP210.084	LP210.084	LP210.084	LP210.084	LP210.084	LP210.084	LP210.084
30773.53	399911.7	52229.53	32303.88	37792.29	52036.62	251569.3	38295.26	371282.4	30626.9
27622.44	373541.3	72221.47	25901.48	61017.36	63891.46	365506.2	37566.44	385254.6	38944.59
42050.29	398268.3	52345.24	30906.09	43441.14	56184.3	234833.8	33340.89	353546.3	52115.33
35003.47	661321	65379.55	36845.66	35655.79	58339.34	228687.5	31297.74	308178	27792.12
27408.52	358944.1	49468.04	29943.93	37271.44	71884.12	361894.7	27614.09	313453.4	31208.08

LP210.084	LP211.086	LP211.086	LP211.085	LP211.086	LP211.086	LP211.085	LP211.085	LP211.086	LP211.085
261819.5	83568.86	90496.89	59761.3	84705.33	93564.3	54481.08	84791.64	64626.62	66117
270060	82132.04	84275.81	56960.98	85180.62	88857.66	52025.7	89400.77	73140.12	70958.17
206738	88689.48	86328.19	57544.03	93824.84	91854.98	53795.04	84024.25	66212.16	70599.84
296375.1	91136.9	83309.67	69980.46	84506.27	101556.1	50048.75	88917.65	72784.9	77932.95
256946.7	41566.09	85327.72	60636.11	94786.56	94968.02	57166.61	89195.99	70885.87	35441.62

LP211.085	LP211.086	LP211.086	LP211.086	LP211.086	LP211.085	LP211.092	LP211.092	LP211.092	LP211.092
71245.33	72866.79	55391.92	64322.47	96059.79	108842.4	92675.43	55496.23	46225.13	68020.05
73661.3	70982.94	57773.61	76417.64	93857.51	96202.74	88179.23	54602.51	50624.08	71214.56
79309.87	72824.79	61071.89	63611.18	97094.33	103285.4	91791.57	60099.51	48099.57	68494.12
85787.55	79637.68	56253.16	59596.45	90654.87	102406.4	92096.81	52260	46382.47	65550.49
83355.9	71203.91	60815.83	66055.02	102158	112772.1	94509.58	57403.38	50239.86	58691.21

LP211.092	LP211.092	LP211.092	LP211.092	LP211.092	LP211.092	LP211.092	LP211.092	LP211.092	LP211.093
79103.55	54500.62	88023.42	55313.92	46205.17	64084.86	81277.27	88181.47	68267.46	48141.83
81126.09	55272.73	89886.93	56938.33	51846.21	71104.78	85122.43	85378.71	62614.22	42815.77
78999.81	57657.19	90502.53	57783.18	53662.13	69329.98	84887.09	91641.53	66053.99	45199.31
77898.52	58857.32	97721.19	57759.72	50335.32	68621.64	83022.18	88693.45	66090.89	48060.94
79119.31	51691.1	100236.2	54188.5	50145.38	67604.3	86361.43	96981.48	69189.05	46527.26

LP211.093	LP211.093	LP211.093	LP211.093	LP211.094	LP211.093	LP211.093	LP211.092	LP211.093	LP211.093
60641.26	69473.48	54954.31	62414.86	62313.54	59428.04	80589.19	76061.96	47120.91	54848.45
61326.36	64557.21	51897.66	66962.11	65650.32	65074.04	79742.19	76640.59	50078.87	56878.86
61160.68	59762.16	51095.5	68605.58	62968.26	61096.14	77211.64	76662.22	51547.95	57402.36
59920.4	65886.8	56943.84	60631.4	58191.14	57889.83	81981.8	74675.38	56769.11	62178.59
58994.38	66155.55	53924.26	62661.03	54828.1	57528.84	87006.92	77964.66	48794.48	58684.41

LP211.093	LP211.092	LP211.093	LP211.093	LP211.093	LP211.093	LP211.092	LP211.093	LP211.092	LP211.093
63900.3	47394.64	63908.69	46928.35	86071.65	52205.1	56810.08	99574	86138.52	58665.91
58216.78	48525.91	63868.03	43512.28	77336.13	51558.51	56148.33	107512.6	86388.64	55514.89
58409.68	42271.35	59679.29	47443.25	85429.52	49947.03	55341.12	109303	79877.12	66751.76
62137.2	44829.79	59801.03	44508.14	82153.71	47977.04	59484.49	107157.9	82403.78	63107.11
66344.07	45025.03	51485.73	42877.25	85407.47	46278.7	57762.3	98720.54	82245.83	59720.95

LP211.093	LP211.093	LP211.093	LP211.093	LP211.093	LP211.093	LP211.093	LP211.093	LP211.109	LP211.132
62926.71	88573.48	73540.24	79201.6	79709.61	69101.42	47958.77	58680.09	157929	55848.08
63305.21	94344.58	70441.63	73581.19	70553.72	84056.13	46028.76	56377.4	38766.6	58542.12
60513.94	94293.74	69474.72	84810.59	76903.63	79350.83	49572.29	53393.71	63643.81	66713.56
57925.27	84224.64	70273.41	70037.51	72084.9	79341.23	53257.24	59951	105638.4	62171.11
66542.91	90306.76	68688.23	76512.85	79379.79	79293.14	48118.99	67924.04	86018.59	63918.72

LP211.132	LP211.132	LP211.132	LP211.132	LP211.132	LP211.132	LP211.132	LP211.132	LP211.133	LP211.132
98310.47	65548.45	74286.24	90504.69	105385.9	92267.24	86848.24	83537.52	111360.2	78804.22
102283.4	67220.31	67524.74	85124.67	129150.1	96307.84	98133.14	81514.78	125132.2	100413.2
99838.64	72737.06	81157.6	89967.92	75880.34	84350.36	104872.8	76938.57	80881.6	78253.51
87296.29	66772.92	73891.11	88857.27	128180	104472.8	85854.42	79332.54	82599.95	68022.75
95415.32	63608.55	72299.13	85986.19	90554.84	98054.55	93994.83	79434.18	87089.09	70972.2

LP211.132	LP211.132	LP211.132	LP211.132	LP211.132	LP211.132	LP211.133	LP211.132	LP211.133	LP211.132
80585.85	90836.96	74263.98	76924.28	81229.94	85661.88	68046.38	85886.18	102456.1	87505.83
115671.6	89863.95	82518.09	82472.07	78931.42	83188.51	65904.8	83957.33	97457.11	111643.7
87269.45	99732.12	127710.9	86219.12	85654.76	79686.11	65473.17	88258.99	116010.2	132548
82530.1	92007.24	83542.66	83252.32	75876.89	81761.33	62049.82	83349.11	104298.5	115135.5
72093.73	97870.61	76593.69	82130.54	78389.05	81060.72	61617.44	84960.37	100536.9	110621.2

LP211.133	LP211.132	LP211.132	LP211.133	LP211.132	LP211.133	LP211.133	LP211.133	LP211.132	LP211.132
87221.57	84250.88	75446.77	86344.74	92113.19	98373.52	56549.41	62413.06	98516.98	80302.23
87193.12	82158.88	64534.69	74124.68	88287.33	99246.84	75624.42	64310.94	89308.96	92893.29
89982.66	84513.3	69521.29	75853.46	101727.8	99029.23	68313.06	71182.87	85838.8	88938.14
84828.05	90169.98	68269.63	82379.76	89621.02	96782.7	70908.95	62774.14	90863.79	87106.19
90855.25	86720.5	71179.95	83541.51	92448.1	114464.2	63668.63	66629.43	88177.54	96590.67

LP211.133	LP211.132	LP211.133	LP211.132	LP211.132	LP211.133	LP211.133	LP211.133	LP211.133	LP211.132
75461.64	95652.16	83108.2	76658.79	62102.51	72050.53	78816.14	56388.9	80179.82	96380.95
76532.08	99233.41	87210.25	79881.59	63662.91	79229.74	80499.47	62566.11	81556.72	93513.77
79461.91	93622.25	90300.19	94356	60228.5	68838.5	80647.53	60230.39	75412.16	95086.47
74509.01	103910	74037.56	71238.34	66574.69	73448.35	81292.86	61320.02	78915.25	90905.64
79982.25	102514.5	83786.93	64502.89	60311.22	69930.48	86278.48	64633.56	84128.74	92076.28

LP211.133	LP211.133	LP211.132	LP211.133	LP211.133	LP211.133	LP211.133	LP211.133	LP211.133	LP211.133
96346.3	76669.89	70519.29	73149.3	59136.2	72220.59	57742.01	82458.18	52893.75	70885.93
93688.55	75755.35	74200.2	75253.99	65291.44	77529.75	60851.85	77868.69	50709.18	70435.64
98339.87	76680.92	79756.26	71408.13	79286.8	70604.7	68200.83	79819.59	53042.43	76039.11
94974.54	68983.24	71938.6	74886.19	57322.34	67343.39	55848.13	78201.14	55520.82	65814.53
100594.7	71444.52	67569.7	73206.46	63050.58	64087.38	51058.66	78573.9	56644.88	67241.93

LP211.133	LP211.133	LP211.132	LP211.133	LP211.169	LP211.169	LP211.169	LP211.169	LP211.169	LP211.169
76994.84	64266.98	47530.69	54625.86	32514.34	31734.46	30561.81	40121.82	28640.84	25258.65
80426.2	66467.13	53752.05	55108.71	33913	31918.56	33850.99	40525.88	28249.17	26199.04
70943.9	64516.13	60164.36	58580.2	32540.35	31735.38	28544.54	39275.05	28578.63	26430.99
69126.75	69971.96	54870.97	56430.74	30206.55	33005.83	29556.62	40245.51	25867.46	27388.61
66469.49	67417.07	51035.58	54516.64	43746.44	53763.64	42343.22	45148.6	26437.11	26700.92

LP211.169	LP211.169	LP211.169	LP211.169	LP211.169	LP211.169	LP211.169	LP211.169	LP211.169	LP211.169
30957.49	35922.99	22193.48	40446.51	25148.12	49477.3	19852.08	30358.61	43086.13	35691.03
33895.48	37749.57	25530.82	40284.83	26488.92	51428.7	21025.79	35096.06	44824.4	30531.3
29845.56	33651.61	25688.37	42009.09	25069.11	52653.78	21481.39	32700.22	44432.49	33150.65
33382.24	41026.7	25843.53	40998.38	23153.71	52057.18	28313.65	32743.53	48522.61	41114
32740.67	39941.97	25575.17	44253.52	26489	55157.8	26300.18	33178.29	45356.34	40902.81

LP211.169	LP211.169	LP211.169	LP211.169	LP212.113	LP212.128	LP212.128	LP212.128	LP212.128	LP212.128
34852.15	43292.77	29400.16	44244.95	11529.85	26509.69	29710.73	27701.04	35338.51	22370.37
31303.02	43290.4	29505.15	42699.61	7314.165	23478.87	26168.95	23956.99	36379.34	20044.27
31308.97	49523.12	31421.46	41280.24	10594.32	27502.82	28739.22	26642.19	36139.53	23058.93
31505.27	46036.41	31725.21	46842.6	9421.909	24202.63	33449.26	24878	38731.88	27111.39
32421.98	45218	30712.83	47127.56	11935.18	32223.69	38272.46	28078.17	44948.79	26521.87

LP212.128	LP212.143	LP212.143	LP212.143	LP212.143	LP212.143	LP212.164	LP212.200	LP212.200	LP212.201
22850.29	25428.45	22987.25	34543.25	26564.82	38692	87906.9	89003.43	101820	97412.09
22970.16	23634.64	23295.42	29051.07	25538.56	39573.22	95072.95	113925.2	118685.3	108262.6
22972.05	26467.88	25335.38	31228.99	25613.55	43322.08	88770.65	108481.5	94900.92	113217.5
23329.73	24195.91	28679.26	36823.93	25380.23	50741.55	87016.04	130632.2	89367.87	113248.9
24983.66	29432	30875.97	36122.75	29698.98	48709.43	88026.76	152780	118251.9	70198.48

LP212.200	LP212.201	LP212.200	LP212.200	LP212.200	LP212.200	LP212.200	LP212.200	LP212.200	LP212.200
103148.9	96809.72	67471.4	78033.68	91842.43	72358.22	74119.89	85220.77	83524.18	95363.53
108440.3	109108.8	98204.03	84216.9	82337.02	69167.4	88172.88	73395.19	80060.12	91904.61
104167.4	120862.7	94308.82	80689.65	85052.05	66377.58	85781.26	67138.79	79002.85	87847.83
136396.8	118528.7	83940.87	113983.4	70709.49	62915.65	88390.04	74841.74	80830.35	90437.54
83166.03	139923.7	90610.85	80613.45	77907.88	92394.35	86275.44	63094.53	87394.5	82309.06

LP212.200	LP212.200	LP212.200	LP212.200	LP212.200	LP212.200	LP212.200	LP212.200	LP212.200	LP212.200
121792.8	81271.98	74994.68	67727.42	85867.74	81877.09	68955.57	75706.47	33118.15	87027.79
94572.57	77298.03	73019.73	76197.82	70131.01	63243.58	65176.62	69523.62	34632.88	70300.52
89207.3	77233.42	75152.98	81609.15	74620.95	64336.34	67650.1	75128.65	33552.54	66926.23
87994.52	78873.41	75469.61	71230.32	71624.61	70269.56	64132.98	75407.07	49083.98	65471.87
90011.69	84832.49	71951.64	77405.65	74119.23	66298.36	64548.31	69394.02	32568.7	72013.62

LP212.200	LP212.200	LP213.112	LP213.112	LP213.112	LP213.112	LP213.112	LP213.112	LP213.112	LP213.112
69988.88	80549.73	46638.27	42902.64	48027.37	48051.52	44431	37251.36	40230.14	39621.84
60619.19	65144.02	47871	44128.89	43108.31	50505.94	47068.19	36386.47	39948.98	42274.19
63035.62	80352.64	52336.31	36278.98	52485.17	51349.22	40145.38	36458.77	41252.82	36778.57
71264.61	77706.64	52470.77	47346.01	50904.22	53255.76	48936.7	39717.73	34666.97	46179.41
68677.95	76915.71	45996.19	45233.73	50007.94	53798.55	50529.99	39822.56	42301.81	44730.67

LP213.112	LP213.112	LP213.112	LP213.112	LP213.112	LP213.112	LP213.112	LP213.112	LP213.112	LP213.112
33147.04	54447.42	48062.46	34349.35	45089.44	47943.42	36973.19	34391.58	51139.23	43728.71
36471.62	53419.04	48025.69	32787.45	50844.83	45439.7	37715.59	31349.25	48900.08	46597.1
33290.24	55940.43	43548.06	36044.85	44274.72	47778.54	38136.99	33008.46	50162.5	48290.32
35698.25	55123.21	46801.76	33974.25	49902.26	50817.24	43322.45	34348.84	54008.58	48421.37
34221.49	56466.95	53967.4	32950.66	50397.02	53138.39	38723.09	33723.92	54611.66	47759.33

LP213.112	LP213.112	LP213.112	LP213.112	LP213.112	LP213.112	LP213.112	LP213.112	LP213.112	LP213.112
40701.56	41117.75	33501.26	36074.94	39394.03	51421.8	54132.82	32509.37	60347.5	70964.34
40311.1	38322.03	33487.29	35197.16	43914.77	52393.06	52661.31	31855.2	60425.91	65038.03
40957.84	43214.77	33303.49	33926.94	42436.67	49996.93	51287.28	37814.2	63370.39	70977.41
39434.56	38649.36	34417.35	38766.84	42713.92	51730.37	52563.93	31002.03	67193.54	66799.46
42684.16	42201.59	30955.6	38390.11	43836.13	49412.87	51820.45	30847.61	60290.43	71126.45

LP213.112	LP213.112	LP213.112	LP213.112	LP213.112	LP213.112	LP213.112	LP213.112	LP213.112	LP213.112
56115.12	41637.54	39607.64	33273.85	41396.17	26957.45	31069.96	54670.87	34200.64	32877.26
61282.48	38696.56	38210.14	34515.7	44526.41	34892.61	30281.07	57181.62	32021.38	28581.67
57565.25	39357.73	41491.96	37195.28	43313.38	34492.43	29860.64	54802.41	36029.61	38295.27
57543.34	39943.34	39386.57	35116.69	42576.95	31808.68	31106.5	55094.58	34929	33325.23
61913.29	41933.15	40770.45	40026.12	44226.7	33269.59	30779.94	60523.96	36287.79	38644.58

LP213.113	LP213.112	LP213.112	LP213.112	LP213.148	LP213.148	LP213.148	LP213.148	LP213.148	LP213.148
49375.6	58108.9	35536.99	45414.66	64682.75	37959	47133.82	42550.1	32393.2	43096.9
46655.31	57741.88	38818.52	42161.98	67281.39	37968.05	47089.16	49434.29	32326.85	43327.76
50757.57	58764.69	37524.26	38590.4	59345.37	31246.4	50620.32	44622.77	35413.82	41088.92
43803.43	53526.1	36895.35	42967.62	68810.75	36304.19	49922.79	43664.5	33367.76	43705.4
47982.19	58602.44	41115.91	44705.49	69135.38	37488.32	51959.44	50111.79	34924.43	45064.81

LP213.148	LP213.148	LP213.148	LP213.148	LP213.148	LP213.148	LP213.148	LP213.148	LP213.148	LP213.148
52357.78	51414.74	67284.73	64150.79	79765.5	41910.4	50452.56	41291.29	49250.18	43266.14
48585.82	54241.3	68470.32	64403.62	80798.5	46305.96	50530.31	45192.38	51703.11	47509.48
50676.45	50342.16	67692.96	58835.39	84262.84	46207.51	41897.33	43224.3	52419.8	45581.77
52352.79	48471.64	68517.71	66895.68	79966.64	43771.6	47023.91	44668.07	53130.25	45189.75
51573.52	52810.64	69646.96	67965.96	84497.95	50925.64	46944.02	42578.3	53073.85	46381.63

LP213.148	LP213.148	LP213.148	LP213.148	LP213.148	LP213.148	LP213.148	LP213.148	LP213.148	LP213.148
32759.32	51190	54366.55	47490.59	40155.44	69330.08	41811.81	56789.06	39476.99	77006.2
37530.26	49834.67	52005.35	44343.74	39417.13	72009.12	44911.22	57506.98	42832.09	75888.2
32120.57	54996.01	55788.95	47811.74	37338.98	72706.32	42921.08	55879.92	45303.95	82038.73
34959.17	60060.91	52783.2	45036.72	38169.88	72349.8	40227.45	56032.97	41333.25	74332.25
35320.52	62384.63	58589.61	49590.92	39484.9	75391.57	45381.8	59828.3	42878.06	80827.17

LP213.148	LP213.148	LP213.148	LP213.148	LP213.149	LP213.149	LP213.148	LP213.148	LP213.148	LP213.160
99793.33	50346.53	38435.62	42876.23	76433.19	51244.67	40493.3	44942.82	50785.16	78818.43
91299.89	47745.74	38766.4	45016.31	85366.91	51677.67	40191.68	48882.51	51243.46	113483.2
97932.52	49151.3	40712.15	46405.41	81430.85	53031.77	41736.84	49733.86	50923.74	126577.2
103517.2	50890.52	38568.18	44696.79	57935.23	32236.52	40291	46682.03	53119.01	98890.75
106372	49644.35	40466.26	53876.04	92082.76	58974.16	45588.31	48132.52	51297.77	114638.6

LP213.160	LP213.160	LP213.160	LP213.160	LP213.160	LP213.160	LP213.160	LP213.160	LP213.160	LP213.160
118474.7	67306.49	93875.55	98970.56	111799.8	108637	80114.73	85999.71	111845.7	89248.1
73005.26	67679.53	135006.7	93179.59	134980.6	88763.57	92624.64	79325.28	95182.52	106102.5
97687.25	111361.1	124877.7	113157.7	73922.15	115506.2	121409.7	112647.9	91908.15	108511.8
120620.9	92007.92	92564.18	148064.7	98883.77	116324.4	109675	77981.16	112702.9	143858.2
134817	86569.9	154104.2	90899.01	102489.6	128002.5	109156.5	136896.7	113926	126221.3

LP213.160	LP213.160	LP213.160	LP213.160	LP213.160	LP213.160	LP213.160	LP213.160	LP213.160	LP213.160
86570.45	90999.55	80751.46	91095.58	124240.2	108955.2	93992.23	107628.1	90533.42	83681.03
83570.64	68391.58	118901.8	91469.72	85075.83	89658.29	90630.42	147425.2	68828.27	89873.4
88540.44	74023.06	124068.5	77396.98	106092	82503.59	161142.2	69218.7	74870.3	89930.62
124445.2	98748.04	100545.1	114572.1	103519.3	85005.65	126219.4	124719.3	71398.55	104107
96736.52	90095.65	70111.27	120126.3	77816.25	134509.7	97451.67	90975.64	86356.74	107684.2

LP213.160	LP213.160	LP213.160	LP213.160	LP213.160	LP213.160	LP213.160	LP213.160	LP213.159	LP213.160
101624.4	136892	79646.86	73073.07	121879	86758.38	113858.7	76274.44	106957.1	84053.9
121205.2	83279.7	93926.99	74329.15	102252.3	91346.58	82560.2	80010.82	88124.32	59511
79498.65	102862	91056.24	70001.29	91648.75	88474.51	91568.73	76462.3	85049.69	69666.79
79051.03	110634.9	74487.06	114548.5	91404.69	110522.1	104492	109545.6	75242.36	67377.63
154273.6	115497.8	112114.4	101764.4	156396.1	110094.6	104437	84565.73	100447.9	77853.59

LP213.160	LP213.161	LP213.160	LP213.160	LP213.160	LP213.160	LP213.185	LP213.185	LP213.185	LP214.143
90979.19	80947.93	79524.48	55967.44	57172.28	91738.35	63956.12	75497.57	102310.9	423021.4
90176.53	68746.65	78064.31	50795.1	72554.71	97738.46	71736.42	85498.51	99121.69	396066.7
96387.05	74692.76	71175.78	47779.83	68399.17	92135.59	65062.17	77495.41	103205.9	426541.7
108128.9	88725.17	104785.8	51166.53	74932.04	93949.07	75685.17	85417.54	101670.2	397343.3
108425.9	90222.19	85875.16	55037.95	78424.8	107050.7	85786.71	88308.55	124433.2	476766.9

LP214.144	LP215.092	LP215.092	LP215.092	LP215.092	LP215.092	LP215.092	LP215.092	LP215.092	LP215.093
298219.6	92860.96	36937.73	87984.22	55527.83	40745.04	65897.94	103346.9	71490.6	44023.54
320637.5	89793.89	36830.41	88765.65	66469.25	48858.8	64727.87	97277.92	70878.71	43688.32
317546.2	53309.06	38313.8	92215.28	69056.15	28496.58	64493.06	53669.43	69926.67	47032.11
323854	92421.47	41277.79	84966.26	68549.9	44749.94	66241.68	108015.2	72114.96	41828.8
394153.6	106426.9	39016.98	101611.4	70326.54	53985.87	71751.06	100612.6	84058.31	42537.12

LP215.093	LP215.093	LP215.093	LP215.093	LP215.093	LP215.093	LP215.093	LP215.093	LP215.093	LP215.093
54875.2	54925.05	50747.91	56469.76	38617.3	46084.84	40251.76	57295.03	35348.6	31945.75
54885.75	50956.55	46103.77	53335.11	42385.34	44507.32	41378.03	58501.51	34806.25	31189.17
52412.9	54206.23	43133.73	49986.3	41387.67	44195.43	39835.34	54171.12	35207.39	32023.63
50510.75	52809.05	45826.93	51433.19	39582.81	47819.69	39298.83	54339.56	35287.57	31933.18
61525.29	64835.72	57269.47	64907.3	47295.18	45810.26	45696.75	63097.58	42787.27	34255.08

LP215.093	LP215.093	LP215.093	LP215.093	LP215.093	LP215.093	LP215.093	LP215.093	LP215.093	LP215.093
46470.89	67584.23	54003.04	46783.53	75450	55139.12	74011.14	43234.17	51665.06	35087.58
52431.21	69400.75	48132.22	49291.52	77254.3	61236.09	71526.57	40410.86	47374.63	36427.95
49768.74	31873.26	52139.55	45341.22	75317.55	52987.03	72859.76	43797.29	32680.99	33945.75
48925.26	70441.4	55580.26	42570.69	70828.46	63246.21	70297.58	41628.88	51108.66	33010.8
52825.65	74369.92	56620.84	52761.89	81759.83	72569.39	72953.6	45214.85	57123.55	38151.45

LP215.093	LP215.093	LP215.093	LP215.093	LP215.093	LP215.093	LP215.093	LP215.093	LP215.093	LP215.093
71860.16	51378	56402.02	49602.84	36078.8	48854.73	41937.65	43465.58	37411.15	81621.62
73322.93	43675.1	58747.06	57607.73	37488.36	50310.37	47969.32	40884.38	36987.42	80380.01
78157.82	45621.26	54050.18	49801.44	36903.02	48606.69	43970.51	44139.94	36582.92	75910.25
73219.37	43968.15	54128.18	48070.79	37921.48	52341.38	44497.09	44695.21	35578.44	79759.8
79978.32	48989.19	60633.1	55418.43	41350.73	49905.85	48681.81	41630.2	39779.15	88162.44

LP215.092	LP215.093	LP215.093	LP215.093	LP215.093	LP215.093	LP215.093	LP215.093	LP215.093	LP215.093
60438.13	63244.44	59856.37	39513.8	39575.45	37853.62	56859.91	37920.33	28754.2	63048.6
63796.65	66184.01	56144.56	41505.01	38875.46	37220.57	61678.43	40905.89	28611.47	66426
40887.02	63154.95	53204.32	41467.95	33996.96	39911.23	56133.54	41047.29	25066.03	31003.8
65377.17	63155.91	52273.24	37464.1	35299.93	32665.78	56989.86	45372.54	28204.41	67775.47
75314.74	77193.71	59203.51	41181.91	43999.41	37839.8	65977.37	49708.8	30788.93	67990.36

LP215.093	LP215.093	LP215.093	LP215.092	LP215.093	LP215.092	LP215.093	LP215.093	LP215.093	LP215.093
46417.99	55909.51	82004.28	47642.48	42180.91	106528.4	58960.28	62747.03	56472.43	70789.74
47868.99	51050.01	87452.01	48635.84	41778.49	100726.4	55739.28	60508.03	58083	68942.92
50100.87	57888.78	51583.14	46395.38	37819.09	54014.24	57687.85	34146.11	50456.22	66512.04
45886.21	55070.02	86883.18	52216.25	42688.28	100198.9	64289.47	66349.3	54648.01	72083.45
50133.51	62357.81	88054.15	55121.39	42194.58	104901.7	68181.96	74136.63	62930.87	79144.23

LP215.093	LP215.093	LP215.092	LP215.093	LP215.093	LP215.092	LP215.093	LP215.092	LP215.093	LP215.093
75779.31	62507.58	64986.98	64296.8	54216.66	73356.83	58692.49	70250.03	31978.18	73581.36
76183.06	57469.36	70321.18	59833.49	57556.6	67469.88	64718.21	72564.32	36995.96	67247.68
50279.56	62727.72	62069.5	34710.68	57370.47	68350.75	64654.53	73004.79	32781.19	37466.88
86470.46	58192.02	60647.32	65606.25	57294.5	66990.26	56931.8	75976.62	32433.56	69296.05
85297.06	68584.8	78013.61	72133.34	60855.77	80252.08	67578.8	80895.2	37320.98	77883.39

LP215.093	LP215.093	LP215.093	LP215.093	LP215.092	LP215.093	LP215.093	LP215.093	LP215.093	LP215.093
88659.07	55422.42	51555.32	47679.14	49328.91	69811.82	48509.07	48443.79	66155.81	79882.27
80107.95	54902.18	50250.55	47493.07	41283.66	69228.88	50177.95	49911.48	67451.61	85195.02
41659.79	50203.97	53849.8	48138.69	48795.64	45731.27	42917.38	53208.42	66230.36	37655.5
82816.74	54520.6	50079.22	51470.21	49777.23	71811.94	52175.26	53801.06	61337.49	81879.47
91389.7	49083.67	52307.94	47232.21	54536.84	81563.16	56026.06	62107.05	69325.26	95723.3

LP215.093	LP215.093	LP215.093	LP215.127	LP215.127	LP215.127	LP215.127	LP215.127	LP215.127	LP215.128
72400.33	77522.56	62077.78	52960.25	59803.48	35097.6	57293.06	60651.2	47319.39	86827.29
76218.39	72236.82	59456.65	66827.89	57589.4	32236.21	50158.49	63096.97	48430.04	83126.62
34246.09	72042.38	58734.15	100717.9	73622.51	34897.67	53932.73	63824.15	49100.22	99109.03
72435.75	66943.97	60907.09	54208.47	57295.63	36414.28	61153.47	63702.86	42065.08	93022.02
91572.6	89766.47	72044.17	77652.08	69184.83	43224.89	59598.87	74352.29	57482.87	66023.37

LP215.127	LP215.127	LP215.127	LP215.128	LP215.127	LP215.127	LP215.127	LP215.127	LP215.128	LP215.128
84218.97	89707.41	89911.73	89990.86	91471.51	46882.33	66656.27	84694.63	83440.76	70099.38
71642.28	102490.1	97232.03	104084.6	73692.93	75682.65	68478.82	73918.09	106657.6	68645.62
75647.65	76733.83	88215.74	76463.81	77463.64	72880.77	62938.25	81283.84	83078.82	62768.07
83059.36	141679.6	94823	86066.69	98121.65	58217.91	82585.52	76786.14	99846.25	90848.61
110801.7	124734.5	81624.7	123525.6	76644.7	66653.87	70762.69	141607.4	82986.09	97784.36

LP215.127[illegible]	LP215.127[illegible]	LP215.127[illegible]	LP215.127[illegible]	LP215.127[illegible]	LP215.127[illegible]	LP215.127[illegible]	LP215.127[illegible]	LP215.128[illegible]	LP215.127[illegible]
56771.36	74303.03	72966.18	73355.86	67722	79321.26	85748.34	65929.32	73525.59	62300.05
84791.51	63094.79	72968.34	60977.7	70260.29	67607.09	117038.8	57612.56	50779.92	67317.96
69659.01	90862.76	63053.48	79662.78	67463.01	89418.64	92160.24	62939.84	74538.8	65373.66
79170.67	83442.05	96504.45	72657.06	66289.84	87602.36	66926.77	85268.19	74706.38	79992.26
93565.46	74733.09	69659.23	81859.51	67789.51	86498.6	81556.42	57628.44	77672.56	75034.46

LP215.127	LP215.127	LP215.127	LP215.127	LP215.127	LP215.128	LP215.127	LP215.127	LP215.128	LP215.128
79541.86	69917.34	57113.51	87218.54	63290.56	75669.44	58688.4	72161.21	66425.26	62718.2
70895.21	92596.84	71728.93	64275.19	60847.79	88443.73	61881.3	73604.12	69721.19	46935.44
95185.33	68951.92	65654.3	83247.24	61477.95	78406.23	67360.85	72852.08	61274.66	56516.35
71127.17	83447.62	86482.36	59130.24	65288.16	85184.87	61787.46	77722.46	61203.79	59859.72
81275.76	77860.66	49795.47	123325.3	66315.99	82785.65	72644.31	87081.42	76382.76	67144.24

LP215.127	LP215.127	LP215.128	LP215.128	LP215.128	LP215.127	LP215.127	LP215.128	LP215.127	LP215.127
62503.65	74010.65	55128.94	55284.76	56176	48840.27	58250.58	42851.43	52710.96	45755.15
66671.93	55201.63	57595.96	50499.52	55320.23	42547.99	60809.42	45278.9	56457.19	53796.15
60882.08	74046.65	56975.47	56721.63	51336.54	59076.15	60333.28	57479.59	53543.05	45691.29
68276.36	70208.96	65582.58	69708.01	56823.78	57804.03	56554.18	42811.37	54059.81	47646.62
78943.4	80156.14	64837.35	61913.28	65482.41	64361.03	66270.73	45490.27	60268.9	50724.09

LP215.128	LP215.127	LP215.128	LP215.128	LP215.128	LP215.127	LP215.127	LP215.128	LP215.127	LP215.128
38170.49	37447.71	41658.51	37019.5	59200.36	59945.86	79564.06	81540.67	51019.02	58495.47
33065.57	44017.01	46600.96	35979.88	65359.19	57826.75	77730.67	75215.52	55063.76	62663.86
36498.53	36660.99	44356.92	40011.02	88490.1	62777.5	82523.7	79806.18	57483.13	62632.4
39106.65	39815.4	53270.6	36685.83	58710.04	62909.91	84041.95	78193.09	54018.85	64943.63
44623.39	40693.52	49568.4	38157.07	70693.54	65203.8	87077.36	95858.97	58974.15	69391.55

LP215.128	LP215.127	LP215.128	LP215.128	LP215.128	LP215.164	LP215.164	LP215.164	LP216.085	LP216.085
37799.74	62917.45	45968.25	40381.52	62260.48	23303.14	22321.93	27216.06	20661.46	20727.84
39743.8	58034.15	54082.19	40085.31	63443.45	28138.53	26321.75	24241.11	17773.62	20392.85
38755.28	64595.51	57831.63	46935.49	65295.93	24947.39	24456.19	25949.57	19088.94	21603.22
43670.07	67617.55	52099.13	42851.63	62931.25	22207.08	27350.13	25588.91	19092.86	23549.94
45627.7	69321.85	54385.89	44865.94	74066.95	25844.42	24518.05	27759.9	22742.68	29039.03

LP216.085	LP216.922	LP216.922	LP216.922	LP216.922	LP216.922	LP216.922	LP216.922	LP216.922	LP216.923
22523.94	27830.81	42508.67	62793.77	44298.25	56747.37	71784.48	53171.61	48950.27	57495.86
19900.41	23978.92	37897.33	54842.04	41912.31	60139.11	76818.66	44601.05	42152.57	52948.63
22654.23	23549.26	42967.71	56607.04	37258.39	55365.85	73772.51	50563.48	44536.81	50937.51
19303.67	24434.12	44358.12	53044.48	40190.94	63674.27	73475.61	46925.24	46799.06	56940.19
25874.97	23696.33	38939	56318.41	43279.16	59111.1	70473.19	48917.51	44800.17	58515.42

LP217.068	LP217.068	LP217.106	LP217.107	LP217.107	LP217.107	LP217.107	LP217.107	LP217.106	LP217.107
65331.86	72968.28	42301.68	57447.96	66239.49	53820.06	58768.13	48671.14	45034.58	49402.77
67608.02	77579.14	33906.44	50317.79	56475.93	81207.56	55809.79	41596.75	39632.34	57116.4
67043.77	78606.6	35008.29	55528.86	57575.13	52844.2	56795.64	41599.52	44756.02	45873.77
64702.62	76379.57	46531.19	69856.14	67040.54	57903.15	92209.37	62173.2	40887.25	80200.75
58941.86	87781.26	52101.23	63816.65	106810.1	95823.7	55118.85	51003.11	45761.58	57202.43

LP217.107	LP217.107	LP217.107	LP217.107	LP217.107	LP217.107	LP217.107	LP217.107	LP217.107	LP217.107
42881.46	39217.27	43602.51	50078.93	55677.16	58582.51	35355.23	40498.54	52663.5	52684.66
55217.08	40293.61	41438.02	60882.58	63833.11	60134.22	27846	40648.29	51926.57	42317.43
37603.89	39934.36	46388.47	67030.61	58878.93	54187.07	28747.67	38997.38	48469.55	49785.73
64175.11	62961.99	45898.34	61405.17	58187.36	56108.24	32034.36	43656.93	49081.58	50225.15
42725.15	40090.17	39438.5	64207.87	56206.54	63306.91	34121.75	36849.34	48476.93	50044.94

LP217.107	LP217.107	LP217.107	LP217.107	LP217.107	LP217.107	LP217.107	LP217.107	LP217.107	LP217.107
37094.32	39112.18	41289.09	40042.58	34191.48	36658.64	46706.76	36122.69	37262.48	22055.15
39017.59	41203.58	39440.34	47619.24	39054.4	34474.66	48468.73	34588.54	37860.62	22583.34
36961.16	45588.58	41092.28	38885.83	37349.93	38600.58	51086.88	36679.63	37458.04	24031.99
36588.74	40019.24	38380.53	39839.8	36922.88	38214.04	44559.85	37526.84	38192.89	22361.9
32214.78	46996.38	35378.04	42521.61	35976.38	36171.77	49446.95	38154.81	39739.3	21176.85

LP217.107[illegible]	LP217.107[illegible]	LP217.107[illegible]	LP217.106[illegible]	LP217.107[illegible]	LP217.107[illegible]	LP217.107[illegible]	LP217.107[illegible]	LP217.107[illegible]	LP217.107[illegible]
29985.72	29167.17	58675.03	41532.57	23288.48	61985.56	87382.82	54398.65	51143.12	65352.07
32740.96	31336.32	52200.9	37213.17	22238.42	91453.06	90624.31	51584.32	44054.12	82555.56
34378.39	30457.31	53108.64	42366.15	23917.84	63653.21	76106.08	56879.83	42102.92	63360.31
33007.29	31008.09	53892.43	39417.06	24880.13	59380.46	68352.33	71593.38	43441.89	85758.98
35068.31	27314.13	49964.75	35977.81	23135.04	66461.02	62956.55	70244.42	51832.25	60789.54

LP217.107	LP217.107	LP217.107	LP217.107	LP217.107	LP217.107	LP217.107	LP217.107	LP217.107	LP217.107
49488.03	60510.52	40503.84	50821.8	50367.93	50296.89	48572.77	38715.37	55994.56	52376.35
47801.88	59119.33	43400.69	49559.98	59644.45	46147.71	53423.06	86817.07	67949.2	49380.9
49656.01	60009.31	37463.17	50866.28	50570.01	45705.43	54182.59	35820.36	57517.86	71743.1
57492.59	63729.31	40665.6	51411.57	52415.83	54841.3	49865.63	43706.28	55075	46294.68
54425.27	61883.05	44912.52	50339.62	51189.55	47433.47	57006.24	37290.01	64570.69	48494.52

LP217.107	LP217.107	LP217.107	LP217.107	LP217.107	LP217.107	LP217.108	LP217.107	LP217.108	LP217.108
51457.22	48032.07	56655.51	35658.65	26549.66	35982.1	66918.56	54408.17	44435.57	37157.79
55186.67	47203.32	69232.1	32408.61	33938.25	37408.46	72085.79	52019.04	40555.67	32798.58
62023.28	56820.05	55322.81	30793.4	33281.94	37326.66	69420.65	54485.16	41824.35	34095.78
48005.18	62652	57340.18	34270.49	32876.62	37464.17	68548.75	55305	40819.04	39932.83
63552.31	51282.33	56082.15	43798.53	30425.77	39196.84	79595.4	54822.71	41372.2	31262.2

LP217.122	LP217.122	LP217.122	LP217.122	LP217.122	LP217.122	LP217.122	LP217.122	LP217.122	LP217.122
57133.09	42078.08	66286.99	36226.93	52809.67	41414.7	46543.28	48478.84	57632.77	68410
54151.11	37324.42	63888.69	38571.14	50595.99	39859.06	45282.61	44536.53	56223.94	70705.01
56941.4	41707.21	67386.18	42437.51	54686.18	38880.19	49331.76	44465.08	57897.61	84524.97
64074.83	47468.2	65656.95	44029.19	53505.4	44119.21	56165.1	49934.93	59724.78	75966.83
44983.27	43502.44	75873.08	38205.15	47919.93	45477.16	53503.46	45449.86	61483.88	83061.62

LP217.122	LP217.122	LP217.122	LP217.122	LP217.160	LP217.160	LP217.160	LP217.160	LP217.160	LP217.160
67727.41	41329.85	51622.84	64107.77	35051.35	29344.64	37100.71	32006.3	24487.13	36785.92
67287.11	41812.53	50510.76	65459	49270.87	48413.03	48612.97	41835	38916.87	54470.3
68527.29	47388.69	57648.65	73869.36	45093.52	43906.36	55392.74	51103.08	39321.29	43641.17
69705.72	47176.84	56761.67	75429.13	41702.34	32761.71	35458.07	28018.38	26064.61	35528.62
69685.7	43171.24	52858.15	72593.97	53102.57	40256.8	45399.97	41809.27	34369.39	55432.68

LP217.160	LP217.161	LP217.160	LP217.160	LP217.161	LP217.160	LP217.160	LP217.161	LP217.160	LP217.160
40163.01	33220.23	34391.35	30425.61	35131.07	30634.68	49532.69	30514.17	41347.58	45740.48
48885.59	51956.05	41819.41	50895.01	46695.11	30585.38	39587.01	31858.2	38150.03	47137.28
66846.2	41023.96	41667.39	35200.57	37273.4	32291.87	62820.02	46934.76	55038.25	58025.16
41540.16	36539.86	32621.51	28651.05	37996.87	30716.63	45212.97	37449.47	39805.67	47024.48
56563.14	40394.25	43258.49	40086.17	51261.28	40615.45	62587.34	48373.34	55414.16	63530.65

LP218.139	LP218.139	LP218.139	LP218.139	LP218.139	LP218.139	LP218.138	LP218.139	LP218.139	LP218.139
19136.22	19761.41	19409.02	17411.97	18336.86	30606.32	23008.5	22054.69	26166.21	22581.37
18145.92	21496.94	20427.74	19027.08	19691.37	29728	23455.03	22117.37	27925.14	23391.5
16491.14	20098.43	23172.59	21612.81	20704.37	33366.63	21962.6	24404.59	23831.28	27644.18
19004.69	19797.96	22528.42	20999.69	16747.54	33938.38	24876.71	21318.4	28811.11	27937.46
18982.86	20328.25	21893.92	19796.65	16138.18	34580.37	22102.21	22576.06	29056.24	23839.52

LP219.1378	LP219.1377	LP219.1378	LP219.1378	LP219.1377	LP219.1377	LP219.1378	LP219.1378	LP219.1377	LP219.1378
60020.77	48331.85	41406.14	43494.11	49753.47	34916.73	38972.8	31998.38	38009.98	34614.07
58275.36	48747.5	36429.62	44068.28	43144.4	32894.31	40299.91	38344.67	35450.76	37941.98
58333.3	49679.1	34126.47	46892.68	49265.1	34426.41	36172.46	33934.44	36143.24	41790.61
60402.19	39813.5	36929.18	48543.61	44825.53	35137.69	32421.39	35926.18	35590.3	32523.67
55175.62	43215.16	38061.1	44023.3	48134.85	35181.69	36628.1	36712.34	35692.8	35231.28

LP219.137	LP219.138	LP219.138	LP219.138	LP219.138	LP219.138	LP219.138	LP219.138	LP219.138	LP219.138
44211.8	54723.5	62618.86	53784.23	48260.81	47849.22	46594.73	42372.74	44037.06	53608.71
54929.38	53529.84	66779.68	53015.66	37800.23	48825.33	55413.15	42918.66	44387.37	44084.89
49917.3	62844.84	63825.22	55438.22	46194.65	46856.04	47486.02	47015.24	43432.78	48106.36
45273.37	53005.74	70843.91	70780.34	58479.36	45516.29	48770.48	43160.25	41616.8	40430.99
45628.66	81625.43	84486.48	47553.45	42347.44	53496.37	58051.87	38874.27	58359.17	48045.3

LP219.137	LP219.138	LP219.138	LP219.137	LP219.138	LP219.138	LP219.137	LP219.138	LP219.137	LP219.137
54867.07	49257.13	46341.18	40063.44	39510.14	51289.65	34986	28434.07	61840.89	43969.96
54616.38	44804.81	44938.43	40954.27	38668.5	49612.48	33217.24	32797.36	54637.42	41953.53
56331.1	42851.44	44616.37	40878.83	46178.48	48213.4	34435.38	30902.35	58989.97	47039.15
52880.95	42666.95	44243.04	38715.05	43110.44	50158.74	31802.27	30427.67	62171.6	42312.22
65198.35	47162.25	53207.3	38004.1	41538.36	45235.21	35806.92	33761.35	63455.09	47001.14

LP219.138	LP219.138	LP219.138	LP219.137	LP219.138	LP219.137	LP219.138	LP219.138	LP219.138	LP219.137
42885.18	49257.11	35325.85	33196.53	40376.18	40122.75	27176.07	35254.62	48333.87	64480.99
41808.09	47536.85	32353.2	35722.5	35057.53	44678.06	22167.25	32137.38	56175.15	56826.63
37974.01	45944.64	35182.18	34697.05	29707.84	40173.55	25261.67	34304.9	61720.33	64959.29
33362.95	50041.08	38894.34	35249.93	36606.63	50525.81	26946.57	35688.11	53709.41	62895.66
40080.56	53393.75	36565.29	29437.94	33462.37	45384.19	28530.77	35156.21	55430.16	66995.37

LP219.138	LP219.138	LP219.138	LP219.138	LP219.138	LP219.138	LP219.138	LP219.138	LP219.138	LP219.138
43519.78	44745.06	28115.74	45646.59	35895.53	54910.41	41605.73	40265.78	34594.25	26240.87
45393.79	46362.36	26283.65	48914.31	41755.37	51267.48	44340.9	37677.08	33640.29	25604.69
42661	40448.52	29179.7	52898.68	37290.25	53878.66	42949.27	46778.02	39309.27	27116.6
46290.84	41956.4	27186.85	51151.46	38373.19	51485.1	46313.9	40205.28	34474.92	24875.27
43513.82	41852.5	31020.07	50188.67	41606.02	55776.03	41078.03	37076.47	36077.9	27401.47

LP219.138	LP219.138	LP219.138	LP219.138	LP219.138	LP219.138	LP219.160	LP219.211	LP219.211	LP219.211
50550.99	39795.68	45765.44	45033.3	38130.33	43272.74	277184.3	337002.7	366922	295846.3
49610.13	39792.27	39463.18	54918.26	42358.13	40065.41	64563.62	316759.5	355372.1	273567.2
49861.31	42223.29	44661.59	48027.26	43269.32	40522.56	304033	281293.2	272018.7	227209.7
51226.61	37950.65	46471.98	52841.16	40659.14	42075.08	333069.6	33725.98	34257.36	38459.75
56749.65	40213.91	41184.33	53773.97	39152	44193.54	382410.2	329514.7	322803.6	279413.5

LP219.211	LP220.039:	LP220.039	LP220.039	LP220.039:	LP220.039	LP220.039:	LP220.177	LP220.177	LP220.177
267615.4	43011.95	33289.86	37751.87	29039.88	30455.63	35119.86	16932.52	18045.45	44870.28
264203.2	31870.94	25084.38	35917.84	26434.29	26127.51	36227.76	18319.2	20526.32	46096.99
256808.3	28407	27506.64	31747.14	32779.34	29223.49	38088.5	17835.54	20344.06	49258.66
57857.31	32382.21	26850.15	38697.26	24584.2	29578.61	39073.23	18878.04	23577.73	49332.02
353938.3	32541.89	22864.35	37276.85	30883.46	33755.04	40725.8	23987.65	29011.3	65964.29

LP220.177	LP220.177	LP220.177	LP220.177	LP220.177	LP220.178	LP220.178	LP220.178	LP220.178	LP220.177
25779.98	26672.75	52075.01	48117.16	54024.43	47005.9	40201.76	38100.26	37077.27	46161.77
27387.89	24873.57	54708.18	51021.96	61023.31	47954.02	40757.88	38695.62	43886.55	41253.56
30141.88	24245.21	57579.1	54372.73	49678.48	53150.37	41050.48	40351.33	34651.9	46866.02
29205.66	29287.66	59018.72	58293.15	51932.19	54053.5	47546.6	40979.34	43180.37	54145.45
37877.54	34917.18	70684.01	71080.25	69101.79	63233.08	56620.69	48541.75	51823.83	62455.29

LP220.178	LP220.178	LP220.178	LP220.178	LP220.178	LP220.178	LP220.178	LP220.178	LP220.178	LP220.178
43548.88	38419.64	56228.59	56668.74	58022.36	57867.54	52447.59	45402.47	45855.21	49520.2
43489.83	43255.95	68233.79	53680.89	59584.12	57430.7	53504.4	45219.64	44254.39	43111.85
46500.41	41511.22	72009.5	55470.66	58748.28	65752.51	50145.45	43779.66	44072.13	52735.85
46269.27	39406.99	73616.17	68044	60284.17	66551.73	54975.51	48772.4	55250.03	53785.08
55698.34	50799.38	91922.27	79044.71	80107.42	82350.75	70874.85	58603.53	52898.88	59551.25

LP220.178	LP220.178	LP220.178	LP220.178	LP220.178	LP220.178	LP220.178	LP220.178	LP220.178	LP220.178
56521.38	31745.56	43428.8	38774.21	28095.33	39669.57	36177.96	31701.43	42506.33	44117.68
52837.45	33786.11	41229.75	38754.16	29501.08	40096.39	30357.71	35218.76	44149.06	45441.86
57544.8	36125.15	44238.95	41858.78	28056.96	43211.22	33165.37	37987.72	49306.12	49364.78
60645.74	35784.65	50788.63	46069.91	31795.72	45575.17	40090.62	39152.44	51399.32	52782.17
77101.43	40562.14	57377.94	56637.2	35135.07	62778.47	43898.23	49335.63	65607.92	67083.69

LP220.178	LP220.178	LP220.177	LP220.177	LP220.178	LP220.178	LP220.178	LP220.178	LP220.179	LP220.179
36972.41	76893.9	48692.67	24538.21	72504.81	32059.12	39356.15	71089.45	43171.35	50096.16
34191.22	70998.87	50750.83	25549.43	70105.17	29689.25	44208.68	72545.88	43953.95	56143.55
39333.23	71671.83	45114.09	25474.69	67748.85	34711.02	45005	75054.63	42968.66	57413.84
41383.1	78749.3	51885.5	27346.29	77302.73	38356.19	49268.28	81115.73	51374.1	62844.5
45659.95	103053.3	62403.03	35116.6	87103.35	47468.87	65467.59	104503.5	60811.68	74683.94

LP220.178	LP220.178	LP220.179	LP220.178	LP220.178	LP220.179	LP220.178	LP220.178	LP220.177	LP220.178
45166.38	43426.18	68097.28	32179.43	32551.1	59832.32	31660.11	44917.86	23191.63	27490.99
36371.37	46837.66	61123.92	32219.73	31834.23	56053.93	32478.34	45490.22	19683.38	23269.33
42581.19	50480.42	67100.14	29880.59	33823.97	65943.28	34587	43641.27	22147.36	29041.7
44930.75	55005.97	69234.97	33862.32	37404.84	66993.01	42785.17	53920.32	22046.19	30461.4
55052.45	64389.91	81288.32	36800.37	44649.81	81542.37	51694.97	60722.73	28360.27	33709.65

LP220.178[illegible]	LP220.178[illegible]	LP220.178[illegible]	LP220.178[illegible]	LP220.178[illegible]	LP220.178[illegible]	LP220.178[illegible]	LP220.178	LP220.177[illegible]	LP220.177[illegible]
45657.91	32356.53	42777.63	44333.96	32186.27	57001.12	32210.66	36023.71	24842.61	36547
48286.78	32173.62	34002.81	52349.59	28468.11	61273.97	27663.48	40563.37	24515.77	36773.82
47967	34793.8	42946.66	54606.18	33965.57	58986.11	29518.79	43854.67	24709.09	36505.24
55783.72	33870.51	44989.64	53105.86	39972.75	73337.71	33657.05	43637.31	27476.42	39491.81
65473.63	39720.49	53589.15	64717.86	44473.35	84571.68	40985.36	50977.34	33934.56	51207.72

LP220.178	LP220.178	LP220.178	LP220.178	LP220.178	LP220.178	LP220.178	LP220.179	LP220.178	LP220.178
28478.99	30485.31	25113.11	37448.1	52327.36	27323.44	26286.53	51335.52	54786.09	46404.57
29499.49	30765.98	25035.43	33887.67	64230.1	28737.72	28555.82	50904.03	56254.57	48309.1
30362.54	35562.35	27725.66	34914.15	63656.16	31459.12	32546.06	56354.46	57620.32	50417.38
30947.6	36067.38	30003.5	39907.58	74691.15	31295.94	32254.96	59016.64	63849.87	55559.93
38528.52	41212.01	36470.29	49645.18	87091.31	38886.14	40619.8	72880.91	74317.85	66720.57

LP220.178	LP220.178	LP220.178	LP220.179	LP220.179	LP220.178	LP220.179	LP220.178	LP220.178	LP220.934
45677.77	52722.75	32843.85	42016.93	36421.29	30964.42	37612.53	35642.7	29668.42	96809.99
45718.93	52340.62	30547.09	42820.06	42167.29	32507.9	37159.05	32306.78	31590.68	88147.32
48351.12	60960.9	34184.48	41705.82	41009.97	34047.86	37755.88	33035.14	33140.08	95856.33
52362.46	67904.06	39551.19	49650.83	43413.82	37348.04	39455.31	40708.58	36041.17	86342.25
60504.25	80214.24	40224.19	60507.65	53442.21	47206.82	51476.77	56099.93	41897.62	95509.5

LP221.117	LP221.117	LP221.117	LP221.117	LP221.117	LP221.117	LP221.117	LP221.117	LP221.117	LP221.117
141305.9	44898	73196.91	36336.69	38813.66	47755.94	48261.09	45132.49	65482.67	55016.6
122607.6	51151.88	75703.99	31053.44	30810.98	55098.6	59421.76	34568.63	59416.4	53466.08
128610.3	44900.39	75453.93	27624.52	37843.35	58760.09	43596.11	40772.41	55469.91	61906.11
126774.8	61495.81	70908.72	47008.79	37147.17	62362.91	48515.6	37740.38	58026.94	50065.31
111591	53185.36	53546.87	40201.13	38664.97	54215.79	40066.68	53382.62	56173.47	57226.55

LP221.117[illegible]	LP221.117[illegible]	LP221.117[illegible]	LP221.117[illegible]	LP221.117[illegible]	LP221.117[illegible]	LP221.117[illegible]	LP221.117[illegible]	LP221.117[illegible]	LP221.117[illegible]
54798.31	64418.65	32977.69	40001.86	51508.59	60908.83	24781.93	39856.56	30686.77	46655.33
57800.53	68993.24	29002.86	37584.53	55775.79	67284.03	21981.05	36250.27	38073.9	46963.84
53236.94	57910.23	32410.4	39139.5	53104.09	67766.96	20972.7	36811.35	34338.24	42550.77
58415.59	57682.81	25691.55	37746.31	56502.98	65848.44	17442.18	37438.62	29982.88	45712.26
59530.64	57707.21	30298.54	38942.36	58244.82	65740.61	25432.8	37764.8	29236	42559.61

LP221.117	LP221.117	LP221.117	LP221.117	LP221.117	LP221.117	LP221.117	LP221.117	LP221.117	LP221.117
48397.34	67845.77	75675.95	70693.43	100710.9	49655.48	75137.98	56482.45	68842.22	67759.7
47429.64	77286.57	76096.95	77181.26	88535.48	57074.86	65368.49	60152.95	52771.07	50947.32
46029.62	69957.71	72540.37	95615.73	59304.49	53569.22	68993.82	63964.82	55535.54	54247.14
47415.42	76720.49	75806.38	80720.23	63426.63	58184.99	66013.39	73138.86	53247.49	53836.7
44840.8	75110.53	100213.1	84102.19	56018.78	56809.93	65397.11	66554.76	61610.83	61590.45

LP221.117	LP221.117	LP221.117	LP221.117	LP221.117	LP221.117	LP221.117	LP221.117	LP221.117	LP221.117
67082.95	73120.17	72955.87	52412.42	67043.19	51685.47	56110.05	71832.85	53626.45	44134.19
61140.45	55866.61	63273.95	45617.07	62530.16	45426.33	53009.95	50835.41	44323.3	47441.56
68660.72	64476.84	57257.87	47327.32	61214.71	49505.39	54215.16	50720.72	52192.15	42780.35
57979.47	62268.93	59198.16	54290.74	85618.53	54214.67	54429.87	49506.35	49119.18	44182.61
54644.71	54764.57	61417.74	54534.79	63875.31	52601.93	49192.69	53341.52	48408.54	45517.61

LP221.117	LP221.118	LP221.118	LP221.118	LP221.118	LP221.118	LP221.118	LP221.154	LP221.153	LP221.154
52444.09	65248.1	52898.24	37890.12	61435.51	51428.62	75016.78	48031.96	51638.85	22438.05
54784.94	62484.37	54060.99	39330.76	61990.26	60175.34	72405.55	44976.57	46268.22	22276.89
53530.59	59348.73	56747.69	39278.39	58766.6	49625.53	66884.61	46934.99	53053.06	24373.6
51967.03	64265.77	52329.01	34941.39	77078.43	54938.2	73483.37	49770.37	53719.89	19461.65
49890.32	58848.32	61150.71	38837.44	58661.58	51489.91	75723.41	50563.6	51809.87	21839.41

LP221.153	LP221.153	LP221.154	LP221.154	LP221.154	LP221.154	LP221.154	LP221.154	LP221.154	LP221.153
23749.58	33038.46	48939.09	30779.85	44228.78	34316.62	43309.13	42217.64	40702.95	35427.31
23995.67	35436.35	45422.73	35336.9	46546.54	32999.69	38302.81	47864.41	34113.76	32227.36
21934.27	34719.49	42954.48	37568.39	47878.92	35850.7	43844.79	46194.46	42724.4	31086.54
23725.24	33487.17	46555.39	33556.67	43926.41	32932.26	39352.5	47216.69	39929.41	33626.42
18116.31	33186.26	51573.65	32963.71	48644.23	37259.6	44671.87	47330.97	37977.17	37544.95

LP221.154	LP221.154	LP221.154	LP221.154	LP221.153	LP221.154	LP221.154	LP221.153	LP221.154	LP221.154
42276.96	22406.84	19362.26	23139.65	35756.05	36546.11	35612.21	43485.72	28011.14	36632.65
40587.18	22597.03	21690.21	22003.43	29571.24	30741.78	36753.63	40131.98	27703.16	36400.79
36448.8	26179.11	22840.39	22471.69	34447.7	36959.57	34960.02	40392.01	26996.92	38590.78
38115.24	29784.5	22310.49	22620.74	31346.3	37104.25	39748.15	41946.73	29213.71	35751.12
42603.46	22546.18	20345.45	19313.44	32104.9	35046.31	36896.1	41817.78	29511.38	38489.19

LP221.154	LP221.154	LP221.154	LP221.153	LP221.154	LP221.154	LP221.154	LP221.154	LP221.153	LP221.154
36711.81	41129.02	21278.29	31698.14	33399.52	46665.28	17729.18	34398.71	33643.29	44694.88
33213.95	39835.94	21158.38	33042.04	35441.97	46975.06	18785.88	34732.58	34193.65	44963.26
33379.18	41586.8	24211.75	35025.51	35868.57	45276.94	19757.55	36338.78	34322.3	44136.92
31796.04	39526.46	20706.42	37283.92	32745.01	44024.74	18592.97	35998.27	29815.62	44345.19
34003.44	40175.58	19964.58	33168.34	37365.46	50706.18	18083.5	33649.52	35003.65	46294.17

LP221.154	LP221.153	LP221.153	LP221.154	LP221.154	LP221.153	LP221.154	LP221.154	LP221.153	LP221.154
29886.81	28738.53	47769.84	45385.43	48149.39	28150.38	21335.51	36754.19	64487.48	28806.78
31769.5	25445.56	54648.25	40545.74	48897.89	30238.36	19407	38229.25	61633.85	28685.43
34782.7	27902.85	56590.22	44454.22	51580.08	28973.57	26142.96	37817.37	69322.69	27034.7
32134.77	26059.11	56902.24	42540.64	52074.01	26535.9	23404.31	41152.83	64208.07	26344.89
32279.88	29906.81	60736.32	47600.56	55600.02	28800.39	26141.27	41211.22	63126.13	27051.13

LP221.154	LP221.154	LP221.154	LP221.154	LP221.153	LP221.154	LP221.153	LP221.154	LP221.154	LP221.153
23918.66	35405.85	33896.26	34375.63	40227.25	35680.67	33711.12	48758.08	27494.4	28490.91
26184.98	36380.97	30577.87	37042.07	37956.11	37321.81	29710.67	47356.48	28173.68	27310.35
24110.76	39557.15	31775.65	34917.59	39087.03	41820.11	24420.1	50762.32	28763.73	30117.3
24963	32293.17	31347.63	40721.67	37031.27	39935.83	28971.21	48034.88	24791.12	30586.89
25047.28	36964.44	35311.65	39611.82	40515.72	40821.82	30582.22	56724.43	31213.94	26213.4

LP221.154	LP221.154	LP221.154	LP221.154	LP221.154	LP221.154	LP221.154	LP221.154	LP221.154	LP221.154
28929.29	59928.43	38258.49	43109.93	33068	60741.67	31346.98	43906.02	72359.29	29859.77
32138.24	57058.92	38170	41895.43	35684.84	60047.13	32283.79	38259.73	73012.09	40482.99
26333.74	53985.31	38302.28	43356.43	35539.77	66476.13	37100.69	43087.88	73964.66	36142.91
29907.39	58462.27	40085.18	42637.04	37680.8	63041.62	34481.79	43510.2	77004.55	35459.88
29863.87	64629.98	39703.72	47580.71	36080.54	64392.03	35208.71	43941.55	78875.39	34806.68

LP221.154	LP221.153	LP221.154	LP221.154	LP221.153	LP221.154	LP221.154	LP221.154	LP221.154	LP221.154
30906.23	63706.57	38806.32	27415.43	54615.58	43941.75	66218.13	48717.86	27892.92	49988.03
29421.79	59797.82	40972.49	26786.39	61582.71	42687.78	69332.44	49846.52	29708.97	58303.96
28806.01	67188.01	41102.3	24138.85	55760.64	44593.18	63823.53	53112.07	28384.73	53036.85
24819.07	63469.54	38659.22	29458.85	58195.46	43032.55	65432.57	52477.35	30115.06	53724.32
29502.28	61070.34	47503.66	24448.23	64875.13	45074.34	69999.23	54828.4	31436.11	59968.75

LP221.154	LP221.154	LP221.154	LP221.153	LP221.154	LP221.154	LP221.154	LP221.154	LP221.154	LP221.19_
30731.11	54839.74	24049.63	23494.94	71951.71	32648.3	37533.82	28694.26	47423.55	37441.94
27560.93	51736.21	26479.04	25977.06	67703.67	33453.59	40739.11	31539.03	43778.14	39294.57
28408.24	51436.24	25268.98	23376.28	74904.29	32048.91	39211.72	31817.58	43401.09	25229.02
25069.84	58533.72	24596.64	26343.47	61845.9	33664.62	37664.38	26281.73	47364.06	35707.56
27525.57	59820.47	25184.6	27239.11	68698.9	32741.13	39902.55	31421.06	45697.63	39546.82

LP221.190	LP221.190	LP221.19_	LP221.19_	LP221.190	LP221.190	LP221.190	LP221.190	LP221.190	LP221.190
38678.95	44677.25	66871.1	45699	29219.7	57423.45	37551.93	31128.25	39987.95	39594.78
40635.89	44489.66	61027.51	48084.85	30692.26	59395.91	35113.13	33961.65	35062	43086.16
28900.78	47056.12	43475.24	45423.61	32090.61	59513.39	35052.11	33608.1	35897.45	37141.31
43400.83	44205.57	68340.63	49228.29	29869.98	62235.82	34422.98	31607.28	41347.14	43060.57
46407.08	44291.18	54923.02	49326.89	31555.69	64519.01	35253.5	34150.57	40099.82	39023.8

LP221.226	LP222.112	LP222.112	LP222.112	LP222.112	LP222.112	LP222.112	LP222.112	LP222.112	LP222.112
33301.21	25198.82	25105.97	21698.58	17069.54	34469.38	24915.63	19932.48	26843.08	23404.41
37655.99	23580.17	24899.59	26871.51	24079.68	37933.85	29348.12	20331.19	32157.84	27693.81
29152.66	23626.6	23179.09	23947.64	22099.29	35572.32	27068.89	19346.06	28118.74	23921.04
32923.53	23547.75	24010.68	24156.49	20722.82	34038.55	29681.8	24960.71	30828.32	28240.66
27002.65	18578.61	21101.69	24361.57	24045.43	36841.26	27926.1	20925.65	32760.99	26432.8

LP222.112	LP222.112	LP222.112	LP222.112	LP222.112	LP222.112	LP222.112	LP222.112	LP222.112	LP222.112
20763.7	39456.69	33308.82	21311.76	36505.91	24494.27	23067.84	23152.28	26623.59	32300.37
18614.85	40943.4	29078.37	22221.82	36337.49	22926.88	24982.2	25543.86	22996.34	36466.39
20432.6	36651.93	28816.09	23362.25	37664.22	21949.99	28105.46	19939.43	25413.74	37288.09
21684.7	41240.34	26041.38	22464.82	35040.19	23677.69	24251.43	24106.84	26430.31	31419.6
20021.15	40383.37	28290.65	24074.58	33892.95	22909.3	25155.36	24933.74	26216.67	34011.17

LP222.1127	LP222.1128	LP222.1128	LP222.1127	LP222.1127	LP222.1128	LP222.1128	LP222.1128	LP222.1128	LP222.1129
21686.55	26054.43	26940.14	19989.35	17595.91	23936.97	17890.27	17692.99	15176.52	20438.76
22070.36	26387.84	30765.4	19615.03	23873.46	25047.44	16377.41	21508.81	19568.03	20191.09
21325.58	26215.6	31817.26	21069.22	20721.47	20460.2	18064.66	22848.23	17974.33	24530.95
22733.2	26462.81	30325.19	22118.94	25337.28	21580.8	17653.6	25300.43	22597.12	23366.2
20637.08	25411.41	29401.75	18758.64	19700.77	24513.43	15775.53	18941.45	18821.6	22653.35

LP222.112	LP222.112	LP222.113	LP222.113	LP222.112	LP222.120	LP222.149	LP222.148	LP222.149	LP222.149
24593.94	26920.61	26694.69	27456.62	19868.22	13700.18	31369.04	21044.84	20837.12	23467.88
23132.56	26341.5	27194.11	26676.61	18020.31	12975.67	36873.93	20623.91	16860.78	20635.49
21941.78	23364.9	23812.56	24032.68	22672.71	26910.32	35417.43	19714.48	17892.75	21066.26
25669.47	24904.82	28016.58	27235.31	22057.36	14019.46	36767.71	18643.29	15509.04	25724.72
25271.19	26294.77	24313.19	23760.7	21625.65	15939.3	36000.69	20506.51	19382.12	23824.49

LP223.063	LP223.063	LP223.063	LP223.063	LP223.063	LP223.096	LP223.096	LP223.096	LP223.096	LP223.097
42304.13	68415.02	66321.6	33015.71	64008.78	50154.63	47153.31	84298.03	96119.06	59308.74
49748.7	73049.31	64230.18	34769.71	67492.64	49146.95	63589.14	53375.84	62897.1	56735.66
50177.46	84268.42	72703.59	40031.72	70504.72	64341.16	69366.32	66788.52	89126.69	56376.44
54363.7	93669.52	71086.51	39531.52	66916.19	63760.6	89165.67	51837.29	53142.07	64429.75
57789.54	92987.29	67828.85	37258.11	71907.22	50192.84	55366.22	60140.15	70179.78	63925.19

LP223.096	LP223.096	LP223.096	LP223.096	LP223.096	LP223.096	LP223.096	LP223.096	LP223.096	LP223.096
70217.4	71699.08	55977.43	39830.08	56065.54	50329.72	41346.91	33519.37	46674.9	47749.65
44797.2	52526.85	68358.29	48152.73	55063.96	46192.05	54324.78	52370.67	52407.99	49269.54
54630.96	53440.97	62795.57	44737.26	55303.25	50629.75	61929.86	52923.88	51184.44	42523.4
51202.37	38583.67	74221.77	43654.77	57847.52	50031.3	54721.52	52305.85	56290.83	46115.33
45380.38	60100.04	67959.5	48463.88	59660.98	50800.41	53806.34	57655.53	35600.62	50771.76

LP223.096	LP223.096	LP223.097	LP223.096	LP223.097	LP223.096	LP223.096	LP223.096	LP223.096	LP223.097
41075.56	52347.33	63966.64	60205.24	48167.08	40716.01	46474.98	52045.3	55928.67	55381.78
40026.65	69609.63	63391.61	38224.13	50305.61	41452.03	52293.21	55517.08	51667.01	50600.32
43725.89	72445.13	63807.91	40106.89	52960.11	38688.62	45759.31	52785.16	61271.93	56837.02
42162.05	70526.26	68580.86	39458.86	41909.12	41596.63	50040.18	47733.13	59815.55	56754.77
44563.39	67912.65	64282.47	40293.54	45816.74	36587.53	50571.89	51853.66	59081.42	62084.27

LP223.096	LP223.095	LP223.096	LP223.095	LP223.096	LP223.096	LP223.095	LP223.096	LP223.096	LP223.096
41474.42	25386.28	36145.51	35063.99	43905.44	44834.15	32467	37815.23	60766.66	38579.92
49509.59	28037.06	36070.1	39938	42577.94	43063.26	33767.1	36127.8	44953.68	39664.06
45282.31	30667.75	36319	40997.93	44824.39	45272.08	45821.16	42644.2	50512.24	40350.31
39893.18	27684.14	37922.66	35155.11	45295.72	41706.58	34459.25	39393.75	50983.64	37593.26
41076.33	25663.52	37026.86	45210.91	41936.28	44591.78	34006.12	40335.42	46930.44	39378.89

LP223.096	LP223.096	LP223.096	LP223.097	LP223.096	LP223.095	LP223.096	LP223.096	LP223.096	LP223.095
37619.88	48484.15	38824.02	42803.82	53369.5	47994.4	25760.37	47159.72	40541.06	43862.82
36047.28	52187.59	37651.91	44586.86	59556.06	44771.83	29094.63	47448.45	44461.84	48064.39
35983.5	49951.6	37952.29	46333.78	63607.4	46429.71	27745.81	43857.29	40199.72	53480.18
47233.7	67642.58	37869.16	43140.89	60933.2	45147.76	27352.92	43285.55	42754.78	56233.34
41673.31	51699.62	38987.71	44351.2	60800.8	42262.42	29569.62	45622.9	40557.24	57810.74

LP223.096	LP223.096	LP223.095	LP223.095	LP223.095	LP223.096	LP223.096	LP223.096	LP223.096	LP223.111
43304.25	35367.11	36385.31	25338.34	31915.13	46124.77	37414.38	46197.27	37556.97	60125.45
44207.55	36226.96	33622.16	27082.61	32663.46	49923.39	42914.43	39602.7	38890.83	51623.95
45693	34323.84	38277.84	27751.49	32469.65	45643.9	44547.27	41272.96	40740.44	53394.55
46686.19	34646.84	32538.38	29046.77	33808.65	46408.59	38828.74	36237.2	40444.91	50311.11
51054.2	37243.36	36487	28652.93	34203.61	46395.01	39305.08	38570.21	42554.71	49780.82

LP223.112	LP223.132	LP223.132	LP223.132	LP223.132	LP223.132	LP223.132	LP223.133	LP223.133	LP223.132
76097.81	58887.85	45339.9	58288.13	35432.62	41277.38	43879.54	60959.95	45072.35	39073.33
68779.12	65418.74	48188.43	59513.57	33985.46	38492.18	39937.39	63339.74	43359.2	33395.86
68158.95	67056.64	49174.51	55871.3	39355.84	44096.8	49862.88	65501.03	43061.21	39670.12
65238.45	59990.97	49379.18	59063.75	28848.87	41395.33	40740.09	63489	47822.36	37603.79
67895.9	62205.88	45818.24	52499.38	34888.62	42679.65	40507.55	54461.54	45890.25	33513.11

LP223.132[illegible]	LP223.133[illegible]	LP223.133[illegible]	LP223.133[illegible]	LP223.132[illegible]	LP223.133[illegible]	LP223.132[illegible]	LP223.133	LP223.132[illegible]	LP223.132[illegible]
50086.5	60782.41	58330.68	34622.79	64891.93	65362.62	45990.03	45845.89	41525.88	83134.6
51492.7	60830.34	54566.01	36486.9	59141.41	61340.97	44370.6	47866.16	53631.43	77429.52
51727.46	56897.68	52119.73	37034.11	60909.3	66047.84	49123.16	51923.03	51564.18	73981.92
51604.77	58520.51	56605.24	37420.3	63249.68	63022.57	48132.24	47917.59	52112.26	80812.18
52800.36	64331.04	50009.13	39504.39	63228.83	72250.43	54179.93	49934.2	43494.38	88678.36

LP223.133	LP223.133	LP223.133	LP223.133	LP223.132	LP223.132	LP223.133	LP223.133	LP223.132	LP223.133
49164.51	58413.48	54158.06	45304.55	60654.16	53453.14	63833.82	62612.91	60801.99	37644.8
43595.81	60734.08	55847.59	41193.13	60424.69	58610.69	64725.2	57734.52	68164.92	39113.14
52854.46	52770.3	56951.08	42396.67	67126.64	57636.86	65041.16	62164.94	68007.04	38194.14
45239.62	53545.19	60527.06	42927.58	65535.37	57244.64	70448.33	61385.67	65057.19	39938.93
46360.03	62782.53	57491.52	48746.39	62259.09	58572.18	63861.8	60762.98	66579.24	37051.9

LP223.133	LP223.133	LP223.133	LP223.169	LP223.169	LP223.169	LP223.169	LP223.169	LP223.169	LP223.169
30834.81	60685.19	63525.5	79224.96	66252.31	69747.98	60326.54	78390.29	66132.68	79755.9
38314.69	63963.26	61799.86	77017.89	72863.82	63856.26	60323.03	81017.42	66201.55	74349.22
37991.96	61434.75	59463.73	74133.05	62858.41	113928	63585.16	81334.52	70301.8	84986.91
36410.74	60462.95	65124.46	85907.71	69255.52	65515.76	66254.1	78953.73	66106.81	86543.7
41022.91	56926.51	64804.45	81227.8	73067.17	70251.7	62680.06	85213.01	64776.2	80644.62

LP223.169	LP223.169	LP223.169	LP223.169	LP223.17_	LP223.169	LP223.169	LP223.169	LP223.17_	LP223.169
58661.77	66804.69	60365.73	62241.86	65728.77	72490.69	70379.55	70837.77	44978.75	60569.01
57455.47	66158.5	54715.12	57763.87	68635.66	72874.09	69414.15	70080.45	50417.57	60295.95
56729.67	67264.72	63275.16	63958.51	70399.24	69837.36	66628.07	74115.01	50563.65	63037.75
56827.01	70632.93	61901.67	57879.96	71061.06	69561.15	72690.95	67825.57	51392.91	61330.42
59801.04	71847.09	59818.28	65111.8	76171.55	78707.63	64820.11	71764.63	47230.95	64469.75

LP223.169	LP223.17_	LP223.17_	LP223.170	LP223.169	LP223.169	LP223.169	LP223.17_	LP223.170	LP223.169
65341.84	59952.74	50961.11	63625.36	61007.84	64752.52	72421.21	39712.9	57407.41	61832.27
70253.44	50954.33	62183.34	63863.84	60794.3	66636.86	68748.92	40748.73	66396.53	57635.94
70469.46	53700.7	62432.1	64771.21	65793.8	65120.01	66392.13	41283.95	65192.86	51949.66
67129.48	53174.39	67928.31	61124.64	58874.84	69174.24	74409.25	44782.74	64729.71	62804.45
65744.89	57346.91	67866.98	65220.81	63771.05	73438.54	76677.19	43803.01	65845.1	59618.93

LP223.169	LP223.169	LP223.169	LP223.17_	LP223.169	LP223.169	LP223.169	LP223.169	LP223.17_	LP223.169
71017.34	49443.07	61141.07	63240.46	68155.62	55777.01	88340.18	45478.2	59389.8	65530.01
69757.07	44645.06	71745.75	57745.08	66251.58	57459.67	96615.74	49005.21	60595.5	66203.19
67552.79	49896.82	64810.79	69577.82	72920.62	58279.34	95877.67	53914.1	62496.57	77916.36
70195.48	50278.92	61840.03	72136.26	71159.83	61082.54	90452.69	48354.6	56848.59	65470.33
72373.16	45086.32	65859.29	69161.49	71934.64	59721.73	94310.2	56101.02	61766.68	61952.66

LP223.169	LP223.170	LP223.169	LP223.169	LP223.169	LP223.170	LP223.169	LP223.169	LP223.169	LP223.17_
54950.94	58862.67	43511.91	40765.39	60043.41	38881.07	51727.48	56484.89	48937.3	54037.56
54050.55	60511.52	42589.38	37758.73	67317.1	33932.62	61070.23	54145.55	44375.68	50368.85
54420.99	61876.32	45923.88	37580.36	68768.61	35352.64	58418.04	57964.7	46311.67	51360.64
56470.76	63963.96	41924.4	41272.33	67432.74	35538.72	63120.63	52364.88	44934.75	52563.07
56209.83	57276.07	47391.22	42745.36	67415.21	37022.48	62049.02	58513.45	45414.61	55127.98

LP223.169	LP223.169	LP223.169	LP223.169	LP223.169	LP223.205	LP223.205	LP223.205	LP223.205	LP223.205
43101.37	44138.98	38504.26	29943.09	31476.68	37257.8	52899.11	40882.96	65850.99	75980.76
41100.96	38118.74	43783.87	30730.8	37881.21	60710.01	69487.98	51999.31	73463.88	95760.17
43495.12	35861.84	40419.47	32387.12	36395.03	67883.93	73773.05	53552.14	73164.91	97469.2
45908.02	40274	43143.59	30759.09	35259.74	66201.64	71501.77	56265.46	77613.17	102134.2
38098.37	37201.73	38131.06	30017.5	34521.24	67225.16	72735.86	54814.72	76811.68	95699.16

LP223.205	LP223.205	LP223.205	LP223.205	LP223.205	LP223.205	LP223.205	LP223.205	LP223.205	LP223.205
67117.09	46182.1	62111.44	73503.54	47407.42	55156.47	64151.61	56828.93	50347.95	73472.3
62835.73	45570.79	57166.38	72977.2	45977.64	46655.72	61543.38	56114.31	44722.08	71307.23
65733.49	42437.6	66245.81	78511.65	47534.34	50993.46	63397.59	55617.27	49116.85	64156.14
70956.26	44893.61	59981.07	74472.86	45864.25	51381.86	60638.19	60310.27	46441.3	60298.92
73369.26	45941.07	64521.2	76633.91	52039.18	50380.49	58511.58	60009.31	49472.74	71401.28

LP223.205	LP223.205	LP223.205	LP223.205	LP223.205	LP223.205	LP223.206	LP223.205	LP223.205	LP223.205
41647.6	84355.66	39574.76	51034.67	60008.87	54210.51	62368.84	54940.13	42816.09	43549.28
48760.36	85500.81	41438.98	46632.41	53053.18	52567.43	59650.22	56528.88	38749.65	51609.6
49169.83	87032.61	40281.58	45238.16	59660.84	55412.54	68356.71	50312.71	45642.7	52075.02
48713.27	87109.72	42863.35	51863.94	54954.49	51422.36	66659.01	55772.84	51917.95	50592.45
49635.23	88670.45	38078.94	48700.98	60871.16	51889.59	64964.65	62085.61	46484.06	54246.85

LP223.205	LP224.063	LP224.127	LP224.127	LP224.128	LP224.128	LP224.128	LP224.128	LP224.128	LP224.128
74867.31	147787.9	132854.9	95451.13	124934.2	117058.5	113798.8	103235.9	95665.84	113883.2
73900.09	175075.7	130227.6	93791.46	133691.9	121588.4	111331.5	107977.2	86189.85	111893.1
72720.24	155457.8	136527.8	97235.62	127917.7	145351.6	155462.8	87443.05	88677.37	126118.6
77062.38	155341.8	133076.7	96946.41	156415.3	111055.2	108328.3	102493	93893.78	120447.6
75692.37	147331.6	134377.4	92655.07	140550.7	116805.6	103500.7	98815.95	87517.9	113742.5

LP224.127	LP224.128	LP224.128	LP224.128	LP224.128	LP224.128	LP224.128	LP224.128	LP224.128	LP224.128
72999.35	38274.63	115234.5	28252.88	115890.9	136112.6	111597.4	34956.12	157113.7	156415.9
70147.46	44601.04	111303.3	28771.27	98392.41	143269	99637.66	35831.61	137769.3	152720.6
57996.41	28535.35	122283	41725.51	124846.3	190102.6	111515.9	30370.22	224895.9	103329
65888.62	37569.76	114721.7	25563.81	97560.82	135372.8	111075	36560.89	154073.5	162494.5
68121.58	35872.67	108338.5	26630.06	90509.79	140787.7	111196.2	30839.18	156295.4	157758.1

LP224.128	LP224.128	LP224.128	LP224.128	LP224.128	LP224.128	LP224.128	LP224.128	LP224.128	LP224.128
97190.89	127982.1	106605.9	59638.69	114380	66629	46874.57	113290.9	117723.7	166172.1
100544	138638.2	109034.2	67322.29	124783.1	65403.17	45578.59	119044.3	111054.2	170867.3
121318.1	127630.8	157288.9	90080.73	135065.8	73374.73	69720.42	133541.3	140614.1	173402.7
98973.37	131669	113090.3	63215.03	124667.3	61165.82	44083.38	125414.6	109475	174924.4
93548.09	119352	113567.9	67821.37	126684.3	60711.41	44030.81	110562.9	114556.1	168524.1

LP224.128	LP224.128	LP224.128	LP224.128	LP224.128	LP224.128	LP224.128	LP224.128	LP224.128	LP224.128
60662.09	96124.53	32972.47	68585.62	45478.69	110828	124443.7	109008.6	145545.3	80744.79
55504.75	94064	30309.37	74981.26	44678.1	123920.8	128738.6	104296.8	141381.3	74296.78
38931.69	90608.81	32671.75	107366.3	42075.93	126689.9	93656.65	111544.4	96382.61	71305.63
58393.16	83492.65	29775.01	64855.59	42751.09	107675.5	126444.7	103665.8	137801.8	74954.66
54293.69	84271.87	26652.05	61390.94	49106.27	109093.8	136162.1	108551.9	130769.4	77687.57

LP224.128	LP224.127	LP224.128	LP224.128	LP224.128	LP224.128	LP224.128	LP224.128	LP225.073	LP225.112
159522.8	115311.4	92404.07	117297.5	101042.3	115916	120779.7	112266.2	45576.17	53306.21
167307.9	118369.9	99109.64	115873.6	95996.47	117778.7	118414.7	130965.2	41489.77	55007.55
188965.5	116869.9	124652	117086	94506.8	114099.1	130040.7	117300	40455.68	50286.49
168668	114537.4	94280.15	119779.9	101833.7	109903	122956.8	117674.9	43632.58	50492.24
186489.7	105713.6	96508.65	123639.9	98518.48	115276.7	128429.7	106765.9	44275.11	59806.99

LP225.112	LP225.112	LP225.112	LP225.112	LP225.112	LP225.111	LP225.112	LP225.111	LP225.112	LP225.111
46096.2	45243.19	60571.77	55726.57	56286.29	44359.78	43220.99	60524.42	54363.04	45823.65
50151.76	43425.58	59721.23	49576.35	50896.64	46909.64	45755.53	49254.03	54713.57	45863.64
49395.39	43103.57	67147.35	43942.43	47918.24	48609.5	45074.11	55788.49	52046.98	44525.59
45510.29	50118.71	56491.48	43091.59	49477.56	44904.62	45233.33	53981.59	50959.4	42693.51
48215.03	46485.98	62432.14	42917.56	48155.9	47244.22	47803.9	53041.24	54889.45	52497.64

LP225.111	LP225.112	LP225.112	LP225.112	LP225.112	LP225.111	LP225.112	LP225.112	LP225.112	LP225.112
34756.86	63786.36	53894.71	60593.33	56857.11	45485.24	45455.43	49591.32	42670.16	39178.35
54004.95	65378.03	53756.86	64939.45	57698.61	43448.08	48700.19	48313.02	37408.33	45644.91
54527.19	65894.47	53572.08	62261.07	57887.72	45733.21	49729.83	51798.09	42839.04	44399.11
52568.7	60507.59	47854.98	68799.5	54840.55	48595.29	50392.44	48643.39	37979.17	37227.25
52807.8	62868.67	53177.22	65471.19	55798.88	46414.96	48219.79	47289.95	36382.09	39080.51

LP225.112	LP225.111	LP225.111	LP225.111	LP225.111	LP225.111	LP225.112	LP225.111	LP225.111	LP225.111
45567.77	50101.93	80959.99	62732.14	44769.57	35802.98	57035.13	40043.44	37482.25	60588.03
45266.37	45022.55	77658.98	61550.41	40535.15	32964.81	59459.1	35966.93	42879.57	54910.15
40647.64	46534.49	73786.56	59187.19	39755	34015.76	59217.52	40624.89	46675.08	47308.23
44600.8	41097.4	75957.79	54737.04	43386.68	26927.18	52594.59	39685.33	39948.53	59341.08
44531.1	45996.5	78124.85	55726.66	40158.71	23137.51	56895.3	36554.22	43255.31	51798.38

LP225.111	LP225.111	LP225.112	LP225.110	LP225.111	LP225.112	LP225.112	LP225.113	LP225.148	LP225.148
51286.01	20961.79	49980.57	48791.2	26969.96	54607.5	51291.41	61234.1	59601.07	45260.92
54273.65	23241.15	53226.78	53984.44	33521.2	54491.15	49893.79	61265.25	64219.66	32994.07
52329.37	29252.13	53302.3	55732.37	31600.73	52270.82	33218.48	61580.78	59675.54	38049.39
53975.39	22302.85	44839.02	53886.89	33057.47	52861.91	48900.58	58888.5	65842.56	53418.27
44964.87	25741.68	52124.57	52964.94	33941.9	54429.43	50611.78	60245.5	67607.29	50627.99

LP225.148	LP225.148	LP225.148	LP225.148	LP225.148	LP225.148	LP225.148	LP225.148	LP225.148	LP225.148
40456.55	45556.22	42364.77	66438.82	54130.75	47599.03	57434.27	60312.31	57375.82	54486.92
41539.83	45291.2	37434.18	56912.83	53190.76	42008.85	60427.96	62497.68	55778.03	54908.82
40846.47	38388.87	37466.75	55374.38	47533.94	42221.54	59413.8	59681.54	57798.31	50414.96
40200.44	46049.39	48090.67	70065.79	53277.69	51814.48	56730.67	71156.96	54083.64	52523.9
43090.92	48945.38	38640.71	62187.49	47651.24	49167.54	54435.28	63574.72	42588.18	60520.52

LP225.148	LP225.148	LP225.148	LP225.149	LP225.148	LP225.148	LP225.149	LP225.148	LP225.149	LP225.148
65420.3	65494.48	61520.78	39139.04	52705.44	38644.63	59503.43	54184.71	48297.71	50383.07
66312.34	60546.96	57585.62	43492.58	53045.53	38617.38	62419.4	49528.22	47149.18	55435.69
66329.77	60719.04	55964.17	41148.04	52025.42	39219.41	58906.92	47628.74	46341.78	54796.67
62259.15	56781.46	51507.54	39474.57	52226.04	34786.15	58277.81	47223.16	47493.48	51969.02
67398.87	62582.27	55147.2	43923.23	60481.3	36658.66	69947.55	50469.93	54349.66	50114.52

LP225.148	LP225.148	LP225.148	LP225.148	LP225.149	LP225.148	LP225.148	LP225.148	LP225.149	LP225.148
53631.38	34293.19	42275.31	57182.68	62652.95	59942.91	40482.24	50907.67	38346.13	78307.92
51966.81	34955.31	45652.48	52235.97	63336.49	52528.01	42301.14	47656.71	36969.52	78834.11
55514.22	33580.16	48688.79	52790.21	60359.8	55787.74	38600.04	48247.26	39427.83	81123.85
54247.19	37093.34	40029.26	55972.04	60925.91	56120.03	38808.67	52357.85	37893.33	78218.96
54063.88	32375.61	41862.38	57059.66	61563.4	54454.37	46563.29	46657.21	41032.02	83035.92

LP225.148	LP225.148	LP225.149	LP225.149	LP225.148	LP225.148	LP225.148	LP225.148	LP225.149	LP225.148
42608.98	48126.09	58392.43	72076.28	48709.67	58314.01	71225.21	45040.22	43877.88	49424.8
42512.79	44963.51	55229.07	70900.29	47373.54	53493.46	65507.24	39852.76	45278.59	46319.73
46888.71	45504.56	57448.42	70970.9	45889.53	54214.86	68455.89	46584.82	43807.17	48869.06
44398.86	47907.15	52666.94	72011.82	43269.89	56426.64	68759.94	46527.91	43447.68	52607.41
44161.85	48774.71	61873.81	84390.06	44099.87	61757.67	68084.85	43589.49	54680.62	47849.68

LP225.148	LP225.148	LP225.185	LP225.184	LP225.185	LP225.184	LP225.185	LP225.185	LP225.184	LP225.184
53053.7	56485	46429.28	63458.9	46387.25	60864.92	56007.99	48466.91	42104.07	52754.16
54516.2	57540.76	48957.82	62930.18	47536.55	59836.85	56463.22	36541.17	46520.92	48761.27
55449.14	55962.67	46999.26	60268.11	47274.54	58080.36	59930.58	40154.44	45849.94	49049.53
54387.39	62389.82	47950.46	63027.9	50588.2	60542.96	58544.97	43061.03	49995.24	52843.24
50910.97	59198.12	55803.39	65194.67	58300.9	66091.64	60144.93	49147.59	52022.66	56392.8

LP225.185	LP225.185	LP225.184	LP225.185	LP225.184	LP225.184	LP225.185	LP225.185	LP225.185	LP225.185
74711.42	85135.05	52228.02	44169.72	35062.26	55207.38	69080.82	32039.47	53403.37	52873.2
69464.9	82694.86	57095.06	43785.31	39404.91	52177.27	67785.29	29535.01	46006.74	56674.88
69420.46	83294.37	40078.54	44873.7	30807.9	34895.89	68454.59	33588.92	43039.04	43220.64
76271.28	85682.83	55862.08	41101.33	37555.11	51846.67	71486.89	30605.04	54250.54	56370.4
79241.75	84419.9	59158.83	49626.83	51011.07	59391.46	81078.2	32526.45	55806.46	66385.08

LP225.184	LP225.185	LP225.184	LP225.184	LP225.196	LP225.196	LP225.196	LP225.196	LP225.196	LP225.196
74335.02	57361.94	56581.17	34516.98	72311.77	80581.14	46527.42	87754.03	49678.12	67149.5
73033.63	61363.16	52466.28	31806.99	83544.19	51455.17	57023.13	39041.32	66989.14	43812.28
75690.83	47824.78	48733.68	26451.59	73117.68	62352.85	57862.03	60400.75	53317.56	50779.65
75146.01	56006.64	50822.81	34977.38	77122.3	50986.18	56262.32	65777.44	54930.82	55656.66
87401.82	64229.38	60017.59	36902.99	80510.17	57714.12	62736.35	76978.48	64893.58	64804.19

LP225.196	LP225.196	LP225.196	LP225.196	LP225.196	LP225.196	LP225.196	LP225.196	LP225.196	LP225.196
87486.57	48287.38	73864.66	52809.5	48456.7	52899.26	48633.25	67165.03	70763.62	84757.64
63861.18	65344.33	46494.57	58894.01	46391.89	72097.25	45703.48	63781.05	50432.75	44616.52
59320.35	49977.25	40250.67	35442.5	44266.58	56608.11	44451.34	51450.18	53435.46	47231.16
60783.83	54557.16	42209.76	38384.76	47734.54	56478.47	52860.69	51223.38	54384.54	48072.69
58772.29	58622.49	41489.32	38781.46	53845.23	60127.69	56615.84	54784.64	58968.99	52157.67

LP225.196	LP225.196	LP225.196	LP225.196	LP225.196	LP225.196	LP225.196	LP225.196	LP225.196	LP225.196
62113.28	57936.36	73084.13	75315.46	56427.86	44096.02	68918.71	71958.07	45022.73	46978.7
60278.41	49620.23	64585.91	69320.39	53273.85	42280.35	65681.19	62997.67	46271.75	43290.51
60356.71	48711.49	65608.1	63838.1	56304.01	40054.23	69750.94	63257.08	44370.35	45601.38
61028.2	57120.55	67156.88	66173.61	55287.66	47075.04	71817.58	68458.55	48179.38	43690.13
69397.69	60848.9	74747.38	73203.09	63436.69	49202.17	81468.7	73017.62	48943.06	49402.44

LP225.196	LP225.196	LP225.196	LP225.196	LP225.196	LP225.196	LP225.221	LP225.221	LP225.221	LP226.144
45270.72	52170.27	36715.55	42291.46	53412.33	60607.06	39100.64	90975.91	54250.98	29341.06
41293.43	48446.61	40901.04	38905.99	52272.99	56696.05	39161.36	85572.68	52855.43	34855.12
43766.95	52525.37	33780.82	32473.25	54828.84	60307.81	37969.47	83810.52	58765.87	34982.03
43620.63	55577.77	39978.43	40345.12	55975.15	65133.72	31785.2	87153.47	55233.53	29311.67
50827.21	62562.48	49778.38	39895.3	59287.61	71708.03	39082.04	85062.62	65405.51	29469.6

LP226.143	LP226.143	LP226.144	LP226.143	LP226.159	LP226.159	LP226.159	LP226.158	LP226.159	LP226.159
22843.49	32666.88	20400.79	16928.33	29113.05	43662.79	22007	32874.88	25474.48	56726.5
27001.62	32638.58	17308.16	18375.65	35599.95	42025.45	22773.2	30437	27001.68	54643.64
26864.24	33130.57	21871.22	16978.7	37540.47	45820.92	23301.61	29848.56	26066.16	51940.61
24246.28	30376.88	19069.36	15930.89	29991.81	44989.39	22246.76	35851.67	28873.94	54961.68
23164.25	31279.33	20269.26	15483.57	33190.38	46124.68	25813.5	35177.52	28702.47	56511.32

LP226.159	LP226.159	LP226.159	LP226.159	LP226.159	LP226.159	LP226.159	LP226.159	LP226.159	LP226.159
31958.9	31660.05	18630.54	21668.34	22369.43	37523.72	32735.04	22807.42	27166.79	15773.48
33878	33235.17	19432.42	20475.24	22000.93	42864.32	30638.91	21893.64	28828.46	15019.77
32307.45	31371.49	20995.61	20091.18	20626.07	41198.27	31929.33	23577.98	28178.19	14220.45
36117.83	32467.47	20276.85	18655.08	22641.95	40313.34	32114.53	25289.75	29902.39	16762.41
32367.76	37183.73	24443.05	25723.09	25411.14	39245.44	36377.12	22111.38	32270.53	19469.66

LP226.159	LP226.159	LP226.159	LP226.159	LP226.159	LP226.159	LP226.159	LP226.158	LP226.159	LP226.159
19901.45	39014.16	67424.54	27643.41	30936.02	44426.54	20963.18	44397.15	40328.92	42701.7
20868.95	38677.42	68057.43	28513.92	32684.28	46935.49	21233.31	41769.77	35824.96	41793.81
20549	41650	62944.98	29278.78	35786.68	41904.02	21699.16	43929.91	31712.22	41934.14
20584.09	39978.61	71260.9	31842.18	32228.63	41550.07	23496.66	46869.06	37398.77	40797.73
24268.9	39966.73	72601.3	33046.21	36603.57	47227.62	23119.87	42688.45	41591.38	43415.45

LP226.159	LP226.159	LP226.159	LP226.159	LP226.159	LP226.159	LP226.159	LP226.159	LP226.159	LP226.159
28187.95	27890.16	18135.48	54540.42	30632.54	22541.04	14972.79	29128.95	34876.88	39306.48
28054.4	31851.95	18703.74	53180.42	30631.5	20422.82	11836.8	30668	33924.76	32797.95
26844.45	28073.68	20265.18	54262.59	32010.76	25140.48	15088.7	28222.87	34088.13	31916.25
29166.65	28620.73	20276.32	55704.74	29692.25	21116.1	14945.77	28580.27	35381.64	35816.35
29187	29452.12	23726.48	49966.12	37396.07	25780.88	17777.23	33391.31	39626.11	43014.71

LP226.159	LP226.158	LP226.159	LP226.158	LP226.159	LP226.159	LP226.159	LP226.159	LP226.159	LP226.159
26184.13	31416.38	29162	27431.21	40874.96	54572.66	55106	25226.12	24716.34	38962.47
26363.59	38382.72	29650.56	28748.84	41611.93	52490.09	56325.98	26194.17	22074.27	34766.89
28887.34	35651.46	30313.09	30324.31	41400.81	52334.1	61585.48	28060.75	26993.14	32359.23
26654.69	33773.93	33410.44	31574.91	43679.83	48176.21	55982.82	29099.58	23569.27	39891.88
30614.46	38123.25	34022.23	33040.48	40787.73	53501.1	61109.62	31691.52	27043.77	34851.48

LP226.159	LP226.159	LP226.159	LP226.159	LP226.159	LP226.159	LP226.159	LP226.159	LP226.159	LP226.159
25548.85	39919.73	41481.82	44042.03	40477.64	32095.73	33916.06	38205.97	18934.11	19482.33
27166.53	40220.24	44369.04	43188.62	44029.5	34129.68	30633.19	33401.94	18827.58	18650.19
26696.19	37169.47	41277.66	46364.84	42608.76	36014.46	32365.04	40261.35	19159.64	19694.33
27720.09	37017.15	42311.8	42699.47	44947.66	31593.33	32514.84	36678.42	20056.24	20421.15
30959.11	38467.4	48705.43	47752.88	47358.74	35852.31	35826.84	44535.69	23300.57	20778.2

LP226.159	LP226.159	LP226.159	LP226.159	LP226.159	LP226.159	LP226.158	LP226.158	LP226.159	LP226.159
43194.89	53000.99	47185.67	15512.65	43708.03	40686.26	36153.71	39694	57911.55	56565.3
39845.17	50210.14	53358	15930.7	43915.59	39159.18	36827.47	41722.28	52898.8	50255.38
40766.68	53727.26	52744.97	14812.35	39181.68	44357.02	38115.93	38263.54	50971.25	53525.67
40425.05	50010.27	57025.32	15655.86	44635.45	42043.38	39949.56	39519.96	54471.9	55635.7
44723.89	50242.59	54012.59	15199.57	43457.13	40708.51	39831.48	40303.49	52486.81	50313.71

LP226.159	LP226.159	LP226.159	LP226.159	LP226.159	LP226.159	LP226.159	LP226.159	LP226.159	LP226.159
15747.15	52279.52	40653.57	24714.13	36796.71	44653.07	55075.9	31518.35	29910.16	39511.06
16766.24	50127.68	37074.65	25338.2	37736.08	44287.33	55164.38	32256.39	32038.27	44688.65
19739.41	49083.25	39031.77	26738.88	36798.67	46614.52	59854.24	35217.78	30819.6	42615.09
18010.73	54467.43	41061.26	27007.77	38159.78	43966.34	52647.68	39492.27	35106.52	46805.15
21326.45	52818.34	43709.34	31459.61	39500.27	47112.28	53914.8	39346.75	37364.46	44757.98

LP226.159	LP226.159	LP226.158	LP226.180	LP226.180	LP226.216	LP226.216	LP226.216	LP226.216	LP226.216
24322.26	30513.11	45046.57	43229.88	30168.28	49138.38	58129.64	60076.33	48268.95	60601.75
23668.14	28279.22	48982.06	39134.73	32249.53	52376.37	65596.5	45437.03	54732.82	61834.73
26554.2	30014.19	48474.92	41827.95	35795.83	49531.87	57373.43	72191.22	53928.67	54543.61
27024.9	31414.88	54588.57	47720.26	34873.41	47813.37	62508.65	46493.56	55524.22	48620.98
27364.42	33270.56	54937.44	43543.31	30221.1	58489.46	50864.32	59599.93	68347.15	63052.82

LP226.216	LP226.216	LP226.216	LP226.216	LP226.216	LP226.216	LP226.216	LP226.216	LP226.216	LP226.216
45442.71	56286.62	44891.72	58331.38	49158.19	51704.06	54373.49	50462.09	57212.12	34582.31
49126.9	53476.42	49943.74	56255.16	47678.84	49029.25	57287.88	52102.67	59722.22	33559.4
50197.65	43048.51	71783.73	72094.87	44813.41	43025.48	50311.88	49208.59	55675.91	37806.54
46332.06	48212.65	40126.76	61862.73	45484.62	51194.07	56148.88	57264.65	57630.31	33409.89
59730.19	79623.55	56143.12	57742.54	48553.41	52237.29	61276.86	60501.58	63146.07	35945.28

LP226.216[illegible]	LP226.217[illegible]	LP226.216[illegible]	LP226.216[illegible]	LP226.217[illegible]	LP226.216[illegible]	LP226.216[illegible]	LP226.216[illegible]	LP226.216[illegible]	LP226.216[illegible]
42617.04	34670.94	51207.34	34791.76	34085.41	36879.39	43395.12	38690.1	44981.35	40482.92
39031.92	34393.16	58176.21	34127.1	34781.84	39069.42	47710.6	34436.11	43710.3	43838.08
41656.37	29068.23	55297.7	37194.8	28541.84	32832.15	45952.08	37628.58	41512.33	47279.58
43792.18	32523.66	54346.93	34347.96	33718.09	42529.22	45711.4	37770.6	46050.41	43245.5
39425.14	33197.75	56505.39	36167.02	39711.67	45158.16	45127.52	34793.85	49874.91	39028.48

LP226.216	LP226.273	LP226.951	LP226.951	LP226.951	LP226.951	LP226.951	LP226.951	LP226.951	LP226.951
52001.54	29324.23	186832	167811.6	104386.1	210588.8	87900.78	206681	241515	224727.3
52045.3	26205.47	113217.1	137335	160738.9	286829.2	83546.76	182811.3	293331.9	188163.2
46429.11	24343.3	161729.8	153079.1	118004.5	255182.4	76525.54	234196.1	219680.2	219834.6
58912.09	21874.46	195784.8	191128.4	117325.4	254992.1	84403.25	211690.4	273833.2	278533.8
50522.99	22478.84	205113.8	140929.3	113535	183531.1	86952.77	246513.6	167794.2	279763.1

LP226.951	LP226.951	LP226.951	LP226.951	LP226.951	LP226.951	LP226.951	LP226.951	LP226.951	LP226.951
117743.3	191467.6	186698.2	220188.4	167950.4	117178.1	170899.7	149972.5	120462.7	160303.7
134900.4	230559.3	242890.5	250641.3	278874.9	137503.6	165456.4	181465.3	114127.2	199208.6
120970.1	200914.3	170497.7	229877.4	157586.2	156558.5	159472.3	143158.3	108325.3	131991.6
130928	156109.9	149628.9	179299.6	176143.7	113308.4	162880.9	192184.3	109073.8	199226
110774.1	291337.4	183335.8	224330.1	159408.1	118371.5	166854.1	112904.5	125751	144298.4

LP226.951	LP226.951	LP226.951	LP226.951	LP226.951	LP226.951	LP226.951	LP226.951	LP226.951	LP226.951
176414.4	210651.7	226018.7	146168.3	197215.3	84145.26	196544.1	179811.2	90226.91	86524.29
184346.6	277459.3	193811.6	160253.5	267690.7	95897.46	169639.6	141190.6	82989.86	97592.24
195546.4	213566.8	231036.5	150672.7	210093.8	89327.29	192807.2	200116.8	85421.13	73581.89
173476.7	307837.7	252651.7	152853.6	157527.9	105662.1	239489.9	205406	79908.72	81453.1
172698.4	204223.7	196800.9	141315.8	231918.7	93014.62	197674.3	214318.8	96031.79	85978.83

LP226.951	LP226.951	LP226.951	LP226.951	LP226.951	LP226.951	LP226.951	LP226.951	LP226.951	LP226.951
220572.8	206674.3	138867.8	121774.9	255577.7	108821.5	130247.9	66783.77	195888.3	180897.7
189771.4	215808.8	127165.5	168059.3	256118.3	144267.6	122248	72014.91	189381.8	170327.1
225796.6	195674.6	138050.6	141953.9	244328.3	105381.5	126839.8	68531.27	207886.4	175183.8
284779.6	144039.5	123012	164147.4	318022.7	124258.7	112270.6	56604.72	211136.8	179698.2
222810.3	198618.7	138237.1	149486.4	232792.3	111866.2	117299.1	64442.63	187389.5	182225.2

LP226.951	LP226.951	LP226.951	LP226.951	LP226.951	LP226.951	LP226.951	LP226.951	LP226.951	LP226.951
67940.35	228153.5	147840.5	197817	135882.3	226029.6	195750.2	108403.6	203882.9	81804
100371.5	219366.7	144644.8	174697.9	215544.8	264589.7	172545.3	84966.83	215480.2	117888.7
67248.21	210653.8	144874	185344.7	146575.4	224244.8	194277.1	111613.7	186336.6	75826.72
68862.7	221940.7	151615.9	192817.1	154105.7	235543.2	186808.6	91911.53	261296.9	103367.9
74719.8	197228.1	140952	189891.1	150615	287164	179565.6	98359.22	173545.2	79395.25

LP226.951	LP226.951	LP226.951	LP226.951	LP226.951	LP226.951	LP226.951	LP226.951	LP226.951	LP226.951
22554.15	201233	86223.54	187793.1	100047.4	26726.1	32588.89	149753.5	105577.7	21771.11
24853.98	191169.3	105272.4	199213.7	109843.6	18906.38	50965.05	168120.4	100877.3	28092.86
19973.97	189866.3	87999.37	194597.6	98904.07	21182.77	30423.7	162448.6	103234.8	20689.02
15649.71	284961.1	86092.74	177771.8	96826.01	17153.87	27465.74	144609.6	98984.84	17212.62
16148.26	200402.1	90254.31	200301.9	104148.8	23771.25	30073.79	145446.7	98683.76	20385.25

LP226.951	LP226.951	LP226.951	LP226.951	LP226.951	LP226.951	LP226.951	LP226.951	LP226.951	LP226.951
26558.21	161202.7	171189.7	98565.37	82256.15	22311.88	98099.98	22134.91	107852.1	28528.14
27958.17	164826.5	141322.3	151204.9	156870.3	16695.68	103905.7	17984.66	100490.1	43370.52
25115.99	170655.4	168368.5	99905.55	88907.9	18887.81	100521.8	19940.71	94994.59	26706.29
21400.39	179130.2	162510.6	97235.15	98498.39	16062.97	90405.24	17061.26	97756.35	22763.47
27124.96	167974.7	171295	102509.7	99397.3	20651.55	100376.5	19803.12	103446.3	27258.64

LP226.951	LP226.951	LP226.951	LP226.951	LP226.951	LP226.951	LP226.951	LP226.951	LP226.951	LP226.951
84629.61	178952.9	50031.73	108369.3	58219.01	125430.7	34013.96	120144.6	127143.4	30845.38
103862.4	253493.8	52248.25	145456.3	59885.64	120157	24323.3	136176.8	104179.6	31383.29
72468.45	188989.7	47309.68	109623.3	56482.27	123901.5	30063.95	107893	127564.8	29264.52
71905.93	192999.2	44527.62	109989.2	46270.84	116180.3	22265.43	123505.6	122377	24578.38
79752.19	190370.8	54268.49	114196.2	48615.71	113389.6	30166.3	128119.3	130701	31869.45

LP226.951	LP226.951	LP226.951	LP226.951	LP226.951	LP226.951	LP226.952	LP227.127	LP227.127	LP227.127
139821.8	196296.7	24046.83	24912.89	17073.94	195506.1	164614.6	76654.17	74579.78	93062.55
119845.2	192107.5	30093.24	26231.8	17959.26	186480.8	150057.8	79996.49	82571.63	84859.95
127742.3	176934	20491.49	23272.77	15195.53	192682	153610.4	86251.02	78034.55	85657.48
127849.4	185941	15111.91	20152.64	11449.2	189547.7	166181.4	85541.77	80634.89	88777.2
138974.8	189435.4	21746.4	27157.88	14475.23	189633.5	165228.3	87137.98	86208.33	97674.66

LP227.127	LP227.127	LP227.127	LP227.127	LP227.127	LP227.127	LP227.127	LP227.127	LP227.128	LP227.128
58474.09	91338.83	74588.6	86266.47	81265.82	90340.41	88548.83	92355.32	83430.01	118476.9
58655.97	95220.19	71408.66	83517.19	78154.5	85790.21	88864.77	130099.6	96923.53	93360.26
58571.73	93809.17	72781.37	87730.87	84122.36	95848.75	92802.02	107621.2	98656.58	97068.91
68076.43	97238.31	82440.96	91370.65	98075.42	98916.42	93763.96	106645.9	117209.5	94506.67
63493.3	83249.63	79074.76	76849.54	84146.89	86114.53	90423.52	142210.7	84417.1	134415.1

LP227.128	LP227.128	LP227.127	LP227.127	LP227.128	LP227.128	LP227.128	LP227.128	LP227.128	LP227.128
129557.7	73988.29	105792	109242.1	72830.45	96945.41	90923.21	111664.2	103495.4	123512.9
119819.6	82756.4	93514.92	102212.4	92333.77	87311.22	71370.15	89065.84	94879.51	108919.2
123470.7	74599.04	86778.05	101200.1	102272.3	90505.98	66414.76	92469.94	97759.12	105676.9
162927.5	82252.7	105921.7	85466.16	102050.1	81422.19	88715.81	80124.16	112940	163690.4
107960.7	89405.38	93101.85	108537.7	102314.8	78867.7	65231.03	101289.2	102662.6	106237.8

LP227.127[illegible]	LP227.127[illegible]	LP227.127[illegible]	LP227.128[illegible]	LP227.128[illegible]	LP227.127[illegible]	LP227.127[illegible]	LP227.127[illegible]	LP227.128[illegible]	LP227.128[illegible]
74036.57	77654	67428.93	121524.3	92391.38	95185.69	93930.2	91069.25	87782.48	109310.6
67970.12	92438.76	68207.74	122676.4	98621.88	98889.02	98434.8	80779.51	92672.63	110151.5
65408.01	89828.81	65685.18	114346.5	101657.1	97119.71	98869.94	86476.82	86659.97	103636.9
77939.47	92146.02	96862.94	77392.19	100425.9	118582	93684.49	85411.01	94228.1	123800.6
74078.72	97736.1	68948.25	129482	101924.4	89219.34	101860.5	96914.34	90192.33	99098.65

LP227.127	LP227.127	LP227.128	LP227.128	LP227.128	LP227.128	LP227.127	LP227.127	LP227.128	LP227.128
96972.58	108931.6	85593.18	81735.02	79286.41	77004.62	92772.58	182419.7	87731.19	113367.9
92392	103606.5	87806.73	90409.61	85197.42	70300.21	92657.03	182041.1	91458.45	109024.5
95179.36	109111.7	95251.61	88454.02	80573.37	64596.54	90686.15	181166	90464.41	112209.8
101561.5	120056.8	91410.17	95268.22	92926.14	82133.84	102273.3	192191.7	93595.69	116028.8
95432.12	104144.9	86463.98	96309.85	86788.02	73821.53	96495.15	187900.5	95630.2	113313.8

LP227.127	LP227.128	LP227.128	LP227.127	LP227.128	LP227.146	LP227.146	LP227.146	LP227.146	LP227.146
133368.8	84833.25	113858.5	89807.27	122695.5	90527.66	89631.34	92855.93	75074.58	100678.8
139688.9	78676.76	100870.7	81995.25	95640.56	86207.93	83246.36	81125.12	71155.67	102267.4
144105.8	81610.08	107852.4	83428.05	97073.16	88606.53	87045.53	83016.68	79761.24	109799.1
138568.3	86947.34	84416.76	69154.35	71716.5	90714.77	86303.19	82550.69	76460.21	102590.5
147998.9	84846.32	103147.2	78206.35	110249.5	76949.29	77722.73	60868.42	88584.37	95863.8

LP227.146[illegible]	LP227.147	LP227.146[illegible]	LP227.146[illegible]	LP227.146[illegible]	LP227.146[illegible]	LP227.147[illegible]	LP227.146[illegible]	LP227.147	LP227.147[illegible]
84626.07	119299.4	72198.07	75523.67	89913.31	63657.95	117916.3	72543.17	96891.5	77860.36
83437.51	123192.2	80214.64	76446.69	88260.76	60390.33	117380.4	79034.17	94858.82	76808.19
86175.79	108984.6	69705.2	78329.51	84322.57	65824.75	115218.3	68593.92	103309.7	74026.52
78358.36	125158.6	74134.03	79381.69	93328.61	71190.05	121563.8	75186.01	86317.01	82583.74
73201.33	108292.3	68378.58	87700.62	57710.41	66976.55	86566.44	69891.94	83018.11	77313.74

LP227.146	LP227.146	LP227.147	LP227.147	LP227.146	LP227.147	LP227.146	LP227.147	LP227.147	LP227.146
70786.24	98873.04	91944.59	63393.93	109605.1	88897.52	92572.14	95579.4	72600.49	91149.15
79409.85	106599.2	87970.24	57872.22	107392.8	81694.33	84018.67	99380.3	73578.96	93946.2
87072.07	116134.7	92865.67	70173.71	102249.3	86702.22	85687.45	105487.7	73905.19	93903.52
85426.83	105273.1	92864.09	70435.29	106916.4	85897.7	89150.59	106188.5	69125.68	92813.39
53857.02	113084.4	98858.21	57724.42	101547.1	76795.76	76450.6	89148.26	62682.33	79824.17

LP227.146	LP227.164	LP227.164	LP227.164	LP227.164	LP227.164	LP227.164	LP227.164	LP227.200	LP227.200
69783.56	75016.11	67454.5	58559.6	137767.4	112992.3	52148.28	94692.13	106286.2	58803.07
67896.02	59696.57	70320.73	66338.95	136920.7	117390.2	41744.1	99877.8	66856.34	63043.21
67403.59	68118.11	74667.19	58519.93	144325.9	120054.8	38631.76	94997.8	63700.19	64186.87
70083.82	70058.74	65763.73	49164.84	155545.5	117631.3	49320.29	98112.13	56613.94	65840.3
65852.7	73349.86	76265.56	67414.21	158320.6	125453.6	46622.45	98031.79	79235.35	69859.29

LP227.200	LP227.200	LP227.201	LP227.200	LP227.201	LP227.200	LP227.200	LP227.200	LP227.200	LP227.200
72730.71	78488.19	66658.88	75220.64	90895.55	85062.64	100448.2	70654.07	75608.69	75495.64
82176.6	74154.35	66296.9	73352.05	75468.09	72715.06	84026.51	75643.47	82398.97	61622.02
77011.47	74792.93	63007.09	75045.44	85376.75	69578.47	88313.28	82603.35	76489.66	68785.01
77680.25	75172.38	65835.1	76328.38	90144.46	81573.04	89578.74	82417.71	73335.69	75816.26
82207.01	80831.97	72198.43	73736.51	93181.26	86181.79	87779.54	78899.26	75026.31	65965.83

LP227.200	LP227.200	LP227.200	LP227.200	LP227.201	LP227.200	LP227.201	LP227.200	LP227.200	LP227.200
85911.61	80682.33	95845.72	89339.16	74266.14	90461.83	59785.87	79537.41	60729.77	80584.74
88740.71	82309.92	95891.55	86021.28	76325.52	84705.73	67739.87	88090.13	54361.41	75762.64
89176.16	78181.16	103383.9	87366.52	72738.55	80227.13	61770.46	85785.53	53647.85	84271.87
87950.21	81672.29	95287.1	84908.85	81017.59	83991.76	68696.84	85305.47	58423.08	76794.06
94623.84	76439.51	101794.3	88476.22	84221.87	93658.48	73606.61	84215.28	56508.52	85952.38

LP227.200[illegible]	LP227.200[illegible]	LP227.200[illegible]	LP227.200[illegible]	LP227.200	LP227.200[illegible]	LP227.200[illegible]	LP227.200[illegible]	LP227.201[illegible]	LP227.200[illegible]
51817.62	61914.93	66770.18	85967.02	54108.17	36108.22	84648.57	66009.46	42207.17	118534.2
49787.15	51451.12	73257.22	78979.07	50467.77	35021.13	84703.7	66526.26	49650.97	104648.4
50904.44	56149.49	68002.51	89338.57	53902.26	39483.96	85196.41	61620.24	47608.82	104253.1
51089.47	59399.04	75318.51	84972.85	55067.36	39511.04	82434.42	62422.11	47629.72	120074.6
55404.56	60889.32	73162.29	96889.18	55630.87	39382.73	97564.45	72298.93	57523.51	127859.9

LP227.200	LP227.200	LP227.200	LP227.201	LP227.200	LP227.200	LP227.200	LP227.200	LP227.200	LP227.200
58322.03	89534.22	57449.61	58538.4	52509.81	65920.76	51573.68	38166.67	56133.83	75758.01
63302.7	86864.12	56856.79	56270.09	48178.1	72804.42	51164.28	41265.49	49630.64	74557.5
65365.45	94313.67	62519.15	57338.39	54402.74	73560.39	49315.72	41417.92	54828.11	85339.56
63854.86	84789.33	61247.21	54032.27	57157.43	65347.14	54918.44	40933.19	53518.31	80554.54
73397.96	92750.12	61584.92	60027.64	52854.74	69633.87	50002.91	42558.85	57151.21	85323.98

LP227.200	LP227.201	LP227.200	LP227.200	LP227.200	LP227.200	LP227.200	LP228.105	LP228.105	LP228.105
52440.69	55339.29	47412.92	88858.38	52087.8	38914.9	45157.55	399834.6	384333.7	400977
50495.74	57204.19	48257.28	83469.88	52302.35	36761.6	45705.65	357184.1	365403	518604
55817.28	50170.55	39468.15	91571.94	50882.57	36389.39	48518.66	377495.4	319954.8	467192.1
55181.66	56983.81	42257.63	89598.25	54052.01	36348.64	45588.88	402805	301775.8	493990.7
57463.32	61593.68	48097.41	92297.71	50232.64	37827.19	52295.77	446634.5	344713	557594.4

LP228.105	LP228.105	LP228.105	LP228.105	LP228.105	LP228.104	LP228.105	LP228.104	LP228.105	LP228.104
212790.6	349640.3	229327.5	315422.9	95528.82	54981.42	174745	73226.49	89212.28	83965.42
287878.1	412261.4	174032.4	221427.1	73997.46	49352.73	158570.4	71158.4	85520.83	76171.22
293154.3	384639.3	223601.5	209728.1	88356.6	55944.29	129441	81361.23	86311.67	70657.51
262921.3	403005.7	161812.2	249123.7	74874.84	64963.5	98901.97	65993.85	67465.05	67321.1
320899.5	504685	155730.3	249694.2	80231.3	55746.66	152501.6	70598.94	88574.37	117240.9

LP228.105	LP228.105	LP228.105	LP228.105	LP228.105	LP228.105	LP228.105	LP228.105	LP228.104	LP228.104
102370.2	175220.3	139549.7	146834.5	132991.9	175527.2	83071.68	149066.2	45293.96	85772.01
103793.1	174788.8	129076.4	174789.5	122645.1	173679.9	81624.78	136092.1	45506.39	92190.19
100071.4	180128.9	127483.8	153970.7	125191.3	172526	80916.8	142254.2	47212.19	93486.45
97683.6	183998.5	138228.5	157519.9	128835.3	178323.2	87061.12	137435.1	45429.26	91035.27
106285	161160.4	133458.4	160589	147849.5	176801.6	86403.21	173864	46808.15	92421.08

LP228.105	LP228.105	LP228.159	LP228.159	LP228.159	LP228.159	LP228.196	LP228.196	LP228.232	LP228.232
152108	118715.4	25846.49	29145.1	31911.12	42497.45	60029.17	26592.84	41356.54	25703.81
136399.2	123480	26263.52	32527.85	32196.97	43019.48	60682.98	23961.01	38793.24	26884.77
124843.9	120421.9	29487.61	31417.32	24896.37	46484.63	58189.28	23238.34	38813.49	27114.07
123340.5	122543.2	29115.23	34334.17	31714.8	46247.23	55198.15	23057.89	33726.87	27859.04
141746.8	124101.1	20353.46	23747.32	35526.24	29576.01	59479.82	19919.84	35949.38	33552.75

LP229.106[illegible]	LP229.107[illegible]	LP229.108[illegible]	LP229.142[illegible]	LP229.143[illegible]	LP229.143[illegible]	LP229.143[illegible]	LP229.143[illegible]	LP229.143[illegible]	LP229.143[illegible]
68377.78	62556.09	46380.29	67771.85	63514.21	49565.6	64354.57	65075.42	72437.08	72694.1
61559.72	71667.68	50724.69	62860.27	67026.41	58029.6	74897.57	71991.75	50690.57	74645.79
59023.54	70757.62	47181.96	73721.96	75003.37	53531.39	66070.34	64943.23	65278.67	74363.15
50071.68	41509.57	47316.11	69751.47	66160.44	59423.56	65075.71	63676.61	51820.52	69777.28
60163.19	74682.32	46589.38	76929.8	66117.73	66082.05	69109.97	64280.86	43848.09	68950.45

LP229.143	LP229.143	LP229.143	LP229.143	LP229.143	LP229.143	LP229.143	LP229.143	LP229.143	LP229.143
75731.71	61222.47	54447.84	44142.04	45661.79	74737.72	68450.33	59994.75	48362.66	49604.6
73243.3	62316.32	56146.53	48744	49080.1	77093.71	69364.35	60906.84	56613.93	44413.7
92525.41	61951.56	62342.41	46835.7	55720.24	108433.2	73386.96	67475.45	50887.74	45639.52
72263.69	59811.91	67890.5	44459.63	49391.39	67294.45	70716.45	52111.22	50433.13	53161.57
77827.54	63914.67	60577.54	49725.08	68046.52	79777.29	75828.85	59441.19	53865.63	54066.53

LP229.143	LP229.143	LP229.143	LP229.143	LP229.143	LP229.143	LP229.143	LP229.143	LP229.143	LP229.1437
33574.35	58436.07	62610.8	55027.1	68516.84	45093.44	56204.6	67912.24	51311.22	41755.28
50035.16	61134.62	65100.45	59252.93	64175.94	46022.65	59920.87	68853.01	54385.55	36637.77
51398.46	76451.97	61616.4	56991.36	68372.14	51933.36	72374.88	65622.03	56256.01	40422.36
46806.64	67102.09	56321.11	53412.1	63007.11	43895.75	62365.26	59273.45	52747.09	40953.3
48313.87	62933.5	69169.84	46551.68	69013.61	43641.11	56085.75	62054.49	60609.46	35816.77

LP229.143	LP229.145	LP229.144	LP229.143	LP229.143	LP229.143	LP229.143	LP229.143	LP229.143	LP229.143
34479.26	50967.28	50643.88	49028.09	62030.34	69440.35	47413.03	58191.28	53714.32	72984.63
32468.22	53786.71	50538.16	55229.26	60019.71	70601.65	47571.74	65613.96	51984.08	70655.26
34704.76	50299.7	46892.66	55799.13	65908.19	66254.58	46005.26	55991.92	57936.37	69600.49
36556.8	52950.69	51882.4	54128.15	62178.94	71142.38	51924.63	59243.58	54402.93	65667.14
40839.87	61273.04	50898.58	49616.86	64456.25	78306.27	49171.22	55148.36	50969.99	64412.45

LP229.143	LP229.143	LP229.144	LP229.143	LP229.143	LP229.144	LP229.143	LP229.143	LP229.143	LP229.143
46046.51	53493.14	33343.68	46289.28	52422.53	73166.31	34415.29	80274.26	48964.27	95527.02
43329.58	46206.96	36235.02	38264.02	47146.39	65303.76	38455.92	86314.94	50832.55	83983.28
52044.81	43611.6	35679.72	45478.74	49381.33	66574.59	37832.52	80405.82	52400.47	94346.18
48873.25	47952.02	33335.15	45840.91	46148.33	71492.12	42399.35	76263.75	52810.23	85131.25
46442.35	45483.21	30287.23	40432.19	50514.55	63124.13	34956.89	80778.51	47721.1	89122.5

LP229.162	LP229.162	LP229.162	LP229.162	LP229.162	LP229.162	LP229.162	LP229.162	LP229.162	LP229.162
59952.4	83876.46	96038.14	72424.67	60949.31	87229.28	97697.4	90828.06	87437.3	54912.43
65308.9	78508.11	84752.16	80704.51	69256.14	77964.41	104842.5	82419.19	75700.64	58778.11
58683.2	80600.81	99990.41	70128.16	75257.11	86281.97	98601.93	98543.06	88648.97	55038.85
63876.99	84729.54	102856.4	84251.18	70254.35	88210.14	102727.2	98403.74	88829.27	60228.89
86645.86	115679.5	123427.2	100772.8	93357.7	108986.9	124790.6	120202.9	113592	83307.84

LP229.162	LP229.162	LP229.162	LP229.162	LP229.162	LP229.162	LP229.162	LP229.163	LP229.162	LP229.163
85763.86	56770.58	68752.93	82380.32	43463.79	85844.58	85804.05	88231.16	53842.9	70261.74
92322.41	60785.5	63263.05	84063.15	47765.62	86235.76	83949.69	72534.55	58852.39	68646.84
98955.59	58594.79	64118.38	77417.67	51718.11	91282.5	76866.07	79176.43	59536.38	67640.63
101470.3	59906.51	72249.64	96617.97	52513.98	87849.64	85080.53	84076.45	60353.13	73854.14
126101.5	84008.68	100270.7	124015.3	69882.55	109297.8	105498.9	108599.7	77080.64	89914.28

LP229.162	LP229.163	LP229.163	LP229.163	LP229.162	LP229.162	LP229.162	LP229.162	LP229.162	LP229.162
70929.71	76210.74	54045.88	58551.6	81527.43	78973.65	102365.2	76062.82	60129.05	79486.14
78447.83	66594.9	50990.92	60327.87	74736.32	82371.74	85898.8	70108.11	59271.72	69108.08
84483.34	67566.95	57172.04	57112.18	72256.27	87847.4	101548.5	71156.95	60804.75	68121.92
79060.25	73255.73	58903.66	63484.85	77348.53	89236.8	107960.3	74409.43	64075.99	68276.31
114000	88593.59	74584.2	79502.38	98668.51	117545.5	135493.5	86347.22	87530.62	89085.64

LP229.163	LP229.163	LP229.162	LP229.163	LP229.162	LP229.162	LP229.162	LP229.163	LP229.162	LP229.163
81923.11	111835.8	76464.49	88814.29	63859.85	81705.85	131116.3	61934.94	77482.48	60577.96
80164.06	111092.2	73092.48	93555.4	65189.55	77210.47	126466.5	68731.33	87896.89	60388.47
83962.15	119111.7	72233.61	91276.2	63955.72	78617.36	122767.6	65542.48	84898.24	69265.71
90397.54	118581.6	76264.87	93969.05	66743.17	85787.06	140026.7	69059.4	82748.5	61235.28
109880.2	155066.4	108087.7	119567.5	85494.97	97387.94	167125.3	91052.8	118240	81410.09

LP229.163_	LP229.18_	LP229.179	LP229.199	LP229.199	LP230.139	LP230.210	LP231.122	LP231.123	LP231.122
61597.13	89885.88	85575.79	51881.64	52186.49	38722.26	18425.86	24690.73	31205.04	37649.86
57861.53	82519.33	87607.71	51234.41	49228.31	40446.55	15860.33	17749.5	31142.19	46213.5
62972.11	90273.11	90752.02	50003.91	52799.17	40659.96	28453.05	23068.96	32162.94	38710.15
62741.66	102226.3	99505.49	56319.95	58329.55	45972.06	19630.05	20383.25	29606.96	39014.97
79582.06	123069.2	116413.8	69380.17	68950.31	37797.82	18934.85	19161.92	29913.24	43385.45

LP231.177	LP231.177	LP231.177	LP231.175	LP231.178	LP231.178	LP231.178	LP231.178	LP231.178	LP231.178
67546.94	101618.3	49814.29	55495.13	44796.66	56179.38	53684.74	52537.29	51191.85	56201.57
68356.87	95022.85	49950.64	48276.86	47656.24	49728.79	46896.16	62880.99	49100.32	52605.32
66800.12	94377.13	51311.7	53880.52	51217.01	43903.06	42477.58	68580.76	50940.72	57472.89
80824.75	106489.1	59688.68	52850.66	47111	48641.86	49029.99	66524.16	55940.37	62201.91
107182.1	126854.6	70698.25	76681.34	65424.46	94007.87	108356.8	83927.95	60195.57	89999.67

LP231.178	LP231.178	LP231.178	LP231.178	LP231.178	LP231.178	LP231.178	LP231.178	LP231.178	LP231.178
45483	62896.82	61460.4	56332.5	56226.94	65933.3	61588.72	71883.98	55780.98	57593.84
47448.77	58866.69	61314.67	48492.68	54453.5	66562.14	66290.3	58727.77	53317.47	56526.69
42032.43	63756.78	58651.51	53416.99	56089.4	66182.02	67098.92	62881.92	55093.37	64810.98
49798.78	65398.04	64335.52	58452.86	60542.83	75404.22	69973.79	65339.25	55309.03	63966.71
60106.77	106141.5	71636.06	64838.04	76599.98	77608.09	73291.39	78944.8	75299.96	86726.23

LP231.178	LP231.178	LP231.178	LP231.177	LP231.178	LP231.178	LP231.178	LP231.178	LP231.178	LP231.178
52413.03	84907.66	57175.64	46859.04	45513.04	40735.7	57829.05	46661.59	49125.34	52222.79
60628.89	84649.75	61005.06	48652.87	41300.94	49367.34	56212.43	50022.87	52603.47	49304.53
60211.2	85008.61	63519.73	49931.8	48278.64	48987.53	62767.93	54047.08	62873.85	51724.55
58223.89	95804.21	66608.35	57803.51	47431.5	49061.55	65852.95	55612.4	64831.65	58620.86
98941.36	112829.5	92612.02	72566.95	63972.75	66790.44	81781.65	66844.89	75295.25	74436.41

LP231.178	LP231.178	LP231.179	LP231.178	LP231.178	LP231.178	LP231.178	LP231.177	LP231.178	LP231.178
51398	59738.09	40677.03	71371.39	59769.77	69253.13	44083.72	43219.85	44440.03	69673.67
46720.9	55745.32	43464.37	70738.36	61822.33	63194.45	43468.07	42811.14	42196.08	71578.68
49474.63	59661.33	50112.56	66954.82	59535.38	70843.67	44458.97	44450.39	41575.37	73282.01
55695.12	65078.52	47376.21	69280.54	57867.55	74299.54	50014.98	46489.42	54316.26	79112.2
73720.36	80833.5	55377.28	90982.88	81030.42	91120.04	58217.22	62798.36	58201.68	92230.94

LP231.178	LP232.153	LP233.153	LP233.154	LP233.153	LP233.153	LP233.19_	LP233.190	LP233.226	LP233.226
47720	27623.21	29405.45	24663.76	31911.46	37573.91	36889.57	29485.02	31102.26	28770.08
44505.41	26728.99	27962.44	26376.84	36252.94	34988.42	35870.2	31485.8	21647.75	21627.17
49612.88	24943.79	25586.42	26791.69	38633.22	41190.97	31938.29	22990.56	37469.85	32485.27
50958.04	27944.07	27406.18	25234.39	38084.4	41786.53	41955.18	26743.35	37104.83	32077.13
66198.37	25243.88	26300.41	25600.55	34791.24	35034.84	37230.68	29840.57	30156.9	35979.75

LP233.226	LP233.226	LP235.132	LP235.132	LP235.132	LP235.133	LP235.133	LP235.133	LP235.132	LP235.133
27215.84	27732.71	49036.06	45454.16	65418.78	62776.79	57735.07	37238.47	45600.4	28260.72
23378.07	29149.58	47010.5	44220.55	38596.25	56892.15	59834.62	46755.6	45490.39	31507.58
25274.8	34294.73	46781.94	48386.08	58646.46	77161.48	57395.71	43579.49	53126.08	35919.79
23626.57	31040	43456.44	39274.68	47106.08	57596.37	58479.32	46323.01	40998.2	32383.71
29643.86	27965.62	46265.02	44639.55	42944.38	63268.97	57228.16	49130.03	47179.54	34435.99

LP235.1329	LP235.133	LP235.133	LP235.133	LP235.1328	LP235.133	LP235.133	LP235.1329	LP235.1329	LP235.133
34429.08	44539.78	27437.22	31766.64	49303.72	34672.31	31048.83	49563.16	42428.75	38167.14
34557.06	36501.48	24962.96	36950.43	49945.28	30030.81	31278.63	52226.57	42653.05	34039.45
32145.67	40399.76	33564.66	36374.64	50039.68	36818.17	30095.34	55652.54	43828.09	34843.96
29643.46	42138.38	28260.86	33492.48	50987.28	32698.26	31088.04	50244.08	44437.25	32883.66
34129.32	43173.47	30544.12	35118.54	49266.75	31425.96	28325.1	53249.62	39557.17	33873.07

LP235.133	LP235.132	LP235.132	LP235.132	LP235.132	LP235.133	LP235.132	LP235.133	LP235.133	LP235.133
38729.67	47567.05	48364.77	36184.81	44310.64	34794.05	48508.51	43518.59	31712.73	33947.75
36874.92	44132.84	42994.66	37579.95	46277.25	36981.53	43012.26	40489.55	31465.29	33249.91
43370.97	38548.8	49989.38	41697.31	43780.84	40818.9	49241.32	45425.86	31466.75	39139.87
37911.41	46811.05	49702.67	37471.19	46185.92	40311.52	42432.4	33302	34760.77	34102.49
36070.72	44631.37	43227.12	36129.45	47261.38	35113.56	41584.97	39487.38	35065.61	32837.89

LP235.132	LP235.169	LP235.169	LP235.169	LP235.169	LP235.169	LP235.169	LP235.169	LP235.169	LP235.169
26518.95	148156.9	129766.3	149003.5	133698.1	202965.8	211980.5	151434	169662.6	181231.4
28987.98	140841.8	114843	138519.7	136614.6	200060.5	194436.1	139312.4	174360.7	166228.5
29814.83	149040.9	119310.9	141318.4	141849.2	204428.4	215057	142093.2	172200.7	181888.9
32127.13	144412	127768.5	152805.9	148701	213824	230307.4	149784.4	192615.3	172951.4
28748.11	181126	168326.3	167284.9	176229.4	263948	261726.7	184420.1	227799.9	228798.8

LP235.169[illegible]	LP235.169[illegible]	LP235.169[illegible]	LP235.169[illegible]	LP235.169[illegible]	LP235.169[illegible]	LP235.169[illegible]	LP235.169[illegible]	LP235.169[illegible]	LP235.169[illegible]
135165.2	133146.5	114518.3	165498.1	159092.8	117299.8	118638	171985.7	136338.7	142988.5
129773.4	123533.1	110285.9	163113.6	148722.4	104909.1	136501.2	173473.7	137548.7	143524.6
131419.4	139176.1	119717.6	174751.6	152955.1	113562.8	136439.3	182529.9	147492.9	137214
143951.5	140186.9	123915.2	189031.3	174336.6	121736.1	136654.3	199514.6	152589.5	151276.6
165960.3	183293.5	162209	220304.3	214553.9	165424.6	159214.2	223048.8	176382.4	171295

LP235.169	LP235.169	LP235.169	LP235.169	LP235.169	LP235.169	LP235.169	LP235.205	LP235.205	LP236.072
122603.3	158067.4	156219.2	173072.5	173286.6	186291.1	193122.8	39828.2	46758.16	43578.77
134403.2	185778.8	154036.6	162022	176720	195164.3	193677	128582.2	169008.4	46961.86
143687.2	190640.2	156795.3	180732.2	187177.3	195805.4	213016.4	135592	165338.7	14148.49
143245.4	191215.1	170680.7	190060.8	202909.8	212930.1	227199.1	142391.7	168388.1	47256.74
172669.2	216577.5	183638	220637.9	219663.6	257205.1	272352.8	162856.3	218009.2	51105.52

LP236.112	LP236.112	LP236.112	LP236.112	LP236.112	LP236.112	LP236.113	LP236.113	LP236.113	LP236.113
65667.64	70363.65	75558.73	65800.11	50977.81	43526.4	77617.97	131967.5	92438.91	70932.51
65252.78	69823.11	83823.63	68239.4	53532.05	39306.42	82505.85	94206.26	93355.1	76561.63
65358.37	72906.92	77237.6	62910.46	48081.49	48349.07	106955.6	93789.1	93565.25	77997.9
67914.07	80769.17	89695.07	66745.91	55901.1	43629.24	78611.55	102253	105482.2	116535.5
64952.86	71008.92	88218.98	77672.39	52635.46	45717.62	101691.8	84340.85	68593.56	82857.32

LP236.113	LP236.113	LP236.113	LP236.113	LP236.113	LP236.113	LP236.113	LP236.113	LP236.113	LP236.113
56523.1	79726.66	82993.37	72972.64	68329.14	73230.63	72100.78	82768.52	59909.66	66217.45
73164.87	74472.94	75984.44	73338.87	68778.38	69762.34	68117.06	90863.05	60333.55	58310.73
92972.24	103724.3	89681.85	87527.99	71781.55	67278.95	55475.98	89270.66	62153.2	69765.25
66820.7	76320.96	87220.16	80089.05	69639.88	69706.07	73080.58	95904.84	58131.69	62584.73
110299	67978.57	104582.7	78504.04	96526.47	73716.86	68709.95	92532.09	74371.76	61888.22

LP236.113	LP236.113	LP236.113	LP236.113	LP236.113	LP236.113	LP236.113	LP236.113	LP236.113	LP236.113
56552.75	54411.46	79387.33	66901.9	67186.34	62416.15	57628.15	80199.64	79725.05	84785.65
64440.97	58705.01	69811.54	69479.14	64403.93	56697.8	64071.43	77285.78	74806.88	79734.14
57779.43	64413.44	72792.76	63064.03	62405.31	62666.77	63884.36	79806.21	71629.46	81362.07
68498.32	61430.52	109462	64123.83	71246.56	61906.29	66356.54	72891.83	79731.34	86955.2
76823.61	77789.51	82942.52	88539.34	77796.99	67423.44	77740.49	100984.9	106106.5	106796.6

LP236.113	LP236.113	LP236.113	LP236.113	LP236.113	LP236.113	LP236.113	LP236.113	LP236.113	LP236.113
62082.14	65250.3	85445.03	58629.22	70107.26	59886.13	87496.73	42657.63	38841.36	39957.25
67934.02	65218.68	79380.54	55888.27	73993.58	59154.51	88984.91	38485.97	42709.13	40879.94
65171.8	65847.11	81164.06	60630.67	72935.44	56593.94	89943.45	44824.52	39724.33	40663.43
65689.21	64008.79	80672.68	62602.73	81262.85	63161.84	82808.64	45771.3	40939.91	34119.92
64289.43	66456.99	85390.93	59491.13	73502.6	58510.52	82743.74	43391.78	34405.38	40664.48

LP236.112	LP236.113	LP236.113	LP236.113	LP236.113	LP236.113	LP236.113	LP236.172	LP236.170	LP236.173
80774.34	62314.2	63013.96	79310.03	93018.26	75617.29	75920.64	21511.47	20560.19	23031.39
75187.24	61374.64	63572.05	72710.31	92481.12	73555.22	73908.28	27019.9	20026.27	20307.45
79433.55	65021.07	63761.69	68031.04	115847.2	114073.4	74050.79	22961.58	20276.51	22596.62
84070.81	67390.54	66221.42	79424.37	107434.6	74991.66	141746.6	25991.04	18035.93	26012.98
84943.44	67891.41	63834.59	74475.17	86569.25	83010.26	54401.17	30336.04	19272.68	28396.79

LP236.173	LP236.172	LP236.172	LP236.907	LP236.907	LP236.907	LP236.907	LP236.907	LP236.907	LP236.907
29928.5	27588.5	18005.6	60972.96	48004.48	52923.53	46898.21	46753.74	46921.98	50912.55
28453.04	21365.87	19127.96	59761.41	43749.99	53595.05	48043.36	50130.61	44623.09	43242.98
31660.24	23406.91	20616.62	76164.76	60136.38	66751.32	62266.9	57798.06	56456.14	54372.37
27807.82	30406.66	19688.74	78406.85	60595.31	77722.3	59301.47	69169.81	64275.17	62962.68
38765.04	31748.53	29482.3	56742.08	41563.66	55203.99	46338.76	50384.04	41672.78	42933.01

LP236.907	LP236.906	LP236.907	LP236.906	LP236.906	LP236.906	LP236.906	LP236.906	LP236.907	LP236.906
66874.43	47557.73	60348.65	73404.01	55444.2	51294.99	58981.7	46927.63	39952.48	30964.7
55328.53	43586.75	59129.94	65434.7	54064.49	45016.2	57823.95	44468.05	37058.6	19775.4
73203.14	64855	74634.66	83121.11	68586.78	55587.07	68344.18	48036.21	51290.12	29417.53
78714.21	63670.09	92182.7	97168.83	70898.67	68803.05	82173.12	66001.87	60034.84	29169.33
54224.89	43844.99	61447.49	68133.5	52484.02	47469.59	54072.23	43402.21	41449.69	21494.29

LP237.148	LP237.148	LP237.148	LP237.149	LP237.148	LP237.148	LP237.148	LP237.148	LP237.148	LP237.148
126696	86627.62	80499.27	97783.99	67696.76	72125.28	71891.21	59219.58	70592.16	78800.4
125116.5	93730.62	79230.99	97691.9	66835.28	80845.76	75845.57	54898.94	67574.46	81082.15
117432.9	92196.44	80678.97	109094.5	66272.36	73202.31	72967.01	59121.69	74823.72	77646.81
131433.5	95517.76	70991.19	102590.8	68525.4	73268.62	72688.79	53676.38	69462.39	71017.72
131003.2	86338.65	76807.99	103944.6	63241.54	73964.46	76466.73	61538.85	69685.77	77284.05

LP237.148[illegible]	LP237.149[illegible]	LP237.149[illegible]	LP237.149[illegible]	LP237.149[illegible]	LP237.148[illegible]	LP237.148[illegible]	LP237.149[illegible]	LP237.148[illegible]	LP237.149[illegible]
75519.86	94242.82	82100.97	75717.65	82666.42	62132.69	76202.23	87965.64	69559.31	83422.02
69679.52	90628.95	80981.33	73295.37	84499.65	68630.62	76431.87	82660.28	62900.1	80520.9
69126.37	102647.3	81595.64	74730.37	81579.11	61382.4	76871.53	86377.43	60586.07	74559.44
79532.76	104325.2	78555.4	74167.14	86301.01	60016.28	76109	84066.78	60527.52	82077.18
78134.44	115744.7	87423.89	77072.88	89867.14	70535.5	82751.97	85465.31	71231.79	86432.34

LP237.149	LP237.148	LP237.148	LP237.148	LP237.148	LP237.148	LP237.148	LP237.185	LP237.185	LP237.185
72015.34	76871.63	72616.28	69980.49	63585.72	50689.32	76942.9	80490.27	72583.49	100238.5
69476.8	77589.66	78440.72	66789.73	59340.03	54708.11	80774.73	83403.1	65943.79	100057
73400.88	84069.46	66057.69	64460.37	67214.61	54015.4	78307.23	85531.12	77266.07	97295.01
68263.93	72111.3	70206.01	63348.79	63602.1	57359.11	77184.67	89044.58	78101.8	112886.8
73471.57	78463.72	71595.33	63635.16	59299.56	50801.94	72375.87	95362.89	78620.33	113276

LP237.185	LP237.185	LP237.185	LP237.185	LP237.185	LP237.185	LP237.185	LP237.185	LP237.185	LP237.185
70622.63	75945.73	89555.63	74187.44	81035.04	82187.96	74510.44	94564.98	57950.57	73326.64
69810.22	80117.5	91393.35	67171.56	80719.09	75338.77	71246.81	79162.37	59309.07	73439.63
73436.14	72701.74	82052.18	66617.43	82527.55	78231.95	67906.66	88805.08	59957.37	76733.27
72438.48	68217.57	95327.4	101748.6	68123.45	87786.5	78276.9	82446.61	63241.14	63087.43
76290.18	70385.07	84129.67	72524.72	90241.96	87145.21	73501.38	94969.99	66047.05	75940.13

LP237.185	LP237.185	LP237.185	LP237.184	LP237.185	LP237.185	LP237.185	LP237.185	LP237.185	LP237.185
71190.95	71128.61	108800.8	52467.35	56965.32	76081.13	63085.66	56175.9	55414.46	74378.25
76600.9	67800.67	100087.3	50276.49	58201.67	76692.4	56250.89	57011.89	53638.18	69105.08
71559.22	66462.01	100934.7	49175.03	58180.01	81258.84	58591.91	52851.6	60323.57	71261
79567.83	64966.21	98480.52	51041.4	58492.73	80372.45	57393.46	56450.12	54876.68	78539.92
81530.49	70276.29	99716.66	59283.47	60850.07	79960.72	55523.1	53466.57	65780.35	79735.14

LP237.185	LP237.185	LP237.185	LP237.221	LP237.221	LP237.221	LP237.221	LP237.221	LP237.221	LP237.221
54376.81	68937.5	80910.79	95271.18	86890.87	86660.15	81345.93	94654.57	63564.71	71649.85
57165.84	62154.15	70376.99	84759.42	76669.94	90012.66	72735.9	74784.86	65385.87	79461.59
58301.49	64906.52	69380.06	96366.98	89258.86	92526.97	79201.44	78462.11	61944.25	86092.15
54587.18	64592.39	77660.69	104989	75384.92	99372.68	77621.4	75318.76	78258.28	95492.91
57077.09	66795.47	75114.56	77789.24	81736.53	105092.1	79377.67	81668.89	71233.07	80777.25

LP237.221	LP237.221	LP237.221	LP237.222	LP237.221	LP237.221	LP237.221	LP237.222	LP239.126	LP239.127
61281.83	86399.99	72037.82	74253.73	65239.5	41488.61	56008.12	94997.48	38595.5	39562.17
67064.75	89066.01	65552.08	65069.03	67483.31	44178.85	62928.59	92980.31	40253.52	40215.37
64867.42	89599.51	68087.49	72217.65	65250.32	44100.06	56262.03	92182.29	38309.38	51493.88
70704.2	98125.08	68968.18	70058.86	68393.08	43673.84	55481.12	99796.12	34628.6	41854.33
74139.27	107233.1	71659.86	72583.5	72601	48231.1	57313.94	96938.05	46479.7	45180.87

LP239.127	LP239.127	LP239.127	LP239.127	LP239.127	LP239.127	LP239.127	LP239.127	LP239.127	LP239.128
66707.3	87107.41	67249.88	86637.69	60186.21	53191.94	42403.38	58288.38	59249.02	81709.79
61758.69	86162.91	65985.68	78152.58	66274.41	38157.93	44056.03	60340.59	56325.03	88102.07
66997.69	88127.15	67164.56	80480.04	57088.8	41893.13	38982.61	63267.88	56868.27	84172.02
66312.56	87782.59	56337.46	76250.96	65245.52	41842.54	41864.58	61489.21	73558.64	75323.93
70263.08	84690.33	66461.19	84913.78	60776.31	34278.66	40805.5	59114.68	60029.88	79582.03

LP239.127	LP239.128	LP239.128	LP239.128	LP239.127	LP239.127	LP239.128	LP239.127	LP239.127	LP239.128
77282.81	68780.97	85730.43	83477.52	48588.36	53962.13	55886.19	71429.91	57007.72	55349.01
54890.05	71343.2	82335.69	66682.78	59400.91	57338.06	52235.16	69422.62	55280.85	61596.3
53802.96	80914.64	80338.01	78295.22	58036.62	60051.27	54298.46	67177.88	78789.43	62483.23
54216.39	76880.71	79187.62	70577.76	56955.68	59836.73	55963.82	64451.63	53314.45	60006.14
55190.94	81430.36	85813.64	80133.72	64498	55296.61	53621.35	68707.27	52032.5	63409.59

LP239.127	LP239.127	LP239.127	LP239.127	LP239.127	LP239.127	LP239.127	LP239.127	LP239.127	LP239.128
75423.25	47746.87	69952.33	66466.52	52610.41	59983.16	57709.02	75330.54	74285.26	78465.68
69291.23	61091.07	81292.21	63239.4	51873.31	54868.88	60046.32	77193.45	74969.47	85341.69
71388.76	55208.49	67291.78	63982.8	55632.27	60514.63	54972.3	75765.66	69541.88	81836.38
71743.01	77885.7	75213.12	65749.17	52923.58	63902.37	52755.26	74387.3	73354	81522.01
74401.02	52280.4	78386.1	70413.88	54818.17	72128.31	55188.67	75179.95	68224.43	83509.52

LP239.127	LP239.127	LP239.127	LP239.127	LP239.127	LP239.127	LP239.127	LP239.164	LP239.164	LP239.164
59803.82	57694.46	68662.51	55623.86	61405.67	52302.96	62013.08	115634.7	109250.1	98668.22
61458.41	53593.48	72790.37	56424.37	63171.85	54652.68	57179.92	86859.45	84559.71	87750.52
58739.91	55720.62	73199.61	61717.65	69437.36	50295.69	62682.73	84121.63	97385.76	104312.1
63460.11	58908.3	73172.71	60324.54	65787.54	53060.1	58897.37	104596.2	93439.86	95043.79
60086.35	54019.66	79961.94	58954.87	72191.2	47597.62	56965.22	88101.92	103224.5	126178

LP239.164	LP239.164	LP239.164	LP239.164	LP239.164	LP239.164	LP239.164	LP239.164	LP239.164	LP239.164
84096.49	97071.15	85767.23	61039.88	65185.89	62225.86	60110.03	60953.61	54968.87	88220.74
74824.29	120392.3	75433.73	49049.17	67588.29	65956.45	61521.07	60902.83	59239.13	80354.46
76016.28	137943.3	63654.93	57832.43	64330.53	67951.02	58906.28	60716.33	59416.74	77258.28
77856.67	120522.4	93851.4	59486.17	69375.03	67664.54	65043.18	64825.59	62583.72	78358.98
73498.47	117500.8	69575.82	56612.79	71863.48	63629.19	65256.06	60570.56	61496.54	85310

LP239.164	LP239.164	LP239.164	LP239.163	LP239.164	LP239.164	LP239.163	LP239.164	LP239.164	LP239.164
40733.89	56746.4	66251.81	87999.35	70415.05	95619.96	72680.85	70332.84	96260.56	55552.52
40295.37	69854.74	69297.22	95991.6	73823.53	92531.83	78696	71647.61	99348.13	56445.88
38368.34	60815.26	63897.25	91223.01	78126.43	96681.52	85435	71836.73	105348.9	55757.15
40441.91	63777.03	72381.88	88305.05	71489.13	94279.12	77145.51	71052.37	94878.36	56103.45
38957.55	68542.14	68603.48	92779.61	69778.58	99116.11	77251.46	80629.87	95802.63	53757.65

LP239.164	LP239.200	LP239.200	LP239.200	LP239.200	LP239.200	LP239.200	LP239.200	LP239.200	LP239.200
69274.04	98612.96	119313.5	105952.9	93466.07	95232.39	84697.03	88642.48	79519.4	71776.29
77512	96791.97	110003.8	102864.1	85648.93	80900.96	81677.15	85105.21	82085.02	72239.2
82912.31	90070.74	121932.6	104736.6	97438.63	86310.82	84111.09	83868.2	84403.74	70325.65
87093.09	87790.91	106768	103227.2	92393.34	94336.42	81958.48	88435.44	89439.62	78371.17
79054.33	93329.26	112406.3	102861.8	101060.9	86217.46	75664.71	79408.45	83822.43	78460.31

LP239.200	LP239.200	LP239.200	LP239.200	LP239.200	LP239.200	LP239.200	LP239.200	LP239.200	LP239.200
88094.54	77910.93	110755	67348.3	73566.61	81005.84	70684.98	55502.84	59951.25	74104.67
94726.67	72812.59	105910.7	74524.67	73014.57	72512.52	76735.85	59601.97	60342.1	68414.5
87410.86	74960.56	99597.46	75352.78	32598.05	86690.65	78874.66	57401.54	64549.39	64780.68
95856.52	72561	107466	73559.16	73988.98	81660.63	72128.33	51971.77	62389.36	71690.49
98150.72	82533.26	101088.4	75867.17	79832.33	82724.44	87267.04	52980.4	59436.2	71325.48

LP239.200	LP239.200	LP239.200	LP239.200	LP239.200	LP239.200	LP239.200	LP239.200	LP239.200	LP239.200
98929.49	72844.94	58312.22	75746.53	60457.68	70282.79	60596.56	72664.31	80948.98	65514.89
109758.8	72795.06	55801.78	79713.88	66369.56	68240.61	63951.24	73512.87	73293.12	72629.43
69760.1	71473.55	50233.87	78331.34	70886.79	67546.35	59375.84	76628.01	74378.58	69504.52
100013.1	78894.36	50496.02	80749.32	65956.46	72523.78	62071.49	75308.05	76430.22	74319.33
106868.2	76199.23	56137.02	80613.84	63362.53	79693.34	63174.48	76731.63	80325.79	73617.5

LP239.200	LP239.200	LP239.200	LP239.200	LP239.200	LP239.200	LP239.200	LP239.200	LP239.200	LP239.200
60515.39	72196.61	67005.31	63746.38	67685.77	79496.71	68636.01	79306.25	76595.7	50439.11
59181.83	66903.97	63837.43	59229.06	80179.16	79765.63	68087.66	80460.63	71928.38	52650.7
60983.81	67969.9	66404.3	64637.32	75111.41	46301.44	72855.76	86690.36	68969.65	44100.58
58503.96	70930.18	70500.75	64431.36	67816.75	71935.37	66120.74	87471.23	70171.38	51813.39
58955.37	76539.2	74354	62242.91	74345.76	81792.96	67483.97	89033.79	69504.11	50031.28

LP239.200	LP239.200	LP239.200	LP239.200	LP239.200	LP239.200	LP239.200	LP239.200	LP239.200	LP239.200
56067.8	69188.95	87040.42	50649.64	67540.98	91212.67	88076.76	87389.66	76206.46	60898.46
60562.24	67375.65	84567.66	45199	76388.94	87153.82	83421.01	75092.46	77514.33	58927.51
54388.19	68182.2	91603.26	48645.88	82139.93	90803.27	84590.95	80888.57	82255.47	54747.97
59255.39	80612.14	86423.77	50291.19	68628.09	93663.63	86762.79	81914.59	76869	54286.78
59981.79	71385.39	79453.51	49137.58	72271.77	96434.21	87537.52	79446.26	80433.04	55722.7

LP239.236	LP239.237	LP239.236	LP240.160	LP240.196	LP240.232	LP240.232	LP240.232	LP240.232	LP240.232
136686.5	48349.83	69250.47	13992.54	18951.3	20632.79	24098.63	35132.91	28673.81	19089.96
138627.4	53104.61	66850.73	12923.95	13552.09	22017.5	23127.26	35592.12	27431.79	17665.04
157287.9	48530.74	70484.74	12860.63	15676.03	21875.85	21523.81	32618.16	29147.61	18613.58
161297.5	52087.08	68667.78	13648.43	14232.66	16058.73	21420.98	35869.26	27621.73	18984.46
149390	45225.54	72063.9	15373.13	17991.74	20591.12	19810.57	34421.4	31751.5	19464.29

LP241.143	LP241.143	LP241.143	LP241.143	LP241.143	LP241.143	LP241.143	LP241.143	LP241.143	LP241.143
42381.61	55458.08	52401.38	52253.79	49137.23	42676.26	43191.15	66222.19	52523.8	46522.75
41942.24	62112.6	48087.87	56296.62	61098.59	42346.02	39642.04	64964.01	53873.99	48714.68
41292.73	54883.69	43471.06	54655.95	48665.41	48529.06	41970.95	56861.61	54504.84	43234.25
44133.52	66034.03	69207.97	103667.6	73475.78	44074.48	51555.13	63362.55	54372.45	47365.73
54068.37	73337.16	53436.49	51577.39	67350.1	48645.72	35947.34	73692.27	78893.39	41370.52

LP241.1437	LP241.1434	LP241.1437	LP241.1437	LP241.1435	LP241.1433	LP241.1439	LP241.1438	LP241.1433	LP241.1438
48084.96	40255.89	51012.82	42663.64	45652.69	46071.16	54515.55	51762.91	55920.25	45906.56
47594.29	44064.45	44683.57	43066.82	47068.48	48232.53	55070.82	52864.37	62929.61	45189.45
53187.96	47164.08	45158.21	41128.32	46239	47744.21	48101.92	53579.26	64121.16	44055.88
49849.9	39559.37	49990.36	45091.12	42509.63	48640.58	51446.89	55278.29	65737.51	48346.26
47313.55	43726.58	48757.32	48336.72	50429.17	48706.95	64373.7	56236.25	35997.56	42527.23

LP241.143	LP241.143	LP241.143	LP241.144	LP241.143	LP241.143	LP241.143	LP241.143	LP241.143	LP241.143
53710.49	47988.19	38605.58	52299.06	43678.48	49416.29	35781.99	55563.86	51630.4	52707.89
50798.98	52048.94	35178.33	53665.05	44095.98	49377.73	31762.04	56361.2	50893.18	54032.39
52799.62	46962.59	33336.39	52450.2	44344.54	49570.67	38291.3	51720.78	53425.54	59977.53
51283.83	58030.06	36528.42	51179.32	42049.52	51880.29	35474.78	51837.93	49711.75	55256.04
53633.18	50999.06	53542.33	52859.72	43160.16	51041.41	35008.55	49736.15	53964.74	54198.04

LP241.143	LP241.143	LP241.143	LP241.143	LP241.143	LP241.143	LP241.143	LP241.143	LP241.143	LP241.143
54002.17	52872.74	46295.07	53099.35	43610.83	49194.86	45748.34	61104.17	44648.74	76764.58
52078.56	56014.73	44354.95	50276.8	42186.46	47103.31	48450.97	53993.16	42664.28	75779.07
52020.92	56651.77	49513.41	50870.77	42474.21	48363.81	44417.15	59155.76	42541.21	71704.64
54152.16	60897.66	47349.57	55006.73	42852.64	48895.11	53477.62	61641.7	42861.34	72715.94
42370.55	56656.06	42023.78	49949.97	39003.05	47225.43	40488.73	63638.9	42590.85	77769.41

LP241.143	LP241.143	LP241.143	LP241.143	LP241.143	LP241.143	LP241.143	LP241.143	LP241.143	LP241.143
79208.64	33419.71	41677.09	42192.97	41382.89	55740.84	44267.48	44979.33	35156.82	47448.44
74242.84	41330.87	39368.67	41256.86	41370.03	59626.9	42211.52	43926.72	42935.81	47900.23
74877.67	36031.56	41985.05	40501.6	41293.93	58634.69	43322.76	43821.62	39270.65	46652.36
73785.58	30336.74	45914.42	38197.51	41973.93	52555.13	45354.17	45623.79	37593.98	43036.74
76680.74	34165.95	39075.34	37293.82	39101.08	54035.09	46347.26	37909.07	36721.53	49234.18

LP241.143[illegible]	LP241.143[illegible]	LP241.143[illegible]	LP241.143[illegible]	LP241.162[illegible]	LP241.162[illegible]	LP241.162[illegible]	LP241.162[illegible]	LP241.162[illegible]	LP241.162[illegible]
33987.45	25463.84	53501.92	56732.75	148881.3	157017	108859.4	140930.3	130493	95187.29
37577.36	22716.61	52084.87	56992.31	149804.2	156439.8	98520.26	122394.5	128842.4	94983.25
35142.38	26258.93	50988.82	55800.68	149210.5	159462.8	112574.1	140808.9	138682.7	89749.27
33432.03	26307.98	50312.18	56293.06	156992.6	179438.9	123700.1	131749	132938.3	103855.9
34693.6	29722.61	54128.65	60971.4	163267.1	177971	117807	144318	148405.9	107321.4

LP241.162	LP241.162	LP241.162	LP241.162	LP241.162	LP241.162	LP241.162	LP241.162	LP241.163	LP241.163
108403.3	110619.4	146947.7	178141.7	134767.9	140645.4	92955.96	92966.44	112875.3	130745.9
113452.2	126073.5	141375.3	177830.1	131591.5	122817.8	96772.28	105276.5	122901.5	113997.1
102835	122085.8	154777.1	174119.7	148460.4	125209.6	97065.82	107230.6	113836	124620.7
109648.9	112763	150609.9	186080.4	153300	140569.3	96823.5	115660.3	108993.7	137178.8
116994.9	124915.5	167336.4	172948.5	147032.6	128308.4	88453.96	112349.8	121187.7	135629.6

LP241.163	LP241.163	LP241.180	LP241.179	LP241.18_	LP241.216	LP241.216	LP241.216	LP241.216	LP241.216
120079	147643	103985.7	109311.8	134534.5	178317.4	130499	221471.7	147173.8	85174.45
124026	150225.9	109737.5	104297	143378.3	151852.5	190375.4	159250.1	154986.8	105234.5
127048.7	140467.6	93336.19	95071.09	137301.8	164411	131481.2	153560.6	142426.5	97973.98
128100.1	152293.9	107546.5	104713.8	137088.9	180201	198204.5	141973.8	155597.6	104753.1
123816.1	153726.9	113478.1	113189.4	152838.8	191785.2	129825.2	208371.9	196752.8	103143.9

LP241.216	LP241.216	LP241.216	LP241.216	LP241.216	LP241.216	LP241.216	LP241.216	LP241.216	LP241.216
102547.3	109698.8	194530.5	170263.9	160315.6	149222.3	154003.3	138463.1	141734.7	88940.34
81043.02	129162	229013.9	240666.9	173713.3	125961.9	143984.7	82396.21	194309.7	99711.74
98231.02	129685.4	159625.8	142984.7	124304.6	140066.7	128203.8	94682.73	117593.7	85567.86
83124.69	168371.6	167924.8	157731	125132.8	154114.4	134196.8	102712	157982.7	89725.85
100904.5	135085.5	177456.5	172967.9	134868.3	160364	139608.4	105084.4	125314.2	94079.01

LP241.216[illegible]	LP241.216[illegible]	LP241.216[illegible]	LP241.216[illegible]	LP241.216[illegible]	LP241.216[illegible]	LP241.216[illegible]	LP241.216[illegible]	LP241.216[illegible]	LP241.216[illegible]
129704.1	73670.39	131440.3	183843.8	130511.4	73407.26	68830.19	75661.74	112970.6	160364.4
109608.5	118297.3	134858.4	138637	158052.2	83018.94	101460.8	85054.57	113037.9	110407.9
93076.66	94501.76	99259.1	124162.8	142700.7	62216.64	64903.39	98354.09	122006	123825.6
83004.04	101159.9	131565.4	124711.1	141502.9	64134.67	67709.65	104584.2	101500.4	120415.9
101307.6	101330.8	177803.5	124109.8	152030.7	66332.51	70257.93	102463.8	135805.2	121371.6

LP241.216	LP241.216	LP241.216	LP241.216	LP241.216	LP241.216	LP241.216	LP241.216	LP241.216	LP241.216
134588.8	126469.2	51439.76	128350.9	155103.2	54196.99	81225.65	121017.2	183190.6	75608.56
142123.9	95408.57	38022.23	129412.3	143268	53188.15	89275.05	127532.5	104256.4	84707.53
137744	104041.5	51399.63	120317.6	157096.9	46967.49	86428.77	143574	111629.9	77986.75
140214.3	100634.1	51332.35	130067.6	158678.3	50693.45	75647.57	135250.5	114960.8	78269.49
165072.4	174668	55165.06	136055.9	172056	52935.78	83816.83	150267.8	129136.3	76697.43

LP241.216	LP241.216	LP241.216	LP241.216	LP241.216	LP241.216	LP241.216	LP241.216	LP241.216	LP241.216
139944.7	193455.7	102792.8	64990.66	65339.98	91890.41	107990.7	200213	52152.92	129748.4
155479.7	148173.7	145335.8	69836.91	52239.98	72942.12	118527.7	99708.2	53931.16	138967.3
118581.5	152372.2	121704.7	68598.11	55694.82	76749.9	91863.58	157930	48868.79	119291.3
103281.2	159904.1	124813.6	66920.77	58035.97	77970.99	84215.74	161464.1	50713.99	117028
142983.7	199281.7	132894.8	70389.16	56771.98	74429.7	99658.05	164692.7	49983.34	126960.4

LP241.216	LP241.216	LP241.216	LP241.216	LP241.216	LP241.216	LP241.216	LP241.216	LP241.216	LP241.216
112933.5	143762.8	145341.5	141512.5	72984.66	101671.3	113002.8	152015.3	117594.7	98841.23
119833.6	150545.9	173635.7	141579.5	82759.29	90276.13	84584.25	150014.5	117630.2	106592
103069.7	136719.8	151108	135376.5	100473.9	115281.3	105396.5	124442.1	123220.8	85393.13
103501	135757.8	153374.8	136146.9	93171.27	100808.2	112247	126561.7	131262.7	80854.88
148437.1	118351	162023.8	150285.2	102693.6	111437.2	118375	156030.3	137055.2	96225.23

LP241.216	LP241.216	LP241.216	LP241.216	LP241.216	LP241.216	LP241.216	LP241.216	LP241.216	LP241.216
150027.2	113786.3	96058.58	89842.68	62901.12	135605.9	113730.7	63182.42	88309.13	77428.92
115352	66396.22	94457.07	89632.24	57972.71	149573.2	100865.1	48302.56	93279.14	81009.35
125479.4	92187.5	101817.1	96891.83	55818.7	102675.9	101914.3	65081.27	91477.19	86922.34
135935.2	92993.35	103941.8	93902.8	59905.77	113422	102519.6	66553.1	97055.05	88976.09
138959.7	98546.11	92644.82	100877.8	61340.64	115212.1	108327.8	70873.17	89734.76	138092

LP241.216	LP241.216	LP241.216	LP241.216	LP241.216	LP241.216	LP241.216	LP241.216	LP241.216	LP241.216
87644.76	84186.56	90073.43	72267.94	80822.05	73997.13	46971.87	62260.69	141027.2	132354.6
138183	81121.17	102910.4	91295.59	117797.7	81618.37	42935.48	56685.98	150023	145087
83428.58	78484.19	111320.8	81898.36	87109.66	77035.87	53572.31	58767.88	133789	120050.6
90896.83	76766.97	113916.3	81673.94	86842.98	81623.46	57223.71	61028.8	133930.1	117657.4
101472	87269.58	117347.2	81852.69	91016.53	81079.53	55159.59	64998.18	144784.6	135559.1

LP241.216	LP241.216	LP241.216	LP241.216	LP241.216	LP241.216	LP241.216	LP241.216	LP242.121	LP242.121
125200.3	173830.8	70294.44	68584.46	56588.74	174116.6	109499.1	85081.5	116340.3	84599.07
154607.9	115666.9	68802.5	48685.18	52305.9	144715.4	96517.29	92062.31	101917.3	96003.35
141458.1	154872.6	78156.39	64121.24	57878.8	169892.8	95187.23	86470.75	81780.15	76621.84
137562.8	179115.1	81660.9	69443.25	59890.14	158486.7	101764.2	88460.01	94450.42	68074.81
142742.2	183848.9	83184.86	67647.52	61180.08	160386.6	98536.76	93804.59	136089.7	100198.7

LP242.121	LP242.120	LP242.121	LP242.120	LP242.120	LP242.121	LP242.121	LP242.121	LP242.121	LP242.121
97062.9	94585.22	85985.88	85915.03	67645.65	66392.92	67217.75	65468.54	60133.43	63210.64
93157.53	105642.3	78967.4	76824.88	83635.7	89310.51	94474.79	91357.52	56379.01	59095.53
79188.49	110647.5	90711.82	95295.58	64758.32	76970.81	91689.03	92273.14	78534.22	55181.34
85779.86	96278	75430.85	84636.94	67160.28	57181.59	76678.06	67876.54	51082.34	62056.6
91500.27	109802.2	119641.6	105215.4	68634.92	86939.88	85245.34	101374.7	64360.22	66934.05

LP242.121	LP242.121	LP242.121	LP242.121	LP242.121	LP242.121	LP242.121	LP242.120	LP242.120	LP242.121
63939.96	51141.15	84139.39	50729.17	62249.07	80309.8	67125.64	63795.59	41903.94	46575.14
63930.17	72151.22	65636.69	62077.43	62509.81	73939.98	60434.93	62744.58	42172.58	50265.34
77198.22	52428.93	82421.25	56581.48	64149.08	76459.63	62728.27	66549.51	58550.31	52581.72
66158.67	53304.81	87543.04	51450.82	62652.02	75698.1	64537.02	66311.65	47248.74	50708.07
79251.63	64536.44	81690.47	55621.28	61537.82	74030.97	64930.8	69741.68	38335.03	50822.87

LP242.120	LP242.211	LP242.219	LP242.247	LP242.248	LP243.122	LP243.122	LP243.122	LP243.122	LP243.122
67520.5	5365.358	12606.42	12749.41	10511.84	27795.34	31310.79	37378.26	30620.46	41697.46
57498	7400.104	15293.33	14697.33	8877.538	46963.35	36128.89	25688.6	28081.15	39915.24
73092.68	9268.518	13161.2	15641.13	11505.01	38787.08	30410.49	27441.36	35900.18	40615.42
67517.64	6606.957	9672.742	16103.41	10215.09	40978.81	35788.54	25048.14	34666.43	33994.68
60272.77	7970.341	13202.82	18820.91	14520.43	35411.42	35953.25	26257.56	37241.8	39365.7

LP243.122	LP243.122	LP243.122	LP243.122	LP243.122	LP243.122	LP243.122	LP243.122	LP243.123	LP243.123
44031.09	28515.36	34954.65	41256.52	41507.88	33128.23	31804.11	34299.83	41442.42	63669.2
52129.57	27348.5	30187.83	44699.93	36961.43	30672.21	29529.05	23904.7	46065.45	54239.84
52108.77	26151.29	29232.92	44355.92	38483.27	28911.72	25419.1	20318.57	41755.35	53715.69
52292.13	36835.23	26839.24	40028.95	36182.48	30393.67	25131.39	24444.27	45834.6	62347.66
59071.08	27362.09	24668.23	42541.38	39302.2	30845.39	24358.64	26296.81	43499.81	55507.53

LP243.123	LP243.122	LP243.122	LP243.123	LP243.123	LP243.123	LP243.122	LP243.122	LP243.123	LP243.159
79848.15	38618.92	34394.47	32428.76	44063.5	41155.56	47274.87	39307.68	41191.71	28732.33
72355.73	37445.65	37692.94	41995.29	40431.99	40430.96	49576.85	37842.56	41798.78	25884.22
71570.08	38964.99	34434.31	45753.1	37710.38	37976.62	52255.96	35257.65	46058.5	24995.25
71486.08	35118.89	29849.66	40035.19	40825.29	40513.06	51145.72	38112.27	49606.29	24757.01
65466.93	36529.21	33115.97	40102.01	42188.09	40088.51	47757.31	38198.29	43236.77	31635.89

LP243.159	LP243.159	LP243.177	LP243.177	LP243.178	LP243.178	LP243.177	LP243.178	LP243.178	LP243.178
30458.63	31085.58	34396.73	22811.05	24420.21	37998.35	32949.53	32051.47	33914.08	31649.65
33369.95	39990.58	33276.68	26133.27	29979.96	40831.51	34173.36	30131.9	33608.49	27959.38
33412.13	35094	34034.44	26263.82	24238.08	38150.13	33396.46	30025.33	34229.51	30254.1
30213.99	37463.9	34197.86	26949.92	29198.01	39060.07	30970.91	27261.07	31524.27	32884.33
33224.28	41113.22	53259.02	42294.56	40593.86	63246.62	50255.01	49706.42	53044.97	45220.08

LP243.178	LP243.178	LP243.178	LP243.178	LP243.178	LP243.178	LP243.178	LP243.178	LP243.178	LP243.178
32697.38	33906.02	40583.66	38475.62	25676.81	40842.42	32871.45	34300.06	47201.95	39679.19
30360.2	29318.67	33752.57	35745.24	25659.06	36765.83	32367.69	28764.1	39402.65	37615.87
34654.99	34020.85	36958.58	36670.22	25206.7	41560.82	32786.36	30847.93	47502.14	40491.73
33143.01	38154.14	37110.34	41253.33	26101.91	44129.37	31052.75	35289.39	44908.76	41156.97
36004.53	43412.48	41669.99	63150.94	47911.67	54206.8	51111.64	48897.03	84258.7	52639.93

LP243.178	LP243.178	LP243.178	LP243.178	LP243.178	LP243.941	LP244.154	LP244.154	LP244.154	LP244.154
35604.93	53152.28	40992.44	43545.61	41026.75	26638.23	23723.75	31680.77	38034.16	39992.14
40867.31	50188.3	44544.95	39946.67	40515.97	29052.27	23048.73	29479.15	36015.51	36722.29
38220.77	52821.12	38205.4	37476.11	44576.04	29202.28	21469.25	28743.68	32604.59	39566.78
38012.69	57137.96	42000.59	42538.1	43216.77	28655.03	22652.6	28566.98	33210.75	35962.69
45794.89	54717.27	48757.45	52879.3	51933.5	29430.04	20420.88	33214.11	33478.74	30602.09

LP244.191	LP244.191	LP245.117	LP245.118	LP245.117	LP245.118	LP245.117	LP245.118	LP245.118	LP245.118
37694.98	34511.01	115225.5	657974.6	203146.1	490040.8	271552.8	397273.5	715327.4	327914
37130.61	29977.98	107848.9	598950.7	232429	426349	248976.9	433749.5	583436.9	336777.7
41439.28	30894.9	123141.3	460689.6	265552.2	513558.2	252753.6	512881.8	502971.1	450124.5
43806.45	32629.5	93135.74	499459.2	218925.1	468200.5	174055.3	555784.9	543480.9	386311.6
44767.19	30165.29	119280.9	650993.9	167815.6	320150	197492.1	361474.3	486999.2	228079.4

LP245.118	LP245.118	LP245.118	LP245.118	LP245.118	LP245.118	LP245.118	LP245.118	LP245.118	LP245.118
377907.4	481588.1	411759	346986.3	367722.9	285322.8	274379.5	525355.6	300093.3	448743.4
277134.6	392863.4	387847.7	455098.7	372734.4	284964.7	234960.8	447177	277653	342331.2
273882.8	430056.9	319990.2	446572.7	371044.1	419565.1	224054.8	528819.6	171225.6	400185.5
257430	393208.3	296324.9	353621.6	372458.7	225728.9	254588.3	528855.1	266302.6	476912
305854.7	390173.3	317076.4	306843.9	246788.8	248246	294738.3	447657	197158.7	386902.1

LP245.118	LP245.118	LP245.118	LP245.118	LP245.118	LP245.118	LP245.118	LP245.118	LP245.118	LP245.118
340099.1	248151.3	264495.2	240263.7	273186.3	215295.1	174919.6	345616.1	420266.2	279420.4
351389.6	317931.3	317626.4	284892.7	389245.8	303825.3	204915	369077.4	389281.1	334263.1
430418.2	251577.6	291991.9	301627.7	234449.3	250154.4	260808.8	393331.3	555714.5	392819.4
376804.7	287188.7	352306.2	219968.9	252072.8	243941	194130.7	257153.5	374119	269494
367153.7	279231.1	357305.6	297157.8	240683.2	222478.3	257091.8	376213	418997	365830.1

LP245.118	LP245.118	LP245.118	LP245.118	LP245.118	LP245.118	LP245.118	LP245.118	LP245.118	LP245.118
578116	307510	305124.4	536235	330563.7	404609.4	332361.1	182034.7	214807.9	360165
350111.2	346032.3	271055.5	504999.1	506110	561018.8	329617.8	151558.5	315650.3	367868.7
391485.7	383955.9	308271.4	493095	405583.3	435577.6	337535.9	133097.9	243997.1	425480.7
322248.9	298118.7	264882.9	522207.1	321616.1	607481.8	302531.3	190954.2	256829.3	358288.5
321140.8	292108.1	253719.6	478100.2	325367	509237.3	246314.4	206632.4	223915.3	378317.8

LP245.118	LP245.118	LP245.118	LP245.118	LP245.118	LP245.118	LP245.118	LP245.118	LP245.118	LP245.118
260302	248000.1	371359.8	212621.6	263010.9	217706.3	267892.4	133965.5	196595.7	174569.4
258660.1	309249.2	323898.2	260125.1	267854.8	207029.7	338829.9	193039	182403.5	180802.9
316261.7	267758.2	271009.2	309871.6	283213.5	249085.9	310028.5	109570.2	216056.2	170682.7
269382	275302.7	284233.8	242575.1	223233.3	140340.9	258027.8	136185.3	194007.3	252517.7
272099	455126	341252	238452.7	197414.5	178618	308638.8	120828.2	197361.2	165688.3

LP245.118	LP245.118	LP245.118	LP245.118	LP245.118	LP245.118	LP245.118	LP245.118	LP245.118	LP245.117
153665.2	197262.7	129117.3	152557.1	163692.6	164089	129909.7	163376.9	151570.1	88383.73
163578.6	202167.5	190346	119669.4	157726.9	152940.6	104843.3	147274.1	148772.8	78024.42
161132.8	200379.7	149876.3	113704.1	219173.5	156430.8	112846.6	132003.4	163901.2	90490.56
162280.2	187736.8	158273.2	185168.3	181333.2	153486	127053.5	136698.1	160933.7	80718.23
135093.9	218675.7	115521.5	109467.8	217076.4	201467.5	120761.6	152104.4	224620.2	78667.08

LP245.118	LP245.118	LP245.117	LP245.118	LP245.118	LP245.118	LP245.138	LP245.138	LP245.138	LP245.138
127313.3	115067.3	93781.51	144635.4	233834.9	346773.3	92955.76	17744.16	17546.09	17782.24
115283.8	129477.8	100529.7	116820.6	246563.4	369973.7	104883.7	15550.72	15879.98	19961.91
97871.59	125072.6	94631.47	112601.7	205138	268686.7	91075.65	17478.6	19362.88	19342.92
97285.55	118862.9	121246.8	124331.8	309183	261424	103617	16219.4	16927.03	18363.27
98108.28	127346.9	97363.54	123311.6	155960.2	324209.2	97363.54	16325.81	20113.91	19851.33

LP245.138	LP245.138	LP245.138	LP245.138	LP245.138	LP245.138	LP245.138	LP245.138	LP245.138	LP245.138
174155.9	188211.9	81126.17	106893.3	190247.6	23546.41	93777.88	138166.9	109560	79956.38
191371.7	212912.3	90279.8	103609.1	178289.2	25315.54	100728.6	147927.8	105543.3	73386.8
189923.4	215954.9	74553.11	100718.4	170958.7	27398.64	93418.16	128827.6	97048.05	64695.65
192769	188591.7	73088.58	91532.57	166227.3	23816.62	85538.88	133261.5	77081.59	61030.27
149967.2	198217.1	81741.46	89981.92	165775.1	22007.77	86655.98	119695.5	83331.29	63354.18

LP245.138	LP245.138	LP245.138	LP245.138	LP245.138	LP245.138	LP245.138	LP245.138	LP245.138	LP245.138
135090.8	28143.75	41775.57	35925.4	26247.63	105902.8	15687.64	58602.03	134263.8	39877.94
137724.1	30971.28	43843.22	32612.6	26670.09	114113.7	17721.72	58306.8	159305.2	36116.64
121898	31934.43	41458.37	30955.97	26100.68	100439.6	13893.86	52813.5	139782	36344.21
127843.9	25280.63	39030.68	33047.86	26566.54	102028.6	12488.42	48383.5	119400.9	35829.5
114484.3	27683.19	37833.43	28591.71	25792.39	99135.95	12992.58	49504.98	121535.6	38152.86

LP245.138	LP245.138	LP245.138	LP245.138	LP245.138	LP245.138	LP245.138	LP245.138	LP245.138	LP245.138
170136.5	23934.87	24313.16	173759.6	46208.61	27970.22	148947.4	321174.9	29680.31	119690.1
182571.9	25034.49	29787.45	186287.6	55370.47	28162.03	145578.4	315964.2	26209.77	143226.9
180378.3	26906.9	24130.66	154100.4	42783.04	25853.16	132854.2	309892.7	33722.15	132271.9
157515.4	22885.63	19197.06	158451.5	35864.37	27393.21	141780.5	305848.7	26613.37	123751.8
165849.5	21188.5	21798.55	184098.6	41992.95	26563.65	154398.1	304246.5	26822.27	128407.3

LP245.138	LP245.192	LP245.193	LP245.193	LP245.193	LP245.193	LP245.193	LP245.193	LP245.194	LP245.193
144426.1	33321.37	34741.9	34183.81	32180.56	33641.47	38860.53	32250.83	39441.11	27035.19
129365.1	34424.98	36408.67	35163.13	31817.55	32872.42	31628.26	32166.64	34675.77	26491.73
128676.5	34940.99	32811.28	37688.55	29382.5	32648.71	40552.43	35497.96	47117.6	26350.36
132131.5	36966.47	39558.73	41528.52	35174.44	30813.93	32666.25	38512.08	41770.61	24028.58
134008.8	46339.95	55773	40335.87	44880.85	43164.3	50266.02	55215.11	56806.14	33917.32

LP245.193	LP245.193	LP245.194	LP245.193	LP245.193	LP245.193	LP245.193	LP245.194	LP245.193	LP245.194
32740.88	36183.85	51367.54	46473	38115.13	34100.32	40505.97	45871.63	58869.77	43028.05
29451.85	32602.23	42091.17	48203.34	41163.61	32381.72	36090.79	36861.76	55988.02	44037.42
28589.56	37525.91	47039.52	48413.98	43070.12	31504.86	45381.77	39117	61085.5	43136.09
33497.38	34212.35	48602.57	46186.3	42949.16	33002.19	41904.11	43992.35	60555.34	45761.8
37544.55	44769.79	64482.83	62649.06	54572.76	50377.44	71463.5	59763.71	86789.75	59521.09

LP245.194	LP245.194	LP245.193	LP245.193	LP245.193	LP245.193	LP245.193	LP245.193	LP245.193	LP245.193
39477.08	37791.15	44869.83	32273.05	26526.12	27898.53	32325.96	30272.14	41172.25	38227.77
36782.38	37095.26	37802.47	27898.4	29195.48	28025.02	28474.67	34097.79	38642.13	33968.03
35963.18	43733.03	54052.78	27322.96	28484.1	29665.75	33961.14	30917.65	46244.36	38593.78
38245.26	41894.71	50491	26063.77	32157.62	26904.17	30838.59	30548.33	42283.69	35425.52
52157.88	57215.92	65641.4	40809.98	40356.72	34053.45	43491.99	39124.22	61943.13	50505.15

LP245.193	LP245.193	LP245.193	LP245.193	LP245.193	LP245.193	LP245.193	LP245.193	LP245.193	LP245.194
45326.39	36134.89	22720.36	37704.92	28122.55	26997.3	25642.58	30787.31	45843.57	35308.54
47428.44	34875.93	24115.97	36461.36	25671.88	28929.97	29116.74	28869.07	41488.13	39051.12
50714.69	34600.35	27137.3	31792.59	29534.13	30003.89	25001.11	32551.83	41905.54	47248.07
52976.71	38540.32	25205.64	39238.34	27712.3	31078.38	26473.91	37707.29	43539.66	45373.02
67542.1	56889.56	33286.74	49510.23	34648.45	38683.69	38744.89	47009.23	62180.2	56559.31

LP245.193	LP245.193	LP245.193	LP245.193	LP245.194	LP245.194	LP245.193	LP246.170	LP246.170	LP246.170
32405.21	31409.3	61973.18	32228.8	42847.11	47830.42	42526.69	36936.49	21138.79	22881.92
25044.23	33656.11	60023.58	26416.97	43832.26	52375.97	38667.26	32157.23	25085.87	27786.91
33132.56	36925.97	59018.96	32084.56	41633.91	55779.64	35647.68	29910.96	23487.37	22779.14
32232.85	32861.4	61237.22	37326.24	42712.26	60798.62	37582.7	35306.49	18754.55	25279.9
36576.52	48598.61	83919.09	41835.12	57348.24	67198.85	53993.84	32024.81	21813.27	23105.86

LP246.169	LP246.169	LP246.170	LP246.170	LP246.170	LP246.170	LP246.170	LP246.170	LP246.170	LP246.170
40637.43	33388.19	27958.16	34514.59	39835.06	31901.04	35515.79	21079.02	39317.4	31702.76
46146.75	35047.44	31277.45	29293.35	41066.79	33607.5	36620.49	24189.97	33960.01	37289.14
45453.31	35528.29	29431.29	31868.07	41957.81	33980.13	34410.91	27390.54	34537.91	36228.66
41758.29	37446.48	33582.16	28471.75	46034.83	27597.09	33427.43	22373.26	33499.85	36862.49
43265.98	36976.67	29068.79	33747.62	46797.04	30805.17	30245.84	25146.32	37590.93	27770.82

LP246.170	LP246.17_	LP246.170	LP246.170	LP246.170	LP246.17_	LP246.169	LP246.170	LP246.170	LP246.170
39537.4	35519.52	27867.52	29733.91	44817.22	32542.08	35213.92	36136.68	31055.02	26972.5
35418.16	37892.31	25318.49	34465.11	43781.86	27824.75	39007.43	35817.72	33650.99	23937.08
36770.88	38387.26	26260.67	31569.86	45758.12	29992.67	39809.35	36604.16	31331.08	26828.93
41223.39	37378.02	25483.39	33102.84	43502.38	27980.95	40477.36	36346.56	29998.7	25357.44
35668.7	35553.3	27610.54	29539.78	41363.33	28008.11	41961.08	39936.7	31895.96	28046.26

LP246.170	LP246.170	LP246.170	LP246.170	LP246.170	LP246.170	LP246.170	LP246.17_	LP246.170	LP246.170
40010.86	38970.78	32541.25	38672.37	41309.09	25439.68	35760.27	37013.37	53542.82	26232.35
41077.59	37641.61	39809.66	37301.26	44601.03	28118.02	34211.55	40387.48	54693.05	28901.19
43182.96	32728.08	33799.76	32591.38	40287.78	27372.47	36327.42	37439.15	52074.93	31009.75
40639.49	31147.43	37902.61	41839.71	49460.75	27601.65	36788.35	38732.42	54257.84	30622.8
40253.03	34323.4	32476.77	36045.74	44005.67	26481.83	32804.81	35150.62	54894.01	26926.68

LP246.170	LP246.170	LP246.170	LP246.170	LP246.17_	LP246.170	LP246.169	LP246.170	LP246.170	LP246.242
42980.39	34035.81	33710.48	24581.7	42936.51	45727.59	34214.24	38484.05	39178.42	43445.67
40874.23	38335.35	33431.02	24994.05	42536.86	46972.74	34509.24	39368.38	41518.82	46056.18
41047.26	32664	34901.29	21429.97	44928.87	47831.78	37551.6	38533.82	39451.81	45532.53
41154.04	35299.41	36748.5	22092.04	39604.02	44958.42	39347.85	38869.2	40893.28	46679.95
42521.29	30014.97	32579.55	27359.44	40020.4	49993.58	36320.35	38589.9	40426.73	45163.62

LP246.243	LP246.242	LP246.242	LP246.945	LP247.206	LP247.206	LP247.206	LP247.226	LP247.242	LP247.242
34428.34	48627	34986.4	13573.59	35329.65	26212.58	23435.72	50844.57	15709.59	32508.61
33592	45962.79	37330.96	12115.59	33611.71	23939.73	21643.09	54191.75	14016.56	35615.89
36373.46	44365.07	35127.17	11742.69	28421.45	27769.4	25181.54	49141.33	15076.39	32151.03
38929.2	40653.69	36831.48	8108.876	26110.98	23759	24919.7	49665.7	15284.51	39907.66
32501.4	44658.56	31327.84	12745.56	37142.1	31273.4	24890.31	46913.89	17061.88	35843.51

LP247.242	LP247.943	LP247.943	LP248.059	LP248.149	LP248.149	LP248.149	LP248.149	LP248.149	LP248.149
42019.52	17217.16	12521.88	24464.84	26896.9	35702.55	47400.04	30185.94	29202.16	28997.36
36931.06	16795.86	9659.169	28433.73	18658.53	31039.92	50379.91	32410.55	26845.49	34423.88
41219.65	21640.03	14895.04	24674.05	27278.57	32192.69	43027.17	40094.35	27450.53	34214.33
35939.62	22921.36	18379.7	23848.83	27967.16	36054.16	48833.71	30966.66	29402.76	36155.23
41816.23	16190.95	12603.44	22450.71	22946.64	37560.89	49999.38	30099.57	31377.72	33157.36

LP248.149	LP248.149	LP248.149	LP248.149	LP248.149	LP248.149	LP248.149	LP248.149	LP248.149	LP248.149
33949.81	46878.6	41071.85	32110.95	29772.76	39050.38	36351.81	36261.52	21366.69	37735.44
35634.99	44006.83	43932.17	34137.17	24712.21	42474.04	30433.13	35498.34	28989.28	31939.08
36851.8	44022.39	44211.02	34437.79	29851	42378.87	30344.86	34417.28	26348.52	32753.1
39956.38	39747.14	45374.04	36286.9	26489	42163.05	31164.89	27453.52	21316.48	29987.68
36677.37	41397.23	45708.52	33160.46	29123.79	42907.29	39630.64	34266.04	21589.13	35517.7

LP248.149	LP248.149	LP248.149	LP248.149	LP248.149	LP248.149	LP248.149	LP248.149	LP248.149	LP248.149
32790.75	31775.85	33427.66	40447.22	28037.89	42729.75	37350.51	35001.16	31742.79	27084.14
34822.45	29177.66	30369.12	40786.05	27922.36	42884.82	39660.76	33812.44	36032.58	23264.32
34256.3	32734.28	30942.89	40488.21	30994.64	40486.54	39933.86	33890.15	32793.6	24937.42
29541.89	28566.04	26902.81	40906.99	28036.44	43133.77	41562.56	37585.89	33692.63	27935.92
32054.05	32536.9	26828.63	39637.18	32666.22	44650.1	36779.74	36122.74	35899.21	24006.39

LP248.149	LP248.149	LP248.149	LP248.149	LP248.149	LP248.149	LP248.149	LP248.149	LP248.149	LP248.149
31351.54	30855.61	48779.42	36635.83	34626.39	35489.73	30777.93	31144.47	42966.47	40262.22
28105.67	34051.71	49944.56	31477.38	34789.14	31235.68	37973.94	31317.92	37229.63	30419.96
31078.65	30244.54	47248.78	28462.47	34124.61	33281.17	36353.12	30403.53	41550.69	36516.53
26765.64	35039.85	50015.39	33890	38961.55	32480.63	34373.83	31974.87	38493.14	38232.37
32348.86	32934.24	54708.14	32238.08	35266.62	35322.82	34244.63	31599.64	41199.16	32731.52

LP248.149	LP248.149	LP248.149	LP248.149	LP248.149	LP248.149	LP248.149	LP248.149	LP248.149	LP248.149
33685.05	36427.17	30059.29	47268.05	41930.7	61101.62	25509.13	38951.71	36415.32	29504.84
39117.26	37545.95	32896.09	47237.61	41247.51	64855.8	28555.4	40510.61	37976.73	32423.48
31547.53	33034.21	31323.34	43619.44	41830.16	64233.15	29610.49	41555.62	41567.14	33174.01
38242.16	32117.03	25996.69	49488.18	40149.45	63147.78	24674.04	41587.35	35789.51	31363.72
38748.03	37290.79	24983.14	52128.69	39414.21	64148.52	24289.73	40754.11	36443.61	29033.87

LP248.149	LP248.149	LP248.149	LP248.149	LP248.149	LP248.149	LP248.149	LP248.149	LP249.148	LP249.148
39170.1	47647.33	37962.07	26646.11	49267.71	45988.62	26976.19	29498.07	36393.15	43034.93
40534.77	47473.13	33064.29	28131.97	54423.47	44425.48	28469.69	30385.94	50453.76	25807.6
37573.97	51272.47	31470.78	25909.96	49781.81	43744.12	30685.6	33137.63	40379.15	38053.21
43829.29	47202.76	37037.91	23851.76	55705.85	42210.32	30090.92	30500.82	40416.44	35595.08
43004.42	53057.93	38957.17	25733.92	53237.74	43970.48	27025.11	29914.29	34333.11	36154.72

LP249.148	LP249.148	LP249.148	LP249.148	LP249.148	LP249.148	LP249.148	LP249.148	LP249.148	LP249.169
31722.47	30132.52	32404.76	36114.4	30612.19	36329.47	26925.76	29344.43	33724.77	39596.21
32930.1	38653.68	37752.74	46051.15	50057.94	40830.81	25703.27	25889.16	29533.96	41312.99
28459.7	27562.68	31823.73	44367.55	31371.04	33415.04	22140.46	27220.94	26051.87	37161.36
29244.04	30893.13	30858.86	40126.93	35681.19	39239.11	27427.05	31202.35	27881.49	38688.64
29949.69	32297.58	37911.72	41332.67	36040.01	41618.87	24467.67	30328.95	31367.37	34798.29

LP249.185	LP249.184	LP249.184	LP249.185	LP249.185	LP249.185	LP249.184	LP249.184	LP249.185	LP249.184
49621.97	66732.88	51458.26	33717.05	48202.38	62849.27	30668.28	31208.78	48471.06	28974.88
47739.71	63548.99	47492.85	30281.08	45165.74	64003.27	43184.81	32162.26	48101.37	36960.85
49047.79	70588.74	51303.76	31706.83	49010.2	62510.72	35764.91	30544.57	51775.92	28179.51
62935.19	61310.88	50485.09	32396.81	59488.46	70194.47	30805.32	28497.62	46625.21	34972.82
49414.03	79037.46	52795.17	34436.96	49373.99	62139.7	46300.99	32399.92	56708.74	42078.49

LP249.185	LP249.185	LP249.184	LP249.184	LP249.184	LP249.184	LP249.184	LP249.221	LP249.221	LP249.221
46218.56	56600.6	32329.27	44494.45	61702.5	33141.92	47492.82	55965.03	45512.91	47682.25
49654.73	55036.68	37193.79	46945	63486.27	35764.37	53740.61	21465.67	32919.96	34592.93
47275.03	58229.14	43087.72	47013.35	59844	28026.01	54635.38	59218.59	53469.07	43662.07
51210.75	65516.06	38953.74	46419.86	77392.99	35665.75	49747.05	35607.72	43044.34	46319.56
55967.98	60685.41	37090.48	50416.24	60629.39	38778.33	54948.39	67520.3	54977.9	51187.29

LP249.221	LP249.257	LP249.915	LP250.086	LP250.178	LP251.000	LP251.000	LP251.000	LP251.000	LP251.000
42112.11	13928.41	16155.59	16290.13	10128.41	131260.2	148464.1	151119.6	123131.2	97392.89
46566.37	8936.776	13816.23	12953.42	9717.484	169641	140454.4	182366.9	129729.1	115567.3
49425.68	18052.36	11260.87	16168.72	10968.14	213853.8	139294.7	199940	121844.2	109089.5
24008.03	11580.67	11787.79	13260.24	10131.05	154164.1	135206.4	153916.2	140063.3	118876.4
46192.25	16900.59	14906.48	14608.17	7171.097	122450	129597.4	131053	139943.8	91481.4

LP250.999	LP251.000	LP251.000	LP251_8.0	LP251.000	LP251.000	LP250.999	LP251.000	LP251.000	LP251.000
105149.5	136815.5	148993.7	108582.6	118343.9	168422.5	88309.74	93034.23	165375.7	146960.9
87854.84	176434.2	181949.1	103570.7	185031	136734.9	107980.4	127819.4	155941.8	158127.2
100768.7	170075.9	143758.5	140424.9	114839.5	136260.2	112644.3	84721.51	161963.7	146483.4
146407.4	201119.9	185329.7	112054.8	115928.2	180919.6	121946.4	93727.4	167977.3	165513.4
116222.7	168572.2	129389.9	106745.8	116837.4	142430.8	162312	90353.99	151932	144790.5

LP251.000	LP251_8.6	LP251.000	LP251.000	LP251.000	LP251.000	LP251.000	LP251.000	LP251.000	LP251.000
145366.1	118684.3	139092.8	114468.1	138460.8	113899.1	138023.1	151082.2	114391.7	154164.2
153459.8	111608.2	145618.6	111240.3	124674.1	112237.3	149068	145476.2	118643.8	133391.5
131545.7	113281.4	154952.3	109891.4	141641.1	119522.7	130602.2	163733.6	122476.1	127418.8
145674.4	111923.7	152271.1	117208.1	147476	118460	150971.1	149754.3	124000.4	119461.7
144177.4	108871.4	171727.6	105820.5	149569.4	121339.3	152961.1	159810.9	136580.3	123233.9

LP251.000	LP251.000	LP251.000	LP251.000	LP251.000	LP251.000	LP251.000	LP251.000	LP251.000	LP251.000
164772.5	105416.2	122478	125403.1	135069	102220.3	113083.3	115965.1	97628	150463.2
171211.5	116436.6	118159.3	127121.7	124486	104537.9	95440.84	107495.7	100097.1	154247.9
144120.5	145441.3	121124.6	128683.1	132530	105011.9	120553.2	123337.1	137130.7	154040.7
171441.6	104200.4	136288.4	134241.3	145504.4	103970.5	110080.8	107594.4	112657.9	151076.2
174618.9	105871	120255.1	134121.2	139905.1	97089.45	108684.7	112841.9	110335.5	155434

LP251.000	LP251.000	LP251.000	LP250.999	LP251.000	LP251.000	LP251.000	LP251_3.5	LP251.000	LP251_3.6
94162.34	128569.1	146033.9	92033.42	133413.2	90030.72	80207.35	85449.96	83160.29	83647.79
88849.64	156353	139822.7	77604.8	132560.6	89969.37	73710.98	77055.03	82355.23	82507.08
99203.27	148277.7	132979.5	90489.91	121127.7	99152.57	74468.86	76339.2	82089.42	81206.82
91983.3	148430	143194.8	89451.09	124791.4	96578.7	75775.22	79934.03	85066.62	81131.3
86247.52	147169.9	141829.4	83976.29	132697.6	92217.67	73178.34	77288.28	84964.76	80148.72

LP251.000	LP251.000	LP251.000	LP251_3.7	LP251.000	LP251.046	LP251.127	LP251.126	LP251.127	LP251.127
95280.97	69922.73	90224.55	72940.5	83313.45	21518.34	68107.6	49914.42	68958.57	66641.2
95312.89	64386.61	91878.44	80828.68	77866.93	21426.17	69018.19	58973.64	70965.96	65795.33
97865.31	73789.24	92118.35	77206.86	85163.49	26071.24	80067.62	54151.28	71092.49	65579.85
99423.51	66738.39	92236.19	77829.69	79184.53	81258.31	75436.56	50556.62	63985.35	58716.25
95121.95	69433.45	95283.06	82336.5	74844.93	79960.39	64872.62	67729.15	72925.65	57856.79

LP251.127	LP251.127	LP251.127	LP251.127	LP251.127	LP251.127	LP251.127	LP251.126	LP251.127	LP251.127
67459	120088.3	81283.81	76369.6	60560.58	81271.49	76996.77	49792.98	50207.67	74546.7
66543.01	124759.6	79044.67	78914.51	59090.76	77051.07	78504.87	50735.87	51494.5	79096.36
66733.65	117075.3	83039.22	75730.38	57647.21	86743.49	75121.49	63657.42	55541.06	76941.57
60647.63	108262.5	79781.27	74081.01	68404.08	73087.63	68366.93	46346.46	55667.38	70211.61
73003.09	115650	73865.26	73181.27	65072.69	81877.79	75655.69	44569.54	50371.88	69970.71

LP251.1277	LP251.1274	LP251.1273	LP251.1278	LP251.1258	LP251.127[illegible]	LP251.1274	LP251.127[illegible]	LP251.1259	LP251.1262
57296.02	55169.01	51756.29	49954.29	55613.22	76817.53	71622.6	61868.93	35366.8	71249.69
57345.11	58221.29	56777.05	57820.43	53036.17	73020.53	63871.97	76361.13	33123.11	72115.01
64795.64	66333.64	55244.4	49869.92	61612.9	73296.57	72386.63	71723.88	38836.41	80244.25
59007.25	56236.46	56602.99	50186.95	65869.67	79203.02	66476.31	67532.08	38958.35	80488.87
56169.21	55429.56	49592.7	50318.14	48438.49	70615.29	59433.45	61799.71	32791.34	69564.37

LP251.127	LP251.126	LP251.125	LP251.127	LP251.128	LP251.128	LP251.128	LP251.127	LP251.127	LP251.128
45586.7	45173.94	57906.35	69195.21	116559.9	98667.41	99967.41	99911.24	82540.57	92704.27
49784.64	51709.29	54663.19	70268	111199.4	103606.9	103540.2	97291.65	86411.44	93224.53
50045.6	49801.26	63723.88	77298.36	139290.7	100015.6	137546.6	95493.76	90204.55	99361.29
49347.34	52870.67	68611.61	67899.76	103529.2	86537.37	108601.8	86841.94	79647.3	93839.92
42685.11	48385.22	54835.16	65509.83	92009.65	87926.22	81963.8	87035.99	77491.67	88857.34

LP251.128	LP251.127	LP251.128	LP251.128	LP251.128	LP251.127	LP251.128	LP251.128	LP251.127	LP251.127
97753.79	67398.41	78428.4	82701.13	69846.46	86911.29	71680.89	66263.22	77279.13	64834.68
90202.46	84191.95	83335.12	79364.45	74658.28	70923.16	77464.07	69622.87	75729.61	62311.7
91376.71	70054.56	81207.43	88510.83	75986.82	70773.16	74339.88	63679.04	71775.23	58328.89
98091.95	89940.85	70114.37	80323.76	80733.17	99783.9	70821.5	73262.16	74130.94	59579.81
79700.65	65106.51	66845.46	77961.21	80826.69	57299.18	72700.92	63443.3	71681.65	56783.34

LP251.128	LP251.128	LP251.128	LP251.128	LP251.128	LP251.164	LP251.164	LP251.164	LP251.164	LP251.164
55168.91	59036.88	71398.64	105844.9	101645.6	68526.71	103348.1	101898.5	91933.81	77555.85
56326.79	57366.19	76450.95	100148.3	102651.4	85910.14	66644.23	99831.64	103663.9	58208.13
55943.56	56290.83	72526.13	86244.27	99015.86	83511.57	75055.14	104409	101480.2	72293.07
54140.55	54273.93	67931.53	95086.81	98044.04	86714.11	74246.51	106006.5	102791.5	68104.43
54807.75	51298.2	72990.25	122377.2	85557.79	97708.74	79230.46	126624.3	107917.9	66789.19

LP251.164	LP251.164	LP251.164	LP251.164	LP251.163	LP251.200	LP251.200	LP251.200	LP251.200	LP251.200
89969.64	66836.94	92732.23	125691.1	47872.59	100503.2	93214.17	75966.43	107343.1	79406.56
92094.03	65663.26	80705.19	121605.9	50713.83	103125.4	94409.05	58666.52	103109.1	78763.6
99256.53	69280.73	88580.55	124674.4	46225.21	80797.41	93761.63	73007.7	73292.67	84162.88
93555.99	70653.8	94610.57	119345.5	47258.43	118839.8	79842.98	62126.62	101110.1	86754.46
104405.7	66446.61	93738.93	123753.5	49138.28	117115.9	104124.1	66891.57	112141	87699.1

LP251.200	LP251.200	LP251.200	LP251.200	LP251.200	LP251.200	LP251.200	LP251.200	LP251.200	LP251.200
76424.21	77025.97	60847.74	70298.83	60262.12	91492.72	70417.78	54643.94	76542.66	69115.1
87501.97	66211.09	59054.14	65932.07	65925.42	101459.7	75668.89	49224.68	61882.44	58127.02
80535.94	76039.96	59265.33	55590.37	67208.28	95619.08	73554.93	51067.8	74983.32	67796.51
62317.55	60651.35	51478.36	55825.93	54114.48	73686.32	70840.1	57585.83	75260.87	72236.63
85775.66	80475.99	55425.08	68333.72	67246.77	110842.7	86290.95	58451	74201.77	71864.96

LP251.201	LP251.200	LP251.200	LP251.200	LP251.200	LP251.200	LP251.200	LP251.200	LP251.200	LP251.200
82669.4	68523.2	89452.59	52685.25	65410.55	73404.44	123071.2	51388.63	89042.58	43861.16
80438.44	71163.6	89135.89	52173.75	73173.77	67768.3	123848	56506.75	90369.51	45024.41
88827.42	68844.29	93608.97	54118.66	66507.56	72526.12	121326.9	51729.5	85122.63	43174.95
81307.67	65511.92	90270.83	48339.58	65826.12	70757.96	126742.6	53528.86	90349.65	42287.79
87442.9	69073.6	99789.6	59659.49	75581.85	75203.35	131460.8	52251.79	90856.84	43918.21

LP251.200	LP251.200	LP251.200	LP251.200	LP251.200	LP251.237	LP251.237	LP251.237	LP251.237	LP252.008
59910.61	85040.94	48688.39	68919.08	53710.14	81861.58	76566.36	82872.77	95070.64	29177.28
52471.43	85466.43	45439.88	67942.69	53493.98	75874.06	82135.77	76751.04	101180.5	43401.99
52551.97	87138.24	47709.5	67459.2	53166.08	83240.29	83596.02	72683.41	93885.87	26647.18
55076.63	84010.39	44621.64	66522.6	57693.72	76839.04	78067.21	66590.31	94585.59	26759.62
56407.04	85870.3	50837.82	70646.62	51831.09	80593.43	86588.25	85432.85	96362.54	18197.13

LP252.159	LP252.159	LP252.159	LP252.159	LP252.159	LP252.159	LP252.159	LP252.159	LP252.159	LP252.159
27193.59	41830.67	48067.35	44821.99	41802.51	32339.45	32471.49	28157.51	32758.8	26752.76
26642	46834.44	42005.37	48010	40284.84	42396.83	30891.79	28111.48	31708.7	24626.74
27081.56	42544.97	45100.27	47357.88	46178.42	38114.87	31646.58	28909.22	35418.5	25470.91
30562.16	38109.05	41035.56	45895.89	35550.82	38479.56	27914.38	33094.35	30967.82	26033.32
24491.26	41284.61	44191.42	47010.81	40695.9	37208.13	30785.86	25482.05	32860.48	23953.58

LP252.159	LP252.159	LP252.159	LP252.159	LP252.159	LP252.159	LP252.159	LP252.159	LP252.159	LP252.159
26332.03	26276.8	32319.74	33434.06	28791.12	31337.53	27411.89	25966.47	17432.67	28108.98
23989.8	32749.53	29992.42	34019.91	26368.03	31267.42	23348.41	26700.4	16520.08	28316.47
21499.51	30813.63	28947.55	31308.48	24165.38	29158.62	26491.92	22430.19	21228.04	23846.24
24890.96	27791.91	30326.58	31149.01	24320.77	31929.94	26639.69	19356.73	18044.65	27248.41
22968.15	28584.42	28672.69	31341.55	24235.98	29951.87	26640.94	27552.75	18241.05	26879.13

LP252.159	LP252.159	LP252.159	LP252.159	LP252.159	LP252.159	LP252.159	LP252.159	LP252.159	LP252.159
21677.05	33473.31	23948.26	28083.84	17189.38	24328.76	26134.97	21591.99	19754.72	22261.99
19541.47	33501.03	15923.45	28525	15855.9	29185.5	22519.49	24711.59	17510.71	22441.95
20796.5	29735.38	20780.15	28621.59	17665.95	23698.14	24331.86	24902.23	17907.56	22690.61
23285.16	25729.31	18765.37	28999.1	16218.56	26790.58	21197.77	22877.92	14601.42	21183.07
19598.58	28264.27	20090.74	23967	16684.42	25415.54	21790.49	26844.66	16996.48	22049.31

LP252.159	LP252.159	LP252.159	LP252.997	LP252.997	LP252.997	LP252.997	LP252.997	LP252.997	LP252.997
23759.86	37185.15	25331.8	84860.01	81935.4	78436.24	102236.1	108632.4	100875.8	95599.49
24324.2	36399.79	26992.54	63878.84	70352.88	84677.77	98211.51	73267.5	92671.29	96994.56
26164.04	38573.72	26936.9	69824.27	60189.67	87327.58	95937.29	57845.28	97330.09	120404.4
23993.61	39950.14	24288.73	66250.53	62865.23	74130.49	96490.18	60262.29	104154.2	108089.6
24368.64	36398.67	27124.65	67550.49	55341.66	120659.2	112703.1	107042.1	88714.09	101952.7

LP252.997	LP252.997	LP252.997	LP252.997	LP252.997	LP252.997	LP252.997	LP252.997	LP252.997	LP252.997
56972.09	67278	81492.62	86512.23	81625.53	67245.24	82826.51	55170.95	95614.1	77410.03
51534.37	72966.71	84434.57	95008.68	72814.61	63045.41	84506.4	62189.68	98374.32	85193.53
66330.09	70734.89	90074.46	88952.27	72421.37	75952.9	78648.74	62550.52	99990.37	79125.3
51590.05	69749.08	89127.17	83702.92	65314.36	63067.82	78551.06	63135.15	96640.69	65795.15
59624.45	91011.01	87876.63	89123.63	77896.95	78052.62	94478.64	62776.98	77241.3	131166.7

LP252.997	LP252.997	LP252.997	LP252.997	LP252.997	LP252.997	LP252.997	LP252.997	LP252.997	LP252.997
75343.61	62810.44	68009.94	50471.28	76624.93	68846.8	62356.01	53513.09	56125.94	57675.49
75875.27	67177.91	68043.69	49946.39	70741.93	64598.22	61976.35	55008.66	56358.32	57965.96
75863.8	65981.19	72595.05	72232.01	74210.04	59771.67	68460.89	54780.57	59435.87	64095.9
84576.92	64870.36	66099.21	48849.42	81649.6	64647.41	62042.48	52087.46	57440.51	58635.5
77118.29	69477.99	63092.19	45848.38	85482.45	83432.99	63473.99	51181.44	53635.16	61398.28

LP252.997	LP252.996	LP252.997	LP252.997	LP252.997	LP252.997	LP252.997	LP252.997	LP252.997	LP252.997
69018.04	51770.56	71655.82	121726.4	105544.2	104563.7	93824.95	113039	78504.21	95490.26
75106.23	48751.14	70427.63	95276.74	113801.6	100510	101463.2	114329.2	98495.53	94785.89
76511.78	57576.31	74782.38	87542.79	105848.4	104503.5	102284.6	109812.8	73131.15	92678.97
65150.62	51870.61	69273.22	113293.2	92177.56	86073.68	99103.51	115308.5	78959.72	88726.59
77668.44	47961.56	75299.31	106828.1	138164	115135.5	88674.82	98139.92	84115.44	111371

LP252.997	LP252.997	LP252.997	LP252.997	LP252.997	LP252.997	LP252.997	LP252.997	LP252.997	LP253.104
89269.17	95119.95	94759.51	75848.59	67544.01	79698.89	105172.5	108708.8	74032.69	53991.55
86047.21	102716	95023.08	98552.29	69326.4	75651.83	105304.1	105384.6	74159.27	50475.01
103224.9	105325.1	97148.82	101854.9	71125.61	74834.98	114986.6	107953.5	80444.05	51696.08
101926.5	105186.6	98026.45	93383.61	68161.77	64106.25	103631.4	104037.8	65382.53	41622.61
101963.1	91799.76	98189.89	104517.1	93028.19	62965.94	107246.2	109589.3	68448.44	56300.94

LP253.104	LP253.104	LP253.104	LP253.104	LP253.104	LP253.104	LP253.104	LP253.104	LP253.104	LP253.104
49590.69	54126.71	58445.8	61094.87	84436.85	73046.18	62533.65	48491.26	90802.85	74251.38
79751.13	80773.69	73923.73	60610.43	84636.58	67012.27	69678.15	49146	67650.3	56432.94
58127.29	62096.94	97955.45	72330.64	54081.44	68235.88	76531.54	61625.88	64341.24	56971.18
86435.37	83285.94	76343.58	65009.35	70104.18	67781.27	89716.73	87380.38	64130.17	87733.69
58276.49	66334.7	69007.88	63599.92	115081	69071.64	59374.75	72798.75	70623.59	58637.19

LP253.104	LP253.104	LP253.104	LP253.104	LP253.104	LP253.104	LP253.104	LP253.104	LP253.104	LP253.104
53910.39	54342.36	54883.79	53532.5	93454.12	69077.27	52206.39	72239.24	52829.57	92935.71
66222.45	62507.57	76652.95	67472.95	75441.13	74809.69	81555.6	73347.84	97029	92202.1
43654.57	74160.28	86038.9	65662.46	79695.91	60180.91	88902.62	53884.13	71816.69	94812.65
69858.62	69406.61	67462.65	49893.5	53654.05	73366.84	69547.87	80531.96	86987.76	101277.9
74788.32	55531.39	50068.02	59809.92	84011.51	86377.02	89381.8	70814.95	117144.9	76581.1

LP253.104	LP253.104	LP253.104	LP253.104	LP253.104	LP253.104	LP253.104	LP253.104	LP253.104	LP253.104
93053.01	61375.55	79282.16	50823.9	61674.3	61121.02	59678.22	59278.31	57946.64	67311.09
80995.44	64973.64	67438.18	67068.68	66165.15	71919.04	51449.63	67533.67	60245	66333.22
57904.01	68257.54	81094.56	55515.75	59089.62	56466.74	38669.82	56004.1	62343.58	66515.63
75147.46	55263.49	62372.21	80931.08	60334.47	68991.18	46575.15	88253.15	56786.4	68867.88
78939.02	71832.32	59330.23	50259.39	57452.23	62570.14	41361.02	63681.59	47562.35	100754.1

LP253.104	LP253.104	LP253.104	LP253.104	LP253.104	LP253.104	LP253.104	LP253.104	LP253.104	LP253.104
79878.62	71922.74	53747.95	70277.52	89251.33	67680.33	61014.05	54137.04	48665.58	72888.3
54437.93	62224.37	62900.78	81979.38	80161.72	58400.46	55207.11	60755.52	58713.26	79061.98
74685.33	83449.39	58339.52	64928.64	73104.72	64449.02	53103.07	68794.54	61881.02	63441.38
80826.67	67680.57	48324.2	47136.1	80128.37	67385.94	56135.17	98299.66	60813.87	78476.57
61785.2	57078.88	68307.69	66381.73	47192.5	45755.8	58964.66	68622.65	68837.56	83628.43

LP253.104[illegible]	LP253.104[illegible]	LP253.104[illegible]	LP253.104[illegible]	LP253.104[illegible]	LP253.104[illegible]	LP253.104[illegible]	LP253.105[illegible]	LP253.104[illegible]	LP253.104[illegible]
48620.33	55445.37	64129.83	78688.61	52980.42	67950.81	75440.27	49255.26	68286.2	51134.96
48152.33	59771.48	62662.88	63534.73	57148.35	74253.5	77682.33	73719.3	54992.71	58950.15
55079.99	55971.33	69542.27	66582.54	57914.3	78712.94	60843.58	50570.13	53902.89	62594.15
48045.62	87011.64	73026.93	53265.57	54883.2	50446.5	90968.14	86855.85	54042.13	63528.38
41327.66	57563.71	67538.46	65630.85	61365.98	88670.92	60539.36	59187.48	72080.25	56027.43

LP253.104[illegible]	LP253.104[illegible]	LP253.104[illegible]	LP253.104[illegible]	LP253.104[illegible]	LP253.104[illegible]	LP253.104[illegible]	LP253.104[illegible]	LP253.104[illegible]	LP253.104[illegible]
65401.95	55511.35	32500.02	56020.74	62522.91	73506.55	60780.62	47883.24	70961.36	59722.64
68228.8	62975.93	40747.54	70549.23	63734.64	72808.12	56946.48	51589.08	79437.85	65741.9
75391.14	105515.2	46339.67	72795.49	74629.15	82108.91	44660.53	44316.89	78445.9	63493.03
64827.12	66186.54	42711.26	61341.98	60234.44	70979.33	63971.59	48580.93	57658.06	58476.95
61559.65	53528.58	28546.03	67093.8	53508.66	85513.41	36809.64	35449.57	74579.41	59190.85

LP253.104	LP253.104	LP253.104	LP253.104	LP253.104	LP253.104	LP253.104	LP253.104	LP253.104	LP253.104
76144.82	99044.91	74732.24	63055.2	38394.14	98243.12	54283.82	83760.21	43378.12	50654.43
94293.21	101304	70489.61	70556.7	42529.15	108115.7	51620.59	79062.02	42080.73	44115.26
80248.22	97136.83	70430.4	78628.48	46288.03	72765.9	54460.51	71075.51	48607.31	52038.31
87624.64	67639.98	74059.83	82020.36	37949.6	57320.69	46950.37	69325.27	53275.67	53455.94
64359.46	70517.26	58633.95	82776.46	34638.51	117332.5	43780.93	61258.91	39531.78	50053.62

LP253.104	LP253.104	LP253.104	LP253.104	LP253.104	LP253.104	LP253.104	LP253.104	LP253.142	LP253.142
63066.69	54235.92	64383.11	47117.87	40881.08	52587.76	60948.29	108274.6	84843.38	85829.64
56515.07	58565.08	55807.59	42427.32	51695	57883.46	52321.9	109741.6	88540.19	88095.72
76038.85	62393.97	68688.26	42786.58	53997.35	52003.11	64770.8	106305.1	84654.61	87123.97
59075.48	64325.53	68325.4	46978.65	53947.3	57772.06	61563.24	100402.1	80606.39	82040.82
51349.53	58257.56	64934.59	43895.15	37065.39	41653.4	57299.55	88083.76	82846.21	85066.8

LP253.142	LP253.142	LP253.143	LP253.143	LP253.143	LP253.143	LP253.143	LP253.143	LP253.143	LP253.143
72723.85	66500.98	80325.29	65274.22	117354	83151.48	64696.61	64393.56	70283.02	68553.76
78146.87	73104.62	79147.47	69132.42	142972.7	92249.4	62270.54	62737.51	66236.32	72355.67
67218.93	72434.67	75979.42	61085.05	122714.2	90152.44	58516.48	55381.32	71351.42	68861.26
76636.41	70056.57	76148.78	74725.27	112366.4	95762.69	58820.07	71099.27	71581.27	70217.41
67860.32	70519.16	66386.85	89063.38	100889.6	99656.68	66530.66	62050.33	76350.38	75881.99

LP253.143	LP253.143	LP253.143	LP253.142	LP253.143	LP253.143	LP253.143	LP253.143	LP253.143	LP253.143
76823.66	95426.47	81413.04	54488.48	90342.11	58319.6	68664.18	77408.63	77596.89	53959.05
75317.46	92682.13	85725.5	51977.25	89032.44	56951.53	66374.2	69802.67	72402.8	55853.81
72377.28	94475.15	87848.08	52482.04	87370.59	58483.63	61911.87	69632.87	75587.89	58201.62
88049.35	86744.68	82047.92	50943.49	88881.43	56261.33	77418.02	67626.11	71952.41	49477.62
75776.64	93202.19	81527.58	50995.46	89127.19	79147.73	65453.74	75252.81	73477.28	55285.74

LP253.143	LP253.143	LP253.143	LP253.142	LP253.143	LP253.143	LP253.142	LP253.143	LP253.143	LP253.162
75310.29	73484.43	49817.78	85507.13	50337.19	86827.38	72883.16	76394.69	100362	75048.65
68955.43	65584.66	54098.68	94947.5	50907.09	91937.65	65918.08	75527.8	107362	70968.72
66020.36	64723.37	46885.3	89202.63	54357.04	84673.2	75775.45	76322.38	96225.03	70557.08
72752.17	66273.68	52065.11	83702.88	54327.02	90312.88	66718.94	76961.58	101073.5	76642.48
72615	65391.07	49485.21	88414.1	54539.01	84314.73	65853.82	78505.39	105411.9	82696.72

LP253.18_	LP253.179	LP253.179	LP253.18_	LP253.179	LP253.179	LP253.179	LP253.179	LP253.179	LP253.179
102812.6	110353.7	82279.2	108235.4	78475.2	104075.1	78621.43	102928.7	55252.59	108479.1
82402.51	100877	81089.03	109143.4	88648.42	107428.5	72411.89	109932.3	52384.82	107601.5
98533.47	94967.1	87983.96	107089.4	89693.97	100350.2	78008.73	100356.6	60355.51	117734.2
104499.7	109382.8	92923.25	115354.2	88702.64	97188.87	80375.17	103146.9	56763.54	115653.4
112049.9	121869.9	96959.4	136350.2	98488.79	111034.6	90569.81	115470.5	62867.44	128891.3

LP253.179	LP253.179	LP253.179	LP253.180	LP253.180	LP253.18_	LP253.179	LP253.18_	LP253.18_	LP253.179
101586.9	91012.43	103947.7	69660.68	84243.71	86065.55	87223.12	127497.8	101923.9	85984.31
103528.3	92051.1	98273.84	68292.19	81140.58	85645.13	94246.22	121806	96936.69	83004.54
111857.9	92961.19	97796.52	70760.12	83466.95	94751.18	85554.19	124072.3	102021.2	85361
111395.4	93355.7	106036.4	75088.19	85742.74	95821.56	94540.08	125801.7	108922.8	92789.87
119913.6	101694.1	111547.3	83070.65	92414.86	105162.9	107056.7	149962	116625.1	103360.4

LP253.179	LP253.18_	LP253.179	LP253.179	LP253.179	LP253.18_	LP253.180	LP253.179	LP253.179	LP253.179
94124.99	94480.16	86636.14	104871.3	91069.05	94714.53	107639.7	97058.51	101869.9	103720.6
100273.7	86900.7	93510.04	101102.3	95315.29	103854.8	107043.5	101086.1	91688.78	106103.2
101523.8	92786.05	86303.72	100106.6	91733.24	113136.2	119821.8	107584.7	97197.5	97590.83
95959.26	98809.94	86628.42	112511	102340.8	107873.1	117696.6	103098.9	105118	102584.9
111131	104641.1	104666.6	126695.7	103059.8	123445.8	132696.9	112893.5	107043.9	115256.6

LP253.179	LP253.179	LP253.179	LP253.179	LP253.179	LP253.216	LP253.216	LP253.216	LP253.216	LP253.216
72051.73	104563.9	75287.72	80512.48	70959	83762.44	113979.6	98906.55	85352.31	97775.34
73679.56	97310.4	65259.76	87346.64	77015.76	88298.91	119170.5	107192.3	81315.97	95928.1
79560.12	109004.8	80117.35	84515.51	79589.99	71215.71	110214.1	97985	65630.03	110714.5
76875.15	108217.2	75905	88288.98	85585.7	91826.59	122526.9	102398.4	104945.3	103370.8
85863.4	122657.1	76331.65	95417.45	89167.96	85326.15	99390.23	92660.24	103695	78218.39

LP253.216	LP253.216	LP253.216	LP253.216	LP253.216	LP253.216	LP253.216	LP253.216	LP253.216	LP253.215
101414.7	96789.41	102790.5	88247.77	88924.04	81892.81	109665.1	98108.56	81083.93	86332.68
100638.5	97050.92	96098.2	86970.22	77451.91	79672.5	123885.1	92078.55	72067.35	85761.15
92727.53	97318.78	106750.4	77549.76	95991.41	84559.84	119642.3	96122.11	73132.48	87211.73
91992.5	105928.8	76926.95	88803.39	62548.15	88172.96	118690.5	68688.78	48765.73	87707.15
77101.69	124650.2	89779.73	98534.98	96990.43	86052.77	123768.3	110929.2	82594.39	63783.65

LP253.216	LP253.216	LP253.216	LP253.216	LP253.217	LP253.216	LP253.216	LP253.216	LP253.216	LP253.216
80606.5	91783.95	63853.24	62233.32	77458.89	107872.5	88739.55	60960.33	81607.46	85428.29
84890.2	91343.19	66849.53	62077.6	66897.44	108364	87290.96	61128.79	89306.98	84864.83
84323.7	97284.05	63780.32	63604.03	66436.73	108264.2	98125.81	60979.75	57946.17	84171.37
82004.41	94005.82	63763.4	69754.23	72245.91	110840.6	100885.8	57254.55	87226.46	83562.39
92481.8	109591.7	80516.37	64452.75	74430.81	119203.1	67097.69	70529.59	99832.16	87679.05

LP253.216	LP253.216	LP253.216	LP253.216	LP253.216	LP253.216	LP253.218	LP253.252	LP253.252	LP253.252
115958.6	108166.7	69696.53	81287.69	88901.78	83688.31	67680.42	29008.63	32032.42	36174.84
103002.1	103101.3	70230.39	72300.3	85195.99	89702.53	72422.81	25721.98	33827.02	24248.95
109495.6	111352.4	79556.1	78859.95	88031.8	82664.91	70984.38	28003.77	32745.54	26787.92
114129.8	116147.3	69210.78	78583.95	84762.8	86103.1	77594.79	31186.09	30045.02	24668.99
119905	124703.3	86103.28	88100.79	103558.2	99300.32	79306.33	25750.03	34654.19	27212.79

LP253.252	LP253.253	LP253.252	LP253.252	LP254.175	LP254.174	LP254.183	LP254.183	LP254.247	LP255.084
28661.17	28667.71	33592.74	32124.54	23743	31911	13068.71	17146.48	21334.63	107322.7
30162.8	31484.66	31798.55	24854.25	27027.58	34481.11	12704.33	18052.69	21095.86	108818
29916.83	30660.34	33288.45	27776.75	27209.5	35246.85	10949.29	16384.42	19957.55	104970.4
27966.27	31316.64	30229.76	33286.02	24675.79	27991.77	11563.37	15109.84	18721.06	114134.1
35138.74	31547.46	34676.53	28448.05	30337.78	28102.97	12936.78	17860.44	18275.93	89488.59

LP255.120	LP255.120	LP255.120	LP255.120	LP255.120	LP255.120	LP255.120	LP255.120	LP255.120	LP255.120
129882.6	141038.5	105498.3	121925.6	100569.8	79283.14	104348.5	118638.5	112863.1	99505.36
137323.5	88455.9	133885.4	117052.2	103741.1	103774.7	99333.97	131445.8	110391	82066.24
116186.9	102671.7	113688.8	148592.3	91633.9	103561.4	164634	183484.5	108208.1	120444.3
79296.2	113045.4	121754	142283.9	145432.9	84203.31	100249	156513.8	95700.02	78191.81
81640.55	103486	107757.5	126287.8	95650.21	74200.23	97461.87	100987.3	111794.6	91982.6

LP255.120	LP255.120	LP255.120	LP255.120	LP255.120	LP255.120	LP255.120	LP255.120	LP255.120	LP255.120
104563.6	108884.1	96005.23	118589.2	130502.5	107609.3	121801.8	124356.7	89459.92	88317.21
101062.3	111335.3	88626.57	123981.2	85107.77	104532.2	119392.3	110214.8	95266.34	60555.41
98038.57	108638.6	72581.45	125467.5	112238.5	112554.5	140792.7	164173	91526.2	64485.59
91982.04	101658.7	85780.8	134155.4	87371.69	110421.3	157174.8	122059.8	143338.1	63593.39
101366.5	99982.22	92090.66	126448.3	108865.1	118015.8	109667.2	112379.8	88409.69	72903.59

LP255.120	LP255.120	LP255.120	LP255.120	LP255.120	LP255.120	LP255.120	LP255.120	LP255.120	LP255.120
95648	121575.5	99762.95	101630.3	114753.4	96832.29	125514	94823.19	101669	109258.9
97608.5	176696	95587.6	117262.7	111085.5	95824.8	136568.4	136221.8	100699.2	124223.8
103937	122712.9	111388.4	84202.51	133858.9	88827.12	130849.1	87729.39	107144	108578
117319.2	181863.2	120123.8	137162.9	139784.6	108193.5	152343.1	84127.82	103342.4	102586.5
86396.24	102480.7	96678.56	92167.13	118068.8	87808.43	122590.3	73605.89	104727.4	110775.1

LP255.120	LP255.120	LP255.120	LP255.120	LP255.120	LP255.120	LP255.120	LP255.120	LP255.120	LP255.120
114841.1	89170.56	91814.5	110152.4	125461.7	111820.6	99600	108485.9	171417	108381.3
118501.8	79625.28	90058.41	105175.3	126000	102164.1	93617.39	108740.3	155914.6	104595.1
121848.9	81488.74	99730.84	121804.8	121340.5	149277	118330.2	111205	210788.1	112689.8
109104.4	81053.29	80423.35	98395.35	119288.2	107526.4	111905	132862.6	166884	109904.9
105771.4	75652.92	90718.3	106955.2	104580.9	112579.6	102613.6	125143.3	161103.4	109280.2

LP255.120	LP255.120	LP255.120	LP255.120	LP255.120	LP255.120	LP255.120	LP255.120	LP255.120	LP255.120
94686.34	79425.69	121597.8	111732.8	107867	120648.6	92512.94	84274.26	107888	92597.05
84049.38	77746.53	165769.8	121367	112414	113218.4	95211.6	79769.08	122653.1	80743.25
91607.36	111038.5	108139.3	167962.8	107227.3	119593.4	100684.1	87372.65	101469.9	82711.62
125392.3	95090.92	122682	128453.1	97605.6	136158.9	103407.4	87924.84	115323.3	80484.85
72234.16	67282.52	127734	98667.84	100946.6	116243.1	96239.73	76146.7	94017.88	80397.04

LP255.120	LP255.120	LP255.120	LP255.120	LP255.120	LP255.120	LP255.120	LP255.120	LP255.120	LP255.120
154367.1	85012.19	96196.81	85295.28	111147.1	70028.73	99686.25	102431.9	107819.9	106068.3
127794	79341.25	137786.7	78091.41	98237.86	66010.31	143866.5	106120.8	93953.85	99106.69
150381.4	78108.94	97282.07	75864.86	112669.9	72502.08	125146.7	90234.79	105201.9	121194.7
169817.9	95282.37	128801.6	82116.44	102987.1	77360.09	117838	98157.72	102826.2	100522
132649.3	82323.01	102057.7	73327.54	94119.95	66709.07	92016.76	93319.32	93103.97	99359.83

LP255.120	LP255.120	LP255.120	LP255.120	LP255.120	LP255.120	LP255.120	LP255.120	LP255.159	LP255.159
86860.68	130991.3	114808.1	106125.6	68841.47	70372.06	93747.25	90210.03	157018.3	187778.4
87480.41	132641.5	109518.1	92648.25	73490.46	75861.53	94625.26	89927.53	186172.5	181423.9
105568	146278.1	124372.1	110018.7	67308.64	82711.21	91630.42	95963.78	160241.5	188419.7
132506.7	144784.8	138707.4	112360	79271.25	79469.46	100872.5	93068.1	164978.9	195367.6
80221.72	129147.8	109946.8	101470.7	74933.46	71997.03	89489.01	89154.79	164854.6	202902.6

LP255.158	LP255.159	LP255.159	LP255.158	LP255.159	LP255.159	LP255.159	LP255.159	LP255.159	LP255.159
109494.9	165064.4	180797.1	90191.86	135285.9	107070.2	141640.7	168056.2	136734	91757.12
104819.6	144159.6	127682.9	98691.81	143315.6	112075.5	144781.6	158548.2	129902.4	80985.79
103806.2	157950.5	182581.7	104474.5	142971	117787.4	153129.9	157540	132718.7	80959.11
104770	166905.4	188566.6	102310.2	150045.8	119761.1	152355.8	165832.9	146644	89550.11
115038.4	172420.2	194244.2	97358.65	142913.5	119731.5	143719.1	182290.1	146934.8	82770.01

LP255.159	LP255.174	LP255.174	LP255.174	LP255.174	LP255.174	LP255.195	LP255.195	LP255.195	LP255.195
109255.8	39423.29	46468.69	68373.91	39720.93	131929.4	206512.2	151386.9	138453.6	123684.1
107194	149435	144369.8	174393	129333.4	130287.2	189488.3	141544.7	136927.4	120087.3
113404.7	148710.8	147965.3	189254.1	141838.2	133958.6	198428.6	132725.9	137483.7	111631.1
111816.3	137549.8	151066.4	192716.1	137965.8	138739.8	195722.6	141506.9	142372.6	110789.9
124638.3	149010.6	170003.4	195815.2	137508.2	146731.5	202817.9	133836.3	160990	123147.9

LP255.195	LP255.195	LP255.195	LP255.195	LP255.195	LP255.195	LP255.196	LP255.195	LP255.195	LP255.195
200508.2	101326.3	208518	125181.3	118625.5	116735.2	115299	112029	106233.6	130798.9
216969	103939.6	208967.1	140554.1	116396	128742.6	127448.6	125285.3	111873.5	113105.3
228440.4	101754.6	199399.8	99140.82	63799.7	120634.6	110973.7	120268.4	116434.6	127066.5
112406.5	102128.9	196749.7	135264.7	127791.6	125445	106919.5	116311.2	118197	143794.4
247918.9	109048.7	205093.2	161091.1	137895.7	130155.4	110802.4	131416.6	121927.8	146589

LP255.195	LP255.195	LP255.195	LP255.195	LP255.196	LP255.195	LP255.195	LP255.195	LP255.231	LP255.232
126342	147297.8	154964.6	185293.9	176092.6	99116.41	100523.1	155181.7	117516.3	132596.3
127428.5	159898.4	156638.1	202459	173781.6	89156.52	105410.7	153574.2	136624.9	143574
130689.1	162423.4	163413.5	197395.3	182791.4	95280.86	107450.9	147100.9	135374.6	145652.4
129127.1	167027.7	148669.8	207935.3	193600.4	103786.8	98052.4	163844.2	125616.9	141000
136588.3	168115.7	166845.2	207125.3	199205.1	100811.2	102964.8	77257.88	141338.9	148065

LP255.232	LP255.231	LP255.231	LP255.231	LP255.232	LP255.232	LP255.231	LP255.231	LP255.231	LP255.232
171335.9	109808	142883.2	199354.9	110165.8	139714.4	138770.3	119456	132927.6	145833.6
159030.5	113593.6	150199.5	189376.2	113606.5	129322.6	152561.2	113963.7	142561.7	150159.3
170316.3	119581.8	140452.6	195821.8	110946.2	129999.7	148756.2	117691.8	140923.1	147877
174120.1	111162.7	133150.2	193394.3	114969.8	147537.8	140491.8	127077.1	145368.2	149833.3
196167.7	130010	147564.5	208802.1	122165.7	142185.2	147473.4	124104.4	156756.5	154526

LP255.232	LP255.232	LP255.232	LP255.232	LP255.232	LP255.232	LP255.232	LP255.232	LP255.231	LP255.232
123742	159162.3	108388.3	117095.7	91705.21	166990.6	211186.9	107043.9	106786.1	98885.28
124791.3	157864	99694.54	114284.6	95687.37	162190.9	214437.6	107566.9	107710.8	106141.7
136955.9	165865.2	108579.3	115769.2	109162	164618.2	221914.9	105658.4	105166.3	106175.1
139131.8	166987.4	116374.5	122848.1	106952	166992.5	221374.9	117103.4	110131.7	109161
142151.4	168607.7	116840.3	118556.6	100069.4	187776.3	232946.2	116453.2	111022.1	109521.1

LP255.232	LP255.232	LP255.232	LP255.232	LP255.232	LP255.232	LP255.231	LP255.231	LP256.087	LP256.154
115880.6	129072.6	71750.92	152167.4	132413.1	155011.3	128006.9	143287.1	8954.049	35525.1
110264.8	129067.2	70230.63	158773.2	134953.7	157541.4	133355.1	141976.7	11400.97	38057.01
112943.7	123659.6	81971.11	160689	135616.9	157693.6	136478.5	148307.6	15035.28	33218.95
115262.8	137615.4	78660.63	168397	141806.9	161317	139231.6	153677.2	11630.95	38872.4
123693.8	145571.3	77442.88	176245.3	148697.3	169971.1	146313.3	161426.7	8116.187	38450.56

LP256.235[illegible]	LP256.235[illegible]	LP256.263[illegible]	LP256.263[illegible]	LP256.263[illegible]	LP256.263[illegible]	LP256.263[illegible]	LP256.263[illegible]	LP256.263[illegible]	LP256.263[illegible]
46342.41	42377.05	245511.7	155132.9	140015.9	190281.5	178469.4	213367.6	113537.6	168470.8
44235.49	42430.01	168827.5	203924.5	108314.4	177603.4	180069.2	151327.4	118153.6	152511.7
50709.23	41439.51	139998.9	158269.6	155402.1	181274.9	192184.5	157953.7	113191.5	163566.5
47100.82	46150.56	138984.5	164103.2	176433.8	172293.8	177322.7	148161.8	103908.6	167813.1
50937.78	45737.26	156097.4	152743.9	176901.2	180459.8	163594.1	155232.3	111206.6	157939.6

LP256.263[illegible]	LP256.263[illegible]	LP256.263[illegible]	LP256.263[illegible]	LP256.263[illegible]	LP256.263[illegible]	LP256.263[illegible]	LP256.263[illegible]	LP256.263[illegible]	LP256.263[illegible]
73946.78	142465.8	130247.3	90682.5	93610.31	96582.84	79919.19	118428.2	102170.8	127237.6
88798.81	123661.7	122692.2	85279.6	92697.54	94999.62	64808.08	113588.4	95195.7	117853.5
85801.48	130274.2	120040.6	82668.59	99649.93	104095.5	77555.34	107865	96414.76	130293.7
85023.48	132330	122455.5	90949.43	103222.8	97944.86	73722.18	111985.5	101406.6	122968.7
93645.82	183340.8	121066.5	132069.8	98439.55	105586.5	78897.05	78562.28	103663.7	121614.4

LP256.263	LP256.263	LP256.263	LP256.263	LP256.263	LP256.263	LP256.263	LP256.263	LP256.263	LP256.263
107672.1	90097.07	115108.5	117376	108940.5	103105.5	117488.7	130902.6	107538.8	73614.01
107751.8	82570.4	107303	116396.3	110940.5	105807.1	132672.8	132385.5	113181.1	78432.14
101664.8	91262.68	115721.4	121386.4	113348.9	110399.4	115010.6	124795.1	121224.8	68216.29
106043	82394.48	105152.6	114548.2	117753.2	105181.5	120969.4	121384.8	109479.3	69520.8
107907.3	98840.9	110210.2	108734.6	109052.8	100335.4	122025.4	124143.2	106655.6	77803.96

LP256.263[illegible]	LP256.263[illegible]	LP256.263[illegible]	LP256.263[illegible]	LP256.263[illegible]	LP256.263[illegible]	LP256.263[illegible]	LP256.263[illegible]	LP256.263[illegible]	LP256.263[illegible]
96761.89	78075.99	128798.1	84876.38	101477.9	84222.92	128874.2	125232.9	79074.57	118601.7
93220.93	76200.68	142111.4	90371.24	106111.1	77072.86	115113	149291.6	93775.03	122522.9
99243.21	70589.53	147662.3	94795.09	106918.6	84788.37	127586.8	139004.8	78733.41	116811.7
92260.41	76546.59	136918.6	89377.39	101253.5	77046.6	117433.2	123415.9	77755.76	119242.5
84640.3	79954.25	144895.2	83647.2	99998.01	73396.98	117352.5	123679	73503.66	123397.2

LP256.263	LP256.263	LP256.263	LP256.263	LP256.263	LP256.263	LP256.263	LP256.263	LP256.263	LP256.263
113477.7	104886.8	56336.45	103042.9	72665.28	58661.82	74138.31	78458.27	83458.38	99061.64
118741.9	105266.8	70459.59	99189.18	78554.57	64155.86	71935.43	77869.64	83132.83	104461.6
113509.3	113228.2	58285.88	102803.7	75165.94	65502.45	79730.1	80628.95	77880.13	103535.4
108849	102027.6	57807.58	99941.41	73788.41	70860.69	74632.93	66679.19	71956.55	95128.32
109447.9	109780.6	58624.43	104408.9	76311.37	65001.13	73043.27	77315.93	76154.47	98898.4

LP256.263	LP256.263	LP256.263	LP256.263	LP256.263	LP257.137	LP257.138	LP257.138	LP257.138	LP257.138
135517.6	90541.91	65348.63	132409.9	81027.79	31383.54	46964.92	34398.43	34069.02	45295.93
136178.7	97936.84	63559.38	125246.1	83526.97	38196.49	49965.08	39079.26	34259.99	51693.3
135678.6	101463.1	61573.92	120934.5	84305.35	30739.15	46232.68	34308.17	37846.43	48909.6
134654.1	92828.16	60463.21	133860.8	86730.82	33793.01	45053.18	34681.58	36795.21	50159.52
129645.9	88489.32	68545.8	132980.5	70068.3	34159.74	47402.78	38454.79	36351.41	52071.08

LP257.137	LP257.137	LP257.138	LP257.174	LP257.174	LP257.174	LP257.174	LP257.174	LP257.211	LP257.211
38183.34	38907.07	50657.76	44275.13	27288.33	41129.43	31976.68	48828.83	35201.49	38021.52
31766.41	37623.66	52299.77	41975.49	21580.83	37814.44	32039.17	49417.18	28066.65	36176.28
37741.16	34419.18	45234.23	45772.44	26914.27	40238.84	31067.64	50892.05	32921.92	42475.43
37380.02	35630.85	54022.87	43423.52	29206.01	42637.48	32433.24	52614.85	26126.22	31400.74
32584.99	34674.67	50026.37	43425.56	29649.5	37043.92	30505.06	55001.71	34356.25	38015.62

LP257.211	LP257.247	LP257.247	LP257.247	LP257.247	LP257.247	LP257.247	LP257.266	LP257.267	LP257.266
31602.19	45837.64	24301.18	26005.95	36553.15	25888.83	62633.6	30316.4	27130.87	28863.89
32737.81	47761.66	25267.88	26419.34	23600.44	33010.88	40179.3	26505.36	42735.15	36644.22
34422.95	53890.37	21506.25	29702.01	31045.07	31988.08	48009.71	29056.25	31486.95	30423.35
26686.36	60091.18	22709.59	28068.08	28419.43	35635.93	50085.45	25667.8	32680.8	29575.19
30249.05	55997.01	13922.54	28766.09	29182.74	34721.31	46740.23	34046.03	27450.28	25422.84

LP257.266	LP258.097	LP258.097	LP258.097	LP258.097	LP258.097	LP258.097	LP258.097	LP258.097	LP258.097
29074.48	70997.7	45761.45	36721.92	39063.19	46796.52	20064.45	27563.45	21452.83	29902.96
28360.16	45314.54	65437.77	35719.47	40911.43	57865.18	19597.79	25613.47	22352.72	31582.94
24620.43	48879.26	63931.62	39622.82	40928.68	56257	19292.54	25135.75	21576.24	31077.07
23897.76	51265.53	73153.4	29593.26	33546.81	50955.1	19156.61	22301.31	22589.82	27241.01
30135.77	50292.33	62811.05	34553.89	40798.14	47009.55	19898.58	25135.33	20750.16	28330.25

LP258.098	LP258.097	LP258.097	LP258.098	LP258.097	LP258.098	LP258.098	LP258.098	LP258.098	LP258.097
124816.2	102198.3	120540.2	74509.35	94405.24	79905.23	80831.07	104559.1	82798.85	75885.47
88605.67	86033.23	100085.2	78766.14	86195.08	81764	77655.49	103107.6	80956.27	78995.39
129365.2	113177.8	133722.7	100297.5	75458.74	75334.54	92223.99	93196.67	100562.5	84015.45
92049.69	102312.9	81635.05	82034.84	99373.84	75803.53	75109.73	76975.1	109045.1	93452.36
83651	124938.4	90630.35	72622.15	95471.14	90916.21	91849.13	86326.29	81996.72	90205.83

LP258.097	LP258.098	LP258.098	LP258.098	LP258.097	LP258.097	LP258.097	LP258.098	LP258.097	LP258.097
88190.75	82759.09	115856.8	68483.5	39109.57	58918.2	87536.74	72104.83	62413.51	84860.45
64508.72	87814.06	88603.59	75079.81	47532.34	52984.07	81578.18	77364.95	56493.96	86153.24
73921.99	77707.43	81813.1	124904.9	43615.29	60353.57	106214.7	90836.7	60340.8	99053.81
83470.13	70362.17	92571.1	68240.26	43702.52	65598.74	77938.78	84489.73	51211.01	104563.4
58250.36	76503.71	112660.9	83379.02	46625.97	54519.29	109322.9	92123.8	69360.61	79679.13

LP258.097	LP258.098	LP258.098	LP258.097	LP258.098	LP258.098	LP258.098	LP258.097	LP258.097	LP258.097
73316.93	71807.87	61476.5	54267.85	80777.86	82382.01	58191.59	62166.93	64228.13	51902.86
72101.32	61331.14	56846.36	49132.98	77595.91	91004.71	60336.93	55228.64	59335.86	51367.48
64413.53	70213.4	64247.55	56176.02	100432.4	93375.98	66993.94	56228.6	55823.09	63791.8
93959.3	58054.64	103112.6	61708.32	71163.72	78597.54	91384.74	43384.1	65516.04	61299.01
68551.45	76476.73	54133.73	71071.74	106291.6	88241.41	56670.62	62464.78	54710.59	57173.36

LP258.098	LP258.098	LP258.098	LP258.098	LP258.097	LP258.097	LP258.097	LP258.098	LP258.097	LP258.098
29034.31	81036.14	44144.36	45498.92	47748.42	42232.82	37683.52	67244.24	29511.34	27459.84
33226.02	70490.13	45470.52	38643.86	63154.51	44848.02	39596.29	67186.07	34379.63	29780.76
34690.62	53673.67	46038.19	44027.97	54637.38	45458.26	38456.4	70874.02	36059.1	33937.19
41087.98	101329.5	42190.02	49555.01	63619.13	37045.79	37570.42	52714.07	38267.62	30657.77
26321.02	71149.94	44327.74	42337.02	55551.05	44731.07	37886.2	66352.31	29590.97	31719.43

LP258.097!	LP258.097!	LP258.097!	LP258.098	LP258.097!	LP258.098	LP258.097!	LP258.098	LP258.098	LP258.098
51475.33	35224.35	41923.47	24945.73	31252.18	31713.05	37351.53	33402.54	26993.66	54223.69
46574.73	34330.97	40640.99	22227.41	30352.85	27441.64	43664.26	30996.78	27941.72	57813.65
42228.65	34729.72	38840.93	23969.69	30318.73	34216.21	46954.3	33945.74	24862.97	58680.28
46100.63	33688.24	31079.71	23688.14	32362.76	42079.65	38061.23	32387.78	26820.1	67726.36
45792.16	35969.32	38591.56	24623.7	30563.34	29779.26	40502.68	38932.63	24345.05	50922.72

LP258.097	LP258.097	LP258.097	LP258.098	LP258.098	LP258.098	LP258.097	LP258.098	LP258.097	LP258.097
43946.52	20790.12	19853.65	41833.81	45739.75	35757.64	34623.09	48028.02	30502.35	20317.04
37048.95	24462.4	17745.24	47786.52	45775.62	36354.91	32735.45	40407.17	28890.74	23263.86
40850.92	18554.73	18501.45	46142.76	45471.73	34495.12	29202.1	43388.33	29520.66	18958.57
37080.42	19492.38	15625.55	44346.95	49426.14	35003.45	33897.21	46039.89	28381.84	19816.26
37138.86	19510.42	17436.67	49239.24	43596.24	32160.98	23593.81	38734.57	32245.46	21132.68

LP258.097	LP258.097	LP258.097	LP258.097	LP258.097	LP258.097	LP258.097	LP258.098	LP258.243	LP258.243
17876.62	31817.35	26758.08	32168.72	17441.79	25179.43	17894	21209.19	62566.19	32429.62
14163.38	37508.24	29068.8	31516.03	18892.42	25598.95	17262.39	19372.57	59676.99	29857.68
18977.99	36544.95	29244.62	33232.19	18189.35	25571.73	17557.15	22828.45	31080.35	28726.54
17074.77	38555.74	30788.98	31418.88	15928.02	30358.84	20172.87	20741.84	41538.72	28171.71
15227.32	28882.39	30223.18	31109.95	20642.53	23701.43	15719.41	22333.05	42339.15	29583.3

LP258.243	LP258.243	LP258.279	LP258.279	LP259.066	LP259.066	LP259.066	LP259.154	LP259.154	LP259.154
29221.54	31787.24	49540.12	46380.79	28230.58	24615.88	21794.05	161031.8	186874.4	189797.3
32253.35	29814.95	56409.35	42423.77	28151.31	30390.66	18776.95	148105.2	202491	251692.2
33481.65	29735.01	48338.85	41660.39	26756.1	30456.23	19084.06	159280.8	190305.9	190085.9
35589.84	27928.54	48317.82	36402.97	27942.81	29070.67	17248.11	138299.1	178728.3	190807.4
30043.1	27655.14	50874.29	37431.96	25785.72	28890.58	19323.21	145784.8	144287.3	195012.9

LP259.154	LP259.154	LP259.154	LP259.154	LP259.154	LP259.154	LP259.154	LP259.154	LP259.154	LP259.154
197850	143278.4	184536.9	126878.4	167284.8	132986.8	103061.2	87433.58	126643.6	117750
186446.3	139864.3	194280	111144.3	179372.7	132932.6	131277.5	97767.4	153002.5	90564.77
172456.2	139316.7	185394.2	129668.3	163395.5	138600.2	94950.02	101601.4	129583.6	114727.6
200619	120862.8	173735	119645.3	150536.2	122902.7	97929.76	92033.39	124167.1	110509.4
185226.7	116256.8	170492.8	120744.7	163352.2	132532.1	98991.38	94224.49	114645.4	117060.1

LP259.154[illegible]	LP259.154[illegible]	LP259.154[illegible]	LP259.154[illegible]	LP259.154[illegible]	LP259.154[illegible]	LP259.154	LP259.153[illegible]	LP259.153[illegible]	LP259.153[illegible]
127753	121252.8	123882.9	105613.3	127238.8	83701.37	116618.6	102105.9	104392.1	60756.18
146464.3	90604.5	103885.6	102251.5	144339.5	77901.44	110417.8	98781.08	138627.2	58634.51
136489	119415.7	140528.2	98393.17	128865.3	77244.18	117254.2	101484.8	106926.8	57905.2
116298.2	110258.3	136425.5	93551.42	132309.9	79316.36	114429.4	93363.91	97457.65	59424.52
123451	120543.2	133134	97101.83	140480.3	74480.08	117328.4	93523.42	103195.5	60509.56

LP259.154	LP259.154	LP259.154	LP259.154	LP259.154	LP259.153	LP259.154	LP259.153	LP259.154	LP259.154
108169.3	65028.13	89944.89	87727.44	93449	71175.78	76475.23	99811.22	80540.16	126979.2
92996.01	64771.98	106551.7	82326.75	81081.84	83971.42	82263.45	95132.88	88022.5	125066.3
109943.8	72852.08	87200.78	71798.59	89368.48	84459.19	76465.53	108479.8	78867.21	122754.4
105595.3	63602.51	87870.11	78862.58	81413.2	77652.87	73128.81	99958.5	74357.15	120161
100816.1	58651.22	82133.31	81211.76	91810.9	71808.87	74520.92	91706.56	79539.39	119235.4

LP259.153	LP259.153	LP259.154	LP259.154	LP259.154	LP259.154	LP259.153	LP259.154	LP259.154	LP259.154
37036.79	71815.73	106563.1	80607.17	43220.77	35635.81	60369.56	46254	40575.43	57055.11
34513.23	67684.91	113881.2	81972.66	50522.12	39846.86	57466.92	53461.48	45140.36	61322.27
37108.12	65493.57	116728.1	82678.27	48973.67	36784.92	61280.52	50331.37	42620.65	59556.17
40127.24	66171.58	106786.4	79494.36	42318.08	37271.3	61039.66	46028.89	41238.67	57374.59
35912.37	67025.42	104834.5	81149.17	41877.4	36969.77	59417.34	48178.91	36548.19	55165.72

LP259.154	LP259.169	LP259.190	LP259.190	LP259.190	LP260.147	LP260.149	LP260.149	LP260.149	LP260.148
43295.81	16173.68	179062	185333.5	40249.25	41229.8	47305.26	84102.77	58916.46	47050.32
36656.97	16973.9	184577.3	170247.8	38894.8	36783.99	50374.82	57230.04	63041.31	48876.28
40936.79	16804.62	174797.5	191217.1	46112.31	37729.57	47954.43	63228.09	59755.72	48415.86
38478	17011.12	183936.7	148655.9	44333.26	35331.83	54549.47	54787.71	63531.4	70828.59
41330.47	15954.45	160650.2	162661.2	43335.69	37285.6	46599.86	54859.05	61048.38	41262.85

LP260.149	LP260.149	LP260.148	LP260.148	LP260.149	LP260.149	LP260.149	LP260.148	LP260.147	LP260.148
53384.73	56101.93	48030.9	77414.04	47008.08	61074.64	53746.59	56641.08	55753.85	63999.99
54921.78	47057.6	43417.9	79102.04	43705.64	62699.52	51192.31	53534.77	62803.99	65239.06
54161.7	46277.53	43973.81	70031.44	42548.66	59130.1	49791.14	63754.16	62684.06	59430.37
54018.06	47996.77	47468.62	98967.39	57967.35	59700.73	57689.39	61297.26	61876.11	58374.36
58358.59	47847.82	45140.83	76057.65	45451.45	57652.42	48356.1	59996.46	61836.55	57539.15

LP260.149	LP260.149	LP260.149	LP260.148	LP260.149	LP260.149	LP260.149	LP260.148	LP260.148	LP260.149
84580.52	47895.91	47058.27	51970.38	50582.86	43819.9	60710.08	72588.59	65582.79	69770.19
73475.08	46235.6	53953.55	47047.78	43225.38	34746.29	42030.52	60947.81	65559.41	65199.11
72415.91	48638.54	49557.94	50042.18	53588.51	32820.45	45074.88	58837.82	66796.14	65660.57
75989	51264.71	46404.97	57801.19	60130.73	48029.39	48034.07	54214.16	54264.27	59051.22
74149.08	51259.26	53549.8	47545.5	44647.28	36813.55	45830.88	56673.19	60925.66	67015.36

LP260.148	LP260.149	LP260.148	LP260.149	LP260.148	LP260.149	LP260.148	LP260.148	LP260.149	LP260.149
46378.43	41068.8	61006.98	52156.16	40025.7	62742.26	31545.59	58256.92	67861.47	72710.99
57758.64	41819.06	54252.93	49960.56	36874.61	65081.31	35632.2	59221.54	73174.89	70359.22
56596.99	37066.75	57724.08	50590.72	38567.9	69388.05	34154.59	57578.7	67738.91	62745.4
75184.07	37690.27	57036.44	55060.4	41555.14	69536.51	48348.11	56692.25	63468.12	68462.37
48678.86	36610.42	52973.03	52605.81	35570.96	59616.33	38628.3	58899.54	74278.49	72291.38

LP260.149	LP260.149	LP260.149	LP260.148	LP260.149	LP260.149	LP260.157	LP260.157	LP260.149	LP260.157
52815.62	35732.41	44540.32	52383	45071.39	61862.91	52960.33	63488.48	50625.58	49335.76
46046.39	37933.56	40343.68	50035.78	47315.48	50223.39	23996.53	17563.26	59037.98	19816.92
47643.58	34080.55	37056.7	52171.87	51898.16	51950.25	23218.98	20330.04	53498.43	30884.93
50820.09	40819.69	35851.47	52561.77	47605.81	50432.6	22270.25	17179.34	46911.4	25385.08
49297.16	36377.46	29926.27	44062.46	49680.79	52259.6	22253.43	20810.83	39836.7	25391.72

LP260.157	LP260.153	LP261.148	LP261.148	LP261.148	LP261.148	LP261.148	LP261.148	LP261.148	LP261.148
22673.3	25812.15	108601.4	74812.27	104925.9	94056.12	76176.12	93342.93	96094.99	52130.57
24169.41	31514.99	132358.2	70622.08	113488.9	140938	92698.69	99318.46	75676.48	59647.08
22391.69	27067.93	133810.9	78738	91890.4	89393.95	67811.04	85066.14	93012.7	52995.08
26492.54	52576.7	92690.29	68069.06	75291.85	82439.68	64395.38	90707.6	92738.29	49320.97
24847.18	27349.31	90672.22	85292.8	67052.86	86037.42	75496.16	94047.96	87168.28	50663.27

LP261.148[illegible]	LP261.148[illegible]	LP261.148[illegible]	LP261.148[illegible]	LP261.148[illegible]	LP261.148[illegible]	LP261.148[illegible]	LP261.148[illegible]	LP261.148[illegible]	LP261.148[illegible]
70026.42	68706.56	91749.02	88368.01	81745.48	107895.6	105990.7	58152.19	84666.87	119030.3
82014.58	79238.68	89522.33	101281.2	109238.2	85998.8	86101.29	64705.01	87735.29	101311.9
64062.43	94779.72	62131.71	79034.73	78307.11	91698.7	113626.2	59708.16	84405.7	106760.1
66642.82	95527.42	59700.72	72017.95	74954.73	102074.9	101917	55933.91	75655.27	111099.8
71560.63	91347.2	60639.9	116109.9	85070.68	102701.3	103658.4	56348.48	77234.17	114272.7

LP261.148[illegible]	LP261.148[illegible]	LP261.148[illegible]	LP261.148[illegible]	LP261.148[illegible]	LP261.148[illegible]	LP261.148[illegible]	LP261.148[illegible]	LP261.148[illegible]	LP261.148[illegible]
110399.4	58147.41	42979.15	68198.13	53773.25	63817.57	56626.02	37904.83	47950.91	70438.99
127969.6	73773.11	50563.48	84256.69	38452.48	74723.09	48809.27	38460.68	52193.42	70365.36
101222.4	54813.27	39978.76	70322.83	51334.06	62670.45	48797.94	42578.48	49395.93	65272.87
106257.4	54944.15	40995.64	64927.75	40572.6	58187.18	46196.94	39207.26	47945.47	65204.11
110658.3	52908.75	41941.48	66284.91	52597.64	59655.02	52047.35	38512.83	46074.65	62610.14

LP261.148	LP261.148	LP261.148	LP261.148	LP261.148	LP261.148	LP261.148	LP261.148	LP261.148	LP261.148
43414.53	70991.68	59485.36	45523.99	67629.97	73120.67	43729.67	39830.45	50250.17	45072.38
40958.42	61216.39	55007.99	43341.62	71647.64	87631.37	45175.23	44018.75	46425.34	46801.58
40503.55	67214.47	61271.87	46636.71	64284.28	66897.14	39103.25	39877.82	51639.31	48154.61
40102.31	69312.42	55455.3	41626.82	65041.29	72981.33	48352.21	41460.32	46716.1	43138.4
41337.96	77563.16	53839.2	39909.2	64107.65	64032.62	38006.22	44320.15	47030.42	46333.43

LP261.148	LP261.148	LP261.148	LP261.148	LP261.148	LP261.148	LP261.148	LP261.148	LP261.148	LP261.148
59623.59	43591.17	77503.07	51363.78	46588.15	54434.82	56641.65	60504.52	63693.27	49510.18
60464.69	50011.81	102160.8	61177.25	53388.65	43851.1	59017.13	55623.14	66053.32	52227.12
55940.21	50984.87	77697.53	48209.81	57421.88	46115.97	58915.15	59347.92	62031.83	48099.85
53706.96	43390.67	63202.4	47079.38	45435.35	51160.93	58123.03	57976.56	54136.59	52194.05
50665.68	52255.14	70504.14	56865.99	56178.13	47405.63	62077.18	57134.22	58837.97	44683.46

LP261.148	LP261.148	LP261.148	LP261.148	LP261.148	LP261.148	LP261.148	LP261.148	LP261.148	LP261.148
29307.34	41554.67	35536.42	36251.01	58783.14	55229.48	78984.29	29337.21	62857.67	62250.72
30990.9	39886.72	39114.51	34749.59	61943.62	58028.63	89557.35	36598.73	59722.67	63072.69
30446.84	38974.8	36599.79	38848.34	55609.79	46436.34	83461.71	31578.83	57094.16	64559.65
26467.55	34967.94	33997.7	33389.88	48872.58	50588.55	76581.3	30160.6	63476.48	58904.12
31545.99	40104.15	34962.08	32282.58	56799	52198.34	76549.86	25804.9	56941.99	62951.85

LP261.148	LP261.148	LP261.148	LP261.148	LP261.185	LP261.185	LP261.185	LP261.185	LP261.185	LP261.185
36030.43	62617.9	39124.35	60026.34	21884.53	40116.38	28982.16	29004.59	44745.19	31194.82
41673.43	64151.81	43937.74	66691.67	23675.71	33591.85	24671.59	32458	43062.28	30363.97
35630.24	62273.9	37670.03	60905.44	21172.17	34493.91	26008.65	28351.53	48774.57	35349.67
35340.54	59245.69	37529.24	56580.91	20719.79	37106.05	29181.53	32366.87	46327.09	37166.62
37434.37	59776.05	35490.42	59959.08	23242.52	36102.67	27697.82	34987.64	49059.66	30469.15

LP262.165	LP262.164	LP262.165	LP262.165	LP262.165	LP262.165	LP262.164	LP262.164	LP262.165	LP262.165
55494.6	52213.38	61172.61	51593.01	66273.1	57701.28	43356.06	84996.81	52307.81	56147.13
65325.08	45028.1	63776.69	43488.03	66324.58	59264.63	43545.14	86201.84	53886.12	56634.13
63846.97	52804.37	55641.09	48586.33	64267.71	56403.29	47972.78	84596.86	48238.33	59325.67
57879.9	49328.49	59873.17	48522.13	63081.34	63537.94	38664.01	96571	53868.81	57849.77
63188.79	52413.12	57372.45	49697.06	61217.41	60044.15	45082.22	86166.52	55366.55	61564.61

LP262.165[illegible]	LP262.165[illegible]	LP262.165[illegible]	LP262.165[illegible]	LP262.164[illegible]	LP262.165[illegible]	LP262.164[illegible]	LP262.165[illegible]	LP262.165[illegible]	LP262.165[illegible]
53212.32	55077.75	107377.6	51036.75	48214.5	68108.02	73466.73	85924.36	87056.02	71498.21
48741.74	48800.68	99966.55	48918.57	44454	67367.86	75406.04	99042.08	96742.2	70560.61
52294.69	52319.49	101917.7	56020.04	51161.4	67010.13	70527.78	89313.17	89219.8	74483.32
54066.41	48525.3	101235.2	53473.92	45442.31	71875.28	69164.33	87386.71	93843.75	72127.28
54046.98	49517.14	105908.8	45701.52	50186.15	67217.52	78002.36	92460.75	90740.79	71356.13

LP262.165	LP262.165	LP262.165	LP262.165	LP262.165	LP262.165	LP262.165	LP262.165	LP262.165	LP262.165
41904.75	60196.21	102008.4	68516.75	72103.63	54081.9	69699.15	58644.62	46363.87	68214.48
41880.11	59647.42	89794.76	68924.58	73659.79	52727.97	73795.53	58859.22	45032.16	75279.5
45339.46	56755.43	96020.2	66273.88	83321.53	51823.3	70974.49	54975.17	50541.92	71314.28
45032.84	65716.75	90426.8	74682.08	71268.49	57518.68	76834.31	58484.19	49889.17	76133.94
48892.32	55781.37	97072.52	68499.89	79298.62	57480.48	69127.39	62100.88	51614.93	77756.54

LP262.165	LP262.164	LP262.165	LP262.164	LP262.165	LP262.165	LP262.165	LP262.165	LP262.165	LP262.165
76941.07	71933.67	53224.65	76891.53	68931.93	72619.25	77264.68	61292.94	94325.38	61097.61
70965.2	67641.69	54891.79	79929.91	64329.47	65547.38	73048.26	74869.6	93040.45	60100.21
78255	71571.07	56505.84	81293.8	72337.82	68179.25	76330.61	69251.51	99965.42	62550.83
77944.15	70804.25	54360.26	72405.97	74721.63	74457.74	84387.99	69945.39	93787.01	65706.07
76023.7	71948.95	53932.7	75367.36	79746.19	67922.03	69963.24	69185.7	99954.99	67898.53

LP262.165	LP262.165	LP262.165	LP262.165	LP262.165	LP262.165	LP262.165	LP262.165	LP262.165	LP262.164
53512.8	64673.16	72302.93	54183	121457.5	52327.17	68873.15	91928.81	74930.76	70233.81
61316.72	70187.66	62405.07	58602.59	123930	53795.04	71166.39	91637.24	75132.2	72222.19
58903.25	66174.95	75555.6	55050.93	132322.5	45460.83	71252.5	87911.19	73257.97	71703.81
55456.95	71971.45	71065.65	54751.68	134071.7	45293.96	70980.52	99314.99	77026.65	74628.75
65299.26	69587.74	68364.62	55633.43	129040.1	52179.22	63984.93	97739.68	70049.74	75263.59

LP262.165	LP262.165	LP262.165	LP262.165	LP262.177	LP262.922	LP263.200	LP263.200	LP263.200	LP263.200
73560.86	38990.86	56950.83	71806.23	72812.2	11510.46	53000.21	49578.52	61144.87	70545.6
72567.9	40469.67	43117.67	71219.68	77930.46	15350.34	52303.67	55883.07	65530.93	67985.97
66531.49	38089.27	50848.14	66161.67	69651.76	14901.72	57518.04	51734.43	61511.14	74247.35
71153.92	43221.47	55099.48	69916.98	72566.59	12590.02	58688.92	50716.84	66704.31	71803.89
70616.68	40826.24	50958.67	74892.76	69317.84	12911.49	59994.62	49423.83	66353.6	78015.65

LP263.200	LP263.237	LP263.237	LP263.236	LP263.237	LP263.236	LP263.237	LP263.236	LP263.237	LP263.237
67326.44	66542.71	46017.45	55516.72	48664.89	84675.73	89304.54	50833.22	58344.47	42823.01
67581.35	69368.85	35418.69	60494.9	51316.23	65223.29	91675.85	50447.31	59695.71	42733.69
67118.58	66215.27	44270.21	54779.79	52207.01	68125.64	81303.56	49207.53	59101.88	42921.88
68267.25	67395.46	47198.79	61927.48	49182.71	64709.33	84462.01	57371.38	61755.38	41129.57
70889.89	84131.74	62377.76	67645.47	56848.37	67821.45	74012.12	66178.24	67226.66	46911.74

LP263.237	LP263.237	LP263.237	LP263.237	LP263.236	LP263.237	LP263.236	LP263.237	LP263.237	LP263.237
55832.11	56327.15	68519.53	53909.54	65087.5	55681.37	50102.1	31319.54	54757.64	79685.03
51136.26	65423.5	63553.9	56713.07	62150.33	52356.45	51514.98	27866.66	47419.94	77872.03
55972.12	63538.36	62946.6	59718.32	57562.71	57352.39	53977.96	30585.62	57551.2	80112.33
59193.04	68181.78	68375.97	56151.75	57592.54	55818.01	50898.79	34628.77	52005.29	74083.84
58691.65	63530.43	71732.21	62433.86	63045.73	59219.67	53034.66	31825.92	58941.59	86332.2

LP263.237	LP263.237	LP263.237	LP263.236	LP263.236	LP263.237	LP265.142	LP265.143	LP265.143	LP265.143
45320.98	39801.77	47818.99	58720.71	61601.11	60712.34	178750.5	170360.6	155127	132521.7
41052.68	39466.42	43587.41	59862.88	66793.8	71913.42	175309.4	170231.4	140876.1	124981.6
40921.99	44182.59	46766.76	59612.26	67546.07	63945.08	185983.3	179895.1	141502.4	122595.3
41109.94	43833.35	48570.66	60968.37	69294.68	65173.13	170494.5	140618.2	135521.3	107278.2
44203.9	43694.82	43539.26	61953.16	71482.21	66949.01	168176.2	166684	169243.6	109619.2

LP265.143	LP265.143	LP265.143	LP265.143	LP265.143	LP265.143	LP265.143	LP265.143	LP265.143	LP265.143
212299.9	119381.4	80672.23	132408.3	60167.16	105677.6	165818.6	81012.88	81894.13	123617.5
164442.5	122743.7	75151.97	101939.3	57902.67	91964.6	157766.6	103274.2	91198.22	108107.6
163077.2	115690.3	88510.33	95583.42	47309.26	108568.1	164746.9	88378.7	90294.68	116224.5
236827.5	178520.5	79783.83	125521.1	94058.84	102033.6	170421	69982.14	76878.51	97024.08
159116.8	138285.1	78320.12	151999.7	53886.41	98127.29	173347.7	70169.13	139167.9	96466.43

LP265.143	LP265.143	LP265.143	LP265.143	LP265.143	LP265.143	LP265.143	LP265.143	LP265.143	LP265.143
94816.36	74424.72	148767	127606	162157.9	134496.3	98672.07	119970.1	105525.5	128316
125595.1	81228.63	145774.5	100612.6	120374	128400	102474.9	111422.8	142353.5	126141
85749.57	93479.36	146662.8	105696.8	117721.9	138483.8	105565.8	126833	109866.7	134729.1
122710.1	70335.57	152710.4	98934.39	113649.8	131260.5	100313.9	111942.6	107611.8	106940.7
79686.23	104287.9	134824.7	97883.95	149559.5	119590.8	104827.3	114250	103822.5	127818

LP265.143	LP265.143	LP265.143	LP265.143	LP265.143	LP265.143	LP265.143	LP265.143	LP265.143	LP265.143
39773.11	161993.9	77331.9	66455.65	92511.86	88707.4	91952.75	77611.37	131940.8	182228.9
38206.99	139716.8	75090.77	49790.93	97851.43	83082.46	97618.92	92580.98	132579.7	135467.4
40931.21	144824.3	76211.3	49692.34	98486.97	93962.37	76058.5	137052.6	133185.8	141940.2
39921.39	142828.4	72305	54950.02	94139.13	86904.9	85560.45	76405.25	130542.8	116288
35391.14	130061.8	74203.05	52759.46	93050.07	86933.87	77710.85	85794.92	127877.3	124973.4

LP265.143[illegible]	LP265.143[illegible]	LP265.143[illegible]	LP265.143[illegible]	LP265.143[illegible]	LP265.143[illegible]	LP265.143[illegible]	LP265.143[illegible]	LP265.143[illegible]	LP265.143[illegible]
66840.2	114506.5	61085.99	99236.62	86942.63	69404.43	63098.08	83735.67	94073.09	63291.83
64445.91	95107.46	62084.17	97006.42	85164.75	71430.04	64442.92	86618.36	94033.57	91896.13
60682.07	88091.56	61273.47	112300.4	86157.2	73976.17	66980.72	91689.29	95891.24	97620.08
59212.02	94968.67	57655.95	93268.89	76815.07	66443.26	63281.46	97093.33	92966.32	87838.27
52756.72	83482.33	59364.68	82087.06	81506.03	62387.71	62455.75	91243.4	96026.84	84331.84

LP265.143[illegible]	LP265.143[illegible]	LP265.143[illegible]	LP265.143[illegible]	LP265.143[illegible]	LP265.143[illegible]	LP265.143[illegible]	LP265.143[illegible]	LP265.143[illegible]	LP265.143[illegible]
68514.57	55654.37	59914.97	91946.33	59775	64108.74	60779.37	85190.67	38916.18	72142.52
68572.24	55626.87	67237.88	85296.8	53710.19	67298.6	61895.95	82853.39	34990.25	78063.78
69309.76	60092.69	67369.38	89342.97	58532.44	72711.24	57456.08	83265.71	39152.47	85465.92
66959.55	56996.89	58782.97	86430.69	62125.83	66239.51	57957.1	83202.6	39883.1	81120.38
71839.37	56024.4	67143.25	83927.74	55643.48	65515.09	61440.17	77655.78	37447.46	77436.64

LP265.143	LP265.143	LP265.143	LP265.18_	LP265.18_	LP265.216	LP265.216	LP265.216	LP265.216	LP265.216
27330.76	138637.6	139557.8	60578.45	90236.23	82994.75	64927.38	53164.29	74843.36	50425.62
31355.48	189411.4	144832.3	51433.17	90050.13	78690.82	61860.38	53425.87	74073.9	49182.21
29762.42	123949.9	125648.3	58857.92	86742.53	72182.55	98271.16	69007.61	60458.61	50503.74
29426.28	105484.6	137919.9	60099.21	89030.74	78055.47	64153.81	50182.82	79099.3	46156.05
30857.81	125276.9	193887.9	61653.58	92272.16	85563.12	58443.62	55801.71	83206.39	61567.59

LP265.215	LP265.215	LP265.216	LP265.216	LP265.215	LP265.216	LP265.216	LP265.215	LP265.216	LP265.216
42970.63	63262.88	66620.15	64448.42	56909.4	63774.17	52841.15	45210.71	44233.28	50165.18
38089.31	38387.76	68701.45	59206.39	72932.99	70250.45	52420.71	44283.78	48052.48	53612.22
36952.15	40175.26	97356.95	59844.45	59845.73	94028.08	59655.26	44746.58	48539.28	54658.09
35685.62	38060.55	71420.35	64855.69	56000.33	63594.71	49707.94	47860.15	51659.78	54789.59
50972.27	37036.5	71327.47	56972.74	58101.58	72022.87	55238.25	45766.7	48691.1	57345.37

LP265.216	LP265.216	LP265.216	LP265.216	LP265.216	LP265.216	LP265.216	LP265.216	LP265.216	LP265.216
46612.22	58915.87	57457.66	79603.77	36944.33	56101.27	33934.68	59081.31	42253.3	52076.07
55038.92	64910.97	62154.06	70823.58	38496.36	49974.01	40549.6	56740.98	45023.31	60096.55
53877.26	58403.11	63367.01	83221.25	40804.79	58177.58	39770.95	48505.36	43362.72	63446.18
50470.86	57650.51	62750.78	77870.91	39462.69	56794.22	37102.05	57896.04	43981.24	56031.93
57705.77	51244.06	62561.88	81942.51	39553.4	53218	41450.87	62300.01	49846.01	61207.26

LP265.216	LP265.216	LP265.216	LP265.216	LP265.216	LP265.216	LP265.216	LP265.216	LP265.216	LP265.216
64670.57	38310.78	65479.37	47825.32	43829.33	38368.23	31999.37	48741.23	38111.22	62360.54
68954.92	35066.1	59451.29	49377.39	50004.08	40771.09	34612.47	47397.08	37465.17	66264.5
66111.09	33015.99	67094.26	41640.39	40365.73	39263.17	29339.82	47819.45	35211.89	63058.62
70999.05	40510.74	63523.75	44309.2	44794.8	44207.29	29619.82	45815.59	38745.66	61622.49
76648.86	39941.91	68267.16	44069.54	54954.39	40324.97	31931.73	50181.34	39539.01	63634.53

LP265.216[illegible]	LP265.216[illegible]	LP265.216[illegible]	LP265.216[illegible]	LP265.252[illegible]	LP265.252[illegible]	LP265.252[illegible]	LP265.252[illegible]	LP265.252[illegible]	LP265.252[illegible]
42198.41	45880.6	40535.8	77363.68	35068.16	43400.58	60699.33	69413.75	76664.01	36411.38
44675.93	47639.74	44064.73	81131.67	40190.79	42630.9	56322.44	64572.91	88926.66	44610.46
42187.2	45084.33	42646.56	71472.91	35126.94	38597.27	57722.69	65760.1	77647.19	41168.07
44920.8	44569.86	43820.03	78867.1	44750.79	50297.23	62881.5	71599.03	74963.45	47309.27
45449.62	45358.25	45174.35	81939.17	37896.34	39006.97	58205.25	65213.59	78623.4	42464.76

LP265.252	LP265.252	LP265.252	LP265.252	LP265.252	LP265.252	LP265.252	LP265.252	LP265.252	LP265.252
62060.64	52003.41	72009.84	66313.27	36531.28	45588.71	72746.95	73914.54	46245.91	70867.85
61411.43	50967.88	71684.3	70241.46	36854.67	47391.87	63916.19	74284.11	38710.29	77502.65
59162.19	53117.89	80196.91	67865.42	36319.02	45928.88	70106.7	73973.26	41149.23	76411.16
65505.07	53609.12	81286.3	64447.26	41851.78	50213.7	77012.13	76044.05	38644.61	71666.52
60170.38	47987.89	70779.87	68887.5	41969.76	45345.41	72202.21	78352.33	43533.56	76374.58

LP265.252	LP265.252	LP265.252	LP265.252	LP265.252	LP265.252	LP265.252	LP265.252	LP265.252	LP265.252
59084.26	36178.6	117943	90846.49	63950.66	84901.88	87287.83	57757.3	57753.09	100561.4
61149.86	35007.38	80665.85	103246.1	113681.1	90541.44	96069.18	54108.36	62787.18	102223.1
63070.57	37176.29	109321.6	78255.51	84803.8	82414.99	103274.7	51732.74	63604.06	104116.1
59742.11	37949.63	96836.96	91079.94	112719.2	71618.53	110802.9	108205.8	61372.6	128524.1
61025.22	39184.45	101695.8	82123.99	86960.1	91247.78	102309.5	48294.48	67594.39	109127.3

LP265.252	LP265.252	LP265.253	LP265.252	LP265.252	LP265.252	LP265.252	LP265.253	LP265.252	LP265.252
55398.91	57840.3	45632.16	55421.65	70444.4	43264.66	61624.17	74197.91	68260.36	44752.7
51440.83	52485.05	42421.57	57714.13	76446.27	45850.12	59736.89	74503.84	71761.65	43498.58
54545.84	51675.51	46389.84	54515.35	68477.69	42141.27	56460.71	77389.34	60370.23	40672.8
60561.37	53626.13	52581.71	74074.18	74731.1	70699.08	69499.98	72490.87	107163.8	40810.62
54457.98	53171.77	45030.05	61916.57	74390.03	46383.29	63469.97	78657.64	72514.53	45255.9

LP265.252	LP265.253	LP265.252	LP265.253	LP265.252	LP265.252	LP265.253	LP265.252	LP265.252	LP265.253
43268.36	27839.92	47277.18	50020.1	31159.95	42394.04	37727.13	48747.03	47796.87	47442.73
39829.56	28664.81	46334.82	54067.96	31708.46	45473.46	35427.39	53249.51	50371.37	48848.25
42917.68	29068.22	46489.87	51405	26492.26	44490.08	42370.54	53162.88	49704.49	51462.56
42469.87	32880.02	43748.32	49902.89	30699.86	45669.78	39128.76	47304.22	51783.6	49893.16
45921.41	30212.47	44861.87	54609.22	29771.2	43843.91	41759.38	52191.41	49263.45	54799.7

LP265.252	LP265.252	LP265.252	LP265.252	LP265.252	LP265.252	LP265.252	LP265.253	LP265.253	LP265.252
32931.84	47322.48	59031.25	40969.21	39051.03	35319.25	42668.81	45088.3	42518.9	37535.1
31269.27	45437.25	54733.82	44922.82	37978.65	31657.96	41666.13	44144.31	41670.84	37297.73
35815.79	50410.63	54817.89	45839.16	39206.7	30506.25	40124.29	47004.82	41029.4	40303.98
39081.51	44588.77	57948.19	53434.8	38309.14	29170.17	48954.12	47905.69	45191.24	37893.07
42070.41	48352.92	61525.89	49806.35	40351.05	34232.1	41445.72	51444.03	43200.25	38829.63

LP265.253	LP265.252	LP265.252	LP265.252	LP265.252	LP265.252	LP265.253	LP265.252	LP265.253	LP265.252
29171.09	33053.11	38264.68	31493.94	33599.18	61680.15	50958.07	69129.46	36179.03	35687.87
34010.41	37205.57	33095.42	25122.7	34861.46	59574.21	49474.34	57315.36	34480.56	40287.27
37046.56	38334.73	34540.36	31565.87	34703.5	64935.07	56812.45	60460.51	33834.31	40058.66
35128.54	32504.99	35811.09	31495.69	34500.61	62922.3	51244.54	65621.01	34428.42	38232.28
32529.11	34690.63	36973.57	30226.51	39578.4	62283.81	53465.9	64107.69	40484.01	43107.84

LP265.253	LP265.252	LP265.253	LP265.252	LP265.253	LP265.252	LP265.252	LP265.252	LP265.252	LP266.178
40550.13	35792.35	48855.17	51382.59	52319.28	49479.74	61303.87	60395.39	76567.49	17392.88
48256.36	36819.64	54745.9	54149.62	55872.59	47189.67	56327.34	57612.32	69231.01	18519.52
48960.34	40529.16	48896.76	56185.18	59426.91	47974.69	57608.5	60058.21	70158.65	15656.73
44542.32	35712.78	48576.14	63996.8	55273.77	49678.68	61596.04	56519.41	73704.24	15538.83
45160.25	39191.01	52763.42	54861.12	54834.1	53904.24	59105.29	61383.84	70063.18	13975.43

LP266.178	LP267.120	LP267.120	LP267.120	LP267.120	LP267.120	LP267.120	LP267.120	LP267.120	LP267.120
24190.19	104888.6	101751	108698	106356.4	119150.1	105440.3	95107.59	82797.69	75968.39
25487.84	92828.16	80073.17	102853.5	110050.3	71776.17	89890.8	108877	94510	81008.16
22369.04	107708.9	114869.6	93554.81	102452.8	95719.8	74028.81	131997.6	93515.31	82022.85
25749.39	116996.8	153737.5	134334.5	108102.5	78611.62	96142.16	104830.1	66381.24	112905.6
25360.35	97993.59	89072.44	73984.08	114900.9	122626.8	139736.9	87863.63	92263.28	135249.6

LP267.120	LP267.120	LP267.120	LP267.120	LP267.120	LP267.120	LP267.120	LP267.120	LP267.120	LP267.120
115032.4	69868.03	90542.87	101252.5	71763.57	89278.59	80455.55	118641	120612.9	87256.31
75051.22	80827.4	88234.49	95328.26	58062.03	87085.83	77624.37	107376.9	89280.02	126447.5
81120.62	126766.6	74725.95	94070.01	56780.58	83194.45	85952.44	100896.7	98910.46	114623.3
121405	63284.77	78959.09	148354.4	70100.16	85091.39	96116.08	76192.94	115962.7	84405.14
102138.1	90618.2	111815.8	112161.4	58897.36	77831.65	86211.18	133472.6	87522.37	90322.25

LP267.120	LP267.120	LP267.120	LP267.120	LP267.120	LP267.120	LP267.120	LP267.120	LP267.120	LP267.120
83933.42	103185.6	87427.53	82109.81	86737.77	137679.7	96648.69	149745.5	121024.4	108197.9
79346.06	99717.53	88460.9	83876.13	107533.7	102726.1	94446.94	99122.21	108134.8	96294.42
80093.42	97712.54	126247	85994.27	106085.4	99177.94	92936.37	90562.25	137204.8	63291.93
84561.55	87719.22	88254.86	112681.1	107188.3	86348.45	135639	144716.1	104408.3	92155.52
89900.35	119918.4	115338.2	70126.33	100640.9	111318.3	72559.3	156835.1	114727.8	67435.89

LP267.120	LP267.120	LP267.120	LP267.120	LP267.120	LP267.120	LP267.120	LP267.120	LP267.120	LP267.120
131184.4	90855.21	102883.2	101376	138072.8	127249.2	85872.71	93314.34	69556.25	78733.54
112680.4	137003.2	96402.74	100593.1	111199.2	91034.71	97828.42	112850	73831.05	101737.2
119026.9	93030.16	99409.96	118134.6	119157	150859	105118.3	76501.99	92748.06	95516.71
95936.25	124502.7	114205.3	108455.1	132682.4	93556.44	82762.05	89423.81	108538.9	98274.62
123697.2	97498.58	84768.06	91035.57	99025.06	91126.41	84744.79	121043.7	94708.93	85015.32

LP267.120	LP267.120	LP267.120	LP267.120	LP267.120	LP267.120	LP267.120	LP267.120	LP267.120	LP267.120
93880	86235.91	115156.5	91617.61	173794.5	103606.2	94758.2	96365.11	98418.14	80319.86
157784.8	85493.06	139250.6	95072.87	99731.94	101481.4	112674.6	87799.53	94038.28	68602.03
149194.5	82177.77	114241.4	88052.86	132090.1	90544.54	81814.48	95091.45	92259.15	79709.64
73505.04	109136.2	93999.65	114779.8	115253	100161.2	81038.43	97500.73	113046	120892.9
87659.43	83302.48	103520.8	81977.72	116799.5	84233.86	86352.78	112869.3	98252.15	92778.02

LP267.120	LP267.120	LP267.120	LP267.120	LP267.120	LP267.120	LP267.120	LP267.120	LP267.120	LP267.120
72326.35	96642.47	75279.99	111296.2	106713.4	87541.69	112052.1	100759.5	92784.85	82399.78
69247.25	95320.15	81288.16	114616.8	111066.9	83938.21	110249.6	94703.92	84354.58	87720.86
79648.83	100368.2	81304.01	102033.2	109963.7	88403.09	113115.2	95906.52	86493.35	90665.16
61506.82	113399	82210.05	132054.6	104401.5	86526.64	108079.1	96200.45	77823.54	111088.3
71389.74	106451.1	77666.25	102718.7	109649.9	77723.94	106013.7	102458.2	85372.08	87279.95

LP267.120	LP267.120	LP267.120	LP267.120	LP267.120	LP267.120	LP267.120	LP267.120	LP267.120	LP267.120
94448.92	91056.6	72168.73	78173.05	95913.48	89946.68	110323.2	71234.56	101876.6	91090.79
89600.9	129392.8	84951.69	82685.1	97873.01	92611.61	87479.31	72739.68	104103.2	86539.44
99142.79	88950.33	116609.8	86846.03	99927.78	94490.17	90860.66	66269.35	102932.6	112313.7
94455.65	78076.84	71289.14	76822.32	70949.05	99991.17	91293.57	61152.45	117027.5	151762
102271.2	86595.58	124275.9	73187.08	99258.23	104206.1	87364.48	71801.13	112345.7	74895.17

LP267.120	LP267.120	LP267.120	LP267.120	LP267.120	LP267.120	LP267.120	LP267.120	LP267.120	LP267.120
91948.41	67213.23	58469.47	91164.79	49640.51	71069.15	64619.96	75721.79	80099.79	93247.89
99505.43	69899.1	53093.69	86535.98	55160.97	71849.66	63179.89	76544.42	80211.25	90429.92
104381.5	70245.25	60357.2	87761.8	48695.02	61670.95	61501.26	75456.37	79428.65	97074.77
95721.44	78114.02	49816.5	82794.79	87159.5	97482.38	104782.7	60700.27	77945.59	96274.73
92743.07	63766.66	44722.75	82600.26	46186.08	68296.78	59869.33	78264.76	74174.68	110867.8

LP267.120	LP267.120	LP267.120	LP267.158	LP267.159	LP267.159	LP267.159	LP267.158	LP267.159	LP267.158
68168.02	55075.26	85244.04	75771.92	84571.62	87184.79	65862.71	47672.48	109579.9	99138.17
70641.66	50728.71	86211.99	62684.27	86371.73	77130.88	66849.14	54420.28	114148.3	88179.25
68391.18	52229.01	83209.54	66327.22	85056.24	88208.24	62215.45	51922.22	118257.9	94096.41
67430.87	67008.28	85659.66	73056.34	89708.24	78551.26	55931.6	45942.53	118838.9	91671.1
58572.61	48615.67	81963.96	74166.15	87807.26	99018.76	63936.8	50721.85	108444.1	97481.98

LP267.159	LP267.159	LP267.172	LP267.174	LP267.195	LP267.195	LP267.195	LP267.195	LP267.195	LP267.195
87635.04	102064.2	886520.3	46461.27	82552.41	89248.8	56260.05	61078.95	75266.95	45647.83
87412.26	100000.2	277669.5	49160.43	82974.4	90607.81	55307.47	70820.55	75852.14	51405.62
89385.16	94820.41	408445.4	52819.97	80308.27	90061.06	58964.35	65976.01	79424.91	47209.35
85232.24	96334.52	641222	50384.78	83055.9	88120.35	62286.69	71322.72	70226.12	47892.21
87192.16	106159.5	513909.6	45103.49	81294.88	94609.77	53655.87	44015.83	78159.36	47214.57

LP267.195	LP267.195	LP267.195	LP267.195	LP267.195	LP267.195	LP267.195	LP267.195	LP267.195	LP267.195
56336.85	95671.2	82159.83	72894.39	82071.14	96759.62	96023.03	87307.48	93926.23	116423.1
52078.61	95419.36	71847.44	69172.63	73059.56	92756.52	91768.36	84972.53	90256.73	95463.89
59032.65	94804.37	78132.39	70791.73	83539.47	91363.86	87319.99	80045.06	89984.19	103965
60363.56	77126.23	72438.43	70562.54	84021.3	94314.12	87895.42	80813.82	87652.83	101921.6
58369.94	78023.98	74789.01	72158.86	78693.39	91374.23	101484.7	87284.33	81176.41	106201.4

LP267.195[illegible]	LP267.195[illegible]	LP267.195[illegible]	LP267.195[illegible]	LP267.195[illegible]	LP267.195[illegible]	LP267.195[illegible]	LP267.195[illegible]	LP267.195[illegible]	LP267.195[illegible]
123251.6	73536.45	59353.06	129741.4	68027.52	117626.2	69792.96	63012.65	61920	65099.31
131066	70200.8	58915.52	127631	79188.74	113297.5	78605.69	59745.22	66583.96	61077.56
122419.3	70386.56	54804.52	130727.9	78378.57	117944	87757.08	63243.07	62564.13	67675.07
121616.9	73193.09	49631.87	124785.8	75584.76	110123.5	79332.13	63243.26	64683.67	60607.1
123725.6	72762.09	60395.28	83003.13	73907.67	109069.9	80227.99	53328.39	65643.88	65322.65

LP267.195	LP267.195	LP267.231	LP267.232	LP267.232	LP267.231	LP267.232	LP267.232	LP267.232	LP267.232
47102.17	54415.5	73207.39	73639.32	106476	81505.84	78196.56	59008.1	72297.68	85974
51165.98	47887.87	58737.5	64237.66	97470.06	74890.43	78658.04	55042.87	66391.74	84704.64
48987.47	49930.75	74885.23	72613.13	107243.6	65666.88	87211.61	56793.31	70134.99	88702.03
54800.1	51873.19	64408.47	66710.15	109550.9	76873.02	59629.46	59960.16	63358.09	82901.72
46676.18	48906.64	61342.6	64986.22	98341.11	54661.01	82111.96	59579.78	51275.04	85706.53

LP267.231	LP267.232	LP267.231	LP267.232	LP267.232	LP267.232	LP267.232	LP267.231	LP267.232	LP267.231
85913.29	80215.39	76765.9	76508.94	83025.12	81350.64	75950.2	51290.52	72212.73	67753.18
99901.86	83468.12	70967.2	77482.86	88338.97	82032.26	83201.19	57812.23	72853.58	66198.23
52301.95	79571.85	74060.8	74223.32	87629.55	76804.12	85627.25	48578.92	80349.34	64844.28
97671.94	79837.06	44680.54	87856.49	86729.03	78730.5	78614.87	59363.18	65449.73	65111.18
70263.76	78179.16	75707.32	78592.18	91006.44	77988.36	78783.73	56859.37	75097.52	59612.27

LP267.232	LP267.232	LP267.231	LP267.232	LP267.232	LP267.232	LP267.231	LP267.232	LP267.231	LP267.232
70632.42	107272.6	68756.37	66031.57	68335.7	45017.09	45234.16	108877.8	82016.93	68266.79
73469.65	106162.6	63081.49	70775.46	68085.78	44518.16	55657.06	108255.4	75917.2	65361.97
61334.82	107645.1	71346.1	65781.14	69766.29	47355.5	38855.02	112116.1	81870.68	67116.02
67449.71	99166.11	61838.67	70650.52	68330.58	47922.12	52517.09	105019.8	81147.53	69675.79
75095.22	105603.4	52560.18	71219.43	67161.41	52441.26	48041.05	111970.1	78237.13	67191.16

LP267.232	LP267.232	LP267.231	LP267.231	LP267.231	LP267.233	LP267.268	LP268.154	LP268.154	LP268.154
48577.86	77108.35	78978.03	73904.59	80404.18	71916.9	98401.02	22198.98	26563.93	19678.87
53153	73595.24	78147.37	75042.12	82080.44	72535.08	110172.9	21367.54	27434.87	19269.02
51189.42	75140.93	82951.31	77971.47	81297.03	72085.09	132292.3	21567.52	26599.86	18788.96
51000.9	74336.08	78610.2	71944.54	78900.2	66790.62	134254.5	21575.42	25791.61	20809.92
51706.9	78470.16	85526.17	69860.36	61510.97	75443.9	139981.5	23277.24	28126.35	23203.18

LP268.190	LP268.191	LP268.191	LP268.191	LP268.190	LP268.191	LP268.191	LP268.191	LP268.263	LP269.136
23958.79	26226.68	21909.51	19070.39	23886.78	25041.14	19667.27	23944.96	15433.12	162819.4
19920.36	25082.99	23785.07	20935.28	24687.49	25775.86	20319.3	26756.03	12385.78	119382.2
20812.79	28376.92	22157.28	21464.24	21082.7	26934.33	20457.27	23015.44	8971.303	106320
23535.95	27360.17	20446.41	23644.97	25418.09	28581.19	21246.52	26869.15	7875.156	154504.7
25464.82	29417.5	24275	22564.89	26122.66	29552.71	22028.53	26941.42	10094.04	119410.6

LP269.136	LP269.135	LP269.136	LP269.136	LP269.136	LP269.136	LP269.136	LP269.136	LP269.136	LP269.136
83802.63	149872.8	133237.2	109634.8	112420.1	106877.4	106112	107792.3	141937.9	106414.6
123018.7	97724.11	110518.4	152980.3	122889.4	105550.9	197305.9	110118.1	120979	111446.7
116266.7	128715.8	143048	94213.9	117273.7	99212.17	110149.9	148689.9	111208.6	136299.4
92660.57	133308.5	101309.6	92181.66	138200.1	143513.9	170132.3	123719.8	102098.3	102919.9
98364.28	87584	138560.2	164662.7	115676.4	88196.57	83094.85	113581.5	125463.3	101209.1

LP269.136	LP269.136	LP269.136	LP269.136	LP269.136	LP269.136	LP269.136	LP269.136	LP269.136	LP269.136
136578.7	161224.9	100723.4	148806.7	95483.95	89164.47	150425.5	81641.52	92846.29	102845.8
130518.1	96709.41	108644.8	114936.7	157721.9	69238.67	141576.6	113104.8	99623.97	132552.5
94504.42	108074.5	108497.1	150998.2	124012	91404.19	141553.9	94308.41	104283.4	91687.11
118015.3	101055.5	76538.99	112791.4	91934.69	93288.31	165526.3	95429.11	98453.73	87469.61
115602	97768.2	98980.46	77443.38	91905.91	93479.13	122451.7	124614.7	102704.4	94686.24

LP269.136	LP269.136	LP269.136	LP269.136	LP269.136	LP269.136	LP269.136	LP269.136	LP269.136	LP269.136
146323	103716.1	97166.47	96406.7	100914.4	134474.1	175239.5	102913.4	97992.13	94961.1
136944.1	105416.1	119505.2	124251.8	83859.48	122030.2	89283.26	106497.4	90958.86	118385.9
140140.8	103299.6	101969.6	115364.3	106746.3	134327.4	90101.19	108573.6	82393.44	92227.97
139711.7	105747.7	138753	125561.2	104988.5	140219.5	95832.6	112498.5	89089.96	86438.25
139440.7	106416.5	96927.35	117227.1	95998.12	125936.9	89719.7	95707.14	85186.5	137477.8

LP269.136	LP269.136	LP269.136	LP269.136	LP269.136	LP269.136	LP269.136	LP269.136	LP269.136	LP269.136
79047.09	122333.1	101887.7	82881.48	133913.3	128063.7	150839	109204.2	105302.6	109418.9
78411.16	110387.7	96621.06	83064.52	79564.12	129716.3	159745.4	141391.7	104301	109093.8
150993.5	183392.2	91327.8	130839.2	128961	131525.3	142932.2	104894.1	131363.7	102861.4
66651.6	111160	102821.9	100299.2	132338.2	119955.8	149411.3	111392.5	100092.9	97101.71
70566.74	105682.6	104093.9	88109.45	133741.1	131294.3	147218.4	112511.3	112472	114925.3

LP269.136	LP269.136	LP269.136	LP269.136	LP269.136	LP269.136	LP269.136	LP269.136	LP269.136	LP269.136
136312.7	127300.5	83827.56	116906.2	94620.93	99253.05	100449	102876.7	114238.3	105002.2
125448.2	128252.8	86588.1	115191.7	101911.5	92349.33	100748.9	131194.7	101895.8	106823.6
134493.2	125941.1	99328.86	119936.8	102493.8	102061	109801.8	114496	110025.2	111220.3
133268.1	113701.5	83153.94	127844.3	93862.7	97848.45	93858.32	121802	108845	112739.2
123123.4	121597.4	85503.42	120018.7	108611.5	98689.98	91495.83	101994.9	113928.2	104043.2

LP269.136	LP269.136	LP269.136	LP269.136	LP269.136	LP269.136	LP269.136	LP269.136	LP269.136	LP269.136
105666.7	110093.8	98878.27	60378.84	99502	66457.04	81954.12	89921.4	78766.79	80131.96
114170.5	104128.2	102532.9	59111.07	90205.79	71565.1	80647.13	86335.59	77996.02	82581.29
113840.2	120859.5	83517.64	66364.78	103221	70558.63	87990.9	94666.5	84176.84	83053.54
116330.7	114636.2	88802.68	63850.46	105017.5	70658.88	81713.84	96907.61	89495.4	86809.26
112021	114499.6	86481.29	62354.23	91026.09	63950.06	75343.49	88264.96	80500.47	74623.44

LP269.136	LP269.136	LP269.136	LP269.174	LP269.175	LP269.175	LP269.175	LP269.174	LP269.174	LP269.175
67222.14	90322.71	59773.51	103747	177487	169270	145767.3	105659	119533.5	131734.4
68094.95	88167.98	65148.28	95277.48	170992.6	162240.7	152880.4	102325.4	129189.3	126189.5
62963.39	86882.8	58938.74	113199.9	180138.6	161096.5	143969.1	88153.85	127007.7	122265
68498.71	93562.6	62035.26	77399.55	109627.7	120051.6	150710	56316.17	129646.7	83061.64
62576.36	77354.18	59397.33	110290.3	192203.9	181941.3	173491.9	99516.29	118977.8	127043.5

LP269.175	LP269.175	LP269.174	LP269.174	LP269.174	LP269.174	LP269.175	LP269.175	LP269.174	LP269.174
128269	151592.6	111458	92079.05	108192.1	113714.7	194548.6	148793.5	142068.3	81116.19
137042.6	131609.7	109113.3	72892.58	104276.5	115725.6	198877.8	130976.3	145451.5	75978.6
131913.3	130705.3	117563.9	86135.99	105469.7	118682.5	196396.4	133305.4	145892.1	71611.94
127312	139625.5	118681.1	89634.56	103562.4	120147.8	203493.1	130109.3	136641.1	79214.48
147997.2	134499.4	128587.8	91731.12	120714.7	112606.3	223437.4	128926.5	145587.8	80579.48

LP269.175	LP269.175	LP269.174	LP269.175	LP269.174	LP269.175	LP269.174	LP269.174	LP269.175	LP269.174
164124.1	176826.5	125896.2	91642.45	136351.6	126076.7	88396.69	109217.3	85812.7	156156.6
164184.9	173789.2	121154.8	97296.44	138006.4	127076.9	88543.2	108569.5	83254.94	148671.4
165115.6	164233.8	132890.8	99410.64	138150.7	128317.6	90743.68	112041.2	75509.52	156590.1
172559.8	170227.4	131053.2	105573.2	138638.1	128891.7	95443.81	109436.4	89845.16	141970.7
182604.6	169071	144213.1	102087.8	148345.9	129687.2	104857.9	122867.9	87479.09	154633.7

LP269.174	LP269.174	LP269.175	LP269.175	LP269.174	LP269.175	LP269.211	LP269.211	LP269.211	LP269.211
98069.27	126345.1	68795.45	132878.2	120831.7	87176.8	98302.29	87146.8	119571.1	81790.14
92704.72	129947.6	71250.65	124581.5	130878.3	81515.98	101135.6	200304.7	164822.6	148078.6
94253.91	123823.5	68553.41	136994.9	127404.1	82416.41	98665.2	192374.6	164697.9	160758.9
98595.55	127321	74492.63	126889.9	124446.2	88718.65	180334.4	184698.8	176379.5	160070.3
103858.9	126528.6	68643.8	128387.7	130939	89900.91	185876.6	200253.5	194488.2	174037.8

LP269.211	LP269.211	LP269.211	LP269.211	LP269.211	LP269.211	LP269.211	LP269.211	LP269.211	LP269.210
68620.72	50764.53	55225.14	116394.5	190047.6	121822.8	127689.5	93016.27	91703.06	70466.9
155857.5	122735	125924.7	120574.7	185873.1	116950.4	136931.5	89756.09	91215.4	74200.77
150728	115030.9	113755.7	124256.5	185689.8	119351.7	132091.2	94728.85	94116.19	77011.02
153023.6	79990.38	132621.4	137542.5	200965.5	123915.6	145401.3	101273.7	92483.75	81882.41
160067.8	125149.4	137499.9	132543.1	198475.7	116052.4	134987.3	90628.25	97268.65	77227.78

LP269.211	LP269.211	LP269.211	LP269.211	LP269.211	LP269.211	LP269.211	LP269.247	LP269.247	LP269.247
121317.8	130344.5	145199.1	111887.1	48104.97	123293	76221.09	135237.3	179713.5	135386.6
121940.9	121468.1	133565.9	108138.4	88012.17	67064.34	74734.24	119713.2	191886.7	129724.2
120120.8	123558.9	134879.5	108735.1	82401.28	120799.5	83042.61	128753.1	190236.9	131177.5
124708.1	73641.36	80285.55	118712.1	74974.15	129265.5	81852.52	139665.3	168915.7	135398.8
127484.5	140569.5	152884.3	130130.2	94831.51	132458.2	77482.88	152129.2	185063.2	149166.5

LP269.247	LP269.247	LP269.247	LP269.247	LP269.247	LP269.247	LP269.247	LP269.247	LP269.247	LP269.247
110972	112431	124719.5	179489.1	119257.6	131385.8	161364.5	166773.8	140873.6	109078.4
103392.3	109384.5	120661.5	191620.1	121051.6	144284.6	160045.4	168298	144285.6	105024.4
100136	112714.9	119552.2	184057.3	122770.8	135261.4	160830.1	156520.8	146116.5	99744.66
110439.6	102490.5	122708	182586.5	122132.7	149091.2	154245.1	149662.5	141602.6	110896.1
106215.9	114992.8	124789.4	199486.6	132119	150556.5	168142.2	161455	144056.7	118651.9

LP269.957	LP270.155	LP270.170	LP270.170	LP270.170	LP270.170	LP270.170	LP270.243	LP270.243	LP270.243
18269.48	16266.97	40093.09	35738.79	28214.32	25953.12	17240.01	20998.1	15902.44	9582.76
17690.52	17387.32	38087.74	43021.2	29203.42	25022.2	18654.14	31635.56	16361.4	7999.678
18091.65	16156.08	38022.42	38938.91	25449.88	22706.54	17675.33	30729.27	17348.92	8624.791
17775.26	14148.61	41001.11	44554.17	28408.05	23077.14	15095.42	30708.88	15743.1	7045.924
13176.97	13880.18	39940.71	39548.53	24325	19582.08	15099.57	30070.75	13788.66	8961.885

LP270.251	LP270.251	LP270.251	LP270.251	LP270.279	LP270.315	LP271.115	LP271.115	LP271.115	LP271.115
27079.82	33560.18	27296.74	35676.73	43780.89	165702.9	383518.3	167434.4	207721.6	200411
29463.1	27207.47	28868.81	24309.34	43883.42	178458.5	238115.2	142522.7	259414.6	354732.2
28591.32	29430.34	19962.52	29126.4	51071.97	242723.2	271368.3	171745.5	196428.6	253169.4
27966.57	30674.18	22498.03	30079.81	53725.33	289629.7	315490.7	215347	270712.9	242221.9
34097.6	34732.81	30241.08	32507.44	54945.66	309588	271409.6	268754.5	229149.4	272175.5

LP271.115	LP271.115	LP271.115	LP271.115	LP271.115	LP271.115	LP271.115	LP271.115	LP271.115	LP271.115
331530.5	236648.1	207198.6	136453.5	299067.1	267605.2	231542.4	339263.3	256022.4	258067
254439	223230.7	176244.1	120180.8	295432.8	378373.8	314405.3	238555.1	256025	199181.7
220280	173823.2	225747.6	112623.8	235589.2	303115.2	205989.4	234860.9	202595.5	229666
285762.9	218405	329630.5	150313.5	243439	211146	276248.7	405498	283216.4	191970
237980.6	277868.5	333234.8	120832.5	315110.9	267046.5	277776	270630.2	298802.6	239414.5

LP271.115	LP271.115	LP271.115	LP271.115	LP271.115	LP271.115	LP271.115	LP271.115	LP271.115	LP271.115
399053.1	207234.2	192085.6	277831.3	208681.8	192260.6	359933.4	379047.6	250184	261118.8
252313.6	341483.6	196123.7	302380.5	290936	257850.2	248325.9	198782.6	274673.9	230057.9
310623.4	325355.8	292907.3	232769.1	255539.7	185080.5	268226.6	220380.6	316427.9	310249.6
263027.5	227271.8	197909.8	268229.8	230633.3	264197.2	240235.4	234725.2	254362.4	188384.6
219729.4	323607.7	205165.5	265231.1	225784.2	176441.1	377876.5	223221	373237.2	222224.3

LP271.115	LP271.115	LP271.115	LP271.115	LP271.115	LP271.115	LP271.115	LP271.115	LP271.115	LP271.115
337500.4	295297.1	333769.1	220041.6	302656.4	284343.5	217229.3	207455.1	241758.6	310348.7
275943.7	293684.7	401740.8	319304.2	236296	228789.4	239733.8	218948	232106.5	234938.3
316955.5	317662.6	308070.1	200480.8	250842	231608.2	252312.7	222021.3	228870.4	317111.7
264062.3	311617.5	263581.7	249721.9	275363.3	243377	238624.7	192774.9	240844.3	260742.6
284951.7	315562.8	327705.8	202754.9	247227.3	199079.4	223301.9	241744.2	232377.5	198958.9

LP271.115	LP271.115	LP271.115	LP271.115	LP271.115	LP271.115	LP271.115	LP271.115	LP271.115	LP271.115
262080	261821.5	159878.7	217117	335935.3	183146	157319.5	198395.3	286237.2	243438.4
295723.2	421111.5	207293.5	214483.7	286147.3	265193.5	152612.1	219277.7	294875.8	218964.8
292571.6	296036.3	196794.3	280258.1	296398.9	189901.2	179481.5	237514.7	302033.7	223027.2
240766.6	254694.3	298421	323251.4	246759.2	222104.8	132564	236850.3	186786.6	251313.2
272348.9	288552.5	232509.4	208653.9	305617.3	186925.6	226722.6	210728.4	234050.3	219237.1

LP271.115	LP271.115	LP271.115	LP271.115	LP271.115	LP271.115	LP271.115	LP271.115	LP271.115	LP271.115
194331.6	184496.9	199035.7	292478.8	187179.7	277770.8	303002.6	221588.6	210929.8	242015.6
212143.9	142017.3	175743.5	274930.3	176074.4	272042.9	208589.4	213346	209950.5	210879
222050.5	148274.9	225318.6	269367.7	228825.2	275056.6	197760	175216.8	214749.9	229885.7
173015.7	188967.9	164722.9	263731.3	240576.8	290365.6	242569.7	177406.1	292009.3	226862
263639.1	154854	200742.6	290737.4	183279.3	254155.3	281147.1	204296.9	207904.4	233372

LP271.115	LP271.115	LP271.115	LP271.115	LP271.115	LP271.115	LP271.115	LP271.115	LP271.115	LP271.115
201055.3	226679	234342.5	214097.4	192928.6	209816.7	227945.3	264134	203395.8	189334.3
197191.4	207539	233252.3	227064.5	183559.3	210265.6	234368.7	209132.8	198263.1	196286.7
192576.7	182014.3	265278.7	228459.8	186373.4	223877.7	238746.9	196797.7	190043.1	187517.6
209835.4	230320.7	162386.9	327841	192377.1	230197.4	260330.5	203345.3	276903.2	192280.8
227879.8	229567.1	219446.1	259834.3	179611.4	240384.5	261159.5	176364.6	184497.5	212512.7

LP271.115	LP271.115	LP271.115	LP271.115	LP271.115	LP271.115	LP271.115	LP271.115	LP271.115	LP271.115
284709.3	260276.6	169616.9	166044.3	141925.7	150329.8	86780.24	127004.3	125309.5	129556
217633.2	226765.3	164043.1	130974.7	137383.4	160768.1	112724.6	127884.2	204497.7	121129.2
216208.5	213159.5	158484.8	133993.7	127607.2	146149.1	79723.19	123975.7	122989.7	101950.3
230559.5	214979.7	131244.5	137101.4	172051.8	142231.1	86075.12	128481.6	126463.6	127815.8
236768.2	215412.7	154379.6	147038.2	139355.3	165597.3	91063.43	113192.3	125934.3	109508

LP271.115	LP271.115	LP271.153	LP271.153	LP271.154	LP271.154	LP271.153	LP271.154	LP271.154	LP271.153
119310	102292.5	62096.49	46753.43	73803.65	68098.53	79832.64	65085.97	53056.41	68449.53
114321.9	111430	91130.53	45794.19	78461.48	63782.68	73863.09	63912.55	56773	69974.57
116223.9	100232.5	102363.9	48629.69	73531.31	64247.2	74864.41	67436.96	53437.64	69879.77
99820.93	107395.2	77969.13	44881.97	67930.08	63205.6	75627.32	57277.05	54032.85	66613.06
119575.8	103422.4	103018.1	48544.45	76463.79	73704.04	71829.48	73821.28	55768.04	70073.66

LP271.153	LP271.154	LP271.190	LP271.190	LP271.190	LP271.190	LP271.227	LP271.227	LP271.227	LP271.227
50885.9	49811.21	92807.98	54255.05	67104.78	92635.36	67322.73	64450.79	68015.53	78298.25
59381.55	52613.84	93687.09	51226.58	69321.85	91341.12	59660.84	63594.66	66641.18	78679.51
61605.59	55216.34	94277.38	62989.22	75415.3	87163.04	76220.76	58152.47	66534.8	74280.33
53304.12	52390.62	98862.59	54153.76	61529.72	90048.35	68389.2	56810.66	71613.52	68961.2
51516.51	54857.84	99368.13	56525.43	76567.97	84806.69	66409.03	61580.28	67015.56	76078.6

LP272.118	LP272.185	LP272.185	LP272.185	LP272.185	LP272.185	LP272.185	LP272.185	LP272.258	LP272.258
25837.42	17462.73	28299.69	25569.79	19319.55	29448.39	42206.32	32461.41	64539.2	28986.51
22669.74	13920.05	29297.68	24545.6	19968.68	33977.59	47592.96	29254.93	59874.61	29626.43
24968.05	18868.25	27151.59	22545.26	19649.5	28349.75	41482.73	31735.48	61735.47	24265.85
21240.67	19524.51	28745.6	21738.93	21120.06	33692.95	46681.1	35779.6	65547.79	25892.85
19904.72	19202.39	25910.09	23000.27	19932.24	29647.19	43671.85	31444.21	69107.95	36334.37

LP272.322	LP272.944	LP274.164	LP274.165	LP274.164	LP274.164	LP274.165	LP274.165	LP274.164	LP274.165
20920.58	16586.79	45814.14	47323.25	46746	56451.44	45375.18	58237.94	37620.91	48368.7
22132.13	11829	43874.77	45380.24	44956.52	57177.98	47525.55	50796.75	37235.95	48328.09
19132.39	12256.53	43528.07	38757.43	58145.43	52662.65	63414.67	54338.68	35143.66	50925.02
20606.6	16118.93	38767.03	53748.28	43634.62	55172.83	48485.04	61826.05	37728.26	47113.81
20583.03	13772.02	41779.25	51385.03	43042.88	57324.37	49869.18	54251.95	43472.88	52681.88

LP274.165	LP274.165	LP274.164	LP274.165	LP274.165	LP274.165	LP274.165	LP274.165	LP274.165	LP274.165
44974.3	54491.51	43271.65	36779.04	38159.62	56555.59	47693.18	43447.89	39770.16	50200.66
41628.85	49031.31	37464.13	33386.14	41646.88	54169.54	37329.88	46347.53	38455.2	56950.3
41734.34	63940.52	50153.16	43066.15	43886.12	61421.68	44746.36	47198	38839.24	57202.12
42729.86	53648.95	46001.39	36138.06	43590.84	58460.32	45665.94	48548.83	32929.94	51216.8
35000.17	53515.58	39324.35	34217.35	37959.5	54618.79	44164.21	45473.04	31458.52	44986.71

LP274.165	LP274.165	LP274.165	LP274.165	LP274.165	LP274.273	LP274.273	LP274.274	LP274.274	LP274.274
54807.08	56396.8	55491.25	45833.44	53113.56	128698.2	127675.9	203792.5	129616.8	247097.9
57105.09	59349.27	59236.44	46531.72	47237.29	109268	125725.4	158926.8	139899.5	152361.6
66076.07	54286.43	63334.69	44399.11	57173.1	131440.2	126417.6	137341.9	109177	281774.9
58362.51	55512.62	56440.82	41706.26	49366.24	88352.12	83967.18	157253.6	139171.3	157745.6
58716.04	53476.9	54183.93	41820.63	41823.06	80274.81	118423.4	194966.7	111481.2	173123.3

LP274.274	LP274.274	LP274.274	LP274.274	LP274.274	LP274.274	LP274.274	LP274.274	LP274.274	LP274.274
126890.4	149535.7	211445.9	80191.62	187982.5	122540	152719.6	144029.9	245064.7	100952.3
113631	158893.4	174152.3	137891.1	197519.6	135836	179786.6	142313.8	158189.2	118561.3
165981.7	217890.8	169045.7	87568.06	276042.5	126229.6	256019.5	174284.6	211853.2	138535.7
115739.8	167378.3	278518.9	117397.7	177421.5	124665.6	182755.7	148143.5	178410.5	123068.1
127585	175275.4	195308.6	157548	172298.2	119753.1	193096.7	141644.2	193678.1	103727

LP274.274	LP274.274	LP274.274	LP274.274	LP274.274	LP274.274	LP274.274	LP274.274	LP274.274	LP274.274
165184.4	125521.9	142665.7	158826.5	117996.3	191666.7	84644.88	130823.8	171471.7	138800.6
145573.2	128716.6	134192.7	152281.2	153921.2	162517.8	90294.6	125556.9	182193.2	194711.7
158769.8	122764.2	162578	179544.7	212655.9	145585.8	87344.16	157735.2	114716.9	173819
165527	165045.2	129640.1	144703.8	159782.8	154557.6	91530.23	117597.5	128159.6	158969.1
169562.9	114569.7	143276	143094.6	101962.5	198050.9	81532.48	123707.8	185577.7	236636.2

LP274.274	LP274.274	LP274.274	LP274.274	LP274.274	LP274.274	LP274.274	LP274.274	LP274.274	LP274.274
98113.03	120918.3	72715.05	184011.2	112688.1	231503.8	119583.4	167442.2	167773.4	197659
96861.11	119381.7	74108.12	139823.7	111200.3	139829.5	118676.4	127030.5	118281.2	154047.2
106598.3	94888.36	79646.28	139157.4	154312.7	149299.7	235866.8	116158.8	120926.2	160664.4
89751.17	117363.8	72910.42	137729	104117.6	153407	114111.9	114121.2	116824	151694.5
100134.5	100345.7	73515.82	137310.6	115667	145116.4	102696.8	130324.4	119096.1	150839.5

LP274.274	LP274.274	LP274.274	LP274.274	LP274.274	LP274.274	LP274.274	LP274.274	LP274.274	LP274.274
179374.9	158151	123134.8	124788	115214.8	98882.91	116569.9	89746.57	128195.2	83829.1
177261.2	147021.9	121828.4	120687.1	114706.8	89237.58	114604.6	95150.65	130393.3	80454.25
144677.7	151863.7	118404	119841.6	116249	110577.8	97926.25	107810.4	130034.6	82687.75
174660.7	139710.4	118477.2	115123.4	119702.9	92588.86	111552.6	91895.85	118437.4	84727.5
211984.2	134848.1	95973.75	150204.5	110651.6	96112.96	112103.8	88232.29	128111.1	81974.12

LP274.274	LP274.274	LP274.274	LP274.274	LP274.274	LP274.274	LP275.163	LP275.258	LP275.277	LP276.180
77926.25	72953.32	89390.52	67039.84	81939.56	115551.3	12054.56	92428.73	21827.75	76403.62
67326.42	73035.01	85221.42	63376.84	84245.01	108848.7	13441.06	96592.43	20218.07	80539.51
67287.1	72832.43	81684.23	57918.11	90136.03	111472.4	12593.46	96901.91	24985.76	82702.65
70450.56	68271.84	82053.8	60251.64	80684.61	96946.42	11010.64	103067.6	20882	80106.23
62946.25	79216.72	84515.11	78899.21	88182.44	105862.7	14140.37	87980.34	23662.75	78875.4

LP276.261	LP277.18_	LP277.18_	LP277.179	LP277.18_	LP277.180	LP277.216	LP277.216	LP277.216	LP277.216
12820.21	54356.09	63195.48	62853.31	71268.77	44893.6	71467.19	59737.16	38917.15	41406.7
10394.33	47822.69	67218.22	67366.06	61470.84	45231.13	69982.66	59076.5	33055.14	42980.74
8286.782	48526.78	72512.1	68487.88	63933.74	44028.03	79242.53	60437.52	39807.33	45280.63
7660.09	39118.1	71968.46	66366.62	75069.93	46573.89	44809.95	31821.46	25174.38	25889.74
10615.86	45237.92	65397.59	65585.5	65194.07	43419.39	49705.34	35544.46	37079.88	40442.74

LP277.216	LP277.216	LP277.216_	LP278.159	LP278.159	LP278.159	LP278.159	LP278.16_	LP278.159	LP278.160
49417.08	62928.41	67574.65	56918.58	85973.94	62336.41	61530.01	58043.51	70170.08	63662.31
52110.9	61510.2	69189.46	83477.98	71316.35	58567.34	99548.84	58909.62	67060.07	54867.52
52129.33	77178.22	65480.89	82559.61	64589.05	65183.67	67446.85	52708.56	72000.95	65090.63
40102.89	66651.53	74770.73	83595.96	64800.61	43733.17	63269.57	56542.75	62629.79	100480.2
38461.56	52191.27	54531.65	61360.82	95136.11	82381.42	64700.5	45270.03	70213.04	64755.87

LP278.159	LP278.160	LP278.160	LP278.16_	LP278.160	LP278.160	LP278.16_	LP278.159	LP278.159	LP278.160
75853.23	81876.62	60579.64	66218.61	77815.69	73440.01	61703.29	59927.81	78962.16	60348.61
108891.6	74436.79	60669.98	80070.26	74745.26	70117.31	67624.1	57767.89	80190.83	60498.65
81140.02	74223.97	61438.89	55781.92	78110.27	68144.71	70732.49	56703.15	70529.65	56075.4
66722.84	79605.07	88273.45	52373.94	74986.49	75603.41	67043.15	65411.13	70158.13	64408.57
76161.92	78309.41	66765.94	49387.79	75757.02	72038.74	64394.01	54039.25	68861.65	60702.24

LP278.160	LP278.160	LP278.159	LP278.160	LP278.160	LP278.160	LP278.16_	LP278.159	LP278.160	LP278.16_
93182.06	55235.2	90126.25	81295.29	48468.19	64764.66	51936.07	99778.3	58018.02	65533.71
93850.07	48967.62	93719.86	82096	42816.39	73283.86	50890.57	100940.3	61612.81	61983.2
89134.56	46196.72	88467.87	86635.93	48151.75	72814.82	51650.83	98848.34	57935.11	65545.13
91163.01	50442.68	99125.2	83154.05	44009.31	68168.87	55944.92	98989.66	65793.37	62461.44
92908.82	49469.48	81614.09	87317.74	51776.38	67772.58	51589.23	101996.7	64517.33	63147.08

LP278.160	LP278.160	LP278.16_	LP278.159	LP278.160	LP278.16_	LP278.159	LP278.159	LP278.160	LP278.245
49717.41	56034.23	41101.75	58491.59	41699.28	47130.15	66912.31	45402.18	68304.7	54954.13
50532.66	61884.75	51170.9	56395.04	42105.27	49823.48	58315.08	54341	71751.42	57261.9
45430.56	47688.82	37638.03	56376.65	46365.55	46363.19	60584.79	45407.75	71034.53	56807.94
49288.23	60632.17	41870.78	58313.79	47064.27	47280.12	64006.91	44242.91	72612.14	61575.57
50269.3	59747.14	44597.07	56282.34	39046.93	46931.5	69459.06	44469.28	73409.95	54758.28

LP279.231	LP279.231	LP279.231	LP279.231	LP279.231	LP279.231	LP279.231	LP279.231	LP279.231	LP279.231
17291971	842146	656393.3	237003.2	15493309	1301529	19451905	15816128	14552705	19423576
17153979	13377056	18547664	15564196	16494807	17689197	17743463	14350941	13879914	20456805
3095081	1477901	1188313	174302	19468352	2334944	3436426	18665923	17120542	21442160
3867585	15988716	19969400	17662634	3010433	19582001	21594314	17146942	15030014	18946945
22179561	15391278	20106279	16727612	19092947	18789400	21154099	16440252	15867902	18324304

LP279.231	LP279.231	LP279.231	LP279.231	LP279.231	LP279.231	LP279.231	LP279.231	LP279.231	LP279.231
10036560	13779888	9174219	9826252	6354889	10177655	9855479	9326263	17850735	8539955
9391394	13112586	8624811	9226612	6445184	9661642	8999870	9121807	17370336	8449558
13282186	197190.9	10227521	291769.7	7635411	332208.3	12364056	12835353	20184757	48830.44
11854805	15024809	8928463	10454539	6226072	10382844	9587131	10736875	18000403	8923395
11384954	14162340	7872821	10583320	5900914	9965056	9233544	10432362	16936517	9086285

LP279.304	LP280.235	LP280.263	LP281.048	LP281.050	LP281.136	LP281.137	LP281.137	LP281.136	LP281.136
15909.64	48274.44	51671.43	42765.22	30315.11	71043.94	61507.12	60230.31	74335.02	47199.65
18505.99	674275.3	48925.39	41849.88	34269.8	45866.79	51719.14	43593.79	73581.16	53866.45
17450.34	710670.9	44676.54	38703.39	31777.45	43633.51	53762.6	39890.47	56810.7	60116.56
12134.74	752616.3	48659.02	41424.86	30962.51	34417.93	58699.08	40241.32	91589.58	61957.23
13476.02	709037	44380.28	37287.87	31722.01	32152.59	51859.24	39053.69	65459.74	55536.09

LP281.136	LP281.136	LP281.136	LP281.136	LP281.136	LP281.136	LP281.136	LP281.136	LP281.137	LP281.136
71800.44	45055.41	45364.28	56838.09	61530.97	58891.99	36173.47	42868.69	56410.6	43044.45
63318.55	40386.64	56381.14	60043.62	54489.25	54636.34	59160.3	40879.86	55233.49	44590.01
68496.84	44579.17	50094.95	63106.95	58373.49	56650.72	56478.52	59357.52	52471.73	47589.09
66629.95	44805.07	47908.1	71028.66	51650.24	57928.62	57546.01	66029.86	57373.17	43173.87
76279.27	45022.56	51313.15	58310.15	57513.36	61010.04	58965.71	57355.65	61522.06	43468.35

LP281.136	LP281.136	LP281.136	LP281.136	LP281.136	LP281.137	LP281.136	LP281.136	LP281.136	LP281.136
52955.49	37831.05	43298.55	62669.01	59735.57	68923.91	41221.93	46516.38	37497.89	44953.86
46453.76	40930.33	58755.03	59378.9	70948.65	58388.5	42774.69	49843.35	36306.7	56375.28
48772.55	36858.32	57892.57	63782.49	69901.31	54167.95	40887.32	65967.22	41971.86	53529.48
47502.19	38857.19	66327.74	65132.95	72994.27	49573.68	41463.83	45702.88	38844.33	47355.39
50668	34394.7	58356.67	58220.59	54550.76	62886.2	37734.49	50414.04	33491.1	48524.21

LP281.136	LP281.136	LP281.136	LP281.136	LP281.137	LP281.136	LP281.136	LP281.136	LP281.136	LP281.137
37371.26	66279.6	48919.74	48133.92	46026.76	51657.42	54042.91	51280.23	75139.21	67212.45
37500.95	62486.11	63310.24	51989.75	53959.04	57896.76	43830.82	51498.38	72609.89	65766.11
40594.43	60035.43	63503.03	51993.98	49132.35	59677.19	47042.16	52230.79	77657.76	65578.43
34739.22	63195.28	63530.92	53625.37	42311.85	52677.53	45252.33	51813.49	77161.5	67556.1
35362.82	62711.21	56334.23	42100.33	52435.31	47056.59	44696.17	48503.57	70426.95	68692.89

LP281.136	LP281.136	LP281.136	LP281.136	LP281.136	LP281.136	LP281.136	LP281.136	LP281.136	LP281.137
60769.13	55227.9	60360.7	44083.89	69251.35	44771.2	37737.59	74066.15	52288.74	64468.44
61687.95	50541.12	59056.09	44649.55	66425.69	43228.64	37494.56	70473.76	49495.83	63325.45
58726.81	50627.39	57744.97	45031.2	67658.66	47231.14	36498.49	74131.91	45120.37	63136.4
60720.41	48775.48	57459.4	41346.63	68395.98	50878.15	34268.46	73110.76	46380.46	57204.7
59476.3	51715.43	60410.4	45176.32	67656.98	42795.01	36174.94	73470.55	50355.83	58682.03

LP281.136	LP281.136	LP281.136	LP281.136	LP281.136	LP281.136	LP281.136	LP281.136	LP281.136	LP281.136
55598.59	48412.52	63522.36	45084.57	73566.81	76121.82	47185	50791.67	52273.45	21757.23
54275.62	52252.36	64405.14	51306.84	76769.07	72042.88	44307.89	52960.53	49418.06	23295.19
61367.61	50509.01	58431.49	53269.44	72699.84	71452.23	47892.81	58461.22	54428.15	23068.67
61881.94	52131.29	58694.71	50692.16	75958.18	70774.7	48061.1	50988.68	53477.04	22066.95
56161.65	49008.01	61888.47	46754.14	71738.82	75101.33	46568.87	47982.94	49612.84	21531.89

LP281.136	LP281.137	LP281.136	LP281.165	LP281.165	LP281.165	LP281.165	LP281.165	LP281.164	LP281.165
39469.55	33941.86	50698.99	86424.69	214213.7	358569.8	212276.7	364313.1	205895.1	289921.1
32475.05	28233.24	42940.86	82090.58	216269.1	323356.3	208241	366430.4	202851.7	278110.6
43308.92	31437.53	47496.63	83495.85	221394.5	314948.7	225993.8	348222.5	216019.1	252410.5
39040.5	34451.05	42666.58	75772.11	222262	334909.6	248729.5	350043	197189.4	252297.5
35335.67	28179.41	40991.73	72984.23	205259.5	316982.1	231582	369230.8	211043.6	286500.2

LP281.165	LP281.164	LP281.165	LP281.165	LP281.165	LP281.165	LP281.165	LP281.165	LP281.165	LP281.165
295438	149256	281485.2	200005.2	246771.1	259060.2	221196.8	266347.7	363938.7	148496.8
296069.8	155060.7	300272.9	208919.3	234482.2	258748.4	212596.1	243288.8	391216.4	149462.5
273871.1	168424.6	291667.8	204037.7	213975.7	241240.1	220423.1	271362.4	358548.5	140481.2
281753.8	167504.7	316690.6	196303.6	241428.6	240023.2	225245	266576.3	364219.5	147339
281883.7	166782.8	313959.7	201248.3	211527.4	264794.6	213085.1	245823.2	360219.7	159629.6

LP281.165	LP281.165	LP281.165	LP281.165	LP281.165	LP281.165	LP281.165	LP281.165	LP281.165	LP281.165
297340.4	229255.8	302538	240372.1	198153.2	272777.3	173328	232497.5	177086.6	265619.3
289639.5	216918	310479.8	254786.9	191270.5	274788.4	177742.1	236324	193741.5	252664.5
291802.4	231455.9	304993.6	242624.2	202823.4	307435.7	176865.8	242549	185640.3	265585.3
300432.4	231168.2	312673.2	265861.6	212940.5	332203	167384.7	242461.7	177975.4	283542.7
292534.8	232874.9	309330.8	255835.2	203558.3	329283.8	184907.6	271523.8	174765.7	278209.3

LP281.165	LP281.165	LP281.165	LP281.165	LP281.165	LP281.165	LP281.165	LP281.165	LP281.165	LP281.165
233950.5	197139.9	243893.9	278997.4	219800.2	179118.6	255748.8	161661.7	349574.1	212399.9
209572	202861	233576.1	291264	226467.3	173915.3	252700.8	155538.2	327259.2	202149.2
225085.7	193683.7	236671.7	275257.6	220910.2	159598.8	247114	166525	344784.9	208594.7
246021.7	225666.5	242792.6	302753.4	208613.1	163283.1	258380.4	164240.8	335653.5	196910
207606.5	213784.9	253288.1	285991.6	199388.3	172148.4	269516.3	150522.2	336817.6	197920.7

LP281.165	LP281.165	LP281.165	LP281.165	LP281.165	LP281.165	LP281.165	LP281.165	LP281.165	LP281.165
208204.4	330631.6	221074	175951.8	242257.1	264565.1	191820.6	128047	174182.9	159921.7
211797	319121.8	203149.1	164875.8	233701.8	265769.7	184532.9	139878.5	177173.6	170646.5
218868.1	320312.3	233358.4	186713	225500.8	251205.5	194839.3	136914.1	168506.1	156585.6
206063.8	357233.8	228881	190087.7	256201.3	278314	193458.2	135299.2	178345	156208.5
199054.9	369149.6	208837	193870.9	246671.3	279659.9	194177.9	134971.9	178154.7	166984.2

LP281.165	LP281.165	LP281.165	LP281.165	LP281.165	LP281.165	LP281.165	LP281.165	LP281.165	LP281.165
131380	134927.4	243578	138802.9	182905.4	153455.6	175121.2	171178.2	169545.9	229384.9
137489.9	133135.9	260099.2	138715	165055.4	145602.2	169330.5	167059.2	163880.5	232945.7
122633.2	136863.7	244901.8	135155.5	184815.2	144545.1	160979.5	175505	166069	221324.5
136926.1	139049.6	255120.8	128703.7	196835.6	140353.1	158284.2	166151.8	167933.2	241695.8
135939.8	135688.8	245193.1	127860.7	183754.7	140920.4	173715.3	170960.9	161743.3	223466.6

LP281.165	LP281.173	LP281.174	LP281.173	LP281.190	LP281.210	LP281.211	LP281.210	LP281.211	LP281.210
66745.66	79039.72	337722.4	275764.2	18274.18	220147.7	244290.3	164504.2	53062	32651.9
71819.69	79217.05	361522.9	269251.3	63299.64	239985.5	258194.4	169563.8	275679.7	183308
68229.38	78595.72	353427.7	287126.8	24005.69	247762.5	249437.4	183492.6	265731.9	163184.9
67598.94	83346.45	362123.9	302127.6	24755.24	119244.1	96278.68	59392.71	275430.6	173672.9
70157.78	81383.53	381863	315422.2	73223.89	129287	268016.1	173753.1	280276.6	178880.2

LP281.211	LP281.210	LP281.211	LP281.211	LP281.210	LP281.247	LP281.247	LP281.247	LP281.247	LP281.247
103663.2	227959.8	232189.4	337233.8	439026.1	330984.6	249784	250272.4	292767.6	284808.8
113968.9	237439.1	237217.8	355177.5	444864.5	331710.3	217433.9	261504	282902.9	284530.4
110178.3	249216.6	245442.4	353282.4	462296.8	359535.5	252793.1	283459	301410	314042.3
44301.06	245400.7	240134.6	111819.1	511654.4	375836.8	232951.4	261842.2	313536.2	303193.6
116040.5	88581.39	263785.5	369614.8	172788	169161.4	251138.6	270907.6	322582.5	311481.4

LP281.247	LP281.247	LP281.247	LP281.247	LP281.247	LP281.247	LP281.247	LP281.248	LP281.248	LP282.168
210238.7	229970.5	76485.68	285334.8	403601.7	263189.8	245249	299537.5	250730.2	128427.6
214606.9	225442.7	190490.1	324565	374132.1	262112.1	246291.5	275308.8	242304.8	141179.4
210513.9	219964.4	188594.9	327064.9	373602.3	276596.7	222239.3	266939.3	255834	121762.7
241758.6	231094.4	193047	333971.2	419311	288923.6	240521	296121.9	243415.4	118175.6
219358.4	240961.6	189257.1	343331.5	173204.2	279028.8	263047.3	269056.4	239842.9	135988.4

LP282.168	LP282.168	LP282.169	LP282.168	LP282.278	LP282.279	LP282.279	LP282.279	LP282.279	LP282.279
130283.8	145545	102992	150903.3	324230.9	348944.5	714521.1	369218.9	428168.8	414571.6
110022.7	136556.7	110279.1	145074.9	424105.6	258202	423088.3	379537.9	382907.1	285115.6
102644.5	136132.7	106540.9	136058.9	277943.8	305485.3	793197.6	475185.9	373881.3	289568.9
108116	137563.7	104865.8	140099.1	279193.1	359387	568079.9	402122.9	438125.4	371092.9
135003.6	152622.3	125373.3	172249.7	526868.5	308672.7	802801.6	330997.5	348581.7	252439.3

LP282.279	LP282.278	LP282.279	LP282.279	LP282.279	LP282.279	LP282.279	LP282.279	LP282.279	LP282.279
269625.7	243217.4	437652.8	826243.4	183405.8	618951.6	428921.8	693710.7	727800.3	511305.7
409221.6	279412.7	565508.7	760406.8	205230.9	634016	324098.2	658080.2	791007.2	587142.3
282116	259125.8	545767.5	638916.7	194382.2	687382.7	386271.6	727985.8	542399.4	352911.1
315864.2	329740.6	511617.6	659783.4	225711.1	837371.2	299497.3	666101.6	526195.6	365792
259220.4	224378	527703.1	925004.7	174695	585713	421694.8	538247.8	509711.4	354408.1

LP282.279	LP282.279	LP282.279	LP282.279	LP282.279	LP282.279	LP282.279	LP282.279	LP282.279	LP282.279
393334.8	455906	281318.3	450601.4	307746.1	451191.2	350276.8	382089	694774.6	475152
301117.2	520933	335943.7	467997.7	349357.5	521834.2	382694.6	256989.2	641218.4	425766.9
319899.6	372727.8	309592.4	441401.4	349291.4	485283.4	573407.4	379301.6	649172.9	538740.7
375883.3	555546.2	272786.3	400180.2	286512.8	476677.1	285200.9	356372.3	651756.7	415408.3
345233.4	465128.2	279536.4	383792.8	308156.8	317796.9	408327.1	366321.2	643308.7	373136.1

LP282.279	LP282.279	LP282.279	LP282.279	LP282.279	LP282.279	LP282.279	LP282.279	LP282.279	LP282.279
255940.1	114154.2	445711.2	320152.9	288776.4	355604.3	399478.9	329134.7	321113.8	348375
343964.4	117168.9	435167	353359.7	476297.2	333007.5	520113.2	325863.3	439282.2	372987.2
341207.5	145223.3	443486.8	471006.5	405392.3	434745.5	461939.2	444405.3	501485.5	364665.6
269675.7	128845.6	357241.8	326655.2	347010.4	314411.8	538152.5	529112.2	668320.7	363889.4
377131.5	116025.8	383118.2	332210.9	342381.9	457062	461846.3	308696.4	336143.3	377407.3

LP282.279	LP282.279	LP282.279	LP282.279	LP282.279	LP282.279	LP282.279	LP282.279	LP282.279	LP282.279
103343.9	302282	551058.1	394416.6	325109.4	439133.6	210849.8	212458.8	490562.7	186954.9
208109.9	376619.5	435798.6	387989.4	250338.4	430346.1	195126.5	258827.9	488080.1	292243.6
208833.1	409755.1	400368	479125.8	264744.8	391974.5	288070.1	266027.9	458661.3	208269.1
188614.9	384545.5	401637.7	415468.7	326256.9	427663.9	213990.4	215233.5	458672.1	301463.4
172387.9	385094.8	384865.8	351273.5	327494.5	383454.4	207989.9	200460.8	508823.5	201382.1

LP282.279	LP282.279	LP282.279	LP282.279	LP282.279	LP282.279	LP282.279	LP282.279	LP282.279	LP282.279
186269	217522	201303	223348.9	389693.3	310248.7	241992.2	315583.1	195788.7	162861.2
209084.3	232317.2	205373.8	225954.3	390222.2	330760	274731	307068.4	215225.1	169513.8
205303.3	213151	194832.3	209019	394267.7	273930.2	247865.4	322142.1	211906.8	168932.4
185694.5	215293.1	287601.3	209308.7	382212.2	185907	246125.4	312773.1	203861.9	173696.3
198646.2	208508.8	181693.1	207696.1	378998.5	308087.4	221464.3	304950.4	199462.7	177012.5

LP282.279	LP282.279	LP282.279	LP282.279	LP282.904	LP283.115	LP283.115	LP283.115	LP283.115	LP283.115
165418.9	222802	159468.3	197802.6	13659.08	135390.2	105823.8	116198.5	130839.9	215143.2
170006.5	232534.8	158790.2	166411.5	16764.89	167642.2	194885.7	120914.1	157949.8	104575.9
163943.8	288208	157102.1	215993.4	11631.55	99208.71	117476.8	108748.7	99460.02	164944.2
177122.8	240013.3	172861	197267.2	6860.812	143399.3	123700.4	107035.6	122902.2	187510.9
191758.9	176129.8	148205.8	210114	11918.91	145060.2	144567.7	139003.1	109850.5	135411.3

LP283.115	LP283.115	LP283.115	LP283.115	LP283.115	LP283.115	LP283.115	LP283.115	LP283.115	LP283.115
145835.8	234368	160676.4	145915.1	97195.29	123201.2	211142.2	169716.7	141309.1	132597.8
115839.2	176155	120306	124091.5	97008.85	135546.2	153030.2	187571.7	133035.5	102438.2
172596	115882.2	150829.8	128206	122779.6	149617.8	113761.1	100957.3	146343.3	113115.9
127654.9	148343.9	186506.2	109541.5	123955.1	165842.1	125207	182670.3	101281	128950.7
169760.6	190345.7	104151.4	114521.7	101928.3	123974.1	206020.2	128723.7	118912.9	154186.7

LP283.115	LP283.115	LP283.115	LP283.115	LP283.115	LP283.115	LP283.115	LP283.115	LP283.115	LP283.115
193469.9	134745.3	192135.7	110171.5	148203.8	144849.9	105075.5	119690.8	109533.7	102375.2
108229.9	189024.3	163624.7	139157.5	82627.78	123790.6	106575.5	151571	152420.3	126184.9
130098.6	123292.9	178653.9	140997.1	132137	166695.1	107519.9	106151.2	157348.1	131380.8
148237.6	125929.8	181658.1	131497.3	141121.9	146805.4	178785.3	141279.1	94220.19	135899.1
114730	176703.9	104329.2	151620.1	110268.6	189701.2	152295.1	114612.3	117601.7	109644.7

LP283.115	LP283.115	LP283.115	LP283.115	LP283.115	LP283.115	LP283.115	LP283.115	LP283.115	LP283.115
100716.6	129885	130963.4	151530	144973.8	177006.7	146580.2	118458.1	129794.4	133799
127927.7	111437.8	145517.8	131165.5	130746.2	120776.8	142359.6	170154.4	127583.8	136696.3
127793.4	149755.3	136452.2	115009.4	147376.4	159252.4	178355.5	138998.5	177161.6	140228.3
99384.83	173355.8	123490	191648.9	132823.1	134976.4	109271.9	128369.8	145462.7	142670.4
183130.6	164003.2	154661.3	130993.8	137173.4	183881.6	171817.7	98204.61	133394.6	119957

LP283.115	LP283.115	LP283.115	LP283.115	LP283.115	LP283.115	LP283.115	LP283.115	LP283.115	LP283.115
108776.8	114367.8	118803.1	158653.1	200377.4	150090.1	125919.9	178349	91517.22	127660.6
113363.6	213508.6	118376.7	156379.8	213076.5	158818.9	118335.4	209378.1	95025.01	83979.79
119525.8	156034.5	214521.6	94260.78	160369.4	181938.2	112449	130274.9	114247.5	89294.41
121145	112200.9	158683.5	112490.5	155322.4	159965.2	102800.2	160639.6	137236.1	105937.1
106843.5	130033.9	152292.7	86217.02	174992.6	129787	150629.2	116214.6	97037.33	122252.8

LP283.115	LP283.115	LP283.115	LP283.115	LP283.115	LP283.115	LP283.115	LP283.115	LP283.115	LP283.115
144842.7	117478.4	116735.4	81480.68	156697.1	155017	133182.9	133122.8	144440	176353.9
188005.4	125594.7	126704.2	80139.58	141696.2	170460.9	135036.9	119763.7	150860	127454.9
110919.1	169789.2	152366.4	76359.98	176670.6	140087.6	167131.2	116139.5	150503.6	120322.8
134027.8	109207.8	165361.5	73066.48	134305.7	182710.9	118384.7	113907.9	86794.58	136671.5
128672.2	155063.2	115242.7	71624.11	122667	173431.3	116283.9	128544	109307.6	145647.5

LP283.115	LP283.115	LP283.115	LP283.115	LP283.115	LP283.115	LP283.115	LP283.115	LP283.115	LP283.115
104778.3	110888.7	73972.2	126877.6	91199.5	153822.1	107061.3	141565	132583.9	117954.7
82584.31	120223.9	65123.99	119734	121560.6	115370.2	123795.8	87232.02	110379.7	134765.5
130520.5	114229	69161.87	145157.7	117807	91027.28	135306.7	107544.4	118266.4	105353.9
117868.1	137273	62463.28	131815.9	108532.5	158927.6	131657.9	125860.1	98827.1	109342
87870.06	189836.7	60700.81	141682.9	121577.9	124720.8	119991	109074.1	114029.2	80246.99

LP283.115	LP283.115	LP283.115	LP283.115	LP283.115	LP283.115	LP283.115	LP283.115	LP283.115	LP283.115
135751.8	128713.7	111909.3	67270.31	118414.3	82910.63	122024.6	58801.6	69897.02	64548.56
91903.14	162735.9	111223.3	59246.09	99827.11	59702.59	94429.84	73051.06	66985.24	63050.12
91711.05	132618.9	109653	48840.6	95350.78	57521.25	82830.22	60558.18	76132.41	70218.34
130615.3	183712.3	113676	57951.54	103241.9	53728.99	114869.3	53828.31	53634.69	55502.2
105564	140067.8	122526.3	81449.69	96701.81	58190.34	96497.93	55542.26	56834.08	54831.43

LP283.115	LP283.115	LP283.115	LP283.115	LP283.115	LP283.115	LP283.153	LP283.152	LP283.153	LP283.152
60702.31	72993.74	66680.73	59129.29	68325.34	63060.22	70099.24	69666.2	75805.45	62615.49
59965.98	68692.91	49666.73	61499.34	65998.43	60992.46	78559.75	57025.72	69468.18	61167.32
62431.5	62562.23	53758.87	56361	58822.54	74609.27	73783.9	58037.31	71932.98	59987.74
54728.37	77219.9	45495	56239.59	46086.16	60301.54	74092.83	58199	70971.77	65846.8
60423.34	60528.03	40886.77	53521.76	68424.8	55484.96	80544.6	73181.86	70411.45	62936.62

LP283.153	LP283.153	LP283.153	LP283.153	LP283.190	LP283.190	LP283.190	LP283.190	LP283.19_	LP283.190
73979.43	73325.15	97436.87	108466.2	96481.07	70533.98	76494.14	59733.28	76312.14	54201.31
67027.29	67853.08	96934.85	102156.9	89175.16	70485.94	76774.52	63687.16	76420.02	52719.53
71381.83	71432.07	101107	101969.5	90663.73	70387.26	73227.83	59684.67	76264.2	59701.51
77860.96	70123.41	99063.87	110163.9	106299.6	67073.42	78850.97	64670.69	79807.18	58234.8
76774.45	68328.28	100729	105636.4	102090.1	81326.58	76851.91	64916.34	80961.03	58844.12

LP283.190	LP283.190	LP283.190	LP283.190	LP283.190	LP283.189	LP283.190	LP283.190	LP283.190	LP283.190
54263.14	90811.49	69739.2	82209.24	92362.76	71336.22	60656.59	87944.05	48558.85	102828
53275.89	88565.87	68069.33	85502.96	81694.09	71038.2	63818.52	97546.05	47371.94	97964.01
58854.09	87927.74	76156.46	79299.76	85643.63	63554.2	64734.75	92325.74	49834.49	102304.5
61398.35	86708.04	69676.41	79825.33	97504.7	68061.65	66782.71	98640.74	46737.5	100115.4
53119.88	91459.72	71356.88	76621.6	97399.29	71300.75	64218.67	94641.19	51104.92	102788.3

LP283.19_	LP283.190	LP283.190	LP283.19_	LP283.190	LP283.190	LP283.190	LP283.190	LP283.189	LP283.190
61073.3	89824.95	67446.36	46633.05	106719.7	71272.77	61383.87	92291.88	98624.74	97763.01
66951.3	91213.33	69399.52	47015.63	110881.6	63117.32	55928.34	100882	87376.54	99678.87
73459.86	91692.85	72032.56	50724.75	112976.3	69369.29	61032.3	94763.31	89671.05	101565.2
68956.51	96076.33	70257.28	50399.07	114320.5	65464.57	61678.25	97265.6	87807.98	100607.4
78235.68	98419.82	71322.67	52491.74	117398.3	70905.63	65070.07	96182.53	99211.8	101243.5

LP283.190	LP283.190	LP283.190	LP283.190	LP283.19_	LP283.19_	LP283.190	LP283.226_	LP283.226	LP283.225
66394.55	67192.24	80106.3	70226.39	83469.7	84952.14	95388.23	62547.33	63372.66	62123.45
65195.06	65396.93	86384	68557.42	88231.75	85791.34	98719.33	65153.36	70020.46	61360.71
71595.67	71519.61	88598.38	65939.1	86103.02	86730.6	96312.32	59743.54	67455.41	57234.78
64484.14	66837.71	84866.44	64892.24	84436.59	84074.97	89217.79	61874.92	69453.45	61862.92
68730.49	70002.99	93850.96	69782.32	86738.47	89893.92	94957.14	63853.17	70159.28	57020.5

LP283.226	LP283.226	LP283.225	LP283.225	LP283.225	LP283.226	LP283.225	LP283.225	LP283.225	LP283.225
92515.77	69595.31	72910.62	99342.04	83047.85	54324.85	102270.8	94881.89	80433	97663.35
99034.32	66309.74	71981.46	103360.6	81689.09	54224.23	102910.6	106233.5	71447.54	91432.7
87638.05	76096.57	77572.6	101801.9	86382.64	56703.64	105604.2	101611.6	77236.73	97606.65
88762.4	70578.24	73922.01	105542	85486.43	55666.44	106092.1	102169.8	75163.49	96848.85
85474.25	72275.59	66025.72	98710.16	88777.26	57151.42	104709.6	107620.7	77169.81	99558.47

LP283.225	LP283.225	LP283.225	LP283.225	LP283.225	LP283.226	LP283.225	LP283.226	LP283.226	LP283.225
65045.26	71846.82	101243.7	84480.72	83231.41	90833.17	65375.89	72314.93	75423.94	67955.38
66382.06	76026.51	94051.39	85961.37	81386.99	87759.58	70307.73	80447.08	70458.99	75068.13
69187.52	74266.82	93922.87	81292.6	84779.21	88456.76	70307.91	80866.13	77058.63	71113.96
65535.63	69398.69	98419.45	88923.51	83274.54	91721.53	70645.53	80721.1	66855.1	69676.57
68068.42	81280.38	93248.81	91593.6	81873.92	89073.9	70107.9	87626.21	73411.66	70248.56

LP283.226	LP283.226	LP283.225	LP283.225	LP283.225	LP283.225	LP283.281	LP283.282	LP283.281	LP283.282
76502.63	98610.86	78521.02	103701.1	69821.04	79585.31	107406.6	124438.2	183168.2	209560.1
69552.34	101091.7	74357.03	106881	72930.24	77473.95	117146.4	124897.5	127732.9	224603.2
68566.35	104440.6	70639.31	105839.8	74825.3	84612.17	177959.1	120825.8	188418.5	232210.7
80613.91	105117.2	76578.2	102949.7	70242.62	78262.1	168942.9	105087.7	190235.6	246452.5
72467.04	103624.7	69177.98	121262	75211.52	83640.22	151247.9	126206.2	192079	235778

LP283.282[illegible]	LP283.282[illegible]	LP283.282[illegible]	LP283.282[illegible]	LP283.282[illegible]	LP284.266[illegible]	LP284.266[illegible]	LP284.266[illegible]	LP284.294[illegible]	LP284.294[illegible]
167296.7	142983.4	132978.5	160660.5	125706	143545.3	135233	121209.3	467672.6	139170.5
160848.8	147809.6	135160	147024.7	121197.7	134502.1	115235.4	133404.1	472946.1	150436.7
121096.1	87806.84	127424.1	206643.9	164390.4	38931.58	36242.1	27531.38	576580.1	146229.1
169688	157607.6	133798.2	139771.7	185090.9	123395.1	119036.4	128957.9	632864.5	164691.5
174129.2	163284.5	131695.6	232275.4	192428.7	142946.2	116737.8	115717.4	656947.7	133179.4

LP284.2947	LP284.2949	LP284.2945	LP284.2949	LP284.2947	LP284.2946	LP284.2949	LP284.2948	LP284.2945	LP284.2949
301573.1	114064.7	72792.69	138107.2	134165.8	165426.5	149007.6	131912.8	81797.54	132632.1
321104.6	110782.1	76308.73	135772.7	144192.1	168531	146208.2	123131.8	79477.58	121145.1
324172.8	127012.4	93784.9	153261.1	144280.5	168318.9	145167.1	130562.4	79636.98	172420.1
351085.5	121156.4	115183.9	125884	147274.7	153718.1	141844.6	119208.5	79415.07	131900.7
336082.9	137073.7	71622.99	152790.4	143278.3	164929	157999.9	128305.3	79508.51	118795.6

LP284.294	LP284.294	LP284.294	LP284.294	LP284.331	LP284.331	LP285.130	LP285.130	LP285.130	LP285.130
95508.17	89128.08	86788.53	82896.52	809415.7	317803.3	210406.5	113009.4	189456.1	111002.9
99360.06	82257.46	82812.13	87013.74	609268.4	329449.4	152783.3	140629.8	161494.2	117796.9
92576.98	92759.55	80265.4	94332.9	517099.7	370131.4	164753.4	128249.1	158217.5	152286.6
96777.48	84183.94	81093.85	88394.88	709877.4	407911	181006.1	126427.5	185291.2	148802.4
94826.14	99877.99	84679.01	87916.36	689061.1	422230.3	209140.9	181207	185981.1	153014

LP285.130	LP285.130	LP285.130	LP285.130	LP285.131	LP285.130	LP285.130	LP285.131	LP285.131	LP285.131
151113.9	137539.4	178425.2	181018	115461.6	126673.2	157137.9	140360	124976.6	172877.4
114670.6	173291	125422.3	179867.9	133404.1	181968.4	117239.6	229981.4	154648.9	167881.9
151352.4	185836.5	166734.5	144126.9	127106.9	125338.2	206068.8	154007.3	135956.1	153573.9
175489.1	130815.7	182913.2	140286.2	107588.3	154800.9	116507.7	183932	132992	178952.9
165707.2	125805.4	175171	146385.7	124581.8	203672.4	109328.9	201620.9	112637.4	162975.7

LP285.130	LP285.130	LP285.131	LP285.131	LP285.131	LP285.130	LP285.131	LP285.131	LP285.130	LP285.130
150027.5	210202	215764.2	239663.2	204444	137103.1	122920	171536.1	150324.6	144261.5
145413.7	155898.9	195517.1	208840.7	254247.8	149359.3	120763.3	140013.9	172279.6	169949.5
154093.7	167652.7	154846.6	116258.8	198181.8	177666.5	119476.5	150682.6	154331.7	191364.3
134129	130673.1	196914.1	196886	193903	143845	151432.6	172907.9	140989.7	220258.6
140587.6	186393.9	127409.9	126685.2	216832.1	148114.6	161947.5	202225.2	160766.4	109289.4

LP285.131	LP285.130	LP285.130	LP285.131	LP285.131	LP285.131	LP285.131	LP285.130	LP285.130	LP285.131
135412.5	173322.2	234241.6	146518.7	108126.6	155622	127783.2	99920.32	155531.9	178846.6
169082.8	190336.7	188054.1	130468.5	156102.4	166845.9	126857.1	133082.6	165527.9	177924.3
155235.3	139949.2	143598.8	127327.2	164585.4	143564	154438.1	129866.2	164713.4	144292
198005.3	127686.4	180051.2	121357.4	130902.9	199784.9	138439.2	242170.5	139845.3	192906.3
206875.5	137056.3	210624.3	124235.1	141582	146476.5	177073.5	143208.8	167181.5	186626.7

LP285.131	LP285.130	LP285.131	LP285.131	LP285.130	LP285.131	LP285.130	LP285.131	LP285.131	LP285.130
157696	109366.7	171481.5	244234.2	182514.6	127588.2	159490.6	149233.7	153784.2	139636.1
158622.5	94778.95	179757.6	153464.9	201615.6	129913.7	132879.7	131578.1	151858.5	133627.9
161611.2	112558.1	153350.8	146367.1	210695.2	188666.1	129251.3	154219.7	176284.4	199986.4
142924.8	87621.04	217348.1	164131.7	185037.2	122895.8	121901.6	154718.1	180031.8	135930.4
138772.6	96284.31	165176.4	143013.7	206723.3	157118.4	144109.8	135848	183871.2	154617.3

LP285.131	LP285.131	LP285.131	LP285.131	LP285.131	LP285.131	LP285.130	LP285.130	LP285.131	LP285.131
165409.5	169122	131357	169300.9	162989.1	171113.7	138738.7	143695.9	144484	132089.4
143060	191643.9	116738.3	150522.2	235352.4	223718.4	138759	154808.4	152424.5	154540.7
165758.9	186403.5	115992.9	139245.1	160424.4	173616.9	133869	128574.7	162379.8	138017.6
170612.9	228608.8	143996.7	163401.7	171851.1	186470.5	158194.3	126475.8	142366.2	127254.8
167889.1	158670.6	97145.27	147438.1	163208.5	163331.5	187223.3	138214.2	171951.3	122743.9

LP285.131	LP285.130	LP285.131	LP285.130	LP285.131	LP285.130	LP285.131	LP285.131	LP285.131	LP285.130
129373.8	125930.9	113775.8	131391.2	250049	140830.1	118803.8	137899.4	153733.9	135120.7
130660.9	132729.9	130070.5	135594.3	172386.6	126066.6	130467.9	120600.1	164577.2	124413.1
126027.1	163303.7	122747.8	120356.8	205491.4	135368.1	133972	142498.5	139737.3	139645
169270.7	146506.5	120671.7	126420.6	179218.6	139879.3	139321.3	137303.8	123204.3	146415.4
156875.5	144871.6	121861.8	132908.8	145400.2	146566.2	117176.9	127982	129203.1	154316

LP285.131	LP285.131	LP285.131	LP285.131	LP285.131	LP285.131	LP285.130	LP285.131	LP285.131	LP285.131
132428	143564.4	97747.38	72353.67	89141.63	79513.25	78465.37	62250.08	86345.88	66419.6
127086.1	154037.2	93371.83	82753.4	78653.7	71213.6	77521.49	64527.17	86420.76	68015.89
125929.5	152629.2	101597.7	65871.05	77055.03	71231.6	73934.14	66313.43	79219.57	59082.3
123727.3	150015	92465.78	66899.68	74288.29	74641.87	73277.26	57981.91	90105.85	65068.05
148819	161118.8	91918.53	80092.56	80105.68	76737.83	74889.84	61336.68	89756.22	63507.74

LP285.131_	LP285.169	LP285.169	LP285.169	LP285.169	LP285.169	LP285.169	LP285.169	LP285.169	LP285.169
73032.15	55716.93	48658.11	55388.21	47810.43	67963.35	39224.13	149103.2	42287.56	76517.2
75947.52	52050.56	52615.82	63444.9	47771.06	70842.98	41397.96	141671.5	48425.29	74748.82
63781.24	58591.83	54850.54	58849.74	47268.24	76570.83	37451.48	144257.8	44156.22	78926.88
65013.13	52571.11	50733.66	66310.41	47068.96	74226.84	34991.28	153544.4	44533.75	76614.99
66840.56	49610.56	52260.6	65377.75	44794.94	72649.43	39220.75	145346	43504.64	72490.24

LP285.169	LP285.169	LP285.169	LP285.169	LP285.169	LP285.169	LP285.242	LP285.242	LP285.242	LP285.278
51982.47	35394.21	31360.43	47839.21	33331.9	42499.63	152709.6	86236.38	124361.8	47218.45
52620.92	32035.18	29775.99	48543.36	36761.29	40497.81	165806.7	82751.15	124073.2	50163.95
50939.38	35761	27795.19	41834.96	36488.76	45469.37	167638.5	86501.47	127840.1	48319.39
47974.29	33938.48	26879.09	50919.11	36343.17	40497.57	144730.8	86329.28	121392.7	48461.62
52966.76	31729.68	31084.64	44303.26	33553.14	40903.45	163972.1	76934	122837.1	48834.72

LP285.278	LP285.278	LP285.279	LP285.278	LP285.278	LP285.278	LP285.278	LP285.278	LP285.298	LP285.298
43468.68	43594.8	15592.31	36852.8	40367.05	40522.9	40282.27	16684.48	36194.27	29909.07
43190.36	40055.64	17374.64	41302.92	40765.7	40717.36	40264.25	19695.16	39729.15	28847.36
48978.4	40702.54	15039.16	36355.88	38910.13	40654.53	39074.75	19349.69	40489.49	28848.45
43112.1	35364.11	14698.43	32289.05	41839.54	33860.48	38701.65	19139.55	37107.12	27703.6
38582.45	40948.91	14954.21	34839.04	38168.54	39033.01	41206.39	20353.51	42004.55	26063.91

LP285.334	LP285.334	LP286.165	LP286.164	LP286.165	LP286.165	LP286.245	LP286.245	LP286.281	LP286.310
146482.5	46078.93	26743.37	32883.7	21135.06	20967.1	23456.15	17515.89	11485.88	37624.44
109224.2	53701.89	24927.71	34630.81	21172.04	22449.62	25920.14	18725.25	13951.37	31823.37
93224.21	64272.99	26608.37	34257.36	19971.21	23426.46	26907.34	19149.87	12944.32	34216.88
78330.28	64086.11	27519.92	35626.27	22807.39	21068.17	26173.42	19277.41	13242.29	31402.26
90832.11	55145.26	27999.56	34416.26	19792.58	23129.37	27374.19	15738.56	13747.13	33094.22

LP286.310	LP286.310	LP286.338	LP287.147	LP287.147	LP287.147	LP287.147	LP287.147	LP287.148	LP287.221
13860.4	36893.74	11738.7	61883.03	93195.36	50423.83	56895.99	56110.65	61839.29	104632.6
13412.35	33061.44	8305.692	67183.17	93939.25	57250.58	49692.8	56561.48	68576.38	89886.07
12637.45	41247.74	9195.252	67883.35	93053.01	48491.52	62580.94	60759.23	60960.96	95501.77
13397.65	34819.61	10532.97	66139.4	93513.12	48462.94	65728.82	54560.43	63146.18	128128.7
14270.11	35333.77	11783.72	75862.77	97795.46	66101.6	68425.16	65910.6	70861.16	83184.18

LP287.221	LP287.221	LP287.221	LP287.221	LP287.221	LP287.221	LP287.221	LP287.222	LP287.221	LP287.221
70070.38	54052.01	79313.51	34721.92	55159.73	73439.07	78254.87	74297.69	64770.99	115901
66089.95	77838.7	78672.47	38895.52	69820.21	70549.85	77151.09	66247.99	52405.49	109912.7
61060.91	53775.53	76697.43	55639.64	54004.51	76125.15	85532.85	71777.84	65196.54	116247.6
62853.13	58604.19	71838.9	36706.01	55729.61	81102.76	72079.7	70433.45	63285.78	112126
71368.17	56590.59	71175.71	48984.61	55817.75	85359.37	75119.57	68221.5	57793.17	97038.92

LP287.221	LP287.221	LP287.222	LP287.221	LP287.222	LP287.221	LP287.221	LP287.222	LP287.222	LP287.888
68901.46	62472.62	67444.58	53927.01	73837.45	52311.59	71979.38	50961.57	69683.49	75136.26
71151.81	48472.02	64125.86	52479.59	67801.35	64088.6	72085.59	45626.24	69434.22	62946.41
73246.74	60529.79	73967.91	62240.71	68044.83	59059.74	68069.14	44631.41	69173.66	104224.3
71496.09	57186.08	64853.3	51851.16	69756.14	57020.01	68790.5	48265.65	69844.12	148441.2
65814.96	58607.39	67846.93	53085.56	71218.36	56499.27	72597.63	48657.49	68387.67	59741.98

LP288.180	LP288.180	LP288.181	LP288.180	LP288.180	LP288.225	LP288.253	LP288.253	LP288.253	LP288.253
43064.8	60920.11	37531.43	47973.42	49402.35	58639.95	121389.4	57939.54	105437.3	89989.96
39714.32	59837.85	35118.82	46346.3	47151.43	56317.47	119428.3	63969.59	89936.57	96846.25
38237.58	63421.14	35032	42762.71	52827.67	51870.23	104185.2	62914.63	132298.7	94640.6
48275.17	57815.03	40963.94	53922.34	53601.16	55742.2	131232.5	80051.78	124340.2	87605.85
37710.1	60615.27	39420.17	49533.47	50303.14	49702.34	114407.4	61566.68	101205.3	95895.45

LP288.253	LP288.253	LP288.253	LP288.253	LP288.253	LP288.253	LP288.253	LP288.253	LP288.253	LP288.921
59308.11	63424.08	82709.88	45877.94	83576.65	56493.81	46762.81	67170.57	54670.55	216794.2
72915.97	54937.21	75162.32	50560.98	88577.21	59554.96	54865.77	67937.4	60023.38	250341
54674.12	55800.75	76436.26	50566.53	106155.5	56943.61	42382.51	66676.08	59575.45	214568.2
42740.91	65455.96	82625.38	46000.07	87836.61	54685.78	43917.99	63761.12	63358.88	337978.4
50672.38	53349.94	54115.92	44808.13	91228.33	55105.07	41036.08	63979.87	61414.89	283169.5

LP288.921	LP288.922	LP288.922	LP288.922	LP288.922	LP288.922	LP288.922	LP288.922	LP288.922	LP288.922
227379.5	208077.2	154247.3	253496.8	228447.5	286093	255586.1	69383.65	228328.6	118706.2
217579.2	213530.3	147957.2	278630.8	241028.4	299114.6	247645.3	77397.43	226492.7	124727.7
333661.4	165253.2	200288.4	286334.6	238486	327578.9	280232.1	67895.33	152033.7	99426.45
268459.3	172586.5	186228.6	229325.3	252675.4	236625.5	270705.8	59232.87	210646.3	153188.4
195339.8	198395.4	157731.2	281233.1	228269.1	295120.6	273512.2	75628.59	226018.3	112405.7

LP288.922	LP288.922	LP288.922	LP288.922	LP288.922	LP288.921	LP288.921	LP288.922	LP288.922	LP288.922
83176.54	84764.14	99685.34	80895.64	81138.95	229977.2	235009.7	92438.17	85678.36	135430.5
85443.14	79721.19	103495.9	75813.77	81053.33	242879	228432.5	88718.6	87714.2	133091.4
61938.95	89765.45	108397.4	94539.45	70406.18	267171.6	246824.6	80316.61	65733.67	102694.8
69782.81	74526.52	111926.4	57734.27	74079.85	277972	232489.1	58417.17	85446.22	127415
80013.49	87640.24	96722.54	75866.25	73830.31	228677.8	234426.1	80953.71	78369.07	131751.2

LP288.921	LP288.922	LP288.922	LP288.922	LP288.922	LP288.922	LP288.922	LP288.922	LP288.922	LP288.922
247742.6	33099.99	185136.5	195191.9	45957.74	167633.6	72870.41	186486.4	42639.69	40463.81
216754.3	36934.64	196719.5	200395.3	40527.75	179199.2	72715.62	208607.4	34687.76	37218.51
256338.7	39117.67	225578.8	177318.4	34855.7	140017.7	71344.32	179064.2	49890.62	41574.72
249152	44242.62	203708.4	258668.5	45777.57	196923.1	77992.96	226341.9	48379.58	31119.6
231449.4	34774.44	189205.5	179941.1	40052.62	197595.1	71594.63	195317.3	31335.7	33729.66

LP288.922	LP288.922	LP288.922	LP288.922	LP288.922	LP288.922	LP288.922	LP288.922	LP288.922	LP288.922
67227.4	233301.2	302907.5	289085.6	226308.1	189882	40444.28	94640.23	210659.8	77674.96
73196.27	241311.3	331576	297796.6	212079.2	200088.3	49224.19	111423.5	225812.3	74177.24
58543.45	230930.8	242819.9	333253.1	312675.7	197640.5	74264.28	83217.73	165118.4	74936.54
52772.39	289537	325760.4	270108.1	244364.8	266193.6	45934.95	138645	440561.5	78759.04
67773.07	214821.8	304732.7	281935.3	208021.2	204653	46507.7	101504.7	207080.8	65571.72

LP288.922	LP288.922	LP288.922	LP288.922	LP288.922	LP288.922	LP288.922	LP288.922	LP288.922	LP288.922
267220.8	218967.5	204166.2	125427.4	188405.7	190954	194482.5	146332.1	190966.4	76473.24
261793.2	226973.1	201541.3	117964.4	187398.1	177378.9	188421.1	151553	174195	83412.34
201995.1	219810	229010.7	189579.6	168881.8	218857.4	274274.8	245241.4	205297.4	68307.08
193803.2	239621.1	239504.8	137057.5	194087.1	188956.4	204202	163647.8	151238	63814.47
260833.7	248852	187438.2	133587.5	185685.9	192299	209643.6	140193.8	183832.2	73735.73

LP288.922	LP288.922	LP288.922	LP288.922	LP288.922	LP288.922	LP288.922	LP288.922	LP288.921	LP288.922
287387.2	190827.4	99286.78	213794.4	126846.9	63408.33	134327.5	247382.8	213494.1	68111.93
286567.9	213679.9	102342.6	198162	158123.4	67022.15	135937.8	251487.3	198386.9	63422.21
235662.2	233642.8	93201.91	187869.4	188983.4	85625.94	139094	245740.5	218783.5	71144.6
267666	171695.2	62586.59	175750.1	168441.2	62491.87	245905.8	183943.5	260384.9	60801.82
283123.5	183334.3	92983.72	190117.1	130187.2	62470.43	138424.3	259777.2	201175.7	72441.11

LP288.922	LP288.922	LP288.922	LP288.922	LP288.922	LP288.922	LP288.922	LP288.922	LP288.922	LP288.922
49926.66	238711.6	215746.5	238630.7	233851.4	75762.22	227336.6	328893.1	159766.8	259470.9
44123.28	247564	212034.6	213412.4	208799	84531.48	236574.5	344638.1	167741.4	255183.6
41506.41	222181.3	176747.2	234597.8	200144.5	63210.19	249567.6	346061.6	154746.6	316759.3
49064	219597.1	183666.7	271045.4	308313.5	63077.63	240501.5	292730	128893	252295.1
35114.22	261954.5	189006.1	220822.1	227094.7	75861.11	224592.5	341074.2	147786.1	246559.3

LP288.922	LP288.922	LP288.922	LP288.922	LP288.922	LP288.922	LP288.922	LP288.922	LP288.922	LP288.922
228638.5	108257.8	45863.97	60256.7	42575.68	184637	171866.3	215838.2	265955	232661.8
225120	115351.3	44378.71	61810.4	47630.77	197683.9	173774.7	201717.1	264621	209411.8
258318.7	132483	54743.35	54587.42	91230.02	264952.8	180257.6	209554.5	230029.7	189310
242844.6	112385.6	42709.14	61868.25	32927.67	308210.1	198517.2	190118.1	297094.4	276105.1
216019.8	100969.5	47319.38	59612.92	40552.5	199213.2	160832.3	204734	267339.1	224085

LP288.922	LP288.922	LP288.922	LP289.154	LP289.164	LP289.164	LP289.164	LP289.164	LP289.164	LP290.160
111256.5	65331.22	274273.4	19521.14	40134.87	35490.84	41882.31	38388.05	77218.1	52909.06
118511.6	68517.83	257252.8	16187.52	34933.57	32651.57	44552.83	31599.51	77195.67	67336.65
99350.98	64674.43	289083.8	15289.48	36873.23	31332.37	46468.52	36760.52	76782.15	59687.47
134122.7	53013.7	263474	10437.88	35664.69	28995.81	43971.69	38313.72	77986.25	78875.49
100556.6	69292.07	247932	14854.81	34890.09	31254.42	47023.91	33600.75	65290.61	60424.84

LP290.159	LP290.159	LP290.159	LP290.159	LP290.160	LP290.160	LP290.160	LP290.159	LP290.16_	LP290.16_
77609.64	71378.37	69295.84	53563.23	82423.14	55868.93	67307.03	75886.45	77501.05	59272.87
69200.26	61668.45	74019.88	67102.32	74275.94	55494.98	67241.16	62831.08	74432.26	55829.76
69175.82	64902.45	70513.76	71698.84	78977.06	51160.38	63468.13	63789.48	77279.94	53127.62
73773.13	58557.9	66826.04	55193.81	71924.41	53730.95	74300.75	74273.86	106388.3	59259.23
86990.78	64770.75	79039.81	63582.65	76187.63	54858.27	65383.66	66656.19	79793.4	58679.68

LP290.16_	LP290.160	LP290.16_	LP290.160	LP290.16_	LP290.16_	LP290.16_	LP290.160	LP290.159	LP290.160
85791.02	70095.68	86116.33	64978.68	66527.08	57594.14	56874.05	46474.88	42638.72	77572.16
84991	71958.25	83169.86	59744.05	65471.54	56245.57	56038.79	43908.51	42921.22	80604.53
74196.86	69547.69	87491.99	59474.32	66783.8	59923.56	52950.79	50920.03	44432.21	78993.04
85899.45	76088.35	93527.89	62570.48	63352.28	55871.26	59002.91	45420.4	44798.69	77377.82
84315.74	76319.97	91647.64	66027.82	63092.33	53275.68	59091.55	44989.33	43987.47	81782.34

LP290.160	LP290.159	LP290.160	LP290.160	LP290.16_	LP290.159	LP290.160	LP290.160	LP290.160	LP290.16_
43832.62	78125.42	42157.18	54658.02	77503.29	53956.04	72698.37	79637.66	65815.3	81040.35
40981.24	73172.89	41789.8	65937.12	79136.58	56445.26	78372.02	78090.66	61134.87	84925.88
46260.66	67774.12	42699.02	70037.52	83345.85	47711.22	78237.82	92295.16	55148.53	84228.93
45378.07	68332.28	40213.29	64937.19	74690.4	49487.48	75160.97	77302.81	64735.81	85087.77
46424.21	67363.93	45720.24	67497.23	71079.43	46814.91	78786.27	76952.73	67122.99	80965.83

LP290.16_	LP290.160	LP290.159	LP290.160	LP290.159	LP290.16_	LP290.160	LP290.159	LP290.160	LP290.16_
72895.6	40232.56	86662.64	56421.66	74789.6	86092.23	75304.13	74982.41	66548.17	80925.22
68648.58	44501.61	88457.23	59056	69372.34	85406.33	69920.39	65684.11	64070.88	83052.07
76251.89	43170.79	85553.34	51287.43	71547.47	85186.63	78575.63	71276.12	64739.35	80530.07
73595.1	43394.69	91887.45	50930.44	69894.14	89177.67	71081.15	70017.76	66104.99	78564.67
72926.26	42147.7	91185.16	57543.65	72816.25	84519.36	76250.76	68641.72	69223.44	79568.87

LP290.159	LP290.159	LP290.160	LP290.159	LP290.159	LP290.160	LP290.160	LP290.16_	LP290.159	LP290.160
91796.54	105066	71347.44	87560.7	112656.8	72281.43	75366.32	50254.61	45079.48	102618.3
93056.77	113039.2	70767.81	91770.32	109405.1	78274.35	71265.3	50511.01	44738.11	102678
93611.45	98886.62	66113.99	92269.06	108392.5	77605.81	77374.54	50651.57	37537.46	104039.9
90304.21	100597	73966.45	87989.38	114661.3	76994.32	78378.03	50741.84	38956.19	99740.69
91357.87	116341.2	71791.01	85233.79	109729.7	76617.49	75914.77	50731.49	39564.44	97655.51

LP290.16_[illegible]	LP290.160[illegible]	LP290.16_[illegible]	LP290.16_[illegible]	LP290.159[illegible]	LP290.196[illegible]	LP290.269	LP290.269[illegible]	LP290.269[illegible]	LP290.269
44876.51	71038.93	51332.37	67838.15	76681.85	50066.54	81699.68	101142.5	77717.97	83620.22
42762.61	71312.09	52458.26	68195.23	74896.9	49661.65	78678.59	105279.5	73584.89	81446.87
45731.12	70897.83	53897.39	64419.79	73742.88	52736.73	102548.1	131702	78913.26	79210.45
44077.76	61787.33	53018.06	64004.2	69977.67	47587.73	90917.24	96541.2	100449.9	132257.3
48613.24	67323.7	53597	63811.56	74765.39	47618.12	70032.49	90054.06	67031.4	78636.95

LP290.268	LP290.269	LP290.269	LP290.269	LP290.269	LP290.269	LP290.268	LP290.269	LP290.269	LP290.269
94717.13	97910.28	80723.24	87912.48	74262.97	89810.6	68299.01	97407.58	96782.13	58252.08
86122.72	89172.3	67363.35	93523.21	80807.18	94824.89	58948.08	84595.13	96567.25	56772.3
124761.7	75442.58	100010.8	114690.3	106906.6	76293.23	75497.12	104637.6	102789.2	56531.03
127758.6	101270.5	82761.72	64159.99	85343.14	90352.5	68257.29	96510.24	92173.43	63066.96
77848.98	91854.1	72255.23	91283.97	75028.34	89704.83	52427.3	98294.87	92531.47	51436.61

LP290.268	LP290.269	LP290.269	LP290.269	LP290.269	LP290.269	LP290.269	LP290.268	LP290.269	LP290.269
58507.16	49820.68	44200.73	63126.22	32801.71	55047.95	68974.7	63352.63	40915.93	44793.76
60693.36	46166.99	49809.57	71404.46	33835.07	52304.08	62166.8	57378.65	47664.33	45427.06
66695.99	46556.72	54235.23	76128.91	40501.55	59126.36	54707.22	59660.86	46184.03	45437.79
58431.49	49327.29	42634.61	65135.19	31888.47	53486.25	61227.75	55571.02	38590.62	50250.2
61226.61	46539.54	45819.29	65805.03	32693.37	53803.05	66144.68	50884.79	43464.71	40971.03

LP290.269	LP290.269	LP290.269	LP290.268	LP290.268	LP290.269	LP290.269	LP290.268	LP290.269	LP290.269
39373.68	44444.08	56755.4	56589.49	37190.83	39984.61	59575.48	45131.73	17764	49741.48
37642.5	45502.1	56438.23	55962.02	36389.77	39792.66	59149.2	36834.84	18082.83	45179.53
36198.75	57035.01	59190.19	60274.26	43743.83	49606.12	66585.31	43508.5	23431.35	41056.25
37069.47	41002.77	53480.57	49797.78	35014.46	37941.81	51139.58	42341.11	16071.73	45442.28
32125.05	38091.63	53615.11	57989.5	36787.25	36059.59	55415.79	37864.59	15925.34	46409.02

LP290.269	LP290.268	LP290.269	LP290.269	LP290.269	LP290.269	LP290.269	LP290.269	LP290.269	LP290.269
34118.7	35536.8	32623.41	39910.78	65332.27	41631.95	46446.77	28221.64	50315.42	76365.83
37002.83	38375.78	33309.83	45886.23	65425.87	46326.53	55216.56	25864.13	52916.26	65248.39
43950.35	35640.18	26904.05	42186.01	56913.87	50486.7	64294.22	25839.81	54480.99	83268.46
31454.19	36161.07	26964.37	43408.34	68053.4	44729.45	48091.03	22755.2	44698.28	66742.76
36222.72	36190.9	30568.59	46771.83	67153.4	46432.85	46513.45	24815.4	48082.65	69068.92

LP290.269	LP290.269	LP290.269	LP290.269	LP290.269	LP290.269	LP290.269	LP290.269	LP290.269	LP290.268
42936.55	82263.72	20859.14	25975.2	61295.7	55689.35	27972.65	37381.24	82279.66	53371.88
44328.46	71688.78	21814.44	24789.82	58829.37	53589.27	30716.76	40533.03	81528.55	48857.18
42540.44	73918.98	20512.62	24028.04	65712.93	59693.21	25881.19	41450.84	103745.3	47520.63
39755.01	69882.7	22004.89	24046.73	56553.8	60200.98	24399.53	38235.01	78458.8	59188.19
37920.92	65506.06	21050.71	24194.56	54896.13	53223.5	28654.32	41663.88	85560.43	48686.43

LP290.269	LP290.269	LP290.269	LP290.269	LP290.269	LP290.269	LP290.269	LP290.269	LP290.269	LP290.269
18493.9	56494.83	51472.88	84203.5	21987.18	40580.71	17734.47	68676.58	47239.51	74799.6
18801.71	61914.6	52847.63	77794.56	22302.78	42448.64	16584.35	63526.44	47984.29	69693.61
23760.42	57867.15	67159.61	98029.06	23706.72	50776.31	18190.81	66870.06	64885.88	66837.49
19279.74	64239.05	53268.91	76434.32	19144.76	36647.74	17285.04	63627.12	44404.96	70741.83
19544.69	60891.83	48674.96	75245.83	16614.48	33206.97	15623.55	69183.47	45364.56	70607.1

LP290.269	LP290.269	LP290.269	LP290.269	LP290.269	LP290.269	LP290.269	LP290.268	LP290.269	LP290.268
39453.79	36641.57	70890.66	22547.53	26760.78	72099.76	52021.31	54004.27	48086.85	28379.71
40955.02	32022.4	70415.66	19484.42	27133.8	69981.77	56118.92	44408.52	45141.29	27470.76
47512.25	41897.29	65105.71	19527.08	33439.68	82178.27	60462.28	60622.24	44075.5	30485.39
38459.65	33982.74	59633.41	21231.58	25621.38	64485.14	52452.19	47965.65	43055.99	27397.79
33889.6	34492.12	61943.55	20279.72	27586.94	68396.72	51566.4	43643.28	43693.34	25503.09

LP290.269	LP290.269	LP290.269	LP290.268	LP290.269	LP290.269	LP290.269	LP290.269	LP290.269	LP290.269
43277.28	23625.91	46710.78	39244.45	32330.73	25368.1	85498.74	30850.96	61739.61	42874.52
49645.36	23352.65	43098.61	39602.58	35278.98	19583.66	82137	29750.42	69714.08	46983.67
49771.68	25321.92	49865.42	49792.09	40641.45	30220.32	106124.4	36001.85	83373.8	55395.64
49492.15	23739.43	42830.77	37059.36	32219.42	21595.6	85144.6	27423.35	64513.27	46696.04
44455.36	26656.86	39783.18	46242.17	30261.97	21275.29	85312.4	27957.2	55023.77	43130.77

LP291.195	LP291.195	LP291.231	LP291.253	LP292.175	LP292.175	LP292.175	LP292.175	LP292.261	LP292.284
29104.55	37083.93	34151.61	72420.87	67211.53	38647.92	31062.85	69339.14	9368.622	25329.67
26648.27	34125.9	32978.6	65561.05	63172.7	36022.04	30399.94	63390.92	11320.63	20529.72
24243.41	34282.74	33806.01	68046.09	72510.86	33431.18	30009.8	64173.8	13579.48	24611.1
26937.84	36433.37	34142.27	64351.42	70326.53	32381.46	34834.01	65458.7	5812.688	21866.07
29809.3	37332.02	36802.02	62854.03	72992.1	32325.57	31767.98	67412.97	12142.44	24052.4

LP292.284	LP293.174	LP293.174	LP293.174	LP293.174	LP293.174	LP293.174	LP293.174	LP293.174	LP293.174
13137.84	75733.74	78391.69	43870.33	74141.07	57648.93	51048.49	75772.27	50347.94	61652.43
12213.54	72415.06	77922.04	48358.7	68841.76	66343.2	49167.38	70046.17	54354.94	67689.09
15573.82	69228.26	76107.22	47609.15	69139.96	67161.42	54690.78	67711.88	47171.97	57705.25
11611.44	75668.48	67176.85	46741.02	93141.2	62676.66	51185.61	74060.69	50191.14	63227.77
10604.28	70493.41	74081.03	39468.74	61793.7	68362.31	54814.73	72104.23	51679.06	58374.77

LP293.174	LP293.174	LP293.174	LP293.174	LP293.174	LP293.174	LP293.174	LP293.174	LP293.174	LP293.174
36807.95	67382.42	44993.11	51457.5	56208.86	79977.32	47245.68	55926.82	58179.28	56525.62
41601.42	66615.45	43288.81	48331.63	54721.7	80328.2	40618.84	60911.43	62629.36	52463.03
44496.95	57488.67	44376.14	48117.52	54788.19	74256.11	40821.44	63289.67	57845.1	53464.67
46512.23	68609.39	45150.45	55429.93	62965.8	83260.68	47107.77	67066.04	52956.56	51660.5
39812.12	60708.13	47309.55	55453.44	55931.6	79316.75	38423.86	60169.11	61754.31	53001.15

LP293.174	LP293.210	LP293.210	LP293.210	LP293.21_	LP293.210	LP293.210	LP293.247	LP293.247	LP293.247
59158.97	64169.33	83284.1	74455.13	51103.45	59678.67	63982.16	89683.92	42483.27	52431.66
58789.13	68297.45	72494	63217.02	45365.93	58007.78	68391.22	87638.1	37366.72	56582.55
61726.78	57726.98	83416.63	83642.87	50207.85	57791.76	64052.55	70349.24	26879.31	38868.53
57146.53	61958.79	78999.5	76422.1	55833.26	53441.64	65074.93	46216.76	43931.32	52345.41
57952.31	67612.14	76951.79	77140.26	53235.78	51468.72	63927.89	92399.03	42517.44	56720.33

LP293.283	LP294.206	LP294.209	LP294.208	LP294.209	LP294.208	LP294.209	LP294.209	LP294.21_	LP294.209
40080.83	208973.3	90895.2	46891.58	130131.8	55005.88	69256.99	93029.33	88083.54	78185.09
38934.04	194438	91254.6	52508.7	122315.5	50990.64	59714.82	94340.04	95470.09	83721.16
40623.88	194141.2	117976.1	52415.61	120436.7	51590.01	65896.04	120446.8	94457.93	81038.49
29453.35	176891.1	89695.76	51532.13	123555.3	43736.28	62600.81	94170.13	99776.38	73186.81
35509.59	181564.2	91953.02	40858.05	114227.1	42489.51	62559.96	86566.53	101102.7	82706.15

LP294.208	LP294.208	LP294.209	LP294.210	LP294.209	LP294.208	LP294.209	LP294.209	LP295.151	LP295.151
53427.37	52906.44	95667.99	64866.61	36165.74	49936.28	37389.57	61177.29	64198.65	119342.1
54115.24	45342.83	86013.94	57376.54	34546.92	52246.42	35726	57225.09	60280.13	110203.4
55697.49	47266.22	86946.81	62403.41	35768.24	49523.45	37874.25	60199.94	65312.41	88606.53
52999.54	52352.53	96912.18	67423.97	38720.59	50472.76	35632.9	62274.65	81026.66	110327.1
37747.14	45957.67	93613.49	65698.92	45521.78	41765.5	36511.46	57607.52	76993.71	81661.87

LP295.151	LP295.151	LP295.152	LP295.152	LP295.151	LP295.152	LP295.151	LP295.152	LP295.151	LP295.152
98489.73	138890	88753.21	78419.55	67904.85	90268.64	91015.75	73039.5	87765.17	77354.27
100345.9	74324.41	61569.41	71099.4	83220.19	132711.2	109245.2	91942.08	84269.14	85373.65
105190.3	88972.02	72564.1	76630.43	111243	68420.4	86177.72	88906.59	92548.05	126096.1
101932.6	63628.78	67315.15	75509.21	69131.96	85034.01	80320.16	87180.01	103457.6	82056.13
69781.81	64966.39	70875.21	75130.54	96672.42	76159.68	56060.88	76700.72	64483.05	77677.04

LP295.152	LP295.151	LP295.151	LP295.152	LP295.152	LP295.152	LP295.152	LP295.151	LP295.152	LP295.151
63588.73	67378.16	78071.38	102708	88489.75	61976.63	88846.17	94984.82	85663.31	105343
110380.2	66078.3	105904.3	74741.2	94945.69	118593.9	90583.25	82467.38	93021.15	91760.06
106039.5	68432.57	85816.41	77545.3	90594.08	79824.5	87034.92	94399.19	87658.2	107155.4
106525.9	65693.67	83592.32	79840.89	79649.3	120668.4	91159.61	89819.94	90820.92	103558.1
96994.98	62163.9	77837.96	73506.82	113526.5	78092.63	85500.41	62399.69	93948.5	105779.4

LP295.152	LP295.151	LP295.152	LP295.151	LP295.151	LP295.152	LP295.152	LP295.152	LP295.152	LP295.152
83367.05	98005.51	87110.82	79791.8	85382.79	70029.54	67909.94	64558.12	81014.56	94589.25
59733.48	66474.39	112980	70017.9	79644.49	64649.47	77906.97	98423.04	67660.53	83262.5
81720.32	83183.26	101876	140304.8	75110.23	66053.51	85462.45	96507.02	70874.87	106086.5
83111.11	83982.93	95091.46	83915.53	130837.1	62847.05	80411.93	95590.6	75820.47	109363.4
78197.72	77027.13	98798.56	78824.35	62330.61	71982.08	77845.91	65999.18	65790.34	81412.46

LP295.152[illegible]	LP295.152	LP295.152[illegible]	LP295.152[illegible]	LP295.151[illegible]	LP295.152[illegible]	LP295.152[illegible]	LP295.152	LP295.152[illegible]	LP295.151[illegible]
76904.03	72783.09	82228.55	89717.68	95262.8	91606.4	68366.15	76036.84	86955.1	137963.3
74544.94	84122.51	79573.24	93651.2	104096.7	96274.27	89873.07	78447.71	80233.62	106861.9
74969.83	76262.34	87432.11	89248.32	115319.2	76507.84	86047.65	72606.68	77274.41	115226.2
76320.51	74178.75	88965.51	97766.76	122183.5	96999.91	77640.36	73858.8	77957.1	107752
75855.89	85155.45	76677.74	84257.31	74435.81	99925.16	70755.67	74512.25	76815.05	105597

LP295.152	LP295.152	LP295.152	LP295.152	LP295.152	LP295.152	LP295.152	LP295.151	LP295.151	LP295.152
92984.67	69759.81	100627.9	67498.29	56247.18	88030.67	109751.4	69787.33	54971.97	93472.4
89903.97	78494.23	99690.65	75030	93616.43	106148.8	101933.4	77262.79	52602.91	80793.79
89190.55	77885.8	96708.65	69635.21	97901.48	64101.27	110623.6	91236.45	66039.91	117776.2
85325.04	105186.8	92711.11	72861.77	95792.13	88556.26	107416	94988.36	61056.6	85370.8
127688.6	61142.33	97323.04	67792.64	76998.6	89445.19	90796.82	76435.13	57178.37	75546.21

LP295.151	LP295.152	LP295.152	LP295.152	LP295.152	LP295.151	LP295.152	LP295.152	LP295.152	LP295.152
67269.76	87340.15	63584.28	59795.44	116729	72290.94	89652.14	74649.48	75183.69	91635.57
66847.87	75129.06	82247.2	78980.27	68535.25	71347.59	83290.16	76327.65	88234.7	91381.26
78922.17	79161.16	84793.06	81077.96	68738.25	74641.01	89926.77	80070.73	88429.23	93118.96
76924.85	89215.5	88389.62	84731.05	60390.89	74109.1	81339.8	79599.68	89432.65	68135.91
74190.88	92305.93	84753.03	72978.28	86413.89	70811.67	91655.92	72727.49	82613.61	93351.86

LP295.151	LP295.152	LP295.151	LP295.152	LP295.151	LP295.152	LP295.151	LP295.151	LP295.152	LP295.151
66539.86	93971.91	55147.43	51530.54	61145.73	60785.03	80596.94	60085.72	59850.42	71080.52
77370.53	82014.96	58764.95	53205.82	75503.77	63999.47	75352.3	63102.65	60795.1	67364.78
91817.99	78477.48	79266.35	59655	74109.94	61308.15	90484.23	67389.63	78601.29	73878.94
126779.5	64841.98	65964.15	62708.35	74311.53	66566.66	87076.47	71783.35	72015.69	72506.98
69809.75	85042.8	52887.97	50311.03	62074.75	51848.32	57835.1	57017.72	64040.4	67405.85

LP295.152	LP295.152	LP295.152	LP295.152	LP295.152	LP295.190	LP295.190	LP295.190	LP295.190	LP295.190
62491.08	108505.4	80111.37	85803.9	136806.6	97158.65	115328.1	111946.3	73577.8	114658.5
89696.95	63929.85	91533.51	85324.62	144986.8	97509.5	111835.3	110445.7	63932.83	98715.04
108780.6	102669.2	90779.93	95177.68	138822.4	82785.24	123586.4	89281.04	78406.65	148398.3
60810.56	107877.8	69118.3	85071.23	139012	87506.71	121546	103563	65282.41	91303.26
60170.81	61500.15	77719.75	96814.18	144675	98587.02	124879.2	98155.52	71074.63	98619.54

LP295.190	LP295.190	LP295.191	LP295.189	LP295.19_	LP295.189	LP295.190	LP295.189	LP295.190	LP295.189
76430.91	110257	85436.21	102786	81760.69	59616.47	89819.82	71636.1	99624.65	90368.47
93246.5	114338.7	83364.9	102116.5	80539.09	58261.92	90374.08	74095.85	96425.27	78340.22
109213.9	140412	90919.6	107607.4	84813.18	65065.22	87609.72	70933.03	98292.51	100078.4
72001.41	105029.2	87030.79	96890.02	74971.05	56945.52	83531.14	65415.73	95636.86	94345.02
74815.98	128038.2	75961.22	105928	77625.73	69203.83	93197.16	66648.68	93780.38	83339.95

LP295.190	LP295.190	LP295.190	LP295.190	LP295.19_	LP295.19_	LP295.190	LP295.209	LP295.226	LP295.226
65213.76	104977.7	85896.95	82986	85622.25	60598.33	56809.88	35584.05	129587.4	96021.67
74342.68	109648.2	86378.23	84237.17	90543.08	70310.49	62337.68	33444.21	125411	114318.3
77185.93	105336.1	86332.01	87446.78	78343.75	71662.57	66999.9	37889.95	137708.4	108504.7
67181.04	108825.6	83621.74	83134.57	74690.95	59990.8	60395.25	34040.61	73415.11	100340.3
74014.73	101331.3	88648.21	88186.33	84255.38	65125.86	62914.54	40116.56	128456.4	101001.6

LP295.226	LP295.226	LP295.226	LP295.226	LP295.227	LP295.226	LP295.226	LP295.227	LP295.226	LP295.227
89111.78	75031.11	69228.32	81167.06	81221.08	89750.87	72105.79	86881.21	102227.4	109565.2
89084.34	80438.54	62219.59	82207.36	86919.54	82624.81	70230.31	79967.76	101315.5	106316
84389.74	78010.68	54720.04	72540.65	87596.21	74877.06	73911.21	77590.16	98382.42	65683.49
91715.18	74734.8	62332.05	73075.33	77560.25	83785.38	59591.81	91834.19	83350.36	98571.66
86072.35	78987.47	63916.86	67556.75	86925.79	81744.88	72210.12	87043.89	98494.02	101438.9

LP295.226	LP295.226	LP295.226	LP295.227	LP295.227	LP295.227	LP295.226	LP295.227	LP295.227	LP295.227
111328.8	81052.51	75132.38	95587.53	83307.27	96542.25	84783.2	96680.63	91158.65	90139.86
118715	82255.91	74652.84	93867.14	86107.47	93852.3	86399.92	98725.72	77963.71	78268.11
119736.8	66802.2	73638.58	89130.45	45362.83	89115.63	84599.9	96651.88	80574.45	87558.53
113774.3	79828.29	72351.57	93492.18	90530.63	96360.96	86764.21	99053.64	81495.66	86786.44
108967.9	77144.35	74665.28	92940.73	83270.58	103963.1	80286.65	93797.36	81834.37	82201.05

LP295.227	LP295.226	LP295.226	LP295.227	LP295.227	LP295.227	LP295.227	LP295.227	LP295.227	LP295.227
100429.4	76464.4	78385.08	82732.98	84960.76	103771.7	87811.47	119693.6	97148.5	65681.69
94284.37	72777.41	87282.22	83076.63	78713.66	104737.6	90364.57	117867.6	89502.3	67052.45
85883.87	75551.68	85612.61	79708.51	77761.19	102977.4	92507.58	119895	98887.88	67229.32
95396.38	71743.51	83682.31	81605.44	84953.94	109962.3	86956.99	116395	96398.91	63544.92
95154.58	67659.83	80656.26	77478.57	81199.1	106857.6	85552.94	131869.6	93064.27	59256.66

LP295.227	LP295.227	LP295.227	LP295.227	LP295.227	LP295.227	LP295.227	LP295.227	LP295.227	LP295.227
75378.22	110105.1	85519.94	84763.3	64722.47	114272	75398.78	70626.59	128177.6	79727.12
67664.51	112288.1	85409.27	92913.63	57037.21	112749.8	77049.49	70143.77	131140.5	79954.91
66828.98	117884.4	80496.38	90760.18	55940.17	116033.3	84842.23	69290.02	130616.7	85360.5
66409.35	107351.9	82015.42	83305.7	59466.34	105623.1	66076	69937.37	122046.3	83470.52
67372.14	105546.1	86638.76	79892.19	61162.42	102628.9	66897.83	69727.54	135232.2	71528.11

LP295.226	LP295.227	LP295.226	LP295.226	LP295.299	LP295.956	LP295.956	LP295.956	LP295.956	LP295.956
88318.09	106627.9	80191.09	69878.5	44475.42	121719.4	67638.04	67248.88	72443.39	89979
88215.38	102227.1	64752.87	70441.47	56946.97	101363.9	63187.59	78307.18	78354.24	99338.59
95166.01	101476.7	83071.78	74803.97	55122.09	136646.2	74308.2	65202.13	76415.41	122081.5
92490.57	100214.4	88456.62	69352.94	41037.95	135859.9	51910.1	75666.13	59147.45	86791.41
83851.98	96760.01	70657.67	68795.88	51094.44	125717.8	54688.39	72901.73	72238.88	96479.17

LP295.956	LP295.956	LP295.956	LP295.956	LP295.957	LP295.956	LP295.956	LP295.956	LP295.956	LP295.956
67886.52	97823.46	38021.43	50128.14	89336.59	109063.7	103135.2	87767.87	117682.1	88274.83
58154.06	100177.2	44175.99	54583.18	116127.7	98915.14	101366.9	94390.8	99523.56	91504.27
113502	97155.77	34985.26	65001.7	116741.6	148781.1	126964.2	106415.8	101050.4	96023.48
79123.89	106868	48281.65	55116.12	112253.5	109467.7	92659.58	133969.9	126149.1	139647.6
71182.95	114956.4	31139.23	42164.49	85599.01	106056.7	117771.1	77336.53	107445.3	86771.26

LP295.9569	LP295.9568	LP295.9564	LP295.9560	LP295.9569	LP295.9567	LP295.9568	LP295.9564	LP295.9568	LP295.9568
98683.35	59448.66	96828.89	106868.6	103659.9	42946.03	65696.14	94419.65	74150.98	104103.8
90883.35	64699.3	123686.1	92282.23	103315	44340.04	67510.73	130987.1	67392.26	104762
98323.16	77783.51	167918.3	168428.4	97192.98	40143.26	122191.3	88226.24	81018.52	121154.5
111874.9	46944.75	87776.52	103356.3	136045	44481.43	81483.94	113963.6	84062.58	101605.4
116592.9	47196.51	86584.69	98763.58	100547.6	46630.64	71521.44	103744.3	74497.63	136647.9

LP295.956[illegible]	LP295.956[illegible]	LP295.956[illegible]	LP295.956[illegible]	LP295.956[illegible]	LP295.956[illegible]	LP295.956[illegible]	LP295.956[illegible]	LP295.956[illegible]	LP295.956[illegible]
128832.7	93367.44	111296.1	100181.5	110736.3	105858	100762.6	82724.87	89482.36	119266.2
111129.5	94865.41	103044.4	109635.8	99627.4	97442.04	92944.36	79063.61	90937.85	101811.1
88978.09	116344.9	88420.16	103676.8	86540.58	103928.2	84968.66	77564.76	127137.7	133294.7
116377.2	111726.1	107043.6	93664.89	117252	125287	91016.64	83751.47	94989.56	118532.9
123353.7	86157.71	119402.8	103096.2	146556.7	148525.4	121689	96881.97	90415.73	125745.5

LP295.956	LP295.956	LP295.956	LP295.956	LP295.957	LP295.956	LP295.956	LP295.956	LP295.956	LP295.956
93739.96	100518.8	89777.47	106873.4	114466.3	77700.78	98400.17	68893.85	89600.09	122578.8
95886.03	96168.91	92742.98	99967.47	121051	99083.21	94253.61	72561.12	92095.88	111647.4
103874.2	104945.6	100751.4	127596.6	126854.5	91942.05	96349.11	61518.27	95821.84	133389.5
91938.3	108373.5	90074.03	104544.6	165080	71049.53	105110.2	78166.16	91686.76	124911.1
86748.1	103346.9	80171.92	108395.4	127930.2	79698.64	106721.9	63280.08	130958.8	124913.3

LP295.956	LP295.956	LP295.956	LP295.956	LP295.956	LP295.956	LP295.956	LP295.956	LP295.956	LP295.956
100440.5	115814.6	84829.48	112257	73942.44	70476.52	105506.3	42208.47	102887.4	74226.54
109832.2	120631.2	76160.97	102079.7	71999.4	71783.38	106527.6	45931.06	109002.6	71089.55
106648.3	123938.5	80292.28	114921.7	71833.22	63775.44	113440.3	44582.66	106514.2	85404.17
111163.1	127940.7	80432.84	136661	76719.52	69041.8	118798.6	45222.97	105686.4	136192
102881.3	137196.3	79414.72	100200.4	70100.09	72302.19	117313.5	48343.7	107515.5	76720.79

LP295.956	LP295.956	LP295.956	LP295.956	LP295.956	LP295.957	LP295.958	LP295.957	LP296.194	LP296.294
81565.78	62250.71	95479.69	132739.7	92580.27	111611.8	73834.43	92036.94	17808.24	18080.11
83202.97	62239.4	92978.16	137620.1	99344.42	103443.2	76393.57	92413.73	14477.69	16883.06
87006.31	68395.58	95242.83	135236.1	100040.5	81129.78	77236.82	89909.29	13373.85	23316.19
90054.07	64036.56	98767.36	134181.9	102140.3	115211.5	75697.24	97096.86	11226.46	22371.1
81499.54	63760.45	92239.55	161747.6	97396.96	107612.8	83374.65	92321.4	13913.96	21451.35

LP296.294	LP296.294	LP297.082	LP297.082	LP297.082	LP297.130	LP297.131	LP297.131	LP297.131	LP297.131
13404.76	20408.88	47245.06	87107.36	51164.92	69667.21	86577.34	93971.22	79334.27	92707.37
11960.39	21346.48	49966.19	85217.34	48556.82	104242.7	72494.61	63250.79	61830.26	88607.63
13563.68	20961.59	54621.61	89255.02	54215.9	71052.12	104011.7	86495.82	79887.22	86765.07
13678.43	15541.08	56067.62	102686	55109.42	75834.62	61446.63	89905.46	54766.59	78165.11
15944.22	19369.05	53160.18	106353.1	53288.72	63790.59	65814.11	103910.4	58425.22	88762.15

LP297.131	LP297.131	LP297.131	LP297.131	LP297.131	LP297.131	LP297.131	LP297.131	LP297.131	LP297.131
77467.83	88714.56	82564	98562.28	92147.17	81345	87802.85	77398.05	89491.18	70794.21
71756.47	88845.34	80667.55	85032.26	92669.89	65467.31	76830.34	87501.16	73028.27	79297.04
72966.55	113499.2	87931.64	108778.1	72345.87	120768.3	85076.38	74396.35	102790.5	62093.49
64844.54	105221.8	80760.46	104252.2	148118.4	87804.44	83506.21	58948.12	82100.57	70851.76
71337.54	97416.29	75392.23	88344.75	97666.99	80239.57	66988.44	77806.59	89145.23	85225.13

LP297.131	LP297.131	LP297.131	LP297.131	LP297.131	LP297.131	LP297.131	LP297.131	LP297.131	LP297.131
62956.32	75794.57	48861.58	71328.84	93957.11	106067.9	81894.32	81896.28	54714.58	77471.44
57083.86	61110.65	63995.1	91707.64	64454.28	85243.06	99244.89	95564.34	72747.22	69778.54
124117.1	76013.65	72284.76	79749.83	83947.93	97649.86	110232.2	62120.17	50330.16	84849.4
86272.49	59917.78	81422.44	55282.07	124030.7	123099.2	73489	73761.12	82931.77	75644.34
95782.8	64800.57	54038.15	81758.13	65664.65	72480.36	101843.1	103939.6	89441.17	79201.94

LP297.131	LP297.131	LP297.131	LP297.131	LP297.131	LP297.131	LP297.131	LP297.131	LP297.131	LP297.131
106333.7	89301.15	77790.15	78414.79	73761.06	106367.9	71892.37	73379.22	57396.67	120932.9
69486.93	85246.37	77819.05	84037.3	63098.12	79630.81	76698.7	77772.47	56119.58	80837.19
110842.7	98230.35	78505.14	88686.67	75376.13	65276.43	86088.47	72804.66	148873.5	111646.7
79759.34	103929.7	90209.04	104889.7	82452.34	65371.99	86767.36	62790.85	94292.67	94556.58
78700.41	77681.38	79295.54	68057.38	74364.61	87734.54	81401.68	69424.63	65708.34	79620.83

LP297.131	LP297.131	LP297.131	LP297.131	LP297.131	LP297.131	LP297.131	LP297.131	LP297.131	LP297.131
116214.1	63957.4	107548.3	106917.1	80045.74	100959.3	124508.5	86514.2	100770.3	82705.76
75343	60233.15	69622.89	81592.11	107824.8	84839.62	81913.87	95165.54	122927.8	76378.17
104380.3	59385.24	95829.86	91582.65	102772.1	88014.26	73526.02	75633.93	92850.5	85680.62
82988.73	74380.97	65919.7	99793.62	76089.46	70801.14	78128.45	120875.2	75367.12	64604.14
79946.38	82440.88	67493.75	80394.84	81869.87	90632.25	75172.22	126140.4	93102.4	118507.6

LP297.131	LP297.131	LP297.131	LP297.131	LP297.131	LP297.131	LP297.131	LP297.131	LP297.131	LP297.131
74073.91	103246.3	90200.77	75799.51	80882.81	55080.91	84733.25	41307.93	64073.58	64238.59
115664.4	88675.29	97512.14	62218.09	79008.18	49049.4	63454.94	45150.97	98700.92	74211.85
99141.59	89218.51	84269.59	78426.85	70576.65	49188.92	85068.54	47612.65	102214.5	66597.16
80662.31	115314.2	77956.26	73361.2	65224.7	91462.3	91620.43	39895.42	98107.72	58838.58
108052.8	89183.13	86821.89	61150.48	97089.62	56413.96	82426.02	51922.89	111517.3	65211.3

LP297.131	LP297.131	LP297.131	LP297.131	LP297.131	LP297.131	LP297.131	LP297.131	LP297.131	LP297.131
80587.82	77088.45	75830.43	115866.4	80636.11	75284.34	42763.36	44844.19	58450.61	63061.19
68900.36	72080.68	103710.9	93001.56	80849.47	72576.27	49319.03	53042.89	57682.06	102493.7
78784.27	89033.95	80894.36	94319.37	61731.31	81108.06	53244.18	59341.36	63406.23	52137.7
59308.52	86390.14	72917	87127.72	67157.33	82988.11	45860.78	42658.02	52382.55	70166.77
76878.62	65907.07	92156.86	75997.58	61458.23	76109.54	37130.85	76215.09	62329.38	58587.22

LP297.131	LP297.131	LP297.131	LP297.131	LP297.131	LP297.131	LP297.131	LP297.131	LP297.131	LP297.131
76213.96	86958.03	63145.36	40192.64	56244.5	85012.44	33668.49	37130.86	31090.1	34551.07
88876.31	75325.02	62107.98	56674.46	44298.66	83331.08	37453.84	41120.25	27933.48	35384.61
81097.81	87423.03	83099.57	33167.09	38313.99	84634	39758.12	36241.95	31915.97	33248.84
82720.96	70491.97	64319.2	38361.8	43473.45	83301.16	36528.3	26461.48	36960.17	30609.16
101645.5	101071.1	72915.26	45224.95	34926.6	63427.87	24620.45	28737.89	23955.52	35594.2

LP297.131	LP297.131	LP297.131	LP297.131	LP297.131	LP297.168	LP297.169	LP297.168	LP297.169	LP297.169
38701.76	57342.84	34913.92	38442.02	58532	66281.3	80261.94	66437.53	64242.58	87212.83
41022.22	39378.6	33406.49	47561.43	58648.16	68919.74	92238.43	63038.34	111631.4	81701.09
29402.74	37584.94	43022.06	38902.8	66246.06	67500.91	81631.48	70682.83	116681.1	85917.32
38207.29	51297.49	53534.58	36749.82	68219.03	65555.75	86545.38	71013.55	114613.9	85130.12
33306.71	36487.34	27847.29	32247.56	67805.86	80209.4	85970.4	66292.66	121542.1	87068.06

LP297.169[illegible]	LP297.169	LP297.169	LP297.169[illegible]	LP297.168	LP297.169[illegible]	LP297.168[illegible]	LP297.168[illegible]	LP297.168[illegible]	LP297.168[illegible]
85914.4	82541.58	68678.63	83178.58	63558.99	73356.32	85859.28	73267.25	56788.02	54783.71
82185.27	69522.15	69828.01	74786.35	65789.98	98946.66	91059.03	71165.97	58185.28	53045.53
76217.43	76441.95	68099.62	80369.91	61742.17	106576.7	93928.69	69554.4	55376.38	58457.07
79957.08	81598.42	76199.43	79654.47	66415.52	103125.7	86842.76	75533.24	59915.54	49867.41
76112.27	82592.04	76742.53	79742.98	67672.73	113826.5	94573.24	73212.79	54448.14	56763.29

LP297.169	LP297.168	LP297.168	LP297.205	LP297.206	LP297.205	LP297.206	LP297.206	LP297.205	LP297.206
98791.3	46536.27	73842.38	80972.12	73935.86	77545.38	87524.07	98284.54	119122.9	79901.8
95716.31	43430.34	63165.1	68333.84	76391.84	86205.45	85440.82	97728.34	110489.8	84308.03
95992.08	47745.02	71349.47	75216.77	67955.81	82063.95	81237.47	101169.9	120169.3	81918.12
103162.2	38875.37	75303.26	76242.91	65634.74	81520.47	86256.13	96485.05	124664.1	84557
99789.71	42061.1	75618.02	84485.47	70293.03	92966.24	84829.65	98934.32	125288.7	81850.63

LP297.206	LP297.206	LP297.206	LP297.205	LP297.206	LP297.206	LP297.206	LP297.206	LP297.206	LP297.206
68228.05	64261.33	57775.51	133044.9	87700.83	73979.88	57632	86503.65	90118.86	71494.84
68123.78	62517.55	62475.06	135342.1	89193.58	76818.49	51235.93	80314.44	82056.75	71508.67
62719.95	57391.44	54194.5	143813.6	88088.03	70015.08	60763.23	78091.21	84205.1	72539.21
64526.47	61732.18	54769.86	148097.1	85083.13	80031.86	61323.93	81586.01	81869.77	73167.42
68911.61	65414.96	56041.22	140552.9	86680.69	82866.07	60792.35	89974.59	91841	71399.54

LP297.206	LP297.206	LP297.206	LP297.205	LP297.206	LP297.206	LP297.206	LP297.205	LP297.205	LP297.241
89111.02	71647.69	90646.26	103772.1	75178.94	86872.64	112768	63571.29	72195.96	123775.1
93343.92	71297.15	87500.74	96293.69	78580.44	91129.58	94319.21	65060.17	73320.22	131771.9
91839.88	65571.64	84793.05	100170.7	77993.82	90439.15	100817.6	66780.7	75980.62	137825.3
84136.7	64578.94	86391.13	109762.9	74874.02	90925.89	103579.3	69482.97	80571.32	136347
90272.85	67648.35	90115.02	103605	76905.67	95459.58	97628.77	57261.78	80501.95	147218.1

LP297.242	LP297.242	LP297.242	LP297.242	LP297.242	LP297.242	LP297.242	LP297.242	LP297.242	LP297.242
91339.44	87856.13	72571.2	109576.7	80825.26	103224.5	115825.9	63475.14	126067.1	90131.32
104207.2	104673.1	78262.02	119837.3	84012.94	102424.3	107480.7	68103.41	103985.7	94228.28
100046.4	96131.2	69649.33	110507.9	77225.02	107985.1	115552.9	62150.17	133942.2	90357.1
100550.8	94825.38	71354.18	112441.8	82060.5	107961.7	107964.8	62769.86	137474.6	92868.98
107633.4	99365.05	87058.59	113397.8	81004.41	114576.7	118826.7	63321.03	129991.3	92095.16

LP297.242	LP297.242	LP297.242	LP297.244	LP297.279	LP297.278	LP297.278	LP297.278	LP297.278	LP297.278
100670.4	82832.32	73840.23	66363.14	140079.7	79250.47	68094.09	111069.8	59195.58	47620.58
96603.56	89002.83	75338.4	67749.21	135470.3	89281.16	67604.68	109984.5	48214.27	47178.52
98003.68	78762.57	73925.2	62957.65	105610.5	87381.45	94983.69	106213.4	63016.11	48934.4
92601.57	85455.92	75647.17	68198.49	119008.5	80312.14	70150.17	111028.5	57720.8	46347.6
98880.63	81257.79	80837.19	65333.45	119463.4	84341.24	68146.88	114226.4	46042.83	44838.69

LP297.278	LP297.278	LP297.278	LP297.279	LP297.278	LP297.279	LP297.278	LP297.279	LP297.278	LP297.279
48984.48	70417.45	99614.28	75256.04	38902.11	81562.36	84952.1	57471.38	51725.99	75749.17
49537.59	69399.46	94751.99	76826.19	38593.03	82522.05	79026.4	56856.84	56694.28	79441.06
66103.12	58774.83	93782.71	108836.3	35893.4	88144.4	81215.2	60852.3	58780.66	75210.19
49180.69	86319.46	109029.8	73302.99	42255.33	78267.49	78602.8	62032.15	54784.14	76612.02
50436.98	66011.71	105274.9	78673.49	37065.67	90765.95	91463.84	55562.02	56040.86	77458.43

LP297.278	LP297.278	LP297.278	LP297.278	LP297.279	LP297.279	LP297.278	LP297.315	LP298.310	LP298.310
121297.3	76199.64	60560.7	76516.92	67584.05	82319.96	65104.45	54968.73	105048.8	607488.2
85894.69	61714.93	73388.66	74540.88	72268.31	71819.21	62102	20276.33	107850.7	696928.1
88041.48	64814.66	70801.04	85486.6	63110.69	73577.28	65537.88	26587.9	138221	1072605
84783.89	62173.65	69749.91	73947.02	61154.94	69975.03	61984.46	26007.38	157675.3	1329472
96616.73	63440.51	70592.16	75851.79	65093.54	75980.01	60105.26	57057.73	170873.6	1457434

LP298.346	LP298.877	LP299.061	LP299.062_	LP299.061	LP299.079	LP299.147	LP299.147	LP299.147	LP299.147
76500.79	24551.43	32040.18	36167.07	40756.47	30303.21	53226.86	80911.99	66778.79	79770.96
78513.97	18761.87	34311.76	37429.53	40460.79	32474.88	92645.64	85549.13	71138.89	80645.97
97167.26	22246.53	33061.41	34909.09	37064.18	26829.01	64726.98	75896.2	75139.39	76183.13
103436.6	23318.09	30032.36	36395.33	37797.96	24877.36	64483.98	65604.19	67037.31	73393.61
109491	18809.71	35023.86	34878.19	36391.31	25489.31	58413.09	46572.6	63479.56	74600.63

LP299.147[illegible]	LP299.147[illegible]	LP299.147[illegible]	LP299.147[illegible]	LP299.147[illegible]	LP299.147[illegible]	LP299.147[illegible]	LP299.147[illegible]	LP299.147[illegible]	LP299.147[illegible]
56250.49	62938.7	51656.5	70621.4	63454.61	49574	86949.79	54823.24	97582.63	78332.13
63531.65	63410.21	51657.03	85715.46	62354.68	86263.59	67821.73	57156.94	81224.57	70527.84
90129.8	51864.73	68343.31	97402.97	66597.31	82082.65	125112.5	65819.71	82006.99	66378.92
61631.41	53403	51534.14	80623.66	58555.63	93081.92	65978.98	54328.13	79931.56	62283.19
65289.45	68074.6	61198.41	82675.39	63232.9	79156.55	78377.12	55692.03	86745.07	68105.07

LP299.147	LP299.147	LP299.147	LP299.147	LP299.147	LP299.147	LP299.147	LP299.147	LP299.147	LP299.147
83790.12	94438.68	73206.15	74206.33	98213.68	76493.33	63168.53	154674.1	89569.96	76966.61
88386.6	85417.29	82761.04	63607.86	84886.88	70351.79	50610.78	58460.54	94526.26	66054.52
91909.57	79440.02	78083.52	77657.63	106434.1	67859.39	59414.09	61756.49	119495.1	53976.56
61720.91	81422.16	77942.91	59731.02	79363.48	77482.77	69229.77	61635.64	87619.23	68514.96
63691.46	87442.56	80921.97	63732.18	77028.93	70466.66	64603.84	65760.43	86762.91	68413.92

LP299.147	LP299.147	LP299.147	LP299.147	LP299.147	LP299.147	LP299.147	LP299.147	LP299.147	LP299.147
51201.36	106067.1	72457.79	55963.81	74518.9	71545.16	76440.08	66353.47	60336.36	107753.2
51609.66	62381.31	73264.97	80812.05	58942.95	49530.01	79189.23	64015.92	64427.78	72123.58
57459.75	68569.44	66639.72	75249.66	60907.7	59166.45	73543.62	66980.99	87738.62	73632.6
51067.96	65922.49	74322.68	69475.14	59285.07	58853.24	72690.06	65548.48	59598.27	70049.57
52360.41	70282.62	62833.4	69227.51	57482.92	54338.89	76541.62	65245.09	62690.59	75698.9

LP299.147	LP299.147	LP299.147	LP299.147	LP299.147	LP299.147	LP299.147	LP299.147	LP299.147	LP299.147
137724.7	67098.99	93341.5	82243.04	55244.44	69800.48	73693.58	80929.21	59283.61	50894.05
81136.37	62347.87	48125.23	74317.4	59796.31	58665.96	75048.6	83220.75	58480.53	56491.97
76192.06	62670.63	53236.97	76854.77	57831.31	64229.66	79529.9	79884.59	57090.45	58783.39
81822.42	58553.71	59311.29	76186.24	59113.93	61560.8	72328.58	84810.43	57813.15	61745.51
84165.5	60068.04	50909.12	73368.01	57932.13	64609.78	71061.17	80116.82	56053.14	59034.29

LP299.147	LP299.147	LP299.147	LP299.147	LP299.147	LP299.147	LP299.147	LP299.147	LP299.147	LP299.147
62642.62	48191.53	30488.92	40188.02	32519.73	40700.88	27288.22	38661.76	123753.2	73616.03
68991.07	50473.48	32887.45	39669.16	40895.15	39803.21	25506.55	38184.22	67494.84	67857.26
65422.31	45557.88	30711.39	34595.94	32532.66	35516.47	24855.43	37035.87	79061.73	70973.17
57456.03	46678.48	32927.37	34444.97	33932.32	36152.07	25169.06	37443.59	76201.89	82160.46
61325.97	48980.08	33349.71	36973.35	33114.43	37472.96	24725.16	39045.55	75379.17	70707.6

LP299.147	LP299.147	LP299.147	LP299.147	LP299.147	LP299.147	LP299.147	LP299.147	LP299.147	LP299.148
65053.08	68451.76	66287.23	66624.1	65807.3	48911.78	61965.02	51283.83	64441.56	21941.81
68055.03	82670.93	55501.41	65192.49	80825.38	64904.84	91756.3	68716.97	63611.77	21052.61
87929.82	103734.7	71464.33	60830.54	81810.4	71675.17	90126.67	68523.83	60798.37	18999.34
62771.78	101601.2	62501.48	62311.63	78783.79	70806.63	94339.54	70470.36	64606.49	14743.37
70095.29	84540.1	60362.6	66513.49	81710.92	67185.5	93481.04	68842.16	65176.47	20547.81

LP299.148	LP299.147	LP299.162	LP299.185	LP299.185	LP299.185	LP299.184	LP299.185	LP299.185	LP299.185
36577.93	59569.74	22833.03	31146.52	35119.33	39374.15	33854.05	39678.26	33427.48	30313.1
40853.16	62194.64	22880.31	29940.1	36167.96	38070.39	36841.83	36865.25	32603.05	31223.71
38913.99	61016.04	19160.81	35784.54	32280.1	36444.79	35121.42	41252.79	35768.73	27969.16
39154.79	56775.31	17576.72	34753.95	31586.13	34324.56	32997.5	40511.92	35689.02	30194.62
37772.67	60200.9	17739.92	34899.76	34939.98	37461.84	34857.49	37880.96	32226.53	27649.2

LP299.185	LP299.185	LP299.185	LP299.184	LP299.184	LP299.185	LP299.185	LP299.184	LP299.184	LP299.185
36999.17	43604.47	38015.01	37234.75	29824.63	37511.72	47032.08	40043.57	32732.17	30236.24
35740.88	33621.8	34830.94	39534.26	28443.68	42202.5	54924.78	43450.28	37019.27	29034.2
36532.94	40208.95	38216.18	36784.11	34133.33	37121.63	51275.09	38730.15	36912.74	27307.3
35937.73	40410.81	36333.16	36323.11	31003.84	38619.25	54802.58	44431.42	33219.5	27094.72
40576.21	35563.94	33355.08	36579.43	31452.02	40137.09	48862.14	43880.86	37979.47	31195.34

LP299.184[illegible]	LP299.185[illegible]	LP299.185[illegible]	LP299.185	LP299.185[illegible]	LP299.184[illegible]	LP299.185	LP299.185[illegible]	LP299.185	LP299.185
45213.04	28706.47	55230.94	28895.15	50744.34	26964.93	29405.13	44771.82	33769.79	30229.57
43342.54	26381.7	49376.91	26667.8	50731.95	25581.5	27720.12	46468.31	33992.92	31089.24
44606.78	25359.74	53942.44	30193.73	51639.95	26687.54	29408.6	46665.6	35198.57	31927.73
46287.25	25815.27	51117.21	29393.95	52946.66	25586.53	24252.64	43043.29	34332.45	31670.52
43372.14	28693.07	54641.72	27767.75	57213.99	24949.05	31649.39	38773.27	30441.05	28627.48

LP299.185	LP299.185_	LP299.257	LP299.257	LP299.258	LP299.258	LP299.258	LP299.258	LP299.258	LP299.258
41634.57	51599.55	65301.94	41849.04	63336.53	79565.7	41891.16	39452.27	54776.46	33523.42
46451.94	51254.01	66203.58	44365.35	59920.53	76436.71	39530.01	40523.69	49061.1	38341.82
43243.69	46134.02	65605.55	44617.35	61547.15	83319.38	35389.23	40497.64	55339.56	36110.98
42868.88	49736.68	61687.99	42240.04	65547.37	81126.83	39074.19	43603.08	60369.81	35004.36
44242.63	44978.76	67534.84	40806.6	61025.22	88055.4	37681.94	41883.97	55600.42	35715.96

LP299.258	LP299.258	LP299.258	LP299.258	LP299.258	LP299.258	LP299.258	LP299.258	LP299.258	LP299.258
31072.86	30983.97	37007.37	49131.82	49286.93	62945.61	37635.9	48004.32	46344.83	25811.1
33900.18	26606.83	34070.62	49491.04	49369.02	69818.43	35153.88	48208.11	46993.89	25276.65
33316.32	27147.63	36127.84	51030.68	43324.18	66570.15	43470.15	47639.06	47669.26	26797.72
30686.39	28506.08	39415.28	47902.89	50096.98	65369.91	40424.2	44230.91	48513.68	27283.66
34278.69	26244.22	43122.55	44810.55	49284.42	78238.15	41792.43	51424.83	46628.26	28187.73

LP299.258	LP299.258	LP299.258	LP299.258	LP299.258	LP299.258	LP299.258	LP299.257	LP299.258	LP299.258
38900.25	69542.75	61657	28818.38	34092.31	40910.85	43269.19	35322.73	56211.18	61322.39
35137.59	71587.63	59975.24	30922.64	29437.26	46845.61	47411.56	35800.64	59335.16	65945.48
37009.43	71441.92	63160.28	26112.23	28968.79	43627.31	40841.94	33349.29	56170.13	63546.86
38619.71	70441.7	61287.08	29057.06	30618.31	41626.04	46050.5	32902.26	56166.44	60856.72
34657.54	72380.6	63225.92	30837.5	31458.02	47343.88	43430.94	34600.53	58912.55	67195.26

LP299.258	LP299.258	LP299.258	LP299.258	LP299.258	LP299.257	LP299.258	LP299.258	LP299.258	LP299.258
44429.46	49971.35	38616.8	34329.11	32750.55	47193.6	45626.67	31555.01	48331	42033.99
48673.94	55210.21	37496.69	36619.6	32429.22	32909.82	48888.6	32198.61	46876.47	40957.1
50553.55	54059.47	38674.67	34614.72	33215.73	42480.54	42082.59	28778.58	51184.91	41255.79
41700.22	50014.46	35771.84	33965.02	35724.89	45004.2	47441.12	29228.74	58940.48	40571.69
45955.33	53059.58	38169.93	40996.55	39055.84	39888.01	47882.6	27052.71	53860.52	43194.31

LP299.258	LP299.258	LP299.294	LP299.350	LP300.144	LP300.144	LP300.144	LP300.144	LP300.144	LP300.144
46391.91	35177.54	35548.26	68726.57	176164.8	137793.1	199894.7	179020.2	370064.3	312680.9
51244.97	38189.93	31909.95	65410.37	233421.2	204427.6	181622.9	188602.3	469521.5	401044.6
50712.37	38578.54	30351.02	67945.39	204805.3	154745.7	253058.5	243318.8	478571	336113.9
48596.14	42414.63	31480.78	59864.93	161826.7	110517.9	201047.4	173797.9	333243.6	370339.4
49419.51	38161.64	27421.13	58807.15	177952.3	123427.4	203036.4	174653.6	318337.9	348504.5

LP300.144[illegible]	LP300.144[illegible]	LP300.144[illegible]	LP300.144[illegible]	LP300.144[illegible]	LP300.144[illegible]	LP300.144[illegible]	LP300.144[illegible]	LP300.144[illegible]	LP300.144[illegible]
205476.7	347869.3	220822.7	290836.6	312961.9	296875	233934.4	319477.2	378190.1	209322.6
241131.1	401975	181943	296941.7	332107.5	382398.3	252812.1	327055.4	285035.8	230925.2
225218.6	414864.9	235955.1	287337.5	363293.2	282236.7	245008.1	351403.3	392535.9	156648.7
206526.7	546193.7	231493.1	431396.2	290774.5	244420.6	251957.3	326350	383917.1	228567.2
187701.7	295048.3	223430.3	340513.5	295243	297214.5	248541	343579.8	395539.6	216736.3

LP300.144	LP300.144	LP300.144	LP300.144	LP300.144	LP300.144	LP300.144	LP300.144	LP300.144	LP300.144
228124.5	239583.1	342150.3	158455.1	212922.8	345516.3	238978.3	294212.2	185083.3	257527.1
255631.5	292164.8	329039.1	209985.5	204962.3	304325.5	262245.5	349610.1	187407.9	241291.7
237035.5	224503.1	274049.6	157921.8	193516.7	322469.1	230372.8	222067.7	193212.4	256518.3
230385.9	224253.5	309122.4	160458.3	221253.2	314764.3	234416.3	392712	201298.3	307513
225551.4	239540.6	303701.4	150350.6	214889.2	331958.3	235470.8	304384.1	195053	265708.6

LP300.144	LP300.144	LP300.144	LP300.144	LP300.144	LP300.144	LP300.144	LP300.144	LP300.144	LP300.144
219184.2	125722.1	284619.4	290488	243258	246918.1	153624.1	240395.3	190152.9	216416
322229.4	121547	373745.6	335084.6	258760.6	227150.8	163151.9	262255.9	245300.6	305338.2
264636	123421.7	276533.4	242323.4	228259.8	228143.1	152798.4	249204.8	172535.3	236241.8
244381.6	112229.7	282262.6	292442.4	228774.7	225761.8	146195	247399.4	186267.8	237359.9
250255	119842.2	312187.9	311015.9	242396.3	236018.7	151858	244365.3	181557	236245

LP300.144	LP300.144	LP300.144	LP300.144	LP300.145	LP300.144	LP300.144	LP300.144	LP300.144	LP300.144
295037.3	271779.8	105099.3	181048.8	231043.9	192565.8	237011.1	149528.8	157276.1	116700.3
308011.7	244994.4	105182.1	174126	231709	209164.3	186974.8	127665.7	167304.8	140402.3
302972.2	269552.9	113743.2	172835.8	231864.7	221015.3	228978.9	146367.9	157284.5	129847.1
308946.6	272441.8	137815.1	170129.9	244217.2	203238.1	240441.2	155839.5	170172.5	135198.9
340249.6	272350.4	106037.6	170797.9	249087.9	216214.3	228168.3	153159.8	168380.5	124293.2

LP300.144	LP300.144	LP300.144	LP300.144	LP300.144	LP300.144	LP300.144	LP300.144	LP300.144	LP300.144
103504.2	150864.9	140521.8	211520	200104.2	146328.8	122632.3	122163.6	76547.8	189264.3
105022	154727.4	123524.8	199555	190464.4	143482.4	127307.2	124861.2	85552.66	231157.8
93432.59	165648.7	139529.9	199035.6	198262	146879.3	127264.3	110653.6	83828.13	162324.5
97159.95	160699.1	147099.6	213514.2	187572.3	139280.7	118300.3	115872.6	80110.8	184968.7
102091.6	169963.4	142934.8	203453.5	179669.1	145439.3	127145.3	117418.2	85835.8	173513.5

LP300.144	LP300.144	LP300.180	LP300.253	LP300.289	LP300.290	LP300.29_	LP300.290	LP300.290	LP300.289
116866.9	110321.4	22640.86	29383.5	104738.2	46380.79	35390.03	35697.52	67185.11	35697.08
151914.1	109462.8	22223.31	32366.43	103163	48936.17	42885.66	39500.86	69283.6	35898.55
126276.1	117571.5	22417.89	30130.84	109933.4	47612.03	44612.96	35744.62	63000.4	32736.05
128249.4	115712.7	20356.32	32388.1	112838.1	54056.47	38085.7	39726.1	73091.52	34977.42
121007.2	100979.5	23548.67	34745.1	117237.7	52884.51	39013.98	40762.18	68667.39	33022.35

LP300.290	LP300.29_	LP300.29_	LP300.289	LP300.289	LP300.290	LP300.290	LP300.290	LP300.29_	LP300.290
32273.47	37118.04	55852.21	53794.01	47237.86	31414.06	32921.54	32147.48	35612.79	46417.65
30421.55	31010.9	56517.62	53021.96	48742.19	34276.92	33275.71	30529.11	30517.12	44539.31
32665.01	37712.82	58027.15	58129.26	50196.42	29633.06	34513.76	35657.86	36673.81	47348.12
32219.54	37260.74	64785.83	61635.71	49589.38	37587.66	40840.94	33440.72	37496.85	49930.24
36206.42	38910.93	60048.18	55914.23	51049.87	36956.17	38897.77	30319.89	35711.54	47544.09

LP300.290	LP300.290	LP300.290	LP300.290	LP300.290	LP300.29_	LP300.290	LP300.290	LP300.290	LP300.290
45797.95	45593.07	36598.46	49471.76	42473.38	51602.01	26266.14	41943.98	40062.79	36645.46
44613.11	49304.32	38085.88	42346.73	39235.21	55485.75	27316.23	35907.04	38279.32	36956.57
45846.4	44425.62	38878.86	49097.73	42259.22	57992.42	30742.12	40169.93	43688.99	42880.87
49796.33	52367.79	39727.53	52036.81	42558.7	57469.77	26281.46	40856.7	45050.2	38796.85
47694.67	52713.04	39903.61	53607.8	46897.69	61514.15	31808.55	43205.19	47776.35	42819.08

LP300.29_	LP300.29_	LP300.290	LP300.290	LP300.290	LP300.290	LP300.29_	LP300.290	LP300.29_	LP300.290
51494.3	36585	43181.31	35183.42	50956.86	28065.43	46815.35	43214.78	53590.8	32393.74
52302.89	30007.77	46377.3	33560.66	52138.76	25828.72	46196.57	42233.09	53461.66	32953.03
53665.81	36930.47	42731.88	35417.76	50026.42	30610.44	49957.72	46565.85	56109.14	31159.49
54662.67	36076.96	47135.34	32581.72	58374.6	28025.02	48733.03	46974.69	60048.81	36253.57
58316.28	39687.92	49125.75	33444.31	56804.07	31565.17	48130.46	47699.23	59231.28	38328.75

LP300.290	LP300.289	LP300.290	LP300.290	LP300.289	LP300.290	LP300.29_	LP300.290	LP300.290	LP300.316
38840.76	33358.8	44049.36	42890.28	25714.53	43374.55	70750.83	47637.68	52113.6	64566.1
39048.22	44096.38	45391.57	40189.01	19764.28	40507.23	68562.25	42318.72	47946.45	66331
42557.26	40070.33	38477.56	39916.33	23296.69	45692.05	67235.59	46050.43	50305.11	59793.5
45753.72	39070.91	39940.19	42758.45	25078.1	46571.39	76718.36	50935.22	52233.97	63454.83
45621.99	43071.81	43500.34	45015.47	25385.83	46608.18	77801.49	48973.1	51793.66	68277.15

LP301.141	LP301.141	LP301.141	LP301.140	LP301.140	LP301.141	LP301.141	LP301.141	LP301.141	LP301.141
1643364	2383420	1087953	2374167	2527737	1690586	2354345	5619213	1031743	2535957
1280222	2983477	1191015	3733122	3121015	1402159	2404052	3821145	1454622	2653987
1435314	2013767	1532326	2507905	3404418	2483833	3332710	4107381	1183594	3896853
1164512	3266713	1082159	3154700	2524786	1807143	2393280	5439979	1070281	2594541
1400051	1928335	1220978	3326509	5054337	2092275	2017695	3576465	1037744	2752538

LP301.141	LP301.141	LP301.141	LP301.141	LP301.141	LP301.141	LP301.141	LP301.141	LP301.141	LP301.141
2388369	5088510	2829470	1885469	2629386	3071551	2962286	4396692	3364057	4172273
2929167	3218443	3474895	2882186	3032158	2667507	3200313	4004302	3665127	3687301
3343896	3529741	3476365	4070570	2585856	1776835	3623562	4054217	3796641	3137023
2979005	4178273	3346926	2807472	3868682	2385818	2271176	3933273	1591533	3103526
4123838	3974030	2566696	2590153	2361319	4354962	3440271	4723360	2067064	3353481

LP301.141	LP301.141	LP301.141	LP301.141	LP301.141	LP301.141	LP301.141	LP301.141	LP301.141	LP301.141
3233346	3782213	2379390	1646112	3517838	4215257	4038935	3370171	2343839	4130889
4447294	5118720	1677001	2040995	6218141	4308309	5406848	3034741	2184823	3638499
4205963	2310826	2434229	2599474	4735979	3369082	4061265	3058378	2656679	3409700
3091010	2719044	2135393	2208923	4698694	3740074	3835760	3263891	1817404	2976258
4197421	4630544	2212036	2865604	4148934	4671321	4417433	3533828	1931570	2830791

LP301.141	LP301.141	LP301.141	LP301.141	LP301.141	LP301.141	LP301.141	LP301.141	LP301.141	LP301.141
3675496	4000587	3021194	3294196	2421161	3210289	3482763	1906087	4776570	3611842
3495904	5666145	2925138	3252644	3276548	2995952	3549567	2267035	3606095	2479443
2751721	6574479	3371526	4261442	3754981	3007545	3527646	1929178	4186545	3603230
3415112	2896689	2734253	3428631	2778156	3045909	4919071	2200356	3690673	3255618
3000579	3552241	3485716	4821249	4430435	3420612	3956029	2995993	5065074	3474769

LP301.141	LP301.141	LP301.141	LP301.141	LP301.141	LP301.141	LP301.141	LP301.141	LP301.141	LP301.141
4215214	2428078	3363100	3079381	1666203	2644405	2704632	4068938	6445418	2922541
2722990	3793855	3252449	3082007	2676327	2494185	2611999	3858924	3103801	5550211
3048882	3179037	2674543	2149062	1909411	3142089	2331901	4555901	5198534	4213673
4279993	4408547	3030467	2947409	2234436	2551889	3169012	4025534	3964730	6349328
5397952	4091256	3387936	2486306	1906133	4035100	2557630	3217298	3885257	4027624

LP301.141	LP301.141	LP301.141	LP301.141	LP301.141	LP301.141	LP301.141	LP301.141	LP301.141	LP301.141
3934038	3323815	3880014	3320788	2114212	2431786	4057341	3786226	2118158	3952218
3829739	3891355	4484233	2950315	2226578	2115011	2890389	2009717	2015469	4273447
5358455	3952769	3463111	4776768	2366473	2798985	4184235	2969207	1878444	4068214
4261380	4157452	3949862	3143703	2155084	2334374	2858089	3948310	1726985	3937511
3529421	4056088	3990006	5443208	1971442	1858487	4017617	3058001	1937153	4179615

LP301.141	LP301.141	LP301.141	LP301.141	LP301.141	LP301.141	LP301.141	LP301.141	LP301.141	LP301.141
4099614	2400022	2468677	2791911	1254134	1994340	3380963	2407227	1918817	3021952
3866823	2293293	1805662	2039796	709636.2	1697927	2809205	2837589	2707886	4162516
4157399	3027263	2556505	2304445	1201745	2013174	3060649	2375997	2989262	5078611
5678718	2733408	2041743	3684169	1001084	1955023	3226385	2318648	2338751	3317784
3652084	2350692	2784118	2978733	1028359	2098105	3544382	3086045	2225837	2915842

LP301.141	LP301.141	LP301.141	LP301.141	LP301.141	LP301.141	LP301.141	LP301.141	LP301.141	LP301.141
2298481	1480879	2468056	1676576	2972278	2921116	737522.7	3743244	2298617	797392.9
1848340	982603.8	1605855	1888931	3248458	2850806	1252451	2366479	3084433	1150325
3166990	1082270	1760078	1805073	2853636	2737345	853495.6	3187781	3032940	970032.1
1693987	938577.3	1719566	1916858	2728423	3453132	696195	2257312	2146030	982502.2
1751747	1126345	1715122	1973937	3775920	3320906	1176873	2667079	2715658	1229379

LP301.141	LP301.141	LP301.141	LP301.164	LP301.164	LP301.211	LP301.216	LP301.293	LP302.144	LP302.144
2725985	563261.4	717401.4	44368.37	47240.65	166529.5	19640.63	16134.99	153877.8	267814.9
2899924	399375.4	1189521	46507.02	51628.16	167009.4	18210.73	25585.94	224036.4	322433.3
2921662	652292.4	932052.4	45111.42	47335.29	154966.1	22230.08	24861.65	212836.6	275417.5
2608572	533663.9	750193.8	49036.72	47257.39	145645.6	20981.52	22984.26	200937.2	420054.2
2734286	631350.6	797213.4	47367.36	47371.94	146911	22622.82	25068.47	190723	342870.2

LP302.144	LP302.144	LP302.144	LP302.144	LP302.144	LP302.144	LP302.144	LP302.144	LP302.144	LP302.144
665535.6	432409.6	636404.8	653311	583978.2	360362.3	729120.2	526735.1	231599.9	534442.1
697656.8	472625.6	456117	684744.4	519178	564540.2	577388.2	697947.3	252318.6	581350.8
441785.8	506200.9	577437.8	343387.4	570147.4	377573.5	525598.1	583775.9	174714	645602.1
434055.3	473473.6	591805.4	392287.9	470647.5	446231.9	492942.1	733274.9	188641.7	660672.2
768568.2	574430.6	469114.5	635813.8	404277.2	577353.5	523867.6	548061.7	238845.8	722824.6

LP302.144	LP302.144	LP302.144	LP302.144	LP302.144	LP302.144	LP302.144	LP302.144	LP302.144	LP302.144
403076.8	723020.1	577833.4	604399.4	515791.5	812317	626694.2	719853.7	457884.2	375068.7
451252	723224.2	559209.1	456535.2	368695.9	722171	549225.7	877194.3	536653.5	725722
352606.2	586307.9	380758.4	541673.9	487930.1	600437.2	442832.9	818665.4	468321.8	536994.7
427603.4	619712.4	497955.2	390083.6	354231.3	570217	552810.4	569614.2	396034.5	327217.6
364064.3	951180.8	404896	487828.3	343682.4	738727.2	758836.9	749800.8	595759.2	534457

LP302.144	LP302.144	LP302.144	LP302.144	LP302.144	LP302.144	LP302.144	LP302.144	LP302.144	LP302.144
417751	739025.3	539963.7	451944	840064.3	785670.5	778894.4	312734	362869.2	647049.8
532631.6	630689.5	434819.1	471729	819821.9	495095.4	573234.7	315391.6	265786.8	657361.4
505861.5	587850.1	554954.1	279254.8	782539.7	677714.5	556436.8	600620.1	372568.4	508867.2
480454.3	556725.6	428181.5	356266.4	672135.8	450960.4	557174.1	309691.7	456645.3	823122.4
407174.8	817482.5	524656.9	346954.1	644846	930532.4	654672.5	276861.3	411163.2	695720.8

LP302.144[illegible]	LP302.144[illegible]	LP302.144[illegible]	LP302.144[illegible]	LP302.144[illegible]	LP302.144[illegible]	LP302.144[illegible]	LP302.144[illegible]	LP302.144[illegible]	LP302.144[illegible]
875020.6	558915.3	858950.8	522444.8	316551.3	505648.9	453997.5	573289.8	454376.7	506474
686383	656287.1	844645.3	428551.4	426555.1	602271.9	351679.4	612678.7	402162.6	487619
559656.3	503846.1	673599.2	457498.6	281740.9	864926	384751.9	739949.4	455898.7	695699.2
793773.3	593132	736034.9	391473.7	317123.1	624047.1	356149.2	546343	381878.3	734045.4
795340	513856.1	537842.7	442880.4	373677.6	561937.9	424665.8	654142.9	303305.5	779499.7

LP302.144	LP302.144	LP302.144	LP302.144	LP302.144	LP302.144	LP302.144	LP302.144	LP302.144	LP302.144
707826.4	723327.6	305188.6	405773.9	321587	520991.7	441511.1	167907.9	290451.9	510989.9
547222.5	386789.5	320314.8	370774	476067.8	442125.9	411010	207850.1	453259	386297.4
643133.3	432822.4	508614.1	349265.9	404901.2	393237.7	429852.4	144548.4	363792.6	389251.3
578665.2	475029.7	376662.4	319261.3	439395.8	382561.2	517491.1	175565.6	322160.8	410702.5
539453	516808.7	359809.8	424141.3	334021	487866.4	334972.9	166237.1	391895.7	608722.4

LP302.144	LP302.144	LP302.144	LP302.144	LP302.144	LP302.144	LP302.144	LP302.144	LP302.144	LP302.144
477539	665974.8	423267.3	537040.5	598099.2	947499.9	610702.1	685444.9	457948.4	192722
324336	644332.1	449930.7	717345.7	642873.6	690629	709743.5	577357.1	577145.3	181613
384810.9	479038.4	496101.7	501385.6	554200.8	746503.9	740253.4	829825.3	380200	223362.1
414124	751641	510572.3	714382.4	730625.8	646777.2	835911.3	803520.3	519702	204650.5
411188.2	710538.1	480938.6	587619.3	773819.5	623920.6	584168.1	604845.6	368011.5	229737.6

LP302.144	LP302.144	LP302.144	LP302.144	LP302.144	LP302.144	LP302.144	LP302.144	LP302.144	LP302.144
226082	781850.3	589570.3	684121.6	581275.2	344404	505985.5	753163.6	259026.8	561583
205134.8	810053.2	510666.7	823055	476340.1	379590.8	622976.6	517399.9	392939.2	566564.1
138216.8	780556.5	453385.6	667918.5	397536.9	327417.3	693669.4	421688.7	261835.6	752711.7
203059.5	670867.8	578876.1	705997.4	534554.6	393037.3	523845.8	527262.1	321081.7	639780.5
178836.3	519502.9	554238.8	753293	735169	438245.5	660822.3	633387	330166.9	603378.2

LP302.144	LP302.144	LP302.144	LP302.144	LP302.144	LP302.144	LP302.144	LP302.144	LP302.144	LP302.144
573593.4	507551.6	801665.1	122076.2	129116	520529.3	142357.4	448075.9	149082.1	476015.9
572171.9	375232.5	583304	101556.5	139663.7	493195.7	128344.4	471356.2	180904.1	476282.8
686294	394861.7	617729.9	132095.4	193337	534971.3	138546.3	418250.3	164845.8	692970.2
558465.8	489364.9	662862.4	129316.8	157247.1	721097.7	122072.3	437164.6	135454.3	499898.1
506310.7	553548.3	605879.9	123101.9	134554.3	518678.2	109282.7	531791.9	165886.1	833823.4

LP302.144	LP302.144	LP302.196	LP302.196	LP302.195	LP302.196	LP302.196	LP302.196	LP302.195	LP302.196
176594.7	122915.3	39405.46	37558.1	39387.97	47283.44	33730.18	48530.17	33095.48	44877.76
175519.7	129791.9	43500.22	38404.76	43195.42	40054.55	35862.07	48581.12	35951.18	40554.11
146305.9	119132.5	42065.74	34041.54	41135.46	39216.5	34358.58	43172.51	31837.13	45891.81
172673.4	102471.1	47900.15	37065.09	43204.22	44875.17	38303.57	42888.61	32374.78	43464.69
172684.4	155426.1	39388.52	32799.35	35148.16	36854.5	35657.57	41831.11	30011.54	45475.7

LP302.196	LP302.214	LP302.232	LP302.232	LP302.305	LP302.305	LP303.144	LP303.143	LP303.144	LP303.144
39338.53	24673.74	36348.3	45425.9	37684.57	36978.44	51903.67	50184.29	37189.45	48055.22
35758.53	49436.26	37455.71	44847.83	40305.96	51879.83	50218.23	49233.74	45792.45	51071.62
37495.74	51856.41	34186.1	45194.29	43878.61	52784.46	46054.93	49182.75	41804.97	43182.32
40473.99	25640.26	31950.1	41969.74	27414.45	62052.55	53521.73	51811.83	43470.85	46754.77
35857.57	28091.71	30719.91	41919.66	35288.89	48703.26	46263.04	54324.46	40185.71	49521.21

LP303.143	LP303.142	LP303.143	LP303.144	LP303.144	LP303.144	LP303.144	LP303.144	LP303.144	LP303.144
54567.56	69489.67	67485.88	52788.84	47353.94	43670.64	58228.99	55326.32	49940.9	50377.04
51010.22	57627.11	63924.98	56031.35	46485.06	38585.89	54821.53	45909.29	49376.13	49473.67
62632.78	58841.83	64704.11	51861.42	47814.35	39833.49	53619.76	53764.12	46132.67	52385.42
53289.85	56899.76	54933.69	54943.56	41780.6	39280.5	49251.7	51634.86	46781.31	50887.98
58568.63	49678.61	51212.39	49530.75	42155.79	32313.56	46440.08	50714.79	49333.55	48448.11

LP303.144[illegible]	LP303.144[illegible]	LP303.144[illegible]	LP303.144[illegible]	LP303.144[illegible]	LP303.144[illegible]	LP303.144[illegible]	LP303.144[illegible]	LP303.144[illegible]	LP303.145[illegible]
44125.18	53592.04	45713.09	48496.89	50664.22	49809.86	46467.47	34840.23	42682.58	30485.64
45544.99	47408.42	43297.77	49718.97	53257.14	48778.34	53963.28	36118.05	40738.74	36510.41
42622.55	52888.87	50182.89	52749.39	51845.07	50230.23	52497.14	37906.62	42044.83	33686.36
45219.86	55891.92	41953.4	59424.47	54056.43	58052.54	43693.79	33622.54	41793.61	32792.34
40882.45	50236.62	50447.57	58701.18	49384.43	55995.96	55142.61	38449.59	38580.46	28583.93

LP303.144	LP303.145	LP303.144	LP303.144	LP303.145	LP303.144	LP303.145	LP303.144	LP303.147	LP303.144
40371.08	47968.37	40801.17	39336.94	44258.11	47570.33	41130.73	25096.67	13535.81	39981.15
38380.83	43726	40426.37	42379.31	43049.17	48467.31	38277.13	32205.29	13334.39	48737.95
37512.4	47530.62	44828.56	46640.54	44665.82	46991.04	42016.05	32298.81	12377.65	50672.98
42755.22	47260.09	47846.64	43844.6	44040.05	42521.34	41274.36	29020.72	9829.6	46091.01
39172.13	45969.42	40440.13	49013.04	47414.51	46837.04	42951.15	31536.22	10896.25	50050.68

LP303.143	LP303.180	LP303.253	LP303.253	LP303.253	LP303.253	LP303.289	LP303.304	LP304.211	LP304.211
42474.49	25460.95	13266.47	25265.62	32251.88	29927	69882.69	24347.36	189936.2	179286.9
42678	27365.89	13034.49	23668.88	30977.61	26764.54	59943.96	22066.28	189313.9	175999.7
38368.2	36146.46	11640.48	22056.89	35041.61	27004.55	62613.51	25522.61	193585.5	176538.1
41475.82	40771.98	13945.5	24783.49	31693.3	26789.18	84662.79	17900.7	206667.4	198807.6
41533.44	45333.74	16878.15	26002.08	34140.08	26441.56	72866.53	18034.87	125100.2	210155.8

LP304.211	LP304.212	LP304.212	LP304.247	LP304.247	LP304.248	LP304.248	LP304.248	LP304.248	LP304.248
149087.9	139336.2	184248.5	124628.1	107336.9	185370.9	105312.2	119262.7	126667.6	134512.1
136967.5	143901.5	178239.1	118620	110470.3	180526.8	87923.38	126432.4	120935.3	126101.5
153367.3	142889.7	189232.1	115595.9	118152.3	163926	104935.2	119332.2	128442.8	134232.8
165652.9	164022.5	192783	128712.5	124163.3	187259	106512.6	127535.9	128828.6	142198.2
186911.2	80201.97	218990.5	146567.7	135498.1	186810.2	131021.4	147032.6	156072.1	153753.6

LP304.248	LP304.248	LP304.248	LP304.248	LP304.248	LP304.248	LP304.248	LP304.248	LP304.248	LP304.248
121793.1	143436	114819.2	120134.6	101009.6	168356.5	125710	138214.3	89665	86856.26
128788.2	141571.3	118642.5	113471	92924.58	166653	107538.5	131105.9	86123.95	88256.68
127955.6	139372	117397.7	127096.9	101683.3	171142.3	114903.3	120972.2	91606.82	84325.72
131172.8	144621.8	124828.4	117904.7	95377.01	171298.5	116306.6	144676.7	96222.13	83812.93
148208.6	154098.2	132748.9	156772.3	107369.5	200617.1	128898.8	151881.5	102036.5	95335.43

LP304.248[illegible]	LP304.248[illegible]	LP304.248[illegible]	LP304.248[illegible]	LP304.248[illegible]	LP304.248[illegible]	LP304.248[illegible]	LP304.248[illegible]	LP304.248[illegible]	LP304.248[illegible]
105328.7	117275.2	102551.8	169492.3	144079.5	100604.5	150030.3	105427.2	139663	139594.7
104947	109219.5	105423.2	160607.7	153928.6	98874.39	136591.7	101430.1	133336.8	133221
107565.7	97850.35	101025.8	163362.8	147664.1	96243.98	137285.1	103298.3	131585.1	133906.4
101088.2	109128.8	106154.5	162107.4	141474.9	103752.8	143029.4	108475.8	134561.8	140111.7
112907.6	123699.6	120310.9	191669.5	182277	99722.82	160466.1	121330.7	150464.9	152743.6

LP304.248	LP304.247	LP304.248	LP304.248	LP304.248	LP304.248	LP304.248	LP304.248	LP304.248	LP304.248
116319.8	153641.4	145146.3	163281.3	126360.2	122368.4	143837	120163.6	112444.7	118367.8
123203.6	126814.5	148085.5	169424.2	122254.3	118407	133493	127966.7	114686.6	129933.6
103783.5	141345	146915	172984.8	121764.1	120295.7	141724.8	110752.3	107771	125071.1
109294.6	144644.4	154166.1	169867.9	133481.5	120948.8	149474.6	130162.6	104726.5	131396.1
122751.3	166341.2	154169.1	197862.7	157777.9	146563.2	166154.3	146419	124211.7	157185.4

LP304.248	LP304.248	LP304.248	LP304.248	LP304.248	LP304.248	LP304.248	LP304.248	LP304.248	LP304.248
147494.9	141926.1	129585	102494.1	158897	116076.2	175165.2	122932.6	173559.4	137982.7
155471.8	140590.3	131065.6	98632.06	155891.4	127303	182908.7	125165	148277	124837.2
152341.9	148135.3	135547.7	98827.33	154601.9	112112.4	180039.2	116196	154286.8	122507.3
161939.7	147015.3	122964.9	99573.81	157224	128625.1	193367.1	129984.7	165774.8	121403.3
176899.5	163727.4	161013	104772.1	173088.8	147846.3	216408.3	129724.6	181401.4	139518.3

LP304.248	LP304.248	LP304.248	LP304.248	LP304.248	LP304.248	LP304.248	LP304.248	LP304.248	LP304.248
124498.7	160434.2	143200.5	145538.4	122298.1	119920	157656	116802.1	103111.3	97049.4
134189.6	153782.9	145499	130445.6	108871.4	130700.5	142741.6	114694.1	91083.58	94014.55
131204.6	150629.5	149491.2	141427.9	115137.2	123851.7	120396.9	115055.6	94934.88	95041.29
132474.6	171821.3	148246.4	155953.5	127042.1	137407.5	165477.9	131239.4	98858.33	108310.5
159409.8	178912.4	179894.8	185493.3	136470.5	154274.8	180251.6	148558.9	124066.3	108612

LP304.248[illegible]	LP304.248[illegible]	LP304.248[illegible]	LP304.248[illegible]	LP304.248[illegible]	LP304.248[illegible]	LP304.248[illegible]	LP304.248[illegible]	LP304.248[illegible]	LP304.248[illegible]
112506.7	83342.12	96182.93	121332.5	99215.63	116260	89473.5	185137.9	104567.8	147402.8
117640.5	81770.48	90364.85	122734.9	104405	114275.3	87165.87	183804.7	101986.5	140871.8
122926	87360.53	94979.21	124330.5	101541.9	118394.6	88607.79	177072.6	94044.16	139381.6
132391.9	84570.43	105197.3	134560.2	109457.7	119474.1	89273.04	195059.5	98928.43	144431.9
139631.8	114892.4	113023.3	160887.7	121707	135944.1	119801.5	218929	126958.5	169210.7

LP304.248	LP304.248	LP304.292	LP304.300	LP304.299	LP304.894	LP304.894	LP305.174	LP305.247	LP305.247
175868.4	171214.4	11035.24	50410.75	18194	79230.94	61019.23	16568.43	41396.91	48676.32
181123.6	168112.2	14558.12	57579.91	16802.77	79650.95	59244.81	14226.58	35700.5	46948.78
173719.7	164506.6	15801.61	56734.92	16041.85	82826.67	78895.08	18385.04	46687.99	41295.72
177357.4	157988.7	9997.8	65819.89	13634.96	89899.52	77349.84	16818.79	42936.04	43818.42
201168.5	193098.3	13308.63	63326.37	17045.1	71100.77	60294.31	16579.29	40305.73	51770.3

LP305.247	LP305.247	LP305.295_	LP305.899	LP306.155	LP306.155	LP306.155	LP306.191_	LP306.191_	LP306.191
34231.52	41278.35	12504.16	31760.44	75398.14	80452.47	96422.95	73291.06	122325.1	101921.2
34803.19	43937.43	11799.72	25714.24	82807.84	73758.93	108243.6	68789.5	128305.6	99907.86
32488.74	43120.95	15928.63	45083.7	72974.53	78973.33	92453.72	68774.43	109759.5	108751
30195.45	41589.63	18817.2	60893.41	77600.68	72647.11	100080.3	79674.11	115264	108609.5
31794.58	41231.55	17364.79	24160.15	79183.36	79361.44	86497.01	76051.11	107863.6	103979.4

LP306.191	LP306.191	LP306.190	LP306.191	LP306.191	LP306.191	LP306.191	LP306.191	LP306.190	LP306.190
44727.91	60093.11	80578.2	84610.32	69968.64	88228.15	112926.6	108342.5	68889.67	57488.14
57497.58	58726.72	86346.9	84982.28	62665.5	85407.45	113039.7	108100.4	79898.85	66702.37
54797.82	55977.13	85433.31	80912.36	65454.54	79527.99	115981.6	99692.18	68331.74	56256.98
51888.05	61838.24	89535.36	81700.09	60391.05	88205.71	117860.7	117064	70222.78	57559.14
53943.92	52937.57	82061.27	100603.1	80848.38	77929.56	104843.4	99183.19	66837.06	54098.04

LP306.191	LP306.191	LP306.191	LP306.191	LP306.191	LP306.190	LP306.276	LP307.190	LP307.19_	LP307.190
57035.89	62924.72	97529.21	85232.13	58846.26	105964.7	40792.9	33804.54	37260.41	35244.8
63340.65	62549.75	106854.3	84836.66	66814.48	115717.1	44341.58	37801.99	37045.53	33331.83
58679.74	70483.43	100956.8	82850.87	66188.6	121120.6	58668.25	29233.55	42717.02	35051.98
60491.41	65313.9	101174.5	88089.86	64816.33	110289.2	62038.1	36165.67	43432.91	38989.95
54395.03	59686.31	104462.5	83163.9	78792.66	114075.8	68873.15	35263.86	41177.46	35981.15

LP307.190	LP307.189	LP307.226	LP307.226	LP307.226	LP307.263	LP307.263	LP307.263	LP307.263	LP307.263
41056.13	48356.61	27665.16	33346.07	40015.08	49627.44	30304.54	53860.29	38289.88	46069.93
40309.97	38878.35	37471.26	39185.78	36010.72	58789.85	38882.81	50226.81	41011.63	50013.24
37364.45	41507.59	41589.72	35717.76	39630.54	50114.18	28821.18	45157.63	35287.28	50214.56
35359.54	38277.3	38086.1	35521.27	35345.18	47375.36	29959.12	54189.34	39028.38	43680.46
33810.98	44500.61	35791.23	35346.83	42770.53	47636.16	33392.26	53491.56	39166.78	42372.9

LP307.280	LP307.310	LP308.206	LP308.207	LP308.206	LP308.206	LP308.206	LP308.206	LP308.206	LP308.207
37517.89	23777.16	45733.13	80264.91	51636.89	55477.05	38734.81	32543.61	53186.39	66030.52
33517.74	24245.39	44000.98	79222.23	48584.86	65300.47	40267.14	33053.46	54760.44	66069.75
37403.27	23298.49	46550.52	85943.69	59304.22	49074.24	36877.91	29967.55	52462.41	72807.43
29003.84	24334.07	44814.7	86005.32	50380.26	49036.2	39995.73	30197.33	56442.5	66308.63
34878.34	24010.51	48020.88	80138.75	48461.16	55956.09	36455.99	27121.65	51404.19	56266.32

LP308.206	LP308.206	LP308.207	LP308.207	LP308.206	LP308.206	LP308.279	LP308.295	LP309.168	LP309.168
39950.56	43320.24	61109.17	51424.15	57746.6	40147.95	30388.58	26907.34	67126.05	78439.31
42343.77	41788.28	66514.5	51606.91	53959.5	41184.03	29231.38	22993.59	66655.63	62256.34
39187.48	39054.89	76851.73	54860.38	59265.48	40631.54	29507.19	20405.44	68315.03	58732.14
41947.87	40409.56	77428.15	47803.98	59770.01	38866.1	26231.73	8973.647	64108.54	56228.71
39730.34	40205.31	64999.09	47527.16	46639.48	41012.19	28403.15	18661.7	66674.71	57284.85

LP309.168	LP309.168	LP309.168	LP309.167	LP309.168	LP309.168	LP309.168	LP309.168	LP309.168	LP309.168
67403.24	58199.61	74814.67	57360.14	64417.28	72838.89	62587.17	86958.47	57710.65	73518.43
72375.03	52164.59	73229.87	59494.3	59243.61	75223.64	62558.92	58441.03	55297.32	68751.45
60356.09	53213.42	70712.79	66324.58	60727.28	76612.85	61881.77	62736.5	59911.78	68707.67
70854.42	49191.11	69507.69	58469.09	65971.6	75296.6	63932.39	59638.23	59917.54	65119.83
69215.76	52744.03	62859.3	60265.03	67112.61	77408.79	57668.26	62563.45	63280.61	65493.64

LP309.168	LP309.168	LP309.168	LP309.168	LP309.167	LP309.168	LP309.168	LP309.168	LP309.168	LP309.168
75983.02	74902.24	68889.02	49892.71	67493.04	52675.31	64894.62	65773.65	63415.58	60036.02
68612.57	79700.87	65180.16	54389.55	69829	51229.61	69370.73	63875.58	64513.16	62257.76
69391.21	78104.26	70900.18	56898.77	80626.13	57440.78	62108.7	66874.47	61941.33	69727.52
102656.6	79835.04	71656.7	68191.97	75089.04	53242.85	62682.71	64938.91	57935.51	70632.37
67077.2	74294.96	71066.03	56419.26	67671.19	45460.6	69033.2	67640.8	67080.12	61794.67

LP309.168	LP309.168	LP309.168	LP309.168	LP309.168	LP309.168	LP309.168	LP309.168	LP309.168	LP309.168
67581.79	67102.02	70862.74	70313.57	59522.72	73752.49	69031.74	69277.17	59790.78	55725.92
68134.29	73236.39	69694.14	66730.82	53358.17	78446.12	71116.7	63856.33	63693.12	46538.07
71239.22	73414.51	76491.65	67338.11	57816.99	72648.9	71020.08	72147.35	65901.95	49799.56
65868.56	70521.13	61506.49	68467.62	54373.93	77605.73	63117.57	66968.79	66822	46786.22
67145.81	68268.62	73519.23	65762.48	50916.33	78281.16	66680.84	61251.49	59042.4	46636.88

LP309.168	LP309.168	LP309.168	LP309.168	LP309.168	LP309.168	LP309.167	LP309.168	LP309.169	LP309.168
77058.06	67144.65	56178.22	73933.18	97398.63	69001.84	69814.73	76247.48	63386.49	49946.98
74297.53	65640.24	59168.11	67888.58	86257.42	65202.69	67854.98	79252.97	62581.8	48907.77
79162.81	58984.49	64148.2	74888.14	90848.61	65839.2	69443.01	83095.02	66602.38	52596.73
81214.99	59352.96	68388.51	82033.39	86644.53	67921.53	67753.87	85030.06	63311.06	58692.67
66869.75	63151.24	54076.53	66158.07	88655.94	67989.95	62064.75	84009.06	61802.64	46015.69

LP309.168	LP309.168	LP309.168	LP309.168	LP309.168	LP309.168	LP309.168	LP309.168	LP309.168	LP309.168
72744.86	76414.48	74612.38	98737.96	42047.09	50231.29	51037.5	73404.68	57534.98	64221.41
74254.25	77115.43	74826.72	96172.53	44002.37	56615.91	60938.85	64068.82	54979.01	58521.96
72072.09	68843.25	77778.15	98161.79	48210.71	56056.82	59466.41	65846.2	61039.66	61970.98
77278.33	72409.51	77856.6	100011.4	40437.39	50427.02	49134.76	70110.43	67346.06	62038.61
67806.65	74050.5	79859.28	92230.89	43251.2	47965.63	54988.76	67832.27	54166.5	54397.06

LP309.168	LP309.168	LP309.167	LP309.168	LP309.168	LP309.204	LP309.205	LP309.205	LP309.205	LP309.205
56428.79	50701.02	47883.31	79805.6	81742.04	53549.5	63684.46	55764.3	67466.53	72842.47
61912.17	46789.76	45768.45	72408.84	78375.22	53654.56	66031.42	57793	61586.72	70849.7
61359.41	48006.35	48164.59	75990.42	78383.34	59014.97	66879.2	54940.85	64812.94	64944.92
59577.58	48011.8	53127.31	75787.69	86000.1	63556.02	63223.15	55730.02	65492.17	63135.32
62850.61	48312.04	44887.44	74884.34	80756.85	50504.64	62735.23	54973.35	75326.15	58170.3

LP309.205	LP309.205	LP309.205	LP309.205	LP309.205	LP309.205	LP309.204	LP309.205	LP309.205	LP309.205
69249.36	61880.2	46292.97	51484.99	47330.21	53924.32	70385.46	63019.79	71690.77	56309.93
57010.74	46687.12	52262.27	72423.31	47610.94	44907.6	39325.1	57006.86	67131.59	54830.31
68934.86	56857.06	51809.39	50892.4	45028.48	53858.32	62841.7	57366.57	66249.76	60077.89
65956.13	57385.67	54509.31	48936.41	41089.96	50752.98	63478.66	55044.52	60849.07	57405.86
69697.58	57183.63	53419.56	53221.45	45504.7	45786.37	61038.29	58898.18	68927.93	59495.7

LP309.205	LP309.205	LP309.205	LP309.205	LP309.205	LP309.205	LP309.205	LP309.205	LP309.205	LP309.205
78121.74	58736.27	81960.17	86314.49	64442.08	71033.93	62522.41	74672.87	49649.83	82007.82
85808.14	52424.33	80287.86	82681.34	66666.56	62402.69	59200.66	72946.35	44711.82	72221.61
75490.92	61123.09	79015.17	93569.55	68899.33	71360.61	56774.29	71619.05	49853.04	74306.52
80262.19	56294.07	81662.18	83468.79	69094.01	72524.5	60510.11	76859.41	54847.19	78302.75
70651.94	60052.71	84755.73	88002.89	61724.09	69075.36	56978.99	67022.27	45813.63	76634.37

LP309.205	LP309.205	LP309.205	LP309.205	LP309.205	LP309.205	LP309.278	LP309.278	LP309.278	LP309.278
59667.88	91971.08	60221.11	47982.69	82332.97	57903.27	70898.99	54633.18	59468.27	46921.42
51246.96	92365.17	62128.53	51751.93	80428.75	54881.99	62893.51	51656.88	59960.17	37943.62
56575.01	88372.73	60814.17	56710.26	87574.58	53743.8	63344.22	57321.35	66677.59	38292.44
50961.96	79977.42	62233.22	55469.81	79685.77	58406.14	64379.48	53312.14	55475.93	43044.14
50694.46	85642.4	67106.86	50832.5	80962.8	52147.93	57137.32	50577.14	59481.2	42441.48

LP309.278	LP309.278	LP309.278	LP309.278	LP309.278	LP310.201	LP310.201	LP310.201	LP310.201	LP310.201
41624.04	47059.82	51323.05	33524.74	47999.33	35276.58	24987.89	23222.02	26520.83	20819.44
42524.75	46974.1	53968.16	35835.68	52650.05	30820.4	28878.93	23430.09	31379.53	21014.56
46347.25	50467.85	55811.96	40469.09	46153.48	30988.17	24574.88	22754.91	30020.23	20995.43
45602.53	48253.7	51477.75	36524.86	48520.56	29437.82	26046.39	25227.78	28701.65	20665.01
45828.64	47473.81	53211.78	36907.95	47522.93	30350.23	27602.27	24614.79	31611.08	17997.63

LP310.237	LP310.310	LP310.310	LP310.310	LP310.311	LP310.310	LP310.311	LP310.311	LP310.311	LP310.310
34478.13	945303.2	131637.5	77811.63	69416.39	75125.69	91376.84	100750.9	81787.65	124792.8
31169.15	901116.2	216673.1	88988.04	75252.36	66260.22	95128.07	101215.4	84046.75	121025.1
34833.31	874092.5	143171.8	100456.3	91306.07	123121.9	104790.8	97448.07	89483.67	136034.4
30402.27	594637	133466.2	94760.43	92670.96	76186.98	93305.71	99510.96	80456.18	140627
32573.88	854828.8	126051.1	93730.2	88752.47	81504.7	97841.72	100163.2	79962.66	131884

LP310.310	LP311.146	LP311.146	LP311.146	LP311.146	LP311.146	LP311.146	LP311.146	LP311.146	LP311.146
208571.8	145941.6	123711.6	148952.7	175530.1	163714.9	136566.9	149207.2	138472.5	98509.96
208045.8	171708.1	112846	183566.1	111680.3	184945.4	143854.2	134573.5	103202.6	147370.6
220118	150375.5	135659.1	138640.5	111032.8	159573.5	131928.1	192918.3	111177.6	148358.8
214237.2	193914.8	154283.3	140586.7	125233.2	189382.8	179123.8	143299.9	131763.6	134894
225891.9	116041.3	113646.2	115944.6	166702.5	124736.1	167937.9	147113.5	139743	113257.8

LP311.146	LP311.146	LP311.146	LP311.146	LP311.146	LP311.146	LP311.146	LP311.147	LP311.146	LP311.146
132740.7	175060.3	131428.8	158636.8	192423.6	104766.5	230815.6	115158.6	147711.1	219200.5
126738.2	126036.2	139585.8	114743.9	128686.3	121591.5	142020.3	131026.4	192801.3	161162.6
129408.7	116823.1	91595.12	183088.5	159727.2	101095.7	106893.1	93424.09	126913.7	123302.7
169475.3	148538	106587.7	123804.7	127683.3	124213.4	137200.7	93268.78	140750.9	164634.9
138211.2	128740	126888.4	110306.3	148638.7	140480.5	133983.1	128637.5	128895.1	171676.3

LP311.146	LP311.146	LP311.146	LP311.146	LP311.147	LP311.146	LP311.147	LP311.146	LP311.146	LP311.146
124827.8	171321.1	121778.4	127067.4	77650.84	111454.8	233730.6	180768.8	156388.6	172319
107194.7	132692.6	122540.7	138085.6	81919.1	124996.5	138234.8	145925.8	150173.5	142754.2
95681.65	200748.7	125129.2	138643.4	77404.76	117217.1	246763.6	173832	119489.1	213421.7
131696.1	155765.6	176436.2	117132.9	94276.21	166781.4	159590.9	138306.2	141736.8	129467.4
133771.1	180175.3	195708.9	116152	90286.28	102199.8	238073.6	173659.4	114119	145499.3

LP311.147	LP311.146	LP311.146	LP311.146	LP311.146	LP311.146	LP311.146	LP311.146	LP311.146	LP311.146
171133.7	128685.5	134083.7	125587.1	110094.1	179180	175599.1	137254.3	156059.9	114168.7
236117.4	171536.6	119677.7	148916.3	108462.8	203225.2	154204.7	131366	155283	188464.9
202964.9	122603.5	99160.69	141406.8	121389.9	167286.3	224769.6	133207.9	107015.5	198800.4
182201.2	145107.6	106267.7	144815.7	149530	145188.3	167016.2	130931.5	172714.6	119884.9
157363.8	164772.5	83640.84	120803.6	109205.9	154494.1	157768.7	130751.3	166308.7	155774.2

LP311.147	LP311.146	LP311.146	LP311.146	LP311.146	LP311.146	LP311.146	LP311.146	LP311.146	LP311.146
104577.7	187675.2	141363.3	151866.8	141256.7	175194.8	105913.6	174120.6	141194.8	137425.9
84938.87	122408.2	144898.6	121501.3	106877	102152.5	122559.3	206883.1	165346.1	124918.3
102921.1	115627.1	123437.7	159982.4	125169.2	132771.1	114449.6	135947.5	148190.4	145509.6
94955.66	128338.7	121317.4	157841.5	180472.3	131364.9	142531.9	142609.9	160253.9	131215.4
143373.4	135915.3	111469.9	117868.3	119299.6	144515.8	153846	164993.6	149945.5	114318

LP311.1468	LP311.1469	LP311.1468	LP311.1469	LP311.1467	LP311.1469	LP311.1469	LP311.1469	LP311.1469	LP311.1469
132788.8	107060.3	175765.9	142586.8	111394.1	119337.5	110635.8	196165.8	130761.5	124393.9
145062.4	161789.7	122378.4	144163.4	112989.4	189039.4	185653.6	155479.7	126049.7	142006
102435.3	130560	185132.1	117321.3	162012	147783.2	124613.5	205448.6	139733.4	145784
166869.3	157261.1	155073.1	143054.7	123109.7	183373.1	130151.4	154871.8	157217.1	136625.5
113140.8	109271.7	133173.6	172870.2	78409.04	195363.3	117329.1	174910	134344.2	138838.2

LP311.146	LP311.146	LP311.146	LP311.146	LP311.146	LP311.146	LP311.147	LP311.146	LP311.146	LP311.147
151849.7	108605.2	177813.6	145571.1	139534.8	107932.2	152722.3	127896.5	88168.84	195666.6
127882.6	87593.54	157079.3	149848.4	181095.3	141743	149597.9	146553	100094.1	159849.2
145598.9	95842.15	154815.9	133012.8	291043.1	132951.2	150091	131129	96255.41	137057.9
164905.2	84507.86	143505.4	202496.7	165501.7	161826.5	141890.3	144693.8	102248.3	155558.9
200709.9	101647.7	169905	152634.5	133695.4	125401.5	149873	179427.4	89285.96	171954.5

LP311.146	LP311.146	LP311.147	LP311.146	LP311.146	LP311.146	LP311.146	LP311.146	LP311.146	LP311.146
79017.23	137671.1	139649.8	120849.2	73813.11	128844.4	57978.5	123921.2	130177.7	82787.51
88611.21	135111.7	151707.4	104316.7	77655.94	204424.9	70416.31	112069.5	162751.4	89319.2
92709.56	122977.2	193767.6	104523.8	109740.8	144069	82780.24	120745.5	159477.1	94683.25
106299.5	127471.6	176824.5	101086.8	83327.15	141222.9	113946.7	104815	158950.5	98991.29
99754.35	170928.6	111905.5	139850.5	82186.93	130328.1	78795.85	147770.9	159035.1	96052.45

LP311.146	LP311.146	LP311.146	LP311.146	LP311.146	LP311.184	LP311.185	LP311.185	LP311.185	LP311.184
106534	75645.29	115562.6	88932.14	130951	114227.6	103171.3	90942.75	86598.06	73208.13
69842.91	69024.28	156186.5	93546.95	60078.72	108140.8	106566	78883.45	90155.32	84809.53
70850.84	80825.76	196054.4	109750.4	73498.4	117903	104370.9	106926.9	81797.08	81002.87
98039.83	64236.23	153147.3	73449.15	82594.99	120614.3	107328.4	82558.48	92699.2	76842.35
67790.32	115446.2	156712	76407.36	74143.85	118183.4	108480.8	88933.51	79970.85	79956.77

LP311.185	LP311.185	LP311.185	LP311.221	LP311.221	LP311.221	LP311.221	LP311.221	LP311.221	LP311.221
96313.01	80397.64	60295.19	74670.28	142101.3	106281.7	76035.9	85795.62	86851.9	120801.7
99804.2	78958.12	64355.49	39421.16	72212.45	52667	37977.57	47548.84	46736.26	64126.9
98715.75	80009.41	60540.59	72392.23	145760.4	107852.6	70269.22	85700.07	81583.03	125861.2
95117.81	86634.49	56382.15	69480.56	133313.1	102218.5	76899.51	83456.45	82514.26	127482.6
89384.33	81087.06	58389.03	71548.6	134163.2	106839.4	69167.64	81510.38	81655.75	126250.8

LP311.221	LP311.221	LP311.258	LP311.294	LP311.294	LP311.294	LP311.294	LP311.294	LP311.294	LP311.294
57982.38	86894.29	131677.5	170451.1	102928.8	163013.6	101739.8	189384.6	102496.5	155440.1
39651.45	53507.26	112945.7	160191.5	109246.2	128330.3	109644.9	178676.3	78547.64	192245.1
65324.81	87519.22	120468.6	167947.3	110439.8	122584.9	97498.63	175989.3	80927.61	183387
64843.76	77981.53	105681.3	195523.2	119363.3	132716.2	101531.1	186272.6	99306.61	186601.1
58093.7	87422.14	120944.3	157877	105944.4	145988.8	103220.3	104522	78300.95	197992.8

LP311.294	LP311.294	LP311.294	LP311.294	LP311.294	LP311.294	LP311.294	LP311.294	LP311.295	LP311.294
107246.5	188349.5	162649.7	98224.33	108245.4	122387	152880.3	138090.4	128418.7	215412.2
110235.7	148700.1	117199	83907.68	92420.57	120205	149974.6	145534	125241.1	208663.1
114378.4	147360.1	94876.25	77478.07	93837.31	116890	140328.4	146990.7	124613	200022.1
109492.7	159248	95055.92	82570.88	88961.95	151042.8	143808.8	145152.8	135618	196953
124129.8	163736.3	99936.29	79212.27	89776.24	126516.6	150237	136833.1	141404.5	256735.3

LP311.294	LP311.294	LP311.294	LP311.294	LP311.294	LP311.294	LP311.294	LP311.294	LP311.294	LP311.294
201857.6	121154.7	93952.31	74955.14	127114.3	114238.7	125613.4	91746.9	88465.95	121927.8
197075.5	115742.1	93268.39	70490.77	123606.7	96004.3	121983	82250.48	91087.25	115220.2
196551.6	106941.1	89943.66	79909.53	119683.6	96601.67	117432.1	87757.71	93693.74	109466.1
201075	123969.5	91787.09	72992.05	119265.4	100199.5	124547.5	88949.41	91271.19	117493.5
204809.1	120066.4	101508.3	68984.03	128890.3	103126.1	123050.3	85702.12	96736.34	120804.7

LP311.294	LP312.230	LP312.253	LP312.289	LP312.289	LP312.297	LP312.298	LP312.297	LP312.317	LP312.326
83032.9	53676.14	24538.25	19765.44	22564.98	32222.57	28193.12	44998.68	39077.76	283105
86519.38	14612.29	24279.74	21770.4	19125.24	30520.44	32097.91	39052.61	38100.8	292344.8
83541.51	19413.5	22576.35	19298.1	24610.19	26904.4	24746.93	31151.53	33986.04	388762.5
91300.45	36363.51	24565.13	22354.64	22749.19	32289.4	27468.55	39355.24	32166.09	440461.8
83047.14	27866.31	23996.52	17358.03	21250.11	33386.77	32661.47	41060.44	35295.77	469905.8

LP312.326	LP312.326	LP312.362	LP313.125	LP313.126	LP313.126	LP313.126	LP313.125	LP313.125	LP313.125
924232.8	32944.99	33014.61	20648.2	26186.99	37027.52	20151.95	16637.73	42460.92	38390.55
1049659	35700.63	37810.73	27047.6	30127.5	40774.64	21797.8	24807.41	48926.67	36403.3
1585643	34055.78	60196.16	22513.44	30507.22	35919.59	16959.72	18811.49	46266.51	35965.19
1940098	33446.44	75387.83	20957.4	26905.59	37211.78	19804.55	15131.75	49369.69	33784.69
2128318	35282.29	82406.57	20113.61	25960.82	41162.17	21998.56	21181.11	47048.64	35322.08

LP313.126	LP313.125	LP313.126	LP313.125	LP313.125	LP313.125	LP313.125	LP313.125	LP313.125	LP313.125
42765.22	34223.19	37250.13	34791.81	46069.82	41310.59	24692.53	48038.68	47630.84	38465.96
39438.86	39329.5	43934.91	36410.23	44624.74	48065.98	26426.65	53855.79	50753.17	42516.71
39038.34	28555.01	41471.38	32937.99	51047.25	45805.28	27453.01	48689.5	48518.38	35126.42
36955.49	30808.55	40117.23	30647.87	45571.33	42665.6	22906.55	51044.78	50435.62	36012.16
37864.62	31370.46	40705.08	34211.61	40148.41	47505.93	24358.34	51432.97	50412.5	33510.47

LP313.126	LP313.125	LP313.126	LP313.125	LP313.125	LP313.125	LP313.126	LP313.125	LP313.125	LP313.125
37917.12	39485.88	40086.91	35658.72	44767.41	49386.54	25906.06	25113.3	22232.05	57490.09
42765.99	43809.5	42063.55	36727.46	49278.96	59483.65	17146.23	23063.69	19959.15	56200.13
36862.35	38581.55	41241.02	36027.12	45295.17	53010.46	18809.97	26050.8	28644.87	56763.01
34454.48	32137.03	39307.76	33652.17	44152	61442.3	22519.08	21577.51	22002.39	60140.36
35788.9	39280.17	38001.26	33252.86	43806.36	48903.28	23043.75	24261.8	21652.74	54717.91

LP313.125	LP313.126	LP313.125	LP313.126	LP313.125	LP313.126	LP313.125	LP313.125	LP313.125	LP313.125
25993.11	17103.69	53952.37	48023.71	42352.4	49973.83	36511.21	28320.64	43895.25	84662.29
26789.02	16658.76	56540.65	46358.21	40814.34	50492.4	41789.02	21873.4	49756.07	81363.93
23775	16698.02	52512.33	44574.76	37357.36	49476.53	36359.65	19829.73	44604.29	81930.4
23196.35	16185.81	51360.23	44324.77	38049.76	49830.9	40971.44	19704.79	42207.94	81608.88
26942.09	15956.22	56658.06	42228.32	43658.18	49124.59	38242.37	23118.81	45976.82	78596.25

LP313.126	LP313.125	LP313.126	LP313.126	LP313.125	LP313.125	LP313.125	LP313.125	LP313.126	LP313.125
26136.74	39674.81	30364.66	27095.81	37641.72	63739.39	46760.89	57687.38	23671.44	49331.26
28992.52	52750	31405.71	29056.77	41401	68155.45	52638.58	54991.67	26810.26	48516.09
26106.72	44644.16	28966.18	26834.09	35495.93	58819.21	49122.2	61590.55	20865.23	45400.35
24243.6	44635.81	27059.01	24478.35	30585.21	57100.47	50364	55538.14	22411.96	41181.01
26395.81	44235.99	25789.38	25138.23	31309.6	64679.18	48157.87	51249.47	21306.95	42838.71

LP313.125	LP313.125	LP313.126	LP313.125	LP313.126	LP313.125	LP313.125	LP313.125	LP313.125	LP313.125
45989.8	70298.37	18791.06	53613.21	49512.44	44864.4	36685.86	50051.57	43049.58	34323.49
48659.62	77814.22	21009.91	53570.93	59184.47	50182.51	37825.44	53017.69	48281.63	38281.36
41703.23	83362.88	19857.84	50401.4	58316.59	43524.33	37634.58	56252.89	48403.68	40764.04
42872.84	73971.46	17752.72	52342.75	49413.96	46645.49	30244.62	49555.29	38382.14	36570.33
42709.52	77456.52	17881.83	50462.79	52571.59	49566.21	31590.63	51492.95	40797.21	37205.93

LP313.126	LP313.125	LP313.125	LP313.126	LP313.126	LP313.125	LP313.125	LP313.126	LP313.125	LP313.126
43288.83	30739.07	49493.59	21180.43	54991.16	34020.97	32460.03	15589.46	62507.3	47015.47
43344.19	40453.52	56630.61	20834.23	60402.83	38069.05	34112.23	15173.85	64608.74	52788.25
42066.9	36998.77	45819.77	15394.22	56030.22	38016.77	33556.03	14795.13	60815.12	46557.19
42002.86	31095.77	49192.02	17666.29	53314.79	37324.76	31705.87	10605.1	58124.23	42494.08
40962.17	32872.46	47027.31	19437.43	57767.8	36915.46	34787.43	15286.06	65217.62	43608.61

LP313.125	LP313.125	LP313.126	LP313.125	LP313.125	LP313.125	LP313.126	LP313.125	LP313.143	LP313.154
35415.28	55168.17	33198.38	62440.06	45079.24	36736.62	42706.8	51306.12	27948.85	120520.9
37252.48	56862.21	37394	63038.12	47201.48	40452.11	48023	52532	31593.47	121195.2
33548.63	57329.16	35840.47	68499.79	40674.87	31826.78	45584.32	54722.63	30938.68	137608.2
36753.3	50739.75	32511.27	64381.32	50034.26	38751.85	40632.47	54707.74	32579.88	129330.5
34546.64	53169.65	36067.55	65791.15	44249.32	35553.02	45392.66	48344.77	29866.27	57262.63

LP313.154	LP313.154	LP313.154	LP313.154	LP313.154	LP313.154	LP313.162	LP313.162	LP313.162	LP313.162
102877.4	107251.1	154103.3	104771.3	96651.56	26796.92	124370.2	108889.9	159596.7	118157.8
114376.8	100145.3	152676.7	107126.7	96935.6	126033.6	92119.84	111275.3	106660.3	117760.9
110601.8	105090.8	155093.3	97674.49	96127.97	119969.4	115735.4	172484.2	145065.3	97845.98
108497.1	108291.2	157243.2	103618.8	105211.4	120529.4	120655	124089.5	188097.9	121281.6
109024.1	110953.9	143943.6	101243.1	108703.1	118932.9	122463	132814.7	144917.7	169925.1

LP313.162	LP313.162	LP313.162	LP313.162	LP313.162	LP313.162	LP313.162	LP313.162	LP313.162	LP313.162
130246.9	135042.5	144290.7	121444.6	98297.6	140705.3	118901.2	107664.1	130489.3	137851.2
115275.3	156818	147505.5	126100.3	100101	104352.7	109034.2	104168.4	133720	143565.4
143561.1	145183.2	123437.4	130660.1	98445.74	102726.9	112117.6	105329.7	139516.9	179329.1
121357.9	119820	165494.7	149695.2	108512.3	99824.41	119259.3	95366.38	130506.3	180881.7
147283.3	146659.3	196903.7	130513.5	108358.8	99783.79	125212.4	115960.6	148603.9	126278.6

LP313.162	LP313.162	LP313.162	LP313.162	LP313.162	LP313.162	LP313.162	LP313.162	LP313.162	LP313.162
161475.4	113827.3	110343	128415.8	101070.2	91901.17	119140.3	85715.08	92732.58	115785.2
170160.6	108861.2	98090.18	133010.2	86731.23	89705.6	118965.4	86072.03	88245.77	112360.8
115140.5	151933.8	110502.7	136195.6	103461.4	95839.89	119167	86961.21	91852.78	119661.9
114753.8	155747.8	96889.62	132235.4	171141.5	77348.08	123723.5	92291.73	87562.75	109347.7
108501.2	110036.2	93112.18	139906.3	84495.83	101648.8	128414.7	91065.19	86153.74	118957.9

LP313.162	LP313.162	LP313.162	LP313.162	LP313.162	LP313.162	LP313.162	LP313.162	LP313.162	LP313.162
125444.2	139294.4	85201.4	71124.34	105228.3	85582.29	82882.44	87239.69	91579.46	142768.4
120972	136463.7	90669.73	68259.22	101158.4	86221.29	71303.36	83714.57	86359.12	210855.1
121292.8	129654.9	89885.94	71142.71	104666.1	79282.01	72577.26	82136.5	81099.23	122000.4
120467	136504.2	83217.54	64619.72	104476	81099.1	71273.31	75172.04	85746.14	178457.7
110240.3	131235.4	88273.59	64423.27	95124.38	84961.89	68942.18	84111.25	81639.56	120970.7

LP313.1625	LP313.1624	LP313.1627	LP313.1628	LP313.1626	LP313.1627	LP313.1628	LP313.1627	LP313.1629	LP313.1624
108649.7	132718.4	93602	142714.7	157329.4	168075	114997.5	153757.4	112733.8	109415.1
136912.5	108511.9	96282.7	119734.6	110164.3	148269	96802.9	143928.8	99328.95	105435.2
120296.1	142148.9	146664.2	130927.8	122720.7	116232.1	147686.6	145263.4	103412.9	85604.29
102482.8	115960.2	89158.35	125338.8	138311.8	126377.6	169458.3	175939.9	80693.71	114691.2
98581.05	121830.4	98631.34	127856.9	121935.5	112065.4	126287.6	175315.8	93264.12	113168.2

LP313.162	LP313.162	LP313.162	LP313.162	LP313.162	LP313.162	LP313.162	LP313.162	LP313.162	LP313.162
192795.4	135521.5	134863.6	99782.58	109977.2	123163.8	153682.1	197778.6	162638.3	128407.4
159437.9	107929.5	136381	108110.3	158816.6	124696.1	156938.2	104711.2	132705.7	127945
141395.2	140588.5	145829.6	110850.7	109404.9	117885.4	155928.4	144130.2	152845.9	121984.4
163630.9	147255.5	132874.2	146059.6	103665.7	124971.7	149679.3	129336.4	170914.5	126111.7
148780.3	148454	130349.8	92498.72	141723.4	113077.7	156176	156566.1	142226.1	125691.6

LP313.162	LP313.162	LP313.162	LP313.162	LP313.162	LP313.162	LP313.163	LP313.162	LP313.162	LP313.163
124861.5	120150.4	153576	130240.9	114901.4	137422.4	154582	157359.2	129723.9	148309.8
120132.5	135639	120935.1	131579.4	175288.6	108531.6	152396.3	167401.3	126279.8	158634.3
135118.2	123770.1	162857.5	113820.3	128918	120578.9	156370.8	141648.7	125130.5	145595.2
108740	115407.6	220259.8	163140.3	129559.3	122921.1	143965.2	154065.4	131638.5	156387.2
143398.8	126456.3	112603.6	142860.3	139865.2	124192.8	141611.1	156244.4	129784.3	148345.6

LP313.162	LP313.162	LP313.163	LP313.200	LP313.200	LP313.201	LP313.201	LP313.200	LP313.204	LP313.237
102530.1	85158.15	90481.39	134168.9	118245.9	107419.3	86686.25	91606.17	114462.9	110409.8
107542	95256.96	94559.09	141309.8	117574	109524.2	91332.11	104913.1	113292.9	105970.6
96559.75	88219.01	90949.82	138078.6	107884.9	102001.4	88336.3	92932.42	106742.9	99647.35
100206.2	81744.63	89680.16	122499.1	106400.5	102851.1	85713.78	86802.48	100557.6	98769.73
104581.2	83585.75	86413.38	123443	105439.1	108280.4	81482.81	85836.76	98875.58	94768.89

LP313.237	LP313.237	LP313.237	LP313.273	LP313.273	LP313.273	LP313.273	LP313.273	LP313.274	LP313.273
112493.8	131953.6	89427.45	1201943	701662.6	804462.6	235412.6	134705.5	471854.8	440192.3
112812.6	113017.2	98979.05	1240203	784757.2	804873.9	231934.5	148720.8	482516.9	396183.3
117345	117378.8	86643.61	1521554	977285.9	959809.5	242439.1	131412.7	473228.8	430349.7
115819.8	111596.3	86265.33	1277573	1095807	925266.5	231059.2	127510	443079.5	408882.8
112233	120260.2	78814.3	896916.5	1171522	593671	251371.7	117067.1	411371.9	403037.4

LP313.273[illegible]	LP313.274[illegible]	LP313.273[illegible]	LP313.273[illegible]	LP313.273[illegible]	LP313.274[illegible]	LP313.273[illegible]	LP313.274[illegible]	LP313.273[illegible]	LP313.273[illegible]
89303.97	215870.5	250949.7	740120.1	138585.1	210588.7	254901.7	251285.1	210293.6	348753.4
97971.94	222884.4	251030.9	722151	141309.8	197113.8	247271.9	240086.7	187099.9	361649.4
95006.15	224212.7	244480.5	745187.2	146387.6	208835.6	260041.3	254624.5	212145.6	359559.9
86367.97	208759.5	228966.5	741225.3	147793.2	181307.5	248959.2	244619.3	183775.2	326894.8
87291.96	209273.5	223336.9	749007.3	131915.8	175514.9	242804.4	222188.6	212161.2	302985.1

LP313.273	LP313.273	LP313.273	LP313.273	LP313.274	LP313.273	LP313.273	LP313.274	LP313.273	LP313.273
276365.8	87541.1	146290	211917.8	242179.1	237562.8	375363.1	239971.7	159950	279025.2
264107.2	86615.59	155867.4	206066.2	243443.2	231454	381542.1	254483.6	169583.5	248259.2
252665	97699.54	155069.5	198504.9	242854.3	237233.8	388397.8	275441	193622.5	273663.1
233240.7	87724.09	165551.4	190403.1	255866.5	234565	356566.7	242111.9	153770.9	257329.9
226173.7	86991.44	160197.1	183731.1	226129	218792.4	345781.1	236493.4	153543.4	253308.8

LP313.274	LP313.274	LP313.273	LP313.274	LP313.273	LP313.274	LP313.274	LP313.329	LP314.180	LP314.276
459521.4	261809.4	100970.2	252498.5	112987.8	139356.2	237822.5	73598.93	16323.61	233328.3
449346	274158.9	97044.86	249355	123218.9	141736.3	251458.8	201985.9	15293.25	182587.4
469150.8	284636.2	108590.8	250782.1	119974.6	131975.9	261772.5	88565.42	15984.47	173532.6
457069.5	253486.6	109491.8	245613	113753	129117.6	244458.7	233169.2	14918.2	209949.6
479266.2	247309.8	98562.38	231711.4	92926.57	139221.7	228278	109602.4	14470.13	191684.2

LP314.277	LP314.332	LP314.332	LP314.341	LP315.142	LP315.142	LP315.142	LP315.142	LP315.142	LP315.142
138832.8	20358.02	20140.75	198508.8	44694.54	29578.81	36845.47	40733.01	43793.79	39926.43
148394.4	21008.53	19815.42	215947.2	44804.14	28089.68	35848.62	42415.6	47150.99	42752.71
164900.4	21280.39	21322.28	265184.4	54729.39	30319.79	36373.07	44140.75	47624.2	36369.88
142311.5	23506.72	19341.75	295906.2	41778.14	36795.84	38837.75	43204.59	54796.34	42205.38
150920.4	22359.72	21964.36	317543.9	49258.29	32894.01	36931.08	36902.53	39462.06	39802.34

LP315.195	LP315.253	LP315.289	LP315.325	LP315.337	LP315.768	LP316.256	LP316.271	LP316.273	LP316.284
15558.98	43922.84	58689.2	20357.49	22317.69	6734.531	17018.57	31669.35	36452.34	224830.9
13943.4	41743.1	65366.51	20572.7	22607.88	7487.562	19511.57	27318.38	38997.32	193475
9882.3	51916.61	68264.91	14941.59	20206.29	11679.08	18041.39	25657.64	40051.83	195000
13754.44	55982.93	78188.35	20822.4	26688.89	14133.26	17501.47	28918.34	42088.93	172359.5
14378.98	61046.19	81907.77	21054.01	23763.63	15695.59	18782.38	25512.88	43616.21	198433.4

LP316.284	LP316.284	LP316.292	LP316.348	LP317.115	LP317.114	LP317.115	LP317.114	LP317.115	LP317.115
192558	34931.9	33049.18	21492.4	72083.85	141879.5	187271.9	123350.5	54911.77	168758.1
170733	30355.65	32000.66	23619.25	88308.43	196275.1	122253.6	159737.4	90135.24	184762.4
141938.1	35400	32827.12	21632.3	70553.2	139077.5	106696	153242.4	81322.42	244734.6
208949.3	38184.5	30105.52	20911.46	84939.57	94333.15	163094.2	211343.7	67411.63	136993.6
165304.4	37918.08	32231.64	21376.54	62416.67	148172.3	116525.5	208961.2	83500.29	184037.7

LP317.115	LP317.115	LP317.114	LP317.115	LP317.114	LP317.115	LP317.114	LP317.115	LP317.114	LP317.115
159243.7	122735.6	156796.8	183199.2	90984.76	157771.4	114885.4	196236.1	64900.32	128203.6
184305.6	101426.3	150700.7	178323.3	113726.4	149746.5	134078.9	221423.5	139792.2	73609.97
153455.8	105328.4	95993.91	189635.8	108931.4	210894.8	120647.8	128926.8	92921.5	79328.46
133405.6	91447.58	219692.3	190482.4	64925.64	147613.1	114744	115006.8	78648.32	67855.94
135647.4	122361.5	169957.6	140619.1	98569.41	121714.4	193837.4	128340	124337	101221.3

LP317.115	LP317.114	LP317.114	LP317.115	LP317.115	LP317.115	LP317.115	LP317.114	LP317.115	LP317.114
52733.36	237195.6	95728.36	136899.8	181538.1	148842.2	165247.5	209219.3	145328.5	148293.9
64477.28	190760.3	141631	108658.1	132909.3	106256.7	221729.9	173773.5	235986	138537.8
41539.72	130821.5	132948.1	98622.47	196725.9	83002.41	218650.7	174230.1	133304.3	177637.6
39942.22	163966.2	152754.7	127401.2	237287.2	129990.4	186813.9	175706.2	182607.7	168668.5
38028.43	174725.4	100915.7	82301.81	166819.7	124019.2	181470.2	187569.1	179858	207426.4

LP317.115	LP317.115	LP317.115	LP317.114	LP317.115	LP317.115	LP317.115	LP317.114	LP317.115	LP317.115
169492.6	141298.8	163629.4	138599.6	216491.1	163158	113967.4	100797.1	115152.7	101043.8
176438.5	155846	124894.9	153215	190679.1	206694.6	120932.6	132066.5	180265	85250.47
140974.9	171633.3	147541.5	216284.8	205932.1	175033.9	161959.2	112350.2	190342.5	123817.1
151006	206283.7	147244.7	191085	185561.1	167859	109982.3	122023.9	142415.9	92196.8
183685.1	157969.9	134651.4	175351.1	212705.7	240133.5	188941.8	87177.82	188679.6	119467.4

LP317.115	LP317.115	LP317.114	LP317.114	LP317.115	LP317.114	LP317.115	LP317.115	LP317.115	LP317.115
169080.4	175031	71315.08	68293.98	130785.7	147891.2	111723.4	289238.9	188597.4	168044.4
124000	292372.5	53459.94	72624.78	134302.4	138246.6	102222.3	189980.4	192459.2	101685.7
146314.4	201064.8	62628.39	90055.72	114197.7	107103	144454.7	183210.4	170604.6	136082
154311.9	153042.3	38884.16	67260.24	101930.6	204533.5	102700.8	222400.7	142444.6	116424.9
125838.2	174357.7	50225.62	70924.76	152939	164296.9	114275.5	180876.1	202304.1	144441.5

LP317.115	LP317.115	LP317.114	LP317.115	LP317.115	LP317.115	LP317.115	LP317.115	LP317.115	LP317.115
142093.8	180498	97088.01	162101.4	157960.2	218613.3	143395.2	152959.6	36719.24	141833.5
148933	257128.9	84518.06	226521.8	126995.2	135157.2	139424	164975.7	50439.6	159882.4
107445.4	210202.2	124225.2	199674.6	112157.5	191249.9	112195.3	128713.7	60120.89	186257.9
159890	170853.8	106138.9	169772.6	126901.9	178623.8	141194.1	154196.7	59037.17	181215.2
176711.2	182730	106190	199682.9	118344.2	194020.2	177816.7	135561	47240.96	258724.2

LP317.115	LP317.115	LP317.115	LP317.115	LP317.115	LP317.115	LP317.115	LP317.115	LP317.115	LP317.115
118796.8	137858.4	44372.09	92698.63	186972.5	243213.7	118998.9	40869.32	57586.55	39546.59
119607.4	141153.9	53598.63	106753.1	174828.8	153411.9	158713.1	60397.89	46482.71	41906.19
162699.2	128421.4	58014.42	134150.8	219316.2	168428.6	115769	46041.59	31603.58	31079.08
99743.97	166470.4	64526.8	165534.2	169415.9	167320.3	184496.4	47917.19	55428.54	32062.47
190164.2	131240.8	61661.74	93477.4	172066.1	178557.5	134653.9	56530.8	54007.46	35498.5

LP317.115	LP317.115	LP317.115	LP317.115	LP317.115	LP317.115	LP317.115	LP317.115	LP317.115	LP317.115
45439.13	159496	149337.1	174454.9	70709.79	232206.5	118067.1	44261.49	216202.4	78476.1
54475.63	189088.9	99654.95	170945.7	44978.31	176737.1	175452.1	43157.07	210824.5	77535.38
56743.13	178732.6	121852.2	251766.1	49589.08	173651.1	141974.7	33372.16	128966.7	55721.64
58353	128411	119349.4	220763.8	62841.05	193663.7	123143.1	37725.76	227322.5	59019.77
50712.23	141804.2	146778.2	154659.5	61096.01	166604	165593.5	38903.96	178616.5	80973.24

LP317.115	LP317.115	LP317.115	LP317.115	LP317.115	LP317.115	LP317.115	LP317.114	LP317.196	LP317.196
96332.22	161594.3	158209.8	173984.2	143109.9	199399.6	156225.1	208273	15949.13	12203.33
124509.6	161656.7	195956	125175.6	153649.5	156194.9	157025.3	168200.9	13456.03	13603.88
94694.67	162011.5	208902.6	166488.6	183514.4	201209.1	145668.2	162760.7	16026.01	14367.59
147526.2	149367.6	191748.7	192572.1	191755.2	173597.6	191493.7	164223.8	13736.59	12084.15
110701.5	175853.6	206291.3	190188.9	179298.4	181234.6	195116.5	144125.5	17079.09	11970.01

LP317.248	LP317.288	LP317.288	LP317.305	LP318.118	LP318.118	LP318.118	LP318.227	LP318.227	LP318.227
19872.22	32525.04	22046.06	12359.63	8257.008	28259.79	29471.01	96167.16	82055.27	106589.7
20649.36	41985.75	37545.69	11195.21	11353.21	33735.98	31035.47	93879.08	73191.6	99395.32
19672.56	44601.71	35238.74	11715.88	10740.32	25510.39	32716.79	92166.34	56733.76	83249.64
19146.81	26889.23	36060.66	4759.807	10632.3	33732.33	25822.94	95976.57	82510.41	108813.7
16890.93	32526.2	28977.66	10158.56	8252.269	35863.32	33744.2	91833.75	86676.41	103692.2

LP318.227	LP318.227	LP318.227	LP318.227	LP318.227	LP318.227	LP318.227	LP318.227	LP318.227	LP318.227
124446.5	78274.46	77857.72	78233	76310.59	111701.1	86735.76	111834.3	92084.31	70404.03
134134.6	85313.87	73035.17	76038.01	76827.77	102661	90942.2	109791.7	93993.23	65845.34
126765.6	54897.93	72194.85	50727.92	45627.37	109227.9	65700.13	77196.03	61720.01	40502.04
131854.9	77646.64	79755.11	85136.78	81170.27	115229.3	93731.42	115954.7	102695	62108.02
131395.9	87965.38	78715.04	84373.62	78652.6	103613.8	89921.52	114987.9	92225.21	65941.92

LP318.227[illegible]	LP318.227[illegible]	LP318.227[illegible]	LP318.227[illegible]	LP318.227[illegible]	LP318.227[illegible]	LP318.227[illegible]	LP318.227[illegible]	LP318.227[illegible]	LP318.228[illegible]
70995.36	88255.95	94012.36	102560.9	94297.68	93374.88	68673.77	75587.89	127266.3	91306.16
72254.19	86025.81	103909.9	102404.6	94026.01	86209.85	65524.71	66264.68	122632.3	90347.48
44858.35	47393.75	67030.11	64468.37	64953.05	97776.77	49192.95	44712.34	76433.48	99040.02
66562.64	87410.12	104871.2	99980.57	98365.42	99078.68	65838.17	70501.23	125592.1	101159.2
65071.39	91409.2	98477.86	100682.4	77129.77	92846.52	65375.38	79418.44	127204.8	101711.4

LP318.227	LP318.227	LP318.243	LP318.300	LP319.190	LP319.190	LP319.190	LP319.284	LP319.299	LP320.170
114159.2	96974.31	80829.73	26363.12	17542.6	10470.3	13290.06	103625	35649.26	71942.47
115753.6	91757.94	79873.56	28862.25	18134.74	7754.867	12649.01	96986.71	31854.8	74264.94
74352.32	70053.49	89944.51	33378.17	24687.91	9104.869	10303.72	110171.1	32945.65	63980.11
110212.4	93386.55	97460.53	30063.35	24212.86	8452.897	11838.93	105936.6	27539.76	74915.83
107626.9	95546.14	100062.7	21990.45	26916.39	10372.16	12263.62	104335.4	34740.27	77682.94

LP320.170	LP320.170	LP320.170	LP320.2_1.	LP320.206	LP320.206	LP320.206	LP320.206	LP320.206	LP320.206
63016.73	55770.02	91045.84	54391.38	92754.71	93799.96	82006.82	86957.54	76153.22	106115
66293.26	60148.62	93408.92	57091.22	77218.28	95274.63	92571.74	99402.8	70850.87	115337.1
70112.93	59308.61	93530.42	57911.71	75659.77	85834.18	77527.4	103252.6	72266.03	105930.8
71325.53	56941.05	97058.98	55241.98	79090.91	87402.93	88537.85	97602.82	71949.77	110398.1
77257.52	54860.52	83926.85	50278	81084.34	78657.87	84983.73	93557.39	78548.05	92825.5

LP320.206	LP320.207	LP320.206	LP320.206	LP320.206	LP320.206	LP320.207	LP320.207	LP320.207	LP320.207
115574.8	65720.07	69547.1	84599.08	128531.8	103036	88071.72	79601.46	91625.11	80257.89
108241.1	61127.4	60984.18	93888	123080.9	97383.15	92522.47	79047.04	96459.49	71439.44
103895.3	64149.45	65873.37	83275.29	126283.5	94615.62	92181.15	71135.12	81879.2	75250.9
104963.5	70577.19	69374.86	79972.14	142333.6	105815.9	87289.32	76659.91	91856.68	76926.26
113998.8	65441.61	61050.71	76960.57	124822.3	92184.62	96353.02	87140.91	89378.27	80310.34

LP320.207	LP320.206	LP320.206	LP320.207	LP320.207	LP320.292	LP320.288	LP320.316	LP321.204	LP321.279
99268.72	71239.32	160261.4	83793.51	71312.41	23138.82	15960.34	25611.76	34920.31	30255.82
101175.9	68054.7	144116.6	76980.77	67083.14	21264.09	18128.21	21296.01	33243.27	33638.55
101238.3	80459.48	143552.1	79236.99	71016.73	34626.07	17799.48	25155.93	32149.51	33286.86
110527.1	77037.48	150751.8	73070.64	71238.57	41050.78	14623.84	28774.32	32576.41	33775.49
106760.4	75256.97	144202.7	76536.26	81606.66	45797.07	17479.67	22387.9	30450.95	32290.7

LP321.279	LP321.278	LP321.296	LP321.315	LP321.326	LP322.186	LP322.185	LP322.186	LP322.222	LP322.222
33664.83	24614.96	38560.95	244621.6	28479.1	83219.57	111014	73949.68	69535.07	63832.35
35766.15	28973.34	38316.49	206682	27746.17	76612.84	108962.1	70599.59	71043.19	68255.6
31317.81	30692.06	34608.06	221322.1	31547.66	81690.85	110205.1	64626.36	67942.47	68661.67
35015.38	24862.38	34289.06	173351.4	31874.31	84781.74	118739	68971.32	67204.53	67921.09
29916.08	23280.96	35223.83	225695.5	29320.97	79730.04	110471	72850.73	62823.27	64746.5

LP322.222	LP322.222	LP322.222	LP322.222	LP322.237	LP323.146	LP323.146	LP323.146	LP323.146	LP323.146
105925.3	75319.15	119210.1	117019.9	30688.96	40549.03	54425.94	45425.9	45862.81	63284.9
104770.8	63388.26	117076.1	105035.3	32342.25	32138.51	39977.75	61484.74	95152.89	42511.6
101421.4	59564.92	120145.3	123673.6	38057.73	39481.23	61502.3	55519.63	53372.91	68698.37
106304.6	68755.58	128491	118259.6	35907.61	43131	48591.64	44559.52	46375.33	53289.55
109124.6	71795.97	129320	111477.8	39881.73	46032.42	53401.34	49242.53	46633.82	53996.79

LP323.146	LP323.147	LP323.146	LP323.147	LP323.146	LP323.146	LP323.146	LP323.146	LP323.146	LP323.146
61971.41	32172.15	54433.66	38263.66	54115.46	73747.6	38443.46	63986.84	47520.53	54933.11
47437.14	51301.1	50895.05	50107.68	56722.33	73659.95	50232.72	70520.12	59896.04	54986.09
55835.35	46557.38	62119.42	41654.34	64180.21	104522.8	51522.82	85962.71	66683.3	78231.35
54681.64	32540.74	50923.92	42875.52	47437.36	69717.58	35649.78	62755.37	46096.55	54740.57
56153.69	36407.92	47121.08	36316.11	55353.84	63906.8	35367.37	59397.03	43717.99	49602.47

LP323.1468	LP323.1468	LP323.1468	LP323.1469	LP323.1469	LP323.1469	LP323.1469	LP323.1469	LP323.1469	LP323.1469
57968.93	60611.46	46768.36	50654.85	51101.53	60944.68	50114.4	54732.83	45309.48	45411.79
38780.13	71321.48	62847.51	83047.66	50587.66	62917.1	62547.87	55644.48	50008.98	38449.94
68371.97	48091.84	61845.34	46878.04	41404.58	80586.03	56424.96	78465.91	36710.33	60429.54
62417.98	70845.78	40860.4	44865.49	47356.12	58503.46	53389.78	52797.25	42980	46092.94
61067.6	75639	40255.65	46644.36	40097.29	67745.43	41857.7	54276.1	41290.54	42870.03

LP323.146	LP323.147	LP323.147	LP323.146	LP323.146	LP323.146	LP323.146	LP323.146	LP323.146	LP323.146
50756.45	44917.57	52897.18	55539.39	49271.07	34185.6	57149.42	70350.85	48685.93	60272.11
67369.12	69610.84	67870.03	48770.64	63387.74	39911.76	52415	69728.57	51481.97	56507.61
63039.88	48586.67	39982.26	54144.26	48261.86	36651.6	63632.21	46236.78	48672.86	51789.11
59450.2	45516.09	54556.53	64218.11	48561.55	38453.27	52850.54	65443.27	45979.41	64271.41
54857.34	40778.02	47008.34	58407.17	50719.86	37760.32	58530.58	61169.08	41098.37	59929.41

LP323.146	LP323.146	LP323.146	LP323.147	LP323.146	LP323.146	LP323.146	LP323.146	LP323.146	LP323.146
58920.18	58343.55	55539.69	47662.43	64218.72	42223.64	39769.64	44926.83	58675.61	50873.82
63228.37	59638.59	85004.89	75221.55	79455.83	48146.37	51618.28	61466.49	53489.77	47217.13
48016.81	88895.57	64462.15	41092.38	60997.48	80949.48	64434.62	80366.08	52812.56	51971.28
60223.23	53197.12	57460.35	48304.29	64419.09	41270	41470.67	43652.71	57309.81	54067.62
60284.88	54963.43	55743.79	42330.46	57547.43	39580.07	40161.01	39296.3	57464.51	51127.4

LP323.147	LP323.146	LP323.146	LP323.147	LP323.146	LP323.146	LP323.146	LP323.146	LP323.146	LP323.146
50842.9	47688.21	42552.89	43390.58	48172.88	51700.63	62014.59	44344.7	53543.3	54440.48
54967.55	49491.51	56787	52396.1	56358.93	56055.17	65077.99	43701.49	49055.63	57232.66
61771.71	50190.17	69274.14	85714.77	40821.84	48882.14	53462.17	53436.84	54617.21	44565.89
44930.81	41630.16	35201.18	40765.74	49825.26	54746.12	51979.84	39402.96	46543.84	46678.85
53114.06	47022.13	47021.51	42112.24	41640.81	50738.71	52382.42	36396.56	46023.82	51917.2

LP323.146	LP323.146	LP323.146	LP323.147	LP323.146	LP323.147	LP323.147	LP323.146	LP323.146	LP323.146
58799.77	37935.05	56315.12	37085.44	59196.23	48228.89	55784.5	58719.61	48409.27	44716.51
65310.21	33891.01	55646.68	43341.11	56287.98	44108.88	58549.58	59940.04	49546.08	38334.3
51595.22	47177.11	61633.66	38917.52	89467.3	65590.6	67231.48	45327.72	60488.66	48835.04
55172.4	37227.61	48870.93	42136.89	52525.16	46762.74	54778.39	66436.96	50882.71	46818.78
56415.67	35017.38	51318.72	39632.3	51852.86	44748.33	56883.11	58124.41	44336.11	38304.31

LP323.146	LP323.146	LP323.146	LP323.147	LP323.146	LP323.146	LP323.146	LP323.147	LP323.146	LP323.147
47937.56	39277.43	67254.01	53339.7	33828.41	62918.08	65618.31	51181.86	43531.26	31995.17
80261.44	31526.21	71896.83	45418.65	52082.96	66956.74	61173.94	62405.32	55527.85	39112.84
65920.57	40707.55	71221.92	58063.15	34589.88	85807.08	57621.97	51780.15	68820.07	25574.7
48106.84	37803.13	53041.43	45902.15	42662.23	58852.84	67746.92	57028.84	38142.29	36502.69
47152.74	34292.45	57123.84	55582.41	31802.6	63940.05	65599.54	55344.79	43113.63	30382.93

LP323.146	LP323.147	LP323.146	LP323.146	LP323.146	LP323.147	LP323.147	LP323.146	LP323.146	LP323.146
57621.14	49053.17	63247.1	58110.89	37046.87	34080.6	35314.93	59686.18	36403	51985.28
54493.1	49815.3	71236.84	62039.32	39674.76	45455.68	24682.55	61425.18	50294.32	60182.79
46330.91	76761.64	48944.31	97619.82	39862.06	35830.41	37153.23	63916.94	29397.42	51074.33
64399.51	50153.93	57300.43	53530.91	37916.31	35122.22	37552.83	66190.67	39514.04	56684.39
59679.4	51075.94	56138.26	54822.76	34239.94	29795.32	33332.78	65447.73	34469.18	53903.04

LP323.184	LP323.184	LP323.184	LP323.184	LP323.184	LP323.184	LP323.184	LP323.184	LP323.184	LP323.185
42180	43568.58	44877.87	58229.66	44622.64	40711.7	41525.78	60467.64	50003.75	68067.97
35963.87	42939.64	41630.5	49188.7	40852.63	41511.5	45338.15	61345.37	45822.21	70189.77
40808.99	47151.84	42202.82	54111.61	44037.68	45077.01	41497.41	58759.65	51889.15	75058.27
38117.53	45438.22	40707.57	52284.52	46231.63	42538.95	40717.51	53623.52	50397.04	68460.63
36743.86	46991.37	42219.56	52208.72	44533.56	40775.98	46559.17	58320.69	47369.67	67588.93

LP323.184	LP323.184	LP323.185	LP323.184	LP323.184	LP323.184	LP323.184	LP323.184	LP323.185	LP323.185
51386.97	44358.48	47366.53	42473.53	53801.97	38173.2	38182.5	35308.77	39179.28	67018.42
51924.47	45200.93	52361.59	46966.76	51688.45	38223.81	38796.95	33169.22	38943.81	69734.89
52008.62	43262.55	48784.94	47809.76	51139.35	37352.61	37853.31	33828.19	39096.51	66798.8
57552.3	45568.44	48658.9	43308.71	48830.23	37278.55	39827.97	32976.92	40951.28	65279.47
54081.6	45477.58	51969.43	49368.09	53409.74	38495.6	35928.02	37053.34	40878.58	66210.25

LP323.196	LP323.221	LP323.225	LP323.241	LP323.257	LP323.257	LP323.257	LP323.257	LP323.294	LP323.294
29854.74	50803.7	79925.56	21310.47	74785	79523.58	80162.58	55157.65	87902.77	27453.39
31632.34	51287.43	74617	20449.72	65860.7	71838.54	61368.8	39334.65	92695.68	30882.33
29288.73	48517.32	74711.73	18672.3	87495.89	78122.84	80013.65	57608.42	90934.3	28364.91
30130.95	50375.67	58210.72	21157.57	83506.81	73083.85	77942.86	52451.49	85885.12	25983.57
30688.15	53607.45	69742.05	17351.16	84246.41	68411.27	78730.73	60469.2	89842.39	32087.28

LP323.294	LP323.294	LP323.342	LP324.202	LP324.202	LP324.202	LP324.202	LP324.201	LP324.202	LP324.201
67846.8	36665.97	52507.77	118759.8	252761.8	580109.1	451752.3	94332.24	130489.9	612204.4
62204.49	33334.56	50896.39	620779.3	940588.5	518690.3	496871.5	529045.5	247511	561526.2
62909.18	42069.03	47264.21	714603.7	876282.2	484881.6	478778.7	555274	232549.7	634146.6
61382.93	29290.58	46945.42	87342.07	873509	579614.5	426553.8	629289.7	261929.2	680094.2
64734.12	33096.67	42960.19	638376.2	799852	507440.2	452083.2	533219.2	268134.1	633705.2

LP324.202	LP324.202	LP324.202	LP324.202	LP324.202	LP324.202	LP324.202	LP324.202	LP324.202	LP324.202
112266.2	483076.3	112876.5	475294.8	202705.2	656003.8	397882.6	92590.91	128924.9	153650.4
444149.2	469408.6	479520.7	497745.5	192989.8	664540.3	431949.9	942733.2	1015485	404869.5
481233	530704.2	482586.5	496172.3	193108.7	655997.4	430505.6	895343.1	1134149	371752.7
435192.8	494638	490879.5	450934.4	210885.6	679312.1	428824.4	897814.1	207871.3	402655.1
481136.5	536655.5	486813.7	462926.1	196985.4	678944.2	451658.7	854416.4	213341.7	363159.5

LP324.202	LP324.202	LP324.203	LP324.203	LP324.203	LP324.217	LP324.326	LP324.326	LP324.326	LP324.326
147113.6	183507	69618.28	435355	773927	1334220	126176.3	94812.63	88869.01	106007.4
851035.1	782021.6	551817.6	433612.7	755590.5	1338089	118469.6	87640.72	84381.96	91547.78
880490.6	807735.5	577267.7	412808.3	786522.2	1364074	119137	102199.7	85628.31	109627.8
879415	862229.6	549507.1	423690.1	760405.3	1374457	110476.8	87156.21	85009.28	93344.56
824522.8	783907.1	554018.9	423848.8	792624.5	1377062	116607.1	112961.5	68315.23	115100.7

LP324.326	LP324.326	LP324.326	LP324.326	LP324.326	LP324.326	LP324.326	LP324.326	LP324.326	LP325.098
116112.2	100711.2	84583.44	97683.66	95322.42	94500.27	75224.64	92191.18	69892.2	32230.76
109211.1	96230.06	91606.94	97127.43	91055.36	96938.43	70713	103612.5	77337.91	28738.16
110799.5	96010.16	83291.64	98442.66	100806.9	94948.81	70929.16	93492.32	76574.1	33332.23
112875.4	100516.9	85448.21	101339	91332.19	92215.84	69059.92	93878.85	67436.9	31565.1
115217.3	97853.27	83059.02	106365.5	101277.4	94906.53	66418.75	89561.4	69869.78	28510.34

LP325.162	LP325.162	LP325.162	LP325.162	LP325.162	LP325.162	LP325.162	LP325.162	LP325.162	LP325.162
88307.97	92224.6	121407.5	83814.92	73901.16	151175.4	88585.09	87439.07	122092.4	110484.9
83598.49	124970.1	71905.98	129473.1	191906.4	162235.4	132847.7	91843.95	90010.9	118047.4
68204.56	131522.6	67604.97	105347.5	139335.7	160535.6	110049.4	164887.5	123740.1	154262.6
82898.68	83565.03	118633.2	76937	150629.5	126058.9	79393.46	94985.91	101176.3	180493.8
82077.35	100061.2	94208.28	117007.8	124847.1	125457.2	115031.6	88631.89	72640.31	94280.52

LP325.162	LP325.162	LP325.162	LP325.162	LP325.162	LP325.162	LP325.162	LP325.162	LP325.162	LP325.162
89384.36	63026.32	85677.55	108518.6	89343.75	119891.9	75727.18	72659.82	113648	79140.29
107763.7	82633.13	60972.59	94687.02	98606.62	110364.4	134422.1	62762.44	78468.23	73731.6
91091.02	95558.06	64693.95	119511	98324.35	209748.9	120641.7	118668.1	91434.19	80232.11
99400.04	111742.9	53683.98	131540.9	102131.6	117897.2	116419.1	114932.3	102228.2	103872.9
96807.17	63795.18	72371.61	117945.5	103100.9	129109.5	92914.33	91838.83	126975.4	87650.43

LP325.162[illegible]	LP325.162[illegible]	LP325.162[illegible]	LP325.162[illegible]	LP325.162[illegible]	LP325.162[illegible]	LP325.162[illegible]	LP325.162[illegible]	LP325.162[illegible]	LP325.162[illegible]
84822.49	78612.86	151987.4	66544.22	101036.2	125111.4	158473.1	85570.72	132987.9	104020.5
103472.8	60332.18	97288.29	122643.9	100086.8	112425.3	92870.61	81112.96	101844.2	137455.2
81648.26	113811.9	89357.44	58932.92	84216.59	78843.6	114329.8	77789.02	80806.91	139699.4
100817.4	100683.2	109100.4	113764.4	85712.32	143923.6	119515.9	120663.6	125227.8	82275.32
75875.51	77386.36	85122.12	48646.56	108947.9	80040.36	96898.96	70014.46	153676.3	67303.26

LP325.162	LP325.162	LP325.162	LP325.162	LP325.162	LP325.162	LP325.162	LP325.162	LP325.162	LP325.162
95594.19	119673.8	101167.9	135708.9	156656.2	156880.2	76368.49	73809.28	123010.7	89299.83
109482.6	123357.7	101214.6	86229.54	90387	165769.5	70374.11	143513.9	137476.7	84623.02
78518.02	114350.5	95294.91	106622.3	124794.7	178185	115776.5	108141	100941.2	73377.15
98478.31	119468.7	81543.69	135082.5	147233.8	114355.4	87710.16	79383.7	115678.2	129314.8
91775.03	114434.2	169393.8	103202.5	97231.87	123442.8	83180.48	113922.7	104111.3	71240.18

LP325.162[illegible]	LP325.162[illegible]	LP325.162[illegible]	LP325.162[illegible]	LP325.162[illegible]	LP325.162[illegible]	LP325.162[illegible]	LP325.162[illegible]	LP325.162[illegible]	LP325.162[illegible]
89741.18	130501.8	126303.9	89633.06	138458.7	91136.72	124895.2	111914.6	77836.04	53175.62
100088.7	111522.7	106775.1	145070.6	134788.1	137218.4	75645.85	75678.26	77320.9	78647.77
118246.4	91388.02	91538.04	80267.16	85203.92	73236.69	78377.81	119854.2	69768.39	87870.19
97820.52	91546.27	121597.8	93104.43	115037.8	123374.2	107242	118171.8	61485.76	87431.58
82220.14	96701.47	118086.8	112326.7	86238.14	93593.79	99790.97	93456.94	69705.84	59432.32

LP325.162	LP325.162	LP325.162	LP325.162	LP325.162	LP325.162	LP325.162	LP325.162	LP325.162	LP325.162
98115.17	113062.4	126635.3	125140.7	111987.4	147221.5	154106.3	133851.6	105906.5	155762.7
99141.69	134032.7	104837	106526.8	106672.1	77108.9	108725.9	136851.9	84137.16	156775.1
81758.33	80655.65	132251.9	87919.16	103486.8	83930.16	98098.1	111937.7	177498	70797.04
85703.18	112659.9	114312.5	112917.6	83283.44	61914.01	106324.6	109912	100094.4	98185.82
105079.6	134936	112666.3	93387.98	82068.49	146771.4	170655.7	163397.3	108742.1	76082.63

LP325.162	LP325.162	LP325.162	LP325.162	LP325.162	LP325.162	LP325.162	LP325.162	LP325.162	LP325.162
117036.2	80355.93	102174.3	92141.37	62441.1	85630.36	127174.5	120305.7	72481.34	106357.2
149062.7	119872.6	111552.5	104288	97301.87	131347.6	140337	130964.8	161273.7	82855.46
145966.6	94347.93	93381.68	121633.1	104925.5	98886.45	84024.19	125089.2	89774.8	141463.6
109165.6	140963.4	110920.3	73060.3	92717.32	85147.46	68652.76	139537.8	71669.94	148973.7
105930.2	76833.73	101524.5	125026	114722.5	98675.02	159777	126149.7	77711.2	97456.12

LP325.162[illegible]	LP325.162[illegible]	LP325.162[illegible]	LP325.162[illegible]	LP325.162[illegible]	LP325.162[illegible]	LP325.162[illegible]	LP325.162[illegible]	LP325.162[illegible]	LP325.162[illegible]
130552.3	95735.48	125317.3	216089.2	173316.2	154605.3	51490.95	88096.14	39428.57	85361.86
74010.91	56933.95	83741.15	215846.9	180331.8	159541.9	68719.21	127589	117795.9	122875.6
116653.9	47714.8	139975.5	232278.9	135913.7	69611.72	120259.9	120421.1	64900.97	150912.7
145629.8	54857.69	83617.04	70769.76	105420.6	95531.6	68829.84	49406.17	133539.1	159811.6
109074.1	86985.73	87929.71	226426.5	135819.7	116204.9	63122.23	114150	130587.2	152756.1

LP325.163	LP325.162	LP325.163	LP325.200	LP325.200	LP325.200	LP325.201	LP325.220	LP325.220	LP325.220
146071.3	103934.1	208379.5	113882	78404.06	94417.18	78232.13	203259.2	139801.1	134588.9
147348	101618.7	202794.2	119617	77332.54	100617.1	76060.45	298982.4	126070.3	133078.7
140092.6	100607.7	211127.8	116933.8	90795.52	96500.03	77159.98	256793.4	182630.1	177443.1
132765.5	101653.2	210284.4	112738.9	79580.68	102076.9	70686.77	282350.4	133340.7	135912.3
132668.6	107143	190914.5	109890.4	79253.19	96871.58	77501.15	151682.4	134961.9	155107.2

LP325.220	LP325.220	LP325.220	LP325.220	LP325.220	LP325.220	LP325.220	LP325.220	LP325.220	LP325.220
126841.7	161660.3	114718.9	125449.2	130834.2	172700.2	114554.8	177731.8	146054.3	157394.1
124347.5	173815	113100.2	117321.3	128612	142378.9	121183.7	182336.7	159611.9	160635
124254.3	182059.6	124078.5	114015	105505.5	158333	187814	153010.6	203073.4	142440.3
99151.19	171769.6	97375.98	131629.7	116722.7	132983.1	147527.2	201888.7	175622.8	193872.4
125725.3	152765.9	119306.7	133189.7	122686.2	141796.2	97091.45	187845.9	168893.1	147440.2

LP325.220	LP325.220	LP325.220	LP325.220	LP325.220	LP325.220	LP325.220	LP325.220	LP325.220	LP325.220
161198.8	120097.7	112365.4	138950.7	127210.6	140044.1	140629.2	168132.5	141827.6	143417.7
157376.8	123087.4	109128.5	139954.2	138450.3	142003	162740.5	178507	145594.5	140516.7
163518.2	113934.7	137868.5	167651.7	136297.2	227245.2	159660.7	163505.4	160791.9	149920.3
146911	128278.2	139739.1	132218.9	132805.1	137130.3	140463.1	144638.5	138902.6	151432.1
147085.3	121291.8	113968.8	138859.6	126196.3	123057.6	140729	163097	138349.6	133874.7

LP325.220[illegible]	LP325.220[illegible]	LP325.220[illegible]	LP325.220[illegible]	LP325.220[illegible]	LP325.220[illegible]	LP325.220[illegible]	LP325.220[illegible]	LP325.220[illegible]	LP325.220[illegible]
118327.7	137109.9	168818.5	128431.4	148069.2	123516.3	154088.1	187267	125510	142592.1
121433.8	124554.4	189995.1	132025	151674.9	122471.1	144790.6	186349.7	115472.8	123151.7
129569.1	139271.5	163780.8	216256.2	130363.8	139467.4	155200.3	171906.8	121645.7	92559.46
124301.5	140368.2	205090.8	162549.4	133247.2	118471.9	153527.7	183692.8	124252.3	147895.9
124691.2	126648.8	190282.7	111378.2	134782.9	118188.9	148986.7	173965.6	114541.9	130502.5

LP325.220	LP325.220	LP325.220	LP325.220	LP325.220	LP325.220	LP325.220	LP325.220	LP325.220	LP325.220
141795.3	115349.4	110569.1	112040.1	116190.2	97580.39	177338.8	122078.3	127784.1	110480.2
143331	105819.1	93748.79	114260	132936.2	95936	167006.8	113115.8	124163.7	118305.6
155295.5	156042.3	97253.04	221791.6	111869.1	100641	112604.3	167588	151899.1	150049.4
159459.5	103633.5	89950.63	113088.1	157643.2	87453.39	256019.2	119997.7	148302.9	105512.5
139342.5	114096.5	84143.22	106984.6	109012.9	99436.85	157889.9	125650.2	131727.5	106516.2

LP325.220	LP325.220	LP325.220	LP325.220	LP325.220	LP325.220	LP325.221	LP325.221	LP325.221	LP325.220
98800.78	107316.6	126915.3	141131.8	135396.9	106854.3	90025.25	80251.81	94869.8	161496.1
118250.9	104582.3	115996.9	140874.4	140099.3	112263.1	91987.62	84233.73	90820.42	147684.5
113557.4	115626.4	117428.5	142852.2	147270.8	121696.4	82412.12	81851.03	94792.5	134720.1
152257	114534.9	117182.4	168494.2	145421.7	124078.9	84632.37	81289.32	93942.8	160157.1
105630.7	112743.8	113941.5	135450.6	148928.4	116620.8	92760.15	84159.36	98748.57	144904.8

LP325.220[illegible]	LP325.221[illegible]	LP325.220[illegible]	LP325.220[illegible]	LP325.220[illegible]	LP325.221[illegible]	LP325.220[illegible]	LP325.220[illegible]	LP325.220[illegible]	LP325.220[illegible]
143316.5	122190.3	134200.2	151455.4	109642.7	89356.6	114011.3	177606.9	167531.6	99985.89
136981.7	130210.2	136636	161772.1	110343.9	94014.1	109649.4	178133.9	177746.2	100656.9
174350.9	80650.62	143083.6	147692.4	108467.1	89827.01	141357.4	172554.8	175439.4	75065.33
176329.9	128714.9	148941.1	151200.9	118064.7	90464.68	112948.9	221288	190112.2	101406.8
119053.6	135840.2	140130.7	146266.5	111884.8	95739.38	126778.1	164837.7	159621.5	102613.2

LP325.220	LP325.220	LP325.220	LP325.221	LP325.220	LP325.220	LP325.235	LP325.234	LP325.235	LP325.235
155529.7	129039.2	108554.7	109680.8	88457.7	90257.68	57588.93	78275.44	102149	85843.21
155508.7	127253.5	114155.1	105196.2	88438.38	87791.53	174706.9	139790.9	93258.2	76611.92
167397.6	131308.4	101565.7	107285.8	94301.11	64904.98	188568.2	153333.4	101895.5	74220.52
158280	143132.8	116656.5	112291	85895.79	93531.32	207653.5	140884.8	92505.99	88150.03
147162.7	130130.4	114221.2	104586.1	96645.05	86409.11	91047.17	65953.86	82860.75	69922.8

LP325.235[illegible]	LP325.235[illegible]	LP325.235[illegible]	LP325.235[illegible]	LP325.235[illegible]	LP325.235[illegible]	LP325.235[illegible]	LP325.235[illegible]	LP325.235[illegible]	LP325.235[illegible]
83097.26	69725.27	65997.11	134042.1	66371.63	56719.04	77890.02	49976.37	68255.57	65607.07
88570.89	99685.48	67541.73	129520.5	79306.48	90036.93	87900.48	112966.9	133763.3	125393.5
90781.46	100901.5	68405.18	150095.2	72004.4	88513.76	90695.66	123249.2	154382.1	154519.1
91724.1	96797.54	66644	151795.1	76776.98	93078.68	96688.86	124193.8	155248.6	157361
67614.89	72489.75	62899.55	66210.77	96893.08	83418.38	88332.94	59279.59	69802.72	62215.61

LP325.235[illegible]	LP325.235[illegible]	LP325.235[illegible]	LP325.235[illegible]	LP325.235[illegible]	LP325.235[illegible]	LP325.235[illegible]	LP325.235[illegible]	LP325.235[illegible]	LP325.235[illegible]
101076.4	64909.58	74579.48	63578.05	71793.3	58320.53	53347.73	59551.64	58872.62	86111.22
102357.2	98919.51	64836.56	102516.4	98168.75	104977.2	161444	90431.14	125818.5	123745
107718.7	99967.39	68009.61	111318.7	107669.4	101196.6	161770.9	95800.83	128574	132038.2
128653.8	102013.9	60486.07	108045.1	104319.1	107493.8	179763	100299.6	145893.8	138296
84991.78	109142.8	86442.5	80396.86	91961.26	79918.07	76298.87	85938.56	53767.32	89743.37

LP325.235	LP325.235	LP325.235	LP325.235	LP325.235	LP325.235	LP325.235	LP325.235	LP325.235	LP325.235
107077.5	67243.4	136415	52738.38	105904.4	144501.9	104109.9	114557.8	87417.58	122751.9
99878.81	118227.8	134545.9	170234.2	115720.1	153180.1	101768.8	118635.1	203798.1	125897.8
103486.6	128791.1	141612.1	170126.8	105569.3	163629	98542.85	108506.2	217918.8	132372.7
111825.2	134925.6	154246.3	162311.6	108504.7	160233.8	101938.8	121802.3	224400.1	119436.5
58890.43	57248.91	85770.9	53602.07	88970.16	70265.78	45051.71	130654.5	70989.61	125283.7

LP325.235	LP325.235	LP325.235	LP325.235	LP325.235	LP325.235	LP325.235	LP325.235	LP325.235	LP325.235
57538.86	43262.78	72244.06	44503.27	105346.6	45475.28	47882.14	46170.1	76106.09	63741.53
99102.27	113009.1	129649.3	146296.3	112788.8	143446.4	137795.3	176107.4	87822.29	130343.2
109334.7	119232.4	139578.9	145654.7	116454	154163.2	149613.2	166398.1	87039.98	148466.6
104472	123144.7	143343	158748.5	116457.9	160679.1	154695.6	171698.8	84553.83	135294.4
97546.16	44817.97	78449.38	45091.41	118929	82122.03	155472.3	51736.23	89297.81	75082.42

LP325.235	LP325.235	LP325.235	LP325.235	LP325.235	LP325.235	LP325.235	LP325.235	LP325.235	LP325.235
54676.73	49895.24	111492	82081.56	51734.51	159900.8	128646.8	124993	70880.43	36341.69
132547.6	136312.9	107572.7	95576.43	154503.4	181136.1	125244.5	138219.1	179491.6	121953.6
147578	138029.8	113779.9	97728.71	150767.1	171210.7	127426.1	144372.8	190061.3	135222.3
152814.7	137331.3	122391.6	92844.36	158591.1	181604.8	139801.1	134841	178243.2	114117.8
153250.8	54645.54	123902.5	59097.38	54372.99	46426.74	25723.69	34684.01	176248.8	111864.1

LP325.235	LP325.235	LP325.235	LP325.236	LP325.235	LP325.235	LP325.235	LP325.235	LP325.235	LP325.235
145488	173664.9	122633.6	46193.96	85315.94	65199.25	115339.6	134107.5	154757.2	140221.5
144984.7	161173.8	127216.2	136983.2	76882.91	151851.4	115328.9	146979.7	165332.8	149501.3
166242.9	175046.8	144331	137602.4	83019.94	131415.7	117547.6	145072.4	167338.5	153130
167793.8	191549.4	148380.8	139137.6	97563.45	156618.1	117639.7	139200	171887.1	144335.2
73732.32	178956	54949.47	130644.4	85795.53	142266.1	126168	138537.1	65474.11	49840

LP325.235	LP325.235	LP325.235	LP325.236	LP325.236	LP325.235	LP325.235	LP325.235	LP325.235	LP325.235
53564.08	118987.9	120688.2	112966.1	107096	100072	126062.1	176483.3	134373.7	140400.2
147718.6	122117.8	116406.7	104547.3	101324	101875.1	121393.8	173989.7	148228.8	155158
153906.9	134022.6	129501.6	113397.7	111785.6	102071.4	123996.5	166850.3	117646.2	154089.4
155195.6	137889.5	126272.7	103902.4	106515.2	101070	125869.8	176355.6	133677.3	145818.5
144778.2	56097.5	61767.66	95186.63	106881.5	101821.2	109843.5	170257.9	122535.7	160171.9

LP325.274	LP325.274	LP325.274	LP325.274	LP325.274	LP325.310	LP325.310	LP325.309	LP325.309	LP325.310
74569.02	105722	71355.71	97282.04	99089.65	328164.8	145821.8	50245.35	73939.76	67031.07
69743.5	98163.3	72176.86	91936.24	96573.96	334232.9	171346	41590.02	61306.91	60575.63
68813.8	99586.25	62904.35	98491.66	94170.2	373568.4	103790.3	46903.76	60947.63	62138.48
66622.97	97129.3	61953.94	91774.47	88635.69	336799.4	143192.4	39610.28	65760.44	56460.52
76373.04	107950.4	70049.14	91144.59	92975.45	337080.6	98714.75	52910.72	75016.07	60317.7

LP325.310	LP325.310	LP325.310	LP325.310	LP325.310	LP326.196	LP326.196	LP326.223	LP326.238	LP326.305
69115.13	131686.2	99727.29	85812.55	63760.91	12043.98	22810.93	18928.51	13595.44	51482.58
66980.96	112985.8	92369.75	79963.41	54166.15	13828.35	23479.57	20045.21	14644.4	55108.51
71279.77	110116.7	96065.77	83139.25	48541.5	10211.13	22391.63	17594.41	13328.37	70240.41
60800.91	109203.1	85589.69	80159.29	49957.98	10246.46	24348.1	17549.46	17289.89	77413.93
66328.75	105309.3	97929.9	86752.05	51233.09	8858.577	22018.05	17768.65	16698.36	81738.39

LP326.305	LP326.313	LP326.342_	LP326.342_	LP326.378	LP326.378	LP327.077	LP327.078	LP327.078_	LP327.077
28138.77	68586.03	146016.5	87879.04	20866.92	73482.09	93028.66	93588.31	102505.4	128285
27956.07	67762.17	159615	98895.97	31475.33	84503.97	86562.55	88585.88	99145.35	131916.9
28682.8	72177.74	235603.2	145882.5	27665.79	134905.3	89556.19	93325.3	102044.8	128747.1
28825.8	59995.46	285970.9	171053.1	25907.99	168166.1	92745.51	97342.27	100159.2	149130.8
29224.26	71696.48	314435.8	189441.7	19482.7	185131.7	35016.04	50468.46	45385.06	50230.42

LP327.078	LP327.078	LP327.078	LP327.078	LP327.078	LP327.078	LP327.078	LP327.078	LP327.078	LP327.141
100984.9	142244.8	128435	110572.9	122717.9	110075	120806	150215.5	138964.1	131782.2
108015.2	139878.5	126870.7	108329.7	109460.2	115447.9	122145.4	146498.6	127336.4	117104.1
110568.6	135960.4	140986.8	119356.6	128477.3	110052.9	129861.4	154323.3	150679.6	120355.7
105256.4	155235.9	128677.7	105402.7	122887.4	112359.4	132964.6	151450.4	148891.5	134038.9
43677.87	56441.71	65131.03	45124.88	59358.53	52454.51	50601.15	71891	59333.28	122193.6

LP327.141	LP327.141	LP327.141	LP327.141	LP327.141	LP327.141	LP327.141	LP327.141	LP327.141	LP327.141
163372.7	123564.5	130954.1	162221.5	117378.4	116253.6	162538.2	148553.7	181547.6	105613.1
112712.4	101087.5	133220.8	184570.9	128611.3	116679.2	219113.6	133105.9	142148.7	69248.24
93028.23	126768.5	110593	208373.9	145827.4	87660.49	130933.8	202853.4	165712.3	76012
163385.8	93220.38	133709.1	238835.5	111533	113366.3	154714.3	157029.5	120715.7	59133.7
125074.2	121371.6	126409.7	152086.5	141244.6	152977.3	107581	132800.8	150079.8	77549.17

LP327.141	LP327.141	LP327.141	LP327.141	LP327.141	LP327.141	LP327.141	LP327.141	LP327.141	LP327.141
136442	145947.1	200945.9	129220.4	101626.8	164352.7	132166.8	134320.3	183854.2	148432.2
119530.4	181526.9	127676.9	126992.2	119843.7	116191	128813.2	140324.8	158034.1	138885.2
114695.7	183107.2	167936.2	163746.7	122614.7	106547.8	155054.2	177472.6	111222.7	134872.2
163824.3	154661.6	142600.9	97906.55	146582.4	143659.9	110486	145447.1	167478.5	189109.5
131965.1	130968	223007.5	110391.3	150014.1	128663.4	158388.9	162067.7	99249.8	146994.8

LP327.141	LP327.141	LP327.141	LP327.141	LP327.141	LP327.141	LP327.141	LP327.141	LP327.141	LP327.141
113000.7	121594	98438.06	159700.2	153581.4	147935.9	144240	110635.9	151317.8	111545.4
160386.5	202235.3	129807.6	122825.5	153503.9	128249.4	156893.4	109539.2	132014.2	156977
149940	141062.9	106856.3	212578.2	160046.6	121575.2	156379.9	104850	122929.6	107832.1
145504.2	238474.2	111803.4	137667.6	156632.9	130914.2	158001	78759.43	159348.4	127340.2
102240.1	198810.9	118360.5	153078.9	193369.7	151210.5	164136.4	120799.8	145098.7	138448.1

LP327.141	LP327.141	LP327.141	LP327.141	LP327.141	LP327.141	LP327.141	LP327.141	LP327.141	LP327.141
141680.5	185984.9	183771.5	134280.2	125444.2	145905.9	162969	167489.3	109187.7	131557.1
128622.9	147955.2	136242.7	137939.3	130233.6	171754.6	168517	135309.9	100103	159031.8
113295	149879.2	129283.8	177763.2	124188	161117	140000.3	141759.4	155785.5	131999.4
115255	209817.4	145135	191345.3	98996.24	129390.4	172670.9	128531.5	118653.3	181806.3
122234.9	143284.8	174619.2	129091.2	129997.6	129318.6	122630.6	167441.6	96460.02	139529.5

LP327.141[illegible]	LP327.141[illegible]	LP327.141[illegible]	LP327.141[illegible]	LP327.141[illegible]	LP327.141[illegible]	LP327.141[illegible]	LP327.141[illegible]	LP327.141[illegible]	LP327.141[illegible]
120238.8	126825.8	133787.6	106448	176859.2	116861.2	163571.2	112543.3	191240.5	129913.5
122520.8	188765.2	166514.3	135565.5	152645.7	111288.1	163229.7	101544.3	203409.7	112197.7
149573.1	115274.2	144788.5	161347.7	110333.4	114195.9	185808	109142.5	146489.9	138269.5
132957	165824.2	144740.3	132200.6	100455.5	126407.7	136137.7	119328.1	190776.1	144604
156805	140710.5	201174.2	131640.4	142640.4	117934.1	237648.5	114504.7	214599.1	179588.7

LP327.141	LP327.141	LP327.141	LP327.141	LP327.141	LP327.141	LP327.141	LP327.141	LP327.141	LP327.141
199505.9	180789.2	144826.9	119705.9	155760.2	128257.5	179420.3	144753.7	108900.8	116546
156021.8	191359.8	169846.7	103805	127360.1	116197.2	252357.7	138981.1	115711.7	156547.4
188423.7	134560.1	143831.9	105685	119524	132451.6	141218.9	119145.6	123192.4	101808.1
163383	130546.7	184594.7	103562.7	130551.5	116948.8	149245.3	164597.5	111071.1	115071.5
220846.4	132321.2	133607.1	127828.1	122787.9	155350.3	160725.5	167889	147243.8	119232.6

LP327.141	LP327.141	LP327.141	LP327.141	LP327.141	LP327.141	LP327.141	LP327.141	LP327.141	LP327.141
85601.64	122906.5	101049.1	152326.2	114119.8	162395.6	140989.3	63466.47	110788.9	90508.26
70730.55	107226.7	143536.4	79344.21	157471.2	123906.3	144180.4	66645.76	72523.41	78165.55
59679.32	128997.9	167265.1	98595.11	156972.7	176012.3	188402.9	81128.6	66017.25	59356.96
91850.4	105221.8	89464.37	95628.51	128230.8	122017.8	152430.7	65140.51	65442.25	71526.7
62562.6	112408.4	137831.9	96067.9	173635.6	104385.1	160137.8	69828.38	66253.82	78314.01

LP327.141[illegible]	LP327.141[illegible]	LP327.141[illegible]	LP327.141[illegible]	LP327.141[illegible]	LP327.141[illegible]	LP327.141[illegible]	LP327.141[illegible]	LP327.141[illegible]	LP327.18_[illegible]
82100.01	79557.25	99501.74	56243.94	45508.14	58005.81	54144.75	79454.82	67821.42	224727.3
68862.27	77322.77	90280.58	59499.89	53225.19	58377.44	84765.67	76073.56	63146.54	246317.6
70810.32	71577.81	100470.3	53401.07	62539.47	73707.67	57513.66	93925.25	59597.93	261867.5
57690.63	98051.44	89193.43	73877.94	43321.37	59887.16	49441.15	66637.08	57825.79	114826.9
72366.03	80886.04	97129.08	64411.64	44946.97	52142.5	56760.51	57215.33	56438.54	254594.1

LP327.178	LP327.178	LP327.179	LP327.179	LP327.178	LP327.179	LP327.179	LP327.179	LP327.178	LP327.179
207089.1	126151.1	86263.72	130435.8	246506.9	202901.1	121956.1	237023.4	113115.6	152506.1
200606.9	134028.1	80528.97	131223.5	177632.6	202098.9	249662.9	232729.4	109608.2	170673.8
205357.9	126901.5	82188.9	125063.6	253073.8	202843.5	251210.2	233292.5	113325.4	149029.6
193697.9	130926.2	83933.44	110832	244245.7	185768.7	227218.8	210590.6	107175.2	131533.1
176304.3	132441.7	84201.65	109882.9	245263.1	181603.3	230289.3	202829.8	121565	142581.6

LP327.179	LP327.179	LP327.178	LP327.179	LP327.178	LP327.179	LP327.179	LP327.179	LP327.179	LP327.195
132937.6	126581.4	167766.6	197094.3	162585.3	95178.94	172984.4	113186.9	185542.6	545937.1
136090.9	135847.3	182592.1	196036.7	161262.9	103246.8	171043.7	109002.6	196440.8	711781
132810.8	139772.6	199904.7	187726.7	164912.8	97108.17	184468.1	105453.7	195370.8	686622.4
143386.4	130153.1	176367.7	188433	147254.3	103258.5	180927.7	107886.6	188123.8	714051.7
136974.1	133256.1	179343.7	183993.5	145521.2	68646.75	161336.6	109827.2	198160.5	1028787

LP327.195	LP327.195	LP327.195	LP327.195	LP327.195	LP327.195	LP327.195	LP327.195	LP327.195	LP327.195
646701.3	410909.2	487224.8	231755.7	427279.6	257765.3	359347.5	311064.3	318461.2	121286.9
637398	366764.5	482207.7	217195.2	314917.9	322433.6	379132.5	290630.3	289243.7	100770.7
542972.6	454790	475482.6	244287.4	441171.7	291300.4	364467.8	402492.1	308102.2	104742.1
690537.1	530754.2	389726.7	226633	280457.8	230252.4	366900.1	274332.5	325339.8	102329.7
713506.8	498260.6	456867.2	235292.4	371173.1	259279.4	361141.4	283043.4	228402	123274.5

LP327.194	LP327.195	LP327.195	LP327.195	LP327.194	LP327.195	LP327.195	LP327.195	LP327.195	LP327.195
169198.3	116525	154045.6	167837.8	170827.7	210377.2	180956.4	137385.5	142548.4	151167.5
156402.5	174301.5	149759	139544.6	123241.1	248461.1	175361.9	149819.6	139888	133548.4
150145.2	108751.2	142579	162878.3	132362	215096.1	208784	236108.2	149952.7	122685.9
128253.1	119233.8	136208.3	163787	145198.7	171172.2	203842.4	161762.1	164101.9	124419.9
131049.1	111062.8	126473.9	181710.5	99992.61	174049.2	162478.5	160425.8	220348	112531.6

LP327.194	LP327.195	LP327.195	LP327.195	LP327.195	LP327.195	LP327.195	LP327.195	LP327.195	LP327.195
89581.57	135627.1	326731.4	165145.9	169213.3	125808.8	111432.1	131587.3	168862.9	157853.9
200961	124631.1	358718.6	167479.9	183547.9	124926.6	123167.7	152420.7	153212.5	167616.7
90558.21	138058.2	325482.3	152665.3	207243.6	117218.4	119903.4	149424.2	175374.7	156954.6
94014.43	124875.7	322630.5	151285.3	166696	119875.1	123203.7	133467.6	168784.4	163778.8
89428.95	132734.1	323319.3	134740.6	159164	137584.6	98158.88	132871.2	146976.5	157292.6

LP327.195	LP327.195	LP327.195	LP327.195	LP327.195	LP327.195	LP327.195	LP327.195	LP327.195	LP327.195
162723	154010.9	148294.9	142833.4	154238.5	118197.5	105795.5	132431.9	117517.7	105287.6
170165.8	143300.8	146637.6	141853.8	158755.5	119747.2	106554.1	140232.2	105693.1	122892.7
165392.6	157214	126835.2	138323	156592	122057.8	127049	143414.9	117867.5	115235.8
182590.4	166725.2	148886.4	150379.6	137208.6	128641.3	115864.5	142810	110635.9	106498
160665.6	136727.4	120635.4	127946	148600.8	110406	109661.5	130025.1	113275	112154.9

LP327.195	LP327.195	LP327.195	LP327.195	LP327.195	LP327.195	LP327.195	LP327.216	LP327.216	LP327.216
132486.8	113830.9	114244.4	130445	162509.3	162681.2	115034.4	300975	259124.3	150058.7
135358.5	112388.9	134324.2	137609.8	167768.9	153438.6	111671.5	291311	277501.4	141146.9
145262	113551.3	130900.9	135729.7	166836.9	161081.8	115121.3	302004.2	297588.6	160277.9
126979.4	112563.4	124963	127072.9	155190.4	174689.9	108809	295511.9	273620.2	156797.7
125846.9	117209	108987.1	135430.2	154320.9	129627.4	98005.8	312262.3	285680.6	151126.4

LP327.253	LP327.253	LP327.253	LP327.253	LP327.253	LP327.253	LP327.253	LP327.253	LP327.253	LP327.289
100540.6	58057.49	52287.04	48098.19	38194.29	31469.2	35609.55	50271.16	45316.48	44499.04
54464.68	50605.07	54788.16	54505.68	33992.35	31813.68	41321.27	46271.4	50466.59	53579.5
95017.12	62633.47	52738.22	42879.22	36378.7	35313.48	40599.72	49772.37	48460.31	49812.74
75940.61	53738.28	45972.96	50026.87	36546.91	29726.13	38970.14	41951.47	53136.31	44310.42
68612.24	54498.83	46933.95	43429.89	33612.53	34469.01	41820.36	51925.65	49700.35	43092.94

LP327.289[illegible]	LP327.289[illegible]	LP327.289[illegible]	LP327.289[illegible]	LP327.308[illegible]	LP327.336[illegible]	LP328.011[illegible]	LP328.011[illegible]	LP328.011[illegible]	LP328.011[illegible]
53193.85	35988.17	62177.12	52739.18	33872.92	26592.6	65732.28	83514.44	95626.27	64226.93
51706.63	35327.26	60082.74	50197.76	35163.73	27139.36	54991.23	70755.77	64648.75	52834.21
50552.08	33362.72	64407.56	45346.07	33343.28	26976.95	60493.16	61867.19	63193.43	60632.1
48457.42	35558.21	58155.65	47669.04	34554.98	26182.79	62809.32	70660.3	91962.48	63424.71
58367.45	32443.3	60704.87	46162.42	32692.13	26776.48	56591.89	69283.07	63578.67	62240.94

LP328.011	LP328.011	LP328.011	LP328.011	LP328.011	LP328.011	LP328.011	LP328.011	LP328.011	LP328.011
55160.84	79202.88	93589.28	70251.47	58024.22	119566.8	63530.08	55827.78	85579	79164.68
56303.03	76966.16	98647.8	51982.39	63882.05	80411.51	59682.1	56368.45	68448.59	79354.28
54041.54	68567.11	94872.75	49524.94	59013.56	80600.91	74806.69	49761.28	63528.54	84009.26
72258.03	67909.86	99116.88	55825.58	63590.57	87166.48	69914.77	53137.54	70636.31	81923.91
63381.93	70299.67	101989.7	54626.03	55499.97	85776.58	73683.32	52904.23	74403.36	83040.79

LP328.011	LP328.011	LP328.011	LP328.011	LP328.011	LP328.012	LP328.011	LP328.011	LP328.011	LP328.011
80342.79	58318.66	94894.53	68228.57	52216.68	61235.42	55340.66	107683.6	66508.99	70991.13
85481.16	67258.55	71207.85	82089.3	68379.29	66298.11	51566.01	112484	69511.27	67533.02
82090.65	58358.96	76773.05	72034.37	74078.2	63430.72	54393.68	112875.8	58099.25	74002.98
77266.72	68721.72	80275.78	98262.48	72165.89	63503.63	56534.26	125450.6	67886.51	80348.38
87983.39	56722.33	75750.54	68977.61	72922.82	64822.61	52892.15	126311.4	67191.07	80404.3

LP328.011	LP328.012	LP328.011	LP328.011	LP328.011	LP328.011	LP328.011	LP328.011	LP328.011	LP328.011
85223.09	95381.17	65296.58	72889.2	74664.42	66307.75	65423.23	84870.86	54444.91	52892.02
82155.64	96767.5	68891.17	74888.93	72639.32	66688.34	63357.33	93116.23	54072.6	46182.81
83803.23	98465.1	69875.83	70967.06	76812.5	64683.57	64764.53	92174.54	57051.8	47386.76
84402.63	101007.1	67632.09	66567.25	70043.18	71445.43	70143.26	92395.16	62591.28	46426.11
90887.22	98263.69	68649.38	77434.12	74374.06	68310.24	78096.96	87318.02	55721.66	53569.66

LP328.011	LP328.011	LP328.011	LP328.011	LP328.011	LP328.011	LP328.011	LP328.011	LP328.012	LP328.011
62049.34	67538.62	55155.87	46089.47	35384.92	53363.43	73212.44	83087.24	68655.87	86800.72
60727.15	58806.82	51987.07	50998.53	38172.49	52116.44	75227.23	78929.02	65991.88	89950.35
59286.77	64368.71	48133	46529.63	35847.54	51095.89	71191.93	88950.46	65518.59	73706.78
65174.52	59399.95	53299.61	52247.97	36934.14	50501.89	79363.35	82274.66	73415.08	93502.8
61205.22	63433.7	54886.72	47967.85	33676.76	59720.95	74940.99	91292.29	68976.74	87812.92

LP328.011[illegible]	LP328.011[illegible]	LP328.011[illegible]	LP328.011[illegible]	LP328.011[illegible]	LP328.199	LP328.199[illegible]	LP328.198[illegible]	LP328.211[illegible]	LP328.212
84210.02	49616.25	42970.85	49928.56	57724.17	84803.2	82880.26	131324.9	76205.17	35662.97
92609.15	44530.71	34565.84	44106.24	52951.62	85963.74	83091.94	145678	77101.72	37520
84051.9	46325.37	35225.79	45275.93	56244.49	89486.31	91084.48	142666.8	77252.34	37596.32
86321	45916.87	38003.66	41582.28	56618.84	82559.7	80372.33	135588.9	80606.26	34371.84
93716.59	43386.7	35148.61	44433.33	58263.84	83132.53	76169.16	134538.8	66293.33	33502.32

LP328.256	LP328.256	LP328.284	LP328.285	LP328.321	LP328.321	LP328.348	LP328.348	LP328.915	LP328.915
21396.23	17024.12	45058.63	16816.74	89795.19	68493.94	21791.03	18345.29	66715.27	41015.01
23504.97	18290.11	51717.53	14779.73	98983.36	68817.48	23195.37	17117.45	64601.79	38331.77
25802.92	17846.43	52261.08	19944.95	97175.19	76322.79	22981.97	18307.68	96554.48	58754.69
23640.33	20360.11	50389.81	18971.09	103121.4	83919.86	23692.27	18425.94	131395.5	60084.14
27357.79	16784.3	54786.4	19686.6	103249.1	79898.85	24078.24	18951.68	50501.69	26385.73

LP329.157	LP329.157	LP329.157	LP329.157	LP329.157	LP329.157	LP329.157	LP329.157	LP329.157	LP329.157
316546.6	477217.6	310166.9	419460	434670.2	394110.2	514784.5	340105.2	405219.1	470649.3
332268	370816.3	305414.7	378399.4	356505.8	386627	397153.7	270820.4	558518	331400.6
380084.1	492403.5	296972.4	294604.5	386112.6	444363.2	340775.6	278890.2	350410.8	491993.7
336278.6	323766.3	374186.9	482178.9	334211	313236.5	452344.7	509752.2	416847.7	343555.1
217298.8	354418.9	288492.1	354509.2	379643.8	423422.1	404457.2	288704.4	521904.4	296166

LP329.157	LP329.157	LP329.157	LP329.157	LP329.157	LP329.157	LP329.157	LP329.157	LP329.157	LP329.157
269916.3	376099.5	590690.5	487574.4	347451.5	260704.7	392391.9	391941.5	549851.3	253191.9
386988.2	466406.4	455190.7	591794.7	377752.1	255016.2	587278.7	358796.4	332975.4	321218.1
464725.6	376768.7	374506.8	395921.8	357793.8	503712.7	538676.1	416400.5	461665	339380
308095.8	351754.2	471110.4	394327	343289.5	288027.8	349036.7	505469.4	452649.9	371704.8
262746.6	339319.1	346669.7	341657.5	494842.1	278011.7	362475.4	372571.5	374011.2	453671.7

LP329.157	LP329.157	LP329.157	LP329.157	LP329.157	LP329.157	LP329.157	LP329.157	LP329.157	LP329.157
357859.7	512800.1	437366.4	281309.3	351520.6	245534.1	288751.1	250425.7	288320	226880.5
347434.7	621563.7	377062	352791.7	288271.2	294433.6	244810.7	210880.9	352769.1	453526.2
551986.2	383758.9	481315.1	425761.7	290185.9	334011.1	277302.5	223449.7	262341.6	291531.7
298403.9	642276.7	482611.7	370933.3	472212.9	256223.5	368791.1	183776.6	344831.3	483274.6
385262	404117.1	482809.3	324487.8	372229.9	303309.1	375196.2	190895.9	388563.2	348442.5

LP329.157	LP329.157	LP329.157	LP329.157	LP329.157	LP329.157	LP329.157	LP329.157	LP329.157	LP329.157
374263	225502.2	466964.8	274932.3	312862.4	241394.8	294579.7	325182.4	450217.4	319698.6
311585.1	158293.3	627792.2	404470.7	319743.1	275521.3	344934	289958.6	261433.7	413989.3
321410.3	136644.2	533203.6	365252	299500.9	307424.2	246259	358665.2	297139.8	388981.6
289754.6	206869.4	549982.7	456520.5	262229.6	269280.8	305133.8	343454.3	341638.3	506864.2
402185.1	175313.5	388423.6	259371.1	362986	311937.5	338967.7	307683.5	388560.8	424013.7

LP329.157	LP329.157	LP329.157	LP329.157	LP329.157	LP329.157	LP329.157	LP329.157	LP329.157	LP329.157
282959.4	298292.9	274554.1	277037.2	166008.2	140041.9	198677.7	142280	151584.5	187988.8
272632.4	348439.8	277528.2	330503.5	150098.7	168494	189209.7	192397.9	184615.1	175257.2
233010.2	369426.5	267413.8	276885.7	146540	179126.3	202873.1	163352.4	157137.1	154473.4
278347.8	302891.2	256457.3	309390.9	124178.1	179354.7	191884.4	129430.3	221092.3	174723.6
367960.7	382319.5	297425.6	286280.4	142053	123178	223344	140540.3	139906.9	187220

LP329.157	LP329.157	LP329.157	LP329.157	LP329.157	LP329.157	LP329.157	LP329.157	LP329.157	LP329.157
99072.35	136030.9	260533.5	600304	548953.4	383669.6	354179.6	517959.5	402364.1	436231
166220.4	117725.6	355615.8	274085.2	370724	409148.8	486141	331396.3	442427.1	379997.6
108886.8	110096.7	385700.9	370272.7	305198.7	322326.8	348176.9	440428.4	420733.1	494767.8
123973.6	112349.6	315314.8	295449.2	503544.3	363539.8	530384.3	342616.8	427228.6	356858.3
101558.7	113058.6	256428.9	437395.7	511642.5	313656.4	450467	460050.1	345710.4	535543.1

LP329.157	LP329.157	LP329.157	LP329.157	LP329.157	LP329.157	LP329.157	LP329.157	LP329.157	LP329.157
361373.4	543717.1	337835.9	313258.1	390739.8	340398.8	331028.5	504020.1	327119	428994.7
489584.9	429374.3	338689	393332.5	767981.7	388261	443286.9	348022.4	299730.6	555897.7
357644.2	322891.4	422084.1	469861.4	358154	382716.8	469412.5	296315.9	263569.9	554144.1
409170.4	556381.9	285198.3	384539.2	640237.2	399222.3	668859.6	306429.9	279066	346001.1
420132.5	450697.3	399773.9	480294.9	401777.5	337319.2	440507.3	487871.1	296590.4	409476.1

LP329.157	LP329.157	LP329.157	LP329.157	LP329.157	LP329.157	LP329.157	LP329.157	LP329.157	LP329.157
283610.1	376874.2	289252.7	266435.2	265801.6	333403	167284.2	175092	401306.8	391309.5
240915.6	278331.9	322278.4	260735	263333.4	290267.6	157886.2	162985.1	405157.4	352166.8
256274.5	508133	347325	265434.7	313517	282051.2	158902.6	145483.9	364948.1	352829.8
334040.5	411187.9	352640.9	349048.1	278255.8	299138.3	163110.7	162514.4	395963	394066.9
366444.9	290391.9	399337.6	314533.9	265284.1	282766.3	149815.9	126673.6	410258.4	396902.2

LP329.157	LP329.157	LP329.157	LP329.157	LP329.158	LP329.157	LP329.195	LP329.195	LP329.195	LP329.211
311661.4	399949.9	250096.3	283838.9	272959.3	324932.2	274661.1	551454.9	240441.5	13093.27
313358.1	374151.9	236441.6	294186.1	283973.8	330296.2	277723.5	538548.9	252735.9	125995.1
307940.9	369423.5	235492.2	326851.4	288194.4	339121	302091.8	526817.9	242513.6	115541.5
316167.8	361567.8	244557.6	306338.5	261476.3	331225.7	294819.3	567353.4	265379.8	109932.1
346186.5	374970.9	267834	291702	282753.3	324712.7	297623.6	590069.1	253503.9	103853.6

LP329.242[illegible]	LP329.242[illegible]	LP329.324[illegible]	LP330.009[illegible]	LP330.008[illegible]	LP330.008[illegible]	LP330.008[illegible]	LP330.008[illegible]	LP330.008[illegible]	LP330.008[illegible]
401689	228110.7	11991.96	83239.56	52397.62	59049.86	66228.51	61832.81	60125.34	58591.13
34456.67	270227.3	8854.267	85265.12	55073.33	63660.77	67339.1	49749.66	52413.87	73227.58
406153.4	247621.3	14026.98	80439.7	60973.21	63775.94	68756.56	52399.5	60267.17	54812.22
388428.8	228454.5	6902.098	86012.56	55234.43	55587.78	61821.86	57033.34	61876.07	61258.24
399132.6	267578.3	10942.92	90994.76	60028.43	79582.71	68714.44	57764.48	63400.82	60777.49

LP330.008	LP330.008	LP330.008	LP330.008	LP330.008	LP330.008	LP330.008	LP330.008	LP330.008	LP330.009
64091.82	70809.71	57460.65	62018.7	83882.51	72303.43	63623.67	51489.19	47155.88	72985.65
63005.91	77539.52	61718.51	50940.71	77287.01	75244	60474.09	54507.92	52006.4	65918.68
62809.92	76095.07	56759.62	64740.52	70327.82	89355.84	66089.95	51267.09	54902.05	77102.89
63611.89	94357.11	60315.47	62835.1	75136.77	69869.84	63336.22	56333.52	53888.17	74883.07
59164.25	81832.93	63707.28	62044.57	78596.86	73688.4	65136.9	55815.6	54059.37	73102.97

LP330.008	LP330.008	LP330.008	LP330.008	LP330.008	LP330.008	LP330.009	LP330.008	LP330.008	LP330.009
49112.56	75175.22	44309.72	78679	54711.94	46494.63	65890.37	62631.51	61499.56	100719.8
48854.57	72159.89	52778.41	81923.4	61562.52	48035.17	77272.06	65275.24	67307.08	95571.52
46061.57	75975.84	51255.26	76961.36	60980.54	41039.49	70780.45	66446.92	63852.81	95806.36
47967.81	70419.95	48366.5	81277.95	59469.34	49285.42	75273.37	71189.96	66310.24	100791.9
44095.8	72485.41	57103.48	81503.94	57681.55	49663.36	77904.9	66113.4	68171.55	101043.8

LP330.008	LP330.008	LP330.009	LP330.009	LP330.009	LP330.009	LP330.008	LP330.008	LP330.009	LP330.009
47083.29	80979.36	104581.3	80391.21	95558.37	69585.37	73849.48	41905.3	90178.34	68484.19
50987.51	79438.32	108606.5	83016.04	98501.13	70846.3	70052.62	55094.18	88890.8	75456.01
56981.91	85626.44	112770.7	77907.51	94346.38	72287.38	70249.51	49997.58	97561.03	67637
51056.65	95377.04	103627.3	82245.8	97948.41	71350.58	71856.73	48615.3	95163.89	71353.42
53780.71	84139	104661.3	76878.34	99978.41	75266.35	73006.63	52527.34	94229.2	69260.47

LP330.008	LP330.008	LP330.008	LP330.009	LP330.009	LP330.009	LP330.008	LP330.008	LP330.246	LP330.263
77678.72	49096.13	54690.49	88796.68	76759.7	75841.95	45130.16	40895.96	45625.47	28919.89
83988.15	53021.88	54062.27	96866.54	73661.77	74078.9	50681.85	37445.95	43524.58	35165.35
84232.52	48959.96	56344.72	100727.1	78063.62	68989.42	46440.97	34914.68	44578.81	35210.11
78058.07	54764.58	52793.91	94717.22	82522.74	80168.23	45465.99	42011.71	34733.4	37989.17
82290.66	52222.89	49916.29	104284.9	80246.84	78183.14	48296.05	34947.2	36545.55	35211.33

LP330.337	LP331.166	LP331.174	LP331.173	LP331.173	LP331.173	LP331.173	LP331.284	LP331.916	LP331.916
25302.55	14025.37	36502.9	62214	56341.08	49651.15	56416.98	1116098	24834.02	33726.22
24826.3	16288.74	34896.27	58625.89	52494.79	49562.51	56395.89	1242170	25219.46	33983.52
25664.26	21376.02	39062.75	62485.86	47789.72	54416.58	51818.96	1629172	27090.98	35649
24253.73	14598.29	34951.81	61267.67	55146.32	54913.27	57520.01	1845985	34988.69	38033.86
23258.6	16538.87	32059.54	57653.26	51360.24	51280.18	63252.12	1932663	32065.38	34157.56

LP331.916	LP331.916	LP331.916	LP331.916	LP331.916	LP331.916	LP331.915	LP332.206	LP332.206	LP332.207
19071.55	33962.46	24856.16	33440.82	23447.21	33878.49	29439.99	78672.95	97720.98	61079.48
17688.81	35332.81	25438.05	35208.55	22362.14	35898.84	22707.02	81777.13	94401.09	41228.34
20894.11	37968.13	28456.93	35630.18	24660.25	35718.04	32653.66	84182.77	81842.54	60282.92
24694.27	46819.09	30414.11	41684.38	31495.16	42148.09	34831.74	77924.04	94351.37	67798.25
21280.51	39118.1	27585.4	36967.55	23589.09	37189.75	31771.59	77928.16	96660.14	48822.29

LP332.207	LP332.207	LP332.206	LP332.206	LP332.206	LP332.206	LP332.206	LP332.206	LP332.206	LP332.207
83322	62234.15	60762.12	78512.87	73690.27	62299.33	99961.57	41910.34	82339.41	56463.45
81777.21	62857.76	69513.14	81425.21	65163.22	65064.5	99164.03	39620.02	71104.98	61008.35
82320.9	61836.75	60296.53	83202.46	63904.75	50216.67	109344.5	39944.8	74181.77	45479.05
81543.21	61957.06	66259.9	84516.21	76231.95	60029.94	103387.1	38171.99	71548.82	53644.29
80644.75	58006.45	59485.85	81651.75	70063.73	56778.93	100182.6	43391	73331.56	58384.46

LP332.207[illegible]	LP332.207	LP332.206[illegible]	LP332.207	LP332.207[illegible]	LP332.207[illegible]	LP332.207	LP332.206[illegible]	LP332.206[illegible]	LP332.206[illegible]
72656.23	61652.83	97967.04	65629.38	68447.14	126007	70819.36	76517.48	82520.11	74360.13
70207.66	58351.74	102116.9	56617.18	61369.15	124157.5	73921.74	68083.44	97824.95	66855.5
68114.44	60652.49	94771.8	60716.68	59193.47	89222.23	69403.21	74824.58	76146.38	65406.32
74752.96	52634.07	99943.68	61770.86	65486.2	121555.5	69172.62	74111.64	93593.97	78719.23
67733.56	61258.3	106118.7	51148	64098.87	119859	74476.43	72517.39	83265.78	81868.94

LP332.207	LP332.206	LP332.206	LP332.207	LP332.206	LP332.207	LP332.242	LP332.243	LP332.243	LP332.243
55665.94	74842.79	67532.34	67105.03	67935.41	73116.72	79498.2	59345.46	58769.72	68337.95
50772.46	64575.6	71864.97	65578.83	63705.58	73288.16	73348.14	53141.05	52793.69	68678.2
59551.69	82111.26	59924.76	67614.18	71956.04	67698.27	70740.07	55993.99	46666.26	68904.66
53586.47	82549.93	69906.09	68461.52	71199.98	77922.3	70646.12	65300.17	54959.87	59210.11
55246.64	78479.36	70992.39	64996.48	70558.89	78277.53	118983	63223.75	52085.45	69798.37

LP332.242	LP332.243	LP332.242	LP332.243	LP332.243	LP332.243	LP332.243	LP332.243	LP332.243	LP332.243
66293.6	69818.52	53051.38	60594.34	71858.51	46883.9	49402.18	59991.41	52661.79	66926.94
75930.41	73574.81	52624.89	67662.13	61343.69	52803.13	58006.3	62137.92	55101.99	59353.32
64353.83	71158.94	53740.27	60645.59	68750.44	51039.52	59580.86	53798.44	54221.04	62498.34
59820.7	71961.47	59662.86	60278.98	65660.57	50271.64	54744.38	59705.48	54898.06	63840.63
57911.5	64292.77	57318.82	70535.75	65307.16	51414.32	60399.79	53910.06	51941.98	66001.54

LP332.243	LP332.243	LP332.243	LP332.243	LP332.243	LP332.243	LP332.243	LP332.243	LP332.243	LP332.243
66793.51	90558.01	47612.87	78624.95	53125.16	83023.18	55338.8	59867.38	65932.77	60442.92
69831.16	82217.7	41510.93	82994.5	58171.17	81867.19	57745.42	67369.26	64195.25	55213.49
69310.93	94174.15	43317.86	88922.02	54088.14	85564.46	55779.08	61578.33	62105.66	54201.66
68854.93	90399.39	46722.85	85564.46	49165.86	91836.07	56220.62	61632.37	60108.41	63103.51
75009.34	81262.96	50079.43	84461.02	53592.88	81597.55	57802.71	60167.93	63503.84	58216.62

LP332.243	LP332.243	LP332.242	LP332.243	LP332.243	LP332.243	LP332.243	LP332.243	LP332.279	LP332.292
48257.36	39002.99	62724.41	62238.21	76665.45	71315.8	79646.61	53233.64	68900.9	79293.43
50983.9	37069.48	61350.44	60307.39	77807.4	68398.24	79665.2	57243.72	66886.07	68973.71
49149.98	44429.83	59597.63	58872.31	79990.36	68736.57	75793.7	60012.75	84579.32	64754.37
46863.6	42096.85	66082.63	61731.28	84863.86	75520.94	81792.56	56899.41	66713.71	46755.67
51879.79	42982.17	62635.42	62706.28	76958.13	64224.03	77548.03	56653.93	57981.69	51331.94

LP332.331	LP332.964	LP332.964	LP333.296	LP334.186	LP334.186	LP334.186	LP334.186	LP334.186	LP334.185
44629.28	12493411	55818.8	16710.02	76795.81	95963.83	69393.73	117260.2	71388.5	124936.3
42627.3	10921452	61131	18675.06	108415.6	118979	95648.65	105729.4	66072.12	131417.8
43460.2	13717734	57315.28	21894.67	86097.35	104140.9	77167.36	99292.65	63168.35	126561.4
46567.48	9428509	48103.93	14329.63	82300.74	103374.1	76317.08	102618	68105.21	132309.2
35041.83	8983783	46525.31	18629.23	86486.25	99926.14	73711.14	114214.5	65090.98	131772.1

LP334.186	LP334.185	LP334.186	LP334.186	LP334.186	LP334.186	LP334.186	LP334.185	LP334.186	LP334.185
92850.54	62358.3	84058.52	60810.39	107960.9	111330	63250.1	63226.87	101284.7	84706.01
97876.68	68153.96	91176.78	73953.03	127522.2	115023.4	75896.77	75482.13	105238.9	97043.88
87010.34	65256.35	85423.94	64994.32	114592.3	108502.6	64458.71	61813.61	97767.67	88875.24
88254.21	64438.49	87005.65	65367.97	103760	112055.8	68530.21	62195.23	99283.47	100753.9
100270.7	67383.04	89763.37	64826.62	119200.9	107924.8	68039.58	59159.4	99786.87	91708.19

LP334.186	LP334.185	LP334.222	LP334.222	LP334.222	LP334.222	LP334.222	LP334.222	LP334.222	LP334.222
93006.78	97203.05	85473.7	128883.8	124895.3	80443.83	83932	71740.5	106892.5	99860.72
100682.3	102562.3	100770.8	142746.5	119110.1	106378.7	100863.3	68129.23	108627.9	101325.4
95803.78	107430.1	102233.8	128674.5	165615.1	90940.56	126314	73101.72	117895.8	93504.08
94623.38	111143.4	85503.31	143716.2	95672.27	101022.4	97632.58	66804.4	106247.2	91423.28
107396.4	99660.19	94776.44	143540.6	150167	74623.83	96838.47	69315.1	109189.9	121498.9

LP334.222	LP334.222	LP334.222	LP334.222	LP334.222	LP334.222	LP334.222	LP334.222	LP334.222	LP334.222
121165.3	110547.5	65070.19	52348.59	141507.5	114520	97258.97	104437.4	78199.12	109694.7
110699.1	115437.5	87000.12	83056.39	154583.9	108549.1	100006.3	102852.2	80358.66	113019.7
114873.1	93001.33	72308.94	90859.81	121825	123333.2	97727.75	95847.19	80712.23	113667.6
134025.3	109951.7	72196.08	85939.29	160810.2	119605.1	97151.17	95350.98	81060.75	114236.8
103006.5	107334.3	77803.18	88013.01	151903.1	107377.7	99425.43	101149.8	83033.27	114382.2

LP334.222	LP334.222	LP334.222	LP334.222	LP334.222	LP334.222	LP334.222	LP334.222	LP334.222	LP334.222
68273.35	73011.06	96268.6	71092.98	95359.08	84620.1	107673.1	79860.24	55347.42	85338.13
68550.08	69056.8	85227.4	69194.85	93905.76	89957.89	108649	79949.43	92035.17	81410.78
80201.69	74343.62	91665.32	75182.47	91423.89	68635.83	100745.6	81358.07	85031.08	86590.85
74796.99	75972.23	91237.26	75181.07	106358	86998.55	110153.7	82144.47	89171.45	85773.8
78740.05	68029.85	89400.32	71476.46	86852.6	80977.01	106822.1	71511.79	88790.67	79844.97

LP334.222[illegible]	LP334.222[illegible]	LP334.222[illegible]	LP334.222[illegible]	LP334.222[illegible]	LP334.222[illegible]	LP334.222[illegible]	LP334.222[illegible]	LP334.222[illegible]	LP334.222[illegible]
92880.51	179923.3	87970.53	91972.49	108085.9	66796.77	83383.46	141764.2	74325.02	92773.77
87330.13	172438.1	81521.14	80828.31	109235.7	62058.75	69207.21	148413.6	73459.55	91873.7
93631.38	182911.3	98414.91	85990.52	123081.9	69860.17	73599.3	123608.4	66171.92	83667.44
94431.53	187092.3	88242.98	84061.21	108962.8	71298.06	75595.95	146902.1	74534.18	94866.2
94956.22	167678	82744.03	77169.51	105110.2	65972.04	68462.46	146758.6	67603.02	96817.37

LP334.222	LP334.222	LP334.222	LP334.222	LP334.222	LP334.222	LP334.222	LP334.222	LP334.222	LP334.222
76276.53	88953.99	112778.2	69982.61	121913.1	92912.86	89183.08	82739.49	112621	99839.04
77409.58	91632.62	114007	63980.05	130503.4	95694.2	88453.13	79137.54	101665.3	107238.5
88028.59	81943.96	109864.7	56020.19	118194.6	87891.91	84643.35	81305.38	105747.8	111889.9
83948.39	93214.72	110841.9	78583.42	135601.5	96193.17	89720.61	83309.81	111787.1	104500.6
68533.52	86948.96	109436.9	77591.68	131671	96932.01	93054.4	83540.31	109285.5	102028.4

LP334.222[illegible]	LP334.222[illegible]	LP334.222[illegible]	LP334.222[illegible]	LP334.222[illegible]	LP334.222[illegible]	LP334.222[illegible]	LP334.222[illegible]	LP334.222[illegible]	LP334.222[illegible]
134427	80335.43	75706.01	72872.66	81977.19	92371.6	94328.77	90720.08	106698.5	63719.75
128127	85457.88	63458	66269.43	77733.02	92708.76	97840.62	92561.97	98335.11	100467.1
137272	92186.48	70905.37	69363.19	76299.63	98308.46	91281.67	106832.9	100934.9	104111.9
129825	80144.29	70397.17	67549.46	74896.03	94586.69	96714.05	92911.97	77377.41	97033.36
137281.7	85087.11	69387.85	68180.55	85919.62	99823.28	87900.47	94082.53	114978.8	102828.8

LP334.222	LP334.222	LP334.222	LP334.222	LP334.222	LP334.222	LP334.310	LP334.308	LP334.308	LP334.309
95692.05	98261.14	88362.25	75000.93	80123.67	110630.5	38994.33	40842.74	50687.04	45016.36
92413.85	101451.2	83104.1	76310.41	79424.89	105266.7	39820.36	38176.18	54239.1	43206.61
70877.89	77336.53	76861.27	85886.44	84398.64	107656.2	46831.04	41709.78	69147.68	49060.85
94721.33	101230.7	89616.19	84594.63	74679.77	112938.1	45409.44	46024.32	74430.83	49930.55
99060.15	92642.16	88705.42	87051.51	74265.37	105706.6	18862.22	35228.71	71766.68	37701.37

LP334.310	LP334.96_	LP335.221	LP335.221	LP335.221	LP335.279	LP335.294	LP335.294	LP335.294	LP335.294
42304.18	2109508	41845.31	49502.6	41419.26	41175.35	20553.85	39254.35	50917.59	27684.39
39498.6	2047825	38056.07	46520.82	42457.46	39576.63	23614.14	34158.12	46684.27	29134.42
44914.58	1866745	31975.23	49488.49	52828.36	43378.04	20466.99	36103.37	46897.99	31678.9
43752.41	1603787	37167.65	52290.12	48745.36	38297.04	20243.86	42248.4	50605.26	30039.16
36794.06	1474518	35248.2	41536.11	43048.54	39066.31	20043.57	33621.84	54961.14	30799.89

LP336.221	LP336.237	LP336.237	LP336.237	LP336.237	LP336.238	LP336.237	LP336.238	LP336.238	LP336.238
106505.5	177800.4	96593.1	104781.5	140576.3	159241.9	174368.2	65188.42	154192.4	203055.7
97635.34	173691.6	79553.38	112066.1	137397.3	151651	176224.9	60375.44	145746.4	187449.3
95951.79	183204.3	96729.43	116457.6	161005.1	173075.7	186648.2	62066.23	153915.5	205641.6
86271.8	198118.9	91364.28	117993.2	141352	104643.8	139681.9	60712.9	133614.5	148104.8
93823.15	106935.8	55263.15	84808.37	149807.9	116013.3	129199.1	74213.1	105233.9	130672.9

LP336.238	LP336.238	LP336.237	LP336.238	LP336.238	LP336.238	LP336.238	LP336.238	LP336.238	LP336.238
119303.8	171505.2	140677.5	164508	154822.2	96205.26	175617.2	154103.9	127727.6	112999.6
121775.3	166002.1	140987.1	149377.2	153150.3	102466.7	197899.2	144712.7	139401.5	120594.6
134482.2	166481	149677.4	152626	166992	107102.3	194013.6	166814.1	144356.5	119910.3
145299.7	193662.9	156494.9	102553.1	164212.3	112311.5	122448.1	175033.1	138992.4	125740.6
85759.13	96402.41	95093.83	162428	108497	87817.2	123055.3	99655.29	96252.43	111886.4

LP336.238	LP336.238	LP336.238	LP336.238	LP336.238	LP336.238	LP336.238	LP336.238	LP336.238	LP336.238
130924.6	139191.4	110935.4	130106.6	131782.8	85830.29	137217.4	163904	182549	122538.4
128723	142544.8	111708.7	134342.6	130647.2	81491.79	138897.5	166653	160506.4	117308.5
138275.1	145119.3	111689	134315.5	131963.6	90913.68	142759.2	165113	184602.3	132506.8
141519.9	154649.5	107125.8	139982.1	138425.3	82256.34	139013.1	168219	173509.2	76448.4
94899.76	101493.3	110209.5	84473.05	78032.92	58890.31	103308.5	109605	137001	139189.7

LP336.238	LP336.237	LP336.238	LP336.238	LP336.238	LP336.238	LP336.238	LP336.238	LP336.237	LP336.238
92302.17	113982.6	124726.9	134406.9	126798	163743	139120.4	232374.4	137457.2	133713.7
95038.13	110171.2	119422	134122.2	133621.8	170378.5	144270.1	234482	139741.7	134478.4
87404.86	114747.3	127641.8	151987.1	122325.8	173987.5	156888	228790.5	138893.7	139853.3
96230.75	107070.9	125977	144810	75873	178545.4	147058.2	246358.3	147203.7	142345.6
61695.1	67061.77	75610.29	145266.1	126778.1	186470.8	88733.15	144617.2	86342.96	83305.21

LP336.238	LP336.238	LP336.238	LP336.237	LP336.238	LP336.237	LP336.238	LP336.238	LP336.238	LP336.238
155990.3	136769	117898.2	145351.1	153938.8	163708.5	85372.08	160447	97420.54	105559.6
158889.6	130178.3	123659.1	139572.2	145774.3	173302.9	83776.19	179990.8	95996.58	110413
160900.7	135137.5	115939.8	146248.9	149999.3	180255	89375.16	186415.7	85276.71	121565.5
166167.2	139336.1	131672.9	148450.3	160119.5	180274.2	90200.78	185429.4	94088.82	114792.2
105911.2	82872.24	76492.07	91035.48	88497.3	110192.2	60231.25	102069.9	71869.55	80929.73

LP336.238	LP336.237	LP336.238	LP336.238	LP336.238	LP336.238	LP336.237	LP336.237	LP336.238	LP336.311
84840.68	121658.1	177293.9	100873	181276.4	107142.1	118033	153819.3	220037.1	17062.84
87120.21	129810.5	169304.5	108094.2	177883.8	107314.6	120564.5	160826.6	204835.4	16740.57
88163.5	136390.7	173396.5	110348	185178.3	110685.2	128127.8	163305.7	214843.6	15611.98
87413.51	132446.5	189628.3	107606	188947	112412.9	92101.05	117768.5	126171.1	16574.07
89356.35	135779.9	135298.4	70698.43	114048.9	84673.03	107579.4	105554.1	229160.1	12685.52

LP336.326	LP336.326	LP336.326	LP336.955	LP337.200	LP337.224	LP337.236	LP337.236	LP337.236	LP337.236
156171.2	274689	74830.89	132763.4	182070.1	33652.84	42195.9	44229.91	42033.32	38409.78
140581.3	328071.6	83571.31	124599.5	254249.6	32493.7	36692.33	45491.52	33510.62	33614.63
108897.1	242912.5	77114.87	115812.4	266483.6	32446.33	30095.93	39425.87	41373.27	35398.56
190617.8	172780	79218.09	107234.4	250381.3	17913.32	34158.39	43630.1	34507.4	35375.89
126549.6	240766.3	71531.57	95572.39	291799.1	21036.52	36476.57	40047.41	35636.37	39804.62

LP337.236	LP337.253	LP337.271	LP337.274	LP337.274	LP337.273	LP337.274	LP337.292	LP337.308	LP337.310
30634.9	38955.18	102401.3	49909.32	67279.41	39362.67	50327.3	48043.11	103968.8	42897.51
38370.95	40179.67	104175.1	40394.27	59764.81	38464.19	47741.18	46935.69	122780.7	46895.4
45342.67	40899.1	121934.3	43815.57	62996.55	39326.98	50749.74	49718.19	109604.3	41437.7
46161.43	44256	127009.5	43374.67	62716.4	40635.21	46460.37	51655.08	103777.3	45184.07
36607.75	43219.12	130584.3	43862.33	68441.85	41539.96	48347.92	45077.56	111666.5	43134.68

LP337.310	LP337.310	LP337.310	LP337.310	LP338.172	LP338.232	LP338.232	LP338.232	LP338.232	LP338.232
56439.01	56638.18	48987.21	53642.32	67746.25	122734.7	144381.2	157973.4	131403.5	117222
47963.59	60639.25	54148.47	50643.07	67852.2	124620.6	116780.1	162750.4	162967.2	120696.5
49358.12	58971.13	48061.74	53056.16	67320.44	117587.9	125105.2	128802.9	160101.8	122201.6
53185.92	57541.29	44807.1	49278.67	67634.37	127354.3	139645.1	149784.9	172594.5	104007.6
49766.95	51504.83	48783.8	51767.65	70898.53	120959.2	136442.5	176473.1	169667.1	109065.5

LP338.232	LP338.232	LP338.232	LP338.232	LP338.232	LP338.232	LP338.232	LP338.232	LP338.232	LP338.232
177767.5	168865.3	170380.7	117619.2	86541.25	83896.8	95826.29	95427.68	127740.9	95496.35
178814.1	164746.4	84346.1	94231.54	86843.63	83854.7	90390.72	91836.69	123942.2	101828
173799.3	174760.6	78624.14	92393.12	92620.83	73734.87	85571.71	92913.33	127487.2	92321.67
200806.7	166902.2	81058.04	100487.5	87034.24	84948.41	96902.78	96175.55	128538.2	81405.15
172488.3	163186.4	84695.39	98820.93	94523.12	81291.66	92672.69	92854.65	118942.3	115934.5

LP338.232	LP338.232	LP338.232	LP338.232	LP338.232	LP338.232	LP338.232	LP338.232	LP338.231	LP338.232
171822.4	108798.2	107633.3	44385.69	83328.85	212770.4	90752.31	114555.8	59080.78	104361.3
156030.1	112262.9	114458.9	50829.89	96021.99	212472.1	102784.7	113987.7	54603	98882.16
147346.7	104556.3	113496.7	42201.53	84322.04	198300.5	96176.57	117856.6	55076.03	102793.7
156735.1	114520.5	108973.3	43671.58	81040.68	222637.6	90450.65	121211	61619.4	112138.1
155011.9	111391.2	108209.6	51161.21	82509.18	233305.8	89509.56	124533.6	58678.75	107104.4

LP338.232	LP338.232	LP338.233	LP338.232	LP338.232	LP338.232	LP338.232	LP338.232	LP338.232	LP338.232
181688.4	150567.9	130228.8	193537.2	188563.2	35751.97	101087.1	151059.2	199750.5	146979
166296.1	141302.7	148686.9	204151.1	181318.5	34442.56	98968.46	172910.8	198666.2	98569.66
162129	142875.2	142248.1	194383.6	165393.6	40181.7	93699.77	159543.9	182575.6	102868.7
175494.9	139485.8	153192.7	195383.3	175043.4	33715.69	170433.9	179300.4	207956	103102
162063	133996.7	158800.5	232582.9	172679.3	33680.63	105701.8	171210.7	187249.8	98079.78

LP338.232	LP338.232	LP338.232	LP338.233	LP338.232	LP338.233	LP338.232	LP338.232	LP338.232	LP338.233
156082	167400.5	160078.7	135254.7	168694.5	158353.6	168973.9	175737.3	151925.7	139236.5
155130.7	150796.4	158054.7	145784.1	166157.4	157114	168297.4	181521.4	153584.3	152834.7
154604.9	156596.8	158353	145064.8	169667.4	158670	168779.6	174687.9	156244.9	137352
159933.1	172149.6	156708.2	155387	178247.6	160724.9	158381.4	172531.1	152976.2	142434
155121.4	183947.4	173332.6	147616.1	169975.1	169375.9	156692.4	177965.9	144071.9	143720.6

LP338.232	LP338.232	LP338.232	LP338.232	LP338.233	LP338.232	LP338.232	LP338.232	LP338.233	LP338.232
105507	75715.44	101938	210906.3	171219.9	78111.57	120905.4	68682.51	157657.6	118359.1
119020.8	78700.96	106454.4	176309.7	189549.8	80840.49	127944.5	67350.42	139362.5	113206.8
103484.6	77279.77	116370.8	183597.9	188840.2	73415.91	115939.1	64194.2	146248.3	112748.7
113428.7	81909.33	125226.5	188622.9	181620.3	71722.02	122921.5	61282.97	151682.2	127790.9
120214.7	77465.53	108889.5	186323.1	192646.7	72162.72	121991.8	67115.28	156163	117833.7

LP338.233	LP338.266	LP338.267	LP338.275	LP338.311	LP338.332	LP338.341	LP338.341	LP338.341	LP338.341
103289.1	25993.03	20874.88	240675.1	21768.85	34152.29	4501113	23842389	4694090	708717.7
101628.4	44504.6	24848.91	222822.9	47899.42	31919.17	3259902	21299327	4576206	568325.4
98414.02	26856.56	23246.42	210595.2	50736.75	33621.42	4462720	27411226	6515451	655305.7
94779.66	26246.75	15648.79	205911.4	54062.15	31075.25	4631998	23561842	6719237	690852.7
94748.41	23678.25	28688.58	183511	29313.56	37881.36	4834711	21947333	5021506	579886.5

LP338.341	LP338.341	LP338.341	LP338.341	LP338.342	LP338.341	LP338.341	LP338.342	LP338.342	LP338.341
471618	669872.1	504974.9	577182.2	656266.4	643743.6	503610.9	793810.9	713518.1	4171099
462242.5	581830.7	564958.5	559369.8	768088.3	635672	599405.6	624183.4	591761.5	2559011
429083.3	645293.3	770359.1	590325.8	908946.6	917928.2	568011.5	944926.2	498237.6	2974784
547058.3	817685.6	492744.6	532961.7	593975.4	745578.4	689643	584614	443169.3	2799724
896419.9	430838.8	625116	476368.9	857505.2	661601.4	447842.7	834119.1	561574.4	2636380

LP338.342	LP338.341	LP338.341	LP338.341	LP338.341	LP338.341	LP338.342	LP338.341	LP338.341	LP338.342
928393.5	696736.5	845080.3	609586.5	910196.7	872824.3	843012.4	549454.7	614665.9	673244.8
696608.8	620860.5	839213.1	468491.5	752669.7	871644.1	821991.6	485393.1	673641.6	700320
754224.3	518852.8	934778.6	536907.9	767024.9	667157.7	807551.9	594404.5	541601.3	688678.3
576199.6	602135.9	842540.6	521265.3	1146953	849469.3	796626.3	642169.5	525173.7	615130.5
819613.4	579244.2	710857.2	491397.5	673577.2	1011417	818447.6	569723.3	494618.2	725864.6

LP338.341	LP338.342	LP338.342	LP338.342	LP338.341	LP338.342	LP338.342	LP338.342	LP338.342	LP338.342
814007.8	547878.5	574389.8	566821.9	430285.2	649219.8	535552.6	711004	522103.4	433504.2
703326.4	692915.7	644818.4	604694.7	423651.4	691664.6	680641.7	695195.8	392107.2	476107.5
529035.8	475940.2	632669.9	569327.1	546715.5	598527.4	718362.9	653984.2	564266.3	528715.9
527103	563605.1	530358.9	434258.8	428739.8	827032.6	589692	768455.1	718843.7	556645
565742.8	824912.1	708076.9	559587.9	380471.5	489904.3	580729.7	586515	459912.1	642234.6

LP338.342	LP338.342	LP338.342	LP338.342	LP338.342	LP338.342	LP338.342	LP338.342	LP338.342	LP338.342
680382.1	537570.6	479527.7	837158.3	576640.9	797314.8	763317.1	589678.6	407089.2	618564.5
581725.8	507087.4	459571.7	597710.3	788877.7	470648.6	791394.4	588569.1	420535	680276.6
560148.2	695097.2	507970	590050.7	546501.5	625525	637964.7	597920.4	428112.7	641618.8
678259.1	524728.6	590460.2	801157.6	644345.3	484588.7	679491.6	576598.6	406074.4	926419.1
561697.4	485249.2	434609.7	530337.3	483117.7	501123.5	551762.3	715702.5	441766	490584

LP338.341	LP338.341	LP338.342	LP338.342	LP338.342	LP338.342	LP338.342	LP338.342	LP338.342	LP338.342
484192.5	192654.7	645000.6	668484.3	528898.6	418619.5	511311.6	396077.4	496141.1	518718.3
631306.2	127332.7	558239.9	731355.8	523329	605078.5	522342.3	361715.3	475797.5	490625
654955.1	144556.5	611434.7	674895.9	511007.1	436664.2	548716.3	372218.5	489731.8	539870.6
466465.1	116764.4	453543.9	645039.8	492262.7	466550.8	536350.5	365773.8	480791	548221.9
547001.4	113517.7	820291.7	693682.4	488915.1	465817	546392.2	519676.8	362729.8	466172.7

LP338.341	LP338.342	LP338.342	LP338.341	LP339.141	LP339.141	LP339.141	LP339.141	LP339.141	LP339.141
145442.4	506070.7	358433.1	996108.8	58778.85	63324.72	62845.83	76910.93	72882.46	91876.78
149705.5	505403.6	332366	1019633	71243.26	55469.61	82146.88	51299.23	69791.89	68841.51
143230.6	548780.7	328422.3	1087470	59939.98	63042.92	56537.71	52874.95	68421.11	73871.08
143371.7	559237.9	365844.7	1019361	75838.88	79032.54	94343.68	68314.43	72083.04	74494.5
159953.1	522036.9	402759.7	1089298	73684.96	66956.6	58173.44	65135.83	80271.9	62873.93

LP339.142	LP339.141	LP339.141	LP339.141	LP339.141	LP339.141	LP339.141	LP339.142	LP339.141	LP339.141
99771.51	74809.69	31321.86	66469.95	70215.45	68762.78	64376.4	79283.44	63435.77	66255.93
76029.56	70386.83	38345.32	62343.35	88109.11	62234.63	61255.7	62203.03	56544.45	77766.03
78770.79	72283.73	44655.64	88680.48	105745.6	60334.9	77369.45	95260.97	76708.48	67032.56
81919.32	86086.54	28130.85	64451.59	50639.16	71013.25	94038.13	98144.72	66741.82	59800.67
65200.87	102844.1	44018.48	49230.75	71276.86	82958.22	83763.49	100194.8	74377.71	72212.4

LP339.1417	LP339.1419	LP339.1418	LP339.1419	LP339.1419	LP339.1419	LP339.141[illegible]	LP339.141[illegible]	LP339.1414	LP339.1418
68678.43	59664.58	87323.28	69400.23	51837.97	53324.84	62191.49	64650.56	50418.51	69646.69
93197.71	81106.72	68847.05	90235.97	74534.22	54520.9	68440.32	88688.39	60794.43	40016.52
74631.82	68152.42	110425.8	90845.1	75446.76	52822.28	69861.85	60301.23	65913.3	70681.72
53288.49	65768.92	65554.01	61267.48	73463.14	64842.13	52068.83	55464.74	62571.44	58204.56
96976.8	88563.2	61167.19	78834.63	64041.17	74306.95	70756.86	78488.58	106905	45137.8

LP339.142	LP339.141	LP339.141	LP339.141	LP339.141	LP339.142	LP339.141	LP339.141	LP339.141	LP339.141
63359.36	65406.23	87121.01	49700.43	59424.71	105394.3	69181.95	75662.08	62818.4	95216.6
79758.96	100878	67808.42	72338.95	85673.48	60365.73	84253.52	68895.35	86938.04	77816.63
90308.77	91819.31	86864.73	54790.97	74218.18	70061.95	66226.59	77674.74	61681.61	102975.7
67464.31	75696.63	67110.51	74046.75	58310.83	71734.42	96457.55	60170.64	92234.05	70048.19
89904.52	66660.35	62724.2	58410.72	57675.61	71568.1	92755.93	65121.64	66875.73	83313

LP339.141	LP339.141	LP339.141	LP339.141	LP339.141	LP339.141	LP339.141	LP339.141	LP339.141	LP339.141
66014.7	51124.64	27064.51	76838.06	80332.46	83496.09	55864.97	33794.76	56998.89	49149.59
69425.44	68158.86	34129.29	60407.99	71735.77	82597.69	80570.26	28479.43	108638	69957.45
78745.14	65244.13	28772.74	70356.66	63071.34	64336.27	55428.89	35247.43	66545.64	91140.9
61304.14	56637.03	28148.28	56370.29	102045.2	63358.39	57063.43	38585.07	63215.13	58640.38
48459.22	87078.24	31709.12	67927.05	85942.42	75416.98	64062.91	27748.12	75595.52	80073.19

LP339.142	LP339.141	LP339.141	LP339.141	LP339.141	LP339.141	LP339.141	LP339.141	LP339.141	LP339.141
73433.48	48196.14	62230.47	65231.76	67050.11	66311.98	86794.71	62407.72	33751.55	94105.04
97501.3	52236.54	61669.35	77965.56	65281.34	64661.86	85675.03	63562.52	42244.44	76772.25
67726.56	103039	65946.57	64215.07	92035.61	96143.71	88474.51	69291.69	35905.92	67363.96
71064.18	75812.54	70621.93	66473.16	82352.82	56832.14	83521.58	49798.72	34234.06	85345.11
77986.78	63141.64	60917.5	79092.38	69058.52	104988.4	72640.32	73457.7	41962.75	84846.66

LP339.141	LP339.141	LP339.141	LP339.142	LP339.142	LP339.141	LP339.141	LP339.141	LP339.141	LP339.141
59867.96	56847.65	65654.53	87263.9	66757.36	70652.38	65660.77	58059.02	67807.49	51479.82
60147.21	46337.04	77378.94	86845.64	101692.3	93669.44	70828.92	48162.22	55034.85	47737.2
57574.17	46657.38	59535.36	81380.18	62949.95	77500.52	61404.29	52150.52	51415.87	58571.69
57453.39	38216.36	70670.42	93325.53	74074.58	67165.09	63958.88	52838.7	53296.65	79150.76
75958.19	54734.73	79015.71	90876.14	74899.37	74754.76	56013.61	54585.36	56053.28	57472.38

LP339.141[illegible]	LP339.141[illegible]	LP339.141[illegible]	LP339.141[illegible]	LP339.141[illegible]	LP339.141[illegible]	LP339.141[illegible]	LP339.141[illegible]	LP339.141[illegible]	LP339.141[illegible]
68935.78	35080.87	45279.81	37055.12	43830.88	37988.94	69142.17	33302.87	44174.49	50230.76
61177.75	45386.17	58007.92	34487.06	45722.49	38249.8	64096.74	48258.88	72743.46	59449.92
62306.88	35857.4	69461.52	42742.68	32736.37	25273.6	51913.65	48679.01	69926.72	53893.84
89989.12	30665.38	66174.35	26339.19	46529.75	29436.83	46173.99	27184.91	53503.27	54574.82
84643.46	43399.49	61212.72	27646.4	46439.85	24128.15	51195.2	28611.92	55480.83	52689.68

LP339.141	LP339.141	LP339.141	LP339.141	LP339.142	LP339.141	LP339.179	LP339.178	LP339.178	LP339.178
59173.88	66182.37	70450.97	44581.1	67853.15	49552.09	36648.13	45349.81	34746.74	54413.68
51221.57	68884.8	64290.49	54912.92	80408.94	43745.02	34380.32	41796.87	38465.03	91177.73
47584.8	69966.19	46789.55	46868.64	67898.4	58184.32	43923.13	43638.91	54192.68	68609.95
57933.61	66952.01	46102.84	40453.22	68135.85	53619.95	37593.43	37881.1	39427.69	55729.17
48208.88	72314.14	49733.99	68229.3	64079.78	36783.98	27577.66	43036.64	51498.21	72404.28

LP339.178	LP339.178	LP339.178	LP339.179	LP339.179	LP339.178	LP339.179	LP339.179	LP339.179	LP339.179
39127.47	58468.88	40174.44	34906.6	65904.31	78885.21	67989.21	58073.66	36315.44	87197.86
36318.93	60435.48	43742.35	36269.99	65439.8	82835.51	70131.59	57770.78	39208.53	65287.94
36831.93	80194.97	45072.16	38208.37	45951.28	87160.65	77342.86	56144.27	34505.53	89770.79
41388.56	61576.96	40750.8	37249.03	100998.4	86021.73	73348.65	59070.94	39353.36	83904.53
35758.6	38493.82	50009.95	34559.34	68554.7	49217.47	65689.03	56513.94	38171.08	90959.36

LP339.178[illegible]	LP339.178[illegible]	LP339.179[illegible]	LP339.179[illegible]	LP339.179[illegible]	LP339.179[illegible]	LP339.179[illegible]	LP339.178[illegible]	LP339.178[illegible]	LP339.178[illegible]
57805.07	45346.05	33494.08	67431.63	85286.55	60726.57	69435.18	69179.77	64737.05	42255.96
60692.9	39781.68	31181.75	67541.57	90058.2	55294.65	66511.23	76093.24	67370.58	40865.87
58864.72	52510.71	31001.08	63507.66	86804.18	58991.28	63283.73	67142.61	67533.67	44554.77
57987.08	42654.85	28681.73	72593.23	81951.8	62153.71	59948.7	66687.57	69333.9	45490.67
57253.98	53917.52	30885.4	69524.27	80878.48	61676.82	66429.17	74249.4	66522.94	44661.19

LP339.178	LP339.179	LP339.178	LP339.178	LP339.179	LP339.178	LP339.179	LP339.179	LP339.179	LP339.178
46185.71	51945.8	56523.88	52375.02	23336.69	44766.28	69501.69	49693.34	73039.68	66709.66
49804.14	42934.72	55863.35	48110.59	26970.12	53241.28	65939.8	49640.26	72388.48	66356.86
42430.23	46824.25	55512.78	49271.61	26648.89	48437.67	80449.37	61356.8	73079.32	67736.62
45439.75	51747.03	60830.05	49290.96	26535.86	50486.92	62843.51	58151.19	72209.87	69744.05
50418.26	38063.3	50816.69	50652.15	25266.11	47352.78	66618.37	54230.75	70758.97	74158.79

LP339.178[illegible]	LP339.179[illegible]	LP339.179[illegible]	LP339.179[illegible]	LP339.178[illegible]	LP339.178[illegible]	LP339.178[illegible]	LP339.179[illegible]	LP339.179[illegible]	LP339.178[illegible]
61365.25	46920.23	69650.93	64095.73	75435.5	36322.17	59628.27	46108.22	91185.72	48006.45
61633.07	47107.86	60959.27	59994.97	69740.74	44072.6	58253.01	44959.33	83873.33	49739.09
68203.91	48599.55	60755.2	64525.86	74535.45	48220.93	58369.6	43189.05	79260.39	54949.73
60863.3	45353.58	64576.67	58894.38	78245.39	44086.69	59483.81	40601.05	77035.96	44520.54
58548.03	53209.3	59343.48	56812.51	73075.91	43843.06	55253.96	42221.83	76780.86	46284.74

LP339.179	LP339.179	LP339.178	LP339.179	LP339.178	LP339.179	LP339.179	LP339.178	LP339.179	LP339.179
53541.09	84743.83	39428.64	68101.52	52288.89	58381.72	75861.3	56078.44	42018.35	32005.9
52158.24	82240.34	42846.68	69776.01	49442.33	54909.37	73273.13	52234.1	46777.17	35632.86
52620.83	91633.07	43106.35	67325.68	47738.2	58563.12	69043.11	61658	49770.44	33240
51628.32	86307.1	44324.4	64437.74	48816.21	55325.1	75983.28	57822.3	45831.16	35965.75
50106.62	79109.08	41002.54	72126.47	48751.89	53352.6	78256.39	52997.79	44314.32	30426.72

LP339.179	LP339.178	LP339.178	LP339.178	LP339.178	LP339.179	LP339.179	LP339.178	LP339.179	LP339.178
75194.91	70098.51	52473.81	67184.77	53368.1	54517.56	55351.36	68980.36	42612.96	65259.01
73436.62	72404.84	50183.11	64865.02	54286.58	49003.73	50515.32	66849.7	42105.36	66877.84
75641.61	71702.64	52091.16	67501.24	57065.85	44528.97	55386.94	72449.91	42595.26	71227.28
76242.79	66381.54	51248.44	67110.34	50559.26	49603.95	57439.03	65425.38	44497.23	63242.63
73099.3	67662.25	41552.38	62897.26	54790.34	52562.93	56226.77	72463.72	39482.04	75904

LP339.216	LP339.215	LP339.216	LP339.215	LP339.216	LP339.216	LP339.216	LP339.216	LP339.216	LP339.216
48317.25	50601.6	44085.64	45701.08	46876.15	67154.31	57086.32	61652.6	60342.98	58011.22
48601.52	47191.76	48649.15	51819.19	37817.44	62428.98	52253.66	59570.14	59666.49	56266.38
46742.39	49950.54	53927.78	53016.86	41881.66	70187.19	52585.23	64827.28	62421.74	59293.83
48854.05	44754.92	45090.93	48727.36	39247.07	69309.23	54805.79	67839.75	59389.45	59096.43
52838.57	42486.61	47530.48	52583.19	45058.05	73404.46	51569.57	67890.49	63719.54	57031.28

LP339.216	LP339.216	LP339.216	LP339.216	LP339.216	LP339.216	LP339.216	LP339.216	LP339.216	LP339.216
56266.99	46958.22	70157.21	58591.32	45377.17	68849.48	53434.31	53148.7	42277.17	49134.57
49958.09	52075.72	68785.95	63194.32	42353.02	66045.83	49569.99	51974.49	39624.67	57538.81
51684.79	48579.31	76020.53	64135.05	40923.37	62042.59	46532.56	53911.94	47150.99	55450.51
55298.8	46688.12	71960.68	54806.06	39698.6	61879.85	51289.52	49692.35	44947.05	52289.72
55957.71	52016.47	70756.02	56574.14	45474.43	58626.35	46567.19	56239.68	44917.22	52857.21

LP339.216	LP339.216	LP339.216	LP339.216	LP339.216	LP339.215	LP339.216	LP339.216	LP339.215	LP339.216
39369.21	35913.56	44501.33	61857.77	50666.12	51540.19	49752.91	84044.65	68705.68	59322.12
34495.86	35777.89	39581.19	57883.69	60955.78	48885.31	51002.26	85104.85	72084.98	64056.44
42667.42	35645.19	42642.13	55950.89	60160.41	51878.7	56218.35	90022.69	61341.08	63045.43
38937.8	35340.45	47247.05	57321.85	53929.21	50131.72	56763.29	82397.12	68108.66	60330.72
41318.09	34862.49	43509.3	55307.24	55915.34	49502.34	53540.97	94123.49	72085.33	58989.19

LP339.216	LP339.216	LP339.236	LP339.236	LP339.236	LP339.289	LP339.289	LP339.326	LP339.326	LP339.325
66443.75	56488.03	71101.03	69745.52	45757.28	103132.9	74009.26	117943.9	45439.39	52229.35
66745.97	53134.95	69728.39	66934.44	40993.75	104989	82194.18	177577.9	152097.1	103943.2
66909.87	59000.33	73468.02	67872.87	48248.37	101260.9	73724.04	103649	60711.99	56345.4
67713.89	59018.99	69251.09	69647.65	43138.48	96750.09	84045.72	203498.4	169293.4	118210.2
68729.81	57969.01	71772.29	68894.78	47091.74	101293.3	80491.94	227040.9	170630.6	125883.5

LP339.326	LP339.326	LP339.326	LP339.326	LP339.326	LP339.326	LP339.326	LP339.326	LP339.326	LP339.326
97166.03	118932.5	66501.78	61357	31262.32	72601.14	40233.84	37804.9	47497.62	120084.9
180189.5	211361.6	156690.9	166092.3	86733.09	121432.5	116695.1	98453.18	77651.04	122651.3
86036.76	95809.14	62308.87	94897.87	73905.68	80779.68	43738.83	52744.24	42290.85	72326.36
200214.1	244881.4	178008.4	178375.4	96549.93	156578.6	135788.9	111152.2	98189.45	135006.9
210670.8	234480.5	191385.7	185364.7	95547.73	141366.6	126394.3	121483.1	95775.88	132416.3

LP339.326	LP339.326	LP339.326	LP339.326	LP339.326	LP339.326	LP339.326	LP339.326	LP339.326	LP339.326
74514.3	46628.34	54980.85	47376.65	61433.23	53351.94	52752.27	66956.77	42228.4	62070.1
123927.3	115650.2	114467.5	68620.1	144300.8	147923.5	92526.47	146795.2	100037.1	149070.1
71046.09	45180.18	58679.23	37075.83	62329.42	77264.14	49527.76	80096.36	71931.56	97366.73
127120.4	141633	128237.6	73703.16	161107.1	160301.9	101769.9	170137.5	115146.6	154494.3
137779.2	127876.6	139672.7	67513.24	164420.4	171394	102647.8	173918	103492.4	152489.1

LP339.326	LP339.326	LP339.326	LP339.325	LP339.326	LP339.326	LP339.326	LP339.325	LP339.326	LP339.326
62259.74	49505.1	90582.81	55870.23	48271.41	59734.66	87139.45	39714.5	58839.63	46532.25
148855.8	126679.3	90096.97	125806.7	117769.6	118317.1	125913.1	94856.96	107382.6	101163.2
52315.38	60970.65	50758.03	54405.44	61345.38	59121.18	66268.92	52144.02	84918	48760.87
148599	138032	100461.8	129856.7	124580.7	136157.2	136161.4	104447	142981.1	116734.7
151755.9	138544.7	95677.9	128130.2	125552.1	140274.4	134110.8	91085.36	144842.4	118685.5

LP339.325	LP339.326	LP339.326	LP339.326	LP339.326	LP339.326	LP339.326	LP339.326	LP339.326	LP339.326
48266.19	46068.65	62876.94	104153.1	38480.25	69398.23	55069.6	57729.91	71232.43	55227.59
120041.1	105116.1	139254.8	114790.4	88468.53	166961.7	132147.5	123947.5	153791.7	116715.9
54405.14	59015.92	62913.82	64843.13	29705	61739.66	44550.67	58146.1	172112.2	66530.67
124696	107488.1	152761.8	139799.6	106711.1	190360.1	128095.7	143271.6	169660.4	128410.1
127758.4	115649	156783.1	136568.8	104347.8	209124.6	134817.1	129645.5	171548	132764.9

LP339.325[illegible]	LP339.326[illegible]	LP339.326[illegible]	LP339.326[illegible]	LP339.326[illegible]	LP339.326[illegible]	LP339.326[illegible]	LP339.326[illegible]	LP339.325[illegible]	LP339.326[illegible]
43098.25	48869.83	42112.79	57088.14	74596.77	43378.55	63631.01	144835.6	56835.67	484413.8
99123.43	143286.1	118795.1	129669.7	166180	126519.4	160121.4	133762.1	142071.3	519536.9
42626.5	144274.6	71270.21	70836.09	162702.7	46347.72	170766.4	80247.74	49677.3	102590.9
110380.6	156119.6	135251.4	141359.7	172944.3	136214.4	160592	157123.1	126440.3	554176.3
103642.2	159097.3	124979.2	145351.7	170243	124501.2	175592.5	159781.4	119299.8	525003.1

LP339.345	LP339.345	LP339.345	LP339.345	LP339.345	LP339.345	LP339.345	LP339.345	LP339.345	LP339.345
1171003	956187.4	574377.9	127477.1	122230.9	181834.9	151289.2	107420.8	155764.1	138703.7
974611.9	585701.5	724001.9	127580.4	138442.8	185166.6	138748.4	151815.2	153554.1	151581.8
1422222	1241716	622345.3	133169.5	134088	177781.2	129976.8	104066.6	158581.2	135918
961903.4	937175	533357.1	97398.54	128904.9	173767.3	133697.4	107931.1	146439.9	195321.7
702934	748198.5	637743.8	110459.5	123504.5	165443.9	153118.9	111326.3	154946	138756.6

LP339.345	LP339.345	LP339.345	LP339.345	LP339.345	LP339.345	LP339.345	LP339.345	LP339.345	LP339.904
154117.5	141711.5	109010.1	173821.5	240875.4	170361.3	139630.1	184814.4	118302.1	26146.38
164196.3	164400.5	104771.2	176614.9	252571.8	247242.5	139434.5	175887.8	127756.7	26220.09
148138.9	135636.4	103956.8	172681.1	248768.6	157456	140570.6	181162.2	137399.5	33345.77
150794.8	146666.7	109095.8	144919.3	249669.5	166567.1	152038.8	184031.1	120776.3	35772.69
158220.8	138339.9	116070.2	152527	269836.9	149761	146854.3	177694.9	124862.2	26548.89

LP339.904	LP340.196	LP340.196	LP340.196	LP340.196	LP340.196	LP340.196	LP340.196	LP340.196	LP340.196
29426.72	82530.95	74100.06	70940.75	66390	75969.04	70849.56	89911.44	114423.1	48825.23
30718.13	89158.18	78415.6	65529.37	89072.02	69670.58	83716.21	94086.5	107801.6	55706.85
34352.41	91957.47	81721.13	57727.06	85273.34	68976.34	86614.02	93807.45	115381.4	47399.1
29896.46	122669.3	82142.53	63907.21	81015.85	69984.68	83820.3	94976.1	113236.6	50792.4
26835.53	88963.98	74787.95	57632.65	81857.68	72485.55	85706.46	86603.94	117443.3	46633.66

LP340.196[illegible]	LP340.196[illegible]	LP340.196[illegible]	LP340.196[illegible]	LP340.196[illegible]	LP340.196[illegible]	LP340.196[illegible]	LP340.196[illegible]	LP340.196[illegible]	LP340.196[illegible]
78107.68	91190.47	65855.32	57114.73	61345.98	96039.02	81986.34	81441.82	62373.05	84713.5
82390.62	95363.81	76923.48	42491.37	63077.03	86164.78	65534.65	77516.51	71210.19	83640.93
81789.06	88262.69	66799.7	46324.27	68721.68	85683.64	70137.86	75870.74	55086.75	84401.68
88839.09	90748.53	64409.21	43768.52	63016.39	89954.03	77340.73	72673.97	61250.99	93120.21
91132.48	91658.18	73165.59	45476.31	66462.42	84401.59	71022.14	77382.87	54538.36	82384.66

LP340.196	LP340.196	LP340.196	LP340.196	LP340.196	LP340.196	LP340.196	LP340.196	LP340.261	LP340.320
61439.89	70048.11	63231.71	70398.31	89253.67	65340.69	62443.49	63390.58	45122.48	27567.29
58358.92	61997.72	56171.32	66787.86	95337.34	57174.58	65757.35	73139.86	14973.74	32822.54
62377.79	66234.41	63857.51	68477.19	91846.78	50702.89	68240.3	67836.99	24278.44	37533.8
60849.79	68009.07	58338.49	73911.51	95096.23	54680.1	67218.97	71703.84	31726.09	40364.06
64250.59	68772.44	63095.22	72943.03	86773.02	56330.37	62569.13	68386.38	33218.17	41824.54

LP340.357	LP340.357	LP340.357	LP340.357	LP340.393	LP341.157	LP341.157	LP341.157	LP341.157	LP341.157
126689.1	248137.8	11531.96	9988.598	120244.6	143570.5	166479.6	151445.1	155512.5	186728.8
135392.1	277538.5	17791.99	12696.02	134088.7	248116.6	200050.1	160008.9	164472.5	211176.5
162454.4	409327.4	19341.13	13149.33	199117.4	121833	113068	172768	259300.2	271831.3
174482.6	488480.4	16976.18	15015.31	241520.8	250723.6	155909.1	161200.8	193741.9	131263.9
182045.8	527536.7	22824.18	17192.42	260650.8	174023.2	172630.3	129363.3	140438.5	199605.6

LP341.157	LP341.157	LP341.157	LP341.157	LP341.157	LP341.157	LP341.157	LP341.157	LP341.157	LP341.157
125552.9	161651.1	158286	169039.2	155538.6	143885	177059.5	127194	138184.9	138298
173853.5	201978.2	112877.6	165701.5	120150.4	198142.4	150926.4	235614	204739.4	163051.7
111045.9	122906.2	187327.9	208211	115786.6	126958.6	125230.9	147492.8	172360.3	112126.3
152782.8	245043.4	141967.3	248386.8	145280.1	155972.9	188950.8	148501.7	126626.4	110251.2
103270.6	140036.4	147201.7	165401.3	171497.6	225156.8	178870.9	174000.5	175358.8	161050.7

LP341.157	LP341.157	LP341.157	LP341.157	LP341.157	LP341.157	LP341.157	LP341.157	LP341.157	LP341.157
138353.4	215592.2	136613	94714.33	115959.5	57869.28	66152.72	169290.8	175715.5	123962.5
122568.4	170546	120354.7	101738.2	109931.7	60937.12	63657.91	128512.4	180315.3	125292.9
144722	249838.5	199110.8	99530.13	82977.72	49417.63	88639.85	90235.25	160283.4	127210.2
110738.8	192218.1	94838.79	134526.8	119299.2	45025.32	70475.28	128438.5	182993.4	105258.7
116355.8	205237.3	110163.6	105260.8	119153.6	75955.01	80500.05	135127.1	169057	125921.1

LP341.157	LP341.157	LP341.157	LP341.157	LP341.157	LP341.157	LP341.157	LP341.157	LP341.157	LP341.157
226091.9	193821.1	212186.5	141792.9	212662.5	145515.2	125942.3	119571.5	193253.6	187946
128063.6	173595.4	212177.9	222969	214728.5	175186.6	147951	139431.5	223580.9	224066.9
189146.1	120742.3	147280.3	124377.7	191854.3	139921.4	133916.4	158413.2	172721.9	289157.5
218423.5	241592.1	176316.7	165152.1	124898.1	132008.5	167384.5	117402.2	206923.8	178672.9
219913.6	270024.8	210242.4	171852.8	198542.6	181047.6	207067.6	121841.5	222739.9	148151.5

LP341.157	LP341.157	LP341.157	LP341.157	LP341.157	LP341.157	LP341.157	LP341.157	LP341.157	LP341.157
247870.3	177201.1	142506.2	178551.8	136480.9	217760.1	167042.1	162101	137716.1	162266.4
171518.6	179114.6	133272.1	147302	196543	126938.8	183935.1	111452.4	113601.5	159161
167573.1	182436.2	111484.7	211167.3	158820.1	189939.4	119788.7	140392.4	155011.4	197230.9
223854.6	127523.5	143686.2	198519.6	203839.8	179390.4	140162	112977.6	137544.3	181563.8
165827.9	231277.2	106798.4	184142.1	147117	175734.8	160942	141748.2	170554.4	176581.9

LP341.157	LP341.157	LP341.157	LP341.157	LP341.157	LP341.157	LP341.157	LP341.157	LP341.157	LP341.157
193111.7	139014.9	175128.5	172032.4	141356.7	208961.5	138184	173860.9	201883	172059.3
301582.6	179460.4	178713.6	191100.6	230704.7	179804.7	130658.3	174185.1	225238	233603.7
173485.2	151771.7	167969.3	182755.7	150970.4	167747.7	117100.5	143139.7	194256.7	227316.7
234871.5	161393.9	153042	124728.2	182594.4	172045.8	170790.2	116084.1	225308	202940.5
170067.5	168997.4	155570.9	135724.5	229215.9	154935.7	131299.7	191655.8	162026.9	159955.7

LP341.157[illegible]	LP341.157[illegible]	LP341.157[illegible]	LP341.157[illegible]	LP341.157[illegible]	LP341.157[illegible]	LP341.157[illegible]	LP341.157[illegible]	LP341.157[illegible]	LP341.157[illegible]
143826.4	218993.6	143641.3	149137.9	112698.5	141118.7	198806.6	153704.9	187403.7	166250.5
146931.1	156458.3	215062.5	189523.9	118192.6	118298	143851.4	110411.2	172860.4	147180.3
166964	176618.4	164169.1	142952.7	108054.6	135217.3	168923.3	119340.3	188851.5	209835.3
143152.2	197093.4	126214.8	136925.6	161217.1	133442.9	171993.4	103359.1	258738.4	166171.8
131482.3	222621.8	102350.5	201848.9	156351.2	158265	179433	116910.9	184775.6	161435.5

LP341.157	LP341.157	LP341.157	LP341.157	LP341.157	LP341.157	LP341.157	LP341.157	LP341.157	LP341.157
145711.3	122830.7	120274.8	112774.4	62797.35	151356.6	71638.66	145631	102280.7	71638.59
208571	107187.5	133625.4	132384.3	71959.75	188844.9	75626.75	153402.8	87575.94	72352.71
204895.6	124932	157843.7	109623	72079.91	133676.6	88647.37	160887.1	92052.67	101843.2
236635.6	131648.5	142661.1	152657.3	94806.69	230142.5	85690.97	197947.1	64671.54	49734.83
177985.2	190214.1	111597.7	166819.7	83902.7	130090.7	86968.66	208396.5	80320.93	90656.01

LP341.157	LP341.157	LP341.157	LP341.157	LP341.157	LP341.157	LP341.158	LP341.157	LP341.157	LP341.157
68053.86	68580.98	69830.17	66418.2	149477.1	91915.43	166900.1	187326.3	183725.5	139066.9
74638.25	73373.75	77503.19	69810.1	164370.6	94379.59	179996.6	198739.2	188713.4	134100.2
67040.17	73884.72	89220.66	63026.45	162205.4	107185.1	186289.8	174557.6	189841.9	129010
66749.54	82810.73	60611.32	77320.62	146509.1	109935.9	165539.6	186398.3	192203.5	135070.8
52872.68	78164.17	70071.92	64727.57	200716.7	82502.33	190266.2	184827.5	187486	131898.3

LP341.158	LP341.157	LP341.157	LP341.157	LP341.157	LP341.157	LP341.157	LP341.157	LP341.195	LP341.194
185492.8	106632.6	96819.38	117565.1	210223.3	64188.3	126698.2	68420.78	172639.5	114447.2
179401.5	99438.94	109023.1	109017.1	187167	63765.24	117832.9	68814.33	158788.2	102882.3
175218.4	108263.6	100669.2	116951	194817.1	60363.88	114738.6	58734.6	159590.7	106409.7
181854.6	108465.1	109879.6	112355.4	195933.4	58698.17	127064.6	62239.08	174532.1	102393.5
176607.7	105487.3	110278.8	115726.3	200632.4	61665.5	126619.8	63669.45	153676.4	114575.5

LP341.195	LP341.195	LP341.195	LP341.195	LP341.194	LP341.195	LP341.195	LP341.195	LP341.232	LP341.232
124284.7	106686.5	170470.1	149270	109327.9	142963.4	125058.5	158846.5	127329.1	175669.7
124815.2	96002.79	162129.3	146662	104765.9	154788	124527.2	152004.1	134462.6	164364.7
128869.2	107378.5	163330.9	153709.7	104468.1	144116.4	122654.5	162620	41887.26	171523.2
137536.7	59896.95	163748.3	140211.9	105123.8	150280.7	120450.6	173333.2	138253.3	179816.3
77965.46	106791.5	138590	149875.6	104084.4	148441.2	128285.3	172787.3	138761.4	186100.6

LP341.236	LP341.269	LP341.304	LP341.304	LP341.305	LP341.305	LP341.305	LP341.305	LP341.305	LP341.305
73824.04	795150.2	1514854	2272546	556303.2	467897.8	718453.9	210831.1	996640.6	716861.7
74104.81	799663.7	1297163	1503150	444677.2	439416.4	921251.6	245861.8	698767.1	539747
73104.98	905848.9	1054167	1579239	552612.7	462568.8	975690.8	312946.3	1068755	691765.6
54980.68	930201.1	1197479	2465599	442844.7	486999.8	1175394	361325.3	912356.4	705720.3
185456.7	943694.9	1887643	2178821	412613.7	327261.3	1101929	312642.5	1161925	561044.3

LP341.305	LP341.305	LP341.304	LP341.305	LP341.305	LP341.305	LP341.305	LP341.305	LP341.305	LP341.305
906813.2	179532.2	159270	1432116	361134.9	445131.2	1062854	346327.9	269205.3	326157.5
654739.2	173843.2	173192.1	838951.7	353522.1	461405.2	759859.4	271458.7	397024.3	297374.5
736662.4	185207.5	139819.6	846164	467056.5	575519	966450.9	365562.3	242546.1	354312.5
782537.9	202666	134810.7	633318.9	396068.4	508666.2	776146.6	486156.5	347728.7	276192.8
758877.6	149800.4	127839.8	849310.2	357627.8	537367.3	632202.5	449645.1	357090.2	254193.1

LP341.305	LP341.305	LP341.304	LP341.305	LP341.305	LP341.305	LP341.305	LP341.305	LP341.305	LP341.305
430150.7	446000.4	96065.89	411850.7	380280.9	191211.3	208966.8	321932	407028.6	347071.3
376553.4	345747.8	97999.58	425667	507866.8	196649.5	226935.8	387260	528201.5	351286.5
418434.6	333965.7	100689.6	403078.4	530729.9	162047.2	184682.5	501670.6	482584.8	336326.6
320924.4	353571.5	101271.6	382108.7	424485.5	191668.8	219596	409556.1	332144.2	344764.2
364570.8	331319.9	102476.3	394437.6	305408.2	179650.1	208858.2	249578.8	290453.2	434897.2

LP341.305	LP341.305	LP341.305	LP341.305	LP341.305	LP341.305	LP341.305	LP341.305	LP341.305	LP341.305
174227.6	362092.1	338803.7	142988.9	346525.9	318957.3	407551.2	96662.13	234461.5	139550.1
193280	350447.7	356934	149587.8	342569.1	315173.9	385700.5	132478.1	225916.2	150075.9
169692.4	408321.6	347281.6	147682.1	360364.9	376435.8	405077.3	129838.3	214786	152891.3
176476	342128.5	338340.4	139797.6	350668.3	292937.3	378465.7	119428.3	242159.9	149462.8
168735.6	304411.4	320787.9	151920.4	343206.2	285825.3	390442.6	119495.9	167136.3	138883.2

LP341.305	LP341.305	LP341.305	LP341.305	LP341.305	LP341.305	LP341.305	LP341.351	LP341.397	LP342.191
127235.4	217939.6	107336.3	108229.2	115769.6	135975.1	123456.7	34614.09	45229.88	35872.41
126055.3	175513.3	103910	123712.3	123872.3	115950.4	111006.8	35394.63	49219.34	35920.39
136604.7	185528.9	114865	115665.7	117449.3	126812.4	114752.9	40176.19	59345.44	36916.79
129047.3	180553.2	101493.4	109703	110112.3	111745.3	105215.3	40573.02	62798.44	38272.34
112800.3	171008.4	104157.6	110062.9	114324.2	131515.6	103915.1	40522.95	65077.94	42085.33

LP342.308	LP342.308	LP342.308	LP342.337	LP342.336	LP342.373	LP343.154	LP343.155	LP343.156	LP343.156
435284	305346.8	133223	43826.45	82517.39	23124.22	171270	132276	136570.1	170913.3
291162.8	339305.5	138245.4	35281.48	86673.39	26398.74	177713.6	137089.7	142160.6	164783.6
310281.4	225882.1	141066	39900.78	94419.62	41936.97	167051.9	133471.4	140312.1	165822.8
361422.7	335417.4	120715.4	40355.92	78302.52	51955.63	163578.5	118819.4	130034.2	153192.7
429459.8	247250	131442.5	48371.51	82097.83	57300.19	157393.9	119907.7	141535.3	145997.4

LP343.153	LP343.153	LP343.156	LP343.153	LP343.157	LP343.156	LP343.153	LP343.172	LP343.172	LP343.172
118796.2	136372.5	106527.4	123796.8	110372.8	174072.6	126316.9	162113.3	56584.91	67564.15
119118.4	136415.4	97132.55	126997.9	106351.6	170262.1	135468.6	156500.5	58160.42	59434.96
109530.2	131842.3	99625.39	56329.86	100034.2	166291	125607.1	115677	55789.49	63298.33
113782.2	132015.6	108393.7	123745.7	108288.6	180916.6	117424.2	105997.2	47821.15	60747.44
116826.4	128389.7	104807.3	129183.5	108021.5	161413.1	125678.2	113173.6	49231.28	64326.75

LP343.172	LP343.172	LP343.172	LP343.172	LP343.172	LP343.172	LP343.172	LP343.172	LP343.172	LP343.173
134737.4	141057.7	146246.1	210741.3	104419.6	169833.7	135024.1	127539.7	144922.9	144635.7
100739.1	95737.52	118363.4	144263.3	106959.3	135263.1	139967.6	151344	205000.5	185782.6
127658.7	150623.1	103540.3	122006.1	137098.1	142701.5	128181	179796.8	212879.3	147181.9
163401	99823.85	130473.2	162496.2	149424.3	142917.4	120689.8	161674.5	195108.1	210264.9
196185.6	165708.2	121976.8	128562.3	105829	183717.8	137783.1	207262.1	219531.6	136864.6

LP343.173	LP343.172	LP343.173	LP343.172	LP343.172	LP343.173	LP343.172	LP343.172	LP343.172	LP343.172
174110.1	185882.8	145362.6	128421.8	82868.93	124621.4	107948.8	184853.6	128882.2	104469.2
148900.3	128835.1	153374.8	132779.6	99453.75	157176.4	181932.4	121739.8	156767.7	132922.7
190190.5	196781.6	178942.6	129238.1	138866	111853.6	143538.2	130425.8	134316.8	158509.2
136926.5	140172.6	168814.8	85201.66	150041.8	111079	128722.2	211797.5	102640.4	187321.7
150337.5	147540.4	174100.3	128888.5	131952.9	152953.6	139058.1	106819.3	154509.2	142809.7

LP343.173	LP343.172	LP343.173	LP343.172	LP343.173	LP343.173	LP343.173	LP343.173	LP343.173	LP343.172
159952.2	135215.1	146369.8	158586.8	161310.6	151221.8	173774.9	154943.4	163190	227734.4
217545.9	170376.9	119984.2	161417.5	150289.2	141302.5	176478.5	161435.7	171069.5	138590
177144.2	127144.7	195362.3	154108.1	165708	168027.5	147678.2	172498	129615.6	163307.5
145863.6	153134.3	143418.7	130391.5	224813.1	119569.4	177200.1	126201.5	150232.2	154714.8
180314.1	98435.54	98942.79	160680.1	131498.4	175276.2	144387.9	114788.6	141711.1	160557.1

LP343.173	LP343.172	LP343.173	LP343.173	LP343.173	LP343.173	LP343.173	LP343.173	LP343.172	LP343.173
116262.3	97948.86	123046.8	135306	142249.9	113841.9	154662.8	111657.8	113676.3	102044.3
137761.7	89320.31	196124.6	159690.6	175768.8	112033.5	137517.5	129364	106462.2	89357.57
157261.4	154625.1	177993.7	129991.1	126371.7	113028.5	147995.6	127462.4	120462.3	131497.9
202798.1	124089	142904.3	172032.4	134239.4	115110.6	213885.9	142594.5	117874	126082.1
132635.4	98267.93	147570.1	175769.1	146980.3	116091.6	122009	108792.5	110023.9	105904.5

LP343.173	LP343.173	LP343.173	LP343.173	LP343.172	LP343.173	LP343.172	LP343.173	LP343.173	LP343.173
97641.3	127856.5	195295.7	178407.6	154989.8	170663.6	113036.2	140879.8	146890.5	126478.7
124641	125925.3	171586.7	189174.1	132483.3	142139.9	123521.6	169170.7	93329.05	137619.1
88440.49	142516.4	164793.9	152840.1	119818.7	107410.7	113326.9	135882.9	108862.1	141626.9
128643.1	121420.7	146973.8	99921.2	122741	115782.6	113209.8	128743.8	124965.6	114977.5
103513.2	165633.4	189392.1	164058	119768.7	114643.3	95520.05	218088.3	108347.8	131908.3

LP343.173	LP343.172	LP343.173	LP343.173	LP343.173	LP343.173	LP343.173	LP343.173	LP343.173	LP343.173
135668	106253.1	89791.1	118725.6	148526.8	103681	59877.49	148772.8	141673.6	108550.7
206878.5	83062.58	112839.7	104855.2	114737.5	136715.3	79147.26	93734.31	130325.5	103874.3
129528.8	109819.4	101973	151118.9	116274.3	130506.2	55467.83	88545.92	132502.7	136593.3
167362.6	107329.3	74334.33	148330.7	145955.1	111349.4	56625.29	79029.29	168408.8	100547
127530.2	104537.5	102167.9	126328.8	118691.6	110902.2	54227.53	118023.6	116273.3	127856.8

LP343.173	LP343.173	LP343.173	LP343.173	LP343.173	LP343.173	LP343.173	LP343.173	LP343.173	LP343.173
117532.1	84217.63	64320.86	79393.53	81837.57	113473.4	54445.14	48978.85	66261.59	56511.16
98411.64	117750.1	71976.59	148899.7	53897.87	125196.6	59023.93	45682.7	98137.24	64963.32
87609.26	74714.72	73464.5	135962.7	51923.89	116135	55353.45	54093.38	66321.94	56967.39
75943.1	85584.43	78679.62	106578.7	58955.27	116212.2	59446.57	45126.1	50981.84	57829
117591.8	84869.4	72721.52	122718.8	62502.23	124096.5	56868.44	47160.39	93182.15	63574.17

LP343.173	LP343.173	LP343.173	LP343.173	LP343.173	LP343.173	LP343.173	LP343.311	LP343.320	LP343.321
55547.02	47114.51	50428.92	38133.17	122388.6	124238.7	50433.97	27584.19	44698.88	28157.38
77194.56	45571.64	50004.52	38118.44	138948.7	118025.5	45435.74	25289.13	45714.16	26467.53
51660.21	45989.76	57893.91	40855.75	134521.9	109958.1	44573.34	19141.01	53828.92	23370.5
42236.08	43079.21	42923.23	36237.25	102397.1	106834.4	41292.47	23234.77	60051.58	25354.18
45976.07	39749.79	43609.75	36901.25	117184.7	168673.6	50901.45	19850.16	71016.91	25604.3

LP343.368	LP344.207	LP344.206	LP344.242	LP344.242	LP344.243	LP344.243	LP344.243	LP344.243	LP344.243
7542.164	51073.74	58916.13	49905.94	32225.47	54915.53	36075.77	40964.39	56189.32	57804.04
8419.446	49927.18	61931.64	48536.23	31151.62	56910.28	38468.96	40766.32	53854.15	50568.23
13170.1	49954.65	57917.77	47922.87	36060.12	50918.09	46987.18	38824.72	53944.87	56061.86
15997.03	43307.08	57334.62	48315.79	33062.13	40837.74	36504.24	49725.16	54071.7	48739.42
17745.74	46193.08	61683.55	44733.04	39924.53	57021.38	30514.22	47730.88	54259.31	52068.8

LP344.243[illegible]	LP344.243[illegible]	LP344.243[illegible]	LP344.243[illegible]	LP344.243[illegible]	LP344.243[illegible]	LP344.243[illegible]	LP344.242[illegible]	LP344.243[illegible]	LP344.243[illegible]
40804.81	42740.1	42107.36	49621.45	45493.03	43148.99	42170.89	44057.27	45108.42	80908.27
45467.71	40666.87	45668.76	59072.18	46527.54	42374.75	41314.26	54619.59	41000.3	70752.21
46820.77	41483.99	44980.82	54169.51	45450.95	40665.96	45872.13	48397.03	39548.29	73309.59
43647.49	38792.95	47781.63	52368.99	44741.75	40495.19	42476.21	45649.82	38574.83	72816.24
40455.05	47912.1	46159.39	58463.92	47539.68	45493.26	43341.2	48211.03	45643.43	78625.43

LP344.243	LP344.243	LP344.243	LP344.316	LP344.316	LP344.316	LP344.323	LP345.152	LP345.151	LP345.152
37764.8	60940.17	68940.82	281553.3	319541.6	132202.9	32450.86	289008.4	386465	442049.6
35742.74	54808.69	62164.96	263021.1	303321.6	149351	29511.44	278722.7	408083.1	381976.1
40224.82	55335.37	66226.75	180091.8	283760	116905.8	34040.31	292597.7	347282	348987.3
32879.5	55206.13	66802.45	229982.9	294026.8	161724.4	32753.34	337702	311206.5	394744.5
44240.23	56718.54	63482.86	239007	247360.4	139554.5	34611.7	364855.4	391953.2	427857.7

LP345.152	LP345.152	LP345.152	LP345.152	LP345.152	LP345.151	LP345.152	LP345.152	LP345.152	LP345.152
339091.9	404236.4	402477.9	349945.7	429291	306912.2	390130.2	374575.5	446499.1	571476.3
307869.3	361625.4	374220	391238	269626.4	383968.6	316612.8	360683.6	370377.5	427375.6
234772.4	386670.4	293119.2	500700.5	308692.3	305265.7	307704.3	491510	379264.2	479417.6
301104.7	346188.5	292873.3	305646.1	380407.1	329424.3	317826.8	466765.8	285283.4	482468.6
327829.7	372910.2	375097.7	363567.5	343511.6	339719.9	278340.9	505349.5	368751.9	500057.8

LP345.152	LP345.152	LP345.152	LP345.152	LP345.152	LP345.152	LP345.152	LP345.152	LP345.152	LP345.152
539617.4	303936	225891.6	466008.2	390821.7	311875.3	371069.2	383848.8	307854.8	346438
412661.6	418725.2	231090.8	383000.9	400933.4	302400.1	419955	446209.8	252648.3	365969.9
468618.8	258001.3	278003.1	358289.4	477610.1	279820	446548.4	482152.5	287034.4	305524.1
384796.5	533474	256840.5	325479.1	329493.8	287047.8	460956.6	317904.5	290864.7	273987.1
415848.3	465297.1	255551.4	380938.9	397308	386395.6	406649.7	438704	261581.2	356103.6

LP345.152	LP345.152	LP345.152	LP345.152	LP345.152	LP345.152	LP345.152	LP345.152	LP345.152	LP345.152
240197.3	441238.9	267456.8	257756.1	282845.4	261633	197791.5	246746.7	232574.8	321072.6
349270.3	377133	357271.2	275821.1	217001.6	271503.7	193759.1	259388.4	221137.5	344436
267251.3	408806.2	240325.3	203179.3	249222	221243.2	166497.3	428214.6	200573.6	245894.4
299306.2	440436.6	398959.2	245771	249147.5	258777.5	164851.4	254980.6	175121.7	339220.7
289327.3	428876.5	275031.5	251104.5	247706.2	254482	187604.5	237235.1	207589.3	365322.1

LP345.152	LP345.152	LP345.152	LP345.152	LP345.152	LP345.152	LP345.152	LP345.152	LP345.152	LP345.152
296877.7	169171.5	124519.3	140372.4	134959.2	112010.1	130175.6	170545.2	92696.24	153894.8
318495.5	170127.7	117803.8	132885.4	146616.1	116274.8	128440.6	176938.5	95655.44	149725
318279.5	167619.5	103179	125494.4	144022.9	127748.4	108409.7	151868.1	81590.35	156269.4
325027.1	160989	108474	118716.5	115304.6	103499.1	100408.3	146683.1	82813.17	120855.6
349643.6	181336.2	109400.1	121645.2	147444.5	117130.8	124966.2	170880	82829.84	147333

LP345.152	LP345.152	LP345.152	LP345.152	LP345.152	LP345.152	LP345.152	LP345.152	LP345.152	LP345.152
121293.8	369542.1	293935.8	489613.6	380631.9	371167.5	445027.4	340028.6	543665.5	471206
119025.6	441583.3	375255.9	382295.1	362337.5	537800.8	352997.2	365393	437142.6	387210.6
114981.9	460881.3	340586	547855.7	392942.4	331552.5	410089.2	323435.5	600111.3	424835.8
114532.3	379884.3	385973.2	362559.4	436671.7	412926	362499.2	324840.8	347073.3	350643.9
118684.1	447402.1	361695.6	413332.4	521364.7	529657.4	586284.8	306768.3	395309.6	346290.4

LP345.152	LP345.152	LP345.152	LP345.152	LP345.152	LP345.152	LP345.152	LP345.152	LP345.152	LP345.152
406149.4	372579.7	321817.5	332900.4	466844.8	494659.6	455068.5	270622.3	281285.9	485501.1
347842.1	406663.6	347555.1	383273.1	418945.6	421540.3	455783.6	223379.9	247151.5	437326.1
457296	348907.1	485314.2	325241.6	462317.8	438387.4	486705	207496.5	472113.2	464595.6
387136.8	539269.8	392202.3	364087	404833.9	340741	430796	266879.8	245052.9	463898.3
414130.2	314427.6	312820.3	385473.3	622531.2	565486.8	425032.5	287423.6	269024.9	436481.7

LP345.152	LP345.152	LP345.152	LP345.152	LP345.152	LP345.152	LP345.152	LP345.152	LP345.152	LP345.152
409726.3	606920.8	285494.2	307656.5	247880.5	226203.8	339721.2	393815.7	450424.7	442992.2
422811.1	394966.7	278482.2	296650.6	280422.4	289356.5	508769.7	364014.5	390599.3	379611.8
341711.1	485823.7	247806.7	385844	363737.9	336179.2	438538.6	327773.1	414096	358317.5
424480.8	495055.7	240737.7	316400.9	331980.4	294470.6	500652.4	299866.8	515994	352636.5
367133.8	326203.8	304373.8	314272.1	306906.8	272657.6	311439.6	377219.1	400503.3	318523.9

LP345.152	LP345.152	LP345.152	LP345.152	LP345.152	LP345.152	LP345.152	LP345.152	LP345.153	LP345.152
283716.4	166394	337997.7	415938.7	259176.9	186717.6	251691.6	250686.9	292613	183232
291119.3	170750.5	361916.2	375180.5	253259.7	181613	268740.4	254627.2	266685.8	191265.7
208079.9	180384.3	357164.8	410269.7	288297.8	174773.5	252636.6	252905.5	275974.9	185122.8
264644	145298.5	370072.4	418918.9	259358.6	186112.3	259249.5	270713.1	284439.9	184832.7
326893.4	162328.4	386643.7	416301.1	273845.3	179451.2	273196.7	272910.3	283362	186096.8

LP345.153	LP345.152	LP345.190	LP345.190	LP345.190	LP345.189	LP345.190	LP345.190	LP345.190	LP345.190
434409.4	248228.6	290037.7	422610	388749.3	291540.9	297036	314534.8	174290	491650.1
429739.4	273035.7	281344.6	412358.6	386109	266751.9	289179.3	318355.3	179398.1	488493.6
409673.1	231015.8	248038.4	379047.5	376220.5	261558.7	317474.2	279741.7	148631.1	480320.9
436408.5	235558.2	258319.1	387445	360227.9	289134.8	101604.7	268648	135383.6	162535.4
442840.1	251595.7	267002.8	362320.5	339931.2	280701.4	342726.7	274540.5	151961.6	517368.4

LP345.190	LP345.190	LP345.190	LP345.19_	LP345.189	LP345.190	LP345.189	LP345.190	LP345.190	LP345.190
101915.8	79423.11	464804.5	207424.7	363662.4	152453.6	381216.7	116058.6	388827.4	524017.3
108418.7	85164.01	429490.1	216525.8	389525.5	172077.6	394241.9	129830.2	406210.9	521785.9
82274.88	75107.04	423727.8	233098.8	392935.4	140658	402416	102815.6	420018.5	535738.2
81764.98	67194.67	424108.2	226941.5	370760.2	142082.5	129030.4	97529.51	400248.2	165594
91910.88	77226.32	417136.6	204147.2	371618.5	147644.2	434211.4	107398	424478.2	536783.7

LP345.190	LP345.190	LP345.19_	LP345.190	LP345.189	LP345.190	LP345.190	LP345.190	LP345.190	LP345.190
101320.5	324939.7	477370.5	217191.9	413429.2	207458	214359.5	263631.4	116756.6	291500.9
102461.1	310828.3	501499.5	237345.8	398970.5	205115	222610.8	271064.4	123649.4	281730.9
83632.2	283005.1	122170.3	197256.2	379940.1	194597	209017.1	223119.1	98898.59	286809
79652.07	288825.1	519744.9	199206.4	395806.9	184528.7	200784.3	246347.7	91931.71	101441.4
83757.47	297450.3	488606.1	226051.1	402265.8	198536.6	215401.4	253595.6	108421.6	286150.3

LP345.300	LP345.319	LP345.319	LP345.319	LP346.155	LP346.155	LP346.155	LP346.222	LP346.222	LP346.222
47323.72	34406.59	47455.67	29743.43	67187.68	68431.62	63775.77	70256.47	96872.56	58522.26
42864.8	27231.69	69942.62	27192.41	65862.79	67491.02	63404.81	102235	53933.39	56851.91
48179.03	40965.73	60466.81	20867.38	62824.74	65073.93	65202.68	94048.92	64439.07	60960.65
49108.02	25725.93	54475.84	21382.08	71292.78	70927.69	60766.69	101510.2	60944.72	58637.57
59896.6	27075.14	46149.49	24452.57	67747.3	69989.72	69007.4	105756.5	64302.71	62548.7

LP346.222	LP346.222	LP346.222	LP346.222	LP346.222	LP346.222	LP346.222	LP346.222	LP346.222	LP346.222
76953.53	90065.68	95033.76	126832.6	60068.28	57288.14	96884.21	65391.54	82810.19	80515.61
70735.26	85438.8	72591.92	127488	63786.11	61738.6	99289.19	75222.11	82044.93	85187.46
78269.93	106926.8	78447.21	127223.1	60867.75	56483.19	103888.5	63482.55	82536.29	77005.72
70100.18	84363.54	70783.96	129369.1	62131.74	55671.15	95632.16	68583.51	86782.22	84722.3
92753.95	93816.63	73040.42	119822.8	58225.45	56842.33	92535.95	56480.09	77720.59	72818.81

LP346.222	LP346.222	LP346.222	LP346.222	LP346.223	LP346.222	LP346.222	LP346.223	LP346.222	LP346.222
71912.8	83284.51	89774.51	85718.71	72807.19	64652.44	80388.58	80872.93	84449.95	53764.02
62709.19	66796.79	57992.5	90850.89	76851.52	63899.82	60135	79022.86	90803.82	53269.31
69029.13	83340.02	87792.2	72471.05	80444.24	68195.63	69499.3	74366.87	85278.81	50990.94
74019.01	89485.07	89922.97	78276.19	77542.69	62319.85	62527.9	81940.63	86789.15	50361.1
68490.09	84481.41	80924.42	71142.29	75493.14	67466.78	63780.53	80921	84661.98	50087.79

LP346.223	LP346.222	LP346.223	LP346.223	LP346.222	LP346.223	LP346.222	LP346.223	LP346.223	LP346.222
52599	89206.32	85351.22	65417.37	73099.02	58893.99	55255.2	51814.94	59256.18	83095.78
53263.06	99420	93422.12	56330.68	77189.63	56921.49	52911.72	51346.93	63085.21	79127.21
50626.29	104303.3	99780.72	61644.42	68083.81	54263	47736.72	46977.78	55369.27	81701.96
49637.01	99585.53	96757.59	67232.96	74436.51	52042.59	56339.46	50053.03	62791.5	88075.82
56979.38	90100.23	88955.5	64281.9	74362.83	53149.68	48777.43	47777.1	60368.84	75649.19

LP346.223	LP346.223	LP346.223	LP346.223	LP346.223	LP346.222	LP346.223	LP346.222	LP346.223	LP346.223
73467.56	94150.45	76922.7	61484.54	60966.56	94579.06	60427.85	77393.85	54179.89	79981.1
67607.54	89537.35	76946.85	65288.61	67396.13	85828.4	61158.93	82843.55	57771	78624.44
75872.1	94653.92	77262.71	63917.8	61410.94	87951.69	64649.7	84944.99	52801.2	74510.02
72210.26	87399.56	75522.74	60412.01	63218.52	83036.83	57459.95	85116.34	56127.64	83691.97
78141.58	87118.97	69719.21	57732.23	57210.15	86075.58	64746.86	82671.27	48652.75	81847.55

LP346.223	LP346.223	LP346.223	LP346.222	LP346.223	LP346.222	LP346.223	LP346.223	LP346.222	LP346.258
69533.98	74581.29	63376.96	86528.63	96532.89	55642.75	68897.86	60691.29	62570.38	84461.7
58397.8	68863.7	58193.03	85189.06	98541.13	58458.57	71682.67	61400.28	51859.81	86868
64651.04	66349.6	61043.17	89482.16	96112.43	61485.14	76357.32	59940.12	58019.22	104813.2
66009.28	71612.28	58643.66	87288.08	102692.1	58766.83	64127	60846.01	56164.09	88925.33
67341.6	70460.45	55384.65	82057.09	97086.15	59313.12	68609.89	61706.78	56030.22	71293.13

LP346.258	LP346.258	LP346.259	LP346.258	LP346.258	LP346.258	LP346.259	LP346.258	LP346.258	LP346.259
70893.35	80259.68	77737.5	92553.34	85426.91	99784.99	86788.93	84984.61	100958.6	80137.53
73525.22	73321.59	72088.93	91909.56	88653.79	100714.4	94190.36	94588.19	93529.43	76507.53
89867.18	87179.11	65250.37	99566.93	86721.43	89146.18	94881.13	93658.45	103056.7	87231.3
82128.06	98945.38	69564.18	89632.04	99589.37	89487.95	117829	100385.5	88252.35	74395.67
85094.88	94792.57	72775.93	82799.24	86267.01	123008.8	81637.28	87929.81	92843.06	80468.95

LP346.259	LP346.258	LP346.258	LP346.258	LP346.258	LP346.259	LP346.258	LP346.258	LP346.259	LP346.259
60126.85	105086.6	56541.07	83916.99	54786.51	60902.45	73075.76	94257.78	91784.81	76158.69
59136.68	68876.91	55131.01	85340.41	58622.37	60462.75	83295.06	87056.54	89045.64	75012.08
52523.35	78018.65	87065.34	67429.53	57618.9	87431.89	131342.2	83284.54	63012.18	74350.82
58436.84	103948.8	63180.1	87515.71	55325.33	56429.11	78362.3	104370	85566.91	55795.15
60899.89	63500.89	60179.71	77206.61	48899.37	55499.18	83365.68	87012.94	104194	72251.87

LP346.258	LP346.259	LP346.258	LP346.259	LP346.259	LP346.259	LP346.258	LP346.259	LP346.259	LP346.259
77997.5	80268.62	80743.92	110741.7	73507.58	78489	107530.6	75679.3	58076.8	73104.46
102473.5	85603.91	71886.61	104685.2	75632.41	81626.21	113554.7	75089.51	66830.14	75238.26
76658.19	82250.52	75982.71	117460.1	70810.34	99389.25	93681.5	83711.45	63126.09	82135.24
74285.97	75485.47	73942.64	114530	72405.43	82885.76	107997.2	70709.2	65396.33	78697.66
77544.67	72195.3	83544.94	110906.6	76253.72	81449.65	134803.2	57038.44	59252.07	77041.69

LP346.259	LP346.259	LP346.259	LP346.259	LP346.259	LP346.259	LP346.258	LP346.259	LP346.259	LP346.259
113470.3	70450.5	86026.09	102703.2	73560.53	75926.22	82784.59	69741.4	48883.16	56256.8
104206.9	67567.28	86908.65	90912.14	74482.42	63478.01	80624.11	62829.55	44837.76	55827.29
110888.9	71850.72	86340.6	89139.3	85037.38	88512.27	80748.74	69139.78	49342.06	61664.66
120603.1	70954.9	86893.69	84950.96	77473.75	76245.88	94869.04	77253.69	45366.51	60582.6
124285.5	72101.67	97381.14	80293.92	77900.43	75367.63	85779.64	70646.86	51329.75	54547.23

LP346.258	LP346.258	LP346.259	LP346.259	LP346.258	LP346.259	LP346.259	LP346.259	LP346.258	LP346.259
82521.17	66663.86	68976.65	93965.63	65623.9	83191.54	99177.73	110271.9	64596.6	84855.65
80425.95	61660.6	68730.57	100957.3	64231.46	81214.27	99841.68	120550.2	63627.54	96026.87
81527.82	67746.45	76186.69	102792	61888.89	82730.98	104448	124497.9	62256.52	94469.13
88190.94	69963.16	67892.47	106553.2	56035.93	82578.8	102014.4	118930.9	70019.76	97658.88
76802.09	63416.27	64324.82	94062.45	60991.91	82667.45	94518.74	126328.4	59493.45	90197.36

LP346.259	LP346.259	LP346.980	LP347.315	LP348.238	LP348.238	LP348.238	LP348.238	LP348.238	LP348.238
95955.26	62891.68	73958.38	66723.6	95752.86	97990.09	93344.77	79782.75	92964.93	113768.1
91806.86	60228.98	68317.22	66259.73	105453	100730.9	93089.64	99437.18	81461.09	94700.22
89537.78	60100.84	59533.57	45526.37	118527.9	121188.6	75898.96	100337.2	89506.94	117395.1
96360.38	54654.66	55380.65	77900.33	163762.4	100358.4	63530.47	96579.74	92528.79	127112.4
90410.36	59569.08	49446.98	71596.83	140968.2	97122.15	103159.2	96839.25	127484.6	108110.6

LP348.238	LP348.238	LP348.238	LP348.238	LP348.238	LP348.238	LP348.238	LP348.238	LP348.238	LP348.238
97873.97	79799.79	98005.2	98987.05	97581.86	102036.2	121166.7	132429.2	82146.92	89496.89
73931	135584.9	89180.02	97762.56	72045.64	94685.65	110583.6	128440.3	84990.64	89473.91
72515.74	130257.1	90865.76	100117.8	104691	90163.22	113377.8	134961.4	88294.82	90834.41
69894.98	112491.9	103216.9	97498.65	87542.79	60433.78	105183	96136.44	77497.07	95297.04
66061.03	170155.7	103812.5	107119.8	104612.5	104734.2	87630.14	112663.3	84652.07	88485.07

LP348.238	LP348.238	LP348.238	LP348.238	LP348.238	LP348.238	LP348.238	LP348.238	LP348.238	LP348.238
67151.14	84660.97	92049.63	86833.19	84366.34	89037.13	97941.28	95735.67	81695.02	87848.69
75527.37	83016.42	77848.73	81593.7	90762.34	101725.2	93307.61	63726.77	97845.15	85112.76
81133.82	85537.02	78570.12	84139.01	120398.8	109653.7	87274.57	94874.27	86651.06	95778.03
112637.5	82778.68	110155.7	78590.11	91043.52	115943.6	71525.5	101082.6	117020.9	92519.69
81174.28	73348.81	80272.6	86028.47	88875.87	152379.5	89622.71	83715.04	102419.4	99117.52

LP348.238	LP348.238	LP348.238	LP348.238	LP348.238	LP348.238	LP348.238	LP348.238	LP348.238	LP348.238
85429.86	107471	133074.3	98591.72	78491.26	65857.75	102167.6	111444	106910.3	85764.22
83093.55	90316.14	134337	89670.86	81243.16	68093.09	94880.95	95550.5	108592.3	88466.02
97989.29	96689.4	104075.2	85140.62	85762.55	76442.36	89039.12	131478.7	115780	89014.68
91921.84	73523.5	108080.7	75113.12	84079.6	70616.23	120280.7	109752.9	124186	68083.8
91832	106861.6	161283.6	94125.78	91512.2	75832.27	91565.13	103416.8	83620.99	85033.19

LP348.237[illegible]	LP348.238[illegible]	LP348.238[illegible]	LP348.238[illegible]	LP348.238[illegible]	LP348.238[illegible]	LP348.238[illegible]	LP348.238[illegible]	LP348.238[illegible]	LP348.238[illegible]
57739.98	106304.2	86060.9	142947.7	73664.56	148740.9	67590.34	85643	63565.08	89993.3
59501.44	102733.2	85958.51	156291.8	81389.9	105318.3	72110.76	88824.93	64095.45	91897.46
69462.68	94203.28	85251.26	142065.9	73245.23	162049.2	72700.3	86023.13	53977.85	111187.4
64412.08	109082.6	116916.6	93297.03	76812.12	151196.8	86476.57	90376.98	60150.47	91746.16
63185.88	106003.6	84146.95	104674.5	68829.07	104807	85739.39	102713.1	69346.25	98897.18

LP348.238	LP348.238	LP348.238	LP348.238	LP348.238	LP348.238	LP348.238	LP348.238	LP348.238	LP348.238
115418	110282.2	81402.09	106933.3	74447.58	90137.43	95365.36	63149.8	105119.1	119287.2
128472.8	110958.9	66047.26	109144.9	72356.68	61183.68	101504	73545.11	98679.02	117082.7
118821.5	108819.9	83107.63	111533.1	70145.16	88525.34	101369.6	69413.95	92155.2	123864.7
121423.7	119037.6	75070.54	108288.3	114933.3	89927.01	89246.35	66707.53	101761.3	124094.8
128294.9	115503.7	81574.08	94836.16	79682.46	95875.92	99227.32	69015.09	118783.7	134423.9

LP348.274	LP348.274	LP348.274	LP348.274	LP348.274	LP348.311	LP348.324	LP348.326	LP348.326	LP348.325
65270.08	74941.46	92069.32	88014.26	90780.95	306355.4	69330.86	90089.61	72920.63	28262.72
60351.16	68350.64	91039.43	88552.98	86527.02	339533.5	65909.24	85013.45	74065.34	26128.62
38979.55	49810.09	65830.52	55632.75	67828.59	403011.2	69578.02	80881.35	71592.36	31548.29
67021.62	78909.14	91721.85	87930.36	99018.51	506375.6	67929.24	84708.88	75645.28	29217.25
69919.33	74779.23	95458.34	92820.94	91879.5	525510.9	58925.77	89222.57	69044.03	27148.31

LP348.326	LP349.272	LP349.311	LP349.310	LP349.310	LP349.310	LP349.310	LP349.310	LP349.310	LP350.172
85247.37	18168.54	23203.77	33061.55	32421.33	38038.47	28390.29	33532.77	27677.34	20567.56
81503.04	17460.66	29945.72	33550.1	36445.03	42015.29	26573.43	35269.42	29007.67	19750.8
80814.56	17978.57	25351.37	35182.14	38919.65	37908.16	31683.05	37777.36	27470	23226.8
78676.77	16331.79	26008.66	36947.26	38321.83	44919.14	33572.79	30102.18	27693.99	18513.24
79545.72	16556.98	26148.11	34543.88	36172	38738.84	32220.2	29828.59	28297.26	20134.63

LP350.217	LP350.217	LP350.217	LP350.217	LP350.217	LP350.217	LP350.217	LP350.217	LP350.217	LP350.217
54932.58	77667.82	60925.33	59641.34	45424.55	71200.56	88795.96	98025.27	72373.85	67771.01
55742.88	74908	65353.49	56555.3	44337.21	74283.96	91001.71	104020.5	71709.9	66290.91
50101.6	85333.38	59174.17	57684.2	47048.89	78811.55	91003.88	104199.4	79250.38	69509.21
49399.1	79261.14	55484.26	57126.91	53137.61	79092.48	98123.21	95043.39	67249.38	62581.69
48852.63	73433.7	64822.77	62280.72	46851.86	83898.63	94618.86	103490.4	66834.8	60296.12

LP350.217	LP350.217	LP350.217	LP350.217	LP350.217	LP350.217	LP350.217	LP350.217	LP350.217	LP350.217
67139.56	109566.9	102616	81690.45	83573.64	58825.03	108568.2	72230.17	75821.33	85814.86
70972.7	105159	104596.2	84346.78	82640.99	52004.14	111743.7	70609.71	76359.05	90038.83
68591.5	99894.7	102683.9	81829.05	81718.48	55472	105209.4	68575.4	75453.87	98091.16
69014.29	99555.01	107882.6	89242.67	79090.79	53456.51	102688.7	71572.02	79893.17	92625.66
64397.23	95796.89	82334.56	95150.81	77717.56	56930.22	108680.7	74613.67	78587.31	94435.24

LP350.253	LP350.253	LP350.254	LP350.253	LP350.254	LP350.253	LP350.254	LP350.253	LP350.254	LP350.253
207255	139927.8	239688.2	265684.6	172837.2	111257.9	150691.8	166924.4	152025.7	215717
189803.3	154688	232270.6	255962.1	215375.6	113866.5	181618.8	191787.6	152192	141799.4
218315.6	159222.1	209840.1	181566.2	229463.6	117689	142858.8	174548.9	137962.3	153377.3
163126.3	150787.1	214982.4	227723.7	260083.4	105333.9	226623.9	154310.2	156887.9	174004.1
222341.8	182472.4	266617	219940.7	163441.2	142037	134081	162804.5	153025.9	142832.1

LP350.254	LP350.254	LP350.254	LP350.254	LP350.254	LP350.254	LP350.254	LP350.254	LP350.254	LP350.254
165307.4	125860.2	145697.9	163163.7	207182.6	159073.8	167251.3	163783.7	185836.6	160461.9
208867.5	123666.5	223352.6	177513.5	191283.2	166419.6	154223.8	166668.2	179439.8	153480.6
206227.6	246217.6	215735.3	154455.8	204728.5	148971.3	147090.8	165064.9	188212.3	163795.8
209888.6	133733.6	216349.2	156287.4	185095.4	160542.1	155960.6	175718	179123.3	157594.9
177379.6	142019.4	203114.3	171745.7	202082.4	152913	153339	162647.3	173809	162341

LP350.254	LP350.254	LP350.253	LP350.254	LP350.254	LP350.254	LP350.254	LP350.254	LP350.254	LP350.254
167613.5	179429.4	119709.4	141785.3	149601.7	158053.3	108726.8	167983.2	169604.6	127409.3
155841.8	185386	104381	130408.3	231416.9	179374.9	104360	133239.2	162763.3	117384.2
175764.5	175921.8	114093.2	130461.2	217772.5	184248.7	109825.4	147021	191630.7	100709.6
188791.4	171378.8	104420	126497.9	215301.6	164476.7	115904.6	141047	155645.7	115870.5
178498.4	166539.1	116600	130759.3	232030.3	173532.3	119520.7	149247.1	159694.1	154290.5

LP350.253	LP350.254	LP350.254	LP350.254	LP350.254	LP350.254	LP350.254	LP350.254	LP350.254	LP350.254
179030.1	132683.4	162049.9	144059.2	110999.6	131881.4	145716.1	168703.2	163640.6	150950.3
173385.8	133512.8	157446.3	167574.3	102451.4	140674.7	145378.4	162180.7	174808.5	152426.1
177575.8	137051.9	155655.1	173776.9	121270.5	150076.8	148699.3	179056.1	165325.8	139440
190181.4	123266.3	170709.3	175459.8	111164.5	145670.4	142615.3	176763.1	152690.8	154372.5
168538.5	109654.5	153518.3	168425	111985.3	144508.6	128271.7	159667.2	169490.8	159108.7

LP350.254	LP350.254	LP350.254	LP350.254	LP350.254	LP350.254	LP350.254	LP350.254	LP350.254	LP350.254
138655.8	84815.7	126244.3	213986.7	111230.2	161279.1	102191.1	128907.7	187890.8	91201.74
135524.8	90815.58	125526.9	206110.4	106277.3	225324.3	85102.41	86466.2	178305.1	92462.48
146258.9	83055.67	128872.4	207015.6	112539	215227.7	138828.6	105214.4	180945.5	89881.9
139700.9	96334.36	113869.8	192622.3	108213.8	230154.8	78613.89	95426.75	175169.5	103393
128010.2	86711.01	107764.4	211352.8	111629	220583.4	77642.15	98029.01	175246.4	85337.52

LP350.254	LP350.254	LP350.254	LP350.253	LP350.254	LP350.254	LP350.254	LP350.254	LP350.253	LP350.316
106800.3	155896.8	163295.2	263270.3	157371	73326.5	79751.43	216754.9	66281.63	156824
128474.1	148987	159073.3	256155.8	156928.7	73617.35	78352.08	215512.4	77540.7	155073.1
106868.2	143350.4	156700.8	250684.4	159419	78103.15	77491.29	208998.6	69626.63	152025.7
102792	151271.3	163253.1	244115.7	153930.5	78212.39	76239.78	218896.5	67572.63	148150.7
109565.1	145356.4	165208.6	242887.9	151216.2	73095.12	73171.54	222056	66008.44	139757.1

LP350.326	LP350.341	LP350.342	LP350.342	LP350.342	LP350.342	LP350.342	LP350.341	LP350.341	LP350.342
233293.7	263028.3	310165	227536	166831.5	147661.7	202162.8	140840	112975.5	158081.3
239752.8	289889.4	306078	176528.9	188466.2	127590.9	240459.4	131759.3	114039.1	152428.3
235885.5	297357.8	216461.9	186265.9	178472.5	136316.4	315843	142285.5	151919.5	152839.7
231660.8	278992.4	217159.1	205147.1	168824.6	137848.2	238507	137294.4	104504.3	145375.2
225072.4	293672	249000.3	187884.1	177935.6	145090.9	228792.4	128897.4	102024.2	143877.1

LP350.341[illegible]	LP350.341[illegible]	LP350.341[illegible]	LP350.341[illegible]	LP350.341[illegible]	LP350.342[illegible]	LP350.342[illegible]	LP350.341[illegible]	LP350.341[illegible]	LP350.342[illegible]
192436.6	107956.1	91485.32	179589	154333.1	145365.7	194134.1	181087.3	84386.72	188856.7
195778	98603.45	145853.2	143070.7	163431.7	137875.4	206001.4	185303.6	83604.06	173134.3
186129.7	94955.69	135613.9	177356.7	156198.6	142958.4	218700.9	181066.3	91727.64	188949.1
173566.2	95854.48	123930.9	182003.9	153697.4	169063.1	196420.3	181411	76047.32	186620
182775.3	99966.82	152565.3	166234.1	143690.3	150129	204499	174441.9	107502.7	166769.3

LP350.342	LP350.342	LP350.341	LP350.341	LP350.342	LP350.342	LP350.341	LP350.342	LP350.342	LP350.341
177964.9	184672.7	146127.2	155874.9	181727.5	160303.8	161857.5	158958.1	150099.8	141853.9
182880.8	191452.4	149819.5	145886.4	181741.2	179974.6	143801.7	161052.6	139619.8	158444.9
182651.8	177035.4	155942.8	147683.8	163660.5	176562.3	156598.9	151034.5	142427	138399.7
183229.7	173301.8	155253.2	149835.5	185308.6	160575.5	160263.7	147760.4	141314.3	140266.9
176061	180498	152222	168493.6	168337.5	179279.1	153225.3	134426.4	151895.2	159293.7

LP350.342_	LP350.342	LP350.341	LP350.342	LP351.159	LP351.253	LP351.253	LP351.253	LP351.287	LP351.29_
166971.8	140712.1	146111.8	143147.5	26163.94	112897.2	37978.14	59446.86	63716.81	49223.31
170460.1	142406.5	171517.5	149216.9	31993.78	120417.2	41063.11	58649.88	53564.13	51613.87
173758.9	146727	143116.1	145848.7	31434.97	66125.86	24439.11	31973.79	59253.67	53996.51
160356.5	132276.5	142768.9	144959.9	30990.93	113175.4	44087.96	62003.26	62180.79	48976.96
163822.9	140259.8	151357.8	147955.6	28609.96	113484.5	32775.63	58846.68	50305.62	48217.61

LP351.289	LP351.289	LP352.170	LP352.184	LP352.194	LP352.196	LP352.197	LP352.197	LP352.197	LP352.196
71045.95	37424.6	154107	190330.2	301616.2	612449.7	901458.5	502366.3	879413.7	617025.3
72338.51	40912.36	148037.8	182383.1	241466	638134.1	848929.2	513205.8	789665	611642.1
67036.84	39734.25	145154.5	198226.5	264740.8	566634.5	808521.8	470654.8	718633.3	565934.1
65239.87	34436.82	141660.7	174654.2	274711.3	491828.4	817478.1	474959.2	774114.9	593216.4
75266.01	37450.52	119900.2	163689.8	244859.7	492525	754907.3	463561.4	786217.2	578026.2

LP352.247	LP352.248	LP352.248	LP352.248	LP352.248	LP352.248	LP352.248	LP352.248	LP352.248	LP352.248
606694	108314.9	932703.1	1160282	456076.3	788489	753712.1	477389.5	796393.1	818019.5
607238	125309.9	724334.5	1347075	797061.4	763815.5	656134.5	562838.7	657997.5	1140815
559375.4	147239.4	523678.9	1003910	659434	1286379	756147.9	484148.5	625850	1107872
631685.4	148531.8	770200.3	1318632	712885.2	627153.1	860178.9	650308.3	732454.1	913691.4
577701.8	140631.5	1038704	1067056	1039680	611204.2	833570.1	472558	842300.6	1167089

LP352.248	LP352.248	LP352.248	LP352.248	LP352.248	LP352.248	LP352.248	LP352.248	LP352.248	LP352.248
451919.9	904310.1	807733	730149.3	819964.7	719009.3	458191.7	408036.6	816716.4	751246.3
662535.7	940677.2	1164545	1107759	838293.3	715359	536082.2	567920.1	1066063	1010725
528053.4	853695.9	649481.7	907965	1051876	976839.2	473017.8	359337	865722.6	1041091
535777.8	612094.3	652529.4	856965.3	841612.3	738074.6	495316.7	494451.8	972971.7	882928.2
513668.1	967622.9	627654.2	764327.6	704995.7	733625.7	441625	641319	923289	1362839

LP352.248	LP352.248	LP352.248	LP352.248	LP352.248	LP352.248	LP352.248	LP352.248	LP352.248	LP352.248
827509.5	1122317	607054.4	432088.5	491542.9	505616.2	939884.2	814359	614594.2	692641
636793	882244.8	851064.9	508617.4	516441.9	453975.6	931032.4	634180.6	482029.4	883150.8
769284.1	1186131	835023.2	449988.2	443880.3	591280.3	1013798	620862.6	508925.3	593746.5
655505.7	846588.4	917313.1	565622.5	295118	420116.5	655001.1	573980.1	361103.6	855901.5
655309.7	1214364	927354	434107.9	463031.6	595778.4	919435.7	717015.3	442380.5	1188845

LP352.248	LP352.248	LP352.248	LP352.248	LP352.248	LP352.248	LP352.248	LP352.248	LP352.248	LP352.248
361012.5	495103.6	508497.2	347952.4	831676.5	652972.9	818911.5	705870.7	673345.8	625444.8
391648.2	448141.9	830808.5	566845.2	505655.6	753946	1353082	710376	854640.9	692598.8
382484	375153.7	1353944	589192.5	561432.5	986534.7	704028.7	818951.6	900923.8	878411.1
430314.1	482493	923682.3	547955.1	574309.1	887702.2	671857.7	607130.1	836887.5	820332.4
358698.3	532941	805461.1	630005.4	467979.7	976120.4	1042581	681966.3	869647.4	668943.4

LP352.248	LP352.248	LP352.248	LP352.248	LP352.248	LP352.248	LP352.248	LP352.248	LP352.248	LP352.248
464403.5	560609.5	688605	484870	884971.4	1091259	541462.8	627558.1	658873.8	684963
896100.2	569649.4	766549.5	501719	649319.4	913218.2	363203.2	385089.7	680158.4	610171
714311.8	722376.6	819659.4	474369.3	699615.8	889776.6	356079.2	414503.9	754372.7	646951.3
1036082	555505	784079.2	659313.8	629458.6	902815.2	440761	535741	748234.9	653681
629131.6	720050.7	817431.8	450033.9	649863.9	922728.3	457835.3	488668.2	777263.5	1083290

LP352.248[illegible]	LP352.248[illegible]	LP352.248[illegible]	LP352.248[illegible]	LP352.248[illegible]	LP352.248[illegible]	LP352.248[illegible]	LP352.248[illegible]	LP352.248[illegible]	LP352.248[illegible]
417151.2	414851.7	864069.6	911448.4	801045.8	466280	867450.2	203800.2	198904.9	340283.2
440979.1	391792.7	737461.5	964315.4	915111.9	478614.8	610455.4	191633.2	222030.8	429385.9
404459.1	418294.2	735876.2	855559	1067317	388113.4	791279.7	200377	216477.8	373514.5
468212.5	448101.2	790430.6	772450.5	604389.6	546193.9	425893.6	198645.6	267852.2	445314.2
478189	455298.3	730364	743061.7	807313.5	541544.1	587836.2	214256.2	233557.2	508173.5

LP352.248	LP352.248	LP352.248	LP352.248	LP352.248	LP352.248	LP352.248	LP352.248	LP352.248	LP352.248
503029.1	162819	601691.8	425684.2	512946.4	134154.3	172910.5	152368.2	153585.5	127617.2
482853.8	127758	609318.2	252118.8	340822.4	123265.2	176541.2	178832.5	155915.8	132456.2
690992.2	95365.39	591452.6	238310.8	722563.8	162989.1	174181.7	155172.8	160382	136593.4
470320.2	129112.6	596797.1	360407.8	562128.4	136271.9	177245.9	110283	107815.3	199714.9
476550.8	153137.6	781893.9	463137.1	589598	142165.3	170592.5	168971	101373.1	130920.4

LP352.248	LP352.248	LP352.248	LP352.248	LP352.248	LP352.248	LP352.306_	LP352.321	LP352.321	LP352.321
194480.3	149255.3	241815.3	183944.1	172658.8	1068838	44560.45	54605.67	46355.51	57234.49
184423.9	151711.7	226826.1	171798.8	180355.5	991301.5	42974.31	55205.38	43696.91	54545.37
126071.4	144111.1	208538.2	151960.5	166901.5	647274.7	47005.54	56401.05	42836.82	52144.51
173383.7	102357.3	188841.9	150266.6	144993.4	920723.1	40179.36	49280.63	39089.31	55791.62
166907.4	137575.2	164353	139178.1	137818.5	1035969	41155.94	52962.85	37376.12	51528.76

LP352.321	LP352.321	LP352.321	LP352.321	LP352.321	LP352.321	LP352.321	LP352.320	LP352.357	LP352.357
44487.45	55520.43	51007.34	45321.77	54100.68	52117.12	34450.43	32039.45	93387.49	107736.7
45538.59	59690.81	56830.85	45710.4	64155.63	54917.41	36629.04	31876.7	70174.96	93542.59
46324	59398.15	52166.71	41271.58	54623.86	56368.69	35492.84	32593.73	86494.01	104540.2
47486.29	54673.72	56479.27	44422.95	52660.59	53340.92	37352.59	30407.92	75106.07	106092.2
46644.2	54751.26	53672.75	41574.21	61528.13	54420.56	34088.34	35081.74	88091.09	96716.84

LP352.357	LP352.357	LP352.357	LP352.357	LP352.357	LP352.357	LP352.357	LP352.357	LP352.357	LP352.357
103401.2	92238.53	160157.4	100799.6	134464.2	86285.01	171525.5	108908.4	97438.93	70003.64
92691.71	89111.68	167211.6	98812.6	97503.42	79969.5	165820.2	104175.1	106855.7	65630.51
89088.32	87314.94	162457.1	95310.54	105993.7	81745.76	177332.1	101840.2	106841.9	63805.22
94798.43	78051.13	162160.5	100321.3	114912.7	88696.62	147723.7	99923.83	105984.7	75339.39
87559.05	90771.95	179298.6	94563	106590.2	85871.32	169823	97945.64	98485.82	65555.5

LP352.357	LP352.3574	LP352.357	LP352.357	LP352.357	LP352.3577	LP352.357	LP352.357	LP352.357	LP352.357
23830.68	83922.08	129935.8	104934	388731.7	292951.8	293171.7	222569.3	178931.5	179284.3
21449.75	89413.43	125970.7	101564.4	228225.2	290072.3	300254.2	228874.2	185214.7	241484.7
19996.94	82387.04	130452.1	102940.1	504608.8	307445.5	371699.8	229151.4	181739.1	187165.3
20640.15	90140.53	123284.6	96552.13	215622.2	284676.8	301053.3	236113.7	191532.8	176828.5
23720.35	83095.26	133110.5	103570.3	353701.7	245267.2	286210.2	177493.3	189094.1	177443.3

LP352.357	LP352.357	LP352.357	LP352.357	LP352.357	LP352.357	LP352.357	LP352.357	LP352.357	LP352.357
170119.1	96151.86	151017.7	140827.4	108392.3	147379.3	112651.4	162235.5	89933.95	73567.34
150211.2	113810.3	156707.1	136805.7	101777.4	141507.2	106417.1	166180.6	91558.13	68812.65
171003.8	100682.4	156171.6	134194.6	105833.5	144805.6	112078.3	150113.1	97796.4	74787.61
164449	109896	147850.1	126652.2	99061.25	134362.2	105655.7	165828.6	87902.64	72647.52
166942.5	109004	140204.8	129939.1	105369.5	145078	106689.2	169115.7	84371.43	69545.46

LP352.357!	LP352.357!	LP352.357!	LP352.358_	LP352.358_	LP352.357!	LP352.358_	LP352.357!	LP352.357!	LP352.357!
158327.5	145268.2	176605.8	96523.28	132452.1	99393.98	191228.8	138612	81610.73	194963.2
175407.5	150486.9	186370	88029.37	137983.4	98841.75	186070.6	144051.6	82054.43	210101.2
158259.1	144195.5	174944.4	95826.84	126784.5	98683.12	184152.8	143345.9	78867.07	195622.2
155640.4	142608.6	177219.6	87328.47	132171.7	94500.1	183774.9	133592.6	82752.59	197211.3
157204.7	146188.3	168269.8	93929.47	121866.1	102708.1	199163	136773.3	84384.99	189548.3

LP352.357	LP353.157	LP353.157	LP353.157	LP353.157	LP353.157	LP353.157	LP353.157	LP353.157	LP353.157
80873.55	47342.32	66531.17	68875.97	64669.29	73209.81	58361.64	62797.86	90175.03	47862.44
88278.58	48225.23	59249.88	67577.54	67568.68	75183.07	47119.98	62921.4	79465.03	45673.66
87711.88	41884.64	64300.46	51308.38	74445.54	63466.74	53369.01	71269.64	55032.57	50987.12
84482.49	49577.88	68741	68993.8	65307.24	78114.89	50309.07	52437.46	96342.69	51837.53
78183.24	47761.66	61404.8	68465.29	66823.55	71668.82	48018.92	49631.19	84719.19	42782.75

LP353.157	LP353.157	LP353.157	LP353.157	LP353.157	LP353.157	LP353.157	LP353.157	LP353.157	LP353.157
41245.61	65192.96	50233.55	86428.03	50502.45	34774.72	33013.03	56478.15	48149.42	56155.64
39623.53	67182.11	48769.08	85528.05	45849.12	34180.58	37416.4	57637.38	50727.02	57073.63
34515.74	42432.75	44749.8	65514.35	36295.32	28266.46	21983	53185.31	37347.2	55107.86
43048.55	77025.84	45010	84125.16	48101.51	34964.25	38822.19	63807.69	52976.89	56790.65
39216.66	70968.39	47185.95	90982.36	46769.42	31953.8	35522.16	52722.52	47386.34	49986.77

LP353.157	LP353.157	LP353.157	LP353.158	LP353.157	LP353.157	LP353.157	LP353.157	LP353.157	LP353.158
52970.48	43948.85	41919.38	47445.56	53371.44	48761.12	39335.12	45384.58	43606.33	39310.38
55832.27	44277.83	46898.08	48141.05	54692.44	43947.75	37746.29	47325.45	39914.75	40100.38
52965.71	36807.56	31052.7	41432.03	57279.25	33717.7	31965	47126.4	28488.15	28783.7
51701.95	45023.87	44635.82	44305.48	49808.73	48835.64	37762.93	46076.69	45399.7	41143.92
49645.43	41788	44432.09	43299.14	52262.77	45617.43	32249.58	38619.73	40651.26	37522.92

LP353.157	LP353.157	LP353.158	LP353.157	LP353.157	LP353.157	LP353.157	LP353.157	LP353.158	LP353.157
52140.1	45414.49	43455.93	52766.84	34196.8	63473.04	61505.67	47522.11	66097.78	58921.73
47964.97	46338.86	45906.85	44606.82	33945.02	62524.22	60437.52	42168.02	64378.55	54130.76
44688.14	42862.83	39150.23	51696.27	19462.02	66906.15	58316.24	47044.72	71804.41	60760.48
54960.48	44833.78	45143.35	53309.46	38073.07	61320.72	66288.88	41669.1	58968.28	51338.63
51696.68	45249.01	36665.19	49346.82	31264.03	59330.02	64203.15	44755.2	65218.93	55379.93

LP353.157	LP353.158	LP353.158	LP353.157	LP353.158	LP353.157	LP353.157	LP353.157	LP353.158	LP353.158
69243.28	68966.53	49376.12	57657.99	52179.56	58167.77	55089.91	34439.84	63468.26	66922.1
67482.63	66647.05	51584.98	46142	49366.47	65144.14	52493.81	34397.7	60770.04	67585.18
69993.79	65089.07	46872.39	52492.52	49577.13	55601.81	25747.25	38991.91	58483.1	52556.66
63172.97	59673.38	50680.76	52482.42	54566.96	59937.53	61889.65	34337.44	57089.21	69996.95
62347.17	63312.89	47750.52	56321.44	46094.6	60226.35	53845.53	33570.99	56246.37	69521.49

LP353.157[illegible]	LP353.157[illegible]	LP353.157[illegible]	LP353.158[illegible]	LP353.157[illegible]	LP353.157[illegible]	LP353.157[illegible]	LP353.157[illegible]	LP353.157[illegible]	LP353.157[illegible]
72547.73	48334.9	37164.21	63450.06	60217.44	82823.36	42226.21	44340.53	62967.47	74958.25
71718.01	43987.25	37539.16	63259.73	56168.27	89039.48	41415.84	42521.34	52589.58	73695.84
57210.78	35759.28	27756.7	57876.09	43420.46	95625.62	25127.85	37843.52	61192.68	66948.09
76301.04	45158.79	39456.34	68063.19	59356.45	78727.11	41516.41	43334.59	59879.14	75513.95
74150.51	43201.55	32431	61571.6	50498.69	80474.87	37729.83	38420.4	57437.08	69517.68

LP353.157	LP353.157	LP353.157	LP353.157	LP353.158	LP353.157	LP353.157	LP353.157	LP353.157	LP353.157
57826.07	65846.52	65980.56	57800.2	77383.36	64496.82	41860.7	68678.87	48370.59	64528.57
63608.46	70172.24	69684.8	55010.43	70791.22	65121.01	38913.58	59735.02	45829.05	70258.55
68094.2	65846.46	68994.37	52704.74	61996.51	49358.37	36685.43	61750.97	42055.05	47396.95
62831.92	65379.3	61147.44	60770.83	70464.82	66386.62	46553.89	63716.71	47924.68	59937.17
56766.79	68341.49	62151.43	54566.69	71400.02	62948.78	39562.74	64208.22	47410.02	64020.44

LP353.157	LP353.157	LP353.157	LP353.157	LP353.157	LP353.157	LP353.157	LP353.157	LP353.157	LP353.158
64038.91	41453.93	71441.64	64085.11	73191.94	61443.13	67150.43	71692.97	51575.48	56290.2
66418.03	48176.01	65281.21	65033.27	77348.34	61864.87	65733.38	71849.57	51716.73	51441.15
61573.55	40653	35606.4	63271.73	55190.67	58315.33	47679.94	74498.49	53323.41	49200.22
66690.6	47588.43	74395.71	61392.8	73650.41	62968.83	69804.86	69085.65	56427.05	48494.16
63398.05	42473.1	68036.97	68925.35	80875.16	60741.34	67136.14	76092.59	48478.17	54041.76

LP353.158	LP353.157	LP353.157	LP353.157	LP353.158	LP353.157	LP353.158	LP353.157	LP353.158	LP353.158
50249.91	51650.43	53022.24	63017.82	36159.48	52289.06	62249.06	251330.7	49186.18	48245.75
45442.98	53563.76	58034.44	58691.34	36235.21	47045.25	60812.73	241671.3	53582.29	44269.74
43313.99	45141.75	59168.83	49385.04	28855.33	51585.08	60924.1	247970	52737.38	43560.5
47422.73	53175.08	58309.05	67148.37	32910.29	53109.67	58942.87	237334.1	49764.76	46175.03
50506.4	62191.05	51538.35	54720.83	33428.95	50549.96	60418.84	220621.3	47584.28	40869.31

LP353.158	LP353.158	LP353.158	LP353.157	LP353.195	LP353.194	LP353.193	LP353.194	LP353.193	LP353.194
29600.23	61189.92	58605.84	54136.3	295401.8	77077.96	90245.98	49593.85	55597.48	42246.91
29299.91	59563.25	66260.93	52013.17	283775.9	54469.6	96794.71	56930.33	46123.79	58474.98
29190.8	58091.4	68143.33	51739.16	319783.9	83724.31	96346.77	43702.07	50973.15	43111.33
32981.16	56092.28	59253.8	49611.19	266811.1	94062.14	92555.53	42871.65	70592.4	51726.27
28342.21	62213.84	70649.01	52673.17	270151.7	88489.09	74453.74	40476.11	55339.31	43306.67

LP353.193	LP353.193	LP353.193	LP353.193	LP353.193	LP353.193	LP353.193	LP353.194	LP353.194	LP353.193
59330.27	50773.48	56330.9	60622.85	60126.12	51539.09	54171.44	60185.17	59283.45	68112.2
52732.86	58879.07	54087.83	51695.49	62465.71	45273.4	68940.21	59978.42	41376.63	56158.68
55755.92	56175.03	47725.04	53949.34	55430.58	52479.98	57025.19	65194.19	62645.58	69904.88
69154.91	66713.01	70746.21	48300.27	49016.24	44081.06	73255.52	91638.08	56010.19	70138.5
67546.44	44045.4	48318.01	56935.56	54261.34	34536	68830.72	59015.63	54170.84	91665.94

LP353.193	LP353.193	LP353.193	LP353.194	LP353.193	LP353.193	LP353.194	LP353.193	LP353.193	LP353.194
62178.61	60223.98	54880.56	56507.75	64191.36	51124.74	52590.13	51266.31	68061.91	44985.88
83685.95	60597.33	67063.51	78252.6	56446.03	66485.04	39945.22	46789.67	56215.66	45826.52
68732.53	56964.63	44771.7	62466	73879.92	53177.2	50833.9	51937.34	72846.31	48809.09
40000.68	62762.62	51005.08	53658.43	77671.01	74602.24	49195.15	45436.69	63754.03	36044.1
67116.85	57509.98	38563.62	58495.65	60933.2	54169.63	62155.89	41501.93	66839.4	37300.3

LP353.193[illegible]	LP353.193[illegible]	LP353.193[illegible]	LP353.193[illegible]	LP353.193[illegible]	LP353.193[illegible]	LP353.193[illegible]	LP353.193[illegible]	LP353.193[illegible]	LP353.193[illegible]
60416.37	46572.44	54983.74	66107.88	55338.28	52467.82	56330.86	64505.06	58638.96	58690.45
70395.84	49114.94	47702.59	53635.37	66331.12	47296.58	48216.35	48239.11	68253.79	58323.58
61601.01	46667.89	56986.36	65255.78	61717.28	62831.48	55426.84	57220.82	63133.25	59246.7
62773.52	54793.2	54078.57	63673.69	50098.4	59830.05	75512.91	58691.76	58492.48	51344.66
62515.87	34754.09	50905.23	51376.57	56548	49599	59920.98	65725.45	56150.33	59263.07

LP353.193	LP353.193	LP353.193	LP353.193	LP353.193	LP353.193	LP353.193	LP353.193	LP353.193	LP353.193
57617.19	63070.42	71608.23	59772.59	64025.39	72103.73	56802.28	52804.1	66705.94	60568.83
56179.92	70711.71	43773.79	59563.24	49802.46	87037.85	57776.11	53194.64	58678.55	72293.62
56891.61	64522.58	78509.82	57211.11	67643.92	75926.04	53667.49	59904.87	64638.68	60655.75
54425.71	62807.98	65241.21	57883.67	50858.63	74239.87	55094.03	54444.94	61308.73	91688.96
86693.4	42476.55	72733.38	55450.56	70746.09	75712.09	47882.83	53271.67	50572.98	45171.2

LP353.193	LP353.193	LP353.193	LP353.193	LP353.193	LP353.193	LP353.193	LP353.193	LP353.193	LP353.193
64003.77	55850.78	40678.99	58945.82	58761.68	60499.4	48706.66	57038.26	54625.06	56294.95
67292.37	42722.14	46241.09	59125.83	57484.1	68408.29	58603.64	37019.11	62468.65	46274.89
70858.88	54452.36	49690.31	61496.41	58760.36	60426.97	52115.22	54261.57	55557.88	54978.69
53241.86	46099.51	62064.01	57761.09	57705.57	62723.12	48124.31	60869.67	53943.01	40122.33
39594.67	58595.24	48185.21	85717.48	63342.72	58059.85	77176.04	56249.62	52079.12	48968.02

LP353.194	LP353.193	LP353.193	LP353.193	LP353.193	LP353.193	LP353.193	LP353.193	LP353.193	LP353.193
56168.84	51038.99	59968.32	56660.47	55971.95	53376.9	42347.42	44603.84	61595.88	37930.33
61498.47	63473.71	52837.01	39679.97	81502.98	45095.8	30435.88	58167.2	58375.01	63792.9
57050.47	46422.66	58819.18	62397.75	58321.3	59819.16	42474.75	47660.53	55806.3	39730.85
40363.57	46550.91	63880.23	62657.08	63043.92	53163.12	43555.34	45969.24	53830.19	39838.21
60798.82	49866.9	59560.04	66291.27	56199.6	67548.95	36439.24	61297.03	52009.48	40203.33

LP353.193	LP353.193	LP353.193	LP353.193	LP353.193	LP353.193	LP353.193	LP353.193	LP353.193	LP353.193
52602.62	61106.25	59321.76	38570.1	63088.41	62172.1	47057.09	48200.64	63810.13	75115.59
48791.26	72871.57	43793.52	35201.1	59449.3	82022.38	53226.33	52141.25	57634.16	77214.04
54798.3	54045.54	63516.54	38627.34	57245.86	68384.56	43319.35	56437.4	68004.5	69385.66
63248.99	55598.2	57874.03	44723.27	53619.27	65373.1	46578.93	55199.06	63003.34	71377.61
46962.83	52835.46	60228.95	36500.39	69056.92	65818.95	52404.27	44901.69	55200.58	75555.56

LP353.193	LP353.193	LP353.193	LP353.193	LP353.193	LP353.193	LP353.231	LP353.252_	LP353.252_	LP353.251
70701.93	42644.79	52867.99	32709.86	80647.17	43748.54	47212.28	354692.5	484754.5	371211.4
72222.59	61685.14	48601.32	30575.56	71246.44	45244.57	47939.58	376659.7	562889.8	361421.6
67742.6	42815.97	55998.77	32357.99	81708.5	50102.2	51409.99	397473.2	560307	425109.7
63240.75	39504.51	49023.06	34103.13	78819.43	47508.49	45357.27	357926	529151.1	346152.6
72202.02	37084.59	50379.64	29070.9	73037.21	42371.28	44162.42	362997.4	490154.8	408465.2

LP353.251	LP353.252	LP353.252	LP353.251	LP353.251	LP353.252	LP353.252	LP353.252	LP353.251	LP353.251
468848.8	497235.6	626711.3	459165.4	286991.1	775849	305943.3	792013.5	350184.6	757134.7
493863.1	497903.3	621425.5	455455.9	285933.8	795159.9	285352.4	822472	333604	776242.9
526268.6	506386.6	612170.2	480812	256152.7	814371.9	256624.3	768256.9	365441.8	814600
471194.4	451913.3	598303	463468	271222.8	683280.5	274617.9	755559.4	305083.1	742906.4
509881.7	444707.3	615594.5	489758.3	274438.6	727134.7	254797.5	732208.1	347046.2	820126.3

LP353.252	LP353.252	LP353.252	LP353.251	LP353.252	LP353.252	LP353.252	LP353.252	LP353.251	LP353.252
198858.9	640483.1	711266.5	589239.8	597841.6	648962.4	478388.2	573550.8	504525.1	896581.8
198769.8	551722.1	712413.1	576883.3	556220.5	707978.4	501996.1	573367.8	507077.5	991657.2
190749.3	611442.6	683300.4	567283.6	541175.8	645426.1	468396.3	593075	544511.1	931078.6
178374.6	597396.8	668626	558620	564485.3	648602.8	448701.7	495705.1	535204.3	929399.8
189510.8	576278.5	631231.9	548000.9	553763.6	600672.1	443426.8	499883.8	518882.9	825899.2

LP353.266	LP353.266	LP353.266	LP353.266	LP353.266	LP353.266	LP353.266	LP353.266	LP353.266	LP353.266
951157.6	407397	759061.4	635667	502021.3	981512	493184.9	326433.4	552874.6	619154.7
1348871	486387.5	805938.2	719916.1	299893	964742.3	533888.6	312409.2	424771.7	985026.6
1163907	518868.8	748176.9	791487.9	426474.5	725991.9	746983.1	433691.3	512871.6	652641.1
1030146	422267.7	607454.8	709223.9	384764.7	949807.2	562065.8	388782.5	410547.1	648101.5
1216535	320842.7	1003523	713825.5	425582.5	882090.7	588070.3	542333.2	627168.5	715243.3

LP353.266	LP353.266	LP353.266	LP353.266	LP353.266	LP353.266	LP353.266	LP353.266	LP353.266	LP353.266
739239.1	552223.7	736955.1	507080	734049.3	903708.7	1177687	519945.8	731850.8	565024.5
449562.1	884317	627945.8	689831.4	725808.6	915047.9	983808.7	713387	712236.2	432401.7
760672.1	879207.3	480860.3	657469.8	681723.7	644553.6	1274173	422146	731299.1	690296.6
629212.2	622460.7	623215.8	483428	614098.3	668333.8	1383303	460356.6	658926.2	694334.6
546379.9	628504.8	766963.2	474046.7	694368.6	611784.5	913219.2	547745.4	640922.7	542537.3

LP353.266	LP353.266	LP353.266	LP353.266	LP353.266	LP353.266	LP353.266	LP353.266	LP353.266	LP353.266
644998.8	537310.9	543990.6	252215.5	1231638	1270588	538633	1065151	574024.1	903148.7
1023409	449106.7	451783	259312.3	874197.5	1180095	320384	717049	699631	735591.4
796701.5	551467.9	423582.3	172012.5	1013593	1568319	449294.8	748222	780467.1	895958.8
758618.7	589866.4	636934.2	261590	885684.9	1266931	413343.6	744600.4	666227.2	975257.7
635828.3	492820.6	588514.6	177665.6	825101.9	1033258	423535.4	900464.4	696460.8	1031104

LP353.266	LP353.266	LP353.266	LP353.266	LP353.266	LP353.266	LP353.266	LP353.266	LP353.266	LP353.266
678295	802881	1092785	527833.8	996392.8	856752.2	797576.1	603762.4	643522	474422.5
812395	957766	1014928	471158.1	608909.3	784336.7	664303.2	701164.4	883921.4	461138.3
608583.6	963549.5	1098584	393823.6	664492.5	680006.3	526129.7	865892.7	1040178	536923.3
791370.9	863603.6	883533.7	408664.3	635166.4	893157	628815	671092.8	686302.4	425303.3
970956.3	625725.4	1073681	488428.2	702623.1	507568.7	639574.9	640241.9	830574.5	563427.2

LP353.266	LP353.266	LP353.266	LP353.266	LP353.266	LP353.266	LP353.267	LP353.266	LP353.266	LP353.266
849003	1292389	488232.1	499031.9	425782.8	943801.9	442596	464899.2	433857.8	884406.3
744787.1	1112744	320257.8	305246.6	694466.4	806565.6	603421.1	522582.1	517069.5	767735.2
508412.8	1178010	327479.2	570121	586249.7	758034.5	709837.1	402820.9	531136.7	664624.8
864632	1343525	327166.7	412830.7	489775.2	1005199	547462.2	408247.2	430847.9	799719.3
676266.7	987835.4	503554.8	523431.2	635083.2	1104694	494091.4	379879.1	451731.2	718140.9

LP353.266	LP353.266	LP353.266	LP353.266	LP353.266	LP353.266	LP353.266	LP353.266	LP353.266	LP353.266
401695.4	404571.4	775876.8	794041.3	728950.2	778467.5	442962.7	589118.8	590026	346357.4
431858.8	439078.6	584782.3	942647.6	774122.5	896716.5	376741.1	726708.3	767173.9	345446
416574.7	552426.4	800826.1	935089.3	801663.3	1225191	433520.6	714348.8	573621.1	353008.8
459067.4	381625.4	527788.2	794625	736814.7	946240.4	463576.4	495224.9	459198.5	354405.7
530135.8	590772.4	571828.6	813717	899713.1	992811	513389.7	682938.6	492006.8	459011.6

LP353.266	LP353.266	LP353.266	LP353.266	LP353.266	LP353.266	LP353.266	LP353.266	LP353.266	LP353.266
570001	377716.8	376792	461456.7	572387.8	576568.7	444153.4	339404.6	520653.9	510903.6
681908.7	466137.9	438941.7	424054.9	782304.9	604471.8	526920.9	409562.2	403575.6	412370.8
741139.7	505206	693043.7	590823.4	693584.2	601046.9	464595.7	341250.5	478192.1	595125.8
708992.6	450466.8	572251.4	433717	716173.1	761528.8	396360.5	482550.4	346657.6	445898.3
618996.7	492015.5	558539	540826.3	675972.3	655535.8	395577.6	514106.5	475157.9	409657.9

LP353.266	LP353.266	LP353.266	LP353.266	LP353.266	LP353.266	LP353.266	LP353.266	LP353.266	LP353.266
246321.8	347427.4	739692.1	524983.5	584530.7	116946	126563.6	252384.5	221855.9	275955.2
285424.8	427304.9	772793.9	590879.2	856306.1	159269.3	192107.4	234061	226070.9	255074.9
185419.4	375848.7	852636.4	542757.5	677805	160775.5	189414.1	254796.6	213759.1	281990.9
153974.3	292539.2	876220.8	754570.2	504599	116568.2	137845.4	183841.3	207159	197421.7
206457.6	405792.4	661078.2	531532.1	963720.4	133052.8	155451.6	250072.8	226199.7	204193.6

LP353.266	LP353.266	LP353.266	LP353.266	LP353.324	LP353.324	LP353.341	LP353.341	LP353.341	LP353.341
425760.2	236619.8	152507.1	389147.3	27307.07	304237.2	338464.2	166190.2	1074412	432371.2
398750.6	156520.9	314801.6	309986.6	442994.5	297103	294434	194807.1	1066632	120374.3
442555.3	190741.4	193040.3	299737.7	399830.4	309136.3	299851.3	141478.6	1087911	445079.7
555918.6	153819.5	135799.4	290751.4	375465.1	261864.7	275103.9	479696.8	1051010	478866.3
461693.3	168029.7	205653.8	348159.1	388395.4	290201.2	310789.9	487991.8	1107862	94972

LP353.341	LP353.341	LP353.341	LP353.342	LP353.341	LP353.342	LP353.341	LP353.342	LP353.341	LP353.341
705410.6	88459.85	818486	872106.6	410961.6	786266	394591.5	966881.8	399816.8	507675.1
682393.2	418556.1	915145.4	843460.3	395972.4	699390.1	475033.8	1022754	408840.1	491772.9
156402.7	431056.9	831119.7	788143.6	131834.4	755474.5	426449.5	1080086	426016.7	501695.3
640469.8	428723.5	879163.3	751775.9	421687.2	758518.6	454666.5	1072095	432033.6	427215.1
681559	71689.87	926787.1	724843.8	462423.9	735768.3	436586.5	1066517	435355.5	493244.1

LP353.341	LP353.341	LP353.341	LP353.341	LP353.361	LP353.361	LP353.360	LP353.361	LP353.361	LP354.199
561133.1	1181657	956048.4	928094.7	38993.93	43721.19	41716.23	30976.64	51103.21	54561
603620.7	1315998	943592.5	937086.6	35620.45	38229.38	45325.35	33471.35	44879.65	50763.85
640946.7	1373897	1024111	906156.9	32925.94	40053.07	42220.42	29017.8	46518.95	54752.37
585322.5	1407867	1033971	943926.6	32321.24	33333.88	47959.18	33021.4	49699.32	30568.72
593149.6	1381452	1052715	905501.6	42198.47	49389.26	36418.32	34163.72	44407.42	50602.47

LP354.198	LP354.212	LP354.211	LP354.212	LP354.212	LP354.212	LP354.212	LP354.269	LP354.269	LP354.269
56091.54	107686.9	48902.09	65326.09	45734.99	55502.91	41178.28	181211	159814.6	214481.5
54620.55	68147.68	39368.5	54712.18	39060.06	57174.46	39593.61	208310.2	159099.4	272503.9
55974.4	64278.01	45350.72	60438.45	40876.07	57229.12	42999.37	137734.3	158114.3	209241.8
50948.25	75542.45	42225.17	60142.68	43853.32	57385.24	42335.14	151111.8	147437	195732.1
50723.91	62215.97	39368.53	55320.79	44159.2	51154.23	40140.24	154864.4	147822.3	175755

LP354.270	LP354.270	LP354.269	LP354.270	LP354.27_	LP354.269	LP354.27_	LP354.27_	LP354.269	LP354.269
193493.5	228317.9	194056.5	146561	127435.5	207029.9	140328.9	106613.2	117169.4	106893.3
245436.7	200208.8	231153.1	163079.6	109435.3	254599.5	161442	108128	126839.4	146716
186337.4	195299	189226.5	150622.9	108822.1	238483.1	175717.4	98911.94	108552.3	111201.8
192409.5	216235.4	179023.1	154182.8	98920.27	227015.9	192047.5	105941.3	120610.2	95501.14
184827.3	206964.3	161899	150613.8	101237.7	206753.1	188886.4	101871.2	119497.4	113232.6

LP354.270	LP354.27_	LP354.269	LP354.27_	LP354.269	LP354.269	LP354.270	LP354.27_	LP354.270	LP354.270
176002.3	99723.53	78599.66	102007.8	77579.47	72769.57	189220.1	97676.45	122548.2	119921.9
206671.9	99842.66	106756.4	110416.9	90481.37	74272.54	138388	87137.56	116381.1	124184.7
139871.4	109551	86037.45	100540.4	94719.82	72826.35	178312.6	90919.32	105073.2	165586.8
183784.4	101976	78941.64	89655.33	87381.71	82199.65	187137.1	87693.81	100189.2	113661.6
173263.6	90624.18	69983.64	103427.3	93620.72	67251.84	161693.9	92900.95	104601.9	106383.9

LP354.269	LP354.270	LP354.270	LP354.269	LP354.269	LP354.270	LP354.270	LP354.27_	LP354.270	LP354.27_
89698.09	158476.1	132184.8	150144.4	72552.14	107043.6	145894.6	77855.3	80588.65	150158.2
81536.49	178757.1	129664.5	179609.4	89084.13	112131.1	157573.2	87348.25	88729.61	138302.4
90280.91	151448.2	144627.5	149527.9	73192.77	109133.7	154892.6	85344.8	93302.82	137682.5
95049.04	149499.1	130423.6	137956.6	84199.91	107788.8	169368.7	71023.02	84411.05	137232.6
89462.02	125416	136669.2	149341.1	75440.27	93754.12	159237.5	76205.12	89341.32	127608.7

LP354.269	LP354.270	LP354.27_	LP354.270	LP354.27_	LP354.270	LP354.270	LP354.27_	LP354.269	LP354.270
91698.1	135041.5	122486.7	143161	210697.3	136696.2	104955.6	161729.3	67208.01	158013
88550.36	139084.9	128476.8	138403.4	211207.9	134589.3	110957.1	162058	116214.1	153813.4
96399.52	132415.3	144001.7	141781.5	223321.5	142028.9	114555.1	158239.2	70693.86	162964.2
87048.56	130364.4	127384.4	140712.5	215602.9	127881	100913.5	160744.7	60861.87	141280.7
80911.38	115795.9	120735.4	124571.5	207629.2	132230.5	108696.9	165161.4	64302.51	149955.2

LP354.27_	LP354.270	LP354.269	LP354.270	LP354.27_	LP354.269	LP354.270	LP354.270	LP354.270	LP354.270
144815.1	79526.19	166654	148916.6	199881.8	83799.37	90954.92	118596.3	92315.37	86576.66
140959.8	79414.47	156615.9	151005.9	139026.9	98074.34	86453.83	108248.5	97592.14	82040.14
163240.5	87367.13	162180.7	148193.9	196148.6	86971.42	93033.75	105078.6	98010.95	77481.82
128746.8	78208.58	143631	153173.9	134336.4	95337.2	82681.96	111923.7	91683.21	80556.73
127788.3	77265.13	152421.3	149132.3	129057.9	84956.29	78441.49	106338.2	98068.08	79507.9

LP354.27_8	LP354.27_7	LP354.2701	LP354.2701	LP354.2701	LP354.2697	LP354.2699	LP354.2701	LP354.27_8	LP354.2699
98772.04	110331.9	101935.7	71108.67	82388.55	64137.66	87272.01	115617	107779.6	70858.91
102814.9	110248.8	103553.6	75192.14	83434.58	51070.33	91801.18	107742.1	106901	77894.86
103226.1	108106.4	109893.8	74901.03	70372.77	58983.62	90974.84	117169.3	112142.1	80667.51
95193.59	115618.6	100323.5	62382.23	73475.08	53021.18	79340.85	99898.03	117790.7	77959.89
95135.92	96282.58	97064.32	71364.85	74375.24	58291.41	81331.41	108489.5	102038.3	77939.12

LP354.277	LP354.285	LP354.285	LP354.336	LP354.336	LP354.337	LP354.345	LP354.373	LP354.373	LP354.373
208294.2	199091.1	203752.3	400703.6	141148.9	69688.65	71064.86	266014.5	533111.2	70586.85
50826.07	174107.2	226763.3	396713.7	136535.9	76001.78	65260.13	292794	614897.3	73486.8
73212.49	210453.8	229372.8	78170.22	135630.6	61858.89	64106.62	469085.9	974313.3	77836.48
141884.9	67888.51	43233.82	250166.8	129130.8	54967.98	60900.84	571760.6	1206441	63611.71
108229.8	72646.82	216085.6	230017.2	127317.9	59604.33	71653.53	634712.9	1327087	67470.59

LP354.946	LP355.069	LP355.069	LP355.069	LP355.070	LP355.07_	LP355.069	LP355.096	LP355.136	LP355.172
298710.6	35211.37	30380.51	25030.2	11742.51	19582.41	21683.27	21475.93	27141.2	39987.21
292933.8	24732.07	31321.35	29408.2	13355.57	20806.42	23310.18	18953.41	20381.16	37603.27
246789.9	37375.23	32869.72	26121.78	11419.4	20926.39	24052.1	21085.5	26078.34	41295.17
214164.5	48428.66	36409.19	27097.46	13346.37	17707.92	14932.18	21858.94	21184.66	39757.23
221333.7	46662.11	38380.68	30029.83	15213.36	22917.55	19476.81	21083.93	21686.87	38779.07

LP355.172	LP355.172	LP355.172	LP355.172	LP355.173	LP355.173	LP355.172	LP355.172	LP355.173	LP355.173
63096.07	62990.25	43841.33	30219.71	65217.95	66032.68	80705.59	105506.8	102537.4	59677.24
67713.93	58967.92	50820.11	33423.34	81774.73	77972.03	114687.8	81540.58	48553.5	57033.13
61565.74	66450.44	45812.66	33078.04	75424.09	84441.66	97635.15	87778.29	87398.39	60640.66
55007.72	64498.49	43495.01	31363.25	94797.38	59353.53	79531.69	70564.97	57313.88	62152.7
62104.37	66454.54	43137.37	27737.73	88324.63	59983.32	93536.83	64543.94	75064.94	55970.41

LP355.173	LP355.172	LP355.172	LP355.173	LP355.173	LP355.173	LP355.173	LP355.173	LP355.173	LP355.173
71823.08	89940.41	67648.23	101019.5	89319.25	63224.69	60021.31	77325.68	56221.31	87269.03
106435.5	68646.24	60813.49	69825.95	73002.01	92694.08	97047.43	63407.65	48916.87	66605.68
91606.59	66406.63	54026.44	80414.95	68969.88	83610.42	90733.86	102770	84937.54	64879.33
58095.6	76443.51	54589.24	69491.53	71733.01	68124.59	81551.56	54252.26	83495.05	78311.58
100093.9	62558.32	94844.11	87766.35	69495.64	98037.7	115572	62892.06	84262.69	90414.31

LP355.172	LP355.173	LP355.173	LP355.173	LP355.173	LP355.173	LP355.173	LP355.173	LP355.173	LP355.173
54658.33	53591.88	120161.6	84628.29	98470.68	112332.2	69386.45	73218.01	70717.36	108143.6
113998.5	61118.85	79816.58	115969.2	57308.08	58037.22	61911.79	77293.69	116045.3	66753.91
73902.51	52047.16	57940.31	72387.13	80672.16	76148.07	52898.4	62122.59	120794.1	87854.73
59248.25	63228.13	57087.96	73768.27	92456.51	73452.22	79666.37	66641.48	74101.01	75255
72781.86	61603.72	72707.4	129271.6	87607.56	87469.56	65277.07	67601.03	57568.53	132195.9

LP355.173	LP355.173	LP355.173	LP355.173	LP355.172	LP355.173	LP355.173	LP355.173	LP355.173	LP355.173
75255.81	87526.11	82265.85	59310.64	106955.3	61385.65	61854.28	61433.61	96012.15	72065.52
87987.63	59627.56	69117.46	69909.09	59652.89	71789.38	60985.39	69880.25	60799.61	74352.73
80745.95	77282.93	83231.73	88848.07	63218.11	82512.94	66941.54	50915.28	76166.93	67384.96
103249.8	79830.39	66864.28	66555.23	61025.46	82173.67	79194.81	59052.14	74329.27	79765.64
94184.42	64703.17	103842	56734.62	73761.11	60381.91	65720.8	67243.87	103121.5	76756.08

LP355.173	LP355.173	LP355.173	LP355.173	LP355.173	LP355.173	LP355.173	LP355.173	LP355.173	LP355.173
28640.26	94437.08	75401.1	106647.3	49458.68	61942.8	53552.87	58352.75	68638.29	47743.23
23619.03	83622.7	77043.17	57424.63	52498.41	71688.56	68914.24	40924.19	77758.68	57112.05
20300.48	62038.02	59510.08	80153.7	37497.19	107185.9	62182.79	99248.26	76150.32	69462.08
25932.98	99488.3	104750.9	69749.45	41779.21	66927.19	47382.43	62542.99	102302.4	49470.23
36655.7	67654.42	133626.8	94162.53	47287.75	77575.34	43115.65	49821.85	71173.95	58893.27

LP355.173	LP355.173	LP355.173	LP355.173	LP355.173	LP355.173	LP355.173	LP355.172	LP355.173	LP355.173
77823.53	79763.85	26537.6	77776.09	60081.84	58581.89	58342.72	72644.78	51607.68	72865.84
80515.87	82081.42	39795.17	58954.68	49632.45	73971.18	52513.86	60850.75	61325.75	62716.72
75009.01	88715.27	40948.28	76229.82	62330.67	68951.86	56279.95	63601.27	56269.33	51423.89
115259.7	77753.55	31127.68	51731.68	60500.37	71634.76	58382.3	74684.34	55011.85	66072.43
89262.65	66775.23	31449.55	80128.48	71273.21	74048.09	60944.15	79179.72	62082.11	62897.58

LP355.173	LP355.173	LP355.173	LP355.173	LP355.173	LP355.173	LP355.173	LP355.173	LP355.173	LP355.173
63707.38	91111.22	39345.81	55968.42	33035.61	57799.18	68079.01	88471.09	58176.19	64921.84
52270.62	73051.41	54368.88	68407.72	33273.16	69823.31	62770.59	89666.52	52395.39	96277.39
67797.96	123279.3	40731.79	41678.09	35633.23	61537.7	73193.71	76228.86	47207.87	74704.67
67028.68	87737.47	34931.23	54896.28	32858.61	62183.16	71826.87	75616.86	48792.83	97883.02
68456.13	72605.43	33035.15	67226.29	31012.94	52456.66	72922.48	109989.4	40446.03	73496.51

LP355.173	LP355.173	LP355.173	LP355.173	LP355.173	LP355.173	LP355.173	LP355.173	LP355.173	LP355.173
61636.38	37274.08	25215.68	28949.59	55429.55	31193.34	39053.77	61936.82	32828.27	65834.91
52713.38	29802.49	28285.26	28628.59	72123.98	21515.46	40981.37	54987.9	34213.96	62641.59
70659.53	41535.82	23132.84	35998.35	82383.02	29891.63	34132.9	70105.84	35339.6	70954.44
64769.8	32074.89	27828.93	26784.98	81964.53	23820.18	38828.28	55254.97	27743.47	66587.05
64248.22	32787.25	24086.46	21228.16	52004.75	29999.96	30068.07	58436.07	30319.29	72940.31

LP355.173	LP355.173	LP355.173	LP355.173	LP355.173	LP355.173	LP355.173	LP355.252	LP355.272	LP355.284
63399.9	73202.24	29028.82	85467.05	54022.01	74534.24	36547.64	39311.14	77454.79	164029.9
63176.32	69022.21	51128.06	61119.32	60729.64	72915.81	50111.9	39769.12	69803.8	125423.4
67773.03	79930.33	37056.45	83356.46	46109.03	86108.22	31034.05	32211.49	74085	144060.7
55490.38	71104.99	29071.61	80682.97	45528.94	75669.25	33937.24	69956.97	76629.92	120353.5
54364.14	57464.92	25128.67	88106.18	44432.78	74696.37	35951.31	67785.82	70444.01	117739.7

LP355.284	LP355.284	LP355.284	LP355.284	LP355.284	LP355.284	LP355.284	LP355.284	LP355.284	LP355.340
79918.86	91884.87	77266.82	64769.68	35647.06	37186.87	42502.77	71174.64	42154.13	33352.05
88652.84	80590.02	73656.7	56342.77	37031.24	38203.19	40215.12	69296.73	42861.73	38023.47
89504.26	87452.72	72141.79	59389.16	44268.41	39009.66	39718.42	77851.5	40633.45	32283.85
93267.3	70748.43	71916.96	57438.24	42161.74	35795.45	42970.3	66238.58	39345.08	29563.46
97916.42	84161.74	75685.47	51845.72	35583.11	33091.44	40173.53	70883.14	42167.32	29280.19

LP355.340	LP355.340	LP355.368	LP355.368	LP355.876	LP355.949	LP356.206	LP356.279	LP356.288	LP356.288
56686.95	23065.49	79905.02	34523	41625.58	50827.57	47680.01	19623.37	37218.65	26963.47
61426.93	21639.55	74345.74	37000.25	35321.26	54359.06	49150.35	23669.37	37092.31	23246.23
75372.82	18388.6	73978.1	27496.68	54455.78	41554.8	44365.94	23656.06	22330.81	24744.52
80961.22	22072.23	78579.3	17664.48	75905.99	31120.38	39645.36	22240.34	32778.44	26504.49
87606.33	17554.35	82931.77	27872.74	30292.53	28656.6	32827.26	22134.58	29886.45	25283.84

LP356.288	LP356.316	LP356.316	LP356.352	LP356.363	LP356.371	LP356.942	LP357.152	LP357.152	LP357.152
22884.81	53274.03	22115.52	47443.9	19097.9	11798.93	48807.65	141657.5	168211.4	207593.9
20004.33	52460.5	22310.39	48980.05	23238.16	25752.51	47922.47	195192.8	147083.2	164467.3
19096.78	46261.64	22092.07	48466.22	21345.63	25606.87	38733.41	186353	172353.6	177622.8
20337.98	53060.24	20624.25	54751.66	18645.37	26552.42	33924.39	152345.5	228174.7	220893
21346.93	51974.1	22732.11	57065.37	23156.88	25236.83	38326.87	139780	128906.7	142270.2

LP357.152	LP357.152	LP357.152	LP357.152	LP357.152	LP357.152	LP357.152	LP357.152	LP357.152	LP357.152
103319	169663.9	138974	172213	123101.1	132082.2	95694.55	92517.76	144505.5	86541.07
114162.6	124467.9	137336.2	132005.4	153300.6	145829.7	103486.7	96456.53	159693.5	102434.7
107914.5	115436.4	114616	178307.3	155681	139196.7	106640.9	102558	132960.1	71785.69
157295.6	122978.4	120403	213217.3	170273.5	152856.7	106475.2	105923	179243.5	76892.12
108728.4	161166.9	138457.1	105738.9	162906.5	125314.5	103109.8	131469.9	234904.8	77447.38

LP357.152	LP357.152	LP357.152	LP357.152	LP357.152	LP357.152	LP357.152	LP357.152	LP357.152	LP357.152
139034.7	107420.7	46483.86	48340.02	162950.5	213501.1	144663.8	201972	144759.4	174393.5
134561	90104.81	45418.66	46284.97	132117	227577.4	292228.3	226230.3	203473.7	237445.9
140940.8	77854.84	41799.32	38760.9	182078.4	133319.6	174241.6	170132.9	230774.3	162464.9
93791.91	108081	38096.31	44867.55	239756.1	149349.3	194705.5	203652.4	197506.5	159755.3
112626.7	99691.11	44627.72	45790.98	184929.7	148344.6	181832.6	185182.1	143268.9	173145.9

LP357.152	LP357.152	LP357.152	LP357.152	LP357.152	LP357.152	LP357.152	LP357.152	LP357.152	LP357.152
198613.1	136674.7	214991.4	237756	192026.4	192332.5	225386.6	247129.7	175000.2	261696.2
197306.6	193493	221757.8	182734.2	113078.3	158763.8	191562.2	174993.7	138454.5	312170.3
206061	157129.6	235481.9	172368	218335.3	199899.9	179769.1	181583.3	189105.6	228008.7
241055.6	213572.1	214072.4	203130.4	104764.4	117888.9	195931.8	166509.1	178507.4	206655.1
230076.2	134311.6	212885.6	218657.9	123686.9	141857.9	196245.3	206395.2	144648.5	207854.4

LP357.152	LP357.152	LP357.152	LP357.152	LP357.152	LP357.152	LP357.152	LP357.152	LP357.152	LP357.152
206846.9	163563.8	151001.3	111551	144971.5	162430.3	178492.1	113128.3	235395.7	106694.6
205903.7	232819	166906.9	135176.2	168086.6	141729.5	175437.7	160782.5	325949.4	101156.9
175144.6	210978.1	213724.4	124648.7	166193.7	160085.6	149696.3	118017.6	192059.1	112386
158315.1	179702.4	170845.7	162599.9	173954.9	176501.4	202967.8	102901.1	249409.8	100495
236515.9	154026.1	185736.4	129218.3	207825.7	151115.7	147759.1	137116.7	187386.4	116427.6

LP357.152[illegible]	LP357.152[illegible]	LP357.152[illegible]	LP357.152[illegible]	LP357.152[illegible]	LP357.152[illegible]	LP357.152[illegible]	LP357.152[illegible]	LP357.152[illegible]	LP357.152[illegible]
212415.8	63106.69	215090.6	130439.4	115111.8	115784.6	200265.1	207704.4	117069.8	169085.7
177052.2	61996.76	270883.6	136936.8	128996.7	129530.2	201033.3	153072.3	107482.9	186986.1
118141.6	61621.03	180400.9	133229.3	121295.9	126586	162083.1	207548.4	147912.2	170394.6
139383.5	56977.87	182460.3	139413.6	120967.8	139894	176386.5	188044.7	127403.5	182442.1
200582.8	60794.51	160305.2	123742.2	117813.8	148319.9	172136.6	214052	140738.5	217752.6

LP357.152	LP357.152	LP357.152	LP357.152	LP357.152	LP357.152	LP357.152	LP357.152	LP357.152	LP357.152
135846.7	123612.1	103952.2	173974.1	171917.8	129028	136676.4	133978.4	133801.3	150733.8
241254.9	95196.16	155728.2	156049	215658.2	139498.4	156431.9	124556.2	134973.9	199429.1
180114.1	118421.4	135861.2	268505.6	173725.4	185568.3	132028.4	130027.3	140682.5	152296.1
264422.1	115883.9	125979.5	160739.6	168691.3	125581.4	157441.2	145490.6	155518.2	147400.7
221118.9	94162.92	156645.7	143286.5	158793.3	122870.2	152325.8	145796.8	109759.6	161738.5

LP357.152	LP357.152	LP357.152	LP357.152	LP357.152	LP357.152	LP357.152	LP357.152	LP357.152	LP357.152
176441.7	40293.41	116959.1	121213.1	149128.4	115850.7	194569.5	55945.26	150484.3	166689.7
217898	45085.9	111120.3	104378.6	118174.3	97170.03	126708.5	77642.64	146172.3	154889.3
219690.4	51193.23	134062.4	90417.38	212251.9	124515.9	119200.9	76392.92	135408	135249.1
166838.6	47872.6	113986.7	117427.4	114421.6	157340.4	115938.7	42779.79	124639.8	129468.7
171619.8	69198.31	111400.5	127731.8	127355.9	226147.4	137584.1	52287.3	121430.7	166663.4

LP357.152	LP357.152	LP357.152	LP357.152	LP357.152	LP357.152	LP357.152	LP357.152	LP357.152	LP357.152
94508.43	66463.29	63928.76	127826.1	128215	58391.44	60036.18	126276	52381.87	67375.8
98855.74	61351.36	62398.88	113961.3	155090	51370.43	62831.13	128706.6	51071.12	54055.99
125655.8	62600.29	72108.3	91236.9	131686	40769.75	55307.32	138886.1	36966.01	60879.54
92314.25	60076.47	64969.5	113018.6	134186.5	50600.97	51261.02	121579	46402.04	52106.72
100119.3	53434.64	90881.33	115888.8	120630.4	55721.82	57244.49	109431.3	52990.89	65731.22

LP357.152	LP357.152	LP357.153_	LP357.152	LP357.152	LP357.152	LP357.19_	LP357.189	LP357.189	LP357.189
66448.61	158731.4	129697	165035.5	143342	99513.33	135782.9	125738.7	101718.9	109077.6
50258.33	160048.1	130788.7	173626	139111.9	100549	136993.3	131512.9	104127.3	105208
65968.77	156378.7	134992.8	159593.6	134594.2	96226.76	143670.7	130981.8	97917.84	118099.6
54069.73	161906.7	127477.8	156978	131556.6	94025.19	76081.56	69297.2	92966.65	62482.33
60882.6	166838.7	149105.2	164151.5	133363.8	106405.4	88913.27	137795.8	106493.8	114575.9

LP357.189	LP357.189	LP357.189	LP357.189	LP357.189	LP357.189	LP357.189	LP357.189	LP357.189	LP357.189
116718.7	141222.2	141873.3	36471.61	46275.71	194568.5	210431.3	137163.4	141129	123755.3
117756.3	141292.4	151608.3	34240.97	48997.5	209031.5	209136.8	133429.2	140994.5	125683
114606.9	137088.5	149323.8	33266.79	46072.92	217763.8	213893.6	64769.03	131152.3	137585.9
65424.94	131437.3	79097.95	30085.58	41537.67	199733.1	216733.5	139276.3	128713.8	117419.5
126994.5	143486.1	155070.2	35717.36	45017.47	218081.2	212036.2	136517.5	130648.3	118315.3

LP357.189[illegible]	LP357.189[illegible]	LP357.189[illegible]	LP357.189[illegible]	LP357.189[illegible]	LP357.189[illegible]	LP357.190[illegible]	LP357.189[illegible]	LP357.189[illegible]	LP357.19_[illegible]
111668.5	175166.1	146133.4	101065	173545.2	111090.3	130983.9	56874.88	143190.7	106355.7
104669.2	171254.7	145746.5	101991.7	180050.2	54637.08	119111.2	62493.01	149788.9	103767.8
117692.8	161950.7	150011.1	100759.1	186406	98807.57	131364	58841.94	152699.4	94823.75
115747.9	187149.7	146318.2	93857.5	184343.4	109520	128506.9	61529.26	139264.9	96867.78
116732.5	87760.45	132126.7	97919.2	176671.5	113524.3	126741	63384.06	145884.8	106261.2

LP357.189	LP357.189	LP357.189	LP357.189	LP357.19_	LP357.189_	LP357.226	LP357.226	LP357.227	LP357.384
75931.58	123471	69353.4	182464.9	42255.56	198786	187823.4	150820.7	169909.6	23110.73
80805.6	135062.9	74765.16	180804.6	44400.42	92054.79	179041.5	167462.3	157473	24719.73
72862.54	139339	68966.33	190400.3	40932.51	189976.6	182538.6	173469.8	189227	40109.03
79469.74	123555.3	81519.26	93979.9	35552.79	206087.1	182360.4	163142.1	176811.2	47534.52
70761.38	136886.3	84339.43	194817.1	40697.08	198775.2	183566.2	145964.8	183834	52672.5

LP358.155	LP358.258	LP358.259	LP358.259	LP358.258	LP358.259	LP358.308	LP358.444	LP359.167	LP359.167
28742.31	48434.15	38973.22	61139.5	48947.02	45213.81	40603.98	30483.27	168505.9	38547.13
24416.93	47986.96	35610.68	61031.37	48266.86	42111.35	37435.34	23753.61	216363.6	40263.81
25766.32	47989.04	44907.1	68954.36	45427.37	41914.91	38541.13	23531.38	179066.8	35690.01
28759.93	51150.78	38492.74	63272.26	45294.94	40872.22	12626.89	19514.71	178164	35130.33
27370.8	47085.99	37732.21	57431.73	47451.91	43920.62	40301.52	24435.6	176640.5	31819.31

LP359.167	LP359.167	LP359.168	LP359.167	LP359.167	LP359.168	LP359.168	LP359.167	LP359.168	LP359.167
54251.42	134613.3	185559.3	166555.7	142708	157523.4	159363.4	143788.1	179479.3	123226.9
65948.26	220881.9	160358.1	183496.2	155051.3	181281	187097	183180.8	198972.1	143449.1
43459.98	221028.1	150655.1	143400.4	129784.8	165714.5	157593	209241	196598.3	176648.8
52571.51	209363	193633.3	137311.6	142152.3	230567.6	161671.6	206806	206275.1	191896
50218.37	134308.2	182040.8	153053.8	158707.9	221015.8	223727.8	220105.5	170734.5	141267.5

LP359.167	LP359.168	LP359.168	LP359.168	LP359.167	LP359.167	LP359.168	LP359.168	LP359.168	LP359.168
181535.9	174067.1	201385	272665.4	138711.1	167872.8	143394	177034.2	187847.6	129023.2
142053.2	205409.9	128379	188251.4	136002.3	180728.1	156474.9	149457.7	167781.5	163155.2
123798	170189	156685.4	160255.5	114155.9	181598.4	229379.7	197104.9	162355.4	144553.5
193469.2	199034.8	185971.5	234321.3	135343	194462.2	200891.2	127333.4	137380.4	179788.7
145682.9	222998.8	125784.4	222445.8	154576.1	205486.4	190369	146109.4	168333.3	151469.1

LP359.167!	LP359.168	LP359.168	LP359.168	LP359.167!	LP359.168	LP359.168	LP359.168	LP359.168	LP359.168
226159.5	230274.6	338812	106176.2	104128.9	147655.4	207318.6	115806.2	163555	198638.8
149698.1	253377	246411.8	145072.5	139516.1	162290.4	281066.6	118216.9	131828	188147.1
190240.7	237288.4	198814.5	122806.4	193382.2	110342.4	210102.5	143719.8	147069.4	136582.9
145594.2	211760.3	262881.2	117743.9	133312.9	139117.4	162134.2	101274.9	181520.3	136519.7
152593.1	185240.1	205716.5	132078.9	129960.8	170549.5	170762.1	135399.4	159809.8	180408.6

LP359.168	LP359.168	LP359.168	LP359.168	LP359.167	LP359.168	LP359.168	LP359.167	LP359.168	LP359.168
144695	180611	230760.6	137212	138065.1	175552.7	157137.7	134082.5	142681	144690.5
122670.1	284964.1	239159.7	177248.1	130933.2	176735.6	80932.21	208070.6	216600.2	147206.3
162236.4	222138.8	278811.8	144849.3	120757.9	158548.7	100467.5	158215.8	244409.2	154840.2
94461.33	248519.6	245103.9	131780.9	132900.8	127901.2	111162.7	183078.1	169103.3	119016.8
131739.7	212815.2	180395.4	144882.1	213362.3	198936.5	87087.26	145568.6	167183	170361.5

LP359.168	LP359.168	LP359.168	LP359.167	LP359.168	LP359.168	LP359.168	LP359.168	LP359.168	LP359.168
298874.4	147560.8	129281.5	156230.3	229906	105899.8	156240.4	121614.9	127849.2	155354.7
201295.3	135697.2	134642.5	110705.3	131187.4	105019.1	169658.5	117672.1	144139.8	126735.5
194966.6	161327.4	147793.8	92020.88	114350.1	139998.2	179815.8	95420.05	149936.1	117242.3
214607.5	181313.9	173076.1	131272.9	112082.4	87694.27	148855.3	123357.6	141785.5	109922.8
202252.2	263722.7	125356.9	121954.8	128421.2	120606.7	206006.9	132395.1	123406.1	125357.9

LP359.168	LP359.167	LP359.168	LP359.168	LP359.168	LP359.168	LP359.168	LP359.167	LP359.168	LP359.168
201714.3	152671.3	81120.96	149650.3	155623.8	179602.8	175375	124123	80996.58	128718.4
195371.1	120721.9	70633.56	142196.7	158727	204649.4	147836.7	143109.1	74061.82	133658.9
191950.1	140474.5	62527.44	151163.7	192882.5	210283.4	197549.4	91640.72	83687.25	160301.3
156991.5	141294.6	56336.27	209354.6	185394.3	200662.9	150140.2	121537.7	71586.08	134188.3
229991.2	150449.7	59068.68	148270.5	199305.5	192161.9	194473.5	138208.2	85682.52	195604.7

LP359.168	LP359.167	LP359.168	LP359.167	LP359.168	LP359.168	LP359.168	LP359.168	LP359.168	LP359.168
142407.5	102975.4	118357.4	95769.65	96815.02	61245.32	124584.2	123684.2	139631.2	49763.67
168263.6	96018.13	114110.7	130412.4	79202.53	59387.58	158460.8	193791.2	162379.1	60694.63
160652.3	93984.75	113782.8	116356.2	95802.89	74516.07	134247.6	168170.2	118614.6	57768.5
158478.3	114408.6	121126.2	70014.54	98492.56	55326.35	153733.8	125323.2	183628.1	40753.26
150991.1	98424.15	128189.4	81169.35	97127.92	63664.82	157568.2	120274.6	147615.7	51510.95

LP359.168	LP359.168	LP359.167	LP359.168	LP359.168	LP359.168	LP359.168	LP359.168	LP359.168	LP359.168
49119.27	89795.44	51397.22	38985.74	67008.65	38197.78	111110.2	159476.2	192130.3	158916.5
46301.1	74141.72	59441.37	46416.72	67059.5	30760.6	118906.8	187471.7	180831.1	161438.7
46932.77	73956.83	44914.78	36091.66	57545.06	55239.68	175307.4	223319.3	309122.5	162815.7
36345.59	53586.7	68579.89	43069.38	54421.64	30291.33	149349.5	169172.8	252432.8	157809.4
38720.68	67846.48	44849.37	49504.58	65630.7	39789.17	124769.7	238278.6	169339.2	152999.7

LP359.168	LP359.168	LP359.315	LP359.315	LP359.315	LP359.315	LP359.315	LP359.315	LP359.315	LP359.315
57476.15	50365.35	1677891	1646194	673252.5	243698.8	351156	1095911	1125185	319138.6
56455.42	47618.35	1778679	1572306	542840.3	292082.6	553164.7	1051049	929712.1	254699.5
46106.68	43429.62	2176967	1625494	759715.8	268186.1	311443.5	774360	1110657	298185.3
54753.37	73564.82	2587455	920355.2	513103.2	377255.1	413037.3	1162194	1070392	323262.9
48648.44	55073.76	2910423	1650420	722123.8	331138.3	304031.5	882568.6	739871.2	288953.8

LP359.315	LP359.315	LP359.315	LP359.315	LP359.315	LP359.315	LP359.315	LP359.315	LP359.315	LP359.315
310493.6	904105.8	873715.7	958737	210093.3	250386.8	1108483	402427.8	1202352	276024.9
385847.1	1536174	1264047	1109286	190354.1	373066.2	688150.9	439600.8	1360144	397492.1
394873.7	1052761	993210.4	956985.2	223613.4	247871.6	939995.9	376518.4	1088399	257733.1
485871.3	1054518	939872.1	1268257	191364.8	316946	933811.5	250006.2	1462324	317545.5
322528.7	1384340	737380.9	860049.1	203632.3	230138.3	733308.4	320345.8	1529169	340221.6

LP359.315[illegible]	LP359.315[illegible]	LP359.315[illegible]	LP359.315[illegible]	LP359.315[illegible]	LP359.315[illegible]	LP359.316[illegible]	LP359.315[illegible]	LP359.315[illegible]	LP359.315[illegible]
936355.7	376489	396945.6	952689.9	858568.1	452210.7	406627.9	416125.2	288539.1	584614
967751.7	284068.8	409083.6	845191.1	950925.3	502992.8	579074.3	485168.9	389284.9	423407.3
901273.6	522077.4	355818.2	761237.5	614952.6	529180.5	539498.6	557124.1	293033.3	616053.5
725859.8	362831.2	419314.3	707749.1	743609.6	444049.8	419403.7	449245.7	245559.9	569112.8
915743.6	382935.6	378886.8	765183.4	1147713	431077	437322.2	498086.1	475772.3	614310.6

LP359.315	LP359.316	LP359.316	LP359.315	LP359.316	LP359.315	LP359.315	LP359.315	LP359.316	LP359.315
328256.9	475094.2	359985.3	443415.2	257165.4	291198.8	390894.4	420152.6	161819.3	378995.9
393777.8	645630.8	341386	434424.3	285892.7	220023.6	344509.8	514682	238379.3	387117.8
280173.3	717087.9	380857.1	345962.1	266438	273689.2	471844.2	436452.3	259191.9	307173.6
237562.8	502607.3	430947.7	357673.1	273178.7	295232.1	418108.1	459426.2	246425.9	327798.6
217488.4	388333.3	356759	567516.5	270685.8	272365	308774.4	464819.9	233042.9	462570

LP359.316	LP359.316	LP359.315	LP359.315	LP359.315	LP359.316	LP359.315	LP359.315	LP359.315	LP359.316
307163.4	362518	336993.9	249985.2	195756.1	247026.9	177271.7	243167.6	260225.9	275466.4
348533.6	539710.1	350797.1	244413.5	189906.4	259942.4	168646.2	279705.9	265649.5	327667.3
287397.1	472238.8	339539.8	297555.4	219778	260852.2	215308.8	257573.6	341094.4	255345.9
349460.6	345619.2	324497.7	218306.2	214194.8	268064.4	179749	232996.3	239649.8	318337.8
271601.1	546183.7	322397.4	208694.1	243354.3	239757.2	183377.7	231247.9	229293.1	298512.9

LP359.316[illegible]	LP359.316[illegible]	LP359.315[illegible]	LP359.315[illegible]	LP359.315[illegible]	LP359.315[illegible]	LP359.315[illegible]	LP359.315[illegible]	LP359.315[illegible]	LP359.315[illegible]
465306.8	220583.6	190066.8	246476	253877.1	255926.9	179665	215788.2	260568.8	223622.5
360943.3	251479.9	207501.8	277501.1	260224.2	272927.8	242078.7	215827.5	267287.2	229845.4
365517.5	303932	189196.4	230904.2	245836.1	272300.8	239103	221819.8	288338.6	231111.8
347670.9	249696.8	176312.5	238524.7	237441.7	235658.8	236065.7	211525.2	270494.6	219032.5
341342.6	237859	176660.5	243049.2	247747	236952.5	248394.7	195772.7	262540.9	212239.2

LP359.316	LP360.237	LP360.238	LP360.238	LP360.238	LP360.238	LP360.238	LP360.238	LP360.238	LP360.238
186955.6	72197.17	79687.33	65476.55	74413.43	87495.81	89705.17	80470.92	69326.81	61605.16
184960.2	77198.73	66022.83	75113.65	63763.66	92434.57	69168.1	71052.96	64639.29	67021.18
237419.8	73646.79	80447.01	97415.61	73930.23	85303.24	65179.56	73766.17	61833.14	59040.17
183268.1	71934.8	77311.19	57395.18	70577.47	80933.52	66299.2	71715.57	74258.25	72840.02
172731.8	69833.28	74337.3	64327.47	69112.99	87849.16	71044.2	83224.21	60111.47	72514.5

LP360.238	LP360.238	LP360.238	LP360.238	LP360.238	LP360.238	LP360.238	LP360.238	LP360.238	LP360.238
91509.21	67455.13	66911.72	61329.79	67648.29	68826.17	91760.36	77638.56	55600.36	48933.02
88458.75	67978.7	62425.6	63237.46	81005.23	65773.91	86055.16	83064.28	63301.92	48532.08
90124.82	58542.14	69332.1	64472.25	69055.2	61702.58	84648.72	80786.44	55058.33	45651.87
91687.33	63844.6	72204.55	65587.96	71039.7	64147.37	82113.19	79671.12	53599.58	49818.25
75123.01	56944.36	70634.31	64442.12	73144.31	64797.66	83835.17	77684.91	59741.51	46784.9

LP360.238	LP360.238	LP360.238	LP360.238	LP360.238	LP360.238	LP360.238	LP360.238	LP360.238	LP360.238
62823.06	66933.46	66307.34	63832.27	48162.05	55595.23	54670.06	68193.44	74106.38	64968.53
61999.98	74884.96	64638.77	58895.42	50140.04	63294.26	55588.08	65554.15	65784.6	64198.44
63334.33	70398.87	57965.36	58190.01	52175.68	58659.7	48732.16	75034.75	68693.74	61432.65
69197.1	76237.4	62783.49	59290.55	45234.82	55697.72	52763.94	62414.58	73066.28	65554.45
62815.95	74586.37	64213.97	59724.53	50070.49	55748.55	53741.52	68049.86	70953.19	62745.27

LP360.238	LP360.238	LP360.239	LP360.238	LP360.238	LP360.238	LP360.238	LP360.238	LP360.238	LP360.238
56255.43	68476.38	58584.81	59559.1	59730.54	65147.09	70516.77	54242.43	82773.6	67260.07
62329.02	70876.2	58470.98	56840.09	69087.46	72006.25	72951.06	45509.33	86142.56	70898.68
57684.44	74223.03	63474.18	55675.29	61392.82	64561.56	75921.56	51979.39	81306.77	73549.85
56296.16	73449.07	60350.34	61369.5	63651.38	68956.63	63561.88	64760.54	81640.54	67419.24
52635.76	71406.71	55638.21	66151.8	55904.83	63170.53	66885.1	56638.29	82001.7	75087.27

LP360.238	LP360.238	LP360.274	LP360.274	LP360.274	LP360.274	LP360.274	LP360.274	LP360.274	LP360.274
72057.74	51329.75	69139.66	188062.2	82122.9	80028.26	63884.5	60589.65	62671.81	58477.02
77037.65	49557.47	82943.58	205515.3	84630.6	75335.73	53816.27	84136.47	73459.68	64237.26
83504.53	52981.94	80149.68	216745.5	83036.89	72852.27	56995.94	81907.13	75465.87	60891.4
75574.72	48275.44	71649.15	218780.2	81818.71	63832.09	79871.56	80293.47	66442.06	60414.99
76197.04	49081.85	91815.85	221180.5	98959.1	75729.24	60042.57	82047.32	65912.26	53333.48

LP360.274	LP360.274	LP360.274	LP360.274	LP360.274	LP360.274	LP360.274	LP360.274	LP360.274	LP360.274
180161.6	75836.63	70660.59	170860.9	154281.7	69535.44	55184.06	51174.75	83468.94	76130.31
184541	75179.76	70323.84	180053.3	156119.8	66950.39	54611.45	52425.28	92618.59	72318.96
192383.7	72284.17	70696.43	190215.4	166181.5	70171.95	55267.95	52841.19	83473.07	74653.23
183290.2	69350.71	63078.47	212281.2	151860.4	70208.09	57412.64	55113	77242.89	74955.69
181761.6	70214.6	69729.58	192530.6	104343.8	56280.7	56751.83	54575.52	67921.53	63168.25

LP360.274	LP360.274	LP360.274	LP360.274	LP360.274	LP360.274	LP360.274	LP360.274	LP360.274	LP360.274
60435.52	75244.59	92871.68	61942.25	86570.57	121136.4	85560.53	74930.61	53257.74	59399.07
57119.39	72567.7	94933.16	71886.43	83194.73	119940.9	108150.9	71884.73	59697.78	61877.63
57188.95	73601.02	105006.1	68899.4	86375.8	124706.2	108136.9	72329.85	54709.29	61772.14
57379.17	76639.88	95984.33	67753.39	84267.12	111820.7	103124.7	77863.46	55524.1	58649.17
58775.62	69319.09	86170.5	58580.46	77022.18	86224.9	100359.2	75370.29	59449.44	62272.1

LP360.274	LP360.274	LP360.274	LP360.274	LP360.274	LP360.274	LP360.274	LP360.274	LP360.274	LP360.274
127750	85276.06	212954.9	81468.27	64153.57	92166.66	77049.98	48303.64	184732.2	202276
131734.1	90161.7	209562.3	77204.53	61478.72	81989.33	89915.04	51022.26	187427	207707.4
124716.7	88314.43	227355.7	84081.67	61463.21	76440.54	90166.58	48441.29	204190.8	209499.4
131184.8	80752.52	228775.7	76972.4	60244.24	84606.56	83976.66	54657.68	184749	202257.2
81815.62	79938.61	213071.2	83064.16	59861.92	81312.13	71274.39	56969.96	192106	208600.1

LP360.274	LP360.274	LP360.274	LP360.274	LP360.274	LP360.274	LP360.310	LP360.319	LP360.319	LP360.319
71081.19	57265.77	76888.74	299455.4	39193.98	57036.74	84711.04	239794.5	189508	157673.5
65914.84	50901.19	72072.09	315022.7	46191.2	56445.61	96249.37	248279.9	195203.6	154644.8
70152.12	53616.76	80643.9	308858.8	40511.43	59368.47	89027.91	280558.3	179134.9	167764.6
69042.05	56515.04	78940.63	307099.1	40152.76	56465.19	76499.56	226972.2	177475.2	150676.4
58801.78	53426.93	80259.35	308375.9	42698.47	51163.29	71763.86	302497.4	221839.6	183750.7

LP360.319	LP360.318	LP360.319	LP360.319	LP360.32_	LP360.320	LP360.320	LP360.319	LP360.319	LP360.320
238583	199288.1	135022.7	130707.4	99278.14	77264.21	67588.73	66105.22	102233.4	85714.31
246225	212874.3	138968.1	123661.4	101408.8	79762.98	71809.36	70379.75	94973.1	85750.46
240778	215800.1	140084.1	122834.3	112539.8	81137.21	65671.45	77112.22	102812.2	88854.66
263691.6	173374.2	165902.4	126281.7	101996.5	84998.34	70430.9	60471.45	100761	77956.69
307940.2	233153.1	130016.7	122547.5	89467.25	80652.63	71310.51	68124.03	87991.54	83905.73

LP360.319	LP360.319	LP360.32_	LP360.320	LP360.319	LP360.320	LP360.319_	LP360.320	LP360.319	LP360.319
51774.96	60061.51	83830.41	78043.75	71947.25	137676.4	93354.42	70910.75	101069.8	59012.23
51485.76	58367.15	77620.21	76449.13	70076.67	167169	98474.89	81364.39	96308.08	63074.19
52153.37	57156.24	80910.89	78737.88	77719.55	145848.2	93929.15	78669.7	97663.4	61716.05
52844.51	62265.44	71723.72	77886.69	81785.14	156992.9	94223.85	66496.64	97607.75	58985.74
49418.5	54203.45	82653.35	81387.53	69307.8	120529.1	88701.25	70910.36	82854.02	57739.27

LP360.320	LP360.323	LP360.324	LP360.324	LP360.324	LP360.347	LP360.347	LP361.168	LP361.168	LP361.168
49970.43	2026264	245874.8	208441.9	224933.4	205782.1	211867.6	527906.3	367047.5	217216.6
58510.64	1812875	239114.4	198862.1	247195.6	212715	206270.3	524799.7	351565	214529.4
52230.28	1708439	234137.3	193060	230324.6	199087.1	206971	492206.6	331057.8	181268
45697.57	1542077	225248.1	322198	295047.1	188894.3	210377.7	463135.5	313411.4	175713.5
47667.1	1871448	251820.8	151242.7	215681.7	186245.3	216213.7	445361.9	300589.2	174086.6

LP361.184	LP361.222	LP361.222	LP361.222	LP361.222	LP361.222	LP361.222	LP361.222	LP361.222	LP361.222
101463.3	620866.8	716082.9	576661.4	644428	567739	408453.4	651745.5	682907.4	609654.7
950508.2	611735.8	734821	559419.1	633386.6	506780.1	392740.6	656276.9	689243.6	621919.4
1019792	639488.5	737173.2	596910.5	657567.9	597203.1	426937.1	617539.3	710540.1	596080.6
992779.3	587273.8	681705.7	592024.3	609034.6	569146.1	417634.7	650186.9	698583	650615.6
1043015	575772.2	674331	626486.9	630366.6	555237.6	434247.4	609286.4	686929.4	616341.4

LP361.222	LP361.222	LP361.222	LP361.222	LP361.222	LP361.222	LP361.222	LP361.222	LP361.222	LP361.222
1065358	979957.1	733678.6	399928.2	663702.5	341585.5	321872.9	324738.7	396604.2	473547.4
1135037	1015832	792928.4	441184.1	695251.6	281239.9	336776.6	324423.9	381801.3	471671.4
1165990	1033214	792093	405621.7	727887.7	336096	327527.7	338131.4	361170.3	462217.1
1196852	1064708	727918.2	455056.2	675065.4	332137.3	307802.4	348921.9	361243.6	464779.4
1181660	1065961	748170.3	453847.6	694969.1	342312.6	338061.7	309927.5	381920.2	456366.9

LP361.222	LP361.222	LP361.222	LP361.222	LP361.222	LP361.222	LP361.222	LP361.222	LP361.222	LP361.222
225617.9	586571.2	391731.9	314062.1	301579.1	326922.3	336450	124037.1	402505.6	359543.8
236705.5	584218.8	420533.3	330717.5	313629.9	329984.1	318619.2	123541	359014.3	340956.3
232057.1	608996.5	415461.1	348712.3	293173.8	312923	349310.9	130985.5	380215.4	351424
233604.3	634557.7	388045.8	328102.3	302448.2	334402.9	334094.4	124204.1	374765.9	349057
241120.5	627352.8	386967.8	329932.3	297112.5	304869.4	349101	128190.2	388052.7	343627.5

LP361.222	LP361.222	LP361.222	LP361.222	LP361.222	LP361.222	LP361.222	LP361.222	LP361.222	LP361.222
366362.1	339010.7	268489.8	77027.99	337449.2	802355.2	365090.8	927896.2	1055870	919856.6
357151.1	332276.2	252168.4	76649.49	368648.6	804814.3	407529.8	893334.8	1058513	836825
383377.6	336142.6	260621.9	75149.89	354092	832922.8	407380.8	966804.6	1062366	913944.4
349055.2	333074.9	269278.4	67978.89	339900.5	786589.9	379056	1006508	1034341	850549.9
343960.4	305926.6	261094.6	80237.57	336790.2	850268.8	388072.2	1046785	1052646	1025837

LP361.222[illegible]	LP361.222[illegible]	LP361.222[illegible]	LP361.222[illegible]	LP361.222[illegible]	LP361.222[illegible]	LP361.222[illegible]	LP361.222[illegible]	LP361.222[illegible]	LP361.222[illegible]
779141.5	465466.4	925109.7	1193030	425568.7	652988.4	732054.9	711398.2	705102.6	831293.8
767643.1	458854	926357.1	1055403	420633.9	658900	672890.5	693826.7	724371.7	821589.6
741693.7	471007.3	947404.6	1168391	414881.1	673122.2	718608.2	703911.7	728657.4	811761.2
778827.4	486814.4	970352.6	1278691	449586.6	679025.8	750110.6	723974.7	739645	856827
771521.2	495762.7	1031407	1247545	452361.3	687279	734380.2	734537.4	809368.4	799235.7

LP361.222	LP361.222	LP361.222	LP361.222	LP361.222	LP361.222	LP361.222	LP361.222	LP361.222	LP361.222
867201.7	568324	589247.3	447448.1	534527	329984.6	503060.8	83275.22	495768.9	323497
898244.3	555955.7	605663.8	451998.2	587522.3	332638.9	506170.7	75560.61	551468.3	364474.7
971378.2	595284.7	640874.8	432743.8	624160.5	350966.8	549953.9	72421.55	556180	354827.9
1031456	618360.8	581697.3	498171.7	621772.1	337089.9	560021.6	66154.43	581088.5	361121.6
973920.8	632045.2	594654.3	493915.9	591620.5	343624.5	559824.8	68347.54	567889.5	355193.1

LP361.222	LP361.222	LP361.222	LP361.222	LP361.222	LP361.222	LP361.222	LP361.222	LP361.223	LP361.222
768685.4	397121.2	643822.3	448129.1	225701.5	402839.6	359176.4	365653.9	775624.6	384857
808630.7	368324.8	649272.8	430639	224434.9	392054.2	345738.6	399049.6	798827.1	385616
753486.6	371486.4	668731.5	451679.3	227341.6	452203.5	361900.5	372574.4	807007.7	380612.2
761757	355720.9	698781.7	464697.6	232653.7	412428.9	368640.3	406764	784535.2	382344.6
781808.2	358433.4	717856.9	453691.6	223007.1	400280.1	355677.6	426282.8	796610.5	381318.8

LP361.222	LP361.270	LP361.332	LP362.226	LP362.226	LP362.226	LP362.227	LP362.226	LP362.226	LP362.226
485468	26148.94	16385.89	148152.4	221588.9	150886.7	93533.48	130147.2	223979.9	253950.5
499922.4	26876.8	14298.44	160360	225338.5	127994.5	84572.25	128704.3	205845.8	241196.1
493112.2	31325.03	15619.32	153276.8	207509.9	126250.2	89691.44	131266	213442.9	224898.4
505560	26907.88	14088.22	147635.3	225337.4	133145.6	90551.04	131979.7	226903.6	258047.7
501955.8	27797.83	13501.29	238372.1	227989.6	133820.5	90254.44	131453.7	233949.6	242153

LP362.227	LP362.226	LP362.227	LP362.227	LP362.226	LP362.226	LP362.226	LP362.227	LP362.253	LP362.253
250277.8	172314.3	145100.3	75080.58	97556.55	99388.44	134175.5	121031.6	136272.2	122293.4
264398.5	189981.4	136358.2	70269.61	93587.15	102503.8	137491.5	124638.4	162882.3	103334.6
258609.1	189520.9	136137.4	69256.94	103645.5	86901.11	125281.1	126945.5	180155.6	157560.7
259847.5	178374.9	134332.9	66405.83	89326.09	89936.14	132549.2	117811.2	128334.8	131346.1
247023.1	179919.6	145770.8	66788.84	89659.02	96956.12	137945.4	125790.9	161441	119928

LP362.253[illegible]	LP362.253[illegible]	LP362.253[illegible]	LP362.253[illegible]	LP362.253[illegible]	LP362.254[illegible]	LP362.254[illegible]	LP362.253[illegible]	LP362.253[illegible]	LP362.254[illegible]
151974.6	124533.3	151307.8	162629.6	200340.5	189111.3	142866.3	148446.5	127089.7	155570.1
135797.7	113594.3	163604.6	140422.1	202116	178439.9	157401.9	103870.7	138382.3	136766.9
202164.3	109536.7	144241.1	229331.3	148011.4	146241.6	143292.7	124753.2	150851.1	122320.4
111716.8	175742.2	166200.1	135961.1	172714.7	167568	240334.8	103052.6	149722.4	102959.2
152720.2	105609.1	148076.2	208788.6	164532	141935.8	164573.7	154193.3	125905.5	134468.1

LP362.254	LP362.253	LP362.253	LP362.254	LP362.254	LP362.253	LP362.253	LP362.254	LP362.254	LP362.254
145704.9	110526.5	175951.4	106224.8	116424.3	122041.6	101585.9	135894.4	143286.9	89644.13
138483.7	101460.4	169978.2	103096.9	117015.1	125692.9	107022.6	142783.3	153092.7	120513.7
159861.9	105320.3	158152.2	197965.6	141763.3	133573.9	112224.2	138737.1	140632.9	122298.7
115984	142919.4	162527.8	117508.4	117671.9	120396.1	128229.2	108684.6	142314.6	90441.33
142380.8	102723.7	171048.5	116366.4	106062.9	121391.9	104450.4	149026.9	146195.2	83838.71

LP362.253	LP362.254	LP362.254	LP362.253	LP362.254	LP362.253	LP362.254	LP362.254	LP362.254	LP362.254
123436.2	104230.4	97709.73	137943	112862.4	108168.4	110458.6	118102.8	89794.65	147282.2
205361	107012.9	93615.83	143374.9	112491.2	110608.9	103708.2	121439.7	94040.34	169496.3
144738.5	103498.9	111210.2	153293.8	117375.1	96873.52	150139.5	109349.3	87288.8	155374.5
109265.6	108233.8	101566.9	146093.8	129495.3	102894	115814.3	104701.8	99632.08	155374.4
111343	104431.8	105749.7	140747.8	94632.48	90850.35	97176.24	113124.5	102372.7	138111

LP362.253	LP362.253	LP362.254	LP362.254	LP362.254	LP362.254	LP362.253	LP362.254	LP362.254	LP362.254
115914.4	96133.44	125275.9	98698.91	90360.1	114287.4	86184.2	97125.28	132087.6	124939.5
128064.9	89991.1	120247.3	101272.2	93275.15	119470.1	90514.66	81948.55	129036.4	127268.8
121785.9	108960.8	122697	114490.4	98983.82	102936	98212.91	132352.6	133111	120081.1
113004.9	97406.01	128807.5	106094.6	115430.6	145159.3	145329.8	93660.42	115943.3	128097.4
167869.2	93637.59	136214.5	105813.8	92851.73	112136.2	81659.05	94503.83	125596.9	128828.3

LP362.253	LP362.254	LP362.254	LP362.254	LP362.253	LP362.254	LP362.253	LP362.254	LP362.254	LP362.254
167586.7	119845.6	133969.9	100787.9	79152.42	126552.3	95087.41	131228.6	166769.7	86963.93
157892.7	122349.1	120470.6	94746.39	83352.63	122040.5	99663.39	128829.8	158684.9	95664.87
191565.2	110555.5	120536.4	88017.19	90781.07	153765.4	122171	131043.5	163096.4	88217.81
180329.4	125459.3	130381.1	98804.21	107126.7	126852.4	103234.1	151155.6	159672.3	117370.8
166725.8	122188.3	130625.4	96121.52	84710.91	119111.8	87547.28	136686.8	165322.7	96950.43

LP362.254	LP362.254	LP362.254	LP362.253	LP362.253	LP362.254	LP362.290	LP362.290	LP362.290	LP362.290
142839.3	220419.2	161649.1	138960.9	57483.95	82881.49	89014.44	112375.1	84771.41	95177.77
160251.3	221788.1	154194.1	141225	60567.9	68371.26	82933.43	120559	80347.6	91118.84
147626.2	218655.4	173512	156766.3	67931.7	77277.07	80309.17	112459	82116.1	89325.1
130226	223063.9	148666	143216.9	59620.34	76788.67	82562.94	113526.5	76723.18	92709.2
172171.7	214651.9	150286.4	136278.8	56458.63	71891.46	82224.22	118174	78959.62	77120.52

LP362.290	LP362.290	LP362.290	LP362.290	LP362.290	LP362.290	LP362.290	LP362.290	LP362.290	LP362.290
116353.6	78743.76	123969.6	106703.8	89261.48	165731.7	114730.5	137991.5	106442.6	191512
112099.1	79107.22	127680.2	98809.43	76038.25	157238.3	123490.2	131814	112375.7	186131.7
117888.1	83685.84	119470.2	101353.6	83074.44	150091.9	120420.2	130105.8	95267.11	181756.9
116651.6	74324.56	124132.7	88647.9	80970.9	150557.2	109241.2	133793.6	96991.4	206947.3
120377.5	80385.36	118273.2	96204.09	85232.2	144529.5	109430.9	129491.5	118684.5	193246

LP362.290	LP362.290	LP362.290	LP362.324	LP362.339	LP362.339	LP362.926	LP363.111	LP363.182	LP363.289
75438.57	112883.2	203556.7	105537.6	139410.5	146598	21438.44	16153.54	160973.7	42127.74
76089.18	113301.8	197728.5	101477.2	142622.9	136801.8	20728.11	19525.42	139228.2	33571.58
77818.84	109749.8	191679.7	96069.13	154300.2	137317.6	16304.65	17743.71	123740	34733.34
81244.36	114299.7	207249.2	96962.8	149949.9	136090.4	17126.64	15514.02	138766.4	36603.25
80564.39	108470.1	204475	94721.01	152278.2	129987.2	16786.39	20528.95	142568	40112.22

LP363.288	LP363.288	LP363.289	LP363.288	LP363.288	LP363.310	LP363.326	LP363.326	LP363.343	LP364.185
44257.26	54303.57	31306.74	41556.46	37140.49	54409.43	24040.84	36390.28	27627.93	36651.01
44630.1	66511.71	32324.94	37895.12	32839.96	42645.31	23224.75	33957.26	32670.82	34939.93
34686.53	50843.7	30934.65	41771.11	38393.67	50606.09	25488.41	40438.43	29739.91	159886
43944.33	47924.63	32146.96	45232.61	36463.49	39626.36	23857.52	36492.29	32929.19	140458.7
46460.4	53016.69	32407.32	44134.48	35921.47	42360.51	20674.85	37715.98	32508.44	137172.5

LP364.196[illegible]	LP364.197[illegible]	LP364.197[illegible]	LP364.196[illegible]	LP364.197[illegible]	LP364.196[illegible]	LP364.197[illegible]	LP364.196[illegible]	LP364.196[illegible]	LP364.196[illegible]
107930.2	94341.59	106940	118380	88482.27	157261.1	80805.36	83595.89	91715.84	93993.68
106701.8	93689.49	106479.2	108134.8	84370.44	154393.4	78302.88	83954.1	88899.28	98407.18
102286	111278.3	98684.38	126833.1	93962.11	164097.4	79442.1	90418.59	93538.71	92806.3
115755.8	109844.2	108886.4	136084	91340.07	84538.56	77460.05	92310.05	98331.65	104585.1
55165.14	60170.7	50969.63	67390.11	59771.84	150814.4	47420.81	44186.89	48725.11	55894.65

LP364.196	LP364.196	LP364.196	LP364.196	LP364.196	LP364.196	LP364.196	LP364.196	LP364.196	LP364.196
134194.9	96161.34	139880.1	109515.3	128261.2	82018.81	111291.4	99211.77	107012.2	141392.2
124707	95534.68	141332.7	108442.9	119384.9	89430.95	114181.3	97600.62	104224.1	136324.1
148617.5	95257.28	141156.1	101496.2	133894.2	92665.2	132910.3	97019.03	115062.7	142457.4
147604.4	102146.7	148434.8	117524.1	134558.9	96376.45	131709.1	110694.8	116527.8	145413.3
75445.22	52913.61	69023.29	58484.6	68278.09	45820.43	63427.5	57984.61	57588.52	74693.23

LP364.196	LP364.196	LP364.196	LP364.196	LP364.196	LP364.196	LP364.196	LP364.196	LP364.196	LP364.196
100152.5	102480.7	116208.3	99605.69	77266.95	114851.1	68439.45	79357.22	70915.25	98469.34
103289.5	113094.6	121109.9	116310.6	80279.15	124644.5	67508.67	72104.21	73862.11	97503.54
112213	114666.2	126385	113000.5	76356.76	138967.5	60113.42	86341.01	68567.18	101945.7
113145.6	113443.6	126313.5	111617	92097.54	133406.6	74378.98	83053.13	76430.16	103936.6
57830.8	65648.84	58705.03	56382.79	43108.58	66165.32	35815.47	40960.19	35178.61	48030.35

LP364.196	LP364.196	LP364.196	LP364.196	LP364.196	LP364.196	LP364.196	LP364.196	LP364.197	LP364.196
98818.07	118760.2	102415.3	122378.6	126936.8	129185.6	92851.11	71407.45	89108.95	110961.8
101371.6	136198.4	113233.1	130905.7	118946.3	120871.4	84406.64	75366.29	85722.81	113180.5
112537.6	131153.3	115528.5	137907.3	130808.7	137357.6	93365.03	76548.48	85554.75	130059.9
107786.6	138332.3	108410.1	139393.2	139112.5	138427.4	89087.25	76581.42	84262.1	129615.1
52394.41	68602.18	64893.94	70792.84	74167.06	71091.93	53085.09	46535.8	45196.38	60111.78

LP364.197	LP364.196	LP364.196	LP364.196	LP364.196	LP364.196	LP364.233	LP364.233	LP364.233	LP364.233
139295.7	119463.2	136598	95717.84	71813.78	147201.7	144479.3	118213	95292.22	156019.8
140373.1	111248.2	150663.6	97509.69	70934.98	155255	131977.4	108006.5	93595.33	141356.3
136108	126886.6	157518.3	98503.86	74018.97	153872.2	131221.2	128234.3	106733.9	147833.3
139891.3	141657.5	142240.6	98474.71	75002.43	146707.2	138407.4	116326.1	118184.3	148172.1
82837.43	56531.02	70598.57	52528.88	40733.72	152387.2	136341.2	114829.7	107658.5	146428.7

LP364.233	LP364.233	LP364.233	LP364.233	LP364.233	LP364.233	LP364.233	LP364.233	LP364.233	LP364.233
78390.24	163491.7	92022.75	128188.9	103181.1	161781.1	150194.1	118006.1	98705.98	120351.8
75447.7	161379.4	89242.9	122671.8	103932.6	162035.2	150628.1	105179.7	98636.74	105586.6
80104.16	162392.6	97915.7	129601.7	95453.44	157931.9	158673.3	112088.5	83118.55	120413.3
76370.14	162206.6	96298.23	113748.6	98864.37	155708.1	155857.9	109125.3	91171.13	122836.6
77555.14	161684.2	99919.78	120728.2	100394.2	141890.1	142570.2	95956.49	99960.9	123300.7

LP364.233	LP364.252	LP364.269	LP364.269	LP364.269	LP364.269	LP364.269	LP364.269	LP364.269	LP364.269
142523.6	184520.1	123341.9	77431.51	159105.1	109506.4	118989.4	75503.52	127250.6	130828.6
145532.9	150815.5	72502.85	82670.58	153769.7	89819.26	76675.94	82726.72	84304.58	126736.1
155962.8	151020.1	75563.61	70101.32	142193.5	83616.5	76307.02	73479.8	132785.7	123584.9
140274.5	151721.1	84364.39	74657.15	136385.5	126247.9	125488.1	83771.8	118855.6	104301.7
133828.5	163970.2	94885.38	83200.9	208110.4	87740.49	73993.14	70801.46	127953.8	161940.8

LP364.269	LP364.269	LP364.269	LP364.269	LP364.269	LP364.269	LP364.269	LP364.269	LP364.269	LP364.269
105424.6	77719.8	130744.4	104049.5	83156.15	91816.29	106873.1	71303.33	70963.28	75705.3
98258.22	75931.87	137810.8	104488.8	75495.68	98722.18	100345.5	78501.18	78251.44	63426.33
71564.4	100448.5	130167.5	102164.9	80230.49	89510.36	106870.7	83743.5	64939.15	96110.84
75484.12	82459.97	119564.9	139119.6	83658.31	120719.7	99645.72	80014.77	101323.2	66441.53
74848.94	79654.67	137258.7	93566.96	82928.79	91288.3	98699.58	73120.43	73072.96	74381.7

LP364.269	LP364.269	LP364.269	LP364.269	LP364.269	LP364.269	LP364.269	LP364.269	LP364.269	LP364.269
93573.13	99808.1	63090.18	113320.6	88950.21	67927.69	92901.08	72018.91	92085.12	80688.65
86760.28	100748.2	57230.12	86622.07	90447.98	69642.94	92775.59	62654.14	87397.85	82837.14
83828.84	106989.7	58189.43	116369.3	92129.29	67518.81	88022.7	65953.87	91765.28	80045.67
81821.34	96643.26	53611.26	111602.7	90849.51	71555.15	86075.65	70201.75	86741.19	75159.7
80018.34	97073.32	65182.45	122935.2	91431.53	60847.5	90419.96	62594.65	86315.58	84449.75

LP364.269	LP364.270	LP364.269	LP364.269	LP364.269	LP364.269	LP364.269	LP364.269	LP364.269	LP364.269
74267.84	89776.52	99411.33	80169.51	110526.1	74511.01	91327.08	112918.9	86751.22	87822.57
67810.68	86898.07	104173.6	83408.93	70528.47	75155.49	83327.73	122791.1	93260.24	66813.47
70545.99	95530.44	101676.4	109569.3	72728.42	86005.32	84063.02	112437.7	92207.59	87425.01
69157.19	93038.18	105868.8	73335.48	83854.66	69954.56	92469.72	120087.8	92283.73	85848.63
72829.17	92628.72	99823.51	78613.81	77727.52	66517.55	81138.39	114973.6	88866.11	84197.58

LP364.269	LP364.269	LP364.269	LP364.269	LP364.269	LP364.269	LP364.269	LP364.269	LP364.269	LP364.269
117077.3	93032.85	89867.98	77412.89	83556.5	83495.11	57404.82	68515.66	73165.44	62264.75
118353.3	87843.6	93333.18	78851.89	91615.72	74652.98	56430.66	72771.61	73076	64640.94
109872	101695.6	96381.82	84692.66	93515.35	77356.18	57816.41	71802.36	72552.82	65252.77
116463.2	98529.34	95701.03	78056.34	85361.71	78549.04	57638.33	74506.56	75995.66	62867.45
119165.7	93086.72	89852.25	81233.04	82371.35	73590.56	54629.26	70923.84	62767.04	56593.08

LP364.269	LP364.269	LP364.269	LP364.269	LP364.269	LP364.269	LP364.269	LP364.269	LP364.284	LP364.342
38308.37	56537.67	86274.42	114036.1	58586.07	83374.32	66882.67	96948.77	15702.5	44890.49
41542.06	52595.31	86190.72	112599	63937.99	93184.34	69825.2	98825.19	14810.69	89331.7
42218.81	51634.95	80969.34	112559.8	66186.44	88951.69	71149.32	99437.8	16992.4	101327.9
39461.5	50253.45	80033.11	110422.7	57490.49	81340.84	64577.15	102080.3	16355.29	89774.54
46248.35	49349.42	80147.32	101347	60429.51	76427.28	59614.19	88227.14	19464.63	91505.41

LP364.341	LP364.342	LP364.357	LP364.357	LP364.462	LP365.255	LP365.303_	LP365.302	LP365.305	LP365.305
117345.8	38924.19	87423.82	40138.76	162009.4	58154.13	188750.4	118955.9	55091.96	33488.57
112192.1	44113.27	86521.24	43096.62	159821	68777.23	200478.2	126042.3	50537.26	33062.1
104242	47216.46	87512.8	38525.24	151631.8	50499.97	182437.8	139977.5	51801.33	30010.8
105055.1	40355.7	70718.11	39738.93	138279.4	65584.23	174992.9	143632.7	47303.3	30970.7
95519.02	39291.85	86098.68	29405.13	156776.7	70109.39	176639.8	152569.8	51982.23	30702.44

LP365.305	LP365.305	LP365.305	LP365.305	LP365.305	LP365.305	LP365.305	LP365.305	LP365.323	LP365.341
31745.84	39449.06	37177.15	38799.71	47512.78	45295.94	42594.18	36885.95	63620.96	84445.9
36581.98	50618.06	37008.07	46346.99	45807.06	45786.94	40705.27	37452.63	62116.01	76639.05
41124.39	41411.15	35904.31	39426.7	46717.51	43426.96	43190.6	35656.33	57311.61	106865.8
31876.76	44063.16	37043.77	39267.19	45088.25	38829.92	40496.64	36286.59	58108.56	70678.21
32421.59	43155.31	37696.77	46218.42	45152.59	44079.42	39766.9	36330.62	62000.47	76568.1

LP366.212	LP366.211	LP366.212	LP366.212	LP366.212	LP366.212	LP366.212	LP366.212	LP366.212	LP366.264
78042.68	51252.01	54772.06	68539.49	44998.13	75627.39	72048.52	61801.91	42424.6	1605009
60986.94	60242.9	58279.17	65162.55	48721.22	78487.8	70785.49	55238.65	42123.21	1387815
77915.08	59181.59	49602.13	60055.25	51709.02	83071.91	69558.93	50306.92	41988.12	2015141
81903.58	55915.1	49663.57	64198.41	49977.01	77044.12	68906.51	58520.38	39780.54	1670239
73284.69	50351.88	50291.18	62411.42	46816.19	72592.35	62421.95	59006.11	50212.82	1630642

LP366.264	LP366.264	LP366.263	LP366.263	LP366.264	LP366.264	LP366.263	LP366.264	LP366.264	LP366.264
285111.8	1433496	1034645	887580.8	1505997	947753.8	1302491	735031.5	996622.1	1482836
299072.8	1668198	1354544	1469881	1014547	1383056	1307662	971258.8	798593.5	1332487
203769.4	1062583	1171229	1189817	1385368	1271772	905897.9	632893.8	776617.8	2086475
225313.8	1399570	1371910	1720044	1717876	783356	1386615	825255.4	1109793	1237914
202978.3	1267291	1435013	1210665	985614.8	883064	1054831	784005.3	1147627	1119884

LP366.264	LP366.264	LP366.264	LP366.263	LP366.264	LP366.264	LP366.263	LP366.264	LP366.264	LP366.264
1119619	1797712	1574642	1183395	1047716	1105734	1069648	853531.7	1352483	1022552
638853.6	1645204	1160112	952058.9	739606.1	1393272	1117585	857943.7	1215701	1000750
588449.4	1425779	2766654	1634484	902723.2	1194157	1065798	800645.4	1494618	904097
700259.4	2183015	1302233	1218554	729787.4	1765125	1356470	759341.8	1145531	971906
840095.7	2311921	1299386	1198851	827397	1735743	929434.3	526648.7	1761547	970237.8

LP366.264	LP366.264	LP366.264	LP366.264	LP366.264	LP366.264	LP366.264	LP366.264	LP366.264	LP366.264
1312998	970340.3	680876.9	264048.9	925615.7	770664.5	736328.2	2216788	1106285	1408499
1059286	912101.3	768044.5	418679.6	1010566	895404.4	714118	1744003	1309231	1892642
1078535	1570538	606786.4	574917.5	772317.8	964764	1179278	2103585	1817023	1688659
1706996	1001631	743177.7	278058.2	1019450	906096.2	993826.2	1561277	1200199	1450275
1067849	1137892	982041.2	355453.8	1017286	789969.3	836650.4	1668079	1032640	1776264

LP366.264	LP366.264	LP366.264	LP366.264	LP366.264	LP366.264	LP366.264	LP366.264	LP366.264	LP366.264
2085769	1062788	1932835	1157334	274945.3	1004707	1289313	1515980	1295232	641305.3
2144350	1003854	1278421	1467207	272547.4	1200189	1328559	1299615	1113054	590820.1
2639672	1133295	1232930	981717.7	225478.6	1278457	1468284	1222412	945346.9	663790.9
1720324	925384.3	1682595	1324178	293065.5	993323.4	1433643	1634503	1247933	928199.5
1976201	1600130	1445059	2053999	220217.1	830145	1046924	1548021	1115357	836426.9

LP366.264	LP366.264	LP366.264	LP366.264	LP366.264	LP366.264	LP366.264	LP366.264	LP366.264	LP366.264
1633259	1611903	1013026	1696689	850365.2	659528.5	1949629	150293	1413827	1118129
1910442	1516175	1021048	1347129	600151.6	414745.8	1609660	208890.7	1534551	1239174
1440295	1276656	1602769	1261653	553966.8	641153.7	1768228	159202.9	1203475	1060810
1290179	1839224	1146020	1299537	781688.5	632214	1778565	207307.5	1276770	1631344
1718030	1228578	1443563	2051982	698271.6	568306.5	1895184	154805.6	1202778	1237297

LP366.264	LP366.264	LP366.264	LP366.264	LP366.264	LP366.264	LP366.264	LP366.264	LP366.264	LP366.264
321670.7	1114862	597644	146982.8	2005643	229668.2	983840.6	1109057	1068010	1306368
278734.4	1778928	699739.7	171085.2	1224636	268312.9	891145.1	982550.3	728764.1	1129020
198503.1	1265335	499864.9	152680.6	1420872	208279	1153868	1264045	791339.1	1242330
330176.7	1057098	840125.8	213765.6	1973886	324487.7	1036599	774880.2	875621.8	1511575
266829.3	1198442	701444.2	161690.5	1833517	387537.2	934950.4	906116.6	920429.7	1653360

LP366.264	LP366.264	LP366.264	LP366.264	LP366.264	LP366.264	LP366.264	LP366.264	LP366.264	LP366.264
925078.1	143865.9	644378.8	1125032	148389.3	1075595	1255957	639001.1	768845.3	968099.4
899564.3	85156.2	611047.8	998825.3	116294	1200395	1142459	902475.4	781268	947536.9
1177747	124647.3	821744.8	1311819	118109.6	1366429	1298451	959988.9	1145687	1016503
1053779	107128.4	627335.7	1048536	149526.5	1153522	772785.4	846102.3	1010677	1190201
1172216	79697.65	653147.9	931119.6	171412.3	1605626	1283092	963162.1	854624.6	1010151

LP366.264	LP366.264	LP366.264	LP366.264	LP366.264	LP366.264	LP366.298	LP366.306	LP366.373	LP366.373
816924.6	1202920	865250.9	188631	200311	409883.7	206078.2	561217.5	547832.4	125635.5
1038446	815794.7	759240.6	206234	219419.9	415670.6	184201.5	539986.6	356541.1	132608.6
853180.2	609253.6	833197.5	226300.7	159042.3	385229.5	175702.4	519347.8	365158.6	133925.2
997281.1	967903	1156725	255562.3	122099.4	446844.2	24295.46	539376.7	374064.1	117505.6
667192.6	702317.4	940597.2	350920.5	107372.9	426903.6	183669.3	557694.8	436260.3	117780.8

LP366.373	LP367.172	LP367.172	LP367.172	LP367.172	LP367.172	LP367.172	LP367.173	LP367.173	LP367.173
30193.97	42948.88	48235.04	41608.13	47990.17	81493.2	61266.82	54003.51	57320.45	48115.87
33415.24	41138.51	46411.15	39592.26	45905.19	74065.19	62979.24	59170.44	55724.82	42887.85
29967.53	46356.07	44456.49	39697.1	51739.46	78714.21	71853.05	77071.43	71632.84	44239.98
36723.29	46774.44	44128.51	44425.97	47092.95	75459.65	65734.82	58768.18	70646.58	42497.45
37320.77	42657.07	42974.57	39167.74	44611.92	72067.56	62800.47	41259.08	64570.84	34596.68

LP367.173	LP367.172	LP367.173	LP367.173	LP367.173	LP367.172	LP367.173	LP367.173	LP367.173	LP367.173
72971.44	78105.47	44292.94	73124.16	84073.88	72646.28	70300.19	88618.4	73927	72356.6
70731.98	69539.63	49023.5	71636.49	73146.63	65266.15	69919.7	84071.28	69698.46	75442.63
73530.2	118958.3	39280.84	47816.93	104943.5	79742.65	80611.08	96162.06	72808.74	98110.09
76056.56	112436.9	32396.75	69835.43	61543.87	85576.44	61717.75	80339.74	72461.76	69684.19
72475.05	67623.19	47237.65	55238.37	86797.62	67952.53	59902.93	85376.2	83415.31	66714.09

LP367.173	LP367.173	LP367.173	LP367.173	LP367.172	LP367.172	LP367.173	LP367.173	LP367.173	LP367.172
59768.2	76430.83	70394.29	37371.55	65479.81	61837.28	72684.06	61126.68	89332.9	94664.15
54179.22	79215.61	64509.31	39937.24	63382.97	59491.14	60624.31	56734.71	80744.47	90144.88
58751.83	61806.45	56315.02	55137.27	59350.12	95126.92	65343.16	82155.87	81991.64	93749.1
76109.07	58500.61	63809.33	36940.11	67420.06	65887.38	71640.78	48647.58	93699.35	75281.83
61160.29	79838.95	83800.25	32322.48	73406.57	69884.08	78090.03	56649.79	76590.27	66109.86

LP367.173	LP367.173	LP367.173	LP367.173	LP367.173	LP367.172	LP367.173	LP367.173	LP367.173	LP367.173
66322.63	73729.36	41983.76	72202.8	35465.89	72107.94	61226.26	47799.55	57630.36	87125.35
70648.33	79573.06	39342.69	61851.22	36367.83	68303.65	59954.48	49255.11	48460.69	75303.07
65346.37	88681.14	37645.11	61912.38	31510.5	68099.53	64451.48	49547.45	67388.69	84158.44
64265.22	121016	38723.93	48758.16	33865.02	64633.98	64292.54	51484.54	55839.03	78138.06
58713.2	57751.95	48622.31	53781.69	41927.47	58825.66	76377.39	40905.17	55331.51	64677.42

LP367.173	LP367.173	LP367.173	LP367.173	LP367.173	LP367.173	LP367.173	LP367.173	LP367.173	LP367.173
35481.61	62025.47	66608.36	56274.95	83362.97	77541.28	69595.93	68821.65	46743.23	77223.7
36259.8	66540.39	64734.41	59777.2	79893.55	73361.68	63557.53	69564.85	44837.16	82627.77
29971.72	72599.59	100776.1	84384.57	86528.18	71742.67	69281.35	97598.76	54538.4	94702.88
48927.62	58222.84	58725.73	62530.99	95283.8	73600.43	56667.36	69783.01	48266.74	100011.9
36593.69	63405.17	55207.9	61356.88	72451.1	71246.79	48981.26	64675.98	37906.79	69567.07

LP367.173	LP367.173	LP367.173	LP367.173	LP367.173	LP367.173	LP367.173	LP367.173	LP367.173	LP367.173
70955.63	65857.22	58498.57	72096.68	56350.92	71907.46	73091.07	87616.56	54426.82	66394.93
77736.96	66618.46	67830.9	67191.36	56630.85	69466.91	78750.42	82734.32	55990.08	61251.73
61278.97	63249.41	75343.05	88420.34	56920.91	93079.43	59129.24	57554.48	55659.41	57676.18
53885.56	74744.51	62442.03	86447.65	39895.61	58128.77	69164.78	78769.64	54486.57	45466.27
60358.98	100303.4	72357.9	52958.92	65859.47	85404.51	113048.9	70485.29	33783.17	72627.55

LP367.173	LP367.173	LP367.173	LP367.173	LP367.173	LP367.173	LP367.173	LP367.173	LP367.173	LP367.173
44332.49	77977.94	58312.69	50966.43	72456.38	36736.02	59204.7	78127.62	90466.32	71525.17
46893.91	79292.99	49478.39	52121.52	73878.12	35256.6	52163.9	70996.87	92533.38	69742.37
44453.86	82599.69	50414.36	62623.75	55351.25	30904.88	75749.21	84380.07	84865.71	91250.04
41091.24	62782.89	57904.44	71545.1	53966.33	39766.07	58656.77	60220.12	81355.82	76128.57
48688.76	103476.5	59803.14	43168.12	83002.81	40354.86	43418.62	95126.13	79723.5	57729.16

LP367.173	LP367.173	LP367.173	LP367.173	LP367.173	LP367.173	LP367.173	LP367.173	LP367.173	LP367.173
60437.31	58239.08	76233.68	72824.69	61189.72	61644.69	67068.74	70482.23	70848.29	43032.33
61113.05	62139.34	79264.05	67627.88	58589.48	64725.82	68005.21	70619.4	74875.99	43246.75
61749.5	54578.17	85807.47	55494.66	80417.92	69176.91	56541.69	76883.19	66599.86	66413.87
61696.1	66628.67	66847.48	90575.22	76806.72	62964.49	64854.62	56125.96	69708.7	40891.09
64958.3	62979.79	83306.34	71490.48	68078.49	45193.76	62824.17	82217.42	98991.42	48684.08

LP367.173	LP367.173	LP367.173	LP367.173	LP367.173	LP367.173	LP367.173	LP367.173	LP367.173	LP367.210
61488.72	44890.18	73259.18	70713.55	71329.04	70386.54	61792.5	73762.67	74371.45	79477.55
60840.4	44619.16	76103.08	60538.65	68580.89	74284.35	63953.01	71466.49	74361.93	58205.75
61437.12	39674.58	65300.83	94655.1	69171.7	68872.04	50671.26	65798.36	76675.76	65890.28
75922.36	40762.62	82482.59	61596.63	66691.59	69735.4	63073.3	67093.24	74718.58	57392.05
69385.18	43883.72	94482.9	65257.73	65306.2	86231.08	89616.98	75199.18	76348.94	54029.56

LP367.210	LP367.210	LP367.210	LP367.209	LP367.209	LP367.209	LP367.210	LP367.21_	LP367.209	LP367.209
78176.49	66060.71	67488.86	69379.76	45728.05	72550.33	28893.97	59150.83	74181.36	37960.08
52637.64	56779.85	79482.03	54591.41	88185.81	75210.21	49503.08	76520.72	74069.56	61170.41
54513.64	52518.68	70668.09	55743.86	83323.12	77896.45	46799.12	77442.19	87322.88	63428.43
76044.79	54335.33	79732.89	63796.63	79835.14	100958.4	49085.25	65691.56	71555.38	67788.63
48991.27	54655.96	82616.52	57397.29	79762.95	81074.99	46435.12	74976.4	71249.76	63747.46

LP367.210	LP367.209	LP367.209	LP367.209	LP367.21_	LP367.209	LP367.210	LP367.209	LP367.209	LP367.209
76565.18	66206.03	61472.32	76717.4	79948.4	43892.84	74046.53	59380.01	70193.2	44090.29
64963.62	71233.93	63805.62	59784.68	77740.24	44910.07	81916.97	73891.54	92159.3	62700.16
79125.3	70807.03	75854.91	59888.25	51259.56	47491.88	72506.91	69564.71	96807.61	69140.32
71427.44	73043.23	64300.44	59226.59	82321.55	46565.74	74457.49	75340.56	90182.95	67036.21
73720.77	73631.37	66908.45	58049.05	76438.52	42451.8	79573.28	66663.08	82501.7	64921.19

LP367.21_	LP367.209	LP367.209	LP367.209	LP367.209	LP367.210	LP367.210	LP367.209	LP367.209	LP367.209
50311.08	81498.84	67282.13	61547.51	49733.85	91321.13	35949.86	36492.98	76710.54	60729.32
72275.23	80971.79	72145.78	82114.62	80484.7	90626.28	38574.35	49351.11	80743.38	55209.95
67444.39	87946.89	72918.26	84216.24	80534.3	88742.7	44103.98	48555.77	82932.6	58268.47
77529.94	82526.06	68063.1	80246.11	80937.75	94330.37	40818.47	49069.58	75705.4	58330.51
67265.79	79387.49	67275.32	80934.78	79403.1	99340.45	43246.11	44455.17	87061.82	51203.44

LP367.209	LP367.210	LP367.209	LP367.209	LP367.210	LP367.21_	LP367.210	LP367.21_	LP367.21_	LP367.210
57888.58	58399.17	47171.47	56739.81	56581.28	53768.49	60490.38	56859.2	58555.11	41166.16
84292.25	127182.4	63581.11	61769.15	61679.5	62474.21	55492.29	68294.66	97252.88	52708.25
82752.61	123094.9	66548.36	43665.9	61519.05	63624.4	58287.12	65099.29	101467.6	57983.5
87742.03	124604.4	63040.31	60543.29	56241.72	56502.22	56329.23	61856.94	98138.42	52925.15
83993.85	121708.5	59922.74	62127.02	61553.11	58409.4	57148.88	55519.64	97043.76	50417

LP367.21_[illegible]	LP367.209[illegible]	LP367.209[illegible]	LP367.210[illegible]	LP367.209[illegible]	LP367.209[illegible]	LP367.209[illegible]	LP367.209[illegible]	LP367.21_[illegible]	LP367.210[illegible]
60098.26	99042.24	75370.25	74297.03	84401.71	56055.66	59194.43	54023.4	55733.33	120906.8
70606.5	79559.89	81730.76	70395.56	78019.66	57708.25	71476.89	63290.66	65890.88	119074.4
62795.52	87426.01	91396.69	72496.7	76359.33	56663.44	69509.97	67182.01	69228.27	118252.2
69441.21	84945.72	96112.48	67072.75	76915.23	58613.61	67018.1	65626.57	64811.36	125071.7
72073.5	90148.98	89485.53	69035.05	82391.6	52040.21	71822.75	62808.67	64908.01	114300.9

LP367.209	LP367.209	LP367.209	LP367.209	LP367.21_	LP367.209	LP367.209	LP367.209	LP367.210	LP367.209
63015.29	50193.57	27060.96	64301.29	32450.89	52760.73	40478.75	76979.45	82443.34	24286.11
71103.08	50703.05	28476.36	58525.38	34265.98	47002.26	44748.03	88661.67	74140.36	29337.43
75495.92	53438.1	32079.4	60606.75	35553.65	48299.28	50741.78	92699.54	78669.94	30483.61
74895.12	54737.59	32555.84	60652.3	38599.37	54457.07	51406.41	96081.13	77878.87	28054.34
70390.83	51390.21	31299.87	56759.02	33321.8	61656.08	55129.08	92878.03	79398.21	25024.47

LP367.21_[illegible]	LP367.209[illegible]	LP367.209[illegible]	LP367.209[illegible]	LP367.209[illegible]	LP367.21_[illegible]	LP367.209[illegible]	LP367.209[illegible]	LP367.245[illegible]	LP367.247[illegible]
79148.32	60521.56	58953.73	59236.87	47898.37	29396.99	65341.93	50716.99	80838.35	46917.36
89937.77	68078.37	58425.22	72724.2	54999.23	33131.61	74746.88	56526.73	93527.96	49900.25
91971.49	69977.69	71310.41	76318.8	56536.83	34915.16	81307.33	52852.28	93482.55	50497.66
83962.9	67390.33	67943.43	74569.48	56008.07	36462.01	76481.4	48892.99	88140.97	50805.06
94999.34	61649.95	59455.6	71590.43	49436.47	31541.42	73340.12	55140.05	85302.16	49883.61

LP367.247	LP367.247	LP367.247	LP367.247	LP367.247	LP367.247	LP367.247	LP367.247	LP367.247	LP367.247
72514.29	72573.04	58506.83	67697.74	72192.83	65763.93	56107.19	45320.83	95785.74	54576.43
64588.41	75674.95	62764.87	71295.75	75851.17	60916.65	56783.3	49094.28	95581.33	49394.5
62134.39	69452.23	60509.95	68196.4	73112.05	61732.46	56601.54	48345.34	95339.53	49743.16
63190.39	70153.64	61570.11	68584.58	71374.53	60949.36	53845.97	52439.21	96710.83	51518.64
60010.63	76086.87	63864.94	69997.16	73877.34	63385.01	54655.96	46258.16	93691.05	49819.74

LP367.248	LP367.247	LP367.267	LP367.267	LP367.267	LP367.267	LP367.267	LP367.267	LP367.267	LP367.267
55619.99	53975.98	259288.6	52886.19	43854.25	209736.9	263123.7	172844.1	266238.7	385182.9
62756.08	51458.65	275984.4	51266.9	61865.34	234423.3	251129.2	197019.1	411212.9	284937.8
58272.23	55353.56	285688.8	54835.08	53592.14	286780.1	369937.2	294347.1	248431.9	322348.5
54747.65	50308.65	275594.1	46952.56	54063.82	235361.8	260997.9	227084	399637.6	311161.5
55302.23	51117.9	207577.2	47221.12	44206.48	347644	200629.7	183984.5	347208.8	397820

LP367.267[illegible]	LP367.267[illegible]	LP367.267[illegible]	LP367.267[illegible]	LP367.267[illegible]	LP367.267[illegible]	LP367.267[illegible]	LP367.267[illegible]	LP367.267[illegible]	LP367.267[illegible]
338069.6	306290.4	243177.8	324657.8	238645.2	325264	387400.9	378518.1	279178	314270.5
251079.5	418945.3	222958	455139.9	273939.5	240471.6	296392.1	318878.5	413290.8	274363.3
329394.5	400401.6	171304.6	321955.9	418657.5	250330.5	245809.9	385289.8	376643.1	545828.9
267773.7	313056.9	242516.7	474481	354567.1	216745.8	280966.4	494761.9	261144.9	326320.9
254558.4	400386.2	218827.9	304970.8	283569.8	254237	222305.6	347138.7	401969.7	268427.8

LP367.267	LP367.267	LP367.267	LP367.267	LP367.267	LP367.267	LP367.267	LP367.267	LP367.267	LP367.267
342964.8	162090.2	249281.5	338637.6	341315.4	332505.7	468488	512471.8	263672.1	227417.4
332591.2	144838.1	486048.6	332741.7	230372.1	283029.6	460085.2	337030.4	274712.2	287308.6
411261.3	245100.6	277241.4	310367.8	355374	286169.7	517108.8	367581	336539.8	300310
478947.5	216482.7	322888.3	342384.1	396764.2	221456.3	301225.3	370979.1	307015.1	331199.6
405965.3	192681.5	447926	336103	288838.4	268553.7	303584.9	382510.8	305069.4	317701.9

LP367.267	LP367.267	LP367.267	LP367.267	LP367.267	LP367.267	LP367.267	LP367.267	LP367.267	LP367.267
274180.7	232251.2	312660.1	228621	265115.4	246017	204122.2	404465.7	414918.4	246733.5
237597	266130.3	261024.8	250913.6	291608.7	322857	186512.6	238272	262024.9	288517.5
186764.7	328429.2	220735.9	152038.7	280689.5	269950.5	175453.7	263282.8	247742.4	263586.6
394144.8	345059.3	492901.9	344438.9	257985.8	344269.7	205093.8	262432.2	376664.7	333833.5
220784.2	349943.9	373480.1	195668.3	263471.1	354019.7	223531.5	342599.7	338753.7	298039.4

LP367.267[illegible]	LP367.267[illegible]	LP367.267[illegible]	LP367.267[illegible]	LP367.267[illegible]	LP367.268[illegible]	LP367.267[illegible]	LP367.267[illegible]	LP367.267[illegible]	LP367.267[illegible]
208711.1	272815.4	193210.7	218691.1	287395.7	240784.7	222762.9	237646.5	246286	216658.8
186234.4	130525.9	181233.4	256178	274213.3	270216.2	194412.4	192934.9	260145.8	206089.7
154437.5	114523.2	269858.4	225747.7	233669.1	266318.6	192379	157865.4	204787.1	250934.7
149273.1	119235	256429.5	268370.9	266881.4	350403.1	191643.4	217221.3	211818.6	188271.8
275765.9	180335.8	159455.7	230808.8	262651.4	283238.6	203760.1	208342.9	280720.5	170482.9

LP367.2678	LP367.2678	LP367.2678	LP367.2678	LP367.2677	LP367.2678	LP367.2678	LP367.2678	LP367.2679	LP367.2679
398109.9	250918.9	147155.5	251050.2	234448.2	202247.6	256024.5	349693.3	243416.7	272194
268364.2	177148.1	191146.9	250384.3	186848.2	282074.8	261847.4	247035.8	258614.2	263941
347841.9	163213.4	215748.4	332615	131151	318157.7	227535.7	214633.3	244148	388785.4
341207.4	182992.5	200538.8	233918.9	138177	299061.9	268174.2	243619.7	284795.8	258946.3
331701.2	183172.7	202634.3	376109.6	157864.1	370891.2	287124.3	245026.1	310134.4	235737

LP367.267	LP367.267	LP367.267	LP367.267	LP367.267	LP367.267	LP367.267	LP367.267	LP367.267	LP367.267
171630	186397.5	323812.2	289783.2	222870.5	151538.9	193370.4	88616.95	150750.3	49051.93
146593.8	181590.3	323368.2	210051.9	177141.6	141988.2	185245.9	129191.7	200187.6	37392.57
120319.9	178553.4	211742.7	307932.2	209893.7	183163.4	176482	116782.1	186242.8	39302.72
169111.8	219037.2	253147.2	204518	196994.9	146599.4	184558.7	113266.9	202355.4	38862.99
142432.4	193472.2	277796	210640.8	194951.2	152128.3	186677.9	104609.1	206930.4	38531.34

LP367.267[illegible]	LP367.267[illegible]	LP367.267[illegible]	LP367.267[illegible]	LP367.267[illegible]	LP367.267[illegible]	LP367.267[illegible]	LP367.267[illegible]	LP367.267[illegible]	LP367.267[illegible]
73944.43	164756.9	40011.36	57309.24	25427.02	40886.77	64872.98	27763.78	43150.62	114903.8
73996.24	154385.3	56262.23	58017.66	27360.74	32602.54	70901.04	24479.3	48789.83	103798.1
72771.15	189531.5	69242.45	59065.38	32425.51	32940.69	58377.25	20810.15	36143.01	90205.9
74660.84	163714.8	60631.5	58944.35	28795.45	30658.09	75659.94	23550.8	39796.78	125932.3
64162.96	149840.1	56639.89	51432.9	25887.43	31885.99	71392.81	22062.29	33456.74	104193.7

LP367.267	LP367.267	LP367.267	LP367.268	LP367.268	LP367.268	LP367.282	LP367.283	LP367.321	LP367.321
27864.82	26725.23	64977.46	201962.3	31628.96	34658.12	247463.7	429842.9	336602.2	210986
31046.14	23867.29	60860.92	230610.5	37326.62	39780.75	239842.6	422630.9	299176.2	229929.9
27628.18	23522.24	40832.59	237125.5	36992.38	43591.69	222196.5	376933.4	318692.3	220342.9
30006.48	25629.72	67031	351622.2	34085.08	40898.65	231469.5	397249	338467.7	225270.2
26888.55	25841.86	62640.19	245184.9	33858.21	42319.77	236769.1	408095.4	317648.6	240764.7

LP367.332	LP367.357	LP367.357	LP367.357	LP367.357	LP367.357	LP367.357	LP367.358	LP368.191	LP368.191
308047.2	42951.84	40560.64	47559.9	55787.27	65643.38	90807.13	63681.71	45849.17	69510.82
300425.3	44214.84	42456.99	55562.5	62055.63	78589.57	86512.85	60995.43	45980.44	69272.11
315217.7	43617.13	42380.12	54448.53	59759.27	70468.46	90483.04	61150.3	44450.7	65020.55
331368.6	43951.6	47832.95	53495.66	51411.08	68632.62	88803.48	56982.36	46544.16	65396.72
331005.4	43647.42	45406.6	58539.74	51984.49	76744.1	88110.43	69703.46	28031.74	46046.93

LP368.191	LP368.191	LP368.191	LP368.191	LP368.228	LP368.227	LP368.228	LP368.249	LP368.270	LP368.270
81693.5	58598.87	61503.82	58003.72	141634.9	122793.7	117589	113881.4	35620.78	37488.34
79883.6	57780.54	65002.87	51073.34	159335.6	117550.3	151041.5	126097.3	32910.68	34072.68
80596.74	62783.92	56435.17	46954.69	155096.8	123151.5	138630.1	114973.3	26260.7	31148.06
83208.82	63471	67264.74	57224.8	157619.7	120349.3	139406.5	118557.7	26977.84	33465.69
50652.56	61699.49	65685.72	53028.33	156904.6	126702.6	153638.5	115276.6	30178.69	32880.24

LP368.270	LP368.269	LP368.292	LP368.352	LP368.352	LP368.352	LP368.389	LP368.389	LP368.389	LP368.389
29246.04	33941.94	56705.49	12809.13	10824.48	26933.87	56656.43	114142	634438.5	20602.16
23232.32	28838.77	12550.37	9249.034	6968.768	29939.15	61251.58	125073.3	723072.9	24469.29
31853.17	25547.63	23359.26	11109.98	13630.19	32150.22	62724.59	192346.3	1154370	21135.06
34379.92	31602.45	36485.66	11556.21	13381.47	27523.42	61281.66	241582.3	1438210	21300.95
31837.51	26694.7	31097.3	9452.85	14976.49	28727.88	67487.15	259959.2	1584184	19980.69

LP368.425	LP369.188	LP369.188	LP369.188	LP369.188	LP369.188	LP369.188	LP369.188	LP369.188	LP369.188
68651.27	129356.8	156262.5	108026.6	109457.9	131948.6	118401.2	149974.4	122163.3	123025.7
80081.42	220072.4	165398.3	117248.4	102204.7	126310.5	163009.7	156240.7	120865.6	135996.7
81279.02	180867.3	165187.4	115501.3	111244.8	115460.3	91322.9	171415	119417.1	126302.5
74464.33	156640.8	183485.9	110905.3	121938.5	108319.2	159257.7	145262.3	150948.1	111674.8
98539.08	114469.9	109499.3	128647.6	144522.4	114668.7	101826.9	166082.8	129000	124422

LP369.188	LP369.188	LP369.188	LP369.188	LP369.188	LP369.189	LP369.188	LP369.188	LP369.188	LP369.188
215349	122742.6	143780.6	138081.8	133951.6	123616.6	157482.1	161787.7	109463.9	151961.5
126817.5	121410.1	143707.7	127095.1	121176.6	137854.1	162238.9	112289.7	119794.7	163217.8
138849.3	171447.4	171339.9	106356.5	163393.2	134672.2	184655.5	113199.4	134295.8	185405.5
135504.7	148435.9	134050.8	147236.8	194937.9	141738.2	149365.7	223849.3	108885.8	134635.3
116092.6	121697	164060.3	196428.9	155615.7	149071.7	133265.6	143900.6	111646.7	119440.5

LP369.188	LP369.188	LP369.188	LP369.188	LP369.188	LP369.189	LP369.188	LP369.188	LP369.188	LP369.188
119407.5	190377.1	102554.5	215537.8	174383.2	155934.9	107946.7	157272.4	223179.1	200529.3
102212.2	140562.4	142538.7	159502.5	179729.9	199723.5	110320.1	156417.9	142006.6	160318
109503.7	151212.7	107154.7	108895.8	112835.5	141502.7	151690.4	186360	134087.2	198800.4
128054.9	131094.5	104178.2	129118.6	119168.7	199980.7	137474.2	201023.7	232216	167882.2
147895.4	157205.1	84289.92	126685.3	141179.6	135011	129514.4	161788.2	130500.1	213023.6

LP369.188	LP369.189	LP369.188	LP369.188	LP369.188	LP369.188	LP369.188	LP369.188	LP369.189	LP369.188
141297.2	112214.3	125523.6	110559.9	122080.2	117540.1	145071.6	195338.5	131707.3	197432.9
141660.5	152791.2	108519.8	109104.5	170085.4	101516.4	108381	114700.2	115787.5	176169.6
182939.1	189062.6	105828.1	166319.7	141445.5	113391.3	160398.8	132966.4	139304	174620.2
109916.1	130241.7	123401.1	142853.9	151468.2	141601.9	172853.4	149715.9	128134.1	161951.1
163241.7	178573.6	149159.4	89526.95	123947.5	167224.1	152121.1	122225.2	128718.9	169788

LP369.188	LP369.188	LP369.188	LP369.189	LP369.188	LP369.188	LP369.188	LP369.188	LP369.189	LP369.188
137641.6	108628.6	98989.97	178685.7	142258.9	161483.6	157500.9	122548.6	172250	106506.6
112019.4	165806.1	85940.62	169449	142176.2	125657.1	172791.8	124144.7	209051.4	100928.5
132321.2	262986.4	99833.42	225997	158212	109634.2	109905.2	120022.3	169487.2	97558.39
104309.1	113211.4	96511.66	135176.3	131191.7	106530.1	150214.2	171260.7	154288.2	93774.36
109289.6	139312.2	96200.72	181583.1	156087.5	125808.4	167659.3	112205.3	173794.5	108163.8

LP369.188	LP369.189	LP369.189	LP369.188	LP369.188	LP369.188	LP369.188	LP369.188	LP369.188	LP369.188
99355.89	185414.3	138116.8	147389.4	80362.56	149159.6	121718.7	162537.4	86313.62	130817.6
109203.9	105344.9	164993.2	145973.8	106148.1	151232.2	168244.4	114430.5	97464.1	85221.72
95069.64	165477.1	145459	163716.4	77729.95	158797.8	146490	122298.5	90657.82	151227.2
89589.59	137269.4	149359.5	149127.3	78055.01	174040.4	133611	115579.3	91833.12	139745.7
90989.97	135579.3	145865	151301.2	79128.68	141083.7	134693.3	130591.7	79874.52	125752.6

LP369.188	LP369.188	LP369.188	LP369.189	LP369.189	LP369.188	LP369.188	LP369.188	LP369.188	LP369.189
149673.3	131752	142065	143511.1	139153.2	137321.8	130346.4	61351.84	107860.7	168714.5
154838.5	168153.1	129841.2	194008.9	187449.8	110579.3	91947.39	54412.66	149626.2	178087.8
152708	176890.1	137692	130027.5	145220.4	122974.1	165808.8	63906.84	107183.9	143463.5
120081.7	160065.4	121801	124613.1	163711.5	149815.9	129548.1	73773.68	100836	179684.7
160671	112618.1	201306.9	149654.8	158481.4	125658	113656.5	65088.23	120441.7	131662.1

LP369.188	LP369.188	LP369.188	LP369.188	LP369.188	LP369.188	LP369.188	LP369.188	LP369.188	LP369.188
88815.83	105549.3	102354.3	97265.62	94890.43	64055.78	96461.68	148441.7	74697.66	74778.89
74672.17	93412.92	95757.45	93394.49	68737.3	50514.2	132530.5	93941.02	59309.16	64497.12
68821.17	91187.74	122481	89128.12	110087.2	60361.04	90753.96	105125.1	78535.44	58661.26
59125.11	101891.9	154534.1	127088.1	98249.65	64310.83	83302.12	114772.5	91738.67	87928.77
70031.18	80667.09	110363.4	86051.95	91312.69	62355.6	87901.2	162123.3	76789.5	64700.49

LP369.188	LP369.188	LP369.188	LP369.199	LP369.226	LP369.240	LP369.263	LP369.263	LP369.300	LP369.300
93068.03	56009.57	84886.84	145369.8	86865.87	225067.7	33509.17	42745.54	49947.16	32362.82
89994.85	59326.42	82519.44	225596.9	122204.9	110300.6	32236.71	40547.32	49367.32	30631.22
87657.66	59252.18	116640.7	162976.7	121745.5	123289.1	30279.13	41171.55	50448.74	28583.04
94841.9	68628.61	62764.9	242167.6	123468.9	262600.6	30232.38	42317.58	53202.19	32928
70657.11	51029.75	74684.51	177141.2	123763.1	265331.8	34697.3	35868.79	40242.67	26930.17

LP369.300	LP369.300	LP369.336	LP369.336	LP369.336	LP369.336	LP369.336	LP369.351	LP369.351	LP369.351
36898.22	27623.25	24458.25	38618.88	55755.48	28430.97	27244.76	68336.15	78855.97	89212.07
33500.94	28028.43	22591.07	39478.11	49727.89	29452.3	31583.76	70742.55	75601.94	91865.12
38331.04	25030.54	19535.05	41392.77	52771.74	31075.69	33340.99	68455.77	120155.7	88053.97
31359.51	26994.05	27472.9	40023.96	51073.68	30094.39	30603.13	75864.9	69113.49	99754.34
35455.02	25021.42	22869.25	39102.92	54821.13	28858.48	31937.59	70674.18	76078.22	83460.33

LP369.351	LP369.383	LP370.203	LP370.355	LP370.368	LP370.395	LP370.404	LP370.404	LP371.101	LP371.101
62901.64	5969.262	29420.78	29103.35	148109.4	23941.64	177349.6	29841.93	142891.9	46682.96
59009.85	6337.731	40098.86	30407.61	131537.5	21116.28	189287.4	28183.93	205282.5	55738.77
55068.74	4784.886	28445.54	22239.17	140795.4	20994.22	246569.6	28594.59	222255.3	68201.62
71254.32	7650.905	31908.85	27673.91	191060.6	23484.06	290508.1	30838.58	379372.1	99894.67
68607.86	4004.424	29891.75	28574.02	156310.6	23102.07	306058.4	30186.1	325187.4	98550.59

LP371.101	LP371.101	LP371.101	LP371.167	LP371.167	LP371.168	LP371.168	LP371.168	LP371.168	LP371.168
54262.25	20378.2	32494.81	98387.29	48811.06	107974.3	74351.03	82737.87	66204.5	62982.14
72415.63	21201.42	34431.62	109534.7	50734.66	62320.32	75003.89	81894.5	82435.75	66518.38
69837.56	25752.22	36379.7	91225.55	44405.75	85888.38	81502.61	104762.6	65346	55606.44
123686.1	30136.93	37530.72	80539.11	46498.77	67099.08	81757.18	85589.22	70508.44	56355.23
103041.4	30540.8	40660.59	81459.52	47101.35	73342.78	76329.43	81931.19	73377.48	55775.01

LP371.168	LP371.168	LP371.168	LP371.168	LP371.168	LP371.168	LP371.168	LP371.168	LP371.168	LP371.168
79818.1	110582.9	80567.7	86394.85	74405.14	113965.4	102319.9	82257.66	117534.8	110574.7
122638.6	86144.06	55307.4	114193.7	63163.73	86594.45	58889.77	58655.73	97780.88	125547.3
76287.53	82603.96	78131.8	81510.71	84528.61	79761.84	88880.06	58877.12	81645.68	119327.7
89760.42	73489.55	55530.07	90521.74	81796.71	98806.16	67870.39	63221.68	80868.41	88881.49
85262.77	87136.56	58785.1	99228.46	85636.31	88804.91	73880.67	64132.6	83880.21	100965

LP371.168	LP371.168	LP371.168	LP371.168	LP371.168	LP371.168	LP371.167	LP371.168	LP371.168	LP371.168
93997.65	57258.77	38396.94	69143.04	79178.78	80041.76	54049.16	49738.78	73202	82449.22
60876.77	56058.96	45375.8	88680.53	65638.56	86675.62	92947.08	57560.78	48061.15	74824.91
140145.8	53325.35	43916.75	57975.14	91841.79	67834.86	90224.13	57112.38	53147.44	68335.79
94055	64015.47	52896.03	55693.5	92908.12	88746.16	80966.5	49164.01	64158.17	84671.37
91430.43	63621.82	54623.28	64478.55	86168.16	81982.1	71787.57	50451.41	62960.21	80345.79

LP371.168	LP371.168	LP371.168	LP371.168	LP371.168	LP371.168	LP371.168	LP371.168	LP371.168	LP371.168
79105.34	72539.64	96828.34	67598.9	82970.2	19812.75	75945.97	149422.6	107531.1	33466.67
54062.48	121567.6	63132.35	79287.27	93493.77	25782.29	131377.5	82634.12	133810.3	27754.16
51543.92	95840.44	62353.95	64876.11	74701.47	25726	83374.55	72845.83	121753	36833.93
54264.65	86167.26	62005.5	61913.46	86704.11	24217.51	87604.51	90556.89	81440.32	28244.95
57854.64	75566.48	60514.75	62027.41	91058.88	24583.63	84171.93	88652.59	81908.17	28927.52

LP371.168	LP371.168	LP371.168	LP371.168	LP371.168	LP371.163	LP371.168	LP371.168	LP371.168	LP371.168
70873.8	107325.5	43398.51	102169.5	51062.22	22009.87	77797.34	61628.51	98606.3	82663.71
128614.9	92634.02	56087.54	112742.7	70011.25	26252.99	51393.79	42450.63	125272.2	99402.16
77017.71	102991.5	62843.35	117907.3	59891.13	27222.3	89407.46	65999.59	114598.4	86601.1
90617.53	81320.81	39933.61	95404.76	62661.73	28337.35	64098.96	64085.69	98498.51	92463.28
94822.87	86493.3	40263.26	93001.97	63333.44	29827.75	66486.07	61004.95	102079.8	88539.58

LP371.168	LP371.168	LP371.168	LP371.168	LP371.168	LP371.167	LP371.168	LP371.168	LP371.168	LP371.168
95235.84	63574.06	37888.99	66165.58	37920.81	103746.8	84410.39	82494.62	99804.2	104345.6
89685.54	109183.4	48645.81	68537.88	28071.76	61821.49	90622.84	62187.32	87997.56	81917.96
94132.1	84260.24	38856.5	85048.96	30764.06	92071.95	65751.36	81812.23	94766.19	77627.24
96848.97	69608.93	27453.83	57576.02	31767.85	66351.19	72632.93	58297.63	87910.25	77269.7
92465.06	63652.85	31912.72	59371.24	32518.6	76480.59	75031.9	58352.9	99841.57	75035.71

LP371.168	LP371.168	LP371.168	LP371.168	LP371.168	LP371.168	LP371.168	LP371.168	LP371.168	LP371.168
28383.27	42317.35	67175.5	123659.9	44779.48	42488.81	40529.13	59125.98	89793.31	24088.53
39313.31	38733.1	84530.5	61246.47	32654.6	32438.32	40435.07	66269.73	76945.32	44065.02
24134.19	34716.05	84007.13	105075.1	23581.43	27797.25	22507.59	76656.55	88700.15	33674.73
35074.05	39505.36	55501.1	97388.59	26218.57	28751.31	24727.48	59218.04	78064.67	36732.79
42068.38	39961.46	59377.35	102073	29919.78	32923.02	24509.77	61369.51	74531.7	36954.52

LP371.168	LP371.168	LP371.168	LP371.168	LP371.168	LP371.168	LP371.168	LP371.168	LP371.168	LP371.168
88773.16	85183.88	61096	95093.28	66100.31	80427.03	61107.54	81879.64	51529.35	65152.27
81653.15	72611.76	59310.57	103752.3	57609.47	73975.69	50834.78	67565.89	50234.31	71559.87
97414.04	72758.91	47609.67	88532.92	74872.33	77002.79	114698.7	160112.8	63532.14	74184.22
93087.84	77343.17	59579.51	94782.06	64798.07	86693.57	47796.4	73140.61	55395.98	75073.18
93283.94	74978.69	55373.4	95662.94	65175.65	90216.26	50325.76	78419.5	61903.66	76678.66

LP371.168	LP371.168	LP371.168	LP371.168	LP371.168	LP371.168	LP371.168	LP371.168	LP371.205	LP371.205
78370.81	30251.98	34247.01	94392.38	95275.62	35953.28	23716.72	58696.36	104644.9	99711.91
60732.57	34572.81	36361.14	79082.5	61587.9	21032.83	33057.23	61320.16	92531.46	106861.4
80751.71	32964.96	23506.91	103924.6	98423.72	21917.53	28002.26	64463.48	97429.97	104816
62924.07	30668.57	29309.9	76224.44	77500.88	20623.43	28536.53	60937.35	93705.3	98717.38
64011.84	31632.55	32651.8	84889.42	83388.59	22177.31	31093.38	61997.04	93182.32	98955.11

LP371.205	LP371.204	LP371.204	LP371.205	LP371.205	LP371.205	LP371.204	LP371.205	LP371.204	LP371.205
142714	65798.39	88380.72	86102.68	79100.98	83084.26	88059.44	43809.64	68169.56	83267.04
157608.3	68048.26	87132.06	83049.48	86219.61	75516.57	94883.68	42057.1	67531.14	78667.66
153573.7	62799.41	86458.56	79815.74	85964.42	75076.09	85668.13	38829.11	66746.4	74464.94
147047.7	61089.01	83645.58	83808.86	79357.06	77654.36	85458.02	36520.66	63341.14	76510.67
144824.3	64619.9	93168.36	71650.41	83630.33	77201.08	86356.68	41068.77	65428.87	84308.54

LP371.204	LP371.204	LP371.205	LP371.205	LP371.205	LP371.205	LP371.204	LP371.204	LP371.205	LP371.205
64169.25	67296.76	59855.57	23748.51	58182.26	120309	89070.66	68002.92	113686	58534.82
69334.01	63332.31	61098.37	21181.67	60628.76	120226.1	78891.25	77159.74	119005.7	58070.75
64293.19	67559.24	60907.92	23525.15	64435.84	116929.2	91063.18	76248.07	109712.4	53685.95
69895.96	58856.34	57228.19	21158.14	58325.72	127753.9	82792.37	75034.19	109076.9	52951.69
60828.75	67133.41	56949.61	21713.24	54816.51	127680.4	85353.8	69952.23	113824.6	49817.96

LP371.205	LP371.205	LP371.204	LP371.205	LP371.204	LP371.204	LP371.204	LP371.204	LP371.205	LP371.204
112298.4	95792.43	65014.72	77938.33	58719.21	70303.65	54570.47	52840.4	29020	75584.67
108755.3	88619.14	56417.76	78001.27	58117.4	66813.08	64350.52	47984.25	28042.78	78608.99
111988.1	89683.92	55148.52	77130.98	69801.09	68815.84	53416.09	54358.01	24733.61	75994.97
106293.7	95896.25	50054.66	87739.59	63720.45	69330.16	56017.5	53633.21	28653.06	75892.52
116678.5	92187.74	61720.76	81185.66	66046.54	63873.52	56891.76	54356.65	25504.19	81150.37

LP371.205	LP371.205	LP371.204	LP371.205	LP371.205	LP371.204	LP371.204	LP371.204	LP371.204	LP371.204
20893.18	103287.8	62453.73	68372.88	48549.69	51496.99	62325.88	76363.41	57639.84	61573
24076.7	98555.54	65216.88	67962.37	55396.94	49847.57	58326.64	75358.71	56990.21	57041.86
21063.45	94547.3	66411.5	67513.35	50600.44	48222.32	54408.3	75969.67	53848.22	55310.63
20505.15	98627.23	68127.26	76682.22	51303.67	47814.48	58108.12	74043.34	50824.57	55335.93
25335.69	96843.57	67355.38	78417.36	52086.04	50332.13	60103.08	73126.24	53252.87	59733.68

LP371.204	LP371.204	LP371.204	LP371.204	LP371.205	LP371.205	LP371.205	LP371.204	LP371.205	LP371.204
68270.27	62110.79	56870.8	105136.6	54811.62	37947.81	27973.44	65424.37	76339.98	66721.33
72066.48	64426.78	60603.06	100198.7	52741.26	38971.37	25310.43	67719.37	81881.22	72312.89
70227.91	69233.31	63486.56	101796	54359.12	32808.68	28633.52	62913.56	82512.65	64504.15
70164.14	59029.91	62290.69	97609.48	50910.07	31902.52	21930.62	64930.19	75490.64	66284.24
64356.03	62895.4	57146.54	105612.4	55617.57	33353.16	27597.57	57246.57	76754.55	71735

LP371.205	LP371.204	LP371.205	LP371.205	LP371.205	LP371.204	LP371.204	LP371.204	LP371.204	LP371.204
37529.7	64254.75	29011.91	19788.97	30475.46	73446.79	80940.64	127611.1	62410.07	63222.42
40466.94	57243.73	28587.55	17254.24	32688.23	73034.57	91521.06	127225.6	69488.37	74651.59
43566.6	61997.48	26257.91	15839.76	25915.6	79373.6	90360.8	116790.6	66852.19	69869.65
34613.39	58103.91	26142.9	14514.32	28971	84145.26	94402.35	117116.7	58867.53	68379.76
40239.13	55261.5	28137.56	17190.7	29614.55	76149.38	90858.18	116512.2	68042.05	66981.39

LP371.204	LP371.204	LP371.204	LP371.204	LP371.204	LP371.204	LP371.204	LP371.205	LP371.205	LP371.204
55727.45	63251.43	69051.79	71752.94	120707	54702.82	93003.28	33263.01	71869.42	48788.01
53819.86	62432.68	67318.36	80626.75	120387.7	46637.33	95982.08	33256.86	68629.31	50829.47
56147.25	65607.25	63561.71	70036.77	119697.9	51850.07	103067.5	32476.92	64102.33	51632.03
54445.74	61743.77	69787.09	76405.51	116524.9	46055.02	96055.86	27696.9	61155.27	50959.61
58419.65	67137.69	70495.66	75411.27	124251.9	51564.15	94546.73	30729.02	67582.97	48488.12

LP371.205	LP371.204	LP371.204	LP371.205	LP371.204	LP371.204	LP371.205	LP371.204	LP371.204	LP371.204
115137.5	52296.81	95012.7	19414.7	74123.25	132032.2	70599.48	70434.91	69165.39	82126.97
116843.2	47868.97	98531.57	21431.82	78364.87	129467.2	73607.02	67862.21	68207.92	86109.62
103219.1	48960.41	99176.64	18832.06	72149.24	126697.4	64134.38	69191.15	69049.96	79002
101342.6	44889	105407.2	17768.63	69483.56	128562.4	68541.39	69795.83	69397.92	77967.6
107529.3	48882.85	99719.02	19249.78	72593.26	134674.7	70379	70166.21	68813.7	84529.84

LP371.222	LP371.315	LP371.315	LP371.315	LP371.315	LP371.315	LP371.315	LP371.315	LP371.315	LP371.315
56244.54	1815601	724843.5	444753.5	637424.7	668213.1	172973.9	175902.9	117232.6	165502.4
57032.26	1037678	627594.2	482660.5	903848.3	687858.2	121253.7	181554.4	120281.6	168452
49181.82	1174772	572749.2	487550.1	621546.8	1150229	101736	217368.4	109751.8	154384.3
28533.43	1161817	697413	341611.4	629755.8	631707.3	139968.5	207832.3	101578.2	155583.7
59717.54	891628.3	806390.5	331505.2	687555.3	625363.6	188690.7	198077.6	116246.5	155061.2

LP371.315[illegible]	LP371.315[illegible]	LP371.315[illegible]	LP371.315[illegible]	LP371.315[illegible]	LP371.315	LP371.315[illegible]	LP371.315[illegible]	LP371.316	LP371.316
140697.2	154972.4	120157.7	212328.5	204296.5	136719.7	140181.5	170807.8	152179.6	202508.2
151939.7	154821	127052.2	219420.6	177613.7	133344.4	126537.3	159284.1	173309.4	229683
229030.1	134854.6	115763.8	236454.9	165102	140241.7	133719	157831.5	156070.3	227665.3
134319.6	125409	122583	232351.2	159734.8	138559.4	116382.8	158698.9	155986.4	228253.5
131258.2	120336.1	118197.8	232952.7	161137.6	177753.6	135704.1	177571.7	174298.8	234057.4

LP371.316[illegible]	LP371.315[illegible]	LP371.315[illegible]	LP371.316	LP371.315[illegible]	LP371.316	LP371.316[illegible]	LP371.316	LP371.316	LP371.316[illegible]
173985.5	192617.2	227743.3	128449	136637.7	135184.7	149108.4	126777.6	136573.6	96502.79
173964.4	253404.7	224514.6	131798.1	114471.5	148842.5	174917.8	131270.6	141895.8	89607.66
173063	180261.2	211806.2	124631.5	141425.5	134735.4	170618	136919.6	128017.5	92143.37
177468.7	193047.9	226789	145765.1	123388.6	127617.3	155174.4	134462.1	142851.2	101334.1
185580.1	199228.8	218636.8	140692.8	137242.3	153453.3	165050.1	131815.1	145729.8	93553.14

LP371.316	LP371.316	LP371.371	LP371.400	LP371.399	LP372.101	LP372.101	LP372.257	LP372.257	LP372.257
138978	149307.1	30154.15	10855.94	25171.14	40265.33	22479.69	35087.3	44605.65	92090.26
155745.9	162058	27223.07	10998.53	23244.66	36328.95	21964.61	33596.19	48752.49	87113.54
152667.3	161258.5	23935.31	17730.91	18597.5	35242.24	19597.46	36539.25	55970.31	90411.01
151525.4	168408.7	33182.59	20539.54	24080.34	33560.56	20018.65	33674.05	50099.25	90227.75
164380.7	167071.9	24293.56	22801.82	19447.26	41499.44	20455.81	35022.58	45043.26	85366.42

LP372.257	LP372.257	LP372.257	LP372.257	LP372.274	LP372.274	LP372.274	LP372.274	LP372.274	LP372.274
52011.48	54611.66	42678.88	33898.83	37238.19	40470.11	71095.23	50511.48	58248.04	60156.27
49215.83	46596.36	46858.25	35520.1	59933.58	61756	68797.85	63470.91	63115.41	54969.3
46221.08	52097.64	44082.41	33968.38	48989.93	53252.14	71519.29	61047.81	67115.9	64071.55
49493.23	50400.21	48458.94	31396.47	63830.13	52311.21	66551.58	61456.48	62737.83	62748.59
45767.94	47450.01	43949.42	34772.26	56796.77	54934.51	74489.89	67993.84	62057.15	57711.88

LP372.274	LP372.274	LP372.274	LP372.274	LP372.274	LP372.274	LP372.274	LP372.274	LP372.274	LP372.274
42741.04	49547.99	48688	55257.24	53283.17	77607.2	49597.57	31505.86	42660.13	62746.77
56169.61	52660.4	42635.19	45010.65	49464.04	56535.25	49615	33678.49	49151.96	50369.86
53869	54557.29	41035.61	51753.13	46345.18	61652.57	47613.01	30774.87	37440.54	58685.55
54714.07	48822.55	43536.7	49363.56	67819.87	55360.8	45864.13	33924.72	44753.98	54914.27
49429.31	51365.03	44629.16	43541.26	45767.94	57595.68	43723.58	31576.49	45185.68	57078.6

LP372.274	LP372.274	LP372.274	LP372.274	LP372.274	LP372.274	LP372.274	LP372.274	LP372.274	LP372.274
45299.17	45883.13	38394.98	45739.37	52188.19	53228.56	47549.13	48365	59297.2	47920.98
46728.06	41530.41	42184.06	53059.67	48214.87	45654.06	43133.68	56763.44	40414.21	137826.7
44735.59	39987.6	43017.31	53037.38	48381.37	45808.15	37989.87	51986.26	44441.15	142393.4
42167.43	42602.47	42024.91	44599.43	51967.17	42986.45	39752.56	49464.51	42466.81	141651.8
43300.52	43439.68	37192.23	49079.21	48010.98	44288.5	38755.52	49980.21	40029.2	151326.5

LP372.274[illegible]	LP372.274[illegible]	LP372.274[illegible]	LP372.274[illegible]	LP372.274[illegible]	LP372.274[illegible]	LP372.274[illegible]	LP372.274[illegible]	LP372.274[illegible]	LP372.274[illegible]
43199.38	38556.32	59830.84	43171.11	38053.79	34820.89	42876.97	82822.86	56768.75	55315.67
50944.52	43229.33	50736.62	50194.52	37335.46	33968.4	43429.12	62888.52	113993	42740.35
51991.83	46570.93	55523.46	47919.11	39150.38	34213.84	49366.08	64786.5	98570.32	42030.16
48971.02	51487.76	49600.68	45148.05	31442.22	39881.62	48950.07	64097.87	103917.4	43780.02
49938.59	49420.99	53626.43	47741.19	36449.75	35949.99	43560.66	69493.37	94105.94	45369.77

LP372.274	LP372.274	LP372.274	LP372.274	LP372.274	LP372.274	LP372.274	LP372.274	LP372.274	LP372.274
46921.24	36344.08	57319.03	49534.52	72409.92	41801.23	38538.06	44334.96	35067.65	39511.09
45903.39	30873.42	52232.38	38719.5	57915.54	40258.23	33425.57	41991.19	31556.01	39941.32
44015.23	30484.77	54099.92	43228.02	61663.66	42417.45	34900.11	43440.88	30893.63	38040.15
46035.98	30321.52	51891.14	36893.61	59815.09	42294.33	36541.8	44285.14	35257.28	38730.99
47940.89	29733.85	51210.96	42177.84	59753.32	43316.13	36075.53	43755.4	37110.71	39014.04

LP372.274	LP372.274	LP372.274	LP372.274	LP372.274	LP372.274	LP372.274	LP372.274	LP372.274	LP372.274
37713.67	42334	40597.78	32949.42	30017.39	48620.28	46691.85	41140.23	31597.1	31323.85
32659.61	36461.39	41048.42	36496.56	30702.4	48961.44	44639.36	43042.38	31302.22	28376.52
30440.84	43040.89	40203.37	34507.14	29757.53	47299.29	38071.88	37833.44	27546.74	30564.17
31601.35	38175.19	39004.81	38888.87	29242.69	40614.73	42253.05	43911.92	29725.59	33337.03
30942.64	34328.06	36441.06	32348.49	25869.74	40917.67	37965.87	37539.51	29478.63	27162.23

LP372.274	LP372.274	LP372.274	LP372.274	LP372.274	LP372.274	LP372.274	LP372.274	LP372.274	LP372.274
49071.04	35254.83	32406.9	119181.9	32608.63	39997.12	45672.76	47663.46	49453	30084.84
79933.32	35169.16	30477.96	117201.2	31395.74	44114.28	36244.5	106925.7	116917.3	29000.58
79973.72	30154.58	34269.18	116025.7	34025.63	45952.54	42260.65	98142.51	124429.2	28526.3
78803.96	36122.08	32586.13	112350.8	38110.93	40842	36296.78	92989.25	110869	35922.91
76974.81	30404.47	29593.66	119176.7	31927.22	41965.16	42149.77	85861.51	105806.6	30171.65

LP372.274	LP372.274	LP372.274	LP372.274	LP372.274	LP372.274	LP372.274	LP372.274	LP372.274	LP372.274
54591.79	46949.74	39078.54	46657.92	38812.6	42649.62	51188.49	31271.02	30955.69	63579.61
50657.04	43735.15	38733.41	43228.81	40538.87	41016.41	41468.41	28632.41	28484.15	80630.68
55228.17	52012.11	39000.44	48494.04	35589.36	40981.16	40649.6	28029.13	35427.67	73259.34
55278.82	47115.97	42334.3	42845.87	39447.85	40056.71	42093.47	32397.17	32393.36	75543.44
55439.63	43936.72	38528.24	44774.72	48710.25	38438.93	45980.74	25316.4	29738.08	72346.46

LP372.274	LP372.274	LP372.274	LP372.274	LP372.310	LP372.319	LP372.347	LP372.347	LP372.882	LP372.882
44396.27	42891.33	33536.22	39580.14	316714.7	251474.4	137212.4	230912.4	29354.04	23327.52
43346.77	143739	36818.12	36393.1	321794.4	250233.7	114896.7	326258.6	24122.89	21113.22
41163.65	132143.5	36563.07	36846.99	296189.1	255559.5	116742.4	279746.7	35104.96	27309.06
42976.46	130702.6	36465.47	36616.72	297695.2	257462.2	120623	283066.3	31491.82	24850.47
42032.71	148326.3	31292.94	33029.91	289760.7	240902.2	129080.6	217718.1	20604.62	22144.98

LP372.882	LP372.882	LP372.882	LP372.882	LP372.882	LP373.098	LP373.098	LP373.098	LP373.098	LP373.183
26893.09	27399.51	19449.44	22442.25	24336.25	38534.05	25672.69	15588.75	17670.54	69335.24
25307.18	24917.56	21364.42	17698.51	22781.59	34766.32	27983.11	15253.09	13393.45	60342.27
29912.44	36260.8	23133.63	27865.56	32932.63	49692.13	23973.41	14648.28	14012.79	68797.03
32997.72	36409.41	23888.71	28035.96	32759.72	65290.66	25648.8	10693.77	8611.867	64915.41
22960.2	26865.46	18441.73	18228.03	21458.24	55539.5	25932.04	18675.93	16393.28	60165.48

LP373.184	LP373.183	LP373.183	LP373.183	LP373.204	LP373.295	LP373.314	LP373.331	LP373.350	LP373.350
129467.5	76883.82	63037.51	50491.56	32887.68	14737.58	30765.03	21674.8	50814.86	24661.13
126576.9	70546.72	65634.95	52514.18	35264.43	8248.368	32472.69	23881.03	65033.86	22346.63
137466.2	72240.87	51859.92	60695.15	30379.94	12034.46	32888.2	22879.35	51920.84	24753.82
130608.7	64627.11	68990.6	49833.8	28436.17	8228.586	25388.28	20193.01	50020.96	16175.93
135745.5	68557.2	50111.42	43511.73	34153	10774.52	25649.14	19024.83	46979.07	18639.1

LP374.098	LP374.217	LP374.217	LP374.253	LP374.254	LP374.254	LP374.253	LP374.254	LP374.254	LP374.253
29027.98	34283.46	49571.31	88805	75440.14	96675.52	83603.53	98292.16	71526.24	93087.32
29242.54	36434.2	57822.68	86155.86	79378.12	117355.8	68782.03	97353.35	110172.7	87346.56
33331.84	33647.21	57027.19	118992.9	154875.4	86876.55	63723.05	128709	71057.48	82842.68
34107.22	33803.36	60196.31	66204.6	83555.6	92325.6	70156.58	97633.64	77869.19	68276.38
33460.06	34155.56	59214.88	92235.11	82295.36	74801.91	73221.28	90213.57	63392.56	83747.16

LP374.254	LP374.254	LP374.253	LP374.253	LP374.253	LP374.253	LP374.254	LP374.254	LP374.254	LP374.254
85095.58	82741.43	71535.2	70433.69	78827.26	76563.5	99400.57	104081.5	83055.96	81086.16
77822.35	86170.65	66332.62	149982.3	82324.81	73866.48	101493.6	91711.1	104799.2	76569.89
73874.66	100180.3	67094.1	95020.62	84261.81	56441.08	83390.35	102518.7	87692.44	81747.44
71949.62	83695.55	61662.29	94921.16	76015.63	79937.01	98595.54	102559.6	85072.37	79585.85
84802.66	84452.55	64888.16	87240.79	79240.66	60739.02	107460.8	102240.2	85221.16	80719.13

LP374.254	LP374.254	LP374.254	LP374.254	LP374.254	LP374.254	LP374.254	LP374.254	LP374.253	LP374.254
96242.22	98445.52	80475.54	76051.53	69293.65	61386.5	70520.28	75414.76	78869.2	74142.03
78931.71	101964.3	80855.74	76140.84	70180.71	66429.61	75793.33	68042.54	76333.37	83197.6
73739.3	99678.8	103257.5	77818.89	74424.47	66808.21	69534.44	69187.51	78414.51	86225.28
72574.71	94700.89	80969.46	72260.76	78624.6	71481.67	89802.68	78997.69	76578.28	84044.34
79140.93	93889.43	84213.17	71483.12	64437.06	61372.18	67760.79	79113.22	67455.71	82338.06

LP374.254	LP374.254	LP374.254	LP374.254	LP374.254	LP374.254	LP374.254	LP374.254	LP374.254	LP374.254
59325.15	65757.5	80472.75	91954.09	93222.45	57481.83	97899.14	66425.86	66633.26	91412.26
53462.59	72507.29	71915.14	85585.13	90101.8	58027.94	89761.2	66306.1	63533.85	86396.98
60227.3	68872.51	77489.43	85286.3	79800	65069.24	92634.4	67479.89	70597.55	93006.35
59623.57	63002.57	84600.03	88040.44	86388.02	67465.95	93447.56	76652.66	68744.03	86375.96
53460.75	90642.77	79465.69	94401.63	89166.87	59634.96	94582.93	66087.4	68574.71	93572.35

LP374.254	LP374.254	LP374.254	LP374.254	LP374.254	LP374.254	LP374.254	LP374.254	LP374.253	LP374.254
81508.96	85871.5	92673.66	75950.91	96758.87	69205.44	70055.04	77162.47	81097.49	92586.87
80899.8	96761.34	95436.28	67379.83	82176.17	65636.5	68680.9	73542.87	84526.95	67936
74960.32	95398.03	94098.02	78499.3	88124.26	68608.03	71854.31	91022.76	78030.63	89791.21
77500.21	87059.3	91766.33	72585.25	91094.31	65414.99	74186.9	73980.23	86160.41	94718.89
78962.41	84603.5	94831.64	68261.11	90310.32	63888.62	67363.11	80155.52	77450.86	114083.4

LP374.254	LP374.254	LP374.254	LP374.254	LP374.253	LP374.254	LP374.254	LP374.254	LP374.254	LP374.254
62541.79	76703.63	102592.5	75184.41	91078.09	67644.38	61420.43	81056.59	74289.77	69873.82
58634.82	74461.41	99212.96	72099.67	68592.79	53719.92	70832.56	81710.02	73889.57	67066.48
60204.43	75016.09	96343.49	75700.05	72534.53	60637.95	71930.23	74765.88	74362.96	67632.64
59694.66	78136.64	100946	70636.75	88075.28	56336.93	66152.59	78637.39	72329.57	61957.01
59513.02	73731.31	101167.3	74833.98	74145.7	60493.44	63595.46	78412.35	74147.79	62437.41

LP374.254	LP374.254	LP374.254	LP374.254	LP374.253	LP374.254	LP374.254	LP374.253	LP374.254	LP374.254
97659.17	74436.74	76445.19	109877.7	82077.49	87647.65	59400.02	55228.53	62891.06	104647.2
91074.46	68816.9	77135.47	117187.7	77898.71	84343.32	61140.64	65381.83	66019.95	100796.1
90492.2	66421.37	78124.92	116005.6	81644	89961.5	64401.38	56835.94	65544.28	97741.6
88951.3	76126.42	78420.56	111948	86467.54	86091.41	62920.82	60069.96	65291.43	103099.5
88680.1	68567.93	68987.82	108094	82229.4	84572.63	66425.49	58523.52	65185.04	95872.9

LP374.254	LP374.254	LP374.254_	LP374.254	LP374.29_	LP374.29_	LP374.29_	LP374.290	LP374.290	LP374.290
81791.49	58092.92	54643.94	122598.9	115106	103551.2	77774.53	49331.28	70155.39	87905.84
76943.71	61202.66	56531.63	119202.9	98471.02	85962.88	83430.33	57251.44	69395.38	92711.4
78523.9	64792.36	55685.17	129517	107724.9	107772	80481.71	50451.71	67410.51	93743.3
79773.77	63123.08	54958.55	125768.6	103101.7	96706.48	91204.79	56985.15	69126.15	91384.68
84224.5	59961.82	59972.26	125330.3	105970.9	97028.52	71504.26	50316.14	68324.4	84888.67

LP374.289	LP374.290	LP374.290	LP374.290	LP374.290	LP374.290	LP374.290	LP374.290	LP374.290	LP374.290
52052.33	97564.11	69437.87	66870.98	106113.5	69681.84	84828	59481.98	112196.2	73474.84
48830.77	96161.56	71106.53	59779.11	96856.89	62611.18	83964.04	57112.5	109439.1	74486.2
41677.92	85678.92	68357.01	64452.42	106807.2	68173.08	83058.18	50359.66	119959.4	72994.33
50386.82	86785.2	73530.94	64955.04	101121.5	69905.95	83655.55	48976.86	125571.9	75860.04
51520.88	98114.37	69863.51	67677.6	91626.45	68463.28	79833.32	50877.2	106888	74530.15

LP374.290	LP374.290	LP374.290	LP374.290	LP374.290	LP374.29_	LP374.289	LP374.290	LP374.290	LP374.290
80848.06	54614.59	90460.26	79393.04	120187.5	89493.9	91014.48	108157.3	91763.98	107589.9
73401.73	49511.15	79590.06	76406.44	115170.4	92498.58	74481.35	90593.61	104691.7	91748.24
75611.31	50269.23	82198.05	72409.9	118650.7	94966.34	79500.08	97645.03	98264.77	101332.6
76316.95	54441.1	84319.04	75368.52	115847.7	86183.01	84573.42	116965.4	94904.58	100059.9
85584.94	52890.72	79259.14	75461.74	117655.4	88666.72	90495.13	103538.6	90441.01	107012.3

LP374.290[illegible]	LP374.290	LP374.303[illegible]	LP374.326[illegible]	LP375.162[illegible]	LP375.162[illegible]	LP375.162[illegible]	LP375.162[illegible]	LP375.162[illegible]	LP375.162[illegible]
81241.99	102381.2	90114.15	109139.7	39789.63	48064.96	32348.49	35097.58	53449.04	51664.48
71200.25	116269.6	83669.48	96959.54	36245.58	49655.53	29918.67	31778.28	54225.49	45792.18
79893.33	102592.3	94088.37	113420	35863.02	44507.59	34140.37	27862.5	54934.82	43370.39
73375.44	107667.9	91267.32	105251.9	36208.01	49869.94	28623.57	31817.34	59364.28	45420.51
76003.3	113653.6	88569.71	108701.9	39181.96	42674.89	30151.32	31576.1	50053.17	44076.19

LP375.162	LP375.200	LP375.200	LP375.200	LP375.247	LP375.347	LP376.232	LP376.233	LP376.232	LP376.233
36165.86	55905.2	43856.92	45458.02	24439.55	62551.45	94715.39	96153.13	48716.44	96896.56
33374.92	59755.2	52800.63	45444.02	23704.85	63656.51	91018.68	91964.78	54838.11	91300.98
35276.3	57242.59	54539.04	43500.69	23187.54	62084.02	101213.4	89004.89	60081.36	97951.64
35960.63	58414.74	54160.66	45829.51	26394.15	59796.62	94992.7	94844.53	50634.88	86418.4
33909.28	62994.09	44993.93	48943.32	21688.51	59815.08	99795.09	87069.94	53023.7	90057.81

LP376.233	LP376.233	LP376.233	LP376.233	LP376.233	LP376.233	LP376.233	LP376.233	LP376.233	LP376.233
92481.9	109301.9	117972.4	66365.38	119060.4	177698.6	107698	113746.4	94462.96	87097.51
87095.29	102807	125135.3	66121.87	117704.4	161870.3	113864.5	116569.4	83157.93	80192.55
88769.96	111209.9	125759.7	71094.98	123708	166711.6	101997.1	115647.8	93824.8	75989.26
74258.17	103925.5	124890.8	62164.68	129961.7	177209.9	105320.8	118114.2	87603.29	80371.18
84569.01	99157.91	132585	64723.14	116972.1	165863.8	104178.1	120440.9	84838.83	86806.4

LP376.233	LP376.233	LP376.233	LP376.233	LP376.233	LP376.234	LP376.233	LP376.233	LP376.233	LP376.232
99503.18	75315.36	68560.84	62493.62	123687.3	58648.53	82905.84	71040.44	68176.07	85018.51
100853.8	71622.69	83782.45	64053.75	127976.7	60060.58	77967.19	71696.13	68377.72	97275.37
96453.15	71460.46	65111.55	65256.47	125164.6	61385.88	88130.4	69489.09	65446.41	88968.95
98752.85	73532.54	67137.03	72727.44	123618.4	65652.61	79064.94	74435.82	71784.13	77879.68
103969.5	75742.31	73510.06	68417.74	130342.4	73134.82	85363.25	81081.36	69761.07	85159.06

LP376.233	LP376.233	LP376.233	LP376.233	LP376.233	LP376.233	LP376.269	LP376.269	LP376.269	LP376.269
84045.09	120977.4	91002.07	68503.52	110749.9	107719.4	150725.3	238271	96000.11	109073.3
78923.31	130107.5	82774.45	80081.44	104839.1	105466.6	94098.61	493698.6	127134	139875.8
85517.91	123912.1	85478.96	67730.7	96304.13	108360.8	107587.1	270502.7	100707.9	135640.9
81427.81	127303.6	84039.75	66592.29	106103	99837.72	165586.9	548795.1	83499.4	114312.7
80606.15	116313.9	92372.45	63038.85	100862.6	103358.8	109843.6	341721.3	98595.95	134359.6

LP376.269[illegible]	LP376.269[illegible]	LP376.269[illegible]	LP376.269[illegible]	LP376.269[illegible]	LP376.269[illegible]	LP376.269[illegible]	LP376.269[illegible]	LP376.269[illegible]	LP376.269[illegible]
230231.3	118984.3	283167.5	132656.1	130352.7	102781	169242.6	247579.4	127135.3	159439.5
98967.24	151200	253697.3	93553.96	146421.7	90682.34	101841.4	137657.1	124472.3	178263.8
152940.6	152974.5	216630.3	123543.4	143874.4	100253.2	127628.7	174378.3	132903.2	125043.2
132288	121627.5	136422.4	130400.7	151050.8	121957	162786.7	182138.3	119735.9	165195.6
251903.5	117321	282975.6	120342.6	143152	71996.57	124276.5	197633.2	121905.1	117560.9

LP376.2699	LP376.2697	LP376.2698	LP376.2697	LP376.2698	LP376.2697	LP376.2698	LP376.2690	LP376.2697	LP376.2699
112512.4	96009.94	119568	96648.61	130277.4	115889.2	104040.5	111472.4	235077.2	106518.2
118550.8	117093.3	108036.2	93551.94	142467.1	114252.7	92122.37	99307.96	231962.8	111500
117962.1	136289.3	90402.57	99003.43	149037	112175.4	98171.31	85127.88	225234.1	111788.1
118326.5	89526.99	128405.1	95581.31	131236.9	123030.8	107859.5	113630.6	230816.4	112445.3
114402.2	89985.7	90067.72	87221.64	144862.9	103447.5	104668.2	97256.21	227541	84618.46

LP376.269	LP376.269	LP376.269	LP376.269	LP376.269	LP376.269	LP376.269	LP376.269	LP376.270	LP376.269
112889.2	103589.7	210363.7	81927.8	145432	99341.8	97200.99	126062.9	138594.1	89793.86
123342.2	104339.2	206460.6	81212.41	118121.1	95607.8	97097.74	122179.6	132708.7	86316.69
124462.1	122420	100691.1	86535.17	138908.4	92359.87	88852.66	115147.1	138360.3	76285.35
134828.3	105071.7	209941	81766.8	153969.8	94613.05	101003.9	123546.4	127456.3	91053.47
118682.5	96044.5	218316.1	89823.55	138973.5	93172.88	97139.56	122091.5	136964.8	91448.11

LP376.269	LP376.269	LP376.270	LP376.269	LP376.269	LP376.27_	LP376.269	LP376.269	LP376.269	LP376.270
663961.7	94233.51	113236.3	106678.1	185772.1	91353.49	121375.1	110608	67123.24	108452
725326.5	92841.96	109828.3	113533.2	197328.4	87396.24	115425.8	107671.2	70382.43	122572.9
957025.3	93139.35	102840.2	91254.9	143801.6	96373.02	115131.2	105961.4	64714.89	120693.5
813933.3	100901.1	104901.7	119016.9	204377.7	98390.03	126600.2	107997.8	70463.69	111691.1
1162475	85648.85	118632.9	115613.8	162225.1	94386.84	132624	115447.6	68755.48	109983.4

LP376.269	LP376.269	LP376.269	LP376.269	LP376.269	LP376.269	LP376.269	LP376.269	LP376.269	LP376.269
99030.81	100482.5	80824.79	113502	55098.71	101685.5	81829.47	76558.12	71973.46	92296.64
89988.73	96176.79	82907.33	117061.6	50736.42	97245.8	85014.03	77517.03	85046.16	103817.2
102349.2	102657.6	85442.02	117237.9	61027.09	104159	81575.16	82015.95	75781.6	99045.16
95650.19	103485	85047.63	111176	62543.35	108472.8	80973.6	74564.95	78030.93	101056.9
98704.74	95848.58	77046.99	118459.8	48491.29	102467.2	83711.49	77214.57	71448.46	101187.7

LP376.270	LP376.269	LP376.269	LP376.269	LP376.27_	LP376.269	LP376.297	LP376.306	LP376.306	LP376.306
61080.86	84142.27	112801.2	228071.8	91782.66	102582	290852.1	223342.9	223291.4	298166.3
59508.68	83771.46	113075.7	236062	95122.72	106972.1	298668.6	258922.8	234080.2	302639.2
60870.8	87844.9	114863.1	260472.6	100664.1	93279.32	273635.1	220452.8	231277.7	312043.3
59471.47	91270.28	114522.8	223155.2	97048.87	97693.53	260290.8	228963.5	257248.1	308216.6
61290.71	82137.75	112031.9	235472.1	88624.15	97934.3	259020.7	209218.6	234358.3	310045.3

LP376.306	LP376.306	LP376.318	LP376.319	LP376.342	LP376.342	LP376.342	LP376.342	LP376.342	LP376.342
167851.4	133650.9	115085.3	82235.33	342366.5	120395.6	241874.3	376214.1	236413.7	206868.5
181311	137302.2	115025.7	74541.61	303828.3	131085.3	232205.7	377890	273066.7	196787
177360.9	136985.2	110142.6	80691.07	301341.2	142817.8	221966	290871.3	205550.5	373211.8
166595.5	130284	122364.7	80706.06	300235.4	118189.3	233305.9	369617.6	225294.1	205091.8
189232.4	129445.7	114701.4	74136.04	345528.3	113744.7	215412.7	355109	251081.2	200977.9

LP376.342	LP376.342	LP376.342	LP376.342	LP376.342	LP376.341	LP376.342	LP376.342	LP376.342	LP376.342
322010	122238.8	118921.3	222971.1	117519.4	88088.27	131285.2	1002884	407778.3	287683.3
208342.1	115956.7	83347.3	219495.8	122677.5	87439.67	122364.1	825519.1	502048.6	316995.1
242288.6	124216.2	85935.35	228900.9	124325.4	82604.92	126318.5	1325388	454661.3	259475.7
219575	119793.4	100255.2	236357.7	122977.9	85431.84	127713.2	1428133	452346.1	282448.1
198517	113738.3	86962.82	227576.4	114793.8	86890.74	121779.2	1560398	605051.1	180779

LP376.342	LP376.342	LP376.343	LP376.342	LP376.343	LP376.342	LP376.342	LP376.355	LP376.456	LP377.158
201890.9	177840.7	159078	291141.5	173652.4	311761.1	147999.2	17843.32	24150.77	22906.21
216864.6	191910.6	172709.2	275050	171089.3	281838.7	153488.9	20305.39	20795.96	24701.25
184917.4	200317.6	172163.2	292858.1	177315.3	320683.7	144850.3	32187.61	16802.97	17041.96
264284.2	194239.5	167552.3	297125.4	175800.1	314481	153459.1	40459.1	15762.46	23551.63
260881.3	156856.4	164178.6	283920.7	174520.1	318508.2	135657.3	43894.82	17875.74	24500.76

LP377.178	LP377.178	LP377.178	LP377.178	LP377.178	LP377.248	LP377.268	LP377.301	LP377.305	LP377.322
45943.85	32347.13	48694.61	78576.95	52246.63	26162.06	105191.1	59267.11	29274.93	20065.96
38840.87	43330.49	52873.51	76426.78	62044.63	16476.24	99408.98	58084.83	30318.74	21159.52
46758.97	42253.02	49129.41	70604.61	56710.33	21407.28	109295.5	55287.16	26610.63	20132.34
46571.2	42374.74	53117.46	74128.18	55524.91	23335.53	109003.8	52386.86	29885.3	19321.54
43444.4	44851.06	51903.86	77082.39	60329.94	20462.52	98317.34	52452.62	28212.26	18164.52

LP377.326	LP377.345	LP378.212	LP378.212	LP378.212	LP378.212	LP378.212	LP378.212	LP378.212	LP378.212
23942.54	125179.5	170137	166963.7	88206.85	282504.7	176509.4	190541.8	129365.5	229241.1
25759.22	111930.1	175056.8	175780.8	144747	266112	86580.71	193919.3	129190.7	211616.2
28724.2	83245.27	161148.3	183221.9	129158.7	270416.8	182297.4	208321.8	132590.4	211952.8
26705.18	110248.3	100252.1	179630	118690.6	92714.92	90702.68	93057.21	76892.68	228249.3
26922.29	107351.9	157167.5	91416.39	128092	246979.7	187990.8	199481.7	145888.6	227452.7

LP378.212	LP378.212	LP378.212	LP378.212	LP378.212	LP378.212	LP378.212	LP378.213	LP378.212	LP378.213
248098.4	230476.9	230527.9	197231.9	88949.41	113884.4	72654.49	230508.5	151613.1	197199.8
241431.6	95861.93	224207.9	215597.1	93090.27	120266.2	104376.2	236683.6	83849.58	194107.7
234920.4	237288.5	223377.9	205321	96067.47	119873.4	218936.5	256650.7	156494.9	200051.7
153691.1	241351.3	233025.4	210451.3	68304.46	85180.42	100917.4	131948.1	82244.71	107008.9
263977.7	230006.4	236665.3	213952.2	90950.83	119955.7	199397.5	286004.6	170664.9	205494.5

LP378.213	LP378.213	LP378.212	LP378.213	LP378.213	LP378.213	LP378.212	LP378.213	LP378.213	LP378.213
154275.4	162217.4	211862.1	288421.9	274053.6	270094.5	256291.9	187876.5	257620.8	443732
164611.5	174167.1	87103.52	284510.7	270910.9	261964.6	248433.8	183094	246062.3	462724.9
151893.8	176099.6	204303.2	280677.8	271891.5	280445.1	270384.2	194243.7	259111.4	455283.4
66685.67	80493.34	221127.6	282989.4	264041.7	131788	128671.4	100208.5	123996.8	223743.6
158720.9	170828.6	200279.4	307510.8	276379	285095.7	255645.1	203218	267599.9	495794

LP378.213	LP378.213	LP378.213	LP378.212	LP378.213	LP378.222	LP378.248	LP378.249	LP378.248	LP378.248
388175.2	120341.6	220132.4	147328.2	153961.9	119550.6	189936	175167.9	183831.7	178940.1
400523.2	123646.8	204698.9	158658.2	159073.9	126282.7	209915.2	176737.3	173913.6	171124.1
392784.9	115134	206160.5	153747.5	153369.7	132980	240588.4	204256.1	187894.1	180551.7
176994	126290.2	209942.5	91768.05	79196.63	133245.5	187124.9	167245.8	171062.4	172384.7
429872.5	133371.5	202050.2	139696.2	175115.9	146161	191736.1	167482.7	148183.6	182591.5

LP378.249	LP378.249	LP378.249	LP378.248	LP378.249	LP378.249	LP378.248	LP378.249	LP378.249	LP378.249
308664.2	267026.9	175109.2	183980.8	144470.2	247680.4	182028.1	141201.4	293764.7	193938.6
299375.6	283127.1	170486.7	185957.9	147832.2	247266.1	185385.9	143129.6	294182.8	220368.2
300781.5	256865.3	193570.2	192041.1	169426	174143.7	197820.2	144734.7	324444.3	220498.7
258863.1	268079.1	187173.5	192356.3	153820.7	254403.9	202733.7	154272.9	308916.9	196782.3
267848.5	258463	174365.6	183685.8	140659.9	260808	188468.8	142663.6	342157.5	233839.4

LP378.249	LP378.249	LP378.249	LP378.249	LP378.248	LP378.249	LP378.249	LP378.248	LP378.249	LP378.249
212741.7	312381	188122.2	369216.6	185708.8	119693.8	311459.2	186081.9	158934.1	183009.7
205675.1	319567.8	190957.5	368655.3	161733.4	121973.1	302903.4	191778.7	174163.5	191593.7
207861.5	305695.5	191364.6	334668.5	179760.8	132351.6	311853.8	197049.2	139535.8	199857.2
229798	323738.5	184963.3	349587.6	180486.9	121138.6	307575.1	207760	169965.2	199026.1
223759.4	317907.9	197207.6	370480.8	175644.9	127696.9	324494.6	193907.4	180846.6	205137.2

LP378.249	LP378.249	LP378.248	LP378.249	LP378.249	LP378.249	LP378.249	LP378.249	LP378.248	LP378.249
272128.4	179046.6	164125	188946.3	190964.6	173770	275121.6	206084.7	186600.9	193772.8
257270.5	185497.7	146714.6	185169.5	191032.6	160961.4	275388.1	207556.7	194204.3	191005
252970	177454.3	162423.8	189451.5	183286.1	170934.4	285944.5	207304.1	299795.8	190816.3
252148.2	188846.6	157788.1	201906.8	193864.6	163048.1	297699.6	209200.3	192297.9	197632.2
270281.5	183123.7	154442.6	189971.1	199292.2	163043.8	279253.6	214542.4	186378.5	214811.1

LP378.249	LP378.248	LP378.249	LP378.249	LP378.249	LP378.249	LP378.249	LP378.249	LP378.249	LP378.249
209957.8	122245.8	145835.1	224107.2	226798.8	266857.8	195699.1	149360.5	152080.4	197387
199617	127446.7	139928.4	233856.2	225398.9	265391.2	186818.4	162002.5	145159.5	205104.6
192513	130548.5	140501.9	232373.9	221201.2	283713.7	193258	152112.2	147169.2	240137.6
207394.4	123350.3	135235.8	242255.2	227396	280324.4	192592.8	165775.1	151981.5	194960.4
207200.3	128044.1	155445.6	239470.6	238107.9	275399	200986	158615.8	138372.6	194311

LP378.249	LP378.248	LP378.249	LP378.248	LP378.249	LP378.249	LP378.249	LP378.249	LP378.249	LP378.249
250067.7	124113.1	145590.9	168688.5	148511.1	233124.4	190053.6	163886.1	164490.5	196295.1
235994.7	130273.3	148730.6	167155.3	151331	223829.2	185480.4	160939.8	168548.2	188074.4
235416	121029.1	145180.1	164220.9	159736	230814.4	189220.9	178406.3	153128.2	197503.2
250606.6	121551.4	144240	168691.4	142349.5	226595.8	209387.8	176092.9	160239.3	198036.9
234226	131174.3	156923.1	171924.1	141339.1	240971.8	177868.9	165125.8	158279.4	192781.2

LP378.249	LP378.249	LP378.249	LP378.249	LP378.249	LP378.249	LP378.249	LP378.249	LP378.249	LP378.249
204984.1	137087.6	129941.4	141800.9	171400.6	86135.31	175007.6	180089.4	176034.2	115061.7
200295.8	131033.6	128953.6	141295.4	176989.3	91469.96	165932.5	164914.6	179149.1	106168.4
207342.5	136179.6	147325.9	141710.7	169012.9	86234.67	161698.2	164980.3	172338.4	111737.4
219934.5	142649.8	134168.8	146935.8	186006.4	88784.3	175577.7	172478.8	188009.6	112866.4
199321.7	130281.3	133092.8	145787.9	170141.1	95250.76	158160	177313	183384.9	89696.81

LP378.248	LP378.249	LP378.249	LP378.249	LP378.249	LP378.249	LP378.284	LP378.285	LP378.285	LP378.284
68622.1	261821.2	194659.4	174279.9	87295.11	102428	82084.08	71498.16	50289.63	47136.89
79197.03	264518.5	174324.1	167842.8	81374.99	105884.7	79004.82	68337.56	57238.29	46135.92
71738.87	272774.8	177998.8	172425.4	89061.3	109906.4	82892.4	63959.41	56404.4	47666
69852.68	270625.4	201977.6	151288.7	84018.21	109453.2	82546.56	55214.14	54663.1	46768.97
74965.37	283834	199633.1	155641.7	82864.51	109400	92813.05	68811.33	57296.18	44765.8

LP378.285	LP378.285	LP378.285	LP378.285	LP378.285	LP378.285	LP378.285	LP378.285	LP378.285	LP378.285
64703.84	89197.12	78638.38	57347.56	57775.25	57720.43	93349.18	102929.1	75920.19	61082.92
56662.34	85329.64	79059.91	65106.25	61109.48	55550.58	93682.3	85893.91	78114.82	71244.64
61095.92	87393.49	76339.69	56689.61	58274.12	63256.86	91939.61	84563.35	78533.51	75740.73
88095.61	92534.62	76389.08	61740.47	60770.24	43495.95	96151.93	98120.17	79389.42	75178.82
54825.86	89332.91	77599.65	65425.77	60015.01	58239.42	97773.25	96244.35	75888.78	66550.79

LP378.285	LP378.285	LP378.284	LP378.284	LP378.285	LP378.285	LP378.285	LP378.286	LP378.285	LP378.285
57414.53	69011.15	44433.28	52849.94	84286.45	77102.55	46622.33	60086.77	81242.56	53189.8
54363.79	64565.4	40162.72	45832.95	88642.11	86190.34	48365.82	58173.54	87166.9	52608.13
60417.91	64381.09	39724.5	62052.3	86306.35	85072.49	48517.35	58498.87	87627.28	56685.93
57874.06	79217.9	44146.33	59721.1	84592.78	88297.51	48226.59	64918.19	90909.91	53932.19
59659.88	61689.18	51654.57	45692.41	80506.03	83692.47	42400.26	66498.11	93392.19	58830.4

LP378.285	LP378.285	LP378.285	LP378.285	LP378.285	LP378.285	LP378.285	LP378.285	LP378.285	LP378.285
52771.31	52662.46	47103.03	59779.52	39793.09	54251.46	57585.25	50480.67	71258.62	62222.11
49893.24	57427.75	50117.17	58528.02	41409.28	57293.19	57714.07	53942.35	71507.34	59965.66
57287.12	54795.58	53859.49	62671.82	36442.69	55871.07	55560.03	54575.84	70807.2	63899.19
53271.12	59829.92	47210.8	64118.42	43764.06	55045.19	64141.38	52571.58	73544.09	50103.84
55072.97	61734.28	49884.13	55585.21	32177.51	53945.89	48799.43	44939.04	67357.45	64936.14

LP378.285	LP378.285	LP378.285	LP378.285	LP378.285	LP378.300	LP379.084	LP379.084	LP379.084	LP379.084
62354.59	51603.41	54564.67	64519.04	52627.67	26368.31	44038.89	33649.44	28071.91	27161.36
63229.91	52652.66	53553.37	66767.38	55659.44	23537.28	39783.13	30864.82	28214.31	24680.69
61951.13	49769.46	55349.57	61820.62	53295.3	24844.78	51083.46	44411.89	35336.39	37414.27
59869.2	59989.51	55428.81	67514.01	51691.59	22541.95	52588.59	35899.17	32421.16	37911.55
64179.41	45009.73	52138.32	71796.15	50845.38	21701.67	41520.5	31911.59	24096.34	22256.65

LP379.084	LP379.084	LP379.084	LP379.084	LP379.084	LP379.084	LP379.084	LP379.084	LP379.084	LP379.084
23719.13	26558.19	33319.92	23987.47	25110.43	32935.5	24939.71	23007.81	34782.03	25518.12
24927.98	25599.91	32641.47	26554.06	25369.3	32065.45	25556.16	20821.56	33813.24	21398.29
33530.94	30377.28	38933.83	28272.1	31639.09	39498.82	28091.62	29595.14	44553.94	29467.12
32683.67	38211.54	37501.11	32320.57	30824.97	46315.17	35848.37	32817.67	39199.07	35445.91
25533.23	27503.78	24695.89	27383.96	18246.55	31842.2	21664.72	20707.1	32719.95	20473.88

LP379.084	LP379.084	LP379.084	LP379.084	LP379.154	LP379.237	LP379.248	LP379.251	LP379.251	LP379.251
28720.67	39288.75	37312.22	17647.28	177907.3	27366.57	34725.19	49722.47	57334.63	54360.06
28814.41	30552.88	31040.72	18441.18	165089.9	28156.07	36504.17	49305.77	55495.77	48792.34
31511.29	42865.88	46499.35	21243.01	201184.6	30762.72	45802	49901.04	60727.15	57310.64
37760.56	41391.83	45876.26	26096.84	199632.7	30941.69	47131.67	51266.44	59577.65	58211.92
30946.69	29627.43	32672.93	17559.6	144015.8	26170.67	50904.85	53133.34	62861.69	63075.8

LP379.251	LP379.251	LP379.251	LP379.251	LP379.251	LP379.251	LP379.250	LP379.251	LP379.251	LP379.251
35415.27	47717.62	37284.43	26534.2	34378.46	26105.89	30240.21	42835.68	32552.98	34185.67
27766.62	41080.59	34954.27	25179.68	27227.07	26430.5	28261.71	40808.16	32436.65	33517.9
32950.38	54043.37	35784.41	27913.89	31883.79	27144.86	34988.74	48131.63	33455.22	36877.21
30316.25	49387.27	44576.18	26208.32	32087.48	30089.38	32693.6	44215.2	25864.33	36526.32
32410.52	48463.61	38086.96	26014.14	31637.99	27876.02	32856.05	46399.61	32616.25	32359.42

LP379.251	LP379.252	LP379.251	LP379.251	LP379.251	LP379.248	LP379.250	LP379.250	LP379.251	LP379.251
39924.08	52242.56	64517.43	49698.86	43467.52	19069.71	47809.1	26828.65	43598.5	34207.74
32310.07	45737.52	60671.96	51412.14	45658.82	20614.9	40619.45	32657.04	39509.51	38025.02
42267.08	49909.38	59858.56	51789.3	44060.71	19392.33	42939.32	28346.63	37312.39	38457.08
44491.93	48044.67	58428.68	48338.97	49593.24	21651.59	44207.68	33106.63	41201.8	37920.4
43470.91	47423.05	65223.22	54677.52	47447.24	20279.28	45089.11	33738.76	42753.55	42115.22

LP379.251	LP379.319	LP379.320	LP379.320	LP379.320	LP380.157	LP380.226	LP380.279	LP380.28_	LP380.279
53490.8	63043.21	40207.62	41680.39	56339.65	36174.78	318608.5	3034842	3714208	480921.6
48582.21	54595.44	47240.86	40115.43	54901.28	38495.69	292697.4	2492971	3805416	619983.8
52209.59	52239.07	42814.73	40268.96	56001.62	52498.37	328175.2	2524110	3545047	481666.7
57743.1	54082.17	39978.59	41205.83	49557.2	61642.76	311266.6	5463506	3594624	547342.6
53890.85	60504.92	47589.28	41350.66	58600.7	52544.19	282074.5	1893660	3498830	443099.7

LP380.279	LP380.279	LP380.279	LP380.279	LP380.279	LP380.279	LP380.28_	LP380.279	LP380.279	LP380.28_
5344969	2254298	1225825	1352398	1642526	1612202	3607446	2743970	2333152	3367698
4639291	2027752	1641298	987500.4	2873245	2583401	2181738	4108194	2372118	3530996
3106518	2303503	1810695	1545966	1634226	1473624	3414891	2212103	2601916	2939015
3884937	2473284	1543168	1264945	1483859	1528112	2875285	2647342	2813538	4198290
3516553	2055318	2684461	1472692	2545927	2534873	3200715	2500765	2099564	4768924

LP380.28_	LP380.279	LP380.279	LP380.279	LP380.28_	LP380.279	LP380.280	LP380.279	LP380.279	LP380.279
1862738	3183386	4005739	3250507	3535184	2656600	2791870	1854766	2309388	2841035
2939545	2301299	2883526	3230377	3894120	2380230	3457497	1762476	2379256	4089022
2352764	3788809	2954070	2589698	4376453	2276003	4127043	1633689	3536894	8886022
2343987	4459845	3777145	2720791	2783663	1826508	3459595	1756845	4169372	2936166
2247074	2754426	3187748	3599478	3444915	1390972	3146919	1991105	3127907	3784468

LP380.280	LP380.279	LP380.280	LP380.280	LP380.279	LP380.279	LP380.28_	LP380.28_	LP380.279	LP380.279
3939969	1246148	3464511	2738204	1679805	2301520	2945825	3179336	4405091	1652978
3546267	1178296	2852929	3516608	1648191	4038783	2669978	3401324	5068576	2108753
5658202	1461054	3412457	3371429	2069882	3585935	4240883	3335941	3484955	1705661
4359926	1478402	3357708	3233672	1425795	3216426	4806386	3842304	4414931	1623629
3008902	1526508	3546214	3377054	1216324	3191301	3704887	5196413	3872681	1405778

LP380.279	LP380.28_	LP380.279	LP380.279	LP380.28_	LP380.279	LP380.280	LP380.279	LP380.279	LP380.280
3653722	2972956	2107066	1294008	3038107	1407673	3410710	404734.6	203357.9	3634495
3658695	4234807	3862099	1931447	3503541	1520827	3544094	350164.4	217542.3	2461771
3923901	3755494	5217568	1229325	5324947	2499866	2547507	270119.7	262630.4	2882991
4599215	3632923	2917322	1363210	3251055	1628120	2731163	340848.1	201125.3	3465265
3632959	3910169	2659104	1372817	3167907	1516002	5375622	297598.1	259344	3460250

LP380.279	LP380.279	LP380.279	LP380.280	LP380.28_	LP380.279	LP380.280	LP380.28_	LP380.279	LP380.279
876537.1	1506178	3216266	2431591	2677166	2329093	3873641	1863589	3005146	2512232
941306.6	1298430	2149519	3601842	2346065	2455311	3240288	1982329	3567317	1736907
1089002	2171876	2159866	3391693	2552164	2588897	3020878	2853055	2577911	2053512
1126470	1378820	2159061	2280989	2644320	3117560	3558125	1533711	2899568	2102339
926792.6	1441898	3917904	3370309	2561333	2481951	3522633	1779567	2952017	1973929

LP380.279	LP380.279	LP380.28_	LP380.279	LP380.28_	LP380.28_	LP380.279	LP380.28_	LP380.279	LP380.279
3467459	482174.9	3627650	292699	2174980	1402392	619388.9	2106079	1214798	460972.9
2332287	743248.4	2604928	228358.4	2402238	2698583	882908.7	2547852	1811667	480791.4
2148230	466573.4	2208474	299890.1	2638972	2370742	657641.1	2624326	2164867	425383.8
2993319	521792.7	2738910	448384.3	2147540	1446051	1109494	2010629	1194963	721363.9
2179177	697655.8	2392316	250773.6	2381169	1721371	759278	1804239	1553547	572903.8

LP380.28_	LP380.279	LP380.28_	LP380.279	LP380.279	LP380.280	LP380.279	LP380.28_	LP380.279	LP380.279
2413540	357377.9	2650904	536695.5	517872.1	2826557	451649.4	1191579	430359.9	1531895
2114216	466912.3	2986485	462569.8	609196	4724476	272493.7	1197321	427783.1	1242893
2441331	624918.7	2410558	339996.9	401614.8	3177443	340029.4	2119020	445443	2320744
2927412	513049.4	2633497	378815.3	690220.8	2738221	251847.5	1954827	720352.1	1848082
2315035	532607.2	2260304	350808.6	428916.1	4144599	416392.3	2164381	390642	1709440

LP380.279	LP380.279	LP380.279	LP380.280	LP380.280	LP380.280	LP380.280	LP380.279	LP380.28_	LP380.279
351321.7	1957519	2543048	1706687	2457496	1375843	2440790	1129677	2557130	1533682
419363.5	2002126	1695949	1942123	3478465	1869480	2532028	729712.3	2002581	1459344
427194	2057345	2046154	2411171	3863621	1470691	2910751	1165657	2060169	1658299
485379.8	2078964	1615961	2031836	2775203	1646021	2649260	958224.7	2021120	1476657
390067.5	1794840	2011117	1379971	2773274	1547512	3173314	961367.4	2106671	1837686

LP380.337	LP380.337	LP380.337	LP380.352	LP381.188	LP381.188	LP381.188	LP381.189	LP381.188	LP381.189
714123	83740.01	218642.3	8563.08	88625.09	59002.04	57097.07	48636.98	62121.05	54524.66
711286.7	79881.94	30534.41	11481.99	45766.41	52994.26	73173.34	58187.54	68527.81	69351.11
727453.9	75892.25	39112.38	14083.74	65860.06	57475.02	54152.87	55469.52	65452.97	47311.42
686114.4	66008.57	50361.07	16237.23	62368.52	58760.44	45164.73	53610.42	60120.21	50781.49
704493.8	69342.68	49201.48	19486.16	57219.46	61817.66	49268.54	59808.98	53167.52	45122.75

LP381.189	LP381.188	LP381.189	LP381.188	LP381.189	LP381.188	LP381.188	LP381.188	LP381.188	LP381.188
52222.88	48846.64	67241.1	49386.34	46760.79	44927.58	63040.41	88095.78	60351.08	68549.26
59366.07	54473.54	59702.01	49554.63	57126.77	76592.49	45891.25	57161.68	72316.17	52286.82
45820.73	60499.1	54028.58	49126.33	53031.54	49861.87	62017.24	53449.19	60817.64	67622.79
48079.96	54449.8	54743.01	42020.61	53985.28	50644.12	56429.89	60138.11	58770.78	62639.31
46696.93	55473.95	49741.11	43417.05	50939.84	51707.54	51730.23	47695.84	56045.45	64706.15

LP381.188	LP381.188	LP381.188	LP381.188	LP381.188	LP381.188	LP381.188	LP381.188	LP381.189	LP381.188
49284.4	76956.8	54820.26	54937.91	58481.52	86068.6	36522.97	69085.35	62705.7	46410.85
77997.66	46102.92	51346.32	53062.98	42949.82	94167.26	40245.27	66191.92	56651.47	68718.68
65084.01	57200.11	55486.37	51598.03	44643.3	73502.08	45490.08	62477.65	56658.97	51496.21
58979.07	54747.5	57307.28	53921.61	48263.1	67759.42	47426.85	55367.38	57460.86	53700.21
59350.88	63942.94	50449.94	44300.3	45408.76	68292.05	45990.94	52849.77	60888.74	54332.75

LP381.189	LP381.189	LP381.188	LP381.189	LP381.188	LP381.189	LP381.189	LP381.188	LP381.188	LP381.188
57794.78	59020.88	51151.73	72345.1	75014.38	72275.29	81250	61374.64	81284.22	66137.82
79979.08	58179.91	73055.57	51249.57	48878.13	43425.65	52254.61	60273.42	59653.79	65414.46
61991.05	58348.37	61750	51179.42	60680.86	56100.73	64923.07	67952.65	48676.24	54741.75
51406.01	54864.6	61540.2	47766.84	59721.54	50811.13	65879.42	74689.57	51008.26	49655.9
48717.58	58912.02	54278.61	47941.64	54613.62	54762.28	69791.36	55537.54	48882.52	44972.59

LP381.188	LP381.188	LP381.189	LP381.188	LP381.188	LP381.188	LP381.189	LP381.188	LP381.188	LP381.188
67730.2	35463.26	44849.43	65607.85	35554.8	65823.51	46148.83	31359.61	38504.78	68522.14
62784.05	43416.42	64609.3	71230.21	39512.61	59566.52	62739.29	28203.88	35978.2	63748.86
52432.5	47864.89	60882.2	56163.38	24307.92	56652.1	52566.65	34983.92	50005.88	67214.69
51951.42	54331.55	62418.3	55024.38	24636.78	47298.51	50603.32	33986.52	48010.77	62423.29
52476.36	46151.21	56791.27	55092.24	24897.44	51327.59	55052.68	32661.41	42644.12	63186.83

LP381.188	LP381.188	LP381.189	LP381.188	LP381.188	LP381.188	LP381.188	LP381.188	LP381.188	LP381.188
49374.32	42188.37	76399.89	59587.61	56664.1	61653.28	69914.75	29241.11	65067.57	65941.31
95454.29	58085.13	81629.39	53601.15	71407.1	54163.75	68695.2	41658	59296.46	85018.69
76801.53	34415.38	72406.9	52859.33	57921.12	62964.33	63563.75	30941.64	84238.52	68811.95
77345.96	45517.7	61896.58	56224.74	59016.12	67793.63	62486.66	30614.92	66675.63	64423.68
75139.2	40258.3	76140.06	48578.2	50252.8	66370.49	61827.48	33127.89	69966.21	66979.3

LP381.188	LP381.188	LP381.188	LP381.188	LP381.189	LP381.189	LP381.188	LP381.188	LP381.188	LP381.189
41671.64	60572.03	61547.23	44309.88	75137.9	66086.57	48433.84	48341.92	50353.24	60725.48
28231.24	55913.77	42088.74	48964.45	83219.25	58015.74	48133.61	65946.44	52348.31	101343.9
38615.77	47448.28	59146.44	53485.8	68124.37	58829.64	60803.29	49689.71	45754.24	56354.06
44213.13	41876.71	54208.78	52223.09	62923.28	58458.81	54306.72	48056.67	48191.27	57084.36
36188.24	43812.32	51797.21	59170.54	65365.44	54063.88	47569.77	47017.2	43762.21	61635.03

LP381.188	LP381.188	LP381.189	LP381.188	LP381.188	LP381.188	LP381.188	LP381.189	LP381.189	LP381.188
40441.58	46016.28	63649.63	58182.85	64890.43	84516.31	38353.99	60348.97	87294.09	35137.62
29701.53	69338.41	68018.48	52915.96	68466.58	64900.63	47113	61930.62	90395.5	34415.85
38327.17	74461.43	62300.56	68398.34	57330.57	69646.55	50901.27	63353.72	73070.57	38153.3
37853.74	70875.12	57730.65	65717.04	57228.31	66627.95	52949.1	64409.74	64988.95	37808.64
30447.55	75449.98	53458.32	61171.73	56421.11	66978.61	41790.79	62523.36	66202.78	39071.07

LP381.188	LP381.189	LP381.188	LP381.189	LP381.188	LP381.188	LP381.188	LP381.283	LP381.283	LP381.283
31987.37	70315.35	48626.33	77988.98	46424.03	57353.12	29503.3	93153.33	127566.4	76782.3
47475.28	57195.02	26145.53	71846.3	46231.05	70060.52	47277.35	115856.3	148534.4	109544.2
44240.23	74830.68	43813.85	65395.33	43740.08	62947.28	38097.56	110700.7	92266.13	81041.09
45462.52	72589.76	46498.22	66301.63	40200.96	59826.7	43609.32	121151	139871.3	93122.83
40814.66	73355.13	41394.08	67001.39	37648.16	57385.4	38721.66	86913.26	137257	157234.2

LP381.283	LP381.283	LP381.283	LP381.283	LP381.283	LP381.283	LP381.283	LP381.283	LP381.283	LP381.283
105830.5	136241	140187.9	94400.29	102556.9	50663.81	85720.4	105635.3	128376.2	315014.1
77146.04	123180.5	110854.8	95572.99	105989.5	69369.53	82859.21	98077.38	116618.6	216788.7
55227.64	128920.6	137699.1	61198.3	82408.88	92958.3	85073.66	109188.3	190569.9	188706.8
52935.68	146608	85523.04	64417.64	119296.3	52402.77	172809.1	139442.9	97703.9	314501.3
58387.1	191969.3	110866	83504.95	110069	52036.22	82862.66	118000.3	99191.62	164449

LP381.283	LP381.283	LP381.283	LP381.283	LP381.283	LP381.283	LP381.283	LP381.283	LP381.283	LP381.282
490363.3	702932.2	1418556	532761	470750.3	1358702	721914.2	882491.5	744930.3	686238.7
548392.2	379879.2	1478961	989972.3	499610.3	1276964	661955.4	750740.5	743192.9	569282.8
501783.5	761877.8	1418752	918176.8	483315.7	1432496	750310.9	873182.3	789359.1	793959
337081	675553	668367.3	671344.5	462312.1	1340051	650860.2	745693.2	754715.2	684005.3
542818.4	753408.1	1323849	974431.6	563850.5	1252011	700507.5	867962.6	775428.4	670919.4

LP381.283	LP381.283	LP381.283	LP381.283	LP381.283	LP381.283	LP381.283	LP381.283	LP381.283	LP381.283
1900594	620258	1104294	720024.7	519051.6	758788.1	655115.5	1602978	696041.8	1339681
835056.2	586203.3	1163890	650110.7	329017.2	846297.7	678259.8	1845646	789662.9	1293636
2111894	618333.6	1177334	576670.2	568916	765106.5	671621.6	1950156	674020.9	1310943
2028471	617800.9	1021717	676254.7	386660.2	798739.8	389836.6	660797.4	679379.4	1356662
2092315	587032.3	958738.4	639100.6	571948.8	780967.3	667521.5	706818.1	625921.9	1328349

LP381.283	LP381.283	LP381.283	LP381.283	LP381.283	LP381.283	LP381.283	LP381.283	LP381.283	LP381.283
980815.8	707906	432063.9	1213210	622234.4	263523.9	822922.8	1832307	796662.2	844700.8
1008860	710342.3	821110.7	1144536	632039	1004552	885967.6	1765763	742130.8	823217.9
1039545	430656.6	783196.5	1233507	666702.9	827984.1	836718.5	1817339	732019.9	872382.6
984130	611439.3	850542.5	1066110	675172.1	763219.3	972812.7	1793468	574673	816355.4
1020763	698272.2	797555.4	1152798	654085	785618.1	570519.3	747678.5	773306.4	760401.3

LP381.283	LP381.283	LP381.283	LP381.284	LP381.283	LP381.283	LP381.283	LP381.283	LP381.283	LP381.283
866556.4	968404.2	1258463	1438094	684122.8	897976.6	953660.2	979743.8	661766.4	1156411
836340.4	1018561	1267972	1586277	735005.2	898619.1	876331.7	947468.9	971058	1248691
662711.3	1032691	1315391	1659182	788185	883935.6	899897.4	957604.4	952170.7	1408091
512401.9	1005671	1251926	1497349	716912.3	832060.9	886598.1	934651.1	768896.8	1266940
844371.1	921066.7	1225357	1463327	792193.9	885260.2	856382	840603.3	976855.2	762791.3

LP381.283	LP381.283	LP381.283	LP381.283	LP381.283	LP381.283	LP381.283	LP381.283	LP381.283	LP381.2837
1321160	818434.2	1137898	946365.9	1162383	727878.6	1115074	358347.3	715984.6	990415.6
1394295	870752.9	1161367	1023025	1181163	761195.4	1137194	635416.4	691024.8	1042616
1432072	907314.9	1202771	1053758	1300016	777817	1112536	661861.2	716745.4	1030339
877154.8	852904.9	1127789	648254	931027.7	810352.3	1152787	622172.4	674550.5	1069904
1409622	811991	1218373	884785.4	1183006	628064.2	1133771	636866.1	684455.9	982156.7

LP381.283	LP381.283	LP381.283	LP381.283	LP381.283	LP381.297	LP381.297	LP381.297	LP381.297	LP381.298
1516201	1728229	612289.3	676127.7	2216010	3968101	2938251	947331.2	2444964	1540271
1075601	1845805	643544.8	635327.3	2247760	4874819	3554214	1496340	1488235	873897.5
1552228	1770478	623396.2	658270.1	418701.4	5146091	3666633	981099.9	2048554	1190669
1468945	1743242	619044.8	634790.9	2024456	5912453	3080373	1228708	1433795	1129356
1486484	1675289	577385.9	692655.7	2161544	6787430	3154628	1375913	2425279	1072500

LP381.297!	LP381.297!	LP381.297(	LP381.297:	LP381.297!	LP381.297	LP381.297	LP381.297	LP381.297!	LP381.297!
1433621	935053.6	939715.1	1888222	878648	705283.9	967780.5	1166166	1369868	755607.8
774207.1	928673.1	1001088	1967832	1055105	1198203	962227.7	899744.5	999685.4	933854.7
927738.8	700865.9	837566	1674829	1499407	758634.2	1040612	962861.6	938960	855443.1
929165.3	920146.2	633449.5	2109082	1210596	1167292	974221.3	894011.3	1258246	1008233
870660.6	990481.6	1169876	1757753	1217443	917717.5	716403.4	906844.5	1080590	824500.7

LP381.297	LP381.298	LP381.297	LP381.297	LP381.297	LP381.297	LP381.297	LP381.298	LP381.298	LP381.297
980177.5	1202669	840840.3	2026066	683371.5	1948953	2126229	1078399	1609128	860757.6
1201473	1154283	993695.7	1908086	883933.8	2040930	2204504	1146310	1746614	985737.5
833169.3	1434013	796148.8	2152264	831018.1	1987877	1412370	1207343	1734749	1191797
1343743	1522843	1043247	3071961	836564.7	1761236	1286142	1050969	2125197	661887.8
1143151	1285362	797170.6	1887771	1048591	2248407	1673732	1269866	1382905	787844.8

LP381.298	LP381.297	LP381.297	LP381.298	LP381.298	LP381.298	LP381.298	LP381.297	LP381.297	LP381.297
863596	2478132	968236.5	1511664	813655.9	766773.4	1428275	1603561	1715664	674106.2
1500830	1670332	699836.7	1451217	1019778	786868.8	2068358	1124310	1933163	689416.3
1133105	1816417	646241.3	1055386	1107756	771374.9	1481904	1822843	1503049	743462
867162.8	1863022	886528	1034920	1327694	660727.5	1768166	2270780	1275273	735973.4
1290353	1948856	693059.9	1250418	943694.3	588098.8	1223903	1666783	1495434	537133.8

LP381.298	LP381.298	LP381.298	LP381.298	LP381.297	LP381.297	LP381.297	LP381.297	LP381.298	LP381.298
1397931	779574.2	1351503	1437757	867098.2	852705.3	1081993	556638.3	543756.3	1321188
1260720	823421.1	979655.5	1172808	1021301	987415.6	1526210	665401.6	756721.6	1163830
1264311	881551.4	1342456	1486307	1064708	1052290	1218442	625428.9	904226.9	1002203
1146770	898547.4	977507.1	1404433	956558.4	641785.2	1627008	727908.3	651602.5	1548469
865623.2	768437.6	968979.1	964296.5	1338284	1009620	1261661	694424.6	752109.2	983560.7

LP381.297[illegible]	LP381.298[illegible]	LP381.297[illegible]	LP381.297[illegible]	LP381.297[illegible]	LP381.298[illegible]	LP381.297[illegible]	LP381.297[illegible]	LP381.297[illegible]	LP381.297[illegible]
1702354	937103.5	1221718	990311.8	1668609	1202095	1326768	833810.6	887924.5	2745723
2005906	665039.4	986292.1	1025871	1048149	1202206	1133772	946738.1	768449.6	2252220
1425010	903073.4	1075058	917082.7	1274024	1729437	1400178	1418543	897537.6	2151215
1809538	639313.4	1176158	1509874	1125026	962877.4	1188565	1187447	780834.4	2149373
1107913	767451.7	804029.5	771055.5	1477114	1651882	1615182	924838.7	789422.7	2183923

LP381.298	LP381.297	LP381.297	LP381.298	LP381.297	LP381.298	LP381.298	LP381.297	LP381.298	LP381.297
663176.7	1330330	1020689	907373.1	925832.2	817125.9	1099273	1036583	521722.6	1741381
586340.3	1374776	845599	548212.7	931339.3	887314.5	1080996	908686.7	609720	1356232
752754.1	1046619	835787.1	620750.2	581937.9	1432861	951114.5	882369.8	589822.2	2092225
701978.7	1313907	817073.2	658756.2	610887.8	790641.4	959774.5	766376.7	643755.5	1494659
838681.6	1084866	714587.8	737616.4	777367.7	911471.2	979465.2	757859.6	552863.6	1720127

LP381.297	LP381.298	LP381.297	LP381.298	LP381.297	LP381.297	LP381.297	LP381.297	LP381.298	LP381.337
1304245	656911.7	709943	724813	509622.2	1196275	912052.4	434972.2	134098.6	1076239
1054525	899107.8	768736.9	727375.6	729828.8	1312835	680141.9	364668	139582.3	1074218
1064805	963945.4	631360.6	642062.1	515195.7	1295079	805026.8	270420.8	145562.1	1075159
1185588	853993.8	522716.2	662240.6	1033158	1191164	568463.6	400651.1	130045.1	993878.8
1539443	1036600	668678.1	680774.7	420730.9	1405239	519693.1	376492.4	134357.1	1059895

LP381.336	LP381.355	LP381.372	LP381.384	LP382.207	LP382.207	LP382.207	LP382.207	LP382.227	LP382.244
624279.1	31719	109756.4	93242.59	116809.7	87872.09	81520.31	75362.42	100069.6	96299.12
601826.5	31888.69	127897.3	95795.45	139024.6	91827.01	76820.15	77450.91	99766.41	133557.7
669284.4	33778.4	116173.3	95554.47	118102	90419.93	78315.01	75506.31	90665.56	100746.8
701329.6	31906.54	110273.8	96124.39	120572.5	89356.22	95860.83	69784.33	81361.88	123793.1
672288.6	29505.56	117727.1	114620.3	124161.1	103437.3	87653.39	72588.72	82377.57	114111.8

LP382.243	LP382.244	LP382.244	LP382.243	LP382.244	LP382.244	LP382.244	LP382.244	LP382.243	LP382.243
118167.3	98138.12	111213.7	82349.33	124242.3	72595.14	71488.63	119952	77309.02	85351.31
182399.9	102397	105997	82524.91	122880.7	67481.85	74329.54	126935.4	78122.15	85395.76
138935.9	121690.1	110307.6	82275.57	110100.2	64890.24	77870.09	138749.6	86172.44	103103
103693.7	85158	101136	79511.66	86189.59	111377.4	73754.39	97954.15	73092.21	85768.86
133822.6	104135.4	130207.7	81371.2	94902.55	65767.75	74581.31	130704.4	75637.1	77742.69

LP382.243	LP382.243	LP382.243	LP382.244	LP382.243	LP382.244	LP382.244	LP382.244	LP382.244	LP382.244
64555.39	75797.04	62626.92	97390.32	88838.17	58925.97	85240.7	92526.78	86503.85	60089.52
65999.06	78007.96	56614.58	89587.54	86063.15	61608.63	79106.84	97028.19	82865.78	63243.73
72494.33	75563.45	58519.91	90189.22	87368.45	60937.34	90223.85	97654.26	90703.25	65980.75
75400.52	78117.18	76641.33	93081.12	77087.88	63038.23	89498.94	97058.52	87521.4	65370.37
67593.92	73411.63	58191.9	95620.73	71124	87853.49	86030.27	102345.3	75698.59	65520.46

LP382.244	LP382.244	LP382.244	LP382.244	LP382.244	LP382.244	LP382.244	LP382.244	LP382.243	LP382.244
90278.48	93770.61	79908.04	64875.91	110909.4	64091.38	86350.41	67920.03	65540.8	73233.7
92120.38	89237.54	77833.16	70409.46	103614.5	61341.52	83352.12	75725.32	63482.53	76732.23
89464.57	100963.7	79518.61	75084.58	105710.7	64025.09	90948.55	74297.63	65914.52	83271.02
90548.03	94779.84	80273.28	71893.64	112193.9	66135.53	86789.49	62938.33	61426.92	77093.19
89210.13	85684.42	71874.8	65978.67	115160.2	66474.16	93634.59	70533.55	66881.8	77564.56

LP382.244	LP382.244	LP382.243	LP382.244	LP382.244	LP382.286	LP382.286	LP382.286	LP382.285	LP382.285
46076.7	81599.39	102141	79826.85	72494.62	144914.7	200765.9	218512.3	165595.5	202027
55161.17	69216.04	105086.6	83399.21	74436.18	161210.9	221527.6	219426.5	153339.2	196570.6
57409.2	68693.58	102981.1	82784.39	78369.85	145666.1	232471.7	225919.5	148463	196429.9
49620.06	69739.82	100147	87409.4	70919.91	163596.8	192586.7	221368.9	146030.2	202165.4
50272.67	76627.68	101735.8	89708.43	74513.56	148855.8	196848.1	199417.6	169885.9	190464.8

LP382.286	LP382.285	LP382.285	LP382.285	LP382.285	LP382.286	LP382.286	LP382.285	LP382.286	LP382.285
187298.2	200027.7	204267	159639.3	205595.4	188663.7	207968.5	298388.6	152178.6	189171.6
186611.9	179873.1	214830.7	159183.1	204463.3	177675.3	217610.8	301600.8	155661.6	164248
179670.2	185941.4	208573	167429.5	221388.8	194934.7	231759.1	298674.5	146665.8	203473.9
194626.4	189840.9	209527.5	184885.5	209771.5	179305.6	194391.6	287131.9	151761.4	169999.5
184639.4	188036.2	188911.1	150755.5	182453.9	163649.1	197840.1	287990.5	142926.5	198994.8

LP382.286	LP382.286	LP382.285	LP382.286	LP382.286	LP382.285	LP382.301	LP382.301	LP382.301	LP382.301
207979.7	320696.3	116235.1	180847.7	168819.3	114742.7	824360.8	286640.4	553048.9	638452.5
213341.2	337332.4	128620.4	190462.4	188837.2	135429.5	775952	354608.9	537942.3	549424.9
210916.2	350437	131451.2	204370.4	181193.5	135292.6	831201.7	401456	818241.6	615719.5
200968.7	334371.9	124482.2	187996.7	171421.2	135356.3	747159.2	448407.3	439250.3	402602.7
206654.7	321633.4	121549.8	181026.8	155269.5	133305.1	761539.3	368582	650404.7	578347.7

LP382.301	LP382.301	LP382.301	LP382.301	LP382.301	LP382.301	LP382.301	LP382.301	LP382.301	LP382.301
214235.7	212829.9	347083	245961.4	373073.8	393161	158854.1	322573.1	429612.3	428826.4
211234.4	266789.1	471480.2	247131	279883.7	344453	173832.9	331520.7	413135.2	280411.8
202519.1	211434.9	413064	280820	406206.6	323300.4	148561.3	245575.1	336229.5	280013.7
234990	258978.8	366712.1	223412.4	396002.6	270662.8	154756.9	268846.5	393277.8	270278.1
263765.2	198191.1	411265.4	224839.8	354675.1	270808.1	149883.9	262370.7	317456.3	280399.8

LP382.301	LP382.301	LP382.301	LP382.301	LP382.301	LP382.301	LP382.301	LP382.301	LP382.301	LP382.301
172171.2	196361.7	292544.8	242225.2	464312.6	217449.3	207775.2	238358.2	246910.6	245864
181224.5	185675.8	216708	258970.1	409129.8	250929.3	314616.5	237199.8	255922.4	201721.3
137306.3	259334.5	277681.6	259453.6	315018.3	235184.2	240374.2	262181.6	191979.8	251831.4
206005	251032.8	306855	263557.7	311368.2	249292.5	213588.6	198011.2	205682.7	242215.1
165272.9	232436.5	233988.6	231926.3	246820	220470.7	208083.5	248451.5	267751.5	234014.1

LP382.301[illegible]	LP382.301[illegible]	LP382.301[illegible]	LP382.301[illegible]	LP382.301[illegible]	LP382.301[illegible]	LP382.301[illegible]	LP382.301[illegible]	LP382.301[illegible]	LP382.301[illegible]
271712.6	246500.2	170443.4	341426.1	187904.2	314841.9	209725.7	294332.1	185808.2	323148.7
152757.1	262887.9	176044.4	335620	200624.7	249686.9	209950.7	304840.1	192860.9	264799.9
183132.5	265637.5	191847.3	368688.3	227125.2	238913	215617.9	249979.4	278696.9	327322.3
168956.2	240162.2	178581.3	338543.4	199253.4	236866.4	217536.7	331595.2	184221.9	324302.5
162569	232471	162740.2	294004.7	193459.1	228978.1	215368.2	311159.5	184374.3	291663.5

LP382.301	LP382.301	LP382.301	LP382.301	LP382.301	LP382.301	LP382.301	LP382.301	LP382.301	LP382.301
209407.4	276333.4	190962.1	304795.5	149697	257109.8	152527.5	134062.5	191087	183357
211482.1	256580.3	202607.2	212419.6	229256.9	266355.4	135765.9	185894.7	194862.2	187833.9
274188.9	399933.7	205207	217422.7	155846.1	267722.6	130476.1	159556.8	200460.5	186347.5
194936.8	264361.6	216060.2	190208.1	146448.5	248200.4	135287.5	135675.5	202147.4	173462.5
199268	244816.7	183924.7	197304.6	151629.7	256259.9	145668.7	126082.8	200863.1	183232.8

LP382.301	LP382.301	LP382.301	LP382.301	LP382.301	LP382.301	LP382.301	LP382.301	LP382.301	LP382.301
190530.6	137160.8	168480.2	207218.6	218891.7	141997.2	217512.2	180579.8	125945.2	241687.3
209213.7	150143.4	213453	218176	198369.1	142574.6	227653	167995.8	137878	236638.2
270681.7	154182.5	225868.5	219033.2	215693.2	141928	256775.9	165626.1	131343.5	233607.8
197391.4	166613.4	215536.9	195598.1	190108.1	137672.4	210914	164382.9	139573.3	226752.5
180609.9	145740	211574.9	184393.1	192902.3	146479	193583.3	173695.3	126308.2	216726.2

LP382.301	LP382.301	LP382.301	LP382.301	LP382.301	LP382.301	LP382.301	LP382.301	LP382.301	LP382.367
185811.4	181080.1	170456.1	196597.1	140831.6	147693.4	160971.1	189973	150993.7	22542.16
156432.4	186193.9	179440.8	197659.3	151150.3	136863.7	163721.7	184939.5	138613.5	24971.03
147503	170076.7	168008.1	211611.2	144152	140629.3	160644.5	192348.6	145832.1	20261.5
146674.4	179588.3	169711.5	189487.5	142117.6	128949.5	158650.7	188689.3	138537	14553.32
145719.6	180239.5	165916.2	176846.3	143381	139713.7	157432.4	189675.8	123325.4	20086.32

LP382.368	LP382.379	LP382.404	LP382.404	LP382.404	LP383.167	LP383.167	LP383.167	LP383.168	LP383.168
24834.74	63773.31	214666.1	12795.19	7269.581	176721	59042.96	69984	68954.87	68783.76
23520.51	56547.26	248137.6	13364.54	6542.789	167781.6	64788.41	66332.93	38356.88	37184.73
37714.41	55281.26	400416.3	20674.18	10745.9	163257.7	58094.25	63108.21	111874.6	38055.94
38465.74	66744.43	490252.2	25147.19	10659.43	175417.4	55879.76	61986.98	47381.45	31137.19
46500.32	57274.83	541450.8	26947.66	12919.94	180108.2	56426.86	67805.06	125673.4	68788.21

LP383.168	LP383.168	LP383.167	LP383.167	LP383.168	LP383.168	LP383.167	LP383.168	LP383.168	LP383.168
73618.88	50318.39	96163.84	50468.27	181035.4	32911.77	254821.3	111733.9	332656.1	252116.4
40021.95	56154.07	39125.79	23434.73	202889.4	71910.13	299309.5	366273.7	328172.1	233300.1
63309.4	63771.18	66010.08	48411.54	73799.15	32467.07	290258.6	355677.2	140160.5	61185.46
60681.49	207570.5	93776.34	24372.84	191725.9	62465.47	291148.6	67311.94	392425.7	249113.8
61558.22	202824.4	106703.2	44622.58	202262.6	65330.35	319106.4	377136	179891.1	272543.3

LP383.168	LP383.168	LP383.168	LP383.168	LP383.167	LP383.168	LP383.168	LP383.168	LP383.168	LP383.168
323320	396701.5	81589.62	377002.7	62658.16	426426.4	55811.21	95530.71	42973.51	50308.42
312245.3	405594.7	373429.2	337019.1	123723.4	437116.9	247008	61178.68	94853.19	172773.5
352338.6	88518.97	92563.17	56350.03	127144.3	418243	71017.41	259526.4	95693.3	182061.6
330652.8	407835.9	360415.3	359012.4	117815.3	438657.5	290659.6	255487.5	83638.44	177249.9
303388.4	406147.9	364723.9	345418.4	129849.1	102729.9	277280.2	259652.7	93230.01	173016

LP383.168	LP383.168	LP383.168	LP383.168	LP383.168	LP383.168	LP383.168	LP383.167	LP383.168	LP383.1687
199634	352435.6	366779.7	290688.1	194617	203951	283902.9	137563.5	161359.6	292740.8
220983.3	402721.3	67481.63	326008.8	199427.6	204693	283947.5	135331.6	49925.94	304042.1
213617.8	130471.5	387372.5	304718.7	204551.4	204656.9	286107.1	146823.7	186569.2	293857
212886.8	402562.2	381395.4	289630.8	87340.9	199549.7	102060.2	139882.6	156779.1	103922.3
216943.5	434737.1	412367.9	306316.1	220882.8	199018.7	312072.4	142638.9	163128.2	302354.1

LP383.168	LP383.168	LP383.168	LP383.168	LP383.168	LP383.168	LP383.168	LP383.168	LP383.167	LP383.168
59140.97	51059.11	184691.3	166712.6	72467.19	187901.7	264354.7	204371.6	171199.5	198011.5
266235	166205.7	178783.7	177466.1	36121.27	181177.5	248597.6	196912	165060.1	217009.6
259238.8	161715.3	186197.7	171801.5	72102.1	184519.4	242092.8	212239.7	166139.4	208791.6
248903.2	164684.2	182285.8	170069.8	66219.8	191880.2	254022.8	212611.9	166210	207020.3
255466.4	155386.4	190826.2	180828.7	73251.03	211417.9	242540	215077	168982.2	198905.1

LP383.168	LP383.168	LP383.168	LP383.168	LP383.168	LP383.167	LP383.168	LP383.168	LP383.168	LP383.168
258761.3	119959.4	259214.8	218377.4	210967.9	226849.5	521404.9	85969.83	342929.6	279958.9
257019.9	125828.1	258950	215296.4	216969.2	58175.58	519652.4	490643.7	345144.9	273084.9
272681	140827.5	244819.8	215748.9	220499.9	242945.2	520489.3	460676	343358.8	285961.9
258161.2	123450.7	203658.6	210482.8	76160.32	242079.3	516713.3	493656.6	318911.5	275329.1
253079.1	145656.9	226205.3	207230	231655.6	240270.9	537951.3	447641.1	336325	289673.5

LP383.168	LP383.168	LP383.168	LP383.168	LP383.204	LP383.204	LP383.204	LP383.204	LP383.203	LP383.204
266310.3	33943.37	43536.88	59636.63	326730.6	285086	329352.3	383805.4	283536.4	554148.8
303130.7	60943.54	41666.98	53759.31	300393.6	546585.2	428024.8	468329.4	194247.7	524923.7
273732.6	35437.8	53928.11	32104.23	348352.3	431932.4	325052.2	554740.3	291519.9	245717.2
264163.6	38009.84	111725.7	52414.68	418412.5	504265.7	453122.4	477477.6	349314.6	353763.1
247893.3	65770.36	108392.6	49614.13	304570.7	340137.5	255148.6	324652.5	262047.6	353206.6

LP383.204	LP383.204	LP383.204	LP383.204	LP383.204	LP383.204	LP383.204	LP383.204	LP383.204	LP383.204
192183.6	514188.5	516729.5	287655.5	383281.1	422474.2	519045.9	280759.2	343662.9	622379.2
284278.1	475042	381759.3	328032.9	483410.8	200506.6	446292.7	279859.9	415594.7	486044.7
219685	356795.9	399582.7	362616.7	398984.2	247233.9	453736	257403.9	469105.9	423917.7
233341.2	432811.1	363897.5	307151	429523.4	221624.1	524008.4	334763.9	400006	388606.2
191932.2	411081.6	536986.9	471977.2	361081.1	219026.3	452902.3	362627.8	324034.6	349007.6

LP383.204	LP383.204	LP383.204	LP383.204	LP383.204	LP383.204	LP383.204	LP383.204	LP383.204	LP383.204
330963.3	249362.2	290698.5	368195.3	390470.8	342068.3	356276.3	351769.6	464556.8	262286.7
405077.8	213450.8	199364.2	405554.3	315151.3	376556.8	398463.2	278443.2	413548.2	252451.7
308286.2	314641	306211.6	517210.8	334416.3	371487.8	623364.7	315279.4	447357	363343.2
447528.4	189126	331811	294873.4	367257.2	304736.1	332258.2	291170.4	386212.9	315929.6
328607.8	213207.9	260576.5	439824.7	398500.4	282820.2	435254.5	344991.6	613433.4	280389.8

LP383.204	LP383.204	LP383.204	LP383.204	LP383.204	LP383.204	LP383.204	LP383.204	LP383.204	LP383.204
451758.9	568452.2	502486.2	282134.7	209554.8	243631.4	345463.7	491909.1	278614.6	313977.8
394757.9	504205	417448.3	492860.1	156268.8	176869.2	300682.9	328798.8	276341	237703.9
440951.3	326832.7	569584.9	480343.8	332861.3	190588.1	353740	457238.3	288512.9	243944.8
259858.9	625053.4	364806.6	387837.9	238375	233454.5	432372	389108.8	260207	211442.9
325060.3	473866.7	463795	392111.4	262094.3	140573.1	338649.7	428672.5	221971	301605.1

LP383.204	LP383.204	LP383.204	LP383.204	LP383.204	LP383.204	LP383.204	LP383.204	LP383.204	LP383.204
489730.6	432988.9	201274.3	168538.7	270833.6	299126.8	412287.6	230592.4	510751.3	161508
421675.4	441987.4	212407.1	308459	195847.1	443327.7	359850.1	425594.3	340263	176018.1
436268.6	473521.3	173496.1	203696.6	207275.1	404886.1	277976.2	250389.2	438682.8	160278.5
588694.4	437293.6	304478	200031.2	209333.1	389808.6	339302.6	209163.8	489144.3	173281.9
435628.7	426331	190723	204746.6	222413.6	383191.8	369562.2	191260.6	446936	142936.7

LP383.204	LP383.204	LP383.204	LP383.204	LP383.204	LP383.204	LP383.204	LP383.204	LP383.204	LP383.204
510827.2	440749.9	446178.1	334432.7	105168.2	414831.7	341110.1	213522.4	229056.9	77267.39
500619	359404.6	412513.3	428007.5	110427.7	413608.3	317137.5	254495.7	424731.8	53199.12
379276.8	334736.2	400859.2	297128	110339.9	458268.7	463916.7	216689.2	319963.7	62308.29
415984.5	359358.9	510587.8	413318.8	81990.16	475701.9	302083.3	310180.2	274290.2	44264.17
368280.5	405866	475198.5	344432	125190.9	480501.7	292164.7	270265.7	325205.9	54727.64

LP383.204	LP383.204	LP383.204	LP383.204	LP383.204	LP383.204	LP383.204	LP383.204	LP383.204	LP383.204
219287.8	187657.8	528111.7	248410.6	118749.4	89206.51	403873.3	92181.25	67082.07	371575.3
297584.1	186362.9	413011.2	172012.1	118275.1	130284.3	443402	112854.1	67962.96	255452.9
242328.3	213165	366058.9	168643.3	97669.19	85436.93	419778.9	98870.75	47550.71	269798.7
276868.3	213716.1	434650	193873.7	104246	103484.4	506397	95581.66	50278.46	189836.9
322018.6	230957.3	619360.8	182426.8	107181.9	74767.85	411501.4	90445.07	85190.59	184664.3

LP383.204	LP383.204	LP383.204	LP383.204	LP383.204	LP383.204	LP383.204	LP383.204	LP383.204	LP383.204
246657.4	212584	210281.1	236067.5	129829.5	166839.7	224030.1	377076.2	73012.72	63216.78
262955.7	194792.4	353820.5	286313.5	136531.6	256313.8	234134	303040	105602.4	61995.99
275841.3	197605.2	179777.4	191868	142670.5	318446.5	241573.9	373589.4	107883.8	59974.74
241428.8	212024.5	235368.3	236038.9	193558.5	211132.1	158809.2	372498.7	91566.24	61169.82
345252	192612	218960.3	255414.8	115260.8	202107.2	245408.3	366213	84280.18	76927.17

LP383.204	LP383.204	LP383.204	LP383.204	LP383.204	LP383.283	LP383.303	LP383.316	LP383.315	LP383.316
115105.2	487750.3	226844.5	90640.46	64991.31	70043.18	85618.92	92524.01	64596.12	57530.78
112366.3	445280.9	216207.1	81589.19	70491.51	75881.03	79576.73	132451.9	71285.76	50171.6
101940.9	382684.4	210530.7	60067.4	63213.19	69400.69	87518.66	145137.5	71693.12	51878.85
82624.88	425851.3	203978.8	84920.15	64513.62	61412.07	82301.04	151625.4	72072.59	51694.11
112718.5	513446.4	197653.7	116692.4	64780.46	64974.31	89236.99	149101.5	71096.25	53098.29

LP383.316	LP383.316	LP383.316	LP383.316	LP383.316	LP383.331	LP383.331	LP383.331	LP383.331	LP383.352
56710.04	46382.7	48009.77	52260.21	51297.64	94783.37	104663.2	67737.08	85454.37	56307.36
48483.71	49277.87	47319.72	60835.29	51582.57	72400.58	96476.56	59044.49	83086.84	52991.86
48277.48	45657.32	54619.42	49085.28	56898.09	85338.03	107735.8	67353.77	84724.74	51395.79
51107.3	54254.33	46000.37	50125.54	48922.03	92538.75	106616.6	64442.08	81830.74	50610.99
48585.05	45621.52	51923.39	51866.93	47871.06	84990.81	100358.1	66292.52	81814.87	48626.06

LP383.352	LP383.371	LP383.399	LP383.400	LP383.409	LP384.207	LP384.238	LP384.306	LP384.320	LP384.326
88992.71	43203.79	1132739	45304.78	17261.56	77911.83	19290.48	88520.62	42719.48	21846.12
81213.88	43309.88	1086939	39126.74	22816.61	77486.21	18983.4	94383.85	41436.08	22528.41
84607.29	44532.99	1032994	40252.86	17854.42	93516.88	17449.94	90054.68	40445.94	23675.31
91078.11	40494.6	1017970	43081.1	17688.48	79204.75	19577.98	80024.5	37123.17	23788.26
116346.5	43363.82	910329.9	35460.4	18414.73	76243.9	17520.6	93238.65	42001.74	23207.21

LP384.335	LP384.384	LP384.383	LP384.383	LP384.383	LP385.183	LP385.183	LP385.183	LP385.183	LP385.183
43501.13	35852.62	64545.79	28149.33	31789.79	183520.8	167926.4	217740.5	240475	262949.8
34354.24	36100.87	66261.35	32422.86	36664.51	145687.5	268881.1	265911.8	304320.7	228252.8
39600.62	42145.15	66976.44	31097.63	33513.45	211919	242963.6	142259.3	313393.2	212042.7
34015.95	49468.66	66364.73	32516.15	29966.07	228553.8	252600	256469.2	210016.2	178067.3
33408.18	44221.99	61577.87	32917.69	37995.95	299766.9	204495.9	224135.8	293199	215102.4

LP385.183	LP385.183	LP385.183	LP385.183	LP385.183	LP385.183	LP385.183	LP385.183	LP385.183	LP385.183
210998.8	227197.2	212407.2	215329.9	290237.3	156561.2	275264.9	306385.5	255946.5	258505.6
193133.8	236088	187703.4	212481.6	267959.5	243801.2	219304.7	243035.1	208443.2	306594.5
152003.1	485785.6	144757.2	298210.3	192115.6	219891.6	246789.7	244938.2	222932	220311.1
194576	253378.3	297979.2	230309.3	282439.6	179478.6	204547.5	240490.3	225206.6	264613.6
173023.5	245549.2	170941.1	186883.6	229040	183426.8	197688.8	284497.9	223794.6	216238.9

LP385.183[illegible]	LP385.183[illegible]	LP385.183[illegible]	LP385.183[illegible]	LP385.183[illegible]	LP385.183[illegible]	LP385.184[illegible]	LP385.183[illegible]	LP385.183[illegible]	LP385.183[illegible]
264138.4	161924.5	159821.7	270705.7	239130.4	184019.6	292467.4	308564.7	216550	335424.3
206797.6	169649	204314	313948.3	230628.5	221361.9	200236.9	322889.5	307869.4	241998.8
241021.6	195176.4	188324.4	232556.1	240737.4	156001.3	219920.6	280524.7	234158.7	276715.1
173830.1	214305.8	187689	256987.2	207524.5	164121	262197.2	364960.9	231854.2	353131.9
158962.7	236867.4	184922.8	282871.4	209341.2	226844	263841.3	297341.3	200107.5	185075.9

LP385.183	LP385.183	LP385.183	LP385.183	LP385.184	LP385.183	LP385.184	LP385.183	LP385.183	LP385.183
219173.9	224987.1	355058.5	275854.7	180711.5	266680	329765.8	240792	277872.9	95193.56
307286	184265.8	205263	230035.1	388952.4	232256.2	219212.3	208044.4	255141.7	102999.1
329674.5	219292.1	162782.4	212789.1	239708.6	324616.9	304480.4	157051.9	240311.2	108216.9
208169.9	167724.5	388080.4	214327.1	199740.7	247079.8	264203.8	248216.2	246063.3	91299.97
219022.6	216309.9	219324	185533.1	229780.9	237503.3	296105.1	192032.6	212271.1	107248.6

LP385.183	LP385.183	LP385.183	LP385.183	LP385.183	LP385.183	LP385.183	LP385.183	LP385.183	LP385.183
205504.5	181760.7	164108.5	212726.6	294333	161562.6	256785.1	261187	188114.4	188029.6
221767.3	218229.4	167202	171428.3	330470.1	188210.9	340205.5	246809.4	159443.5	191865.6
225385.1	221182.8	188052.8	175098.1	256441.2	141194.4	226507.8	207369	183844.7	230335.4
209054.1	198179.7	263627.7	172282.6	234405.3	201719.2	291726.5	214535.8	144563.8	192415.8
261832.3	190579.9	181063.4	181556.2	221786.3	198871.4	179172.3	184257.8	210760.7	207310.9

LP385.183	LP385.183	LP385.183	LP385.183	LP385.183	LP385.184	LP385.184	LP385.183	LP385.184	LP385.183
178927.2	143474.5	158757.2	245306.9	157625	254393.9	274989.4	225811.5	202145.3	163309.6
176632.7	172977.2	183718.5	200884.4	274005	316954.1	219734.3	300478.4	201219.2	209580.4
282622.4	152853.4	206028.1	242559.7	167960	281618.6	268428.5	176612.8	272366.7	257085.7
251459.1	142468.7	157658.2	259401.5	232212	330649.9	247051.6	257012.5	247782.7	218149.7
188079.6	145469.6	156902.7	192250.5	173506.8	259699.4	247266.3	228315	290550.1	242561.7

LP385.183[illegible]	LP385.184[illegible]	LP385.183[illegible]	LP385.184[illegible]	LP385.183[illegible]	LP385.183[illegible]	LP385.183[illegible]	LP385.183[illegible]	LP385.183[illegible]	LP385.183[illegible]
249438.1	226264.7	173701.5	229343	165930.3	186084.7	144680.4	150332.5	210705.9	182884.1
176125.9	184328.1	164650.4	172454.1	121439.3	249915.8	156676.8	115078.3	235099	149474.6
175175.5	186149.5	188968.6	166909.3	122399.6	196717.5	164808	140210.1	227284.5	166226
223448	190355.1	199822.7	151487	106401.2	217609.7	177165.7	165153.2	187747.9	153124.9
150341	194842.2	235514.2	250026.1	109058.7	175343	170526.5	201153.5	252215.6	162272.8

LP385.183	LP385.183	LP385.183	LP385.183	LP385.183	LP385.183	LP385.184	LP385.183	LP385.183	LP385.183
205763.8	106605.9	196909	164280	73602.39	126645	71157.28	174694.6	186566.4	86528.45
224626.4	110512.4	208673.4	186446.2	60964.54	161651.5	77411.3	178258.3	198541.2	86459.17
229816.6	103606.6	210620.3	151231.6	85893.78	158884.2	70591.77	167783.8	152104.1	87211.17
208003.9	102218.8	212058.7	163362.9	89442.49	158994.4	70058.28	140667.5	139196.2	72692.09
195832.3	109286.5	223537.6	177572	82791.56	186079.3	72100.72	158695.2	174334.5	89521.34

LP385.183	LP385.183	LP385.183	LP385.183	LP385.183	LP385.183	LP385.208	LP385.221	LP385.221	LP385.221
70620.54	114249.9	66191.49	102660.9	87092.59	135926.9	156992.3	75827.65	190421.1	251954.5
76851.38	117933.4	108520.2	101769.1	90530.23	119040.5	125962	32040.04	67855.3	258440.8
70656.8	98701.29	80968.13	74657.32	89805.2	110021.5	139284.6	73257.56	182581.8	254528.3
73660.93	101896.4	85675.55	78491.07	79461.64	103885.3	136322.8	67808.74	106532.2	244309.9
74120.46	104444.9	85198.43	76438.46	83366.1	111345.5	143814.3	66965.41	191546.4	255449.7

LP385.221	LP385.221	LP385.221	LP385.221	LP385.221	LP385.221	LP385.221	LP385.221	LP385.221	LP385.220
46353.83	64267.3	139759.9	242082.5	213710.4	164062.6	202359	186538.8	95612.47	56230.31
24556.37	64164.98	46784.81	237230.7	188898.5	161823.8	210875.7	188889.8	102871.9	168778.1
43921.25	67156.24	130995.1	247482.6	205070.6	155683.5	195612.9	187016.5	89703.26	144861.2
37228.4	58712.2	127488.5	106627.4	66677.18	157319	200139	176039.8	93656.97	160972.3
40655.63	64368.48	135907.4	260996.3	215578.9	147381.3	196334	179499.2	103423.7	158197.5

LP385.221	LP385.221	LP385.221	LP385.221	LP385.221	LP385.221	LP385.221	LP385.221	LP385.221	LP385.221
200710.8	140480.4	56649.36	228482.4	93629.38	187502.6	71871.94	195007.9	75101.87	261672
212619.2	57777.33	134373.9	238119.5	93920.56	71789.3	30806.53	185792.4	69362.58	241201.7
200767.2	146878.5	119259	245314.9	87304.25	188639.4	64081.12	193158.2	68833.09	262542.5
220853.3	141189.4	118393.8	255646.2	79330.21	183830.3	53770.82	205554.8	63430.3	241868.1
199298.4	147545.7	127563	274607.2	91181.93	182849.2	64555.25	214328.3	65771.36	248209.4

LP385.220	LP385.221	LP385.221	LP385.221	LP385.221	LP385.221	LP385.221	LP385.221	LP385.221	LP385.221
164738.7	102217.9	122575.9	195193.5	150518.1	100582.2	117383.3	118611.9	258556.6	192457.7
157162	107749.5	129258.9	213131.6	144487.9	106543.7	115882.8	125928.2	246409.5	182502.8
170165.9	95386.25	119375	188806.5	157284.5	91290.54	106719.7	95608.65	261114.4	180301.2
162854.6	99209.79	124446.7	186770.8	150112.5	88861.24	92947.46	98059.64	260326.2	177918.9
184477.1	101398.7	126922.2	188297.7	144399.8	96755.73	108575	105166.3	274916.1	194056.2

LP385.221	LP385.221	LP385.221	LP385.221	LP385.221	LP385.221	LP385.221	LP385.221	LP385.221	LP385.221
201353.2	220191.8	159873.8	144503.3	258329.1	201187.3	185768.7	118641.6	107321.1	221329.4
209897.7	233757	159085.1	145607.6	250513.3	173598.7	186291.7	129955.2	123214.8	230578.8
188709.3	229487.7	155805	147007.8	233718.8	175365.1	188009.6	129702.8	108393.5	201780.4
201550.2	241834.4	162094.7	144440.5	249016.2	179628.5	182181.7	119719.2	110019.6	217774.7
206584.7	232522	167698.8	140523.6	248130.5	179018.8	178925.9	140647.7	102201.4	209431.3

LP385.222	LP385.221	LP385.221	LP385.221	LP385.221	LP385.221	LP385.221	LP385.221	LP385.221	LP385.221
158837	163854.2	160022.7	135891.9	240897.6	164695.7	204295.3	213878.3	80886.32	234708
154777.3	200293.2	60013.62	152188.5	235514.3	169543.9	212435.6	195363.9	89818.51	224998.1
150913.8	180468	151983	135528.3	229815.5	156979.6	201081.4	196877.7	84073.59	232888.1
151014.3	173130.9	139662.1	140443.5	216764.9	172509.4	209821.2	210673.7	79748.37	240843.8
134682.2	174843.6	153165.2	139418.7	229256.9	182289.8	215004.9	184997.3	87004.82	106482

LP385.221	LP385.257	LP385.258	LP385.350	LP385.350	LP385.350	LP385.350	LP385.415	LP385.415	LP385.415
171806.4	26647.42	24439.19	17186.17	28742.01	33716.04	30264.53	32071.49	5355.077	23578.71
169986.6	22821.41	25276.34	25866.73	26777.43	54956.78	30158.71	48004.64	6859.284	28788.73
165298	24184.45	16146.91	31519.68	28200.71	56596.57	34779.69	55386.3	6673.967	29885.96
150869.5	23987.65	17972.41	34461.95	23158.69	60978.79	35551.83	62265.65	9440.574	35496.71
159134.1	27412.52	25523.05	29001.26	25015.05	48189.54	29049.92	53106.13	11710.99	30190.92

LP385.415	LP385.414	LP386.166	LP386.326	LP386.363	LP386.363	LP386.363	LP386.363	LP386.363	LP386.419
18256.41	17380.78	16660.16	100170.3	26219.22	33246.48	24815.1	26284.64	46398.67	10200.92
18441.94	21198.69	15780.34	106338.4	27845.71	35189.1	26598.51	21388.56	50527.38	10500.98
26673.47	20953.15	16074.88	109831.5	23798.87	33581.86	25123.36	20279.16	48273.86	8994.663
22004.25	19975.72	12052.48	104634.3	26346.36	27297	24123.5	23691.95	43534.18	9212.873
28966.01	25724.2	14623.36	103330	26157.99	32204.2	26572.01	24067.42	48198.52	11454.3

LP387.199	LP387.199	LP387.199	LP387.199	LP387.199	LP387.199	LP387.199	LP387.199	LP387.199	LP387.199
199606.9	106272	118834	127939.2	190797.2	160241.7	155207.6	143364.7	151692.9	139860.5
189464.1	145793.8	160475.5	110651.7	144159.3	165188.5	159184.1	172463.1	217338.4	151694.4
170586	145227.3	148723.6	125280.8	155969.3	166077.2	124991.9	164159.1	164944.7	145125.9
94611.38	102200.9	136897.5	133423.5	241900.9	142959.4	148629.4	150542.5	177830.1	193857.6
154905.9	160039.9	150864.9	107834.7	106561.6	134215.7	152161.1	193330.2	154419.8	186067.1

LP387.199	LP387.199	LP387.199	LP387.199	LP387.199	LP387.199	LP387.199	LP387.199	LP387.199	LP387.199
133112.3	178006.6	73849.87	183912.7	160527.6	134191.1	123566.5	137877.2	123638.8	70756.2
136381.6	178914.1	90122.81	142693.1	140322.2	191178.1	197452.9	107826.8	197553.3	70871.71
102522.4	231585.4	87357.16	98770.89	158523	149873.8	134545.9	101688.7	114472.2	116458.8
107632.8	135809	69247.43	115672.7	170716.8	151576.8	171878.4	109565.1	178833.3	103777.8
108951.6	226655.7	86129.4	99909.71	178450.2	136740.2	155902	123429	141669.2	68760.37

LP387.199	LP387.199	LP387.199	LP387.199	LP387.199	LP387.199	LP387.199	LP387.199	LP387.199	LP387.199
202245.9	184965.7	108466.5	164279.2	120752.5	115507.6	125840.5	121571.7	104808.4	110164
189821.7	137878.9	159691.2	162692	121928.8	127158	158183.5	113042	133950.6	130927.3
167353.1	172663.7	131901.3	160348.7	170826.2	162356.5	115433.3	114514.5	130577.6	107479.2
141099	182355.2	129953.9	190564.2	126672	133852.7	140132	118737	121957.2	148393.5
119457	166644.4	121784.8	115351.9	177505.7	117695.8	109045.9	130606.5	96667.36	112998.6

LP387.199	LP387.199	LP387.199	LP387.199	LP387.199	LP387.199	LP387.199	LP387.199	LP387.199	LP387.199
191526.6	191891	164234.5	130061.5	186364.2	191898.9	106696.3	50844.86	134572.9	107488.5
178806.7	149150.5	189984.5	123943.1	132335.8	173770.7	143006.5	51452.69	135441.9	93466.67
158807.7	186448.1	188599.7	133320.1	144053.5	121593.4	108312.3	51753.47	141605.2	93549.52
190664.1	172421.3	181866.4	116582.7	187616.2	128826.2	98824.75	53034.6	112693.4	87399.28
140866.1	191879.6	242494	145366.2	156453.8	157562.3	82923.5	55989.3	195419.7	95772.23

LP387.199	LP387.199	LP387.199	LP387.199	LP387.199	LP387.199	LP387.199	LP387.199	LP387.199	LP387.199
110232.3	193074.6	89646.96	156873.7	122579	136143.1	144844.8	100295.1	138208.5	46219.02
167106.4	146476.6	96325.53	152936.7	112471.9	130446.1	96780.85	135900.9	138466.8	38840.1
117073.8	142074	145327.2	191745.4	88728.8	100618.5	97929.97	89419.91	138226.6	46857.38
114653.4	170069.4	89011.38	173864.1	117619.2	98447.8	132159.4	109083.9	132567.8	40653.73
121935.2	146728.7	92533.59	131561.5	158890.4	115437.2	93211.46	135941	148545.5	44120.23

LP387.199	LP387.199	LP387.199	LP387.199	LP387.199	LP387.199	LP387.199	LP387.199	LP387.199	LP387.199
89643.52	130661.5	145369.5	159119.1	99926.2	46279.7	98573.83	144258.8	110753.2	144156.6
112498.8	110353.8	213403.7	114464.2	102775.3	41780.85	99777.61	234877.6	140068	138244.7
119407.3	160192.4	192555.1	138432.6	105968.5	51141.3	121668.1	175359.7	108887.4	110752.1
104909.6	153560.8	164715.2	85504.45	102761.4	50012.07	89235.79	147604.1	111350.8	127126.9
80067.8	172846.2	160882	104467.9	110310.7	49494.55	80231.15	129447.2	118998.5	140990.5

LP387.199	LP387.199	LP387.199	LP387.199	LP387.199	LP387.199	LP387.199	LP387.199	LP387.199	LP387.199
146851.7	147787.2	158954.4	123927.1	59969.96	129116.5	30839.59	145231.3	134834.9	138056.9
88617.52	135649.4	143550.4	121410	62410.84	139925.6	38145.5	143865.4	121228.5	110562
96037.63	144999.9	177505.8	99418.61	81408.42	122821.8	43433.96	147451.1	129831.8	177972.2
101120.6	168424.5	164817.9	77891.96	58482.23	147896.4	47062.14	151078.9	111219.8	129881.1
96125.52	123956.5	126831.8	94520.37	60414.31	146377.3	30654.53	117028.4	139275.6	136761.6

LP387.199	LP387.199	LP387.199	LP387.199	LP387.199	LP387.199	LP387.199	LP387.199	LP387.199	LP387.199
36722.91	88833.69	161524.6	118682	146612.5	45855.01	45871.9	44223.82	75656.17	50427.37
35934.53	106617.7	106320.8	132821.6	111850.5	55445.22	39725.35	36130.73	88352.27	62010.67
44107.68	102603.3	138539.4	151092.8	102134.9	40380.85	37043.24	39303.92	75660.05	47153.75
43990.55	95322.51	115527.5	211288.4	124755.9	43098.37	47571.14	20924.83	68256.7	47146.91
51240.24	73968.44	178932.4	187721.1	113995.2	47976.15	36468.51	34631.17	84281.23	54272.27

LP387.199	LP387.199	LP387.199	LP387.199	LP387.199	LP387.310	LP387.330	LP387.347	LP388.269	LP388.269
48399.67	40231.93	55544.49	50446.55	45610.95	16422.79	19109.78	42106.36	106082.5	106589.2
50237.37	43222.32	51802.48	52732.72	51207.03	18242.19	18276.38	42843.92	110816.8	103362.1
59314.8	44456.25	41799.04	35904.59	39026.97	21930.55	15774.65	51952.08	113397.4	95775.65
40116.81	45336.63	47341.38	31556.4	48664.82	25985.36	18047.9	50727.31	113277.7	90931.04
60743.81	43289.18	44132.7	33863.15	48330.95	26127.72	13857.73	49563.64	122672.3	104603.9

LP388.269	LP388.269	LP388.269	LP388.269	LP388.269	LP388.269	LP388.269	LP388.269	LP388.269	LP388.269
130591.3	70404.39	100949.9	106315.7	79562.76	89010.17	97055.79	70510.6	62094.91	93676.16
87802.91	73505.06	101952.8	116536.9	78631.02	70247.8	90266.1	63770.18	63436.74	99662.23
98318.23	64159.2	101472	94255.06	81236.48	90493.18	118276.8	71180.82	68235.82	99580.63
116309	63195.1	89989.61	88382.28	91629.07	89358.98	100717.6	61125.02	63674.75	99682.6
122270.9	62867.03	100338.5	101337.5	78016.87	85771.34	91550.69	68336.46	62757.33	107627.9

LP388.269	LP388.269	LP388.269	LP388.269	LP388.269	LP388.269	LP388.269	LP388.269	LP388.269	LP388.270
97126.93	100621.5	99176.24	73597.2	72727.27	91204.59	68298.07	79940.46	94448.41	87348.15
92017.61	98717.41	147131.3	61604.14	71148.52	77157.84	68659.5	97500.68	93472.71	91950.47
94356.94	106577.5	69928.33	65258.63	79809.65	90697.88	79491.97	84007.7	88073.31	90980.08
94326.46	95879.67	101774.3	68256.22	73772.27	89727.33	65840.18	71592.96	89586.75	87432.71
112976.6	99639.88	93808.33	62962.57	70008.91	66281.46	66235.25	75440.39	95567.22	90565.85

LP388.27_[illegible]	LP388.269[illegible]	LP388.270[illegible]	LP388.269[illegible]	LP388.27_[illegible]	LP388.269[illegible]	LP388.269[illegible]	LP388.270	LP388.269[illegible]	LP388.269[illegible]
81735.89	71580.81	131710.3	109012.1	75710.25	93676.63	77709.04	57353.64	75645.71	59143.79
83136.28	71414.09	142480.8	109972.8	67274.91	96870.01	80098.71	59236.27	70244.29	72254.33
81073.5	65910.24	132852.4	107484.3	68846.92	98098.18	68104.29	61595.21	80064.06	64139.35
77685.36	81927.52	134037.2	100732.3	73706.15	94006.93	73787.64	52135	71577.29	59700.86
78356.69	71202.75	144262.4	100829.8	72730.08	91703.55	75191.6	53805.34	74557.56	65687.38

LP388.27_:	LP388.269:	LP388.269:	LP388.269	LP388.269:	LP388.269:	LP388.27_:	LP388.270	LP388.269:	LP388.269
82592.23	55662.22	76237.49	86643.7	116146	78112.34	68809.46	69135.33	84735.05	79872.53
73121.52	60053.8	65255.33	78020.17	108093.8	69478.72	67470.11	65191.87	88761.31	79949.77
75721.63	67444.57	67575.05	79395	113707	79612.81	64003.99	65092.52	93143.56	78062.91
74338.58	61094.7	72896.98	79030.96	108197.5	72405.54	64592.44	77640.9	89648.36	82243.61
76053.85	59736.55	77605.16	78721.27	110263.2	73935.54	75735.79	64959.88	90478.53	81673.31

LP388.269	LP388.269	LP388.269	LP388.270	LP388.27_	LP388.269	LP388.269	LP388.270	LP388.269	LP388.270
57160.34	79566.58	78094.05	68599.09	79675.37	78121.43	71787.08	79572.85	67493.86	99060.59
56820.93	78183.18	84399.82	65239.35	78077.18	80206.02	73050.23	83916.2	68750.11	100474.5
50797.2	83881.85	87973.02	80981.12	79661.32	84339.64	77296.66	87013.44	70556.54	104738.8
55913.75	90483.84	78586.68	65452.8	84274.83	84117.85	72352.89	81328.15	70052.39	102177.6
51481.11	72917.61	74990.57	67797.74	80131.07	85998.07	70187.39	79608.69	75380.76	96575.54

LP388.269	LP388.27_	LP388.269	LP388.269	LP388.306_	LP388.306	LP388.306	LP388.305	LP388.305	LP388.306
82085.81	70856.98	66926.53	67498.11	82120.37	89863.32	61419.72	86040.88	113221.4	104072.4
83850.68	69799.14	71607.21	81454.28	82315.26	84139.15	61871.83	85478.97	106295.3	99852.33
83825.72	70799.4	73330.19	77751.85	76130.04	83475.9	64021.48	87491.43	116192.2	101218
83415.87	70908.84	71916.07	65111.27	84853.91	86127.33	60667.39	82722.32	118104.4	100759.1
79632.31	65495.3	63847.83	72940.62	77999.25	83177.27	60733.5	84636.88	114668.3	107941.2

LP388.306	LP388.305	LP388.342	LP388.342	LP388.342	LP388.342	LP388.342	LP388.355	LP388.379	LP388.378
126989.5	144594.8	161242.6	123908.8	93834.74	86802.41	88368.61	34588.55	39295.86	18473.43
119664.4	140526.1	160959.2	112781.6	95738.39	78330.24	74135.47	32904.95	39786.52	17462.78
120229.5	128415.4	162117.2	118927.8	94852.22	75975.04	82591.57	30826.09	37646.22	12843.56
123304.1	138374.6	160638.8	119266.9	88610.41	77749.58	86777.03	29023.54	41347.46	12004.49
128164.7	143362.7	158402.6	126408.8	91671.29	85493.6	88218.06	20169.39	36620.29	11706.67

LP389.112	LP389.112	LP389.179	LP389.179	LP389.179	LP389.179	LP389.216	LP389.216	LP389.216	LP389.216
24394.72	12464.59	52841.63	35753.27	51289.74	26243.56	35827.73	50888.5	40774.1	35990.99
35319.56	18458.35	46567.26	35744.29	48765.41	33425.78	35119.39	55074.63	42517.86	39479.51
45841.1	21859.34	45598.13	34044.76	53420.17	32448.7	37376.31	51228.8	37561.55	38473.26
68592.85	35460.12	44762.29	36061.49	51173.52	33464.74	36214.92	57349.25	43576.84	38757.47
71358.62	30652.96	42898.68	38081.56	45103.38	32744.92	35077.05	51112.66	36934.13	40069.28

LP389.301	LP389.342	LP389.346	LP390.212	LP390.212	LP390.212	LP390.212	LP390.212	LP390.212	LP390.212
28866.19	16076.65	24701.88	40383.01	54738.12	51692.9	57585.61	56453.56	50243.63	56643.98
22894.48	13032.45	27394.18	42467.62	70242.74	43341.23	58321.3	40405.94	41975.31	40451.51
24941.21	14998.67	27377.56	60389.01	80248.93	64545.88	85924.27	76854.59	68772.63	73494.35
24239.47	13049.35	25207.39	55154.73	79731.12	64835.36	79331.92	65604.63	52805.98	78267.62
22324.74	13406.5	27094.34	60405.53	86648.15	69222.22	90696.4	67739.77	70116.97	70814.45

LP390.212	LP390.212	LP390.212	LP390.212	LP390.212	LP390.212	LP390.212	LP390.212	LP390.212	LP390.212
49567.76	59566.9	47322.46	74458.23	87684.42	73340.08	40200.32	103513.4	81241.87	89852.28
62280.96	50640.01	49139.58	52423.88	52279.59	41376.26	64073.63	61043.52	46345.21	55875.82
89225.63	85734.4	66978.77	79183.05	92887.97	67634.52	57553.36	102078.6	68371.13	96961.11
80021.76	78672.53	68419.87	77770.28	86151.57	66490.32	51475.3	105864.1	70925.43	82466.42
98789.43	78554.88	73064.92	72477.61	83950.52	65347.9	59225.6	106049.6	65732.99	95275.21

LP390.212	LP390.212	LP390.212	LP390.212	LP390.212	LP390.212	LP390.212	LP390.212	LP390.249	LP390.248
94312.41	38035.86	52134.84	49007.76	52728.3	61957.94	44510.6	73949.08	77571.39	92423.66
60115.97	61415.76	45591.48	58974.28	67234.1	102030.1	49708.09	49491.48	85092.7	84010.21
93302.15	74500.4	66939.8	92917.7	85854.17	82336.01	72551.7	62720.68	78975.33	94221.71
97108.7	71012.15	66013.91	76056.47	82954.02	85655.35	72965.42	67314.06	74293.11	93239.51
99955.77	78512.59	62968.65	91863.98	89827.67	83760.16	73184.22	72618.9	74123.32	80499.16

LP390.248	LP390.249	LP390.249	LP390.249	LP390.249	LP390.249	LP390.249	LP390.249	LP390.248	LP390.249
93662.68	94222.04	98704.78	74978.48	81442.94	84688.3	120317.6	72424.51	64623.65	83680.16
94336.41	82876.12	96972.35	77036.4	83179.07	82658.27	122811.6	62578.81	69039.17	111588.1
102902.1	88576.99	98901.14	72192.13	83577.5	75619.84	119691.7	62854.91	63863.57	75922.49
142228.5	82782.46	88378.13	84386.59	83391.25	60330.39	127674.9	59191.77	64766.95	88641.45
62719.23	88441.73	87280.26	60787.84	81224.53	82024.87	128237.6	68428.08	60651.94	79298.68

LP390.249	LP390.248	LP390.249	LP390.249	LP390.249	LP390.249	LP390.249	LP390.249	LP390.248	LP390.249
87224.41	73062.9	93901.93	76462.62	49851.71	79799.54	100225.8	72113.98	112608	76667.81
89359.7	80509.76	95846.38	87418.29	51147.76	74278.16	84340.08	77623.21	97362.41	79858.51
81705.43	73689.49	96700.69	88953.87	49719.45	78954.41	90031.95	74556.86	99327.11	77758.35
80162.61	87415.95	102657.9	88407.49	86878.24	81063.21	89611.43	75641.86	109146	70103.94
122743.2	65778.34	95583.7	80592.33	50608.94	79598.98	100492	70852.32	116446.7	71193.53

LP390.249[illegible]	LP390.248[illegible]	LP390.249[illegible]	LP390.248[illegible]	LP390.249	LP390.249	LP390.249[illegible]	LP390.249[illegible]	LP390.249[illegible]	LP390.249[illegible]
71799.22	106919.9	83924.29	55083.14	92045.3	78551.73	64961.36	89624.86	73412.96	133733.4
68582.48	92860	89567.62	50442.86	88364.01	75635.87	66940.6	87330.91	85841.72	126084.5
72580.09	86110.6	88370.43	49635.75	99399.13	83548.95	67608.58	88162.93	72795.81	133857.1
67033.03	90986.43	84876.39	47993.37	111355.1	80120.14	65315.37	93612.98	71281.96	138964.7
73897.51	85936.54	83855.81	45378.82	85074.69	76040.22	72407.24	86828.75	78857.81	119727.5

LP390.249	LP390.248	LP390.249	LP390.248	LP390.249	LP390.249	LP390.249	LP390.249	LP390.249	LP390.249
84155.68	94280.99	75415.37	60609.2	97244.98	65165.78	94358.28	129744.6	78388.59	70023.85
77158.78	83210.35	74081.27	76287.91	96567.67	65278.67	119682.5	123711	78050.72	74753.54
68364.15	88599.74	72425.94	70237.02	94830.05	70802.82	97912.15	124471.5	86751.26	73926.83
70464.49	87041.46	68278.17	69621.19	90105.9	64377.93	93607.31	127158	81834.08	77278.91
69630.02	71772.93	65730.56	75711.97	117647.4	62104	93528.45	116820.8	71601.22	76819.98

LP390.249	LP390.249	LP390.249	LP390.249	LP390.285	LP390.285	LP390.285	LP390.285	LP390.285	LP390.285
72445.44	66862.41	98219.78	91729.29	156530	137537.8	114226.5	143538.5	144353.4	131371.1
83087.68	58288.91	91319.81	88491.98	146372.4	128150.4	104148.5	141873	114439.8	118662.2
81811.23	60054.02	95727.24	95633.67	179556.8	148411.9	111334.6	137155.9	129046.4	173728.3
69362.38	54044.15	82724.87	90369.3	165469	124884.3	146708.7	171256	153019.5	133095.9
72098.45	61267.3	88615.76	88870.39	177174.2	117486	123073.2	136787.9	160341	112766.8

LP390.285	LP390.285	LP390.285	LP390.285	LP390.285	LP390.285	LP390.285	LP390.285	LP390.285	LP390.285
107103.3	166741.5	121386.5	136996.7	119652.1	118120	91037.87	122386.2	118620.3	135043.9
103020.9	173200.1	115923.4	205532.8	124269.9	118380.5	90901.66	123011.4	111442.7	129144.3
102373.1	173934.4	130333.6	112161.6	159959.7	123670.4	92710.91	129055.1	124398.2	144023.5
109926.4	178154.2	143733.7	142925.7	137310.3	123687.9	90713.53	123340.7	120997.1	130152.1
155457.5	166754.3	119509.7	151452.7	104661.3	132182.9	158556.5	124610.1	116097.9	178273.8

LP390.285	LP390.285	LP390.285	LP390.285	LP390.286	LP390.285	LP390.285	LP390.285	LP390.285	LP390.285
150741	121366.2	136111.8	94760.97	155535.4	131364	91583.14	147760.4	126380.4	124282.6
116362.8	127895.7	117878.4	103703	131385.3	135845.6	95194.61	150490.9	114390.4	117794.4
133368.5	117555.6	128364.4	113253	139379.9	122126.5	93559.19	154250	99466.57	115850.2
113825.5	122035	121303	96786.37	138490.3	140768	108685.2	142701.1	126088.3	103801.7
113720.5	113538.8	112226.5	97919.09	139271.6	136607.6	100877.1	142881.5	135757.1	103765.2

LP390.285[illegible]	LP390.285[illegible]	LP390.285[illegible]	LP390.285[illegible]	LP390.285[illegible]	LP390.285[illegible]	LP390.285[illegible]	LP390.285[illegible]	LP390.285[illegible]	LP390.285[illegible]
103869.7	134987.6	92662.49	115206.7	130580.5	130766.4	115455.5	125120.3	102391.6	113561
108667.4	129627.6	88276.85	109565.5	121889.7	124192.7	97073.89	128134.9	105238.6	118574
104702.7	101815.3	82144.13	113271	127450.2	135176.8	127398.2	117243.1	111264.9	116656.6
107768.1	126327.2	86657.88	114280.4	132978.1	135448.3	103308.7	135728.6	104320.2	112774
104945.7	117817.7	92567.93	107207.9	125877.9	139500.9	98688.22	123672.8	97983.34	112835

LP390.285	LP390.285	LP390.285	LP390.285	LP390.285	LP390.285	LP390.285	LP390.285	LP390.285	LP390.285
97406.59	111584.9	114666.9	103797.7	103223.8	149310.5	71512.92	120177	117117.7	93999.58
85088.8	120378	116528.5	114932.3	118560.2	129868.8	74402.04	113756.3	102411.2	97542.07
90654.36	108968.5	114055.7	117200.3	118204.2	135048.1	77775.3	84304.95	100945.2	104842.7
86482.27	105432.6	112129.8	114619.1	122715	134487.8	66298.87	121564.3	102608.7	86647.18
88006.77	115786.9	116925.3	111082.4	118446	129813.1	72522.19	125641.5	92693.71	99715.23

LP390.285	LP390.285	LP390.285	LP390.286	LP390.285	LP390.285	LP390.285	LP390.286	LP390.321	LP390.321
90052.87	112673	102611.7	101137.1	86966.85	113896.6	164678.7	94919.1	74709.23	155583.7
103720	87216.87	95703.13	95449.05	86023.11	106738.2	162588.1	88152.3	133784.4	150619.1
117505.1	118910.7	118134.9	91977.06	83742.07	103878.6	171290.9	89958.7	134306.3	153385.7
101335.1	100516.2	94083.84	93348.2	88758.2	111363.1	178346.7	104492.3	119234.4	153771.2
95922.48	108047.8	101856.5	99131.22	92380.64	105358.3	167496.7	83612.18	115195	144048.2

LP390.322	LP390.322	LP390.322	LP390.322	LP390.371	LP391.214	LP392.216	LP392.228	LP392.228	LP392.228
163487.2	116080.8	105435.4	164757.4	16997.91	229911.3	42690.13	156613.7	117036.6	77989.53
144544.3	118197.8	91142.9	159002.7	17848.89	233165.3	56805.38	164201.7	110421.6	85579.24
148485.5	105781.9	81788.96	149520.8	28257.21	238202	56666.46	161307.3	120848.7	89285.69
147750.9	105771.7	91347.83	155885	36102.58	179221.1	59696.98	159438.4	107572.1	82636.87
140907.2	122517.5	94245.77	148628	39901.13	194608.9	67191.87	157179.7	113497.1	89158.73

LP392.227	LP392.228	LP392.228	LP392.228	LP392.228	LP392.229	LP392.228	LP392.228	LP392.229	LP392.265
62611.71	112805.7	116251.7	130908.9	115774.2	80311.4	92504.94	79307.51	138221.4	1038470
68860.21	120458.2	107473.3	131935.1	106017.9	82753.56	95816	77701.57	146543	982656.4
71906.53	117088.5	114442.2	122080.9	118036.4	83863.42	93647.7	77895.72	141658.2	987574.8
72135.18	120338.4	120575.7	125440.4	120471	81968.92	96027.75	81032.11	162578.3	921293.5
58507.27	122633.2	115131.2	127288.5	136052.2	80365.03	92839.99	75165.87	135073.8	987124

LP392.264	LP392.264	LP392.265_	LP392.265	LP392.264	LP392.264	LP392.264	LP392.312	LP392.313_	LP393.219
1424353	1546971	1126613	2516118	2176156	2073925	1347180	1899811	1817217	597464.7
1314934	1463535	1060001	2652584	2168084	2032617	1279474	1805989	1684159	552721.3
1155591	1368004	1025978	2364741	2075441	1967427	1187295	1782769	1660869	771254.7
1144586	1352099	1081108	2259619	2066490	1992043	1290347	1967917	1790032	698370.2
1357336	1585169	1222462	2377892	2237057	2179186	1450591	2268953	2007745	537631.7

LP393.317	LP393.333	LP393.372	LP393.372	LP393.372	LP393.372	LP393.372	LP394.207	LP394.207	LP394.207
286659.6	136207.3	51475.16	31283.33	43878.68	40743.33	51546.29	113551.9	105544.8	88984.8
271296.7	132368.6	49664.95	31846.83	40936.74	37438.13	46221.24	94500.94	108576.6	74703.58
264142.8	129272	48647.77	30163.07	38666.73	40408.38	48182.81	109108.7	111458.4	89562.7
284574.8	113845.2	49761.66	36098.13	39797.05	34333.72	47433.47	102564.8	115742.4	84291.34
315690.8	111161.8	48520.81	32000.45	45146.8	41159.04	47207.82	113716.4	118547.8	84580.68

LP394.222!	LP394.243	LP394.243	LP394.243	LP394.243	LP394.243	LP394.244	LP394.243	LP394.243	LP394.244
143631.2	181542.5	84047.32	89133.98	117922.1	70489.29	143551.3	77488.95	78653.95	89567
126057	158183.7	77621.24	103352.6	94145.65	78771.03	128844	80462.33	73055.98	81107.16
188915.6	154502.8	78802.52	89820.03	94395.03	76684.6	138837.7	79314.31	80581.45	80739.81
156965.2	145023.6	86060.34	77551.66	94277.29	68774.92	128483.5	80211.46	85954.6	87701.22
126747.9	196373.6	69429.94	87645.92	96213.42	70081.87	135900.8	69551.9	79416.24	85188.7

LP394.243	LP394.244	LP394.244	LP394.243	LP394.243	LP394.243	LP394.244	LP394.244	LP394.244	LP394.243
104001.5	114688.6	127635.5	85159.83	102135.3	96501.39	121488.7	126204.1	66904.56	93556.72
82564.51	119452.7	123065	66080.15	61540.34	97982.07	115813.1	95729.7	63734.28	98229.11
75598.19	106631.3	121665.1	65200.1	63425.62	100079.9	124632.7	105026.7	63253.01	96328.08
69501.48	114102.1	123769	68859.95	64097.73	97773.97	121086.2	97578.81	110908.6	111504.4
69095.48	117104.7	126254.8	71275.49	66561.89	105218.8	128617.1	103513.5	52154.75	99031.49

LP394.243	LP394.244	LP394.244	LP394.243	LP394.244	LP394.243	LP394.243	LP394.244	LP394.243	LP394.243
161579.1	95386.64	97338.48	73157.4	102154.7	75798.23	103066.9	91721.66	70690.49	101749.3
155252.1	79517.46	97426.02	58485.07	101024.4	76429.51	96442.07	93160.69	80306.46	99587.85
177046.1	76033.78	96838.28	59349.02	98766.18	69555.52	89812.88	96565.78	71503.15	103106.7
172146.1	72439.49	94914.05	60761.51	97652.33	73755.48	91480.35	94612.35	74704.63	97573.17
136693.8	72496.45	90662.64	53939.37	99013.67	71471.09	87957.61	100964.4	74593.31	98777.49

LP394.243	LP394.243	LP394.243	LP394.244	LP394.243	LP394.243	LP394.244	LP394.244	LP394.244	LP394.243
108314.7	87052.26	90902.19	56356.27	80138.88	78108.3	56621.1	87173.73	70043.43	84491.37
110183.6	90417.24	92056.08	58617.96	88202.63	96108.99	55929.71	87526.61	67097.32	83780.25
105144.3	94091.89	90692.08	57592.45	84466.06	93279.37	59071.95	90291.61	79425.07	90779.09
104812.8	92866.09	86792.94	63931.47	78469.91	85904.5	56314.35	105438.4	69539.98	82840.24
106581.9	92213.31	86346.28	56792.34	81135.78	88153.58	60056.56	87047.71	70808.03	85350.43

LP394.243[illegible]	LP394.244[illegible]	LP394.244[illegible]	LP394.244[illegible]	LP394.244[illegible]	LP394.243[illegible]	LP394.244[illegible]	LP394.243[illegible]	LP394.243[illegible]	LP394.244[illegible]
73379.21	82624.57	52333.71	52267.86	57322.02	135032.9	61920.8	72646.71	77028	78716.91
74510.13	86876.86	57617.91	56770.4	56745.9	115140.5	55507.39	72423.56	76428.47	72712.79
76132.63	80721.07	55375.56	54067.65	54884.97	125822.1	58813.28	76297	79862.84	73316.31
78544.04	76867.83	54591.31	56030.08	62185.76	127967.1	57554.46	73583.45	85893.9	71958.5
72029.59	76678.57	54549.47	56182.9	59196.36	128141	49586.12	66801.97	80863.44	72425.06

LP394.244	LP394.244	LP394.244	LP394.243	LP394.244	LP394.279	LP394.279	LP394.279	LP394.28_	LP394.279
78605.83	97713.1	107392.9	91595.4	72319.36	117825	94127.66	105880.3	152958.7	81495.16
73793.22	105261.3	100683.3	95762	70326.02	119781	93049.19	115190.6	192421.9	92471.39
72599.99	108297.3	98478.2	84988.21	77458.26	123645.4	104851.2	98680.65	144815.7	74383.84
73182.92	103712.3	106996.3	92196.64	73487.5	117018.5	83722.66	97759.58	128691.3	101243.3
68858.55	106305.9	96326.3	94597.45	68026.99	101590.7	72053.89	113179.2	148710.3	63904.47

LP394.279	LP394.279	LP394.280	LP394.279	LP394.279	LP394.279	LP394.279	LP394.28_	LP394.28_	LP394.279
136676.8	204055	160270	118585.6	92055.54	94520.54	112096	62143.04	85169.13	96607.11
129530.5	128748.1	135232.9	112307.9	91568.92	70486.69	69213.62	56598.14	87137.56	90500.62
136878.9	120435.5	139364	115441.5	105687.3	84051.66	116628.3	60716.96	88430.34	87304.96
132979.3	119846.9	131953.6	160788.2	88023.37	95374.07	113258.8	92480.72	89549.06	88040.36
103598.6	128773.9	141030.9	108556.2	82964.75	89846.49	115054.7	59867.58	89637.32	90473.76

LP394.279	LP394.279	LP394.279	LP394.279	LP394.280	LP394.279	LP394.279	LP394.279	LP394.28_	LP394.279
73199.17	91711.2	80247.81	97387.69	89060.54	139741.5	82426.21	99303.33	89833.4	96337.13
78350.66	89970.1	77478.67	76641.6	109633.4	119634.2	90471.73	107805.8	88964.65	104978.4
70635.91	84809.11	77456.62	101590.3	93941.6	118694.3	85186.97	97407.92	79627.08	92343.07
75207.16	80800.16	76480.36	101009.7	92674.71	125607.3	84123.92	107296.7	101972.8	95201.51
73185.31	85614.14	72594.44	100713.8	94048.41	126524.5	72502.48	110854	88395.67	96834.08

LP394.28_	LP394.279	LP394.28_	LP394.280	LP394.280	LP394.279	LP394.28_	LP394.28_	LP394.279	LP394.280
119089.9	112373.9	69863.2	75174.96	80739.11	81024.99	93312.55	113685	82613.51	136319.2
126241.1	119929.5	71548.8	73171.25	71528.29	80217.23	88591.76	117010.9	95389.66	132770.1
123943.4	116450	73878.52	70891.31	79716.84	76544.75	88499.8	124652.6	95928.07	131464.5
113302.4	119670.3	67768.08	84308.39	78851.59	80441.51	84851.17	118230.6	99653.51	151471.7
120474.5	112558.7	81077.23	60476.32	79568.98	78508.81	89311.05	122665.8	98262.63	138206

LP394.280	LP394.28_	LP394.279	LP394.28_	LP394.279	LP394.280	LP394.28_	LP394.279	LP394.28_	LP394.279
107081.2	124161.1	136942	89183.67	66090.85	109430.6	86694.31	89418.62	96926.26	67845.57
107746.2	116499.9	125244.2	83034.19	77327.8	101520.2	71639.36	95504.84	93954.08	63509.06
108117.3	112770.8	138658	85363.68	78256.94	119340.1	87854.66	83928.81	101045.3	67058.79
108960.2	115413.8	125688.8	86219.18	67475.06	113902.3	78197.96	89820.83	96217.96	67252.36
113641.6	122244.6	135260.4	93843.59	78386.43	116722.4	77666.82	91092.68	93759.19	61113.54

LP394.280	LP394.279	LP394.28_	LP394.280	LP394.28_	LP394.279	LP394.279	LP394.28_	LP394.279	LP394.279
78929.35	66263.95	107158.1	68246.13	63920.87	100318.2	68798.75	58027.38	83932	52302.49
88050.57	69746.76	107764.1	77476.99	65668.89	101979.6	70212.41	62014.66	85661.42	57298.78
79308.67	68347.49	105573.5	73311.86	68093.77	99947.66	70870.08	58030.22	80247.89	48710.06
95275.39	66744.64	90477.51	69640.84	68655.88	95682.85	70564.98	59048.45	71769.33	50806.18
96254.65	65542.28	97391.6	69945.84	67312.82	96073.36	67042.12	57222.93	80650.24	52667.28

LP394.293[illegible]	LP394.293	LP394.293[illegible]	LP394.293[illegible]	LP394.293[illegible]	LP394.293[illegible]	LP394.293[illegible]	LP394.294[illegible]	LP394.293[illegible]	LP394.294[illegible]
122158.5	142470.5	90739.22	99086.98	103582.8	90497.42	123900.2	112298.9	97209.54	145337.4
126773.3	137571.8	92956.99	97534.97	108219.2	89535.16	128576.2	111817.2	99720.87	128209.6
99642.14	138920.3	89076.75	99326.26	109131.4	93505.86	113680.3	108740.7	97424.3	131309.3
116515.5	143972.1	81177.28	94522.58	105827.7	112383.6	113707.6	107700.9	94489.18	154235
130257.8	129663.9	95441.5	94676.66	115429.8	92409.71	127918.1	113775.3	99694.53	144583.9

LP394.293	LP394.294	LP394.329	LP394.337	LP394.352	LP394.886	LP395.220	LP395.242	LP395.277	LP395.276
69583.25	59364.53	57085.16	57771.2	61946.56	135540.8	154450.2	43105.23	100825.9	84802.34
69639.34	67278.33	61350.47	22051.35	63287.21	121333.6	141395.8	44760.47	91740.77	81162.19
68452.84	60833.98	15952.21	61787.48	74284.25	134849.9	134669.9	49314.17	121990.7	82155.65
69173.93	63045.04	56117.45	21184.32	75497.96	158470.1	129668.5	52701.03	76438.81	86044.83
67962.56	60049.86	58160.91	22258.19	80413.09	107017.2	134035.9	52961.35	94878.15	89349.97

LP395.315	LP396.222	LP396.222	LP396.222	LP396.223	LP396.223	LP396.223	LP396.223	LP396.223	LP396.223
56376.16	94179.06	83385.49	78788.96	106439	122989.3	97760.71	100321.7	194639.6	69553.17
53207.98	102200.9	91934.45	73256.8	113846.2	112246.5	98697.18	89648.14	207413.7	74544.72
54226.23	104506.9	94043.68	72778.6	118612.1	115427.8	104124.7	100742.4	188501.4	69964.58
59620.24	95346.14	90320.23	80867.17	107791.9	125460.4	104710.1	102323.4	182409.3	77237.16
37783.6	102385	94472.08	72546.27	108877.2	134070.8	103566.5	95731.35	206631.8	70290.69

LP396.223	LP396.223	LP396.258	LP396.259	LP396.259	LP396.259	LP396.259	LP396.259	LP396.259	LP396.259
152767.8	106632.1	65732.17	93752.63	88222.8	119626.6	91206.68	94678.24	61299.87	112864.5
153593.7	104108.4	87692.54	85023.77	103758.4	122186.9	109184.6	104824.4	68574.26	124829.2
144409.4	111472.8	106555.4	106119.2	92571.18	125031.4	98817	114896.8	68843.31	100904.8
151678	102326.6	91481.4	95234.69	83098.09	118466	86552.28	101759.7	67940.9	106202.4
135701	105296.9	100931.1	99744.93	85046.12	113252.2	89949	106018.1	67490.59	104070.7

LP396.259	LP396.259	LP396.259	LP396.259	LP396.259	LP396.259	LP396.259	LP396.259	LP396.259	LP396.259
125901.7	73433.99	52860.38	54731.54	51420.34	77722.18	69199.77	102872.1	77361.99	50048.99
134408.8	69412.55	61124.78	58298.88	65031.51	87767.92	68653.4	101254.3	78959.49	53142.16
138928.6	69062.58	48942.73	57961.23	48190.45	79414.5	68710.93	114614.8	76233.31	50787.01
137813	65475.17	55828.63	51746.88	53086.6	85330.58	65596.91	106295	76495.6	45203.26
143017	73512.67	56275	54587.86	48549.21	74356.88	56090.74	104716.3	76648.25	52334.2

LP396.259	LP396.259	LP396.259	LP396.259	LP396.259	LP396.259	LP396.259	LP396.259	LP396.259	LP396.259
100472.4	62260.39	59643.2	83914.42	51885.73	64282.09	72155.95	58351.18	62431.57	76324.05
103931.9	56141.01	62268.01	83728.61	54383.3	61060.41	71211.68	48316.64	61923.24	76741.4
109686.8	64915.38	64479.41	86645.73	53285.19	70268.81	68205.95	60990.79	61207.08	77155.85
117661.2	54230.97	64329.03	82691.57	53054.19	69819.91	66119.69	51479.63	64612.67	79818.31
115913.6	63750.46	60079.32	80807	55436.85	69766.29	71818.94	54286.26	66519.61	74261.32

LP396.259	LP396.259	LP396.259	LP396.259	LP396.259	LP396.259	LP396.259	LP396.259	LP396.259	LP396.259
79316.24	69889.98	78917.44	79301.82	44368.3	54960.56	90817.17	51704.89	40046.2	80883.21
73783.01	64074.76	83997.06	81102.86	44662.55	54951.68	90224.83	54643.17	43713.53	73858.71
68768.22	71735.41	77189.32	90865.01	41142.91	55823.03	82163.08	43910.07	42536.21	79220.57
69772.63	66598.63	73650.54	76474.74	37823.95	57130.1	89980.19	49549.19	42623.75	72948.31
63783.55	63435.59	77857.01	79489.87	39035.58	54630.72	91592.78	54810.75	41030.61	75000.87

LP396.259(	LP396.259.	LP396.259!	LP396.259!	LP396.26_:	LP396.259:	LP396.259:	LP396.26_'	LP396.259!	LP396.259!
91319.88	64580.95	45957.06	54464.14	93887.72	63166.95	43550	78300.52	33291.76	60121.1
97153.86	73942.36	50468.54	48899.43	98160.95	60988.79	40206.54	73957.18	31917.29	59881.94
86742.22	67905.34	41258.16	52684.89	99217.99	57912.82	38847.55	76154.71	31522.47	65350.87
92163.95	62422.21	38491.65	51402.33	94518.21	58521.79	33413.1	75562.84	31553.17	61318.39
86778.9	65249.84	45793.61	59657.49	97485.95	59407.99	42956.57	72812.46	34001.21	57946.71

LP396.259	LP396.259	LP396.260	LP396.259	LP396.259	LP396.259	LP396.259	LP396.259	LP396.259	LP396.259
56021.51	53337.26	39406.19	31681.3	69143.13	54763.26	50347.38	72594.58	74133.95	42156.15
56684.68	51354.43	41676.47	31309.72	64885.35	52848.83	47139.84	60960.46	74303.93	40082.39
57429.69	50327.53	46932.22	26407.22	61279.67	50865.03	43424.16	73846.65	77509.57	47632.28
54241.88	42505.26	37549.85	25844.93	59870.52	53977.96	43702.28	73241.9	72715.03	38520.71
60142.14	53178.65	41931.58	29043.04	59691.79	50319.3	44284.44	67140.75	72401.12	41836.07

LP396.259	LP396.259	LP396.26_	LP396.26_	LP396.26_	LP396.26_	LP396.259	LP396.259	LP396.259	LP396.259
67769.56	75559.75	36945.64	37504.03	45077.3	60389.97	74082.51	50214.66	80956.8	63570.88
68393.82	79452.73	37768.23	36387.67	49443.51	65128.48	75745.04	43404.04	84286.54	59763.34
68185.63	81066.31	35767.18	37140.96	45887.29	72220.46	67500.02	44188.22	80724.44	67351.2
69644.52	77031.99	29271	30919.63	41349.03	67984.93	71060.58	51211.84	89626.23	64679.27
70490.25	74246.58	35245.63	32303.29	41392.14	65032.15	66295.8	39914.11	80681.97	59016.64

LP396.259	LP396.26_	LP396.259	LP396.26_	LP396.26_	LP396.260	LP396.259	LP396.260	LP396.259	LP396.260
34917.66	39683.71	61832.51	39652.39	64742.59	39241.77	63005.49	48995.11	46066.97	81188.84
31148.45	41400.83	64131.4	40890.6	60995.48	45661.74	60570.32	48446.13	46197.93	71456.77
33847.52	36605.2	61673.74	38118.65	63325.13	35151.26	64998.21	45738.31	51905.92	76033.75
29104.7	38481.55	59818.1	39284.34	59381.99	33752.31	64579.94	43305.58	44289.85	79249.73
29883.51	41880.36	61726	39893.51	67849	35603.27	62141.81	46020.15	44581.68	73846.83

LP396.26_	LP396.259	LP396.280	LP396.279_	LP396.332	LP396.383	LP396.420	LP396.419	LP396.419	LP396.420
57851.26	111058.1	80382.26	78023.08	44699.08	21170.7	44619.54	19060.85	15627.13	6842.477
55830.68	112945.9	76879.99	77406.45	42997.51	18476.29	43092.76	18494.35	13631.23	6173.954
53447.58	101338.9	69424.71	77615.09	41880.27	17584.93	41628.88	26690.37	26719.98	7922.519
50043.84	108587.6	63675.63	76426.64	39413.23	23063.99	35488.58	26791.72	36776.23	12473.52
58167.68	117630.6	63087.73	78918.22	39943.36	25683.4	41518.35	29845.88	35957.98	13302.32

LP396.420	LP396.883	LP397.183	LP397.183	LP397.183	LP397.183	LP397.183	LP397.183	LP397.183	LP397.183
13715.35	68683.16	102164.1	105534.7	91301.4	87038.02	101833.2	91272.82	57963.96	89077.56
17527.34	63790.26	99569.84	65362.88	71140.13	85932.29	101632.9	69502.07	64670.41	92235.6
14275.95	74153.23	120265.3	79772.81	105745.8	83178.86	70101.85	67556.4	90373.42	112724.2
13357.06	89865.47	74688.22	67628.96	105521.8	121512.2	112969.5	74404.58	62426.17	71609.98
12632.04	53905.56	97681.9	87574.9	75900.5	101387	79515.47	94874.52	107454.3	66014.13

LP397.183	LP397.183	LP397.183	LP397.183	LP397.183	LP397.183	LP397.183	LP397.183	LP397.183	LP397.183
73122.03	84629.1	101949.6	94490.45	96594.05	96288.54	94047.2	105022.2	101834.7	69570.56
67865.26	93699	80510.29	93196.22	85246.04	103373.3	98793.5	83703.65	79341.93	96198.82
75469.7	87267.44	57879.92	79697.06	82516.22	86801.25	83934.22	98169.9	80111.41	82743.7
74242.13	74590.64	88191.75	88531.09	70837.63	78108.15	90894.79	136063.5	67042.09	82219.29
122541.4	74505.63	93711.89	80263.73	68023.5	79906.83	114170.5	82478.55	101973.2	98082.37

LP397.183	LP397.183	LP397.183	LP397.183	LP397.183	LP397.183	LP397.183	LP397.183	LP397.183	LP397.183
113407.5	111625.9	48057.41	116522.4	88704.18	51031.61	85655.22	108120.6	88102.68	87545.39
106364.2	95027.78	44492.44	87914.51	73478.25	60048.82	103637.8	85300.72	74344.7	82820.97
105048.6	104104	42016.63	72828.02	75709.71	57055.31	102946.2	148217.1	119606.4	87271.67
105071.5	108929.8	37115.51	79635.2	99221.9	45689.13	70166.35	162190	87461.25	118280.5
140181.4	118617.5	52914.38	67628.05	79910.43	50479.52	111536.7	121211.8	139007.2	65507.89

LP397.183	LP397.183	LP397.183	LP397.183	LP397.183	LP397.183	LP397.183	LP397.183	LP397.183	LP397.183
38672.6	71564.33	115386.2	45041.51	101742.3	75316.4	81406.99	61517.06	92639.23	122233.2
46278.89	72598.57	90766.44	39941.24	91996.32	91065.71	68482.9	71646.22	97488.6	118859
40342.34	77607.49	82304.81	45686.15	82848.18	86331.35	66702.06	75535.77	110777.8	110589.6
44088.17	83966.83	76444.32	41586.38	90287.67	53914.33	85892.99	63212.95	122833	116147
54512.01	67776.54	70830.61	46405.52	101707	101949.9	66050.56	73129.04	114454.7	97989.1

LP397.183	LP397.183	LP397.183	LP397.183	LP397.183	LP397.183	LP397.183	LP397.183	LP397.183	LP397.183
162531.5	47308.33	105830	111278.2	98244.18	55912.09	81223.52	99876.2	93681.72	107470.4
99999.5	45852.2	89080.52	111430.5	104376.3	55864.51	86885.78	73629.32	83293.87	87166.17
144048.1	32850.1	100437.9	120897.1	115403.1	39131.54	90599.74	93078.58	116435.6	83054.29
123462.1	38231.61	126679.2	94259.22	96587.1	31155.05	96382.69	71948.39	93289.32	125556.5
109179.1	44072.67	110834.8	133955.5	113007.6	41019.07	97810.38	85971.19	120896.5	88843.19

LP397.183	LP397.183	LP397.183	LP397.183	LP397.183	LP397.183	LP397.183	LP397.183	LP397.183	LP397.183
55229.23	47242.17	38369.02	94683.05	85226.21	34793.35	82629.85	84196.02	103501.6	31796.47
61903.25	63864	28412.72	104480.6	92561.5	35258.62	83341.04	98705.96	88459.6	28566.95
42401.36	49732.02	29441.34	85287.96	94141.36	45612.45	95153.92	91737.74	82062.73	31724.12
37791.85	49793.91	31919.14	77334.54	94784.98	34106.72	91250.72	141754.1	105483.6	27562.86
47175.56	59107.59	34119.79	94944.18	109742.1	50792.11	80300.69	65462.73	111041.1	43739.12

LP397.183	LP397.183	LP397.183	LP397.183	LP397.183	LP397.183	LP397.183	LP397.183	LP397.183	LP397.183
120831.6	113776.5	74449.9	64327.34	42247.7	62527.32	42987.64	89042.39	79607.94	105026
111260.5	106141.7	76349.41	36136.22	31597.61	52422.96	44205.77	86516.89	82138.19	78476.12
124716.7	96245.9	54880.51	51673.35	42254.35	63527.86	36587.28	88977.06	70797.03	67216.73
74455.25	85173.91	55038.56	36683.5	39519.24	73480.79	52077.97	72656.89	58441.4	72853.59
102793.1	100658.6	69187.7	38618.83	34345.79	54498.42	44838.34	97292.84	59810.68	82851.62

LP397.183	LP397.183	LP397.183	LP397.183	LP397.183	LP397.183	LP397.183	LP397.183	LP397.183	LP397.183
101280.3	99890.26	80360.5	81874.66	75398.63	90794.21	66399.88	109143.2	62674.33	69107.4
127661.9	101821.2	67284.15	84248.62	82135.6	104288	123903.2	88208.39	52146.66	73322.23
130105.6	90032.98	80417.59	100585.8	79354.6	95262.6	71275.55	117270.7	61530.27	71699.72
92266.02	74687.99	85086.8	81720.05	121919	91582.35	79422.76	75894.31	90315.55	71345.06
105625.1	81247.61	73703.84	76151.1	77733.6	100869.5	87994.68	110472.6	60303.17	72220.08

LP397.183	LP397.183	LP397.183	LP397.183	LP397.183	LP397.183	LP397.201	LP397.201	LP397.216	LP397.230
76851.11	120135	28496.79	90991.32	83846.7	103267.7	10144.06	30137.53	67715.84	230577.4
84825.24	73642.54	51242.59	119030	83613.92	98804.7	38229.28	30644.1	121960.2	234840.4
85224.58	72669.04	39002.13	108091	69456.89	101997.3	33705.44	26213.72	125651.5	237567.5
54043.85	60401.9	41171.4	96068.06	72225.29	126069.8	34496.99	26308.44	117369.4	195563.5
74536.17	79766.81	31871.57	131640.9	108362.5	92865.5	33113.75	26081.37	82494.44	199748.6

LP397.258	LP397.258	LP397.271	LP397.293	LP397.294	LP397.294	LP397.331	LP397.331	LP397.331	LP397.387
24967.45	45083.85	225797.7	65684.42	60123.88	32067.06	41288.17	57767.75	55877.5	16210.19
28490.11	46425.72	232719	59437	59450.78	33522	42162.15	61511.65	52242.13	12501.81
26646.67	47597.44	256010.5	70171.25	61686.12	38840.65	40322.41	61154.8	61150.75	14944.12
21525.71	38843.75	260156.4	39497.61	41840.8	26178.65	37373.69	58854.68	50687.74	10969.11
20796.98	42958.02	306910.4	58221.76	69979.39	34073.4	37744.87	56175.48	60654.49	8985.564

LP397.415	LP398.238	LP398.238	LP398.238	LP398.238	LP398.238	LP398.238	LP398.238	LP398.238	LP398.238
83673.07	96170.73	114638.5	106997.1	137583.6	81210.64	90808.5	110290.9	74767.66	88051.14
69390.8	91925.22	155696.2	78218.91	100847.7	88124.93	92730.97	120878.3	81980.08	94727.04
78662.74	91401.85	146551.6	82472.49	95396.78	81042.85	83043.96	123767.7	73799.27	90848.84
60937.97	107594.3	166422.1	70347.94	166053.1	89515.24	87057.39	115281.1	75594.29	95531.65
69901.3	87246.76	141737.4	84169.34	94053.24	80145.1	83133.28	109963.9	76687.74	96966.67

LP398.239	LP398.238	LP398.238	LP398.238	LP398.239	LP398.238	LP398.239	LP398.239	LP398.238	LP398.239
110766	56163.96	78934.85	92947.13	108057.7	88420.2	75635.96	68918.5	90922.67	83034.09
117038.5	59841.76	78891.85	100539.4	98446.49	85900.58	84201.94	70749.49	95454.05	74236.13
112171.6	58012.93	80638.01	99195.73	100182.8	77792.5	83307.64	71728.96	92567.89	85447.15
105469.9	57744	72169.55	116227	108867.3	85979.45	82076.79	74090.59	95072.55	83585.19
112045.4	53203.51	70821.31	104081.9	115155.6	85405.36	79926.45	75031.84	93566.48	80586.83

LP398.238	LP398.238	LP398.238	LP398.239	LP398.239	LP398.239	LP398.238	LP398.238	LP398.239	LP398.238
63333.61	118564.9	89924.08	88025.92	77027.5	86193.89	90496.03	92451.36	84701.54	61577.97
68189.8	112627.4	74340.98	90288.61	76655.26	89449.08	96576.33	71049.9	86358.77	67274.72
67781.35	115229.2	70535.45	88114.76	71476.63	82615.44	100264.5	81182.89	88852.33	65127.23
67366.7	107390.7	76223.08	96740.81	74793.75	85414.26	88355.79	77028.18	81795.98	56217.81
67103.79	117781.9	73451.67	96173.39	80007.65	84008.95	93296.63	78094.88	81806.65	58220.3

LP398.305	LP398.399	LP398.436	LP399.163	LP399.163	LP399.163	LP399.162	LP399.163	LP399.163	LP399.163
16178.28	131867.6	99552.56	90424.58	129175.8	109992.3	124315.1	114579	169726.6	103814.1
16913.4	130772.3	67494.53	96197.41	113659.5	110588.9	122997.2	98758.44	177777.7	112458.1
14733.36	132202.8	119978.5	92097.13	114470.4	112479.5	133321.5	102049.2	161771.8	115000.1
11883.12	113331.7	99786.64	86675.27	116836.3	102221.2	123054.5	104962.8	158069.1	101858.8
16410.16	125929.7	144590.2	92286.61	115311.6	99629.31	126868	105801.4	156478	97696.9

LP399.163	LP399.163	LP399.163	LP399.162	LP399.199	LP399.199	LP399.198	LP399.199	LP399.199	LP399.199
97426.82	127877.3	119508.4	177409.2	113938	147994.9	134040	138774.8	123223.7	131896.9
98414.97	127594.9	120962.7	159164.5	101041.7	116733.7	123183.3	109015.9	168101.9	138121
100345.9	126756.1	124889.2	168430.1	89093.94	128929.8	89971.14	158425.9	112187.9	147566.4
92221.16	106611.3	121475	150727.5	96783.71	100325.1	137829.8	126513	146455	167685.8
104397	130242.6	117876	148557.1	114312.2	109910.7	95927.64	124252.2	120882.1	137486.8

LP399.199	LP399.199	LP399.199	LP399.199	LP399.199	LP399.199	LP399.199	LP399.199	LP399.199	LP399.199
161391.6	146969.6	87694.11	155550.4	118786.4	103753.6	166550.8	159358.5	142651.3	103444.6
200112.5	96817.66	105393	104104.8	115993.8	129234.1	146558.8	99064.38	140166.1	86927
176274.9	116111.1	132111.9	89212.68	217684.8	162641.4	175153.7	112270.6	179444.5	100721.3
142191.3	76846.26	137403.7	96651.75	159913.4	110496.8	129293	139420.4	132933.1	112900.8
129198	89723.36	89411.4	135544.1	187217.1	103118.3	145598.1	111432.9	161513.9	88780.86

LP399.199	LP399.199	LP399.199	LP399.199	LP399.199	LP399.198	LP399.199	LP399.199	LP399.199	LP399.199
117893.6	140656.5	62305.27	152351.7	108341.7	91866.2	133948.4	37303.99	133173.2	229566.9
115507.3	151393.4	39485.83	169275.9	95305.6	105091.4	126835.8	42294.86	171951.6	146208.1
135714.8	156119.9	49050.18	112398.1	105972.7	122810.6	100509.2	33279.44	143534.3	159448.8
154551.6	145901.2	49442.9	90149.67	85000.31	130293	149525.2	36865.1	161579.9	170958.7
111089.1	139084.6	47361.51	157419.3	97536.64	78216	116403	28988.22	130923.6	186129.8

LP399.199[illegible]	LP399.199[illegible]	LP399.199[illegible]	LP399.199[illegible]	LP399.199	LP399.199[illegible]	LP399.199[illegible]	LP399.199[illegible]	LP399.199[illegible]	LP399.199[illegible]
107607.2	151542.9	175704.6	71640.99	94965.41	208154.7	116526.9	106913.5	86061.29	222124.8
86347.12	158706.6	111664.9	93749.64	130806.5	137813.8	90495.07	84393.77	118904.3	161280.8
96974.04	194219.2	123594.9	66255.61	115552.4	153110.8	129018.5	119097.4	90661.85	201823.5
114835.3	144946.2	120220.6	91514.15	103727.7	182688.8	124962.9	113243	95482.27	168046.5
92111.45	159956.5	127553.8	129336.8	118600.4	108672.9	108999.4	87603.04	103296.8	156497

LP399.199	LP399.199	LP399.199	LP399.199	LP399.199	LP399.199	LP399.199	LP399.199	LP399.199	LP399.199
69071.1	172494.6	157679.2	123228.5	150437.8	164372.1	138862.6	112233.1	127821.6	125386.3
110855.2	175046.7	157346.4	110043.4	111085.2	171951.3	168897.1	121992.7	116245.6	134159.4
75410.73	157565.9	107915.6	120844.1	126219.9	156668.3	136576.9	106123.9	113039.9	173028.8
88814.05	153363.3	163181.6	115977.4	113499.5	167210.7	140944.2	125514.5	159737.1	132169
78631.11	123383.9	125557.7	107810.4	105003.4	165676	140213.5	162885.3	137695.7	125674.4

LP399.199	LP399.199	LP399.199	LP399.199	LP399.199	LP399.199	LP399.199	LP399.199	LP399.199	LP399.199
169363.8	91364.93	144499	93231.9	105566.2	117806	172915.1	220016.1	78093.85	34034.38
97543.84	101078.7	147157.6	82067.74	95182.49	116330.6	172091.9	131408.5	88606.14	63355.25
113295.7	134238.7	153664.9	111526.6	90763.19	84649.54	137045.9	148711.4	70591.08	43614.13
98319.31	97022.26	158933.6	88392.14	97641.16	87107.72	124641.8	125309.4	89169.12	44530.15
77402.31	119608.2	149028.9	87685.24	82054.03	106153.8	171272.7	123101.6	81319.12	33531.41

LP399.199:	LP399.199	LP399.199:	LP399.199	LP399.199	LP399.199:	LP399.199:	LP399.199	LP399.199:	LP399.199:
102288.1	121165.1	135317.1	99796.88	93791.61	110334.8	146255.9	116814.9	101150.4	140537.4
96835.71	154199.5	101795.7	146640.6	101446.7	122613.2	114678.4	124133.5	141412	167354.7
129537.4	178802	106986.5	127552.1	84413.67	107234.9	121364.7	101487.1	137013.6	105083
117939.3	138807.4	75062.33	124622.4	106673.8	184888.4	121631.2	71729.6	95209.18	217634.6
110808	155173.5	71195.94	103681.4	94525.47	169099.9	141212	82881.72	117005.1	164077.7

LP399.199	LP399.199	LP399.199	LP399.199	LP399.199	LP399.199	LP399.199	LP399.199	LP399.199	LP399.199
59608.61	40224.67	125664.5	128477.1	130689.6	73524.6	47280.11	64897.84	92282.41	92625.39
62298.15	42129.13	202580.6	137877.8	194214.8	45123.15	52219.98	53847.81	130283.8	114824.2
86560.66	39706.03	103085.5	119466.9	117783.5	49983.79	43306.9	50048.86	84400.45	86402.29
51582.68	45684.77	136936	119712.8	119207.2	68693.94	45557.83	48468.65	105736.8	104217.4
56651.32	44240.53	146282.4	120670.5	153635.9	56748.31	50277.58	43875.19	102511.8	108722.6

LP399.199	LP399.199	LP399.199	LP399.199	LP399.199	LP399.199	LP399.199	LP399.199	LP399.237	LP399.250
76222.01	41250.98	138307.2	98438.36	169521	65605.55	135410.5	70586.09	99765.6	35321.06
92253.39	46849.36	96995.93	143406.7	126513.1	54551.03	112387.9	74091.49	110413.4	32783.28
68739.77	28732.38	133411.3	86578.01	163772.8	61832.38	133186.6	48123.97	108425.1	34476.9
89791.51	57890.9	167962.3	138447.7	131515.8	51310.76	117334.4	68307.05	111548.9	32337.86
89651.27	33423.83	141416.6	140164.1	106942.2	93383.62	120856.1	50635.18	108384.9	30502.87

LP399.253	LP399.259	LP399.269	LP399.331	LP399.358	LP399.358	LP399.394	LP399.430	LP399.431	LP399.431
40512.34	28748.62	20975.69	23532.82	68049.86	82790.45	5840.147	13136.35	47981.28	31190.65
43262.17	33273.2	19300.76	23307.23	85335.94	76699.32	4035.951	13232.24	53502.04	25098.55
40604.94	34386.55	16554.86	18977.23	76455.79	83747.77	6875.405	21356.21	61000.27	32441.07
41048.04	42291.37	14243.8	19282.28	65296.12	56767.59	2422.774	24375.93	61392.72	31362.01
34661.42	44497.56	15714.99	23366.46	80774.59	61674.79	2688.624	28054.64	61799.71	28809.8

LP399.430	LP399.439	LP399.472	LP400.269	LP400.269	LP400.269	LP400.305	LP400.305	LP400.305	LP400.306
13405.35	21450.3	92956.14	38721.84	48597.24	46073.15	46490.26	37820.58	56142.45	50480.79
16476.23	26599.66	92667.4	42117.69	46983.74	48796.36	46366.56	39301.91	54018.65	53298.72
15349.13	21214.87	79068.57	40224.33	48815.62	48922.3	46893.91	37155.08	60975.73	56630.26
14200.22	18543.74	75253.04	38283.58	47343.61	50438.5	51186.79	37955.07	63828.65	65592.76
14333.23	25640.91	78219.15	43502.26	46580.85	46565.66	46152.68	39715.8	55356.06	59509.47

LP400.305	LP400.342	LP400.361	LP400.398	LP400.434	LP401.177	LP401.178	LP401.178	LP401.178	LP401.178
51607.81	208333.7	16240.31	10116.84	19629.77	139853.2	120136.4	163573.4	174418	120873.6
49474.34	204042.4	15296.38	12851.72	19839.98	152567.9	118075.8	111859.6	190249.4	92340.69
45387.02	193218.3	13648.23	12126.4	21321.33	124735.4	84067.51	162715.3	156932.6	122548.5
44015.83	200522.4	12250.71	4521.272	18372.94	139326.1	112076.8	133859.4	164411.2	122485.8
45740.54	192516	13996.83	11252.1	22338.27	132839.3	110910.4	127279.3	211386.4	151964.3

LP401.178	LP401.178	LP401.178	LP401.178	LP401.178	LP401.178	LP401.178	LP401.178	LP401.178	LP401.178
157889.6	127260	126350.7	165377	107857.3	220066	178867	170316	157086.6	163546.1
175175.9	195544.7	138944.6	121716.6	178374.7	143515	166721.7	160275.1	132223.4	210050.1
153937	91148.32	169941.4	202921.5	130070.7	189854.7	142353.8	162043.5	158184.1	137619.1
163581.5	123939.2	105636.1	169596.1	148816	168765.5	162699.6	192817.6	206684.7	179703.7
132271	117271	142189.6	163485.7	203283.5	129374.8	213552.2	158089.7	115935.2	187471.6

LP401.178	LP401.178	LP401.178	LP401.178	LP401.178	LP401.178	LP401.178	LP401.178	LP401.178	LP401.178
80424.56	129295.5	147355.2	225951.6	172502.8	129540.9	195194	189418.3	171078.2	218709.3
79970.93	137803.9	179393	146403.7	190365.4	149421	234813.9	194582.7	156376.1	188044.6
101535.8	123361.7	129216.2	302959.9	248832.1	193556.3	178435	202560.3	128513.8	211357.4
124387.4	109765.9	160562.9	221501.2	224109.9	126527.6	258752.1	230331.6	186375.8	232440.6
106462.9	119164.2	120167.8	165063.7	163460.4	135204.8	182107.4	155434.4	139611.3	199932

LP401.178	LP401.178	LP401.178	LP401.178	LP401.178	LP401.178	LP401.178	LP401.178	LP401.178	LP401.178
155472.8	129228.3	171843.5	212265.8	166805.7	85483.17	166433.4	131053	219458.9	129429.1
108214.2	166266	125311.5	165523.2	256277.7	178005.2	173938.7	175007.1	134665.3	233184.8
136882.3	168478.5	108620.8	147435	258615.3	156746	150290.5	204960.7	209435.2	204334.6
111179.3	127925.2	127213.9	257904.8	165675.8	102592.4	174866.9	130892.3	163033.3	141348.5
137046.7	118607.5	151300.6	160128.4	160187.7	96645.89	157724.5	140381.7	192582.7	232539.4

LP401.178	LP401.178	LP401.178	LP401.178	LP401.178	LP401.178	LP401.178	LP401.178	LP401.178	LP401.178
163340.5	97762.89	126166	136715.6	163064.3	105491.5	226518.1	101612.8	232437.2	132340.3
136714.5	88195.6	113328.8	152078.4	189839.5	135512.2	238486.5	121688.6	224305	131808.7
218288.6	87342.4	193615.3	179176.4	204843.9	96822.49	213439.2	116221.4	190302.8	158158.4
222359.7	124870.9	189075.1	172077.7	182168.8	108081.9	248761.1	86953.47	234537.5	144799.6
204364.9	84738.48	191330.3	158840.6	178024.8	100948.2	254909.6	128028.1	145542.5	186144.8

LP401.178	LP401.178	LP401.178	LP401.178	LP401.178	LP401.178	LP401.178	LP401.178	LP401.178	LP401.178
96013.99	159210.3	178653.3	204390.5	145812	143497.8	118673.9	61504.54	125728.4	153353.9
119672.7	84661.47	127060.8	173471.6	123649.4	190931.3	89032.54	85807.86	161377.4	148377.3
106440.7	151688.5	165143.7	166696.7	203971.9	167783.1	104151.7	64792.24	146347.1	113219.4
81457.62	101869.3	213521.8	278271.1	152156.7	177821.2	124539.2	53570.12	160825.6	109346.8
99277.82	123908.2	143517.1	204457.4	241224.2	241471.2	172308.1	56533.67	131539.7	162299.4

LP401.178	LP401.178	LP401.178	LP401.178	LP401.178	LP401.178	LP401.178	LP401.178	LP401.178	LP401.178
109201.9	46275.59	95937.8	194706.6	154879.6	126695.7	53425.8	96560.3	158628	63000.63
136380.9	48176.69	173984.5	146328	217893.9	191459.6	67493.28	107003.7	132197.8	55178.45
101430.7	46278.52	156585.4	168622.5	178434.6	127778.3	58020.74	94295.9	144054.5	42233.08
147030.3	45621.46	163525.5	213566.4	136762.1	110808.6	48610.6	115565.9	102461.4	42433.23
94614.24	63247.26	127830.7	174095.6	159878.3	120365.3	55497.42	131841.6	121757.1	62884.08

LP401.178	LP401.178	LP401.178	LP401.178	LP401.178	LP401.178	LP401.178	LP401.178	LP401.178	LP401.178
173429.8	60731.53	186293	160150	59490.48	69176.43	55016.37	136918.8	47097.08	58088.59
182216.4	47610.3	170401.2	208989.3	43197.31	74657.62	48675.26	114555.5	61178.03	61701.91
161521.2	51258.8	197736.1	167490	69491.09	59782.15	72359.25	103800.9	47452.73	56307.21
165497.3	49050.97	134361.2	167223.5	55446.9	70353.22	42983.33	100282.3	47833.8	48036.86
142869.7	55974.03	239064.4	109936.5	50085.53	64215.68	46644.1	124269.2	66194.19	55166.69

LP401.178	LP401.178	LP401.178	LP401.178	LP401.178	LP401.178	LP401.178	LP401.178	LP401.215	LP401.215
123551	152202.8	136391.7	44119.09	139398.4	58668.35	90444.43	183765	71209.87	57120.3
156551.7	157193.8	115527.1	52682.52	210982.8	67742.06	82257.34	167746.1	71580.48	29475.1
131216.4	205225.7	179074.2	40750.48	209383.9	57402.28	59192.85	187703.7	62277.7	54483.63
168147.1	167072.8	144399.5	37991.32	214052.6	77676.7	68651.79	130369.5	55525.75	51536.63
138528.7	128937	115816.5	46116.22	191186.9	69560.45	75313.87	191989.5	61437.45	57672.49

LP401.215[illegible]	LP401.215[illegible]	LP401.215[illegible]	LP401.215[illegible]	LP401.215[illegible]	LP401.215[illegible]	LP401.215[illegible]	LP401.215[illegible]	LP401.215	LP401.215[illegible]
123187.9	86824.25	107571.6	39021.9	52192.7	67225.76	93345.65	147389.9	155092.6	49344.79
137221.3	86632.43	111661.9	36166.12	40335.68	72258.34	97182.83	149292.7	57079.42	38493.88
131159.9	75811.61	114788.1	37805.69	38407.6	65082.73	89152.09	141275	172847.3	38788.66
128782.5	81360.06	99297.28	35878.68	35592.72	66806.15	92471.04	106562.2	169888.1	32828.57
88893.7	94828.79	98932.31	39867.98	45016.14	65113.83	98586.04	136018.5	171361	40955.55

LP401.215[illegible]	LP401.215[illegible]	LP401.215[illegible]	LP401.215[illegible]	LP401.215[illegible]	LP401.215[illegible]	LP401.215[illegible]	LP401.215[illegible]	LP401.215[illegible]	LP401.215[illegible]
118107.1	98363.3	49540.74	110594.9	42383.97	125564.2	73033.57	103107.8	119692	176440.4
117642.1	59487.72	49337.84	109906.5	40616.44	142026.4	81467.39	104289.3	120881.8	178101.1
128473.8	97574.53	51059.64	105868.9	42496.47	134388.9	75405.51	92677	121482.2	179404.8
114248.5	97285.51	45476.93	98880.8	35853.32	137035.1	67526.82	94483.97	117606.6	176439.3
118214.3	102918.4	36304.19	105901.8	39601.05	136587.5	78547.26	95277.41	132250.3	171327.9

LP401.215	LP401.215	LP401.215	LP401.215	LP401.215	LP401.215	LP401.215	LP401.215	LP401.215	LP401.215
115570.6	182147.1	153081.8	142188.5	184910.8	124057.4	159105.5	86983.38	182814.2	78412.3
110079.6	169333.5	156528.2	157922.6	175349.9	120085.8	161903.5	96604.52	193587	59108.01
108192.5	170433.1	157541.8	163191.7	180361.3	124557	158894.9	84375.89	168986.7	79938.5
108352.6	170900.8	153368.3	148890.2	189407.8	132712.4	178141.8	88868.47	183331.3	78848.05
118307.5	181359.8	160781.3	147265.5	186199.9	127576.4	132604.2	86707.28	173689.8	89231.56

LP401.215	LP401.215	LP401.215	LP401.215	LP401.215	LP401.215	LP401.215	LP401.215	LP401.215	LP401.215
42244.58	128093.2	136537.6	170623.5	120892.6	80361.81	136690.9	155479.7	68295.21	135670.8
44850.42	129762.7	137659.4	116699.2	127809.9	80108.6	141855.5	154578.2	66806.24	134049.8
39281.95	138705.4	138911.6	185791.5	126274.8	67696.47	135153.3	148180.6	58175.71	138562
39415.04	137940	137307.4	170233.5	116885.6	78473.05	135531.9	141382.2	61320.24	144807.6
43412.97	134987.3	134477.6	178689.8	120352.2	76623.17	145003.5	156594.3	67154.27	142618.4

LP401.215	LP401.214	LP401.215	LP401.215	LP401.215	LP401.215	LP401.214	LP401.215	LP401.215	LP401.215
117602.1	117301.7	146690.6	122445.5	184731.6	49711.28	102306.7	215433.9	82834.63	120091.8
122161.4	111452.7	143181	106175.4	176181.5	54801.13	103621.7	213163.1	78523.69	123217.6
118157	118835.6	128275.2	115404.5	176481.9	49565.45	91410.96	193176.6	78743.69	121823.4
115348.6	121827.6	132791.4	122781.7	175036.6	44684.74	86323.27	214001	73618.19	125459.1
118713.3	122930.8	145911.4	117325.3	172618.3	48796.23	91824.58	210645.2	82995.11	123676.2

LP401.215	LP401.215	LP401.215	LP401.215	LP401.215	LP401.215	LP401.215	LP401.215	LP401.215	LP401.215
85763.48	148330.5	110376.6	142878.8	114096.8	89127.25	123535.9	237266.9	50838.63	57658.21
86719.7	153829	117836.5	161311.6	113949.5	81320.91	130335.2	225105.9	49158.09	56541.48
90775.41	148217.4	109209.5	156858.9	110946	89712.3	125983.4	249727.8	43948.95	49767.26
86864.66	93507.57	106458.5	174333	109553.4	88895.2	127919.5	239477.8	42119.98	44475.46
90086.46	156185.1	116984.8	162011.1	115015.9	96647.46	124987.4	231236.3	44642.69	51593.21

LP401.215	LP401.215	LP401.215	LP401.214	LP401.215	LP401.214	LP401.214	LP401.215	LP401.215	LP401.215
100249.5	137236.3	123590.1	104693.4	241731.1	91687.04	160136.5	99985.3	156818.2	154602.2
99950.36	128777	136969.4	109872.5	230427.7	108954.1	158317.3	104614	164330.6	164138.5
100433	135808.2	116641.7	104787.1	236030.8	109751.9	159656.8	107384.8	168105.4	156639.3
106123.8	147351.8	113460.9	106556.3	243812.9	102667.3	165002.3	108859	170378.2	140503.8
99109.5	129328.1	110172.9	113674.4	252371.8	102632.6	167775.7	110114	163571.7	150824.9

LP401.247	LP401.341	LP401.345	LP402.248	LP402.248	LP402.248	LP402.248	LP402.248	LP402.248	LP402.248
77663.55	56658.04	41613.39	46911.88	43753.25	38823.56	77641.28	75758.17	57775.12	53146.13
80579.25	50207.22	43841.77	42209.32	40256.95	30717.91	77906.61	75482.53	49624.37	53355.76
66837.29	52593.57	37504.81	51699.68	44793.39	38995.3	74092.32	80382.95	54506.13	50952.58
59526.83	39052.54	36652.74	42882.62	37853.12	38283.68	80545.72	71048.95	55815.76	54930.42
65597.77	48826.4	32523.46	44155.86	41590.18	29382.09	78198.34	72385.68	53931.97	56913.69

LP402.284	LP402.284	LP402.284	LP402.285	LP402.284	LP402.285	LP402.285	LP402.284	LP402.285	LP402.285
105752.7	100286.1	86821.45	92163.34	58120	83597.74	89408.25	82674.8	77259.8	67362.29
78354.42	92757.78	78658.33	90258.84	91975.91	81084.42	76548.55	85231.19	79490.39	61185.49
119854.9	85674.58	60200.59	122665	92006.37	147595.1	77514.36	68963.59	73302.02	57766.51
89555.54	136534.5	76390.3	71984.12	105284.3	85726.07	79311.92	87371.6	56357.38	65135.81
148900.7	117233	94205.01	75446.87	65833.87	80316.95	87327.76	85420.56	71007.6	66257.43

LP402.285[illegible]	LP402.285[illegible]	LP402.285[illegible]	LP402.284[illegible]	LP402.285	LP402.285[illegible]	LP402.285[illegible]	LP402.284[illegible]	LP402.285[illegible]	LP402.285[illegible]
93097.81	75034.16	83977.24	74100.98	123982.4	92536.89	122040	74679.11	60200.92	70105.17
121754.3	74799.76	84660.79	135748.9	106065.1	92191.02	111396.6	81444.02	64498.01	72598.96
87249.94	84978.31	80791.39	74051.53	117366.3	93197.74	95096.08	81759.79	69884.14	69540.34
92319.55	70569.27	71319.15	78207.03	109446.9	88527.93	98750.15	69456.99	58579.69	69636.47
84336.9	73104.58	86738.52	77002.51	113158.3	93664.85	120198.4	90364.16	77089.88	71423.45

LP402.285	LP402.285	LP402.285	LP402.285	LP402.285	LP402.285	LP402.285	LP402.285	LP402.285	LP402.285
90052.9	85345.88	57291.7	82481.92	88677.93	119650.4	71039.46	95190.62	84009.34	69570.06
87222.39	54764.71	66388.39	89786.98	88318.44	109066.9	73966.4	73696.83	79931.39	84846.1
86929.94	64595.07	65268.09	97058.32	87514.46	102360.2	74621.56	74013.22	74586.15	86888.67
85901.61	53437.73	65433.9	82597.64	83780.05	116697.9	82574.16	76315.15	72935.2	69656.16
92675.98	55152.5	66036.14	81251.85	85349.04	105234.5	75284.27	73918.44	70424.2	59068.41

LP402.285	LP402.285	LP402.285	LP402.285	LP402.285	LP402.285	LP402.285	LP402.285	LP402.285	LP402.285
83050.25	86924.58	59639.76	84881.11	67864.46	67497.23	83977.94	84556.42	65325.05	79079.04
85730.63	79686.73	57939.94	75220.16	70668.17	61831.03	82590.6	91783.85	64544.84	85220.68
78525.67	70972.54	67432.91	77269.92	81026.83	57327.85	82973.54	82468.21	66065.99	90859.77
83749.82	81095.86	58541.64	73525.28	72485.84	76651.79	86713.85	84874.21	77017.73	76565.08
83118.24	84814.66	58156.37	80445.39	60216.24	61274.93	76295.08	86677.3	73046.7	87574.16

LP402.285	LP402.285	LP402.285	LP402.285	LP402.284	LP402.285	LP402.285	LP402.284	LP402.285	LP402.285
74731.53	98296.04	48029.07	49440.17	97745.51	118308.5	77146.67	96771.01	79004.11	75453.6
76057.73	102992.2	48297.36	51920.29	92115.72	117960.2	79355.45	105684.9	80446.98	74874.84
73301.43	105226.5	54135.42	47131.97	94579.12	122652	78947.2	105089.9	78249.62	74506.45
75993.31	101380.1	46869.01	48417.05	107790.5	111631.9	72636.48	100890.1	79264.15	75974.45
73030.91	98825.08	44561	52190.51	88740.41	114396	80003.32	103272.4	77748.86	73385.99

LP402.285	LP402.285	LP402.285	LP402.284	LP402.285	LP402.285	LP402.285	LP402.285	LP402.285	LP402.285
59129.09	59880.34	84750.22	65589.98	63642.42	62544.9	49154.39	74091.69	85582.58	52591.81
60039.85	59641.45	84105.09	50781.23	64601.06	69228.85	49583.38	71534.45	86770.37	46687.78
57598.82	55752.11	81569.3	51231.57	55985.6	74747.59	51597.59	71840.07	82492.51	51019.34
62217.94	60836.96	86720.89	53057.02	67971.16	62007.02	50480.67	68759.1	79800.83	53793.25
52716.21	55941.49	87636.39	52783.17	55046.02	60604.77	51446.16	66072.77	84880.12	44930.13

LP402.358	LP403.193	LP403.194	LP403.194	LP403.194	LP403.193	LP403.194	LP403.194	LP403.193	LP403.194
98900.28	351500.2	250367.6	201914.1	232500.1	240111.5	347039.7	457976.2	222143.8	437655.3
104902.9	280945.1	218320	244211.9	231815.5	221028.3	284101.8	347177.5	234467.6	237356.5
107098.6	331757	223041	156274.1	176863.1	285543.7	345697.6	365870.9	193756.7	248720.8
91724.93	372819	188546.3	288552.6	208014.3	202949	392984.4	349433.2	255434.3	339419.8
99720.21	297978.5	236854.9	217235.2	216904.3	345935.7	363663.5	425790.7	243442.2	360769.4

LP403.194	LP403.193	LP403.193	LP403.194	LP403.194	LP403.194	LP403.193	LP403.194	LP403.194	LP403.194
427321.6	261143.6	228835.8	349472.8	318424.3	182251	328990.2	234891.6	272904.3	481991.4
256761.2	265249.6	352874.2	498724.9	378591.6	221790.4	336384.9	186056.1	274785.1	260926
340687.9	257643.5	336577	350708.2	413862.7	234749.6	435627.7	333452.5	249392.3	345808.6
254265	263010.2	384248.9	396963.4	510857.8	205790.5	279216.3	221315.8	222900.9	331791.4
351776.4	299890.4	265386.6	358149.2	246202.6	215315.4	316252.6	370019.4	245684.9	345581.3

LP403.194	LP403.194	LP403.194	LP403.193	LP403.194	LP403.194	LP403.194	LP403.194	LP403.194	LP403.194
299703.6	461429.8	352759.6	199345.7	325822	266942.8	148753.1	315370.2	210809.6	335838.2
258120.7	308776.7	390933.1	233934.3	389186.4	370118.7	183935.3	295534.9	262317.1	421760.1
297644.9	305199.6	328378.3	217292.6	282435.1	272178.8	176079.9	312137.7	219693.7	404444.4
293227.5	499762.8	390079.4	177498.8	406421.2	312725.6	231182.7	445299.2	239016.9	319881.3
227204.1	359662.1	382061.5	200863.9	346898.8	291314	152267.6	292324.2	276228.2	415329.4

LP403.194	LP403.194	LP403.194	LP403.194	LP403.194	LP403.194	LP403.194	LP403.194	LP403.194	LP403.194
339232.7	488364.7	362385.4	317379.8	361851.1	305956.9	388187.6	199882.9	81767.85	270394.4
276117.8	323276.9	329162.2	253152.8	236006.6	293960.2	300798.7	216498.2	65856.68	276199.4
376903.7	395033	267487.4	223839	352252.4	363653.2	343618.2	198073.6	64260.18	259294.4
344863.8	424594.2	306281.3	227728.8	337194.3	367766.5	395635.5	204939.1	89810.41	443156.8
344614.8	414962.6	324812.8	234303.2	348910.8	290914	597107.4	183573.6	50509.36	311515.9

LP403.194	LP403.194	LP403.194	LP403.194	LP403.194	LP403.194	LP403.194	LP403.194	LP403.194	LP403.194
364242.3	160842.5	319052.5	405990.1	173888.3	291503.4	353629.9	195370.5	213231.8	251669.1
288651.8	151108.7	260746	245953.6	319027.6	248488.9	469134.9	181023.2	214561.1	256549.3
313053.4	183302.4	472446.9	364503.2	217879.8	310659	488391.9	153534.8	232617.3	217122.1
292808.1	149381.3	417115.7	361952.9	149239	278183	338636	194548.5	210394.8	292957.5
287622.5	188563.1	235646.4	289443	131081.1	367168.3	381043.6	224583.9	214835.9	296149.3

LP403.194	LP403.194	LP403.194	LP403.194	LP403.194	LP403.194	LP403.194	LP403.194	LP403.194	LP403.194
233952.7	314621.1	158849.2	229273.7	169416.2	231944.2	184239	304501.2	212438.4	162890.2
236588.8	256739.6	255880.6	250947.3	163845.5	232761.1	229231.4	309705.9	244916.9	174722.7
308420	408044	218749.3	375536.2	127354.7	245710.6	192200.7	275364.7	253419.6	229715.5
264375.8	234411.7	215699	187056.6	143488.9	217545.1	283122.4	239066.8	246728.6	139868.5
226601.7	296808.4	173747.1	317859.8	183494.6	216335.3	252977.2	323113.5	264234.9	202492.1

LP403.194	LP403.194	LP403.194	LP403.194	LP403.194	LP403.194	LP403.194	LP403.194	LP403.194	LP403.194
220638.8	293592.3	151466.8	124165.9	155459.8	253836.4	163009.6	189006.7	173582.4	158765.9
203745.3	326200	173029.4	121105.8	177289.7	280054.5	174490.8	212977.6	169820.1	163701.6
196495.2	308181.3	145163.1	132252.9	255612.2	188994.5	167508.6	185476.8	154024.1	184839.3
217842.4	353978.3	126373.1	149475.8	187050.2	286653.8	236067.8	160099.1	160150.3	169473.2
252008.9	302416.7	205989.7	119298.3	171161.3	264490.6	162192.1	191704.3	172793.9	176185

LP403.194	LP403.194	LP403.194	LP403.194	LP403.194	LP403.194	LP403.194	LP403.194	LP403.194	LP403.194
91700.58	84820.72	89303.16	71049.54	71229.1	201472.9	58228.52	93157.69	57420.92	83946.16
104639.1	77525.26	62696.73	56890.23	71406.16	203219.2	59574.68	83135.53	62374.27	93876.03
88132.54	96411.34	60537.67	86930.57	63455.31	193602.1	53030.94	98183.1	65569.54	84470.07
56412.08	75951.47	91351.06	52878.53	58339.92	142999.9	68476.55	65178.48	48736.12	73151.29
79856.44	94041.64	81240.39	70994.6	64591.67	211238.5	58409.86	92219.42	51330.87	79591.48

LP403.194	LP403.194	LP403.232	LP403.278	LP403.354	LP403.353	LP403.353	LP403.353	LP404.197	LP404.197
87463.89	70552.41	80571.72	15425.32	37916.1	57933.14	53510.58	44715.01	45023.37	63443.85
93565.34	75380.78	119969.1	15327.27	39774.78	53896.72	50745.92	42699.86	38106.33	53271.43
103607.3	67492.05	99435.24	17653.65	37897.71	53339.53	46809.59	41876.58	40202.54	55826.69
84486.57	56214.36	106732	16236.25	41215.43	56422.77	48538.97	39576.24	45003	64256.82
78426.05	104610.1	78562.54	17114.21	40514.44	59817.79	49885.84	36082.79	50324.19	54260.24

LP404.197	LP404.197	LP404.197	LP404.198	LP404.209	LP404.263	LP404.264	LP404.264	LP404.264	LP404.264
56664.98	45449.93	38999.27	36707.96	36962.48	142167.7	112636.5	99421.28	130529.7	120657.2
59198.86	48562.99	50410.6	32876.11	22567.86	130080.8	112528.1	150725.6	115622.5	125312
56910.37	49221.81	49547.31	32520.73	35285.6	112507.8	117078.4	131409.7	130574.8	129873.5
54414.48	40713.96	47524.16	35454.78	25094.81	126023.3	113924.8	124625	142052.6	153962.1
60396.34	48129.76	47034.44	32802.57	33392.94	154417.1	120133.7	90756.72	115540.9	122281.1

LP404.264	LP404.264	LP404.264	LP404.264	LP404.264	LP404.264	LP404.264	LP404.264	LP404.264	LP404.264
142382.4	87249.84	105055.8	96237.5	126283.2	116595.4	118711.1	96538.77	113724.6	99127.21
110318.6	79196.37	98543.45	104491.5	132081.6	107876.6	108005.3	82363.98	110388.6	93333.78
115115.6	90927.93	124075.4	102738.7	106740.2	114553.6	74930.67	84232.67	110740.1	128661.8
100684.6	85129.21	104017	98350.84	161573.9	152742.7	85111.43	91898.16	111745.9	109277.5
104271.5	76830.68	98846.87	96086.42	139631.4	105129.8	73710.15	82897.58	116641.7	91267.27

LP404.264	LP404.264	LP404.264	LP404.264	LP404.264	LP404.264	LP404.264	LP404.264	LP404.264	LP404.264
119659.7	114089.7	104628.1	126270.6	100209.2	74339	84540.25	115018.2	98652.56	103772.5
106143.3	105407.7	98734.67	103916.5	108299.6	74050.15	88523.46	115745.5	97684.49	97235.55
109805.9	106892.2	99846.45	122077.1	105027.9	85940.35	92444.01	106174.2	84954.2	102980.2
117765.6	107102.6	96345.72	106379.7	108216.1	71258.15	83093.96	94685.98	81207.9	102844.4
106753	103056.8	98947.84	112621.7	105772.5	75518.23	97120.53	132062	93258.61	98964.02

LP404.264	LP404.264	LP404.264	LP404.264	LP404.264	LP404.264	LP404.264	LP404.264	LP404.264	LP404.264
85664.65	102031.8	96525.89	82813.86	108270.9	96565.9	86864.41	65790.62	75763.27	95719.49
84244.58	126629	96878.69	83960.21	103437.2	100761.9	88337.85	73674.66	79111.68	98290.8
84890.79	106925.6	95003.3	83594.22	101174.7	86415.32	90740.98	65571.75	75400.19	104277.2
91746.36	110246.6	80442.87	77213.83	99163.26	98432.31	86971.92	72644.76	73375.4	105997.3
94008.24	111175.2	88602.31	84413.76	99386.22	96566.92	89419.1	64955.45	67597.04	91233.37

LP404.264	LP404.264	LP404.264	LP404.264	LP404.264	LP404.264	LP404.264	LP404.265	LP404.264	LP404.264
99300.69	99142.94	110569.9	82779.67	101409.9	114971.6	87232.02	98116.82	114865.1	90510.83
103452.7	93658.44	115214.8	79281.2	98624.16	106464.5	87862.06	102176.6	123492.8	81143.84
109540.8	108770.7	111575.2	79087.72	100466.8	120565.6	92149.78	96110.81	122919.9	75909.23
101280.1	109375.1	107446.7	81277.14	101398.9	78857.15	89544.68	97390.67	124400.7	83116.72
92965.23	99297.42	99406.04	79810.44	91194.96	117194.3	89735.38	107901.6	120251.6	84610.34

LP404.300	LP404.300	LP404.300	LP404.300	LP404.300	LP404.300	LP404.300	LP404.301	LP404.300	LP404.300
128444.9	116901.2	121925.7	137672.2	103055.3	111506.6	139292.7	126303.3	170795.7	115447.3
114675.2	100562.3	115867.2	97903.94	94086.17	81007.83	105443.1	129838.6	89148.06	85121.94
139004.7	107923.3	121881.4	129093.5	93589.58	133124.9	131792.3	133317.5	129797.7	114467.2
117073.6	105930.7	119906.6	121686.4	79431.02	123665.7	142156.8	127279.7	117321.8	100402.5
103241.3	114490.6	105978.4	119325.8	91597.69	111236.5	101992.3	84877.17	131612.4	103703.1

LP404.300	LP404.300	LP404.300	LP404.300	LP404.300	LP404.300	LP404.300	LP404.301	LP404.300	LP404.301
104937.8	142447.6	80993.41	134605.7	81787.03	116500.8	70991.12	125682.7	88631.17	81457.24
83960.11	83692.65	72790.25	123688.7	94906.22	120555.1	99055	92712.21	97006.24	88347.15
116832.5	149303.9	104746.2	103495.5	105762.2	101185.2	109459.9	95424.28	94995.54	77191.09
126353.1	148227.2	101502.2	129668.9	114650.2	131826.2	123675.1	88806.69	85029.54	80857.84
126949.8	149373.8	68316.62	115086.5	89676.61	116412.7	116294.2	96828.21	108995.6	78976.36

LP404.301	LP404.300	LP404.300	LP404.300	LP404.300	LP404.300	LP404.301	LP404.300	LP404.300	LP404.301
82271.03	87202.63	67345.5	95824.69	95697.33	92700.87	111754.4	67265.43	91422.65	107201.5
74698.62	93444.44	78307.52	96031.8	154004.7	101159	69556.39	59419.86	79989.09	116893
83536.9	95923.61	79440.89	105151.8	108596.2	64790.54	118310.4	64314.42	92674.6	132610.6
81030.21	96297.8	87786.08	119903.2	123654.4	99315.15	120494.6	65527.35	89722.97	125364.5
84864.4	69688.62	81305.93	89974.69	132098.4	94474.61	99303.32	60289.58	90908.89	120174.9

LP404.300	LP404.300	LP404.301	LP404.301	LP404.300	LP404.300	LP404.300	LP404.301	LP404.301	LP404.300
89869.08	120953.4	78161.3	132243.3	99367.53	132691.4	116281.7	74939.59	72771.1	72733.8
74277.9	84067.34	68739.02	79324.53	89658.4	122006.4	120837.3	75556.8	91050.33	69959.45
80858.81	78781	85040.64	134281.9	99100.3	137742.3	115560.3	73526.77	82726.71	78150.17
77941.92	98183.54	91113.19	141286	94628.05	134046.8	117992.3	65631.57	90428.42	70970.97
91558.5	88348.75	93491.98	121181.1	64530.41	103278.8	136135	77877.72	93290.15	76179.61

LP404.301	LP404.301	LP404.300	LP404.300	LP404.300	LP404.301	LP404.301	LP404.300	LP404.300	LP404.300
94998.32	64776.95	85662.02	103273.2	86850.91	80265.7	69684.67	68664.96	113992.7	144594.7
71009.14	51162.04	65445.28	76195.83	58929.63	62718.06	58192.97	60474.11	87205.76	78913.64
101849.4	64496.04	84444.01	106036.2	91951.42	81056.56	84142.52	70866	119700.5	131653
99748.36	54417.29	89262.94	95948.25	99602.63	83039.81	82550.94	65690.09	118642.8	126860.6
96420.12	91787.98	76334.75	101622.3	69118.56	85036.44	36938.8	69453.99	85317.32	139688.7

LP404.300	LP404.300	LP404.300	LP404.300	LP404.300	LP404.300	LP404.300	LP404.301	LP404.300	LP404.300
82522.14	113880.3	77912.9	61299.54	117367.7	112104.5	123145.7	114151.1	112698.7	71891.62
55474.96	96156.66	51837.97	35300.18	110763.4	49580.02	94744.7	119238.9	89847.26	62839.24
86477.14	122208.2	79522.09	65490.24	121389.7	60280.73	134032	67015.9	107038.7	70717.12
80865.29	106568.2	71499.09	63937.39	121305.1	112524.2	136091.3	113401.1	102386.3	73263.31
81056.7	103035.9	75031.18	67776.73	107365.2	117296.9	58062.47	112401	97404.8	67892.83

LP404.300	LP404.301	LP404.301	LP404.300	LP404.300	LP404.301	LP404.300	LP404.301	LP404.300	LP404.301
67128.19	88116.8	80977.9	84493.93	87097.59	102137	61740.38	74340.91	106757.1	85010.13
54268.69	67268.53	53514.45	43373.37	74919.4	93821.83	55089.92	45542.75	94278.45	85468.99
68969.52	97149.97	94143.67	90902.83	86224.76	102762.7	73749.28	80305.5	106199.7	82006.67
63257.62	84189.33	79363.82	95624.51	88965.85	104017.5	64073.67	84305.34	105964.3	77368.21
60881.58	84503.98	84626.62	81799.27	83644.13	98498.41	67876.88	73997.45	97157.47	75504.52

LP404.300	LP404.300	LP404.301	LP404.300	LP404.301	LP404.301	LP404.300	LP404.337	LP404.337	LP404.373
97383.48	117043	107458.7	98759.91	115183.9	71813.03	87610.75	127384.9	122781.9	219260.2
102111.5	67822.29	60496.6	94255.12	65994.23	52265.09	58274.98	135853.5	120995.6	232691.9
99832.2	120012	102667.5	102153.4	116330.7	76055.48	92162.18	132171.1	117949.2	260164.9
101255	115400.1	104759.2	101199	114866.1	73763.05	92954.44	132655.4	119212.7	266961.5
95613.64	116877.9	97988.65	92965.23	112504.1	78415.45	87962.27	125439.4	115142.4	273627.8

LP404.373	LP404.373	LP404.373	LP404.386	LP404.451	LP405.210	LP405.211	LP405.209	LP405.279	LP405.301
58505.74	60630.38	142846.4	22071.11	40854.55	25935.38	30525.43	11377.32	49801.34	38499.32
56659.93	69171.62	146430.2	26516.24	46384.74	33291.23	30632.52	14402.92	48923.44	43029.18
60946.14	74458.55	137154.2	24219.33	42702.9	28665.91	35907.55	13555.13	63381.6	40836.19
62471.5	74383.55	127896.3	21144.34	30192.6	30166.22	25346.6	14043.4	20433.1	34042.56
59941.36	69238.03	127390.7	25210.88	34977.46	30683.2	21261.01	13738.84	61381.83	36338.79

LP405.301	LP405.302	LP405.301	LP405.335	LP406.243	LP406.243	LP406.243	LP406.243	LP406.244	LP406.243
48122.45	37012.29	35984.02	40099.74	79060.61	75030.68	90127.65	69203.67	60730.35	62656.67
48304.29	41415.31	45019.46	40154.22	81432.89	67628.9	97477.75	75924.44	54421.08	58053.09
46387.76	36822.92	38223.66	39538.07	77082.71	72559.97	88757.09	64516.16	56571.75	56373.87
46547.6	34954.74	40566.76	36483.18	76760.06	74331.07	92365.9	71780.91	54525.53	66670.32
45195.26	40862.22	40521.8	37172.1	74763.6	83938.03	88769.44	66572.96	56509.15	54844.34

LP406.243	LP406.244	LP406.244_	LP406.243	LP406.279	LP406.279	LP406.280	LP406.280	LP406.280	LP406.28_
82621.9	72309.7	47960.87	73060.24	277214.1	247293.9	269847.2	207543.3	197404	195725.3
83078.27	67886.22	48058.22	69288.03	242816.7	320279.1	215812.9	204974.4	208378.2	297068.3
85901.32	73003.34	57446.82	69377.33	227020.5	252824.6	325820.3	201688.7	191695.5	234142.9
80008.55	67174.38	52795.03	67570.23	223542.3	330426.3	186371.5	299323.2	207788.4	306628.3
74888.89	65866.17	55815.07	62624.91	256442.8	371904.7	334886.9	207956.8	261113.3	232624.9

LP406.28_!	LP406.280	LP406.280	LP406.279	LP406.280	LP406.280	LP406.280	LP406.280	LP406.280	LP406.280
243758.1	235814.5	269070.7	203338.5	210320.3	199471.8	116624.9	198196.3	240441.5	201647.6
194261.6	196528.1	278498.5	173649.4	175866	232069.4	157553.3	271885.6	306383.7	193246.5
229541.7	220214.8	231544.9	200105.6	200390.5	197447	163747.4	199726.8	211621.4	222466.6
259566.4	202962.5	278837.5	206451.4	165285.2	208238.4	159223.3	243208.4	285734.2	275561.3
219717.3	225049.6	287687.5	204397.2	166492.9	225791.3	145419.5	223415.1	210401.9	260101.7

LP406.279	LP406.280	LP406.280	LP406.280	LP406.280	LP406.280	LP406.280	LP406.28_	LP406.280	LP406.280
254868.9	220790.1	232457.6	235019.9	201699.2	188645.4	239028.4	210168.4	151677.9	252482
195200.7	220813	212624.1	222636.2	222885.5	204288.6	246254.1	259648.6	165944.9	235417
250739.6	185415.5	253975.7	299038.7	200957.3	179858	184726.4	203302.9	167257.4	211690.8
218568.8	242427	219081.4	231982.6	207807.8	251650.2	205179.1	206572.3	168778.8	247456.7
245162	229360.3	230224.2	243790.5	228223.4	187008.8	306826.2	233251.6	168308.7	249278.4

LP406.280	LP406.280	LP406.280	LP406.280	LP406.280	LP406.280	LP406.280	LP406.280	LP406.280	LP406.280
305442	279447.5	203314.2	244882.6	239152.8	237348.4	256181.2	180616	214911.7	167585.3
267579.4	246325.4	194257.2	331475.9	240468.5	238926.2	281583	207936.6	223780.4	183637.3
201337.5	260625.7	205146.5	216499.8	230106.7	261495.7	195513.3	229370.9	288157.7	186394.9
286836.4	274981.4	188103.9	249111.9	243611.8	245347.3	258672.4	256501.6	201417.4	234716.9
217748.9	259152.5	273265.4	203011.6	248884.7	255356.7	167549.9	212129.6	188603.4	280815.8

LP406.280	LP406.280	LP406.280	LP406.280	LP406.280	LP406.280	LP406.280	LP406.280	LP406.280	LP406.280
216306.9	217956.1	209553.6	188399.9	220457.3	224439.7	220514.3	216483.9	204292.9	208472.9
182949	215414.3	248857.8	199910.8	238916	240630.3	184302.3	211294.2	187525.2	239032.6
190364.5	258334.3	215870.6	205128.8	215448.4	210780.5	244585.8	234119.5	193490.6	192936.4
131179.8	188366.6	202500	211038	211428.1	233081.9	202844.9	198690.9	172797.8	183877.1
168687.5	249206.4	187622.2	211995.1	253317	276480	180478.7	201950.2	204318	214380.2

LP406.280	LP406.280	LP406.280	LP406.280	LP406.280	LP406.280	LP406.280	LP406.280	LP406.280	LP406.280
181650.5	236988.8	264352.6	211047.3	252888.2	228172.9	218635.4	166527.8	186801.3	255505.6
191204	222619.1	225164.1	214948.6	255945.4	285348.6	230720.9	209403.7	208771.5	248077.5
210871.4	198746	232178.5	211818.7	235906.2	344871.3	198060.8	142439.1	232868.1	247550.1
214752.3	214170.8	202961.8	207621.3	479991.3	298054.8	218682.7	203252.2	175249.7	250358.4
187086.1	215910.8	221068.6	315382	243269.5	268865.3	252541.7	234943.5	212642.5	214234.8

LP406.280	LP406.280	LP406.280	LP406.280	LP406.280	LP406.280	LP406.280	LP406.280	LP406.280	LP406.280
225989	204501.3	211168	360021.2	132456.4	208275.4	159897.6	221941.4	248926.8	187348.7
242130	202226.2	205588	202427.6	135465.2	208407.2	158200	244544.1	220241	179725.3
243287.9	229169	196627.7	244862.1	112549.6	231705	167649	169520.5	227374.4	178333.8
235025.5	254166.6	209158.3	230261.5	133402.7	231810.5	151014.5	232520	182845.1	174943.7
203313	213188	239497.5	211966	148495.6	228121.9	139407.6	267266.8	210033.3	168132.3

LP406.280	LP406.280	LP406.280	LP406.280	LP406.280	LP406.280	LP406.280	LP406.280	LP406.280	LP406.280
317862.6	227647.1	331080.7	208491.6	196839	129093.3	205109	126132.6	190746.7	143546
143331.2	229737	212741.6	181295	180077.4	141240.9	185500.4	134586.1	186456.3	109531.3
200938.6	228675.4	229519.1	327746.7	220421.8	161992	329037.5	121535.9	188386.3	128139.5
176703.6	230475.6	190883	192454.5	198078.2	163191.1	202455.2	121378.5	200810.2	92877.66
203545.8	161585.6	180845.9	210094.3	194768	149233.4	168708.2	173180.1	217820.4	96076.8

LP406.280	LP406.280	LP406.280	LP406.280	LP406.280	LP406.317	LP406.317	LP406.317	LP406.317	LP406.317
153431.6	131445.4	117652.7	161281.3	125172.9	182710.2	202909.8	142513.3	202344.2	198565.8
121614.7	119514.1	119166.5	199303.6	117571.7	174265.7	200738.1	150847.9	193664.1	197481.3
144544.4	127347.6	124501.4	151184.9	127028.6	182143.4	183854.3	130652	184815.3	198074.2
108655.9	126460.7	108199	160187.9	112984.6	177407.9	190583.6	130626.2	191265.7	189287
124725.9	111313.4	108549.4	147512.7	123368.3	166135.6	190038.9	139239.3	196776	187470.4

LP406.317	LP407.185	LP407.204	LP407.262	LP407.282	LP407.351	LP407.352	LP407.388	LP408.188	LP408.222
206473.5	176839.4	21059.78	56481.55	42876.89	38860.48	27181.58	33082.43	86482.11	102393.5
205385.1	140885.9	13069.02	44235.01	38597.26	34775.13	31052.77	30556.51	95200.25	94922.66
206315.7	224041.6	17188.89	50648.34	39321.08	33777.65	29651.21	30318.89	55137.42	94511.1
188682.6	174833.3	16036.7	23710.21	41945.71	32328.77	30502.88	28792.03	58472.49	77562.47
198870.4	151869.1	17818.73	50004.51	43427.98	36197.88	24424.17	28129.45	96543.33	95192.61

LP408.223	LP408.222	LP408.223	LP408.223	LP408.222	LP408.222	LP408.223	LP408.223	LP408.222	LP408.222
115067.6	46607.55	82999.91	135923.9	74271.78	73545.68	60193.82	63949.79	83985.11	61233.36
119934.6	65320.04	90317.4	116167.9	71796.07	68966.98	59001.25	70133.41	86299.83	57848.8
122344.2	45928.18	82601.67	126161.6	79227.49	69101.88	59281.62	77330.17	86696.66	63996.15
96467.71	56682.26	93522.4	113545.6	76628.45	68528.44	59151.67	70004.3	83179.5	58173.16
114300.9	50754.89	94092.56	135849	71742.11	60630.26	61008.03	75753.73	80597.29	53141.7

LP408.223	LP408.223	LP408.223	LP408.223	LP408.223	LP408.223	LP408.223	LP408.223	LP408.224	LP408.223
97006.42	71515.64	87302.78	76011.65	107253.8	73241.96	85852.09	83408.31	67027.47	69730.24
94972.75	72516.18	76559.32	76805.66	115905.8	67806.28	86349.61	80571.08	71171.09	75613.13
107310.5	73457.22	75595.74	76953.01	119349.6	67337.69	79602	77580.29	60176.21	79669.39
99545.9	109472.3	77424.08	74653.66	118936.6	68144.96	82792.7	71291.18	66677.4	78306.62
97232.26	71340.43	76956.05	75624.09	113244.4	67418.95	76562.17	78756.13	70431.78	76597.57

LP408.223	LP408.223	LP408.224	LP408.223	LP408.222	LP408.224	LP408.223	LP408.222	LP408.223	LP408.223
74454.72	96279.53	63791.37	67267.1	73172.07	78479.34	44282.07	58559.21	66311.89	66785.49
74180.39	92609.23	79172.3	59632.62	78616.97	84771.98	51420.22	55282.07	58534.86	69198.61
68067.75	97886.17	78330.4	66650.42	70116.1	90273.11	48595.53	57547.76	69842.19	65716.88
74562.68	89179.14	78103.5	61424.26	71600.56	78074.83	43347.69	58350.32	63749.33	73565.2
76266.15	90244.56	74962.65	62324.74	75721.72	89401.31	41725.49	59734.78	69594.47	65013.32

LP408.223	LP408.223	LP408.223	LP408.223	LP408.259	LP408.259	LP408.259	LP408.259	LP408.259	LP408.259
61612.47	66635.46	54258.84	130671.1	216361.1	125835.5	215647.1	340129.7	129347.1	115694.7
68375.52	59102.29	48397.52	128614	201486	139056.5	210301.2	120028	133547.5	117649.6
67365.88	62804.74	50312.86	135047.5	202278.9	141659.5	208765	339238.8	127615.6	111444.1
61041.1	62253.43	48704.96	129972.5	206882.1	145845.2	210041.6	352769.5	122404.4	118066.7
64304.23	75091.3	50993.07	132952.7	192449.1	148783	156540.8	335480.4	121095.3	92671.75

LP408.259[illegible]	LP408.259[illegible]	LP408.259[illegible]	LP408.259[illegible]	LP408.259[illegible]	LP408.259[illegible]	LP408.259[illegible]	LP408.259[illegible]	LP408.259[illegible]	LP408.259[illegible]
100719.4	155392.5	143157.4	194478.3	141027.8	198060.3	114211.6	241222.5	119516.1	113172.4
120696.4	155138.7	141626.3	188975.7	132233.5	207932.3	114902.6	211891.1	138974	136940.6
120200.2	154668.4	149467.6	209423.1	141554.6	186618.8	108633.2	213773.9	137706.1	140796.9
100763.2	154722.7	116316.4	161450.6	133430.5	189067.5	109787.9	230025.3	141982.7	146825.3
103886.1	140839	141193.4	202703	127338	179963.8	122075.2	243466.3	133960	135949

LP408.259	LP408.259	LP408.259	LP408.259	LP408.259	LP408.259	LP408.26_	LP408.259	LP408.295	LP408.295
214786	184421.4	132930.5	145964	156739.8	169434.1	126900.3	180767.6	151689.5	208612.6
210726.2	176445.9	125684.2	150525.5	173814.8	143801.7	138144.3	170699.2	152526.1	345372.3
198080.9	170309	128696.6	139944	170897.2	139066.4	130088.2	189678.7	127392.9	335848.9
196952.9	188010.1	141138.1	151403.7	150038.2	159856.9	141569.8	178149.2	204234.9	238252.1
207599.5	180934.6	132241	145609	158057.5	140553.4	133796.7	173946.6	186181.7	245124.5

LP408.295	LP408.295	LP408.295	LP408.295	LP408.296	LP408.295	LP408.296	LP408.295	LP408.295	LP408.295
194662.5	211399.5	316743.7	144902.2	221661.6	181395.4	245711.8	235411.7	355294.8	219084.8
200027.1	191366.6	371796.5	146556.7	245614	250625.2	236080.9	277075.7	254581.4	194814.8
172176.6	195612.6	281701	251965.8	217049.4	244975.9	349724	184629.7	269231.7	186768.2
240561.8	263014.9	318598.6	155598.9	259176.2	208824.3	257059.7	176343.6	273185.9	196440.8
152600.1	204870.1	248707.1	142870.7	379709.8	187528.4	265960.3	166173.3	557957.5	181623.8

LP408.295	LP408.295	LP408.295	LP408.296	LP408.296	LP408.296	LP408.295	LP408.296	LP408.296	LP408.296
245909.2	154662.6	158165.3	182944.3	303017.2	253801	178722.5	165110	195292	121663.9
306381.9	170758.8	176218.3	164192.6	182821.4	270170.2	171212.5	232079.3	149443.6	162496.4
490619.2	164560.9	142653.8	177502.8	169920.6	246747.5	149555	190501	223759.7	128068.7
249999.9	147923.4	172454.4	172889.4	194884.8	297807.3	170831.3	160823.8	161195.5	124204
227417.3	150829.3	180673.9	156154.1	197333.9	293857.5	162323.8	261318	218255.8	136082.4

LP408.296	LP408.296	LP408.296	LP408.296	LP408.296	LP408.296	LP408.296	LP408.296	LP408.295	LP408.296
159814	177461.3	140027.5	206615.4	191551	185017.7	186268.4	193335.3	283144	120448
215981.6	159810.4	129610.8	202540.4	129272.5	132380.8	210099.2	181588.3	155931.5	159381.9
187489.1	172152	113897	186927.1	160636.3	114209.1	150860.8	193722.4	131092.7	133178.3
180591.9	160360.4	136516.7	196679.5	140374.8	117895.7	182649.4	183543.5	171536.8	159532
238494.6	171276.9	129536.5	182004.1	161456.6	169215.1	213788.4	161650.6	181954.3	104568.3

LP408.295	LP408.296	LP408.296	LP408.296	LP408.296	LP408.296	LP408.296	LP408.295	LP408.296	LP408.296
185798.9	173876.5	142237	174269.1	143781.2	146661.2	204973.2	128941.3	117191.9	204035.3
200567	170073.1	140320.1	220277.8	156479.8	151256.6	190332.5	169919.3	132274.7	214429.9
166941.1	177050.3	162063.2	188351	152042.2	125853.6	188694	143376.8	138330.7	189215.8
173286.9	176014.5	144071.4	165736	154293.3	151242.6	199991.6	147302	127211.6	203022.8
161974.3	175705.7	160860.6	150680	151186	140394.5	186089.3	150083.8	127004.9	162396.7

LP408.296	LP408.296	LP408.296	LP408.295	LP408.296	LP408.296	LP408.295	LP408.296	LP408.296	LP408.296
212073.8	176986.7	228828.6	157207.8	171485.4	213062.5	150294.9	201903.3	143254.1	176659
210994.5	177477.5	115745.6	153483.2	148854.1	151245.6	117944.6	214021	163643.7	168740
177514.4	198030.7	101686.7	115416.9	171724.8	144450.2	141155.8	186367.7	171597.6	176427.8
195931.1	171896	112745.1	165087.4	184248.5	143726.8	169225.6	201638.4	157677.1	149004.4
247612.4	170780.9	123583.1	152631.3	173088.3	146673.6	163068.3	215515.5	164319.2	180052.6

LP408.296	LP408.296	LP408.296	LP408.296	LP408.296	LP408.296	LP408.295	LP408.295	LP408.296	LP408.296
183833.3	162271.9	156692.5	149035.1	181692.4	221419.7	151473.8	152774.7	112580.4	69395.72
202585.7	137242.6	141523.2	141365.1	162071.4	200878.9	111020.8	139601.9	96772.83	84769.65
198679.9	138387.7	171726	207666.6	153027.6	220446.5	157336.7	155816	118847.8	109250.2
169592.9	114860.6	125644.7	152927.8	174972.7	173090.3	143871.6	159772.8	115090.4	120510.3
224289.2	130083.6	130854.5	145559.8	296282.5	264044.1	127604.7	134976.7	86108.18	107564.1

LP408.295	LP408.296	LP408.295	LP408.295	LP408.295	LP408.295	LP408.296	LP408.296	LP408.295	LP408.295
159149.4	175016.3	56323.28	91786.97	102106.2	102767.3	182286.8	117158.3	151389.1	106641.3
140956.6	170699.7	108317.7	102982.2	98452.82	72204.57	66295.73	131343.2	157713.7	103881.6
187145.9	180534.8	60774.31	105138.4	103852.4	96830.9	180813.8	127044.3	147076.4	108371.9
158482	194678.9	93066.46	97403.71	101449.8	100267.9	182959.3	122840.7	139378.3	90574.78
166160	171471.2	70809.7	95287.93	76492.46	110108.5	169991.6	68461.67	158318.9	96060.24

LP408.296	LP408.309	LP408.309	LP408.332	LP408.368	LP408.368	LP409.258	LP409.292	LP409.310	LP409.312
106206	141985.9	198833.8	235276.4	57677.49	59156.17	48026.99	60986.2	92050.35	30009.85
104138.2	142695.7	183752	234072.2	54112.53	55627.27	39717.82	64243.14	96972.71	31890.23
101863.3	32220.93	187741.4	251541.4	46961.36	64303.85	36098.6	68377.28	110164.5	34465.94
95287.4	152308.7	197666	84913.03	52538.31	70467.36	41093.51	67170.16	98806.67	29669.02
94307.94	147308.6	186807	254341.1	49096.38	70475.09	36680.76	67285.11	83083.01	28114.74

LP409.311	LP409.329	LP409.33_	LP409.367	LP409.367	LP409.368	LP409.368_	LP409.368	LP409.368	LP409.383
27452.06	151099.2	59612.93	33406.33	46167.06	33475.35	70918.37	38079.86	41722.11	46903.96
28806.08	162693.9	54803.75	32560.5	49584.28	30323.31	72866.18	37037.92	34926.47	48316.02
29327.51	170445.1	56921.65	33278.17	45275.85	30258.67	76043.76	41008.53	32298.61	48309.14
26533.56	182623.9	61838.84	29638.87	42542.54	30155.27	68519.33	39238.6	33551.65	45752.32
28441.31	185974.9	61544.9	31070.16	45148.03	30522.99	74973.26	39540.85	34305.01	45483.95

LP409.382	LP409.383	LP409.404	LP409.404	LP409.404	LP409.404	LP410.152	LP410.238	LP410.239	LP410.239
36800.56	34042.26	37865.54	37424.79	42483.01	49874.92	33470.98	194043.2	126109.8	110981.8
33719.61	35788.59	35105.23	31184.56	39340.36	47854.87	33232.89	188429.9	117838.1	109176.8
38835.37	29593.66	37030.45	34526.84	44257.87	44810.32	45969.01	199840.3	113060.9	107021
31872.63	28843.73	42565.87	35884.84	39082.1	45901.95	38381.81	176704.3	166485	113971.6
36523.6	30674.59	39094.6	36677	37312.16	44472.29	28959.73	210034	108883.7	100709.3

LP410.239	LP410.238	LP410.239	LP410.239	LP410.239	LP410.239	LP410.239	LP410.239	LP410.238	LP410.238
80553.61	89418.85	124207.4	92095.58	94637.83	118638.4	110936.5	78257.71	84201.72	52767.95
85883.91	87910.26	123302	88851.47	88691.31	123519.8	113760.2	76689.53	76816.31	62199.06
88885.19	87863.77	114650.4	95069.81	87929.31	112492.7	118982.7	75067.76	72436.64	63704.36
98493.03	81622.76	129503.4	94773.15	103944.3	123672.8	125961.1	86041.17	88715.58	67313.46
87461.89	85454.93	121833.5	96916.48	90627.75	110628.3	112003.3	76240.1	76686.22	58259.67

LP410.238	LP410.258	LP410.275	LP410.275	LP410.274	LP410.275	LP410.275	LP410.275	LP410.274	LP410.275
89893.42	65742.1	104629.2	108589.3	129057.2	119754.2	125271.6	100872.1	90722.03	98152.05
88236.69	114219.2	110648.6	126138.2	150770.5	165097.7	122951.9	106926.4	107556.8	109832
92114.99	55805.51	105544.3	116643.8	109188.2	120699.6	111414.1	104704.8	86615.42	92257.79
74649.69	109966.9	106118.5	120833.4	115861.9	114874.9	114274.9	107172.3	88731.71	91730.34
81345.71	109122.7	103493.7	101299.1	144851.6	128805.7	161899.4	82528.2	127442.2	79926.43

LP410.275	LP410.275	LP410.275	LP410.275	LP410.274	LP410.275	LP410.274	LP410.275	LP410.275	LP410.275
125291.2	150139.8	78100.79	88494.72	84230.55	98034.45	59283.13	82163.93	105450.4	84265.53
117020	120300.4	69176.46	91502.81	148272.2	86989.15	70072.99	85339.68	123524.5	100533.4
121610.6	144125.3	75604.42	88687.3	82760.94	94505	56241.4	76843.79	98127.65	77372.4
125218.7	162647.8	76655.89	95192.15	86598.63	100461.6	53414.44	80506.96	98766.73	70041
136078.8	141904.1	70756.1	114050.7	93917.31	112020.5	71438.68	85632	91990.84	81490.1

LP410.275	LP410.275	LP410.275	LP410.275	LP410.275	LP410.275	LP410.275	LP410.275	LP410.275	LP410.275
170281.7	69131	91544.57	86374.42	84064.96	78667.25	77876.94	71896.38	53815.45	64867.9
168899.7	76009.23	99590.14	124520.1	125492.6	103243.1	71209.62	121195.7	58325.44	79268.04
160645.9	80196.46	80339.19	89521.98	86939.28	78145.03	79606	67498.26	62384.76	61331.03
158660.8	72646.89	82746.6	93634.86	80474.58	75971.44	77322.16	71220.77	65578.11	62427.63
148102	70251.38	91418.68	109677.9	66237.3	72176.83	74282.03	107474.4	56072.42	104238.4

LP410.275	LP410.275	LP410.275	LP410.275	LP410.275	LP410.275	LP410.275	LP410.275	LP410.275	LP410.275
92444.63	72038.12	65205.9	93301.75	89356.83	58131.8	89090.38	100900.1	78265.4	66948.98
113022.5	76168.9	90546.98	109199.1	113350.4	70022.96	83379.49	94647.84	108873.8	72894.88
94783.93	68628.46	72562.99	89288.09	95820.88	64940.07	81683.91	91860.86	81197.16	66802.68
95478.22	70714.66	74477.5	103304.6	90194.59	54634.97	87749.33	90583.13	73234.29	65948.94
122727.2	70404.18	80388.03	62034.22	88268.75	71479.68	84206.56	97129.87	71118.78	68945.42

LP410.275	LP410.275	LP410.275	LP410.275	LP410.275	LP410.275	LP410.274	LP410.275	LP410.274	LP410.275
76590.89	81231.52	83462.99	75011.05	66524.36	72677.93	82337.64	68191.15	101054.6	72902.86
71363.38	84161.17	100702.4	68211.47	79606.64	61078.58	113689.9	69413.23	74516.76	104349.9
69211.95	82022.69	92820.86	74069.67	63658.72	58807.89	74436.06	64489.29	103519.6	71823.31
72742.08	77807.59	89202.15	72567.31	61796.97	56393.31	82236.55	66231.21	99871.61	76144.38
73754.81	81764.16	86697.31	69791.18	86468.51	98867.34	78201.19	68511.65	80602.29	69597.56

LP410.275	LP410.275	LP410.275	LP410.275	LP410.275	LP410.275	LP410.275	LP410.275	LP410.275	LP410.275
69102.55	71255.64	64222.65	92791.59	76799.76	88827.8	75150.08	98250.76	88702.06	79976.68
76083.05	65019.86	70541.92	92421.84	73015.87	92187.27	112307	93727.32	88028.53	82655.53
62213.25	71004.18	64067.24	88160.97	71256.4	82546.5	73939.91	95918.54	80974.8	68290.51
57704.27	69464.24	60783.38	102809.1	78274.09	91490.74	76712.87	91320.15	82935.56	67362.51
46366.45	82232.9	49760.61	63277.5	63814.04	65963.3	70525.18	95547.82	68113.43	94031.72

LP410.275	LP410.275	LP410.275	LP410.275	LP410.274	LP410.274	LP410.275	LP410.275	LP410.275	LP410.275
91033.28	85416.7	82654.67	64384.49	64969.2	59253.61	54299.06	52012.44	54150.06	51260.16
96673.68	65479.47	79142.12	85910.38	55744.42	70391.43	69753.41	45803.25	59872.97	60087.08
84323.49	69022.82	85405.07	65284.31	65955.21	54396.36	55518.2	58581.83	53503.88	50384.17
94636.27	74303.52	75444.06	67225.74	66540.92	68933.22	46674.54	56669.44	47933.21	50169.65
125575.8	75306.5	75924.68	71213.59	59290.43	61451.58	52877.49	48676.7	72665.37	51097.06

LP410.275	LP410.275	LP410.275	LP410.275	LP410.275	LP410.275	LP410.275	LP410.275	LP410.275	LP410.275
62431.41	73544.27	77272.49	81151.38	62677.29	73263.79	63553.29	70013.22	52965.59	67670.25
54076.47	76926.76	78606.27	69344.48	64968.82	74864.91	94171.18	65510.34	54199.87	69542.62
59327.88	75278.6	61366.25	82651.76	70089.86	76535.08	71484.28	67662.4	58788.91	67730.36
65119.86	65958.02	60520.28	75880.29	69949.63	85089.35	72878.22	65004.23	48698.21	68533.83
54226.49	72653.7	57249.71	76956.46	67423.04	82046.65	70580.37	68818.78	49322.19	64817.19

LP410.296	LP410.332	LP410.386	LP411.199	LP411.199	LP411.199	LP411.199	LP411.199	LP411.199	LP411.199
78901.05	125255	7297.822	67619.86	44974.65	69100.08	45619.72	44800.83	68126.76	44601.34
68038.99	121309	10902.97	66445.79	44217.31	70131.53	45728.17	45070.27	67054.29	41355.04
74608.05	122180	9365.152	77628.63	45560.1	68694.28	47070.64	42617.33	69072.36	40977.77
73277.11	117042.1	5657.114	67286.21	46154.95	67543.38	54587.58	48141.17	67198.73	42876.52
73842.41	110718.2	8305.572	67440.81	48784.99	67169.91	49905.38	40955.14	71129.45	40429.67

LP411.224[illegible]	LP411.244[illegible]	LP411.275[illegible]	LP411.275[illegible]	LP411.274[illegible]	LP411.275[illegible]	LP411.394[illegible]	LP411.431[illegible]	LP411.431	LP411.431[illegible]
61000.08	118185.4	26763.13	26188.04	35928.29	38198.64	9786.463	395357.7	38804.73	45771.88
63190	146072.4	36386.76	28188.86	36367.92	41204.18	11021.48	370986.7	37946.78	46504.59
67299.13	116949.6	30400.34	29756.11	38274.22	42478.5	14688.71	326462.9	36728.39	40254.71
66313.13	108200.2	35593.49	26359.4	32829.32	37559.49	14087.36	299427.3	33052.79	38996.94
70050.78	115114	26815.14	32538.74	37147.67	39164.23	16891.02	320066.5	25662.41	34527.62

LP412.150	LP412.218	LP412.218	LP412.218	LP412.228	LP412.252	LP412.269	LP412.305	LP412.305	LP412.378
19016.98	23434.56	28858.89	20669.95	10178.04	170918.7	23648.04	37442.87	50919.89	23693.36
20172.05	30336.74	25722.32	24892.53	10897.31	113102.2	27349.36	24004.74	52308.91	25950.3
14498.49	30827.64	23673.27	21300.6	11373.66	142349.1	26166.52	38563.2	45810.03	22122.01
17318.69	27604.11	28319.7	23922.21	11100.11	159831.9	19721.3	35179.13	39857.87	25838.69
12309.88	25173.23	31293.65	18815.29	14049.44	138383.3	25419.59	30705.87	42855.14	23285.6

LP412.426	LP413.177	LP413.178	LP413.178	LP413.179	LP413.179	LP413.178	LP413.178	LP413.179	LP413.179
21173.15	46418.49	85027.69	67526.96	109501	87912.96	99208.98	65765.69	76030.78	76051.76
30377.97	37376.09	75147.26	65058	79955.68	90383.13	75778.87	76782.77	71368.52	74954.92
29908.76	49331.32	89439.75	68663.75	92983.18	83970.61	70344.01	72731.52	70549.5	73910.07
25271.7	35248.31	87463.88	64350.18	98911.61	92381.56	74165.14	70640.33	67301.45	79136.16
24957.56	46224.61	81742.99	61147.86	90494.32	82736.73	72773.49	74799.32	78294.7	75639.34

LP413.178	LP413.179	LP413.178	LP413.179	LP413.178	LP413.179	LP413.179	LP413.178	LP413.179	LP413.178
61656.07	79269.53	53507.71	111737.7	98054.09	65455.35	73897.16	33047.46	87425.09	76126.04
98964.95	90614.45	46229.61	77061.62	65260.13	45078.26	89905.16	32826.07	90750.99	78918.88
93142.32	92492.38	53813.5	82992.89	66871.34	52302.81	74848.46	33901.49	86427.74	66236
91940.54	85459.27	54247.77	81245.55	64596.28	46857.46	74570.32	30514.69	83648.11	75340.03
99584.99	98029.35	54589.2	77269.92	66645.89	53287.67	74360.05	30369.84	87904.89	73980.26

LP413.179	LP413.179	LP413.179	LP413.179	LP413.179	LP413.179	LP413.178	LP413.179	LP413.179	LP413.178
107967.3	58768.55	106418.1	61825.28	90377.33	57299.72	34481.15	77106.72	64202.65	37438.91
88337.78	92575.52	79745.33	71368.23	85542.25	48173.9	25829.96	68404.67	67790.57	30459.98
78799.96	87786.9	84272.7	82798.41	81240.82	49193.85	27243.4	70188.36	68138.4	25383.79
86195.15	88720.02	82434.77	80153.9	78249.9	48463.81	29134.88	70598.79	71475.96	23246.65
87423.58	78414.43	87790.28	76426.99	83307.25	49506.75	29187.61	71760.42	70649.94	26362.86

LP413.178	LP413.179	LP413.179	LP413.178	LP413.178	LP413.178	LP413.178	LP413.179	LP413.178	LP413.179
71270.27	136361.9	90447.75	44160.99	38880.39	84997.72	47901.66	68516.28	53866.67	137267.5
70802.44	78093.71	72736.53	51429.5	47031.23	72646.54	55276.59	59005.75	60699.3	117830.2
61743.51	82129.92	71875.19	50604.87	41729.65	72606.37	59207.17	60840.97	53023.2	116184.1
64870.36	92326.96	73901.29	47693.28	41234.14	74742.84	60833.27	56875.4	57403.16	115483.3
62069.63	95146.21	71490.81	53712.75	44754.88	63158.06	61647.16	65486.55	66007.95	130043.7

LP413.179[illegible]	LP413.178[illegible]	LP413.179[illegible]	LP413.179	LP413.179	LP413.179[illegible]	LP413.179[illegible]	LP413.178[illegible]	LP413.178[illegible]	LP413.179[illegible]
65212.01	96380.6	77258.87	49615.66	71742.84	83278.92	59096.61	90674.43	26989.21	100217.7
81955.37	75187.95	60405.67	57830.53	66842.3	80819.9	69603.47	71395.39	28865.53	75458.57
80247.1	80696.87	72650.68	55725.57	67044.28	91250.36	67667.01	68057.69	29422.2	78720.38
84405.35	87580.62	64947.44	63191.65	67296.26	89401.16	75546.66	66777.29	28571.58	80723.29
86685.03	82963.86	66629.44	64976.19	75418.57	93323.01	69766.81	70432.29	32662.37	70610.52

LP413.179	LP413.178	LP413.179	LP413.178	LP413.178	LP413.179	LP413.179	LP413.179	LP413.178	LP413.178
58091.21	27185.29	89846.92	31396.85	18455.7	81626.66	64739.99	91593.4	36391.03	29050.66
65779.33	28508	59090.61	22327.51	24174.29	73403.17	56373.4	74785.9	48996.85	23181.48
63773.16	26833.05	64749.05	25194.2	25669.71	77263.69	50978.96	78239.7	47098.98	22104.97
75103.07	26036.1	59824.77	21586.96	18393.57	74211.27	55686.99	81184.81	49700.6	23638.59
70184.49	29426.31	59298.57	23296.62	22606.05	76742.93	59933.49	81532.8	49059.63	26531.38

LP413.179	LP413.179	LP413.178	LP413.179	LP413.178	LP413.179	LP413.179	LP413.179	LP413.178	LP413.179
53007.2	91998.26	24888.94	43355.05	62457	57488.81	76698.08	65036.37	37416.2	44715.65
56973.18	75983.3	25637.91	60386.26	85919.04	49223.02	62905.91	68996.04	37174.16	66792.68
56135.1	85045.79	23219.6	58642.89	81843.03	48751.07	64032.02	68535.49	32859.85	57319.12
60067.99	84011.98	27037.07	56615.29	80275.27	46406.3	65475.98	63142.66	38357	59378.6
65332.24	90611.8	23711.24	59303.55	72469.51	48450.61	57669.7	69498.28	36207.4	57591.31

LP413.178	LP413.179	LP413.179	LP413.178	LP413.179	LP413.179	LP413.179	LP413.179	LP413.179	LP413.178
21195.24	55865.03	128284.2	49246.75	67231.76	44388.6	91489.68	79446.36	64307.13	33895.36
27126.68	64379.25	95152.37	63222.56	53949.4	48136.71	63030.47	94244.37	70506.69	31757.69
25670.02	63254.86	100814	58845.7	49131.33	50715.88	62818.4	103267.7	63965.05	27501.51
24338.98	65235.57	98538.44	54660.94	45694.94	54471.96	68220.46	99840.45	62184.47	31202.2
25385.46	68536.89	100864.3	60410	55820.74	52715.64	61901.31	92617.39	68731.34	35557.16

LP413.178	LP413.179	LP413.178	LP413.179	LP413.178	LP413.178	LP413.215	LP413.215	LP413.215	LP413.215
30489.9	105487.7	56172.53	72496.25	32508.09	58735.08	70545.38	110875.2	59316.28	80860.34
30200.47	118801.8	85843.02	64809.75	29613.94	61971.11	75608.13	78828.47	63120.83	88058.79
28670.25	110560.3	88728.45	78302.71	28795.11	70201.25	81645.26	108792.5	64293.4	85667.93
28674.65	118203.4	87112.56	73996.37	27278.01	58011.62	71496.26	107430.5	59456.31	83572.7
30876.87	114262.5	90136.57	64549.1	29059.29	65491.63	78121.96	118602.6	62248.57	85745.46

LP413.215[illegible]	LP413.215[illegible]	LP413.215[illegible]	LP413.215[illegible]	LP413.214[illegible]	LP413.215[illegible]	LP413.215[illegible]	LP413.215	LP413.215	LP413.215[illegible]
62404.19	74839.2	81118.13	76759.62	71433.71	81627.53	70358.1	74444.58	75140.2	67668.36
66633.26	78707.14	75119.16	79120.39	71568.9	66131.87	64487.52	75126.77	67573.29	67956.27
65773.49	74538.21	97574.42	78740.45	69585.45	71862	62375.64	77785.38	62029.9	70549.76
61919.78	77129.43	91184.01	78934.15	71708.63	71117.84	72161.29	78500.5	60900.16	63716.4
58883.41	81557.7	87202.85	79346.85	70350.57	68199.51	68126.86	81851.83	64228.02	65134.17

LP413.215	LP413.215	LP413.215	LP413.214	LP413.215	LP413.215	LP413.215	LP413.215	LP413.215	LP413.215
96822.41	83064.39	82942.07	129771.4	85997.69	66781.24	78848.05	94074.68	71927.7	64236.52
95202.96	83113.07	91138.32	118468.5	74574.06	59799.74	75446.22	94284.61	60684.71	67081.11
97954.21	87438.65	85567.8	115196.3	79345.82	62582.8	82635.01	75179.52	72730.48	64961.61
92207.03	91001.79	87509.71	125087	75243.05	65140.39	78990.28	91286.43	71419.86	64572.41
91999.52	81277.53	89261.19	107161.2	73570.92	60719.75	75130.02	88795.56	76700.26	64611.85

LP413.215	LP413.215	LP413.215	LP413.215	LP413.215	LP413.215	LP413.215	LP413.266	LP413.266	LP413.266
82027.59	85993.66	100313.5	72275.98	79667.09	88138.77	62791.89	3116228	2689047	2466986
84621.22	87067.08	93133.11	75181.72	81626.47	91327	58598.34	5257592	2112595	1597534
79137.09	78409.21	100579.1	74710.52	77622.65	89277.86	61542.38	2820984	2236411	1948410
80475.98	81804.86	100479.7	75269.71	72083.96	84793.96	61937.29	3014502	2707236	2158264
77658.93	85261.99	92904.95	68961.43	66691.73	84218.87	62795.1	2857885	2866305	1640275

LP413.266	LP413.266	LP413.266	LP413.266	LP413.266	LP413.266	LP413.266	LP413.266	LP413.266	LP413.266
2552749	2221931	3644581	3326988	5035510	3587894	3552497	2831917	2848265	2335107
2165148	2484223	2842863	3055688	4454204	3327090	3064285	3986518	2808455	4185208
3274831	2225131	2514774	2912201	4106330	2569787	3375877	2582680	4042383	2739852
2243328	2727978	3297621	3256044	7774780	3784276	2909591	3366592	4400974	2317029
2459694	3255647	4112962	2360554	4859723	4342925	3112815	1986519	3266250	2309645

LP413.266[illegible]	LP413.266[illegible]	LP413.266[illegible]	LP413.266[illegible]	LP413.266[illegible]	LP413.266[illegible]	LP413.266[illegible]	LP413.266[illegible]	LP413.266[illegible]	LP413.266[illegible]
2890593	2431298	6117070	3081709	2438504	2252859	3296481	2144215	2070286	1743952
2799048	1833019	5476790	1962294	2998225	2579953	4368960	2539487	3338886	2392583
2567199	1998874	4433941	2663876	3061616	2142806	3435834	1851976	2298038	1990055
2647394	3349914	3495879	3054182	2645444	2780894	2603999	2171827	3667698	2710729
3586460	1578141	6851062	2008944	3424089	2849023	3931594	2236413	2261294	2741966

LP413.266	LP413.266	LP413.266	LP413.266	LP413.266	LP413.266	LP413.266	LP413.266	LP413.266	LP413.266
7440986	3546316	2811040	2023756	2395693	2312133	2380901	4411514	2815873	2012863
7968978	3601231	3413198	2360211	1895948	1703528	2823338	3153021	4202334	3381689
9287362	2781153	3037745	1699107	2739024	2029899	3078144	11420409	2741160	2719180
5515615	3531454	4341635	2085413	2422443	2352983	2049468	5029988	3212301	2576579
11773158	3026965	3231041	2414289	2677350	1724775	3058633	3409687	4025034	3962179

LP413.266	LP413.266	LP413.266	LP413.266	LP413.266	LP413.266	LP413.266	LP413.266	LP413.266	LP413.266
2619462	4745961	2822942	2506884	2385906	2461245	2756025	1553773	4135585	3596392
3207516	2625656	2237408	2097829	2690512	2415499	3454759	2501294	2856365	3341133
2507931	2852032	2656220	2302216	3780207	1775061	3146779	2507095	3516399	3256388
3206436	2560716	2701664	1903946	2488383	2026029	2933123	1813923	2083432	2944747
3787949	2503929	2329282	2392629	2589437	2450815	2753876	3064144	2730230	3676115

LP413.266[illegible]	LP413.266[illegible]	LP413.266[illegible]	LP413.266[illegible]	LP413.266[illegible]	LP413.266[illegible]	LP413.266[illegible]	LP413.266[illegible]	LP413.266[illegible]	LP413.266[illegible]
1853504	3954913	3930177	2537086	2247147	2812784	3505383	1859269	3248783	2977235
2742726	2708306	3502725	2426570	2944152	2789707	4787130	1832912	3449058	3507153
1985366	4112241	2715997	2305229	1960838	1983178	2487248	2324857	3913362	3881118
2632040	2942288	2981262	2529413	3243122	1794093	3907558	1617113	3692225	2799258
2729114	2879309	2875128	2201859	2038733	2229383	3685571	2410932	2761310	3315642

LP413.266[illegible]	LP413.266[illegible]	LP413.266[illegible]	LP413.266[illegible]	LP413.266[illegible]	LP413.266[illegible]	LP413.266[illegible]	LP413.266[illegible]	LP413.266[illegible]	LP413.266[illegible]
3240391	1873254	1604544	2793351	1973972	3344291	3640452	1729347	2095356	1875260
3399444	2066943	1774299	2304819	2279189	3055048	3974021	1773537	1997603	2084544
2762086	2310345	3318103	1907654	1874030	3474382	2741595	1636922	2015680	1548064
3635512	1912902	1799025	2577495	1946787	3183711	3113828	1528345	1533822	2084932
2890685	2226639	2205690	2711351	1893598	2675598	2766560	1662421	1901558	2811923

LP413.266	LP413.266	LP413.266	LP413.266	LP413.266	LP413.266	LP413.266	LP413.266	LP413.266	LP413.266
1992948	1850942	3640004	2242365	351410.3	4861583	246046	2165437	230561.2	315546.2
2153791	1874454	2123933	1821866	308201.1	5461211	280759	1997904	225941.5	302143.5
1359179	1299773	2564764	1788425	341134.4	5147328	216908.8	2271509	217330.3	326286.8
1922942	2330774	2300511	1686649	319573.3	4309669	227763.9	2256530	254598.1	311804.3
2622864	2013512	2481646	1749624	314553.3	4400413	239453	2456046	269163.9	330908.3

LP413.266	LP413.266	LP413.266	LP413.266	LP413.266	LP413.266	LP413.266	LP413.381	LP413.381	LP413.418
348164.6	195702.5	223222.2	319465.3	182050.1	208105.6	207246.8	32065.81	24552.39	26811.76
310459.1	192562	249778.5	223444.2	171930.8	185206.2	200850.6	31273.22	21969.37	26539.68
433589.8	208516.7	243950.1	182005.5	175294.1	174968.9	179979.7	32468.56	25000.34	25460.02
315415.2	207112.7	249030.9	216854.9	171828.9	185925.8	198429.7	28747.82	23648.78	24528.96
335486.8	231330.6	295278.3	237273.6	211436.5	252324.1	220077.8	26716.18	24064.26	26415.7

LP413.446	LP413.446	LP413.446	LP413.446	LP413.446	LP413.446	LP413.446	LP414.233	LP414.233	LP414.233
12666.91	21426.58	53258.46	35123.74	23377.61	19881.99	11293.18	92681.38	183919.5	138169.9
18552.59	18563.62	55113.47	32591.81	24029.88	17602.62	13884.06	90647.91	186773.3	142791.8
16241.83	19964.22	71036.37	34469.77	29442.23	17564.64	13841.32	99481.22	172280.8	150877.3
19040	17672.81	80027.16	33808.19	32510.25	12807.52	12905.98	90495.96	184366.8	143178.6
17518.99	21079.65	79633.11	32587.9	41722.08	19939.92	10044.33	96823.57	191183.7	145724.1

LP414.27_	LP414.269	LP414.269	LP414.269	LP414.269	LP414.269	LP414.269	LP414.27_	LP414.269	LP414.27_
867044.9	523474.8	525418.2	628404	804649.9	625024.1	691702.3	822302	441760.3	932147.8
674629	775877.4	519183.1	764162.7	824545.4	737592.2	840503.2	620678	487108.6	599957.8
945831.5	785730.5	558878.7	588290.2	782168	875840	966632.8	830797.4	556143	1099157
814535.9	565129.4	626211.7	592156.5	722460.6	639721.8	903083.9	638517	793920.4	528009.9
567372.7	599090.8	766107.4	821149.1	692962.4	534238.5	708490	722656.2	641942.9	695267

LP414.27_	LP414.269	LP414.269	LP414.27_	LP414.27_	LP414.269	LP414.269	LP414.269	LP414.27_	LP414.269
628704.4	3010562	1149648	617836.7	815717.1	1967925	680270.5	1411253	1133736	830360.2
497184.3	1163321	726943.5	718044.2	861839.3	1187538	712253.4	1621132	997749	605263.3
630554.4	1168359	592163.6	471713.5	949532.7	1137284	518076.1	1223995	684640.9	790354.7
888203	1687374	592468	563811.9	806236.6	1245482	483418.5	1290675	786430.9	832250.3
651222	1243163	775903.9	536056.3	828004.7	2069066	608409.7	850315.7	1313713	824304.1

LP414.27_	LP414.269	LP414.27_	LP414.27_	LP414.269	LP414.269	LP414.27_	LP414.27_	LP414.270	LP414.27_
860044.7	543186.9	668972	565520.2	805149.3	2465390	825081	980242.7	1030159	887575.6
1441056	506243.7	729230.9	497697.2	554108.7	1941829	633705.8	974911	981039.1	1069001
715056.3	597293.2	874341.4	627887.4	843000.1	1390440	1196226	970286.2	921084.2	697482.7
839108.1	517440.2	794488	659428.1	576110.3	1349546	780107.6	802729.5	827925.6	1080854
1532439	527461.5	759522.3	705266.2	644775.9	1525023	663186.9	693970.4	1151078	1000295

LP414.27_	LP414.27_	LP414.269	LP414.269	LP414.27_	LP414.27_	LP414.27_	LP414.27_	LP414.27_	LP414.270
952020.2	806127.5	832790.4	691325	815958.2	750271.8	795598.4	870513.3	685500.7	946887.6
705555.1	704605.3	619590.7	736168	1137746	609648.2	773625.7	985195.7	616751.8	758611.3
785912.8	692669.1	703322.5	1023818	992948.7	838061.3	870138.4	919069.7	510574.5	887437.5
1038815	1158588	621976.1	601162.3	900394.8	1023815	888041.1	730896.3	657944.1	744581.3
863516.8	795994	798656.6	835354.1	1001877	1090120	1060511	1087619	641049	1059430

LP414.27_	LP414.27_	LP414.269	LP414.27_	LP414.27_	LP414.27_	LP414.27_	LP414.27_	LP414.270	LP414.27_
578804.3	586775.8	697747.7	573590	601864.1	641377.8	457651	628248.3	780004.1	588877.4
485204.3	551757.9	849726.4	624278.6	477395.7	640640.4	591771.6	613559.3	629758.7	363350.4
676644.6	741956.3	992087.8	402248.3	443465.1	605982	817987.5	900628.4	486197.4	520320.4
557192.9	555155.7	835005.6	594372.9	372793.2	559144.7	507758.1	625015.9	531044.8	502033.9
732582.2	611091.7	652731.2	794298.6	505645.2	638076.5	761144.4	644005	533775.5	418338.6

LP414.27_	LP414.27_	LP414.27_	LP414.269	LP414.269	LP414.27_	LP414.27_	LP414.27_	LP414.269	LP414.27_
1004491	541259.2	655626.5	570995.6	1262968	662008.1	663645.4	557173.3	584234.1	669441.8
663663.9	717652.6	551769.2	674776.5	1438991	641313.4	1236328	511810	519753.1	782210.8
807590.8	590436.9	517569.3	502373.9	1344273	718699.2	725938	647473.2	422376	753398.2
593334.9	577657.4	489983.7	568063.9	1385935	479175.8	785941.9	580567.1	506274.6	593644.6
958706.2	498798.7	428408.9	725582.5	1328587	515757.6	623479.2	543617	542453.3	856500.5

LP414.27_(	LP414.269!	LP414.269:	LP414.269!	LP414.269!	LP414.269!	LP414.27_:	LP414.270	LP414.270:	LP414.270(
567099	590348.5	70678.89	80827.99	96207.71	107953.5	94549.51	58359.72	59373.98	130217.3
536745.2	501343.3	71806.69	75922.27	98234.95	84985.73	89280.74	56734.21	49956.96	128303
582089.3	487453.3	66542.32	76075.04	96747.7	101967.1	88572.14	54094.58	56034.67	136206.2
458270	467400.6	74076.34	97031.15	97738.74	113618.4	92278.9	48033.07	54171.14	136911.2
776649.8	467014.7	64106.65	79866.21	92672.38	108958.6	98359.95	57137.19	59873.28	143030

LP414.269	LP414.27_	LP414.270	LP414.27_	LP414.270	LP414.270	LP414.270	LP414.358	LP414.357	LP414.369
74219.5	76723.39	62183.56	142688.5	63651.86	62961.36	54274.69	119850.3	58767.36	28905.27
63700.95	73484.3	73699.65	138400.9	53596.77	61425.82	55372.23	130911.2	78345.79	30804.54
58923.47	79004.13	61051.12	131943.3	61143.34	54830.44	52535.92	116528.7	70639.42	28044.97
68095.19	80207.95	61270.18	134948.4	56662.92	62265.99	63968.02	116548.2	68240.77	26604.69
76645.31	86320.68	61051.31	163400.9	66507.85	65379.42	59013.65	154129.5	91740.86	27135.87

LP415.194	LP415.193	LP415.194	LP415.194	LP415.194	LP415.194	LP415.194	LP415.194	LP415.194	LP415.194
107279.7	201338	151053.1	113154.9	126445.8	145257.6	131129.7	110699.6	121787.8	158759
77802.42	152705.5	102319.8	175158.8	142291.5	187288.4	233191.7	95986.69	152604.4	243563.8
149774.3	197872.6	128041	111209.8	166574.3	179173	113120.8	160240.1	79634.92	168211.6
78539.63	151562.7	191020.9	90055.83	131073.2	157219.8	95269.74	102915.9	98880.08	205669.7
119984.9	152116	135880.5	155275	145803.1	137037.7	147060.2	137271.6	125852.2	133917

LP415.194	LP415.194	LP415.194	LP415.194	LP415.194	LP415.194	LP415.194	LP415.194	LP415.194	LP415.194
145770	50364.57	189587.8	222541.7	57721.38	111033.9	36828.55	73245.82	132597.2	109375.6
118257.7	75815.23	238228.6	110607.9	97875.6	105188.7	91514.79	101257.8	197330.1	171044.3
140791	44028.07	190738.8	202110.5	105330.4	114918.6	97232.52	103883.5	110049.8	73543.23
93936.9	22666.61	111037	113114.8	48399.67	78459.11	33037.81	57767.74	112857.3	93222.56
126804.1	41125.09	134339.1	105836.4	42757.99	124818.6	35360.39	41675.77	110255	113924.4

LP415.194	LP415.194	LP415.194	LP415.194	LP415.194	LP415.194	LP415.194	LP415.194	LP415.194	LP415.1947
51476.92	114244.8	33116.05	58652.2	230959.3	128882.4	117273.9	122332.3	46665.72	56647.5
85033.04	215942	68585.7	97021.4	158566.3	156771.7	141437.7	182421.5	85813.14	104739.3
76363.46	212397.9	77792.37	37166.98	204984.5	164305.6	143487	151710.4	40196.13	113156.1
29024.61	79569.96	28769.28	37102.24	199985.8	172685.4	145462.6	104926.5	33467.17	45468.18
73147.74	92316.52	24930.16	47880.62	192810.7	134770.6	160441.3	132869.7	40437.98	56491.88

LP415.194[illegible]	LP415.194[illegible]	LP415.194[illegible]	LP415.194	LP415.194[illegible]	LP415.194[illegible]	LP415.194[illegible]	LP415.194[illegible]	LP415.194[illegible]	LP415.194[illegible]
249962	201041.1	319069	272298.4	100414.9	166701.6	42077.27	59208.15	314789.7	96833.83
327785	209666.8	179283.2	248509.1	174603.2	121031.5	82040.8	103738.1	325069.3	92280.07
178257.4	208310.3	342472.4	149598.3	89064.5	157421.3	33375.95	105672.6	365094.6	36295.43
308645.7	228193.9	321221.5	157042.5	92737.07	124769.6	76432.18	51436.04	176104.7	40009.69
194831.3	232927.4	149690.6	251184	95321.05	108166.9	34419.48	102221.2	205320.3	42108.66

LP415.194	LP415.194	LP415.194	LP415.193	LP415.194	LP415.194	LP415.194	LP415.194	LP415.194	LP415.194
150828.2	257197.2	212935.3	185012.2	50817.77	287770.1	332872.5	320760.1	158391	145830.2
266060.5	199344.8	337766.6	218080.9	86451.59	261324.7	189337.9	329180.2	123008.7	269843.6
268705.3	189046.5	258844.8	294604	38453.75	162771.8	272749.8	333601.1	149668.1	261678.3
265770.6	290187.1	317745.2	229934.2	41826.93	267928.1	145030.6	191032.6	148533.7	228318.7
197171.4	199545.1	312441.4	144248.2	51362.61	137505.3	350161.4	189035.2	87209.51	242657.1

LP415.194	LP415.194	LP415.194	LP415.194	LP415.194	LP415.194	LP415.194	LP415.194	LP415.194	LP415.194
337387.4	207022.3	328310.4	183475.7	329503.9	217873.3	161003.6	380845.2	153559.3	271983.9
154302	351935	246127.3	131663.1	294026.8	129178	134451.1	211430.5	344789.2	207729.3
280666.8	168273.6	361957.3	203392.3	144586.9	339987.9	283294.9	200968	356946.9	279749.1
205445.3	191467.7	393512.7	116604.2	305098.3	336921.9	290194.6	207717.4	129926.7	253677.7
312068.1	363448.3	215478.3	127290.6	173213.2	325576.7	150333.3	198558.4	345893.8	146098.5

LP415.194	LP415.194	LP415.194	LP415.194	LP415.194	LP415.194	LP415.194	LP415.193	LP415.194	LP415.194
203359.7	234336	287981.1	254666.9	146951.9	205965.8	156990.2	288314.5	361595.9	242147.3
126173.3	229394.5	165076.1	221363.1	162374.6	376139.1	272623.6	126520.9	369251.8	481460.2
201933.4	136627.3	279310.9	507609.6	169961	369842.7	268173.9	290659.6	377240.3	432659.1
117758.1	138946.3	188644.2	448467.8	113443	389152.3	276433.2	291916.7	347250.5	458904.9
135988.9	234938.4	279164.6	451130	126610.9	197094.9	180094.4	287431.7	132800.4	211290.8

LP415.194	LP415.194	LP415.194	LP415.194	LP415.211	LP415.211	LP415.211	LP415.211	LP415.211	LP415.211
627619.4	489467.4	437977.1	219442.3	257547.9	244130.8	586950.2	359917	534837	273844.1
258349.4	191964	427712.6	218755.1	200861.6	321649.7	454912.2	361353.6	404929.1	308428.6
271275.1	460624.4	427247.6	224381.1	226398.7	264525.7	531899.5	259796.6	522778.4	269915.1
682407.1	262866.8	431618.1	208338.6	356702.5	295236.3	345140.9	501041.4	541579.5	377983.1
315810	287393.2	207747.1	215713.5	269219.9	397042.1	503363.1	356347.2	374986.2	241338.8

LP415.211	LP415.211	LP415.211	LP415.211	LP415.211	LP415.211	LP415.211	LP415.211	LP415.211	LP415.211
362984.1	516723.1	432514.6	290287.7	381355.5	708709.9	277483.4	341217.3	362613.3	430172.8
398456.9	505426	543540.1	260278.4	409519.7	542937.6	279556.1	373964.7	263877.2	355382
298525.8	507410.8	388451.8	272322.7	398064.2	625400	429197.8	340345.8	381323.5	276584.5
340047.6	691801.1	472331.5	492187.2	480897.7	777661.2	434858.3	298649.9	231036	389417.5
336563.8	587488.4	615471.5	267427	423685.8	470389.2	262454.1	199819	260290.6	499458.3

LP415.211[illegible]	LP415.211[illegible]	LP415.211[illegible]	LP415.211[illegible]	LP415.211[illegible]	LP415.211[illegible]	LP415.211[illegible]	LP415.211[illegible]	LP415.211[illegible]	LP415.211[illegible]
401892.3	392806.5	465553.4	281201.1	195763.8	497018.8	239347.6	378943.2	256110.6	187154.5
457406.6	356933.1	627504.5	257576.9	199562.2	506660.1	250000.8	447103.8	248519.2	186765.5
383552.5	394702.7	388743	315189.4	197859.7	490679.4	249341.1	335638.5	191401.4	179850.9
524843.2	613113.6	540907.6	258231.8	183150.6	625664.4	253579.8	335461.7	246841.4	190498.4
384517.8	322903.7	415973.9	273774.9	189068.8	466807.5	254362.4	358630.4	245777.1	172810.1

LP415.211	LP415.211	LP415.211	LP415.211	LP415.212	LP415.211	LP415.211	LP415.212	LP415.211	LP415.211
278991.5	372715.4	397114.3	256832.5	358706.6	400074.9	287286.5	480837.2	332578.6	399160.4
240984.6	372705	405158.5	485809.1	367045.4	320027.2	272483.4	494308.3	357118.4	358897
260639.1	242849.6	414492.7	414419.1	378521.5	382137.5	285798	452975.5	363006.4	402200.4
317961.9	268330.7	410262.2	407250.8	396273.8	372360.2	273860.5	560961.9	338043.1	357042.2
256110.5	338728.3	415446.8	406797.5	378107.8	402904.8	326327.8	432382.4	345896.4	351272.8

LP415.211	LP415.212	LP415.211	LP415.211	LP415.211	LP415.212	LP415.211	LP415.211	LP415.211	LP415.211
350075.6	378408.4	250835.4	341158.6	281077.8	184802.8	326773.4	172322.1	225771.5	274685
333172.1	379884.6	263283.8	203903.1	277549.8	192324.4	304188	187379.1	217168.6	244719.9
340245.1	372293.9	265367.3	215313.9	283673.5	181177.2	312057.5	170000.7	208947.7	240371.3
320314.9	423476.9	258572.3	217626.1	260080.1	196272.9	299482	167319.7	188459.6	235777.4
376316.3	361786.6	254353.5	210926.9	261999.3	195026	296956.7	175700.7	181928.6	213628.8

LP415.211	LP415.211	LP415.211	LP415.211	LP415.211	LP415.211	LP415.211	LP415.211	LP415.211	LP415.211
190522	238828.8	146179.9	283684.8	231493.4	269758.1	209812.4	200143.1	190944	133830.4
195028.8	244716.8	151677.5	293091.5	253811	259770.3	278224.9	208956.6	205680.3	137438.7
198539.8	244065.7	166217.1	273291.1	248948.3	264728.1	212514.2	206996	191136.4	134948.8
185093.1	241129.3	153211.8	297430.4	248064.4	258069.1	197736.8	206004.6	233439.6	121633.8
189771.7	251757.4	133502.2	290316.2	300403.3	266206.2	200483.8	205041.6	193510.9	136514.3

LP415.211	LP415.232	LP415.236	LP415.232	LP415.272	LP415.272	LP415.272	LP415.272	LP415.272	LP415.272
129658.8	304362.4	278283.7	532325.7	63519.49	88694.85	66594.76	118045.9	92765.94	137665.5
153526.4	293395	289003.6	537873.8	64167.93	86543.42	54864.81	131407.9	75390.12	123939.9
150110.5	279428.1	290081.6	540080.6	57651.59	92265.41	66203.11	131569.6	80144.95	115500.8
126117.9	309830.3	289940.3	490118.3	58036.66	83689.99	61763.61	131164.3	78840.39	120003.4
145349.6	265211.1	303704	490372.9	67235.49	76429.73	63162.55	133246	83205.89	126793.9

LP415.272	LP415.272	LP415.272	LP415.272	LP415.272	LP415.273	LP415.273	LP415.341	LP415.341	LP415.361
78930.18	113634.6	263296.6	235629.3	178557.8	182598.1	192307.1	264967.1	64122.13	21703.22
69576.98	99022.33	243235.2	223577.6	195284.3	167240.8	195126.9	261388	76134.56	21174.08
67989.94	106150.3	237449.2	217803.2	184855.5	170884.8	183831.8	242342.4	71071.9	19503.31
72854.53	110747.9	198754.3	215492.1	192818.5	167290.6	204667.9	213584.6	63561.81	18910.66
74142.01	92716.02	258721.1	225657.5	182657.8	176073.8	175962.9	216676	63600.5	23004.2

LP415.389	LP415.444	LP416.198	LP416.214	LP416.215	LP416.215	LP416.214	LP416.214	LP416.214	LP416.214
42330.07	64384.71	51027.06	95832.05	139083	140646.2	70089.19	104868.9	90336.52	79945.58
42759.55	57753.01	134097.7	110589.7	177346.2	135012.1	77219.27	84988.41	95606.07	74524.81
24155.63	81387.15	131108.9	101954.5	133235.8	122658.3	77326.91	83222.59	125163	75651.59
15842.6	75169.11	127130.6	103560.5	109037.3	163083.2	57471.03	87900.45	99444.35	75969.17
14511.07	50445.42	120018.7	77474.61	119502.6	103207.9	91524.29	143441.7	91858.86	78167.11

LP416.214	LP416.214	LP416.214	LP416.215	LP416.214	LP416.215	LP416.214	LP416.214	LP416.214	LP416.214
156817.6	71853.01	129688.4	156003.7	126462.3	120559.6	87741.92	108526.6	109678	101776.2
123383.3	146386.4	121333	152643	127558.1	140685.5	84803.75	111251.8	118322.5	122021
122763.2	71616.66	108564.9	105319.4	99131.28	94569.79	86464.21	121865.8	98577.75	95472.28
157426.1	62778.55	189208.1	190948.3	103998.6	197097	84200.32	98160.41	100338.9	96149.53
142646	105932.3	86811.55	153930.6	149623.7	109424.3	77741.69	113797	98590.29	101427.8

LP416.214	LP416.215	LP416.214	LP416.214	LP416.215	LP416.215	LP416.214	LP416.214	LP416.214	LP416.215
104330.7	93203.46	74079.75	121833.3	87513.18	107768.9	90151.24	118854.9	62265.82	89210.66
82692.11	116603.6	78797.24	108661.3	80428.45	97591.15	85982.66	113417.8	67853.82	84641.75
82356.9	108106.9	72061.66	82460.26	80697.02	90524.16	81850.93	113109.5	57110.99	80159
98972.57	101525.4	67789.33	111353.8	88551.4	118669.2	90699.2	104453.1	56827.12	78126.34
75409.24	95386.57	70719.07	88360.5	95067.12	105744.2	84305.4	116164.5	64880.04	82079.64

LP416.215	LP416.214	LP416.214	LP416.214	LP416.214	LP416.214	LP416.214	LP416.214	LP416.215	LP416.214
135948.9	96610.67	95957.85	81405.69	74333.44	98045.68	63994.25	64658.16	102854.3	72027.81
134327.2	104322.7	100794.4	79969.66	72460.07	117846.5	65600.39	56066.83	97631.13	65593.02
144269.9	98529.41	95818.69	84567.12	77129.43	96044.86	55702.48	63484.76	102203.1	52246.36
132941.9	95259.98	100196.3	82519.78	80839.99	82625.91	57700.57	67993.15	97454.64	70912.51
141280.2	85659.25	100467.3	88034.63	92603.37	86013.31	67488.46	59346	92176.37	65394.55

LP416.214	LP416.214	LP416.214	LP416.214	LP416.214	LP416.215	LP416.214	LP416.214	LP416.214	LP416.214
96390.9	58632.88	98189.93	69832.22	85007.39	108052.5	83085.59	98932.48	84754.09	53113.54
101149	61740.27	97419.39	75675.64	84853.77	103757	76141.83	98134	92070.38	51761.22
90744.24	55384.45	99701.88	76905.86	93171.53	72228.38	77461.59	97665.62	93064.05	60288.23
86095.4	55684.75	88441.18	69203.06	84325.46	105537	74676.55	94783.1	77664.7	51420.98
103975.6	59157.22	82694.62	73818.87	87886.99	105678.3	71660.03	120853.2	78190.37	52136.29

LP416.214	LP416.214	LP416.214	LP416.214	LP416.214	LP416.214	LP416.214	LP416.214	LP416.214	LP416.214
67224.29	50452.7	77410.13	60547.42	64980.99	67257.3	67391.25	88043.99	62811.69	51246.43
65669.5	51960.49	79491.44	60567.36	66230.93	62025.22	65976.48	85351.64	57710.89	62207.84
64576.55	49548.19	79771.85	55515.9	61734.12	81980.12	71170.16	79625.36	50861.97	61831.93
64950.59	51143.12	81073.34	55430.97	64102.62	63211.44	64752.4	76832.72	59680.79	53227.23
61842.9	58475.26	82514.88	60526.67	60397.88	64595.1	62164.42	82163.95	59356.36	54939

LP416.214	LP416.215	LP416.238	LP416.300	LP416.300	LP416.300	LP416.300	LP416.300	LP416.300	LP416.300
57086.68	72625.16	69142.21	63110.51	90979.31	53347.53	62433.69	68977.9	91464.66	74723.08
60899.52	73507.51	60256.83	90034.85	60243.98	57401.56	87383.79	84470.28	68632.24	73915.27
56478.36	74612.15	64326.73	73977.98	96216.52	55060.34	62143.63	63284.31	93418.07	80223.94
57076.17	64987.94	61850.51	56907.41	88289.41	60466.75	62243.48	72703.32	57236.29	79711.85
53434.26	70844.67	62434.33	83234.06	84248.84	53441.97	64867.74	62983.98	59297.76	76432.45

LP416.300	LP416.3_3.	LP416.300	LP416.300	LP416.300	LP416.300	LP416.300	LP416.300	LP416.300	LP416.300
72236.45	54289.2	64265.54	66883.74	71609.58	75746.76	65300.97	64497.79	77574.47	68363.5
66680.46	63515.14	63123.74	67984.43	82866.49	68351.06	69234.21	61155.69	79384.36	73140.72
113870.8	56779.36	60274.35	72314.82	88641.04	94065.56	69383.07	66694.27	75066.58	76880.75
65986.31	55254.36	63939.54	66103.21	75443.45	74381.57	68409.84	57821.68	78744.56	68896.14
71814.9	58778.31	61356.12	52815.66	81952.09	77745.45	69126.8	60922.63	68244.97	68629.13

LP416.300	LP416.300	LP416.300	LP416.300	LP416.300	LP416.301	LP416.300	LP416.300	LP416.300	LP416.300
59929	68663.55	63783.42	51292.6	62480.67	56767.94	109934.8	60279.21	56219.98	59052.7
62863.03	85311.46	68418.63	55934.09	65312.32	60602.34	108900.4	71728.07	58754.85	57915.89
61767.12	67183.22	62654.44	66364.18	104228.8	70096.88	102505.3	48173.78	59514.28	61668.23
58880.31	71501.17	66619.14	54788.92	63997.69	57911.2	108031.1	69353.88	51102.21	57371.54
96859.11	73830.47	54505.62	54558.25	61669.07	55492.55	122995.2	67257.04	49637.85	50298.12

LP416.300	LP416.300	LP416.300	LP416.300	LP416.300	LP416.300	LP416.300	LP416.300	LP416.300	LP416.300
85292.02	73691.22	73788.91	69557.74	62101.24	52584.9	58745.85	66707.71	69508.76	69971.28
75976.41	60188.02	71039.61	63586.74	66548.34	53554.21	63697.33	62319.7	62170.62	80482.74
79242.36	71987.2	67858.48	67668.32	60914.21	51537.35	67708.02	68135.78	65281.84	73766.84
73234.6	68242.61	72218.89	70973.54	60706.72	55211.57	62965.99	57005.97	64429.43	66838.92
71263.59	63302.19	64402.1	70570.48	59440.43	48274.24	61202.86	63047.36	62695.29	66659.87

LP416.300	LP416.300	LP416.300	LP416.301	LP416.300	LP416.300	LP416.300	LP416.300	LP416.300	LP416.300
85840.98	68885.63	53564.52	59930.93	87902.54	88684.77	55644.91	75940.89	74930.05	79712.05
83228.99	75858.04	54825.37	62234.63	85353.84	93688.57	56931.39	73552.34	62897.1	82582.09
82556.88	64243.06	54186.19	52497.8	79759.8	86776.35	56140	70061.17	66761.02	71938.9
81789.57	61510.53	54169.92	51569.91	77286.09	86088.94	53012.62	72776.4	68201.78	74825.92
82076.4	70915.61	49695.8	55924.56	80202.35	83549.61	57654.22	69047.36	66814.45	75153.76

LP416.300	LP416.300	LP416.300	LP416.300	LP416.300	LP416.300	LP416.300	LP416.300	LP416.300	LP416.300
58233.34	60196.47	63442.47	72046.23	63649.56	72988.19	56135.39	45811.23	43394.42	52316.31
60854.25	62913.74	61734.37	70362.39	66070.96	73860.91	64662.9	42640.74	46494.87	47041.31
53758.85	62231.58	58169.91	67553.38	66158.17	81417.47	59282.72	48710.92	47310.04	53342.4
61729.99	61392.27	59723.64	69731.45	64490.91	84838.58	52611.49	45313.59	48311.21	55309.16
62310.74	63748.16	61453.6	66341.62	61065.36	76186.37	52732.5	44296.4	45263.38	45161.3

LP416.373	LP416.374	LP416.373	LP416.388	LP416.388	LP416.392	LP416.393	LP416.41_	LP417.173	LP417.173
115952.4	49730.76	185160.3	42413.13	28297.09	24910.62	76336.01	35528.31	148084.7	141168
128880.2	54649.25	191174.4	43102.32	31018.13	26044.74	71538.61	31242.35	144519.9	118119.1
123691.5	53463.43	194514.3	40626.82	33250.66	28065.38	75369.12	38337.13	44455.69	32367.44
125963.6	46402.1	175817	38979.12	27438.15	24352.69	67770.23	27789.96	126696	130803.7
117051.3	45728.28	168355.5	40356.51	27480.65	26788.19	69788.48	30429.85	125736.9	125525.2

LP417.209	LP417.208	LP417.209	LP417.209	LP417.209	LP417.209	LP417.209	LP417.209	LP417.209	LP417.209
76232.81	129396.7	72766.66	99411.76	134012.1	118670.5	123297.8	79384.69	85516.9	95797.47
77204.44	109575.6	75495.62	98035.79	143145.7	116500	123158.5	93913.04	91940.19	92977.74
67004.73	120276.6	72706.21	90767.65	151788.9	127843.6	123847.1	83482.48	81592.44	97110.29
67412.91	123725.9	75148.1	93118.62	137313.8	125207	114968.9	79812.07	84402.71	98918.85
75563.98	124383.1	79209.13	88156.96	140956.6	141531.7	121919.6	91466.85	79593.12	108859.3

LP417.209	LP417.209	LP417.209	LP417.209	LP417.209	LP417.209	LP417.209	LP417.209	LP417.209	LP417.209
54744.56	134405.9	70759.02	72242.45	105122	95202.51	60181.71	69322.33	89144.7	152451.4
56508.56	138445.7	59827.87	75770.17	107541.2	98353.13	61229.35	74672.54	113525.4	154494.5
60474.51	131208.5	63008.89	86553.66	115884.7	97611.6	64734.5	76078.77	116913.4	147423.3
50786.58	124936.6	58536.02	77313.74	117860.9	94815.03	60017.66	76251.03	108181.8	144907.6
60193.54	132823.8	69552.07	70829.18	109054.6	98034.05	63294.98	89009.94	107503.7	169513.3

LP417.209	LP417.209	LP417.209	LP417.209	LP417.209	LP417.209	LP417.209	LP417.209	LP417.209	LP417.209
100502.6	84597.81	126929.5	122265.7	85382.57	81750.45	113385	113236.8	66011.97	117301.9
101130.7	73485.73	120394.5	112815.9	94351.27	88903.32	113117.7	107304.4	72208.24	121450.8
96744.89	87950.02	113539.4	118874.2	85767.65	88698.59	112375.5	121451.8	76787.89	113837.9
104859.8	76139.53	117613.5	120679	83359.27	85278.61	124192.7	115989	62240.78	113068.3
107205.4	73128.48	109789.2	115422.6	78378.85	89092.81	116390.3	99097.27	74402.6	111325.9

LP417.209	LP417.209	LP417.209	LP417.209	LP417.209	LP417.21_	LP417.209	LP417.209	LP417.209	LP417.209
85035.62	78041.16	141388.2	81449.93	71706.6	31189.67	51588.14	117660.7	98841.94	84850.2
87895.75	78633.9	135855.4	80319.43	79608.7	35442.74	50934.51	114699.1	93627.57	80183.14
89241.84	80674.8	126377.9	73380.49	72346.95	28133.19	48704.2	117248	92850.09	85576.79
89448.03	85239.41	117680.7	77895.27	82858.15	25818.33	43761.37	112638.1	95539.04	77233.97
78641.77	84642.22	121777.9	78567.29	73613.23	28135.86	44501.4	110812.8	99106.01	82356.08

LP417.209	LP417.209	LP417.209	LP417.209	LP417.209	LP417.209	LP417.209	LP417.209	LP417.209	LP417.209
99753.05	62678.4	89428.82	61641.12	72145.29	113243.8	60238.23	62137.35	124857.9	81340.92
102025.2	54914.31	93410.09	61695.31	74059.97	103447.2	62862.67	59815.22	131061.9	82435.92
100379.5	64642.61	91274.65	59300.24	76057.91	107374	57277.54	58187.71	121943.8	86021.47
95172.98	64055.34	106253.2	60344.56	68225.41	114055.2	57038.19	49579.79	126516.8	83206.72
104537.1	63423.86	98422.26	67651.8	75644.44	109609.3	59360.2	58405.55	132049.1	84579.53

LP417.209	LP417.209	LP417.209	LP417.209	LP417.209	LP417.209	LP417.209	LP417.209	LP417.209	LP417.210
78798.99	102085.4	103848.4	81779.44	58221.91	78018.12	121951	82772.51	24768.32	16088.74
78350.5	107673.5	99105.96	81622.17	61490.06	82870.04	129864.5	82440.02	24169.95	21319.33
74354.65	107953.5	113576.4	88306.75	57719.53	71123.34	120942	84808.89	25812.28	18423.75
63538.71	95511.4	99449.91	84526.99	55896.7	75426.95	127537	84928.21	21355.63	16143.95
73430.38	106007.1	108847.8	84429.2	60459.46	83429.15	129409.3	89557.97	27162.49	18857.92

LP417.209	LP417.209	LP417.209	LP417.209	LP417.209	LP417.21_	LP417.209	LP417.21_	LP417.209	LP417.209
55562.59	49662.43	48809.65	34059.22	127975.8	20728.31	22859.9	18853.97	88918.8	67150.88
62478.5	49024.02	49095.22	38333.49	119889.5	20220.43	24250.36	17134.11	93011.85	60412.73
51431.73	46631.95	48064.86	33457.64	126126	14926.57	19591.95	17223.13	90277.05	61623.51
53486.08	42986.82	48389.07	26285.39	127484.6	15440.05	18407.21	14646.33	91331.46	61710.24
56640.98	46227.99	46875.24	35328.14	127875.1	18132.73	25164	18187.06	90425.49	65484.82

LP417.209	LP417.209	LP417.21_	LP417.209	LP417.210	LP417.209	LP417.209	LP417.209	LP417.209	LP417.209
95219.86	105340	25267.55	56902.63	22424.18	34966.42	77454.82	109003.7	105210	35852.53
97906.41	107193.5	24819.87	55378.03	19644.38	34899.42	79345.27	109448.9	97809.38	38249.72
100645.4	105595.9	25577.41	63091.76	22881.22	29921.61	81204.33	113434	104096.8	29883.08
94661.02	113016.3	20224.26	56887.6	17654.17	29340.08	75768.84	108477	95994.09	26776.78
104146.8	112774.2	25319.56	59022.63	25829.63	34429.19	76404.26	108476.7	99139.7	31690.13

LP417.209	LP417.21_	LP417.247	LP417.247	LP417.367	LP417.368	LP418.243	LP418.243	LP418.243	LP418.243
38014.17	43016.75	151501.7	104042.1	37134.69	37881.53	58874.02	67962.05	66312.86	60324.75
37705.86	38305.33	34376.41	25525.29	38730.54	41964.41	66283.43	59135.1	65662.95	57301.07
27122.72	40928.04	154969	104588.2	38993.92	37290.9	62319.98	65578.59	59903.99	46257.41
30591.88	35764.34	149049.5	101482.6	28772.27	35229.08	65507.49	56771.55	63712.88	56035.46
35912.03	39873.23	148625.3	116833.9	26453.4	30617.03	66781.94	63838.15	64239.46	53959.34

LP418.243	LP418.243	LP418.243	LP418.243	LP418.243	LP418.243	LP418.243	LP418.243	LP418.243	LP418.243
42863.71	61257.67	42028.69	51112.26	59869.83	54949.98	44136.51	47571.95	45264.43	61353.17
45715.89	60102.67	47649.52	46612.34	64574.2	54547.81	46488.76	44939.74	43926.8	61767.73
41843.08	59092.74	49047.88	47389.21	64807.63	52394.85	42450.24	46259.42	37664.78	61497.74
41760.63	58569.52	47087.71	54841.9	63058.82	51395.9	38990.43	43857.72	46326.86	64344.94
43782.81	67735.8	43404.32	51168.78	61308.06	54891.91	41409.59	43878.89	49881.79	63020.47

LP418.243	LP418.243	LP418.243	LP418.243	LP418.243	LP418.243	LP418.243	LP418.243	LP418.243	LP418.243
62073.23	53003.16	39902.82	56942.85	49646.73	44933.33	45273.97	38463.87	70333.45	50610.04
49448.58	50139.29	45169.78	50297.02	47688.32	42716.75	38566.19	40167.25	75143.25	55937.27
50292.31	49873.87	41662.48	49823.78	51647.51	52006.71	39249.98	38634.36	68633.54	49565.46
54854.31	53617.73	41101.15	54105.79	52481.94	45389.94	39552.39	38070.47	63558.9	51814.08
55828.65	51812.22	44491.64	52114.65	46818.56	48602.68	38638.47	40538.92	76327.42	50504.15

LP418.243	LP418.243	LP418.243	LP418.243	LP418.243	LP418.243	LP418.243	LP418.279	LP418.280	LP418.280
58343.12	53980.15	81056.44	53241.31	36771.97	51286.67	42302.77	169601	144013.8	97003.95
62324.11	46886.26	81028.22	54824.67	41662.96	39069.94	35001.53	156846.1	152835	109246
59859.76	52680.02	75391.22	55114.66	38261.61	52062.25	35665.95	137090.8	134338.4	77356.53
62076.12	49860.7	71648.7	59686.63	38711.04	42997.89	34817.53	159182.7	132138.2	106574.7
57931.61	44510.83	69351.65	56824.87	35731.1	42210.32	34290.99	143068.5	126397.4	109222.5

LP418.279	LP418.279	LP418.28_	LP418.280	LP418.279	LP418.280	LP418.280	LP418.280	LP418.280	LP418.279
87791.8	132904.5	148757.3	146978.5	112061.2	122796.5	126597	98468.72	100318.7	106865.2
93565.67	127162.6	108632.3	137374.3	133789.4	103213.6	122285.1	93369.39	99940.53	101788.5
86447.49	108693.5	102860.1	148220.4	123670.2	95432.66	130027.2	111691.9	103808.2	103054
91910.62	115379.9	125197.5	143771.8	124900.7	107666	123265.9	97017.01	117688.1	84946.73
82637.55	92150.66	102456.9	147002.8	140979.7	102494.8	117918.6	102808.6	115903.4	93405.12

LP418.280	LP418.280	LP418.280	LP418.280	LP418.280	LP418.280	LP418.280	LP418.280	LP418.280	LP418.280
110573.9	100295.6	102197.3	101070.8	98500.79	109771.3	112948.6	87690.86	125332.5	80656.31
106550.2	96344.57	106204.8	99825.39	102138.2	101964.3	115066.1	96518.3	117261.1	89872.74
103920.2	94747.98	112088.5	105205.2	92936.45	96172.34	114768.6	133027.1	116601	89133.46
97718.01	96397.58	120944.6	109005.3	141571.8	99204.94	133647.2	86384.35	124357.6	82482.48
103770.3	98335.59	112065.7	108246.9	94125.38	87750.38	109834.8	92188.12	116657.6	83821.58

LP418.280	LP418.280	LP418.280	LP418.280	LP418.279	LP418.280	LP418.280	LP418.280	LP418.280	LP418.280
105369.3	98275.94	93219.93	109627.3	120650.1	136388.8	61673.46	102253.7	112053.7	81032.72
106867.6	92529.17	93034.49	69852.18	104776.1	122723.3	67636.18	91407.53	100434.9	85130.41
100589.6	96097.48	88224.57	85192.85	98753.33	126970.8	63428.15	117737.2	92858.38	83090.63
95535.73	93539.29	88125.96	67056.16	91095.18	124764.8	59556.93	93209.76	91308.22	85092.01
99867.91	99165.59	85288.19	78566.22	106892.6	115703.6	59942.82	96853.7	93732.99	76399.11

LP418.279	LP418.280	LP418.280	LP418.280	LP418.280	LP418.280	LP418.280	LP418.280	LP418.280	LP418.280
105994	133425.2	80134.69	121053.8	85622.72	102232.9	60458.44	91098.99	63501.36	84424.6
103988.1	135336.6	83604.86	114237.4	83497.5	97368.24	57207.89	98703.33	63589.97	70555.96
114295.1	157104.9	81552.04	123627.1	75383.71	101326.9	53522.35	97845.81	63392.33	82085.01
111863.5	146779.6	80925.16	121806.9	85341.32	85707.03	54103.5	98370.94	58410.58	79917.35
102422.3	134857.4	83967.88	126785.7	83041.84	98049.92	62253.33	102758.1	57050.29	83309.1

LP418.280	LP418.280	LP418.280	LP418.280	LP418.280	LP418.28_	LP418.280	LP418.316	LP418.316	LP418.317
87806.8	86978.67	115116.1	125354.9	110085.1	129944.8	67907.81	136377	170711.3	96121.66
82147.6	75583.85	114017.6	125783.8	113284	121056.8	69634.57	132902.4	104560	93523.87
92872.36	84212.18	111154.3	121404.1	115103.2	117213.9	60553.98	125609.1	167765.7	96719.6
88357.38	86949.36	109072.6	122736.7	107138.5	117407.9	65596.44	126119.5	173519.2	93875.93
86121.22	80339.78	112882.4	119103.7	111997.7	115181.5	70576.63	124494.2	160753.3	96534.92

LP418.317	LP418.316	LP418.316	LP418.316	LP418.316	LP418.352	LP418.352	LP418.353	LP418.353	LP418.353
73349.54	101287.1	81274.3	96295.78	87347.77	58012.09	59843.5	70621.21	47283.03	56445.27
77189.27	99053.22	85893.26	87408.85	58669.53	65117.08	55308.62	66723.41	40695.97	46888.04
66192.75	103400.2	84307.77	98382.17	93195.94	62445.94	59923.97	76716.49	45436.52	55919.53
70747.69	96861.16	78848.33	94749.74	90056.55	59885.19	50343.93	59122.17	44164.73	59784.01
69417.6	98132.26	81720.59	94320.34	92359.23	62863.56	57495.05	66570.02	43520.11	48843.22

LP418.353	LP418.353	LP418.353	LP418.353	LP418.389	LP418.389	LP418.503	LP419.189	LP419.189	LP419.188
36572.96	34451.94	41983.78	33447.17	50005.15	47739.52	37252.34	127303.4	226657.1	487741.4
34747.48	29927.92	37363.58	36478.77	51122.49	50888	39910.92	156141.5	175872.1	296067.3
38007.77	27682.7	35395.36	43436.15	49059.51	47076.52	38034.23	113415.5	179224.8	305879.4
35089.27	28357.72	30388.22	36566.99	53509.12	42601.47	29532.94	190535.9	231815.3	367895.5
35958.95	27739.33	33487.08	34459.56	49422.38	49264.41	35725.44	273024.5	191018.8	349433.1

LP419.189	LP419.188	LP419.188	LP419.189	LP419.189	LP419.189	LP419.189	LP419.189	LP419.189	LP419.188
203382.3	152389.3	198563.9	152868.4	297461.5	224870	309797.8	208755.8	279010.8	159138.6
154539.3	181920.1	199089	170934.6	416999.2	219217.5	386292.3	243625	316653.6	192884.4
183121.2	291856.2	170657.3	219207.8	304275.9	246979.9	242492.5	199057.5	290893.7	150809
136626.6	170867	200146	206306.8	340729	220583.8	252027.3	210818.6	321036.2	183445.6
167595.9	187073.3	192671.9	270516.4	376943.7	237093.2	371693.9	191359.5	305858.6	139929.1

LP419.189	LP419.189	LP419.188	LP419.188	LP419.188	LP419.189	LP419.189	LP419.189	LP419.189	LP419.189
308673.4	382947.1	240561.1	148901.8	201390.8	232068.2	235980.2	224027.3	389475.1	258363.2
413298.4	254318.5	201263.7	187896.4	429238.7	155329.9	147886.7	158284	242574.7	245213.8
385041.1	283396.7	177610.1	193359.2	318818.8	202060.1	147315.4	204376.5	274972.1	254047.2
272236.4	234615.3	200413.4	213453.9	258683.8	137466.4	223240	116662.6	276858.4	211764.5
272373.7	183592.1	140807.5	214252.9	185938.5	219224.6	133672.9	153813.8	246054.5	247476.9

LP419.189	LP419.189	LP419.189	LP419.189	LP419.189	LP419.189	LP419.189	LP419.189	LP419.189	LP419.189
243038.7	238671.5	323123.1	321867	232430.8	178133	310178.7	257982.8	137554.9	135102.7
276551.4	196116.7	334684.7	287712.7	201833.6	216602.1	156402.6	222837.2	165146.8	135014.6
283357.4	299102.2	415727.9	298311	235315.8	247756.3	146699.4	298819.7	190895.4	131953.9
214424.4	180787.6	326080.6	251835	195508.7	222621.2	213790.2	208954.9	177560.5	162680.1
235683.3	159908.9	216904	237601.6	189884.5	176367.7	161872.9	221700.1	134905.3	188164.9

LP419.189	LP419.189	LP419.189	LP419.189	LP419.189	LP419.189	LP419.189	LP419.189	LP419.189	LP419.189
194593.1	151728.8	236959.2	299603.1	230212.9	341039.7	133968.5	230550.9	197393.7	192404.1
247622.2	257015.3	225619.3	246705.7	231216.1	229730.3	131299.9	232057.9	212512.7	205548.6
196671.4	180339.8	253195.2	261385.1	360267.8	321478.9	168324.7	229989.4	204953.4	295351.4
216648.8	156247.2	199782.5	314938.3	258059	206655.7	125846.7	256082.9	200131.3	235139.1
293554.4	136756.7	264828.6	259921.6	248426	313953.9	120971	223270.8	202745.6	207074.6

LP419.189	LP419.189	LP419.189	LP419.189	LP419.189	LP419.189	LP419.189	LP419.189	LP419.189	LP419.188
165879.1	110214.3	118831.4	122236.9	218107.9	117343.5	173933.8	177353.2	158029.2	61141.75
167630.1	147389.5	109491	120473.8	318413.9	120891.6	207715	152031.3	136616.2	63764.09
124869	183969.4	121238.7	137492.3	256248.7	110754.6	214268.4	185431	122546.4	76480.93
174836.9	146970.8	167073.5	138685.3	264702.5	172514.5	190320.1	156333.9	139943.7	57539.1
146002.5	151288	155687.9	104076.7	371966.8	119421.7	185624.6	165361.9	149125.3	49821.07

LP419.189	LP419.189	LP419.189	LP419.189	LP419.188	LP419.189	LP419.189	LP419.189	LP419.189	LP419.189
172202.9	160156.3	277207	245742.1	284409.1	155495.7	165395.9	200828.3	141937.7	218238.4
181586.4	162496.7	247752	144331.9	199010.7	151260.5	124313.2	187905.6	147562.6	220695.3
131063.3	149311.9	241034	186617.7	198272.3	186419.1	118623.7	209106.8	97061.67	237189.6
140026.1	184118.4	285231.5	159148.6	271485	221082	110267.3	200458.1	103312.2	249168.8
163390.1	144483.8	311476.9	190264.2	218725	119145.7	161475.8	184752.2	109961.7	236381.8

LP419.189	LP419.189	LP419.189	LP419.189	LP419.189	LP419.189	LP419.189	LP419.189	LP419.189	LP419.189
298433.8	68841.63	182443.3	92883.46	196231.9	245967.3	126027.7	145959.1	109369.4	145684.2
190769	67338.29	178732.6	97694.06	163124.2	155851.9	135151.4	124244.9	116015.4	147580.8
269359.7	54014.93	171770.3	98545.82	180212.2	216620.9	189167.2	163418.8	83926.01	159122.9
219299.8	45452.83	152072	89538.37	181014.4	225146.7	120644.4	135834.7	97726.16	132341
215020.5	72862.04	169767.6	111241.3	206704.4	237444.7	144457.6	115302.6	141541.6	142200.4

LP419.189	LP419.189	LP419.189	LP419.189	LP419.189	LP419.189	LP419.189	LP419.189	LP419.189	LP419.226
60818.75	139660	51485.44	60865.78	62523.91	162625.2	42795.41	103035.4	67430.17	152443.9
50267.03	132384.3	43441.94	57545.69	83042.59	161082.5	60462.4	73855.7	67997.62	31871
41231.48	135103.6	50865.98	55242.35	65966.31	156059.6	41591.39	53416.46	46620.39	152079.7
44860.42	151677.9	67188.17	62436.57	51519.25	155881.8	44693.23	55970.87	57357.92	144932.3
39340.02	136252.4	42579.75	45481.88	77306.82	161560.8	43263.99	78842.18	66836.82	154906

LP419.240	LP419.315	LP419.315	LP419.315	LP419.315	LP419.316	LP419.316	LP419.315	LP419.315	LP419.315
45859.26	4123670	5265570	174232.8	225356.6	334470.4	209183	300407.6	243956.6	211571.7
46392.1	4030896	5445324	201915.5	222330.6	309816.9	213955.1	276635.3	241869.4	181688.3
43726.82	4229779	5971577	185922.4	231187	353438.6	193035.3	304568.4	233522	214005.5
57588.59	4297621	5383300	178873.1	236729.6	360808.9	210296.8	302353.8	256167.3	183416.4
57825.08	4161221	5231012	187385.3	236950.6	354159.4	194772.6	322496.5	267420.4	213105.1

LP419.315	LP419.315	LP419.314	LP419.316	LP419.315	LP419.315	LP419.315	LP419.315	LP419.315	LP419.315
275226.5	258484	204080.2	216770.8	259247.1	269984	196076.1	135479.3	173645.6	176943.9
251950.3	250615.8	194824.2	212162.1	258224.3	268542.4	202307.9	145849	162747	172517.6
258551.2	241086.2	186153.2	234080.8	257272.9	281620.7	212824	134122.4	154363.7	182193.5
265516	284395.1	219656.2	232831.4	261921.6	293391.2	206973.2	149115.1	158503.3	187017.4
269531.5	276457.8	211201.2	241274.6	258867	294203.3	213457.2	146733.5	174003.8	186380.5

LP419.316	LP419.352	LP419.391	LP420.180	LP420.192	LP420.192	LP420.193	LP420.192	LP420.192	LP420.259
293026.4	17528.88	15038.27	192670.7	60386.22	40622.35	47166.23	85706.61	49859.85	139371
303606.5	21144.53	15311.26	178828.9	63081.17	37729.59	47746.08	87294.9	47012.41	565251.1
296117.5	16349.12	15349.05	235237.2	60973.07	45173.57	45258.98	85652.55	43921.3	555056.9
312866.1	17002.62	15783.41	218171.7	59025.32	40341.7	47954.75	99392.87	47015.13	151483.8
327127.3	22036.81	13143.37	178939.9	55939.5	41541.86	49796.15	93923.16	42433.97	549670.9

LP420.259	LP420.259	LP420.259	LP420.258	LP420.259	LP420.259	LP420.259	LP420.259	LP420.259	LP420.259
155874.9	398211.7	128222.1	173468.5	189068.6	112185.7	171508.6	129489.1	188487.6	217920.1
140537.7	411831.3	95401.57	148807.4	188286	121030.2	187958.9	125100.1	191223.7	235115.5
174832.9	413469.5	124616.8	156911	195713.3	122402.8	187676.6	136007.8	196858.8	221115.4
175067.6	254712.9	128025.5	165981.1	197823.4	110226.9	193877.7	93826.65	194313	176288.4
83717.43	192903.3	120859.7	164966.2	126121.4	121722.3	182903.5	151929.9	182611.8	128661

LP420.259	LP420.259	LP420.259	LP420.259	LP420.259	LP420.259	LP420.259	LP420.259	LP420.259	LP420.259
192707.4	120331.8	149317.3	135209.5	137648.4	89902.47	183083	206954.1	111546.3	91196.53
127237.3	122119.3	156847.3	116924.1	275530.4	95409.35	190862.2	217533.4	114795	82073.27
197619.6	131399.4	156942.4	142513.2	272483.1	101821.7	168898.6	216471.5	112653.6	75305.1
190881.2	123322.3	154326.6	127762.4	275688.5	91642.83	191210.9	222882.6	109305	88248.33
178754.4	129339.6	143508.8	135016.3	263864.6	88951.43	186495.6	155864.6	115202	74361.12

LP420.258	LP420.259	LP420.259	LP420.259	LP420.259	LP420.259	LP420.259	LP420.259	LP420.259	LP420.259
137534.4	231806.6	249228.7	106589.6	141477.4	199768.3	106645.4	163193.9	157473.4	169847.8
139822.7	233506.1	262381.6	111986.7	143795.9	194747.1	89726.19	172080.9	142300.3	155837.7
127133.7	218060.7	256294.8	120342.6	142595.9	185115.6	95324.11	168464.7	160549.5	175172.3
130802	232079.9	264338.3	88096.25	127579.1	167572.9	84416.24	175831.5	159489.9	166213.8
127068.1	228716.7	266738.4	119040.7	131328.1	180892.5	91715.61	161573	157662.6	164709.6

LP420.259	LP420.259	LP420.259	LP420.259	LP420.259	LP420.259	LP420.259	LP420.258	LP420.259	LP420.259
129496.5	140695.7	119769.6	171518.1	143956.2	192080.7	115999.3	121149	182932.5	107671.7
137203.7	159269.8	155606.6	203074.6	152027.3	198138.5	113256.7	140917.9	174807.9	125366.7
125009.8	139740.1	152975.6	198566.8	152941.7	219733.6	110842.7	141151.7	179048.4	126141.8
87863.44	135352.3	137864.3	202949.3	102093.4	215498	111887.9	144112.1	175534.4	126988.4
126720.7	135668.6	146613.2	190104	141053.8	125547.7	113952.4	136374.4	122370.2	127205.2

LP420.259	LP420.295	LP420.295	LP420.295	LP420.296	LP420.295	LP420.296	LP420.296	LP420.296	LP420.295
133172.1	346587.4	175677.8	155510.9	289085.1	205168.3	237220.6	170178.8	204962.4	157123.7
132159.6	167514.2	162372.5	186273.2	263953.8	203437.6	191826.7	166919.9	211503.5	118057.7
137805.3	210054.6	165460.2	210137	235872.2	156099.5	194261.8	170036.3	260870.5	159395.6
133388.1	132844.9	169303	198843.6	302276.8	196798.6	173912.3	162410.7	213367.5	140475.7
139304.6	237403.7	148468.7	171671.3	319332	203261	193200.3	168443.1	203202.5	125913

LP420.295	LP420.296	LP420.296	LP420.296	LP420.296	LP420.295	LP420.295	LP420.295	LP420.296	LP420.296
233624.8	230233.8	207607.4	147424.9	269772.8	279684.9	128069.7	161990.2	199915.8	131793.5
189249.2	202316.7	232317.1	155259.4	249338.2	252068.7	116643.4	148790.9	174360.6	143843.4
217730.6	244011.7	203973	271651	258903.5	245265.9	122090	160479.8	162103.7	145116.5
197501.7	285923.8	231274.3	150041.6	333272	284253.9	126483.2	161503.7	163690.4	146399.6
117951.6	231168.8	217372.3	158327.4	265144.7	285352.4	115952.6	158510.4	196878.6	119836.1

LP420.296	LP420.296	LP420.296	LP420.296	LP420.295	LP420.296	LP420.296	LP420.296	LP420.296	LP420.296
145545.2	174778.7	168009.9	183275.6	103767.1	273492.2	161285.3	630054.3	160800.3	136347.4
160188.4	247755.2	168092.8	171563.7	121893.6	278144.2	156687.3	656515.9	156452.8	136216
166554.1	236150.1	151525.7	167331	124821.1	126088.4	155354.8	613741.6	148438.9	153491.2
187737.2	237866.8	167150.3	164127	126988.8	291688.6	161384.2	585933	152131.7	145490.5
139641.7	250232	234303.5	162171.1	120575.5	293767.2	174408.3	585369.4	160709.5	133598.5

LP420.295	LP420.296	LP420.296	LP420.295	LP420.296	LP420.296	LP420.296	LP420.296	LP420.296	LP420.296
140563.1	144942.9	241563.1	178536.3	201356	165620.1	183357.1	160751.5	142772	119911.2
123751	140524.9	189116.1	162237	164970.7	165901	192288.7	198343.2	180094.2	127628.3
111979.6	111052.3	168272.9	154895.9	181025.5	172576.1	191299.9	166450.4	182047.5	114194.7
117882.4	145806.2	185471.8	159921.2	160901.7	163312.4	284941.4	166815	181898.9	153787.7
118975.3	141738.2	143774.6	153832	161021.6	174483.6	203259	152481.3	179381.3	114034.4

LP420.296	LP420.295	LP420.296	LP420.296	LP420.295	LP420.296	LP420.295	LP420.296	LP420.296	LP420.296
138298.6	153259.6	123483.2	197652.7	100915.2	134956.9	114394	124368.3	167359	176564.3
146672.8	159450.7	97576.34	179081.3	110308.8	145832.2	126148.2	125639	150612.2	144583.3
121530	197415.3	132927.9	142348.2	97102.55	155260.1	113621.2	171858.8	152437.6	141732.5
136103.3	149447.7	123122.9	193027.4	108122.7	125976.5	124827.6	107679.9	128917.7	163994.1
126105.7	161233.5	131387.2	195624.5	111268.3	135958.8	114907.3	112272.9	138196.5	143987

LP420.296	LP420.296	LP420.295	LP420.296	LP420.296	LP420.296	LP420.295	LP420.296	LP420.296	LP420.296
134921	206948	152343.5	134656.5	171775.8	184291.5	159404.3	212107.5	187458.2	116514.8
140041.2	142862.2	157013.7	142920.9	174125	138809.1	126807.3	209417.9	182009.9	102348
142452.8	130638.5	147315.2	192381.5	172414	133514.5	116591.2	207775.7	199401.5	105414.7
147699.6	129275.7	143310.5	142852.9	180778.2	125230.2	126409.5	210997.5	172887.7	97720.68
136334.5	126677.2	168076.9	146031.2	160963.5	130230	116535.2	222532.3	181251.4	99427.3

LP420.296	LP420.295	LP420.296	LP420.319	LP420.319	LP420.332	LP420.332	LP420.332	LP421.141	LP421.180
173381.9	102972.4	357342.9	1023714	532510.3	148342.8	204297.8	74658.36	19902.02	25430.57
156603.2	89498.74	365919.5	696079.2	622235.6	152177.3	204994.5	77934.28	15954.54	25635.62
146976.2	99176.42	377141.9	842637.2	636758.2	140000.4	195718.5	88641.02	16693.58	37977.09
146810.3	88094.88	378932.1	815207	596656.7	148855.6	193048.7	80416.16	13221.29	39093.1
149815.2	89437.29	365844.2	743598.2	418162.3	142196.5	193345.1	75740.32	13494.67	34067.87

LP421.251	LP421.253	LP421.298	LP421.298	LP421.298	LP421.299	LP421.320	LP421.328	LP421.333	LP421.416
598808.5	141072.3	64243.02	52543.97	74903.74	46108.68	389582.4	48132.47	16631.32	27938.56
495277.1	146354.3	52857.19	46575.63	54534.18	63916.84	362412.2	57167.09	18932.93	30016.29
632207.8	182799.9	57661.66	49580.55	57579.98	56571.61	475253.7	54526.87	18765.71	29530.31
630473.3	201236.2	56801.31	42975.59	58113.84	57285.39	473732.5	50856.64	18146.07	26491.41
433967	223140.2	62990.21	46340.45	59533.07	50725.78	357910.6	53668.58	19505.44	23506.4

LP422.181	LP422.183	LP422.237	LP422.238	LP422.238	LP422.238	LP422.239	LP422.238	LP422.238	LP422.238
41749.55	60638.99	34707.48	78957.01	72966.32	63456.64	53507.41	37701.14	42196.54	53487.57
36788.32	63314	34387.33	66674.52	78811.89	65275.09	56571.58	35218.92	51059.52	49259.38
38715.77	55079.38	30820.96	73806.49	77890.63	55667.32	57644.97	36971.79	43875.7	45723.93
40353.38	52764.86	33786.87	71198.97	76570.63	62975.8	64490.4	34322.24	45821.63	54014.39
49505.1	53984.48	33429.85	71017.94	83661.83	61742.29	53785.57	38378.73	49004.1	51759.53

LP422.238	LP422.238	LP422.238	LP422.239	LP422.239	LP422.238	LP422.239	LP422.239	LP422.239	LP422.254
41981.44	59937.25	87898.76	33581.95	50046.22	56334.35	56859.29	43363.25	62739.97	190395.1
38361.01	59630.79	80451.15	34903.39	52629.38	54882.05	62170.62	42796.16	57019.86	190197
45032.33	58096.48	83362.87	39486.59	49945.04	45267.28	67637.2	44015.61	57045.74	143067.1
42220.51	61427.28	69668.83	34455.9	48109.57	60313.55	67128.53	48202.28	69158.32	137039.6
48693.63	55131.99	84091.24	34911.18	47235.98	50338.12	65800.25	40054.86	61273.66	167120.8

LP422.257	LP422.274	LP422.275	LP422.275	LP422.275	LP422.274	LP422.275	LP422.274	LP422.275	LP422.275
162539.9	101994.7	127360.1	133592.4	126949.2	102479.2	106388.9	90220.01	173077.8	125326.4
158039.7	146416	170021.4	115137.2	117026.1	124546.7	134404.8	116555	107688.1	89854.39
151310.6	226150.5	167791.6	251948.6	125292.5	120014.7	111699.2	106593	117837.9	123102.5
155551.1	118121.8	116364.5	133452.5	99607.99	135938.9	112199.3	122095.8	107538.7	126823.9
136196.6	161539.7	107307.1	167470.4	116083.6	110944.9	120185.9	100497.7	105666.3	121221.1

LP422.275	LP422.275	LP422.275	LP422.275	LP422.275	LP422.275	LP422.275	LP422.275	LP422.275	LP422.275
85621.49	76217.9	145654.5	99004.3	99807.25	107184.8	110902.6	117869	147648.8	122627
160998.3	104219.4	146372.2	136468.2	87559.75	117205	114563.6	91906.92	152571	150448.8
87811.41	87537.83	131263.8	98972.74	80826.95	114849.8	115803.3	113771.9	155570.1	124529.1
108853.5	78495.74	168121.5	126959.8	77737.52	103616.1	113921.6	121557.5	124125	125801.8
87617.78	102647.3	141371.5	100622.4	81116.32	96050.95	106577.4	119152.8	151092.2	113290

LP422.275	LP422.275	LP422.275	LP422.275	LP422.275	LP422.275	LP422.275	LP422.275	LP422.275	LP422.275
84188.45	96389.08	150562.7	111977.6	83376.98	118326.5	74106.5	101264.7	108076.8	100434.5
96499.61	94263.29	139565.5	119180.1	106675.7	106349.6	101705.8	99355.43	116352.3	101345.6
90136.94	91606.84	141180.1	114642.7	114205.5	104108.7	103197.8	107537.7	108945.4	96635.86
82996.77	153752.4	139783.5	114135.6	79520.44	166124.6	60897.17	90994.99	106990.7	95441.66
89978.5	89395.7	156721.5	111744.8	82531.26	102159	65877.65	98325.37	99432.39	101078.7

LP422.275	LP422.275	LP422.275	LP422.275	LP422.275	LP422.275	LP422.274	LP422.275	LP422.275	LP422.275
86505.14	104365	143271.5	93056.46	101612.8	105954	79227.57	62408.35	77340.51	73172.45
85750.02	112695.7	153011.5	94631.5	96317.98	100669.7	86402.55	66840.16	83953.85	72916.42
83388.31	114800.5	148294.2	88975.99	104802	100892.5	81295.53	61879.51	81265.75	71273.85
99697.92	105201.9	141556.2	93670.71	106066.5	91968.1	74731.69	57591.83	78773.2	72680.02
86643.71	110187.5	142157.1	88114.38	105619.9	107670.4	99145.95	57301.28	88518.32	73055.97

LP422.275	LP422.275	LP422.275	LP422.275	LP422.275	LP422.275	LP422.275	LP422.275	LP422.275	LP422.275
105490.7	80189.52	78401.65	95785.75	99973.48	79704.26	99257.8	113688.2	80923.6	91431.57
97714.15	78700.05	86123.72	89426.21	97793.68	91490.72	98064.44	108528.6	91115.12	98404.66
91430.01	86483.14	79400.41	92789.74	98502.46	88289.85	100269.8	105598.4	86654.32	89968.49
92741.23	70355.34	80701.06	82775.62	100542.5	80518.77	91475.81	111092.7	85446.42	85205.61
110280.6	88974.38	68293.55	99882.79	93561.93	77499.16	105551.2	120777.8	87026.71	89307.59

LP422.275	LP422.275	LP422.274	LP422.274	LP422.275	LP422.275	LP422.275	LP422.275	LP422.275	LP422.293
147130.6	133203	89240.99	101400.5	68263.68	85290.69	153287.5	85758.12	94739.04	36737.19
146320.2	126593.8	85930.87	98386.93	69951.08	79090.24	152454.6	89466.1	95631.19	102141.2
149942.4	128663.8	86599.68	94523.47	68407.76	95210.09	144343.2	76426.81	85041.37	118316.1
134741	138802.6	96539.28	93777.88	62465.97	83217.89	150375.8	92778.18	88220.96	122605.5
138310.5	121875.4	87516.83	88937.31	61929.09	87803.52	143911.6	75896.76	94831.79	105614.7

LP422.311	LP422.312	LP422.311	LP422.311	LP422.312	LP422.312	LP422.311	LP422.312	LP422.312	LP422.311
132871.6	192712.3	88466.12	60820.57	101608.7	109571.2	116665.4	97358.99	128179.8	66612.82
124544.2	177950.7	88358.31	59142.72	102302	120999.4	102393.6	99252.41	128135.8	65880.51
135382.7	181373.8	103289.7	58024.62	98763.34	115559.4	108868.4	91688.32	129015.1	61448.76
130580.6	192443.4	92454.95	56400.56	96874.94	114232.6	106927.3	97087.32	133454.7	61482.55
131736.5	190984.4	100106.4	57781.69	99054.84	116636.1	104848.8	100234.9	132941	61425.63

LP422.311	LP422.312	LP422.312	LP422.312	LP422.312	LP422.312	LP422.312	LP422.312	LP422.312	LP422.312
71869.24	73505.59	90823	123326.3	98559.85	97840.89	113285	85375.32	78448.36	149503.7
76901.24	78120.88	91467.15	120238.7	94114.93	98343.21	117126.9	79073.78	69280.22	144270.2
83745.56	81173.7	88282.9	118745.1	101919.2	85431.54	114883.6	84923.25	78973.23	150117.1
77668.87	80203.67	86002.37	126124.6	90935.73	93911.64	108695.1	88773.46	80371.54	155284.2
82009.5	78529.49	98608.3	124780.5	98428.56	104323.1	102217.8	79974.47	81484.59	141448.1

LP422.312	LP422.311	LP422.312	LP422.312	LP422.311	LP422.311	LP422.311	LP422.312	LP422.311	LP422.311
84618.23	71013.7	76242.66	67029.51	123495.5	104712.3	112928	88153.94	94831.82	86852.53
81631.11	73739.82	75076.88	70068.71	132319.7	116290.5	102413.6	86811.55	98873.06	83504.32
84301.46	64907.87	80824.19	68511.42	132305.8	116951.8	110841.5	96219.15	102828.7	88117.07
87202.98	67751.08	78747.07	66155.73	128357.5	113075.1	107891.4	90566.08	92091.88	85645.5
81273.12	64690.97	81807.57	70580.3	132426.4	110935	104877.7	90251.36	97612.03	89809.51

LP422.311	LP422.311	LP422.312	LP422.311	LP422.312	LP422.311	LP422.311	LP422.311	LP422.311	LP422.311
117054.8	162915.7	95808.04	78227.23	96629.02	100881.8	55626.57	83994.48	83347.55	164984.2
121722.4	148674.9	102773.7	82271.16	101548.9	90739.42	61732.49	85132.42	87276.61	152366.5
119544.4	169410.8	103365.5	80434.23	101586.1	93615.69	55721.05	83658.42	99313.01	160287.2
123015	150727	105138.2	81117.6	97859.72	85807.09	48905.9	93153.58	97703.49	149512.3
122596.9	167761.9	91014.45	79827.08	103670.8	103228.8	50011.76	87460.35	100786.8	158283.2

LP422.312	LP422.311	LP422.310	LP422.311	LP422.311	LP422.312	LP422.311	LP422.311	LP422.312	LP422.311
75571.5	109452.9	85601.57	139634.1	79835.25	64632.03	67362.99	62048.21	82766.84	52178.57
69644.65	106015.5	82902.15	143484.1	77488.59	67129.15	67771.1	62316.73	86432.58	53895.03
67613.63	104500.1	89026.19	146902.9	83059.38	73066.57	71424.1	59865.76	82708.98	53523.73
80016.37	110380.8	92764.21	144939.9	81834.29	64081.38	70369.22	62561.65	86781.46	53538.33
73880.86	112612.8	95910.35	147988	83321.22	66095.86	70364.14	69143.91	82611.19	63775.96

LP422.312	LP422.311	LP422.311	LP422.312	LP422.311	LP422.311	LP422.312	LP422.311	LP422.311	LP422.311
69138.72	77585.25	81143.53	92171.12	99918.41	67980.62	85541.43	87936.63	85245.43	65078.27
68270.32	81591.7	74702.77	89334.06	94694.73	68691.31	78463.41	91174.27	87100.86	61471.54
66252.69	73520.65	84332.44	95879.81	102976.3	69983.55	91325.46	86957.9	90464.78	68424.13
68715.21	83547.4	75308.8	96909.98	105671.7	69917.06	87333.16	88937.29	96380.59	66950.93
63958.91	78110.11	73769.35	89876.53	94216.06	70238.86	85668.51	80051.45	78640.25	58521.53

LP422.312	LP422.311	LP422.311	LP422.312	LP422.312	LP422.312	LP422.311	LP422.311	LP422.310	LP422.312
90073.68	95094.51	96164.51	105583.7	100643.7	83409.13	59175.39	56585.97	67921.22	83452.26
92374.49	98423.69	97141.79	98486.34	99473.11	69275.71	62916.07	56142.04	73982.4	90358.53
97158.55	97831.43	98316.91	107219.4	113456.1	74296.93	66375.18	55118.29	64778.67	86041.14
89887.73	92619.89	100246.9	106667.6	107923.1	81352.59	56256.5	61373.02	55798.31	81297.98
96898.35	91661.14	99832.96	104722.2	106231.7	69059.59	56177.22	54994.45	70306.73	93082.53

LP422.312	LP422.311	LP422.311	LP422.311	LP422.311	LP422.312	LP422.325	LP422.421	LP423.178	LP423.237
149263.8	90516.39	89259.65	66332.63	48844.06	109575.6	86882.05	24671.72	35442.86	45647.74
153185.6	82285.52	89830.49	60425.87	60179.86	113217.4	138723.2	24242.66	33086.37	47314.73
150778.5	97615.98	89580.68	66112.37	54357.21	109810.1	140491.3	19390.72	34396.75	45062.79
159372.4	95026.02	90430.45	74931.53	50952.86	107698.5	143093.3	23265.99	29760.08	45116.91
147934.4	90112.26	90881.8	62900.54	50590.71	109338.7	58343.1	25166.58	29081.48	47833.99

LP423.251	LP423.308	LP423.307	LP423.310	LP423.311	LP423.329	LP423.383	LP423.383	LP423.383	LP423.383
126190.3	73593.86	50337.01	50786.28	42401.57	33028.77	39869.66	35138.61	40244.35	49343.16
123294.2	62746.36	51463.5	55162.95	43939.35	42128.09	37774.37	33908.2	45426.21	57465.61
111760.9	74381.47	57185.29	49805.81	42814.93	33286.32	43176.34	31338.69	42060.64	52341.62
103102.1	62181.69	56775.9	51890.11	41350.31	31091.61	35777.93	32957.71	44804.34	50440.83
90050.16	51643.26	54349.81	56953.55	45049.35	29525.27	39504.08	35139.98	41822.47	49918.1

LP423.419	LP423.42_	LP423.42_	LP423.419	LP423.419	LP423.430	LP424.151	LP424.217	LP424.217	LP424.217
37352.14	39981.63	43841.68	28649.6	79557.77	27054.15	23360.55	27992.44	28877.13	36143.18
31564.46	39940.51	46651.14	33193.44	70439.96	30682.56	20598.13	30909.29	21147.5	38840.91
36111.49	40956.06	46808.82	29644.69	82307.69	28251.32	23978.02	24949.14	29798.01	38858.88
29803.05	39129.74	43415.77	28760.23	70757.72	24105.09	26212.7	32331.78	25135.8	37327.17
34450.18	42836.42	45053.81	28942.85	72765.23	27162.22	23118.59	37338.12	32785.97	44687.09

LP424.217	LP424.217	LP424.217	LP424.217	LP424.218	LP424.217	LP424.217	LP424.218	LP424.217	LP424.218
35613.14	34666.47	41763.41	51428.78	57671.89	45574.86	27987.32	25789.47	24034.83	47167.81
37084.99	35624.51	45597.07	49563.29	48116.19	40552.95	26827.48	23782.15	27333.3	45809.31
38661.43	39510.96	48273.78	55159.13	52503.73	42977.17	24888.66	24563.52	24532.15	45736.55
37341.84	35377.59	39848.77	48243.95	55567.91	36981.27	25702.54	24902.66	23596.55	51218.22
39888.17	50629.71	40513.51	54367.83	48289.22	41533.48	33170.02	29906.3	24424.69	45071.89

LP424.217	LP424.217	LP424.218	LP424.254	LP424.254	LP424.265	LP424.290	LP424.290	LP424.290	LP424.290
33165.83	42021.7	29645.55	215316.3	89467.04	134193.8	281077.5	274125.8	146944.4	164860.4
34021.68	43684.01	30501.2	217051.2	85596.75	140694.4	152097.3	146465.4	176982.5	198911.7
38092.66	44868.05	32276.14	227878.9	91886.81	120259.7	200858	291218.5	136286.8	241556.3
36039.59	41106.37	27430.8	234957	85609.71	122551.7	220319.7	159754.1	146739.3	187641.3
38561.55	41213.49	26704.96	222120.8	95178.15	125063.5	162361	246340.3	135144.3	175032.8

LP424.290	LP424.290	LP424.290	LP424.290	LP424.290	LP424.290	LP424.290	LP424.290	LP424.290	LP424.290
164113.7	286739.2	173788.3	241529.8	328038.3	245901.4	153980.8	108210.6	259492.4	159682.6
258608.9	289292.8	158757.5	277902.6	333880.9	235802	168296.5	107378.5	257875.6	163460.7
171709.2	299691.7	143413.1	230717.5	482185.9	234048.5	169599.3	113117.5	228967.6	150974.4
127223.3	190549.7	190770.7	333021	365767.1	219074.8	162965.5	115184.4	198473.6	140465.7
156091.9	217712.7	141596.8	329604.6	252828.5	210593.1	141435.5	104133.7	217665.2	168285.4

LP424.290	LP424.291	LP424.291	LP424.290	LP424.290	LP424.290	LP424.290	LP424.290	LP424.290	LP424.290
139204.7	178504.1	200074.5	167828.5	158237.7	147611.3	212764.5	160173.3	92379.97	108118
140666.8	195907.9	184488	167016.9	238126.1	143278.4	144427.4	117354.7	90191.05	98680.96
144187.2	193569.9	177748.6	157239.9	165978.8	237758.3	162776.3	113612.5	88508.02	87822.18
137068.7	247722.5	199115.5	137247.8	125834.8	157064.4	147477.8	146735	86624.4	95861.63
155507.9	198517.9	198591.2	151792.6	153887.1	132087.9	170106.4	113520.7	86918.61	84485.13

LP424.290	LP424.290	LP424.290	LP424.290	LP424.291	LP424.290	LP424.290	LP424.290	LP424.291	LP424.290
104289.2	185811.6	88138.53	124227.6	156795.2	172891.6	157661.3	88899.68	134866.1	143657.2
105497	150355.1	95801.84	175369.4	160700.2	160744.2	167281.9	93031.03	139312.4	154137.2
109560.9	156495.2	94907.46	116415.8	159432.4	162424.4	155232.3	100481.3	129859.2	158689.3
125313.4	151825.6	100515.2	134224.6	157071.6	214474.4	161457.4	106853.1	141104.2	171820.8
106330.2	151044.3	88431.89	110005.4	154589.3	156888.9	149474.2	89186.76	122969.5	146741.7

LP424.291	LP424.291	LP424.291	LP424.291	LP424.291	LP424.291	LP424.290	LP424.290	LP424.290	LP424.290
132080.2	173086.3	174745.5	152948.1	138534.4	141492.9	98872.29	176607.9	145288.5	97276.32
127536.6	167838.1	205087.4	194362.4	135879	134776	108901	166835.3	141256.9	105344.6
132438.3	178651	174784.3	142489.8	112415.7	133700.4	192557.1	150531.6	143658.8	105702.4
180680.7	214439.1	199814.9	143055.7	181990.6	148642.7	100449	175310.8	122271.6	108369.7
134347	163771.4	184127.9	190617.3	126335.8	126730.8	92971.5	163624.6	135364.7	98603.12

LP424.291	LP424.290	LP424.290	LP424.290	LP424.290	LP424.290	LP424.290	LP424.291	LP424.291	LP424.290
139174.5	198770.7	128631	169754.4	119674.5	178215.9	99485.42	123620.7	157293.3	95040.22
143897.4	220282.7	158627.5	156880.1	111124.1	119638.5	114671.2	129351.6	163137.5	101129.2
157566.3	194860.1	118357	148542.2	124592.5	131245.8	104245.9	140252.2	159264.3	91338.79
137540.9	191249.4	116808.9	148102.7	121172.8	146366.6	114826.2	118269.6	129336.4	96019.03
135736.2	196452.6	116126.2	140071.6	120370.7	109168.1	103900.2	122926.9	148218.7	89865.42

LP424.290	LP424.291	LP424.290	LP424.306	LP424.306	LP424.311	LP424.31_	LP424.326	LP424.326	LP424.327
120103.9	107437.8	133811.9	128417.8	94801.73	88857.7	162181.6	246398.6	214833.6	242006.6
114653.3	115365.9	135301.7	123004.3	88517.64	95264.21	164817.6	249183.3	218060.1	245867.4
114168.7	105864.4	134300.3	122197.6	84065.98	94005.84	156782.2	251471.6	235020.9	251586.4
102257.2	99575.32	134408.9	112104.8	84959.27	100856.1	142773.4	261574	207342.7	252167.6
114990.4	101590.7	144893	114278.2	78633.66	88246.5	147053.9	270704.8	235313.5	48725.05

LP424.363	LP424.363	LP424.363	LP424.451	LP425.214	LP425.214	LP425.214	LP425.214	LP425.214	LP425.214
75792.22	49172.87	41288.27	9090.441	71180.77	79246.92	73617.4	94993.95	92462.64	60526.31
75405.63	45631.36	50049.29	8444.667	42760.97	62393.39	83364.38	114023.5	107735	79455.15
75985.93	39455.64	55369.76	11141.06	50744.65	94593.17	94294.29	82149.15	77218.74	100572.9
74669.36	35784.54	50508.93	17727.87	56801.47	92538.17	85528.41	97125.57	103505.1	57051.04
69722.29	34284.3	53357.14	13346.46	51634.34	79321.82	79068.67	81255.15	69471.32	83920.44

LP425.214[illegible]	LP425.214[illegible]	LP425.214[illegible]	LP425.214[illegible]	LP425.214[illegible]	LP425.214[illegible]	LP425.214[illegible]	LP425.214[illegible]	LP425.214[illegible]	LP425.214[illegible]
74994.65	84699.47	81996.71	70514.41	42968.84	101584.8	83111.57	102851.3	51421.67	57993.11
85120.22	127444.1	87658.04	125006.7	36198.75	65735.98	53306.61	94164.59	53728.7	66654.68
105319.5	133386.5	68383.24	58458.17	30328.56	93246.63	49905.95	69382.47	66569.37	60774.27
110979.6	88865.07	69613.85	68404.63	35982.53	73405.05	81669.64	72403.24	56760.7	85452.13
82282.12	81324.34	57534.22	81218.21	44356.41	92428.3	59715.05	116013	53971.13	63365.14

LP425.214	LP425.214	LP425.214	LP425.214	LP425.214	LP425.214	LP425.214	LP425.214	LP425.214	LP425.214
129535.3	100170.7	96753.03	89760.65	79826.97	111831.5	68240.55	89201.34	108592.3	81049.34
95801.21	108135.9	84823.48	73902.07	107968.6	98276.71	78294.19	93652.66	96207.6	70581.71
59708.85	63225.01	109410.3	118140.4	75754.93	117642.4	63378.53	93365.86	70489.44	62176.76
95039.11	73999.54	103564.7	74516.09	90566.78	88826.57	64956.01	84688.16	127848.5	83450.93
78645.22	108086.4	113471.4	71432.71	101310.7	85832.74	71326.15	124869.5	73484.44	66266.12

LP425.214	LP425.214	LP425.214	LP425.214	LP425.214	LP425.214	LP425.214	LP425.214	LP425.214	LP425.214
39456.62	79934.71	89635.39	62714.73	72659.24	77219.34	51527.32	61412.97	66882.73	92539.06
35241.38	88066.14	141334.9	78608.78	72799.06	65892.25	45080.32	78746.66	75347.24	69500.9
42310.28	56354.57	120050.2	112575.7	95815.2	62936.88	46238.44	73951.7	80875.44	96020.44
43717.1	64237.27	93124.86	95815.06	95822.71	69823.45	49785.02	50848.82	85234.17	82011.97
29719.46	75569.64	63616.56	100192.7	67420	93855.14	43810.49	57554.4	62256.16	79964.6

LP425.214	LP425.214	LP425.214	LP425.214	LP425.214	LP425.214	LP425.214	LP425.214	LP425.214	LP425.214
52075.53	88653.49	90227.15	99369.43	53216.16	109582.1	125334.8	84215.41	84492.28	70428.02
50305.42	84528.32	71127.16	77163.82	60672.84	74756.13	115159.9	78015.14	78181.84	103033.6
40503.55	94767.84	70590.12	109938.8	39885.63	57820.08	87716.19	93360.75	130871.5	74939.51
49849.72	81837.56	57729.95	77302.1	52540.87	86513.19	88400.75	114812.5	57504.57	80820.37
44932.66	116143.1	85804.33	57773.22	40018.61	66879.68	95093.19	95004.67	55535.02	69501.6

LP425.214	LP425.214	LP425.214	LP425.214	LP425.214	LP425.214	LP425.214	LP425.214	LP425.214	LP425.214
60420.88	48797.51	62106.39	101690.7	52193.55	72907.58	67938.35	38493.68	64978.52	78194.87
46241.03	44744.34	63741.71	91096.92	45690.63	63283.77	79904.61	43679.31	90608.3	114221.4
45020.45	52753.77	78311.6	86795.83	49165.26	81697.5	73265.59	40895.45	55996.67	67593.89
42928.82	38293.03	106142.2	80689.76	70776.83	64754.59	68994.84	32610.54	63838.86	73919.08
41842.51	38887.08	60069.64	74291.89	69373.85	83864.64	87981.03	40280.14	73375.67	96513.5

LP425.214[illegible]	LP425.214[illegible]	LP425.214[illegible]	LP425.214[illegible]	LP425.214[illegible]	LP425.214[illegible]	LP425.214[illegible]	LP425.214[illegible]	LP425.214[illegible]	LP425.214[illegible]
63657.31	87099.36	29631.74	84354.74	116844.3	90370.29	67405.91	56014.31	78168.47	68509.46
80886.07	85557.55	52649.03	71671.03	100648.5	80356.9	61742.95	49306.42	83077.39	75800.53
106480.7	87820.72	43616.09	103143.9	84622.33	100294.1	66624.79	55969.27	86736.23	89897.48
85293.92	87049.46	26280.11	103240.6	117719.8	119810.4	74254.33	61868.09	96680.22	93583.32
84411.02	93723.87	45252.82	63450.85	106787.5	92049.02	51018.11	55977.87	77566.12	66948.52

LP425.214[illegible]	LP425.214[illegible]	LP425.214[illegible]	LP425.214[illegible]	LP425.214[illegible]	LP425.214[illegible]	LP425.214[illegible]	LP425.214[illegible]	LP425.214[illegible]	LP425.214[illegible]
68238.84	42555.23	74819.75	64754.81	68621.94	48145.7	101740.3	77075.39	90833.99	44794.36
84465.6	72876.22	101363.4	76297.64	80032.59	52817.16	108354.4	70526.63	85818.47	43855.31
126280	42359.11	73762.55	74396.74	81506.9	43612.78	70829.06	94770.28	88192.91	45582.33
96520.28	45592.19	95090.07	65376.11	67425.83	49740.85	106893.6	78785.08	87351.85	34154.39
71146.01	40370.03	96831.01	43804.09	58486.12	43831.52	98614.71	75715.42	82510.46	37767.5

LP425.214[illegible]	LP425.214[illegible]	LP425.214[illegible]	LP425.214[illegible]	LP425.214[illegible]	LP425.214[illegible]	LP425.214[illegible]	LP425.214[illegible]	LP425.214[illegible]	LP425.214[illegible]
87524.81	81990.01	63190.22	113359	65923.16	64738.43	85643.87	81530.26	87065.91	104136.9
80090.03	57297.53	74612.95	79109.52	74123.53	66735.19	120100.3	67605.98	98265.62	129331.6
81915.83	99088.67	72384.37	68660.51	73318.74	67007.99	55734.74	71749.11	89620.48	100436.1
75290.31	67449.85	76527.27	91224.15	72727.16	74475.45	117633.7	71441.95	72874.83	100112.6
65480.94	86518.45	82740.94	80452.7	99873.58	70965.42	95027.18	82333.99	68431.34	105903.3

LP425.220	LP425.292	LP425.293	LP425.293	LP425.293	LP425.293	LP425.309	LP425.363	LP425.365	LP425.446
44771.99	58025.61	53367.89	46357.57	66854.59	69493.62	20372.65	196478.5	21515.26	320468.7
49069.7	56761.86	46877.14	40340.46	78873.71	56183.09	20533.76	155063	14583.59	281911.6
48817.77	57520.78	54886.17	45594.78	65584.14	68109.95	19063.81	318532.2	17965.43	253823.5
43751.86	55962.62	52466.88	52557.95	69688.38	68793.97	20171.37	147169.4	14951.95	232959.4
48501.34	55031.3	49673.3	48042.66	61104.16	69713.12	19565.89	154972.1	13339.8	240435.6

LP426.233	LP426.233	LP426.233	LP426.234	LP426.233	LP426.233	LP426.234	LP426.232	LP426.233	LP426.234
98219.61	71534.85	111000.1	134827.5	71000.09	67255.55	122111.1	105051.4	61134.97	130033.8
77521.9	66961.57	100375.2	117989	77421.67	78997.63	111297.8	100984.8	81937.65	142696
100696.8	77054.75	115670.6	147742.4	75609.17	80514.64	113086	107017.3	74262.45	146731.7
85907.5	78763.58	99782.78	151214.1	76957.11	69888.43	164736.4	107472.7	72691.46	144160.2
79520.86	77728.41	132898.6	157559.5	75095.93	71937.14	158340.4	95243.87	66421.3	148843.2

LP426.233[illegible]	LP426.233[illegible]	LP426.233[illegible]	LP426.233[illegible]	LP426.233[illegible]	LP426.233[illegible]	LP426.233[illegible]	LP426.234[illegible]	LP426.234[illegible]	LP426.233[illegible]
93694.47	89673.66	74005.42	94399.15	79587.75	108939.1	86230.12	94582.09	91923.12	64028.56
80557.59	91086.11	67933.74	95411.66	78311.19	106276	102378.4	98341.18	92950.3	72385.91
83471.01	98590.54	65870.8	93604.18	79566.85	110286	90199.34	98421.46	87807.63	67834.36
89092.83	104294.6	77963.13	93858.64	77691	119083.2	80571.25	108401.9	102333.9	70652.63
80873.93	79185.7	79699.43	70351.26	98276.6	109409.8	89376.15	98626.85	89037.41	79642.36

LP426.233	LP426.233	LP426.233	LP426.233	LP426.234	LP426.233	LP426.233	LP426.233	LP426.234	LP426.234
82479.63	92394.48	79464.84	79585.82	79747.1	43808.14	104271.5	92371.7	114538.4	58952.18
82415.93	97569.23	75511.96	79499.14	84884.84	40364.9	100742.1	92908.28	114951.1	60724.75
82548.26	93300.02	72066.58	76934.79	78886.77	49055.88	104961.2	96973.71	113081.3	59716.36
82649.45	96225.58	77645.22	74741.32	79329.39	46817.46	96251.65	98130.6	116590.7	63603.29
96745.54	98409.09	79989.72	74652.84	80367.55	56523.97	99226.71	93442.09	106789.5	57633.51

LP426.233	LP426.233	LP426.233	LP426.233	LP426.233	LP426.233	LP426.233	LP426.270	LP426.270	LP426.270
45743.72	71098.32	74182.94	56689.77	83912.93	80518.62	98317.13	70772.07	104460.4	70476.19
41319.56	68508.24	68340.49	64077.31	88031.53	85690.95	89964.21	68392.2	107745	71625.53
44696.67	73885.89	74109.35	68568.47	88649.58	88463.03	96760.69	69598.32	102054.5	71850.7
43639.96	64085.79	77184.45	64710.74	78741.64	82543.24	83908.91	69037.21	95581.63	75470.58
44777.37	75067.69	74220.65	60765.79	89565.33	85453.64	93695.7	60074.39	93074.67	71108.49

LP426.270	LP426.271	LP426.269	LP426.321	LP426.367	LP426.366	LP426.430	LP426.432	LP426.430	LP426.430
55541.1	32767.96	95453.57	46577.39	66361.24	51662.78	141587.3	222907.2	22633.31	27141.38
58855.39	33324.16	102285.9	47948.85	64435.45	54017.09	130225.5	255219.4	21874.66	28878.97
60194.46	31814.24	97354.27	48353.64	62880.66	56979.25	147679.5	262601.2	23789.78	31923.44
59723.69	35249.42	91792.72	46234.36	58797.41	41738.73	129854.6	238214.9	21605.94	27117.53
54125.83	32840.09	88344.58	19515.34	61406.52	52293.65	144167.4	229823.2	22407.11	29030.93

LP426.449	LP427.157	LP427.194	LP427.194	LP427.194	LP427.194	LP427.194	LP427.194	LP427.194	LP427.194
196395.4	24696.19	76572.92	82654.48	94068.85	104548.6	56045.53	77230.23	81275.43	111117.7
198213.6	64878.86	75520.03	71804.29	93561.75	99133.73	58777.75	75562.17	76536.6	102707.4
173888.5	65206.14	78927.28	79950.26	85446.66	105999.9	55110.05	90820.34	81750.87	102952.1
166824.8	58241.46	72317.26	76313.99	89337.51	105873	70181.11	70963.47	72412.25	104323.4
171193.8	62546.71	76858.69	82349.79	94238.08	96638.29	61869.51	83400.31	77649.47	105482.1

LP427.194	LP427.194	LP427.194	LP427.194	LP427.194	LP427.194	LP427.193	LP427.194	LP427.194	LP427.193
78549.33	68356.83	70098.34	72003.89	64060.9	80846.44	79869.77	88652.94	67135.75	89045.43
77845.26	53327.14	68467.57	75440.6	63490.72	79407.45	75116.34	84431	54410.08	100300
80386.5	52960.4	63764.57	78965.97	59966.97	70735.3	76569.89	75206.85	55241.76	96909.67
81456.14	54787.26	63127.1	76684.12	57819.85	78174.34	76678.63	82804.44	53363.11	89967.14
76444.18	54637.64	63487.99	75231.03	58580.36	69158.02	78502.16	94420.47	65368.36	87395.63

LP427.193	LP427.194	LP427.194	LP427.194	LP427.194	LP427.193	LP427.194	LP427.194	LP427.193	LP427.194
60481.69	130287.1	87351.85	86111.58	53923.78	95920.31	64784.86	84617.51	59809.48	149672.4
63613.91	129379.6	93607.78	85096.36	57292.29	99252.27	72358.81	81556.77	59350.81	137382.1
57714.83	123833.9	87763.29	82487	54098.06	105587.1	67822.76	79809.05	59741.07	146546.9
63272.64	127667.7	97224.72	86962.38	56869.07	83087.18	66159.41	86898.35	51689.65	142711.4
58919.63	127903.4	91486.32	89441.97	50560.5	99177.07	59654.74	90118.8	56831.39	141012.4

LP427.193	LP427.194	LP427.194	LP427.194	LP427.193	LP427.194	LP427.194	LP427.194	LP427.194	LP427.230
103967.8	68095.11	60989.95	93412.29	82160.77	104662.8	92975.07	162953.2	80610.09	86590
105904.8	73835.26	64318.75	103032.7	75082.41	109971.2	86830.3	150307.9	74212.21	94672.76
105996.2	71195.51	73074.88	99138.34	78580.46	107072.2	88966.04	160382.1	78021.2	82829.65
102937.9	71874.65	71944.21	93700.46	85058.21	100500.7	85496.2	154882.5	73978.22	85636.79
94074.33	72365.37	74266.84	92467.68	78058.1	99042.9	82624.68	146637	71110.79	115304.5

LP427.230	LP427.230	LP427.230	LP427.231	LP427.230	LP427.230	LP427.230	LP427.230	LP427.230	LP427.230
69193.45	94382.11	67506.19	35167.18	69734.82	85960.27	104479.2	117951.4	76446.36	62085.66
104456.6	79645.99	80251.99	39101.94	66924.69	74654.71	65655.94	69520.98	88239.2	73136.1
85931.82	101666.2	57882.51	38233.11	81193.8	79085.72	122863.7	106838.3	69415.09	55668.26
63735.82	61980.89	99282.88	19995.85	58396.52	117701.2	65199.37	93200.29	64770.39	83464.06
75164.9	79440.65	88473.38	32210.56	72278.52	95078.35	101070.5	76917.72	74948.17	74693.64

LP427.230	LP427.230	LP427.230	LP427.230	LP427.230	LP427.230	LP427.230	LP427.230	LP427.230	LP427.230
78673.54	101297	59626.95	87350.58	81760.83	89199.6	59973.97	57389.2	104150	95853.51
53117.69	88884.43	56047.41	90254.56	63944.54	77746.44	76399.58	63600.14	116353.3	79884.45
78927.74	94755.79	53016.55	95330.84	76922.41	76157.93	103836.6	93153.19	96364.95	101243.6
69547.52	105493.9	89047.72	70976.69	83708.61	76204.07	57259.34	82279.64	82631.12	89472.14
82802	81180.4	68752.32	106647.8	108140.3	72258.42	73660.92	81376.33	79534.8	78647.93

LP427.230	LP427.230	LP427.230	LP427.230	LP427.231	LP427.230	LP427.230	LP427.230	LP427.230	LP427.230
73369.85	48342.55	98322.84	84780.65	31348.08	112883.4	60830.4	70212.15	73317.73	111915.5
72327.07	79838.05	80597.22	85826.18	25863.52	79373.8	69759.04	78483.53	67292.8	74766.55
64455.92	93856.55	73227.56	87437.79	36953.54	62999.35	54563.42	64051.42	91051.22	88613.91
68837.2	77501.06	93059.62	116602.2	28635.64	68464.25	63769.67	64980.18	90561.96	76951.78
89617.46	71536.24	65932.46	77357.34	38111.54	79134.24	82490.66	97260.69	52730.12	81048.38

LP427.230	LP427.230	LP427.230	LP427.230	LP427.230	LP427.230	LP427.230	LP427.230	LP427.230	LP427.230
65176.64	119150.4	104105.3	78728.42	77443.93	118875.4	60161.34	83487.17	74873.95	101415.1
100366.2	122604.2	76911.04	89140.34	58536.85	72558.41	59026.54	110932.1	100284.2	90492.3
95990.26	102293.3	94118.38	71245.31	56689.63	143273.1	69457.47	83046.69	97586.11	92760.4
73790.94	103023.7	103001.1	69758.4	57679.38	84409.61	54553.7	107942.6	60425.71	70319.37
92950.47	87173.52	70463.68	80976.26	70322.14	86083.03	61324.13	91356.83	96209.47	104745.9

LP427.230	LP427.230	LP427.231	LP427.230	LP427.230	LP427.230	LP427.230	LP427.230	LP427.230	LP427.230
94433.68	118267.1	30658.5	96487.9	95045.04	131286.7	86575.03	55181.57	63620.48	51529.98
95571.8	94857.61	43705.26	91486.07	125692.7	105292	93416.22	27588.55	78544.78	90575.31
83019.59	96781.56	37229.94	80097.17	113906	91419.81	74627.43	43348.68	49546.96	65688.24
81525.4	88160.55	41409.77	52470.8	82756.36	120423.3	83251.44	30246.87	79707.96	69286.77
77968.93	70954.54	29548.03	93382.46	136616.4	83152.77	87661.44	28657.65	47212.98	69996.77

LP427.230	LP427.230	LP427.230	LP427.230	LP427.230	LP427.230	LP427.230	LP427.230	LP427.230	LP427.230
87590.78	114334.1	38683.5	85589.77	22703.89	44055.54	70210.07	88118.64	58609.46	62252.25
100222.5	76346.22	37279.05	64192.48	29345.7	33627.31	125887.5	97187.68	54541.28	55322.74
76186.97	100853.4	32340.57	73396.81	33031.41	45845.3	82143.67	90499.9	57903.71	73145.75
112608	91758.22	36582.52	59154.27	28734.9	51364.38	72434.87	141576.5	49678.75	72600.32
110342	91680.75	56697.71	72615.17	34791.39	49626.95	103727.8	92632.55	51334.87	70204.96

LP427.231	LP427.230	LP427.230	LP427.230	LP427.230	LP427.230	LP427.230	LP427.230	LP427.230	LP427.230
45731.74	46850.9	67082.22	100649.7	67553.73	70033.37	86786.18	63508.56	65365.1	69442.61
25432.13	51357.63	52282.71	84611.34	74836.89	65144.63	71886.48	50729.66	74793.33	52890.36
22809.78	51872.42	80275.59	63045.69	59374.18	80751.42	105771.4	60055.9	80158.77	60938.39
28779.63	42461.08	79332.56	65785.48	74353.85	70780.15	83945.48	65463.61	76527.13	56337.51
37565.79	41561.9	64047.02	94271.49	57350.87	71198.85	81288.31	64181.36	68052.15	52741.68

LP427.230	LP427.230	LP427.230	LP427.230	LP427.230	LP427.230	LP427.230	LP427.230	LP427.230	LP427.230
61597.34	39158.56	28024.22	128837.1	46220.21	50525.85	48702.19	84519.65	79558.82	28698.3
49468.84	37729.92	40994.35	116958.2	40645.48	58690.15	33797.06	73196.97	96364.15	34820.31
71029.14	40844.6	40551.4	108953.4	43829.85	59525.62	43150.34	81304.99	61285.84	37755.6
88314.63	42035.03	26480.62	85854.51	51466.13	60207.9	36660.04	68608.51	97528.78	29838.92
56894.61	41426.72	40763.9	88592.36	41768.45	64093.58	31513.11	92893.2	88555.91	36669.79

LP427.230[illegible]	LP427.251[illegible]	LP427.251[illegible]	LP427.251[illegible]	LP427.251[illegible]	LP427.252[illegible]	LP427.290[illegible]	LP427.303[illegible]	LP427.363[illegible]	LP427.377[illegible]
45931.71	59575.42	404066	133580.2	26154.83	21172.06	42164.83	19881.47	28331.27	58889.34
43188.59	61273.55	464756.9	144842	26368.35	20528.55	50554.48	25256.82	23795.23	61934.17
37292.42	74548.16	704744	204498.6	28733.8	21544.54	52067.09	22095.32	19982.75	59624.71
38201	81887.46	858432.5	229232.8	28514.16	17537.67	57573.68	19988.03	21116.46	64096.15
39099.15	87921.87	935978.9	257405.3	27782.2	20990.37	59220.96	21339.95	26022.24	59621.79

LP427.378	LP427.378	LP427.378	LP427.389	LP427.389	LP427.434	LP427.435	LP427.462	LP427.462	LP427.462
1117279	267992.4	136128.8	113843.5	40664	65639.69	50794.51	7610.739	7303.946	6298.534
1709733	252264.4	133315.9	105789.3	38235.64	750132.8	54607.37	8498.571	8144.579	6984.488
1010135	230337.1	127549.5	105216.4	49905.51	108425.6	48539.47	13296.69	12730.31	10874.16
1379473	287027.8	136000.6	96404.74	33127.92	128186	38798.54	16155.28	15447.3	13127.12
1408528	241964.5	138225.2	101539.9	32532.12	817920.4	46628.21	17919.81	17141.03	14588.83

LP428.248	LP428.249	LP428.249	LP428.249	LP428.249	LP428.249	LP428.248	LP428.249	LP428.249	LP428.249
103059.2	80702.26	124948.3	91611.71	46513.68	112067.3	69749.86	63200.86	65628.58	41217.47
94598.01	86247.94	119715.7	130934	59066.85	119489.1	61935.71	74442.14	78461.27	38363.62
90878.09	104201.9	123843.6	87574.16	54745.13	105522.4	65879.72	71193.08	84662.07	39402.54
72776.45	78694.87	108470.6	69443.18	45450.58	76240.13	67323.74	60961.03	55020.16	36664.72
86549.69	83637.02	108858.8	83243.24	47164.67	106844.5	60457.37	68799.23	68171.18	40681.31

LP428.250	LP428.249	LP428.249	LP428.248	LP428.249	LP428.249	LP428.249	LP428.292	LP428.301	LP428.311
36956.41	102651	64522.76	40322.79	64567.98	59633	103024.1	23252	34256.21	35828.08
32901.09	98112.75	61660.7	34292.75	66422.77	62963.72	91383.77	20861.48	39556.91	34668.49
37165.59	90726.34	48435.93	35684.35	50396.63	64636.51	75201.66	27105.26	49181.84	47823.95
46194.38	102076.4	65974.53	39250.34	46608.33	58122.35	57417.36	26736.07	38993.49	44876.21
42911.09	103965.7	50798.39	33958.88	58337.7	59695.74	96373.7	23478.6	44367.56	45967.98

LP428.337	LP428.338	LP428.338	LP428.338	LP428.338	LP428.382	LP428.393	LP429.173	LP429.173	LP429.173
31727	40228.81	30194.39	56983.51	23854.49	270085.1	23867.25	119855.4	75217.46	99883.82
42175.88	44345.31	28408	58843.34	24864.35	276763.3	27815.41	113795.1	71003.08	98513.81
38777.11	40109.35	39070.4	59416.51	26033.36	378539.1	25340.3	107129.1	66890.81	94462.39
47591.36	58924.74	48878.41	70458.88	30608.1	259661.1	24047.86	109373.4	67627.91	88937.52
39194.69	46097.86	41357.42	59291.71	32274.46	268691.8	24687.41	101598.9	72348.79	95882.36

LP429.174	LP429.173	LP429.174	LP429.174	LP429.174	LP429.174	LP429.174	LP429.174	LP429.173	LP429.21_
154874.2	97281.19	201114.9	146559.9	180854.4	172243.1	188987.9	139826.9	184074.6	117156.5
154290.1	98318.87	186594.6	154105.1	180486.4	180495.7	182450.4	128873.1	183702.2	112522.8
142225.4	94196.1	192495.6	146338.4	174938.1	166380.9	165944.6	123580.4	171869.8	114763.1
149437.9	87125.21	181742.9	135763	170552.3	173009.1	181364.2	129434.8	171291.9	113289.4
145442.1	88450.28	203784.5	139489.4	187051.3	149197.5	170603.2	120304.7	151657.8	128314.1

LP429.21_(	LP429.210	LP429.211	LP429.209	LP429.240	LP429.240	LP429.239	LP429.240	LP429.24_	LP429.239
145677.1	234695.1	30182.98	76983.71	363560.4	417917.6	386407.9	346009.9	397367.5	172738
149568	220196.1	34865.01	71724.41	504829	378266	373332.8	337032.2	436104.4	170905.9
147677	205027.3	34962.39	72209.33	615332.9	430235	305724.6	336622.9	299753.2	182495.1
150125.3	189559.1	33610.46	68249.01	535891	375054.3	332970.9	343686.7	382866.2	185059.6
141522.8	201823.4	37958.65	74874.48	454456	440134.7	260531.2	365294.3	432099.5	182521.8

LP429.239	LP429.24_	LP429.24_	LP429.239	LP429.239	LP429.239	LP429.24_	LP429.240	LP429.24_	LP429.239
155651.1	188804.9	212220.7	217507.8	228043.9	177365.2	218296.7	146743.4	283911.7	93234.07
146418.2	233108.6	217418.9	236298.8	257670.8	171501.3	226607.1	176868.8	214015.7	171073.7
136610	244744.2	212843.8	283564.6	243946.7	162328.9	234125.9	173730.8	223301.1	175629.9
146729.1	230404.2	201265.1	206188.4	243628.7	143233.6	219591.6	176106.5	232365.3	166370.3
122497.6	234751.4	201712.6	221298	251416.5	167385.3	219565.7	177250.9	211844.4	179199.9

LP429.240	LP429.240	LP429.240	LP429.240	LP429.239	LP429.24_	LP429.240	LP429.239	LP429.240	LP429.239
228950	174162	196782.8	210216.3	192716.6	251304.5	192331	179299.4	201398.9	169777.2
228452.3	164363.2	198592.4	210027.9	178940	199922.3	216318.7	171252.8	195221.5	165363.7
241944.4	172633.6	198918.9	216094.1	164811.5	194073.1	193091	168276.1	195493.7	177393.7
216921.9	168564.2	200736.3	227226	152223	213473.5	195776.2	140388.9	192885.3	173367
211737.2	178156.3	183194.1	215228.3	154843	211635.4	183968.7	166820.7	201150.5	173296.6

LP429.250	LP429.288	LP429.385	LP430.126	LP430.126	LP430.126	LP430.126	LP430.126	LP430.126	LP430.126
331378.9	15118.09	32039.81	38678.33	31593.16	46553.15	46587.92	32341.6	25636.9	28853.32
298501.7	18961.31	32619.14	40261.8	33590.73	48490.89	42283.87	24423.49	24607.11	33481.99
290952.1	14411.26	36721.76	31812.31	32244.59	45081.73	42304.39	26203.05	23034.78	30667.79
277882.6	13585.26	27577.07	34093.66	26017.68	45532.7	40824.23	30743.03	23042.78	26919.54
283618.9	13131.43	33652.17	39775.8	31018.09	45202.66	41439.03	28645.4	23855.26	29366.02

LP430.126[illegible]	LP430.126[illegible]	LP430.126[illegible]	LP430.126[illegible]	LP430.126[illegible]	LP430.126[illegible]	LP430.126[illegible]	LP430.126[illegible]	LP430.126[illegible]	LP430.1264
36879.17	37838.18	46653.61	35973.73	41859.76	40963.13	37774.34	29624.82	42133.63	58019.57
33334.61	34671.71	38859.13	36003.2	48256.38	39134.76	32418.58	32083.05	37693.99	62054.92
35458.59	38126.22	46272.52	35874.41	45780.38	39932.61	33213.92	27134.25	39068.27	62705.91
37829.45	38400.67	44653.79	40663.25	42190.67	42370.76	30230.2	29605.66	36673.57	59504.32
35779.81	34498.68	43579.23	35286.82	38861.75	40655.06	30993.07	31662.84	38118.95	67423.95

LP430.126	LP430.126	LP430.126	LP430.126	LP430.126	LP430.126	LP430.243	LP430.251	LP430.275	LP430.280
37670.52	65058.95	29332.57	36877.59	38618.94	32113.43	139782.2	77645.44	92057.25	72615
38964.45	70893.14	28968.75	34602.94	38298.61	29413.2	135514.5	70589.29	96588.03	62864.6
38610.59	61751.69	30774.32	30508.81	34931.17	29284.19	142618.1	75437.78	90818.68	71407.52
36824.89	61678.13	31594.03	34624	35244.63	25004.39	152645.5	79414.35	91853.27	74797.65
41372.61	62791.77	28227.74	39737.69	40198.34	30472.08	134644.3	77978.1	86953.5	77091.98

LP430.280	LP430.280	LP430.280	LP430.316	LP430.316	LP430.316	LP430.316	LP430.316	LP430.316	LP430.316
115925.3	63259.89	80037.54	64765.71	76823.01	72928.86	67919.66	76208.52	97542.4	81467.55
118068.8	73848.12	79898.5	59460.88	64644.09	66548.9	109255.6	103585.9	59219.51	79576.5
111664.5	67669.52	77361.13	96991.7	58497.73	78429.69	73995.31	66432.52	63710.8	63656.03
107450.9	70107.59	77892.14	112712	83169.81	81607.93	68474.32	76673.74	69864.06	70875.68
118407.7	71619.32	78338.98	56163.58	70330.69	76246.55	87922.69	70986.98	57482.82	72305.48

LP430.316[illegible]	LP430.316[illegible]	LP430.316[illegible]	LP430.316[illegible]	LP430.316[illegible]	LP430.316[illegible]	LP430.316[illegible]	LP430.316[illegible]	LP430.316[illegible]	LP430.316[illegible]
84172.1	81954.58	62383.23	67413.62	74612.76	64422.08	54339.87	90748.08	96800.89	59500.6
68931.25	54122.75	70294.61	84671.31	67228.33	66479.14	56954.5	97646.94	70794.18	58357.41
66359.62	52197.68	63956.79	80473.26	65535.14	61832.02	57256.31	94634.16	70059.3	63169.54
74615.12	71338.83	62024.43	76289.41	68088.96	61755.18	78690.3	97769.2	61270.27	59713.8
76925.24	53651.47	76218.63	79046.92	68258.95	56481.96	72428.94	92504.8	69802.18	61247.08

LP430.316[illegible]	LP430.316[illegible]	LP430.316[illegible]	LP430.316[illegible]	LP430.316[illegible]	LP430.316[illegible]	LP430.316[illegible]	LP430.316[illegible]	LP430.316[illegible]	LP430.316[illegible]
60651.03	74072.57	64350.27	70634.9	64579.59	56303.16	82859.4	63909.39	62978.33	56444.34
62956.68	60032.59	57863.93	56489.19	64560.01	60151.67	62578.25	66349.61	68605.27	70966.51
62043.6	57680.68	61954.61	60039.74	59091.92	62101.92	60127.67	76058.88	80730.1	75155.29
63845.42	58361.18	74029.81	55495.58	64409.74	60498.33	57851.6	62436.84	75610.01	68266.02
59137.79	56011.92	58252.34	56456.04	62586.21	67559.36	50547.4	66404.68	75503.61	68299.8

LP430.316	LP430.316	LP430.316	LP430.316	LP430.316	LP430.316	LP430.316	LP430.316	LP430.316	LP430.316
57084.45	77167.8	87122.54	58495.78	88196.29	53415.49	57968.03	46236.75	70525.42	75682.99
56534.8	66379.05	65683.36	60925.78	82984.6	57788.05	58711.25	69121.05	59773.48	55761.52
56320.71	69873.69	70221.11	61383.24	75027.5	53580.11	57293.41	63648.62	54752.71	55463.09
56354.03	72946.32	67985.49	66746.43	85499.46	51397.54	55513.4	67841.14	68190.56	57786.33
56298.96	77060.73	58105.58	63217.21	78374.9	54966.85	54898.51	57814.27	59586.15	50457.84

LP430.316	LP430.317	LP430.316	LP430.316	LP430.316	LP430.316	LP430.316	LP430.316	LP430.316	LP430.317
80222.98	51097.84	76420.74	79363.69	67228.52	60272.7	53235.15	49315.16	60959.24	57713.78
76577.98	50877.69	83750.82	45220.34	68894.65	62401.21	53617.53	45228.05	60600.17	56075.57
82601.81	50821.21	70488.58	48064.39	74896.57	60873.8	46108.7	47926.83	58144.28	61252.13
77727.21	83616.12	63903.75	41113.66	72774.06	57624.71	52116.28	53892.26	60045.91	65636.32
80623.11	45336.32	68469.11	41064.54	63902.55	63176.73	56737.17	48512.92	58218.65	59085.07

LP430.316[illegible]	LP430.316[illegible]	LP430.316[illegible]	LP430.316[illegible]	LP430.316[illegible]	LP430.317[illegible]	LP430.316[illegible]	LP430.316[illegible]	LP430.316[illegible]	LP430.316[illegible]
65878.96	53342.31	51535.07	57089.6	63700.6	83000.64	36303.12	51281.29	47503.75	79134.52
55775.63	46915.08	51242.94	51867.92	59682.64	96864.32	39677.02	52601.11	58533.15	73734.46
66322.49	47037.69	49533.15	54353.81	55687.79	90535.84	35877.74	53395.98	56610.51	69736.17
59039.99	51474.15	53152.18	50829.26	60302.9	88249.51	37150.5	53777.76	47483.93	70720.72
54358.67	43271.02	52364.21	52639.56	70685.19	90686.56	35191.23	52637.84	47885.6	69322.08

LP430.316	LP430.316	LP430.316	LP430.317	LP430.316	LP430.316	LP430.316	LP430.316	LP430.316	LP430.317
59445.36	67132.95	59927.68	53591.31	80599.54	68020.09	37284.98	74433.75	44997.86	50847.49
63553.78	60065.96	66108.98	58254.83	46072.95	61858.1	32629.91	63242.93	45711.41	51037.55
61961.22	49557.08	60297.97	64536.82	49014.54	60350.47	38809.48	63217.33	43219.17	50145.93
61235.43	54535.18	54741.33	64468.61	49230.17	59922.87	36919.24	64916.77	39568.07	49834.6
49171.97	56224.17	56128.05	61518.45	47467.37	69725.05	37639.11	61461.41	46448.16	49773.59

LP430.316	LP430.316	LP430.316	LP430.316	LP430.317	LP430.316	LP430.316	LP430.316	LP430.365	LP430.389
71045.09	45367.7	55193.63	39470.18	45002.27	33133.31	58374.83	39234.08	56517.81	425172.9
70851.54	44452.71	56822.72	34822.62	44658.02	35217.1	50900.11	43291.09	55144	564089
69846.33	42198.79	58236.73	42511.11	45492.22	34219.71	55169.03	41606.15	60895.82	331486.7
70593.47	39005.98	58969.55	40251.16	42349.06	30956.91	50877.61	40653.48	58619.63	319706.3
65162.21	42896.62	53111.99	34853.67	39050.65	32287.91	51720.07	40812.23	54722.79	420685.3

LP430.404	LP431.169	LP431.178	LP431.188	LP431.189	LP431.188	LP431.189	LP431.189	LP431.189	LP431.189
31404.92	110200.4	691353.3	136941.5	171377.2	74253.69	174395.8	126250	83725.2	119468
26221.59	130526.3	682717.4	136558.8	170412.7	84516.22	149339.8	127293.5	92819.62	107567.6
27100.95	113651.1	653235.1	127655.8	173830.4	79534.44	168355.1	127932.7	84648.97	113500.1
29219.08	101034.5	589222.3	136340.1	172960.2	74656.18	172411.3	110138.9	72606.15	103544.6
26978.63	128417.6	595171.6	135595.6	171533.9	89792.14	153384.7	121038.1	86580.58	113583.9

LP431.189	LP431.189	LP431.189	LP431.189	LP431.189	LP431.189	LP431.189	LP431.189	LP431.188	LP431.189
119181.1	98303.91	122798.4	195008.4	90727.62	83451.35	86570.27	132054.5	110221.1	127964.6
114094.3	111123.7	107626.4	178260	89781.57	81095.07	90624.7	137021.1	108862.3	117835.8
125953.3	104535.1	107722.8	189680.9	81016.71	84706.25	81718.41	135521.6	114890.3	123464.2
122182.1	105059.2	105870.9	187048.7	79861.02	89626.94	94706.95	134879.4	121987.7	127984.4
118121.4	96027.2	113478	179262.9	70742.71	88335.63	88543.69	127097	121183.4	114451.3

LP431.189	LP431.189	LP431.189	LP431.189	LP431.189	LP431.189	LP431.189	LP431.189	LP431.189	LP431.189
112101.5	111652.7	120059.2	106164.5	58415.48	118365.3	497312.2	76025.78	59292	74035.88
114062.9	117128	112680.6	106994	59602.42	128735.8	473847	77778.61	60330.29	77141.25
107825.3	114748.4	102570.4	106634.6	53939.51	116888.6	448789.9	72287.61	52582.15	76920.63
103543.5	113311.7	119164.9	95292.18	58442.68	115255.7	398949.6	78245.4	49585.2	63983.26
107858.8	114071	109260.6	107792.6	58966.94	119514.8	416994	78877.9	55501.03	75073.55

LP431.189	LP431.189	LP431.189	LP431.189	LP431.189	LP431.189	LP431.189	LP431.188	LP431.189	LP431.189
92650.71	74505.29	95482.03	70154.29	127843.7	86404.97	80463.24	109018.3	112556.8	114764.8
93002.21	84921.04	109924.2	70042.45	113538.2	82900.14	82060.05	102209.9	115288.7	116437.2
86463.44	81799.78	101457.5	67810.26	125885.1	70829.73	76466.79	105915.2	109000.6	118076.6
87775.17	78999.8	97260.43	62136.62	110650.4	78652.79	70920.9	87246.75	117474.4	113621.5
92513.65	75728.01	99635.56	65385.37	113748.6	83608.98	83912.13	105938.5	116501.2	117485.2

LP431.189	LP431.189	LP431.189	LP431.189	LP431.189	LP431.189	LP431.189	LP431.189	LP431.189	LP431.189
97784	147422.1	77804.77	126332.9	122504.5	86770.32	102379.2	93472.61	112666.6	113903
93238.74	147043.2	76393.57	118648.4	123145	102933.1	108276.8	92107.22	108167.3	108857.6
82324.26	151540.1	71937.13	127214.3	120357.7	106252.9	112511.3	93106.18	96434.51	112771.7
88049.24	136678.9	69340.8	124819	119270.8	98106.55	95689.7	83237.67	105136.7	103421.7
94562.56	153719.8	75862.85	128701.4	117602.3	102771	106775	95037	110222.7	110898

LP431.189	LP431.189	LP431.189	LP431.189	LP431.189	LP431.189	LP431.189	LP431.189	LP431.189	LP431.189
125045.4	125809.6	96690.37	78066.87	69440.34	121213.9	101925.3	87693.2	144978.2	75117.37
133506.8	125007.3	104885.4	71566.82	67150.02	122950.7	98198.21	88032.26	167407.1	78894.35
122665.9	124059.1	91370.37	64319.22	70707.78	128528.3	102644.1	82424.66	156715.4	72011.88
130833.5	126668.6	100344	65978.8	71603.58	131457.1	111344.1	79401.92	156194.6	73168.56
115553.4	134881.6	98926.25	67121.28	84720.24	125406.8	110113.6	80807.24	162659.4	72248.04

LP431.189	LP431.188	LP431.189	LP431.189	LP431.189	LP431.189	LP431.189	LP431.189	LP431.188	LP431.188
121732.3	90184.4	75087.09	102408	54108.62	93317.2	130385.5	105059.8	61260.57	42605.86
124652.2	90583.82	76966.95	99546.14	56237.58	88943.69	131462	120053.5	57637.08	50502.77
114852.2	88115.51	83912.01	91343.51	50051.99	93340.7	129578.3	104768.6	58056.11	42813.84
107822.1	82735.66	69348.44	90600.04	46549.6	95650.18	126193.1	112777.8	57258.29	42145.61
122726.2	85457.33	70468.91	96650.31	54948.76	99455.12	139053.9	117732.8	57243.39	38603.35

LP431.188[illegible]	LP431.188[illegible]	LP431.188[illegible]	LP431.188[illegible]	LP431.188[illegible]	LP431.189[illegible]	LP431.188[illegible]	LP431.188[illegible]	LP431.188[illegible]	LP431.188[illegible]
29173.14	36605	28187.46	25977.8	77824.43	75602.97	26311.44	21249.79	27951.69	22085.26
25723.82	32917.99	28893.33	26800.26	72748.18	73700.19	26231.99	18091.21	28459.53	23039.83
23421.96	31619.52	30138.27	25697.25	65626.78	66866.31	20103.78	21483	21974.39	18968.26
23582.93	28765.72	24974.55	23802.08	71073.86	75376.86	24514.95	18694.18	20914.49	17220.68
22753.06	33270.9	25510.86	27230.5	75538.33	71910.11	21372.05	17340.78	24081.54	24466.91

LP431.188	LP431.188	LP431.188	LP431.188	LP431.188	LP431.209	LP431.226	LP431.226	LP431.226	LP431.226
34260.13	51666.42	217902.4	24063.14	36269.75	125277.5	125276.8	146364.3	125372.3	158997.2
36354.19	51298.32	326253.4	22718.42	32639.8	111768.6	152657.3	142690.2	126072.7	178269.7
29310.32	46259.96	236248.7	20318.18	29039.92	169762.2	135188.9	131376.1	123110	169477.6
32623.03	44148.19	308654.1	20721.41	24297.18	214974	138165.5	116497.7	117568.7	166366
31262.88	47306.65	225320.9	24200.77	32496.6	184269.6	146839.4	130284.4	115380.6	150563.3

LP431.226	LP431.278	LP431.384	LP431.443	LP432.172	LP432.212	LP432.238	LP432.238	LP432.238	LP432.238
269778.2	18234.01	149100.6	38073.1	86822.85	104294.2	85136.78	230361.8	92168.07	158009.7
236243.6	19749.44	153562.7	30117.79	95058.05	105491.6	168309.2	179071.1	102822	169661.7
233558.3	16475.35	143612.1	25203.94	84625.46	102194.7	149364.8	149084.3	104813.5	174994.3
232069.1	17708.56	106992.4	22816.86	101281.2	107427.1	159330	146264.6	140992.6	229459.7
246120.6	17727.55	130357.3	27694.71	100687.2	103991.1	143371.1	142083.7	100934.9	117830.8

LP432.238	LP432.238	LP432.238	LP432.238	LP432.238	LP432.238	LP432.238	LP432.238	LP432.238	LP432.238
145364.2	241394.7	224305	167498.4	111182.5	122517.6	230512.8	150947.9	126763.4	178717.8
117431.6	155548.1	197927.1	127122.7	201479.2	112628.2	225637.2	145523.8	165986.4	178756.2
142686	176586.8	200792.1	164485.6	157286.1	141789	213152.2	149762.2	109009.5	210550.3
129614.1	248712.3	206583.9	225688.7	180345.9	136145	234764.5	144185.2	120516.7	260410.9
158921.4	194590.5	241486.4	158462.3	141000.2	253940.9	226884	135680	153533.8	205447.6

LP432.238	LP432.238	LP432.238	LP432.238	LP432.238	LP432.238	LP432.238	LP432.238	LP432.238	LP432.238
211609.3	194415.1	179231	132453.6	110818.5	171243.8	209756.9	124149.4	155026.8	257331.6
133195.6	141916.3	159709.2	124921	90674.04	155278.1	156485.2	162281.8	232051	132492.9
182873	206434.1	169336.2	208314.1	130220.5	210238.7	139077.8	169535.7	170673.4	140303.5
226186.9	137862.3	179515.1	119590.8	120438.3	143213.7	166569.8	168603.8	169460.1	231356
192396.3	183817.8	178803.1	125090.2	105351.3	137846.6	195481.8	164142.2	200811.2	142070.4

LP432.238[illegible]	LP432.238[illegible]	LP432.238[illegible]	LP432.238[illegible]	LP432.238[illegible]	LP432.238[illegible]	LP432.238[illegible]	LP432.238[illegible]	LP432.239[illegible]	LP432.238[illegible]
167262.9	149713.2	108442.4	91949.84	88136.61	215354	161646.7	114705.8	160026.6	177230.4
175655.7	158981.6	123629.5	151021.5	114057.8	211400.7	132452.2	119138.8	160659.6	132746.2
136743.7	111447.7	127581.7	91265.38	112705.4	126550	132697.4	145517.7	159943.2	141188.4
139081	153860.7	98678.77	129047.7	101812.9	213911.3	132200.4	117366.6	159066.5	129765.4
154759.3	168112.6	106513	111823.3	105509.9	139380.1	121196.2	135984.1	180130.1	138806.7

LP432.238	LP432.238	LP432.238	LP432.238	LP432.238	LP432.238	LP432.238	LP432.238	LP432.238	LP432.238
154261.3	131401.9	61052.34	84268.15	167469.8	125184.9	164449.6	164154.6	177499	157501.1
150410.2	130794.9	62691.5	83599.85	113746.6	124630.8	144311	150623.1	133925.4	168351.7
110261.6	152655.6	43388.1	96170.84	116307.4	156900	126367.9	166494.7	187706.9	135892.4
196877.1	142895.5	68640.2	88174.16	111767	150811.9	138067.1	182527.7	137220.8	146715.8
150093.4	141666.5	70819.33	103942.3	117227.7	145724.4	142304.1	151159	131982.5	196931.7

LP432.238[illegible]	LP432.238[illegible]	LP432.238[illegible]	LP432.238[illegible]	LP432.238[illegible]	LP432.238[illegible]	LP432.238[illegible]	LP432.238[illegible]	LP432.238[illegible]	LP432.238[illegible]
114907.9	122092.4	100820	122908.1	113959.8	128209.3	142543.5	85260.27	126849	130227.9
123678.9	133205.4	107316.2	100375.6	122894	137399.1	130574.7	101888.8	122101	92450.64
120498.3	112599.3	85172.39	142970.9	113147	146210	84321.63	85417.06	119423.2	97143.38
126193.3	150392.9	110154.3	111451.7	116756	135070.2	143428.6	105381.6	138128.2	95579.19
137583.7	140151	109232.8	121051.9	106961.7	129700.7	132716.4	107011.2	119932.5	103157.2

LP432.238	LP432.238	LP432.239	LP432.238	LP432.238	LP432.238	LP432.238	LP432.238	LP432.238	LP432.238
122358.1	91085.75	112725.9	77843.58	84076.69	101824.6	83848.43	130157.7	178406.1	36794.9
165585.3	82677.22	106666.5	76044.11	84566.8	97926.62	84516.95	122618.4	133357.6	33523.94
101317.5	94875.15	107665.1	83664.57	72808.9	96822.62	157369.1	127471.9	95335.33	37912.08
85117.65	70361.84	106083.4	88755.34	86156.42	105647.9	122182.2	123619	144684.4	35723.59
98065.41	73975.47	105143.6	79199.11	75337.26	111610.8	85804.97	146112.1	140251.1	31914.25

LP432.238	LP432.238	LP432.238	LP432.2383_1.85
64819.62	138635	36727.4	56262.16
70961.89	82777.46	45119.75	53806.8
76001.48	82854.19	50792.57	51640.16
64974.77	79153.78	36224.78	48538.54
67345.1	82149.24	44647.08	51301.42
