# Supplementary material for: Untargeted Multimodal Metabolomics Investigation of the Haemonchus contortus Exsheathment Secretome
Source: Cells. 2022 Aug 15;11(16):2525. doi: 10.3390/cells11162525 (PMC9406637; doi:10.3390/cells11162525)
Supplement: Supplementary file 1 [file cells-11-02525-s001.zip › Supplementary Table S6 LIPID -ve (LN).pdf]

Treatment	LN203.935	LN204.987	LN204.989	LN204.989	LN204.989	LN204.988	LN204.989	LN204.989
PBS	62525.96	30141.19	27574.61	36083.12	45856.41	46562.09	34825.11	42790.6
PBS	56011.09	34726.44	35698.06	37383.83	36969.86	35071.79	47848.97	45979.27
PBS	43385.87	32156.5	30933.67	41358.33	42052.95	38775.78	48104.13	42172.72
PBS	55059.45	32456.03	37404.16	49902.69	42607.84	41265.17	48877.51	47196.73
PBS	39433.61	28333.3	34655.57	42917.21	42561.22	36965.21	54432.18	46479.37

LN204.989	LN204.989	LN204.989	LN204.989	LN204.988	LN204.989	LN204.989	LN204.989	LN204.989	LN204.988
41471.73	32319.96	44165.85	35765.33	23284.9	32382.88	29914.57	31225.51	27590.71	30050.27
32925.16	35221.32	28172.74	27357.42	22615.58	29502.1	26402.69	26392.96	29854.94	23699.39
32496.47	32836.39	28616.38	25378.09	19473.21	27855.14	28420.8	26483.08	31162.97	23942.28
32006.92	37075.36	29901.8	26303.08	23311.22	30224.14	26849.52	26604.4	28737.14	25093.56
31033.28	35762.38	30820.89	24684.91	22714.39	32015.78	27640.64	24736.76	33726.33	25418.79

LN204.988	LN205.839	LN206.972	LN206.972	LN206.971	LN206.972	LN206.971	LN206.972	LN206.972	LN206.972
49944.19	17327.86	449114.3	116261.3	111791.9	129325.2	189653.9	101437.3	144905.7	86128.94
49568.75	14941.86	319042.8	122073	121952.9	161503.2	143094.8	99616.97	171967.3	129029.8
43969.26	15435.52	357650.6	103455.1	161788.6	186668.7	115784.1	98605.25	135963.3	125481.9
46749.9	17376.06	368491.9	97244.51	98649.8	147213	130164.7	114654.2	160247.4	108463.1
48782.41	19612.02	464683.5	110024.5	136755.4	150363.3	117712.1	87889.38	166474.8	86086.32

LN206.972	LN206.972	LN206.972	LN206.971	LN206.971	LN206.972	LN206.972	LN206.972	LN206.972	LN206.972
128737.7	152985.4	109529.8	73538.08	54308.9	97608	158454.8	174403.6	131744.1	109541.3
167074.8	178445.8	108270.8	75755.02	89065.67	148772.1	104662.3	130661.7	137456.3	121833.5
149944.2	174491.8	120617.3	68845.85	44781.92	154460	168072.4	167598.1	129746.6	144294.1
147280.7	170962.5	90064.31	68407.14	71678.59	133217.1	117293.7	199180	141291.9	174390.5
137392.1	170165.5	108910.7	72277.19	61452.35	134231	119008.8	166487.1	133407.1	145493

LN206.972	LN206.972	LN206.971	LN206.972	LN206.972	LN206.972	LN206.972	LN206.972	LN206.972	LN206.972
149372	98020.65	96927.31	105606.4	76848.77	116743.2	120302	150914.3	100020.5	172298.9
132482.7	125904.4	84211.35	100237.7	86822.87	113410.9	161805.4	111521	69953.75	141171.8
133570.6	109794.4	94325	128210.1	158158.8	122951.3	95318.05	142015.4	119116.2	179492.6
90819.04	88122.5	61597.2	102812.8	103536.9	173459.9	118762	102936.1	81829.66	205543.1
126656.6	124270.7	111364.6	108662.9	89307.14	110963.8	133287.3	107783.6	98034.41	243366.3

LN206.972	LN206.972	LN206.972	LN206.972	LN206.972	LN206.972	LN206.972	LN206.972	LN206.972	LN206.972
113037.1	126862.3	168358.6	150952.6	94200.91	190435.3	126531.4	126591.6	110519.7	131702.1
98481.18	91530.53	186785.7	180152.9	113979.4	188579.2	152346.8	135800.7	120642.3	137122.1
149975.9	110594.4	245304.6	133986.9	84267.91	174986.2	112483.8	173085.8	107711.3	144483.6
96619.48	108864.4	130592.4	122473.6	99676.2	164293.2	109585.8	157718.7	116865.7	117750.8
112396.6	149158.5	142645	123597.3	109027.3	177590	164420.2	128945.3	132468.9	88463.33

LN206.971	LN206.972	LN206.972	LN206.972	LN206.972	LN206.972	LN206.972	LN206.972	LN206.972	LN206.972
77068.51	96065.64	133079.3	136056.4	244297.3	105777.2	129007.9	120335.2	123207.4	148664.4
59804.03	134241.3	102479.6	153103.3	250944.5	134666.4	101794.6	119511.6	97189.82	99721.55
72420.69	108288.1	147255.6	164303.9	187710	118144.7	121034.4	128912.8	141673.3	144140
78773.44	119488.1	115874.1	104194.9	189218.7	182842.3	180887.9	120122.9	147408.1	133872.8
77320.09	135853.3	94622.93	171697.1	221083.1	85655.87	134327.9	133674.8	115103.6	103326.7

LN206.972	LN206.972	LN206.972	LN206.972	LN206.972	LN206.972	LN206.972	LN206.972	LN206.972	LN206.972
171627.7	133529.7	177640.6	188276.8	106451.6	86413.91	119924.6	127725.3	115103.3	95149
151144	166981.1	139304.7	119465.6	121800.4	115129.6	90641.95	122163.2	139361.1	102086.5
120034.6	118869.4	117208.7	157200.6	154556.9	103659.1	130056	134197	210879.3	105869.9
98927.94	94922.36	81861.86	205327.1	132489.9	154671.2	107928.9	123282.5	117754.3	115474.5
108386.3	121314.8	129959.3	126518.4	106165.9	111491.7	110545.5	121918.4	108242.3	106600.6

LN206.971	LN206.972	LN206.972	LN206.972	LN206.972	LN206.972	LN206.972	LN206.972	LN206.971	LN206.972
66916.01	107459.2	168626.5	103444.9	149679	91527.68	142875.5	143790.2	43114.05	108797
48302.87	201477.8	115374.7	94930.22	198190.8	102838.3	198392	146838.4	45660.1	108313.5
61475.35	154781.8	114609.1	104547.8	130053.3	117909.6	152161.2	162888.8	51265.56	184758.2
69629.73	138821.1	166654.1	83083.08	125404.7	138711.9	133239.2	157522.8	45990.81	107303
61789.23	169427.9	122516.8	104288.3	135906.1	96095.79	175492.5	201069.4	60771.49	135033.5

LN206.972	LN206.972	LN206.972	LN206.972	LN206.972	LN206.972	LN206.972	LN206.972	LN206.972	LN206.972
131696.8	188698	237482	110194.1	87591.05	79126.88	158536.8	134425.9	133343.1	157629.8
155334.4	224918.8	169744.7	156386.6	109967.2	131400.9	99217.28	182502.2	140704.1	106759.1
112068.7	140096.3	177369.7	149589.1	119075.4	153941.4	113983.9	195723.3	170570.1	100116.8
133504	110142.6	123357	149796.7	110164.7	101739.3	109607.7	168683.6	186937.4	111046.3
137152.1	204792.8	144864	144998.6	112359.9	108217.8	116545.3	153073.5	98382.96	117779.4

LN206.972	LN206.972	LN206.971	LN206.972	LN206.972	LN206.972	LN206.972	LN206.972	LN206.972	LN207.836
74451.59	143191.9	51997.89	124683.5	97143.53	97138.82	153324.7	214125.1	158018.4	23801.85
98640.49	139733	71544.53	100107.7	150759.4	123689.8	151642.9	182621.5	224152.5	20344.24
128773.6	172610.4	79051.39	146217.8	135703.6	90901.36	155805.7	205963	204899.4	21100.32
139307	141058.6	54683	133848.8	149660.7	104283.4	134430.3	225642	160257	21045.77
113629.4	105311.7	56147.67	157938.6	142812.6	78809.52	186939.8	150803.6	169323.1	28307.92

LN207.917	LN207.930	LN207.930	LN207.930	LN207.930	LN207.975	LN209.926	LN210.883	LN210.948	LN212.881
147220.3	25663.62	20527.72	26516.52	21061.12	13255.27	30755.71	31990.78	25576.9	22261.64
124310.6	23836.62	20543.94	23076.01	22473.68	11432.3	31523.73	31151.93	24646.37	17699.71
150016.8	26331.12	19399.32	23406.13	22291.2	13250.58	31995.3	32674.78	22634.59	19772.69
148135.2	25782.75	18158.41	23444.53	20947.85	13269.13	36676.62	30842.49	23573.32	18586.99
166776.5	27582.29	18233.72	26131.01	19924.85	13209.07	38475.39	35454.81	24091.94	20797.3

LN212.910	LN212.978	LN213.014	LN213.055	LN214.879	LN215.007	LN215.007	LN215.007	LN215.009	LN215.009
18996.53	12392.76	23876.94	15505.31	16331.8	305823.6	243643.2	235612.8	302378.9	177521.9
19651.29	13387.27	23354.38	13052.56	14126.1	286013	239929.4	229868.3	209404.3	181104.7
19291.76	15817.88	25120.56	11105.68	15440.97	298022	250691.6	227973.5	191132.8	166181.2
21979.57	14397.77	23369.9	14799.42	16107.8	304896.3	245690.4	220098.3	182987.3	231134.6
23083.98	12414.32	24044.32	12371.81	19798.24	295148	253831.8	226588.9	233432.2	164556.3

LN215.009	LN215.009	LN215.009	LN215.009	LN215.009	LN215.009	LN215.009	LN215.009	LN215.009	LN215.009
176111.5	137033.4	231372.4	168125.5	173745.5	186303.5	149264.3	134730.2	272912.8	175459.7
261316.9	115634.1	273887.5	271029.5	234109.8	219595.5	209876.6	164132.3	293117.1	176276.7
249450.1	139443.5	170999	140230.8	200921.6	381939	135719.2	191662.9	219500.6	169919.8
192850.3	118524	179848	176130.6	158987.2	295829.7	140593.9	132758.9	192340.6	203177.5
165568.4	116330.8	197938.7	209820.6	164817	185013.8	184920.9	266331.4	258524.9	211730.5

LN215.009	LN215.009	LN215.009	LN215.009	LN215.009	LN215.009	LN215.009	LN215.009	LN215.009	LN215.009
254466	213256.4	173146.8	181746.4	168998.6	199777.9	210388.6	189779.4	257217	248110
245354.5	205046.8	205186.6	274234.6	183026.7	209361.8	230169.5	176264.8	216002	235633.1
169016.8	229650.2	234048	256730.9	185068.7	210826.6	305551	191588.1	225961	331545.1
297590.8	196691.4	166360.6	187888.2	166554.4	190674.6	238115.6	212915.8	234970.3	225357.8
247066	218517.4	200958.6	263907.6	197186.4	217013.5	165014.6	185174.7	195684.4	272138.8

LN215.009	LN215.009	LN215.009	LN215.009	LN215.009	LN215.009	LN215.009	LN215.009	LN215.009	LN215.009
201395.5	276899.1	241191.6	133091.8	176278.7	144698.4	165564	206698.2	230663.5	145905.9
217956.5	353423.6	193193.1	187973.6	146556.6	146671.3	188989.2	257047.9	234177.4	161546.8
203494	177923.1	212621.5	126522.9	148228.1	206388.1	118086.1	310164.1	227007.8	205160
210391.9	206868.1	338959.8	197362.8	183685.9	205628.7	136985.9	244867.4	269362.6	129310.9
212504.2	170397.8	233895.8	163257.1	154014.4	190695.7	171804.4	277639.7	201447.3	181193.3

LN215.009	LN215.009	LN215.009	LN215.009	LN215.009	LN215.009	LN215.009	LN215.009	LN215.009	LN215.009
197250.5	156816.4	172697	179535.7	179154.6	294865.2	138907.4	209860.8	163925.1	233893.5
339682.3	153964.9	182028.7	227764.5	175545.3	244215.6	113548.7	164568.5	191464.7	200297.2
282456.8	146908.5	163316.6	210237.9	265038.5	315952.9	119426.4	196258.4	148119.3	123175.1
231355.7	167438	154184.1	256053.1	180191.9	247915.3	116394.9	139759.4	154544.4	139534.1
322582.3	181942	168767.1	239033.5	253594.4	229262.5	147032.1	158135.1	167779.1	140735.7

LN215.009	LN215.009	LN215.009	LN215.009	LN215.009	LN215.009	LN215.009	LN215.009	LN215.009	LN215.009
115417.4	136771.8	151160.3	204388.4	182880	134557.5	242458.1	143934.7	219673.5	227191.6
186109.1	143111.3	174865.9	142552.3	324064.1	140425.6	251569.6	141532.2	262104.5	140904.8
159693.7	119688	203394.1	174472.4	273966.1	137855.8	225995.2	110146.2	227389.1	176856.2
104085.8	135702.9	129020.3	210281.6	239790.6	128483.5	232345.3	138246.5	158987.3	153231.1
134534.8	154212.3	132675.8	207302.6	238201.8	149191.3	202536.6	131039.2	375509.7	215674.9

LN215.009	LN215.009	LN215.009	LN215.009	LN215.009	LN215.009	LN215.009	LN215.009	LN215.009	LN215.009
186347	135315.4	262373.8	189424	161772.1	283235.8	302225.6	235566.3	110381	208641.3
136047.5	140798.5	306185.8	228402.7	170659.2	204230.2	196301.4	273744.4	138957.5	224271.8
127979.4	171901.2	203728.4	176183	180746.1	282677.1	227852.8	169567.9	133531.2	245396.7
131274	161881.4	249645.4	249358.6	206096.2	169441	254552.3	182634.3	97245.08	188249.9
111003.1	116301.3	225262.5	139957.6	134341.3	162311.6	278980	208374.2	129272	221672.2

LN215.009	LN215.009	LN215.009	LN215.009	LN215.009	LN215.009	LN215.009	LN215.009	LN215.009	LN215.009
213036.9	303690.6	135370.7	199399.9	179268.6	198113.7	166314.8	144921.9	294835.2	201994.9
229463.7	243727.2	206642.1	217589.4	172781	156704	209013.1	137474.3	292208.2	203323.4
201847.6	231303.6	179651.4	226007.5	158446.8	153102.9	185395.6	165625.3	227079.7	239768.3
239711.1	202854.8	194098.8	214052.5	216651.7	206189.2	184695.6	180947.8	185611.8	165039.3
168556.9	222801.8	123614.5	202231.9	270579.2	153265.9	172449.6	158645.4	194467.6	248252.7

LN215.009	LN215.009	LN215.009	LN215.009	LN215.009	LN215.009	LN215.009	LN215.009	LN215.009	LN215.009
235135.1	382010.4	221434.6	182335.5	185346.5	207835.6	221135.4	203239.6	160188.4	158845.5
220214.3	174803	156578.3	182351.1	214324.8	211549.3	174608.7	248794.6	257158.7	135628.9
158669.4	258452.6	214137.9	159301	147678.1	202021.9	225894.1	253813.6	195007.1	127070.8
192369.7	233958	143080.5	170276.4	164495.4	162113.1	231154.4	245178.4	185350.6	127650.1
214644.6	205188.7	165687.2	186335.8	204654.6	219146.1	188926	198188	199555.1	115416.6

LN215.009	LN215.867	LN215.992	LN215.992	LN215.992	LN215.992	LN215.992	LN215.993	LN215.993	LN215.993
239770	137056.2	47875.82	68538.45	61893.04	49209.4	50305.67	55178.09	70769.19	89291.19
152610.4	122127.6	49746.43	79003.25	60851.31	50676.96	38096.18	70628.56	36890.67	64113.67
163784.6	126484	50662.27	70941.85	54982.8	45994.68	33839.96	54746.77	50774.24	76996.82
237975.5	111075.6	46077.19	72429.41	55235.76	47933.97	36753.5	85575.62	59417.11	98291.32
220061.6	160796.3	48805.16	68574.89	53874.33	49359.68	36146.99	72746.19	40937.71	89086.99

LN215.993	LN215.993	LN215.993	LN215.993	LN215.993	LN215.993	LN215.993	LN215.992	LN215.993	LN215.993
87079.5	47922.8	60475.05	63678.9	40882.29	54483.16	68103.04	70094.94	61092.93	70231.34
94621.53	58085.06	54621.71	53541.52	53860.13	78327.72	54994.74	75375.18	68678.85	62440.71
74311.25	66848.58	109232.1	66284.63	38761.61	77598.28	70889.3	59827.24	85881.51	71911.15
65479.54	55357.02	93987.82	65835.03	42900.53	90006.71	67068.9	86889.13	55165.66	72127.14
96894.02	90076.45	87646.09	54170.18	42246.89	41476.14	55035.39	106216.9	54489.06	71720.36

LN215.993	LN215.992	LN215.993	LN215.993	LN215.993	LN215.993	LN215.993	LN215.993	LN215.993	LN215.993
76812.65	76097.45	57855.64	91587.12	65336.13	69212.43	49273.08	51545.47	43335.78	94570.33
64265.62	79930.52	52145.17	74338.47	74825.45	49197.73	72000.77	52196.17	58479.33	70094.34
77156.15	54533.82	43071.55	80334.72	69158.81	71953.27	70325.63	66380.24	59950.75	63177.99
61716.87	80477.63	90079.64	49330.65	63432.42	93508.97	87761.79	39413.09	63882.54	107310.8
78378.42	87416.07	68298.81	70307.48	64041.37	79284.88	56122.22	43631.9	40728.35	62806.46

LN215.993	LN215.993	LN215.993	LN215.993	LN215.993	LN215.993	LN215.993	LN215.993	LN215.993	LN215.993
101464.7	45880.73	58243.93	103841.2	38473.49	55295.39	97703.48	72745.06	53246.28	72968.04
77596.8	61093.91	77208.78	59416.62	39981.25	64672.22	55682.3	85616.46	51800.5	80027.64
91345.02	50957.94	60109.2	88066.19	53160.98	62150.68	63187.17	75512.48	55799.84	59303.33
65644.45	60608.09	40157.97	77909.3	43944.3	61539.98	52466.32	71634.88	54177.47	71632.61
107577.6	78832.75	74198.45	75444.88	39037.03	56583	68410.22	82015.8	64759.41	69540.96

LN215.993	LN215.993	LN215.993	LN215.993	LN215.993	LN215.993	LN215.993	LN215.993	LN215.993	LN215.993
53207.15	51842.48	55634.63	38483.05	67424.62	73455.99	60171.11	103365.9	86107.96	54780.1
71185.51	58028.07	46423.98	45295.67	59126.46	72177.56	53682.85	50094.34	85966.29	56932.43
62788.52	66185.41	38652.93	47455.73	82199.3	69866.21	43508.5	50748.44	53906.01	49624.62
54120.33	78824.38	46705.17	48355.66	54110.78	75652	55970.64	59377.65	44072.66	45292.64
53699.79	52857.04	46299.62	44337.71	100018	73755.59	51786.54	46670.61	78353.97	48470.29

LN215.993	LN215.993	LN215.993	LN215.993	LN215.993	LN215.993	LN215.993	LN215.993	LN215.993	LN215.993
48267.86	59251.58	46178.6	48106.33	58362.47	66645.48	44401.68	63937.84	40174.74	87876.19
69548.31	44171.93	45953.33	41942.78	64573.57	99879.9	54076.99	54163.54	77853.35	73591.71
46409.96	50174.78	41584.7	52870.5	49597.31	63021.49	52768.29	101561.7	81514.08	86352.96
61216.72	63852.7	52997.71	47050.68	74469.22	87783.25	50347.75	61093.91	51753	70614.11
54693.07	41947.85	46179.36	58097.23	53525.63	76119.35	44909.83	59207.5	66447.93	67865.24

LN215.993	LN215.993	LN215.993	LN215.993	LN215.993	LN215.993	LN215.993	LN215.993	LN215.993	LN215.993
84401.8	39900.7	60187.6	42663.26	45576.67	70962.64	42332.98	45286.35	77352.97	58727.29
72569.97	48500.29	48339.28	45622.3	40605.41	66725.29	61955.35	40626.95	60754.3	66392.48
89388.42	54220.19	58106.5	32912.49	65854.67	66085.76	31955.77	46040.27	55660.61	54072.89
91581.64	29498.54	69317.08	55211.78	81279.51	51541.77	57138.22	50965.3	66346.21	55514.26
80339.55	57183.41	58135.3	38890.85	53768.65	67713.03	47201.82	45729.46	56781.64	93151.83

LN215.993	LN215.993	LN215.993	LN215.993	LN215.993	LN215.993	LN215.993	LN215.993	LN215.993	LN215.993
59923.3	57692.56	41189.42	85612.2	68467.27	62450.99	79618.26	56198.37	59359.97	60486.45
83730.71	69388.31	50160.33	76783.43	93768.94	62105.47	76412.44	57397.18	49486.83	66923.86
53196.52	43198.25	47563.11	69898.6	65862.12	81597.88	80766.86	69269.32	40192.35	75459.66
53513.42	71179.08	60761.52	97941.11	89799.37	55555.61	66984.18	48417.26	37624.21	49849.79
53159.54	49045.33	39307.01	92526.06	73065.92	64190.58	67806.73	56528.81	62232.22	63481.98

LN215.993	LN215.993	LN215.993	LN215.993	LN215.993	LN215.993	LN215.993	LN215.993	LN216.012	LN216.012
42825.63	54638.97	116502.8	38829.98	89523.8	77022.09	40572.05	65380.64	62732.3	58777.45
48697.79	81083.04	76024.84	39089.28	87111.86	54516.71	45552.97	53868.26	61797.07	53843.21
47867.18	78337.86	61769.78	64365.65	93795.53	50449.13	32557.08	95813.07	64411.3	53186.62
41776.72	84349.68	81662.91	36211.06	60162.55	59288.52	40460.9	60361.67	63095.95	51996.24
59444.99	78113.24	61024.87	55389.4	54606.59	54099.01	46683.14	78006.61	59385.57	52910.12

LN216.012	LN216.012	LN216.012	LN216.012	LN216.012	LN216.012	LN216.012	LN216.012	LN216.012	LN216.012
57675.79	64724.59	44037.41	44984.05	20237.08	50928.56	64744.32	28506	40171.7	14707.59
60950.93	71747.24	48226.17	47636.97	58943.03	49043.46	61787.29	32241.25	42596.82	45333.51
56421.31	65916.67	41136.97	48739.2	56927.48	52944.2	61050.72	30694.55	43718.07	40489.16
54383.73	73387.58	43985.56	50350.65	53256.85	46457.79	65370.49	29148.46	33205.95	42331.58
59638.42	71131.13	40091.3	48070.64	58757.97	47731.49	62668.6	31977.77	38154.8	41528.74

LN216.010	LN216.012	LN216.012	LN216.012	LN216.012	LN216.012	LN216.012	LN216.012	LN216.011	LN216.012
66851.23	56732.7	36899	43283.44	68301.2	35401.32	15441.43	15302.74	61608.08	49075.64
71250.61	56365.04	35211.29	47136.21	68387.76	33247.22	44054.88	45838.82	54881.38	48955.07
71189.77	60959.54	34317.1	52323.51	74285.58	31183.84	39771.23	40471.12	53509.02	48310.03
66246.64	55172.6	31654.02	51365.21	68680.18	32887.08	43509.99	41125.25	53098.98	49579.73
65377.23	58683.73	32843.69	50971.85	68149.89	34381.08	41391.81	41502.45	52686.44	45499.63

LN216.013	LN216.013	LN216.013	LN216.853	LN217.863	LN218.987	LN218.987	LN218.987	LN218.987	LN218.987
67249.08	57279.08	44273.96	45738.84	100890.6	28090.84	20630.92	24815.39	31436.42	22868.46
72279.07	61117.95	45028.06	40444.89	80502.9	27483.72	18482.19	25628.19	31087.1	23132.13
70200.4	61961.75	45635.35	41374.29	83692.84	29628.95	22295.5	27527.17	32643.49	22479.04
62115.85	61617.36	45175.77	44029.58	97970.4	24063.51	20808.63	26674.56	34079.92	21835.28
67798.18	59779.86	48943.66	61029.73	101629.9	29502.5	20745	23774.68	35413.51	22718.63

LN218.987	LN218.987	LN218.987	LN218.987	LN218.987	LN218.987	LN218.987	LN218.987	LN218.987	LN218.987
19661.5	19707.75	19816.41	24149.8	25082.8	23613.21	18977.51	38663.82	18770.88	45227.26
20149.86	18607.94	22402.51	25379.6	23166.49	25019.23	22643.63	36649.64	16294.94	44083.29
21660.46	21083.69	18432.71	22106.54	26182.34	25274.39	19743.26	38220.86	17070.09	40932.66
22611.02	21968.73	19671.96	22885.86	26831.76	24446.24	21018.79	39124.9	16873.29	46505.74
22903.72	22448.74	20269.82	21637.93	25579.94	25516.81	22226.69	40858.66	19493.6	41521.03

LN218.988	LN218.988	LN218.988	LN218.988	LN218.988	LN218.987	LN218.988	LN218.988	LN218.988	LN218.988
53106.42	63844.38	43917.55	60187.7	45244.06	43932.91	47638.42	81669.88	44129.38	56060.06
76599.93	66095.68	44253.18	53400.14	48178.89	43869.33	41418.15	71709.76	32796.5	54712.67
41946.5	49980.66	48594.53	38968.99	48823.06	41770.42	31625.8	61346.1	40867.49	45981.98
51012.84	54214.38	44919.31	62273.85	44764.94	33970.2	33641.11	66847.18	47314.45	57455.4
81254.31	58976.6	40398.7	72166.22	40016.2	38010.38	36278.5	39237.7	42130.52	64552.53

LN218.988	LN218.988	LN218.988	LN218.988	LN218.988	LN218.988	LN218.988	LN218.988	LN218.988	LN218.988
36164.41	59406.16	54565.39	44126.86	45780.06	67300.3	36301.56	16721.18	30849.63	24808.7
30125.79	36207.62	66224.37	41515.6	42581.81	61452.84	57544.8	16623.35	26522.71	20785.05
25143.98	41558.28	52254.32	37386.6	53160.13	54267.04	51015.12	20584.12	32287.67	24135.79
23409.35	42912.12	46796.51	39156.36	58202.01	50731.61	49822.66	20961.14	29331.21	23444.64
22599.99	41668.64	51498.66	39046.28	56037.26	47980.3	49990.37	23853.62	30245.55	26222.87

LN218.988	LN218.988	LN218.988	LN218.988	LN218.988	LN218.988	LN218.988	LN218.988	LN218.988	LN218.988
49227.8	51270.45	22836.07	27858.88	23162.96	44330.32	32587.21	38588.87	17449.79	38155.28
33235.07	43114.14	28126.37	19854.47	39173.99	45024.35	25887.34	33366.09	28497.41	32805.21
37325.42	32253.7	20552.14	21923.69	33314.53	46097.73	25705.41	30648.83	20382.86	37526.36
37352.16	30627.61	20442.2	22285.58	31390.1	38496.38	24787.11	27737.09	21891.71	33676.84
42073.96	31788.55	19768.19	23609.96	27661.22	41883.43	23633.04	31148.57	19485.92	36057.88

LN218.988	LN218.988	LN218.988	LN218.988	LN218.988	LN218.988	LN218.988	LN218.988	LN218.988	LN218.988
19917.22	15490.49	27035.21	27307.06	12670.36	27508.37	36396.22	27486.55	36094.65	46979.38
29435.61	19584.56	20793.97	37710.23	15480.19	38635.53	41631.78	25064.67	31512.95	43409.75
24929.14	17611.98	24709.87	30207.21	21782.3	30080.1	28964.18	27697.16	36194.36	33436.26
24248.03	19703	25897.86	38690.29	23078.7	31130.46	26596.02	25165.03	34659.21	35355.87
23594.64	18329.09	26667.71	35685.24	20858.65	30680	27619.65	27213.56	33964.15	36967.62

LN218.988	LN218.988	LN218.988	LN218.988	LN218.988	LN218.988	LN218.988	LN218.988	LN218.988	LN218.988
20275.96	30969.56	32657.42	45917.97	33573.77	20768.16	56500.31	44541.14	25937.04	30991.95
24082.57	38473.15	45467.51	64401.14	41013.17	19871.25	44058.83	48737	33655.97	40946.36
18413.44	44582.4	28067.16	48424.3	37906.34	23051.61	42452.5	46824.06	27585.65	36801.53
18402.18	33581.34	27068.73	48605.03	44920.73	21649.31	36398.82	43927.3	28934.07	31402.88
19867.85	37910.22	32058.11	45715.6	40184.34	22901.65	42359.46	40222.2	29990.68	35706.16

LN218.988	LN218.988	LN218.988	LN218.988	LN218.988	LN218.988	LN218.988	LN218.988	LN218.988	LN218.988
40520.34	33185.37	40801.39	13195.02	48570.99	24615.75	41230.9	23761.68	24487.54	14247.61
38144.53	24350.38	50514.13	20951.2	40875.82	23230.38	27419.78	19169.17	22720.91	21105.98
30960.21	28013.69	46495.79	19601.18	39470.18	29869.3	32160.84	28699.79	26689.71	16991.4
34973.04	27723.7	44716.35	20102.95	39881.18	28506.26	35111.15	25949.96	23216.91	15559.81
30870.83	28097	40229.72	18865.23	37414.72	28889.54	34901.05	27600.76	25268.33	18279.14

LN218.988	LN218.988	LN218.988	LN218.988	LN218.988	LN218.988	LN218.988	LN218.988	LN218.988	LN218.988
29057.63	42957.44	13319.68	36751.68	12040.69	30178.39	29757.29	30235.96	48550.86	42943.07
20066.61	37396.21	12316.02	41291.69	13598.02	34146.11	24136.2	34409.31	33958.22	44457.46
22420.17	35910.25	16506.7	36690.19	17933.99	24286.37	23461.95	38232.4	37994.91	59286.35
21135.28	36683.28	17594.58	32984.44	19094.81	24794.23	23355.25	33170.99	36391.13	49191.8
21171.34	35998.37	15622.13	35128.55	17153.15	29435.71	26712	32199.82	32280.43	52339.34

LN218.988	LN218.988	LN218.988	LN218.988	LN218.988	LN218.988	LN218.988	LN218.988	LN218.988	LN218.988
25064.4	35034.07	28208.77	38903.59	24267.9	48954.68	16392.6	32812.58	33303.1	28319.48
18596.3	54615.63	30667.27	23226.38	22027.28	37355.66	23386.27	28736.34	37128.79	37448.14
27936.37	34023.84	20727.73	28716.07	18199.42	40788.13	20701.14	30241.45	38380.15	30721.09
27110.55	31405.21	22192.27	27552.58	18445.5	42831.62	19909.63	29427.02	32532.9	28731.13
27632.36	31489.46	23289.16	27717.86	16954.91	44286.29	20193.85	24718.48	36540.02	29117.41

LN218.988	LN218.988	LN218.988	LN219.845	LN219.845	LN219.845	LN220.965	LN220.965	LN220.965	LN220.965
17315.64	30853.42	23571.38	27912.75	12367.63	12003.26	25715.36	34950.01	33172.31	26223.77
17610.19	20414.01	31499.22	25883.73	12122.41	12532.7	23236.06	33802.72	34846.48	22729.43
22239.53	23849.07	25323.26	25433.32	10892.9	10507.59	25917.36	35963.12	34761.23	22468.16
23175.25	23386.77	22366.5	26317.77	13304.87	12506.4	23060.21	28620.8	31840.88	23760.85
24285.31	23848.58	25453.4	31794.91	13575.5	13121.29	22964.64	30314.6	29216.48	23791.33

LN220.965	LN220.965	LN220.964	LN220.965	LN220.965	LN220.965	LN220.965	LN220.965	LN220.965	LN220.965
30787.01	43204.89	34334.59	26917.06	28665.72	33007.14	18944.36	25340.32	26430.05	25432.02
29784.93	40889.95	33617.21	22666.42	29279.77	34304.75	18103.25	24501.03	25666.42	22562.25
29618.04	36876.47	31467.56	22574.01	29908.23	32860.55	18518.74	27613.34	27447.44	22734.72
28759.1	35037.2	30808.89	22923.53	30357.11	32019.8	18971.81	24547.21	30017	22863.76
28956.77	36177.82	30377.33	23156.44	29252.3	32384.17	17301.19	25130.81	28367.56	24239.89

LN220.965	LN220.965	LN220.965	LN220.965	LN220.965	LN220.965	LN220.965	LN220.965	LN220.965	LN220.965
21307.12	38836.32	26873.05	24311.99	19669.35	26800.59	24326.67	28773.1	19834.08	20682.95
22639.73	34667.58	26420.36	22041.48	23246.36	25030.41	25320.87	29820.29	18945.28	22822.57
21462.94	36309.76	24894.88	22436.67	19870.32	23432.92	21352.29	29311.14	16908.37	22705.49
23077.21	33053.26	24016.96	23165.5	22675.79	26058.44	21707.29	30040.98	16267.82	22677.31
21870.63	32962.22	21474.87	21588.49	23961.45	25237.37	22894.37	27798.51	15772.81	23357.9

LN220.965	LN220.965	LN220.965	LN220.965	LN220.965	LN220.965	LN220.965	LN220.965	LN220.965	LN220.964
21597.24	17271.28	25728.18	24813.08	20975.47	35510.15	26106.63	20599.31	21028.79	25709.5
24102.19	20687.01	27290.75	24866.15	23133.04	33362.11	24763.34	22186.61	20089.13	25730.73
22005.33	18454.72	24678.19	23232.39	27012.86	33105.68	23809.96	20278.93	18960.48	27636.28
23072.88	18121.43	24898.56	25304.62	24527.41	36134.4	23663.84	20728.32	20187.94	26411.41
22010.71	19063.59	24188.46	23214.58	24215.01	34587.51	24497.24	20374.47	18488.73	25043.6

LN220.965	LN220.965	LN220.965	LN220.964	LN220.965	LN220.965	LN220.965	LN220.965	LN220.965	LN220.965
27313.31	30508.96	30697.72	25405.69	23178.32	22991.3	20715.96	31341.89	23158.68	18113.66
30529.88	29945.78	33118.79	22929.54	21281.46	22210.77	20537.52	34731.55	26098.07	17052.16
27005.78	28667.11	26066.83	21575.68	18966.8	21904.24	22612.19	29654.57	26022.85	18132.38
31620.03	27652.3	27448.58	21959.04	19538.72	22474.67	20426.85	31693.05	25824.96	15587.79
27045.45	30446.1	28221.83	18970.75	19240.42	20297.71	20862.49	33260.13	25357.74	15411.67

LN220.982	LN220.983	LN220.983	LN220.983	LN220.983	LN220.983	LN220.983	LN220.983	LN220.984	LN221.842
27510.52	34821.12	34689.81	31778.35	40476.11	41928.19	26237.57	34209.15	38938.6	22506.68
28039.79	30455.59	31158.6	29240.66	41322.27	42394.7	22963.12	36206.49	40778.68	24130.51
28069.93	30810.38	32065.5	30065.4	37610.96	39455.57	25111.25	34921.58	39700.37	21758.94
25876.61	33942.03	35022.56	33140.95	38595.95	42759.46	24801.7	35815.07	39001.34	23915.98
28563.39	36678.82	34932.41	29327.23	36833.16	40960.3	26762.86	34239.75	40680.28	25511.49

LN221.842	LN223.027	LN223.027	LN223.028	LN223.028	LN223.028	LN223.028	LN223.028	LN223.028	LN223.028
11006.01	37225.11	31249.78	19669.74	19433.05	17978.01	11403.05	19193.78	23559.23	17239.23
10396.46	34959.39	28477.79	20767.05	20706.23	20623.77	13165.64	18978.26	26261.98	16663.76
10530.95	38072.62	31649.82	25345.19	24391.12	23160.89	14208.2	18404.56	21300.92	16691.36
12565.1	34159.62	31469.6	26233.19	28690.33	25775.22	12229.76	16936.01	22961.04	14575.92
13334.77	38399.21	30320.25	28610.68	29553.43	27603.28	14990.87	17553.83	23989.64	15683.73

LN223.028	LN223.028	LN223.028	LN223.028	LN223.028	LN223.028	LN223.028	LN224.908	LN224.996	LN224.996
24091.64	20440.32	16206.47	21620.1	24767.2	28885.96	22038.64	63672.77	55534.72	66207.93
24467.31	19516.53	14694.59	24701.08	21636.09	30287.66	20759.41	54731.97	34919.24	40347.47
24601.9	20755.45	15395.71	22301.45	23613.32	28652.81	18708.06	53422.84	49492.24	67720.4
24040.5	20019.81	17605.87	26056.12	23217.97	33389.72	19733.23	65173.5	42519.91	43613.62
24500.26	20946.36	17486.63	25443.6	25659.06	33917.77	19817.16	65487.3	43703.49	43987.44

LN224.996	LN224.996	LN224.997	LN224.996	LN224.997	LN224.996	LN224.996	LN224.996	LN224.996	LN224.997
37798.53	25500.92	25601.48	30700.83	27247.15	52450.15	39617.45	51001.14	36303.34	33157.37
37865.76	22958.85	27306.48	32843.79	26631.26	47004.54	32683.61	38513.98	33581.46	36883.08
41425.53	25022.63	27995.16	31734.66	30456.33	50735.1	50798.89	39670.38	36166.92	38202.24
38609.07	25511.28	30930.76	38677.02	30720.3	33977.29	33554.23	43644.92	37454.08	36794.19
39782.44	26658.01	29342.24	32688.96	27045.85	35241.09	35729.76	41939.57	38245.79	36441.9

LN224.999	LN224.999	LN224.999	LN224.999	LN224.998	LN224.999	LN224.999	LN224.999	LN224.999	LN224.999
31078.21	37914.47	35289.4	26458.73	42821.81	44325.67	31868.31	42042.39	52433.56	40341.91
32612.17	37963.43	34208.08	30733.96	40987.3	46090.94	33063.36	43550.32	55095.12	39467.84
33551.65	40882.38	37378.97	30784.58	46571	47575.85	36780.8	41891.03	58020.75	45023.28
37202.43	39493.83	37628.91	29369.83	44936.12	46094.46	37280.11	43353.54	57567.71	48007.21
36619.54	40730.22	41267.44	27518.73	44580.29	50798.12	32267.25	44110.44	56843.42	49426.76

LN224.999	LN224.999	LN225.024	LN225.024	LN225.025	LN225.037	LN225.077	LN225.077	LN225.077	LN225.077
47398.41	31296.15	27662.76	24010.78	20317.01	32120.69	171083.4	108416.6	121167.1	119180.4
45559.54	32196.1	28703.11	25514.12	23465.69	33021.53	147635	139484.7	121412.6	127157.9
48792.02	35699.81	28393.1	24685.35	20346.88	28344.94	177432.1	108543.4	109497.6	123098
47426.33	36383.93	26549.56	25415.32	19454.75	31067.95	175090.5	110600.4	118807.8	117655.6
52129.8	35546.81	28758.53	23427.14	21333.61	30845.32	172652	105119	113205.5	119509.9

LN225.077	LN225.079	LN225.078	LN225.078	LN225.079	LN225.078	LN225.078	LN225.077	LN225.078	LN225.077
96335.03	122801.8	91235.32	169722.8	173006.1	116164.6	178088.1	95082.51	129621.8	184328.6
92442.76	111910	77619.63	168343.7	176293.8	117162.3	186174.8	92242.83	124224.5	190581.9
99170.63	102322	119253.9	181459.7	177978.1	123760.4	171790.2	102624.5	129633.1	188170.8
100875.1	109547.6	92237.86	189606.1	187942.1	123723.1	188600.6	101768.1	131302.2	190777.6
96616.53	112116	93252.7	204922.3	180072.3	123443.3	190245	101913	145570.8	182555.1

LN225.078	LN225.078	LN225.078	LN225.078	LN225.078	LN225.079	LN225.077	LN225.077	LN225.078	LN225.078
113149.7	95412.67	99891.31	121563.8	114523.1	57667.42	117005.6	92320.54	72846.48	109002.4
108313.2	158149.9	97082.99	114986.3	118846.1	60280.35	120212.5	105371.4	71680.98	147805.8
114013.8	101224.1	105627.3	117516	115976.8	60561.18	110351.4	99434.35	78774.11	105501.2
118252.2	102570.9	101897.9	115305.3	127385.9	58129.24	116857.4	102690.7	76716.11	101547
116623.5	104966.7	107773.1	115781.6	131025.1	65261.55	114888.4	99559.7	75805.97	105915.2

LN225.078	LN225.079	LN225.077	LN225.078	LN225.079	LN225.078	LN225.079	LN225.078	LN225.079	LN225.079
50533.92	44382.59	111628	113925.2	55052.22	64830.59	49187.5	116055.5	114589.4	204547.1
48018.33	50033.87	118345.1	111666.5	54288.34	65427.63	52265.66	119079.7	117913.7	205184.2
54734.53	50441.18	115597.6	118688.8	58870.57	67037.09	49777.83	110018.4	125239.2	202769.1
53133.09	55423.52	126185.1	112424.4	57501.97	70098.64	55316.57	123691.8	115609.3	203315.4
52110.64	58288.71	118127.2	111507.7	62345.99	69812.59	54741.31	113102.3	108421.3	200943.2

LN225.079	LN225.078	LN225.078	LN225.078	LN225.078	LN225.078	LN225.079	LN225.078	LN225.078	LN225.078
118147.7	92681.47	51458.66	102638.3	138714.5	95406.72	89270.22	57824.87	98544.92	72953.16
116368.6	98387.99	59269.84	99784.14	132338	102042.6	94888.02	56351.66	100794.1	72652.38
114046.2	88675.87	59184.1	102054.6	135534.3	106152.6	95428.54	62023.88	105010.4	74474.04
126959.6	88790.43	58904.5	103534.6	138035.9	99806.95	95223.2	59662.87	107996	80907.78
117877.7	96058.75	54330.52	100283.5	130397.9	96461.69	93373.46	61751.7	99893.02	78976.93

LN225.078	LN225.079	LN225.079	LN225.079	LN225.078	LN225.896	LN226.963	LN226.965	LN226.965	LN226.965
44952.22	51285.38	74072.96	54158.58	58099.09	246212.9	62033.32	143959.8	125738.3	162012.8
45149.66	48054.46	79659.6	59332.68	54557.55	239411.5	46783.41	145478.1	123860	159115.8
47679.65	51041.01	76903.34	60159.86	55885.12	208405.6	53030.01	147027.3	118688	156628.2
45930.74	57635.18	72108.64	61635.73	57535.01	289342.5	71160.08	143612.8	125768.3	148148
49160.7	50979.06	72675.94	62708.98	57860.87	316887.8	44397.03	136297.8	120193.2	145118.2

LN226.965	LN226.965	LN226.978	LN226.978	LN226.994	LN226.994	LN226.994	LN226.994	LN226.994	LN226.994
137582.1	169043	140079	137493.3	55539.94	50897.89	113884.7	96511.58	90611.52	81193.06
124141	160405.9	140662.4	133358.8	54343.52	43934.19	125159.2	87546.02	102880.8	86439.36
144828.5	160245.9	136529.9	139653	37233.33	94328.24	138028.7	140372.2	102775.2	87904.26
133735.4	151816.8	141087	134795.9	93501.68	116311.5	144623.5	86466.59	101234.5	88006.29
126146.6	158571.9	127694.6	128915.1	46464.47	48172.91	125365.5	90306.02	146567	85102.38

LN226.994	LN226.994	LN226.994	LN226.994	LN226.994	LN226.994	LN226.994	LN226.994	LN226.994	LN226.994
81357.01	64368.41	72518.33	81221.95	61685.14	54766.14	139475.5	111041.2	107578.8	71307.52
86576.64	97233.65	85181.37	71933.02	66034.26	53704.05	90289.41	71053.68	60393.99	72735.22
162430.3	77162.2	76620.63	65819.52	50407.75	78077.56	94784.23	89461.25	80194.99	54935.69
161499.7	58761.01	146421	110411	103087.9	106549	95327.67	141904.7	137532.4	117105.5
95365.78	73558.68	72254.34	73448.51	57039.32	61758.26	114142.2	81240.34	74873.99	36644.55

LN226.994	LN226.994	LN226.994	LN226.994	LN226.994	LN226.994	LN226.994	LN226.994	LN226.994	LN226.994
101702	76270.26	94141.34	79796.45	52356.7	41888.29	55399.47	44800.95	81531.11	75230.8
63740.71	62726.07	63725.55	98860.58	58352.08	58880.94	50107.52	61826	46787.25	102935.8
74481.53	45687.57	79064.23	57302.81	79465.13	55772.45	63610.71	39200.26	128379.9	99589.49
65661.02	142029.9	125634.9	70363.39	133721.1	102362.6	133414.4	97875.07	130604.3	132150
95517.84	56672.62	49818.73	67494.46	33228.44	54721.54	52532.78	33054.37	56762.7	91906.25

LN226.994	LN226.994	LN226.994	LN226.994	LN226.994	LN226.994	LN226.994	LN226.994	LN226.994	LN226.994
23955.55	102511	40779.8	78622.63	63500.53	57958.09	91529.38	60935.22	53530.42	70318.97
21436.59	102633.6	36094.93	27175.8	67463.64	43161.18	89330.32	60001	54642.02	97184.49
17275.11	100401.7	27269.58	24495	45566.5	57946.26	118711.8	56158.24	46393.51	62959.23
49526.36	83221.32	65408.32	75647.82	104399.6	115694.8	81025.98	123693	127694.6	75524.09
22496.54	96631.09	21823.86	23230.1	40037.57	54373.23	69740.41	50598.39	48894.87	49869.45

LN226.994	LN226.994	LN226.994	LN226.994	LN226.994	LN226.994	LN226.994	LN226.994	LN226.994	LN226.994
135556.8	38634.29	86439.73	44100.03	87284.9	119542	51146.61	57374.06	47189.34	91877.05
65391.08	55920.49	81026.84	43931.56	85701.22	78685.87	106392.1	67575.42	115565.8	125328.4
65391.71	48548.11	96601	46543.8	99097.69	112113.8	88098.01	57824.95	48807.41	126321.5
66396.85	110236.1	69519.03	97084.46	94376.64	85387.04	122322.4	75123.34	115283.1	143306.1
81707.38	52517.72	83717.66	54308.32	98247.83	107866.1	47565.82	106898.8	41089.8	88551.72

LN226.994	LN226.994	LN226.994	LN226.994	LN226.994	LN226.994	LN226.994	LN226.994	LN226.994	LN226.994
37821.01	26314.9	92078.6	41064.16	131988.3	98207.5	66831.33	59545.75	127878.5	110348.8
33024.63	27893.63	97052.22	36536.47	145483.7	72013	62259.35	44312.3	132697.4	98384.31
20882.93	25179.47	39594.95	43848.4	102194.4	124475.8	102622.6	51759.59	83290.11	103087.7
75797.44	66287.64	79897.78	95632.68	85194.9	103641	108602.3	92864.99	77592.1	97952.56
22070.23	29319.85	79725.23	51258.82	114617.4	97382.57	60623.09	46923.5	90681.31	86533.32

LN226.994	LN226.994	LN226.994	LN226.994	LN226.994	LN226.994	LN226.994	LN226.994	LN226.994	LN226.994
42793.23	39057.41	51778.13	37592.5	96154.79	52044.13	112187	69094.1	87573.46	24262.36
76375.56	33833.02	60159.71	37255.83	93008.07	55009.51	110446.1	84758.49	116639.5	37811.91
40162.89	24354.03	47831.44	89279.2	28045.61	48134.42	86423.54	125327.4	68068.65	70990.54
97944.3	67476.78	106893.6	82925.89	83979.75	113834	81040.12	112522.1	65443.35	66816.67
48123.85	28914.54	44042.35	40031.65	79421.52	45218.24	89818.63	114171.1	95915.06	23570.17

LN226.994	LN226.994	LN226.994	LN226.994	LN226.994	LN226.994	LN226.994	LN226.994	LN226.994	LN226.994
58506.57	110272.4	36878.27	74246.56	63318.5	110880.9	61147.92	92729.11	44301.13	44030.16
59169.52	76025.76	24486.51	74084.38	103465.8	68930.2	130943.1	86453.27	38419.35	115765.8
34109.87	118850	29445.62	89409.56	95009.63	75476.93	59698.93	74181.47	39570.96	52051.04
127681.3	125156.8	83419.98	141287.3	103691.7	123512.9	136864	135502.1	100491.3	106757.3
47779.55	88571.47	25360.63	57958.99	78549.32	43564.07	75847.67	85832.96	34112.94	61550.74

LN226.994	LN226.994	LN226.994	LN226.994	LN226.994	LN226.994	LN226.994	LN226.994	LN227.893	LN228.991
63694.15	74500.6	24773.02	23278.38	58989.09	83122.09	50337.71	31035.26	77144.15	35755.11
63942.22	67993.98	23519.72	29192.02	42004.88	35546.32	52300.99	25431.63	74352.79	41200.69
59407.79	61487.14	34029.7	21768.15	32308.81	49789.66	54658.4	26088.86	67723.23	40027.26
111310.9	138762.8	73152.85	52190.08	114296.1	81123.07	135516.6	28840.79	87503.66	41569.59
117533.6	54344.22	22433.95	19472.37	103488.1	37434.28	85255.28	28615.26	94964.02	41428.92

LN228.991	LN228.991	LN228.991	LN228.991	LN228.991	LN228.991	LN228.991	LN228.991	LN228.991	LN228.991
28551.74	39352.58	43756.69	33476.33	41741.69	36288.13	41775.08	31043.14	40804.93	32446.12
25956.06	37732.17	42782.11	34217.88	40594	33953.58	41791.13	33289.71	39391.95	27847.15
31572.05	40242.92	47062.12	35704.46	33274.53	37510.44	43375.54	33346.52	41412.57	36501.17
33706.05	44965.78	50576.34	37565.67	41491.43	34986.86	40965.07	33211.85	43277.01	36900.25
33076.84	46073.76	53715.58	36985.06	41896.47	37912.9	42893.88	36616.1	42155.37	36976.64

LN228.991	LN228.991	LN228.991	LN228.991	LN228.991	LN228.991	LN228.991	LN228.991	LN228.991	LN228.991
35220.48	30519.21	37369.67	40213.41	27835.45	39677.45	24603.83	27065.01	24581.2	26638.05
34402.01	30371.1	34121.69	41610.4	28463.49	41704.99	25484.85	26130.69	24592.53	29152.11
36566.52	31436.61	35870	40432.67	32588.68	41683.2	27812.13	28506.36	23902.99	29515.59
37283.95	30907.32	40380.48	46248.61	33188.43	41027.1	27796.86	29151.27	24358.72	29487.78
39714.94	32945.87	38700.47	42965.82	32673.16	38906.78	26666.18	30181.8	29378.26	30051.2

LN228.991	LN228.991	LN228.991	LN228.992	LN228.992	LN228.991	LN228.991	LN228.991	LN228.991	LN228.991
23759.27	27342.63	33773.6	20599.2	20438.43	17059.72	12480.96	20943.05	16433.16	14236.3
25968.05	27794.05	35017.6	22239.9	20033.77	15598.15	12240.72	20864.88	16302.63	16034.23
27358.98	29514.77	33812.39	22668.6	22313.78	18078.79	10264.53	20883.27	17332.14	13681.9
24602.52	29795.9	37238.7	24105.66	22661.27	15837.71	10671.73	22432.01	15266.52	14626.5
24469.2	27936.66	35793.47	23309.47	20439.15	18695.8	9581.666	22044.97	16655.19	14099.78

LN228.991	LN228.991	LN228.991	LN228.991	LN228.992	LN228.991	LN228.991	LN228.991	LN228.991	LN228.991
16100.86	18622.14	23346	16829.25	17005.84	14110.16	15980.61	23346.41	11959.26	15507.47
18663.49	19883.18	22242.46	14876.32	18562.24	13552.08	16500.14	23080.6	15720.97	15309.18
18301.03	19510.32	24149.25	14634.28	17050.02	14002.38	15616.22	24702.39	12664.99	16141.95
15997.99	22604.77	22444.14	14103.84	18701.32	13912.6	15626.16	24087.38	13298.73	14467.37
16566.25	21242.15	21160.93	14865.93	16423.85	13798.48	15546.79	24366.52	13220.63	15441.84

LN228.991	LN228.991	LN228.991	LN228.991	LN228.992	LN228.991	LN228.991	LN228.991	LN228.990	LN228.991
25622.32	26283.89	22675.95	29977.52	19628.45	29532.61	24872.23	24197.67	12084.74	28948.83
24873.25	24952.83	22904.53	29165.05	20088.08	30334.25	25409.83	21190.07	12902.26	29984.26
26754.56	24039.64	22887.61	25680.51	18748.92	30773.65	22700.8	23729.69	11679.66	28741.92
23718.93	24190.43	23157.85	27392.89	17611.99	31148.58	24829.41	21116.92	11130.99	28474.99
25410.34	23750.76	23183.9	27674.95	20105.97	29544.46	24139.87	23396.16	12452.11	28497.73

LN228.991	LN228.991	LN228.991	LN228.991	LN228.991	LN228.991	LN228.991	LN228.991	LN228.992	LN228.991
21892.42	23454.93	13976.44	20240.36	15307.5	15518.4	15017.27	18353.92	24047.38	22313.26
22364.73	21366.52	13575.81	19267.25	17366.04	14774.87	13500.4	18902.64	22338.67	19697.49
22585.23	23235.9	15068.53	19531.11	16432.88	16834.4	13528.87	19553.79	23500.67	20251.21
21445.83	22878.78	12857.76	19931.86	17957.23	14996.06	13784.26	16092.7	18652.71	21456.87
22342.81	22929.32	13701.71	18953.33	17752.66	17139.26	13263.86	17700.93	20387.76	21787.67

LN228.991	LN228.991	LN228.990	LN228.991	LN228.991	LN228.991	LN228.990	LN228.991	LN228.990	LN228.991
22977.82	24360.94	13200.79	19709.5	36012.67	28406.92	12712.84	17653.4	11532.17	25181.63
19992.43	26947.75	10792.21	21931.66	35752.18	27361.98	13929.52	16777.19	13553.9	28194.82
18373.29	29382.9	13135.05	19807	34178.58	26849.11	11364	15700.07	10322.41	28892.52
19636.15	24179.84	12904.03	20380.21	35608.9	24915.93	13652.57	17401.98	10876.98	27965.98
20032.43	28426.52	12274.42	21588.25	35223.64	24397.99	12278.7	17407.01	10118.7	28479.79

LN228.990	LN228.991	LN228.990	LN228.991	LN228.991	LN228.991	LN228.991	LN228.990	LN228.991	LN228.991
11359.07	17517.21	11421.06	26684.5	19110.97	36381.9	25713.06	21490.22	26428.15	39537.81
12923.89	18274.51	13502.41	28626.68	21397.87	34966.38	26304.79	20510.01	23546.19	35107.04
10051.95	17061.92	13949.75	27009.79	22625.31	33284.37	24458.68	19104.3	25252.46	35905.87
11171.45	17594.82	12568.74	27689.66	21911.1	34134.21	27002.63	17999.4	24884.66	33785.4
10815.76	16225.96	12895.59	27294.52	22524.12	30960.24	26340.01	17017.26	25456.11	37409.82

LN228.991	LN228.991	LN229.008	LN229.144	LN229.143	LN229.144	LN229.873	LN229.936	LN230.047	LN230.047
23137.28	24991.99	35960.99	23669.06	19005.63	19517.23	26479.95	33262.34	46323.36	54600.23
24935.12	25150.2	32877.85	23474.33	16796.44	20658.54	20946.45	23852.51	42100.15	52874.96
23419.11	22524.69	34303.74	22970.26	18161.08	17009.19	19473.56	18936.37	52442.83	50599.53
24848.59	23385.57	34555.31	21606.05	18328.77	18260.16	24003.96	17174.03	52582.65	53621.92
25102.63	25610.18	32947.19	21174.6	18067.24	18827.18	24558.37	27130.36	50504.73	48887.94

LN230.047	LN230.047	LN230.047	LN230.047	LN230.047	LN230.047	LN230.047	LN230.048	LN230.048	LN230.048
57059.96	42812.87	60316.98	52251.31	38694.6	33378.06	94970.57	56906.53	109613.6	62338.39
59712.63	53023.71	59459.6	48262.36	40606.19	33136.84	93096.64	92097.78	104294.2	78746.93
52391.81	49691.75	65341.91	53208.87	43603.81	34895.49	93359.25	86390.94	162451.1	72346.82
57047.7	45589.97	57905.38	52073.45	46152.63	32508.21	90622.59	78127.99	130100.1	69404.95
58135.69	55105.79	59099.25	50499.99	36525.2	35208.23	93201.74	79436.21	120649.6	104055

LN230.048	LN230.048	LN230.048	LN230.048	LN230.048	LN230.048	LN230.048	LN230.048	LN230.048	LN230.048
30999.11	33053.01	98687.39	98960.43	88664.36	128355	94366.08	113472.8	98826.97	135943.4
27755.3	42627.45	123187.9	108529.9	74603.71	133184	122400.7	103750	99285.23	96567.24
26156.02	37572.69	155024.5	120401.6	100226.5	82570.01	89122.45	135951.4	96802.28	134364.8
28739.42	42541.81	130338.6	94274.22	67564.49	95591.6	81930.51	124574.4	111669.6	119780.6
52165.6	32019.7	141510.9	87287.55	62019.65	113779.1	141126.5	91828.37	138318.7	110346.7

LN230.048	LN230.048	LN230.048	LN230.048	LN230.048	LN230.048	LN230.048	LN230.048	LN230.048	LN230.048
72601.47	136890.9	101574.9	19550.01	136733.6	49716.39	118911	21389.87	84072.09	108273.9
82624.22	72841.87	118109.5	18254.66	162695.5	72168.65	115783	22202.42	58453.98	95371.51
52434.04	86735.95	71700.52	16488.58	96291.8	50645.41	145783.1	28927.63	51088.25	106097
77311.82	153744.6	95271.63	16873.69	100700.8	72688.33	110780.7	24324.75	62907.46	106877.3
81338.42	106822.5	96501.24	20522.54	126339.1	56533.55	107892.3	30892.74	55997.07	133875.6

LN230.048	LN230.048	LN230.048	LN230.048	LN230.048	LN230.048	LN230.048	LN230.048	LN230.048	LN230.048
78622.8	175349.4	54837.25	42807.72	72720.89	36566.46	74823.08	70223.76	142811.6	44802.1
105076.9	144010.6	63845.27	37164.33	86156.28	63049.67	45823.94	52453.8	106076.1	45194.52
102479.6	166235.3	35236.2	50664.75	88987.33	40133.29	56634.64	56901.18	88365.04	46685.7
89762.75	170814.7	41147.17	49885.12	63026.08	54530.35	83792.95	45391.91	114623.5	42188.06
94971.18	118911.1	48272.99	54916.46	67925.41	56159.58	57705.34	41693.11	132044.3	40091.23

LN230.048	LN230.048	LN230.048	LN230.048	LN230.048	LN230.048	LN230.048	LN230.048	LN230.048	LN230.048
51762.65	67319.91	92238.53	55038.92	52237.01	57708.98	31262.66	66535.36	119508.5	83297.99
62174.52	82576.62	111707	38607.62	61162.35	41945.99	32739.88	40058.69	154369.4	186001.6
66286.99	104859.2	97326.31	40944.76	79082.12	43273.28	30006.18	43439.37	103309.9	126624.8
59651.53	65725.22	85741.04	51183.96	53403.9	38304.98	34166.42	57912.6	122236.9	94659.96
59754.51	67765.96	99434.3	57372.7	86915.76	46069.33	26782.03	43225.54	85873.9	111698.7

LN230.048	LN230.048	LN230.048	LN230.048	LN230.048	LN230.048	LN230.048	LN230.048	LN230.048	LN230.048
111728.2	114358.5	44280.18	155885.2	28198.19	29823.08	104984.1	83965.09	90559.35	33853.07
75595.56	109783.1	39788.97	108499.5	30519.09	26940.71	110891.5	93860.87	95392.06	39080.37
116532.9	145666.5	60168.23	105393.3	29499.74	27435.66	109792.3	71890.45	102579	25052.83
118270.9	132048	50928.2	121294.9	31724.49	28504.44	89535.84	73086.4	87442.61	39517.27
111421.3	91056.62	32779.1	98981.42	34480.66	34948.84	117013.4	69876.22	96613.32	26907.1

LN230.048	LN230.048	LN230.048	LN230.048	LN230.048	LN230.048	LN230.048	LN230.048	LN230.048	LN230.048
31153.27	86762.93	128583.3	54319.96	108771	29888.67	38648.62	68725.73	24486.25	150259.2
25197.09	101800.6	86045.13	48193.95	94693.36	34089.02	25787.12	72197.86	21823.75	151066.4
43298.98	95238.72	129136.8	46651.3	106744.9	50808.78	28462.72	52230.66	20047.1	123976.9
24524.35	119358.9	100727.1	45365.44	85895.89	32030.26	27818.71	71682.7	24567.67	123343.2
26481.17	60933.17	93316.36	64902.33	72476.22	24537.41	28713.56	52512.27	29456.32	161444.8

LN230.048	LN230.048	LN230.048	LN230.048	LN230.048	LN230.048	LN230.048	LN230.048	LN230.048	LN230.048
52379.01	93003.43	57658.78	78656.76	53930.07	110619.2	51181.66	36803.28	19178.94	43656.77
67663.89	75648.05	72989.73	63013.18	47427.45	89853.68	48594.4	24271.7	27601.29	67020.24
72066.79	102374.1	67305.7	48126.89	43436.2	110284.2	64631.3	25985.59	25539.22	52364.89
47663.59	101575.3	66552.04	48724.09	42852.56	136855.3	51507.29	26136.17	22519.49	58880.01
53307.51	87400.57	65628.56	48674.9	55787.85	100808.9	47203.97	27354.45	21245.55	58107.36

LN230.048	LN230.048	LN230.048	LN230.048	LN230.048	LN230.048	LN230.048	LN230.048	LN230.048	LN230.048
55697.34	35378.99	62254.55	49398.53	61794.87	47763.46	102042.3	61314.25	43283.85	41537.72
90714.21	38392.56	44284.34	39865.18	46348.76	59949.6	73299.03	68718.01	34313.61	109063.3
86505.94	36983.38	48085.17	56474.64	56665.14	52543.4	121211.6	54098.72	59837.38	49968.67
85261.43	65633.34	38414.23	50152.25	51712.09	83118.06	77565.92	74482.19	46767.62	58106.34
58736.53	57486.83	32993.35	43219.4	68007.27	63892.71	98991.96	66072.56	49567.23	60501.28

LN230.048	LN230.765	LN230.881	LN230.955	LN230.985	LN230.985	LN230.985	LN230.985	LN230.985	LN230.985
37779.97	36100.4	12198.95	27106.22	36846.05	51471.81	42663.6	48971.67	52704.34	40233.5
76380.08	30647.83	11257.68	27310.43	41857.22	44010.06	39818	41729.2	40834.09	48938.2
63146.22	34134.94	13092.15	27175.95	36560.26	34019.52	51452.22	38386.99	48338.34	57791.5
62601.62	28335.92	10415.1	26992.62	41531.35	40638.71	37287.87	44253.32	44325.11	49670
51931.73	30880.93	14301.54	32159.57	36532.19	41331.13	37257.76	44868.91	45028.24	50992.92

LN230.985	LN230.985	LN230.985	LN230.985	LN230.985	LN230.985	LN230.985	LN230.985	LN230.985	LN230.985
51951.45	29265.14	26870.32	46553.52	32362.04	30532.05	26565.06	33815.93	40079.05	40479.07
44772.01	31373.95	41226.36	33158.56	33488.66	32014.19	24122.86	32221.24	31831.05	34939.55
38721.83	36093.34	41489.15	29768.49	27988.72	41617.9	43072.94	30306.7	43957.26	44992.1
43274.57	35616.21	38937.63	34885.65	32199.56	33083.03	29293.6	34293.06	35795.71	39542.14
43298.42	33312.08	36877.54	38440.64	34150.87	30620.87	26815.63	34789.5	34653.44	37658.5

LN230.985	LN230.985	LN230.985	LN230.985	LN230.984	LN230.985	LN231.005	LN231.930	LN231.931	LN231.931
29007.03	38561.59	26957.38	34724.26	43424.62	36605.77	22727.1	349496.5	882683.3	146939.2
28159.54	32755.77	27808.05	32839.62	46472.87	36876.94	22416.16	401393.5	1211603	195836.3
31841.04	35388.99	25880.26	33214.9	42751.34	38946.02	23455.04	250152.9	690829.4	213583.8
27604.56	33166.61	29030.83	32052.18	45868.73	37147.91	21468.38	336646.4	791585.7	165579.2
28770.63	36418.73	28163.51	34661.58	44987.75	35474.28	23588.55	371184.3	1013358	169430.5

LN231.931	LN231.931	LN231.931	LN231.931	LN231.931	LN231.931	LN231.931	LN231.931	LN231.931	LN231.931
210786	185331.2	170978.5	175088.3	166527.8	165186.6	122463.3	149715.9	220460.9	225908.3
168051.2	137467.6	143432.7	253675.3	170204.4	149725	140495.2	188911	147453.4	176990.2
164072.6	177412.4	217772.4	235850.4	156531.9	159239.1	156110.9	112598.3	132476	126747
193112.2	148726.7	227218.4	170506.1	175026.8	127092.9	158846.7	148598.4	152102.8	132407.5
147931	168089.8	202599.3	216743.3	168466.1	162905.4	122638.4	161601.9	183236.7	174997.9

LN231.931	LN231.931	LN231.931	LN231.931	LN231.931	LN231.931	LN231.931	LN231.931	LN231.931	LN231.931
194679	167604.1	158848.9	174066.5	188441.3	136429.1	299009.4	162713.5	140083.1	143036.6
158571	205214.2	199999.8	162111.8	153643.6	174713.7	176503.1	154052.5	126944.1	234180.8
126795.2	183603.2	160658.7	149139	165454.9	126049.4	161293.6	154170.8	204777.7	125549.3
150893.7	156738.6	159224.8	156522.6	203624	103181.9	197673.1	183508.8	172040.4	158422.2
150392.8	109847.6	165905	239043.6	145016.9	166189.5	196596.7	193951.4	167020	129965.2

LN231.931	LN231.931	LN231.931	LN231.931	LN231.931	LN231.931	LN231.931	LN231.931	LN231.931	LN231.931
127652.7	147071.6	130506.5	206953.8	116832.4	129635.7	222491.6	151729.4	115871.7	110503.9
119433.4	193576.5	156208.7	140509.1	146059.3	150838.6	168120.3	188312.4	123685.5	161039.4
224381.1	177744.1	130216.9	142181.9	209763.4	93499.96	175711.1	100794.2	118871.7	100818.1
169529.3	120810.1	130398.5	119501.4	181463.1	130688.1	171177.7	144852.9	136852.3	158077.7
125867.8	167548.6	154025.6	159167.2	208657.8	124721.3	201465.8	129228.5	185135.3	103245

LN231.931	LN231.931	LN231.931	LN231.931	LN231.931	LN231.931	LN231.931	LN231.931	LN231.931	LN231.931
204238.5	127104.9	192284.2	247316.1	190770.7	140788.7	144950.2	124314	192057.8	154598.4
219616.4	190909.5	229947.2	130541.6	150599.6	188830.4	149256	170417.4	228206.5	185481.6
209108.4	179740.7	164174.7	135232.8	124477.6	219848.6	135410	126338.5	165526.8	137164.1
167601.8	188463.9	150588.2	139128.6	135609.7	200688.4	112853	178954.9	205373	137648.1
170565.1	147801.6	222476.1	196622.3	139582.1	130892.6	149021.4	157875.7	108418.7	159238.3

LN231.931	LN231.931	LN231.931	LN231.931	LN231.931	LN231.931	LN231.931	LN231.931	LN231.931	LN231.931
171652.3	187992.4	195331.4	172346.6	216463.7	138926.3	183681.5	150655.9	214297.2	148387.5
160745.1	239531.9	178426.1	149248.4	199969	186524.6	169033.1	118734	160055	106846.9
150646.3	164944.8	277332.2	191958.4	149980.6	118385.2	148431.5	124723.7	132736.4	131831.6
157385.2	152230.5	265162.8	194355.5	129103.8	144468.3	214381	139837.5	161056	123020.7
131845.2	159546.7	217283.8	150519.3	244467.2	174599.1	166992.5	117401.1	166660.9	108590.7

LN231.931	LN231.931	LN231.931	LN231.931	LN231.931	LN231.931	LN231.931	LN231.931	LN231.931	LN231.931
150635	140733.7	208327.7	255254.8	137343.6	130633.4	159076.2	136839.1	238504.1	125548.1
114936.3	151341.6	165029.3	191240.1	191828.2	120923.7	185962.6	165025.3	239692.8	184517.4
145672.4	124981.9	160435	173148.9	213391.4	169341.4	150746.5	108327.9	154820.1	136039.9
104960.2	177516.9	158316.7	192676.9	191010.4	120293	144375.8	134376.2	202696.2	100075.9
114787.7	127748.4	157859.2	196492.8	131160	118195.6	183874	150237.3	208717.8	102737.4

LN231.931	LN231.931	LN231.931	LN231.931	LN231.931	LN231.931	LN231.931	LN231.931	LN231.931	LN231.931
146919.5	162858.9	208733.2	140962.4	206597.8	113433.9	167981.1	121635.9	155990.8	131736.7
130793.4	141459.3	262587.2	256480.5	188217.9	159236	279866.1	127673.5	149733.1	147408.1
139086.7	185261	222182.3	176860.6	167716.1	103402.1	151392.9	131559.9	229165.8	111351.1
142802.7	151298.8	217576.3	255220.2	132197.3	92207.7	148246.6	140912.9	228798.1	132043.5
123498.9	175520	264711.3	238928.5	225433.5	180660	147865.5	147753.7	251718.4	138447

LN231.931	LN231.931	LN231.931	LN231.931	LN231.931	LN231.931	LN231.931	LN231.931	LN231.931	LN231.931
172938.2	139057.4	175534.3	146079.6	196599.8	158519.9	229540.1	154718.6	171762.3	256486.9
204796.4	126771.8	159147.1	189685.9	174492.5	221562.7	197151.8	151671.6	163468.6	219735
170237.4	112988.1	203397.1	161216.6	190130.3	144546.5	182562.9	192355.6	174628.5	195813.2
144474.4	224270.1	167223.7	96630.5	149584.8	200946.1	245931	183145.7	180712.9	233617.6
163112.2	165494.8	178187.2	136720.3	194921.1	157859.4	200629.3	143234.7	188659.6	210588.4

LN232.763	LN232.914	LN232.930	LN232.931	LN232.931	LN232.932	LN232.931	LN232.931	LN232.932	LN232.931
65505.85	54858.06	50582.21	15872.68	16351.6	43985.52	20919.15	16602.18	43371.79	23474.37
52675.71	49699.2	44103.29	15224.81	16068.81	45935.95	21219.25	13409.27	45116.02	20894.8
56669.5	53880.18	37250.52	12120.97	14757.15	48080.41	23868.42	13748.1	39919.41	21033.52
42287.81	54251.85	42418.37	15443.14	17496.1	50352.47	19418.92	12306.1	37456.72	20442.73
47845.81	56931.74	34695.56	15826.37	18297.04	46486.04	19202.98	12749.42	37767.54	21942.64

LN232.932	LN232.932	LN232.932	LN232.932	LN232.932	LN232.932	LN232.932	LN232.931	LN232.932	LN232.932
51099.22	42974.3	29188.39	39180.47	34391.96	30214.99	40975.32	33983.89	24221.83	32084.01
48792.63	45691.02	28026.9	41332.9	36050.42	30730	39915.33	34588.35	26529.21	31192.76
47906.78	41502.68	32654.14	38305.15	32898.34	28562.1	36596.21	35062.97	22901.17	32235.19
47905.05	38696.47	27231.55	41941.07	31177.24	28208.85	38524.24	34123.61	24033.27	27531.12
48177.91	41189.53	26926.89	41632.51	38925.51	27387.64	36724.77	32483.4	23888.05	27018.68

LN232.932	LN232.932	LN232.932	LN232.932	LN232.932	LN232.931	LN232.931	LN232.932	LN232.931	LN232.931
34884.84	28174.76	28310.65	21659.26	23464.82	52808.19	22463.53	31966.33	23833.95	30724.98
33789.26	29627.19	29470.95	23160.51	22608.58	56317.5	20729.38	32378.57	23678.25	29713.19
35596.68	26884.35	26765.84	22295.05	21493.57	52454.46	22200.78	32587.25	24396.85	28448.03
33014.93	25984.13	24845.81	17031.45	25376.45	51490.26	22841.93	32519.21	22645.69	26805.7
36762.57	26522.4	28254.04	20294.74	20690.3	52612.06	19070.6	34056.77	25756.57	28770.21

LN232.932	LN232.932	LN232.932	LN232.932	LN232.932	LN232.932	LN232.932	LN232.932	LN232.932	LN232.932
56528.25	22910.58	65249.84	35150.96	42312.72	30796.61	40743.74	43918.77	28904.3	48792.6
54542.14	20549.26	61450	34603.06	35536.76	33367.58	40837.57	44416.94	27364.8	45475.13
52762.49	22055.09	63283.55	35738.74	38797.24	31764.36	36959.94	45867.77	28726.96	47237.15
49938.15	22167	60638.2	37738.73	44042.05	31720.78	38463.49	50129.05	25414.92	46695.92
52174.63	20848.91	54972.36	31514	41338.97	31324.84	36359.32	42169.25	25487.88	41996.95

LN232.932	LN232.932	LN232.931	LN232.932	LN232.932	LN232.932	LN232.932	LN232.933	LN232.933	LN232.933
43152	52204.51	22197.06	30899.73	49294.26	59017.17	31423.4	45604.64	45611.61	41352.25
40641.69	54292.88	22541.18	31830.18	49366.91	57588.55	32340.57	46228.43	45054.22	43660.66
42466.2	53248.82	23051.27	32186.13	44362.61	56510.53	30646.37	45549.1	42753.15	37654.9
39435.77	53179.48	20143.03	30677.84	46261.78	52617.64	27644.4	42601.62	43270.38	39273.8
34621.15	53409.92	21328.89	30900.34	51564.84	51373.2	28196.03	45202.46	39958.41	38845.42

LN232.933	LN232.933	LN232.932	LN232.933	LN232.933	LN232.933	LN232.932	LN232.933	LN232.933	LN232.933
45718.68	45875.11	43294.04	48407.29	42512.98	54354.47	23753.67	39777.53	45600.45	54659.75
46254.75	48121.09	44543.96	51949.48	44925.15	50978.5	25456.81	38398.94	51881.42	56966.47
47678.13	41819.83	42774.41	44126.9	45372.72	47011.6	20678.99	43788.78	44310.2	55635.15
42060.21	45688.75	43521.16	41010.98	40599	50025.04	22114.32	40115.05	45277.45	56441.66
40582.79	42993.16	41053.83	43329.93	42019.74	47111.85	22608.37	38107.13	45312.2	55175.42

LN232.933	LN232.933	LN232.933	LN232.933	LN232.933	LN232.933	LN232.932	LN232.933	LN232.933	LN232.933
47280.08	38313.51	33794.06	40053.03	45607.72	44832.57	28157.84	48990.8	67873.8	35115.98
48982.9	30946.44	33791.51	37959.8	47173.03	44883.98	29663.21	51032.24	65468.61	36033.76
43979.29	30915.71	34115.99	37558.5	43841.47	43391.77	31469.11	46851.54	68045.06	37846.55
50622.17	30476.09	33821.17	33725.15	44536.4	40916.56	27536.16	48755.98	62507.12	34952.76
45580.2	31446.85	30224.78	39745.68	45233.98	42773.72	28407.38	48922.23	64911.57	34641.29

LN232.933	LN232.933	LN232.933	LN232.933	LN232.933	LN232.933	LN232.933	LN232.933	LN232.933	LN232.933
64298.92	46493.81	62652.33	43429.46	55957.25	56704.55	38248.96	39383.22	31886.01	40079.56
63945.01	45339.59	65506.05	44044.72	55405.67	59875.27	39731.34	41621.33	31116.72	37005.49
60958.62	50159.8	58229.25	44755.21	55847.85	53844.17	42993.52	34100.44	29361.9	37857.71
59604.99	47247.86	63801.65	42923.59	54846.49	58361.73	38257.98	33093.57	30928.32	37420.22
62312.21	46322.98	60863.04	41654.29	56087.25	60347.49	37822.06	36848.26	26557.79	39374.68

LN232.933	LN232.933	LN232.984	LN232.984	LN232.983	LN232.984	LN232.983	LN232.984	LN232.984	LN232.984
43292.57	48445.15	23908	39454.39	27995.66	27241.66	30937.21	24921.34	17017.89	24069.19
43420.26	49561.52	27518.31	31794.34	27540	37439.91	30084.22	23946.32	17991.59	24023.62
43379.34	50186.19	27279.19	31413.7	28404.4	36243.01	33270.09	25285.34	16275.81	26677.72
44120.4	45708.63	27837.97	28402.86	28839.16	33350.33	30589.44	24669.71	15095.08	23176.92
42448.08	49298.4	23955.55	38148.98	33039.75	27952.03	29937.56	24926.73	17388.86	24265.76

LN232.984	LN232.983	LN232.984	LN233.154	LN233.154	LN233.154	LN233.154	LN233.154	LN233.154	LN233.154
23411.9	23384.28	30636.23	97905.93	104475.4	129782.9	133651.7	108458.7	118745.7	89266.38
22101.83	25216.72	30098.5	85027.81	161752.3	137475.1	88801.51	67362.35	149028	108676.2
25166.11	25215.67	29503.9	70176.7	82177.9	96879.2	108265.7	101091.2	128546.7	114050.8
23407.18	27598.64	32869.28	74664.62	86373.37	107856.1	122069.6	106440.1	147956.5	109634.9
24242.72	22474.83	30384.9	127339.3	226715	110774.5	154734.8	149986.4	172943.8	112468.3

LN233.154	LN233.154	LN233.154	LN233.154	LN233.154	LN233.154	LN233.154	LN233.154	LN233.154	LN233.154
142090.3	82990.26	108724.8	88562.49	113112.3	109338.8	95468.36	71577.83	70308.31	59148.23
165446	89467.94	97530.34	112287.8	100278	58101.16	121125.9	78831.13	85782.5	57919.29
137865.4	89863.46	146775.8	104808.1	105853.7	75579.36	131720.9	85375.96	94536.7	75668.95
131721.6	125480.8	162695.4	111050.4	114264.1	92475.69	118254.1	96162.76	85170.02	56516.38
149058.1	148680.6	157559.9	125249.3	104040.9	90489.34	136715.5	102621	125430.2	92256.24

LN233.154	LN233.154	LN233.154	LN233.154	LN233.154	LN233.154	LN233.154	LN233.154	LN233.154	LN233.154
110064	64980.97	90081.09	137554.7	73725.69	108686.7	84158.43	98873.52	135428.3	74735.72
118048.3	61000.36	110470.9	124809.5	90540.3	87576.77	90268.18	96450.41	104432.3	85208.23
119429	101061.1	114846.5	145958.6	77842.46	83386.94	119424.9	101430.8	97056.55	67938.4
137900.2	122114.5	125140	177292.3	85756.56	107199.8	103449.4	111091.2	85452.29	87217.49
127371.3	93930.21	176616.3	190511	107127.3	148156.9	146215.2	168180.2	121647	125557.6

LN233.154	LN233.154	LN233.154	LN233.154	LN233.154	LN233.154	LN233.154	LN233.154	LN233.154	LN233.154
95071.17	111652.2	114315.8	84301.53	79382.25	52614.53	106052.7	66636.37	89478.95	97184.51
129853.7	96802.51	109342.4	90849.51	81203.37	53260.79	168535.8	58734.5	80648.14	57046.02
127424.9	78748.61	91648.89	129735.6	80811.01	49200.06	100476.6	51318.77	70113.72	82516.44
130765.5	94042.43	115315	139683.7	75012.34	72384.09	128356.8	64279.2	87923.6	73207.08
145693.9	127521.5	191005.5	159433.3	75883.36	98716.65	143099.6	90114.36	117336.2	88481.62

LN233.154	LN233.154	LN233.154	LN233.154	LN233.154	LN233.154	LN233.154	LN233.154	LN233.154	LN233.154
107494.7	63308.19	90839.76	115609.3	68440.61	95772.08	105533.5	122312.3	76817.39	113063.6
103738.1	76484.9	90639.33	89214.47	70222.91	103034.6	96678.43	98767.76	78420.7	95149.35
90315.77	76186.02	143756.8	85339.54	81088.81	139033.4	135657.7	123358.8	76993.6	106498.3
115910.5	80390.96	106177.6	90635.37	84543.5	147965.1	135542.3	121990.8	65572.01	107932.8
179902.5	92388.65	129252.2	165254.1	103129.6	141021.2	135467.8	127976.9	114790.1	138161

LN233.154	LN233.154	LN233.154	LN233.154	LN233.154	LN233.154	LN233.154	LN233.154	LN233.154	LN233.154
102126.8	129808.6	86525.47	123215.9	101334.8	77559.9	115124.4	52090.04	99362.89	70229.91
121640.1	82625.12	76805.17	114057.3	119226.4	58956.14	65017.41	69139.04	94566.04	76749.13
167324.3	79910.59	87158.86	105669.1	106057.4	79481.79	85243.32	51687.63	126274.8	112615.3
154608	125006.9	100285	111515.9	129743.1	76197.56	97347.31	69095.48	99156.74	83528.92
166490.9	173691.7	151861.4	133029.1	158617	73839.96	115621.9	122731.2	130799.4	106381.7

LN233.154	LN233.154	LN233.154	LN233.154	LN233.154	LN233.154	LN233.154	LN233.154	LN233.154	LN233.154
88572.77	71244.56	102957.2	67395.17	129399.5	76455	75097.49	123720.7	82352.12	80417.34
93553.71	71186.47	125645.6	126814.6	113863.7	63656.94	72350.35	137884.7	127670.6	93641.48
109018.2	85364.8	156712.7	92296.88	117770.4	79988.02	86031.43	121290.5	74218.15	49789.45
125036.2	112418	113438.8	88967.88	117514.9	102528.9	94985.81	140780.8	94182.05	54832.55
126174.1	110977.5	134103.9	121637.3	146371.2	106827.3	120783.7	141875.4	149637.5	71028.85

LN233.154	LN233.154	LN233.154	LN233.154	LN233.154	LN233.154	LN233.154	LN233.154	LN233.154	LN233.154
88195.46	74760.56	93295.26	88188.82	96724.43	76206.51	119990.7	111855.6	82248.48	99207
117689.2	73693.94	71582.76	107031.9	127526.5	89762.37	132438.2	96533.89	78719.23	120001.9
123173.6	82007.88	109365.3	76287.92	105297.2	126369.6	149912.6	101352.3	81121.97	93656.93
91987.53	122259.7	92528.87	91280.52	98879.29	85041.01	155247.8	92689.26	85803.49	102078
138629.5	109682.9	92359.57	144122.6	147105.2	96468.99	154096.2	116662.3	107405.4	123446.9

LN233.154	LN233.930	LN234.761	LN234.999	LN235.925	LN235.925	LN235.925	LN235.925	LN235.925	LN235.925
68293.43	44584.96	50759.11	16352.27	723009.5	172840.4	113746.9	171103.2	142400.9	124838.4
75702.06	46443.08	39543.46	17171.45	798732	149065.4	113433.1	170265.5	189314.5	111934.7
63747.62	56614.31	39401.71	16178.96	844292.8	148162.7	129515.4	157826.7	244761.3	89584.27
69987.23	71150.42	31025.37	15490.16	979569.6	105261.2	108405.5	160488.7	179022.5	79935.09
92499.84	68256.43	36657.33	18917.86	1072474	209022	77991.73	141165	208822.8	107760.7

LN235.925	LN235.925	LN235.925	LN235.925	LN235.925	LN235.925	LN235.925	LN235.925	LN235.925	LN235.925
137446.6	237059.1	175801.8	132775.9	108138.2	162859.4	68958.26	134254.5	141746.9	154071.8
154501.4	172755.9	137138.9	138456.7	138386.4	129357.9	72577.42	138498.8	213580.2	158253.9
114507.6	118289.6	125810.3	148970	143004.7	162821.6	74263.3	165044.5	179051.4	164265.4
139204.8	156525.7	118842.2	164852.8	166809	140390.6	46529.44	150277.4	151471.8	192221.7
160876.9	102920.5	178458.1	152107.8	134044.1	202226	97909.79	192093.3	134941.6	111754.3

LN235.925	LN235.925	LN235.925	LN235.925	LN235.925	LN235.925	LN235.925	LN235.925	LN235.925	LN235.925
234434.5	158043.4	179359.1	201003	203409.3	178079.5	247494.2	155719.5	63354.78	133604.6
159279.1	146221	156240.6	269030.9	185549.9	226975.3	163016.5	169527.5	74947.37	122140.6
184934.9	130596.7	174196.3	176359.9	204867.2	227613.4	153936.8	209838.5	69152.25	136497.3
185665.9	164211.2	149602.3	224145.7	189962.9	218162.9	179034.1	177699.3	73670.67	115066.5
163878.2	160489	138426.7	172201.1	179246	154178.3	258833.7	176861.8	78039.82	188091.6

LN235.925	LN235.925	LN235.925	LN235.925	LN235.925	LN235.925	LN235.925	LN235.925	LN235.925	LN235.925
201547.2	134186.3	131716.9	177633.1	156477	160328.3	110368.6	79151.19	226283	187269.5
145349	191206.1	150225.8	210585.7	202503.9	179294.2	150316.4	55366.67	139141.7	199929.4
162868.2	182713.4	178066.2	132233.4	149899.1	170317.6	177687.4	86477.3	187850.4	171997
157052	128240.3	187326.7	111456.3	173164.8	105900.8	101673	71630.5	256374.6	150673.1
139496.6	159214.3	147663.5	162735.2	139031.6	110174	157033.8	82137.05	150484.5	147305.8

LN235.925	LN235.925	LN235.925	LN235.925	LN235.925	LN235.925	LN235.925	LN235.925	LN235.925	LN235.925
181715.4	157531.2	202948.9	145299.5	163220.3	99546.55	262349.1	115919.1	155344.7	186355.1
220065.3	224310.9	147967.4	135833.8	158038.8	161274.8	156753.8	184169.5	144669.6	145434.2
213322	154394.5	130654.7	188625.7	201782.5	136243.9	179326.6	151317.3	172367.1	170923.2
160643.4	114530.5	180353.2	178885.5	166207.4	173450.1	125590	123725.1	116209.1	210977
205303.9	137616.9	128815.1	146444.6	221008.8	128050.7	171894.2	191969.1	152343.2	118227.2

LN235.925	LN235.925	LN235.925	LN235.925	LN235.925	LN235.925	LN235.925	LN235.925	LN235.925	LN235.925
216482.7	134831.6	141841.8	142503.3	186631.1	210127.5	179037	149204.8	158968.3	165886.3
212594.8	167245.7	105071.1	187680.3	127711.3	207822.4	172150.4	172386.7	151402.8	178772.9
246393.3	155601.3	134564.2	139715.3	114951.6	187038.6	144581.6	165214.9	163997.1	141138.4
175684.6	127112	143461.3	121832.6	129310.1	186897.5	202256.3	151137.4	113614.9	160351
222392.7	150821.5	153007.9	164158.9	172683.7	182557.7	192479.1	128450.7	134450.7	143701

LN235.925	LN235.925	LN235.925	LN235.925	LN235.925	LN235.925	LN235.925	LN235.925	LN235.925	LN235.925
189939.5	123768.8	142440.2	153178.8	135673.4	51628.65	116867.9	74269.88	195575	141179.9
138750.1	174595.2	174648.7	158155.4	201766.2	55536.03	144412.3	65730.8	172842.2	162702.1
188789.6	135706.8	198592.9	141437.3	155629	76671.17	189140.2	71243.71	239029.3	152000
133733.9	137556.8	153839.6	120229.5	196238.2	52743.61	225564.8	61875.22	211739.1	167334.1
173780	169795.8	146344.1	170211.1	164076	69742.19	131889.3	65492.23	136722	115736

LN235.925	LN235.925	LN235.925	LN235.925	LN235.925	LN235.925	LN235.925	LN235.925	LN235.925	LN235.925
181630.6	190603.1	196415.1	259994.3	185967.6	209331.9	163991.3	147804	170617.7	155018.2
166005.5	152245.5	184947.2	189227.7	157030.8	161543.5	153678.7	167796.7	169811.9	190230.9
160267.9	161668.4	228949.1	153693.2	174571	141186.7	233624.1	185912.9	163868.5	230355.6
177814.3	121966.1	151442.7	159643.6	160684.2	181769.5	114495.8	168429	164325.5	155720.6
283142.8	156542.4	142026.1	173890.1	148505.6	191626.7	163062.6	199858	137722.6	142889.9

LN235.925	LN235.925	LN235.925	LN235.925	LN235.925	LN235.925	LN235.925	LN236.928	LN236.978	LN236.978
150742.4	161452.4	156403	173580.8	181175.5	179350.4	175711.7	17653.58	49819.8	55420.62
175104.1	162566.1	154124.1	178655.4	187836	164230.7	128906.5	16454.33	42250.61	66364.97
180165.4	176402.1	155318	137112.1	185458.2	162561	151896.4	16658.59	45455.36	62128.76
159391.3	160284.4	171385.7	126110.4	201294.8	201326.3	177654.7	22228.17	44481.7	54161.55
170973.8	216305.2	171496.3	184431	183778	212318.5	116244.9	25202.44	45375.05	56284.31

LN236.978	LN236.978	LN236.978	LN236.979	LN236.979	LN236.979	LN236.978	LN236.978	LN236.979	LN236.979
37872.1	38772.27	55344.26	53735.87	29116.76	32792.17	34081.12	54623.04	30542.44	31160.56
30679.92	37234.93	51875.56	52078.2	27993.46	34622.27	35962.67	60459.39	33804.33	33261.5
32594.07	38213.51	53454.38	51477.83	28763.36	33761.21	37310.45	57176.88	30203.98	34898.09
31552.69	36431.62	52008.64	54237.64	28177.57	34670.06	34135	56783.76	30249.84	30241.88
33225.6	41528.17	56817.96	52267.39	27389.11	35982.62	35298.52	57694.01	32803.53	31333.12

LN236.978	LN236.978	LN236.979	LN236.979	LN236.978	LN236.978	LN236.979	LN236.979	LN236.978	LN238.915
35030.31	25757.39	32184.49	27565.26	46895.98	29598.64	35930.63	29899.62	20031.23	15196.73
40481.96	26566.58	33228.04	25574.08	49839.26	25848.66	40395.45	26675.32	20608.22	10878.03
36504.35	31134.54	28504.51	22861.95	52149.71	28152.48	37684.86	33172.87	20062.46	14144.92
38065.4	29906.68	31731.09	28448.54	44741.87	27844.23	38141.79	35312.91	21897.64	13874.97
40377.49	28486.44	29396.72	27215.43	48059.79	25658.19	35383.52	27638.1	22782.75	13619.67

LN238.931	LN238.931	LN238.931	LN238.931	LN238.931	LN238.931	LN238.931	LN238.931	LN238.931	LN238.931
134669.7	130156.9	90551.01	169190.6	184593.3	184912.6	229643.2	265644.7	215955	179768
102966.3	118451.1	85408.11	151465.7	125238.7	259550.6	169795.1	156744.6	186564	241867.9
128028.6	108370.2	87593.31	129720.6	143103.1	208239.7	177009.7	162694.2	185453.4	184005.7
101939.9	121740.8	80143.08	143340.9	146477.9	248788.6	161828.2	167487.1	173223.2	220019.5
128322.2	114038.5	84177.14	156970.7	150958.6	228267.9	194565.2	155875.5	291264.3	230168.2

LN238.931	LN238.931	LN238.931	LN238.931	LN238.931	LN238.931	LN238.931	LN238.931	LN238.931	LN238.931
210362.7	85482.1	147856	211573.3	158935.6	163782.7	164047.5	125914	164620.5	121142.4
210634.1	80034.74	160576.1	209140.9	151277.2	152655.6	158438.9	131225.3	149432.5	112922.1
304478	110941.8	123778	227037.4	165403.9	189237.4	131740.6	121779.6	206183.1	177234
195465	77917.42	147422.7	192862.5	185357.9	167729.4	161087.7	116468.3	142622.3	108197.8
209161.3	102512.6	133144.7	206911.5	170602.6	157164.2	211309.9	158903.9	175888.4	161729.3

LN238.931	LN238.931	LN238.931	LN238.931	LN238.931	LN238.931	LN238.931	LN238.931	LN238.931	LN238.931
187234.9	110100.1	189719.8	205738.5	76859.37	151164.4	167425.2	183707.2	147423.7	262846.8
190102	105356.3	193303.5	165012.8	113957	145919.2	171110.6	206076.2	154294.6	224208.3
213457.2	104161.9	219224.8	196226.8	113056.4	185175.7	179162.3	188730.1	172176.5	167641.6
165564.4	121125.6	180884.6	158577.3	102936.7	149451.2	168385.7	188617.7	150090.3	207836.2
246669.1	128178.7	244742.3	183895.9	99490.52	170895.8	181613.3	178136.8	176378.5	233693.2

LN238.931	LN238.931	LN238.931	LN238.931	LN238.931	LN238.931	LN238.931	LN238.931	LN238.931	LN238.931
206586.7	145255.8	180817.7	83178.95	164770.6	207961	179988	170772.8	187428.3	191645.5
168106.9	134684.7	182913.1	92718.02	164819.4	182699.9	182363	155692.6	214716.6	165475.5
182701.2	138713.5	185331.9	72563.7	169821.4	179018	174879.5	150690.9	231214.7	180779.6
158579.6	149001.1	170575.7	79935.02	168617.6	181700.2	176198.4	151578.5	206832.7	165732.5
162521.5	153292.2	174130.3	129997.4	227238	165427.2	185420.3	203418.5	213641.4	160177.3

LN238.931	LN238.931	LN238.931	LN238.931	LN238.931	LN238.931	LN238.931	LN238.931	LN238.931	LN238.931
287335.6	134282.1	87353.97	132444.5	160062.1	109993.5	119285.8	164024.9	105636.4	131630.4
180134.5	136983.2	94117.59	127452.7	156781.2	112256.1	93527.6	164975.8	100453.4	123778
171561.7	166515.6	140633.2	157163.1	168230.9	108946.5	92986.65	147963.8	111431.1	134536.6
186338.2	134032	100992.4	123683.5	147171.2	112460.3	96663.26	172761.7	111397.3	116927.6
175770.3	129886.2	96290.63	117963.7	129659.3	112261.9	86214.15	150179.2	125988.3	119906.5

LN238.931	LN238.931	LN238.931	LN238.931	LN238.931	LN238.931	LN238.931	LN238.931	LN238.931	LN238.931
167622.5	89167.66	137763.2	149103.5	86737.62	101003.7	151134.2	94737.37	206921.7	131442.1
106706.5	87223.17	137653.5	90741.99	140599.6	116414.2	153034.5	99240.05	203054.7	119779.5
110334	100237.1	139539.8	83043.15	89779.03	144136.5	194100.3	95439.36	189739	154460.4
101592.9	93070.7	128204.9	97602.46	89351.47	113932.8	143108.6	94749.91	210803.4	125039.1
104374.6	105401	152028.9	94968.22	76557.99	107015.9	162603.5	90383.22	205669	116813.8

LN238.931	LN238.931	LN238.931	LN238.931	LN238.931	LN238.931	LN238.931	LN238.931	LN238.931	LN238.931
109840	103675.2	111976.1	105638.9	142304.3	91768.91	54243.17	56714.39	40652.05	28954.06
112221.8	105197.1	125307.3	106225.5	142904.7	95551.21	57570	55674.46	37004.35	30819.88
107060.4	131948.3	131794.4	104846.2	135814.8	91855.42	60339.27	52410.71	41506.72	29240.71
114983.7	98799.76	115420.4	109427.9	145907.6	87954.29	52439.2	50541.89	37129.48	27031.14
101540.3	79901.98	111603	99590.93	137088.3	90359.85	54154.12	49324.36	36925.13	39965.93

LN238.931	LN238.931	LN238.931	LN238.931	LN238.931	LN238.931	LN238.931	LN238.994	LN238.994	LN238.994
45824.82	49759.71	39682.05	25242.38	34036.43	53484.04	14187.8	42356.3	63406.09	42334.7
42291.55	48036.03	41320.1	25282.28	34489.04	54826.77	14633.11	48951.1	80090.36	40772.39
39252.09	58593.89	36490.93	27551.58	31000.55	53373.25	13794.33	44732.62	60138.4	41813.3
37858.38	49742.9	37230.92	24655.53	27555.39	47940.07	12313.06	48348.35	61510.78	43288.92
37000.59	41809.2	39924.82	23929.65	27255.98	49727.5	11239.16	46476.94	59635.74	40926.72

LN238.994	LN238.994	LN238.994	LN238.994	LN238.994	LN238.994	LN238.994	LN238.994	LN238.994	LN239.059
41583.18	36668.43	52486.49	50234.43	30279.39	46807.17	39534.74	41147.1	46405.9	25406.76
45840.31	41846.48	46435.73	48593.54	27134.21	44495.28	35045.07	38667.94	47759.59	29757.34
40017.42	39063.79	48520.1	46298.92	30400.89	44097.77	36387.47	46165.89	41278.79	42574.97
43030.2	35447.73	44125.49	48957.47	26867.7	46421.29	36490.44	37441.74	48294.62	47367.25
44707.25	36744.72	48285.3	51442.94	26226.53	43120.09	33488.75	41627.6	43010.14	47960.95

LN239.059	LN239.059	LN239.059	LN239.059	LN239.059	LN239.059	LN239.059	LN239.059	LN239.059	LN239.059
21846.38	26833.77	17087.47	13217.97	12552.62	9140.011	16052.48	17848.85	11528.59	13051.24
26776.59	32185.01	17509.31	13786.43	12212.63	10407.41	17158.02	19125.36	12851.24	11860.02
38042.6	46975.28	21015.79	19006.69	16148.56	14880.59	20926.21	16794.25	12043.71	14073.57
43764.38	50705.06	23342.03	18946.6	19648.73	17062.79	23975.23	17341.43	11680.16	14601.89
45891.13	56024.6	24646.52	19957.21	19822.12	17403.84	23905.05	15086.68	12878.62	14609.38

LN239.059	LN239.059	LN239.059	LN239.059	LN239.059	LN239.059	LN239.076	LN239.076	LN239.076	LN239.076
14967.47	17234.67	17039.37	24386.66	16298.31	15198.89	17157.19	16587.32	23885.01	19867.93
15321.38	16152.57	15474.8	28240.97	16195.17	15946.33	19073.38	17152.64	18396.75	16919.62
16034.84	18093.93	17434.76	32039.33	20320.92	13774.24	19497.15	15997.39	19380.27	18272.16
16946.62	19596.33	17188.11	33613.98	24781.38	14542.21	20164.04	15181.59	19199.77	19263.61
16817.49	18425.62	15079.69	36721.26	26406.23	14236.21	19224.69	15416.03	19594.81	17890.57

LN239.076	LN239.076	LN239.076	LN239.902	LN239.949	LN240.910	LN240.927	LN240.928	LN240.928	LN240.928
18375.64	14584.98	16365.72	20281.42	18891.28	302903	57516.76	67246.69	79951.61	58757.24
17233.37	15704.67	15371.19	16483.66	19041.94	263888	59597.62	65602.04	68127.73	57561.57
18257.6	15884.47	14653.32	20028.92	18105.16	236748.8	61964.06	74398.36	74802.26	61709.78
18950.32	14592.29	14959.79	19575.9	20304.36	397606.5	58580.42	64205.62	70391.56	57214.78
18335.26	15289.12	14081.77	22415.25	21269.27	366994.3	55460.98	61673	71025.93	60324.52

LN240.928	LN240.928	LN240.928	LN240.928	LN240.928	LN240.928	LN240.928	LN240.928	LN240.928	LN240.928
57348.44	49101.73	58938.75	58145.36	49651.8	47661.4	46104.01	32748.56	39799.88	57135.29
48683.58	45639.51	63725.03	62350	54325.35	40327.51	49756.51	31217.78	37329.82	57630.2
51536.85	46777.22	61176.28	57533.16	46474.29	48950.37	43836.28	32677.29	34969.51	55013.17
52746.43	48814.57	63360.43	61871.48	47784.49	41286.69	46097.63	33365.42	32661.81	52333.38
53683.55	43421.99	56576.28	58561.95	49333.95	47400.82	46130.88	29928.02	32911.48	55619.3

LN240.928	LN240.928	LN240.929	LN240.928	LN240.928	LN240.928	LN240.928	LN240.928	LN240.928	LN241.01_
68709.28	58139.58	64573.83	48796.15	61018.29	47215.31	47958.22	77879.29	42202.81	47115.25
65743.36	52479.05	64719.31	48219.28	67772.3	47222.94	45823.46	78365.68	47684.01	101090.2
66943.49	58644.46	65337.27	48212.5	61943.15	45275.87	49195.57	78437.96	45678.67	49120.92
61540.26	57789.17	64808.12	46524.69	61223.53	47306.25	45873.08	74087.06	41557.32	54693.98
67705.59	52048.43	60417.38	53241.72	58929.18	45630.94	46965.55	75143.16	41769.99	45098.68

LN241.01_	LN241.01_	LN241.010	LN241.010	LN241.010	LN241.010	LN241.010	LN241.010	LN241.010	LN241.010
64651.97	37093.83	62650.08	67566.15	47580.94	54871	54584.9	42014.73	62912.6	48507.08
40961.95	41018.61	59408.84	63243.8	47489.4	61049.14	72312.54	42023.42	69159.71	53920.37
60322.88	31330.56	57029.55	74416.53	48047.97	62694.2	52712.38	47331.08	72574.34	54535.95
66734.89	32323.66	56830.7	60937.86	47121.55	62426.03	54920.83	43207.84	63485.29	52745.38
63156.67	37366.54	53013.09	71648.98	50849.54	53841.76	52745.99	47533.37	72737.01	53372.55

LN241.009	LN241.010	LN241.010	LN241.01_	LN241.010	LN241.010	LN241.009	LN241.010	LN241.010	LN241.01_
43599.28	46664.35	41687.56	50365.29	51048.78	36565.58	36445.88	29931.94	23018.96	39456.59
47707.94	57694.61	48703.04	35047.77	50926.04	34172.74	38088.11	34091.38	32369.97	39130.62
44858.21	55240.97	44616.59	53360.04	49172.84	35288.79	34790.39	31181.29	27069.13	40595.62
45738.34	57850.84	46739.53	49835.34	48643.63	36086.16	37257.33	30889.6	26304.68	41539.08
42569.68	51511.86	44757.28	47188.08	46085.95	35043.85	33108.54	31224.32	24193.38	42333.93

LN241.010	LN241.01_	LN241.01_	LN241.01_	LN241.010	LN241.010	LN241.010	LN241.010	LN241.010	LN241.009
40305.13	44259.12	40649.95	34854.53	38894.9	31420.56	28365.09	29923.63	36777.77	48909.49
45152.58	40566.58	37403.45	34413.99	38616.54	30364.4	32040.12	31108.35	36550.4	58294.6
41694.85	39320.53	37745.75	34091.89	35862.89	34517.88	30571.74	31313.55	39531.15	42831.77
38300.73	41173.42	39650.31	35730.39	38780.43	31820.93	32562.4	27377.19	37224.36	45613.11
38425.15	41783.96	42576.72	37071.23	39564.7	32513.13	29143.64	30494.64	35129.3	53962.8

LN241.010	LN241.010	LN241.010	LN241.010	LN241.010	LN241.010	LN241.010	LN241.010	LN241.010	LN241.010
14836.47	22190.04	22660.79	15618.18	24943.66	28797.81	23733.09	32529.28	30081.19	15188.54
19050.68	20026.75	22978.86	15633.45	28621.67	26710.51	24944.91	33948.76	24969	15712.7
16388.93	20931.03	20022.87	12167.22	21599.67	24437.19	23053.41	31027.48	27945.73	16125.01
17474.08	20220.01	20802.58	13365.7	24798.49	29471.99	24084.89	34532.77	25944.13	16165.73
18221.43	18087.3	19899.78	14250.69	23169.56	27850.8	23560.16	27414.65	25892.08	14821.82

LN241.010	LN241.010	LN241.010	LN241.010	LN241.010	LN241.010	LN241.010	LN241.010	LN241.010	LN241.010
16520.99	36145.2	35203.87	35809.77	19426.61	14037.08	24937.32	17976.59	24531.6	14076.98
17046.7	36650.59	38617.45	31816.1	22735.84	13430.71	26894.65	19547.04	28250.33	14003.06
17769.55	33819.08	37824.54	36533.38	19951.82	12601.28	24964.89	19337.96	24326.42	12493.02
17187.88	39052.79	35048.76	32400.61	19593.53	13532.13	21847.18	18491.34	27614.82	12420.1
18291.23	34277.05	35794.05	35151.32	18845.14	12890.74	24949.74	18123.11	25944.51	12420.22

LN241.010	LN241.010	LN241.010	LN241.010	LN241.010	LN241.010	LN241.010	LN241.010	LN241.010	LN241.010
21293.97	30418.91	25159.93	12613.83	29875.67	17452.88	23226.06	24090.58	33672.64	23121.28
21535.72	31375.65	26808.31	13572.08	29682.44	16224.44	21661.27	23245.12	29974.19	25288.65
20455.33	31722.05	25472.9	12619.3	30475.81	13389.64	23264.35	23773.26	29106.62	24026.7
19696.26	31352.84	25455.09	12360.96	30709.94	13623.83	23002.72	21612.72	29280.6	23153.5
19342.36	30046.32	24874.36	12987.72	32123.02	14120.39	22914.4	22884.91	26176.23	24377.64

LN241.010	LN241.009	LN241.010	LN241.010	LN241.010	LN241.010	LN241.010	LN241.010	LN241.010	LN241.010
26116.73	64138.22	28733.97	33866.67	16231.58	35023.92	13365.22	32011.82	19281.35	21420.41
27485.04	68150.41	31696.9	34206.99	15325.6	36671.83	15589.7	33197.75	17022.81	23538.96
26262.28	64881.99	29231.86	34286.25	15957.97	34768.91	14077.66	32523.54	19860.5	20681.46
26751.27	67116.5	28907.74	34114.25	15081.83	40340.83	14979.19	35862.3	19918.43	20810.05
24030.24	62443.5	28813.29	33697.84	16466.04	34137.04	12773.06	32962.06	21338.26	23319.1

LN241.010	LN241.009	LN241.010	LN241.010	LN241.010	LN241.010	LN241.010	LN241.010	LN241.010	LN241.010
35142.44	26027.76	51445.02	20414.85	20417.67	22090.1	14427.6	15006.36	21152.04	12679.79
31553.55	24408.62	52383.3	21442.1	21850.65	21766.45	17862.16	15737.84	23843.6	12843.62
36085.71	22540.69	49980.26	17443.22	22366.79	20532.14	16394.68	15132.17	20518.43	13896.09
32391.77	25484.64	47328.78	18718.41	20310.38	21027.82	15864	13650.68	20868.07	10969.17
33810.92	24697.22	51482.31	20063.33	21370.35	22394.02	15269.56	15284.77	22159.97	11460.43

LN241.010	LN241.010	LN241.056	LN241.056	LN241.056	LN241.056	LN241.056	LN241.910	LN242.887	LN242.942
29483.93	27898.84	17033.29	18382.7	18905.19	16234.28	18322.86	19999.07	32642.17	365205.4
28377.68	24351.22	18986.8	18286.25	18428.2	14355.9	16487.76	20191.66	130268.4	370018.4
29523.53	28793.49	16453.96	19074.82	18034.61	16546.35	17438.13	20551.61	132663.2	372226.2
29015.96	27560.59	17196.21	18629.43	16456.18	14813.46	18180.39	14297.74	13697.18	354944.7
29955.76	27372.38	17560.7	17177.27	16658.56	15743.43	16554.21	31956.71	107193.9	361007.6

LN242.943	LN242.943	LN242.943	LN242.943	LN242.943	LN242.943	LN242.943	LN242.943	LN242.943	LN242.943
481601	380847.7	509470.4	509267.6	267439.7	484429.5	309553.7	261600.5	267720.9	480771.6
455990.8	342526.5	602464.1	462436	260406.3	528830.5	307013.3	264331.1	249484.7	452317.1
602178.3	396437.1	470805.8	448845.3	294177.3	421919.3	545604.9	309642	256232.7	556012.1
432681.4	363263	408776.3	510232.8	234706	387849.2	420220.2	305097.1	272163.7	614852.9
502272.9	329942.4	543038.4	431767	240109	325572.6	320959	272147	266650	540491.6

LN242.943	LN242.943	LN242.943	LN242.943	LN242.943	LN242.943	LN242.943	LN242.943	LN242.943	LN242.943
374207.7	371406.9	494922.3	374189.3	321687.4	371462.6	429114	516811.7	348626.2	361550.5
413749.3	422635.3	774448.2	212001	228607.6	382344.3	500402	576399.1	399800.6	386524.3
370935.2	408959.7	400568.5	217314.6	246369.6	470415.6	335826.4	322989.1	351050.6	550355.9
512291.3	524013.4	525281.7	290113.3	306747.8	380108.4	482952.2	622522.7	346516.3	326633.1
422777.2	432571.9	524925.1	226275.8	286652.7	554943.7	481180.1	566019.7	288648.3	447762.8

LN242.943	LN242.943	LN242.943	LN242.943	LN242.943	LN242.943	LN242.943	LN242.943	LN242.943	LN242.943
221058.9	428418.3	365272.9	291356.8	462678.7	577404.7	414061.7	566075	294041.2	433557.1
377176.2	407145.4	337557.1	269305.5	412813.5	548263.5	288968.5	417386.1	481395.3	457454.3
274072.8	375924.6	360252.6	229638.8	475026.2	486737.3	326151.8	501476.3	395681.9	580369.2
177429.3	371473.5	406422.2	248664.8	507059	433927.4	470105	452633.5	460474.6	289813.6
202481.9	435254.5	379247.1	346138.2	342729.1	604995.7	457267.8	514144.2	429524.9	346673.6

LN242.943	LN242.943	LN242.943	LN242.943	LN242.943	LN242.943	LN242.943	LN242.943	LN242.943	LN242.943
354598.6	365525.7	594579.5	70156.52	373333.8	387958.1	314544	510757.9	533716.8	449873.5
325949.5	340588.6	343111.8	94585.3	416651.8	499586.4	332104.8	361192.6	435814.1	456826
331353.3	372419.7	364096.5	99057.13	609407	662585.8	347730.4	462611.3	471279.4	341638.3
286068.5	363862.2	400496.2	93590.44	469237.6	452103.6	313056.4	425070.6	435832.3	505463.4
368785.7	355734.5	407819.9	108480.9	408221.8	423353.2	325973.8	399351.4	380622.6	445779.5

LN242.943	LN242.943	LN242.943	LN242.943	LN242.943	LN242.943	LN242.943	LN242.943	LN242.943	LN242.943
336388.6	354925.7	590100.2	346176.5	311387.5	540553.2	280074.7	385637.2	349225.1	287400.3
419270.4	435375.6	423859.1	443277.8	352584	403932.6	474686.9	483208.7	382290.6	283892.6
172746.7	437057.9	646990.9	407912.2	328416.3	447651.5	247132.9	532454.4	422379.3	304373.7
213307.8	512219	460938.2	374707.6	303648.3	593842.4	256738	499138.2	427960.3	318425.9
249096.5	442521.8	392937.1	509368.1	299623.9	554587.8	293629.2	385311.3	342601.3	291786.5

LN242.943	LN242.943	LN242.943	LN242.943	LN242.943	LN242.943	LN242.943	LN242.943	LN242.943	LN242.943
367498.6	428088	530601.3	112092.7	680052	469034.3	467208.6	401834.7	114200.3	356146.1
295380.7	476758.9	472574.3	157758.5	453415.9	392732.2	475143.5	377994.3	115142.5	318561.6
303588.9	311100.4	469536	108848.1	355117.2	479411.5	434358	396018.6	85960.74	273907.8
252237.1	321364.6	413117.5	146913.1	485396.9	535398.7	609324.9	467086.7	105843	245563.3
326346.6	323558.9	321524	93241.34	440652.5	316175.7	440245	587262.8	118704.2	317533.8

LN242.943	LN242.943	LN242.943	LN242.943	LN242.943	LN242.943	LN242.943	LN242.943	LN242.943	LN242.943
469796.9	350478.1	365992.4	244728.9	234391.6	211338.9	136408.2	352035.8	142365.9	406849.1
442919.5	201267.9	369002.7	282787.6	402288.8	203466.8	65952.75	443729.8	101160.1	394848.6
393317.5	292957.7	438388.4	267435.3	343364	268035.2	70601.51	456724.4	110102.5	367402.8
612169	271352.1	497518.2	356838.8	234978.4	227615.7	124883.7	433780.5	121690.9	413892
459664.3	328018.2	334622.5	235512.3	264562.7	259289.1	80988.13	345926.9	93599.32	454390.4

LN242.943	LN242.943	LN242.943	LN242.943	LN242.943	LN242.943	LN242.943	LN242.943	LN242.943	LN242.943
474685.7	306063.7	621750.7	410582.9	632186.2	450716.8	437624.6	402132.4	487828.1	414368.1
437475	324914.4	436866.1	419648.2	376833.1	360038	625118.4	532335.1	608897.9	349714.8
560491.3	461235.8	610685.8	331352.1	402053.4	494227	494553.2	472809.2	507755.7	449023.9
727808.8	306216.8	426820	416516.8	384573.1	585163.7	444988.5	466521.1	450523.7	470403.8
536801.5	294686.9	490266.8	389264.8	446269.1	397124.4	464951.6	367549.5	474044	480444.1

LN242.943	LN242.943	LN242.943	LN242.943	LN242.985	LN242.986	LN242.986	LN242.986	LN242.985	LN242.985
83277.61	327568.9	335885.7	495234.3	49479.2	25794.81	18168	19777.97	22399.69	25967.69
88533	292492.7	534904.3	613818.3	38414.6	26347.28	17817.9	19766.82	24639.82	25256.6
86490.53	304204.6	621600.9	488715.2	44522.36	24755.99	21145.91	18123.34	24192.01	28305.94
75145.75	327529.7	442674.3	445533.9	43752.34	26177.82	19262.57	19510.56	18807.72	27905.07
72322.27	356686.8	509835.7	688797.6	46383.63	22964.17	18273.45	18773.08	21660.01	26778.04

LN242.985	LN242.985	LN242.985	LN242.985	LN242.986	LN242.985	LN244.984	LN244.984	LN244.984	LN244.985
37161.29	28324.4	27558.53	37771.01	13079.41	73002.65	12338.59	33819.29	59617.28	42939.51
38740.98	27147.93	30211.77	39227.53	12768.92	73665.33	14527.12	55346.68	71538.13	53178.42
38859.05	25465.38	31987.15	35615.95	12540.69	72754.44	11875.83	33769.28	68619.22	44797.6
39374.65	28103.13	27709.93	37750.86	12374.4	71834.27	10339.62	35397.52	65674.04	36702.92
38567.47	23911.6	33339.92	33709.92	12599.3	75333.97	16239.54	48266.89	63602.65	55923.96

LN244.984	LN244.984	LN244.985	LN244.984	LN244.985	LN244.984	LN244.985	LN244.985	LN244.985	LN244.984
47817.04	54943.08	46134.55	51245.14	48582.06	86516.23	55973.81	15241.54	55547.56	98783.62
72067.2	42178.4	39697.45	63740.04	33243.23	65583.11	54466.35	19096.48	36541.91	70936.26
62545.64	38866.4	47239.32	61588.32	41012.08	53334.74	50236.52	19866.9	39325.16	64629.53
60517.61	29494.03	37906.38	67417.66	24629.1	44558.85	67396.38	16610.91	64428.11	68851.26
61573.72	45995.12	46874.14	68181.87	33010.27	66504.42	42311.44	22007.93	45676.88	64462.47

LN244.985	LN244.985	LN244.985	LN244.984	LN244.984	LN244.985	LN244.984	LN244.985	LN244.984	LN244.985
29076.98	47923.28	60710.7	86859.38	66308.26	23733.46	94210.06	70232.13	41959.61	39870.47
26837.2	50744.35	67582.87	71849.7	83006.11	24739.25	87024.79	71991.52	51023.3	47202.09
28360.36	43238.79	52812.22	67647.44	59323.21	26316.78	75567.75	60813.32	48948.66	63637.25
22365.16	35042.69	50114.65	104869.8	61268.04	20636.31	77718.84	51030.9	63144.82	45651.16
29714.01	39064.93	73549.21	66963.9	74183.85	24637.97	81322.36	69962.23	48978.39	42583.22

LN244.985	LN244.985	LN244.984	LN244.984	LN244.984	LN244.984	LN244.984	LN244.984	LN244.985	LN244.985
38495.08	17591.08	66812.61	77510.31	62682.37	39902.37	87097.61	50237.94	22662.86	44921.16
43201.01	25690.57	65634.08	119537.5	86434.23	48907.39	104695.6	51068.03	18526.75	55773.97
40934.76	20094.66	68071.81	64448.4	81327.98	36123.23	84869.11	58746.9	18959.09	45223.87
46071.55	31934.78	64844.37	79827.41	104620.9	40256.03	99574.11	53889.03	18222.34	51906.41
47154.75	18461.54	60237.38	84997.97	73940.07	39257.44	89191.21	43333.29	23585.45	46357.6

LN244.984	LN244.985	LN244.984	LN244.984	LN244.985	LN244.985	LN244.985	LN244.984	LN244.985	LN244.984
81472.69	82559.88	49314.71	70117.15	35072.38	37223.79	25346.27	50177.77	20971.5	97473.03
68689.56	56637.87	69326.69	71653.43	35661.17	43593.87	31390.05	70431.14	27117.29	110434.4
64604.01	60081.07	56761.05	75595.97	37117.81	39083.04	30881.67	56047.35	20440.09	97731.99
63364.29	65802.65	73299.13	105924.2	35309.71	47455.45	46351.21	82236.97	23346.23	96316.87
49304.67	46171.82	57745.82	58441.91	39208.67	33965.12	37863.88	53723.99	28303.94	84078.34

LN244.985	LN244.985	LN244.984	LN244.985	LN244.985	LN244.985	LN244.985	LN244.984	LN244.984	LN244.984
55545.41	41422.08	80363.93	48358.98	35341.77	22629.79	42985.54	61483.87	51122.71	49444.85
40302.74	42486.54	74448.66	47484.19	33531.98	25308.38	37183.82	37413.91	73036.1	48140.71
40763.14	49717.37	92039.21	47029.09	35574.01	21219.01	43365.72	38379.23	61368.99	52902.78
38219.69	48374.77	79741.69	61779.5	31593.34	21729.4	50231.54	40893.88	64787.5	43350.26
41333.48	52020.35	94029.44	76210.34	41523.92	28325.93	36135.55	39600.98	51466.55	57688.26

LN244.985	LN244.985	LN244.985	LN244.985	LN244.985	LN244.984	LN244.985	LN244.985	LN244.985	LN244.984
33717.99	50692.53	54773.54	18630.28	28923.99	58158.29	56845.17	35953.71	50655.24	59998.89
55302.96	55273.39	57477.99	27430.54	19048.91	51237.87	70142.15	31141.54	44918.26	68766.67
35228.1	58654.14	48658.89	21338.33	21708.47	46119.98	52375.55	30061.26	34058.31	67752.11
36076.54	45741.17	64767.27	17552.17	18848.55	48745.87	54737.24	27785.79	28186.52	115276.3
37951.41	55127.8	58379.13	18810.09	21814.15	43156.37	50095.57	36954.9	31760.69	74888.26

LN244.985	LN244.985	LN244.985	LN244.985	LN244.984	LN244.984	LN244.985	LN244.984	LN244.984	LN244.985
25097.94	61765.87	43255.3	33338.83	43519.61	43187.47	28372.43	88614.04	52241.13	47441.12
24774.53	44081.7	55635.13	40438.27	67666.57	54323.11	42005.58	83384.97	37481.13	64154.45
23405.58	60825.31	41495.56	36901.58	42349.68	44566.09	40398.11	74664.04	43521.69	50916.37
22022.61	48349.77	47073.13	34512.46	42482.46	32024.71	41448.58	84674.66	44060.8	55524.19
25706.51	60758.45	47124.72	30576.84	48560.74	60836.17	36820.31	76085.15	41114.35	47251.41

LN244.985	LN244.984	LN244.984	LN244.984	LN244.984	LN244.985	LN244.985	LN244.984	LN244.984	LN244.985
45317.11	43257.99	76475.6	97097.94	63612.95	39349.78	67816.46	83695.47	124878.7	18008.33
34730.48	53990.11	56124.25	53438.62	77249.87	28046.09	76030.96	98163.47	69284.22	13102.6
39111.12	36741.87	64032.09	58118.05	71156.47	39419.49	67846.45	81865.38	84706.49	15040.41
32443.33	37750.2	70317	58326.72	62589.26	54496.49	58949	82301.67	98756.63	15064.84
29908.73	49671.77	62218.9	54971.42	65557.06	38914.59	60998.68	98024.83	86659.89	15603.9

LN244.985	LN244.984	LN245.983	LN246.886	LN246.965	LN246.965	LN247.000	LN247.000	LN247.000	LN247.000
42549.88	74184.11	42126.52	10197.77	11951.04	20376.48	41685.77	15463.75	27379.6	19575.58
42434.83	74921.29	38224.07	10123.14	12445.08	18466.15	46208.06	19968.42	24837.5	29510.54
46256.22	70321.28	42735.61	10042.21	11876.8	18613.28	37283.24	18602.92	29183.49	26600.74
48244.75	82289.41	42401.72	13385.99	11387.31	16326.75	53843.41	19584.83	23185.43	26320.6
57861.02	62371.7	44653.62	12285.37	11298.93	18936.76	34778.5	21485.11	22062.72	25447.47

LN247.000	LN247.000	LN246.999	LN247.000	LN247.000	LN247.000	LN247.000	LN247.000	LN247_12.	LN247_12.
36308	13836.76	50703.38	46514.2	33779.47	50828.53	24151.65	40706.63	65632.99	58649.51
43202.55	11202.93	44901.48	44295.33	20621.26	40696.87	25517.28	45803.82	67373.28	96352.14
35000.06	12816.28	49352.01	41131.14	28633.5	61640.34	24139.86	40439.89	65505.82	45182.72
33980.95	14804.39	59228.12	29643.33	22495.17	60016.08	24887.3	41583.52	69365.5	47962.17
46514.62	13835.33	89030.82	50689.72	29692.95	48783.18	22357.79	63227.51	54516.49	43010.5

LN247.000	LN247.000	LN247.000	LN246.999	LN247.000	LN247.000	LN247.000	LN247.000	LN247_12.	LN247.000
34067.29	19900.35	49177.6	52165.06	34043.09	33664.48	45417.2	44045.24	56009.37	60456.68
34648.79	23842.23	47681.77	60724.61	22095.43	62008	35017.38	39738.82	45548.14	58144.96
31286.01	17508.9	53847.46	54057.1	27331.77	39019.51	35262.73	37984.27	69212.39	46871.02
35304.02	12873.59	52723.23	81826.99	25878.5	38388.93	31852.87	38401.31	51385.53	40816.71
26186.17	17510.43	36439.78	58191.63	27889.27	40190.37	39122.92	31439.34	72940.96	41120.97

LN247.000	LN247.000	LN247.000	LN247.000	LN247.000	LN247.000	LN247.000	LN247.000	LN247.000	LN247.000
41759.27	35768.32	52430.12	29939.89	33588.81	22148.63	43867.37	43240.57	41330.47	16876.17
54374.46	26593.38	47213.1	16942.81	35182.57	30343.34	35766.47	44236.52	57602.37	21955.37
54225.11	30787.78	40562.84	18763.88	39728.7	24835.92	27204.11	43914.24	50251.29	18083.77
51116.14	29041.07	59122.69	17367.68	37428.89	26611.76	27244.64	40449.68	50577.29	16476.64
52408.78	26416	36927.75	17120.26	37188.87	28755.11	28179.26	38172.16	49454.89	14713.54

LN247.000	LN246.999	LN247.000	LN247.000	LN247.000	LN247.000	LN247.000	LN247.000	LN247.000	LN247.000
38116.72	52960.29	38190.12	35898.13	38374.62	31632.54	52675.38	16167.24	67069.53	26685.34
53212.56	38893.21	33154.26	27478.17	34135.56	45383.31	74382	18626.93	43880.19	42597.21
48591.64	48532.34	39646.13	23716.62	45062.44	44051.36	67668.21	18162	36440.82	29362.26
39152.41	41854.77	51037.75	35393.05	47973.37	54672.84	46932.89	14537.09	43492.12	33828.34
55178.66	63492.98	31809.87	26351.43	54516.05	47485.06	45428.52	18607.72	52866.62	29275.92

LN246.999	LN247.000	LN247.000	LN247.000	LN247.000	LN247.000	LN247.000	LN247.000	LN247.000	LN247.000
143825.2	44225.61	32348.38	36967.7	17876.31	52185.15	30828.25	64615.94	30053.64	28098.17
59806.06	51230.19	31654.48	30066.93	25182.61	36245.8	23220.14	55506.05	38095.17	29653.45
53177.68	47780.19	25706.68	34996.57	23492.53	46699.64	29015.31	67510.38	44347.17	28611.42
76212.13	46391.22	27362.78	32975.61	21169.74	53139.01	31064.12	46475.88	33137.24	31368.39
57655.68	47937.98	24137.09	29252.96	24796.87	54406.72	30353.69	53352.08	36320.29	37964.95

LN247.000	LN247.000	LN247.000	LN247_12.	LN247.000	LN247.000	LN247.000	LN247.000	LN247.000	LN247.000
16624.52	34371.74	37340.84	45289.35	51399.01	28292.79	26863.75	25222.12	42663.61	30814.62
22123.62	36327.83	39144.77	58860.42	53413.56	25446.55	23672.2	41577.81	41765.2	23169.26
18253.21	31130.49	44888.09	60432.04	51112.57	25958.19	23606.99	28644.28	44651.15	32798.41
14805.63	25553.71	31191.42	55238.1	59131.63	24423.29	27330.68	23720.64	55794.99	24998.16
15345.03	36035.99	40728.68	76588.92	44019.02	34605.18	31943.27	28425.57	38954.55	33449.29

LN247.000	LN247.000	LN247.000	LN247.000	LN247.000	LN247.000	LN247.000	LN247.000	LN247_12	LN247.000
16808.8	19217.08	37576.82	30914.75	51817.76	30589.02	23416.88	20537.47	55511.31	25996.3
16795.01	24315.76	51826.52	26694.51	47931.98	28866.82	19272.32	25014.06	81361.26	24199.77
19882.08	16843.27	44018.57	33615.4	44645.35	33635.04	30518.92	21263.44	53611.86	31311.31
20170.02	19989.05	32412.49	32086.19	55590.57	27477.63	27222.77	23314.83	59679.39	24598.4
13865.4	19465.02	43857.17	31053.66	46838.34	29754.01	22230.49	21745	51183.13	34493.67

LN247.000	LN247.000	LN247.000	LN247.000	LN247.000	LN247.000	LN247.000	LN247.000	LN248.886	LN248.960
30055.55	31336.5	26329.09	40134.82	36535.94	36050.07	36047.51	57720.61	44526.53	725114.8
42303.61	29707.67	48322.99	38288.22	37757.67	24688.21	40233.36	50968.17	55872.99	1120123
34463.99	32589.07	30056.2	38727.45	37440.19	26310.4	38990.59	40297.37	58344.77	796463.6
33851.63	52096.35	47451.87	35780.55	22077.92	31758.48	25646.09	35219.65	68819.25	823114.7
31041.54	37642.85	30591.28	40200.55	42867.93	27756.49	33638.54	40668.25	61774.95	968793.4

LN248.960	LN248.960	LN248.960	LN248.960	LN248.960	LN248.960	LN248.960	LN248.960	LN248.960	LN248.960
1073746	768045.7	1065655	369310.2	1110881	890549.7	570041.4	462561.8	1235020	1013215
650958.5	892132.9	936020.1	509873.8	898267.7	781112.4	565841.8	429508.8	1587222	1328751
817897.9	891590.8	910418.3	505987.1	951063.2	683321	639365.5	405135.8	1430627	1095627
753374.1	725903.3	1730491	563497.1	1184905	498710.2	569005.5	332715.9	1301986	921377.7
1058804	676137.1	1272617	578744.1	1290305	603523.9	561566.1	273505.3	1395350	806431.6

LN248.960	LN248.960	LN248.960	LN248.960	LN248.960	LN248.960	LN248.960	LN248.960	LN248.960	LN248.960
421841.8	529524.1	844377.3	1460494	872558.6	1151757	1182656	1591796	1419921	276635
408839.8	606892.7	1025894	1002829	1189936	883390.2	1815165	1195811	1162467	300198.2
410365.6	579180.6	742207.1	1168371	1127333	748249.5	1159467	1292594	1268569	254641.1
340851.7	546770.1	575536.4	1181948	1211592	913789	1235767	1489456	1184946	248811
473179	658605.2	1252039	1411004	1062570	938777.9	761226	1430553	1439745	350286.2

LN248.960	LN248.960	LN248.960	LN248.960	LN248.960	LN248.960	LN248.960	LN248.960	LN248.960	LN248.960
401477.2	1080724	716530.8	444755.5	1278559	255343.9	248995.1	1308401	1248385	1616305
411626.6	1446671	725061.4	598153.5	997357.1	208095.8	311869.5	1635707	964058.7	784654.6
494156.2	1195794	622099.7	446223.6	822366.1	156676.5	359791.4	1223254	1066641	900779.7
616620.4	814985	765138.1	462983.3	904896.6	167016.7	252509	1066301	1667331	979910.7
548889.4	1303256	590390.7	372318.6	1023701	163568.1	254472.5	1074559	1874636	754241.6

LN248.960	LN248.960	LN248.960	LN248.960	LN248.960	LN248.960	LN248.960	LN248.960	LN248.960	LN248.960
894577.4	214318.2	961065.2	1338627	588329.3	1081499	644127.5	1318190	247727.1	569413.8
642366.2	208702.2	839401.4	1084543	751118.9	984796.2	873697.9	760898.2	272458.9	607311
573389.5	192197.9	841606.8	1223717	723933.6	1026653	790546.7	740278.5	237508.7	860842.3
540006.4	213790.3	638694.9	881392.9	836575.6	923110	677473	723451.6	242672.4	863863.7
841140.5	173210.2	656813.2	1255389	558554.6	913509.5	1035798	789165.7	441557.3	668551.4

LN248.960	LN248.96_	LN248.960	LN248.960	LN248.960	LN248.960	LN248.960	LN248.960	LN248.960	LN248.960
884965.2	1506201	605959.6	1102250	331875.8	861156	1380304	804395.2	919112.4	694852.2
1507261	1079434	434958.6	1104454	332054	1010324	985923.8	732751.4	1375522	908187.7
1420830	1403716	629478.1	1463169	381453.5	650017.2	1181544	1316066	1409180	775278.3
1070780	1139855	772917.4	1339114	261864.9	766568.8	1005449	815083.3	1318049	1163737
1017378	1180810	655010.9	949224.1	310442	883392.1	980081	588862.3	867687.1	937721.2

LN248.960	LN248.960	LN248.960	LN248.960	LN248.960	LN248.960	LN248.960	LN248.960	LN248.96_	LN248.960
919355.3	1302812	961974.8	654973	1160325	1474332	1259470	971443.8	997636.6	718593.4
1374248	1372521	1101709	767879.3	1274465	1396469	827549.8	981421.8	1704651	656760.2
1289117	752902	831322.7	582075.7	1147932	1011370	1006462	907583.2	1320852	557564
907446.5	967524.4	906016.9	523404.1	1029729	1024526	1409975	1199226	1815677	786194.5
1103043	1216959	927980.1	997180	1083580	1157190	1370633	797065.2	1532228	503435.4

LN248.960	LN248.960	LN248.960	LN248.960	LN248.960	LN248.960	LN248.960	LN248.960	LN248.960	LN248.960
552458.4	863516.4	526646.2	665358.8	763696.9	527053.7	945262	1004582	1103620	1327457
488170.1	1052773	615035.4	756925.9	629107.4	487049.4	868399.4	1152970	1327951	1109703
595696.9	714653.9	439148.4	766087.1	705163	955413	1484572	976668.4	1777667	1092761
679505.8	1164027	736301	634612.7	833146.9	744820.1	1017252	1006311	1238242	1416347
556689.9	894042.1	786162.4	686288.4	731975.9	827330.5	1129850	1111784	1668156	973294

LN248.960	LN248.960	LN248.960	LN248.960	LN248.960	LN248.960	LN248.960	LN248.960	LN248.960	LN248.959
644321.5	702554.2	884035.3	839978.4	708194.4	701901.3	638325	203351.7	768784.5	1002031
961539.9	956286.3	639088	657596.3	1011758	866725.5	595115.3	253639.7	818545.1	1256669
893122.2	741104.6	601635.3	591606.1	540157.3	687183.3	554591.7	131759.5	933454.1	1092508
1060035	558665.7	803782.1	629014.9	709488.1	733687.1	588866.8	160565	1059030	1329386
890508	721745.5	740869.2	710759.1	745284.9	683427.9	611485.9	141630	1782608	1065012

LN248.960	LN248.960	LN248.960	LN249.149	LN249.149	LN249.149	LN249.149	LN249.149	LN249.149	LN249.149
1117845	840482.4	1024795	73716.03	51683.02	30022.55	55535.53	41467.07	34217.33	31621.73
934973.8	830747.5	914351.6	38527.47	67125.36	26179.65	54243.81	44553.64	59499.74	39335.81
1148256	836948.8	1057359	44138.86	60234.28	24575.44	62393.07	41109.3	53886.18	41420.16
972275.2	876609.2	1119762	41018.17	50544.61	30722.13	54989.52	46805.33	44801.34	45969.01
994279.1	1033404	832758.1	42704.98	58786.66	27746.85	60272.1	43908.29	51821.06	49816.91

LN249.149	LN249.149	LN249.149	LN249.149	LN249.149	LN249.149	LN249.149	LN249.149	LN249.149	LN249.149
50200.72	33000.1	48180.56	51417.08	66271.46	41213.38	44540.44	33140.54	34397.81	52384.2
49581.81	26458.96	48771.98	40212.51	47518.05	45087.47	46793.14	37306.98	26266.64	32617.4
40136.97	39039.69	44210.78	46889.7	47433.29	59073.04	56178.64	41413.9	27108.28	40303.94
32819.75	31419.2	84168.7	45733.39	58317.04	49206.85	44370.24	33890.28	26856.22	46186.47
41181.18	34274.38	75601.59	60425.94	78638.5	48512.6	54210.78	58759.15	36757.82	39632.62

LN249.149	LN249.149	LN249.149	LN249.149	LN249.149	LN249.149	LN249.149	LN249.149	LN249.149	LN249.149
44823.81	50723.18	38235.79	52400.43	27103.67	47361.8	40555.88	38328.27	60195.93	30150.07
50705.76	43994.26	35357.12	43550.84	28680.8	38759	25683.33	55179.62	45617.87	38118.33
37091.16	46902.47	43227.51	38298.22	32244.92	52305.59	33773.61	57657.07	44118.68	44079.06
50161.62	46043.9	39288.94	58766.48	31810.18	49048.66	32307.13	64782.91	49630.53	29505.49
74310.81	54575.59	49328.71	52492.28	30295.07	53737.76	52410.43	61615.69	83334.82	45619.94

LN249.149	LN249.149	LN249.149	LN249.149	LN249.149	LN249.149	LN249.149	LN249.149	LN249.149	LN249.149
39624.13	57683.55	34136.53	48932.88	37183.32	35726.06	43968.85	58265.8	50757.71	34571.21
40596.44	58166.79	44786.31	31519.2	45022.81	45012.26	40837.99	55403.23	40886.54	29263.15
42093.43	64226.1	45440.57	39565.33	55328.7	33975.18	48320.96	45501.59	33801.62	34148.09
38337.65	57007.56	35582.33	49621.06	51482.25	37998.5	54386.7	47751.46	41404.28	32668.71
75138.03	72329.52	57910.91	42485.52	51963.94	47230.4	51095.54	48450.97	56336.55	35971.36

LN249.149	LN249.149	LN249.149	LN249.149	LN249.149	LN249.149	LN249.149	LN249.149	LN249.149	LN249.149
65938.83	51751.23	63747.29	79802.12	43940.59	48561.09	35489.12	40842.51	45843.81	40938.93
62002.23	47080.37	44067.22	51469.5	46686.14	48383.46	46793.13	32428.43	45019.71	40669.13
60044.8	50431.48	44873.71	50330.4	50242.31	43174.55	45600.51	37856.55	43610.19	53940.4
53835.85	56796.97	58176.05	65624.22	33186.65	40969.03	48689.99	52502.7	45001.78	55497.04
80297.3	57972.22	55257.76	67576.42	68453.6	60854.59	48834.45	59929.92	61143.2	53580.05

LN249.149	LN249.149	LN249.149	LN249.149	LN249.149	LN249.149	LN249.149	LN249.149	LN249.149	LN249.149
42507.41	55360.49	40348.68	42078.62	47250.59	44998.39	37631.66	54996.95	49215.68	46582.45
34296.57	70707.74	37770.98	27887.56	28754.5	31496.43	29292.14	42187.13	32274.26	39584.72
39756.67	52005.58	43207.94	34081.54	41539.11	32610.58	28884.19	51158.13	35614.62	38762.23
35648.42	51033.52	44585.8	30112.14	39199.81	45508.1	36074.92	54684.35	34259.04	50220.02
46113.55	52426.96	63300.4	41996.59	48184.32	47421.02	41882.47	61934.37	46487	49685.22

LN249.149	LN249.149	LN249.149	LN249.149	LN249.149	LN249.149	LN249.149	LN249.149	LN249.149	LN249.149
43950.78	43695.57	36256.92	41792.73	34915.11	48015.94	45222.33	61193.92	39877.88	34316.72
34153.92	47218.36	47634.43	40047.67	31605.68	53163.15	41260.4	51162.65	35517.37	25919.63
30286.54	30970.68	29207.74	49849.87	52265.65	40080.75	48365.96	50210.45	36568.49	33763.34
33826.89	30259.92	34853.12	47982.72	39658.65	43117.89	36084.24	51199.6	34444.62	27875.7
40998.85	42353.11	43284.98	62308.61	54060.43	49942.34	45371.18	66843.44	39045.37	40484.81

LN249.149	LN249.149	LN249.149	LN249.149	LN249.149	LN249.149	LN249.149	LN249.149	LN249.149	LN249.149
49438.3	46068.86	47354.21	25687.84	59385.64	39287.24	33665.64	36402.94	46482.24	40188.26
46589.4	64776.91	49959.29	35532.49	48973.22	42465.94	32042.97	38267.44	41658.81	47698.59
51587.18	44956.6	44791.05	36806.16	44589.52	34913.93	27688.7	38120.59	50276.05	52248.01
54542.57	47147.41	44607.54	38722.91	60172.22	39804	35476.5	47674.85	45567.41	36099.46
88548.42	66968.57	60235.14	34787.63	66848.51	50496.08	37358.78	52988.21	56701.56	41116.5

LN249.149	LN249.149	LN249.149	LN249.149	LN249.977	LN249.977	LN249.977	LN249.977	LN249.977	LN249.977
32353.71	31795.34	57101.04	64974.76	92205.56	116516.9	160334.4	125455.8	119059.4	150010.3
36755.42	31775.66	33458.54	54881.49	89377.68	157378.7	133774	124350.9	147157.2	126598.8
37349.13	23351.39	36004.31	46342.72	77379.78	103127.4	167408.2	123624.1	107241.8	121223.3
39026.01	41064.41	48661.19	53267.21	142517.1	95504.06	148107.6	115191.7	111591.5	139709.1
41341.83	45046.16	43997.3	76411.91	128533.5	118496.1	134814.2	102855.5	128414.1	123045.7

LN249.977	LN249.977	LN249.977	LN249.977	LN249.977	LN249.977	LN249.978	LN249.977	LN249.977	LN249.978
122494.6	121956.2	148658.7	127640.7	118730	64274.1	56875.68	148096.8	118405.7	76175.73
111588.1	102628.6	134525	123666.3	118847.1	119973.7	34328.02	134248	89278	110576.8
103709.7	105089.7	102481.9	121232.8	141430.2	71777.24	56984.56	108699.6	90084.21	93244.67
132344.2	122983.7	119062.5	135024.3	131471.7	84412.01	38965.65	129163.4	72294.36	98419.96
72591.09	135411.7	144555.9	94477.53	105413.2	81906.72	32605.41	87705.24	89558.59	68054.61

LN249.978	LN249.977	LN249.977	LN249.978	LN249.978	LN249.977	LN249.978	LN249.977	LN249.977	LN249.978
83946.75	125243.3	143129.4	46752	76849.11	115719.5	60736.72	132383.6	138046.3	94497.32
80187.02	150821	152512.1	70424.94	56078.56	118526.8	67865.09	129850.8	159507	79493.36
100651.6	104449.6	143065	54416.38	42636.55	171578.8	54139.37	135370.4	160735.9	92749.19
79186.17	117906.4	128830.1	55689.84	50830.66	124534.9	40000.91	120367.3	181779.5	94485.59
80634.16	110884.9	212157.9	64054.79	38419.09	111669.7	51773.67	141737	199262.7	103835.7

LN249.977	LN249.977	LN249.977	LN249.977	LN249.977	LN249.977	LN249.977	LN249.977	LN249.977	LN249.978
111006.2	126179.4	108623.4	139459.9	108422.2	180694.3	82246.9	179243.6	88318.82	43604.85
140501.8	145434.1	127947.6	149280.9	97346.89	155887.8	70792.32	118448.2	125885.4	42569.65
126669.8	123970.5	100794.6	118718.3	94192.72	130805.5	76124.11	127912.7	98072.18	59159.4
134681.9	155934.8	115212.5	130562.4	86952.71	176833	73193.93	122047.5	72439.15	53594.38
107233.4	118564.4	113092.4	146866.1	62056.32	177279.1	68985.6	131348	123157.1	52770.02

LN249.977	LN249.977	LN249.977	LN249.977	LN249.977	LN249.978	LN249.977	LN249.977	LN249.978	LN249.977
140021.6	118602.5	113622.6	83127.83	94868.58	106264.9	187443.6	94831.23	63983.46	121434.2
154647.9	161587.4	128063.6	83829.83	93358.11	89639.31	166360.4	71626.95	65125.42	152213.7
106079.3	148169.5	98941.52	64266.07	104007.4	102622.2	161686.8	95535.16	56333.88	111537.5
105578.4	135712	89839.97	76737.96	83956.42	108479.4	163633.7	84765.67	62565.58	115258.8
116629.1	117768.4	146278.7	146647.7	112511.7	109418.3	151580	78204.14	68218.7	116389.4

LN249.977	LN249.978	LN249.978	LN249.977	LN249.977	LN249.978	LN249.977	LN249.977	LN249.977	LN249.978
157707.8	120599.3	68257.39	122207.3	156208.5	95632.28	64450.35	127621.4	173541.8	43451.04
94019.55	150771.5	46634.21	108248.7	150375	75463.94	72342.11	79632.41	145797.1	40149.5
106509.8	102550.4	55553.55	144613.1	118668.3	91010.29	80610	127849	112222.8	49776.24
79554.19	131567.5	51918.61	119820.8	159398.1	89695.34	82140.09	92237.91	143564.2	34477.13
79653.26	136674.8	42990.8	115969.6	124023.8	93291.13	77505.8	89225.41	140257.2	41761.66

LN249.978	LN249.977	LN249.977	LN249.977	LN249.977	LN249.977	LN249.977	LN249.977	LN249.977	LN249.978
134618.4	108328.6	148058.6	86887.34	154045.4	105334.8	222345.2	90469.74	122218.4	167599.8
99053.16	81876.56	133727.5	86226.06	200521.8	121739.8	178810.1	100503	107565.8	149603
105824.4	112644.2	152988.9	86601.4	142980.5	138137.3	135480.3	103349.6	214979.5	205391.7
136890.9	126891.6	148970.5	90140.96	171345.2	96964.27	137563.2	97905.3	111612.1	133916.9
147172.8	96070.62	130206.8	112827.9	142313.8	109296.8	126611.6	101193.2	102977.6	115305.7

LN249.977	LN249.978	LN249.978	LN249.978	LN249.977	LN249.978	LN249.978	LN249.978	LN249.977	LN249.977
121124.4	112124.9	53701.13	120809.3	98962.96	39138.58	54482.88	134701.3	130114	93383.36
73016.41	90954.14	56122.07	108366.4	136218.2	61366.28	42116.61	83631.41	128002.9	88205.84
85180.34	86969.15	64377.38	169046.7	108143.2	42941.23	54684.15	75326.06	120634.3	91805.2
107281.2	104286.8	39834.5	113948.3	73321.88	45926.64	42227.06	81887.61	131617.3	80849.66
105964.7	85275.06	60959.46	102416.8	123391.3	46970.5	35472.85	79134.83	127489.7	98290.59

LN249.977	LN249.977	LN249.977	LN249.977	LN249.977	LN250.144	LN250.144	LN250.144	LN250.144	LN250.144
173264.2	98874.24	119848.7	87715.9	133115	26568.9	40158.01	75748.85	41650.57	47295.23
116705.2	106733	117751.2	77724.38	131228.7	20693.9	54592.76	79031.05	42443.08	39755.4
135177.9	120837.7	127765.5	84857.33	123613.4	32018.82	57938.92	84185.52	50843.59	54928.56
173136.5	100120.7	122180.2	109186.4	127868.4	28922.22	53734.43	83078.97	50562	52097.76
179642.4	112913.9	113170.3	73881.47	128640.3	24415.82	52901.55	66807.2	47412.42	59644.24

LN250.144	LN250.144	LN250.144	LN250.144	LN250.144	LN250.144	LN250.144	LN250.144	LN250.144	LN250.144
66639.85	163436	38307.74	51527.12	79876.64	54794.79	84819.92	54227.87	92506.43	61081.39
59249.26	91458.57	49346.64	52954.72	71332.54	66509.51	59612.5	41556.37	99599.48	80139.94
58102.67	106728.2	43129.91	47564.57	88359.08	52118.8	96187.64	66600.4	81012.26	75819.14
98554.39	99849.69	50418.33	50477.47	121912.6	55917.85	85354.5	42875.12	81054.52	76641.78
57886.25	124111.7	37940.25	54511.09	76034.38	75120.13	76120.28	54676.81	91053.17	55928.71

LN250.144	LN250.144	LN250.144	LN250.144	LN250.144	LN250.144	LN250.144	LN250.144	LN250.144	LN250.144
69798.2	91910.17	94387.02	77651.58	82710.19	95284.36	64675	60012.58	70590.2	45490.11
94706.1	99086.61	119236.5	70870.05	62865.26	87319.03	131675.4	79528.79	62879.72	66683.83
56869.53	82051.44	163376.4	88257.75	88712.74	101567.6	81618.04	77996.37	59892.23	42272.12
61118.04	95421.01	99050.94	119959.8	78425.6	91462.16	61710.97	87847.15	67351.67	58267.35
87945.06	109195.9	94140.62	72516.2	88161.93	84218.8	77382.33	74551.74	55531.01	41051.38

LN250.144	LN250.144	LN250.144	LN250.144	LN250.144	LN250.144	LN250.144	LN250.144	LN250.144	LN250.144
51526.76	40518.52	36034.45	65049.16	72628.74	70415.01	94840.41	74985.02	74851.88	44705.86
55670.69	54973.34	48832.54	63985.34	76443.2	65829.21	99981.65	105631.8	66565.1	46033.75
48437.94	50390.43	41040.42	58691.18	67924.02	65975.7	68365.82	79503.62	64082	51716.6
77179.69	41453.5	38941.4	73117.43	71444.87	81267.56	103617.9	113090.4	86461.91	46294.17
60338.86	56875.55	39133.53	76498.82	82770.11	60397.52	102540	79017.15	64511.32	57595.22

LN250.144	LN250.144	LN250.144	LN250.144	LN250.144	LN250.144	LN250.144	LN250.144	LN250.144	LN250.144
91955.13	57749.99	71526.47	95883.11	39326.82	51044.61	65014.77	75965.59	40254.46	60005.1
81985.98	97427.08	43903.54	80196.58	43726.31	60054.47	64742.38	128986	32970.5	58178.79
66212.26	46779.82	57034.38	72533	35452.94	60528.02	55433.55	75550.9	54425.7	78392.12
69913.88	63326.48	54556.14	72919.15	39198.27	70993.84	72484.1	62478.91	51556.19	66332.23
86472.09	54642.44	70359.88	70861.56	39726.65	46798.02	71761.08	63468.05	36586.01	72193.03

LN250.144	LN250.144	LN250.144	LN250.144	LN250.144	LN250.144	LN250.144	LN250.144	LN250.144	LN250.144
114877.6	44522.83	103187.7	58614.17	38578.07	70155.97	88746.59	50426.73	46073.22	84861.86
102192	61742.75	67495.87	48836.31	62418.62	55955.42	93630.36	47312.67	52018.52	144919.5
103933.8	51124.62	61331.63	52407.1	60094.4	90926.78	82671.39	50805.45	56961.05	119526.1
89095.69	53318.38	77862.52	61892.27	47427.9	67863.03	81269.73	37594.83	86314.05	133473
107673.2	64275.88	66639.46	61771.61	50852.69	77851.73	135194.5	52304.71	42474.32	62047.88

LN250.144	LN250.144	LN250.144	LN250.144	LN250.144	LN250.144	LN250.144	LN250.144	LN250.144	LN250.144
129646.9	55101.76	57570.73	45126.12	48386.76	143141.6	27464.24	94847.27	66851.52	100584.8
100815.9	57825.88	46887.88	41529.51	57554.95	142718.3	45250.07	66020.57	45682.26	101306.2
123378.8	47315.41	76624.9	51776.75	45535.52	145803.9	32685.09	96291.97	52335.03	94558.44
119561.8	43378.06	47487.49	54276.01	47845.44	135859.2	40962.85	57859.99	64349.66	96648.57
125906.9	51072.04	64436.99	60557.03	40530.89	124630.4	38986.19	88400.61	54863.8	101640.8

LN250.144	LN250.144	LN250.144	LN250.144	LN250.144	LN250.144	LN250.144	LN250.144	LN250.144	LN250.144
67081.48	48542.53	32205.36	62130.04	123535.7	96418.67	43489.75	55197.23	47353.6	68209.71
57892.9	39969.42	41608.55	68370.25	136633.2	116527.2	45377.76	61616.94	48829.07	47498.03
64352.83	47455.18	40090.15	70974.87	121657.2	94576.93	55157.34	82980.14	67726.98	49542.23
47491.64	40584.41	29499.34	73019.57	179722.2	89893.85	43419.43	49982.14	40892.59	49879.92
62481.24	66140.85	43450.54	64544.37	117330.9	61325.21	50972.43	53498.79	67073.7	46717.36

LN250.144	LN250.144	LN250.144	LN250.144	LN250.144	LN250.144	LN250.144	LN250.144	LN250.144	LN250.144
65565.99	52867.25	85283.47	86601.48	38793.71	51285.09	81874.43	39907.81	38469.52	38635.78
58390.6	59512.51	64458.99	76105.14	37259.34	50747.3	108467.9	49850.02	42983.76	38022.33
63274.84	64056.44	105921.5	71469.71	38855.8	51640.04	74565.22	37139.41	42156.69	31596.96
66516.72	45867	61882.85	69702.89	43122.95	53556.32	115015.5	32366.49	46735.79	39373.74
67515.82	47552.53	71712.93	77184.01	40556.06	45404.13	109079.2	36685.1	63686.64	39854.99

LN250.883	LN250.994	LN250.994	LN250.994	LN250.994	LN250.994	LN250.994	LN250.994	LN250.994	LN250.994
11008.13	36120.59	57100.77	35860.4	91371.31	34489.45	25185.9	53808.75	36331.52	59343.6
9755.734	46333.66	45378.93	26432.74	44803.45	37604.4	30746.29	51480.57	52218.85	60965.96
10301.69	43590.06	49968.94	25319.55	50151.91	35997.84	24311.17	38668.62	42968.78	66205.06
19169.59	39396.98	53177.46	25709.57	52922.27	40472.48	24384.93	41361.27	43313.24	66410.93
11597.06	49389.63	47870.74	28447.19	51525.18	39416.88	25944.35	45340.96	43921.49	73243.42

LN250.994	LN250.994	LN250.994	LN250.994	LN250.994	LN250.994	LN250.994	LN250.994	LN250.994	LN250.994
25029.73	80787.58	56650.13	23022.91	35845.68	59905.5	30440.8	25867.76	22904.15	31774.95
28187.48	57768.69	78750.59	19304.74	34521.12	40219.69	31739.97	31150.47	29742.31	23537.37
26288.55	59795.54	59506.03	25077.48	34577.79	34393.52	25664.27	21872.67	26034.62	22224.51
25794.03	62292.46	66125.66	27212.21	32277.41	34533.25	20389.14	27379.53	23462.87	23826.44
23791.98	56564.72	64120.41	24001.33	31641.3	38259.05	24272.23	24030.44	26817.8	22282.81

LN250.994	LN250.994	LN250.994	LN250.994	LN250.994	LN250.994	LN250.994	LN250.994	LN250.994	LN250.994
39488.97	49533.05	25075.82	25046.53	59010.88	18738.49	27909.92	34647.09	33389.07	40794.25
30471.79	57032.77	48679.38	23684.83	61104.49	11546.87	31223.37	22875.75	29605.75	23446.92
28306.94	50344.67	29331.8	25844.81	62547.23	11292.45	28015.82	24793.01	35236.37	33673.96
31503.78	46772.81	29134.37	23310.72	68804.3	10399.31	24413.92	26080.43	36292.54	32565.89
29821.06	45794.63	34184.12	26145.48	62422.55	13208.21	27515.75	28718.09	36742.22	30071.03

LN250.994	LN250.994	LN250.994	LN250.994	LN250.994	LN250.994	LN250.994	LN250.994	LN250.994	LN250.994
29758.45	60926.62	43668.71	21993.5	23518.77	29327.9	65010.81	39015.53	53171.6	18230.57
41632.22	72888.71	42990.44	15692.22	42884.63	44886.55	53275.59	34950.49	31389.94	13147.13
40427.93	53285.04	26278.37	16735.54	33085.73	40877.19	60848.34	37523.9	50664.49	15266.98
35211.88	62343.79	27598.97	19194.39	30471.75	39442.74	54496.06	41879.75	46987.81	14645.67
36512.82	54853.42	29315.17	17985.88	33360.72	40928	56664.68	38264.86	46446.56	13449.06

LN250.994	LN250.994	LN250.994	LN250.994	LN250.994	LN250.994	LN250.994	LN250.994	LN250.994	LN250.994
43517.65	60037.33	22773.68	16177.94	42883.68	15469.09	76454.3	34689.49	17700.3	35680.24
34563.59	63425.03	24030.67	18110.33	34822.4	16632.76	51075.88	35863.29	15540.21	25894.78
42416.62	45692.81	29718.36	19128.7	36064.63	16796.76	54938.99	29709.05	17411.38	29381.98
41511.37	51059.04	25946.14	15927.34	40344.3	19243.75	56820.13	29355.21	18383.56	34299.56
43397.9	53089.29	28719.36	14642	39683.84	19408.24	59572.32	29849.22	19118.4	30535.31

LN250.994	LN250.994	LN250.994	LN250.994	LN250.994	LN250.994	LN250.994	LN250.994	LN250.994	LN250.994
49274.58	40753.11	52672.77	37390.15	51169.67	52808.8	31095.5	17502.42	39799.19	17104.19
65011.51	56156.24	36513.74	43111.78	27858.92	36330.97	30301.11	15041.89	47567.65	14776.19
50584.33	45747.6	43370.21	38068.72	29718.18	29521.18	31477.57	13773.29	40090.17	18263.24
50670.07	40223.57	43389.51	39028.7	28543.17	29059.72	25385.76	13507.37	41223.58	18866.24
54279.39	44794.35	40635.17	38344.31	25933.72	32310.41	23460.92	13764.43	38437.33	15212.33

LN250.994	LN250.994	LN250.994	LN250.994	LN250.994	LN250.994	LN250.994	LN250.994	LN250.994	LN250.994
16132.07	35725.88	15549.19	21466.62	51595.65	39176.27	37056.11	12991.5	49739.82	26345.19
20161.55	43095.01	20833.39	30565.03	56640.53	19178.76	42192.38	15061.22	49316.35	27047.34
15067.36	33710.6	17205	25099.69	53019.53	27682.73	45518.88	14216.2	43772.06	27071.23
14707.82	34688.66	14372.38	23005.79	50662.25	26430.12	42448.09	13787.09	37020.33	26612.08
14514.64	32732.43	15202.33	24468.15	51522.44	26463.52	41992.33	14594.92	39037.99	27425.08

LN250.994	LN250.994	LN250.994	LN250.994	LN250.994	LN250.994	LN250.994	LN250.994	LN250.994	LN250.994
40161.93	25103.14	36623.45	66923.28	47185.34	22651.21	27165.21	42923.87	12639.14	43409.26
30130.82	34860.59	33190.07	49125.94	47935.24	21367.98	29120.52	30193.49	14153.83	60445.72
39094.59	29288.84	38348.17	52306	44648	21970.76	27819.84	28777.99	11972.02	48446.99
40344.79	30308.06	39045.62	45445.91	43252.58	23345.43	32044.39	31924.84	11445.68	50091.25
39561.24	30808.79	36589.02	49146.93	42263.82	23693.49	26375.82	29778.27	13189.84	52660.22

LN250.994	LN250.994	LN250.994	LN250.994	LN250.994	LN250.994	LN251.147	LN251.147	LN252.916	LN252.973
25494.97	64338.51	29087.32	48646.26	17828.87	32960.84	19926.11	16282.09	27121.05	22833.33
19037.86	47563.97	26413.53	51364.91	13022.09	36186.58	23540.33	15911.86	25555.79	13864.36
22724.54	55615.03	26047.74	46196.38	16024.78	33567.57	21346.84	16348.89	20169.03	25061.87
24427.32	57504.72	26618.87	47367.89	13889.49	30548.09	22004.65	18388.41	24582.85	26641.81
21352.61	55275.42	25344.59	47828.7	14744.36	29993.02	22339.42	16035.47	28835.76	18118.45

LN252.973	LN254.100	LN254.100	LN254.100	LN254.100	LN254.985	LN254.985	LN254.985	LN254.985	LN254.985
20082.39	15064.92	13039.03	15772.63	20311.07	42391.81	37588.9	48902.69	56591.54	52362.02
17155.59	15896.67	12116.03	19493.15	17309.02	41268.16	34213.75	45069.38	61584.84	49429.86
20956.62	14688.99	15081.71	18632.09	20303.04	44219.36	41308.88	42503.86	60506.34	53088.43
20614.55	16653.91	13750.7	16157.92	17568.73	51651.36	35818.73	50100.08	62960.12	50823.57
13341.58	15687.18	11120.34	15307.3	17929.04	44247.14	39936.82	53723.34	56808.33	50676.36

LN254.985	LN254.985	LN254.985	LN254.985	LN254.985	LN254.985	LN254.985	LN254.985	LN254.985	LN254.985
50351.33	30429.03	46724.64	49252.09	47898.6	38696.32	64553.51	38530.17	38520.11	37708.31
48271.56	31144.76	50488.8	49481.21	45850.88	37153.23	72552.89	41779.43	35584.52	39587.77
46861.7	31446.8	48593.65	44165.56	47769.48	37225.84	65196.03	36041.58	32046.7	39486.01
52993.84	26972.98	48357.56	50436.57	44926.04	41998.52	67564.36	37852.61	35610.22	42179.4
49883.08	32549.34	58805.74	46828.78	45446.01	40624.71	69458.88	42146.09	34600.4	41573.58

LN254.985	LN254.985	LN254.985	LN254.985	LN254.985	LN254.985	LN254.985	LN254.985	LN254.985	LN254.985
36455.75	41985.31	38900.79	27671.04	33954.99	41681.84	32666.04	35849.42	40753.6	26011.83
33786.8	40609.31	37383.22	30030.37	35853.17	46819.01	33154.26	35833.91	37142.41	34056.92
32761.13	36106.14	40537.24	31706.28	34410.72	42314.19	31122.66	37219.85	42751.86	30346.91
31814.85	38331.85	39540.54	30929.03	32522.96	41686.65	32986.14	39716.29	39157.78	27426.4
30194.09	37593.5	41880.04	29127.36	40364.14	42814.44	30006.06	35279.64	41951.32	27435.62

LN254.985	LN254.985	LN254.985	LN254.985	LN255.232	LN255.232	LN255.232	LN255.232	LN255.232	LN255.232
36350.66	33709.43	24312.7	26172.97	58461.93	155961.6	416977.9	108029.1	96754.33	151079.6
40379.13	35477.65	22962.38	24580.25	80590.1	132784.6	437259.1	102380.8	137756.2	86741.19
36159.65	33746.74	23009.36	25566.2	82957.21	151980.7	454013.3	117958.8	112105.1	105122
36757.63	32080.41	21625.13	24878.97	80196.86	181691.1	409613.8	145118.9	144100.1	131502.6
36288.97	33846.84	25753.89	23249.69	94702.93	186296.7	417953.5	166696.5	143226.8	121854.7

LN255.232	LN255.232	LN255.232	LN255.232	LN255.232	LN255.232	LN255.232	LN255.232	LN255.232	LN255.232
161146.9	95513.09	106533.6	101522	142863.7	121236.2	80246.64	120092.9	125958.5	91753.87
114267.3	99800.31	127716.9	97618.75	110101.2	106680.1	94092.9	106623	109386.5	91947.34
130846.8	112781	143317	118958.4	119565.2	130192.3	101626.4	117825.4	152708.1	132585.3
178518.8	125190.5	149125.8	112251.7	138561.1	155932.9	130699	135607.6	186769.1	107229.2
146863	123508.3	163723.6	121505.6	135382.8	153667.3	122240.7	128204	201383.4	134957.7

LN255.232	LN255.232	LN255.232	LN255.232	LN255.232	LN255.232	LN255.232	LN255.232	LN255.232	LN255.232
124201.6	151970.6	126596.6	78756.75	70453.82	125755.6	127259.4	72748.64	93718.44	166196.1
113318.2	121156.1	89802.88	78487.07	72282.41	122903.9	127382.8	80455.23	111814.8	157070.3
133038.8	141669.1	134274.3	98818.76	79191.4	132983.3	135461.4	101080.9	106572.2	152897.8
178313.7	154635.2	138801.1	90577.22	93984.65	171868.3	146413.9	99243.06	121133.1	182033.2
151409.6	149152.4	139456.2	103361.1	103059.4	177940.4	164348	104998.3	127177.9	227608.2

LN255.232	LN255.232	LN255.232	LN255.232	LN255.232	LN255.232	LN255.232	LN255.232	LN255.232	LN255.233
89942.13	104462.9	129917.7	109743	139663.2	88647.66	78631.92	66522.91	165376.3	79223.91
91677.9	110436.7	127280.8	130556.1	148244.4	82641.92	96298.28	56840.08	131314.9	71925.09
94188.12	116720.7	130613.5	158412.1	157677.5	129289.9	121403.1	71346.9	181276.1	78138.54
135958.7	162108.4	137915.7	155723.3	178206.4	105944.8	123389.8	91047.67	194166.5	89230.08
128411.1	158253.2	150209.7	176662.9	195995.8	119454.3	139738.7	99942.08	190895.8	96737.34

LN255.232	LN255.232	LN255.232	LN255.232	LN255.232	LN255.232	LN255.232	LN255.232	LN255.232	LN255.232
112588.1	93143.41	159586.6	119219	130130.3	120655.8	119474.6	118393	128782.9	79825.83
121284.3	120748.8	164592.9	101050.2	133057.7	121216	127181.9	123742.6	93148.55	112201.6
123846.5	158758.6	130873.2	123796.5	162409.5	148701.6	145065.9	140223.7	114956.3	104001.4
132906.9	134868.5	147885.9	139430.2	175102.3	177777.2	141285.5	138180.5	113216.3	115699.4
167494.2	149204.4	154890.6	164294.6	180071.2	173694	167938.9	146467.6	167838.7	114915.3

LN255.232	LN255.232	LN255.232	LN255.232	LN255.232	LN255.232	LN255.232	LN255.232	LN255.232	LN255.232
93102.85	96330.74	106086	112159.4	100313.7	135928.2	87114.86	118197.9	88385.08	126945.6
98321.17	100614.6	99250.65	155671.4	112011.8	141394.6	100350.9	95391.89	91104.27	106513.9
125554.2	100280.6	103001.5	134693.7	118574.7	134510.2	109292.5	108955.3	104285.9	127364.7
143736.3	120616.2	126643.5	152463.7	142793.8	155905.1	133651.8	118624.9	111802.5	146849.4
141093.2	126301.7	117879.9	164092.6	124127.4	171645.5	144268.7	127770.5	114558.4	159521.8

LN255.232	LN255.232	LN255.232	LN255.232	LN255.232	LN255.232	LN255.232	LN255.232	LN255.232	LN255.232
136576.2	177497.3	133785.7	97482.35	102480.8	140525.2	67749.28	136777	142632.8	101138.3
105649.3	158603.6	112174.7	102683.8	123036.5	122047.1	83325.26	153456.6	132640.6	132422.5
117622.1	134662	134553	117152.7	150024.6	129334.5	88843.81	197647.8	162291.6	108942.2
142446.3	193191.3	146377	125023.6	158194.5	166791.6	101025.2	149621.7	160987.3	122188.5
144192.3	166653.1	155198.4	134775.1	146364.8	155751.5	105198.2	166247	168157	124597.2

LN255.232	LN255.232	LN255.232	LN255.232	LN255.232	LN255.232	LN255.232	LN255.232	LN255.233	LN255.232
197055.5	125541.7	100232.5	129279.1	106233.9	128962.4	152487.5	65792.28	71405.14	116006.9
131348.9	90030.96	124687.1	132877.2	120737.2	103881.4	149134.5	53356.41	75390.53	115554.1
163994.7	123406.5	114287.1	150135.9	110544.9	117294.9	155075.6	71330.03	88426.6	140830.6
177698.4	116793.4	146242	182352.3	131001.3	130362.8	182558.5	78610.32	98863.82	145032.1
185603.3	163651.9	127084.5	210053.2	142620	163248.7	199224.3	88836.68	100506.5	146074.5

LN255.232	LN255.232	LN255.232	LN255.822	LN256.236	LN256.236	LN256.236	LN256.236	LN256.236	LN256.236
128487.5	97244.85	126882.5	26843.11	71920.74	18439.54	16802.19	14967.83	17197.28	14567.76
112509.8	71021.92	111778.1	20803.94	75145.68	16224.7	17231.73	15937.9	16026.13	15362.99
148419	80761.92	146443.1	23759.42	64414.64	18626.5	19478.83	17018.94	16995.12	16182.17
172654.2	93533.59	143048.3	24407.09	65313.92	21045.72	20079.28	19707.44	18603.43	18153.54
187674	126406.1	142874.5	26750.09	67493.27	23119.06	20908.23	18599.52	21939.5	19255.59

LN256.236	LN256.236	LN256.236	LN256.236	LN256.236	LN256.236	LN256.236	LN256.236	LN256.236	LN256.236
13192.32	16483.36	22111.93	14257.36	13545.51	23821.13	15693.43	25213.79	16844.95	17798.52
12241.62	13363.79	23434.92	11624.49	15026.79	23490.2	16914.73	24909.83	14934.77	19273.51
15203.44	15223.68	26262.92	15983.98	17658.3	24238.07	19832.27	27870.43	18490.3	20890.48
19063.92	19350.02	27246.37	14352.69	19284.74	29485.74	18836.47	31228.32	21766.22	27691.63
18377.02	21675.01	29332.09	16269.8	18000.11	27584.21	23536.16	30370.47	23167.97	26610.91

LN256.236	LN256.235	LN256.236	LN256.236	LN256.236	LN256.236	LN256.236	LN256.920	LN256.954	LN256.985
16516.14	24575.52	16284.63	19787.06	16728.69	15452.03	14905.16	59428.43	69065.28	52066.89
15974.15	24639.87	19200.3	18377.35	16881.01	15634.59	16243.91	56892.53	64905.25	52626.16
17504.12	25522.36	19996.18	25089.4	17595.37	17690.95	17120	61618.82	78644.31	51526.45
20160.4	28399.46	25086.51	23886.39	20894.82	20537.56	17645.23	55484.28	77856.76	53101.28
23000.27	29742.18	26205.44	24511.39	23071.91	22011.41	20797	70072.51	75306.81	55665.16

LN256.985	LN256.985	LN256.985	LN256.985	LN256.985	LN256.985	LN256.985	LN256.985	LN256.985	LN256.985
51874.05	44422.1	44250.92	44787.22	41887.83	44024.18	50289.18	43291.08	42294.55	35260.99
54379.94	44500.12	45211.18	53593.48	40769.95	45238.36	51824.24	40060.71	43659.96	31777.23
50744.6	45835.43	50572.56	46978.35	42848.12	38771.77	46936.39	43927.71	41105.37	31556.14
54076.46	43476.32	46721.96	46767.38	45450.68	46907.84	49195.59	39006.18	42085.07	35079.54
54815.53	50309.72	49686.3	47209.56	36549.6	43872.99	51766.23	41556.57	39888.34	31259.58

LN256.985	LN256.985	LN256.985	LN256.985	LN256.984	LN256.985	LN256.985	LN256.985	LN256.985	LN256.985
36708.52	27671.65	39065.8	29317.13	28686.76	35190.63	36838.94	40545.28	29853.98	42459.06
29586.58	25490.44	45933.78	28827.19	30356.53	37373.87	35738.39	39282.56	30340.02	42767.21
30065.49	25086.97	38889.55	30260.06	29092.88	36703.8	33811.22	42107.25	28783.19	37219.61
33632.61	30400.6	39861.13	29045.93	28008.39	35698.54	34716.79	39704.97	28261.35	44839.76
34005.38	30729.45	40319.25	28265.34	31951.44	38036.86	35132.33	44018.76	31402.65	44704.21

LN256.985	LN256.985	LN256.985	LN256.985	LN256.985	LN256.985	LN256.985	LN256.985	LN256.985	LN256.985
30633.18	46707.01	26038.74	34537.38	20996.37	29040.95	21855.95	23233.84	24869.77	35222.46
30273.42	51038.5	24179.7	32130.39	22531.88	27251.35	22085.08	23480.74	26484.45	35951.94
29257.28	46118.37	27433.15	32229.67	20852.94	30192.82	21164.85	24354.7	27508.23	35497.52
30808.01	48113.73	26820.55	31000.54	21892.61	24751.37	20569.58	20998.82	27883.33	32025.93
30019.56	45645.87	25276.99	31031.69	22649.34	29635.07	19130.3	22723.36	28612.67	34780.22

LN256.985	LN256.985	LN256.985	LN256.985	LN256.985	LN256.985	LN256.985	LN256.985	LN256.985	LN256.985
39111.64	27149.08	24190.19	22127.46	26364.02	25627.9	26526.69	22839.56	46338.25	40647.09
37183.88	27538.97	28613.52	27171.25	25022.34	24808.19	29424.75	24268.27	47777.3	44808.93
34505.43	31575.3	24723.82	26846.18	26041.18	26285.79	25296.2	24962.17	41596.84	37463.51
35458.68	29273.88	22961.05	23305.52	22142.23	24479.5	25175.13	24020.09	39122.86	38819.29
36187.78	31000.1	23506.78	25453.94	22783.53	25217.76	27900.37	21822.32	42858.62	40524.54

LN256.985	LN256.985	LN256.985	LN256.985	LN256.985	LN256.985	LN256.985	LN256.985	LN256.985	LN256.985
25240.19	37214.33	31959.71	32581.72	54205.34	24561.92	34953.08	33394.7	30789.63	42435.12
25327.39	34026.93	35154.19	36725.91	56580.3	27767.67	35161.16	29329.09	31520.54	45026.97
24085.58	36759.67	33073.2	32340.24	56889.36	26512.78	35010.16	30774	29752.39	43557.92
27741.19	36153.02	35565.71	33809.74	47602.91	26609.3	34886.19	33103.52	32367.98	42563.5
24405.5	31957.28	33037.61	36198.22	48161.16	25183.68	34892.58	30210.11	31656.82	40448.14

LN256.985	LN256.985	LN256.985	LN256.985	LN256.985	LN256.985	LN256.985	LN256.985	LN256.985	LN256.985
26416.04	31227.86	33336.2	56623.7	43097.6	66598.18	24605.3	36042.37	24908.99	31460.61
28996.4	29165.72	39386.27	54550.76	40182.49	64995.32	26586.35	37642.31	25818.82	33851.59
27925.3	29514.39	37070.92	52729.9	40944.66	65464.34	28013.09	37291.41	25369.8	31255.43
27291.64	31017.26	34696.82	49733.82	41699.1	59459.49	28739.88	41343.98	25507.38	30688.58
25061.95	30293.81	31415.93	51339.18	41515.76	59453.02	24532.82	37172.12	24346.47	33991.07

LN256.985	LN256.985	LN256.985	LN256.985	LN256.985	LN256.985	LN256.985	LN256.985	LN256.985	LN256.985
32345.4	35397.29	32891.03	23470.22	31945.82	25654.58	40219.27	20936.22	53258.8	30781.84
30331.19	32360.1	32484.37	25419.63	32998.31	26438.69	37615.81	21352.65	53893.52	28388.22
28135.02	33782.92	31879.56	26125	32100.27	29068.69	40031.48	18391.13	48083.99	31928.97
28879.18	34044.92	32156.04	28254.42	32680.7	29044.09	39930.68	20078.58	51712.71	29794.94
27824.48	32237.68	33767.62	23166.58	35974.28	23981.34	34309.2	19402.81	50871.75	30911.41

LN256.985	LN256.985	LN256.985	LN256.985	LN256.985	LN257.102	LN257.102	LN257.102	LN257.102	LN257.819
36635.05	33693.51	26612.74	65582.88	72107.3	15783.26	22327.07	15462.08	21601.9	27239.74
36380.2	34427.82	25444.01	60679.52	71935.73	14011.3	20562.12	14549.73	21097.9	21826.03
38964.58	35689.48	27192.95	59338.7	69354.36	16558.67	19819.84	15097.63	19397.78	21173.39
35969.37	33293.87	25059.75	58124.92	75147.73	16120.14	20644.18	13035.56	18262.9	25095.77
35662.66	31420.63	27997.43	60411.04	69128.67	15619.14	20241.8	16017.77	16912.43	23271.37

LN257.856	LN257.917	LN258.915	LN258.915	LN258.916	LN258.915	LN258.915	LN258.916	LN258.915	LN258.915
15651.23	42498.63	930422.3	228890.8	419712.4	228469.1	321580.3	414783.3	192075.8	241013.2
15605.74	32041.23	1349762	198106.5	329806.2	263450.1	315689.5	499350.1	213291.6	235325.2
12586.97	41607.85	1363880	237706.3	296015.6	217319.5	362493.4	495840.9	208645.2	327310.4
40516.7	38031.16	1171398	203638.1	292172.4	208721.3	305019.2	528429.9	228965.7	287891.8
36118.93	52431.16	1253491	189678.5	310342.1	195279.7	371949.6	576637.8	207420.1	215629.9

LN258.916	LN258.915	LN258.915	LN258.916	LN258.915	LN258.915	LN258.915	LN258.915	LN258.915	LN258.915
598852.6	133147.9	251665	438790.7	149962.4	335713.7	325832.6	242224.7	366805.3	213687.6
638593.2	129400.8	211889.4	409616	151371.4	307869.7	248173.5	249758.1	375281.6	232911.4
631039.6	146814	231401.9	371411.5	158571.2	328664.4	394269.9	246306.9	367241.4	248707.1
752733.1	138264.8	270416.7	321858.5	148636.6	310527.8	226527.8	256350.9	386830.8	258571.2
631523.2	113424.3	253021.8	449692.6	148135.7	282777.5	252791.3	271362.7	374658.7	240709.9

LN258.915	LN258.915	LN258.915	LN258.915	LN258.915	LN258.915	LN258.916	LN258.915	LN258.915	LN258.916
251648.2	390738	279443.7	282661	211025.5	165523	142981.4	239668	210874.4	146667.1
178365.6	383053.1	260285.6	269968.4	224618.9	164435.7	124599.1	232212.5	207438.9	156401.4
173398.9	280704.3	262494.3	289253.8	263555	181325.8	140451.5	235045.9	179088.2	187443.2
253874.7	400375.9	291644.2	298915.2	224322.5	160352.8	138425.4	289080.5	217388.5	159448.9
140448.2	410681.8	260779	285035.7	222144.5	165328.3	111779	216819	205058.2	151026.4

LN258.915	LN258.915	LN258.916	LN258.916	LN258.980	LN259.000	LN259.000	LN259.001	LN259.000	LN259.000
154871.4	125443.6	115072.7	233101.2	90262.73	67779.13	94480.18	109565.3	84506.4	99094.54
155875.9	129252	128185.7	236265.4	91119.04	184834.1	59361.83	73428.9	86471.32	76219.78
155908	134504.2	121617.1	239278.2	96687.58	141287	54113.03	80032.67	100777.3	103335.8
156171.4	136913.5	120780.3	231720.2	97535.51	110974.1	48048.46	58670.09	86571.62	81861.46
146509.3	119755.8	101608.2	211176.6	93127.97	134185.1	97788.22	65738.13	112703.5	74727.43

LN259.000	LN259.000	LN259.000	LN259.001	LN259.000	LN259.001	LN259.000	LN259.000	LN259.000	LN259.000
79952.99	64416.55	38481.44	49997.52	46429.71	71279.45	96729.7	96247.79	103975.1	114273
70910.51	59338.09	55248.67	52749.06	52109.31	46512.12	90015.37	91698.13	82988.75	112027.7
92346.5	69732.87	65803.27	82212.75	51668.44	53540.1	144114.7	69215.72	118427.3	148973.2
68758.6	56948.98	55431.4	48095.25	65567.88	58242.94	85352.25	80874.28	101113.5	102079.1
69709.78	90174.2	46494.06	50048.6	63202.75	66119.81	89211.63	64801.67	109943.2	85478

LN259.000	LN259.000	LN259.000	LN259.000	LN259.000	LN259.000	LN259.001	LN259.000	LN259.000	LN259.000
97026.53	64293.45	75083.01	91762.62	99640.36	103123.8	72022.12	78177.71	82368.76	114846.2
109227.9	54004.39	67530.28	87132.66	125880.3	112845.2	54933.06	71180.83	97483.33	147640.5
124020.6	66417.09	59081.9	75779.21	95937.93	202474.5	57578.41	67204.3	90399.45	81531.63
116294.8	65041.33	58430.59	67710.5	102174.6	117568.5	41805.39	78167.58	87473.18	114053.7
99309.98	64185.33	55831.76	98678.24	95580.16	121991	51307.91	65586.66	106722.9	138910.2

LN259.001	LN259.000	LN259.000	LN259.000	LN259.000	LN259.000	LN259.000	LN259.000	LN259.001	LN259.000
51902.16	18383.34	92140.81	40802.34	109827.5	77098.08	110017.1	111575.2	44478.66	46154.52
66851.32	26281.66	64693.45	26555.73	123573.8	60441.24	86675.45	117153.5	34628.72	46218.11
66464.6	25346.68	76995.09	31116.94	111517.9	51584.66	94944.67	191543.2	33282.03	60776.05
59231.83	17244.89	77334.77	31975.03	139136.1	61348.81	95512.62	133693.2	26559.66	54562.79
60550.88	15748.46	77629.02	39848.64	234875.7	59068.64	138176.7	124092.3	48055.54	58588.54

LN259.000	LN259.000	LN259.001	LN259.000	LN259.000	LN259.000	LN259.000	LN259.000	LN259.000	LN259.001
45363.99	93568.46	45342.49	93232.19	101824.9	115629.6	76483.64	46108.08	48243.82	20112.11
54450.83	95279.2	24189.26	126595.9	113432.4	114542.4	95002.05	34494.6	39779.67	30254.15
51746.11	67370.33	32733.78	91655.71	114911.3	101935.7	72552.2	38151.15	51554.9	24577.3
56088.16	75332.14	28550.11	80035.79	109323.8	89559.88	82226.27	38025.96	53965.75	26030.44
60049.05	74095.44	35612.26	110611.7	91013.36	75919.99	90284.24	64422.33	55675.01	39084.48

LN259.000	LN259.000	LN259.000	LN259.000	LN259.000	LN259.000	LN259.001	LN259.000	LN259.000	LN259.000
107702.4	67571.54	42736.69	152337.2	68080.24	131268.9	37100.42	70379.16	53823.4	63056.73
69724.93	61962.66	48165.02	115506.9	73359.18	126324.4	42734.29	65222.65	71877.45	49851.82
125588.4	75032.02	48575.62	94253.17	100694.9	120045.6	48038.61	91948.36	42679.58	86564.72
79082.8	86598.03	50605.72	134464.9	66266.67	114118.2	40918.96	64190.74	45280.9	62150.51
73685.66	80886.55	64684.3	86556.32	91686.15	100994	52175.94	61243.26	41044.35	56903.71

LN259.000	LN259.000	LN259.000	LN259.000	LN259.000	LN259.001	LN259.001	LN259.000	LN259.000	LN259.000
137562.3	49389.33	80746.98	111407.5	110852.9	22007.15	23901.12	72678.17	54983.76	61979.55
148770.2	47226.28	103607.7	119244.3	112748.2	28068.79	19626.63	70509.1	62383.69	52350.57
120446.9	43814.64	97887.35	120396.7	115759.7	27896.52	20275.61	65345.03	85387.14	68404.8
111392.1	54528.79	64658.86	136651.9	83026.64	23850.96	23139.45	69669.98	57871.37	60240.27
99032.95	50961.75	96994.63	104880.5	74809.12	27481.79	22849.62	115858.1	54939.33	57871.02

LN259.001	LN259.001	LN259.001	LN259.000	LN259.000	LN259.001	LN259.001	LN259.000	LN259.000	LN259.000
84201.13	34100.63	63176.16	113966.4	76502.31	51032.5	31204.47	97262.67	161940	47843.93
70030.86	42830.38	56859.72	108154.2	76204.02	55499.38	30836.17	87535.81	130873.4	96165.02
66397.41	35037.22	70426.55	87464.44	74606.41	69640.27	30691.59	94911.11	120588.9	44112.53
59175.38	40243.56	49003.33	100977.1	74567.07	52247.35	32148.89	81183.36	106205.8	73125.13
59318.08	38668.72	52561.62	98413.85	78666.24	48700.44	29364.21	131618.7	95846.16	73900.82

LN259.000	LN259.001	LN259.001	LN259.001	LN259.000	LN259.000	LN259.000	LN259.000	LN259.918	LN260.915
38178.53	55236.75	31538	51641.89	82084.58	74623.25	63083.36	108270.7	31815.11	127144.5
32912.95	50812.05	53797.25	80396.29	105378.5	108084.4	71590.16	116195	29482.89	111567.1
33464.56	57625.72	27396.69	54022.92	84102.55	115936.6	82288.91	83259.85	37761.37	108964.7
34817.93	55621.18	35320.61	50395.52	104449.6	94726.67	69400.27	76660.25	22816.6	105695.4
39010.74	62062.4	28897.98	62717.38	138010.2	88233.8	77817.04	78626.44	32933.39	92814.97

LN261.134	LN261.134	LN261.134	LN261.134	LN261.134	LN262.857	LN262.911	LN262.992	LN262.992	LN262.992
15300.17	15160.63	15853.7	14992.59	16016.24	51784.08	42055.26	43367.61	44561.85	38341.08
15210.08	15225.98	13076.46	16740.97	15412.98	48226.55	52274.12	45198.25	42074.12	39625.11
14464.55	12157.74	15232.87	15438.11	17801.14	49162.65	44369.09	47606.21	46107.11	42302.5
15661.75	14683.2	16963.56	14219.83	13613.8	16276.96	39726.22	46845.97	44115.78	42771.42
16395	12882.92	15133.88	15663.7	16242.59	18851.41	42503.68	45122.95	42544.78	43096.89

LN262.992	LN262.992	LN262.992	LN262.992	LN262.992	LN262.993	LN262.993	LN264.934	LN264.934	LN264.934
40744.76	26335.97	46482.6	30614.7	50852.98	26203.74	44129.35	22244.69	36451.69	33399.09
37313	32677.22	46091.54	28388.69	51239.91	24232.73	46402.22	23226.22	38173.41	31697.36
38166.41	29329.23	45157.07	31275.13	51349.87	26509.38	49405.04	33015.4	42924.7	35699.11
39236.19	24378.33	47890.13	30281.39	51563.59	25385.54	45752.28	24156.28	46041.63	49303.07
40326.75	34193.87	45875.59	31884.94	53133.79	26085.78	47420.4	22986.15	38993.93	31030.02

LN264.934	LN264.934	LN264.934	LN264.934	LN264.934	LN264.934	LN264.934	LN264.934	LN264.934	LN264.934
35779.37	35095	40419.59	23520.87	38066.81	27427.32	40293.29	55938.6	50887.59	47735.48
33536.49	35998.05	39054.12	26528.38	39238.61	28186.53	40288.29	53145.64	51773.31	48014.63
36753.88	53985.03	45737.08	25946.55	34817.13	26002.25	43374.27	37066.24	57441.79	48796.44
38950.33	32022.01	48296.9	28173.76	50428.63	39313.48	41310.94	53528.75	51887.96	45331.27
37461.82	32537.72	35054.07	24805.86	37411.09	26015.12	41213.11	56732.11	48652.14	49280.2

LN264.934	LN264.934	LN264.934	LN264.934	LN264.934	LN264.934	LN264.934	LN264.934	LN264.934	LN264.934
54484.96	48637.28	55879.82	31272.4	33898.88	43900.45	38738.82	40253.29	63865.34	43194.37
54932.26	45198.22	56784.4	31709.64	33367.02	44091.12	43182.29	43324.22	63761.42	42169.39
48444.75	51807.56	57237.61	33141.42	34968.25	42749.22	38303	40941.44	61678.46	44306.23
55724.95	47215.3	54714.39	35185.26	32530.45	41207.02	43937.11	41677.05	60320.69	45010.82
53399.2	50545.31	55128.85	36165.43	35291.32	41413.5	44946.31	39892.96	62356.51	47709.61

LN264.991	LN264.990	LN264.991	LN264.991	LN264.991	LN264.991	LN264.991	LN264.991	LN264.991	LN264.991
33706.07	80137.06	87490.55	41713.02	83653.98	73563.32	66682.41	78408.48	156208.9	68146.56
27293.7	118117	59175.43	36798.1	83924.95	104827.8	117570.5	85200.13	146622.1	92210.3
22063.26	80520.71	61402.22	41028.32	80413.05	83294.42	106015.9	77522.72	127086	97460.3
20613.8	113277	81355.69	58728.44	132311.2	109705.4	65625.08	81560.14	123742.8	114167.7
27650.96	73732.19	70002.49	45698.39	61328.13	108544.8	61394.05	83562.11	106334	79530.38

LN264.991	LN264.991	LN264.991	LN264.991	LN264.991	LN264.991	LN264.991	LN264.991	LN264.991	LN264.991
64785.08	38603.54	72055.49	55786.43	58251.36	102125.7	39180.58	52507.93	37760	63077.56
71131.35	35058.13	74013.82	54217.39	55101.67	91208.06	37714.72	50260.36	56247.17	62730.59
55728.96	47994.99	100212.3	48689.07	54112.78	103502.6	56465.91	43055.9	41338.7	40462.75
50895.46	38459.72	88615.11	49422.99	51106.73	124719.7	52495.32	61035.68	44603.34	53801.39
78367.58	40330.01	76998.16	66367.65	86933.19	134387.7	46424.83	55654.12	45455.8	62425.5

LN264.991	LN264.991	LN264.991	LN264.991	LN264.991	LN264.991	LN264.990	LN264.991	LN264.991	LN264.991
85508.9	44749.98	88479.81	106586.5	57247.16	74036.39	130043	50658.24	71884.03	43634.13
88396.71	42940.56	60705.57	84780.14	47584.56	69094.6	113719.2	66370.72	67668.73	51070.34
70293.78	45392.95	102070.8	102963.4	60300.94	80975.2	169373	61296.38	78336.73	46392.35
115929.8	60120.34	57523.59	75963.85	49696.24	77063.33	112488	55718.17	90449.07	59692.36
104286	54231.38	88953.57	90334.64	72670.18	74464.23	108812	66648.46	75519.65	51740.99

LN264.991	LN264.991	LN264.991	LN264.991	LN264.991	LN264.991	LN264.991	LN264.991	LN264.990	LN264.991
48214.88	67208.39	81859.82	76010.86	99541.94	153152.5	85555.65	113649.4	156746.5	72934.59
50588.01	110872.8	96183.92	105336.5	83755.12	103328	112095.7	130291.6	105610.5	112037.2
56356.76	85891.11	100893.7	140987.3	98886.34	131067.1	95440.06	119380.2	118223.1	77943.14
56797.28	79073.9	111049.4	128153.6	91997.09	95878.52	104903.4	96986.18	160073.1	104261.2
49078.31	80774.6	88949.76	91472.66	82155.4	87047.53	106754	114503.3	106797.5	119058

LN264.991	LN264.990	LN264.991	LN264.991	LN264.991	LN264.991	LN264.991	LN264.991	LN264.991	LN264.991
114523.1	158082.3	132185.8	68647.13	99707.11	56449.06	31932.69	64534.26	172273.6	102479.7
92615.8	121740.6	129002.5	62737.84	72016.38	74648.94	29241.95	51198.92	85659.95	104199.3
88811.24	144753	105183.6	50232.15	93256.83	69956.5	26082.84	47341.81	112670	82792.32
104798.9	92933.35	157380.4	49114.24	102393.3	65955.83	31689.31	69698.16	143858	100079
111156.9	151067.4	145299.2	59765.47	106485	49274.06	33354.22	77616.83	120973.8	113629.7

LN264.991	LN264.991	LN264.991	LN264.991	LN264.991	LN264.991	LN264.991	LN264.991	LN264.991	LN264.991
68543.69	94149.16	61941.52	103444.5	91883.76	58626.83	88230.86	59141.87	136649.3	130661.9
70639.12	85553.31	86319.37	55819.89	85760.52	53276.03	111472.5	74547.51	77062.87	98398.14
77651.93	71384.72	46682.74	65231.41	80705.75	51920.83	88764.07	53291.6	89310.21	144317.6
70913.16	80530.85	48385.68	77271.6	74150.03	65480.52	74341.85	64225.1	129102.7	105068
74536.85	80261.57	42228.56	74803.97	126419.1	75227.03	124159.9	75795.79	119963.1	92156.99

LN264.991	LN264.991	LN264.991	LN264.991	LN264.991	LN264.991	LN264.991	LN264.991	LN264.991	LN264.991
40449.42	60789.63	93339.34	55830.92	46096.86	48074.85	48319.66	50964.9	34765.74	42465.45
33500.25	48406.87	71320.86	48118.59	39891.18	67594.65	35562.63	53017.82	42146.86	27504.95
35033.67	67180.99	63555.72	46138.87	38890.43	41564.87	45302.7	59855.26	35638.26	31796.96
33665.68	54412.73	71663.64	52943.18	45935.17	48022.91	43728.78	86561.35	28002.81	31277.91
48700.33	47145.35	74951.49	63630.36	39733.81	51432.5	44827.68	65884.34	40619.56	31641.47

LN264.991	LN264.991	LN264.991	LN264.991	LN264.991	LN264.991	LN264.991	LN264.991	LN264.991	LN264.991
37236.24	72716.54	42203.25	43363.61	82541.85	54183.79	73064.91	45413.15	29483.51	32585.82
37071.29	59344.98	32040.42	49255.97	87657.26	64829.39	86687.1	29834.44	34897.86	31438.31
30234.19	75572.15	33110.92	56407.96	75538.2	68589.35	86483.56	50763.83	37014.51	32904.94
28433.1	76372.87	37813.87	38470.84	79480.09	52910.06	75208.74	64039.69	28228.83	25433.99
28906.28	79874.44	39297.14	43805.66	75975.6	52904.81	62120.55	30328.93	27989.09	29634.58

LN264.991	LN264.991	LN264.991	LN265.851	LN265.851	LN265.851	LN265.851	LN265.875	LN265.950	LN265.950
78443.83	86980.57	35220.81	29581.46	11009.7	27274.99	12702.54	32429.45	44957.23	56344.45
78412.7	82777.83	37190.91	29101.49	10947.93	30074.9	10270.14	40600.37	79265.25	61293.92
74461.41	86217.45	32455.21	25458.73	12506.26	32128.12	12205.9	39212.03	48864.84	60178.72
74759.76	95252.11	32112.95	27648.37	9704.504	20850.08	13439.01	31251.38	44365.89	54441.1
73109.4	86851.21	30458.51	29316.25	13254.39	23309.08	11757.43	36322.93	37037.19	55043.15

LN265.950	LN265.950	LN265.950	LN265.950	LN265.950	LN265.950	LN265.950	LN265.950	LN265.950	LN265.949
85598.09	69475.53	90945.65	67165.62	56655.26	72949.15	73544.45	73557.75	61639.96	38845.92
82862.89	69274.85	94223.43	77451.71	58206	70727.1	66641.54	66797.84	70914.6	41369.36
81214.01	67165.23	91976.52	69458.25	54042.3	67921.6	71007	64390.16	62253.76	39582.17
74810.86	68185.5	93961.36	76784.87	56053.7	67705.47	64445.15	56316.68	59532.65	35790.51
71796	60392.6	86125.17	67836.41	51166.71	64811.54	63544.68	59055.98	51907.22	36155.28

LN265.950	LN265.950	LN265.950	LN265.950	LN265.950	LN265.950	LN265.951	LN265.950	LN265.950	LN266.985
65910.64	59215.49	87526.24	61316.79	46357.03	63639.83	55973.09	56954.45	68759.22	26381.92
67343.79	55949.04	97127.01	62176.83	46913.59	60782.22	62679.13	61377.61	62164.41	27704.28
71239.83	59271.38	87038.02	59965.05	42288.53	58986.47	58413.04	56810.46	63938.26	26386.55
68383.36	54260.93	88481.9	55323.22	41747.04	62110	51362.23	61654.44	63656.5	24602.38
62626.91	51242.42	80032.49	49675.66	46720.42	55722.84	54832.57	55379.14	60052.01	23459.54

LN266.985	LN266.985	LN267.005	LN267.005	LN267.005	LN267.005	LN267.005	LN267.847	LN268.984	LN268.985
37889.07	49962.09	49243.68	44415.9	37088.07	35961.1	35200.28	15162.87	47749.15	59687.75
44044.59	50949.85	53637.35	50410.62	38345.75	34436.44	33533.31	11826.59	58250.34	45132.64
34737.85	53145.09	54670.2	49639.54	40633.33	38520.43	35249.36	11080.36	53225.09	60588.73
40010.16	52225.15	53691.75	51944.33	46545.78	38321.28	38154.2	14068.19	46418.89	71881.12
38588.9	52095.34	31066.6	28801.63	15968.25	18243.68	35409.04	15476.23	47497.88	52414.59

LN268.985	LN268.985	LN268.985	LN268.985	LN268.985	LN268.985	LN268.985	LN268.985	LN268.985	LN268.985
24210.09	42113.23	38222.41	30563.04	42035.23	55015.38	46216.66	68885.9	52070.4	33568.84
25120.29	34745.45	35535.8	24092.74	46983.62	38613.37	55240.42	67654.52	52105.67	42444.18
32707.64	36055.41	45728.78	22228.47	58617.95	29592.39	63280.5	65754.63	51067.17	43663.53
31484.85	40671.82	29078.39	18723.56	60252.59	62576	52308.88	65607.18	52908.18	42413.6
31308.94	35734.26	35227.71	23689.34	48872.65	38475.58	65016.79	56264.76	43991.36	40333.9

LN268.985	LN268.985	LN268.985	LN268.985	LN268.985	LN268.985	LN268.985	LN268.985	LN268.985	LN268.985
86909.94	102485.5	80288.27	79227.94	43855.58	20962.3	51503.32	25090.36	55685.41	75153.36
84487.48	82094.94	53116.76	53863.76	40818.9	28418.15	50227.5	19042.33	60989.91	63931.64
102880.7	77636.22	51471.52	56848.83	44681.55	22269.36	62312.29	12819.06	86941.72	58034.34
77960.38	84291.18	39631.77	52535.93	25654.59	32291.1	73587.18	20297.02	47673.27	65769.48
110696	90678.15	47345.76	54792.14	36574.45	32531.91	45237.46	19693.07	73220.75	104184.2

LN268.985	LN268.985	LN268.985	LN268.985	LN268.985	LN268.985	LN268.985	LN268.985	LN268.985	LN268.985
63953.39	59967.89	68498.66	35858.85	42354.19	89675.13	30287.43	64278.46	19698.24	49289.71
47958.19	53216.53	53272.77	26987.18	43200.37	83528.29	38538.19	58183.33	22957.44	39912.09
48065.69	50209.68	51759.61	33610.51	43932.87	64413.56	47350.68	75293.38	21145.64	36319.25
69113.28	68315.42	56960.24	23469.82	37183.51	91930.92	42811.24	45073.36	28143.03	31051.71
49125.12	55398.04	56848.93	23882.22	38281.82	108588.8	48793.24	62830.82	18690.01	35105.32

LN268.985	LN268.985	LN268.985	LN268.985	LN268.985	LN268.985	LN268.985	LN268.985	LN268.985	LN268.985
66925.26	89824.7	16779.12	37663.66	42580.04	41307.86	56834.19	23024.39	70547.41	21501.95
75545.84	65919.65	19609.7	37734.89	35854.02	41436.73	50804.61	16854.1	58497.79	30703.21
120125.3	60221.62	17980.78	39518.51	32901.54	41659.27	71195.12	15314.67	89667.29	37701
80097.64	76175.54	18801.62	26624.01	31081.05	26033.86	52997.45	19605.79	70044.69	32254.81
68118.95	61907.72	14185.63	37598.86	29300.25	43697.39	52463.71	18204.78	65094.45	27181.72

LN268.985	LN268.985	LN268.985	LN268.985	LN268.985	LN268.985	LN268.985	LN268.985	LN268.985	LN268.985
33692.6	45386.65	39816.27	24579.94	49941.59	36217.32	73149.85	39219.03	70482.77	27847.31
37284.6	36905.93	44126.13	23371.96	60941.6	27645.99	85869.06	36093.01	65295.34	30013
34118.34	51031.11	38378.8	27277	64336.24	38742.58	103478.8	52716.52	82450.98	27642.1
23385.22	38475.16	45158.77	19075.29	51487.39	25609.74	145524.6	33789.63	77544.32	32894.78
41743.79	34369.85	48664.44	21413.72	49815	35288.03	80555.87	37773.48	57265.83	61101.62

LN268.985	LN268.985	LN268.985	LN268.985	LN268.985	LN268.985	LN268.985	LN268.985	LN268.985	LN268.985
68768.18	68543.25	20135.84	85879.95	42919.94	42738.89	46976.56	48700.99	40924.45	55654.59
64410.13	81610.5	21364.57	75361.22	42239.91	45872.07	48467.19	55981.39	53121.16	66836.33
74102.93	86480.17	20266.63	61327.35	47825.41	48041.03	44695.51	55953.42	47668.46	65945.62
73703.64	86482.03	23481.95	69921.05	47418.66	38260.36	36581.49	71563.52	46910.11	59129.22
76611.42	98534.27	19202.42	92038.15	53446.02	45841	46803.73	64719.55	58459.47	68617.26

LN268.985	LN268.985	LN268.985	LN268.985	LN268.985	LN268.985	LN268.985	LN268.985	LN268.985	LN268.985
53559.92	37253.1	23021.77	19058.18	39934.77	63975.74	46685.31	35753.6	33718.88	25852.64
41370.72	36913.99	21902.47	18106.6	34760.39	60646.38	28905.72	43620.49	34274.43	22261.54
35728.67	35862.64	23281.79	17144.33	39084.81	56000.76	33330.7	44360.4	33114.3	21277.38
52114.97	27997.5	20451.04	20757.23	34268.9	59048.74	35884.84	35734.62	45797.11	21946.2
56611.38	29549.16	18435.27	14674.42	32149.98	56060.98	50426.25	52496.69	36334.89	18679.77

LN268.985	LN268.985	LN268.985	LN268.985	LN268.985	LN268.985	LN268.985	LN269.212	LN269.211	LN269.212
16517.61	32298.24	53896.34	17422.46	19848.03	27386.76	76252.31	15932.81	53912.23	38093.58
24534.7	34693.25	59426.2	21630.07	21015.37	37538	66393.66	12572.05	46994.58	37033.49
22604.74	29205.42	48419.71	24556.91	21480.97	37910.32	69198.29	13387.96	50463.81	33518.8
16691.7	37047.33	52381.6	18291.98	19497.77	39620.14	59290.78	15734.63	54597.75	38794.38
17914.83	34521.39	66229.07	20489.06	17337.14	35924.31	95822.69	14744.87	55479.08	40721.57

LN269.212	LN269.212	LN269.212	LN269.212	LN269.212	LN269.212	LN269.212	LN269.212	LN269.212	LN269.212
39100.7	35394.33	38664.64	34899.77	25784.43	33935.62	28239.98	32872.44	38206.04	22133.93
30688.34	37524.25	39381.97	34079.33	41122.34	30840.38	46408.06	38944.45	31275.17	21181.66
36439.44	35826.43	31099.51	32737.19	34430.66	33337.21	39583.13	42001.23	31333.47	20542.87
37567.37	38903.62	33118.89	34393.59	37743.12	35792.92	44143.09	41069.39	33262.73	22374.12
36524.28	40879.66	35001.09	36024.58	38427.06	36811.18	46937.29	41514.57	33610.28	23531.23

LN269.212	LN269.212	LN269.212	LN269.212	LN269.212	LN269.212	LN269.212	LN269.212	LN269.212	LN269.212
47337	34917.65	40879.67	28130.01	31597.25	22693.81	25417.63	38931.05	44913.78	28181.45
35495.14	41132.54	42544.62	32853.11	29122.34	25731.36	27448.06	32207.08	27914.28	29891.6
34397.32	30888.69	38025.48	29337.02	35727.16	24361.7	34270.66	34930.9	27849.65	30893.13
36619.98	34262.2	42875.95	32168.44	34587.89	27494.09	36455.66	38974.56	31969.01	32571.27
38335.08	35789.55	43353.84	33091.25	36395.12	24672.77	38978.43	39292.71	33754.21	38929.58

LN269.212	LN269.212	LN269.212	LN269.212	LN269.212	LN269.212	LN269.212	LN269.212	LN269.212	LN269.212
39665.81	30974.21	33923	35689.54	30691.37	25051.45	38787.57	35869.56	28173.88	31053.18
30624.4	34896.78	40410.73	36869.86	37748.49	30411.41	33340.78	32632.67	31974.17	33960.56
35464.34	39763.9	37699.95	34130.39	38927.84	27766.92	37738.31	34490.82	33259.37	38608.94
40006.41	45782.49	40978.17	33767.44	40223.63	30031.27	43673.07	34152.08	30719.56	36928.65
40741.87	47853.74	42845.48	36771.91	41126.88	29822.67	43837.41	35970.62	31735.58	39268.7

LN269.212	LN269.212	LN269.212	LN269.212	LN269.212	LN269.212	LN269.212	LN269.212	LN269.212	LN269.212
35906.41	36881.12	27874.53	31891.74	47217.23	35800.56	33298.84	33161.8	35744.72	33908.09
26197.38	39126.01	35196.49	26289.29	29686.07	50754.58	39508.44	33907.28	35652.8	30071.99
31644.14	31346.59	36722.17	30775.96	33580.28	39237.3	36091.25	35923.97	36781.87	30055.21
33621.14	32950.68	38123.38	34674.75	36821.69	43262.1	35666.46	35969.7	39381.8	35358.65
35541.1	32867.56	38920.09	34211.55	34554.97	46676.18	39562.38	36972.63	38019.84	35867.06

LN269.212	LN269.212	LN269.212	LN269.212	LN269.212	LN269.212	LN269.212	LN269.212	LN269.212	LN269.212
22634.18	32310.93	34097.41	27522.21	40132.43	38933.8	33823.59	34857.7	17226.09	28155.85
18837.02	37485.14	16715.88	31399.73	27589.09	40553.56	30383.6	35415.59	22151.33	29873.83
20527.41	35635.39	20903.96	34830.5	32565.36	39407.93	36730.85	36246.35	18588.13	33136
18603.4	41160.77	23237.89	37231.86	32390.42	43613.1	39415.87	37488.22	20370.86	32883.86
20601.65	38078.55	25340.74	39558.81	34954.51	41249.27	42089.71	40612.16	21084.26	36086.88

LN269.212	LN269.212	LN269.212	LN269.212	LN269.212	LN269.212	LN269.212	LN269.212	LN269.212	LN269.212
36493.81	29629.38	32405.24	26308.12	37388.54	15490.36	32634.38	31896.69	17393.44	26523.7
40060.64	26836.76	29206.8	33483.36	30397.8	23603.32	38100.85	33363.98	14175.67	24537.38
44706.27	32490.35	36976.27	30055.57	40663.22	20414.55	45951.53	34464.51	18395.99	24940.79
44337.73	37546.36	38704.57	32536.51	42301.32	22615.48	48243.04	35127.71	19235.84	27728.07
47944.19	38501.29	39618.79	33241.47	44479.79	20392.69	49310.29	37149.06	18910.26	25978.83

LN269.212	LN269.212	LN269.212	LN269.212	LN269.212	LN269.212	LN269.212	LN269.212	LN269.212	LN269.212
33644.3	31932.48	37670.31	30365.66	40037.13	32599.33	40122.98	37490.48	33499.1	37505.17
31249.41	16937.95	47248.49	40924.35	45381.89	34519.82	33152.24	32547.87	31034.6	31560.4
34366.62	21353.98	41063.49	41172.47	40474.61	36550.71	32958.9	39689.11	30971.98	33349.32
36079.39	20562.18	44273.53	45573.68	42995.26	38429.4	35643.74	39936.45	36045.97	36898.8
36971.84	21578.48	47278.1	41074.02	47977.41	37723.08	36818.89	43195.32	40821.49	36818.39

LN269.212	LN269.212	LN269.212	LN269.212	LN269.212	LN269.212	LN269.212	LN270.980	LN270.980	LN270.980
56315.19	28838.35	28885.94	31530.15	29971.61	27248.91	34750.69	32585.69	32753.48	27634.12
42789.26	35387.07	38486.07	32180.02	28103.19	26834.57	32408.93	30543.23	30469.42	28784.02
50210.7	38076.37	33655.56	35069.34	33362.34	33464.64	34170.36	34491.15	31161.13	27909.08
54384.22	39724.53	35623.54	37594.93	37254.18	36062.14	37225.23	33389.61	32600.73	27902.04
56311.77	43906.93	39914.07	38719.21	36462.17	35371.14	40065.97	31786.31	29758.98	29129.36

LN270.980	LN270.980	LN270.980	LN270.980	LN270.980	LN270.980	LN270.980	LN271.923	LN272.98_	LN272.98_
37537.69	27950.76	17612.14	35466.31	29494.13	26227.95	25851.92	17597.8	33876.59	50942.25
39591.16	28480.81	19378.09	35195.91	33040.35	24824.19	24582.85	15220.2	16681.76	25973.94
38031.2	28402.56	19000.87	38434.4	35401.61	25935.37	28733.75	15707.51	36857.93	49115.86
41080.79	28612.28	19929.65	37588.69	37688.98	28445.19	28201.4	12458.39	36540.54	53707.64
37232.09	30579.18	18836.21	39251.85	33094.56	28527.88	26378.76	11466.93	34656.72	49912.13

LN272.98_	LN273.016	LN273.016	LN273.016	LN273.016	LN273.016	LN273.016	LN273.016	LN274.858	LN274.889
39897.47	29925.83	37576.21	66592.95	41789.86	29182.92	41895.47	27432.48	14121.43	14036.46
15788.88	30783.33	34299.28	67709.42	43513.62	34888.49	41474.55	29744.48	9999.08	15694.16
39377.19	29082.25	35608.95	68935.6	46339.5	27695.56	41223.49	28610.63	15959.24	18039.1
37060.49	33256.46	36139.52	65642.75	44958.43	31977.77	45452.53	27900.81	19507.58	17791.63
36266.59	33782.8	33754.91	68316.44	42721.92	27942.81	44573.49	29037.93	14907.65	19481.66

LN274.960	LN274.975	LN274.975	LN274.975	LN274.975	LN274.975	LN274.975	LN274.975	LN274.975	LN274.975
12513.13	26160.68	15192.34	25891.07	23835.78	19893.05	33019.46	28798.1	27560.02	24149.35
11286.87	24752.71	16756.09	23668.31	25415.41	20484.76	35759.54	30216.68	29654.99	23821.45
9615.634	27981.48	18433.27	22521.65	22089.36	18457.55	33909.68	26673.7	26585.11	21686.99
10967.32	24547.91	14672.14	24051.23	24468.03	18822.48	33260.71	32312.7	24784.05	25303.04
11810.47	26330.96	14977.77	24843.57	22228.52	16833.94	36485.32	28419.7	28891.96	21563.84

LN274.975	LN274.975	LN274.975	LN274.975	LN274.975	LN274.975	LN274.975	LN274.975	LN275.113	LN275.113
24315.75	26722.04	17404.67	33339.69	36390.85	20757.85	25002.28	30039.15	16570.16	20895.91
25084.09	26429.47	16762.4	36994.02	37707.93	20227.41	24156.7	30204.08	16250.7	18174.96
25408.86	28757.36	18420.52	31602.03	37104.48	18978.95	22900.45	27648.3	18527.22	19381.82
27949.07	28632.18	16599.29	33322.62	38464.58	21308.22	26671.16	28403.97	16059.94	18337.7
25461.06	26614.91	18181.97	31668.44	36172.07	21411.41	25894.89	25776.27	15501	17464.14

LN275.149	LN275.149	LN275.879	LN275.905	LN276.990	LN276.991	LN276.991	LN276.991	LN276.991	LN276.991
23102.43	16288.42	85129.04	60276.6	76037.6	70918.17	41353.73	63323.94	55649.22	19500.4
25466.92	17745.79	87278.71	60969.11	98067.05	70340.63	56453.12	52442.96	47520.02	14752.82
22980.05	15926.05	93858.29	45930.5	82291.31	49122.78	42478.05	61010.98	50506.92	18322.82
23716.36	14462.26	58361.31	65835.36	128214.2	66388.63	45155.51	70361.34	60716.6	14639.73
21638.99	14795.83	50610.22	69114.65	130647.2	57822.3	44384.08	66858.12	56647.91	17557.31

LN276.991	LN276.991	LN276.991	LN276.991	LN276.991	LN276.991	LN276.991	LN276.991	LN276.991	LN276.991
87208.61	99865.34	25362.87	72237.58	171043.8	103146.6	90433.04	50434.26	63247.79	118727.6
80212.27	77196.67	49126.46	50754.34	112482.7	86084.24	58549.44	40250.04	60041.81	111198.3
89248.46	95276.9	25304.26	66095.78	91989.44	60120.74	77434.09	62259.21	88587.89	115590.2
79724.1	84127.46	25035.07	50503.47	89759.24	50662.06	78546.32	39153.18	62595.66	166678.4
79938.06	95569.51	33532.41	62206.83	95064.91	70590.53	78096.34	52781.17	53944.12	154686.8

LN276.991	LN276.991	LN276.991	LN276.991	LN276.991	LN276.991	LN276.991	LN276.991	LN276.991	LN276.991
73061.06	37355.04	49357.81	48576.8	131635.5	89155.62	101507.3	71277.24	18657.29	63271.15
93792.05	36234.02	36619.04	32466.98	77270.17	62284.74	100200.1	107230.9	25670.85	59655.13
87875.35	29701.74	33583.81	36964.48	83293.65	82826.97	106282.8	68884.63	25233.78	68638.34
134505.2	34923.96	34574.9	37954.42	115457.8	65948.35	108983.3	92526.23	26253.05	59658.92
87143.5	26786.61	42278.2	31698.68	109798.4	97678.48	95975.67	77219.03	31794.32	59672.56

LN276.991	LN276.991	LN276.991	LN276.991	LN276.991	LN276.991	LN276.991	LN276.991	LN276.991	LN276.991
88864.87	98809.07	66116.86	72838.49	58105.14	47951.09	93779.66	150258.8	40331.22	31462.21
119713	135992	63604.46	73075.77	50948.03	50503.82	71254.9	114142.7	30410.39	26735.12
102521	82943.03	87030.2	118398.9	70085.61	62776.22	63435.14	100201.2	25351.76	31774.19
101881.8	131132.6	57206.87	63131.97	45784.62	72735.38	101139.5	139161.7	36159.24	30024.67
136861	107346.3	64422.22	86754.05	76782.78	59875.06	81303.61	173067.2	28147.84	20525.17

LN276.991	LN276.991	LN276.991	LN276.991	LN276.991	LN276.991	LN276.991	LN276.991	LN276.991	LN276.991
189438.3	34660.02	179325.9	55394.22	124031.5	45134.33	142939	125334.7	82958.26	44116.64
127718.2	30796.58	105214.5	48149.62	115613.1	46539.26	103502.5	80145.76	95392.68	58192.79
122721.9	42873.15	134249.3	51980.02	117071.5	52814.78	99016.47	148026.4	110308	51485.57
110615.8	35690.39	197061.3	80644.72	73985.48	45884.09	141648.3	78583.41	96851	63053.35
218137.2	32892.61	97807.84	47541.02	131231.4	40665.11	134117	69827.82	74672.2	75510.68

LN276.991	LN276.991	LN276.991	LN276.991	LN276.991	LN276.991	LN276.991	LN276.991	LN276.991	LN276.991
105517.8	89093.02	71069.09	89278.27	79018.16	56685.8	53590.08	129552.7	46762.54	80736.39
89683.53	71211.49	75685.41	116561.4	52828.31	52999.27	51853.57	168856.3	46885.56	92273.66
112708.7	115987.4	62482.38	87707.14	66044.01	62739.37	54910.33	158878.4	49602.95	74127
144910.6	76701.58	63086.53	85974.89	83051.16	43373.41	55510.82	168497	60164.56	49867.47
79690.58	76661.08	44657.42	131043.2	70846.69	53027.27	60877.33	116109.7	62292.6	49719.83

LN276.991	LN276.991	LN276.991	LN276.991	LN276.991	LN276.991	LN276.991	LN276.991	LN276.991	LN276.991
54432.01	39640.24	150129.9	58819.94	42899.41	109417.8	105871.9	76315.75	25474.87	37696.11
58904.05	21231.19	152940	73140.47	57047.82	81567.2	116882.2	82050.64	35518.47	37600.95
51608.08	20306.4	125269.6	63916.56	61924.3	90206.12	138937	86280.85	21146.32	49219.52
38391.63	19975.98	138976.3	43818.64	62606.81	102854.8	115705.8	95828.25	23835.01	46299.77
45774.97	28673.34	152843	80499.12	42956.29	68606.36	89972.68	61448.49	28464.44	44060.49

LN276.991	LN276.991	LN276.991	LN276.991	LN276.991	LN276.991	LN276.991	LN276.991	LN276.991	LN276.991
63603.98	78596.56	48969.78	132472.8	87821.09	125661.8	70985.98	56025.31	101170.9	94939.13
65514.05	98782.1	60670.58	107489.4	111747.3	79310.92	60105.91	59148.55	85546.75	110141.3
53708.19	55961.36	48352.55	93655.82	93388.77	66239.39	55747	47106.78	109610.7	84645.73
64094.24	95265.52	55199.75	95167.18	91340.81	90760.85	68512.35	53998.89	76019.08	119526.7
70963.27	80951.04	56313.74	85297.25	83419.47	60700.59	73995.29	66846.32	98046.5	66655.08

LN276.991	LN276.991	LN276.991	LN276.991	LN276.991	LN276.991	LN276.991	LN277.128	LN277.128	LN277.128
91754.79	45715.42	87031.45	142625.6	83303.47	55597.04	74880.88	26113.48	17564.6	20125.19
89288.97	58000.62	73945.54	131528	61444.52	65011.84	68959.11	23435.1	18013.45	20861.9
133431	42340.11	136484.8	136918.6	76555.99	63938.13	58273.13	24235.62	16504.79	20473.78
109161	58540.86	102346.4	173091.8	70128.93	76572.19	74675.11	24552.56	15306.75	17924.56
108449.1	45306.5	73695.89	139830	88699.88	56155.78	95455.7	25246.48	15749.99	19515.21

LN277.128	LN277.876	LN278.902	LN278.909	LN280.966	LN280.966	LN280.966	LN280.966	LN280.966	LN280.966
16488.78	11080.11	13639.65	13155.65	44840.17	61355.07	52680.63	39829.69	35585.99	42789.58
14960.67	10684.57	12706.87	14549.48	49559.06	59420.34	52783.56	36480.55	37910.97	43997.18
15593.86	11238.52	15284.78	14405.58	46025.75	55400.23	56160.61	39999.83	38198.14	45450.8
16427.31	14037.55	16562.09	13488.72	50712.72	54889.34	52557.29	36570	38745.27	45298.32
16885.15	16400.88	18691.03	12559.55	44462.37	58537	54946.89	39032.31	36181.01	48566.45

LN280.966	LN280.966	LN280.966	LN280.966	LN280.966	LN280.984	LN280.984	LN280.984	LN280.984	LN280.985
41145.26	48264.7	58332.77	37529.53	36970.28	37246.3	15293.08	17194.98	11416.32	12458.1
41264	49154.86	64353.27	33002.58	32831.1	36133.78	14094.04	18959.75	13771.74	11514.51
40106.14	50903.64	62608.38	33352.98	37430.69	41701.44	15200.48	18932.09	12309.84	13272.33
46594.61	50236.4	61273.87	37681.32	38557.81	34395.01	14830.68	20848.8	13052.42	11678.44
41623.83	52326	61712.4	37872.44	38724.76	36381.91	13720.38	18014.32	11455.84	11471.68

LN280.984	LN280.985	LN280.984	LN280.984	LN280.985	LN280.984	LN280.985	LN280.984	LN280.985	LN280.984
30296	17149.29	19306.85	20420.76	16728.23	25161.31	26514.8	15333.92	23799.04	34179.87
32789.91	18273.7	22993.55	23254.43	18299.59	24143.1	28851.44	14804.11	23951.45	37431.15
30993.47	16608.7	20981.37	21609.46	17168.09	25199.86	27088.58	17714.64	23901.61	34444.29
30591.39	16047.72	20081.68	22565.29	16913.88	25474.52	27872.14	16576.18	25283.13	33614.57
34553.91	16669.1	21530.99	17712.54	17629.7	25222.91	27383.22	15833.34	22299.58	36233.46

LN280.984	LN280.984	LN280.984	LN280.985	LN280.984	LN280.984	LN280.984	LN280.984	LN280.984	LN280.984
28723.29	25739.91	41131.03	11450.3	15219.22	20660.94	20103.76	13796.41	33411.7	19049.44
29283.12	25277.28	45343.92	11962.62	14650.47	21720.57	18737.3	13560.89	37252.95	17543.34
33716.49	25964.07	40572.59	13402.33	15132.41	20310.58	18461.23	14155.48	37615.42	17466.74
28322.98	25894.28	46850.28	11865.49	13497.21	22552.3	18133.44	13315.06	37418.6	15061.47
30818.41	26173.47	40477.44	12395.47	13884.49	20388.68	16262.52	13138.02	35293.29	19153.22

LN280.985	LN280.984	LN280.984	LN280.984	LN280.984	LN280.984	LN280.984	LN280.984	LN280.984	LN280.984
25261.27	40473.08	33213.79	42662.28	26267.33	17923.89	25186.21	25452.3	25134.55	37580.46
25703.34	40863.75	34767.29	46583.82	25637.5	19622.76	23966.85	24876.02	25015.29	34515.7
23974.9	39718.93	33824.51	39215.41	27196.08	20434.68	25585.56	26684.88	24675.64	34493.03
26236.97	42141.22	35173.49	43211.59	29951.09	21712.49	27585.82	27379.12	23548.59	32104.27
25534.71	39262.61	36634	47578.08	28041.52	21145.92	26987.14	26768.79	24560.86	36950.75

LN280.985	LN280.984	LN280.984	LN280.984	LN280.985	LN280.984	LN280.984	LN280.984	LN280.984	LN280.984
13947.62	23417.96	13841.89	27820.56	24675.32	38735.5	20106.22	33628.19	18354.97	33099.55
15167.57	25581.52	14120.72	25732.91	23049.97	39949.29	19203.71	30579.83	20231.75	32024.86
16918.69	23357.15	14354.02	25489.54	27693.7	40602.47	19312.19	31424.33	19342.2	40179.92
16847.42	26186.65	13149.86	23058.26	25377.54	44202.67	16280.49	29389.92	20719	33666.92
18002.39	22623.27	14410.47	24975.21	20644.26	40090.25	16476.57	32783.53	18108.08	34361.06

LN280.984	LN280.984	LN280.985	LN280.984	LN280.984	LN280.984	LN280.984	LN280.984	LN280.984	LN280.984
38697.85	34711.51	12929.47	29153.49	28266.81	15632.72	33371.91	19782.03	34930.21	17708.13
36653	33594.34	13251.1	34180.91	27360.31	16847.33	31723.36	17986.56	36035.67	19672.88
34269.68	32160.41	15002.98	29475.63	26684.97	15328.04	31353.54	19029.46	37695.33	17215.78
37879.21	28685.57	12987.81	29104.39	30552.45	16105.74	31866.52	19841.59	38493.71	16944.8
36942.64	34127.62	11699.41	31167.43	27833	14262.27	32482.06	18578.08	37621.89	16678.47

LN280.984	LN280.984	LN280.984	LN280.984	LN280.984	LN280.984	LN280.984	LN280.984	LN280.984	LN280.984
22405.73	20751.44	42251.54	21184.33	27417.07	31023.9	40542.76	36927.75	18775.19	32316.62
22530.39	23053.22	39885.98	19505.42	25481.88	32939.19	43198.01	38835.61	17339.69	32999.05
23481.7	22435.85	43081.9	18816.87	26348.83	29087.62	42973.78	38594.45	17925.79	35513.59
25028.95	22154.04	41133.61	19890.34	28180.29	31702.81	43097.91	34662.35	17249.58	35063.22
23620.58	23495.04	41833.23	18790.84	27418.88	29732.8	40141.13	42228.46	17505.31	33427.96

LN280.984	LN280.984	LN280.984	LN280.984	LN280.984	LN280.984	LN280.984	LN280.985	LN280.985	LN280.984
21903.68	26238.04	21749.19	37194.65	25489.69	35971.83	34306.88	20375.24	23150.79	40103.78
21654.92	27127.16	19299.86	34630.85	26906.7	31400.02	34347.79	19968.44	21375.32	40406.59
21938.24	26814.25	20093.24	34674.04	26699.27	34844.97	32200.49	21065.19	22697.88	40105.67
22506.63	28406.31	20878.76	36081.7	25106.94	33413.21	36218.91	19819.47	21037.8	41219.71
21464.47	25204.85	21760.03	34039.64	25719.51	37317.51	35399.32	21438.18	17872.26	44131.7

LN280.984	LN280.984	LN280.984	LN280.984	LN280.984	LN280.984	LN280.984	LN281.248	LN281.248	LN281.248
16383.97	46001.19	34940.74	36772.49	23196.66	28568.6	39188.41	45215.08	20885.67	16388.94
13655.15	46851.64	32790.28	36073.79	23326.01	27980.56	36597.58	50209.46	18332.31	14144.59
15039.44	43566.99	31843.1	33570.95	21053.14	27947.4	37376.58	58554.16	26181.51	18352.32
16428.12	48763.12	32576.82	38035.86	21270.83	26720.16	37726.24	56690.16	29311.92	24048.62
13495.39	47007.34	31457.25	35736.37	21711.41	30373.57	37523.66	57125.19	31477.27	24386.17

LN281.248	LN281.248	LN281.248	LN281.249	LN281.248	LN281.249	LN281.248	LN281.248	LN281.248	LN281.248
27675.2	40918.45	30441.43	20280.87	31348.14	18106.95	27503.93	25349.11	31519.6	27572.71
28738.97	40887.45	32845.82	18275.53	27988.45	18104.34	29010.67	25168.64	29862.31	27884.19
34530.45	47241.99	37384.97	20244.51	38349.65	24315.72	34018.96	31650.26	38235.09	30787.68
38470.53	53832.65	41703.8	25556.69	41471.69	27620.64	38893.98	34922.51	44100.81	34544.07
40408.8	59527.95	46886.86	26131.27	42752.87	30715.66	42481.45	37978.67	47042.08	37285.91

LN281.248	LN281.249	LN281.248	LN281.248	LN281.248	LN281.249	LN281.248	LN281.248	LN281.249	LN281.249
36353.76	31201.68	15664.64	31741.1	23103.4	23444.26	28099.92	30746.59	22959.87	25367.44
34175.1	32492.79	15163.15	28000.47	23792.01	24355.41	26165.48	33252.5	22482.62	25343.8
40474.22	37492.72	19542.32	30745.68	28520.18	27343.7	31925.43	38012.55	24667.72	28315.63
44271.3	40383.83	25011.79	35280.22	31925.58	32069.41	37825.57	44122.51	30228.42	31738.27
48229.25	44700.21	28664	40679.12	32561.62	32932.09	37787.46	46345.33	31057.63	33859

LN281.249	LN281.248	LN281.248	LN281.248	LN281.248	LN281.249	LN281.248	LN281.248	LN281.248	LN281.249
24821.86	35690.05	34011.38	43034.26	33987.76	28062.63	31720.38	27977.44	25055.65	26700.05
22673.78	33464.7	31273.98	38901.86	34076.05	28473.06	30436.01	26358.51	28755.04	27958.8
27813.64	42566.31	36672.4	47028.27	38780.58	32484.21	35235.79	31608.9	34981.03	33209.9
32460.37	49856.05	43806.66	55176.25	47821.38	35221.88	41782.45	34591.73	37236.98	38359.34
34051.22	51319.78	48415.17	60945.19	46838.37	40487.42	43970.84	38549.67	42250.3	42013.85

LN281.249	LN281.248	LN281.248	LN281.249	LN281.249	LN281.248	LN281.248	LN281.248	LN281.248	LN281.248
31877.01	43772.15	28038.83	14736.59	24079.42	27102.39	27139.66	38478.28	29624.76	34410.02
33327.25	37450.81	27470.14	14184.02	24850.85	26693.5	25512.96	38342.73	26368.87	34944.62
33758.26	47722.5	35341.3	15726.78	29416.76	35241.38	30048.29	47478.84	35073.95	39820.48
40350.23	55112.96	39524.55	22773.24	34526.28	38338.9	33173.13	51220.61	39129.7	47199.69
41163.06	58918.11	41292.8	23749.35	37818.68	38463.05	36187	56065.24	42241.96	51446.69

LN281.248	LN281.249	LN281.248	LN281.248	LN281.249	LN283.264	LN283.264	LN283.264	LN283.263	LN283.264
44098.5	23748.02	33931.07	39652.61	33479.81	691858.1	80089.81	43292.16	101221.8	261442.9
45151.19	22488.59	35124.05	40821.08	31596.78	633683.3	84946.54	42958.36	143833.6	239943.2
51489.7	28038.19	38070.03	48419.28	35691.11	764543.3	98894.63	58009.1	133086.7	191287.9
60437.56	30957.09	45981.43	51722.05	39686.92	645431.7	101267.5	60656.99	141734.2	206733.8
61945.12	34728.91	48525.52	51633.21	42986.97	756550.9	118625.5	66725.87	146212.2	209071.9

LN283.264	LN283.264	LN283.264	LN283.264	LN283.264	LN283.264	LN283.264	LN283.264	LN283.264	LN283.264
113934.8	170431.7	87791.19	109348	152368.4	99112.44	157466	138223.1	113665	81392.86
121358.2	136585.2	146710.1	90144.5	152736.2	117831.3	157633.6	119120.2	141118.1	100478.4
110863.9	136713.5	102110.5	133413.2	150112.8	117018.8	118504.2	91541.16	149679	107455.8
129658.1	161432.5	115086.5	125116.6	146370.3	128938.1	152871.4	125899.7	122053.5	115678.4
136066.3	193283	118707.7	120273.6	159230.6	129523.8	166773.8	119289.8	122995.3	133232.2

LN283.264	LN283.264	LN283.264	LN283.264	LN283.264	LN283.264	LN283.264	LN283.264	LN283.264	LN283.264
50096.24	107120.2	92165.66	112733.9	76220.11	169779.9	134724.4	68228.94	127831.4	52719.27
61377.1	120996.7	105077.5	142111.8	71609.58	136875.4	114096.6	75503.79	131675.8	50484.65
67882.97	105280.6	94648.05	168413.4	101359.7	163036.4	121779.7	79820.03	120760.8	55143.95
75570.03	122516.7	93643	151163.9	95045.17	167989	117845.5	83219.81	146390.6	61403.98
76050.19	126683.2	103828.2	157669.7	111662.7	196549	135318.3	93119.77	179002.6	62978.69

LN283.264	LN283.264	LN283.264	LN283.264	LN283.264	LN283.264	LN283.264	LN283.264	LN283.264	LN283.264
142014.6	119352.4	67219.83	99767.09	96640.74	76694.3	62033.94	92187.17	96653.93	88816.45
112781.8	101395	59936.02	108208.2	95559.49	93503.55	54456.07	165293.1	107309.4	114671.4
120563.6	133470.5	71605.92	115975.3	122804.9	90452.87	64888.31	118924.1	123359.5	107000.3
120135.4	116662.5	84484.81	134533	139196	105855.8	68412.7	124658.7	136060.5	113576.8
154041.5	116390.7	87208.89	133366.4	146884.2	159172.4	69820.47	138155.8	138762.7	135618.6

LN283.264	LN283.264	LN283.264	LN283.264	LN283.264	LN283.264	LN283.264	LN283.264	LN283.264	LN283.264
80672.69	106311.2	105533.6	81251.75	126639	96465.86	51693.58	110535.4	100147.5	98708.33
99102.37	98792.52	116406.6	73945.79	105456.7	108227.2	61547.02	106303.9	117006.2	104860.8
100337.1	133733	139090.7	89969.67	121418.4	100434.1	67533.82	127148.2	142281.4	117200.6
121329.4	134541.1	163765.9	100685	142141.2	124690.3	81838.15	132933.1	138041.5	115207.1
140337.9	171873.1	179712.3	115290	170494.9	120859.5	88923.24	134278	169878.3	152612.7

LN283.264	LN283.264	LN283.264	LN283.264	LN283.264	LN283.264	LN283.264	LN283.264	LN283.264	LN283.264
73123.79	108881	130876.1	112159.6	48817.84	93468.14	118524.5	87495.53	101683.4	171137.2
57637.9	106200.9	143431.2	92458.25	54845.97	97435.52	159445.6	101400.6	83026.15	160163.9
55784.99	123789.8	197825.5	121252.2	59651.34	107594.9	137717.8	97018.91	130260.6	187168.2
68319.1	121434	165648.7	103960.9	71655.25	105904	159928.4	119123.1	116277	188481.8
74692.64	144767	161410.6	117070.1	73178.97	115438.1	155993.4	134204.2	116033.9	161242

LN283.264	LN283.264	LN283.264	LN283.264	LN283.264	LN283.264	LN283.264	LN283.264	LN283.264	LN283.264
107727.8	112184.5	79954.67	78800.71	85904.39	89581.37	100153.1	113651.3	105877.1	108740.2
94967.89	94524.89	97996.82	72497.49	96161.16	79765.09	84894.32	135597.7	105334	105971.7
117592.1	116738.5	94092.43	85054.54	119302	88282.59	95511.57	102461.7	111723.9	113695.5
119765.4	130024.6	104736	92831.18	122374.6	103520.9	109874	126638.8	121536.4	122462.9
132007.6	129142.7	145388.7	97941.36	127925.6	111794.1	112349.4	129643.5	130153.9	140536

LN283.264	LN283.264	LN283.264	LN283.264	LN283.264	LN283.264	LN283.264	LN283.264	LN283.264	LN283.263
83637.76	52448.25	109996.4	71263.59	220127.6	126776.3	155062.7	90269.48	54648.53	135842.8
91268.15	42573.27	113516.4	74937.55	152985.3	108288.4	176258.9	93517.67	48235.7	132536.5
93107.82	57685.31	100953	78569.42	178970.5	113310.5	156969.5	103030.7	62525.59	138467.3
104225.4	68901.34	104660	90411.11	198504.4	124733.7	166376.3	112488.8	65131.26	152344
108670.2	71429.45	124684.6	89521.38	189165	166945.1	172534.3	126176.5	70006.38	176055.5

LN283.264	LN284.267	LN284.267	LN284.267	LN284.267	LN284.267	LN284.267	LN284.267	LN284.267	LN284.267
70408.87	19818.8	16595.87	14988.61	16755.81	24595.49	21069.34	18013.06	19410.76	19426.18
68293.77	24361.86	15412.83	11918.59	16603	24997.02	21240.63	21045.49	19588.34	17674.71
71038.77	25053.93	20056.26	17381.81	17267.58	29173.57	26688.84	20641.29	22121.43	19553.52
76972.29	26086.26	22061.98	19565.65	22375.37	29832.09	24366.84	23200.01	21391.69	22843.43
94310.43	29795.11	23604.54	19881.29	21857.29	33721.3	27880.9	24442.75	23441.93	23894.17

LN284.267	LN284.267	LN284.267	LN284.267	LN284.267	LN284.267	LN284.267	LN284.267	LN284.267	LN284.267
16112.14	22297.39	14554.9	13800.75	38415.01	16548.39	13479.79	16576.9	16502.93	126433.7
14599.63	19149.15	15483.08	12659.77	39049.54	15470.93	12646.34	17570.25	18584.4	124353
16475.2	21664.47	16646.64	14309.69	41268.67	17810.75	13923.77	19220.81	20600.92	135601.1
16725.49	25853.99	18818.96	15574.93	41011.72	19805.1	17042.37	20762.38	23038.91	130461.2
19117.7	24016.77	19460.1	16448.74	45307.27	22316.84	16060.13	22560.97	25178.16	121368

LN284.267	LN284.267	LN284.267	LN284.268	LN284.267	LN284.267	LN284.267	LN284.267	LN284.267	LN284.267
16778.14	15110.31	15015.25	12972.27	20998.67	17437.95	16865.91	19535.86	21354.4	16803.16
11996.62	12980.49	14388.39	16026.27	17983.89	12596.06	17885.11	16670.28	21696.66	14375.13
15378.52	17145.7	15819.76	14951.86	19106.41	17953.3	20709.57	20883.26	23053.15	17021.03
15412.62	18899.55	19135.58	18258	23324.98	19135.98	23840.09	25442.01	25128.2	20585.78
20316.97	20570.67	18540.3	19101.13	20716.1	21366.69	21476.48	26173.58	26980.41	20117

LN284.267	LN284.268	LN284.267	LN284.268	LN284.980	LN284.996	LN284.996	LN284.996	LN284.996	LN285.206
19037.66	36266.93	34330.56	16346.54	36541.89	35213.95	41040.06	37551.31	57587.73	15314.86
20732.94	34160.48	30917.16	14978.15	39732.44	37123.47	40095.46	41262.81	51585.01	14527.42
23012.46	37891.57	36426.9	20698.46	38387.7	31044.49	35363.78	38670.43	51010.79	13158.55
24698.86	38991.66	37141.87	21970.96	40038.8	34133.86	39289.1	33526.2	51883.74	15024.19
22486.78	38452.14	38511.67	21328.87	37280.08	36881.07	40231.84	39283	48973.46	14504.11

LN285.206	LN285.206	LN285.206	LN285.206	LN285.206	LN285.908	LN285.908	LN285.908	LN285.909	LN286.992
12029.01	22024.27	17992.5	23537.27	13404.63	88527.05	19869.08	20511.56	17335.81	56568.99
11522.5	20852.38	16799.48	25318.26	15277.82	48703.58	20583.04	21222.5	16896.74	60522.34
13174.7	19256.13	17374.58	22457.76	11894.9	54246.56	18923.08	22747.58	17611.92	56954.66
14098.11	20965.43	16476.73	23570.56	11737.06	46425.01	18844.7	18694.1	17355.03	62656.67
13239.91	20599.49	19540.42	23220.52	12268.11	71107.71	21662.34	22839.9	18162.6	58995.91

LN286.992	LN287.15_	LN287.150	LN287.150	LN287.150	LN287.150	LN287.150	LN288.859	LN288.934	LN288.952
30401.15	21371.22	21360.32	17535.35	23958.37	28376.46	14969.08	23524.38	30136.76	34200.38
32650.23	21108.26	20897.3	15509.37	23139.41	29743.57	14277.54	17943.05	26097.17	33309.33
34285.07	18804.08	22016.13	16957.46	22552.36	28894.47	13072.28	19890.59	30206.77	36522.69
34758.05	19591.5	20749.82	19735.55	25471.22	29930.16	12878.18	22161.72	29674.34	31698.52
34049.33	16270.1	22201.38	16836.65	24624.72	29281.02	16253.8	24099.03	34071.46	28997.55

LN288.952	LN288.952	LN288.952	LN288.952	LN288.952	LN288.952	LN288.952	LN288.952	LN288.952	LN288.952
34063.56	41239.63	40384.91	26705.51	20272.21	51948.57	38535.77	32414.49	30033.1	45011.74
31218.54	45921.29	39797.7	22965.11	19978.54	52742.49	35725.24	34896.25	32234.52	46972.96
32736.04	42451.19	39937.62	24765.98	20106.78	51255.37	39285.82	32838.15	32260.82	46335.64
34457.69	44280.62	40179.56	23714.56	16776.03	56765.76	38104.4	32288.15	35080.49	46799.95
33596.3	38373.36	39409.25	25221.04	17503.53	52856.22	38091.28	33775.58	33190.15	42253.17

LN288.952	LN288.952	LN288.952	LN288.952	LN288.952	LN288.952	LN288.952	LN288.952	LN288.952	LN288.952
34814.66	27410.85	26732.7	46786.57	44942.55	37905	47032.16	42369.64	35017.68	36358
33978.21	27773.34	27698.68	44049.44	42666.51	35298.19	43239.91	40038.89	38118.84	37282.3
34161.06	30977.05	29544.8	45301.67	44193.49	36655.42	44114.19	41331.64	37593.5	35933.55
34436.54	31706.63	27635.96	44175.73	45268.42	37521.17	43317.85	37789.99	35296.27	36920.7
33126.22	28964.51	26776.4	45917.55	47231.69	36620.97	43352.45	41925.58	32906.49	39475.03

LN288.952	LN288.952	LN288.952	LN288.952	LN288.952	LN288.952	LN288.952	LN288.952	LN288.952	LN288.952
32715.66	33871.43	44630.62	44585.21	41659.32	50326.55	32439.78	43862.67	42241.01	30865.9
35589.78	29447.71	47113.02	46518.35	44301.88	52447.15	34513.66	43249.91	43685.72	30010.3
32848.4	27450.08	47455.19	51590.34	43479.97	50933.42	35202.97	40914.46	40730.97	30445.08
38822.47	30884.71	47020.53	48088.8	43046.48	51429.88	34546.14	44824.81	46568.69	32085.72
35597.22	29889.89	46547.27	43753.29	41299.23	50267.22	36657.72	40331.63	36027.19	31182.41

LN288.952	LN288.952	LN288.952	LN288.952	LN288.952	LN288.952	LN288.952	LN288.952	LN288.952	LN288.952
57126.94	26728.54	31881.05	32329.51	35307.6	49761.94	54411.33	39440.31	23383.64	32654.46
60639.2	27758.37	31210.8	30415.02	35603.43	51725.02	55389.9	42481.08	27383.99	34237.01
58409.52	24594.65	30317.1	30447.61	31835.02	47486.96	57179.75	42337.31	24485	32208.49
61006.05	24548.43	34056.77	29904.48	34659.5	49167.49	53336.76	41956.65	24133.05	33045.5
57429.64	26994.76	33297.72	29681.79	34427.86	46927.23	55278.95	39864.02	23152.63	32522.94

LN288.952	LN288.952	LN288.952	LN288.952	LN288.952	LN288.952	LN288.952	LN288.952	LN288.952	LN289.129
25755.54	23032.65	28158.66	42970.77	51791.09	30925.37	43203.74	44319.94	24077.47	24969.35
22934.42	23495.11	24525.96	43299.16	47282.29	31724.2	42389.48	46484.85	23951.18	25261.77
25252.77	24365.31	24712.63	43804.81	50449.17	29684.12	42969.06	39229.38	25485.19	25720.43
24781.94	24109.99	23312.95	43777.88	55270.44	34481.2	43501.88	48861.09	22439.25	26051.27
24589.93	22329.84	23820.19	44950.16	49267.93	31257.87	42106.12	45689.59	25683.02	22143.64

LN289.129	LN289.129	LN289.129	LN289.129	LN289.129	LN289.129	LN289.129	LN289.129	LN289.129	LN289.129
25991.95	27921.2	32219.5	30415.89	27069.24	28138.55	37682.07	19387.64	40213.82	29487.11
27655.52	31622.07	31913.69	31297.91	25366.52	26788.53	37469.75	23070.95	38883.9	29739.38
22691.11	31462.41	34054.09	32879.08	28665.26	27215.17	37283.01	21538.06	41333.28	29191.74
22458.71	29663.28	31536.86	34699.89	27731.53	27205.87	36860.85	18587.73	42008.27	26740.85
25381.73	28285.81	28973.08	33780.5	27874.16	24486.96	38185.38	21344.4	38336.63	26263.21

LN289.129	LN289.129	LN289.129	LN289.129	LN289.129	LN289.129	LN289.129	LN289.129	LN289.129	LN289.129
25380.11	21847.75	37476.85	25614.96	18334.87	25229.98	29180.87	52780	26371.99	24845.56
21905.62	24845.72	35161.48	29166.31	17950.88	23675.36	30054.95	51878.61	27448.24	28186.92
23185.68	19178.66	33927.94	26743.95	20270.55	22287.8	26079.51	48986.42	29612.61	23067.13
23983.77	23664.17	34214.97	26448.48	19196.74	22730.01	31755.19	49349.2	27149.93	27597.43
23111.01	22644.04	34756.37	28846.87	19366.65	22875.88	29543.76	51236.95	27086.38	27292.84

LN289.129	LN289.129	LN289.129	LN289.129	LN289.129	LN289.129	LN289.129	LN289.129	LN289.129	LN289.129
41621.36	33021.94	21589.24	31926.09	28466.63	20851.77	31528.01	21339	20520.49	13054.15
39669.98	34185.32	24316.27	31690.8	26220.7	20104.68	30105.29	22384.85	19893.5	13212.92
37010.4	33098.12	20808.35	32972.39	31730.97	19305.14	28842.17	23219.48	22428.31	14781.13
36957.13	38382.21	23458.74	31086.52	29901.33	22548.96	34142.07	20786.73	21642.86	15607.6
39187.01	32312.78	23623.18	30741.72	25798.39	18920.69	30865.55	21478.17	22344.45	13267.98

LN289.129	LN289.129	LN289.129	LN289.129	LN289.129	LN289.129	LN289.129	LN289.129	LN289.129	LN289.129
24607.77	15396.25	31198.93	37948.76	34763.48	21079.43	33801.69	28951.02	50209.78	25347.4
23034	15215.25	29792.16	36534.98	32637.57	25517.1	32947.81	29582.49	50066.78	26480.12
22786.71	15360.02	30590.56	40129.95	34205.37	24192.14	34818.76	28114.1	46768.46	25783.09
26838.61	18706.44	29152.69	38435.28	33685.72	24000.06	35477.13	27612.41	49013.35	26369.74
26492.86	17679.3	30583.38	37745.74	34904.32	27168.68	34262.7	27860.03	50052.88	27405.08

LN289.129	LN289.129	LN289.129	LN289.129	LN289.129	LN289.129	LN289.129	LN289.129	LN289.129	LN289.129
26703	30733.05	36106.93	24032.11	23873.55	41868.15	26276.95	23227.88	38457.39	13670.92
27639.5	32186.04	33682.33	25467.58	29623.23	39792.8	25059	24590.11	41774.94	14377.62
29556.02	33059.41	31764.84	23426.51	26432.54	43677.37	25199.26	22569.68	42154.83	16230.41
30840.84	34643.77	34875.89	28215.03	27863.32	41872.93	26085.11	23979.89	37283.42	15968.02
31526.56	30610.83	33563.63	25740.52	28084.17	40879.62	23322.15	19383.72	38332.79	16579.8

LN289.129	LN289.129	LN289.129	LN289.129	LN289.129	LN289.129	LN289.129	LN289.129	LN289.129	LN289.129
40997.39	16223.79	23140.66	25090.55	31821.8	14179.77	29176.07	33760.31	26772.33	22078.13
40295.92	16707.77	24213.25	24069.07	30936.59	15178.44	30098.03	36523.79	27694.75	23165.63
41390.81	14079.39	27588.94	24949.01	31455.36	15453.29	29471.19	33488.15	29634.35	20625.93
41693.71	15643.72	25031.08	24126.79	34561.96	12657.19	28733.63	35588.51	27220.7	21482.58
39015.57	13504.27	23379.79	21302.86	29315.03	13680.46	28739.91	37325.74	27928.38	21876.7

LN289.129	LN289.129	LN289.129	LN289.129	LN289.129	LN289.129	LN289.129	LN289.129	LN289.129	LN289.129
18822.97	21842.79	17401.39	26331.71	48837.03	36789.47	28529.8	25407.32	24383.41	20574.38
21446.35	23112.34	19388.26	26701.2	45183.57	39706.15	30805.65	25460.58	25084.33	22872.86
21287.69	25876.11	16878.94	25505.13	45771.6	34495.13	30267.38	27996.68	21307.94	22203.37
22671.47	22671.25	18326.13	25991.1	46076.77	38584.52	26905.53	24880.15	22917.05	22591.22
22306.74	24152.44	17260.17	28344.58	42519.43	36043.94	30570.03	28210.34	22980.67	18915.24

LN289.129	LN289.129	LN289.129	LN289.129	LN289.129	LN289.129	LN289.129	LN289.129	LN289.129	LN289.129
20844.45	44573.52	20129.04	27733.81	45056.94	37731.38	21692.35	30127.77	32112.21	20561.4
19903.93	44838.85	20856.1	30656.07	40787.54	32267.14	20987.94	28135.59	31082	18957.71
23036.3	48260.58	21252.81	30290.08	40933.8	36772.64	19142.11	24627.43	32434.12	21159.24
19855.73	50180.36	19583.24	29253.21	45907.33	36734.45	22756.48	28153.1	35940.81	22333.06
21814.62	45812.93	18930.26	30926.74	43947.26	39401.67	22125.33	24687.26	29125.4	22121.72

LN289.129	LN289.129	LN290.853	LN291.006	LN291.007	LN291.007	LN291.007	LN291.007	LN291.007	LN291.007
31534.37	13552.38	116564.4	68113.67	56620.86	53614.69	61800.28	69045.85	49607.72	71956.51
33875.87	12556.9	91144.15	94364.36	58785.43	45803.14	57519.73	81749.62	71232.61	55306.23
29849.53	12859	92493.73	69822.13	53516.76	57053.83	61557.77	70505.05	65087.33	58878.58
33039.3	12568.98	110912	95242.54	52331.32	51093.5	63452.58	73060.13	57605.14	74532.3
30016.27	12664.08	159030.7	94601.45	42396.07	35508.68	59207.72	51163.73	64700.5	101284.7

LN291.007	LN291.007	LN291.007	LN291.007	LN291.007	LN291.007	LN291.007	LN291.007	LN291.007	LN291.007
19114.19	36693.84	85633.35	70907.68	73161.01	62317.28	40657.73	71257.3	48668.53	28143.46
17151.61	51089.42	144784.9	76596.39	108871	87410.53	50096.45	63104.94	51757.49	31708.97
25284.85	50243.35	106818.8	54847.92	78282.81	88158.73	46629.99	83739.56	49071.01	33602.73
14876.34	45879.66	111900.7	70126.7	68526.67	72919.3	43760.73	64631.16	50050.21	31145.24
16982.23	49699.36	91565.66	103696.5	74962.22	50496.72	55061.36	91664.46	37997.99	32485.25

LN291.007	LN291.007	LN291.007	LN291.007	LN291.007	LN291.007	LN291.007	LN291.007	LN291.007	LN291.007
112253	75002.93	96054.27	68434.73	53980.92	84906.42	65965.29	77408.68	93518.43	118978.5
74209.21	44870.43	91004.82	70580.69	48636.43	79488.47	28944.61	60833.11	70597.07	99770.82
120745.9	48553.38	65708.8	46065.66	71446.41	88348.39	46058.35	55350.46	102042.7	73935.83
75397.83	48930.97	60233.38	55205.23	55648.62	78828.68	33629	56894.43	79461.16	81673.83
73629.1	54500.03	84563.69	36359.85	56089.11	104997	43649.03	61934.18	74006.49	111171.3

LN291.007	LN291.007	LN291.007	LN291.007	LN291.007	LN291.007	LN291.007	LN291.007	LN291.007	LN291.007
26425.97	64684.45	55884.89	94249.52	112534.9	76304.2	111650.2	104367.2	83408.59	53951.5
31587.77	83678.64	51057.68	91888.59	97486.04	81098.27	77670.49	75338.4	58154.03	80977.35
33139.18	103652.6	42169.43	89633.21	81384.09	55289.45	90480.2	73144.44	83612.89	56016.59
31533.3	82988.88	54211.72	74349.44	81887.69	70288.95	79953.84	89138.52	70709.45	60074.45
31500.15	90982.91	63691.89	61956.4	59836.13	68554.63	99188.04	75621.24	85885.05	58866.95

LN291.007	LN291.007	LN291.007	LN291.007	LN291.007	LN291.007	LN291.007	LN291.007	LN291.007	LN291.007
76757.1	58640.21	43124.12	111404.9	112305.1	134083.8	21506.31	65466.55	62583.09	45149.94
87802.48	60844.34	69106.72	99905.11	98754.03	102689.9	34513.43	56528.29	54051.37	52542.25
121338.9	49189.37	82167.06	155328.5	83425.91	101958.7	20305.78	45709.95	62714.33	41339.86
80317.65	55935.05	53258.7	95895.06	114719	113130.7	23094.49	41466.8	31427.54	40839.57
88201.11	69919.51	55324.46	96754.96	126149.9	171600.8	27918.08	62299.99	45822.04	35294.48

LN291.007	LN291.007	LN291.007	LN291.007	LN291.007	LN291.007	LN291.007	LN291.007	LN291.007	LN291.007
44609.4	71093.88	54469.98	117973.3	44918.42	90943.6	78338.32	62898.46	53932.65	76412.33
45576.68	99650.83	57617.68	124914.4	63196.58	114136.9	81838.65	40547.81	71058.18	106279
43913.91	106331.8	40650.97	113736.2	45548	87574.95	63835.54	64758.69	46514.4	102964.9
44154.3	71060.92	40532.01	101952.9	44439.84	84326.43	82094.03	55174.63	54600.35	106619.8
36919.64	72244.95	56991.39	131449.5	44558.33	77314.14	80379.64	46937.66	65703.2	159417.8

LN291.007	LN291.007	LN291.007	LN291.007	LN291.007	LN291.007	LN291.007	LN291.007	LN291.007	LN291.007
64304.3	23324.54	68289.43	70958.41	31117.24	41046.21	49093.82	25591.28	39393.04	31905.02
80490.81	18673.73	58712.51	69154.3	32993.61	42551.58	56749.89	22856.28	49182.39	29484.39
73952.8	30471.71	71846.6	104662.1	43240.52	47360.34	47168.56	23724.4	33364.56	32102.96
58762.34	22319.74	68498.31	73417.59	36257.3	40164.6	45124.09	23530.35	40614.92	31065.03
57834.91	25915.36	67232.04	78459.99	37171.46	44076.83	45910.91	25304.77	46401.98	29593.68

LN291.007	LN291.007	LN291.007	LN291.007	LN291.007	LN291.007	LN291.007	LN291.007	LN291.007	LN291.007
37885.59	59066.88	61278.71	39781.06	41916.91	21678.73	42621.93	59873.65	53646.45	16427.48
47905.95	56005.67	52977.78	51475.71	43425	33257.24	45589.21	46720.11	75735.97	20349
40792.57	56889.01	60165.91	53020.01	73665.13	29912.13	56228.84	45357.16	81937.47	27660.73
39957.93	61580.13	53429.37	48438.04	50378.52	22172.76	47390.69	66096.59	54771.49	23577.31
39931.94	62458.14	54927.32	44349.04	38412.63	27227.78	58988.51	83028.57	68532.6	19198.31

LN291.007	LN291.007	LN291.007	LN291.008	LN291.007	LN291.008	LN291.008	LN291.008	LN291.008	LN292.868
32393.79	21072.63	38039.94	15294.25	15604.48	23600.15	14464.17	79931.14	33190.08	19596.14
18520.17	33741.35	38086.04	15790	17454.04	22236.19	16497.17	76861	35362.45	14169.94
24792.55	37651.69	40008.52	17052.06	18159.89	19758.25	17212.08	79764.92	33036.5	16752.64
21277.59	28413.95	41228.43	14504.37	15882.24	22207.94	15761.32	81976.7	34555.5	21560.42
27051.04	28105.23	41403.25	17290.23	17762.7	21202.18	18021.34	80842.97	32203.4	24524.5

LN292.986	LN292.986	LN292.986	LN292.986	LN292.985	LN292.986	LN292.986	LN293.884	LN294.830	LN294.953
62378.32	52843.38	41882.23	38654.79	53967.56	43616.83	44270.7	12656.08	21435.23	70005.55
61345.6	50288.55	47890.38	43080.79	52873.85	38615.98	39768.59	9252.056	17862.13	73189.97
65558.18	47135.16	43865.68	39543.92	57130.9	42743.89	38757.47	6690.652	16043.86	72898.49
64276.56	52592.42	41118.55	40411.63	55938.35	43056.82	41070.16	10622.1	22329.14	70690.94
64059.53	47861.09	44525.91	38581.01	57374.34	42217.39	43344.43	14449.72	26917.63	66725.57

LN294.982	LN294.981	LN294.981	LN294.981	LN294.982	LN294.982	LN294.981	LN294.982	LN294.981	LN294.982
62584.35	47599.96	50457.43	61001.33	71816.33	65531.74	56214.63	68385.14	46831.5	45304.98
83282.26	50390.42	69265.79	53439.51	47216.85	38688.66	34919.08	33684.8	34320.4	22644.21
61158.59	49914.19	42834.6	61418.83	69830.73	59852.09	55589.99	71906.65	48558.72	54104.18
62230.47	52647.21	43333.48	58767.99	75633.01	64073.93	56454.35	72634.18	42823.85	50454.64
61384.56	50474.98	44440.12	64794.81	72062.54	64725.06	49976.3	69341.87	41943.51	52636.29

LN294.982	LN294.982	LN294.982	LN294.982	LN294.982	LN294.982	LN294.982	LN294.982	LN294.982	LN294.982
66181.25	54466.04	27776.84	52240.94	57072.37	38833.48	20906.53	56828.81	38630.76	71525.59
54545.94	30256.81	13645.4	37405.31	47196.1	28325.6	12317.18	33621.08	28001.53	33842.97
67744.09	55865.11	28284.3	57913.96	56542.2	41807.98	24017.74	60572.87	38745.47	79907.79
61058.85	61028.85	29418.85	64579.87	55332.56	39624.28	25889.1	56915.58	41538.6	78010.81
67898.49	55422.62	27371.64	57135.71	52388.23	41611.32	23941.53	61561.25	40570.38	74071.52

LN294.981	LN294.982	LN294.982	LN294.982	LN294.982	LN294.981	LN294.982	LN294.982	LN294.982	LN294.982
76705.3	20311.44	27651.79	56379.44	85725.81	57461.52	48407.54	45504.26	45325.34	32404.49
67612.69	15370.95	18690.54	25885.52	53141.49	41001.91	21434.01	28744.87	16839.32	17192.21
78203.55	22276.05	31004.38	51629.21	91586.31	59491.08	61500.64	46571.7	42497.34	30545.74
83911.83	19916.4	31471.58	52166.63	91559.59	60113.95	58733.44	52987.43	47584.58	31065.67
82442.47	20798.9	28630.37	51023.7	88115.07	62838.02	64212.98	48929.09	41010.78	29264.89

LN294.982	LN294.982	LN294.982	LN294.982	LN294.982	LN294.982	LN294.981	LN294.982	LN294.982	LN294.981
63636.63	80259.29	54285.82	17095.09	33658.14	65354.23	58114.82	52755.47	27458.65	54391.42
36968.04	46509.89	32361.33	14863.2	15278.39	31946.06	67314.32	31746.37	14798.14	36446.38
68602.43	80909.11	60681.09	14706.26	36551.59	62809.42	54073.23	45030.79	23354.5	53411.04
68952.79	86088.22	53324.12	16172.76	32407.34	65670.25	56167.84	53589.48	21179.14	56275.25
63055.29	86533.21	57660.11	14810.55	35650.07	63466.25	54785.84	44026.3	22658.07	58751.19

LN294.981	LN294.981	LN294.981	LN294.981	LN294.982	LN294.982	LN294.982	LN294.982	LN294.982	LN294.982
62048.9	62332.11	44430.04	72750.88	69932.75	61081.63	57762.93	60927.28	65253.59	58763.25
74940.93	45445.77	52084.91	44503.06	38899.41	29541.06	26471.76	30778.23	35337.18	35250.2
66866.5	64243.7	53703.57	79112.44	71284.9	57049.24	56712.85	59858.89	69877.47	63070.96
64884.93	61085.04	47003.6	75410.81	72952.71	57306.7	52357.05	66297.41	65248.81	62738.99
66290.2	69104.96	48770.51	72657.52	66107.39	57028.26	55110.76	69459.94	66392.53	68268.51

LN294.982	LN294.982	LN294.982	LN294.982	LN294.982	LN294.982	LN294.982	LN294.982	LN294.982	LN294.982
59597.19	24356.77	54189.46	32155.07	37615.83	51476.57	58536.67	43858.01	66050.28	50221.46
32685.36	10085.25	38123.91	19038.25	21342.02	26541.44	27002.94	23948.85	41060.31	52052.63
62275.4	25402.32	57935.44	31346.47	37973.14	51842.2	64899.71	46326.21	73067.66	53591
68905.84	21472.22	59621.29	29255.42	37606.57	53163.46	69698.58	45164.19	65707.78	57926.62
63600.28	24360.37	53873.76	30651.64	38618.89	50876.4	64589.48	46239.26	69094.95	50218.03

LN294.982	LN294.981	LN294.982	LN294.982	LN294.982	LN294.982	LN296.890	LN296.890	LN296.890	LN296.890
69138.67	36957.6	70943.51	82255.05	49977.85	35035.07	38858.93	39341.54	32628.09	34798.41
69044.27	37453.4	45202.11	61694.61	30664.31	20642.14	33167.86	35279.11	37127.88	35223.26
72382.44	37617.91	71318.92	82843.61	53812.92	34181.78	36133.21	38047.35	34223.19	35637.1
71549.11	39891.52	68004.41	72925	52360.07	33812.88	39805.06	38775	34938.32	38338.2
71671.46	38170.51	70154.57	82376.85	52669.7	33025.96	36646.7	41805.17	34004.18	34949.03

LN296.890	LN296.890	LN296.890	LN296.890	LN296.890	LN296.890	LN296.890	LN296.890	LN296.890	LN296.890
39345.35	33372.15	32800.46	37668.52	24991.44	31710	18479.79	15851.67	37912.94	26658.94
37312.02	36986.98	33700.43	35739.94	26008.4	31976.86	18923.78	16565.42	32810.4	26306.1
40143.41	30726.26	30368.66	39631.5	25083.5	30925.02	16978.38	18547.82	38628.03	25679.31
36714.89	36370.51	32051.19	39832.92	26806.44	32627.64	21111.04	14762.02	37850.5	24739.08
39284.65	33823.69	29936.43	39089.03	25810.21	30356.46	18268.46	17483.45	33867.15	29620.86

LN296.890	LN296.890	LN296.890	LN296.890	LN296.890	LN296.890	LN296.890	LN296.890	LN296.890	LN296.890
32794.35	33637.99	42245.31	23086.73	13806.76	33971.46	24560.18	19538.33	27136.35	23746.1
32088.88	35361.94	44217.64	24167.05	16027.56	37064.57	26440.53	19983.3	24817.83	24141.35
34217.37	30249.82	42936.6	26825.62	14583	31631.14	27694.03	18158.62	26234.28	24359.93
34233.48	33477.14	41577.33	22989.01	14447.51	36044.76	25946.04	16802.33	25107	22637.56
31899.9	32486.59	42070.3	22286.54	13473.71	38926.32	28889.14	19363.8	24339.82	25461.26

LN296.890	LN296.890	LN296.890	LN296.890	LN296.890	LN296.890	LN296.890	LN296.890	LN296.890	LN296.890
31186.6	27558.02	24593.62	40730.94	46100.74	41324.08	28595.94	16767.08	26547.41	13872.49
28086.36	30839.11	29065.54	39348.82	44552.5	37955.21	25427.34	18757.17	34247.15	18664.13
28700.02	29983.16	23349.92	37991.45	42547.46	34717.76	27564.51	17749.17	32822.79	18375.91
27120.85	26301.06	26532.72	37898.41	41628.45	36488.66	29265.84	19316.72	34954.8	18568.34
30241.36	29474.49	28272.01	39019.37	41289.21	40812.92	26643.22	20399.89	33287.87	17300.27

LN296.890	LN296.890	LN296.890	LN296.890	LN296.890	LN296.890	LN296.890	LN296.890	LN296.890	LN296.890
49174.34	23506.49	38976.64	39626.66	28814.28	24698.85	29763.09	31393.32	24044.61	27101.25
49165.51	25199.91	39071.26	42204.65	27001.21	26007.11	31602.28	31929.7	24156.61	27110.7
50266.01	26486.43	36872.69	40188.35	30283.63	28031.49	29772.36	30946.99	22685.13	28376.51
48920.41	26451.79	40539.39	45193.23	24849.88	25692.66	32128.88	31088.55	24499.75	26669.12
50727.29	26242.94	36958.46	42467.52	25986.72	21887.53	32603.51	27733.67	23261.63	25332.69

LN296.890	LN296.890	LN296.890	LN296.979	LN296.996	LN296.997	LN296.997	LN296.997	LN296.997	LN296.997
25243.97	28663.82	31256.3	55291.24	36085.85	48263.19	45817.53	44920.19	37072.16	36834.61
25097.38	27260.38	30815.85	52417.94	45803.88	38383.09	44107.72	30835.16	49083.74	26784.83
23330.56	25318.82	33483.37	55276	41257.22	59326.85	50457.64	50968.85	37884.98	39560.2
24507.3	25951.59	34871.58	57115.68	49360.43	53225.93	42829.87	45126.55	43289.65	40204.75
24627.77	26185.53	36272.92	54713.69	45050.77	48993.95	50000.55	39499.6	42156.6	35453.1

LN296.997	LN296.997	LN296.997	LN296.997	LN296.997	LN296.997	LN296.997	LN296.997	LN296.997	LN296.997
31819.75	49288.31	35454.48	47369.57	42094.43	41521.66	59126.86	50283.84	31019.84	54586.46
26387.66	52787.99	43781.63	38391.58	78096.92	53679.6	47901.89	48673.85	33199.31	67350.65
43604.83	46619.47	35959.43	44555.03	57108.94	42118.3	50960.23	61203.48	38714.52	75116.62
35339.16	41225.07	26969.34	39010.56	49781.06	41477.94	46601.41	44535.68	27996.95	56652.35
31549.83	43245.57	31380.47	67425.14	59133.67	35256.78	76317.94	41737.4	31140.15	66800.35

LN296.997	LN296.997	LN296.997	LN296.997	LN296.997	LN296.997	LN296.997	LN296.997	LN296.997	LN296.997
49049.77	23141.5	40225.54	67633.15	27177.1	39917.22	40684.86	52079.39	40089.61	39829.05
39803.35	23301.9	32089.33	74505.08	21056.83	52406.15	52203.56	40261.28	65886.39	54959.14
41664.75	39941.47	38264.97	64534.72	25630.47	46547.97	38809.14	51910.71	32277.03	40309.7
42100.6	21348.04	45505.75	61649.28	28538.19	33568.05	44591.97	39689.66	44035.41	41292.91
37896.03	34910.05	37073.65	58983.06	21305.02	38698.07	56487.63	57445.89	44625.12	44838.17

LN296.997	LN296.997	LN296.997	LN296.997	LN296.997	LN296.997	LN296.997	LN296.997	LN296.997	LN296.996
55535.87	23664.1	42083.21	65554.76	51727.21	33687.6	40280.46	37803.2	25667.08	63950.25
37426.94	31978.91	27326.6	74949.57	52252.74	34964.08	33034.4	39171.55	32129.01	57812.39
42845.03	26467.12	30513.21	42669.86	65447.61	39687.08	40086.25	29877.08	32577.27	66812.54
47665.79	29744.79	26956.65	51594.26	53311.71	46264.64	39350.65	31864.19	32128.54	52447.74
50524.3	35400.76	25127.6	85985.2	55258.3	48757.31	33559.13	39385.2	30735.26	54892.49

LN296.997	LN296.997	LN296.997	LN296.997	LN296.997	LN296.997	LN296.997	LN296.997	LN296.997	LN296.997
29961.34	43215.12	65667.87	52473.66	26673.62	41061.57	27460.03	51032.85	102152.6	37748.82
34826.73	55366.4	51688.31	45126.8	25875.1	39461.2	35885.62	59811.94	61487.29	28586.8
39854.05	41552.26	60807.52	73681.89	32225.81	37892.73	32551.92	50009.23	62239.1	37336.43
30250.57	46352.87	63448.99	49204.03	29541.26	39749.78	34495.13	47601.01	57825.48	36569.73
41511.85	36933.56	87905.33	53593.61	39218.68	32479.45	33081.53	33711.56	68894.54	31599.48

LN296.997	LN296.997	LN296.997	LN296.997	LN296.997	LN296.997	LN296.997	LN296.997	LN296.997	LN296.997
46055.26	28255.14	40106.48	44212.86	29944.76	39543.4	29775.9	49643.79	40094.19	34967.57
44311.52	47559.48	46133.38	51119.64	35730.43	29029.8	23775.13	31948.24	27178.3	30023.42
46776.03	22997.14	53601.27	42919.73	24586.6	45357.38	26270.22	31181.39	28798.93	29856.57
47297.24	30244.43	50814.22	42992.15	28174.78	28280.87	24648.81	33438.87	27163.01	20735.17
53474.48	32467.97	56114.23	46302.89	28388.07	33347.41	43842.39	35635.47	29914.81	32346.86

LN296.997	LN296.997	LN296.997	LN296.997	LN296.997	LN296.997	LN296.997	LN296.997	LN296.997	LN296.997
11465.15	33840.63	15039.25	33642.6	25801.88	19348.85	52224.75	19309.47	17639.97	22686.88
10874.31	19195.2	17463.05	33074.95	25965.2	16500.21	26779.44	14891.75	20654.24	17843.59
11702.83	23013.87	22097.82	29236.68	26275.98	23450	38869.18	14488.81	13293.84	15974.82
10493.85	28776.91	15973.04	28002.57	23600.4	20540.51	35944.79	13771.55	14897.43	16644.49
11249.26	32481.14	19534.59	28008.67	17411.54	18724.63	52703.32	21274.42	13680.59	20393.92

LN296.997	LN296.997	LN296.997	LN296.997	LN296.997	LN296.997	LN296.997	LN296.997	LN296.997	LN296.997
46082.18	12919.03	26781.32	29385.37	27615.86	33830.63	39193.28	24174.26	16812.04	14592.99
39106.59	16735.98	47899.76	35776.4	42145.06	34542.6	41840.51	26191.39	17043.63	17219.66
32609.76	13214.42	44957	26078.54	23788.95	42099.86	37764.89	26808.78	22315.35	20094.98
35881.76	14747.95	32668.09	24972.08	29540.44	36051.11	36933.04	28180.48	17589.42	16362.54
36290.98	15030.52	30622.99	27316.96	32531.5	41533.7	36663.59	29794.73	18218.4	16748.25

LN296.997	LN296.997	LN296.997	LN296.997	LN296.997	LN296.997	LN296.998	LN296.998	LN296.998	LN296.998
18047.23	30177.83	28435.57	25606.98	19433.86	32943.77	17969.28	17563.79	34620.8	45391.35
13930.9	25761.7	28834.74	33691.98	16242.94	31750.96	19791.72	15119.2	34577.97	42819.02
16293.4	22704.38	27767.88	39429.41	21720.8	34413.43	20380.27	18553.2	31248.93	38857.44
16915.83	28641.46	26190.35	27641.02	17485.34	33991.83	19823.16	18512.17	30328.59	43003.49
20494.12	31148.62	28935.22	35811.4	15803.95	32269.49	19593.93	16443.44	33150.76	41057.36

LN296.998	LN297.047	LN297.047	LN297.047	LN297.242	LN297.243	LN297.243	LN297.243	LN297.243	LN297.243
20464.95	22270.78	26974.29	21426.22	36187.82	16104.96	42973.81	56028.35	67534.6	59783.28
19836.68	21375.64	26025.14	24181.34	36425.21	25408.22	41183.39	55548.11	69122.01	87328.9
21919.31	22472.41	23792.03	19402.66	38378.28	20886.1	66007.85	65858.98	77275.79	68803.85
17445.82	17316.49	26689.31	21107.72	38388.52	22629.47	47024.82	68012.14	68517.25	83301.49
20821.53	22169.77	26716.87	19373.49	42802.17	24925.12	71473.14	68850.7	72527.97	82612.5

LN297.243	LN297.243	LN297.243	LN297.243	LN297.243	LN297.243	LN297.243	LN297.243	LN297.243	LN297.243
44749.94	54667.79	49702.93	59674.9	35814.59	57548.78	54615.68	48145.26	65930.88	42402.07
52997.38	55174.41	64417.25	41012.27	41090.66	49635.08	65035.59	56671.45	79162.03	32019.75
58715.91	64609.2	55038.24	54483.71	40092.84	65986.48	56583.29	74984.79	78399.73	35132.08
56821.74	60687.38	49348.07	54317.85	44611.44	62338.88	53726.2	61614.55	78274.03	37650.45
69658.39	62374.49	72387.12	56792.25	45589.22	62747.89	57880.02	68571.01	77796.91	39692.7

LN297.243	LN297.243	LN297.243	LN297.243	LN297.243	LN297.243	LN297.243	LN297.243	LN297.243	LN297.243
55259.35	64756.53	53477.81	53188.24	55758.13	38204.17	60431.81	65829.89	50313.51	55805.29
64099.56	51022.22	43366.77	51847.9	45334.55	39681.02	51141.27	63528.17	43467.99	55093.58
58534.29	68943.46	73557.67	81746.69	51909.74	52366.66	53444.63	73921.6	43453.71	55202.77
60788.57	57512.75	55760.56	66790.64	64093.93	51274.73	56073.62	64007.26	55380.75	61193.4
65349.34	62329.05	54238.9	74335.01	63649.77	52293.76	67885.5	75946.47	53198.09	63760.61

LN297.243	LN297.243	LN297.243	LN297.243	LN297.243	LN297.243	LN297.243	LN297.243	LN297.243	LN297.243
52934.67	80819.53	59206.66	29802.13	39533.92	69566.92	44658.93	34927.97	55936.75	53536.91
65966.21	55961.53	54130.83	32671.74	30332.63	53629.2	51020.84	32296.37	63758.84	65269.41
68037.25	79562.74	61042.08	40835.88	39566.6	64171.91	61379.24	40050.91	69898.22	54531.27
67595.94	65785.28	72626.69	35970.13	41771.29	82487.02	53957.83	37094.17	65611.24	61278.83
76908.22	81652.28	61079.88	37205.9	47713.79	90373.38	58302.16	38315.26	59946.17	53694.13

LN297.243	LN297.243	LN297.243	LN297.243	LN297.243	LN297.243	LN297.243	LN297.243	LN297.243	LN297.243
74850.13	66301.23	31851.39	62139.56	43469.64	54655.77	83770.03	57217.18	44162.96	64012.65
60353.59	61078.76	41734.33	75138.08	49382.77	44011.27	56135.74	64939.68	51883.33	38423.27
55912.42	54243.12	48398.97	63249.21	69169	50959.45	52722.94	89431.51	49577.63	51296.43
59335.24	74308.95	42642.4	67500.16	59141.8	44174.18	62402.79	60706.49	60138.82	47823.65
73753.16	90111.78	44615.77	82613.29	55875.3	48063.54	68630.35	75361.8	67461.99	65722.73

LN297.243	LN297.243	LN297.243	LN297.243	LN297.243	LN297.243	LN297.243	LN297.243	LN297.243	LN297.243
41529.22	60102.3	64820.12	48489.82	60117.75	54275.5	47895.25	64807.01	32672.56	42607.35
48992.37	43704.54	42384.33	47432.15	50878.1	59559.9	47056.24	39614.83	34492.94	48939.69
52345.98	52431.81	53952.75	54387.29	48014.32	49062.64	66831.91	50375.33	49095.04	48565.73
48934.49	65628.19	52913.6	57038.32	56032.89	51141.27	60821.44	55408.79	42472.38	57849.94
48750.91	59894.05	46749.83	80138.22	54274.61	69835.62	66099.88	54904	42036.69	64718.91

LN297.243	LN297.243	LN297.243	LN297.243	LN297.243	LN297.243	LN297.243	LN297.243	LN297.243	LN297.243
54925.45	64375.96	50535.88	60576.54	54910.59	61649.58	65095.87	38121.55	35666.52	49042.69
56279.79	71426.96	46382.79	52396.56	57215.69	75576.78	61764.24	43645.92	40838.47	45444.31
56810.14	71110.67	61126.19	66945.41	53722.67	71023.76	61079.72	39839.39	55513.18	56474.02
72340.17	73935.08	60383.29	66136.89	59145	73514.55	70644.8	46807.53	50075.52	59210.2
70817.53	77463.46	67915.08	61451.05	66963.9	64147.17	75672.41	51070.49	47894.08	57832.19

LN297.243	LN297.243	LN297.243	LN297.243	LN297.243	LN297.243	LN297.243	LN297.243	LN297.243	LN297.243
46477.19	76218.85	32468.73	49617.25	63504.93	51483.92	50553.93	66291.54	43276.51	71046.59
48405.65	46488.37	22708.78	52328.37	50656.21	43839.78	57614.32	61242.22	54695.21	58410.78
57089.77	61874.38	36373.12	53410.32	72540.99	60408.32	58359.46	54197.59	48998.7	76213.3
50621.8	62076.01	39234.77	59644.7	67814.98	46647.07	55317.39	57417.59	54821.39	81379.98
74486.32	59479.38	38071	57881.18	64835.55	61114.32	57867.51	65751.2	64870.91	81480.79

LN297.243	LN297.243	LN297.243	LN297.243	LN297.243	LN297.243	LN297.243	LN297.243	LN297.243	LN297.243
42191.24	61917.4	50831.1	69731.78	45088.96	45442.05	54216	52638.04	28261.47	38081.71
51021.76	40221.22	40025.88	60528	54594.33	41273.75	48640.23	49025.27	32501.53	62742.8
54917.21	51635.33	51498.88	59531.13	50617.91	45844.48	52124.88	72484.84	31911.98	50211.81
52671	50184.61	54686.24	60861.78	59401.12	45356.34	54360.48	64770.01	36012.58	52965.12
63392.44	52589.76	51890.01	67815.08	77335.12	47648.88	57019.74	64722.99	34049.97	60414.31

LN297.243	LN297.243	LN297.826	LN298.855	LN298.994	LN298.994	LN298.995	LN299.919	LN299.919	LN299.919
52135	27024.07	32859.63	20011.36	26627.58	28193.29	29478.5	85149.96	56789.76	82676.32
39760.3	28607.59	25658.19	14891.22	23142.94	29934.6	30805.92	80914.75	71072.17	76139.34
52382.66	31121.97	29190.29	17488.92	26164.26	27146.33	31560.5	68675.94	89310.83	59792.17
47654.76	32584.12	30030.48	15785.41	24087.25	29419.38	27473.09	135749.7	65461.93	73799.84
56302.57	33723.74	38631.47	14709.03	25489.84	32371.43	28952.65	81482.15	61439.3	95194.99

LN299.918	LN299.919	LN299.918	LN299.919	LN299.919	LN299.919	LN299.919	LN299.919	LN299.919	LN299.919
46460.71	78227.6	97833.02	74285.64	58741.23	51012.59	51544.24	102447.5	47071.26	91739.76
53744.16	76910.64	66619.1	62044.16	53222.47	52806.95	84421.15	48517.53	41838.84	88424.12
64941.53	99472.2	63478.94	69033.27	64659.05	65258.95	68499.72	65011.09	50665.14	74964.37
62271.96	78095.98	48884.74	51136.86	64230.28	54822.21	78957.32	73029.54	38976.36	67524.88
57839.56	90914.13	61045.75	66764.53	72393.4	84332.91	69359.49	48454.13	44234.13	62739.74

LN299.918	LN299.919	LN299.919	LN299.919	LN299.919	LN299.919	LN299.919	LN299.918	LN299.919	LN299.919
47391.88	79559.34	127990.3	114650.2	59544.74	83342.14	59778.08	61366.27	64241.07	73551.52
70083.28	66364.76	69245.4	112722.6	87879.91	83235.25	61206.78	53096.65	65200.36	64753.47
58560.55	54842.76	75769.29	62128.5	78109.84	47425.53	68613.25	55825.6	76106.31	53665.93
68681.42	51828.82	66897.56	76589.76	58883.18	57420.48	65870.79	72152.71	72834.33	66456.17
69241.05	63907.26	117134.2	81077.21	100442.8	75593.55	57263.39	51726.8	58374.28	70075.13

LN299.918	LN299.919	LN299.919	LN299.919	LN299.919	LN299.918	LN299.919	LN299.919	LN299.919	LN299.919
90783.77	89925.95	90976.88	57743.82	52370.01	56806.36	87230.85	63591.3	70340.71	69414.11
76641.35	74533.98	79771.95	49015.42	68499.34	62603.41	69971.92	63211.11	53942.75	97331.44
91755.78	67750.79	79615.46	77236.91	40160.27	68232.66	56570.21	68547.82	63323.55	102608.3
59529.65	75238.59	83480.81	60907.67	67780.19	90386.92	61419.63	47750.18	58771.82	70129.07
63845.51	57323.57	71460.23	51227.39	57848.8	75679.85	75435.65	52872.13	58158.97	119498.1

LN299.919	LN299.918	LN299.919	LN299.919	LN299.919	LN299.918	LN299.918	LN299.919	LN299.919	LN299.919
56194.92	70738.47	59434.93	68872.71	66481	64476.79	56518.48	60844.41	59545.11	63358.04
56699.31	51891.35	89973.08	59706.94	69350.68	66191.64	58266.13	79343.69	58259.63	71751.2
56702.88	76279.53	46057.93	68215.13	49880.18	57702.76	52665.08	54936.1	53232	53930.3
63191.98	53250.29	69661.34	51585.67	84295.52	96324.18	68114.97	49498.24	82432.24	76576.29
61053.17	53062.21	63824.71	56043.98	66004.04	64914.49	50166.06	82536.19	65889.22	60991.56

LN299.919	LN299.919	LN299.919	LN299.919	LN299.919	LN299.919	LN299.918	LN299.919	LN299.918	LN299.919
82775.1	54826.05	84958.91	79071.76	85134.48	70361.47	50123	61268.95	61236.66	69495.91
55380.47	63436.48	62563.85	76636.2	57819.28	48906.73	59672.76	58463.17	54628.34	59472.07
63208.31	72672.52	63192.72	72592.79	64897.28	55860.56	51371.03	52537.61	83452.96	52300.79
65598.01	59601.37	79839.07	55843.96	62954.15	59871.92	48360.83	66264.48	54111.51	60844.32
89655.16	66270.28	68283.76	52317.44	59897.77	74302.64	63246.58	84934.21	71874.33	65273.54

LN299.919	LN299.919	LN299.919	LN299.919	LN299.919	LN299.919	LN299.919	LN299.919	LN299.919	LN299.919
64133.3	85638.55	94923.34	61132.18	80595.5	110389.5	81448.96	77367.18	85056.96	40994.25
73753.08	75992.34	75505.75	45886.83	58406.57	78384.66	95074.99	99344.48	67169.16	51082.98
47480.63	82992.1	59303.56	61915.52	58804.26	71013.79	73447.03	42039.15	55999.16	73614.46
49186.71	102271.7	78496.31	43916.38	44163.01	82030.94	71987.18	56113.99	82430.16	54986.34
82354.12	55777.73	84233.93	107659	48419.25	95508.54	110113.3	67916.38	72780.18	44926.3

LN299.919	LN299.919	LN299.919	LN299.919	LN299.919	LN299.919	LN299.919	LN299.919	LN299.919	LN299.919
56954.71	73838.36	45117.84	72057.52	60692.28	65601.85	49915.31	80588.3	51704.08	78840.59
73815.51	69556.3	62610.55	76219.3	70381.13	72729.43	61455.79	70120.29	59693.48	63543.53
77196.17	89825.92	77881.69	53271.77	51492.17	56480.68	60520.28	48970.99	72371.83	57081.98
71788.83	67285.38	55002.48	84539.75	57048.97	55688.92	60578.44	55040.82	64762.46	54732.88
53493.4	98828.83	57913.18	77664.39	56931.24	78919.52	52121.49	61397.6	68744.43	69387.66

LN299.919	LN299.919	LN299.918	LN299.919	LN299.919	LN299.919	LN299.919	LN299.919	LN299.919	LN299.919
64107.55	69010.72	70890.87	80568.66	61980.8	72128.59	99657.34	43055.37	86477.59	56868.55
72137.83	61796.12	48249.69	61249.78	68092.47	69259.36	66722.61	49056.16	72273.56	59187.83
78150.57	66337.09	85151.2	61404.43	58613.6	53483.92	57861.65	41344.11	62247.72	56796.98
79286.35	69975.86	53022.55	92632.36	59691.9	68146.51	58786.37	49399.75	61103.95	61645.55
76120.54	46597.16	67112.14	69864.24	53125.52	57457.81	52316.17	43465.71	67379.62	71961.22

LN299.918	LN299.919	LN299.919	LN299.919	LN300.830	LN300.902	LN300.903	LN300.991	LN300.991	LN300.991
52586.49	109730.6	93305.11	65622.07	15651.74	56277.41	53310.16	12384.71	77750.94	44369.22
52632.41	59146.18	95305.98	69633.03	17587.7	56584.15	51806.84	19099.03	88769.35	44487.38
50406.93	68600.92	61056.27	70170.5	15107.91	52651.13	50488.26	13454.4	90733.01	46928.61
51750.7	63974.41	39242.4	74707.58	15564.07	54274.22	51765.55	14674.27	61070.12	38942.25
60220.02	69069.1	64449.5	68440.61	17288.34	56102.94	54390.32	22388.27	42166.62	45049.59

LN300.991	LN300.991	LN300.991	LN300.991	LN300.991	LN300.991	LN300.991	LN300.991	LN300.991	LN300.991
37312.81	32178.92	28682.51	25015.51	64431.82	50514.76	52608.35	58697.98	35024.09	44624.2
44901.3	29465.41	21251.55	20094.02	71138.19	44574.35	44337.21	63282.89	39350.58	38708.68
61307.72	19207.88	21606.95	33297.87	105690.4	42163.44	53614.92	57997.04	46797.67	48765.38
46848.43	20005.94	24612.25	27864.68	72952.31	37096.95	43546.16	58137.95	39688.3	44224.37
36984.04	18761.81	26153.08	22048.43	92166.08	35007.55	48050.67	53034.55	37862.91	38162.24

LN300.991	LN300.991	LN300.991	LN300.991	LN300.991	LN300.991	LN300.991	LN300.991	LN300.991	LN300.991
65589.71	33264.72	51283.4	38133.79	43658.96	50901.24	69641.94	61160.2	49368.36	49928.93
55139.64	26583.22	43380.58	61260.47	57518.34	38658.19	65168.86	41613.34	57887.45	39190.8
54276.27	45051.97	53093.16	76770.34	35833.01	50718.47	63434.53	33287.23	73636.62	29670.35
53696.74	38551.45	52917.13	50511.58	39163.7	47177.96	56033.64	39469.61	51546.29	44326.26
55373.38	35895.03	43580.05	52205.68	43295.41	46057.66	77228.49	47444.14	51353.46	41888.95

LN300.991	LN300.991	LN300.991	LN300.991	LN300.991	LN300.991	LN300.991	LN300.991	LN300.991	LN300.991
39180.61	50504.86	74721.69	77774.22	35322.46	29156.5	39654.18	73254.6	37304.93	58013.64
42038.41	50910.43	67041.37	66094.4	38568.56	33628.18	50840.9	63799.99	31666.49	57531.04
42355.44	64380.35	82883.33	67882.88	36135.24	28226.66	58788.43	57924.81	49304.39	49763.07
43368.4	43777.05	62073.12	68360.02	44534.26	24973.97	44026.24	61138.94	38496.44	44216.77
28886.68	59621.16	65727.92	65806.19	37784.5	35306.6	48542.4	93337.05	50209.84	51652.85

LN300.991	LN300.991	LN300.991	LN300.991	LN300.991	LN300.991	LN300.991	LN300.991	LN300.991	LN300.991
42032.31	24850.24	65725.66	40112.78	46526.67	63165.89	54515.5	56249.87	52364.91	32269.82
25819.7	26600.58	67144.93	55517.62	42511.03	75421.22	64517.14	58514.89	56659.47	30298.21
28381.05	29870.51	46761.96	38962.56	63347.81	57535.49	50262.16	51418.69	51058.72	50285.65
31842.39	26487.12	59230.08	47517.41	39796.02	50101.1	55386.93	50836.33	65928.94	33798.53
44043.98	31868.1	67001.48	35972.03	47389.38	93790.51	56972.73	47799.19	53720.36	46947.17

LN300.991	LN300.991	LN300.991	LN300.991	LN300.991	LN300.991	LN300.991	LN300.991	LN300.991	LN300.991
85167.23	67556.11	32555.55	87937.12	34942.11	44506	47615.88	43137.22	51040.04	53653.3
97463.18	46531.06	29669.49	91489.7	35875.93	48960.64	66004.11	40652.4	49866.14	55152.81
79781.38	41653.98	18714.88	106306	28761.75	34263.73	44306.81	40996	41006.19	62672.2
104526.7	49636.09	20736.16	77043.2	32261.31	41886.58	69258.65	46953.51	39233.53	61461.59
101960	57554.34	21451.1	84332.84	35238.75	39176.7	67683.26	39444.35	56472.76	70424.68

LN300.991	LN300.991	LN300.991	LN300.991	LN300.991	LN300.991	LN300.991	LN300.991	LN300.991	LN300.991
93147.54	65699.69	38081.92	58116.41	30253.71	91521.21	32661.58	83888.67	94061.86	93045.81
50452.94	49849.43	33060.79	47945.6	23537.98	77771.14	28771.71	82512.56	66069.2	72239.93
86014.51	44339.19	30342.28	47816.16	20411.28	87209.14	39585.34	84545.84	108932.2	82994.28
54836	55814.35	36207.07	46620.96	28302.72	83967.4	31532.84	83464.17	73075.39	60844.74
71517.83	50951.84	44850.4	51960.07	24656.73	103234.2	41711.27	70645.12	62459.57	81014.9

LN300.991	LN300.991	LN300.991	LN300.991	LN300.991	LN300.991	LN300.991	LN300.991	LN300.991	LN300.991
33390.33	79540.47	62152.3	35959.78	40765.69	59166.19	28821.17	34331.73	71803.89	73292.59
32360.1	70206.57	102434.6	39828.99	34748.74	56550.06	32084.02	52693.51	60211.54	84333.2
34213.63	74668.36	84580.84	41033.41	38179.91	73439.04	40342.76	37652.42	106632.9	87594.64
33277.07	68920.23	79272.66	30656.01	38429.73	64639.86	42798.33	37436.47	59827.11	74243.58
32259.25	60484.61	72579.86	42729.25	30916.24	49687.04	30319.4	37011.11	61310.02	85198.85

LN300.991	LN300.991	LN300.991	LN300.991	LN300.991	LN300.991	LN300.991	LN300.991	LN300.991	LN301.166
23329.31	79079.05	71347.65	43755.69	48987.28	90615.8	21702.5	20072.71	63025.81	28959.69
20813.8	59197.48	52153.75	41361.55	46680.9	56173.78	25875.18	26780.76	44775.82	30075.51
18376.44	73551.48	82574.71	32705.97	53518.33	70449.27	32859.57	25012.65	69024.76	35485.18
22704.68	67309.85	57152.33	36764.73	56125.69	62717.88	27564.89	23223.13	65703.3	35802.33
16075.9	66209.61	60248.69	43234.75	54595.64	62342.89	19221.53	26274.05	68276.26	38687.63

LN301.166	LN301.165	LN301.165	LN301.238	LN301.238	LN301.238	LN301.238	LN301.238	LN301.238	LN301.238
11960.46	19126.69	21711.95	73299.71	12906.37	19762.06	22630.59	9900.409	24152.31	18663.8
12405.17	19019.07	20775.87	61420.87	11672.91	19833.94	23905.27	14513.56	22525.06	20103.28
13614.5	23856.41	25179.74	66042.12	15172.72	23549.03	28326.63	13114.42	26924.61	25241.04
17511.56	24952.34	27865.1	74701.7	19299.15	24732.91	30418.24	15721.27	30920.79	26584.25
16690.74	26419.76	26364.26	74162.46	18237.95	26870.94	31911.53	16196.42	32527.4	29520.63

LN301.238	LN301.238	LN301.238	LN301.238	LN301.238	LN301.238	LN301.238	LN301.238	LN301.238	LN301.238
11308.47	21424	12239.6	23705.11	22977.52	24917.67	19923.16	22066.27	21888.12	17858.99
10851.17	22906.49	11977.16	23918.39	24460.84	25600.65	19955.78	22844.76	25273.11	18088.82
13119.51	26779.21	15739.87	29593.6	30545.5	30970.35	27403.19	25887.21	27361.11	21795.02
18042.56	30568.3	17628.07	31610.65	32993.35	34214.44	29915.38	30227.65	30318.92	25251.92
17452.66	32327.2	17795.48	34169.47	35184.79	36028.22	30081.93	33926.55	32526.24	27278.9

LN301.238	LN301.238	LN301.238	LN301.238	LN301.238	LN301.238	LN301.238	LN301.238	LN301.238	LN301.238
18677.54	22420.39	17672.25	20714.55	18978.41	24686.86	20499.66	23658.39	21958.17	19344.05
21137.83	24991.2	17464.22	21989.8	17471.5	25530.21	19295.69	26754.02	22451.33	21293.03
23754.52	29778.52	22097.96	26976.43	22379.18	31834.91	25540.81	31968.35	29067.2	26355.77
28350.69	31658.9	26766.4	31394.51	25309.86	35790.74	27538.67	33581.96	31389.05	26221.53
29351.17	34278.77	29191.68	32342.68	27494.62	38067.81	30350.84	36576.21	31954.29	30112.44

LN301.238	LN301.238	LN301.238	LN301.238	LN301.238	LN301.238	LN301.238	LN302.942	LN302.987	LN303.913
19957.43	31232.33	20258.47	20060.29	36259.94	21869.41	22428.65	9746.93	41300.2	68413.73
18690.99	31164.22	21695.59	20754.3	33014.39	22467.87	23886.73	9620.428	38293	63557.46
24810.52	35508.25	26721.63	24219.08	40705.29	25572.62	27606.82	11333.17	41237.62	68150.32
28605.68	40294.35	29718	27464.08	42948.23	28986.44	31515.5	12342.01	40938.84	59402.95
29495.57	38288.44	33014.6	28879.48	46514.65	30751.05	34406.91	12933.4	42592.9	84758.45

LN304.869	LN304.908	LN304.913	LN304.914	LN304.914	LN305.002	LN305.002	LN305.002	LN305.002	LN305.002
100448.1	70875.89	67122.73	92496.9	99677.08	61533.49	46524.24	35593.77	62251.39	34304
18680.73	88339.6	65642.22	91870.34	99615.35	57851.88	49228.14	38124.29	61346.66	35564.09
98654.13	92301.3	68966.59	89889.19	93707.17	56142.69	48043.53	35029.3	60006.76	35371.39
86425.36	143142.3	62199.53	87046.56	95709.36	58474.88	48342.9	41623.05	60995.59	35938.74
103081.5	121482.6	62271.16	91788.69	94496.88	56261.29	46010.86	42233	58125.47	35497.38

LN305.002	LN305.002	LN305.002	LN305.002	LN305.002	LN305.002	LN305.002	LN305.002	LN305.002	LN305.002
52273.14	34236.15	29154.95	57707.85	29400.79	32274.75	25788.02	29099.45	35905.87	36749.03
49715.2	30350.68	31495.02	57038.85	28122.05	31868.53	26435.06	29687.58	35511.45	34153.23
46374.06	32746.01	26571.44	58668.89	29788.77	33407.25	24866.24	27751.35	37535.17	34556.76
51488.66	31510.09	27925.92	58391.57	29598.85	32401.86	27910.14	28096.23	37271.49	38771.03
51012.1	35505.72	27417.2	59193.81	31648.27	31603.55	23243.71	27827.34	35221.23	34700.15

LN305.002	LN305.002	LN305.002	LN305.002	LN305.002	LN305.002	LN305.002	LN305.002	LN305.002	LN305.002
49794.41	32814.53	27546.2	32578.91	38004.06	32837.9	30221.12	32291.1	30840.65	36920.19
51028.34	33804.37	28009.58	34681.36	39650.1	33467.74	30010.52	34346.21	30689.69	35502.09
49628.88	35357.93	24498.22	35322.86	40341.83	34131.01	30015.5	31280.42	32652.54	38120.47
54044.8	33131.76	27245.68	36341.73	37622.91	30014.51	29262.99	32981.79	30293.96	38015.02
46505.74	30354.56	25995.1	33353.25	35677.18	30407.34	31031.9	32669.97	28330.67	37332.21

LN305.002	LN305.002	LN305.002	LN305.002	LN305.002	LN305.002	LN305.002	LN305.002	LN305.002	LN305.002
41983.95	27358.87	33873.19	26800.3	35721.74	48797	47544	33257.49	27153.39	37889.04
40834.71	30181.11	33976.84	27395.78	36406.74	47614.51	46220.79	34670.88	27591.17	42556.03
42157.05	29406.45	30949.58	28032.59	38885.77	49930.9	47251.11	34987.93	32190.88	35295.58
40956.61	28497.79	31395.42	27356.69	37028.99	47864.13	50260.78	35346.19	28438.07	38443.35
38583.81	29767.8	29619.97	25869.44	40337.61	49523.71	44775.61	31006.9	30371.36	36717.33

LN305.003	LN305.003	LN305.002	LN305.003	LN305.003	LN305.003	LN305.003	LN305.003	LN305.003	LN305.003
35437.64	13488.53	25881.61	21476.49	17322.36	13054.32	33408.99	33474.25	28324.81	31326.29
37174.11	11829.36	25207.76	24589.02	17581.24	12812.91	37790.37	32634.9	27363.6	28667.66
34602.54	12547.52	21749.1	23124.64	16469.4	14776.78	32022.33	34951.62	26705.71	30517.82
34369.82	13631.63	21611.82	20549.43	14925.58	14383.84	33167.45	32317.11	27406.66	29731.5
34324.62	11606.65	22136.85	21976.46	16145.23	13840.88	33296.12	38435.33	27176.96	30277.75

LN305.003	LN305.003	LN305.003	LN305.003	LN305.003	LN305.003	LN305.003	LN305.003	LN305.003	LN305.003
23645.9	18329.25	18388.1	19429.08	14806.42	14494.38	22922.19	18633.57	20804.97	28652.7
23335.71	17153.32	21626.93	19747.02	14413.33	12409.85	21483.21	17826.43	19338.78	28892.21
25838.75	17379.79	20117.89	19940.07	13166.86	10457.76	21784.83	17955.47	21519.49	28997.54
24754.36	15267.74	19757.45	19689.21	14457.12	14497.97	21257.73	15310.77	17832.51	29210.71
27112.9	18421.76	20458.32	21050.39	11859.63	10961.1	20744.78	15740.19	18956.87	27310.13

LN305.003	LN305.003	LN305.003	LN305.003	LN305.003	LN305.003	LN305.003	LN305.003	LN305.003	LN305.003
23388.68	26222.51	19193.46	21858.68	19644.28	16306.05	36299.33	30965.05	13705.21	28154.27
21121.64	28808.01	19837.38	20965.38	19258.29	14045.25	38438.78	31680.9	14940.88	30252.16
23413.42	26283.36	19980.07	22186.11	20381.16	16840.32	40209.88	30772.89	13909.31	29510.41
21026.74	24175.85	17753.59	21497.06	17010.94	15962.53	38330.74	30577.22	13941.18	27010.26
22602.51	26929.08	18215.39	19742.32	18907.64	14559.17	34997.13	31290.28	12334.25	28575.69

LN305.003	LN305.003	LN305.003	LN305.003	LN305.003	LN305.003	LN305.003	LN305.003	LN305.003	LN305.003
19408.01	21323.89	20469.62	19509.2	24931.49	15523.44	16866.25	24977.32	24330.49	25464.3
17753.03	18233.31	24412.78	20583.14	30006.88	15559.84	17862.65	23861.65	24566.89	23930.94
19931.46	21641.9	21544.21	20231.45	28614.67	16771.67	15422.57	22682.87	21939.26	25529.68
19031.78	20408.11	21954.61	20253.65	25126.54	13489.74	17929.71	23161.74	24269.37	25077.39
16454.19	19987.53	23699.85	18682.82	27143.19	15352.63	17379.89	22550.14	22082.16	25072.47

LN305.002	LN305.003	LN305.003	LN305.003	LN305.003	LN305.003	LN305.003	LN305.003	LN305.003	LN305.003
24569.59	32744.18	24820.93	19495.74	19768.09	22458.72	19235.2	36797.25	17895.51	28965.35
26198.43	30197.18	24665.27	20495.13	19086.2	23305.76	19732.19	30187.79	19109.54	29597.98
23859.87	28136.39	22845.24	20600.64	19397.38	22355.21	19155.3	31415.31	16851.32	30567.26
27851.81	33210.04	23904.9	18907.07	18767.8	20229.07	18211.87	31989.41	16695.48	29950.83
26151.2	27359.2	26219.7	21764.92	19699.46	21983.33	18650.86	34999.78	17130	26286.56

LN305.003	LN305.003	LN305.023	LN305.023	LN305.023	LN305.023	LN305.023	LN305.023	LN305.023	LN305.860
23121.37	28885.23	36344.23	36051.99	40475.97	31984.62	35639.39	37031.41	36135.54	35082.12
21117.54	27571.99	36452.32	35475.87	43965.18	31557.75	34066.54	33056.09	38941.74	31638.22
21029.13	27619.87	34112.98	34966.5	44736.03	28337.61	35822.2	32702.16	35186.6	33952.02
20377.3	23317.87	34510.53	33105.99	37376.13	30902.01	32386.37	35241.28	37363.95	43841.77
20963.2	24669.12	35313.48	33477.3	41104.22	28544.03	29790.67	32496.32	34785.09	54465.43

LN306.056	LN306.057	LN306.906	LN306.919	LN306.919	LN306.919	LN306.918	LN306.919	LN306.919	LN306.919
17579.41	18468.01	65988.09	92020.8	149202	75024.67	232041	150454.1	215982.4	217125.6
18914.46	17017.87	31793.5	94125.37	150972.7	63927.48	213877.4	175395.9	218672.6	213354.7
19285.63	19376.33	42570.19	115473.1	170169.6	63733.24	183984.4	127240.2	217015	176187.3
18349.58	19214.71	44185.68	136090.3	124962.2	91512.97	190646	141476.8	233827.8	221415.7
16134.66	18148.98	57936.71	114411.1	133343.7	76183.53	190693.2	187664	194775.4	210717.2

LN306.919	LN306.919	LN306.919	LN306.919	LN306.919	LN306.918	LN306.918	LN306.919	LN306.919	LN306.919
298087	196268.9	110549.5	70872.26	201078.9	207347.3	232898.9	163932.3	92393.9	79941.97
214956.3	213726.5	95735.25	72820.25	184653	181953.9	164982.3	158679.2	93660.82	80087.28
214289	255285.7	64470.09	71853.13	152179.3	196292.1	180668.4	144245.6	92875.45	84393.18
230427.8	174890	126678.5	66792.1	168258.4	185240	228970.7	147266.1	84835.66	82526.75
227378.4	300269.7	84987.71	68657.3	154168.6	175611.6	224918.6	169601.1	87473.03	94432.65

LN306.919	LN306.919	LN306.919	LN306.919	LN306.919	LN306.919	LN306.919	LN306.919	LN306.918	LN306.918
176668.6	146155.4	189957.9	176515	196483	210785	142339.8	174990.2	207951.8	151501.6
182498.9	175770	215489.2	200212.7	132143.3	278944.2	196335.1	210479	201627.8	151480.8
224011	173091.6	181170.6	191407.6	182570.5	220615	140118.2	145097.6	163581.5	142159.7
181611.9	155877.1	221792.7	190057	188156.1	146936.5	181384.2	186523.3	253816.6	149441.3
170690.7	132420.5	208328.9	150835.3	189936.2	193863.7	151845.4	229450.8	159840	144150.3

LN306.919	LN306.919	LN306.919	LN306.919	LN306.919	LN306.919	LN306.918	LN306.918	LN306.919	LN306.919
164375.2	233939.2	208519.3	167331.5	213720.2	112830.8	143362.2	245343.4	98425.39	202266.9
137187.8	222770.2	189423.1	159700.5	218157.5	161752.5	134736.3	194397	117852.2	196924.9
131356.8	253489.8	202134.2	212484.2	227526.8	122389.6	146138.3	196317.8	104431.9	251186.6
133095.3	243314.1	199668.3	112804.7	226780.8	110669.2	152642.7	267229.1	91814.18	181235.7
141866.3	188432	217911.5	145482.3	193882.7	162229.9	134985.6	187006.3	101368.1	175721.9

LN306.919	LN306.919	LN306.919	LN306.919	LN306.918	LN306.919	LN306.919	LN306.919	LN306.919	LN306.919
150389.6	113502.7	154963.8	125230.2	151514.9	142278.8	108573.8	108014	122245.3	148913.5
134159.3	160068.1	98488.8	151341.6	241816.6	158852	149920.6	122337.7	136176.2	137900.9
145494.7	157594.3	147967.5	122374.1	184764.3	133017.9	114832.3	129465.4	126057.6	135219
154239.4	157963.9	154778.1	139186.6	160363.8	144687.6	114926.3	109383.7	123291.2	163063.6
150543.8	157792.3	144003.9	88407.06	148725.4	139117.7	104662.2	115015.7	121735.9	128031.1

LN306.919	LN306.919	LN306.919	LN306.918	LN306.919	LN306.919	LN306.919	LN306.919	LN306.919	LN306.919
122834.2	110617.7	142337.3	108520.2	110203.8	101630.5	127068.2	86510.07	99297.73	120139.2
115615.2	115085.7	166541.5	120164.3	132369.8	100564.2	135754.1	79590.87	100611.7	126954.9
122570.9	111300.5	144168.9	105522.9	114030.3	115579.5	128723.1	84265.27	101720.4	121537.6
122197.3	107869.1	145839.7	101804.2	119419.5	99601.72	140870.7	88779.01	104383.3	122647.4
115136.6	104877.4	101663.5	96762.17	115556.2	95556.42	128168.3	80618.56	91707.78	127591.2

LN306.919	LN306.918	LN306.919	LN306.919	LN306.999	LN306.999	LN307.14_	LN307.140	LN307.14_	LN307.14_
55378.54	87819.64	66649.85	137935.5	33725.2	28878.15	26596.84	20231.07	20455.79	15869.56
60355.62	91262.57	73343.22	135979.9	29857.04	26024.38	23972.19	19344.07	19574.82	16227.1
65791.02	89684.65	68566.14	130071.3	35800.77	26151.47	30154.91	18627.12	20957.37	15677.52
59845.67	84349.29	60065.77	142576.8	29932.18	24979.37	24811.8	17530.38	18860.82	14676.21
54212.84	88448.4	64331.03	141468.2	32536.11	26258.46	27446.98	17924.78	19206.02	15376.55

LN307.140	LN307.140	LN307.140	LN307.140	LN307.140	LN307.140	LN307.140	LN307.855	LN308.863	LN308.917
23480.77	16132.85	23555.32	14039.86	14354.85	13418.68	20132.79	395710.7	120523.2	41346.58
26546.39	15237.23	26138.73	14357.05	14043.23	15074.08	22187.04	283827	108585.5	43842.42
22595.64	18090.94	26784.99	11768.84	14440.39	14339.13	19262.28	413488.3	137421.1	45072.15
26745.89	15674.33	25840.84	10637.47	12193.1	10237.95	20013.3	464904	148137.5	42520.28
21484.21	16057.39	23013.86	13444.26	12906.58	12185.41	19183.49	422139.6	181930.5	45057.6

LN308.997	LN308.997	LN308.997	LN308.997	LN308.997	LN308.998	LN308.998	LN308.997	LN308.997	LN308.997
99342.61	79907.63	62646.9	109662.5	128433.4	79600.7	40222.52	103228.2	87454.41	92895.36
193939.5	89727.84	56422.74	83422.86	99879.57	72077.03	38086.27	94199.21	83933.67	95035.9
167321.9	69888.61	69714.64	112895.8	96250	51850.93	29002.52	91810.49	86432.77	99244.16
126783	64285.13	65435.39	69664.94	123673.8	77618.91	33830.93	102159.5	60322.71	121523.8
117185.6	59847.45	49646.79	67408.54	113499.5	72194.96	36729.7	82961.51	80859.49	105192

LN308.997	LN308.997	LN308.997	LN308.997	LN308.998	LN308.998	LN308.997	LN308.997	LN308.998	LN308.998
118867.9	113932	72541.55	124369.5	53066.33	60919.16	19841.31	108830.8	27108.42	46473.02
84975.81	133240.1	54560.7	134515.3	47309.63	42118.4	24961.61	71029.51	34918.24	46009.78
69033.42	71059.91	99435.46	136905.7	62388.74	61146.39	22302.63	111009.5	33802.23	43811
73866.8	80607.53	70233.99	159480.7	43220.63	38947.42	25125.32	109944.7	33551.2	37941.2
89004.26	62076.86	77079.06	137686.6	60961.57	45358.89	25344.53	84827.68	22165.99	45175.33

LN308.998	LN308.997	LN308.997	LN308.997	LN308.997	LN308.997	LN308.997	LN308.997	LN308.997	LN308.997
44785.16	116715.7	173532.1	118792.6	105934.8	92488.1	103414.8	128993.3	78732.08	131795.5
34887.66	122745.8	126136.4	92937.05	125236.1	107855.8	124305.1	99860.36	108505.5	126523.9
37966.75	124873.5	123789.5	119493.8	113176.4	101820.1	191807.7	113911.1	71645.26	80993.32
47068.1	123081.7	177629.4	129416.2	84160.88	137055.9	123077.3	99257.67	94091.04	117771.8
50581.16	115180.8	155299.7	105789.9	99149.86	61434.64	87099.14	139188.7	84234.59	103650

LN308.997	LN308.997	LN308.997	LN308.997	LN308.997	LN308.997	LN308.997	LN308.998	LN308.997	LN308.998
97335.1	104879.9	56696.52	78710.41	124190.9	50368.14	133727.8	57233.77	54651.91	69622.46
122082.1	121467.3	74491	60475.37	112406.3	82885.09	80693.58	44990.71	42650.91	63007.87
112308.8	90607.82	72303.54	88023.59	130125.5	73836.66	104052.6	58811.77	100905.2	49596.57
75016.45	93591.82	90078.98	83582.43	112475.6	48198.35	101262.7	47443.89	60616.21	74480.09
141302.3	84130.15	52678.87	66467.43	95821.48	62184.08	89559.81	64296.61	53530.91	48524.32

LN308.997	LN308.997	LN308.997	LN308.997	LN308.998	LN308.998	LN308.998	LN308.997	LN308.997	LN308.997
97279.95	161731.3	109124.7	113657.8	97387.9	63124.2	35368.52	129773.8	127771.4	64224.68
93578.22	83027.93	81808.53	87055.75	103169.4	67583.43	36196.17	130490.1	111375.3	77107.2
111117.3	96146.2	97945.14	89962.07	94751.6	61967.41	23426.03	114968.2	142119.7	64653.05
102804.2	91130.58	83526.11	93698.25	60320.96	89880.42	38520.29	100151.3	123742.1	53862.19
115447.8	97737.04	99563.96	63145.52	95799.76	64193.28	35993.68	81027.44	86709.47	68995.9

LN308.998	LN308.997	LN308.997	LN308.997	LN308.997	LN308.997	LN308.998	LN308.997	LN308.997	LN308.998
27144.42	164764	80819.24	80581.67	106905.2	93404.09	64657.49	156101.5	98466.13	49057.57
33709.42	164658	73440.24	56180.86	86751.6	47220.34	81774.49	162499.1	148509	69467
20760.59	139114.8	71679.48	86642.37	81361.14	59962.4	91609.22	129879.5	189401.6	65952.21
35344.88	131482.3	69528.27	54056.27	119229.6	59639.64	67696.35	135380.7	136642.4	71367.22
31480.23	181636.9	58135.56	89961.7	105850.6	87626.26	57842.34	107903	124079.3	56668.4

LN308.998	LN308.998	LN308.998	LN308.997	LN308.998	LN308.997	LN308.997	LN308.998	LN308.997	LN308.997
31388.02	33617.72	41914.19	109843.7	58072.99	163036.3	86959.52	33919.74	114184.5	160191.8
37137.84	31771.47	41314.69	132685.9	70636.45	123939.4	95210.79	24075.35	112645.5	132799.5
30845.83	28699.66	36596.65	112674	70829.74	154358.5	118649.6	44364.51	104615.7	146632.2
28274.79	34071.62	32864.62	113523.1	70426.51	112397.5	136375.2	57163.25	109515.6	188011.8
44653.21	30044.17	34701.66	131710.9	60863.49	137958.2	106736.7	23747.03	132734.8	133739.8

LN308.998	LN308.997	LN308.997	LN308.997	LN308.997	LN308.998	LN308.997	LN308.997	LN308.997	LN308.997
61226.65	74228.24	88729.99	126033.1	172326.3	33103.75	88437.27	82054.89	155975.6	142632.9
58961.01	80382.59	57671.29	131432.8	107737.7	36941.37	61684.97	89448.53	119058	110372.7
63159.05	47088.42	73416.09	141929.7	133076.9	43899.59	62125.54	72119.27	92818.83	99844.99
55475.56	58606.26	69569.55	109413.4	128350.3	35487.82	78245.99	79165.85	94000.45	115420.2
63832.65	52339.99	125774.9	100309.1	112965.6	38728.04	76275.15	67953.83	101926.5	103524.6

LN308.997	LN308.997	LN309.855	LN310.858	LN310.931	LN310.930	LN310.931	LN310.931	LN310.931	LN310.930
72186.35	105357.3	33637.81	142347.9	232760.2	293426.8	289829	145418.2	157910.3	384605.4
84985.81	118784.8	26539.96	143335.8	185981.5	256100.2	188744.8	133152.4	204882.8	361408
72521.08	136389.4	27333.33	148812.5	211489.6	349221.4	205780.3	161895.3	261023.5	316955.6
73026.6	135340.1	35175.14	153645.8	184195.8	389648.5	243919.7	195326.1	173576.8	356426.5
61039.25	92194.17	32850.38	189150.1	243801.1	231621	244534	194186.4	210173.2	214864.8

LN310.930	LN310.931	LN310.930	LN310.930	LN310.931	LN310.931	LN310.930	LN310.930	LN310.931	LN310.931
357448.6	248223.1	263513.8	300201.3	300560.3	195279.2	214318.3	437370.2	204218.2	135582.9
415285.7	236783.6	354673.4	248542.5	350464.3	176032.5	187874	324210.6	298006.8	194114.9
339762.6	286933.9	239237.9	205524.4	628630.2	247620.2	207322.3	357910.4	312119.9	132416.1
436147.7	225955	273046.4	456272.9	389477.8	196817.4	327993.3	247793.2	346799	195049.8
257313.4	219180.1	292411	369759.1	390465.3	207435.3	239342	301665.1	227907.2	171994.4

LN310.930	LN310.931	LN310.931	LN310.931	LN310.931	LN310.931	LN310.931	LN310.931	LN310.931	LN310.931
284965.4	150502.5	258967.9	372284.9	270864.4	301743.8	350164.8	148701.2	256688.9	144588.9
200069.2	154782.5	273436.5	249437.4	267602.6	241837.1	351414.4	164158.9	397049.6	175391.2
285255.4	144506.8	376623.8	286825.9	319399.6	355055.2	245702	119973.2	290329	178939.6
256188.6	120645.6	323162.6	218734.6	300029.3	368525.8	267978.6	194986.9	296702.6	117232.2
305091.4	116653.9	282205.5	220847.1	221297.6	296913.7	217408.7	125160.4	283808.2	134486.8

LN310.931	LN310.931	LN310.931	LN310.931	LN310.931	LN310.931	LN310.930	LN310.930	LN310.931	LN310.931
289980.5	256172.5	239133.1	188216	231365.3	288110.4	255063.1	82693.84	349560.2	271354.1
286305.9	291424.8	188093.5	108230.1	234262.2	412647.5	281976.7	88521.21	275831.6	249952.4
329273.4	288669	244246.2	159442.5	195489.1	383562.6	374118.2	121799.3	326888	459096.4
245276.6	346840.2	190087.8	141941.4	182564.1	320661.2	321322.2	96769.93	334846.6	328730.3
295843.1	284243.9	163773.7	172107.6	211995.5	386271.6	257691.4	56278.52	326381.1	393755.3

LN310.931	LN310.931	LN310.931	LN310.930	LN310.931	LN310.931	LN310.931	LN310.931	LN310.931	LN310.931
414809	338875.9	291287.4	324210.9	312366.7	293370.8	428182.2	175180.7	342783.4	420349.3
344918.7	264740	303330.7	355553	272294.2	238689	329366.4	175825.6	350039.1	257581.5
392442.9	291068.1	255293.3	212780.8	297725.3	396520.8	379490	129106.6	296226.5	389750.9
273357.1	282782.8	306575.1	222216.5	318584.9	221951.9	302034.5	156655.8	345056.7	301091.4
362484.1	309826.8	321185.7	190538	293075.4	241642.7	338801.6	194626.6	333494.8	253052.9

LN310.930	LN310.931	LN310.931	LN310.930	LN310.931	LN310.930	LN310.930	LN310.931	LN310.930	LN310.931
66763.24	299472	338300.9	361269.7	277838.2	150686.1	169082.8	261729.6	352617.9	142620.2
67627.14	355988.6	285844.3	197973.8	390947.8	174098.4	257596.4	191701.4	301400.8	78920.14
62592.25	268691.2	184611.4	187384.7	275678.4	143064.5	169808.5	137228.9	306830.1	93989.63
45859.9	285281.9	329218	215365.3	300367.1	141263.9	153665.9	187827.1	430352.1	75954.85
98386.7	355325.2	327282.1	220218.4	384955.4	146702.9	319156.8	167608.5	331289.8	68589.17

LN310.930	LN310.931	LN310.931	LN310.931	LN310.931	LN310.931	LN310.931	LN310.930	LN310.931	LN310.931
71055.64	427207.9	199718.1	313375.5	160239.4	215282.9	352263.9	298750.4	155694.7	150917.4
48928.64	274258.7	421996.9	287794.3	192460.3	275703.1	301831.6	274091.8	129614.6	136639.8
71881.35	406336.5	293867.5	346748.6	157540.7	259192.2	299658.8	328887	176631.1	145576.7
57555.72	316151.9	283789.2	241733.4	212065.4	262248.4	544186.9	239312.4	210126.7	82812.16
60262.57	259900.7	288572.8	333265.9	149853	254382.8	230552.2	308969	140564.1	102716.6

LN310.931	LN310.931	LN310.930	LN310.931	LN310.931	LN310.931	LN310.930	LN310.931	LN310.931	LN310.931
303792.1	329466	78519.43	254701.1	450484.6	172262.5	98533.35	381560.1	292869.9	318905.3
331845	223600.7	67337.15	301876.7	276190.9	147016.5	110658.2	324086	306956.2	341442.5
393432.1	191360.1	65115.44	238310.9	288920.1	163237.5	99498	322749.3	311662.6	278651.4
277362.8	368653.1	58944.79	248071.6	291089.6	193550.6	107328.5	281742.1	302652.9	406639.6
356723.4	261867.4	62182.35	236448.3	259117.1	118659	74147.54	273285.3	299868.6	288328.2

LN310.931	LN310.931	LN310.931	LN310.930	LN310.931	LN310.931	LN311.012	LN311.013	LN311.013	LN311.013
229593.8	281998.2	280179.3	81313.53	149647.1	368924.1	42003.58	33220.33	22513.05	31771.63
294806.9	262061.9	317096.3	60143.95	189868	300612	45139.37	29866.35	26353.84	34650.34
310008.9	424184.2	279016.1	100268	196941.2	366195.9	38988.13	27663.5	25401.18	36982.87
294529.1	347250.6	242017.6	54059.84	197446.8	390740	46913.6	29502.05	23300.55	34405.31
257112.5	340649.3	339124.5	76918.84	189042.4	352996.6	40797.62	28737.61	25501.51	38003.02

LN311.013	LN311.295	LN311.851	LN311.861	LN312.859	LN312.991	LN313.01_	LN313.009	LN313.009	LN313.009
34321.65	13116.71	12416.4	13788.27	14718.32	43482.1	45065.04	45202.84	38975.06	52337.66
33302.17	15262.92	13400.34	10787.15	15043.36	45081.77	48198.5	43000.43	34786.34	50596.27
29613.88	15610.98	13260.7	10034.57	13549.45	40423.02	43720.55	41206.73	38879.98	51335.65
34059.99	13552.01	11994.27	14689.86	14458.42	43711.39	45949.75	47806.26	37162.76	54677.36
34327.12	13806.76	10844.36	17682.04	15944.23	44114.51	47959.64	46604	43000.96	57656.23

LN313.010	LN313.010	LN313.009	LN313.009	LN313.010	LN313.078	LN313.078	LN313.078	LN313.078	LN313.078
30508.57	37984.64	38080.16	44954.97	26188.14	35601.56	17103.75	18091.4	13566.96	12566.91
27378.66	37255.99	40600.79	42021.45	25393.53	46711.92	21992.53	23161.38	13066.34	12063.76
29571.01	35041.01	39829.62	47909.07	25766.04	65319.37	32814.81	29586.78	18623.44	12346.63
30237.3	34980.76	41256.6	45915.35	24906.64	75061.66	37852.72	35258.13	22337.51	14245.85
30831.79	40021.89	42046.08	47868.01	26526.25	76254.85	39277.09	38270.17	23379.15	16664.68

LN313.078	LN313.078	LN313.191	LN314.987	LN314.987	LN314.987	LN314.987	LN314.987	LN314.987	LN314.988
8874.382	20163.3	23805.29	24124.89	35281.96	40955.24	19805.89	31358.46	52077.37	43121.4
11142.22	24327.49	21789.43	26318.86	38717.74	42267.34	20930.89	32404.64	59742.02	49369.73
10143.28	33918.29	27095.03	25266.91	29895.65	43713.18	22717.41	33288.04	55322.55	48726.37
11120.86	38708.97	17694.42	25626.62	31473.92	42111.26	23995.74	31062.69	56540.3	41739.14
12773.2	38428.73	19582.52	23666.35	32692.79	41524.7	24944.36	27367.73	64306.49	41256.94

LN314.988	LN314.987	LN314.988	LN314.988	LN314.988	LN314.988	LN314.988	LN314.988	LN314.987	LN314.988
35956.23	57773.33	38924.04	35739.6	33198.64	33698.1	40540.68	31202.9	53168.83	29624.03
53326.96	47671.06	41392.85	32427.32	26894.99	41659.12	51661.39	38645.95	83358.4	26670.18
33978.81	46010.51	34124.7	33237.76	34104.13	39550.63	41367.09	34273.41	60101.92	27318.26
38778.76	48829.24	32430.23	37292.39	34455.73	35656.15	42224.64	36730.35	54425.61	27004.97
39337.29	55907.33	37926.2	49380.55	30392.08	49364.36	46869.08	29120.64	57983.8	28081.19

LN314.988	LN314.988	LN314.988	LN314.988	LN314.988	LN314.987	LN314.988	LN314.988	LN314.988	LN314.988
28540.18	26526.1	18454.47	30331.65	31573.02	48458.84	50527.55	32447.86	35124.96	27973.17
23191.62	33889.56	18616.7	41961.56	31915.18	55882.76	78134.34	26363.59	23076.43	40603.35
29400.77	24527.48	18574.92	35570.05	27488.29	52188.21	49858.62	29361.83	37036.28	27694.79
31879.01	24275.32	20662.39	34646.14	26271.69	50162.94	49155.38	29778.55	32863.44	26379.21
28183.81	22164.19	13203.64	40183.79	24318.63	52285.6	57033.64	31866.66	24784.75	20943.54

LN314.988	LN314.987	LN314.988	LN314.988	LN314.988	LN314.988	LN314.988	LN314.988	LN314.988	LN314.988
31072.31	46988.46	32715.71	39431.11	22252.37	20668.9	27743.61	13640.37	17484.58	36518.03
27554.01	44851.36	28900.78	44036.32	23409.61	21686.3	27558.34	25797.56	19757.9	46144.92
26047.29	44749.98	32001.7	38875.61	23913.02	21136.15	28667.04	15901.17	15680.12	35327.08
31326.1	45066.19	31974.85	35692.01	23353.02	20806.23	25431.18	12241.63	15959.43	37295.74
26413.07	48025.87	50887.74	43813.24	29933.58	21598.91	31688.6	12933.99	13944.86	40535

LN314.988	LN314.988	LN314.988	LN314.987	LN314.988	LN314.988	LN314.988	LN314.987	LN314.988	LN314.988
13118.01	50867.47	27462.95	55809.74	22229.84	24941.06	31556.18	51255.4	33378.26	14079.63
12793.99	34113.11	23924.01	67633.08	12488.22	40082.94	35855.66	52058.19	53724.23	15044.23
16112.63	46019.9	30746.53	63455.57	22177.75	23382.36	31923.53	52520.96	36772.42	15205.91
13818.17	56096.7	29263.56	54454.24	21825.05	22636.75	27278.61	52262.53	31782.36	13978.65
12094.57	51902.07	25668.38	59989.6	16700.14	25353.08	35029.4	68723.43	53532.84	13782.56

LN314.988	LN314.988	LN314.987	LN314.988	LN314.988	LN314.987	LN314.987	LN314.988	LN314.988	LN314.988
19662.25	39187.16	39037.9	44163.57	35650.25	70687.12	57185.76	19203.7	14609.57	19366.26
14341.83	38577.18	54469.61	37369.77	44705.88	40088.71	92292.35	20746.62	15840	28165.25
16253.66	42779.48	42218.21	40644.36	34782.29	64381.08	55054.01	20386.82	13330.34	19701.23
17071.71	37697.77	35343.95	45084.16	33654.1	62556.36	52403.12	18305.04	12234.67	17409.71
12869.59	48656.02	32362.72	51131.46	38535.72	65649.15	53658.49	18676.98	12788.52	20218.04

LN314.988	LN314.988	LN314.988	LN314.988	LN314.988	LN314.988	LN314.987	LN314.988	LN314.988	LN314.988
22395.48	40839.04	45779.01	31046.24	32812.36	43013.69	41812.26	14658.95	30368.56	17698.28
19750.91	37719.9	62082.96	31920.56	42040.23	37635.17	61397.73	14553.91	30193.32	17638.04
17848.93	39578.42	44627.78	30653.46	37447.86	38629.69	44906.57	14478.57	29622.66	19150.58
19116.13	41777.04	46248.24	32568.49	36029.42	36867.92	44017.79	14397.99	31798.61	19189.48
26279.34	31692.65	43688.55	29340.03	37154.42	47576.16	46159.35	13963.21	38748.16	23988.67

LN314.988	LN314.988	LN314.988	LN314.988	LN314.988	LN314.988	LN314.988	LN314.988	LN314.988	LN314.988
33099.32	50938.64	36153.28	34991.98	16908.05	36119.89	14240.39	31044.24	36643.46	20662.12
35928.06	44848.76	33905.29	29811.17	25996.36	48383.3	11927.18	42231.35	36835.63	16598.66
36249.24	45510.14	38588.47	32308.17	13494.44	36097.88	11135.05	29604.89	35469.38	17147.87
36107.93	47859.05	40948.07	30931.14	14147.85	31324.99	12492.68	32849.01	38054.33	16936.33
30990.1	36063.45	39973.55	31668.71	14755.91	41854.39	11925.11	35473.32	24887.83	17574.97

LN314.988	LN314.988	LN314.988	LN314.988	LN314.988	LN314.988	LN314.988	LN314.988	LN315.075	LN315.075
32554.14	31563.9	37643.35	39098.88	35216.14	21979.35	32142.72	24305.99	20313.47	13957.57
47770.89	28512.89	35491.59	34246.69	43957.01	25327.44	25108.49	42438.8	19423.07	16897.89
31166.6	29559.56	35852.87	37550.49	38661.57	22865.4	32577.98	24473.06	19416.88	17237.17
29086.87	27882.54	36273.05	34842.48	32694.85	23717.61	33646.82	23183.64	19161.51	19063.32
30165.1	28013.59	30647.23	35043	38502.63	21177.58	28907.16	25652.58	20945.8	17813.34

LN315.075	LN315.180	LN316.800	LN316.869	LN316.874	LN316.874	LN316.875	LN316.875	LN316.875	LN316.874
16232.98	18379.18	35860.56	41803.89	246659	361689.2	91839.63	62445.61	85517.36	177394.4
17767.9	18090.39	35915.59	47321.84	245948.4	348397	80485.53	69285.19	78233.64	185122.4
15354.99	18838.95	39499.46	46647.18	251517.9	375687.9	83619.36	64830.67	86139.97	179622.3
16955.83	18673.39	41080.8	30773.63	306711.1	386013.6	85754.22	60859.56	73154.33	191979.3
17226.53	15980.46	49156.94	45921.24	42708.41	57205.67	75072.87	54011.56	74925.35	184179.5

LN316.874	LN316.874	LN316.874	LN316.874	LN316.874	LN316.874	LN316.874	LN316.874	LN316.875	LN316.874
433401.3	274933	129618.1	130772	411832.8	383614	279774.2	348870.9	117729.6	285580.1
406892.9	253453	125479.2	122761.9	409025.8	409542.3	294054	329266.2	115816.6	267114.5
417009.2	264689.2	129668.4	124042.6	414667.5	404766.8	302914.3	362629.4	113357.7	292729.5
395745.4	261627.5	125890.4	110396.1	461023.7	404781.3	284680	360722.1	110798.8	271611.6
419462.1	265344.7	110283.3	117054.8	402811.6	399836	308600.8	361808.5	97405.1	274614

LN316.874	LN316.874	LN316.874	LN316.947	LN316.947	LN316.947	LN316.947	LN316.947	LN316.947	LN316.947
213632.6	86911.13	192376.1	707875.5	715649.3	820594.6	496284.4	250377	292606.1	265377.3
217215.7	87313.18	192750	1115238	668441.9	799881.1	440909.6	359064.3	299125	304966.3
216754.3	84074.23	181109	787489.8	705747	888985.6	460761.3	303635	270544	316774
220313.6	83713.08	200613.5	914531.6	675311.7	797997.9	442708.5	312704.5	385247.2	280444.4
213270.4	68565.19	200760.5	744954.5	660917.8	736849.8	544902.7	337777.5	386858.4	253134.9

LN316.947	LN316.947	LN316.947	LN316.947	LN316.947	LN316.947	LN316.947	LN316.947	LN316.947	LN316.947
649183.3	563612.2	686708.2	349345.2	485028.2	674915.3	600705.9	508072.3	833197.5	850660.2
484874	495548.6	472458	318474.3	747193.6	583371.9	726014.3	373172.4	686391.1	576476.8
633750.8	585665.9	437125.6	314423	830235.5	625036.8	569223	375581.2	866601.2	512824.4
408364.3	473663.7	427617.9	375908.1	597348.7	806121.8	459397.7	422555.7	938983.3	670648.2
526410.2	435608.3	1039221	418283.4	507960.4	470000.4	833108.5	462550.5	681467.5	476063.6

LN316.948	LN316.947	LN316.947	LN316.947	LN316.947	LN316.947	LN316.947	LN316.947	LN316.947	LN316.947
357456.1	333785.9	814029	385684.9	808864	710778.1	748465.6	533954.3	698993.9	300359.6
446010.5	544233.3	833532.3	497201.7	741954.7	913235.9	578482.3	664712.3	660160.8	325841.7
294944.6	393578.7	747997.4	328695.4	818855.2	871850.6	560451.9	646273.4	607453.6	455081
255874.4	316223.3	857363.1	355323	671963.9	512299.6	601965.5	762161.3	573042.6	379245
427152.8	500548.9	908592.4	468130.2	572596.3	565450.1	428872.9	699635.6	706222.2	261575.6

LN316.947	LN316.947	LN316.947	LN316.947	LN316.947	LN316.947	LN316.947	LN316.947	LN316.947	LN316.947
604354	541139.7	636650.5	337090.8	807170.6	464314.3	745931.3	382760.8	606034.7	183533.4
513258.1	353691	524263.1	464021	769651.4	371535.6	629715.3	459441.8	570398.8	150746
885439.7	542402.9	680667.4	385011.8	1034829	432743	502817.9	379559.6	587948.4	214416.2
664055.2	558685	562976.9	425919.6	539674.8	531195.9	834993.6	406429.9	852310.7	291765.3
614360.6	559596.8	656567.6	315128.8	825901.8	391843.3	821968.9	449093.4	809649.5	206549.6

LN316.947	LN316.947	LN316.947	LN316.947	LN316.947	LN316.947	LN316.947	LN316.947	LN316.947	LN316.947
414516.7	302888.3	349053.1	621530.3	636631.8	270616	558262.4	725866.5	566555.4	209108.1
429743.3	524917.2	400802.6	534623.3	418574.5	386030.7	499879.8	880978.4	546021.8	133096.1
420840.5	406009	298633.4	554707.1	457095.2	272493.3	515751.5	809594.6	437721.2	134351.4
524883.1	512088.7	472803.9	570091.2	481693.1	242682.8	422824.1	705841.4	547918.7	172465.1
550096.4	397153.5	328898.8	592453.2	394319.6	276253.3	564450.1	754331.4	504739	142233.7

LN316.947	LN316.947	LN316.947	LN316.948	LN316.947	LN316.947	LN316.947	LN316.947	LN316.947	LN316.947
425775.4	399942.2	790904.2	397285.2	604378.3	665336	525283.8	497551.6	579520.4	545958.5
467554.4	451857.5	515664.5	393313.9	737320.3	708607.4	575909.8	709907.1	692492.4	554189.9
433785.7	663861.4	501672.3	375347.7	682240.3	667292.7	595373.8	644130.1	557113.7	501283
483727	391531.1	544018.3	359422.8	705917.1	1042618	612163.4	512921.8	633266.6	577008.2
417186.4	479953.5	507290.4	328407.1	701485.3	469072	639651.8	789802.7	536858.3	904870.4

LN316.947	LN316.947	LN316.947	LN316.948	LN316.948	LN316.948	LN316.947	LN316.947	LN316.948	LN316.947
637097	656667.4	282192.7	149452.8	121196.1	78637.33	285615.6	250455.5	119615.2	862630.2
616284.7	635031.1	290441.7	179102.2	84627.69	79807.76	430506.4	377401.8	121428.1	670146
697285.7	675681.6	322423.4	114234.8	103133.4	87498.7	344652.1	331469.6	87768.09	656957.6
786913.8	589314.8	293252.4	175121.1	126419.3	56047.17	370866	344958.2	83129.29	690485.1
764583.2	703924.9	327628.3	95951.16	86834.62	40085.98	404149.1	283497	107425.1	625892.2

LN316.947	LN316.947	LN316.948	LN316.948	LN316.947	LN316.947	LN316.947	LN316.947	LN316.947	LN316.947
431937.2	714333.5	117197.9	102586.5	246551.3	442386.6	301832.9	511727.4	418740.5	402094.7
439319.6	734690.9	195141.2	144295	324396.2	503037.7	223337.2	340826.3	555355.1	330379.5
342969.3	775186.6	87157.89	104806.9	221827.9	398794	211648.8	433556.9	441416.1	309594.3
366184.4	615276.4	158411.9	137383	323754	368055.9	199420.1	414689.3	568416.1	326269.7
341400.6	778498.3	144335.2	110335.1	234868.3	383763.1	172282.7	451582.7	772666.4	504355.9

LN316.948	LN316.947	LN316.948	LN316.948	LN316.985	LN317.160	LN317.160	LN317.160	LN317.160	LN317.160
65615.88	431176.8	497134.5	594684.8	35331.75	33553.62	25708.27	34298.07	28883.39	35272.45
90133.19	365243.5	555453.8	578796.8	31652.42	28619.9	29569.96	32676.85	29726.82	27954.5
102412.4	545652.8	488759	599626.5	33713.2	38486.47	34109.72	48949.92	36684.81	31256.91
80526.86	476760.3	498883.7	580063	31191.55	28609.13	26835.59	29911.65	34608.56	28405.63
83305.98	419462.1	577671.9	585637.1	34294.35	27140.86	27483.74	30615.8	31750.17	27033.99

LN317.160	LN317.160	LN317.160	LN317.160	LN317.160	LN317.160	LN317.160	LN317.160	LN317.160	LN317.160
34875.61	29334.88	34276.13	53599.99	16409.84	18419.04	21303.94	29113.22	28120.09	37109.65
39917.2	30612.28	25530.79	46889.17	20613.9	12640.13	27179.61	37650.79	24137.64	41291.39
43722.59	23740.15	38353.43	48741.69	18871.92	17403.26	31233.93	37634.47	32302.94	34281.79
38171.29	30725.35	22985.48	48267.6	15713.94	17726.77	23478.09	36342.35	27644.96	40444.81
39612.06	32505.04	21535.72	52591.16	15390.66	13741.06	22902.01	36820.94	25421.61	40537.53

LN317.160	LN317.160	LN317.160	LN317.160	LN317.160	LN317.160	LN317.160	LN317.160	LN317.160	LN317.160
15742.79	42639.93	45168.02	41608.99	47726.63	40940.9	51626.99	43972.52	45523.64	37518.1
20035.97	39406.16	41395.25	32609.76	35448.59	21687.18	45224.2	51983.57	41587.76	42977.18
23169.78	42188.01	33658.92	39085.77	32226.07	28875.07	42221.35	48778.94	45937.28	58875.58
17661.2	38529.63	43484.59	32101.69	34295.42	28213.84	48298.64	51986.58	40659.61	42496.32
17281.61	40115.85	43497.72	38958.36	37518.96	23194.14	49553.92	50309.82	40947.41	36496.69

LN317.160	LN317.160	LN317.160	LN317.160	LN317.160	LN317.160	LN317.160	LN317.160	LN317.160	LN317.160
41177.65	36232.64	39998.97	30005.26	23124.58	30803.77	30234.05	27594.58	29586.09	26868.43
44106.14	24427.88	31586.28	25492.28	27433.83	40069.31	31436.82	30024.13	36698.87	28741.51
51170.6	19427.61	31567.28	32164.06	26493.34	49025.77	34382.12	30355.8	40042.29	34660.38
39546.01	20189.85	35144.38	22266.32	24917.82	32457.32	29200.4	31487.25	32772.13	24498.3
36317.18	22031.28	33028.37	24435.37	27659.68	30782.68	34193.34	27579.64	34528.79	26116.86

LN317.160	LN317.160	LN317.160	LN317.160	LN317.160	LN317.160	LN317.160	LN317.160	LN317.160	LN317.160
33386.03	71786.22	43776.88	33245.63	19491.76	15573.98	33791.84	52497.71	18066.8	22806.17
34906.04	59264.79	26697.88	37239.19	18349.61	13309.68	37641.42	36461.85	23317.57	21928.28
41084.94	49742.74	35234.46	37594.16	18419.59	14260.63	48047.58	43205.91	29243.43	17940.93
29345.4	54421	27005.1	34624.86	18088.08	14156.89	37878.26	35432.18	20782.85	17561.94
31634.14	57238.63	28780.33	35910.54	16764.34	14888.63	38077.44	34073.61	21113.02	17486.56

LN317.160	LN317.160	LN317.160	LN317.160	LN317.160	LN317.160	LN317.160	LN317.160	LN317.160	LN317.160
55865.22	43018.41	31856.01	19480.4	40298.43	41114.3	45821.1	29277.28	34204.23	30198.82
56759.85	49915.37	30606.6	20093.72	35894.72	46655.12	45950.89	30088.04	43576.6	27404.01
52989.65	48666.39	28331.02	24134.09	40089.51	38706.03	49822.47	26226.44	67962.61	26857.6
57435.04	49840.28	24922.98	20324.29	34351.59	44247.95	43242.23	31206.25	38009.32	25991.76
53966.35	45378.04	25975	17547.99	32784.45	43298.69	39059.26	26250.44	40577.68	28233.19

LN317.160	LN317.160	LN317.160	LN317.160	LN317.160	LN317.160	LN317.160	LN317.160	LN317.160	LN317.160
20633.42	36952.43	43753.88	33106.33	62191.09	22215.16	28316.26	22456.26	39305.14	44638.66
15912.2	31260.87	36137.13	32254.68	46529.06	23450.95	30121.96	22968.83	34531.89	43275.46
15359.62	22256.73	31801.37	27805.77	43876.86	30013.9	47561.96	22720.66	38248.46	45572.45
19337.01	27704.69	32546.96	31591.64	42762.1	25064.59	30067.29	22644.49	36086.78	47065.94
16087.57	30087.35	34013.81	31733.39	49473.11	25533.94	26736.85	24489.27	33010.92	43746.94

LN317.160	LN317.160	LN317.160	LN317.160	LN317.160	LN317.160	LN317.160	LN317.160	LN317.160	LN317.160
39488.24	45207.6	29004.74	39904.93	27785.92	30780.86	41392.16	18281.96	32081.14	43014.6
61652.28	47310.98	42645.38	44012.36	28461.86	32831.06	35312.13	18493.23	32225.97	40151.24
68189.34	53504.85	51830.2	40003.49	44486.87	38032.18	36924.18	14111.43	27822.04	40191.62
57812.22	45129.37	40024.33	41505.33	26866.37	33336.88	36097.32	16352.36	30433.14	39722.09
64198.56	47095.72	39189.7	42112.81	26370.19	32617.18	32610.07	19009.62	29571.53	38340.68

LN317.160	LN317.160	LN317.160	LN317.160	LN317.160	LN317.160	LN317.160	LN317.160	LN317.160	LN317.160
42143.3	34089.19	50506.39	41706.43	23720.04	43867.82	49652.83	24396.11	40777.56	25144.24
48978.14	36180.89	31637.34	39821.62	23302.55	41239.6	48091.75	26417.67	31342.79	26380.01
35515.25	47098.74	36245.68	57083.37	25818.89	41877.68	47127.41	25304.35	35091.53	28692.64
42175.07	36345.36	33444.41	39392.05	22885.72	42518.74	42633	24650.32	30795.54	30084.43
45976.72	33928.45	34525.47	40499.47	24093.06	38810.98	44241.07	24067.11	31059.93	25962.85

LN317.951	LN317.951	LN317.951	LN317.951	LN317.951	LN317.951	LN317.951	LN317.951	LN317.951	LN317.951
21567.73	35903.81	28654.87	35153.27	32133.64	27206.59	18756.23	42138.35	32530.05	31151.51
19918.47	39481.47	30658.96	33765.02	30812.57	29451.47	17731.05	41538.59	32033.96	26991.61
23412.44	37446.91	30292.06	33296.43	30249.14	24918.31	16365.53	40184.31	33053.13	27661.35
20480.8	35599.16	28890.61	33410.62	26452.55	25592.66	19123.87	45286.2	31863.17	30028.06
20087.6	36580.02	31377.56	33913.78	28539.29	26810.62	16064.06	43928.51	30382.87	25678.45

LN317.951	LN317.951	LN317.951	LN317.951	LN317.951	LN317.951	LN317.951	LN317.951	LN317.951	LN317.951
37904.94	31242.04	23122.34	41573.74	36773.47	24885.01	21772.68	36173.66	24333.7	40474.68
39433.3	30445.09	25120.44	43363.24	37508.03	26352.26	22411.62	35227.52	28793.45	40208.73
38484.46	33249.74	21799.57	41935.02	39169.09	26756.04	20929.26	40618.29	29053.33	40029.53
36010.23	31015.71	20500.79	38696.11	40223.83	29804.79	23478.76	38639.15	25087.85	41117.64
37984.53	31841.89	20193.72	40839.84	40642.97	27871.24	22288.72	35834.72	25377.22	43377.53

LN317.951	LN317.951	LN317.951	LN317.951	LN317.951	LN317.951	LN317.951	LN317.951	LN317.951	LN317.951
39024.36	44462.23	26014.31	41698.47	32581.56	36554.6	33960.87	50163.52	42241.33	27831.24
35750.16	50268.74	27951.2	42776.03	33526.71	40456.6	34627.38	47082.66	41739.54	29661.26
37370.68	46944.66	27855.83	39752.21	31886.94	36744.96	31855.27	49511.39	42390.63	31074.13
35791.63	46154.21	24963.24	42182.8	35700.25	35766.08	30499.55	48702.84	41537.51	26453.63
31814.09	40649.72	26056.39	35844.23	33513.77	35351.53	31569.21	47837	42096.06	27083.27

LN317.951	LN317.951	LN317.951	LN317.951	LN317.951	LN317.951	LN317.951	LN317.951	LN317.951	LN317.951
32652.17	33990.61	42719.74	35849.91	37237.97	34329.16	46329.66	55163.73	42552.51	36356.96
37392.28	34437.1	46063.77	35501.44	41650.68	37791.24	50304.83	58147.06	46468.24	33081.33
35033.18	33942.6	41196.58	36503.01	38340.89	33676.33	49003.53	53341.93	43277.93	37158.04
36084.7	35993.79	40871.75	33478.07	38413.54	35166.73	46583.7	53781.55	44777.81	35528.4
33962.97	30438.51	43968.92	32552.24	36856.73	33768.11	48884.15	49178.93	42387.17	37271.31

LN317.951	LN318.483	LN318.797	LN318.872	LN318.872	LN318.873	LN318.890	LN318.891	LN318.891	LN318.981
38613.49	374746.3	18526.97	11842.78	12380.14	15930.84	21056.3	17028.56	13361.06	53032.98
38410.32	383401.6	18562.36	13529.63	10837.5	17023.45	17779.21	17273.64	13458.74	71925.98
40835.78	366825.2	23655.44	10455.8	13770.96	17026.61	21211.62	15401.15	12388.08	41745.74
40218.81	327889	25956.97	12639.98	12248.55	15623.15	23125.03	14931.24	13111.2	68860.74
35632.38	323438.2	30601.01	13299.53	12262.83	16816.25	22971.34	16202.35	12841.68	61172.67

LN318.982	LN318.982	LN318.982	LN318.982	LN318.982	LN318.982	LN318.982	LN318.982	LN318.982	LN318.982
17714.67	47886.54	42218.2	23577.03	55787.56	58268.32	86039.75	36083.94	55617.83	27318.25
17040.65	59206.91	44599.38	28913.14	44203.55	78814.44	73691.67	48630.09	61022.49	38812.55
15549.59	81456.7	40942.61	24500.97	39000.15	64995.44	66707.43	30719.82	65344.16	49371.23
25567.94	53588.46	57251.58	40966.3	58978.22	64699.39	81682.35	34371.85	51681.31	39662.67
14509.49	37638.62	53730.8	20912.39	40152.73	69140.97	71884.81	46004.53	71294.08	28877.32

LN318.982	LN318.982	LN318.982	LN318.982	LN318.982	LN318.982	LN318.982	LN318.982	LN318.982	LN318.982
59322.4	28648.06	60646.23	46060.6	61731.28	16664.44	60869.26	60317.35	41150.91	41868.8
87922.56	29991.95	67538.78	35343.15	64871.2	20932.92	39185.34	47533.03	61540.25	25304.56
51282.23	29679.11	66339.49	28080.72	59294.64	19769.83	42674.53	43887.89	47827.76	32330.14
77707.21	35372.37	68424.1	27614.9	121003.1	15572.29	52144.75	54846.08	34303.33	30566.22
56381.87	31702.87	58557.52	27435.44	64314	20690.9	54400.01	55051.71	30536.95	45312.46

LN318.982	LN318.981	LN318.982	LN318.982	LN318.982	LN318.982	LN318.982	LN318.982	LN318.982	LN318.982
27920.28	89185.56	63866.98	38367.96	78225.45	81967.64	72126.24	62465.75	54770.54	39393.92
46644.65	111167.3	39306.72	31365.79	65187.27	63498.31	77993.9	57523.05	46962.62	35392.68
49079.51	104308.6	42188.63	47112.63	69730.6	77271.66	94343.84	54245.1	55689.22	31802.39
40285.18	102267.6	61885.57	33879.61	69297.77	83952.35	91944.12	62600.56	62842.47	43918.26
27763	78321.35	47742.15	43056.18	70456.31	71224.2	97378.67	55744.31	55048.52	40612.6

LN318.982	LN318.982	LN318.982	LN318.982	LN318.982	LN318.982	LN318.982	LN318.982	LN318.982	LN318.982
37428.11	54415.38	70456.05	31831.02	46740.42	55222.47	51111.87	16556.73	40397.06	22168.21
32661.88	50643.32	77483.7	37840.8	62507.46	49590.15	54818.21	17756.55	26833.51	19484.55
45857.32	51792.24	72526.45	38129.5	48448	94877.87	55777.39	20931	26167.03	16711.96
37086.2	47727.91	76171.24	28942.37	48825.28	45271.93	68528.43	21160.2	26190.69	20297.13
47410.21	53822.29	63707.09	28651.11	30666.28	70411.48	59692.05	18260.75	30440.57	18494.06

LN318.982	LN318.982	LN318.982	LN318.982	LN318.982	LN318.982	LN318.982	LN318.982	LN318.982	LN318.982
32107.51	58879.59	35112.88	86744.58	59218.52	24699.75	68455.35	49888.51	85747.53	22562.06
36430.33	88962.42	34784.81	52755.69	51335.4	53590.94	70795.95	41550.42	62608.27	20257.49
39345.63	71394.33	38589.11	64021.42	64219.33	25196.91	92689	48825.23	56477.23	19465.94
39002.57	63363.55	41144.61	60244.02	54829.88	24749.63	62319.74	40814.2	68890.6	26837.03
37244.26	59367.87	37450.4	58041.98	74522.02	27373.99	75786.97	42323.24	58666.08	23382.73

LN318.982	LN318.982	LN318.982	LN318.982	LN318.982	LN318.982	LN318.982	LN318.982	LN318.982	LN318.982
38448.92	40796.04	21723.44	38888.45	53073.56	51302.7	39173.48	55531.37	42549.74	55693.57
58155.69	35663.32	17809.28	29503.06	40213.83	33461.51	32944.35	39264.46	38971.79	83452.46
56101.4	34369.55	16131.21	38271.18	51961.39	39014.9	30425.21	50581.46	51467.09	63599.28
63124.45	27404.87	14505.19	37879.28	56398.63	45135.35	34955.73	36678.19	41896.26	58579.36
48936.24	42195.7	21219.34	38744.77	68855.29	42996.71	31402.81	38793.55	45769.03	82854.54

LN318.982	LN318.982	LN318.982	LN318.982	LN318.982	LN318.982	LN318.982	LN318.982	LN318.982	LN318.982
51208.32	65111.89	36009.38	33286.5	36796.64	74503.9	53940.29	40891.18	48708.01	90390.81
51765.99	53408.74	41648.83	28437.07	35206.45	88704.06	38781.4	43978.22	57493.79	115016.8
41886.25	57359.13	36845.31	34095.65	32798.39	81318.64	39642.04	50377.25	37021.89	106822.4
70964.73	59808.95	37536.66	32288.03	43106.11	79820.17	43408.43	39244.96	47516	101590.9
38140.75	75987.6	34535.06	30758.59	33045.94	105825	45917.76	45090.71	54325.78	97966.11

LN318.982	LN318.982	LN318.982	LN318.982	LN318.982	LN318.982	LN318.982	LN318.982	LN318.982	LN318.982
45545.03	34086.66	36647.83	71215.11	53953.3	24414.96	46573.78	47952.52	55369.11	59523.07
36493.21	37987.42	49005.22	47903.28	56896.74	23462.19	55001.09	43191.53	36839.16	64761.43
43084.67	36661.03	49302.16	57125.88	56330.31	21729.94	58288.7	48937.59	51947.62	64556.27
39003.12	43648.47	35387.08	66703.04	64078.03	25043.04	51530.08	47844	71979.09	49588.4
41224.88	30183.89	37298.27	68645.64	58922.34	25977.47	60115.76	57630.53	46808.61	66461.59

LN318.982	LN318.982	LN318.982	LN318.982	LN319.175	LN319.175	LN319.175	LN319.856	LN319.876	LN319.889
23644.35	32125.5	22532.91	34227.38	17189.02	21866.2	24655.38	23758.62	26112.49	19888.86
24358.68	33725.65	23381.16	53131.24	16142.71	18776.07	21662.83	25202.78	15136.52	15252.13
24663.81	19128.81	27325.96	44124.7	17169.98	21106.09	24901.63	22491.99	16678.79	15604.8
23216.69	20169.99	21153.31	36475.67	13533.2	23465.85	23085	21428.79	23467.97	18931.22
27164.68	21062.44	21797.98	34111.24	13979.54	20997.49	24985.89	18337.98	21771.94	17517.9

LN320.885	LN320.977	LN320.978	LN320.997	LN320.997	LN320.997	LN320.997	LN320.997	LN320.997	LN320.997
541459.7	84158.43	38560.34	53420.95	34845.58	74063.22	68349.58	61099.58	43289.21	36135.34
352647.6	83160.95	40472.65	51526.77	33885.25	72899	65711.58	54753.73	52503.8	34612.54
583650.4	88730.65	41274.92	57401.78	39604.52	80124.95	64836.53	62832.3	51054.1	40009.49
401673.4	89265.08	47243.95	51821.46	37432.47	68687.17	70988.1	57694.92	57485.08	34056.39
611502.6	89578.3	39781.97	57787	41314.69	72020.55	74461.87	64742.99	53681.3	38355.73

LN320.998	LN320.998	LN320.998	LN320.998	LN320.998	LN320.998	LN320.998	LN320.998	LN320.997	LN320.997
47408.36	25837.97	33161.26	40491.34	41180.9	41653.62	28472.73	27723.84	53084.25	66861.06
51085.33	27555.99	26599.37	41331.36	43167.96	40690.76	28378.3	24549.75	53653.1	69607.85
50509.81	27716.27	31142.16	47351.53	44824.53	42812.49	28748.86	28722.03	51948.96	60412.93
52213.26	30393.92	29485.22	42871.32	43989.62	43669.64	26279.86	29466.42	52937.9	61728.32
43855.96	29465.76	25912.64	45606.26	40888.79	38983.75	25630.51	26006.18	51216.49	66403.1

LN320.998	LN320.998	LN320.998	LN320.998	LN320.998	LN320.998	LN320.998	LN320.998	LN320.998	LN320.998
31226.26	29711.48	38822.23	11355.37	34238.72	30864.41	34169.25	34908.5	26148.99	43527.84
32414.77	29597.83	41243.88	13711.37	31119.5	28365.66	36494.04	32831.44	25709.01	42682.24
32210.31	30569.06	42299.44	13457.18	32723.57	31951.37	33567.5	37326.04	27739.19	41602.17
33004.66	30844.52	42244.85	13594.29	33321.67	28443.11	37195.01	35246.99	26255.95	42070.49
32641.58	32459.39	45286.54	12825.04	29723.71	32430.62	35914.61	33182.63	31735.37	41558.98

LN320.998	LN320.997	LN320.998	LN320.998	LN320.998	LN320.998	LN320.998	LN320.998	LN320.998	LN320.998
26853.49	37118.58	23049.08	33627.76	20120.88	26391.12	27997.81	23486.44	29223.52	61499.24
25932.69	40787.89	24897.51	37010.5	20992.48	25086.23	31458.79	22847.93	26229.71	67711.93
27576.45	36340.13	24502.23	35497.46	19971.09	27310.98	27663.2	24179.22	29388.21	67495.23
25315.25	37872.06	21071.75	38911.83	20271.76	24668.34	28120.04	24121.53	28300.85	70858.55
28625.12	41516.64	26876.96	33260.12	21970.8	26490.48	29581.96	21539.22	26824.92	63497.88

LN320.998	LN320.998	LN320.998	LN320.998	LN320.998	LN320.998	LN320.998	LN320.997	LN320.998	LN320.998
30296.99	37739.21	38660.42	42022.07	28317.02	33445.05	21898.12	37196.03	20069.36	32429.14
27503.67	38922.12	34874.68	43005.34	27956.6	36868.04	19328.68	38613.14	17257.13	29480.98
27367.45	38369.46	35618.14	44153.15	28775.58	35442.05	24341.48	40948.44	16243.5	28879.92
32460.36	37100.21	35326.95	43358.43	25630.05	37548.05	27567.73	40237.57	18816.36	29443.65
26509.22	40585.82	34917.28	41831.69	25542.61	40838.99	22128.09	41279.84	13635.15	29060.06

LN320.997	LN320.998	LN320.998	LN320.998	LN320.998	LN320.997	LN320.997	LN320.998	LN320.998	LN320.997
50842.12	57035.92	28701.43	46464.27	47013.78	61649.68	47639.79	27881.73	35560.54	44836.98
49435.95	54790.01	28840.41	45275.61	43069.24	55051.6	47423.46	29228.3	32798.66	45387.14
50404.31	57763.24	32178.94	50253.45	50687.34	58958.92	54372.84	30493.34	34641.63	48855.71
46198.51	60728.04	30304.51	47697.93	47282.57	61627.86	53599.92	30651.29	37287.85	48685.36
40750.3	56011.7	30608.57	46721.9	48981.7	60428.73	48246.21	28498.25	34484.45	51292.54

LN320.998	LN320.998	LN320.998	LN320.998	LN320.998	LN320.997	LN320.998	LN320.998	LN320.998	LN320.998
27220.18	33289.73	32731.84	19636.62	29409.74	40101.83	26961.12	22901.39	33192.84	15422.33
27802.79	30713.55	34485.42	18231.05	34489.72	37530.52	27726.93	26088.99	35957.44	14622.3
28931.57	35579.01	32619.63	17915.68	31027.22	37256.82	29729.23	25563.67	34190.1	15249.76
27307.86	38875.36	33486.54	17692.58	31921.25	39810.81	26875.92	26885.81	36103.7	14635.56
27558.68	34753.52	31817.33	15935.42	32300.93	38391.91	29778.38	25633.81	36385.33	11162.95

LN320.997	LN320.998	LN320.998	LN320.998	LN320.998	LN320.998	LN320.998	LN320.997	LN320.998	LN320.998
47597.76	48913.06	12939.23	32042.61	14000.01	47182.62	20487.51	40136.68	34734.96	18675.96
43999.14	47027.16	14200.29	31057.89	17186.5	45982.37	22980.23	37786.07	34017.52	15718.82
42364.19	49720.46	13852.03	30824.69	15596.59	47564	22917.32	37693.94	31215.32	14727.28
43984.03	54060.8	13393.2	31194.03	14720.99	46795.64	21503.64	35811.72	36201.92	17502.63
43043.25	50844.9	12345.67	29525.16	17340.02	44088.86	22328.65	39159.74	31875.33	14534.91

LN320.998	LN320.998	LN320.998	LN320.998	LN320.998	LN320.998	LN320.998	LN320.998	LN320.998	LN320.998
23855.02	30329.08	32145.92	30418.43	44072.06	17382.36	40640.93	30813.38	28045.97	44698.96
23832.09	31178.66	29875.28	29596.76	40468.68	17330.19	36820	35375.46	27969.87	41276.62
27274.01	26410.03	33677.75	28847.27	40927.02	18351.24	40921.86	33352.04	28629.45	37408.86
27325.36	30400.54	28991.74	28629.36	38881.17	19198.6	40913.31	34838.12	23242.57	40216.21
29418.34	28494.81	26840.79	27464.09	39846.07	17912.41	37489.1	34543.51	24722.09	41982.81

LN320.998	LN320.998	LN321.210	LN321.871	LN322.872	LN322.886	LN322.992	LN322.992	LN322.992	LN322.994
14069.17	20850.53	12231.35	279200.7	65313.2	50113.28	30841.64	57133.63	57168.3	21494.22
16643.03	22978.82	14565.67	263064.2	59912.34	54248.4	32230.56	59133.79	58467.15	22996.88
15434.11	21518.36	11322.42	225899.1	57020.28	47688.29	29331.23	57209.95	58605.1	18378.01
13150.98	20696.85	11789.68	258858.2	55639.34	34517.78	31825.99	57072.96	62464.08	16255.48
14027.06	22968.41	12105.35	319980.6	65084.3	82877.02	30474.33	59209	63906.72	24894.68

LN322.993	LN322.993	LN322.993	LN322.994	LN322.994	LN322.993	LN322.993	LN322.993	LN322.993	LN322.993
39292.51	34250.22	34588.43	20510.67	20365.84	42259.04	72275.75	38320	48686.6	47794.9
48745.39	37362.42	26511.01	30751.94	16619.19	51594.78	62697.31	32868.49	60516.98	35478.8
38842.36	36323.16	21245.77	21857.62	18695.56	33336.96	71758.98	30064.79	46585.84	26545.16
43350.01	28358.15	24501.37	18050.5	13190.01	48700.41	54782.51	34583.15	68655.69	42281.94
47580.33	38517.81	27747.17	22104.52	16909.98	47334.78	55970.54	35252.33	59929.05	38294.38

LN322.993	LN322.993	LN322.993	LN322.993	LN322.994	LN322.993	LN322.993	LN322.993	LN322.993	LN322.993
22186.88	27069.47	51119.96	29881.21	17525.07	41113.65	58874.51	43569.69	37083.81	61614.26
27668.48	31238.03	58169.47	30231.52	20860.49	55171.16	68282.5	42547.9	41118.85	72023.69
20760.03	25661.34	59231.74	29865.16	20284.3	35560.88	40313.76	56266.79	47966.29	55149.59
32852.69	28252.99	52772.67	31702.43	24897.36	48204.35	75595.88	39899.49	53075.35	52884.39
29197.07	28912.87	59472.44	31985.33	20114.37	54018.68	69920.05	48024	43160.17	78385.29

LN322.993	LN322.994	LN322.994	LN322.993	LN322.993	LN322.993	LN322.993	LN322.993	LN322.993	LN322.993
49621.12	15855.28	24825.85	35676.41	39495.39	37253.08	39528.91	28180.47	28703.77	28184.89
42389.06	20953.37	26357.38	36475.22	52908.61	35093.45	41573.85	33701.88	36200.09	29168.89
47761.4	13888.4	39527.06	30163.55	57335.38	39724.96	38831.95	21852.56	35740.8	19819.39
36389.77	18991.4	28780.64	29837.09	48306.61	25803.04	39275.39	29317.73	45719.71	35059.96
39236.6	18823.22	25745.02	38739.95	46454.61	39381.59	43356.27	34967.19	34523.36	30754.13

LN322.993	LN322.993	LN322.993	LN322.993	LN322.993	LN322.993	LN322.993	LN322.993	LN322.993	LN322.993
48187.44	46523.27	47142.06	59971.69	34088.68	69745.31	40271.67	39858.49	37464.25	61680.56
49200.09	49822.32	55404.46	47333.81	37135.21	85770	39659.57	35099.62	41723.09	52473.21
51751.94	42705.94	62615.94	40954.1	31064.69	69293.04	34685.79	27592.42	26644.93	54823.09
37770.6	42945.43	47361.11	42455.94	37390.74	60933.16	33247.56	50057.88	26125.53	29313.62
48023.44	53457.34	63317.78	42638.5	34372.07	91235.05	41173.63	35550	43942.53	47553.17

LN322.993	LN322.993	LN322.993	LN322.993	LN322.993	LN322.993	LN322.993	LN322.993	LN322.994	LN322.993
23433.64	30335.62	49219.59	40643.42	72397.78	45367.39	23884.96	43425.16	34069.16	86277.03
28216.57	35717.76	55274.38	63679	71343.67	47339.92	29003.66	54177.3	33236.59	74028.06
21833.72	42836.11	39495.91	37366.16	65832.3	50796.64	29979.66	48449.3	26034.95	70363.19
30954.63	31967.07	57693.05	54365.65	72884.38	46656.6	27979.23	80071.58	22918.64	66014.01
29799.97	32275.99	57147.54	57899.07	65516.03	46647.69	30011.76	54073.16	33342.5	71427.42

LN322.993	LN322.993	LN322.994	LN322.993	LN322.993	LN322.993	LN322.993	LN322.993	LN322.993	LN322.994
43235.38	37525.77	17206.24	63312.12	36862.17	35502.76	35618.9	41488.95	38638.26	16309.63
60857.54	45484.35	16807.78	62455.84	26498.97	40799.22	42939.49	43767.64	37830.45	19455.1
34020.2	43188.31	16046.06	43605.74	32900.11	38216.28	31364.08	29808.37	28114.4	23859.33
46744.89	36279.87	17927.25	69764.27	26347.64	41983.87	59621.9	57416.32	28291.45	14929.87
60568.78	44110.96	17868.27	59446.41	29460.5	39911.69	46764.81	48187.3	35860.76	18237.62

LN322.993	LN322.993	LN322.993	LN322.993	LN322.993	LN322.993	LN322.993	LN322.993	LN322.994	LN322.993
49559.4	25748.6	34281.74	47032.47	79748.69	57582.4	43641.32	31957.33	18396.61	67776.36
56763	29525.6	25524.49	47809.76	80256.6	57906.96	42802.16	41698.68	23202.17	52121.41
49958.83	21228.53	32172.42	43598.03	42882.07	64623.91	35211.61	41482.33	15199.38	37304.89
51864.69	26227.84	38352.67	49217.73	66793.98	51806.21	42845.66	37763.27	14625.91	55039.55
54235.17	30753.11	23800.75	43336.73	86382.49	56685.95	40619.7	43450.91	24421.4	53172.52

LN322.993	LN322.993	LN322.994	LN322.993	LN322.993	LN322.994	LN323.012	LN323.012	LN323.013	LN323.013
14285.21	42836.55	21721.17	54641.63	75999.94	22407.57	63106.2	55616.34	56802.13	43155.11
11779.21	40643.39	19289.26	67535.86	72198.71	25339.03	61911.96	96231.52	70434.71	38133.42
13747.84	39670.09	16523.43	38566.52	61838.43	35536.28	62943.15	65747.05	51139.58	41712.81
18087.72	29037.81	17434.78	52574.09	65062.97	22810.44	66748.37	70002.55	59206.48	39601.82
16614.94	45357.1	19116.2	67908.62	69756.47	24759.76	70858.83	56104.51	49349.98	48103.66

LN323.013	LN323.013	LN323.013	LN323.013	LN323.013	LN323.013	LN323.013	LN323.013	LN323.013	LN323.013
44436.91	19681.63	37363.54	69534.44	36057.22	47509.03	45776.11	17491.56	43558.41	64304.72
35299.97	20214.89	30172.35	76924.28	42933.88	76690.94	35027.98	12035.43	39083.27	89491.6
38013.86	19992.87	33724.49	71721.94	36611.56	44467.11	45549.87	16101.13	43401.92	68285.32
37976.72	18092.52	37595.71	63468.13	35923.62	47312.88	43880.61	14116.93	36792.79	60110.96
33594.74	19457.99	28485.98	91314.82	28153.89	48910.21	41417.51	9718.862	29710.61	74651.37

LN323.013	LN323.013	LN323.013	LN323.013	LN323.013	LN323.013	LN323.013	LN323.013	LN323.013	LN323.013
53164.94	29571.88	59625.52	41024.43	48750.91	49551.07	41319.11	37208.22	52122.52	37931.64
40495.81	22847.13	65686.96	38595.6	52206.18	47459.15	59761.23	24545.35	45210.38	45272.88
48212.04	28331.23	84525.94	44489.57	52595.92	56087.49	41959.79	29114.6	46693.92	48084.48
47639.28	31485.56	83794.93	46906.59	55715.72	50564.44	45561.67	30574.36	56272.06	43843.23
56252.79	27564.01	67875.19	42827.92	56155.21	37971.21	36422.03	31300.21	44261.15	37060.36

LN323.013	LN323.013	LN323.013	LN323.013	LN323.013	LN323.013	LN323.013	LN323.013	LN323.013	LN323.013
38592.48	55122.59	48022.35	33556.32	35837.19	63579.85	50080.74	73934.56	52500.87	42582.35
39388.43	65167.76	50617.5	29070.86	40294.04	63636.85	44210.11	75608.67	51756.12	37042.85
38941.75	53429.81	44125.08	37228.19	34811.97	58838.11	47945.84	67627.23	52317.38	40870.8
39759.44	49002.14	47053.38	35413.78	35466.46	62157.87	48084.28	67678	52832.18	42334.46
29484.28	57288.56	43619.87	31713.11	29603.68	62256.64	38454.22	101435.4	61461.71	58588.99

LN323.013	LN323.013	LN323.013	LN323.013	LN323.013	LN323.013	LN323.013	LN323.013	LN323.013	LN323.013
36884.98	55360.23	73094.04	40653.55	24040.28	33516.78	44001.24	27199.99	47396.43	37906.44
44350.98	56115.28	106813.9	29615.46	20478.32	23577.75	56445.42	22824.27	79118.82	30915.75
40762.61	59804.02	73023.61	37471.51	21387.16	37291.06	48681.91	25443.22	45935.33	33875.15
35015.39	54740.43	75260.98	42060.56	23114.66	36507.64	43527.83	22290.84	49641.26	33820.73
42594.7	52064.15	75892.35	32870.79	20539.94	29572.8	44183.19	26982.72	110207.9	35315.99

LN323.013	LN323.013	LN323.013	LN323.013	LN323.013	LN323.013	LN323.013	LN323.013	LN323.013	LN323.013
15165.67	76090.03	42589.54	49453.26	46074.3	68507.56	37225.72	26294.91	50470.23	22856.68
17925.11	71214.51	62817.78	47823.2	32886.17	52894.51	56474.96	25914.6	66846.51	15667.63
15622.1	73587.79	40763.7	46437.44	46281.69	48788.78	36462.2	29933.95	57805.59	21079.55
16812.92	76032.23	44380.38	49425.39	45900.77	45758.54	36430.18	28841.78	59089.12	23584.82
15257.39	83087.14	46992.63	54506.15	37765.18	57717.44	29792.62	44255.37	46117.96	18165.22

LN323.013	LN323.013	LN323.013	LN323.013	LN323.013	LN323.013	LN323.013	LN323.013	LN323.013	LN323.013
36839.38	35791.32	37307.2	53380.11	35526.21	52063.32	51408.09	41637.83	64582.59	67110.56
29344.01	46097.84	38252.17	36902.17	54661.22	56030.74	32545.81	37074.8	77917.63	64466.17
34456.82	39384.9	32524.8	53329.87	34216.99	63128.49	52235.66	41941.25	63630.15	60015.76
34575.52	36741.89	34647.26	48447.35	30820.25	55370.5	48801.97	43254.86	63069.36	59718.41
28952.39	35435.59	39663.67	33420.18	49510.51	48256.56	28258.86	45887.87	64030.76	91151.66

LN323.013	LN323.013	LN323.013	LN323.013	LN323.013	LN323.013	LN323.013	LN323.013	LN323.013	LN323.013
27809.08	21650.75	58446.53	52191.9	21129.24	55137.43	70575.52	28670.18	36032.06	29121.52
28016.43	21035.81	65802.04	53692.47	17340.21	78942.45	78821.91	31123.79	19934.31	38624.72
32892.94	18479.22	63443.17	55910.98	19805.53	54337.61	71107.85	27874.7	30673.13	31806.82
33721.84	19633.44	57357.77	55519.95	20656.91	52273.52	75021.77	31913.41	31977.58	30477.15
35533.63	31624.51	67047.53	40968.35	16933.37	49590.16	80158.74	26757.88	30548.1	34898.55

LN323.013	LN323.013	LN323.013	LN323.013	LN323.013	LN323.014	LN323.013	LN323.013	LN323.220	LN323.220
37534.59	48380.11	19681.24	35484.1	54925.43	23171.43	38759.24	58110.54	167791.7	23912.66
30476.43	45688.79	26710.31	44244.28	61607.77	17243.92	38785.4	75403.5	176494.2	24488.51
37234.58	44856.85	21416.21	35514.82	54577	24206.1	42862.53	58585.36	209999.2	24800.25
35582.84	45067.89	19775.08	36930.23	48510.28	19807.64	39218.95	64344.18	146061	24961.44
51237.96	48991.23	20227.66	29127.71	90657.25	22943.16	40630.65	60703.35	148064.6	28001.67

LN323.872	LN323.908	LN323.908	LN323.908	LN323.908	LN323.908	LN323.908	LN323.908	LN323.908	LN323.908
30137.27	35318.71	22888.22	21973	22716.37	40920.8	27828.13	17717.59	24352.51	31799.05
22458.67	34725.3	18667.01	22956.1	22606.99	38887.71	30277.24	17383.19	22488.11	28972.15
33321.98	35530.71	21384.1	22014.85	24173.72	42392.93	29642.35	15654.76	18906.89	29271.62
29910.19	32598.64	19530.46	18793.55	22454.06	36960.12	28308.01	16655.38	19169.52	28555.5
25443.42	46141.33	19517.75	21496.16	24447.65	47883.16	31033.96	18052.8	17204.55	27949.64

LN323.909	LN323.908	LN323.909	LN323.909	LN323.909	LN323.908	LN323.908	LN323.909	LN323.909	LN323.908
29325.69	31603.22	24186.06	19312.96	27384.9	30047.36	41251.36	24628.79	23706.73	23353.73
30972.69	29401.35	24669.74	18744.09	24697.85	25486.98	34659.83	24878.83	24600.32	21960.36
27366.36	23862.71	25136.74	19710.92	27411.29	31412.91	42019.95	23115.4	22829.02	24969.43
24991.61	24417.81	24820.96	19160.3	26497.19	27148.96	37949.09	23277.38	25857.9	21729.3
26954.06	30453.55	25456.32	19904.18	27410.24	27472.89	41634.76	24078.37	22687.42	22859.08

LN323.909	LN323.909	LN323.908	LN323.908	LN323.908	LN323.908	LN323.908	LN323.908	LN323.908	LN323.908
23121.64	30224.86	29254.73	34779.74	21690.94	21728.73	31263.11	18962.58	29255.81	29225.46
25678.56	32234.88	30545.64	31909.11	20821.11	24856.29	31032.65	20939.2	28155.28	29362.33
24922.25	26474.51	28899.85	33106.59	20087.42	22287.28	27811.88	18629.84	26648.44	29167.71
21605.52	28524.58	29179.41	33135.39	20469.39	20486.16	25691.6	17764.11	26596.89	25119.47
25335.28	28468.28	29783.45	32090.01	21323.21	23701.77	31452.81	18596.14	28491.05	27059.75

LN323.908	LN323.908	LN323.908	LN323.909	LN323.908	LN323.909	LN323.908	LN323.909	LN323.908	LN323.909
27697.22	17961.47	26268.7	22193.71	28293.5	18308.72	21907.42	21670.49	37076.12	26588.7
26595.18	17780.45	19661.49	24924.63	25353.44	18516.78	22160.58	24295.86	32507.33	23643.31
24575.92	16724.31	20639.95	18876.47	24713.55	16966.41	20740.59	19225.11	36625.77	23500.68
24115.2	17502.18	23303.09	19869.66	25918.14	17802.35	21001.16	19791.29	32669.74	26322.54
25903.51	17249.79	26137.62	21520.55	26686.51	17022.24	21157.87	21525.16	32644.99	24208.55

LN323.909	LN323.909	LN323.908	LN323.908	LN323.908	LN323.908	LN323.908	LN323.908	LN323.909	LN324.223
16597.36	20189.75	28776.99	43361.03	19927.75	28476.8	30957.48	40561.75	31031.41	25376.47
17077.06	25440.94	26812.3	43941.62	21606.15	26448.25	33314.4	39894.77	31647.92	24808.11
15217.03	23187.98	27378.37	42778.3	18654.07	26990.09	34527.54	39406.59	29229	30402.91
16172.8	22117.72	25415.43	38310.35	15617.02	27561.92	30520.91	40623.97	30009.88	20952.13
17331.52	22772.2	28854.5	40679.03	19423.74	26102.41	30450.69	40105.5	32909.46	24139.05

LN324.834	LN324.881	LN324.882	LN324.882	LN324.882	LN324.907	LN324.989	LN324.990	LN324.989	LN324.990
39533.39	46748.05	21231.89	22355.39	29045.75	25960.65	29721.25	24435.66	56871.57	48582.73
32967.73	44489.63	23479.8	20491.84	31912.09	32242.85	47491.74	29295.94	61386.84	41720.66
36134.62	47134.58	22175.92	18836.01	31190.61	43251.96	29252.01	25171.39	46389.27	35579.03
40299.59	49599.4	19728.96	18468.59	30333.35	29164.99	30404.75	25243.35	41356.57	32535.09
51692.79	51772.2	22821.07	19781.29	31629.08	42779.13	30801.82	26605.15	44617.16	31593.25

LN324.990	LN324.989	LN324.990	LN324.990	LN324.990	LN324.990	LN324.990	LN324.990	LN324.990	LN324.990
25541.91	57944.51	55472.36	33049.94	29935.1	38384.18	37708.4	27441.01	22105.75	50226.49
38533.26	45301.05	43333.29	30425.75	34629.94	39008.93	30242.75	19165.01	32502.87	43574.52
35150.57	45116.71	41778.64	30759.42	32433.08	35950.78	28128.13	24472.95	26147.68	42115
31916.12	48461.3	44625.23	30227.96	26596.81	38074.41	25851.09	26139.09	23817.66	42646.52
33430.56	47820.94	45557.8	29828.95	28873.15	34181.83	27976.49	24545.82	27806.47	39585.55

LN324.990	LN324.990	LN324.990	LN324.990	LN324.990	LN324.991	LN324.991	LN324.990	LN324.990	LN324.989
33320.09	32011.66	47149.97	66802.1	18964.19	14583	18519.74	30952.16	33157.33	66824.32
29453.2	22834.98	51411.35	35211.35	24990.03	14545.16	36471.39	26913.95	33374.17	72016.3
28586.92	22438.03	48913.33	38621.51	20477.74	12133.26	19472.99	30617.12	23748	53811.37
29147.17	24924.44	43982.96	42557.57	21525.59	10545	16554.85	28070.83	28454.89	59822.07
28991.37	23890.57	47824.31	37125.68	18176.77	10700.56	18520.81	27667.39	25256.69	59441.83

LN324.991	LN324.991	LN324.990	LN324.990	LN324.990	LN324.990	LN324.990	LN324.990	LN324.991	LN324.990
13187.69	17087.24	23807.74	43357.87	49718.22	46898.31	25035.83	29711.95	14538.78	36571.18
16766.73	28667.92	23497.64	47082.73	33867.81	37360.81	25674.62	27623.36	14898.49	44727.99
12610.27	17257.05	29037.9	36742.13	51306.56	39338.04	25933.46	31333.46	11682.73	35864.61
14795.49	17910.19	27962.48	38435.69	49880.24	42424.58	24249.4	28078.5	14127.39	33565.78
12865.51	16432.76	29238.47	41206.67	42857.95	39422.38	23173.96	28509.25	11258.69	29360.31

LN324.990	LN324.990	LN324.991	LN324.990	LN324.990	LN324.990	LN324.990	LN324.991	LN324.990	LN324.990
25393.06	85167.02	10082.92	21635.65	30409.01	30778.15	44280.51	18195.52	34034.5	34904.87
23097.77	67859.29	12516.4	30083.36	25565.65	27221.17	40629.99	16466.88	56308.64	27195.01
21400.92	65436.97	14026.46	25426.24	30455.8	25810.87	37411.33	13836.9	37990.93	44014.5
21020.15	62096.36	12463.25	24559.71	26238.69	24853.94	38993.41	14564.34	39784.84	36975.03
21959.46	67656.44	13477.76	24143.64	33893.13	23611.49	35592.33	17836.42	40866.92	37716.21

LN324.990	LN324.990	LN324.990	LN324.990	LN324.990	LN324.990	LN324.990	LN324.990	LN324.991	LN324.990
25180.6	36935.44	35067.47	38490.94	36108.51	27025.06	26129.88	15708.58	17389.05	41957.72
31411.99	56316.79	43415.58	27809.08	43305.67	23855.21	36354.76	11279.46	18797.22	50230.26
28417.71	35057.02	48316.91	28011.69	33698.97	22991.29	25671.57	10571.71	15022.63	53281.71
31592.23	34727.4	41229.81	30575.66	36167.34	21432.15	25748.31	12307.61	16455.9	49495.82
30621.81	34538.1	44167.8	27005.26	35886.44	24341.76	27195.12	10768.41	15206.16	47300.33

LN324.990	LN324.990	LN324.990	LN324.990	LN324.990	LN324.989	LN324.990	LN324.990	LN324.990	LN324.990
33611.31	31207.94	21050.88	40975.62	47860.64	49098.93	50491.47	14869.1	37658.61	41450.97
31246.39	43460.39	16191.38	37827.48	31859.89	81251.8	28713.65	12786.99	31346.81	33587.49
26666.05	30291.05	18092.38	41364.96	35738.35	67053.69	27908	15178.97	29345.43	44266.58
29075.52	33513.27	18686.97	38264.72	38202.34	61927.32	32238.53	13709.74	32874.44	46809.98
29281.89	29860.04	18134.19	38621.57	35233.7	65324.11	30973.72	13250.62	31933.33	48845.94

LN324.991	LN324.990	LN324.991	LN324.990	LN324.991	LN324.990	LN324.990	LN324.990	LN324.990	LN324.990
31380.74	53721.69	13012.7	33588.61	27214.34	50786.14	31746.28	32763.13	27772.14	29506.24
19686.62	36230.21	16629.66	39577.28	18531.32	56041.43	37209.11	39923.65	43135.69	28141.57
25403.83	38410.12	12503.23	32264.41	24960.15	43285.04	32843.42	39361.89	34881.94	27165.18
22890.31	44558.87	13941.47	30896.79	21221.55	44037.28	29160.39	41526.69	36357.1	25752.69
27139.94	45846.87	13342.79	32122.05	25232.55	45379.56	28316.81	36682.05	31937.07	27344.92

LN324.991	LN324.990	LN324.990	LN324.990	LN324.990	LN324.991	LN324.990	LN324.990	LN324.991	LN325.004
30451.13	24378.77	14658.2	45139.32	34242.28	25676.25	54429.25	27820.75	16458.61	14962.84
21990.17	30156.45	23174.76	35213.16	33594.38	19315.64	55231.61	31609.39	22548.24	11399.85
22788.65	30615.31	14051.26	36752.57	29658.29	15606.28	53591.34	28381.64	18171.15	11893.76
20444.24	26111.92	14481.62	37585.17	27000.38	19907.38	51006.89	28646.91	20069.47	14663.57
22808.27	27282.45	10668.84	38775.15	26155.18	16398.04	47202.87	26694.03	17016.47	13177.07

LN325.832	LN326.829	LN326.903	LN326.903	LN326.903	LN326.903	LN326.903	LN326.903	LN326.903	LN326.903
32072.36	364954.6	144326	164878.8	106963.3	142284.6	157410.8	192216	130283.5	146580
30392.6	307334.1	168105.8	199070.2	101439.7	145805.2	162833.4	189344.8	130311.5	165122.9
36302.51	366007.9	216615.3	127811.1	134846.6	143990.4	197690.4	134395.3	180928.9	125781.2
38071.87	427629.4	152504.1	148312.4	104270.1	144681.2	150992.5	178483.7	138970.5	160087
39812.66	519407.8	217136.8	144218.7	61199.09	132509.5	195212	174821	113466.3	170031.4

LN326.903	LN326.903	LN326.903	LN326.903	LN326.903	LN326.903	LN326.903	LN326.903	LN326.903	LN326.903
209886.1	213534.7	132363.1	189571.4	179878.7	150498.3	186503.3	154327.9	116011.2	131321.5
223716.4	236305.3	125513	190881.3	181254.2	153159.6	196020.5	137266.9	105643.2	137799
261484.4	234879.7	119839.1	194766.5	199321.4	154728.5	185710.9	147275.7	131488.7	136443.2
232580	239612.5	123874.6	187982.8	184750.1	163784.3	198049	140443.8	97043.34	143252.2
180892.1	235254.2	117646.7	191458.1	176029.5	159850.7	201296.5	147228.1	93084.73	126260.4

LN326.903	LN326.903	LN326.903	LN326.903	LN326.903	LN326.903	LN326.903	LN326.903	LN326.987	LN326.987
170446.8	187982.5	154552.9	178369.3	138919.6	134541.4	57088.91	64359.33	73174.23	108562
174110.3	189069.1	154374.5	173962.4	143403	126274.2	60472.29	61552.01	79026.38	107928.3
175858.1	195208.4	173028.7	174911.4	137341.7	133145.6	63125.82	67671.84	75435.93	104038.6
170934.1	199497.9	174330	175409.7	136859.3	119449.9	53109.52	57321.61	71614.93	104586.8
151208.7	185793	166907.5	168390.5	126567.9	116968.8	52403.65	59331.89	79559.41	107450.6

LN326.988	LN326.988	LN326.988	LN326.988	LN326.988	LN326.988	LN326.988	LN326.988	LN326.988	LN326.988
108230.1	64514.79	55197.35	79396.5	64743.91	59749.13	35219.72	92485.81	80436.92	68846.59
96372.41	55543.01	42569.89	99902.51	58021.85	53790.08	43250.56	97609.58	52690.64	66027.07
69436.44	34702.39	62949.28	109094.1	88585.93	63587.61	51912.53	58796.92	54917.81	58518.34
78533.58	45811.65	54062.67	81580.24	58617.57	50392.11	46433.13	80948.69	62778.03	69109.71
85051.33	32487.98	31775.66	101146.7	73854.95	48203.37	31767.69	94024.09	84729.24	65450.66

LN326.988	LN326.988	LN326.988	LN326.988	LN326.988	LN326.988	LN326.988	LN326.988	LN326.988	LN326.988
57721.72	46608.04	47759.43	105491.2	90462.47	66680.46	74734.9	57801.86	91155.98	85664.06
70339.46	29974	74448.37	72288.76	48716.58	104420	62812.27	64882.98	87672.66	85955.73
72531.27	46300.07	63453.36	59254.16	54813.55	112326.2	61538.92	55993.65	80317.61	101691.4
53665.49	41308.06	51778.69	76426.37	76508.06	93703.91	61146.65	47201.01	85833.28	76700.92
67197.32	38352.55	61362.56	94060.68	56127.2	57972.17	90480.29	60944.2	117725.8	71919.56

LN326.988	LN326.988	LN326.988	LN326.988	LN326.988	LN326.988	LN326.988	LN326.988	LN326.988	LN326.988
32307.86	68796.1	75110.63	66745.39	16694.26	93582.01	55650	80467.32	47746.08	67211.49
30860.95	63691.79	94840.55	79387.58	17913.88	68034.54	55957.93	87123.63	40268.14	96187.6
28373.64	75774.99	79994.02	73201.44	16562.45	82146.4	50682.54	110040.8	44599.46	86486.59
22909.69	82903.63	88945.31	59161.87	18056.44	105532.6	79165.27	132744	35945.52	69869.05
25850.49	62990.54	92988.51	56678.9	22938.55	80912.8	45870.03	126787.7	44065.34	88064.06

LN326.988	LN326.988	LN326.988	LN326.988	LN326.988	LN326.988	LN326.988	LN326.988	LN326.988	LN326.988
114818.5	60021.91	31353.01	30448.42	38624.4	92609.53	76547.1	58193.71	42531.03	85314.56
108188.8	72612.93	36433.5	39434.77	48749.34	61865.28	91883.12	66588.32	55091.97	97552.82
99415.28	70706.11	32792.16	38378.72	31320.37	86739.38	62566.03	75376.51	54587.96	83075.32
94552.9	56741.29	29029.96	71049.62	48304.98	64859.61	71318.88	46973.01	61098.95	127171.6
142780.1	57104.42	24790.25	48768.23	48434.09	75689.76	70041.46	57533.78	44747.8	77341.18

LN326.988	LN326.988	LN326.988	LN326.988	LN326.988	LN326.988	LN326.988	LN326.988	LN326.988	LN326.988
60985.8	30564.23	48660.9	57958.5	54676.66	62872.45	64618.82	113988.5	54965.04	72464.99
66949.01	22672.7	44325.24	82216.45	57299.7	38605.18	40988.44	61480.37	51500.57	57667.63
64720.24	21664.62	41585.93	81787.81	53321.07	35205.93	43950.72	83484.9	42121.13	61615.38
60961.62	30937.18	52730.54	99097.88	38544.29	59064.11	42938.95	72255.12	55396.55	70525.18
67409.33	33012.69	37053.37	87556.48	46567.71	59439.5	47932.77	97023.98	42617.56	67609.99

LN326.988	LN326.988	LN326.988	LN326.988	LN326.988	LN326.988	LN326.988	LN326.988	LN326.988	LN326.988
31849.99	41735.09	110594.9	55940.81	75304.66	73988.37	142133	29952.41	54795.08	44109.8
31554.04	59858.61	119623.2	60352.03	89345.27	74718.33	130966.2	28045.07	39981.73	48853.75
42893.39	47090.02	82878.15	61185.78	109701.2	84861.16	113976.3	28726.09	67611.34	46915.43
33677.56	36073.95	86896.06	73351.49	74908.49	82538.46	95318.31	28426.95	34354.52	43650.48
30412.93	48999.91	66351.44	116484.8	65427.36	93552.11	85887.68	23354.41	37393.53	41376.46

LN326.988	LN326.988	LN326.988	LN326.988	LN326.988	LN326.988	LN326.988	LN326.988	LN326.988	LN326.988
49205.24	69230.43	53566.16	63681.35	148383.4	129254.8	46628.37	71335.58	28258.05	79664.1
51896.79	71586.27	48776.84	59688.12	96409.76	106842.9	41209.77	46053.18	30135.21	81376.11
58852.14	64962.75	66959.85	41102.46	128172.4	117426.8	58739.44	60088.53	34104.12	83846.63
36266.99	79619.02	57623.2	60365.97	118706.4	122418.6	57059.16	40573.57	30383.15	86024.59
43253.72	68847.14	57985.21	61170.74	112946.8	105133.4	46752.03	38848.08	22198.44	89976.15

LN326.988	LN326.988	LN326.988	LN326.988	LN326.988	LN326.988	LN326.988	LN326.988	LN326.988	LN326.988
63562.45	106957.6	23300.17	27792.78	82212.35	64682.07	88724.45	20544.78	60581.9	45651.65
97359.1	122283.7	21811.75	25825.22	110741.5	57411.94	76156.17	21869.8	43629.07	34737.63
79586.88	114121.2	22147.97	27981.43	91273.07	62474.71	85390.7	32154.93	71217.03	45927.54
55309.47	126050.8	26772.73	28184.4	101353.1	43308.88	92038.91	27126.34	46892.37	38735.38
70392.09	113056.9	28800.67	30866.16	87941.71	61630.46	114011.9	21135.02	43301.35	44724.22

LN326.988	LN326.988	LN326.988	LN326.988	LN326.988	LN326.988	LN326.988	LN327.830	LN328.826	LN328.903
35840.77	23902.84	24676.8	74582.8	90592.38	38036.52	39930.96	32315.94	104791.6	55968.26
24892.06	21986.43	24001.59	72474.67	146386.7	49628.09	40914.14	26922.4	110579.8	45753.61
25522.83	25129.06	24770.88	99267.13	89234.74	43759.05	44464.75	29838.74	127509.3	43997.47
16962.41	17760.1	22291.09	85616.23	89507.89	49171.99	33704.59	36745.27	141456.5	43252.33
21290.12	20404.37	20246.62	72837.07	115489.5	51505.82	40931.11	37985.32	159196.1	45402.38

LN329.269	LN329.270	LN329.27_	LN329.27_	LN329.828	LN330.273	LN330.806	LN330.826	LN330.899	LN330.899
88110.8	14326.42	18615.65	14362.48	8702.878	12807.4	24338.26	31008.37	21858.53	26013.88
75069.92	14263.85	15626.83	14453.5	6499.677	11872.92	21807.07	24790.38	20568.23	24929.24
76385.92	15949.04	19732.36	15948.75	7650.121	11801.47	19561.54	26166.09	21529.33	20333.12
86614.59	18120.43	24037.61	21652.44	11334.63	11371.42	24470.14	25658.73	20044.84	25771.86
87610.44	19373.48	27193.56	17817.4	10816.53	13383.46	26464.64	40228.67	19345	26026.36

LN330.899	LN330.899	LN330.899	LN330.899	LN330.899	LN330.899	LN330.899	LN330.899	LN330.899	LN330.899
23521.17	22676.07	18812.01	15083.21	19752.78	19184.31	16959.87	31784.06	24757.21	29414.87
25239.62	23445.3	17996.5	13225.97	20461.48	18007.38	18924.28	30719.4	24538.05	26305.51
24967.72	21364.87	17002.11	13992.2	17380.8	17613.27	15270.06	29537.76	22119.37	26010.48
22784.31	21513.36	16532.91	13723.42	17575.77	15020.95	18472.61	31246.53	20794.35	26810
24872.91	21210.81	16299.14	15146.22	20940.53	17737.41	13639.48	29607.21	22661.25	28224.33

LN330.899	LN330.899	LN330.899	LN330.899	LN331.176	LN331.176	LN331.176	LN331.176	LN331.175	LN331.176
14632.65	14877.07	17937.16	14237.81	19281.6	24234.08	18548.35	29943.87	12714.71	28129.44
13999.36	13064.41	15629.86	14666.82	16928.76	24768.3	16753.42	26482.55	15386.15	27180.01
12865.79	13846.69	16958.89	14685.18	18220.8	24163.97	17801.9	30004.53	16196.95	28505.08
12794.25	12988.58	14362.57	14072.39	17073.53	26329.38	18160.71	30246.4	11740.95	25916.69
12967.43	14799.99	15994.14	14670.92	15530.19	25090.48	19478.59	33008.43	15594.98	26353.93

LN331.176	LN331.176	LN332.868	LN332.997	LN332.997	LN332.997	LN332.997	LN332.997	LN332.998	LN332.997
21910.77	24075.64	15961.81	14990.13	44501.3	36807.1	61090.99	77996.57	25929.94	91933.42
24268.81	25598.75	13756.27	13170.22	59423.03	42491.15	66822.99	63855.11	17620.68	69141.86
21060.95	21771.15	13272.81	13798.91	59584.97	34826.45	100110.1	58570.29	21836.17	92898.37
21682.37	25076.51	15767.87	10943.78	44474.42	39132.21	69634.59	50724.14	22922.9	67034.08
24564.52	29017.16	19287	15189.93	54656.32	39111	71188.8	57774.2	20512.79	67298.51

LN332.998	LN332.998	LN332.998	LN332.997	LN332.998	LN332.997	LN332.997	LN332.997	LN332.998	LN332.997
16909.92	18949.26	37485.61	64355.6	24787.24	43756.99	54165.32	124251.4	33924.61	89848.62
11227.29	15829.55	37875.24	57691.7	21435.75	68007.44	54243.69	75996.27	29078.92	95643.9
10155.92	16527.82	52652.07	58640.05	33276.16	41844.88	42047.49	86424.28	44029.81	109856.9
13349.68	18391.57	41929.7	49247.25	21114.8	52889.18	51944.96	89050.02	24766.47	86377.67
11720.98	18042.12	43365.48	53457.46	23026.56	51937.25	48490.3	90736.31	27620.54	87758.67

LN332.997	LN332.997	LN332.997	LN332.998	LN332.998	LN332.998	LN332.997	LN332.998	LN332.997	LN332.997
70602.05	47400.45	67512.22	39689.12	30414.93	42462.63	86611.37	28951.44	85574.87	50108.84
37789.15	41099.55	73994.18	49319.12	36099.04	53963.63	58708.28	48349.88	60567.79	43301.35
54271.3	31439.85	90692.2	43667.72	30309.79	34297.44	91748.57	47568.81	71463.18	52261.93
52967.73	51606.73	90136.14	43069.71	33175.91	44275.28	62867.03	38930.83	66961.74	48161.25
51430.78	48886.23	82313.78	42212.27	32284.46	43543.21	61087.35	39212.72	60205.11	52736.45

LN332.998	LN332.997	LN332.998	LN332.997	LN332.997	LN332.998	LN332.997	LN332.998	LN332.998	LN332.998
42731.79	45846.17	39494.71	60421.74	79834.77	38516.79	52335.81	51715.29	27307.4	24586.51
29100.21	42464.84	40080.24	76276.97	91297.74	36929.56	40764.28	45273.61	31902.55	23257.74
43888.75	44846.7	51276.35	41633.23	55366	45804.71	58086.91	46101.91	43539.07	17744.24
29375.05	34772.24	42023.64	54603.87	60687.28	48488.81	55627.04	35327.53	37667.18	22423.04
30998.39	34709.12	39168.91	52083.23	57918.95	45843.81	58509.87	34668.81	33918.98	20831.79

LN332.997	LN332.998	LN332.998	LN332.997	LN332.998	LN332.998	LN332.998	LN332.998	LN332.997	LN332.998
64296.52	37289.27	25899.17	50067.25	23459.53	31348.63	22323.18	25139.17	68160.12	28804.46
70925.87	49101.28	32690.73	47155.56	25879.28	24352.89	26571.46	47333.76	58755.58	25021.15
64402.56	42086.45	44609.69	46002.67	16244.26	25778.25	17877.68	22409.68	61836.81	32153.92
70534.78	36074.9	30292.55	48297.39	19439.04	24221.16	20448.15	33190.61	51549.94	27842.75
68021.05	41242.63	29355.56	46174.42	17004.56	22072.01	20161.36	31985.97	41538.15	27023.59

LN332.997	LN332.997	LN332.997	LN332.997	LN332.997	LN332.998	LN332.997	LN332.997	LN332.997	LN332.998
55651.23	48192.13	42832.97	56859.82	51854.85	47488.95	47561.25	77780.45	54054.45	19801.03
47842.96	47707.29	63681.56	73361.49	61116.77	55802.26	45533.43	61430.08	51005.08	17781.36
65130.61	37921.78	74867.35	66709.43	87236.97	60854.72	35837.08	69962.78	56804.12	23603.2
43456.18	49950.51	56157.76	62643.11	55248.02	50198.22	39643.75	79551.34	61959.66	17993.07
45522.97	47856.67	56318.84	66535.25	53645.6	38267.74	37829.46	75968.71	56001.01	19136.08

LN332.998	LN332.998	LN332.997	LN332.998	LN332.998	LN332.997	LN332.998	LN332.998	LN332.998	LN332.998
70328.04	64051.33	76695.31	53500.49	31633.53	54505.12	46569.56	40561.16	19899.77	50132.7
48839.15	38690.09	73864.63	54183	60099.55	64878.75	64201.6	34703.36	19723.5	54784.24
58471.12	51585.43	54987.35	75125.73	41734.18	62841.7	50295.49	44918.95	15321.51	53966.81
49005.91	48761.35	65908.06	59737.06	39846.41	58270.1	55560.81	38352.49	15894.64	54760.42
48665.68	44991.28	67735.51	52539.24	40069.52	54088.82	57420.22	42805.45	16281.79	48358.67

LN332.998	LN332.998	LN332.998	LN332.998	LN332.998	LN332.998	LN332.998	LN332.997	LN332.998	LN332.998
42006.91	37760.31	43484.22	24161.05	44288.48	32047.91	61581.21	71591.74	39612.16	27633.84
35197.53	34676.78	43685.78	18229.9	50832.93	28833.88	54201.91	76837.82	31901.69	30731.69
29571.52	38679.08	27270	17074.77	41378.85	47727.03	68125.39	49745.02	45643.35	27870.13
33859.92	27501.38	36782.68	20720.07	38606.34	32392.03	60502.09	74978.79	32012.37	32181.91
38265.93	30397.11	36165.65	18673.61	32819.08	33135.41	65148.74	78532.44	30724.83	32671.74

LN332.998	LN332.998	LN332.998	LN332.998	LN332.997	LN332.998	LN332.998	LN332.998	LN333.155	LN333.155
31476.53	38625.7	38508.55	37046.14	58367.38	27904.92	22648.41	18036.17	62887.77	40924.41
31013.68	36100.61	38055.91	73102.4	61512.57	33931.61	21137.6	21097.05	58091.59	42633.98
40739.22	40239.98	31228.24	65595.48	63713.16	28003.65	20820.36	15980.93	65077.74	45062.84
33418.47	39401.63	35472.71	46164	65167.3	31286.24	18832.48	20831.73	58609.11	51476.75
31994.26	35424.55	31423.57	47866.91	60225.13	30241.31	22422.5	19742.02	57980.47	45625.92

LN333.155	LN333.155	LN333.155	LN333.155	LN333.155	LN333.155	LN333.155	LN333.155	LN333.155	LN333.155
30195.99	70120.22	14854.41	49091.65	43396.63	55684.24	73794.66	49831.88	41892.59	45120.92
30695.26	32165.66	14362.05	60871.39	31274.08	52528.2	74474.2	52996.49	45777.01	46249.72
34518.83	40807.97	12516.3	59430.65	39430.61	44194.51	81743.47	62245.04	31990.46	45851.68
27808.56	35797.41	14638.43	69408.68	29374.6	68104.22	59676.28	48350.76	50186.11	42622.98
30056.15	40914.77	17085.82	53656.72	31406.48	57500.74	55266.65	41840.05	41013.26	43041.73

LN333.155	LN333.155	LN333.155	LN333.155	LN333.155	LN333.155	LN333.155	LN333.155	LN333.155	LN333.155
61284.76	43624.36	63696.92	48054.1	105366.8	72454.87	66000.8	105530.5	42327.46	41381.63
54438.02	55674.73	70095.71	84168.36	89815.65	47299.1	57819.17	70737.33	36510	35610.53
56005.02	33863.3	79116.95	52160.87	80214.43	65679.72	45990.41	78261.13	32205.46	38234.23
44877.94	37118.63	60217.56	42256.33	104298.6	66531.74	52614.23	83564.54	45563.73	53739.98
48723.56	35070.72	60447.1	58928.55	71195.91	51152.85	54084.61	66853.55	42680.37	29556.02

LN333.155	LN333.155	LN333.155	LN333.155	LN333.155	LN333.155	LN333.155	LN333.155	LN333.155	LN333.155
54624.55	51015.34	47656.21	139355.1	27804.83	27721.32	41500.19	29924.63	36796.16	91219.41
68451.89	68678.5	71965.07	57205.14	28213.69	32190.28	47828.02	25918.81	54579.62	76593.69
77554.95	72014.98	77671.73	67992.89	27755.5	40467.46	54651.62	18814.57	42400.12	59508.76
59203.31	71243.23	49071.04	78684.18	34798.43	34792.62	50549.49	28114.67	50430.5	95100.86
53703.69	54627.78	59538.26	71824.13	25549.42	35605.55	42540.88	29988.22	52960.47	83470.2

LN333.155	LN333.155	LN333.155	LN333.155	LN333.155	LN333.155	LN333.155	LN333.155	LN333.155	LN333.155
64106.86	62121.25	45766.87	33884.49	98502.54	43595.87	57828.16	36456.32	32480.72	47322.45
38605.26	85841.62	38193.44	41808.7	88873.18	59002.36	51611.6	42538.93	43715.35	50878.26
49376.25	75820.28	50107.1	45243.05	101436.1	62980.31	61859.96	31138.29	41230.65	47685.17
53648.64	52899.96	60880.57	55810.74	72646.33	69184.06	68118.87	45344.85	45953.36	42847.43
41062.58	53383.77	42025.07	34260.37	96941.71	50820.81	57311.27	35870.77	36843.24	53143.78

LN333.155	LN333.155	LN333.155	LN333.155	LN333.155	LN333.155	LN333.155	LN333.155	LN333.155	LN333.155
63975.5	103057.4	94308	38738.05	93706.21	53321.49	54226.87	71418.64	20947.22	66115.42
74434.81	78483.96	91885	61753.59	85884.27	56439.46	46377.19	49332.54	30662.43	78022.72
50210.35	56718.51	75095.29	43788.51	72170.09	39705.78	42527.29	45732.39	25530.93	41805.62
84779.55	88983.73	102010.9	39026.81	62937.23	60760.22	45882.04	47902.16	25306.44	51803
62897.65	86061.21	83682.67	49020.06	69991.75	52944.56	39419.72	55410.09	21855.89	47491.82

LN333.155	LN333.155	LN333.155	LN333.155	LN333.155	LN333.155	LN333.155	LN333.155	LN333.155	LN333.155
27826.73	47635.79	44527.1	60110.59	42708.56	73303.45	66356.34	56313.52	48336.76	63592.15
24990.64	42880.81	45103.21	56698.14	49904.24	66833.5	61880.44	59266.64	59447.01	60585.49
35283.47	38153.81	39577.92	49040.37	35795.86	62199.16	72071.51	40076.52	68413	54399.29
25306.14	43550.34	49384.45	52730.81	55221.26	56831.56	71597.88	38445.55	68928.37	69630.19
25635.76	40941.06	41383.72	44763.6	39245.74	64169.86	58978.58	40933.82	46424.14	58970.69

LN333.155	LN333.155	LN333.155	LN333.155	LN333.155	LN333.155	LN333.155	LN333.155	LN333.155	LN333.155
42272.23	51464.07	36353.28	31642.67	70366.21	22044.43	30100.52	42286.71	45592.35	57128.23
28142.86	71720.74	35307.96	28668.16	68326.38	20310.41	36604.82	47406.48	43725.4	56704.92
51582.15	64417.92	40194.97	33648.18	56766.45	21265.03	34899.15	33849.92	30235.22	52429.13
33997.61	48376.59	40804.18	28668.81	56232.06	20826.39	27742.38	37457.75	42839.82	55935.34
31516.88	48862.58	37775.77	29170.87	71536.23	25084.77	31668.66	39876.67	39791.05	57357.74

LN333.155	LN333.155	LN333.155	LN333.155	LN333.155	LN333.155	LN333.155	LN333.866	LN333.894	LN333.895
38672.8	58916.78	77135.09	20279.32	40606.44	56193.21	29029.12	19346.92	23802.36	16054.88
45825.11	51200.93	51256.09	32547.85	44876.15	45494.5	24480.73	17697.85	25193.3	15049.38
47872.93	54491.95	63824.01	27850.44	36028.95	69889.89	32800.89	18062.61	24886.91	13937.04
42092.01	69869.03	58860.29	21038.29	51411.7	38218.02	25851.95	22493.09	23451.99	15565.31
43636.06	56203.32	63177.9	25480.8	43323.04	41068.36	23165.17	26086.58	18440.24	15022.64

LN333.937	LN333.937	LN333.938	LN333.938	LN334.863	LN335.134	LN335.134	LN335.134	LN335.170	LN335.170
65629.39	66601.04	63985.15	53248.81	452499.1	23210.11	25831.04	16708.86	18740.74	19956.27
76594.39	69021.31	53865.54	57160.44	418500.4	18238.99	28888.51	18156.23	22152.54	21523.85
72780.63	66562.87	55327.52	49324.13	470331	22135.44	26631.17	17414.49	18933.72	19696.05
68622.64	65526.3	51058.3	47253.88	528269.4	21090.33	27499.12	16486.32	15560.29	21207.74
65527.83	79876.86	49842.09	49888.7	644497.1	21703.28	26672.7	14371.94	18600.21	19731.24

LN335.170	LN335.170	LN335.170	LN335.170	LN335.170	LN335.170	LN335.170	LN335.170	LN335.170	LN335.170
18646.61	17218.43	19540.49	15538.17	17034.98	20117.64	14242.43	25136.26	17924.25	16305.51
17893.56	16620.16	19785.62	17288.58	15847.12	21163.99	14859.37	24757.59	19186.49	17132.74
17062.68	18206.72	19328.54	19457.44	16699.88	18321.06	13106.62	23032.39	17910.69	13561.35
19565.35	19034	15544.71	16186.83	15794.84	17961.25	15425.21	24602.92	16637.74	15760.83
24336.72	14840.22	18943.47	17619.27	15516.44	18121.23	13508.71	22777.26	15165.32	14549.52

LN335.170	LN335.170	LN335.170	LN335.861	LN336.858	LN336.931	LN336.972	LN336.972	LN336.972	LN336.972
23352.64	20642.73	11948.31	394509.1	4195595	26767.22	33852.05	60640.01	70471.76	50995.02
22394.65	20944.66	14817.16	363314.4	3447392	26503.34	33902.69	61644.41	73431.31	53069.26
24742.16	18933.04	12131.06	350237.9	3742906	26526.29	34397.21	56344.81	71804.62	49686.44
24632.95	19348.83	12176.8	422603.1	4467628	26648.75	16610.47	54203.27	75847.28	50382.33
20834.34	20766.78	11357.85	512083.7	5340806	27008.18	35821.85	62645.21	71279.83	47099.31

LN336.973	LN336.973	LN336.973	LN336.973	LN336.973	LN336.973	LN336.973	LN336.973	LN336.973	LN336.992
26894.09	36768.31	29775.25	35226.53	38569.02	35524.91	72803.3	34034.75	34748.02	38197.88
25202.57	42078.83	29719.31	36508.3	37297.89	40189.42	76561.2	32935.7	35520.67	34411.25
26823.84	36555.88	26402.43	35195.88	33060.78	41433.31	72790.55	31059.11	34969.46	35886.14
27569.24	38369.42	27097.61	32321.72	37727.09	41747.98	70846.18	33646.66	33763.73	35716.69
22975.3	38785.72	27152.92	31171.69	36911.06	34015.89	68695.86	34297.37	37655.19	39881.13

LN336.992	LN336.992	LN336.992	LN336.992	LN336.992	LN336.992	LN336.992	LN336.992	LN336.992	LN336.992
18938.76	23144.4	19129.22	23524.23	38392.89	21533.34	32006.05	21993.13	25120.07	18766.71
21117.79	23317.58	15900.28	25337.26	33025.64	20651.7	31453.65	21410.27	22525.93	20972.43
19761.22	23699.15	17403.9	24510.58	35228.3	21521.97	31731.8	18549.15	24878.01	21295.44
20882.8	23285.31	17696.02	24271.14	34732.8	21113.67	30460.3	18270.31	23322.28	19686.84
21557.35	23896.11	16348	22316.15	35260.26	20958.05	27315.07	19096.21	19218.99	22309.75

LN336.992	LN336.992	LN336.992	LN336.992	LN336.992	LN336.992	LN336.992	LN336.992	LN336.992	LN336.992
27794.04	48495.37	35284.8	17459.77	40964.09	12248.78	37904.57	42206.1	56833.86	13290.28
28402.52	56308.68	36535.58	16166.94	41657.85	14179.69	36917.13	45558.78	57949.02	12653.14
28462.03	50740.84	33920.11	16290.92	39416.19	12419.9	33963.3	41602.12	63094.1	11089.98
27423.14	51991.2	40354.67	14776.77	40250.85	12788.39	40542.81	49232.8	58776.94	11696.63
29032.87	53366.97	32940.38	15394.87	37386.69	12305.61	34179.38	42716.84	63171.01	12991.86

LN336.992	LN336.992	LN336.992	LN336.992	LN336.992	LN336.992	LN336.992	LN336.992	LN336.992	LN336.992
38720.95	32118.42	15923.52	44601.67	24918.76	33039.72	19750.95	14729.52	17288.82	20849.69
41065.84	31830.38	14440.44	39765.7	26474.45	34951.36	18371.12	18194.34	20080.73	22684.1
41013.8	30268.99	16232.38	43000.76	23699.76	29417.8	17967.22	13179.13	19231.23	20073.09
40785.98	31917	15012.62	44950.84	22681.03	33607.1	20092.04	16882.11	20265.49	19739.95
36651.56	34374.77	15197.83	43033.02	28153.45	29625.98	20448.88	15680.78	17452.4	20904.42

LN336.992	LN336.992	LN336.992	LN336.992	LN336.992	LN336.992	LN336.992	LN336.992	LN336.992	LN336.992
28760.17	32635.63	33738.03	34560.54	26112.88	24511.1	29975.53	41001.55	17070.88	37104.44
31633.32	32585.84	31770.6	32025.36	25774.86	25475.16	28404.77	42214.13	17786.61	35237.57
27713.01	30509.51	31832.95	33994.13	25270.44	21545.18	31989.9	39536.22	15539.77	37280.24
27542.37	28394.97	32604.57	32967.3	24415.84	24822.95	30517.97	41744.15	17033.29	34012.31
26203.04	29973.82	31014.5	31710.4	24363.15	21363.63	26454.55	39331.93	17697.24	35788.31

LN336.992	LN336.992	LN336.992	LN336.992	LN336.992	LN336.992	LN336.992	LN336.992	LN336.992	LN336.992
57004.5	22940.48	43165.92	25699.89	24060.58	50180.65	20215.19	28852.32	25374.77	47265.12
65392.76	24680.56	41306.95	23887.36	21601.09	46113.81	18125.04	28130.76	24425.41	45349.55
53014.23	21483.75	39661.73	24395.72	26562.24	46986.61	20003.13	27650.05	25771.86	40266.39
58745.67	23453.1	36622.79	22342.85	24416.24	46507.82	18349.24	26212.47	24454.19	42259.77
56805.74	24384.15	46584.72	23731.59	23859.14	50486.62	15182.98	33552.13	26208.63	47953.87

LN336.992	LN336.992	LN336.992	LN336.992	LN336.992	LN336.992	LN336.992	LN336.992	LN336.992	LN336.992
36385.18	14259.75	14728.31	26456.9	33487.88	29927.5	23771.21	11776.74	33326.11	25577.78
37796.38	12363.62	11937.39	26582.97	34512.5	30665.99	24593.26	17084.82	35725.09	27210.45
33993.74	13316.11	13537.23	25265	36503.1	29809.05	24083.44	13239.92	35346.73	28782.66
33284.5	12677.15	10311	30551.84	34954.72	30261.42	26266.09	12966.72	33626.59	27563.22
37211.46	12937.25	12316.75	25628.33	35988.05	28494.84	25722.95	14164.22	30122.84	27012.56

LN336.992	LN336.992	LN336.992	LN336.992	LN336.992	LN336.992	LN336.992	LN336.992	LN336.992	LN336.992
34634.06	36727.25	46689.46	18316.06	24899.13	25516.45	16486.83	35881.14	28124.96	32952.89
32443.97	34736.79	45232.98	18809.47	23596.54	27795.38	14555.35	32997.2	27206.85	33365.68
33372.6	36108.68	44990.17	19380.58	22506.02	25556.75	13735.79	31851.22	28926.99	36927.71
31210.24	38436.65	47834.31	18575.13	20999.26	25202.8	17282	33483.2	26722.3	35371.04
32626.73	34466.76	42806.78	16736.91	21916.93	25621.45	13970.76	35743.06	27712.8	33431.21

LN336.992	LN336.992	LN336.992	LN336.992	LN336.992	LN336.992	LN336.992	LN336.992	LN336.992	LN336.992
28705.34	25516.29	23222.61	29837.25	38966.1	43916.36	46027.8	38249.37	33097.5	21901.45
27842.88	23990.52	22007.5	29814.62	39262.82	51341.61	47148.67	39762.25	37430.7	19849.38
27442.98	24260.89	23410.03	28307.82	41822.71	50441.36	41580.1	39381.2	35092.3	20733.02
26838.21	24917.81	20619.23	32209.88	40505.94	46822.9	42698.61	39490.58	33981.57	21380.8
28106.72	25083.21	19567	28308.54	39599.64	44898.35	40254.61	43603.48	34489.73	21423.56

LN336.992	LN336.993	LN336.992	LN336.993	LN336.993	LN336.993	LN336.993	LN336.992	LN338.858	LN338.988
25101.02	19503.19	42298.06	31325.18	32794.14	25699.42	29581	31458.93	1153118	39925.45
20648.9	20436.43	43093.12	32492.71	30100.79	27138.61	27531.89	31251.74	984595.9	39120.9
23996.84	19134.22	39755.07	31297.27	32701.34	25409.64	30225.9	31733.99	1079448	43375.15
24869.5	18451.38	44330.96	35026.21	34253.45	26710.95	29893.82	27942.57	1094117	41597.67
22881.92	19679.16	39375.14	29078.66	31520.63	26313.19	29004.49	31001.37	1292062	39290.15

LN338.988	LN338.988	LN338.988	LN338.988	LN338.988	LN338.988	LN338.988	LN338.988	LN338.988	LN338.989
24407.36	23624.06	56179.4	53178.45	34559.84	15012.69	36131.76	20873.84	22659.04	21131.32
26352.87	25291.38	55042.91	56930.38	34680.65	11953.66	41138.17	22424.55	23752.74	17778.9
20173.11	23666.18	49497.46	29088.31	33230.28	12191.75	38188.77	24181.17	21473.28	23584.43
26137.36	23038.11	58925.71	52696.3	31882.05	14292.56	41667.5	25166.98	23018.73	17894.63
25226.21	26253.89	57547.91	56217.11	36244.94	13021.24	43738.9	22490.49	24748.52	18718.18

LN338.988	LN338.988	LN338.988	LN338.988	LN338.988	LN338.988	LN338.988	LN338.988	LN338.988	LN338.988
44092.83	29374.4	47333.55	60001.41	35740.97	45247.65	34235.85	15058.09	28105.68	20696.68
47774.67	29162.44	46463.4	53612.63	38671.37	46269.06	35395.18	16243.42	29850.76	19999.48
43793.99	43125.2	53329.32	64989.54	50183.06	39997.8	36932.27	16214.5	34105.94	18241.62
45814.12	29332.17	52318.55	52469.77	33503.95	44520.35	34224.54	14549.55	29402.67	19352.45
47060.34	30813.36	47033.42	55162.9	32619.49	49854.48	33634.14	14818.2	31289.21	19331.59

LN338.988	LN338.988	LN338.988	LN338.988	LN338.988	LN338.988	LN338.988	LN338.989	LN338.988	LN338.988
43996.87	42510.13	41237.01	44519.51	44050.18	23817.52	39806.88	16053.51	41662.65	25293.24
41441.07	39851.22	45274.76	44176.05	47534.08	23281.08	41527.46	15339.16	40464.6	23905.41
43635.32	44086.71	59083.47	48778.91	45766.91	20853.6	33502.79	11818.08	41337.49	36884.96
41918.66	42656.57	43483.17	47998.85	40782.5	24827.29	42414.81	16685.61	44046.21	26676.32
42139.03	37279.26	43218.36	44027.02	42060.89	25823.89	43766.08	14592.09	45705.52	30428.24

LN338.988	LN338.989	LN338.988	LN338.988	LN338.988	LN338.988	LN338.988	LN338.988	LN338.988	LN338.988
14880.85	19121.59	48656.93	36912.85	45669.99	19742.55	38122.35	23652.27	20897.75	43326.87
12121.95	20226.65	49473.04	41018.37	40888.76	19055.21	39170.94	23316.45	22456.15	39769.42
11538.83	16178.19	45079.09	51539.62	37104.31	26740.93	37052.88	22940.91	12932.96	40066.56
15238.83	17739.14	47602.04	40925.12	41873.01	18798.53	41945.64	21373.9	24389.41	40684.37
11557.92	17612.78	52102.64	38253.09	39242.06	18764.49	43558.32	23575.63	19307	42427.71

LN338.988	LN338.988	LN338.988	LN338.988	LN338.988	LN338.988	LN338.988	LN338.988	LN338.988	LN338.988
68106.3	40292.2	33197.42	24195.62	38588.54	43704.74	53095.7	56309.47	53180.84	57253.73
59454.48	38497.59	30883.85	23361.98	38530.53	47799.69	50344.64	57799	53695.06	55731.02
68707.11	44136.97	36759.55	25720.72	25980.64	36426.54	51285.81	57441.19	41112.47	53702.09
62512.16	44126.44	29848.5	24540	37829.44	43192.52	57710.54	60826	53025.66	55730.17
62994.76	41874.47	29993.66	23834.82	34628.66	40084.3	58279.63	56201.66	57705.72	54255.82

LN338.988	LN338.988	LN338.988	LN338.988	LN338.988	LN338.988	LN338.988	LN338.988	LN338.988	LN338.988
41015.17	65219.33	29767.73	36282.39	35449.95	31948.59	17125.05	33865.32	27223.66	52775
42635.91	67243.97	35600.71	37631.7	32271.47	32734.26	19238.62	31250.64	25414.48	56774.91
38055	80958.48	36892.07	35904.16	32750.35	32492.6	19853.05	35049.81	24803.55	84902.94
45462.12	68435.44	35317.08	35829.64	33046.76	32864.77	18690.85	32867.05	23682.13	54221.47
44038.62	65205.79	36910.2	37247.88	32427.1	32722.25	17293.89	31795.52	25429.98	59390.13

LN338.988	LN338.988	LN338.988	LN338.988	LN338.988	LN338.988	LN338.988	LN338.988	LN338.988	LN338.988
18142.04	47397.17	51874.85	31064.49	25309.57	26817.76	47191.9	24894.13	27434.56	82000.93
19312.7	46978.82	49100.88	31251.06	31659.17	25903.91	50591.78	24278.45	31989.54	80924.84
21073.59	45673.46	45365.36	35217.27	27071.38	24834.88	44342.4	19581.29	26178.45	66565.05
20673.23	47927.56	45379.41	34728.71	28092.02	24252.47	45614.38	24084.03	28932.11	75756.13
18262.95	46692.88	47599.06	34158.61	28456.46	26325.86	43595.32	21129.61	25281.69	73956.99

LN338.988	LN338.988	LN338.988	LN338.988	LN338.989	LN338.988	LN338.988	LN338.988	LN338.988	LN338.988
39364.04	38800.31	28438.08	27592.14	18209.13	70346.4	28471.59	28712.78	26877.71	69004.03
39450.57	38313.92	28339.9	33365.77	17658.76	67035.42	26106.63	29602.98	26490.33	65185.36
29857.62	42167.88	47962.13	29202.5	16220.18	65302.48	25225.7	30390.49	27283.6	69842.08
43783.7	38666.86	29985.98	32151.18	17327.3	65665.36	28707.78	32246.02	26107.32	67863.09
43355.62	36693.31	26550.26	32302.21	16739.74	64820.79	26287.67	33263.26	28783.01	72232.08

LN338.988	LN338.988	LN338.988	LN338.988	LN339.199	LN339.232	LN339.232	LN339.232	LN339.232	LN339.232
63988.38	31466.61	46547.3	45721.41	64066.9	21047.21	20547.31	17807.64	18269.35	20652.93
58630.31	28474.82	48508.26	47074.51	58024.51	18580.4	16895.62	19989.58	18923.05	17095.25
53193.93	31862.87	51674.98	72157.55	68501.27	19099.24	17886.31	18923.84	19588.58	18750.05
66736.95	29115.69	50524.82	44699.69	73065.43	18835.52	19503.2	17103.22	17937.05	20485.46
66938.98	31541.87	43033.27	44322.49	77911.14	18712.58	18409.14	16462.74	17571.35	18069.28

LN339.233	LN339.233	LN339.326	LN339.858	LN339.912	LN339.912	LN339.912	LN339.912	LN339.912	LN339.912
15139.72	17651.23	10332.25	68261.77	26104.05	22974.31	22446.48	28304.68	18008.92	21701.75
15850.33	17907.32	12044.8	67725.4	22360.86	20753.52	26386.47	30828.7	20458.98	27353.54
15866	14775.08	12579.14	60512.86	22029.47	25439.75	20725.55	28202.77	19283.21	23913.17
14373.84	15490.08	14652.95	55894.59	26580.96	22202.73	23942.7	29611.81	18133.54	22601.86
12553.43	16293.34	14284.13	82823.16	27437.88	22748.17	22515.21	27702.32	20260.51	26364.05

LN339.912	LN339.911	LN339.911	LN339.912	LN339.912	LN339.912	LN339.912	LN339.912	LN340.210	LN340.853
40281.64	14363.06	17194.86	22117.86	19284.37	28115.7	22321.68	31787.11	21363.23	419333.9
41448.82	13554.65	15890.53	25838.39	20398.95	26615.21	24063.84	29957.55	21619.11	338826.6
39881.76	16354.59	14127.38	21080.74	17191.63	27900.61	22711.72	28830.73	22770.81	347673.2
34680.77	14051.24	17581.87	25107.44	19189.76	24365.1	23113.05	29105.93	17250.58	350268.7
36053.61	12505.07	13899.54	25177.12	18452.64	28514.32	23547.07	27052.84	19338.26	468165.2

LN340.941	LN341.004	LN341.003	LN341.003	LN341.004	LN341.003	LN341.003	LN341.003	LN341.003	LN341.003
32800.26	91025.36	93858.64	75305.42	47428.74	98723.44	70593.18	75656.48	97038.86	92356.46
143000.2	59388.55	97870.2	68793.59	62768.51	121561.5	53866.38	85201.08	83384.42	123247.9
148956.7	68501.85	133562.2	73117.5	72491.08	118475	80154.49	66674.29	93622.77	116091.3
151727.1	60357.03	86961.42	64297.19	59432.34	95776.31	66110.87	73058.03	126544.6	98160.02
11390.57	53082.63	87536.88	109269.5	39427.47	101658.1	71719.53	69216.65	77346.09	69630.04

LN341.003	LN341.003	LN341.003	LN341.004	LN341.003	LN341.004	LN341.004	LN341.003	LN341.004	LN341.003
148495.2	112528	48449.38	91233.69	24452.77	75483.74	34514.13	126187.3	60581.96	105779.7
113983.6	152216.1	60458.21	103854.2	19065.44	79202.09	39339.97	131343.8	44115.28	78969.67
110011.5	99511.56	65979.56	91146.34	14681.09	98454.56	27208.94	126233.6	65068.2	64102.91
87793.03	117752	78124.33	109897.2	20410.25	79332.27	30605.11	102370.1	40408.18	83338.31
104348.6	131984.4	62240.73	102289.5	22419.21	86998.41	30507.92	124508	52632.35	104123.4

LN341.004	LN341.004	LN341.003	LN341.004	LN341.004	LN341.003	LN341.003	LN341.003	LN341.004	LN341.004
76994.78	109931.5	146973.5	56539.32	61294.44	95911.48	90035.68	117720.6	39527.52	70541.68
79550.27	108969.5	111606.8	77133.46	60574.75	109781.4	127749.9	92263.82	31059.67	70102.32
48348.9	88547.41	109048.9	71882.05	52811.32	74205.47	106637.1	91810.64	30266.17	48093.22
72296.55	80924.07	150127.8	48035.94	55765.11	131160.7	99178.87	103642.3	30913.6	61299.15
69592.53	85717.89	130440.4	43872.96	76239.87	125281.9	78656.58	86801.63	29243.55	64426.18

LN341.003	LN341.004	LN341.003	LN341.004	LN341.004	LN341.004	LN341.003	LN341.004	LN341.004	LN341.003
128422.6	92540.06	102436.6	88805.7	49312.92	78149.44	123483.9	57644.56	65925.05	148382.3
98288.35	60832.44	114193.1	101321.1	46999.42	63545.2	106591.5	53637.45	49573.31	109357.5
120208.9	61906.31	89238.48	82470.1	53165.56	55403.2	113326.8	88666.43	110876.4	134137.4
99437.94	75234.71	92798.34	73446.24	40163.97	65646.82	236678.6	84974.24	83574.84	186403.3
123453	75502.74	140079.8	76197.23	67327.06	93685.73	158070.7	58683.79	83112.42	131658.6

LN341.003	LN341.003	LN341.004	LN341.003	LN341.003	LN341.004	LN341.004	LN341.004	LN341.004	LN341.004
78878.13	76697.7	33822.01	81150.56	76987.16	33213.85	56175.72	55159.34	57471.33	65219.3
79093.76	95646.13	53310.92	134771.3	80726.49	28110.28	36150.77	45674.62	42648.16	69888.89
83609.88	83285.8	42361.7	146580.8	75120.95	29511.64	47973.93	54449.09	46174.57	61243.25
142194.6	127847.9	47603.47	124417.7	89736.59	21617.02	47469.33	62825.83	71023.27	42264.58
108334.7	97566.81	44289.14	89177.61	98071.73	32944.61	55115.33	59512.63	56841.71	83402.28

LN341.004	LN341.004	LN341.003	LN341.003	LN341.003	LN341.004	LN341.004	LN341.003	LN341.004	LN341.004
96848.2	64323.98	131792.7	101143.9	97840.34	73260.5	87206.33	121747	90252.47	29928.47
115212.3	67629.42	137009	99993.95	137475.6	73270.71	94098.29	112765.6	73154.54	39873.47
125168.1	94441.47	132755.5	105852.6	136790.9	80792.89	89479.66	121612.7	89389	23442.65
97098.42	71817.59	97137.4	83248.5	97369.53	77641.25	81014.92	135512.1	83490.48	43506.26
74673.27	79797.9	148890.6	95414.63	95111.84	62741.03	119279.5	89515.07	107074.6	25633.37

LN341.004	LN341.004	LN341.004	LN341.004	LN341.004	LN341.004	LN341.004	LN341.004	LN341.004	LN341.004
66381.09	66146.96	43586.47	33771.82	74236.1	60866.89	88672.92	65998.46	61875.81	66545.84
90940.42	62064.79	56697.08	30690.59	58123.54	60817.03	66166.27	83089.54	50162.87	49011.9
72257.95	64987.18	43991.27	42292.78	80415.64	88663.85	76988.91	88222.75	48047.07	59532.53
64201.84	69926.07	39358.41	34811	72903.5	54404.05	82234	63012.73	62077.59	53651.8
70513.35	61808.02	50640.07	24957.54	50751.25	72792.98	89233.16	72483.53	99678.57	52096.19

LN341.004	LN341.003	LN341.003	LN341.004	LN341.004	LN341.003	LN341.003	LN341.004	LN341.004	LN341.004
47795.07	139808.1	128826.7	32543.95	26602.47	100317.3	57936.54	57958.23	48968.6	42879.7
40054.03	151947.2	86102.25	29392.43	42451.82	97557.52	52822.86	63951.6	60308.94	34697.76
66666.36	154282.9	100462	23762.75	36902.31	116800.3	67982.68	87452.43	44493.93	27974.62
59064.47	168378.6	86330.7	24279.99	31484.96	110680.6	64206.7	70163.12	51313.4	36581.28
42583.1	164384	88135.48	27333.93	38383.91	96476.54	47904.38	69364.13	51090.45	35634.58

LN341.004	LN341.004	LN341.003	LN341.004	LN341.145	LN341.145	LN341.145	LN341.145	LN341.145	LN341.145
37279.3	50463.8	69088.18	37998.99	56000.56	188619.4	182867.9	124614.4	100131.3	151645.1
33972.59	60121.65	85525	31724.54	86899.71	193734.7	184883.3	128088	104437.6	126631.8
33368.38	58863.14	68047.79	31989.32	61892.55	166575.8	158831.3	122274.4	100752.8	143019.1
54886.53	76089.27	71185.61	31637.93	72709.94	184587.8	188412.9	127755.8	98510.23	126125.8
34472.5	54882.19	71763.65	45998.22	68448.82	176128.6	180193.4	109516.9	96864.41	121964

LN341.222	LN341.269	LN341.269	LN341.269	LN341.269	LN341.270	LN341.855	LN342.852	LN342.890	LN342.890
28622.65	162651.3	207634.1	141462.7	92246.85	159846.4	35420	102255.6	17545.28	32191.09
27240.69	265538.9	253020	140050.4	89501.88	158609.2	28298.78	79739.15	19919.5	29093.64
34037.73	208690.2	184840.4	143057.8	96429.12	164881.1	27515.38	56366.79	18559.55	25630.75
27543.57	189537.9	184852.5	133228.7	96309.66	156479	36919.61	101986.6	15177.4	26274.22
28399.27	208522.6	199512.8	137500.1	87834.06	160131.7	30367.51	67881	16783.51	24433.43

LN342.890	LN342.890	LN342.890	LN342.890	LN342.890	LN343.819	LN343.893	LN343.893	LN343.893	LN343.893
20335.92	18802.07	30745.48	33059.57	29332.99	36633.01	20243.53	20499.09	23429.63	17185.18
20852.67	17972.68	32291.13	28335	27496.79	32193.31	19365.41	22277.62	19276.86	13388.56
20475.14	17632.36	33015.17	27595.28	32076.54	37874.9	18066.85	23845.32	19642.21	14799.51
17630.62	19684.33	34787.18	27325.25	30070.66	44049.91	16911.03	17716.02	18656.5	13241.5
21138.15	18621.35	32608.04	28818.66	29062.66	46329.33	16485.57	20133.71	20059.84	14487.11

LN343.893	LN343.893	LN343.893	LN343.893	LN344.851	LN345.015	LN345.817	LN346.177	LN346.849	LN346.864
16491.3	22961.97	18053.22	15127.39	38650.19	50582.47	10973.18	20938.54	14949.78	18556.6
20433.85	20593.84	19035.24	14145.34	44984.57	45751.48	5952.362	20100.67	16019.57	18060.88
16462.35	20995.26	17970.24	13945.08	43961.39	21518.68	11795.37	18356.01	13428.38	17317.31
16444.47	20012.47	17713.44	14606.37	35860.43	51523.92	11079.36	18178.03	15790.12	10620.49
14424.77	19340.79	16592.28	12792.83	34515.27	46755.8	12153.51	18841.72	17797.1	13975.86

LN347.013	LN347.013	LN347.013	LN347.013	LN347.013	LN347.171	LN348.859	LN349.186	LN349.186	LN349.186
40584.83	36995.45	36197.43	31296.51	37195.6	36324.78	139699.1	19510.6	24708.23	25849.38
40610.74	31702.41	39486.02	32340.19	40015.88	37044.56	90915.93	18839.76	25727.68	23484.15
43743.02	33673.45	33411.39	31211.47	35933.56	46568.93	130817.7	19815.9	27257.7	25412.44
36525.46	37721.28	37239.31	31308.07	35285.03	51082.53	162842	22211.96	29024.25	25389.45
39544.05	30919.79	38523.65	28408.88	35103.41	53831.52	189579.7	18978.03	26405.35	22170.72

LN349.187	LN349.187	LN349.186	LN349.186	LN349.186	LN349.187	LN349.186	LN349.186	LN349.186	LN349.187
24152.45	25548.69	30650.18	23012.92	21318.72	24697.9	23353.99	24583.37	24013.92	19417.42
24280.7	24041.78	31594.89	25928.54	21588.33	25287.32	27555.89	25694.32	25150.18	18981.38
22201.72	23106.74	32317.64	23419.18	23316.72	24443.97	25997	29186.88	24151.42	20408.26
22670.04	23986.7	28093.33	24687.17	23359.07	29050.98	25682.04	26940.7	23975.1	19486.37
21466.06	22663.61	28532.09	23973.28	22211.93	23659.72	24371.49	23830.81	23709.64	19606.87

LN349.866	LN349.903	LN349.945	LN350.857	LN350.910	LN350.987	LN350.987	LN350.987	LN350.987	LN350.988
122294.3	61681.99	18027.75	29400.65	26936.37	118461.8	126659.5	55241.06	86427.19	49627.3
102872	51332.67	71089.45	23900.42	27248.12	120953.3	122096.2	56372.72	80687.07	89153.94
94820.58	52598.68	59823.11	25645.35	30365.6	123767.6	122806.9	59397.35	82947.6	51760.78
123325.4	61807.42	63196.03	32514.86	33729.81	114607.3	123670.3	58656.4	79161.52	57863.78
141061.6	54735.7	63561.98	40725.83	47391.01	115531.1	120014.3	63488.73	80748.78	60013.88

LN350.988	LN350.988	LN350.988	LN350.988	LN350.988	LN350.988	LN350.988	LN350.988	LN350.988	LN350.988
19380.42	31655.98	53340.09	137251.4	47480.99	43679.01	54088.51	38465.55	85883.68	35351.76
19873.64	34438.17	52142.67	96563.31	82989.68	39380.55	40631.65	42528.79	78333.09	39761.77
19866.18	47553.15	29414.41	104620.5	68382.36	46880.99	42140.8	29586.8	65131.95	45134.63
20812.57	31332.66	56127.34	108065.2	83809.16	53420.41	43819.36	42178.88	119294.3	41219.52
24539.84	26017.72	51977.47	121853.3	63973.33	79702.28	40395.79	33293.71	78961.56	45311.19

LN350.988	LN350.988	LN350.988	LN350.988	LN350.988	LN350.988	LN350.988	LN350.988	LN350.988	LN350.988
39386.01	61644.4	49552.06	55170.82	81208.21	79998.44	48391.89	48410.5	40445.67	70866.23
49383.69	90317.74	34397.28	50300.37	92552.92	75789.97	42980.88	46172.84	48707.63	49868.87
40219.14	76868.67	63714.22	53443.05	70660.4	57831.89	43166.68	27863.52	48638.86	65950.54
51496.36	108933.3	37896.8	54239.74	104891.3	68872.81	35296.36	29145.53	44888.59	79201.96
47027.06	65832.28	72439.91	37912.2	59105.61	58035.72	51769.13	34107.11	36888.2	45423.22

LN350.988	LN350.988	LN350.988	LN350.988	LN350.988	LN350.988	LN350.988	LN350.988	LN350.988	LN350.988
63660.61	57354.22	86974.58	60598.3	66251.88	48547.96	104284.2	69354.8	85051.75	30086.71
88972.67	59688.06	62326.83	47253.45	97073.8	32588.74	55826.93	70975.22	75380.24	20610.46
61508.22	48370.48	85077.13	78422.19	81888.08	39526.06	63660.57	79620.58	70142.83	24339.41
70130.05	61660.64	83723.17	58373.95	105033.8	44222.04	77524.79	77486.82	82634.69	24286.15
72315.79	53340.47	51661.92	59740.3	86048.34	48429.6	53277.93	59216.62	78263.65	24380.9

LN350.988	LN350.988	LN350.988	LN350.988	LN350.988	LN350.988	LN350.988	LN350.988	LN350.988	LN350.988
24435.82	58066.4	55293.11	49696.06	40086.92	53654.2	25398.84	40956.81	30419.63	72912.1
23847.2	48605.23	60961.09	64126.38	43758.63	59368.89	43720.23	42688.45	38400.62	107697.4
29808.91	57020.22	50874.31	65251.05	50516.99	45885.85	25925.32	39160.91	39242.41	47011.28
32301.9	41169.21	70890.89	49081.91	46213.74	50527.12	34798.84	37395.5	42099.09	72302.88
29857.08	46293.23	58029.91	58259.47	43371.28	51519.68	26232.01	43163.85	32239.65	78566.57

LN350.988	LN350.988	LN350.988	LN350.988	LN350.988	LN350.988	LN350.988	LN350.988	LN350.988	LN350.988
110441.5	55009.86	65971.25	25260.54	46010.07	45203.6	139368	25069.95	19042.77	33812.48
66546.85	84357.5	51919.99	23672.5	50038.17	60578.05	84431.25	22675.36	25593.83	37032.12
59440.52	92570.44	46372.74	23762.16	45644.23	42534.62	110471.6	25052.52	21315.17	35307.81
74090.92	72405.32	53071.19	28769.56	54148.85	44742.41	120007.8	28482.63	24863.5	42106.09
56001.92	57158.58	72550.44	24108.23	35525.3	48985.87	84812.71	21607.84	23698.22	36127.42

LN350.988	LN350.988	LN350.988	LN350.988	LN350.988	LN350.988	LN350.988	LN350.988	LN350.988	LN350.988
54454.02	36148.13	51389.83	35708.56	63771.82	48186.26	60447.89	38739.87	97147.23	56814.52
59297.15	36006.69	41211.7	41336.37	79703.23	44974.53	97231.5	35905.99	122954	56812.7
44005.37	26360.61	40453.64	42803.89	71310.3	92068.36	70094.71	48502.27	95083.09	42587.48
55165.7	33981.94	37941.11	43167.62	45708.13	43961.92	87779.47	36818.44	119974.5	47981.33
51052.02	52051.6	49758.65	31557.92	73427.18	54777.17	78953.28	38668.53	107263.6	36975.85

LN350.988	LN350.988	LN350.988	LN350.988	LN350.988	LN350.988	LN350.988	LN350.988	LN350.988	LN350.988
50681.99	48124.61	23712	36874.76	35083.57	46254.77	74784.68	49673.07	24474.02	37422.51
83391.37	51083.96	20333.2	36045.02	41907.63	56216.78	70580.74	61389.09	23849.62	34800.51
70462.23	59141.26	25529.56	32268.9	44957.32	50367.22	78994.5	54266.91	18107.73	32817.67
63504.22	48715.34	29645.76	47937.36	37640.6	80912.29	62761.98	55194.94	20038.47	33356.18
69730.3	55155.79	22561.44	36374.85	67270.32	60204.42	73122.22	48304.39	27536.71	38548.33

LN350.988	LN350.988	LN350.988	LN350.988	LN350.988	LN350.988	LN350.988	LN350.988	LN350.988	LN350.988
18013.57	55138.8	30460.97	73835.04	63401.66	89380.4	79250.67	87981.72	85856.02	129126
23589.24	65546.02	38615.59	71943.47	82226.45	78158.46	95572.93	95709.82	64998.11	106404.2
20457.81	64649.91	51376.92	76107.69	56646.7	77938.7	98428.2	78350.57	84083.31	104360.1
29654.12	55916.85	38223.53	57383.03	66191.11	87684.26	110979	105013.8	69610.21	129762.2
25996.22	88429.25	36677.86	94339.85	77062.97	88679.98	133799.9	84560.97	83778.07	105258.1

LN351.251	LN351.853	LN351.866	LN352.852	LN353.182	LN353.181	LN353.182	LN353.848	LN354.851	LN355.000
251544.6	36064.28	42332.02	81480.2	17950.27	17802.4	14163.38	282115.7	22818.05	33217.53
274444.1	28518.98	33692.45	92314.98	17391.19	15815.59	11533.5	242352.1	15567.39	36226.08
242834.9	30381.61	37356.48	80700.13	15577.12	18685.02	12713.28	252397.8	19016.54	26483.01
248669.3	32958.85	42605.25	88254.87	19768.1	17492.59	14275.62	314036.9	22670.58	35781.26
221910.9	47266.46	32401.25	53886.58	17700.01	16728.15	12582.21	376507.7	28540.11	29417.39

LN355.000	LN355.000	LN355.000	LN355.000	LN355.000	LN355.000	LN355.001	LN355.000	LN355.000	LN355.000
42795.24	37415.71	42700.76	17801.89	41665.62	45183.15	31214.47	42079.73	36463.29	36571.79
38975.21	36786.11	49943.67	15882.84	42813.27	57168.79	25827.64	50813.58	49055.11	33543.94
34416.36	34917.61	36101.51	13102.69	36187.21	55991.08	25409.61	41939.22	45286.97	40069.1
44920.63	40347.87	47473.41	14849.06	44280.93	58316.34	25112.81	55918.84	48163.97	32294.02
34162.19	36063.01	38326.6	11599.74	45607.69	50514.33	19610.62	36040.77	37075.63	31318.36

LN355.000	LN355.000	LN355.000	LN355.000	LN355.000	LN355.000	LN355.000	LN355.000	LN355.000	LN355.000
30456.42	69593.47	26340.39	41315.37	57556.15	51823.91	25851.13	41438.63	31644.34	57913.39
39427.94	88049.88	26687.2	43525.48	64346.48	74132.63	30625.58	36825.66	33022.41	60966
39227.16	73604.34	42594.98	40277.01	50524.97	57540.68	37428.55	35286.12	28271.82	41382.01
36965.16	77508.47	29035.88	41931.57	62756.26	72047.37	30717.43	35245.94	31543.25	56313.38
51502.35	69882.94	26318.13	47423.86	45102.56	52463.47	26914.77	30312.15	38553.01	32214.68

LN355.001	LN355.000	LN355.000	LN355.000	LN355.000	LN355.000	LN355.000	LN355.000	LN355.001	LN355.000
30131.39	60866.57	51457.23	67059.69	49048.1	39205.78	41423.63	63065.9	24924.41	45948.89
35036.76	45967.21	53775.76	57873.06	53444.81	44116.22	48004.25	59472.75	18967.39	29724.9
38037.16	39332.52	32365.15	44542.41	28774.09	42494.49	34685.85	50110.62	30194.69	30392.35
37415.36	42422.92	53293.76	59621.51	52337.52	42692.18	49262.01	64170.71	18881.96	35155.93
25701.32	43472.39	42090.6	50846.22	31662.16	36568.56	42441.25	66137.15	18113.32	31763.18

LN355.000	LN355.000	LN355.000	LN355.000	LN355.001	LN355_12.	LN355.001	LN355.000	LN355_12.	LN355.000
21627.75	19662.89	24870.1	36393.16	29556.82	54436.28	22128.83	29152.18	73037.52	77497.43
23248.48	31702.97	35455.58	38558.04	25060.66	59799.45	20795.47	37364.61	60118.72	66318.97
24567.76	32764.19	36210.78	37038.02	26556.52	59159.43	19984.45	26520.96	59932.8	70751.6
20822.19	29923.22	37149.75	41958.39	30131.76	56181.35	19449.85	36157.09	65046.19	67891.87
20436.38	22073.16	29826.4	29782.69	23425.38	55272.4	18872.29	39097.45	100080.5	71206.37

LN355.000	LN355_12.	LN355.001	LN355.000	LN355.000	LN355.000	LN355.000	LN355.000	LN355.000	LN355.000
46950.63	64490.01	21148.46	40903.75	40897.15	19387.41	36768.62	49619.08	40210.82	47568.97
54645.46	71878.37	18398.81	58430.13	49045.25	27101.34	31082.85	65138.54	29200.61	57765.09
64413.59	57594.87	17710.17	59220.82	32723.91	28993.67	28326.64	47465.7	28849.1	50472.13
55190.33	61152.91	19436.78	52514.14	54340.9	26072.93	33104.64	64882.57	30523.74	47371.61
59162.36	63792.38	21768.97	35354	46175.61	27767.21	30864.64	72915.37	25570.25	59797.5

LN355.000	LN355.001	LN355.000	LN355.001	LN355.000	LN355.001	LN355.001	LN355.000	LN355.000	LN355.000
37068.37	17123.33	42067.87	18503.67	27793.04	17864.71	15156.97	49981.81	38139.89	30383.45
37572.45	19968.06	38144.9	17219.82	22055.02	22022.53	15333.99	55971.07	45856.31	32480.61
29514.74	16858.46	36491.09	18339.46	25823.77	17876.23	18674.45	56091.42	46499.14	36550.33
34705.27	16895.23	39802.79	17118.84	23104.11	19208.71	15180.98	54428.86	41135.32	31479.17
30307.14	14247.49	36349.94	14992.32	27544.11	15504.17	19714.48	58351	40718.64	38822.61

LN355.000	LN355.000	LN355.000	LN355.000	LN355.000	LN355.000	LN355.001	LN355.000	LN355.000	LN355.000
36520.41	41021.08	44676.17	27626.78	32681.82	42045.63	15756.74	32698.14	43500.01	33273.1
40686.03	52426.22	47534.4	28555.64	34092.46	32878.67	17382.18	33108.2	54766.77	27383.72
39669.64	43037.92	44907.9	23164.67	39388.72	34118.31	15974.22	24905.23	51552.46	26140.78
39638.12	48758.81	44799.06	30197.37	34176.32	36006.66	17443.46	31096.36	60835.5	30625.33
30662.76	46918.98	39278.53	26839.72	29437.09	26235.66	14037.79	30701.56	59080.96	30262.78

LN355.000	LN355.001	LN355.000	LN355.000	LN355.000	LN355.000	LN355.019	LN355.019	LN355.019	LN355.02_
28257.52	28462.6	45700.24	41736.11	32585.94	36021.94	60272.39	55139.46	63077.55	41433.05
28967.56	31171.84	39611.95	41082.57	33298.81	36087.41	63519.37	62095.55	60530.12	49169.08
33062.7	30020.58	37648.54	64079.96	25823.63	36985.56	62486.52	51096.64	67143.8	47339.67
27844.91	27656.25	35354.57	42482.56	35149.88	35694.76	63102.47	50392.88	67305.42	42904.8
23903.3	27408.19	26389.57	26874.1	33356.04	40297.57	61957.3	50714.94	65641.4	44199.82

LN355.019	LN355.019	LN355.020	LN355.019	LN355.02_	LN355.02_	LN355.019	LN355.020	LN355.020	LN355.019
43786.04	32399.47	22784.29	28857.45	72193.11	39976.13	61875.4	64432.03	36298.87	38492.23
40430.88	23659.03	27399.65	25632.55	75172.92	40948.44	48532.1	63981.38	42304.13	43364.21
40840.11	32139.48	22422.61	30468.51	70386.65	44051.79	65447.82	65521.97	35788.56	38119.07
39579.39	34066.56	22161.25	26755.3	73204.89	42692.84	64575.69	67730.52	35719.87	43515.19
39369.28	33990.03	21580.6	27115.37	77439.47	38066.96	62618.78	64711.34	34664.7	36605.62

LN355.02_	LN355.020	LN355.019	LN355.020	LN355.019	LN355.019	LN355.020	LN355.019	LN355.019	LN355.019
30638.16	23772.36	32680.67	38227.63	60878.26	59242.17	42950.57	88406.11	58069.09	30241.27
23320.21	20022.68	33568.57	37587.59	58712.67	61494.41	38904.25	108847.9	60583.97	27560.75
32188.6	20362.23	35525	41123.41	64547.27	58792.42	46919.85	90119.75	59521.69	33305.57
34265.13	20793.83	36842.3	40067.35	67772.95	57750.34	44102.87	97034.81	57962.43	30333
30961.08	20782.22	35538.92	41897.62	69690.1	52764.73	42222.49	89141.49	52368.5	31539.55

LN355.019	LN355.019	LN355.020	LN355.020	LN355.019	LN355.019	LN355.020	LN355.020	LN355.019	LN355.020
85686.2	29161.68	20680.56	31931.62	55741.95	80981.86	59402.04	22633.57	49905.35	17105.59
82908.69	29411.28	14624.07	30498.09	52172.84	101167	65452.22	21149.24	57161.17	16827.46
86537.42	28877.06	17082.23	31810.15	57840.4	83160.03	56669.4	22477.53	61221.92	14878.16
85486.13	28669.92	18598.26	31148.87	59168.52	81005.03	59604.66	22873.96	50837.58	14640.34
81000.22	31753.22	16650.72	31880.79	59498.86	79352.99	54768.66	23488.71	55557.33	17405.85

LN355.020	LN355.019	LN355.020	LN355.020	LN355.019	LN355.019	LN355.019	LN355.019	LN355.020	LN355.020
46965.52	40289.99	22200.01	31508.79	51898.14	36426.33	60165.7	43341.98	32463.84	26402.75
33940.22	35901.13	26155.18	26295.15	58812.49	29250.73	62784.12	54020.9	36620.65	20176.14
49430.59	41544.34	25051.74	34095.15	61780.12	36389.92	61150.91	46425.36	33347.47	28822.8
43359.47	40089.59	23556.13	31687.93	56894.07	36740.21	64818.28	40377.67	34146.55	27085.94
49243.48	39128.8	22527.26	30160.17	59786.45	39631.11	59010.73	44342.85	34226.64	29239.63

LN355.020	LN355.019	LN355.019	LN355.019	LN355.019	LN355.02_	LN355.02_	LN355.019	LN355.019	LN355.019
18370.88	51558.27	63255.26	55831.87	61437.59	41567.12	25407.59	25551.76	25938.53	35786.84
20026.02	38771.77	49468.1	66501.11	62201.33	45029.7	25033.01	24338.32	19810.58	32160.77
17550.56	53646.08	70103.05	58202.13	62874.27	37971.08	26395.68	25205.57	27095.41	40688.41
16999.83	56301	67857.82	57601.21	62225.19	40012.57	26300.62	21992.23	23937.45	38632.11
18380.79	54288.47	65609.72	57339.06	63683.63	39233.04	25092.5	25329.87	25748.77	35548.13

LN355.019	LN355.019	LN355.019	LN355.019	LN355.019	LN355.019	LN355.019	LN355.02_	LN355.019	LN355.019
26508.89	31816.25	33581.19	39292.84	30594.97	40466.27	48682.82	32741.15	38741.23	43883.77
26542.97	33560.8	33090.13	35501.3	34250.62	37308.94	43606.99	30754.19	32384.72	47927.13
24038.93	28300.92	38409.91	38832.31	32728.39	39480.62	44994.61	33944.87	35113.4	46496.77
24943.37	30379.46	33971.64	38414.55	32737.25	40516.88	44525.96	34445.09	36779.72	47141.23
26667.71	32129.68	33622.2	38612.93	32223.09	40145.51	46097.23	31563.57	33788.14	45341.7

LN355.019	LN355.019	LN355.019	LN355.020	LN355.019	LN355.020	LN355.020	LN355.019	LN355.019	LN355.019
26362.24	39322.49	42345.3	21775.25	18631.71	42126.61	20150.66	46859.48	38684.38	40844.97
26435.28	37849.51	47622.64	22685.02	13554.47	39926.89	18404.71	49735.31	37249.76	42990.87
26097.54	43097.81	44735.19	21238.59	16146.68	39090.59	21613.35	48662.58	38895.69	40206.69
28319.07	40357.6	44828.13	24645.03	17717.99	45285.75	23909.11	48545.76	37720.42	41857.38
23934.97	41147.08	42289.28	22097.45	16922.94	39984.72	21468.59	48258.35	38880.97	42188.67

LN355.019	LN355.019	LN355.02_	LN355.019	LN355.848	LN356.997	LN356.997	LN356.997	LN356.997	LN356.997
34319.72	41110.17	18478.27	38304.36	63716.95	80510.86	61677.75	60047.64	32104.8	29354.84
35914.8	43287.78	14743.88	39565.05	53030.27	76755.56	63425.82	57521.65	32053.99	27722.19
35143.72	43179.27	16159.58	42601.18	64682.92	57062.82	62709.32	92645.43	28596.86	27654.48
33120.59	39777.05	15267.69	41936.79	60034.39	68236.67	66995.8	58916.39	33665.67	27850.79
35262.88	42204.57	15550.28	41686.35	65164.15	74863.56	58408.39	69502.16	35931.1	28942.45

LN356.997	LN356.997	LN356.998	LN356.997	LN356.997	LN356.998	LN356.998	LN356.998	LN356.998	LN356.998
21382.6	22616.98	15911.7	49000.57	38576.12	57457.93	47915.79	33723.28	35348.47	52141.48
21616.64	23212.28	12387.09	45074.72	93297.35	59089.59	41528.15	34665.31	37977.84	56868.78
23514.85	22187.55	12650.56	60410.93	74539.83	50846.09	42222.91	42328.39	31084.54	67107.31
20563.05	23766.85	15261.98	54540.96	40140.26	53092.02	46015.85	32111.22	33557.23	47687.37
21169.45	22303.85	14572.56	52745	46776.38	52972.75	47945.3	33168.86	29628.42	44895.5

LN356.998	LN356.998	LN356.998	LN356.997	LN356.998	LN356.998	LN356.998	LN356.997	LN356.998	LN356.997
39179.08	45444.47	32543.95	52076.49	35191.9	30147.79	22008.39	48039.38	27484.23	47097.63
46795.1	50403.45	23223.93	58034.88	31912.12	27027.69	25332.23	69075.59	32771.37	68333.3
42101.04	49740.79	38997.56	70698.67	35042.85	36529.79	21199.64	38481.68	38553.36	51391.3
39678.49	45269.71	33329.62	53133.15	29007.28	29676.15	21478.36	49805.95	27117.69	50333.58
43330.64	43805.98	34133.78	50776.49	31137.64	28440.92	19172.42	57840.21	25458.19	48314.82

LN356.997	LN356.997	LN356.998	LN356.997	LN356.998	LN356.998	LN356.997	LN356.998	LN356.997	LN356.998
45750.9	43340.24	42729.68	67501.21	48049.57	55914.55	51773.94	39426.26	52223.74	24755.24
81494.45	44111.91	51719.8	72700.55	37564.45	49682.31	47749.01	47682.67	58522.23	42121.35
58356.49	51214.95	58225.71	55625.84	35284.4	44027.24	72898.61	38735.17	63062.33	32330.28
42198.51	44291.23	44028.84	57310.44	40894.7	53331.02	56271.39	39000.85	58006.05	28145.14
42431.23	46010.17	48332.76	60155.18	42804.65	56397.71	63593.09	44322.27	54331.21	28729.29

LN356.997	LN356.998	LN356.997	LN356.998	LN356.998	LN356.998	LN356.998	LN356.998	LN356.997	LN356.998
53291.93	43993.05	44516.55	37380.49	18029.41	35154.88	39959.98	17612.47	77525.88	42525.4
53622.34	56131.53	52597.46	32920.8	22625.31	30919.22	42432.39	28906.43	88564.71	43121.14
62913.04	61033.28	64587.83	46326.05	30614.99	36601.97	56088.9	22404.76	88547.02	56615.38
49496.45	40151.41	47110.53	36365.3	17774.16	35616.99	39073.07	19657.04	78846.37	43542.33
46541.21	42109.55	40616.57	39518.94	16828.17	39617.55	39222.22	17238.47	88816.76	42293.06

LN356.998	LN356.997	LN356.998	LN356.998	LN356.998	LN356.997	LN356.998	LN356.997	LN356.998	LN356.997
55332.89	60227.66	27251.9	40685.88	40283.83	57404.26	32621.68	59178.68	28041.26	57159.08
51038.95	66572.46	32971.22	41207.88	40501.14	92183.18	36215.2	69477.3	31378.14	61934.31
69535.18	63362.13	28158.78	34478.41	40102.84	53870.86	44562.23	83293.44	30258.15	49018.63
54864.68	66220.26	29539.42	38726.57	43655.85	61235.03	32844.2	71466.93	25817.39	58241.48
47748.61	62496.08	26365.85	42782.76	41451.46	62987.09	35397.09	66572.11	30961.76	57342.96

LN356.998	LN356.998	LN356.998	LN356.998	LN356.998	LN356.998	LN356.997	LN356.998	LN356.998	LN356.998
39091.31	57181.35	33872.27	30623.41	38668.69	33513.09	62735.11	40429.62	48796.19	22723.2
46056.2	58416.68	40186.42	27747.62	41173.34	36172.38	58836.98	51005.28	52807.11	38080.71
53564.5	65218.8	51054.2	37678.72	51534.19	36718.77	65835.16	37793.54	47484.88	28914.69
47742.71	59529.28	35580.56	27189.69	37938.79	27407.3	64438.48	46492.79	50796.67	21787.72
45329.6	52775.59	35612.58	29551.93	36244.93	29069.15	65484.09	45656.36	54006.66	22002.28

LN356.998	LN356.997	LN356.998	LN356.998	LN356.998	LN356.998	LN356.998	LN356.998	LN356.998	LN356.998
35419.42	54184.65	31554.01	31058.86	37700.74	43801.96	19297.18	32381.1	33770.53	38194.74
25970.3	61017.55	27506.18	45827.07	46729.54	37793.07	17942.68	33699.91	24918.01	37283.09
32859.38	62726.31	42260.69	35252.16	26777.33	41782.05	18680.97	31644.01	33650.16	36248.01
30129.62	55305.16	29994.01	34660.47	33397.81	42703.22	20312.99	33056.45	33963.28	36291.91
35124.88	54794.16	29384.54	32321.49	31626.45	41308.73	19589.33	31251.61	27209.16	35045.2

LN356.998	LN356.998	LN356.998	LN356.997	LN356.998	LN356.998	LN356.998	LN356.998	LN356.998	LN356.998
53785.8	43777.06	24443	52612.72	19859.49	36175.89	26217.1	25776.93	20826.8	32422.83
65151.04	48570.29	23589.71	38603.6	22247.39	54790.19	32977.85	24157.96	18677.55	33540.71
51616.91	35904.53	20740.25	53032.84	21685.3	33656.25	29949.13	19712.18	23591.5	28681.24
52780.72	43028.05	21178.34	46881.48	19746.38	36044.69	30527.51	24353.92	24270.12	30228.08
50909.56	45063.26	18361.19	47726.79	21160.91	35136.14	29989.6	21029.34	20316.39	33052.1

LN356.998	LN356.998	LN356.998	LN356.998	LN357.015	LN357.191	LN357.191	LN357.191	LN357.191	LN357.191
39653.2	17427.04	77597.35	94553.68	72794.92	17289.63	20692.74	29177.47	19410.96	34213.06
37477.34	18853.07	84321.53	85167.58	23763.73	23716.39	20415.24	27941.6	20035.36	32502.05
33885.75	18252.97	82714.77	94878.73	73571.86	17929.81	18913.45	27797.38	20592.12	31744.53
36788.93	15995.4	75803.01	87357.35	70516.13	22335.66	21422.42	27238.95	18725.42	34052.99
36311.99	18372.08	85181.42	98891.63	75188	21181.31	21367.25	26191.26	19663.59	28720.19

LN357.844	LN358.843	LN358.994	LN358.994	LN358.994	LN358.994	LN358.994	LN358.994	LN358.994	LN358.994
17704.74	41556.8	96060.96	72905.75	54700.25	98950.33	85241.77	115175.6	127707	80602.72
14393.37	36155.75	78476.83	60561.76	66788.14	109087	86407.97	90209.78	152196.8	68233.57
15077.51	40613.82	133784.8	79347.7	68334.44	100929.9	76969.15	71767.13	94297.5	87126.35
21951.04	28206.66	86809.48	59475.08	85289.14	107055.6	111818.3	121985.3	118088.8	64041.98
21636.17	58490.44	110942.3	74682.45	44062.43	113279.2	79506.47	83758.67	135001.7	64101.67

LN358.994	LN358.994	LN358.994	LN358.994	LN358.994	LN358.994	LN358.994	LN358.994	LN358.994	LN358.994
74589.7	97542.1	62341.31	63432.44	122158.9	68218.39	77743.42	58628.57	102349.7	54775.21
86706.13	83464.73	43691.92	67355.67	90774.5	90990.66	119264.1	100785.8	75813.32	60773.86
126894.6	112410.7	54966.43	70474.59	79237.22	78584.22	61506.94	75816.16	109354.5	60268.5
75170.67	72061.87	53641.55	61080.01	104433.8	62589.52	88672.84	65464.6	104021.5	46597.57
131901.5	97644.6	80968.05	69981.58	107368	68192.37	114209.6	92292.42	111191.4	76600.85

LN358.994	LN358.995	LN358.994	LN358.994	LN358.994	LN358.994	LN358.994	LN358.994	LN358.994	LN358.994
76960.29	56880.3	82426.36	126845.9	66161.76	68472.89	26831.04	90215.8	82997.91	102735.3
50046.11	55277.12	63607.4	138687.1	67906.68	75806.67	26193.36	99658.92	89990.42	84110.88
61140.07	61804.65	62506.12	118996.4	61754.07	112867.9	16371.77	56193.93	77876.1	162405.3
104124	39153.62	81723.96	125855	64534.85	72518.46	20754.52	113063.5	132312.5	122262.8
73404.87	48817.99	64391.71	97157.15	92550.08	69039.62	39754.68	79035.13	90499.79	131563.8

LN358.994	LN358.994	LN358.994	LN358.994	LN358.994	LN358.994	LN358.994	LN358.994	LN358.994	LN358.995
153368.1	101332	74645.02	110955.2	80973.67	111034.7	79409.62	90692.16	71730.73	27408.51
96296.1	116456.2	54543.83	175856.6	67010.02	133531	86501.63	98864.86	58970.61	39989.6
96767.84	93977.19	51167.86	112033.4	61413.16	79151.91	75575.52	92737.98	62871.79	28363.06
127113.6	93791.7	79914.58	109367.5	57772.3	121939.3	72023.03	123752.7	86485.23	37733.52
100880.8	84058.04	57636.56	139669.2	83973.56	99706.32	108356.6	91806.59	60830.11	45030.76

LN358.994	LN358.994	LN358.994	LN358.994	LN358.994	LN358.994	LN358.994	LN358.994	LN358.994	LN358.994
105907.1	52953.87	95793.56	54243.81	130020.9	64792.27	84324.79	79233.89	104104	64597.63
82075.18	48244.29	86619.5	52787.7	105042.7	62882.99	94934.79	80953.09	88983.37	67640.08
134926.4	48372.75	110807.9	74731.15	76212.8	66897.9	83794.18	94087.12	168833.7	73743.27
101412	57407.86	113528.9	46903.87	115559.8	90436.99	116100.2	90848.98	137714.4	70942.71
101092.5	43080.72	71897.65	59940.01	111748.6	76382.89	87618.55	83366.37	81695.26	77538.91

LN358.994	LN358.994	LN358.994	LN358.994	LN358.995	LN358.994	LN358.994	LN358.994	LN358.995	LN358.995
156820.6	140726.5	115539.9	83790.77	33745.1	86183.16	74934.62	90229.62	35347.05	34513.79
99550.88	106098.2	155159.8	56336.52	34958.57	99423.27	98646.66	76964.82	38790	43820.07
123909.5	141937.3	150863.8	49380.07	28597.23	64428.64	78314.63	95404.08	52418.69	31048.84
110538.8	138733.4	119400.6	46578.14	36972.99	68839.45	79179.08	54129.61	35339.33	31083.84
116458.6	258006.8	140020.8	58764.44	32960.29	74330.99	115940.4	55845.51	39734.02	41535.54

LN358.994	LN358.994	LN358.994	LN358.994	LN358.994	LN358.995	LN358.994	LN358.994	LN358.995	LN358.994
127393.9	135139.8	64063.46	148516.3	100177.7	34083.83	153092	88483.37	44891.76	37581.98
87934.45	97397.58	99083.2	118668.9	110931.6	28711.73	164166.5	120546.5	36689.74	36238.36
111169	118520.5	97446.12	126033.5	123818.4	50173.17	164763.9	111392.2	29661.16	39918.65
86753.51	100245.2	88032.9	171922.6	139126	38539.1	162107.7	132159.3	28802.99	34999.8
108713	98540.37	90118.77	137093.7	100464.1	38306.03	182697.5	104398.4	36954.39	37176.86

LN358.994	LN358.995	LN358.995	LN358.994	LN358.994	LN358.995	LN358.994	LN358.995	LN358.994	LN358.994
74352.73	41798.28	33552.94	151783.6	138764.3	31623.61	62676.84	28512.36	51339.85	194509.6
62909.52	33380.92	42185.04	183119.9	105980.2	33606.57	54750.96	29881.98	60015.43	144153.6
85391.67	43600.62	34337.36	152020.3	81026.84	25325.79	68323.89	33207.6	57665.56	166437.5
69398.79	35528.48	30964.53	140986.4	123847.4	36651.17	57724.72	36992.62	53817.02	165470.4
66018.22	33370.46	32650.21	124561.1	99494.58	40104.58	57260.88	30304.33	68144.49	166001.4

LN358.994	LN358.994	LN358.994	LN358.994	LN358.994	LN359.154	LN359.152	LN359.974	LN362.875	LN362.969
76857.43	133559.8	73021.01	92364.58	51976.42	12485.52	13892.04	16816.63	188119.9	42981.68
103254.8	177542.1	52131.06	81989.01	79887.96	14191.5	12681.09	12722.74	226812.7	43456.72
78866	129525.7	62197.87	115666.8	86679.77	13411.89	15027.79	14221.33	154305.4	44962.64
89124.55	146736.2	55479.41	87587.81	55281.66	12958.13	13200.44	16682.84	147166.5	38579.17
120146.7	120943.7	54470.89	86244.55	61013.4	12027.96	15363.71	19246.38	175787.5	43292.65

LN362.969	LN362.97_	LN362.969	LN362.969	LN363.875	LN364.489	LN365.022	LN365.246	LN365.884	LN366.869
48356.19	38439.57	39716.13	45947.77	28686.34	67855.1	46664.76	16163.91	36747.36	11454.15
48147.7	37273.11	40930.16	45213.82	28643.36	76443.65	47074.31	14138.31	31316.33	12322.22
43518.81	38420.54	41969.89	44697.4	26748.36	72371.22	46957.74	17438.4	30280.28	13373.25
49427.76	38018.29	34809.63	45753.9	27023.81	62794.44	52360.65	18736.36	31624.7	12987.3
46006.47	37110.24	45025.22	47163.11	37816.17	66691.52	50191.82	18181.54	32736.88	15044.96

LN367.019	LN367.019	LN367.019	LN367.019	LN367.019	LN367.019	LN367.019	LN367.019	LN367.019	LN367.019
41265.6	25932.83	42570.2	25939.02	59909.98	48217.75	32573.29	45218.17	26078.27	55930.63
42415.71	24997.88	44851.96	27566.35	52587.88	50519.95	32019.23	47014.97	28404.63	55089.21
45925.93	27511.28	40364.62	24302.67	56668.01	48789.98	30440.45	44534.65	25609.14	56938.81
44296.22	26291.91	43101.54	25927.6	57662.76	53024.08	31978.19	47477.99	23742.56	59409.53
40306.24	27324	44406.65	28216.34	56553.86	51801.56	31151.46	46357.25	24994.98	55744.64

LN367.02_	LN367.019	LN367.243	LN367.357	LN368.241	LN368.889	LN368.997	LN368.997	LN368.997	LN368.997
24972.36	40832.44	73900.06	16225.69	29531.6	52859.24	38908.72	26505.33	51893.75	31575.61
25606.29	41781.55	68579.66	13772.96	25785.85	51743.03	36522.79	26028.14	52754.26	33216.42
24868.59	39980.54	75672.93	16051.29	22102.81	57217.39	33468.17	26433.64	52414.69	31890.51
28696.77	42585.21	75638.94	15354.98	26868.92	53417.64	37252.33	26000.48	47184.32	31267.14
23942.8	40502.66	81917.76	12915.5	22293.8	48891.15	36244.85	27266.91	45127.87	33296.46

LN368.997	LN368.998	LN368.998	LN368.998	LN368.998	LN368.999	LN368.998	LN368.998	LN368.998	LN368.998
22575.08	50534.49	16991.08	44639.65	55308.52	17291.65	26394.72	38365.81	56394.24	13577.06
24454.09	46420.06	15300.74	44537.04	51792.2	17189.84	25793.58	39086.02	54215.93	13867.53
24396.07	49794.97	14794.27	39957.2	50066.99	17196.23	27062.8	34600.98	57963.56	14769.47
21375.59	48289.31	13287.75	40937.24	53969.95	18309.6	28620.03	37562.48	57684.07	15345.11
24157.89	50828.7	13121.54	44012.21	53714.2	15636.66	29238.28	35461.52	55263.14	10915.82

LN368.998	LN368.998	LN368.998	LN368.998	LN368.998	LN368.998	LN368.998	LN368.998	LN368.998	LN368.998
26361.17	35153.88	43220.41	42531.55	25712.29	22951.62	35737.98	28021.65	19222.58	37578.93
26351.67	32635.85	44841.46	41073.53	29331.66	25237.86	33971.23	28657.04	16803.67	35766.48
29536.7	30856.24	48544.31	42806.87	28203.02	23960.6	33619.94	29232.07	15008.01	32608.85
28184.07	30710.88	44015.01	39027.36	27213.05	25977.28	34379.88	28898.39	17466.91	35122.93
28010.59	33318.41	46970.23	40808.45	25448.75	26800.56	35229.39	35049.52	17443.74	33276.8

LN368.998	LN368.998	LN368.998	LN368.998	LN368.998	LN368.998	LN368.998	LN368.998	LN368.998	LN368.998
20277.44	45650.37	28278.6	66133.36	45233.26	28139.69	58619.79	26356.68	27764.23	43654.43
17089.06	46047.68	30140.38	68227.57	48330.85	32479.55	55342.47	26116.82	28484.36	44720.07
18812.93	43255.44	28000.23	65183.72	49063.87	29906.29	57170.22	25902.48	28866.89	42701.8
18801.89	41579.63	31868.01	61950.79	49147.39	28028.5	47912.36	28875.8	27964.82	46304.51
19006.59	39620.01	29728.02	65597.62	44005.11	28516.88	53598.5	28003.05	26980.35	43591.5

LN368.998	LN368.998	LN368.998	LN368.998	LN368.998	LN368.998	LN368.998	LN368.998	LN368.998	LN368.998
40785.35	45605.82	32161.1	33217.83	43583.74	39928.59	49061.88	12967.13	23673.33	51642.64
48123.11	43792.25	32182.36	31949.98	38479.56	40461.29	46138.88	13490.78	23678.96	49221.7
43008.71	44791.64	33317.66	33867.47	42347.43	38586.82	50499.94	12784.99	25117.57	48243.07
47189.12	46049.49	32198.98	32613.28	41544.6	39832.15	50240.36	11614.65	24121.95	49483.65
39675.33	45245.17	31848.88	31876.56	37845.33	35153.91	50987.4	13405.68	23967.6	43125.01

LN368.998	LN368.998	LN368.998	LN368.998	LN368.998	LN368.998	LN368.998	LN368.998	LN368.998	LN368.998
30428.69	37885.26	38161.98	44045.61	41852.16	42589.37	56572.41	38667.55	17610.33	37827.06
29112.49	38030.46	40003.22	41487.67	38997.24	43130.98	54630.55	35802.67	20299.13	38037.89
28456.49	37703.01	39177.45	41576.56	34648.83	42822.37	61252.66	41609.84	19510.47	35121.9
26136.2	35910.76	41789.9	44189.7	36990.7	44389.31	54627.03	39271.2	18544.67	40988.42
29046.44	40506.8	41033	44387	39685.25	45603.72	61227.83	36458.54	19571.04	36023.58

LN368.998	LN368.998	LN368.998	LN368.998	LN368.998	LN368.998	LN368.999	LN368.998	LN368.998	LN368.998
34132.38	32839.51	66779.98	38753.39	24361.44	37388.2	19232.38	34975.42	47465.83	26505.51
32583.88	31260.72	64241.69	37141.72	25283.56	37844.71	15016.21	32547.71	51054.91	27120.18
38627.17	33904.97	67636.3	35524.56	22699.35	40099.23	16969.62	34656.26	47124.08	28958.62
36963	30560.83	68379.8	34436.17	22837.71	39134.51	18076.1	35391.38	51322.68	27689.95
35834.37	29114.48	68150.29	35881.04	22989.1	36462.05	15853.9	33146.56	46188	23952

LN368.998	LN368.998	LN368.998	LN368.998	LN368.998	LN368.999	LN368.998	LN368.998	LN368.998	LN368.999
42195.85	49586.82	20951.13	31017.76	38294.02	30764.95	16403.89	51220.05	27605.96	19348.56
42060.05	46959.2	21020.94	33719.13	38303.88	34696.69	17066.49	48599.16	27627.16	19417.61
38703.78	47397.86	22504.65	33757.01	39870.4	31313.92	17991.37	45522.59	26879.83	18806.58
39700.85	49281.45	23170.56	32158.26	38155.15	32687.15	16547.96	51298.62	29791.1	19471.33
38878.14	46498.22	22028.49	31103.99	39737.66	30688.47	16946.83	43439.72	29378.83	20396.67

LN368.998	LN368.998	LN368.998	LN368.998	LN368.998	LN368.998	LN368.998	LN368.998	LN368.998	LN368.998
21591.86	22960.18	43019.33	37876.99	53568.76	27742.84	27599.18	37620.14	16981.47	62833.66
22362.42	21584.44	42351.57	37527.33	49755.04	27265.12	28543.69	38053.91	19082.29	65720.96
18993.07	22542.33	39381.85	37800.52	49125.16	24850.13	26689.21	37564.2	16948.93	60169.84
19559.65	21157.56	40973.47	45118.81	52469.36	20935.39	29571.1	38750.16	15745.96	63126.26
18645.74	22051.5	38665.21	38437.91	52249.24	24179.86	25464.09	40552.15	16138.51	62092.62

LN368.998	LN368.998	LN368.998	LN368.998	LN368.998	LN368.998	LN369.016	LN369.016	LN369.016	LN369.016
29590.47	59065.65	24861.24	30454.95	65560.95	48640.3	37959.82	37015.24	58173.13	44123.28
28959.51	58297.53	28911.5	29172.18	62077.56	46878.82	38721.96	34971.02	58940.44	44665.52
30439.29	63863.9	27686.15	29609	60730.36	44737.2	38600.68	39898.86	58182.84	43859.36
28697.35	57546.83	25235.43	27292.44	61371.74	41496.59	39694.09	38110.88	60737.55	48319.87
30296.34	60095.59	27363.54	30403.36	64749.92	41859.19	41178.97	38904.44	60128.34	47386.5

LN369.213	LN370.002	LN370.838	LN370.869	LN370.868	LN370.994	LN370.994	LN370.994	LN370.994	LN370.995
32863.09	14899.45	38447.73	18980.34	14769.97	46634.98	57502.93	61021.45	35342.03	16420.27
33061.42	18358.74	21201.88	20477.54	13102.42	37675.81	50199.41	64018.01	33022.53	17821.51
33591.41	20233	25996.64	21887.17	12865.14	44552.8	53112.13	51539.84	32151.67	15066.88
30474.16	22479.76	36693.29	20547.03	15356.98	43841.96	53928.57	62910.91	32084.68	11304.67
27042.42	26068.49	43020.4	18701.19	13668.69	44169.89	52953.02	61513.62	29418.78	9270.321

LN370.994	LN370.994	LN370.995	LN370.994	LN370.995	LN370.994	LN370.994	LN370.994	LN370.994	LN370.994
52848.33	49957.06	25908.41	34470.24	24208.92	83352.02	79164.29	60128.36	48347.61	22058.1
43677.76	48579.58	25443.35	32031.96	26707.67	67945.47	51800.51	59193.46	42840.79	25591.13
56967.8	46372.75	22418.25	42108.86	22836.28	74699.9	43983.36	72655.37	51547.11	29179.58
55625.89	52641.79	29596.91	53328.5	24931.05	82682.51	58669.93	82554.81	55191.88	27956.76
62792.41	32472.92	22821.19	34542.45	29391.99	65715.53	77310.57	82017.99	63218.95	31215.92

LN370.994	LN370.995	LN370.995	LN370.994	LN370.994	LN370.995	LN370.994	LN370.995	LN370.994	LN370.995
64109.44	32622.82	22719.76	45386.08	52663.11	30356.43	59422.97	18319.92	35757.62	16652.36
37938.25	29100.93	27437.71	31876.85	45775.14	24860.39	62067.37	17784.64	34742.04	25609.81
37663	28896.95	22226.26	38477.36	44475.98	34212.39	46499.05	16077.12	36166	18532.29
40623.66	31358.07	24557.53	34052.55	59315.25	39172.75	83016.74	22347.96	40749.76	16712.46
38171.51	27508.43	24280.34	46982.33	50067.06	42171.43	48627.4	18611.05	33186.59	16593.64

LN370.995	LN370.994	LN370.994	LN370.994	LN370.994	LN370.994	LN370.994	LN370.994	LN370.994	LN370.995
38105.48	59442.64	51144.43	56469.43	74560.43	37063.58	37423.78	29537.98	35564.76	23056.2
30983.7	67063.65	53993.87	46555.83	58216.88	29917.93	39198.65	35932.04	41867.22	20849.88
27752.22	83762.27	46843.18	54734.28	55515.63	35203.18	41717.49	25910.83	39646.52	19033.41
40998.14	102161.4	65825.84	57658.74	76476.07	41153.63	40832.29	37606.72	55251.64	18075.35
26575.74	120789.5	52220.78	63450.52	72701	36916.31	43240.31	29519.99	41656.29	15274

LN370.994	LN370.994	LN370.994	LN370.994	LN370.994	LN370.994	LN370.994	LN370.994	LN370.994	LN370.994
37665.32	56876.9	40559.54	37100.62	43216.34	51741.31	24697.87	48161.36	35260.51	37171.31
59154.16	40679.13	36167.04	34303.64	37061.04	58083.26	46398.03	30590.04	35005.93	50769.58
38368.6	43939.74	40944.3	38519.22	45934.2	51642.15	30482.96	32776.85	29939.64	33455.04
35146.46	59052.96	39267.37	36272.24	67992.53	57436.99	28303.69	31551.85	34269.33	47449.76
32452.38	40559.71	45760.54	46474.51	36591.07	44207.04	30337.47	36840.36	44030.31	27910.62

LN370.995	LN370.995	LN370.994	LN370.994	LN370.994	LN370.994	LN370.994	LN370.994	LN370.994	LN370.994
18506.61	20750.16	43237	36234.84	27251.89	50508.21	63919.15	41590.04	69098.54	55485.13
15297.79	20746.08	46234.02	44627.68	29669.18	47961.47	80253.35	50252.29	66042.28	54505.26
20795.65	13686.77	40688.32	43033.07	29700.28	49048.69	76635.87	40993.66	67229.28	52810.59
14592.87	15240.35	45566.92	39683.12	30983.32	47642.07	52732.55	41926.85	89837.47	64711.71
15746.68	14375.43	42519.2	40927.25	38408.73	46223.28	96970.69	43064.06	66084.93	41961.7

LN370.994	LN370.995	LN370.994	LN370.994	LN370.995	LN370.994	LN370.994	LN370.995	LN370.995	LN370.995
72066.75	32567.8	48864.67	67675.96	37954.17	45161.32	43879.4	25443.54	16523.95	29806.27
54073.92	30729.54	64703.64	56231.18	34899.58	30875.3	78908.39	24996.93	14521.04	40372.22
66666.92	28379.82	49033.13	60986.78	31056.91	36239	61424.77	20954.19	19081	40176.3
59244.75	30712.91	51705.59	58156.91	34077.85	31101.99	76583.3	19346.89	27661.28	46516.28
80232.68	30885.43	57198.48	56811.98	33018.25	37952.29	52149.19	21754.68	19119.88	29158.66

LN370.995	LN370.994	LN370.994	LN370.995	LN370.994	LN370.994	LN370.995	LN370.994	LN370.994	LN370.994
30354.13	42519.2	68781.31	39010.35	64990.6	37182.76	27000.64	50670.47	32124.14	29715
27343.68	60027.39	95044.55	55108.94	45798.36	38285.89	36044.5	41246.68	37016.24	37789.86
34569.64	40515.3	68797.41	40875.78	34299.12	36604.67	29378.45	43965.22	32787.02	30609.2
32793.04	38330.63	88863.29	38490.91	50555.98	39918.89	38397.13	33690.04	35512.39	32117.62
50926.73	67943.79	74865.66	37920.88	40430.99	31878.14	26633.05	38700.15	43473.09	29099.24

LN370.994	LN370.994	LN370.994	LN370.994	LN370.995	LN371.848	LN371.900	LN371.901	LN371.900	LN371.900
32923.33	63293.96	89933.88	63798.45	19815.08	40817.99	43114.88	27610.1	38261.13	15832.78
40094.13	61109.07	54799.69	49580.86	17387.34	49555.51	47022.05	32787.13	37303.94	16201.46
38332.13	56952.75	50192.14	51289.09	17834.47	56686.93	39321.48	30753.35	35149.93	17161.85
31273.91	77468.82	50211.98	42751.8	17243.78	32828.77	45648.43	29365.26	34735.67	15515.64
32194.73	54801.58	60047.7	44509.48	17906.2	42239.83	44672.44	30828.89	35481.4	13878.93

LN371.901	LN371.900	LN371.900	LN371.900	LN371.901	LN371.901	LN371.900	LN371.900	LN371.901	LN371.900
34654.33	32233.24	35647.85	45971.39	36766.03	44194.47	40596.66	39131.23	17428.78	29496.42
33965.95	34483.41	34005.45	46444.23	43213.88	47254.32	37553.3	41854.06	17557.79	36955.49
27180.4	32142.02	30648	47563.54	39950.76	43632.76	35999.37	37688.23	15616.99	35717.37
29674.26	32535.48	30501.67	45727.85	38590.71	41458.99	39413.57	41793.69	12824.4	30784.65
30006.77	33568.79	35572.28	45717.72	39702.41	43599.06	37220	32297.87	15013.11	31728.66

LN371.901	LN371.900	LN371.901	LN371.901	LN371.901	LN371.900	LN371.901	LN371.900	LN371.901	LN371.901
29745.72	29033.04	54200.6	39332.63	31033.92	57109.58	27095.98	32334.78	44193.31	35429.78
31031.47	33082.1	57540.65	44927.93	31387.73	60867.64	30598.86	32524.47	40275.39	37068.88
30480.52	25384.42	55715.33	40862.72	34821.29	59213.31	26041.47	31147.11	39361.71	34869.61
29452.55	25752.98	51952.62	42456.16	30454.16	54972.61	26880.35	28831.04	39133.7	39023.92
29336.52	29001.99	51809.59	39547.77	30936.7	56977.75	24749.38	28235.81	39333.53	37578.34

LN371.901	LN371.901	LN371.901	LN371.901	LN371.901	LN371.901	LN371.900	LN371.900	LN371.901	LN371.901
35564.37	41000.04	20271.8	26092.68	39016.5	27749.02	43067.14	40664.47	36973.21	20967.12
38473.17	44329.51	21946.52	26833.81	41686.24	31789.12	42027.97	43143.25	40459.34	21659.16
31617.64	43441.22	20727.83	24137.71	39646.22	29354.48	43243.75	39649.11	34389.4	21867.43
35254.11	45806.46	19698.11	22612.08	42445.12	28395.34	44930.7	41019.16	33132.73	21199.2
33187.06	38487.57	21873.43	23572.97	38802.84	25803.48	38321.37	39772.97	35366.38	23108.14

LN371.900	LN371.900	LN371.901	LN371.900	LN371.900	LN371.900	LN371.901	LN371.901	LN371.901	LN371.901
27215.27	58251.92	40677.62	37556.63	55299.71	35026.05	27799.09	28667.89	30846.61	37142.48
25309.68	57452.3	38901.69	39985.1	54881.01	38260.64	30397.77	26959.48	35472.65	41890.35
23268.73	58115.09	38805.08	38005.62	52324.13	38019.22	26943.24	24508	35132.25	39076.7
24359.42	56397.8	36926.42	39339.04	52274.33	36122.92	24924.85	21913.39	34348.97	39157.78
21848.13	58008.77	34715.48	40310.64	53999.15	38031.96	23992.68	26134.39	32148.03	40737.66

LN371.901	LN371.901	LN371.901	LN371.901	LN371.901	LN371.900	LN371.900	LN371.900	LN371.901	LN371.901
48914.04	36176.05	21474.57	27199.9	34530.28	38858.45	27611.2	43934.63	45612.63	23223.04
48444.92	39689.66	28723.64	27896.76	39418.59	41083.48	31927.62	43298.21	42843.52	25297.49
47713.27	36587.87	23931.61	29561.25	34364.53	35870.99	27341.95	44469.29	39180.65	23861.22
53544.88	33751.52	20638.08	23813.06	31962.68	36030.18	32120.25	41929.03	39312.67	20695.6
45808.24	36528.43	23405.93	28141.7	36224.52	36891.38	33505.55	39165.87	41939.96	23455.05

LN371.900	LN371.901	LN371.900	LN371.901	LN371.900	LN371.900	LN371.900	LN371.901	LN371.900	LN371.901
31728	49305.62	35466.67	32371.98	50474.93	21891.74	41586.91	42511.45	28348.67	39966.01
30366.91	48003.4	38171.74	37230.47	52577.62	24409.56	46306.67	44238.92	27663.3	43645.77
33313.17	50209.97	35709.74	35854.98	50320.7	22601.91	44239.51	42629.87	25194.22	37204.78
31592.82	48730	35545.99	38993.12	45853.12	23924.23	41230.78	41029.55	25964.39	42089.02
30785.1	47993	35248.2	33676.78	44662.25	22514.11	42702.5	43935.79	25165.92	36917.87

LN371.900	LN371.900	LN371.900	LN371.900	LN371.901	LN371.901	LN371.901	LN371.900	LN371.900	LN371.901
39348.03	18191.85	36930.23	48943.85	26190.41	30168.49	42008.38	18307.63	49129.33	44002.78
43076.53	18633.47	34544.94	50817.88	28456.55	31607.27	46013.64	22858.47	49818.19	49405.29
38430.96	17372.42	36046.05	47339.62	26410.23	33187.08	46782.78	20851.18	42715.97	46629.27
41654.11	18211.52	34736.18	49856.29	26195.01	29956.28	42860.18	18465.68	43751.94	47234.34
42297.42	14050.46	39550.8	48232.02	24860.09	27223.19	45657.75	20477.92	47384.76	45738.69

LN371.901	LN371.901	LN371.900	LN371.901	LN371.901	LN371.900	LN371.901	LN371.900	LN371.901	LN371.901
28715.84	31083.29	36567.29	29413.22	52772.83	52097.75	25345.94	24713.97	27514.48	46474.13
30643.01	35440.24	37993.73	35115.55	53263.16	52062.85	24952.77	26981.94	29309.27	52623.35
29705.71	30735.5	35125.45	29745.61	50220.93	51351.86	26021.16	26906.49	24970.35	44869.35
27679.93	29561.06	39566.98	31533.5	50865.58	50678.23	24440.52	25461.13	24388.13	42004.82
28421.56	30867.63	31705.31	29141.03	43487.06	49195.59	25770.61	25642.94	23729.08	45005.38

LN371.901	LN372.895	LN372.991	LN372.991	LN372.991	LN372.992	LN372.991	LN372.991	LN372.991	LN372.991
59388.68	20085.94	47894.34	58550.26	79949.75	68194.08	74908.09	82382.36	34645.83	46750.68
58482.46	17661.24	50539.08	56371.48	76652.54	70727.92	71608.09	83990.87	33761.49	45250.58
62421.02	17630.97	49025.5	55198.54	76783.23	66160.2	72097.07	88995.96	32511.1	50808.21
56677.27	16199.21	51998.12	57895.6	75495.47	59576.22	76085.16	88035.87	35277.18	38601.9
61666.81	18800.53	28724.83	56937.25	84972.39	67418.82	35944.74	85141.47	18646.07	41393.74

LN372.991	LN372.991	LN372.991	LN372.991	LN372.992	LN372.991	LN372.991	LN372.991	LN372.991	LN372.991
38915.29	63980.12	51261.62	108613.5	48135.28	94155.22	57837.46	109981.9	49751.37	89479.84
37690.17	61293.9	80898.63	110162	44583.07	88481.5	64280.06	100513.2	46887.96	97422.55
35253.04	62679.66	91114.45	106303.8	43909.66	85085.03	55330.06	103019.4	48150.86	89910.2
36673.93	62218.33	88426.57	105138.9	44093.27	82944.27	60346.8	96766.05	51759.87	88854.69
21541.09	65132.38	75299.39	105556.1	44966.5	93842.4	65105.26	92609.78	57504.88	86873.04

LN372.992	LN372.991	LN372.991	LN372.992	LN372.991	LN372.991	LN372.991	LN372.991	LN372.991	LN372.992
35954.09	40373.97	57681.36	89097.88	78015.58	63951.54	49367.26	52017.79	85728.95	69068.28
38790.84	43616.84	62120.36	98581.45	77526.31	63657.24	53701.13	51209.8	81269.25	68525.48
41629.35	44314.38	56321.7	83454.23	82976.86	58905.41	51346.71	50918.71	82223.35	65036.45
41640.04	48006.32	57773.65	89314.72	70251.25	66694.16	49671.45	52501.86	84670.99	63015.5
36252.52	47676.56	55984.25	75237.24	69468.88	58779.2	43951.13	49628	85701.2	65815

LN372.991	LN372.991	LN372.991	LN372.991	LN372.991	LN372.992	LN372.991	LN372.991	LN372.991	LN372.991
96840.87	44651.35	104395.8	92094.17	108660.6	66721.25	24618.65	51931.39	35454.08	74103.12
96434.93	42054.46	106739.3	87719.21	110809.5	68863.39	25360.31	50724.07	37599.1	83859.35
96075.22	43000.2	100991.5	87230.08	112721.4	66415.13	24589.55	49117.45	34736.82	82880.28
101927.5	39835.11	99813.41	91465.53	112989.3	75318.88	22599.43	50933.92	38229.52	80473.09
88167.37	36793.22	104575.5	87182.28	109446.5	68250.12	21566.23	53292.96	39991.92	71480.86

LN372.992	LN372.991	LN372.991	LN372.992	LN372.991	LN372.992	LN372.991	LN372.991	LN372.991	LN372.991
78150.02	77600.84	81243.42	51140.48	51199.9	63692.05	60461.3	87827.28	63065.29	31225.18
77579.96	78710.99	77826.42	50949.75	52206.78	56754.23	61035.5	101972.9	62586.96	31964.02
81387.72	83796.91	81402.34	48263.82	54478.44	61840.75	61061.84	82613.04	62116.51	25458.65
80611.49	78699.94	80123.19	52167.91	50631.71	62026.85	59974.17	92406.36	61767.84	29932.62
80647.92	72879.99	79202.5	47480.83	50829.86	58713.07	65305.65	88697.35	61034.08	25084.23

LN372.991	LN372.992	LN372.992	LN372.991	LN372.991	LN372.992	LN372.992	LN372.991	LN372.991	LN372.991
43451.39	26776.28	65568.05	61027.43	75672.15	42673.88	67807.36	47283.98	84898.2	92145.91
42683.04	24010.58	69316.85	61226.31	76529.16	41253.48	64790.31	44973.49	85860.68	90700.05
47184.28	31031.7	65694.5	53205.83	76401.86	48017.1	76421.8	44410.33	84876.28	89083.81
44343.56	26474.29	70714.98	57207.14	78333.42	44792.47	68530.39	38416.19	85261.12	85940.12
47305	30346.64	66755.84	58798.21	77652.9	44489.21	62921.82	43984.73	76958.91	79150.1

LN372.992	LN372.991	LN372.991	LN372.991	LN372.992	LN372.992	LN372.991	LN372.991	LN372.991	LN372.991
21141.01	102701.3	149934.3	59661.76	56506.84	30986.7	83609.44	97828.55	34195.71	69091.18
21852.99	104585.3	144664.1	62790.62	56820.26	32814.53	81662.6	99818.96	36105.72	70671.41
22917.14	103678.3	148493.4	59277.48	56822.71	37825.34	80309.89	99480.71	35853.8	73583.66
20861.34	107735.4	146614.7	64685.9	57296.45	31419.66	84961.34	101208	36458.5	70106.43
24072.61	98174.13	136555.9	58303.2	56854.84	32539.9	81516.12	91011.16	37665.41	73142.81

LN372.991	LN372.992	LN372.992	LN372.991	LN372.991	LN372.992	LN372.991	LN372.991	LN372.991	LN372.991
46749.15	48659.41	32462.41	83832.23	141573	52064.05	71654.13	50819.77	114700.3	23785.41
45440.98	41943.98	34828.33	85770.51	148064.5	55093.48	74605.38	57311.37	117167.4	20159.89
48992.35	43994.98	34383.39	86935.79	138213.1	58436.85	73479.63	54394.14	117304	16638.34
42375.99	43856.47	35315.08	75197.63	131830	53546.41	75767.62	59444.52	116405.6	20679.49
48313.53	41758.3	34866.08	75153.07	139742.3	55642.13	73639.99	52038.25	121514.1	19041.76

LN372.991	LN372.991	LN372.991	LN373.009	LN373.010	LN373.010	LN373.010	LN373.010	LN373.010	LN373.010
57963.75	68236.19	18650.62	128847.1	88768.44	62048.96	96316.84	108980.2	50146.66	138314.4
60581.55	70355.09	21818.66	164985.9	75816.5	103666.5	105095.9	78993.65	61349.91	148131.7
55366.65	82157.53	18721.06	132251	51040.68	170050.1	121801.6	77199.92	44545.09	77319.45
55779.26	71888.9	16547.92	90706.01	59968.55	86158.37	71546.68	87864.89	97680.64	118486.5
63740.97	77256.68	20254.61	177040.3	70885.07	91459.05	96593	108329	51043.16	62785.06

LN373.010	LN373.010	LN373.010	LN373.010	LN373.010	LN373.01_	LN373.010	LN373.010	LN373.010	LN373.010
71146.13	94367.12	25586.31	97316.14	151802.5	136145.2	75638.52	51305.44	34414.59	176518.5
88112.78	125658.8	22028.95	94796.26	103649.3	177572.6	64458.95	61730.85	67782.17	150417.3
76369.47	79503.84	15020.3	99251	114122.5	137605.1	47693.89	58495.34	46575.24	154561.4
77380.52	72696.43	14154.31	69211.35	112610	126584.3	55784.67	72921.67	40558.01	143729.5
91352.13	95265.06	16980.19	82865.89	113587.7	103397.3	87990.03	50170.32	52507.31	154969.5

LN373.009	LN373.010	LN373.010	LN373.01_	LN373.010	LN373.010	LN373.010	LN373.01_	LN373.010	LN373.010
115887	60174.54	67710.13	165766.1	65918.68	123163.1	69022.92	107378.8	74224.51	107014.4
192731.5	47829.35	71013.07	120136.3	63352.44	160206.1	70074.76	107418.4	51963.22	110686.1
119671.1	63373.25	70049.14	126921.4	55661.69	159516.7	79871.6	170450	76469.94	78661.43
130376.5	57435.26	74088.59	168218.5	53607.99	95973.49	83301.31	117845.6	58194.29	100155.3
173974.5	51570.44	73729.55	100164.1	45746.48	73099.76	85294.39	129707.2	59274.01	125408.8

LN373.010	LN373.010	LN373.010	LN373.010	LN373.010	LN373.010	LN373.010	LN373.010	LN373.010	LN373.010
59283.52	110402.9	164032.8	80585.56	71917.26	112888	77287.06	58983.92	40663.68	92302.8
64276.03	84248.55	117637.5	68124.39	72817.14	120014.1	86649.29	66611.22	51087.6	88412.14
44080	116230.6	107557.9	65048.98	72745.73	93275.3	75258.09	53472.38	43701.16	98116.14
50252.54	110032.3	114584	90497.26	65777.7	74093.11	99682.54	83815.65	59684.96	98066.18
47915.83	67351.38	121597.2	130788.6	59878.68	94679.97	82636.73	56987.74	59642.16	88257.5

LN373.010	LN373.010	LN373.010	LN373.010	LN373.010	LN373.010	LN373.010	LN373.010	LN373.010	LN373.010
84929.16	53541.27	70460.98	71893.14	32216.74	106861.7	73293.18	22966.82	158781.3	46749.41
112143.4	67662.59	56565.19	59196.57	25653.47	103513	51921.05	29960.32	97068.07	67769.91
107197.3	53552.97	105347.1	81288.1	37136.06	85060.75	68629.56	38757.33	103469.3	64217.37
155051.8	57608.27	49579.48	62235.48	23610.97	129279.7	61970.55	39602.5	112113.5	58655.37
80409.09	66137.15	87575.38	55947.85	30513.22	95177.52	51984.93	29711.73	106916.4	39015.61

LN373.010	LN373.010	LN373.010	LN373.010	LN373.010	LN373.01_	LN373.010	LN373.010	LN373.010	LN373.010
65316.78	129902.2	78911.97	37666.45	67114.2	129622.2	53905.98	77677.76	102423	49258.17
71646.84	120678.2	110215.8	29558.81	56555.86	164409.2	76011.97	90810.22	87307.97	64366.45
47531.11	91508.43	123210.2	38232.75	91206.2	132708.4	59088.5	71046.61	71557.29	49865.42
66159.07	87847.65	126660.5	50404.6	76477.36	128966.9	50677.76	107896.6	76510.96	60760.31
75673.78	108575.9	96807.42	37474.28	78994.09	134816.7	57766.03	99094.44	73225.73	47367

LN373.010	LN373.010	LN373.010	LN373.010	LN373.010	LN373.010	LN373.010	LN373.010	LN373.010	LN373.010
100136.1	53155.1	93457.18	65765.09	22697.03	73842.02	73631.91	38901.98	77773.33	64012.23
120553.1	55307.17	97464.18	102022.7	22373.86	73862.36	76806.65	43559.56	105711.5	53437.5
93607.84	51359.67	101695	91648.4	22452.86	59103.93	66002.93	44151.23	141830.1	50155.62
69441.57	56663.46	90476.77	59301.61	28243.86	144278.2	82340.15	33075.02	98880.09	45480.67
103063.6	86638.98	107712.2	73982.57	21836.17	82972.64	67309.02	31272.17	83774.38	61728.7

LN373.010	LN373.010	LN373.010	LN373.010	LN373.01_	LN373.010	LN373.010	LN373.010	LN373.010	LN373.010
26285.23	82241.93	53449.03	33465.62	121004.5	130585.1	70364.87	30957.95	75971.17	27400.4
40385.6	69114.06	54316.11	34689.57	156857.4	86642.63	100648.4	42333.05	52706.31	26862.74
39258.61	61982.57	65428.54	25533.57	158798	78370.84	90763.18	42915.88	45926.2	32494.36
29963.93	51365.58	50635.62	24605.92	135195.6	98122.29	90278.58	35468.2	65337.93	33052.92
31301.06	58841.65	50022.58	26652.53	170595.5	119013.9	67097.96	36494.28	56153.51	27342.31

LN373.010	LN373.010	LN373.010	LN373.010	LN373.010	LN373.010	LN373.010	LN373.010	LN374.893	LN374.906
43944.86	86860.49	33248.65	98017.5	66068.66	39745.58	123937.8	61640.22	35099.75	82899.84
34430.9	64271.13	34284.13	110996.6	58628.39	46683.91	73623.81	46929.87	36284.31	77988.28
33927.36	59212.29	43950	81348.74	64426.66	40963.59	121063.3	47204.12	19526.59	82356.2
27689.03	72685.46	36510.03	135460.5	107061.9	35669.11	97884.73	63172.41	26325.22	77687.36
36355.35	66080.93	39940.26	118983.4	83728.46	46904.37	83622.73	50394.02	51121.86	85214.41

LN374.906	LN374.906	LN374.906	LN374.988	LN374.989	LN374.989	LN374.989	LN374.989	LN374.988	LN374.989
59761.87	165460.6	175471.8	55250.2	39084.94	32887.75	37585.52	32428.34	46439.98	30314.86
58211.32	186623.6	178912	36289.32	29562.18	31433.02	39284.23	27199.46	42802.54	26799.66
65574.42	171050	171654.2	36247.94	33193.6	37702.4	33965.19	29711.19	32534.9	29670.58
70502.62	174412	173835.2	43941.66	32884.77	39988.54	34341.75	25710.66	27025.97	37941.2
65765.05	183358.1	149458.2	44646.01	36984.65	39793.55	29082.04	39530.2	27413.69	33302.92

LN374.989	LN374.989	LN374.989	LN374.989	LN374.989	LN374.989	LN374.989	LN374.989	LN374.989	LN374.989
42854.32	23642.18	18398.95	64041.28	37761.75	40376.36	48721.48	22714.97	41841.69	46293.74
59364.7	34004.15	13680.62	46409.14	40993.99	46717.19	46591.1	30443.6	38935.04	40168.93
44098.17	28420.91	16965.54	34860.7	36259.63	36740.71	38832.86	26441.03	44462.51	35598.98
34194.21	25356.93	23384.86	52659.54	36496.16	40825.25	54534.71	24801.92	36517.87	31429.56
46509.48	29170.04	19505.16	50133.25	30309.08	34488.26	47861.78	20111.43	62436.81	46497.02

LN374.988	LN374.989	LN374.989	LN374.989	LN374.989	LN374.989	LN374.989	LN374.989	LN374.988	LN374.988
13439.08	33716.35	34813.55	16063.86	21359.78	26696.75	25338.4	30556.07	50825.63	57907.05
20823.72	38341.9	30886.7	19659.95	15701.12	36135.4	23654.41	24521.16	57048.87	46777.37
17657.36	32098.53	45053.97	13577.8	16982.47	27958.07	21618.68	28686.91	58234.1	45721.72
16447.32	27100.73	46722.6	15112.57	15886.5	26971.56	24871.25	29138.34	58857.99	60034.3
18341.17	24342.93	52125.21	16915.15	18173.55	21318.31	20569.08	25918.44	50644.79	60803.83

LN374.989	LN374.989	LN374.989	LN374.988	LN374.988	LN374.988	LN374.989	LN374.989	LN374.989	LN374.988
50114.64	18994.16	24609.55	51901.24	41845.48	61009.9	32024.61	28942.88	30335.42	51832.25
53004.99	16592.59	35090.5	49050.79	58640.93	48013.74	48552.44	34793.89	20125.64	55832.45
44369.09	16470.81	31992.77	50627.89	42808.82	48568.62	28792.92	30040.12	22178.09	51814.48
56446.58	21107.34	31454.93	45503.31	56067.26	51783.61	32820.31	45071.34	23319.59	49240.72
59397.62	16538.75	41249.96	49153.29	47008.31	48028.31	30464.86	25603.11	22643.31	55081.94

LN374.989	LN374.989	LN374.989	LN374.988	LN374.988	LN374.988	LN374.988	LN374.989	LN374.989	LN374.989
52776.67	37037.81	32975.46	33551.14	69388.66	34947.73	69580.52	32731.89	14672.51	42490.38
37880.77	43128.8	37396.21	39756.71	74031.83	59733.7	67724.6	47162.26	17185.79	38790.92
42524.48	43346.77	31837.82	37127.25	59277.57	44993.3	55833.41	36891.81	16120.29	40302.44
30584.81	36751.85	21277.74	42516.9	58127.75	35782.16	73744.43	36693	17223.3	52748.63
34768.67	37372.43	29801.78	39094.52	46222.35	46651.33	54715.2	53857.13	13224.23	39299.46

LN374.989	LN374.988	LN374.989	LN374.989	LN374.989	LN374.989	LN374.989	LN374.988	LN374.989	LN374.989
52660.25	68961.2	33208.91	41759.28	32870.01	29915.66	20729.64	39091.44	35545.83	24375.94
31852.15	93858.19	43647.32	29891.48	36212.52	33758.25	14883.85	34658.06	31888.56	18818.73
45166.17	58620.92	44615.06	37233.01	26334.95	22576.99	16910.26	42530.16	24688.72	19404.95
43201.43	45119.42	58062.46	33291.72	24930.39	30685.08	20863	29423.83	24607.4	18994.49
51543.22	88372.55	41167.12	27765.75	24674.17	30613.52	15909.91	42418.12	45229.86	19102.25

LN374.989	LN374.989	LN374.988	LN374.989	LN374.989	LN374.989	LN374.988	LN374.989	LN374.988	LN374.989
24090.06	21267.94	58897.94	28114.26	24357.03	55150.88	33602.37	31835.53	43805.49	42327.46
42430.73	27635.38	52489.7	29630.47	20950.51	49946.11	59723.99	41427.68	45822.04	29150.41
30747.11	21147.49	56201.7	28596.29	18543.36	41548.78	52588.1	27801.38	43741.42	30688.96
41230.92	20677.48	42725.81	30019.17	12820.45	41966.95	39648.07	30962.32	48728.1	26313.72
30847.74	31426.28	70954.01	29177.48	22707.3	42099.9	42811.67	36480.84	40434.35	35136.95

LN374.989	LN374.989	LN374.989	LN374.989	LN374.989	LN374.988	LN374.989	LN374.989	LN374.988	LN374.988
45620.82	41380.49	44935.36	47588.14	31688.21	45674.37	42149.26	17010.87	62754	56000.6
29427.64	26471.46	48127.63	47241.97	28816.7	57948.1	66284.34	18116.74	73858.15	81316.74
37358.68	28860.39	43552.67	54953.99	34220.63	53549.89	40667.84	20680.71	49971.34	64288.3
50069.66	27397.61	47424.8	61736.28	30693.59	43243.67	58638.56	19551.6	53994.23	68948.38
37503.1	29842.97	39024.9	43638.67	37032.93	46914.41	57001.41	26517.38	58408.56	82667.27

LN374.989	LN374.989	LN374.989	LN374.989	LN375.275	LN375.843	LN376.442	LN376.851	LN376.902	LN376.902
53068.82	35456.54	18893.03	28264	15194.79	45130.15	17556.49	36149.25	40264.58	58612.93
50034.2	37977.55	20913.3	45862.06	22877.95	52985.6	20977.33	27952.62	43083.46	63783.85
46372.06	27936.52	18835.43	25966.4	24425.39	51183.52	18330.24	41863.47	45599.21	56642.74
59093.42	25931.63	24239.61	38417.04	26265.12	42175.94	15696.77	35157.78	40503.87	57198.53
39566.04	24802.36	17126.15	28177.09	26683.49	53451.48	13263.9	59564.45	39359.84	54826.31

LN376.903	LN376.903	LN376.903	LN376.903	LN376.903	LN376.903	LN376.903	LN376.903	LN376.903	LN376.903
33786.8	42343.29	27579.23	56423.83	44760.14	40761.15	37738.12	26085.25	28726.11	46736.18
37317.15	45012.22	27053.54	53220.52	44338.73	40408.03	37704.77	23774.32	33470.78	44214.5
31742.04	45343.93	30933.35	55699.55	38366.99	37972.35	39888.09	26100.31	27563.09	45092.59
35422.91	40535.22	25138.21	55473.94	40535.87	37053.77	36057.68	24873.89	28374.35	44548.49
34802.72	41077.88	26470.43	59595.28	41154.59	37667.73	35967.55	23634.26	30167.1	43238.63

LN376.903	LN376.903	LN376.903	LN376.903	LN376.903	LN376.903	LN376.903	LN376.903	LN376.903	LN376.903
35951.65	42586.19	35207.52	40259.8	35045.1	39272.6	27574.11	32383.49	39520.88	34580.96
36894.63	44329.38	35406.47	39460.5	36094.34	33109.08	30780.61	32874.34	41154.48	39775.79
32998.34	46898.46	32453.51	40600.79	37631.76	40094.12	26930.77	32417.07	39751.34	32103.23
33369.14	42447.82	31077.29	44145.29	34694.31	37234.35	27898.17	29138.32	42694.19	37211.67
33252.33	45380.75	38419.81	35660.72	35557.86	35563.72	30025.39	33115.7	39256.37	36433.81

LN376.903	LN376.903	LN376.903	LN376.903	LN376.903	LN376.903	LN376.903	LN376.903	LN376.903	LN376.903
31657.88	26933.14	19167.94	55328.78	55308.66	38617.16	45542.01	43137.67	25206.9	31107.34
32005.2	26635.93	23095.95	62056.58	49771.15	37754.6	41401.49	42406.66	28395.2	29445.42
33068.05	27580.48	19536.64	56877.41	51591.89	36244.55	45487.27	46749.6	27296.98	26903.83
34484.04	26998	22215.07	57766.99	48493.09	41577.39	47956.94	51229.71	28619.02	30419.96
34115.57	27057.57	20913.37	59872.64	48339.13	37883.05	42050.51	48290.23	27305.18	28099.25

LN376.903	LN376.903	LN376.903	LN376.903	LN376.903	LN376.903	LN376.903	LN376.903	LN376.903	LN376.903
26982.02	45190.06	38569.64	40589	29328.96	25697.25	42892.14	54494.77	42448.6	31360.69
32343.52	43269.34	34989.3	38423.23	28408.33	25252.09	40703.55	56226.76	42569.66	31095.64
28874.72	43453.45	35859.67	37118.71	26281.02	27470.39	35736.48	49356.05	40866.78	29418.35
29974.39	43198.62	33717.65	40931.82	31369.96	25437.86	37205.7	48406.32	46751.61	30952.58
26147.42	41337.16	31449.35	40386.91	29352.85	23638.02	35800.7	48281.3	38369.96	28158.42

LN376.903	LN376.903	LN376.903	LN376.903	LN376.903	LN376.903	LN376.903	LN376.903	LN376.903	LN376.903
30174.44	26590.75	22534.64	31620.81	22617.46	32936.32	48424.25	60416.85	27717.51	23470.07
30089.43	29182.05	19820.12	31909.23	23234.02	37300.76	51169.67	57277.6	25458.79	22309.43
33075.51	23435.97	21096.19	30190.1	21741.76	34805.19	46826.62	55302.59	27488.09	22135.3
30535.49	26680.54	20903.09	30670.85	21904.13	33707.32	47184.36	57312.05	26339.22	22238.05
27503.48	25387.88	20787.97	28059.17	24948.31	35874.28	50839.93	58079.71	27796.32	23496.78

LN376.903	LN376.903	LN376.985	LN376.985	LN376.985	LN376.984	LN376.985	LN376.985	LN376.985	LN376.985
22442.26	25062.36	11417.96	35872.4	105772.5	64924.37	41579.2	75030.4	85824.05	47102.57
24900.25	24527.06	16198.68	55287.78	66162.08	49657.73	43489.25	97547.05	60261.71	49639.47
25554.59	22660.61	19902.9	53026.53	77719.15	57328.76	33867.52	77180.68	58235.77	56363.73
23558.59	25837.5	16827.01	36579.42	70071.42	51364.68	32861.8	91749.87	62505.07	47146.59
20693.37	24269.44	10489.11	56353.9	56714.58	68744.15	35400.22	80826.09	52833.82	60198.19

LN376.985	LN376.985	LN376.985	LN376.985	LN376.985	LN376.985	LN376.985	LN376.985	LN376.985	LN376.985
32059.4	25538.82	82309.24	48115.64	58633.86	64547.14	58843.6	57621.82	35801.88	97400.14
25183.64	39585.8	87922.08	37124.31	52318.78	85194.27	52324.66	61220.82	56269	104156.4
20707.17	40954.09	74219.84	51552.63	58360.13	84327.11	68613.96	47317.5	67663.34	125262.9
19275.24	30451.63	50900.71	60390.72	54568.26	81594.38	71216.71	57030.58	51084.27	93314.81
29264.55	31715.41	71335.49	40372.71	48802.88	89549.12	79457.24	71756.87	47348.86	93003.16

LN376.985	LN376.985	LN376.985	LN376.985	LN376.985	LN376.985	LN376.985	LN376.985	LN376.985	LN376.985
42431.69	63480.55	43216.88	86455.14	28173.9	45340.35	31533.55	44773.7	19865.1	23897.93
29547.5	60082.96	55611.73	115052.3	22837.12	40291.93	57238.36	34447.47	14077.46	33189.91
55811.28	61208.35	50907.02	100458.7	20927.69	62496.7	30069.8	31970.86	21719.17	58925.4
25311.06	55166.14	55452.37	117982.4	33474.03	41371.43	43173.6	36595.92	21653.04	34173.22
49341.95	77535.99	40245	78819.01	25851.77	36098.5	38410.59	31831.41	19781.36	40690.82

LN376.985	LN376.985	LN376.985	LN376.985	LN376.985	LN376.985	LN376.985	LN376.985	LN376.985	LN376.985
16475.39	59830.29	65309.23	50838.37	79275.26	65469.12	68650.78	31216.29	56875.74	60181.76
22187.17	59653.47	67521.21	40110.99	49808.43	72879.41	74720.17	30257.33	45692.74	47732.62
17842.13	39051.45	68190.19	33855.99	65484.73	76849.63	48656.88	20822.21	66471.33	52607.1
23888.03	42338.86	69421.44	32224.55	73255.56	60768.94	68826.32	26907.76	49922.23	61248.94
27571.26	39967.98	51346.78	40315	102998.7	70174.86	59560.36	21803.96	64119.18	44358.58

LN376.985	LN376.985	LN376.985	LN376.985	LN376.985	LN376.985	LN376.985	LN376.985	LN376.985	LN376.985
96143.62	85636.56	46547.41	70577.99	58781.43	36876.01	54071.03	90786.36	45025	72740.3
56044.08	86528	44817.64	57215.51	49229.96	33301.35	65485.65	87935.81	27283.62	73488.88
74163.21	86344.8	57532.76	72147.19	43770.93	33657.83	56098.37	115179.8	44967.49	85778.15
105156.6	90576.24	58363.09	46912.99	68376.11	28796.27	78649.11	115112	43694.04	65232.86
55851.06	87724.51	46930.75	69297.22	59418.36	35253.98	73512.62	97043.97	34112.87	56239.68

LN376.985	LN376.985	LN376.985	LN376.985	LN376.985	LN376.985	LN376.985	LN376.985	LN376.985	LN376.985
43155.7	59300.58	34310.33	15292.23	48313.98	64340.04	64327.31	36035.35	24108.99	28518.81
46389.74	84802.76	62796.65	13323.02	41564.08	47947.46	41064.72	37148.91	28606.8	42127.3
57528.72	58282.69	34936.53	18745.69	64200.75	46308.59	61483.65	31698.01	27407.25	38240.4
55461.02	74526.14	42057.69	16569.58	36789.06	37211.94	45651.83	48565.98	23609.76	27762.8
42409.08	77125.11	42236.44	20050.04	44478.23	37689.79	51391.57	45471.75	17356.09	42344.12

LN376.985	LN376.985	LN376.985	LN376.985	LN376.985	LN376.985	LN376.985	LN376.985	LN376.985	LN376.985
28242.15	83735.59	53660.88	81034.19	43157.96	77476.94	51053.64	30709.29	69973.77	54339.14
27296.79	45552.32	123290.1	61760.81	44165.43	54225.13	49269.79	43825.49	63375.34	46026.41
33092.69	49299.88	48923.54	59828.04	32159.35	44531.36	59406.22	36189.14	49745.94	50162.01
31146.38	67280.19	56483.85	67397.92	31263.48	53695.45	51855.21	24578.64	100983.3	47875.2
36325.83	82294.6	56403.28	80592.08	43739.49	65539.98	54901.03	42300.27	60682.82	39719.52

LN376.985	LN376.985	LN376.985	LN376.985	LN376.985	LN376.985	LN376.985	LN376.985	LN376.985	LN376.985
32459.97	63443.52	42486.37	58746.65	58536.52	65393.56	46664.81	94862.74	27236.39	29285.66
41218.98	74166.66	36392.13	56680.68	52886.87	46540.72	78825.44	53641.67	29221.24	25552.88
35730.18	55482.07	41506.67	53659.08	65599.64	42135.28	45276.17	75707.52	27129.96	28971.74
39201.36	51056.45	66814.5	56427.63	66074.62	51033	33810.9	68331.45	28129.98	27458.72
42474.06	69867.21	47690.14	49036.14	38964.03	60100.92	53467.81	64634.36	37418.62	27389.34

LN376.985	LN376.985	LN376.985	LN376.985	LN377.004	LN377.005	LN377.004	LN377.005	LN377.005	LN377.004
85581.29	73645.21	28186.1	18430.72	26746.42	23653.69	49913.47	24772.25	26319.2	55607.77
139173.9	102278.4	15628.39	15236.57	32913.1	24275.77	50261.78	21540.19	28179.92	49915.75
90311.08	79052.37	21149.26	13732.21	33529.08	26894.7	47368.19	23055.04	26759.79	50843.79
101166	76705.74	19599.58	18025.31	31245.5	20326.55	46109.64	22000.75	24419.64	51982.92
92213.9	89641.08	24300.21	24410.68	29918.56	23952.24	43184.16	24755.39	22405.3	47276.02

LN377.004	LN377.005	LN377.004	LN377.004	LN377.004	LN377.004	LN377.004	LN377.004	LN377.004	LN377.004
12140.67	53617.94	50912.99	43502.82	54777.87	42356.31	33186.17	32892.79	82421.5	44887.78
10946.82	51779.19	54861.47	48945.44	57478.84	37583.19	35255.94	27829.98	76759.86	48230.21
11148.73	52217.62	47982.08	45443.2	57034.76	33223.35	35704.53	28645.07	89962.61	41962.95
14216.7	50514.51	52750.7	43546.3	55823.83	34633.1	32442.27	26293.33	74108.27	42597.69
13441.39	46046.04	54160.64	39489.59	55795.19	38808.69	30860.77	29999.21	72142.9	44157.54

LN377.004	LN377.004	LN377.005	LN377.004	LN377.005	LN377.004	LN377.004	LN377.004	LN377.004	LN377.005
74405.16	38710.81	39150.91	34815.06	30827.39	39536.24	46616.25	33554.13	34304.95	23532.14
80737.39	38688.11	40696.81	35931.48	32585.43	42332.17	44849.11	34670.27	56034.44	23038.86
66228.09	34308.65	37634.59	36470.9	26477.32	40093.49	46190.55	31722.83	54563.41	24942.08
68924.15	38337.64	36704.27	30503.98	30703.94	39896.52	45724.95	34959.22	55421.44	22031.77
66601.07	37265.28	33801.63	34147.75	31588.46	35723	38850.57	32936.87	56555	23089.65

LN377.004	LN377.004	LN377.005	LN377.003	LN377.003	LN377.004	LN377.004	LN377.004	LN377.004	LN377.004
36300.9	37207.14	49141.43	88720.04	64820.43	41137.86	41067.92	32688.01	44896.92	35651.26
41495.22	37590.77	47194.83	90230.96	74489.36	39753.88	44858.24	31930.32	41793.61	36635.38
37044.23	35260.98	48201.34	89337.14	68203.99	37001.18	43515.71	30637.41	36899.04	31166.49
36960.67	36701.05	43948.51	91965.22	67540.62	40361.54	46488.25	31384.03	39881.13	31623.6
36207.8	36302.74	44466.08	81627.94	63332.35	42725.14	42786.02	30127.99	38249.38	34275.69

LN377.004	LN377.004	LN377.004	LN377.004	LN377.004	LN377.004	LN377.004	LN377.004	LN377.004	LN377.004
61103.83	30409.73	48512.72	27500.31	64279.51	76409.81	98042.57	75401.84	51501.98	72155.48
60243.65	30368.02	47670.8	32403.77	70746.12	75738.78	97576.06	72169.4	45456.67	68577.5
62270.87	30097.11	47308.54	30366.29	64673.35	80560.22	87346.24	68999.24	49957.68	64683.01
60682.4	28085.33	46723.08	29051.1	65336.56	76149.1	89994.4	76804.78	47370.32	65289.76
58893.78	27533.41	44785.63	30191.91	60020.33	76638.25	86582.7	68703.89	49129.55	63640.93

LN377.005	LN377.004	LN377.005	LN377.004	LN377.004	LN377.005	LN377.004	LN377.004	LN377.003	LN377.003
16719.31	63885.89	23512.3	41298.28	49005.16	18535.45	30582.58	64125.95	75536.42	100400.7
19520.19	59522.21	26427.06	44180.61	51340.25	18572.28	31388.5	65348.49	76161.35	90418.09
18865.55	60677.18	23549.03	42495.83	45884.77	18586.41	26726.1	63998.17	71926.07	92972.16
17390.29	60506.19	24084.84	43839.28	48169.09	17963.42	27390.27	63394.06	78712.6	93645.03
15513.96	58619.86	24660.83	42030.74	49070.07	17588.87	26533.77	62162.65	74059.33	89769.33

LN377.004	LN377.005	LN377.004	LN377.005	LN377.003	LN377.004	LN377.003	LN377.005	LN377.004	LN377.004
61854.63	23037.41	43325.07	37723.12	78901.91	84230.19	65104.27	26772.18	97129.34	54858.02
61032.34	22991.02	45725.49	35781.11	77555.25	80817.85	65780.09	26079.26	99610.88	48273.47
59049.2	20943.62	44280.8	37007.4	79406.26	82943.37	62699.34	26934.23	101281.8	50600.54
54841.4	22581.75	41581.61	36566.18	76904.69	85175.04	58908.18	25361.94	101191.9	53065.12
59084.97	22961.88	40556.48	38692.69	80056.56	81401.68	60270.24	27125.7	97626.27	47957.86

LN377.003	LN377.004	LN377.004	LN377.004	LN377.003	LN377.004	LN377.004	LN377.003	LN377.004	LN377.005
104557.2	66047.59	81714.46	44701.17	59150.06	53322.84	92382.17	73718.55	37446.97	20078.74
92387.88	66598.67	77544.81	46856.27	60990.63	52311.45	81923.46	75746.54	38840.5	21969.36
89627.95	64515.14	74514.68	47937.23	60040.73	52656.5	79672.12	73128.35	39015.32	19938.5
99845.66	61914.38	71127.79	41962.56	59586.6	53496.19	80861.5	71171.43	34005.38	19224.5
95759.69	65935.72	77813.83	44969.71	57148.11	51202.11	72392.06	71079.92	38575.23	18860.24

LN377.004	LN377.004	LN377.004	LN377.004	LN377.003	LN377.004	LN377.005	LN377.218	LN377.218	LN377.218
77606.14	68793.27	110720	68779.81	106432.1	84800.47	24997.02	16572.81	18508.7	17837.07
77567.97	63273.13	106205	66568.52	108851.2	86405.27	24680.07	26368.46	29071.48	24803.08
79115.14	67247.01	111647	68395.63	101486.2	83482.97	25478.49	25959.44	30203.07	26105.24
76111.87	62691.87	104538	69831.47	106803.1	83656.21	24868.8	25426.56	29432.4	25626.14
73829.88	64225.65	107519.8	62196.73	98127.08	80633.03	24630.32	26908.11	29146.7	26386.86

LN377.272	LN377.272	LN377.272	LN377.273	LN377.272	LN377.272	LN377.272	LN377.273	LN377.272	LN377.272
605875.8	1058288	490727.4	496048.4	574520.9	170350.9	736612.5	376538	402811.8	507547.8
538531.8	1749214	372846.8	536403.7	719312.3	152149.8	850344.6	407750.8	365800.1	510905.5
551334.9	1262125	699344.5	544864.3	597502.5	193182.5	803968	420207	523348.6	575868.5
456263.4	1371418	522204.6	583424.4	613885.4	190493.9	581264.9	471094	652063.1	647950.5
590921.7	2007894	562926.6	357404.4	846605.2	118112.7	635737.6	341541.7	594050.6	495667.8

LN377.272	LN377.272	LN377.273	LN377.272	LN377.272	LN377.272	LN377.272	LN377.273	LN377.273	LN377.273
535613.6	610476.7	317953.5	682650	786877	756416.5	409758.9	604639.1	281492.5	450527.1
589894.9	834553.4	469319.3	535325.7	644135.3	1237239	632494.7	614133.2	368334.8	500621.4
570025.6	725693.4	368526.2	906395.6	645048.5	1049825	469639	480389.6	595794.3	420523.7
627750.7	852425.4	418081.8	694651.9	737714.9	769282.2	515363.2	406800.2	509561.8	653540.7
576661.1	866436.8	358140.7	695854.7	1195794	659153	478495.1	547539.6	375251.1	347146.4

LN377.273	LN377.272	LN377.272	LN377.273	LN377.273	LN377.272	LN377.272	LN377.273	LN377.273	LN377.273
299025.4	529963.6	818299.9	966044.6	387610.4	738859.7	618345.1	498252.8	374046.4	363271.2
438971.8	737109.7	614319.9	1111220	431234.7	634284.7	504808	509325.2	531472.9	658488
293666.5	548196.1	697675.9	1276306	327718.5	737817.7	392389.2	390235	543647.2	632529.9
434033.4	613158.5	551760.5	1208710	398570.9	700412.2	557247.3	451754.7	444186.6	376506.9
376233.2	623424.3	780932.5	1555599	416575.4	660238	420764.2	562285.8	511851.7	444658

LN377.272	LN377.272	LN377.272	LN377.272	LN377.272	LN377.273	LN377.272	LN377.273	LN377.272	LN377.272
604345.9	783385.7	473013.2	888669.5	375863.4	367578.1	651098.5	313645.2	697334.1	395923.7
542123.2	732955.8	714090.6	588076.1	384314.9	439355.9	656909.4	280481.9	346159.9	634110.8
918235.6	804291.8	761945.6	718266.2	418496.4	400970.6	839444.2	348249	497938.8	453263.3
836605.6	852345	891458	713334.3	391039	389436.4	700491.3	356686.3	361034.5	523318.4
613592.6	675597.2	747203.7	617238.3	329737.7	448828.1	626222.5	438939.9	558940.7	452740.8

LN377.273	LN377.273	LN377.272	LN377.272	LN377.273	LN377.272	LN377.272	LN377.273	LN377.273	LN377.273
346047.1	410672.9	565098.6	559698.6	211956.2	652781.8	800552.3	1114764	265363.6	332849.4
543086.4	409695.4	759026.1	383792.8	159123.1	672240	793540	909912.1	237399.4	533767.9
507461.3	391513.4	813609.9	556148.8	230148.1	749281.4	622992	1536481	262265.2	472589.8
408399.4	410162.1	875908.2	433186.3	237693.6	692277.8	814464.6	918710.1	378746.3	404208.4
358847.2	447102	970554	559889.6	193120.8	860070.7	1302006	870167.5	281658.5	321559.5

LN377.273	LN377.272	LN377.272	LN377.272	LN377.273	LN377.272	LN377.272	LN377.273	LN377.272	LN377.273
395328.4	454280.2	522155.3	459202.1	398358.3	820731	812693.7	278411.9	698475.3	459083.8
314612.9	336418.7	567494.2	436484.1	423758.8	899957.6	592683	254624	613962.4	605152.9
379008	464339.9	814813	444805.8	495847.9	1280778	721237.1	228589.7	570964.4	422562.6
303616.9	347101.4	1067569	404979.1	473048.3	1370079	735297.1	232046.2	584794.8	354866.9
426652.9	375516.4	423875.7	376153.2	467122.5	816473.7	539292	396558.9	732931.9	611707.6

LN377.273	LN377.273	LN377.273	LN377.272	LN377.272	LN377.272	LN377.273	LN377.272	LN377.273	LN377.273
701960.7	492525	383507.5	520547	1293325	739966.8	593590.8	531483.1	1399294	395533.4
889678	432135.9	387613.8	694445.4	694789.4	988457.7	509431.1	615437.5	575517.8	457857.2
670721	386629.8	488420.9	590148.9	1066750	1057381	350725.5	918853.6	602952.1	368671.7
1360246	417063.2	340028.2	505600.7	1125046	798910.9	467266.1	528110.1	633510.6	366156.2
1089060	339718	429318.1	625269.2	784064.7	1171775	671992.6	512868.4	629433.5	338634

LN377.273	LN377.273	LN377.273	LN377.272	LN377.273	LN377.273	LN377.272	LN377.273	LN377.273	LN377.273
423414.1	333257.7	416982.6	696475.6	589860.9	417903.2	625203.3	451333.3	512880.1	828601.9
387702.8	314398.7	333505.6	461925.4	815066.2	418186.8	771670.6	419106.7	635409.6	821759.6
577719.7	444923.7	441477.5	560771.6	599012.7	416310.9	651531.9	488006	459335.8	873680.5
413173	350269.1	408069.7	612098.1	642733.4	353492.4	623127.5	433115.7	354336.6	836049.4
595500.7	487208.2	641752.6	443875.2	524014.2	422537.4	639557	473851.7	296297	863607.6

LN378.275	LN378.276	LN378.276	LN378.276	LN378.276	LN378.276	LN378.276	LN378.276	LN378.276	LN378.276
97041.07	23427.09	134541.2	161595.6	90798.18	125437.4	173706.1	60698.08	69298.07	64998.39
90262.63	38391.18	135205.6	277202.1	91694.12	129176.6	104038.7	52546.4	80429.84	100451.4
93342.02	29385.1	95474.31	365251.3	72423.97	204354.7	134318.4	68193.25	107702	86914.15
96845.66	30055.8	133778.8	236598.3	81929.99	122923.1	180531.3	75603.65	69099.77	68838.39
107799.4	40704.78	125825.9	228634.4	111008.7	102548	152588.5	93849.91	103757.2	75132.33

LN378.276	LN378.276	LN378.276	LN378.276	LN378.275	LN378.276	LN378.276	LN378.276	LN378.276	LN378.276
76950.75	111939.5	115865.5	100886.6	187451.7	124519.4	105038.2	100725.5	190320.4	216029
97953.36	92397.7	88800.51	87195.47	335263.4	102049.6	156703.5	156232.1	191223.6	156547.6
99640.57	109976.8	104320.9	87504.44	241253.5	103716	155137.1	143044.5	187633.8	138581.4
117382	82869.72	76034.41	76428.01	163070.7	111526.8	127661.2	128528.8	245036.3	144581.9
118590.8	82031.06	69449.06	83442.68	207086.1	117517.1	119635.7	127669.9	319292.8	165780.7

LN378.276	LN378.276	LN378.276	LN378.276	LN378.276	LN378.276	LN378.276	LN378.276	LN378.276	LN378.276
132086.9	122107	116240.1	179135.6	95745.12	214902.7	163610.6	117960.8	143542.3	91304.77
142031.3	112642.6	128805.1	140451.6	94158.15	160769.4	93375.2	144655.1	107766.1	65732.68
114833.7	164670.9	141552.7	129024.1	76704.2	122076.6	111883.7	130114.3	93383.87	121509
107370.6	162319.1	105689.9	172482.4	98428.41	154229.3	123796.8	100924.5	101156.9	78717.28
133961.4	225338.9	143020.5	114079	101540.6	155046.6	128774.6	111488.5	119108.5	73304.95

LN378.276	LN378.276	LN378.276	LN378.276	LN378.276	LN378.276	LN378.276	LN378.276	LN378.276	LN378.276
120667.1	130286.1	68646.78	112430.5	129555.3	211446.8	84534.27	79969.21	126028.2	55397.15
137849.7	101343.2	87605.94	65752.42	138422.5	187432.9	97193.54	116826	71530.56	77793.77
87171.9	100951.5	74380.38	107765.8	116457.7	180874.1	80531.77	101544	82769.81	63687.39
91780.12	89315.37	62371.41	69446.78	116407.9	187135.9	112777.9	83527.75	80088.94	90721.47
81672.58	113473.3	108388.4	92059.59	110578.7	172806.4	74741.46	117890.5	63863.44	75149.06

LN378.276	LN378.276	LN378.276	LN378.275	LN378.276	LN378.276	LN378.276	LN378.276	LN378.276	LN378.276
85962.18	175058.4	124187	202204.5	129015.4	60101.84	98483.85	91564.86	75104.04	83217.32
76950.65	121765.4	107584.5	188129.9	180070.4	112424.7	120098.7	99462.06	94147.12	90201.52
98284.15	124184.8	82957.49	211560.1	100454.3	72633.1	138917.9	103768.7	101243.8	95439.56
80755.12	148654.1	80739.9	225062.7	159320.8	73086.34	94029.76	88822.91	73657.38	108866.7
91076.65	165745.9	82863.33	215068.6	113671.4	76772.8	110851.6	95858.41	81935.97	112465.1

LN378.276	LN378.275	LN378.276	LN378.276	LN378.276	LN378.276	LN378.276	LN378.276	LN378.276	LN378.276
133352.4	162333.4	113624.2	115315.9	217901.1	190513.8	97322	113810.8	99558.35	50485.54
80808.15	152134.8	70749.63	140231.3	143962.9	212405.2	91055.08	131289.1	86144.81	42651.84
127126.3	144229.9	97391.47	114036.4	159653.8	237153.8	149814.5	122171.8	83585.07	43060.51
104904.4	219601	95305.72	116716.5	213007.8	227338.5	92098.19	120866.7	86051.15	44820.5
78579.73	144655.5	84621.4	111994.7	190458.4	213064.6	90672.05	134861.5	82312.65	60856.57

LN378.276	LN378.276	LN378.276	LN378.276	LN378.276	LN378.276	LN378.276	LN378.276	LN378.276	LN378.276
131685.9	97772.62	99886.26	137895.5	162443.8	97339.38	94642.7	165780.7	220544	211984.4
125827.6	125676.7	118914.4	97859.18	186807.6	73482.08	83762.83	245110.8	179919.6	131653.5
156034.8	96002.02	103989.6	118099.5	170316.9	87363.5	108614.6	163641.5	200490.8	145631.3
139522.6	92814.04	163103.7	124988.5	201351	98102.69	125240.9	196274.3	286189	152091.5
136608.4	93585.27	112857.6	115623.5	184894.6	87177.58	94131.88	183249.5	155390.9	201035

LN378.276	LN378.276	LN378.276	LN378.276	LN378.276	LN378.276	LN378.276	LN378.276	LN378.276	LN378.276
79532.24	103068.8	169254	57336.65	84227.73	184007.2	127999.9	96277.76	122406.5	65727.04
65738.3	97974.03	104920.5	70759.35	87038.61	223467.2	119190.7	83096.57	114154.1	68271.33
64861.14	92493.54	139267.4	55876.57	72774.39	269406	129904.2	68471.39	92741.43	76520.66
89090.64	69000.67	95905.76	68720.04	96375.04	196289.2	115227.8	98455.39	121420.2	67864.34
76986.16	127235.9	101121.2	68039.69	87115.29	175178.3	129781.9	101017.9	120313.7	57247.4

LN378.276	LN378.276	LN378.276	LN378.276	LN378.276	LN378.846	LN378.918	LN378.918	LN378.918	LN378.918
78513.88	99547.14	44880.8	93768.16	63483.92	56541.14	151852.5	236779.2	98958.17	294393.3
78682.83	115600.8	50105.75	102535.1	86428.53	52786.3	125013.8	229678.1	162107.2	219045.2
76749.11	81161.77	54327.37	105424.6	59905.89	80139.48	140681.9	281456.4	115686.8	194843.6
82064.73	99873.06	51377.68	125326	56384.4	77905.57	132212.9	228493.2	110547.4	233110.2
86641.59	106152.6	51962.31	85985.02	70326.92	73440.32	134822.3	237941.6	98657.15	206783.9

LN378.918	LN378.918	LN378.918	LN378.918	LN378.918	LN378.918	LN378.918	LN378.918	LN378.918	LN378.918
161544.3	139776.9	153609.5	281086.7	257991.3	322576.7	275544.8	251320	228682.7	247113
141760.3	113480.5	143902.7	295813	148391	241499.1	184152.5	295701.6	244036.2	192074
221166.9	155415.4	129690	200473.1	200547.6	249568.3	186393.2	230949	237915.6	204963.8
183968.1	144615	149844.3	266662.9	211524	227953.9	202400.4	235631.1	206074	180029.2
171387.8	120934.5	210335.9	351179.5	223294	215812.9	248716.4	273685.3	272923.7	246067.8

LN378.918	LN378.918	LN378.918	LN378.918	LN378.918	LN378.918	LN378.918	LN378.918	LN378.918	LN378.918
294813.2	229453.5	162354.5	217668.4	219559.4	206418.5	158098.3	243751.3	236540.3	316995.7
168210.1	191767.4	242623.8	251917.9	263287.4	236221.9	248261	204774.5	226830.5	247966.6
198896.1	255746.4	218377.8	161813.9	221998.4	208415.3	219405.4	227440.4	279913.9	224204.9
283396.6	207956.7	190062	365702.4	284305.9	242189.3	194231.8	255012.8	275661.3	196735
234643.5	199642.5	254122.2	156454.3	149929.6	229010.5	207633.7	164325.1	280428.2	232069.7

LN378.918	LN378.918	LN378.918	LN378.918	LN378.918	LN378.918	LN378.918	LN378.918	LN378.918	LN378.918
216660.8	197472.2	218591.9	231466.6	218410.8	273719.5	304105.7	222193.5	164590.1	302875.8
217137	243675.2	179016	342167.4	242138.6	293898.9	262832	224451.4	154827.8	260684.6
194896	221453.6	191537.5	244660.6	273267.3	228077.7	291326	175513.7	131057.4	247207.2
192851.8	258926.9	184169.5	232162.1	315228.7	273319.9	214605.2	202541.8	129293.6	183454.6
258207.1	201972.2	186087.5	313179.6	189611.4	185956.4	148656.4	280663.1	237286.5	174384.7

LN378.918	LN378.918	LN378.918	LN378.918	LN378.918	LN378.918	LN378.918	LN378.918	LN378.918	LN378.918
185989.4	97485.13	130466.7	97860.84	307772.7	69288.39	82926.75	122095.5	221035.1	89704.86
231053.6	124669.3	127652.7	119066.9	219895.5	71419.74	46062.52	152583.7	270080.6	146245.9
183769.6	84840.56	130220.6	78382.73	297546.5	70645.89	49033.72	106900.7	207203.8	121273
196564.3	101370.5	152192.5	83274.73	230140.4	73912.23	58899.46	122424.6	298016.3	120983.4
189256.9	105585.8	124890.8	71064.56	332894.8	51311.7	46108.81	159327.3	188431.5	85814.31

LN378.918	LN378.918	LN378.918	LN378.918	LN378.918	LN378.918	LN378.918	LN378.918	LN378.918	LN378.918
194953.8	286780.5	160158.5	182071.6	256050.3	162101.1	207363.4	289736.7	201135.8	112454.8
230228.6	231339.9	97089.72	176795.5	177582.2	176648.4	221704	188056.4	234196.4	97224.27
257180.7	172420.1	82541.09	168474.5	258892.2	236269.6	187445.3	168957.2	196707	107828.8
218795	201888.3	116080.4	174315.1	237126.7	214216.2	290388.8	170394	323234.5	127083
218917.9	196112.9	104152.1	179049.1	259488.4	260919.4	184300	208187.5	243250.9	109647.4

LN378.918	LN378.918	LN378.918	LN378.918	LN378.918	LN378.918	LN378.918	LN378.918	LN378.918	LN378.918
152330.6	174327.3	193039.1	73946.73	254874.7	212345.1	107223.1	230484.7	239560.9	87680.25
112787.4	178309.8	244001.4	100087.7	222645.4	233007.7	102329.6	197405.3	210214.5	69785.1
101632.8	215190.1	218214.9	67905.11	244492.4	281214.6	126222.5	210182	269229.5	68487.92
99321.89	162122.7	231967	81220.68	252022.1	207040.8	119544.8	232152.9	208043.9	88630.66
166716.2	158594.4	185711.8	74306.88	296513.6	201628.2	86690.68	183003.1	197011	55352.53

LN378.918	LN378.918	LN378.918	LN378.918	LN378.918	LN378.918	LN378.918	LN378.918	LN378.918	LN378.918
151032.4	79388.51	57665.89	183907	53452.51	118545.1	91588.83	85476.68	188631.3	46364.02
235573.9	50599.16	46838.67	299265.7	51503	124259.3	79857.42	57291.05	584873	60542.73
272114.8	44955.01	48349.73	203026	104049.9	87466.31	124938.7	49194.1	184382.5	58191.75
191222.7	76482.71	47687.09	188554.1	71866.13	95904.22	97053.04	62376.09	243107.5	30916.68
181028.6	31909.71	53573.63	188798.8	53095.33	111940.7	85635.9	65395.15	220449.4	20733.46

LN378.918	LN378.918	LN378.918	LN379.001	LN379.000	LN379.000	LN379.000	LN379.000	LN379.001	LN379.000
230313.4	322629.5	234261.4	15195.65	31679.73	44026.07	28056.47	38619.91	18777.62	44904.62
297760.5	208396.3	255222.8	13908.86	27931.58	44300.09	30086.68	37658.75	18047.28	48991.26
278926.8	272047.6	244036.3	13501.49	31468.41	39302.21	30089.38	44174.49	20663.7	39508.03
230851	217721.3	283269.8	12990.07	30127.28	45372.03	27970.03	40849.6	18472.1	45453.07
242885.2	284612.3	245494.7	15265.62	29869.81	38286.27	37661.55	30261.3	20730.11	29559.41

LN379.000	LN379.000	LN379.000	LN379.000	LN379.000	LN379.000	LN379.001	LN379.000	LN379.000	LN379.000
29254.58	36661.34	29330.87	39289.82	53282.53	48309.96	27326.65	51753.58	22497.13	42853.72
26572.09	35234.55	31022.18	36563.71	45731.34	54832.05	26914.96	55832.13	23799.53	41413.8
38670.08	26241.83	38881.96	36954.8	40386.79	44586.01	23663.56	48569.35	29090.03	48165.85
27996.01	31721.6	34706.15	43116.03	48216.13	54144.48	25568.48	55636.16	25245.16	40520.76
36408.96	38765.06	34057.71	51379.04	40134.98	54791.96	28365.65	50204.15	29367.92	35640.99

LN379.000	LN379.000	LN379.000	LN379.001	LN379.000	LN379.000	LN379.000	LN379.000	LN379.000	LN379.000
38416.61	40411.04	34456.13	23442	30902.68	22269.33	48126.7	31226.54	46726.66	29140.15
35772.64	41140.35	31740.73	25944.62	26810.1	23621.68	50303.22	32780.6	42382.23	28700.58
37533.82	40100.87	39402.84	21054.92	28176.98	24821.94	59057.94	38382.29	53938.93	35336.66
37604.72	41491.37	35781.72	23176.47	27776.8	26843.96	49771.45	29403.58	41488.69	32084.93
47789.95	39316.1	32876.31	21472.71	25404.63	28706.26	43121.96	25430.14	51686.25	23557.03

LN379.000	LN379.000	LN379.000	LN379.000	LN379.000	LN379.000	LN379.000	LN379.000	LN379.000	LN379.000
45616.35	52346.61	29812.81	35618.1	38879.01	26693.94	28125.5	29328.67	35357.02	53955.45
49756.4	52227.25	27155.79	34271.68	38024.13	30732.65	23384.47	25625.11	34704.32	55050.5
47528.24	58300.53	25489.86	40641.86	33128	28522.99	38350.14	32080.28	44662.14	50883.9
49568.42	53521.04	28093.39	33064.26	42984	26671.86	23414.1	30004.56	37514.85	51926.49
34721.85	56617.02	28895.04	34151.08	41714.16	29182.09	28541.19	40187.23	31915.97	80788.15

LN379.000	LN379.000	LN379.000	LN379.000	LN379.000	LN379.000	LN379.000	LN379.000	LN379.000	LN379.001
42776.15	44063.91	53923.36	25836.61	59267.9	22098.75	25795.7	38496.24	37307.02	24263.31
46802.34	48562.97	55363.38	27263.28	60403.33	27999.99	25926.13	41516.12	38542.02	19345.36
38409.24	55752.97	41684.15	26150.84	68126.35	23651.6	23467.99	46400.04	35550.86	17981.83
45132.09	46551.4	54265.31	27028.35	58991.22	24391.8	20594.05	40650.05	36083.56	19403.33
46101.32	46366.28	47428.39	26959.63	51920.78	23052.42	31740.55	44843.2	51937.13	14466.86

LN379.000	LN379.001	LN379.000	LN379.000	LN379.000	LN379.000	LN379.001	LN379.000	LN379.000	LN379.000
32090.95	23478.9	30524.31	65586.46	36164.5	42208.36	35630.81	23976.05	22542.63	41690.41
26980.21	21780.52	35102.08	62412.02	34927.05	49644.94	41244.78	22622.87	22300.8	37244.26
26046.4	23211.72	35333.76	58749.16	51334.71	64234.19	36485.71	34067.88	20964.38	35208.35
28700.77	21077.38	30830.08	59975.58	37896.77	43557.46	34586.29	22851.32	20801.12	40364.29
35669.55	21259.64	32207.26	80294.3	44997.46	36536.92	32921	20572.92	26555.45	47919.84

LN379.000	LN379.000	LN379.000	LN379.001	LN379.001	LN379.000	LN379.000	LN379.001	LN379.000	LN379.001
39536.6	45625.93	46068.62	14161.62	21447.28	22864.97	37496.34	27416.93	49659.42	17128.26
38358.36	50211.8	41534.82	11026.68	19096.19	21370.74	35035.86	28806.91	50569.04	17864.04
50684.59	46943.89	44608.77	15749.21	20392.36	26514.68	35518.9	29476.56	58862.79	18545.67
39223.07	50092.59	40726.73	13541.22	21247.78	20307.43	41899.54	27950.19	46996.08	15537.8
36347.1	51006.07	52419.82	13932.41	18724.13	26029.35	34440.71	21394.58	43567.84	14624.76

LN379.000	LN379.000	LN379.001	LN379.000	LN379.000	LN379.000	LN379.000	LN379.000	LN379.000	LN379.000
54357.19	24466.36	18515.07	29389.28	44235.56	32638.28	27933.4	23362.84	35036.59	37309.97
61201.94	19260.88	18623.42	30923.74	49097.75	28628.25	28710.68	24951.18	30616.45	40090.66
86541.06	22478.83	22337.76	36806.26	60033.38	18086.39	33909.22	31859.82	40817.57	49966.98
54553.74	23512.22	18036.89	25625.63	43839.65	31753.95	29182.84	24441.6	35425.34	37110.9
58906.08	20934	21403.19	26727.76	36082.8	22758.46	27699.79	27783.54	31226.89	38454.55

LN379.000	LN379.001	LN379.000	LN379.001	LN379.000	LN379.000	LN379.000	LN379.020	LN379.020	LN379.020
58679.03	17764.21	48183.33	16174.82	48637.42	37259.27	31249.07	44812.02	51029.49	52109.41
51758.25	16091.88	50238.47	17193.14	41433.49	35991.2	31861.89	39133.91	50460.91	51542.12
56780.69	18805.3	54433.07	18763.05	39752.34	34877.13	29314.9	40293.29	47211.63	51196.45
51995.27	16828.07	53280.37	16168.31	45091.38	37262.43	35915.95	41792.35	44263.91	48788.61
61145.64	13996.44	61892.65	15114.24	57594.34	41389.65	31638.01	37301.31	47126.56	53129.82

LN379.197	LN379.197	LN379.197	LN379.197	LN379.197	LN379.197	LN379.197	LN379.197	LN379.197	LN379.197
57402.2	52982.74	35801.54	32363.71	61192.76	52440.37	42046.55	92356.87	35260.99	44277.17
57928.31	54580.79	32004.95	42780.19	64915.61	50196.05	45181.81	92529.76	32385.68	44571.2
56194.76	54752.54	31494.23	31930.41	60890.38	48250.2	47812.95	95350.31	32336.28	39531.37
62009.45	52499.22	30667.25	37292.52	61921.83	50902.87	42811.59	87567.31	37738.05	40913.81
60376.74	55532.33	33987.48	35616.52	65679.72	48241.99	48149.31	96142.65	38297	38832.99

LN379.196	LN379.197	LN379.197	LN379.197	LN379.197	LN379.198	LN379.198	LN379.198	LN379.198	LN379.198
39859.94	34264.29	44787.25	44637.51	50197.07	24427.47	35564.51	31771.41	21520.38	25688.78
35938.67	33586.08	46724.68	43759.18	49073.54	19221.34	35525.46	34601.02	27327.7	25189.45
33100.12	31768.33	49391.45	47376.3	48808.78	25021.06	39596.93	31206.5	24102.57	27067.74
33522.03	32894.68	44946.07	43645.59	52346.36	23198.74	38785.32	30544.64	21772.29	24935.24
31130.48	33310.77	47047.24	42797.12	50047.61	24749.09	41128.33	30634.32	25294.4	28744.8

LN379.198	LN379.198	LN379.198	LN379.197	LN379.198	LN379.198	LN379.198	LN379.198	LN379.198	LN379.198
33035.88	29795.1	56785.84	17848.45	28019.84	17735.02	30612.59	17785.77	26474.46	27854.07
33861.55	33280.16	47439.9	14623.9	29804.9	16305	28914.04	18610.55	28723.3	25519.26
32260.87	29064.72	54748.23	18936.49	30356.32	16395.52	28818.66	17698.99	26595.36	25536.22
35311.03	31852.73	56131.69	21539.38	28520.81	13919.06	26784.67	17502.23	28745.74	25430.27
33320.92	36071.82	49588.26	17709.6	30552.84	17214.52	28866.16	16337.44	27773.7	29697.62

LN379.198	LN379.198	LN379.198	LN379.198	LN379.198	LN379.198	LN379.198	LN379.198	LN379.198	LN379.198
11122.16	42529.15	45975.75	15834.64	25958.46	26797.58	31220.76	25629.55	25999.8	20490.85
11075.54	43410.42	45452.62	18097.2	23621.92	28914.23	35446.64	25713.22	27716.72	19186.21
12580.19	40540.65	46535.12	16118.26	25796.83	30552.42	34989.12	28546.91	25616.33	19136.27
12942.5	39796.84	46583.25	14208.53	24001.7	30121.82	34174.7	29409.97	24174.42	18829.47
11409.09	39397.4	51865.37	16442.02	24855.45	28930.94	33529.29	26829.43	22941.19	20975.25

LN379.198	LN379.198	LN379.198	LN379.198	LN379.198	LN379.198	LN379.198	LN379.198	LN379.198	LN379.198
29648.8	35134.81	25052.3	26200.05	45976.52	27300.38	34488.2	16127.8	42436.65	28694.46
31899.52	33674.22	26421.17	27044.85	42572.29	26566.33	32905.14	14141.8	44897.58	28120.76
30016.9	32424.72	23659.1	24639.08	46535.19	25429.74	36544.96	13619.18	48103.64	28882.99
32208.2	32627.07	24884.84	24152.3	47482.92	28091.4	34154.78	14917.1	43323.75	30921.64
33257.43	34311.52	21117.28	26252.65	40744.33	25905.67	32533.56	14524.83	45698.25	29565.76

LN379.198	LN379.198	LN379.198	LN379.198	LN379.198	LN379.198	LN379.198	LN379.198	LN379.198	LN379.198
20635.01	21816.63	34446.88	14844.79	34446.62	76841.14	16985.06	36713.56	46584.38	35401.77
22014.6	20127.55	40099.77	16123.32	31554.26	73001.11	17159.33	37267.54	48456.12	34658.77
20454.35	21241.49	40080.97	14585.22	33602.04	74450.32	19031.4	36772.85	52205.97	32369.33
23968.14	19914.03	41610.76	13767.2	32706.65	76876.8	19798.36	39685.95	45125.9	39339.85
23741.25	19993.78	35855.58	14724.09	32508.36	70071.05	19537.59	36334.49	45385.82	38705.39

LN379.198	LN379.198	LN379.198	LN379.198	LN379.198	LN379.198	LN379.198	LN379.198	LN379.198	LN379.198
49025.2	29956.25	24545.6	29603.5	24473.43	16975.63	46861.59	39293.22	23838.34	40732.11
48911.95	29257	24426.02	25733.05	26832.19	15067.83	47926.18	41302.11	22821.95	39942.23
50550.94	29059.1	24609.76	27855.32	25846.5	18285.63	46165.49	39850.38	23919.89	46808.78
46748.15	29999.85	27125.17	25136.99	25932.78	16281.18	48757.21	38211.68	27657.58	48376.36
51393.83	27493.67	25163.3	26757.25	27279.01	17924.28	47488.74	38399.92	22713.76	43832.55

LN379.198	LN379.198	LN379.198	LN379.198	LN379.198	LN379.198	LN379.197	LN379.198	LN379.198	LN379.198
37269.69	18267.8	36657.05	19952.62	45158.3	14179.27	76705.43	23130.48	40240.95	14973.9
37507.79	20759.17	34916.99	21199.6	44652.47	13278.1	74283.94	26504.43	40147.65	14537.54
37275.58	17373.2	36173.16	20965.42	44464	14879.17	76488.47	25488.31	39251.08	15022
37121.97	17240.32	39119.41	25912.49	45354.17	14544.73	77990.14	23703.09	38195.7	14240.87
37954.3	19468.23	37169.39	23712.1	47252.46	14254.61	79586.72	24222.92	39288.63	13775.8

LN379.198	LN379.198	LN379.198	LN379.198	LN379.198	LN379.198	LN379.198	LN379.198	LN379.198	LN379.198
15568.67	36646.17	23714.19	59690.28	64880.56	35906.77	28402.27	46375.1	37670.68	46623.64
16697.24	41579.56	23869.71	57372.42	65447.65	39031.48	28768.36	50538.64	36383.95	46415.76
16667.86	37663.95	25664.87	63547.7	71473.85	41271.93	30583.19	61254.31	41758.79	51629.1
14877.33	40041.93	27340.07	56885.39	71295.81	37818.42	29747.56	52818.44	37286.69	49529.66
17186.95	36662.4	21518.21	55702.45	72367.87	40703	30977.14	48439.12	35123.55	47686.69

LN379.198	LN379.197	LN379.267	LN379.268	LN379.268	LN379.268	LN379.268	LN379.268	LN379.268	LN379.268
38142.35	35668.81	67080.64	263451.2	243301.9	372222.3	280965	278519.2	191271.7	315226.3
42368.48	37058.79	73799.41	254030.3	422010.7	296178.4	255819.9	287265.4	207681.2	266251.1
41617.1	33461.85	69125.09	248109.5	231046.6	485901.7	297306.7	329524.9	211872.3	332379.8
36006.05	33156.65	68255.03	263526.7	249269	299573	291469.5	323509.7	208755	314681.9
40440.29	35682.76	77619.09	302326.7	252362.8	374875.8	288335.5	339191.3	212876	324243.2

LN379.268	LN379.268	LN379.268	LN379.268	LN379.268	LN379.268	LN379.268	LN379.268	LN380.978	LN380.978
153782.6	159880.4	148786.2	183307.3	91703.31	126278.8	124522.1	135886.6	42293.44	70058.12
151584.6	181158.8	147679.9	168976.9	96346.2	135781.9	130520.5	124724.8	49959.75	68834.1
152217	185409.9	154897.9	158189.9	94364.86	118950.8	136112.3	134129.7	45192.37	73029.34
164381.1	183740.4	153703.7	171932.3	95472.96	137590.4	129420.2	133163.8	49344.26	76867.16
159659.3	188552.5	149291.6	160606	88983.93	132221.5	128774.1	146210.9	49960.93	80546.24

LN380.978	LN380.978	LN380.997	LN381.035	LN381.035	LN381.105	LN381.178	LN381.232	LN382.994	LN382.995
42941.81	73898.39	22398.85	39381.58	51149.97	16106.52	55131.1	15967.85	46159.85	43746.56
43230.87	73069.12	22719.77	38540.09	47720.25	13689.84	46815.3	15106.98	55829.22	39032.52
43878.57	76179.4	22263.68	16789.29	17218.93	13993.6	43763.54	16641.23	46920.97	45921.83
42992.32	76435.9	22086	39555.16	52967.61	13502.27	46840	11502.73	63940.18	29265.85
41564.02	74649.63	22462.25	44491.5	49050.72	13927.62	45287.13	10347.2	47214.32	41085.84

LN382.995	LN382.994	LN382.994	LN382.994	LN382.995	LN382.995	LN382.994	LN382.995	LN382.995	LN382.995
41625.3	46640.91	62670.51	50353.87	33465.88	30682.35	59775.23	44352	45149.1	28791.59
50156.9	48386.99	62676.41	37231.23	34637.72	26866.57	67466.77	35004.35	53701.75	38951.45
43096.52	42344.71	58392.96	48652.08	35618.3	33123.91	59719.5	38684.17	47584.52	30902.56
50285.84	67908.03	66160.25	40078.27	39486.87	33881.84	61187.33	41955.74	47382.43	32996.23
36557.84	47658.63	57809.01	50295.23	37216.95	33083.43	62401.79	39454.26	42986.97	29982.7

LN382.995	LN382.995	LN382.995	LN382.995	LN382.995	LN382.995	LN382.995	LN382.995	LN382.995	LN382.994
19383.29	50910.65	54291.62	24029.07	17836.61	19744.48	52581.89	38123.31	31717.87	50762.95
17050.25	52071.06	59217.39	22665.03	19507.42	19654.37	50810.53	45406.53	27784.11	60127.88
17346.94	49750.03	50682.36	23427.05	15284.06	20085.32	45224.68	40397.33	28640.28	44159.35
20828.02	51641.35	65753.94	26321.97	15092.34	18565.47	75144.76	43108.4	31414.21	45862.6
17963.03	52148.93	48704.45	21662.92	13415.46	16800.23	44105.52	40400.9	28388.09	45847.78

LN382.995	LN382.995	LN382.995	LN382.995	LN382.995	LN382.994	LN382.995	LN382.995	LN382.994	LN382.995
36917.57	41318.79	27180.98	61169.86	32905	45315.27	37190.95	18148.24	49596.82	51049.13
54578.6	28584.54	30004.17	66793.98	32585.3	54023.66	34377.73	18939.81	69960.58	34592.3
45776.73	42303.05	27192.43	60369.44	32087.39	49628.78	39587.5	18950.66	49706.16	56527.87
44845.81	40961.49	33766.18	66399.19	31220.38	52326.55	37733.9	18422.45	64529.3	42656.25
46170.07	36402.26	27992.58	57074.96	31394.52	48012.35	34705.47	21361.55	46933.21	56678.01

LN382.995	LN382.994	LN382.995	LN382.995	LN382.995	LN382.994	LN382.995	LN382.994	LN382.994	LN382.994
28668.53	36268.76	26776.94	28875.86	20076.94	52775.38	16625.69	51556.65	43840.6	62707.07
26814.69	39230.21	25758.08	33253.15	15822.81	46917.24	14568.86	45247.68	35051.14	56378.35
28598.75	38342.15	28785.31	25568.48	17641.62	44630.65	19619.88	49898.11	44529.1	70394.61
31875.59	46461.85	28199.12	40446.54	15422.07	36770.72	18473.58	64468.93	35771.22	70717.75
26919.37	36191.59	29198.49	25517.96	19813.33	51716.37	17723.09	56320.76	40854.52	61498.12

LN382.994	LN382.995	LN382.995	LN382.995	LN382.994	LN382.994	LN382.995	LN382.995	LN382.995	LN382.995
60168.85	54994.7	37610.04	50221.61	58802.68	36929.24	49845.27	30968.63	30261.33	25346.09
63802.36	42843.21	44637.26	51279.03	64043.86	40994.82	53927.91	33640.86	35075.69	29997.08
69825.55	53896.18	34988.78	49858.31	60102.99	36300.07	44572.46	34137.35	34483.8	26236.51
63894.13	62282.19	45982.84	49596	65855.87	49076.73	48614.24	35659.24	24981.83	31111.38
61963.44	49632.94	38424.66	48124.86	60093.65	38804.34	46982.18	30321.66	34062.09	29639.88

LN382.995	LN382.995	LN382.995	LN382.995	LN382.995	LN382.995	LN382.995	LN382.995	LN382.995	LN382.994
19044.1	39073.32	25393.94	33611.33	48316.31	25547.28	21485.91	37677.31	20849.37	42866.88
24081.79	57273.66	34007.42	23059.03	50004.61	30263.16	22164.35	42222.59	19294.89	54063.51
19130.77	38006.3	26392.85	29309.66	46115.5	26363.42	20650.44	39239.1	19869.74	42413.32
24261.44	40214	29462.8	32613.9	62069.8	24680.78	15510.07	46546.86	20503.13	55294.88
17752.58	39454.17	26069.5	33209.29	47739.05	27847.36	18388.81	39008.22	21473.13	41107.79

LN382.995	LN382.995	LN382.995	LN382.994	LN382.995	LN382.995	LN382.995	LN382.994	LN382.995	LN382.995
25761.57	53486.33	17619.08	93352.32	31996.75	35186.18	26259.1	58085.29	38305.15	19719.35
40376.82	44816	15219.89	80213.75	27642.77	40700.2	34409.69	63952.81	37099.05	30579.19
26405.05	53757.79	16414.81	85679.76	29373.11	35649.09	28540.26	62337.11	36605.57	23150.53
43496.58	40150.3	16687.86	80724.76	35100.66	44484.2	26565.08	69857.29	40615.82	21979.82
25607.55	55329.91	15059.01	82825.1	29574.62	34545.87	27691.76	61915.46	36789.78	21906.23

LN382.995	LN382.995	LN382.995	LN382.995	LN382.995	LN382.995	LN382.995	LN382.994	LN382.995	LN382.995
28138.64	34292.43	14248.27	15164.56	19786.22	33353.7	24732.53	62406.57	30494.38	29533.9
25917.81	33289.17	18787.65	20379.29	22588.06	62367.61	27730.03	67164.69	36632.52	28967.01
32674.41	28323.6	15421.04	14628.38	18244.19	34129.88	29541.19	62001.86	28738.93	31631.77
29549.38	33988.59	15877.45	16601.83	17261.63	34020.35	23197.8	77129.67	33178.04	32901.32
30478.86	37531.42	13203.57	13874.66	17524.56	31979.07	24549.33	60738.87	28254.37	31158.11

LN382.995	LN382.995	LN382.994	LN382.995	LN382.994	LN384.845	LN384.862	LN384.862	LN384.862	LN384.862
48661.98	24495.47	76383.93	17940.78	55633.76	30072.13	221489.5	134510.8	153858.3	210206.9
36782.82	24853.37	76422.46	23564.71	58495.4	31277.71	215236.6	135759.2	142964.9	213314.8
41646.64	25566.1	78915.21	16621.77	59322.42	30004.01	197606.8	112935.1	141967.7	172894.2
54858.59	28128.94	102175.3	16799.81	56420.94	34891.57	178608.2	136951.1	126536.9	176896.1
41259.78	23789.07	74568.95	16915.95	55835.06	37115.68	186760.2	22837.15	116935.7	185569.6

LN384.862	LN384.862	LN384.862	LN384.862	LN384.862	LN384.862	LN384.862	LN384.862	LN384.862	LN384.862
69802.89	241436.3	194379.1	163428.4	230835.1	48961.2	227328.6	39554.09	22409.44	49317.3
70595.5	246499.8	211473.2	158716.9	206557.1	47847.57	217906.8	36999.21	23818.87	49211.83
69114.13	255557	183413.2	155407.6	183231.1	52354.49	216014.9	41228.41	21227.14	46528.15
62316.74	234804.4	182041.1	151949.2	209618.2	44631.55	204499.4	36249.72	22220.52	47340.66
67634.99	218781.4	191319	140624	175435.4	44084.91	204925.2	38728.14	19916.1	46235.38

LN384.862	LN384.862	LN384.862	LN384.862	LN384.862	LN384.862	LN384.862	LN384.863	LN384.863	LN384.863
43024.07	176975.6	159682.5	43252.34	66430.11	151778.1	40455.79	80668.9	89496.02	68330.21
41543.09	184785.6	161586.7	44310.43	67024.31	146472.9	43573.47	84585.67	88825.59	63193.31
44593.62	167339.4	151190.6	40376.83	60918.62	143271.9	42310.78	75499.11	88526.96	68313.3
47719.32	173123.8	169385.1	37087.59	56895.66	136329.2	37794.07	71778.55	89679.16	63921.81
38148.24	170484.3	150762	43904.32	54954.45	129793.7	36807.86	72299.17	82123.52	63286.4

LN384.935	LN384.935	LN384.935	LN384.935	LN384.935	LN384.935	LN384.935	LN384.935	LN384.935	LN384.935
324969.2	136646.7	458487.8	364123.7	183902.5	298230	255226.9	156161.8	437557.8	198143.5
329569.4	169783.5	332241.2	234572.8	142837.1	312029.5	344499	172205.8	392429.1	190436.6
306457.7	171454.5	410358.7	416033.8	179605	413916.9	330446.2	159718.6	495050.5	145518.2
341418.8	146781.9	444821.3	272085.7	149905.9	392350.4	369040.3	239321.4	574679.2	152008.4
292078.5	142950.4	230367.5	260275.4	186902	496185.7	227763.3	197543.1	526486.1	134585.5

LN384.935	LN384.935	LN384.935	LN384.935	LN384.935	LN384.935	LN384.935	LN384.935	LN384.935	LN384.935
614262.7	565405.3	537759.6	358658.6	434203.1	520682.8	427247.5	189913.1	499297.7	409608
488706.5	469089.7	498381.1	385915	499162.3	400227.6	431616.2	180776.6	364649.9	436577.4
395801	458841.4	420868.6	352289	454062.7	531367.3	385486.4	233762.3	439971.6	495588.2
482117.9	468006.5	497375.7	400754.6	628193.8	417553.7	305756.3	218354.2	421591.7	541380.7
466916.9	517637.2	477921.3	380900.1	413288.6	511398.8	372062	154269.3	414795	406359

LN384.935	LN384.935	LN384.935	LN384.935	LN384.935	LN384.935	LN384.935	LN384.935	LN384.935	LN384.935
401119.6	374281.1	340521.3	290679.2	331951	422957.3	414406.4	315535.7	249876.1	413881
401156.8	400400.1	357709.6	303898.2	432792.1	426395.7	526409.2	398824.4	200591.4	411069.2
417807.8	353804.9	420866.4	312470.1	376666.7	389212.9	368475.3	301339.6	274437.6	359747.5
390079.8	424149.7	374857	309278.6	280555.3	387452	402029.6	290469.4	309509.7	492001.9
388139.1	352495.8	414709.4	306980.5	394148.9	415560.7	284281.9	305998.3	251784.8	596750.7

LN384.935	LN384.935	LN384.935	LN384.935	LN384.935	LN384.935	LN384.935	LN384.935	LN384.935	LN384.935
491772	333315	486360.4	457931.4	304805.1	416200	310437	350662.1	254831.2	395199.3
335942.8	323510	407942.9	455288.1	373756.9	473724.4	453013.4	340539.5	227461.4	620746.3
401957.8	296103.4	600678.7	438595.7	405527.4	423161.5	273372.2	273768.7	263750	384032.1
409362.9	441059.3	391308.7	407211.9	432994.4	423772.4	271305.4	326356.1	231969.5	415148.5
574531.8	312453.7	498366.7	402036.1	361339	659100.4	296484.6	387561.6	224219.2	438433.3

LN384.935	LN384.935	LN384.935	LN384.935	LN384.935	LN384.935	LN384.935	LN384.935	LN384.935	LN384.935
353399.7	332531.5	461421.7	204991.2	332754.7	255573.9	237183.8	400069.2	308354.4	226306.6
295443.9	178066.5	356339.6	331808.2	365224.8	279777.8	285425.6	313588.5	263026.9	255396.7
289667.8	203734.5	523092.6	244705.8	408635.2	269230.5	234906.9	313553.5	318585.6	231168.2
266785.3	178225.8	332626.8	261656.4	307443.4	355192.4	282305.9	306845.5	395122.9	278576.4
242465.6	179890.8	508508.6	242814.7	303999.7	269630	378617.1	235056.2	434390.9	209987

LN384.935	LN384.935	LN384.935	LN384.935	LN384.935	LN384.935	LN384.935	LN384.935	LN384.935	LN384.935
336645.9	100315.2	221073	208924.8	426479.5	216475.5	214358.3	198245.3	324361.5	188709.5
276930.5	83637.56	249024.8	282078.3	459610	214408.7	221194.2	198873.5	367288.9	217501.8
300200.7	82886.12	174020.7	180134.1	358747.8	174991	209318.5	199637.4	318968.8	177114.4
271618.2	99897.56	163774.9	199517.8	363981.1	163906.7	278684.5	204828.2	335641.9	217980.2
250589.6	126835.9	225584	150311	363769.4	163645.9	304656.8	169140.4	395509.2	205982.1

LN384.935	LN384.935	LN384.935	LN384.935	LN384.935	LN384.935	LN384.935	LN384.935	LN384.935	LN384.935
209674.2	226745.2	201396.3	211859	199716.8	228282.7	183705.6	168153.5	184074.1	94701.62
159628.6	238671.3	202990.8	178482.6	226574.7	266127.3	173593.5	125152.3	213485	64209.71
175551.2	184451.4	192880.8	163045.8	243489.7	227749.1	208927.1	124220.6	226165.9	70270.84
134600.4	266929.2	202538.2	165313.7	170923.7	227162.9	147762.7	110288.9	257022	66965.56
175501.2	202913	152448.6	148354.8	227632.7	243478.9	237410.8	106092.3	230964	67327.1

LN384.935	LN384.935	LN384.935	LN384.935	LN384.935	LN384.934	LN384.935	LN384.935	LN384.935	LN384.935
58689.13	52475.41	35238.59	49358.25	52590.99	230969.4	72577.59	37796.13	24390.12	67654.54
70383.88	74341.86	26739.07	48717.23	50135.36	215190.1	55140.18	36823.56	20303.71	60767.6
61518.3	72220.68	35224.73	36268.96	61185.86	197017.1	60831.58	35154.12	23227.81	59545.86
62415.96	77455.76	32516.88	46811.59	53539.92	203898.2	59744	33710.33	22723.51	63318.5
72787.89	64376.6	31360.55	37570.2	54820.69	227941.4	61689.58	31992.18	22471.08	61428.54

LN385.190	LN385.844	LN386.194	LN386.471	LN386.840	LN387.025	LN387.026	LN387.025	LN387.026	LN387.025
115711.3	22878.82	18330.11	145622.4	253445	46595.73	30320.18	36982.74	28179.63	46978.55
130517.1	18577.36	17141.11	152328	253794.8	41244.57	31408.23	35740.08	27856.16	37716.08
117851.5	20141.23	18156.77	144716.2	248295.6	42836.23	30656.22	42295.56	30533.3	39170.65
117502.6	17272.32	16324.28	136022.4	295761.6	42467.16	29898.47	37748.13	29220.65	41584.82
97888.25	21120.2	16326.44	117683.3	328333.2	45443.09	29382.38	32899.17	30327.1	39827.73

LN387.025	LN387.025	LN387.026	LN387.026	LN387.025	LN387.025	LN387.025	LN387.026	LN387.025	LN387.026
35916.7	39797.99	31746.5	29930.66	41316	84817.81	28181.81	34473.16	33822.66	39694.71
38656.14	38452.67	28840.31	35251.82	39005.85	81225.03	28115.79	36844.59	35353.38	33234.32
36549	39441.09	28476.94	31532.25	41884.09	82136.95	27221.33	36173.4	35055.32	39256.37
38145.04	42499.99	29620.96	32357.03	39934.35	81494	27826.82	34778.24	33864.09	39365.77
36676.22	40855.78	25909.97	25441.57	40961.84	84189.69	26608.35	28460.85	31835.96	34486.89

LN387.025	LN387.026	LN387.026	LN387.026	LN387.025	LN387.025	LN387.025	LN387.025	LN387.025	LN387.025
47197.86	20345.42	31326.7	24025.26	52622.32	42844.23	45562.75	61746.79	35345.18	38944.56
48608.66	19189.41	30823.29	20638.79	55155.35	41519.22	45397.31	61137.29	35039.47	41957.02
49632.3	18316.36	29648.34	22192.99	53126.39	39092.68	44043.01	59717.74	33456.47	35915.75
47489.21	19132.26	29612.89	23748.05	57825.96	42690.31	45047.54	62998.19	32960.03	37915.57
47261.5	17995.94	26780.03	19823.19	49199.45	38375.06	49564.5	67022.87	31080	35547.32

LN387.025	LN387.026	LN387.026	LN387.025	LN387.025	LN387.025	LN387.025	LN387.025	LN387.025	LN387.025
57887.91	51412.65	37365.08	58677.3	37455.3	47834.13	33401.46	43345.99	56305.9	64169.3
55098.57	53734.9	38938.16	61310.06	37661.14	49293.41	34221.12	40191.48	50636.3	63321.98
54875.74	55305.19	38766.19	50605.27	35298.46	47699.37	34348.9	42684.06	51271.59	62092.5
53805.62	57627.22	41794.81	62243.4	32965.64	49662.91	34033.9	44212.39	54099.93	62003.99
48563.54	50227.25	37475.83	59721.07	33887.15	45591.36	32821.29	42229.32	54730.59	55484.92

LN387.026	LN387.026	LN387.025	LN387.025	LN387.026	LN387.026	LN387.026	LN387.026	LN387.026	LN387.026
37806.42	37995.68	38858.93	42951.84	33968.31	53650.28	31946.49	31771.68	46537.2	26040.24
37449.46	36458.49	41398.94	42869.43	35010.9	54026.59	33255.3	33097.89	48538.39	27205.35
36482.99	41677.36	40145.12	42104.22	34213.12	53295.04	30085.67	34552.47	48321.45	25514.12
40372.05	40406.46	42559.4	43433.65	31141.86	54713.34	29616.14	29447.14	49876.11	25655.44
32561.51	34377.22	40542.57	46438.86	29296.57	51868.95	28944.66	28077.15	41754.13	25134

LN387.026	LN387.026	LN387.026	LN387.025	LN387.026	LN387.150	LN387.151	LN387.202	LN387.215	LN387.842
31563.61	34925.44	43041.36	59545.66	34877.08	59793.71	108597.4	62714.4	17005.69	31338.31
32709.37	37534.83	43569.9	59211.74	33412.85	52698.87	114806.4	73174.69	17659.77	28653.99
30388.1	36543.95	38219.63	61972.54	34917.73	55781.06	117920.1	71479.79	19855.95	25782.95
29494.71	36018.28	39120.26	61356.26	33077.48	52866.87	111461.1	74447.33	17267.99	35944.06
29274.91	33267.69	41807.57	63914.74	31983.2	57059.34	110808.4	72153.16	16045.55	33544.27

LN388.841	LN388.873	LN388.984	LN389.004	LN389.005	LN389.004	LN389.005	LN389.004	LN389.004	LN389.004
95251.93	103659.2	71378.11	24559.81	19550.75	53697.67	29824.51	71242.62	37204.62	30975.06
88118.75	112280.5	68217.69	30771.22	24441.67	42052.11	22755.27	52473.23	36444.84	42893.18
93480.79	122833	72792.44	33824.29	24393.25	43352.4	22279.37	54125.49	35675.76	42144.21
97720.32	96964.5	68784.44	34902.61	22954.78	46932.72	20681.26	50458.48	35563.02	42035.65
115660.4	150881.3	70060.71	26061.12	27464.4	54878	18042.13	55391.22	42330.01	48461.01

LN389.004	LN389.004	LN389.005	LN389.004	LN389.005	LN389.005	LN389.004	LN389.005	LN389.005	LN389.005
32181.12	30936.7	15040.73	45195.21	35242.12	19179.93	10612.88	31176.53	24475.43	11236.54
30677.12	32274.16	18778.08	47840.61	31356.64	29556.95	15549.51	27019.66	24982.66	18706.76
29829.25	31492.92	17568.28	47089.06	31221.16	27971.98	14918.7	25842.2	26377.09	19707.87
31485.82	31705.62	18484.88	49453.63	32643.15	29284.25	16003.99	29339.68	27074.21	19173.99
32169.75	44939.44	12716.15	39605.82	36222.38	36796.47	13512.31	35666.58	21940.85	13060.28

LN389.005	LN389.005	LN389.004	LN389.005	LN389.004	LN389.004	LN389.005	LN389.005	LN389.004	LN389.005
35760.12	32994.97	39402.06	26210.52	36106.85	76761.77	19854.2	21903.46	69876.57	17301.48
22654.89	35457.34	40568.89	21532.48	44820.28	54167.93	17345.19	17178.82	61581.1	18423.14
22262.72	30791.69	39645.06	23895.54	47496.22	54415.58	14878.54	16744.25	57403.25	17496.03
25058.05	29297.23	39606.65	21606.92	45914.9	60189.37	16389.85	17474.14	64891.78	17551.51
21526.97	37616.17	40925.72	29160.92	42516.87	54763.99	16626.1	19256.11	75306.47	19872.22

LN389.004	LN389.005	LN389.004	LN389.005	LN389.004	LN389.005	LN389.004	LN389.005	LN389.005	LN389.005
28664.3	43866.18	40339.38	32147.38	49046.91	20034.2	35265.54	14977.15	21697.4	38094.22
33998.35	37232.78	35671.66	35867.48	43221.68	25717.3	39932.19	17151.4	25587.65	29907.25
36354.18	35633.68	34044.46	35052.41	45402.8	25724.1	36059.02	14584.83	21322.04	33724.7
36502.15	38101.88	34562.87	32790.28	44503.19	28709.84	37763.15	14270.25	21464.19	32411.92
37876.08	38047.57	52930.3	38373.09	53709.9	25631.19	44098.44	13723.47	24578.58	28168.97

LN389.005	LN389.004	LN389.005	LN389.004	LN389.004	LN389.004	LN389.004	LN389.005	LN389.004	LN389.004
31036.03	39104.67	30952.08	40877.29	30120.73	37935.32	32644.37	21408.78	38093.65	43493.02
27752.53	28310.86	28706.87	39975.38	32741.66	44594.74	38464.5	19171.09	37956.18	42179.78
29196.18	28358.24	27650.33	36460.39	34335.56	44088.03	36571.53	22305.32	39469.17	43618.13
25411.52	31621.51	28164.27	38252.61	34243.12	40643.1	35724	20657.62	42193.27	40767.19
35589.7	32951.47	31784.48	37905.19	26942.64	41956.16	32535.23	21002.61	40345.88	52692.54

LN389.005	LN389.004	LN389.005	LN389.004	LN389.005	LN389.005	LN389.004	LN389.005	LN389.004	LN389.005
13253.05	47471.36	14487.3	33015.91	35038.09	15750.61	30990.38	21377.89	37368.78	22364.61
18273.77	32241.7	14748.87	38264.54	33686.38	24887.45	36044.49	22042.07	38495.88	27918.05
17483.96	33460.99	15217.01	35317.22	38143.99	22083.52	36365.6	22613.9	39098.33	27862.24
14896.12	36863.91	14754.63	35871.07	33463.45	23198.54	31778.44	21023.44	39332.29	28910.37
20983.39	35114.28	13610.23	42576.67	32342.87	13592.9	53065.31	23473.27	50617.55	33149.77

LN389.004	LN389.004	LN389.004	LN389.004	LN389.005	LN389.004	LN389.005	LN389.005	LN389.005	LN389.005
32727.86	26941.35	31994.81	32173.42	16249.51	54149.74	23737.83	23054.38	33611.01	25791.16
36237.88	22701.06	36827.93	28205.56	13834.94	43674.7	26910.35	22742.41	18695.42	32597.94
39455.01	22264.61	33337.89	27548.98	12930.73	42101.93	25895.16	22187.22	21711.86	31249.12
38394.08	25160.64	34579.76	27994.94	14149.68	40434.2	25508.7	22770.81	20697.59	30750.86
33675.93	25004.04	35152.98	37160.35	16707.79	44662.53	25555.1	18374.85	30914.56	42345.82

LN389.004	LN389.004	LN389.005	LN389.004	LN389.004	LN389.005	LN389.004	LN389.004	LN389.005	LN389.005
41092.05	41742.65	41069.34	44325.31	52456.99	20865.27	35882.54	26229.73	27593.58	19563.17
45307.84	41940.76	37415.85	35489.05	44640.86	24075.82	38019.93	28561.01	27933.27	24830.79
48490.13	41782.31	36333.64	39873.25	38354.23	23226.33	37662.24	28081.31	28862.98	28375.24
45413.19	42167.05	36747.37	43358.92	43821.67	25861.27	33293.92	28539.87	26834.81	28433.51
46891.82	32388.26	35215.58	44393.95	36309.12	29913.86	33380.82	30567.97	34924.73	24046.26

LN389.005	LN389.004	LN389.005	LN389.004	LN389.005	LN389.859	LN390.837	LN390.870	LN391.000	LN391.001
62832.19	41454.96	34931.32	24940.71	27886.48	12075.1	30760.54	41559.01	208646.7	100442.6
51575.47	41704.87	30524.04	30116.86	31257.98	15923.86	24572.82	39827.34	149173.7	78715.12
47666.98	41804.49	28423.16	34739.33	31491.01	15285.23	22000.04	40273.87	141207.6	132194.2
51374.83	47574.81	28471.78	34274.41	30558.49	14828.4	20437.02	33068.66	172830.1	93692.15
47571.29	47592.73	25948.17	18965.37	31803.04	13563.71	22695.79	30177.57	133931.2	71922.17

LN391.001	LN391.001	LN391.001	LN391.001	LN391.000	LN391.001	LN391.001	LN391.001	LN391.001	LN391.001
119675.5	62545.52	56523.71	71321.26	152174.3	113739.4	58910.95	24179.08	66557.92	69520.21
79470.08	59789.87	60511.44	68060.73	143790.1	99300.47	78817.01	19125.69	81269.82	114330.8
68742.85	78568.24	64930.09	81457.56	142948.4	85410.68	63573.32	37056.64	71667.68	123548.6
60933.71	52085.39	74253	86121.11	147393.8	102470.3	71475.31	18526.67	91598.89	111286.9
67115.79	103668.5	70760.69	61006.02	138830.8	110246.4	47312.25	17050.34	75377.39	83904.13

LN391.001	LN391.001	LN391.001	LN391.000	LN391.001	LN391.001	LN391.001	LN391.001	LN391.000	LN391.001
64988.45	138330.3	112148.4	88822.23	91413.64	51869.23	112707.9	106654.2	172358.9	70872.94
40267.02	150548.6	122570.5	116794	213664.9	54486.34	124538.9	145008.4	122418.3	90462.61
64628.04	108145.8	91003.58	125246.8	89264.59	47556.62	118884.2	106416.2	128661.4	66528.74
40854.8	111505.5	108755.9	156600	89285.41	80072.26	104898.7	102440.2	125500.9	66865.26
51998.91	112628.1	84636.37	105935.9	104783.5	49986.39	100880.6	166081.4	102571.6	67254.12

LN391.001	LN391.001	LN391.001	LN391.001	LN391.001	LN391.001	LN391.000	LN391.001	LN391.001	LN391.001
91347.86	101589.1	42329.92	41329.73	112250.5	91168.21	146225.3	88470.3	100591.9	31693.82
119313.1	97760.02	39430.07	44402.13	108271.4	136090.3	174119.6	91027.4	103354	37080.42
104758.9	97259.02	32869.84	51166.55	95850.02	93508.46	114345	70741.41	96481.37	26552.64
116089.9	126838.5	38373.25	41541.47	88715.17	140036.9	133647.1	92361.38	84047.96	37094.35
94319.18	101743	33274.48	42051.35	133911.1	97456.52	161596.3	88315.61	91748.94	36116.79

LN391.000	LN391.001	LN391.001	LN391.001	LN391.001	LN391.001	LN391.000	LN391.001	LN391.001	LN391.001
181853.1	90133.58	143285.8	71335.46	67959.1	103574.1	154912.4	160815.6	99051.98	107288.1
133822.4	103620.3	86917.76	60770.8	51848.17	103055.8	162031.3	93009.16	129213.6	92739.07
185542.4	75712.11	95304.31	62922.02	78644.71	144710	170373.2	107032.6	113559.2	95076.1
119665.9	97567.91	132581.2	70940.3	59875.75	124491.9	158953	122152	107803.6	77795.42
195505.8	66718.9	94908.41	76377.45	65148.41	127660.8	144675.1	129594.6	158303.9	81060.04

LN391.001	LN391.001	LN391.001	LN391.001	LN391.001	LN391.001	LN391.001	LN391.001	LN391.000	LN391.001
40010.32	91716.49	96187.5	51124.45	30306.77	114089.9	62783.42	37696.66	146181.8	68710.66
46873.85	85935.45	115617.8	63582.74	37703.31	65239.29	85661.9	30368.94	147829.9	91414.02
56328.77	66356.99	112514.6	53552.59	31543.71	75236.32	61746.87	31508.26	160590.3	69766.59
41717.7	73918.16	121249.2	72976.75	37048.08	74385.81	61814.75	30983.54	122884.5	67262.87
51708.39	73938.99	97132.34	66543.85	36826.38	77513.26	75808.06	45155.8	192237.3	109766.2

LN391.001	LN391.001	LN391.001	LN391.001	LN391.000	LN391.000	LN391.001	LN391.001	LN391.000	LN391.001
40835.74	44901.45	58798.01	32316.01	223655.9	130816.6	132077.8	67781.54	142590	41457.15
30577.23	64501.02	51277.29	33591.89	144922.4	118524.9	103612	67967.19	133234.1	71599.67
54195.72	68769.74	73519.51	37142.71	113363.1	133270.7	111619.1	81511.06	112528.6	43531.8
38519.16	71357.3	48368.3	35549.31	165880.7	165915.1	81602.46	75468.64	97062.02	49284.37
41602.56	63657.02	68915.77	26905.15	146949.2	113446.3	114442	66706.52	110139.1	43303.8

LN391.000	LN391.001	LN391.001	LN391.001	LN391.001	LN391.001	LN391.001	LN391.001	LN391.001	LN391.001
128453.8	82009.31	30973.12	85713.64	79755.78	79370.22	104891.7	54344.03	44773.26	94506.42
94315.83	63813.72	49524.33	66934.21	113046.7	113496.1	84498.03	77090.47	37823.48	162095.8
102019.6	65066.31	33062.79	69996.97	85603.48	73661.2	85590.43	66311.16	41690.76	100243.9
128168.4	65624.31	34123.81	79203.05	89218.71	80719.65	70276.82	66484.38	32483.16	93959.47
126366.6	92979.22	28169.85	54112.47	92185.1	83126.54	90515.4	74051.72	32946.35	96381.84

LN391.001	LN391.000	LN391.001	LN391.001	LN391.001	LN391.001	LN391.001	LN391.001	LN391.001	LN391.001
91326.2	106860.5	59545.63	35308.78	73433.05	50763.79	90336.02	69669.09	94798.55	110628.9
91665.66	101098.2	62593.63	35253.56	69655.56	69340.88	93715.49	60990.35	98426.33	93990.1
87200.94	95559.96	56905.09	32724.93	59291.84	58493.32	82222.2	66924.37	119772.3	139837.8
78180.1	81848.16	64933.99	33815.18	76360.62	62987.04	97319.45	55283.64	112936.9	149845.4
74083.72	102087.8	55480.19	38347.66	49826.01	48038.55	73438.15	68970.35	83212.44	118340.7

LN391.000	LN391.001	LN391.001	LN391.134	LN391.160	LN391.160	LN391.160	LN391.160	LN391.160	LN391.160
102181.9	62192.33	38767.16	13366.97	55860.17	40936.44	47979.67	40840.15	33806.88	59484.01
103101.5	67009.73	38629.03	14953.69	54209.13	34411.58	49318.02	45187.22	39202.96	60344.85
102815.9	69742.72	30199.32	14376.7	64480.48	41109.42	45403.95	45141	38322.95	58945.23
135642	99996.32	38527.86	12691.66	60974.48	40584.53	47408.33	46347.98	38365.61	61316.98
92693.58	58724.22	28784.52	10282.33	27167.63	38016.74	50528.4	46439.92	35601.74	61509.26

LN391.160	LN391.197	LN391.197	LN391.197	LN391.197	LN391.197	LN391.197	LN391.197	LN391.197	LN391.197
44338.94	42697.93	49297.74	29889.29	46804.87	28404.57	37465.79	37846.46	62431.13	68835.34
53568.2	45670.44	44665.32	31525.71	46789.58	29950.33	33809.9	36977.27	58927.55	74892.92
52000.82	34843.12	43267.71	34354.03	51471.08	32509.16	42092.1	28336.71	74676.56	84530.96
56714.35	49791.78	50014.81	31898.25	41246.43	31521.02	36001	34688.32	57508.47	74292.45
45886.22	42219.56	48503.43	30230.72	42405.5	27728.19	36706.26	38718.45	62130.27	69644.19

LN391.197	LN391.197	LN391.197	LN391.197	LN391.197	LN391.197	LN391.197	LN391.197	LN391.197	LN391.197
49382.36	12000.03	23038.82	36208.11	33903.71	61727.9	46532.53	33718.78	42942.76	55959.91
46670.43	14768.24	23094.7	36516.29	34189.97	58082.86	46036.55	32962.45	39906.2	56580.74
47039.82	11663.97	30521.34	28541.58	32806.18	61580.97	48853.05	41918	35171.99	47145.26
49515.88	8247.86	22173.37	41119.71	29789.81	56134.19	44500.6	33620.99	42294.93	54502.77
46281.54	12668	24924.62	37273.88	28922.94	58087.36	42378.98	37332	40293.71	58607.89

LN391.196	LN391.197	LN391.197	LN391.197	LN391.197	LN391.197	LN391.197	LN391.197	LN391.197	LN391.197
47436.86	54944.89	27852.22	18297.15	27713.93	34683.95	60786.07	58705.28	38130.96	24518.77
45900.95	58623.73	32942.32	19899.13	27798.95	34538.34	63483.65	60861.12	39658.7	24723.79
50938.39	58651.5	27180.52	31506.04	22827.73	36430.75	58836.33	74589.83	42025.29	26074.78
49846.23	53857.63	30641.62	20388.94	23693.76	35447.5	63502.31	59282.86	34528.79	22582.95
52550.77	53247.22	32549.08	17978.95	25724.98	35021.63	72756.5	59967.13	37800.74	20606.5

LN391.197	LN391.197	LN391.197	LN391.197	LN391.197	LN391.197	LN391.197	LN391.197	LN391.197	LN391.197
55953.54	44122.48	42950.11	31751.11	40471.39	30081.58	26292.33	28617.38	24240.33	27026.18
56804.54	42570.75	41355.89	30624.06	42596.73	29413.79	27510.59	27977.09	30450.87	27224.26
59441.68	47264.06	44442.53	36968.49	45639.38	26164.01	37601.63	34997.94	21918.55	31354.96
61249.68	45489.61	41245.57	32327.25	40712.82	33135	26341.48	28482.61	26762.62	28176.95
54068.07	45222.92	34597.44	32729.11	38653.27	29304.72	23009.86	30518.49	28125.04	30776.43

LN391.197	LN391.197	LN391.197	LN391.197	LN391.197	LN391.197	LN391.197	LN391.197	LN391.197	LN391.197
48368.39	78558.23	30630.96	41388.19	42099.23	27857.63	28191.45	62171.38	74648.54	24315.21
50251.3	84027.11	27231.1	44369.01	46309.9	31383	27370.43	55845	80307.71	22469.02
44697.15	82504	33899.97	42940.36	49015.31	32460.44	27057.02	46608.97	74339.65	23733.07
46897.25	81480.78	29296.44	42504.83	41677.28	32527.65	24672.01	64919.62	75020.52	25096.29
47549.94	80359.52	31465.74	47568.18	39899.59	29044.18	28265.3	64783.99	69397.54	22626.45

LN391.197	LN391.197	LN391.197	LN391.197	LN391.197	LN391.197	LN391.197	LN391.197	LN391.197	LN391.197
35664.11	53716.5	39791.72	38931.31	29466.83	54951.08	33119.27	23925.02	70733.88	61252.14
30028.67	55666.87	33299.05	40336.67	33174.29	62984.71	32483.93	22510.46	76047.72	62182.52
32912.42	37713.6	40761.01	35848.6	45226.53	73740.64	41207.22	25415.73	76419.46	56667.57
33933.84	52622.98	34508.96	39367.88	30050.67	55543.51	33062.32	22681.38	74631.86	59727.12
32154.65	54962.34	37318.19	43661.11	33419.53	50694.41	35621.25	22890.3	65179.04	66199.31

LN391.197	LN391.197	LN391.197	LN391.197	LN391.197	LN391.197	LN391.197	LN391.197	LN391.197	LN391.197
67330.62	40681.52	61658.06	33122.98	52719.07	47732.7	26182.83	31080.47	47422.96	30124.6
60897.08	41709.43	55946.55	30700.43	55492.96	42961.27	23022.35	35473.56	45197.83	28837.7
62539.77	38721.66	87439.7	37189.7	53989.35	49157.76	24752.56	31714.82	38765.64	30216.61
67748.65	40045.06	61119.42	35866.18	54434.92	46151.87	26402.1	37629.46	46548.21	29079.64
64552.15	34946.71	59154.68	38054.29	51873.04	40830.81	26213.13	37780.19	44318.38	27719.55

LN391.197	LN391.197	LN391.197	LN391.197	LN391.197	LN391.197	LN391.197	LN391.197	LN391.197	LN391.197
32965.4	42322.52	13561.8	19129.46	38865.25	35950.11	15009.86	45976.27	31243.22	14658.33
33737.73	39329.47	15424.54	19569.17	36668.34	33851.64	20166.82	42660.4	34342.77	15429.07
30456.51	37080.17	19816.69	17751.67	37372.37	24418.91	13956.71	47673.7	32803.11	18500.29
31749.89	39533.95	13856.84	20106.76	36420.42	37766.62	15192.07	49203.91	31783.25	16150.54
30448.77	43156.12	14040.95	19535.02	35842.09	31619.03	14772.36	47564.74	30840.62	14758.64

LN391.197	LN391.197	LN391.197	LN391.197	LN391.197	LN391.197	LN391.197	LN391.197	LN391.197	LN391.198
18426.58	44244.52	16281.95	18815.78	39590.68	18839.51	15286.49	21614.7	28492.9	26451.07
14618.78	42351.56	18907.21	17295.15	38532.78	17419.87	18243.42	22127.18	27613.86	25187.26
15520.79	52166.01	17074.72	25968.66	46528.28	16400.15	16315.01	22311.42	32138.17	30534.11
14355.43	42493.85	16251.59	17182.82	37853.58	18439.03	18724.3	20751.17	29905.57	26948.74
15456.1	39272.53	17917.32	16375.71	41846.89	19103.11	17671.56	20608.91	32966.82	27858.45

LN391.208	LN392.211	LN393.176	LN393.277	LN394.262	LN394.262	LN394.262	LN394.262	LN394.817	LN394.890
190807	30170.07	32868.51	73705.71	14806.28	18960.83	15261.69	15331.38	52220.81	62524.7
200104.1	32102.04	26991.32	79302.18	16343.35	20379.79	13412.75	16175.06	40054.65	88336.26
200804	32984.6	31993.73	82812.67	14571.59	19651.86	11675.93	14728.8	45557.27	89572.71
163704.4	25931.15	33527.18	86426.66	13937.89	18401.55	12842.6	15567.55	76923.85	71405.58
175029.7	28868.67	32002.27	83546.19	16786.09	19336.57	16985.3	15484.67	74351.43	71983.32

LN394.890	LN394.890	LN394.890	LN394.890	LN394.891	LN395.192	LN395.192	LN395.192	LN395.192	LN395.192
108585.5	91248.95	109603.6	170270.1	125426.8	61736.25	59405.97	32435.99	71994.17	10364.82
116507.8	100446.3	112587.6	195822.2	122592.8	52121.44	61491.84	46820.3	55916.06	14902.68
125730.6	96684.66	146248.8	158097.8	133391.9	49497.08	64544.75	43029.12	52407.03	12562.86
111471.5	94907.97	111113.2	168059.3	123432.7	53123.48	64483.06	39755.07	43370.69	11211.89
114585.1	87659.17	99884.23	161670.5	126729.5	51542.3	61722.12	32615.81	55389.76	14116.3

LN395.192	LN395.192	LN395.192	LN395.192	LN395.192	LN395.192	LN395.192	LN395.192	LN395.192	LN395.192
79952.74	61672.07	85031.87	59091.68	64846.87	27984.36	70410	66638.88	77145.81	71559.94
69612.87	52268.42	62354.87	79348.63	106462.8	37862.53	50346.19	60931.97	73473.29	41337.12
75396.58	49293.97	53487.86	54120.39	91310.25	29974.9	65354.83	52184.7	76762.45	51699.97
77358.02	54026.96	52034.1	55748.13	85253.54	33189.66	66763.06	52055.54	73900.61	53948.36
70423.46	47913.12	55236.08	55668.76	91077.54	30395.05	66443.55	54469.29	70089.76	52089.79

LN395.192	LN395.192	LN395.192	LN395.192	LN395.192	LN395.192	LN395.192	LN395.192	LN395.192	LN395.192
29165.93	52034.54	66759.23	73080.95	52955.11	53010.26	39590.91	32874.41	31016.38	45374.41
39342.84	59752.91	73584.4	74952.28	40063.64	59866.99	45301.99	25695.49	32250.68	46403.08
27951.53	54787.89	63407.54	90318.24	45001.87	47356.04	45188.11	30047.26	30222.36	35999.22
28973.87	57342.06	55578.71	87636.77	45087.24	48660.28	46568.54	28079.27	28040.9	39629.86
28441.61	53193.16	63811.14	85597.25	47554.13	45849.09	46644.26	27964.44	28708.79	37535.79

LN395.192	LN395.192	LN395.192	LN395.192	LN395.192	LN395.192	LN395.192	LN395.192	LN395.192	LN395.192
52919.8	31855.13	32713.21	31527.86	43023.91	26574.76	28168.19	71462.7	50192.66	29003.18
55075.79	45047.4	46908.39	28406.37	54397.53	33841.93	24613.62	59126.97	43055.77	34600.44
54133.24	28066.98	45884.05	31036.44	49839.37	27861.42	26748.41	71407.71	55087.91	32906.76
56668.33	27320.16	48328.37	26990.75	49566.31	31857.92	24553.58	74903.53	51270.4	32289.97
49934	28736.74	39981.4	30374.44	49679.38	30747.39	23208.52	71403.32	56207.24	31608.42

LN395.192	LN395.192	LN395.192	LN395.192	LN395.192	LN395.192	LN395.193	LN395.192	LN395.192	LN395.192
36065.72	20077.16	31263.69	21218.36	52248.56	52699.83	34896.72	30979.26	40708.82	39447.46
47589.27	24708.84	29757.33	18403.36	51816.5	39783.81	46116.41	37202.97	36388.56	26066.02
40998.73	22162.86	33230	17116.68	51064.58	37851.57	34706.27	39811.71	44679.02	26719.86
40478.99	23732.61	30529.96	17476.94	51252.69	34691.52	32618.75	40632.69	42954.68	28573.05
43177.36	24230.69	34391.74	18720.54	49116.37	37831.11	34535.12	44023.03	41317.37	26756.06

LN395.192	LN395.192	LN395.192	LN395.192	LN395.192	LN395.192	LN395.192	LN395.192	LN395.192	LN395.192
30842.82	29279.44	44792.26	33761.77	50466.99	21189.78	17014.42	44216.95	15954.46	28115.04
35962.84	26358.36	36462.81	36068.75	52643.27	18957.91	18965.2	31212.86	22086.38	24912.32
34928.41	26803.78	44175.96	33360.45	28722.32	18928.44	19460	37985.53	15157.16	27858.34
34300.54	26672.1	45541.72	33403.24	34426.36	22699.22	20425.96	37300.14	14765.43	32920.38
33824.48	29113.42	36341.49	36641.19	29362.61	17502.99	18688.79	34510.29	15200.56	32408.87

LN395.192	LN395.192	LN395.192	LN395.192	LN395.192	LN395.192	LN395.192	LN395.192	LN395.192	LN395.192
45067.62	55598.44	48557.68	46555.91	63545.17	32469.26	46350.07	28054.46	36326.96	47157.25
55235.66	59850.46	66179.63	75391.81	64069.07	32201.34	52846.44	31450.86	69600.1	44699.22
43820.4	44809.71	44037.94	52330.97	50522.72	20087.81	52795.29	28220.43	33937.93	41840.25
43798.61	42350.25	45913.08	54524.78	50614.08	17875.35	54024.51	32579.01	38763.1	45899.42
42754.04	46530.1	46218.39	47137.4	50760.47	19810.09	50179.73	28999.1	36746.66	46313.59

LN395.192	LN395.192	LN395.192	LN395.192	LN395.192	LN395.192	LN395.192	LN395.192	LN395.192	LN395.192
30241.34	21574.53	20602.79	60129.77	45022.64	18209.55	35218.44	38002.01	39436.91	31246.18
31016.84	17873.15	17133.53	55316.4	37130.26	21802.99	67979.4	23857.27	33797.88	35821.95
28054.61	19068.47	20034.85	52715.74	39150.44	20110.71	44039.39	24587.63	32554.16	38939.04
29903.54	20242.33	20226.67	57106.05	36034.47	21406.26	45289.74	21947.51	31036.89	38398.02
31162.02	21426.45	18901.83	57944.27	40492.11	18775.61	46054.07	22285.94	36447.44	38560.62

LN395.192	LN395.192	LN395.192	LN395.192	LN395.192	LN395.192	LN395.192	LN395.192	LN395.192	LN395.193
29769.11	27908.71	34645.63	16718.68	32002.61	17042.39	42927.8	48314.15	24264.59	26646.46
23610.64	27589.01	43050	10859.44	33220.8	17394.39	42619.72	49586.26	26020.18	28012.96
26220.68	29187.05	37354.25	16942.11	32794.72	16855.21	41985.73	64657.07	27352.99	27811.95
27856.7	29801.36	36582.14	20852.66	34444.38	17347.36	40259.67	67350.84	28537.17	27624.41
26610.9	26888.82	35452.52	18515.29	33157.7	17082.45	41547.23	62470.53	28218.12	26880.24

LN395.193	LN395.193	LN395.193	LN395.193	LN395.192	LN395.192	LN395.193	LN395.275	LN396.815	LN396.991
26567.39	35188.04	41617.02	46939.81	43395.91	52369.92	42961.59	24281.07	24671.28	16857.83
23865.98	35292.56	44442.37	41556.48	41419.18	51469.02	45351.41	24876.15	21276.38	16817.3
25933.76	36464.46	45338.09	50001.56	43429.31	55612.71	46199.12	23307.43	26602.85	17907.39
22874.26	38119.54	43975.34	39983.43	40944.52	54941.36	43452.99	28737.26	17396.16	15918.34
27881.1	38051.02	42005.58	44416.17	40741.69	51597.47	42580.66	23399.11	21651.28	17303.73

LN396.991	LN396.991	LN396.991	LN396.991	LN396.991	LN396.991	LN396.991	LN396.991	LN396.991	LN396.991
14460.91	40346.1	30295.24	27916.09	32924.34	25303.3	32485.79	27945.01	36393.33	41557.35
14940.33	41781.49	30010.55	24497.11	27767.96	26086.13	30970.86	28579.57	39407.75	36352.54
13483.7	41035.32	29113.71	25887.72	28443.93	26383.76	32574.09	28437.35	45122.84	39462.49
14886.3	38985.58	30224.41	26152.59	29216.52	23185.86	32305.98	26284.62	43893.44	39107.44
15095.17	40365.45	31120.33	26174.19	29684.09	26514.64	29925.1	30274.68	39408.84	34675.38

LN396.991	LN396.991	LN396.991	LN396.991	LN396.991	LN396.991	LN396.991	LN396.991	LN396.991	LN396.991
47552.14	19552.67	21866.14	38051.82	31011.08	15568.3	27293.29	35760.78	30520.86	25880.09
46684.57	21023.58	22115.53	37643.26	32030.04	15492.76	28353.71	37985.17	33098.06	22805.48
51014.56	20633.02	21106.46	32229	35010.88	15167.08	28694.85	33904.42	33321.67	23868.04
51801.64	18593.32	23180.61	35483.09	33500.75	15105.33	26249.9	34553.17	33111.78	25335.4
49831.07	19984.92	21631.49	36275.57	30324.45	14334.11	26105.39	34332.09	33942.41	24554.96

LN396.991	LN396.991	LN396.991	LN396.991	LN396.991	LN396.991	LN396.991	LN396.991	LN396.991	LN396.991
26524.88	23904.64	27485.73	24853.9	33671.15	50370.59	30460.73	29047.53	19195.67	19487.69
22693.09	26693.89	23161.45	21901.89	35687.74	53564.95	32744.81	28582.59	17264.09	20101.89
26316.59	25674.06	25229.23	24701.28	36260.59	51516.67	30909.58	25948.2	18091.01	21038.95
25569.6	27642.4	25947.66	22692.02	36917.99	51354.31	29804.89	27784.84	18307.71	20449.24
27427.07	26627.99	23581.51	22450.86	36079.62	51657.92	29959.09	25670.02	16236.63	20915.9

LN396.991	LN396.991	LN396.991	LN396.991	LN396.991	LN396.991	LN396.991	LN396.991	LN396.991	LN396.990
31407.1	28748.02	26423.23	26846.98	40260.51	34969.31	42101.5	12787.53	26580.87	28013.02
33140.17	26648.45	23433.03	25559.92	41495.83	32807.75	41309.51	13046.27	26229.31	24937.86
32359.54	26221.19	24597.99	26804.29	43923.77	34043.25	42733.93	12089.82	25251.31	26579.75
30223.01	26598.03	26148.96	26037.16	39063.21	31233.11	42659.04	11848.02	24968.36	25802.28
33344.54	26694.89	26282.06	27876.52	42457.62	33831.72	43049.57	13065.87	24081.24	26419.11

LN396.990	LN396.991	LN396.991	LN396.991	LN396.991	LN396.991	LN396.991	LN396.991	LN396.991	LN396.991
47280.43	39421.7	19197.51	54304.84	18417.09	24788.53	19428.63	22799.92	20999.87	26896.33
45524.57	36737.33	21135.49	55140.78	18399.16	22387.24	18726.01	26464.74	22048.52	30640.42
43864.07	41220.09	18400.75	50938.94	17949.11	24149.42	20594.9	27988.64	22746.47	27744.62
46540.4	35384.1	18482.25	53840.02	17945.81	22429.43	19131.91	23610.79	20880.78	28364.83
46847	34851.77	20921.26	56592.8	16640.03	21305.72	20664.45	24047.32	22751.2	26865.27

LN396.991	LN396.991	LN396.991	LN396.991	LN396.991	LN396.991	LN396.991	LN396.991	LN396.991	LN396.992
25783.67	36346.11	22689.6	37092.13	35875.23	56234.84	32661.56	29428.02	21214.16	16093.64
25362.05	42433.55	20852.32	35092.49	32407.59	51603.83	34349.04	29694.97	20902.24	17508.81
23750.71	36596.66	20906.34	37359.56	34381.77	53815.1	30876.68	25803.13	18924.22	16059.93
25962.48	37081.77	22410.64	39014.51	34273.04	54212.04	32411.2	25256.05	20570.33	16673.72
25136.44	37899.97	21382.66	35877.65	33916.57	49678.03	33410.11	27157.59	18526.33	13762.25

LN396.991	LN396.991	LN396.991	LN396.991	LN396.991	LN396.991	LN396.991	LN396.991	LN396.991	LN396.991
17577.57	48077.74	20677.44	13453.85	14740.45	23500.15	39126.78	35425.26	21666.78	25922.48
17150.27	47492.94	21584.83	12320.69	14086.47	24639.18	41302.61	31719.21	20927.32	25036.38
18108.89	43573.53	21193.48	13664.74	14931.36	24994.75	38386.49	31455.62	21316.04	26799.16
18414.2	44721.68	21272.78	11369.74	13594.33	26976	43020.37	32991.67	20928.46	24179.63
16964.24	46156.94	23372.85	11199.3	16561.16	25392.35	38366.42	30930.98	20180.46	25663.79

LN396.991	LN396.991	LN396.991	LN396.991	LN396.991	LN396.991	LN396.991	LN396.991	LN396.991	LN396.991
29150.27	25863.33	38566.97	32770.81	22500.86	30968.01	20242.73	41567.35	34584.58	92031.25
31319.79	22502.07	36197.78	32516.66	21797.34	32078.15	19924.84	42571.37	35250.76	89800.78
32971.94	27235.99	29928.74	32825.2	21596.83	31164.43	17532.95	39769.98	36868.37	86100.41
34024.6	26724.03	34812.86	32651.9	20874.53	32110.15	21378.47	40633.14	34921.08	90834.94
29751.39	24152.54	34017.84	30526.1	20141.96	29623.99	20223.35	41021.94	34104.24	86961.25

LN396.991	LN396.991	LN396.991	LN396.992	LN396.991	LN396.991	LN396.991	LN397.244	LN398.833	LN401.004
44506.56	55868.42	39376.89	14835.51	35200.52	39041.72	38836.76	56035.46	20411.17	37369.31
47697.64	60090.86	37973.83	15471.04	36118.62	39554.36	36428.1	54354.58	20692.03	21095.06
42776.39	58521.58	38875.76	14289.69	34147.8	41762.39	39600.13	55607.61	21961.6	33110.71
46712.78	56449.35	39077.77	13363.15	35890.74	35528.24	40266.74	41005.42	19563.47	28065.72
45587.24	51185.05	38111.57	13070.01	35296.44	39051.5	34577.09	49710.81	21708.95	24683.88

LN401.003	LN401.004	LN401.004	LN401.004	LN401.004	LN401.004	LN401.004	LN401.004	LN401.004	LN401.004
43067.36	11403.27	27963.24	40205.19	44851.19	34147.62	68345.85	71035.45	42645.31	22773.79
33684.56	13725.4	32126.66	53333.35	41489.88	50096.67	49464.34	45226.63	65042.18	30567.52
44213.58	14306.12	30836.52	29003.95	34395.35	42851.65	58461.9	34240.2	46231.92	29527.14
57825.67	12071.16	34402.24	52025.52	40166.35	42372.8	62540.83	39222.71	48285.37	33877.23
38731.26	15150.38	30472.47	43291.84	34284.87	37190.78	50183.99	36194.26	46588.71	25950.64

LN401.004	LN401.004	LN401.004	LN401.004	LN401.004	LN401.004	LN401.004	LN401.004	LN401.004	LN401.004
45123.01	42468.98	48318.6	29469.31	14031.61	75609.23	54261.52	43813.18	40380.17	20164.94
30352.37	40555.13	45213.09	31016.98	20181.84	56706.74	57015.67	26880.7	46913.22	26932.92
44198.99	54280.25	96448.06	33303.49	14428.45	52954	44340.79	32010.64	50579.27	29118.45
35249.22	51831	55505.41	44670.24	23311.6	40447.45	48775.43	28289.86	37948.1	19760.5
40592.74	40666.67	47615.17	40316.03	22296.41	47471.29	53562.56	34389.4	43981.12	22366.24

LN401.004	LN401.004	LN401.004	LN401.004	LN401.004	LN401.004	LN401.004	LN401.004	LN401.004	LN401.004
38001.25	54371.63	37188.87	28444.17	45098.09	45968.95	46284.95	90230.35	31669.53	40686.19
26740.27	54472.66	38344.26	35839.14	42627.81	48149.57	40426.21	73494.79	55897.8	34516.14
19845.45	44982.99	44165.05	39013.8	40527.99	56295.67	64340.6	72142.23	53937.15	28097.28
19202.17	75372.57	37409.11	34551.45	39315.93	38791.16	58149.06	68106.02	49356.8	39296.94
23473.1	43296.81	41534.76	34424.99	34777.74	37262.63	46046.71	61372.82	44314.75	37957.01

LN401.004	LN401.004	LN401.004	LN401.004	LN401.004	LN401.004	LN401.004	LN401.004	LN401.004	LN401.004
18912.77	52940.72	73632.59	28816.58	42653.2	27993.89	28594.94	26541.83	36223.54	58006.07
21148.44	40255.73	70107.78	29275.21	48175.48	23579.5	31145.06	32183.51	57049.97	52769.81
18151.43	51534.78	66883.18	29126.84	44335.98	22427.29	21908.78	32316.38	43478.6	77660.26
22210.45	44139.3	82893.51	38879.94	40106.33	37099.65	33766.23	33020.19	42937.38	50445.54
21225.49	46753.56	64778.22	35517.69	34615.28	25451.26	28520.61	31391.56	38887.26	57253.07

LN401.004	LN401.004	LN401.004	LN401.004	LN401.004	LN401.004	LN401.004	LN401.004	LN401.004	LN401.004
14974.41	58443.19	30623.36	38483.71	65067.29	66656.95	58297.75	13303.95	33087.84	32346.44
13015.53	86289.93	35017.44	56852.47	51630.08	84876.02	53015.74	17822.97	40564.78	33089.09
23102.61	111334.6	33453.64	52753.83	64206.37	62611.43	41708.66	16063.28	31066.18	38126.07
20410.33	43353.59	32821.73	46571.42	57535.16	72494.54	50675.24	16535.75	34401.12	33871.23
21222.96	56604.91	31110.46	45464.42	71474.97	64626.31	48734.94	17278.1	36241.17	34882.26

LN401.004	LN401.004	LN401.004	LN401.004	LN401.004	LN401.004	LN401.004	LN401.004	LN401.004	LN401.004
50517.45	51114.72	23780.41	56477.02	39462.18	18317.76	38142.82	28308.81	10232.26	36722.32
60788.92	45878.08	24794.33	31536.12	44896.03	24791.79	47960.23	31033.31	16525.78	52899.71
60453.86	62114.38	38469.4	25943.96	56157.29	23768.33	49840.9	42459.04	19791.11	42882.35
51220.78	41013.5	29042.1	45160.96	52593.21	22284.82	50559.94	29228.2	13599.95	52364.07
46085.19	44878.09	26063.63	34644.17	49391.84	27096.81	44101.95	26877.61	13567.37	41797.6

LN401.004	LN401.004	LN401.004	LN401.004	LN401.004	LN401.004	LN401.004	LN401.004	LN401.004	LN401.004
26615.19	33153.49	56424.1	20514.58	23923.58	15065.87	37114.31	35161.95	75641.3	46547.76
38063.97	36507.98	56836.42	24044.89	34777.76	19309.96	31496.23	26861.8	72122.07	59061.61
30912.13	31805.24	55579.94	25604.41	24429.28	12918.95	25346.72	24163.65	63710.55	36751.96
30541.02	38446.55	58199.99	16071.16	22573.57	14554.55	26475.7	23093.43	52682.25	36220.04
32406.33	25742.41	63943.2	19179.69	28353.48	18446.93	32232.31	24479.68	46680.73	34880.22

LN401.004	LN401.004	LN401.004	LN401.004	LN401.004	LN401.004	LN401.004	LN401.004	LN401.004	LN401.004
17015.53	20468.66	43754.97	50904.58	42271.5	22906.81	16894.7	44832.94	33966.03	34721.8
14217.55	28656.61	32150.64	42713.09	55310.72	26517.38	17017.36	65337.33	35173.51	25012.31
13624.11	42750.97	45686.03	56985.78	46324.04	20290.34	16488.11	44337.07	46934.19	29571.4
11722.33	23544	38773.01	49859.5	45592.14	33514.15	18621.04	46393.99	34272.82	26201.16
17286.01	35776.94	35398.32	42534.04	47816	24310.19	16138.15	44109.97	39264.67	32943.59

LN401.004	LN401.004	LN401.004	LN401.163	LN402.166	LN402.850	LN402.984	LN403.000	LN403.000	LN403.000
34278.43	52321.31	45742.55	82188.98	14039.31	63591.37	18097.96	35376.59	50752.34	59840.98
31658.22	31869.67	47579.34	77408.36	15192.5	78509.79	16691.93	41595.65	69892.97	53104.89
29624.1	45776.55	48380.91	86486.9	16750.51	68592.22	17071.09	38845.06	67676.18	50853.05
28407.14	42105.95	34489.31	73322.76	13538.39	90771.12	18800.28	35860.4	68983.81	47596.39
36865.4	40546.41	52993.34	72727.76	14783.79	92663.52	16038.67	38184.77	70526.23	50513.01

LN403.001	LN403.001	LN403.001	LN403.001	LN403.001	LN403.001	LN403.001	LN403.001	LN403.001	LN403.000
38460.54	18621.92	41917.23	39377.73	39114.57	25714.97	14251.61	63324.61	49273.23	57734.85
30786.49	14652.05	37150.95	51409.32	36632.12	29849.39	17960.67	50961.31	47496.1	57322.09
28540.2	13917.95	34724.92	49151.04	34760.6	31376.12	16118.27	52627.25	51774.89	61898.12
27689.65	15402.93	35334.22	49185.23	34650.82	32677.32	15515.14	46463.27	48683.29	57296.06
30731.7	15538.3	31330.73	51510.27	34808.23	32164.41	16257.97	42337.61	45001.41	59148.5

LN403.001	LN403.001	LN403.001	LN403.001	LN403.001	LN403.000	LN403.001	LN403.001	LN403.000	LN403.001
50209.44	37029.96	39996.15	21429.67	67027.22	51635.43	14557.75	36824.86	71010.66	28352.05
52601.9	26465.89	38742.35	16685.25	50975.45	69164.81	15638.61	33992.03	61958.86	26612.64
51108	28645.01	40198.71	15655	50492.95	67789.89	17965.83	32102.13	58004.47	28375.24
46314.3	27219.31	41758.1	16553.78	47021.64	71242.62	16098.3	35339	65431.44	32062.99
47448.61	27845.88	42851.26	16552.1	46785.05	73316.28	15067.5	30879.49	59297.45	29272.8

LN403.001	LN403.001	LN403.001	LN403.000	LN403.001	LN403.000	LN403.001	LN403.001	LN403.001	LN403.001
39216.23	16664.62	32710.1	44854.68	30640.67	50764.93	44804.64	52642	29132.95	32102.92
24928.12	14781.54	26461.35	37743.18	34428.73	50209.15	37206.69	50465.92	18693.04	49963.39
26053.08	14964.44	23126.46	44906.84	34739.09	55753.4	44746.35	52673.11	21409.55	46115.37
25237.48	17561.03	24712.88	45769.42	30851.72	52162.31	43448.9	49564.12	16070.9	47432.99
28139.5	15577.97	24847.36	42550.82	32559.19	49029.94	38660.48	50164.58	17151.09	47032.94

LN403.001	LN403.001	LN403.001	LN403.001	LN403.001	LN403.001	LN403.001	LN403.001	LN403.001	LN403.001
60542.08	55188.22	57865.52	22053.34	34807.31	53370.61	29699.39	35312.65	45096.3	36609.94
50490.55	47786.36	41295.14	24938.82	31820.29	42091.44	36610.63	33594.7	38434.04	30045.76
53469.32	46390.01	41317.52	24108.62	28283.48	42527.93	34999.9	31705.11	33541.87	32328.34
51076.82	44119.42	41205.16	24036.09	32627.06	39402.88	34600.08	32452.15	37631.67	36365.08
46705.74	45208.59	39066.37	22852.15	28351.42	44230.68	33713.46	34054.59	36314.93	33053.71

LN403.001	LN403.001	LN403.001	LN403.001	LN403.001	LN403.001	LN403.001	LN403.001	LN403.000	LN403.001
22152.01	26186.53	17379.21	54533.59	52543.05	21463.43	35362.32	34813.39	59018.57	31418.86
16272.72	20780.97	20740.99	41459.45	34510.87	34947.91	59624.22	31556.09	54884.25	23726.31
14395.56	21339.37	20777.68	45611.94	36374.17	32209.08	53285.19	32946.16	56158.43	25430.02
14698.58	23832.47	20795.36	45428.17	35762.41	33083.68	66614.26	31653.63	49953.52	26074.7
10583.63	24990.22	21739.96	47588.11	36955.51	33599.63	58319.03	31400.86	54976.54	27147.94

LN403.001	LN403.001	LN403.001	LN403.001	LN403.001	LN403.001	LN403.001	LN403.001	LN403.001	LN403.001
40117.16	42031.54	31988.18	22685.23	35107.45	47393.6	30635.81	52802.71	39681.31	26694.23
41580.44	41050.63	18816.54	24047.63	29931.9	35090.3	37717.8	47087.4	39946.64	23403.49
42238.56	36421.01	19297.71	22751.5	36122.34	35016.14	38030.93	52949.07	38438.88	25297.04
45175.99	34969.7	18479.27	25122.91	31555.55	42904.07	35591.08	44194.54	42660.08	25380.8
38853.12	35813.31	19430.29	22584.91	31979.42	36954.49	36361.69	50674.1	36603.19	23236.21

LN403.001	LN403.001	LN403.001	LN403.001	LN403.001	LN403.001	LN403.001	LN403.001	LN403.001	LN403.001
38492.81	47631.12	31582.85	36625.96	28808.41	33962.13	32980.05	24209.67	35660	19058.69
44059.15	36792.09	34133.13	40132.81	24595.77	34790.37	30188.76	26481.84	34577.6	17685.62
47208.61	38263.04	34686.78	45300.06	22987.72	29593.12	33142.48	28265.17	30602.19	17673.15
47052.56	33999.48	29524.02	47574.44	23857.2	33664.03	33528.77	27257.19	33539.62	16118.74
52914.92	35785.67	31892.81	47420.09	26708.36	30968.73	32212.98	26033.04	34323.89	17356.11

LN403.001	LN403.001	LN403.001	LN403.000	LN403.001	LN403.001	LN403.000	LN403.001	LN403.001	LN403.001
20836.25	39134.16	42262.07	65615.73	19970.58	58861.62	64354.35	30618.16	41830.01	23690.86
18963.57	38972.87	37687.05	65644.67	26501.98	45854.57	54235.02	31264.06	38291	26766.51
18588.88	39592.02	38517.87	64667.81	25841.26	43092.55	60940.26	30642.84	39167	25662.74
16960.49	39815.76	36276.37	59308.79	25416.21	45565.83	58260.41	31173.65	38489.19	24391.93
16470.78	36014.31	42690.04	63340.37	25345.31	41635.37	62725.32	30021.09	38023.15	26395.04

LN403.001	LN403.163	LN403.306	LN403.305	LN403.461	LN403.848	LN404.845	LN404.999	LN404.999	LN404.999
29923.72	48977.5	92230.66	35187.36	16963.99	51902.5	763685.6	44065.32	23540.14	73143.7
27197.88	42721.66	106155.2	34993.02	16037.2	55377.33	717371.4	45840.66	22305.4	70395.89
29714	49861	130478	37040.95	15704.49	59973.67	740536.9	46055.04	23220.18	62828.4
29418.38	42412.15	121695.6	34483.64	12625.53	60766.78	912390.9	44945.64	20721.73	65904.12
27366.7	47852.85	145862.9	38861.67	11685.63	66118.43	889311.5	40816.84	21954.39	68784.96

LN404.999	LN404.999	LN404.999	LN404.999	LN404.999	LN405_5.8	LN404.999	LN404.998	LN404.999	LN404.999
30178.41	74035.86	18100.44	46782.13	45555.99	38748.32	42547.36	57969.43	20275.11	20546.81
30157.19	72981.92	18586.96	46305.53	45168.67	35893.23	39790.7	60478.08	18971.85	18785.62
32076.58	69385.72	18431.98	41880.34	46798.87	35835.05	42781.56	60696.98	21482.44	17476.23
30501.06	70563.93	17152.52	38168.77	47325.98	38664.49	41269.23	63499.8	21560.75	18205.86
27577	70789.17	15356.92	41679.02	41787.78	34585.06	39587.83	58294.81	19834.15	17098.2

LN404.999	LN404.998	LN404.999	LN404.999	LN404.999	LN404.999	LN404.999	LN404.999	LN404.999	LN404.999
47808.8	49698.23	56330.81	29464.58	43881.23	79681.37	39033.51	73335.46	43488.01	64249.82
51262.65	53861.74	60913.97	27670.31	40244.01	79162.98	43543.93	74588.58	42785.9	61315
47315.61	53456.22	58056.2	29848.53	42383.18	74138.1	40387.74	75014.25	41119.1	62793.93
48767.2	53009.47	59263.55	32562.44	44912.37	73515.62	37644.42	71259.86	37092.96	64218.91
45021.2	47147.99	54802.57	27080.55	38938.6	69846.92	41015.52	70081.48	44307.31	54473.53

LN404.999	LN404.999	LN404.998	LN404.999	LN404.999	LN404.999	LN404.999	LN404.998	LN404.998	LN404.999
84133.83	67925.82	63061.72	72309.77	57615.21	34737.12	24777.99	58392.72	103241.1	52397.74
87917.27	77175.03	65377.01	65487.01	57999.28	39972.44	21797.31	53268.7	102350.7	56368.85
82693.08	70467.02	62673.3	69319.09	60206.81	36119.23	24490.46	59470	103764.2	54332.05
87408.21	74087.3	61967.67	60871.59	53019.5	38241.32	22455.42	57557.73	103606.9	55315.86
85858.23	70284.48	61209.71	69319.24	54598.28	34080.86	21450.89	51850.84	100064.7	54098.78

LN404.999	LN404.999	LN404.999	LN404.999	LN404.999	LN404.999	LN404.999	LN404.999	LN405.000	LN404.999
25122.25	34824.85	54767.56	49826.2	38282.04	29424.31	29483.58	26114.85	43452.99	38474.78
25757.78	38780.48	52224.95	44902.1	32472.14	29793.19	27954.2	32072.08	35013.81	40484.96
22333.65	35506.6	47792.14	47416.73	37698.12	27932.22	28736.86	30971.3	38383.45	38756.35
23112.57	33361.67	48392.91	44913.33	34381.72	29827.8	30470.86	28639.83	39706.54	38247.68
21685.76	36962.46	50994.4	45866.28	33217.14	32237.74	30022.36	31382.7	38059.66	39135.76

LN404.999	LN404.999	LN404.999	LN404.999	LN405.000	LN404.999	LN404.999	LN404.999	LN404.999	LN404.999
42693.24	54006.02	46063.31	28686.33	44198.99	74911.22	66778.06	52551.52	51343.67	28119.17
39411.01	56049.74	43889.64	23674.25	44039.42	76798.21	67914.56	51273.55	49229.11	30520.07
40926.01	53495.42	43832.84	29695.72	46518.35	76965.17	63106.73	51044.77	53059.26	28321.22
42515.61	54999.04	47732.71	28815.23	45096.75	69749.27	60593.07	54658.22	45354.61	29848.65
40869.85	52154.08	42161.32	29563.75	47172.36	72844.96	62328.88	49133.55	46607.78	29568.9

LN404.999	LN404.999	LN404.999	LN404.999	LN404.999	LN404.999	LN404.998	LN404.999	LN404.999	LN404.999
46863.81	22881.68	32316.28	40635.87	39460.03	67326.09	78328.45	94024.86	29365.59	72564.63
42616.42	30660.78	32485.77	49253.83	44017.04	59792.52	84160.97	97164.57	30618.13	65406.77
41579.73	25392.38	27745.62	41877.89	42677.79	70205.05	87746.52	88528.74	24082.13	67209.38
40593.99	28246.73	27930.7	40789.97	40880.28	66584.26	91484.59	93285.91	27469.94	66558.18
43670.62	27510.7	28141.88	44379.44	36663.37	62408.27	88658.34	91849.67	28742.38	64635.78

LN404.998	LN404.999	LN404.999	LN404.999	LN404.998	LN405_7.8	LN404.999	LN404.999	LN405_3.8	LN404.999
68869.1	41869.46	25120.63	41234.39	78478.22	41909.91	91091.71	36213.2	32795.54	63354.71
74276.39	40402.55	24590.5	43914.71	72218.03	41876.2	93281.49	37782.32	28742.83	62726.65
76132.94	38737.57	25471.78	39346.8	74642.31	43956.37	90998.69	38199.97	29145.23	70907.47
82569.7	39608.15	24993.27	42782.34	77659.64	39068.93	90419.01	34894.63	31128.46	57865.8
66159.72	40161.58	25777.39	44227.67	76746.12	38182.01	91579.01	40005.45	31353.39	63718.69

LN404.999	LN404.999	LN404.998	LN404.999	LN405.000	LN404.999	LN404.999	LN405_2.4	LN404.998	LN404.999
66736.13	47940.11	78602.07	52900.73	55856.72	37193.96	54355.36	27550.52	82803.09	69744.03
63069.09	38318.5	72746.07	53730.67	50110.8	36171.28	53470.54	26693.72	81051.93	67468.33
60700.53	37934.46	80151.7	56514.31	56257.83	44800.06	50103.41	29687.32	79190.9	70672.58
58272.85	45296.14	83045.93	56833.71	49528.72	38504.3	55065.5	29387.12	77249.66	64596.77
60058.5	40894.69	73497.45	55686.56	53971.85	34760.69	52117	23076.93	77890.67	68209.53

LN405.015	LN405.016	LN405.016	LN405.016	LN405.016	LN405.016	LN405.016	LN405.016	LN405.016	LN405.016
59922.12	55766.72	41154.85	25340.14	48579.87	31862.53	43570.8	41705.57	53292.19	86528.38
87544.24	39366.22	40249.01	12464.69	50424.84	52275.84	87582.41	64929.2	67368.55	102879.4
91326.83	61190.32	39290.37	14841.72	71205.89	37867.36	31292.69	58940.59	48989.53	70568.88
59635.76	72647.22	44865.17	13561.21	57403.36	32589.07	58401.05	48412.91	55507.31	85909.43
75350.91	58534.55	44720.34	15793.68	53835.45	42184.99	41587.68	49174.76	55659.98	71843.86

LN405.016	LN405.016	LN405.016	LN405.016	LN405.016	LN405.016	LN405.016	LN405.016	LN405.016	LN405.016
42634.05	83876.05	37727.88	67607.32	49180.55	106832.3	58932.77	48654.97	58985.43	54225.44
34533.77	64591.44	41780.62	63166.66	41112.44	82465.88	61746.29	35492.2	54002.93	47082.58
50289.48	72992.63	38597.98	87346.38	42005.1	103699.4	60874.29	60208.96	68166.93	69959.67
46326.77	81817.24	43427.69	54990.94	40117.05	102414.5	54442.27	47450.94	94643.41	63910.11
30337.18	76011.16	34975.43	86458.49	44463.79	104622.6	59559.75	55964.27	70555.25	57096.98

LN405.016	LN405.016	LN405.017	LN405.016	LN405.016	LN405.016	LN405.016	LN405.016	LN405.016	LN405.016
88276.69	74830.16	30982.7	63858.3	45756.69	121791.8	43653.23	28218.1	47732.9	43551.85
82863.21	60173.06	16388.36	75977.88	37137.6	86415.32	39625.07	32793.24	47123.04	49058.53
105998.1	68586.94	23433.61	79777.47	51437.03	121132.9	32950.29	29096.48	51843.2	62279.98
78593.76	59429.15	30303.6	83200.95	34529.84	67772.8	52475.24	37661.2	44602.87	42036.94
65720	57271.13	21015.03	67423.09	46866.41	77087.53	43646.94	30278.29	43373.15	45253.05

LN405.016	LN405.016	LN405.016	LN405.016	LN405.016	LN405.016	LN405.016	LN405.016	LN405.016	LN405.016
61877.41	51368.93	33162.72	44431.11	37656.79	45161.13	43239.68	35972.43	68996.41	63421.16
61790.03	69022.65	32426.84	55454.33	47497.88	74074.11	40069.8	34129.78	56668.13	51580.62
51657.17	62593.8	33994.03	46162.9	49442.94	49153.26	36053.27	38830.8	64960.15	61778.04
74988.58	68446.65	29606.23	29772.97	52942.97	60504.17	39366.65	27736.96	63832.51	93170.56
52234.76	60465.9	32629.42	46209.86	46353.6	48591.84	41426.84	27804.45	70357.01	68473.26

LN405.016	LN405.016	LN405.017	LN405.016	LN405.016	LN405.016	LN405.016	LN405.016	LN405.016	LN405.017
86028.71	73289.72	22276.81	29746.77	34465.31	63104.42	52443.68	46910.53	78447.4	30108.5
128610.9	64310.91	15092.07	19537.63	49339.82	36183.32	40948.69	71886.86	65905.53	14134.69
112511.2	68040.34	24220.03	17746.42	40473.31	64504.75	50513.6	64898.56	70524.89	15468.72
81376.47	91216.75	16082.84	20358.62	39324.09	40436.75	47459.16	65551.53	73366.07	15447.86
92754.92	57418.16	17174.36	25052.6	42770.25	45066.16	45407.08	49620.28	61184.26	18832.95

LN405.017	LN405.016	LN405.016	LN405.016	LN405.016	LN405.016	LN405.016	LN405.016	LN405.016	LN405.016
24065.77	36048.53	30605.7	75429.09	37690.34	49095.06	66192.2	76641.24	23695.22	86607.48
26495.75	47383.88	43405.7	84508.76	34713.27	51069.65	49217.49	63869.47	25244.19	91621.83
25592.41	41691.95	40746.44	51022.28	48702.29	37087.54	48680.8	90266.72	28551.59	73555.5
26024.8	53692.56	40977.23	57729.09	37938.23	54132.77	57977.77	77533.94	31681.65	93580.89
20158.29	42184.84	36264.01	57855.4	37345.13	50893.3	52382.26	74171.63	24228.4	81465.36

LN405.016	LN405.016	LN405.016	LN405.016	LN405.016	LN405.017	LN405.016	LN405.016	LN405.017	LN405.016
21015.55	64837.78	29559.91	88076.21	47927.98	19648.53	44485.54	33426.68	16688.4	43371.87
30653.48	98057.49	56631.7	62211.74	53965.68	13999.9	65662.15	30155.28	19894.47	55994.64
29978.64	61400.12	59157.52	59999.44	51770.73	18354.42	76816.9	36236.11	20807.2	51608.02
22926.77	67289.86	35954.74	80657.46	74333.95	27456.14	94200	34794.3	15321.05	36961.73
22218.13	66272.89	48466.19	68074.01	60711.26	21470.81	66396.78	31860.84	15880.35	45120.15

LN405.016	LN405.016	LN405.016	LN405.016	LN405.016	LN405.016	LN405.016	LN405.016	LN405.016	LN405.017
41558.64	40716.98	82414.89	21014.88	50582.96	67348.5	72374.91	42239.91	109117.4	23123.5
49920.7	61531.79	69806.69	15019.34	60579.78	138240.1	41432.42	50405.27	83068.66	20670.16
42278.86	44800.18	112627	16486.3	57233.04	70167.4	51783.97	41588.34	87976.51	21290.61
45264.36	54922.38	72504.98	19334.6	53458.95	77372.23	52923.88	43790.88	92706.08	24692.94
51986.23	53567.99	69754.1	15698.65	52620.9	99353.84	57048.41	46385.87	83734.45	24766.98

LN405.016	LN405.016	LN405.016	LN405.016	LN405.159	LN405.176	LN405.175	LN405.175	LN405.175	LN405.175
33798.28	49890.47	56108.15	30361.47	15553.48	25616.13	23601.93	30925.24	28617.41	25706.73
46396.85	53786.55	69235.47	33610.89	14390.92	28325.67	21362.38	30902.5	26478.27	28597.02
42317.26	52708.5	56040.3	33984.72	13934.11	26202.16	20321.54	36661.52	26982.27	29220.77
39491.23	53823.5	54939.5	36677.67	15420.8	26121.8	19716.15	33180.05	30380.13	32767.3
43019.51	54079.76	55338.56	32493.35	12783.21	28986.82	19767.91	32181.6	27096.2	29229.98

LN405.175	LN405.848	LN406.793	LN406.846	LN406.994	LN406.995	LN406.994	LN406.995	LN406.995	LN406.995
32475.11	70062.23	170465.9	189004.5	34087.87	21373.65	39629.8	36121.78	27530.21	28921.68
34708.78	61792.47	152824.6	199209.4	39173.59	23694.88	36102.19	38360.96	30908.27	30691.71
34311.54	84332.36	159625.4	265891.5	34263.31	20437.06	34184.68	35663.66	24814.05	27535.81
35039.76	72875.64	173711.9	381538	33657.12	18077.92	37093.51	39700.74	25869.08	27860.98
33463.54	86424.33	200199.1	373035.2	31096.52	19110.35	35442.01	34646.4	29283.66	27745.78

LN406.995	LN406.994	LN406.995	LN406.994	LN406.994	LN406.995	LN406.995	LN406.995	LN406.994	LN406.995
18451.23	48131.42	22296.69	32971.54	24912.97	40150.42	16012.74	38292.67	27981.49	13482.86
20451.81	46886.53	22984.61	35366.24	25571.99	42053.24	17609.86	35986.37	29645.88	13495.21
19570.85	43007.56	20994.16	29148.32	23794.94	40986.41	15344.94	34605.83	30340.99	11213.82
20161.9	44888.97	22913.82	32598.11	24679.68	44276.16	14065.41	29548.23	26522.02	11573.71
20402.33	42209.05	21238.72	35769.08	24938.13	40899.19	18212.32	31495.23	27386.8	11310.52

LN406.995	LN406.994	LN406.994	LN406.995	LN406.995	LN406.995	LN406.995	LN406.994	LN406.995	LN406.995
16855.16	31822	35957.13	23675.59	25693.04	35795.03	24200.19	50817.88	33298.96	29997.75
14389.21	34911.99	47247.47	25235.44	27019.42	40228.55	29062.17	53338.03	34853.37	30391.08
12316.93	29893.08	36084.94	23476.66	25361.19	36190.71	26307.96	47955.01	32457.22	28192.9
12906.62	31394.8	40191.12	23669.84	24555.74	36830.38	23904.14	46323.96	31144.05	27542.42
13854.75	29537.7	34935.8	19947.66	24912.23	36352.48	22619.19	49666.29	30290.71	27135.34

LN406.995	LN406.995	LN406.995	LN406.995	LN406.995	LN406.995	LN406.994	LN406.995	LN406.994	LN406.995
27627.08	28058.69	17307.68	46033.91	36422.39	27517.82	30952.18	31842.25	34759.59	32478.87
33341.1	29532.51	20717.76	46938.84	38813.86	28570.82	29712.52	32787.23	36440.32	32500.29
27624.93	29505.32	18882.16	46371.54	35404.8	31215.6	30588.41	36848.19	37744.16	36583.77
27568.45	28944.37	19438.78	47116.35	34589.2	30696.23	27141.18	29974.45	31385.53	30406.12
29286.96	29110.21	17285.26	44425.28	37971.07	28702.61	26474.82	31195.84	34830.46	32595.53

LN406.995	LN406.995	LN406.994	LN406.994	LN406.994	LN406.995	LN406.995	LN406.995	LN406.995	LN406.995
17874.48	29785.87	47974.73	45275.55	54947.61	23518.86	26059.73	37578.48	20431.48	27198.25
20854.05	31632.54	50042.82	56103.55	57839.26	27964.01	29152.42	39660.93	21472.59	28303.95
17081.94	25513.14	52439.37	52188.86	58905.31	25790.95	26737.27	38863	18010.83	24273.82
16373.35	28416.08	46013.71	52119.24	60052.27	28011.25	24862.67	39053.3	18290.09	24191.79
18585.13	27988.12	50934.58	50781.77	57026.19	24530.79	25279.68	39668.58	22590.76	28172.84

LN406.995	LN406.994	LN406.995	LN406.995	LN406.994	LN406.995	LN406.995	LN406.994	LN406.995	LN406.995
30432.27	35425.52	22047.95	23561.97	34164.28	22522.57	17927.52	33847.47	28549.68	33399.95
30643.58	39814.44	19493.74	27489.95	36571.32	23985.57	19839.15	34390.7	27262.43	36795.23
31776.46	36278.53	21481.08	28599.16	36562.16	23197.02	18030.49	31709.47	26393.82	36906.51
29615.62	37389.43	19157.23	25671.79	35155.15	21998.09	20135.69	31275.82	28101.55	31148.19
30237.66	35969.01	21967.43	25737.86	32712.9	21065.02	17582.21	30424.31	24803.48	31999.29

LN406.994	LN406.995	LN406.995	LN406.995	LN406.995	LN406.995	LN406.995	LN406.995	LN406.994	LN406.995
31012.38	27743.66	24932.93	26212.03	24515.85	26859.08	33657.67	41540.67	28713.08	28381.68
36266.45	29036.57	28989.52	26214.8	23432.46	26867.78	39192.36	42767.39	30046.61	26935.36
33278.53	27669.36	25404.6	26869.53	21787.73	26102.33	33079.13	36809.52	27538.68	27850.83
33025.5	24994.61	24477.77	27036.15	23918.94	26968.97	31582.99	41836.92	25616.97	29899.47
28044.43	27778.41	26303.11	25691.67	18253.97	28018.37	33745.53	37708.28	27821.32	29067.33

LN406.995	LN406.995	LN406.995	LN406.995	LN406.994	LN406.995	LN406.995	LN406.994	LN406.995	LN406.994
24470.51	30278.42	28313.85	19655.71	42299.06	29700.78	14894.28	46599.65	28330.92	37520.68
29510.88	29185.89	30083.85	20020.38	45725.23	30731.19	12439.89	54173.47	29372.51	42894.77
28163.26	25928.56	29616.76	18929.78	45178.64	29931.86	11863.23	47201.69	30396.49	40117.84
24320.03	27172.52	29644.3	18983.39	40755.6	31027.7	13870.77	48503.56	27904.54	37343.29
26625.07	30497.91	30162.64	19060.04	45434.64	27297.49	12849.8	47565.09	31518.23	34419.46

LN406.995	LN406.995	LN406.994	LN406.995	LN406.995	LN406.995	LN407.192	LN407.192	LN407.192	LN407.192
21124.39	16251.92	63078.1	23140.47	14579.56	30097.28	70621.43	78817.19	13452.21	96280.68
17733.12	18136.94	64528.23	22398.47	17300.84	32009.95	101501.4	84102.46	18932.84	81182.69
18665.7	19195.38	61203.86	21700.33	15368.59	29013.68	70510.74	68541.1	13877.37	85168.71
20204.72	15665.52	59495.43	21542.6	14229.59	28931.81	66879.41	78572.89	13372.02	95613.62
21604.76	15422.51	64879.66	19426.59	16715.77	28636.72	73851.31	76172.9	15991.48	83487.98

LN407.192	LN407.192	LN407.192	LN407.192	LN407.192	LN407.191	LN407.192	LN407.192	LN407.192	LN407.192
61531.56	81251.62	82352.56	103617.3	117587.3	51566.72	59719.08	72682.87	46293.89	66100.23
86289.39	88838.71	94400.41	59091.48	65009.93	51326.9	58800.61	40824.63	60018.65	51623.45
88374.91	92018.43	77934.73	90200.99	76993.1	49339.27	70693.42	54343.64	59688.89	67761.62
92146.49	99960.75	77596.13	95889.72	74453.43	52822.66	70675.47	60236.42	65115.74	74386.67
85493.45	101353.7	76283.2	88816.69	78962.39	48484.44	70885.28	58601.97	65785.53	68982.43

LN407.192	LN407.192	LN407.192	LN407.192	LN407.192	LN407.192	LN407.192	LN407.192	LN407.192	LN407.192
42362.22	56710.81	43138.41	59272.27	70898.48	45219.82	44200.42	28857.62	18777.58	92395.04
49698.07	55817.54	48037.12	110318.4	78178.37	37166.34	36529.81	41596.4	25417.58	75981.32
49899.26	60260.13	46478.04	67691.61	62949.74	48413.67	39766.4	35352.39	25163	68020.08
50181.53	55934.66	43214.52	60146.15	62076.22	52397.21	40055.27	36117.13	24209.98	60856.06
50870.2	56410.28	46793.56	62069.14	61651.9	52755.47	44949.09	36902.33	25786.33	68187.55

LN407.192	LN407.192	LN407.192	LN407.192	LN407.192	LN407.192	LN407.192	LN407.192	LN407.192	LN407.192
90812.75	25284.19	36156.13	70283.37	33984.74	64265.45	36400.97	47002.01	17027.27	31136.66
77077.24	24532.32	35509.32	52950.88	37871.16	65018.24	32041.97	26678.03	16858.52	19398.7
82786.05	24388.61	36173	60381.69	38994.31	60658.32	33131.29	37650.81	15972.44	27607.7
81679.14	28488.84	36046.05	46386.6	38186.67	60745.6	37788.77	38637.59	17927.1	24549.62
71157.7	24681.85	31321.24	60726.98	38363.33	58024.1	37730.75	40262.16	16197.76	28421.26

LN407.192	LN407.192	LN407.192	LN407.192	LN407.192	LN407.192	LN407.192	LN407.192	LN407.192	LN407.192
76079.17	57703.94	54438.52	74559.17	26586.6	52147.12	52341.53	69460.92	90657.64	36987.98
78719.41	49380.51	35890.05	73245.65	22359.18	43912.56	43986.26	50946.06	68234.23	36421.65
67147.31	57444.54	46724.22	66905.24	20859.68	41632.69	42129.23	40205.83	57555.25	43308.73
57800.73	52729.65	50366.35	68481.76	19821.45	38513.93	45590.38	44597.83	56013.99	44365.12
60694.5	49847.1	44641.69	70912.95	24078.91	36518.5	42839.76	45982.34	63200.58	43136.28

LN407.192	LN407.192	LN407.192	LN407.192	LN407.192	LN407.192	LN407.192	LN407.192	LN407.192	LN407.192
61292.03	34783.17	50777.51	39841.64	60182.16	60442.12	26209.7	53822.02	24492.63	46064.5
40811.54	38713.1	61417.31	55053.71	54940.15	56000.4	18124.04	33608.28	39179.99	53384.02
39673.32	39042.61	60599.79	41491	53479.92	57043.19	20725.2	41096.51	29476.61	38686.55
35727.38	38814.51	61006.98	44686.72	54759.12	59904.59	23395.47	43674.03	24308.47	39807.43
41501.72	39764.24	61067.84	40783.66	54135.16	59925.73	21944.71	45701.92	27613.62	38908.78

LN407.192	LN407.192	LN407.192	LN407.192	LN407.192	LN407.192	LN407.192	LN407.192	LN407.192	LN407.192
48667.55	32725.72	40160.31	32915.17	62739.79	70224.66	46060.53	35296.72	54732.28	39640.25
63971.79	65519.34	47734.17	32259.87	44514.45	46592.26	55676.3	53576.87	71598.54	42890.06
55606.44	38257.65	42665.9	35245.49	51105.91	39100.24	52280.9	37555.47	58072.63	41817.23
54129.09	34668.03	44253.07	34716.38	55820.93	40234.9	52087.74	34919.18	65068.24	45771.72
52900.39	30636.71	44175.73	39042.89	52374.28	39043.29	53923.19	39540.93	57539.63	39390.79

LN407.192	LN407.192	LN407.192	LN407.192	LN407.192	LN407.192	LN407.192	LN407.192	LN407.192	LN407.192
35998	46578.27	17516.06	31986.44	48792.99	78053.83	24180.06	30137.74	96530.48	53599.78
32807.84	52488.95	34699.24	32184.19	30495.86	113379	15778.3	29469.45	65752.36	42853.84
36314.55	42670.06	19940.92	33029.31	39147.24	64833.14	16698.15	15945.4	71263.55	43261.53
37401.27	42879.76	21192.21	34266.4	44083.52	62664.44	16787.43	16931.9	73477.84	42215.85
33269.3	42708.46	19084	36408.07	40561.6	62264.9	16417.28	17347.52	70129.66	43553.42

LN407.192	LN407.192	LN407.192	LN407.192	LN407.192	LN407.192	LN407.192	LN407.192	LN407.192	LN407.192
60709.44	58699.33	36646.28	51501.93	33042.09	18795.87	26302.37	32971.57	30173.81	78147.78
60235.87	51802.91	55366.42	45847.26	42000.09	16852.53	23255.25	56041.4	42740.22	57517.55
57174.82	61689.45	38989.44	37769.26	42435.74	20833.62	25283.86	43777.97	43065.78	58102.91
56474.17	58805.6	37695.58	39508.2	43913.7	17277.27	28136.23	41272.55	40358.51	51767.27
58040.36	64610.11	38281.37	35526.18	39221.12	19577.38	22547.97	39705.12	39378.14	54985.02

LN407.847	LN408.197	LN408.788	LN408.822	LN408.842	LN408.991	LN408.991	LN408.991	LN408.991	LN408.991
17913.43	22373.81	98463.73	104984.5	148930.2	45052.11	92153.23	55262.93	22413.73	106715.9
17478.02	21546.78	91917.42	150731.4	149248.5	42440.86	102307	54183.85	22358.82	121122.5
20111.96	21616.68	94759.56	116627.6	152399.2	44859.49	108447.4	74040.34	20203.84	59083.37
17980.86	16337.09	106330.2	128188.1	156413.5	70105.12	61608.58	64495.56	17889.05	65542.16
19633.98	20439.95	132026.6	161349	183253.3	34352.38	61072.87	62040.73	22262.26	70677.94

LN408.991	LN408.991	LN408.991	LN408.991	LN408.991	LN408.991	LN408.992	LN408.991	LN408.991	LN408.991
77026.29	46774.33	78588.91	73569.5	54210.66	95193.22	21922.56	47723.61	83862.22	123911.4
82382.2	50088.13	89134.8	92155.07	82361.99	107295.9	43419.66	43975.86	62197.12	62363.51
63341.03	47258.9	84089.55	78069.89	77396.84	127214.4	20262.75	44745.48	61791.69	107012.6
78083.61	69579.84	87884.99	92209.54	68668.11	118778.5	32254.03	47801.12	74771.43	108670.8
123828.4	43784.67	83141.89	133294.6	77956.41	118416.7	28989.19	41616.35	42460.47	87680.4

LN408.991	LN408.991	LN408.992	LN408.991	LN408.991	LN408.991	LN408.991	LN408.992	LN408.991	LN408.991
44951.67	54073.63	25955.91	48097.87	67987.34	74676.69	49761.88	45677.62	81574.29	44243.75
34907.78	57233.08	26664.54	38158.51	129304.4	78273.53	64768.88	38444.21	63635.17	55521.04
51547.42	46392.04	28793.48	39260.08	84673.82	69577.14	63641.67	42407.16	67143.51	47338.02
49582.24	61441.6	28882.8	49887.7	73271.24	81834.06	66791.7	51853.22	62104.37	55426.78
40810.68	60008.26	21287.8	56842.74	67953.33	68756.82	57642.12	41708.38	53971.78	50849.84

LN408.991	LN408.991	LN408.991	LN408.991	LN408.991	LN408.991	LN408.991	LN408.992	LN408.991	LN408.991
88003.05	64083.43	82661.28	50484.64	75325.53	57523.09	34982.97	24749.83	71364.38	70252.79
66661.42	68299.94	61102.22	45903.68	87509.62	45222.42	46174	22199.07	69159	54966.74
75838.36	49592.67	93916.95	64157.6	70851.81	47220.65	33037.82	31880.89	73183.78	97169.63
69078.49	45765.64	66484.43	49168.4	84201.02	48087.8	35202.19	37958.18	109856.1	67755.85
69603.8	68258.3	58202.31	46995.88	81614.81	62661.39	57688.39	16711.78	76590.86	66035.13

LN408.991	LN408.991	LN408.991	LN408.992	LN408.991	LN408.991	LN408.991	LN408.992	LN408.992	LN408.991
132851.9	50870.68	133179	31772.94	82102.66	59582.9	77993.3	26514.3	35115.92	81308.62
106229.5	46485.42	84291.16	28940	88359.27	75043.78	62422	34557.81	38157.06	75519.33
108008.6	50608.69	107383.7	29415.32	74386.45	60840.56	55425.94	26614.43	43344.29	60994.49
73723.21	60423.81	97832.41	22532.32	74528.61	59519.36	81058.71	25264.82	42149.8	97950.08
177580.6	48906.2	109610.5	38317.37	82611.51	47940.98	81756.59	41584.37	41240.51	56177.35

LN408.991	LN408.991	LN408.991	LN408.991	LN408.991	LN408.992	LN408.992	LN408.991	LN408.991	LN408.991
64782.7	59737.99	65306.46	56011.9	75346.55	57633.8	32817.4	42225.26	103104.5	71904.35
85922.14	33803.03	44772.32	65055.86	60921.31	60435.03	32288.16	43533.69	62867.2	77483.48
87395.5	54210.54	63543.62	77560.1	49363.06	49537.38	30174.83	39905.33	97574.34	106462.1
88332.82	59958.16	59805.1	69843.2	63650.26	126540.5	32662.95	26044.47	65107.15	81383.34
88484.54	45897.18	82620.13	65164.69	77740.47	44095.05	36871.77	33546.35	86248.51	64482.28

LN408.991	LN408.991	LN408.991	LN408.991	LN408.992	LN408.992	LN408.991	LN408.991	LN408.992	LN408.991
85103.27	46015.69	87151.04	80486.39	32258.78	42844.01	105681.9	92021.7	49455.47	43986.26
102904.2	50539.25	102997.9	113226.8	33554.86	49570.16	122399.6	163798.5	40229.21	54116.47
136801.7	74433.89	96473.27	70230.72	40840.21	45883.42	67395.17	90817.48	49357.95	40538.44
114673	58878.67	107723.7	75798.6	32402.94	40663.01	92986.35	99347.29	41799.54	50386.59
124908.3	60676.2	163457.3	72174.43	34861.41	47238.14	107772.7	96402.99	43649.65	62863.14

LN408.991	LN408.991	LN408.991	LN408.991	LN408.991	LN408.991	LN408.991	LN408.991	LN408.991	LN408.991
78189.78	73614.22	133639.1	46772.38	90380.13	43710.06	53536.3	83052.76	43726.23	35599.66
91949.92	137483.1	108719.1	40356	114826.9	53812.93	48425.75	101865.9	74169.81	42919.59
76532.51	125548.6	169536.2	44257.15	105782.5	60044.29	47983.21	78058.73	54471.44	38752.42
82345.53	91365.44	131108.8	35781.79	87134.22	43861.92	73636.13	88159.18	89999.05	42356.83
73867.13	87855.64	101226.6	48508.65	78506.24	49965.97	38284.01	99932.61	47559.46	45816.64

LN408.991	LN408.991	LN408.992	LN408.991	LN409.136	LN409.209	LN409.311	LN409.405	LN409.405	LN409.824
42338.06	34510.99	26900.36	89910.53	16437.34	66488.64	44170.07	27523.05	21911.02	19144.23
54364.55	55264.34	39456.38	79100	14870.46	94161.94	41654.7	31755.88	20307.26	15868.6
39226.08	39131.93	26378.57	88598.09	16021.48	87611.84	40667.24	29797.92	19317.25	16802.56
48871.06	47160.88	26729.51	85701.19	13315.91	65176.63	34456.49	29879.79	20365.23	17958.83
49961.3	46412.34	36849.32	86734.14	14364.26	81326.22	36701.66	29650.66	20005.51	17183.29

LN410.789	LN410.823	LN410.841	LN411.026	LN411.026	LN411.207	LN412.839	LN412.967	LN413.004	LN413.023
24136.68	21105.15	13104.33	54990.8	47613.6	14387.46	11624.69	52781.09	36915.16	16356.09
22601.15	19541.73	29683.75	57737.13	47775.82	16013.54	13103.16	48617.27	40880.52	49708.43
22272.1	18298.36	22140.38	56620.47	46947.35	13532.28	13152.63	49834.3	37578.96	49901.51
22968.35	17746.31	24518.97	55578.33	46749.58	14695.54	13415.49	45376.02	38582.96	46930.29
25595.3	17866.29	25595.3	60506.21	43761.42	12522.64	11132.5	46164.58	37189.54	44697.65

LN413.023	LN413.023	LN413.023	LN413.023	LN413.023	LN413.041	LN413.222	LN414.225	LN414.322	LN414.322
17594.15	16134.19	17575.89	17576.93	28093.74	56522.75	206321.5	34003.71	13971.72	11282.46
38018.13	47151.98	39494.95	39170.3	64466.14	53581.27	224956.2	36403.47	12576.86	10156.52
39529.18	42129.86	39322.85	39062	59797.6	52128.79	219224	40614.07	6479.162	5268.992
37059.19	44154.41	39946.18	37453.63	64293.28	54853.16	185927.1	36680.64	2411.774	2550.008
37467.13	42984.54	36660.44	41164.07	60433.03	57402.94	217226.4	33329.42	411.76	411.76

LN414.982	LN414.982	LN415.001	LN415.001	LN415.001	LN415.001	LN415.001	LN415.001	LN415.001	LN415.001
44872.63	56330.25	17875.27	22228.66	38046.03	33335.56	63058.7	50555.65	41188.56	79689.82
50377.16	56837.75	15901.1	22924.1	35808.5	28957.54	39513.22	54421.66	44860.25	58037.76
51115.77	54003.63	12877.59	26999.3	40376.96	29675.67	48680.66	57326.65	41986.17	61102.36
46647.45	48449.05	14662.78	26403.52	42372.42	31183.75	39463.95	63141.66	42776.26	60660.83
45972.13	59091.3	14180.72	30184.33	38084.16	29640.2	41029.1	57137.33	40390.39	64098.13

LN415.001	LN415.001	LN415.001	LN415.000	LN415.001	LN415.001	LN415.001	LN415.001	LN415.001	LN415.001
42342.48	46247.59	36408.23	50411.38	47272.54	41078.43	63951.31	68269.84	40634.97	25789.42
49966.23	33555.73	35672.93	31965.14	42754.09	61197.64	44218.39	61954.94	40261.42	32312.28
46150.45	33054.37	40136.48	32863.94	41422.88	63214.09	43547.25	66073.08	40862.74	29261.04
52521.19	36258.94	42611.23	29252.56	40685.6	59718.5	45598.73	62186.87	36090.31	30477.28
43521.52	37429.67	42888.45	29692.89	47370.42	68098.06	45612.34	66619.59	39281.93	28798.22

LN415.001	LN415.001	LN415.001	LN415.001	LN415.001	LN415.001	LN415.001	LN415.002	LN415.001	LN415.001
68711.34	28702.84	48802.21	28354.01	29700.9	50060.91	59958.81	11793.3	40490.95	80255.09
48838.85	21055.99	52952.26	33568.44	41039.74	47216.69	58310.51	12071.13	28116.72	58665.71
49363.07	22337.51	45102.69	30708.17	45502	40933.4	56789.24	13561.19	31282.53	56748.01
47639.96	25351.5	46790.11	31016.87	45652.79	45688.88	54320.11	13921.09	29904.99	56670.84
50229.91	22275.06	44740.14	33283.92	43464.92	43214.95	59464.16	12429.59	30524.81	51801.25

LN415.001	LN415.001	LN415.001	LN415.001	LN415.002	LN415.001	LN415.001	LN415.001	LN415.001	LN415.001
50321.35	29419.17	44986.82	20370.04	21287.99	20649.39	30570.28	22641.75	54997.33	73806.07
61201.89	30214.28	44381.27	25767.47	19700.94	19879.09	30345.89	33358.63	44530.1	56605.77
53524.55	29696.21	42786.07	25826.67	15021.02	22258.66	28598.64	35054.6	44196.66	49718.79
60935.13	32411.33	38664.82	27755.44	18278.15	21689.22	32561.17	30975.07	44292.43	49405.91
53071	30251.11	46472.44	29348.86	17527.49	20457.52	28654.36	33020.87	44934.35	49951.26

LN415.001	LN415.001	LN415.001	LN415.001	LN415.001	LN415.001	LN415.001	LN415.001	LN415.001	LN415.002
43941.35	45981.26	44599.32	48434.4	22527.71	43287.1	31460.61	36234.96	36738.77	28867.99
49145.01	46434.54	42423.88	43622.85	26817.62	59090.95	44812.9	39914.79	35695.45	20522.95
52244.74	38854.61	45449.65	43349.03	28139.53	57198.43	49017.07	42609.66	35554.18	18071.45
53677.55	41781.55	43341.5	48193.17	27656.5	54304.07	46013.75	43200.7	34341.55	15889.57
54897.62	44882.86	41870.47	43621.95	28209.95	57142.77	42087.93	38627.15	29172.68	17893.2

LN415.001	LN415.001	LN415.001	LN415.001	LN415.001	LN415.001	LN415.001	LN415.002	LN415.001	LN415.001
32021.67	30256.43	50284.28	28169.08	49169.33	40531.85	26510.4	18522.37	49381.24	51805.77
24884.11	24270.21	48004.54	32276.79	62509.48	33213.41	22592.13	22477.24	41267.69	39737.23
25639.47	25816.06	43547.85	31508.43	60566.68	32809.74	25100.7	21825.54	34841.19	37224.6
25504.45	27680.89	44766.13	28239.26	61223.16	32642.8	24282.86	19694.58	40862.84	39646.6
22395.99	27588.09	47511.93	29049.38	55001.69	33377.77	23552.35	20564.2	38147.17	35178.47

LN415.001	LN415.001	LN415.001	LN415.001	LN415.001	LN415.001	LN415.001	LN415.001	LN415.001	LN415.002
22869.44	32556.55	35549.92	26781.46	30195.87	39564.61	41217.79	22959.92	18231.28	34916.56
23198.83	37109.28	39312.83	27905.28	32001.01	27340.55	40223.14	20843.62	25092.15	29707.31
19285.26	33729.19	36766.05	25427.42	28285.47	24867.28	42161.84	18429.97	25582.52	30581.79
23436.97	35652.36	36036.99	25997.74	28873.12	27023.94	35145.27	20397.83	23617.23	32432.32
25609.58	35844.78	33428.73	28746.13	29601.15	28207.87	37646.41	17941.08	24356.76	32230.66

LN415.001	LN415.001	LN415.001	LN415.001	LN415.001	LN415.001	LN415.001	LN415.000	LN415.001	LN415.000
31223	27558.48	28418.67	54844.02	35062.61	44075.32	24630.3	59751.37	32660.44	73656.34
29095.43	26983.34	27262.76	50607.5	33924.72	33425.65	35884.43	63152.76	33618.66	59675.21
27524.15	22759.12	27341.51	44991.7	30622.14	35929.35	28011.64	55849.5	33084.78	62019.26
25867.66	24397.19	27525.38	45223.72	35144.18	35301.76	31702.92	57819.25	28049.07	66612.6
27391.74	25717.82	28485.16	51999.81	27065.15	37528.59	31054.88	64742.33	34670.91	69555.28

LN415.001	LN415.002	LN415.002	LN415.001	LN415.001	LN415.001	LN415.001	LN415.036	LN415.036	LN415.036
65674.55	17161.71	15618.15	27850.25	42288.47	16698.69	28411.32	48547.11	53364.94	49624.64
56660.07	15812.16	18971.33	30018.2	40674.79	16705.64	31537.99	46324.42	54697.6	55027.35
53379.55	15396.25	17128.78	32767.27	40463.11	15943.31	29327.53	46411.32	47754.47	50449.27
52004.21	16312.8	18870.38	28893.22	38644.59	15562.82	30108.15	51072.86	54103.12	47056.71
51546.97	15441.74	17882.5	29159.01	38192.74	14659.37	31489.07	52160.57	49400.26	48456.33

LN416.846	LN416.997	LN416.997	LN416.999	LN416.999	LN416.999	LN416.998	LN416.998	LN416.999	LN416.999
59452.81	50497.7	41162.07	41955.32	33501.18	15524.62	43275.26	56661.53	24763.45	29811.35
48049.59	45967.04	46804.57	46003.13	31462.73	20336.1	42345.28	66413.48	36379.65	31914.16
38544.36	47684.88	48271.87	35713.12	32423.52	15113.14	43032.26	60422.56	29960.15	23877.35
52700.85	54246.22	28275.11	38326	33574.51	27123.73	47171.94	61608.43	27905.26	33387.86
45724.64	46489.93	45095.31	47095.89	31009.97	17440.73	40466.9	53351.27	26653.96	37058.3

LN416.998	LN416.999	LN416.998	LN416.998	LN416.998	LN416.999	LN416.998	LN416.999	LN416.998	LN416.998
45172.29	28723.68	34135.84	44978.98	51267.18	22217.41	52503.57	35348.59	37585.08	21768.31
54208.06	28927.19	37544.84	40530.6	74999.94	32484.59	59644.95	36649.8	54099.81	20321.67
60180.76	49917.81	35042.42	50117.42	48146.72	23226.67	54724.43	33620.61	51612.64	23820.09
44699.27	35538.87	50992.27	59457.77	44122.41	23492.06	45847.81	48464.64	34026.47	19006.37
48156.68	25185.68	30564.26	44374.44	55781.75	22034.75	51859.96	34174.23	37060	19591.05

LN416.998	LN416.998	LN416.998	LN416.999	LN416.998	LN416.998	LN416.998	LN416.999	LN416.999	LN416.999
38280.16	34575.41	46241.22	36183.61	44671.24	39796.24	44325.73	34561.04	19308.84	25933.9
32437.87	35573.57	32114.97	36952.55	49061.27	36417.03	42627.67	40921.67	16114	24175.41
39211.88	26092.96	31124.88	42226.53	45317.56	40932.18	52814.48	29310.5	15426.74	23459.23
35326.42	32793.82	36946.79	46631.93	57676.48	28902.64	39052.66	34166.35	20051.15	27901.66
36525.56	36325.11	42441.74	34726.92	48593.51	37459.24	42559.77	33161.16	15224.54	22779.25

LN416.999	LN416.998	LN416.999	LN416.998	LN416.999	LN416.999	LN416.998	LN416.999	LN416.999	LN416.999
17566.3	26930.03	33229.16	40073.09	32932.27	35084.16	25238.05	15756.4	23995.84	37415.94
22798.35	32653.38	25290.2	43799.72	28779.95	34146.83	29538.02	21323.91	40708.84	33081.66
19621.6	35321.1	27066.35	51461.32	36956.15	33704.51	31141.15	13825.11	23712.44	34534.83
17789.5	30427.81	33768.24	45196.93	25715.81	45054.71	41560	15026.31	22285.13	34509.42
16273.96	25218.35	28995.1	40313.26	29146.73	44616.21	28823.76	15038.39	27983.95	29528.25

LN416.998	LN416.999	LN416.998	LN416.998	LN416.999	LN416.999	LN416.999	LN416.999	LN416.998	LN416.998
37079.48	17239.18	63807.75	50936.45	32879.24	31396.67	38373.96	30441.4	29663.55	62558.8
30487.25	18206.69	76413.08	40010.76	48612.64	42693.03	63444.14	42700.53	32210.38	45243.79
29946.09	21868.32	59546.24	55683.04	32318.46	39571.41	45658.85	49056.93	26667.89	69263.58
32095.82	17445.12	64967.64	45685.4	35364.46	37516.69	49251.13	50327.87	34189.04	66701.78
43908.3	17160.29	56336.88	55771.51	37118.36	28337.4	39125.91	35985.4	23771.52	60883.4

LN416.999	LN416.999	LN416.998	LN416.998	LN416.999	LN416.999	LN416.998	LN416.998	LN416.998	LN416.998
16066.04	25016.46	44032.45	19703.51	31180.85	21486.57	18295.6	37794.97	28810.56	43442.24
18111.38	27483.64	49484.98	26383.32	26877.16	20544.02	12257.13	44094.16	34820.29	36796.76
19081.97	25923.68	65625.54	16891.85	18942.98	29757.59	13506.86	35299.99	29846.92	43605.3
15854.85	27679.68	51738.1	19257.25	26750.62	14368.41	13698.18	44268.28	31494.53	46741.84
17115.42	23114.33	41911.01	20741.68	31642.43	18705.5	15713.82	38311.49	32657.8	44269.9

LN416.998	LN416.999	LN416.998	LN416.998	LN416.998	LN416.999	LN416.999	LN416.998	LN416.998	LN416.998
58244.43	29954.44	36395.52	44775.23	32257.92	31118.85	18910.47	51171.7	44485.28	38736.66
79963.68	26857.78	31435.61	38269.05	21152.39	37683.52	22737.19	53957.82	52310.51	35396.34
61271.16	34033.56	36922.89	32684.19	34123.61	36568.01	15452.81	46915.92	51907.43	47110.41
69107.72	25294.78	46970.61	44785.87	19252.49	31090.87	14043.79	45016.03	49853.76	37503.08
56071.95	31981.04	36281	45336.31	29718.44	30971.58	18297.3	52854.27	45761.78	40834.45

LN416.998	LN416.998	LN416.998	LN416.999	LN416.999	LN416.999	LN416.998	LN416.998	LN416.998	LN416.999
49724.96	34640.59	41906.41	28869.06	29128.86	38061.53	42428.2	32579.54	35652.09	24425.94
40166.86	45145.6	33407.45	24541.46	28043.42	31219.52	40527.39	23774.76	33118.63	27372.09
46600.03	57152.73	39591.83	25131.13	34856.26	25231.94	37480.28	29616.6	33134.83	28222.57
51610.32	35427.84	37679.52	26991.07	35776.67	33264.71	43831.97	26770.41	35382.96	25624.98
50809.57	34948.67	47067.78	28920.87	27127.79	33965.74	42522.32	27726.03	34199.19	24807.8

LN416.998	LN416.998	LN416.998	LN416.998	LN416.999	LN416.999	LN416.999	LN416.998	LN416.998	LN417.016
42303.93	32610.26	53150.66	47243.02	25813.18	15075.4	33170.67	46701.57	27125.36	55126.97
47871.25	30236.44	37459.14	48108.4	27617.13	14216.69	30259.68	48750.83	24839.48	52361.69
43683.55	33025.35	52528.8	46527.91	37658.96	13171.79	33589.48	41843.14	29651.01	61227.16
44333.4	32653.32	47859.81	43069.25	29979.59	17825.69	30602.14	48045.8	28165.77	56857.56
45106.64	29930.92	44450.17	46818.37	26784.7	14738.44	31598.76	49883.41	25672.23	56640.63

LN417.035	LN417.035	LN417.208	LN417.212	LN417.212	LN417.212	LN417.212	LN417.212	LN417.212	LN417.212
52246.13	44132.35	22549.4	29805.11	27402.32	17646.47	21199.49	21508.97	18787.87	18518.48
53123.16	47340.73	19792.28	29641.36	28918.39	16128.35	20822.22	20721.34	19195.95	16116.42
49771.46	47383.84	23086.39	26918.8	27579.17	18295.05	21613.27	20052.17	18420.99	18302.41
51004.3	46313.34	18690.92	29588.92	28393.34	17194.9	19176.4	23618.46	17353.04	18491.13
43665.35	43333.34	17876.89	27468.14	28311.11	16233.21	19588.43	21256.76	14999	16368.3

LN417.854	LN418.994	LN418.995	LN418.995	LN418.995	LN418.994	LN418.996	LN418.996	LN418.995	LN418.995
35868.96	40445.46	40477.31	30327.9	16095.87	64398.04	27242.21	18066.39	29654.97	51877.09
25994.02	38506.98	52054.48	27869.57	16057.55	57699.79	22690.76	15419.43	23103.22	38653.43
40860.3	39581.87	48863.03	24470.46	14840.01	63160.13	24299.65	17943.72	24298.73	41588.48
31748.8	40290.38	47170.16	24558.79	14936.74	59329.58	22150.92	18634.25	23587.72	43434.4
37360.36	40932.09	52079.01	26975.6	13548	62904.93	23431.3	15999.75	23955.38	39834.08

LN418.995	LN418.995	LN418.995	LN418.995	LN418.995	LN418.995	LN418.995	LN418.995	LN418.995	LN418.995
60791.84	51661.99	21910.34	59847.08	56867.44	37961.32	31415.19	46170.21	34124.41	36954.95
54968.32	54235.57	22990.3	43607.64	59586.91	29698.75	32647.85	39913.68	31870.73	35539.43
56258.04	54158.9	22234.22	47492.04	66071.34	30721	32393.46	40989.92	33280.63	34578.94
58058	51340.45	21249.4	46465.9	65826.01	30767.89	33242.41	34433.13	34861.29	35226.8
61076.84	59320.57	23044.97	46709.63	62280.12	27721.87	32557.15	38064.51	32579.87	34030.11

LN418.995	LN418.995	LN418.995	LN418.995	LN418.995	LN418.995	LN418.995	LN418.995	LN418.995	LN418.995
45548.12	31709.93	37393.51	15978.62	49793.44	75091.37	35024.29	46484.28	30651.25	41037.95
42877.39	28767.28	37011.06	12990.28	59101.51	49794.19	29519.41	34060.9	28200.5	41581.87
40039.18	31650.78	38900.45	16267.99	62233.71	48152.82	31809.2	31712.34	25033.64	42243.92
36181.48	33813.63	37218.21	15153.43	55434.36	52484.73	30974.13	32310.71	26130.31	35971.58
37179.75	29632.74	36594.8	17046.26	57349.99	49558.5	32621.89	34116.8	27704.15	42177.62

LN418.995	LN418.995	LN418.995	LN418.995	LN418.995	LN418.995	LN418.995	LN418.995	LN418.995	LN418.995
37187.3	30583.41	56103.4	32132.21	40966.1	39953.87	40223.95	57406.64	38738.83	12357.63
47127.88	30278.95	51310.46	33604.69	40755.94	39937.85	31067.34	57893.18	43227.95	14929.88
49834.35	30666.14	48530.07	30093.37	39097.29	39484.18	33765.65	56541.04	41469.53	16426.55
49920.82	31552.38	48058.55	31733.44	35993.16	39927.14	31101.17	55698.71	44473.67	16279.86
46291.61	27915.29	46623.48	29583.97	35827.18	44209.69	31044.62	55056.42	39858.52	15366.66

LN418.995	LN418.995	LN418.995	LN418.995	LN418.995	LN418.995	LN418.995	LN418.995	LN418.995	LN418.996
34030.32	25309.71	60471.63	26088.58	26278.92	71011.72	50741.75	15761.98	28745.03	23241.8
32990.51	17080.24	41911.5	33730.87	20854.17	44473.32	63696.86	17777.95	31141.99	17382.05
33346.45	18564.23	45726.58	31676.28	18477.19	44804.19	59126.03	15877.42	32766.62	15230.97
32463.99	18165.74	39590.71	28841.01	21291.67	44843.53	62792.49	18984.21	32028.99	16940.59
37787.36	16949.65	42316.58	29330.78	20273.2	44542.69	61680.23	15763.29	32888.25	16500.7

LN418.995	LN418.995	LN418.994	LN418.995	LN418.995	LN418.995	LN418.995	LN418.995	LN418.995	LN418.995
22889.41	31471.95	70610.45	21827.66	35010.68	20660.6	41419.8	36593.46	31964.86	41537.89
27926.3	26263.35	75802.46	17696.95	35411.37	19620.07	40239.11	31777.89	38926.08	40713.1
31790.81	26108.1	79141.65	18258.82	33949.8	19261.79	43708.09	35492.9	37590.51	35770.66
27478.35	24252.5	73787.06	17568.75	32064.1	22095.78	38894.07	30066.88	40040.2	39296
30898.26	25031.57	76255.27	18091.03	36475.75	19690.38	44865.91	32081.67	38042.73	41080.42

LN418.995	LN418.995	LN418.994	LN418.995	LN418.995	LN418.995	LN418.995	LN418.996	LN418.995	LN418.995
54229.48	29921.13	60706.72	61540.4	23954.87	21187.62	14731.14	38163.81	63093.78	42423.79
42756.64	30678.58	55969.6	54284.3	20071.07	20420.35	16059.3	39053.61	56916.02	47041.6
39163.41	28011.98	61076.81	57699.28	19517.63	20908.54	17696.15	33124.92	55496.47	42043.16
39859.1	28066.45	57924.31	58742.18	20277.21	21533.89	15690.49	38349.2	58511.43	44158.01
41399.94	26384.66	59288.43	57366.91	21554.56	22733.86	14213.15	33031.01	55415.76	48000.08

LN418.995	LN418.995	LN418.995	LN418.995	LN418.996	LN418.995	LN418.995	LN418.995	LN418.995	LN418.995
60689.76	37405.99	25741.74	20164.6	32353.69	40396.58	32493.67	51949.33	23506.29	46405.12
56325.43	35465.67	24813.35	21690.02	35923.66	42680.54	29097.96	56753.48	25687.4	49714.63
49035.7	35792.93	21112.92	19763.76	34381.84	41029.78	33068.92	51803.19	24236.59	46136.88
52295.42	39513.45	20198.24	20706.86	34715.66	43582.58	30782.51	60855.44	26267.77	46747.9
55403.39	37217.31	24055.96	20208.94	31537	44807.97	28395.04	51995.58	24635.86	47502.12

LN418.995	LN418.995	LN418.995	LN418.995	LN418.995	LN418.995	LN419.166	LN419.227	LN419.227	LN419.228
57522.18	53990.25	89369.81	26221.68	29790.82	31406.1	69949.49	32304.51	35615.08	41019.69
49208.25	42693.59	79739.67	30968.45	36932.72	33297.92	67349.95	32447.59	36055.69	45791.97
47098.05	43094.68	83024.46	25652.84	30224.83	30133.04	222583.8	31815.67	34269.12	42221.11
47563.57	40436.5	84092.24	30518.34	34680.33	32177.7	198738.2	38906.34	36174.23	44458.95
51539.78	44912.6	85915.16	28089.21	36239.82	32032.56	11595.82	29666.12	36153.01	44223.04

LN419.228	LN419.228	LN419.239	LN419.802	LN420.242	LN420.296	LN420.789	LN420.841	LN420.992	LN420.992
43479.83	32343.59	280598.8	11139.16	57881.6	10240.65	18916.02	23360.11	36102.07	24018.71
45642.96	30499.33	271850.4	11197.18	62010.43	13562.5	18940.89	27626.41	39211.21	24255.1
45975.25	29778.7	274675.4	10072.49	61716.1	14735.66	17904.35	26096.59	37545.93	26058.27
46130.94	33290.38	288565.1	10277.19	48627.56	13485.55	22754.25	29533.19	38212.31	24383.17
42782.78	33494.95	241923	13766.88	55307.99	16680.33	25897.61	35402.16	36784.88	25327.09

LN420.992	LN420.992	LN420.992	LN420.992	LN420.992	LN420.992	LN420.992	LN420.992	LN420.992	LN420.992
22286.98	27140.89	20814.66	27619.6	36651.23	36179.21	22796.85	24526.52	33104.97	54738.22
20774.83	26645.3	21720.21	25889.36	40337.78	37495.8	21495.64	23302.1	33290.16	59503.13
22795.81	27553.6	23370.12	26572.29	36810.36	33279.26	21353.04	21902.89	33434.01	58321.11
21325.36	27457.8	25133.27	26275.17	42476.72	36515.31	21170.12	20061.99	34252.45	60594.95
21505.12	28086.75	21249.52	26061.04	37978.27	33248.52	19045.51	25267.71	32983.75	57209.02

LN420.992	LN420.992	LN420.992	LN420.992	LN420.992	LN420.992	LN420.992	LN420.992	LN420.992	LN420.992
42180.69	19396.27	19645.38	34288.29	49233.05	25764.4	24307.25	16072.77	26612.37	33305.78
37775.61	20369.79	22232.17	35095.51	52792.36	23443.22	22414.09	16868.24	28877.95	34464.42
40246.51	21396.31	21949.53	34457.54	51142.02	25894.92	23337.11	13799.8	26881.9	32399.8
36162.74	20215.46	22240.33	36087.5	53764.85	26800.35	24859.7	14196.18	24726.68	32908.57
40132.01	18735.46	21292.54	36628.16	53705.52	24111.91	25397.74	15613.58	26401.27	34922.01

LN420.992	LN420.992	LN420.992	LN420.992	LN420.992	LN420.992	LN420.992	LN420.992	LN420.992	LN420.992
69098.93	35683.22	44128.64	30087.4	22374.07	25663.01	26293.9	29341.92	30759.82	57832.09
71231.77	38826.32	39936.94	35472.16	21903.18	25265.04	25918.45	29243.98	31614.02	56820.67
72501.62	40538.23	43190.32	28707.98	23007.15	25427.51	25221.76	28671.98	31572.71	52373.39
73106.27	34197.46	40664.81	30431.5	22407.64	25161.84	27355.66	31096.05	30810.68	54965.12
71720.55	38618.39	46600.6	26074.32	21493.25	23532.38	25475.42	29010.34	32092.45	54169.96

LN420.992	LN420.992	LN420.992	LN420.992	LN420.992	LN420.992	LN420.992	LN420.991	LN420.992	LN420.992
41082.97	30408.4	27760.99	28432.89	26525.38	39164.05	44912.06	36301.78	41389.67	62092.2
36219.75	31073.84	27765.11	30626.13	29299.87	39825.2	42576.68	37674.58	43403.23	56780.29
39108.94	31572.52	27336.94	27672.3	28049.48	38635.78	44164.11	37454.14	43179.31	62272.46
42468.88	28553.89	31478.78	29395.11	26409.49	39961.49	42978.39	35397.94	42354.92	51606.95
38497.15	29896.3	29630.55	30418.95	28537.07	36436.96	45963.47	37790.47	39775.66	62387.99

LN420.992	LN420.992	LN420.992	LN420.992	LN420.992	LN420.992	LN420.992	LN420.992	LN420.992	LN420.992
57371.6	48496.09	52155.49	29383.99	41829.15	27991	35529.75	50762.37	65457.63	35159.45
62025.14	49785.16	51602.81	24323.95	42195.39	24960.73	32779.41	47856.48	63365.79	37374.66
60868.42	51923.39	49915.64	30025.3	39883.71	23829.97	38467.12	50869.22	62377.03	34772.58
64803.94	54955.31	53555.56	28276.65	38029.82	26178.32	34761.78	51652.87	65363.68	33736.35
56365.83	49385.94	57110.58	25791.14	44108.55	25463.54	35455.27	49543.6	64374.08	35577.82

LN420.992	LN420.992	LN420.992	LN420.992	LN420.992	LN420.992	LN420.992	LN420.992	LN420.992	LN420.992
42296.7	39226.58	22944.3	46507.46	35658.84	24344.09	56491.8	35289.36	37331.34	40250.8
43172.18	36367.34	23033.56	45678.44	35644.72	22858.84	54248.11	38039.68	35050.14	41329.1
43563.67	39581.95	24619.34	45960.55	35205.78	23161.82	54391.88	36675.05	38112.47	40205.55
41178.33	37913.7	22105.01	43931.19	35141.99	19180.26	55827.16	37567.16	37638.38	42105.34
46656.27	37307.67	24713.52	43068.24	34958.46	23055.15	53323.64	35148.19	35623.03	41004.3

LN420.992	LN420.992	LN420.992	LN420.992	LN420.992	LN420.992	LN420.992	LN420.992	LN420.992	LN420.992
13887.18	18775.45	33924.46	22109.54	66376.68	30165.07	28121.76	43267.11	32762.55	18240.18
14301.48	18055.99	33416.72	22478.2	61759.14	27120.98	29650.64	46388.4	28562.14	14303.72
14681.32	21230.07	29953.86	23203.04	61523.74	30754.94	27841.46	44888.68	30231.08	16195.37
12190.85	18749.91	31491.31	20699.15	63078.14	31633.09	29538.33	43801.45	30841.55	15312.4
13539.92	19087.48	31985.48	24916.27	59477.03	26108.24	27913.16	45797.86	26461.13	19545.96

LN420.992	LN420.992	LN420.992	LN420.992	LN420.992	LN420.992	LN420.992	LN420.992	LN420.992	LN420.993
25565.13	11890.25	42700.91	21865.24	45503.31	34201.59	79391.8	44605.43	32226.23	15977.22
29225.28	14941.1	39093.91	22117.04	45703.67	34585.71	77519.95	46561.69	28968.57	17668.8
28089.5	15838.31	43251.13	21820.53	45590.24	37772.29	78487.04	48372.49	23788.36	15758.46
28490.65	12338.29	42779.98	21345.23	50840.68	41962.43	81589.06	45842.7	28509.29	14887.14
28685.52	13922.8	38014.03	23001.25	45902.47	34498.29	71938.23	45365.11	26339.63	17633.42

LN420.992	LN420.992	LN420.993	LN420.993	LN420.993	LN420.993	LN420.993	LN420.993	LN420.993	LN420.993
18401.85	18177.6	20592.53	18001.23	31228.04	34457.75	20426.47	14246.59	21492.19	12266.2
15756.54	17800.32	17655.22	15798.61	31680.75	34937.87	21980.33	12983.78	19259.9	15078.99
17899.13	20464.46	18576.5	17325.35	31908.36	34074.2	20647.65	14524.23	18573.61	13446.21
18410.11	17184.91	19879.17	17738.41	31639.61	34013.38	20328.25	12380.4	18703.84	12287.09
14471.03	18862.88	19077.23	18094.02	32112.14	34853.56	18111.14	13338.26	16355.96	14194.56

LN420.993	LN420.993	LN420.993	LN420.993	LN420.993	LN420.993	LN420.993	LN420.993	LN420.993	LN421.027
15371.26	14421.75	38634.62	26735.22	40928.93	32105.98	25685.33	26398.05	26193.62	53580.51
16460.66	18242.65	39677.35	24088.69	39547.97	33013.39	25290.35	28530.81	24825.96	52011.87
16527.42	14575.08	39903.28	22861.86	46208.09	31547.29	26313.17	27967.77	23759.33	56244.29
15218.94	18008.12	38895.89	27222.14	40029.88	35665.52	23583.18	31758.14	25560.44	57894.21
14988.78	15564.91	40240.11	26166.85	41253.08	34047.73	24485.38	28333.14	28584.3	58496.64

LN421.028	LN421.029	LN421.245	LN421.796	LN422.798	LN423.006	LN423.007	LN423.007	LN423.007	LN423.007
38703.01	49246.25	19623.95	73415.73	13618.88	105792.7	145867.8	150395.1	173757.7	190788.3
42990.97	50058.15	19055.36	65686.22	7214.775	121177.5	140520.8	113487.8	127022.7	133098.7
44948.19	53958.11	17702.23	65343.4	12019.76	99491.08	202577.8	126308.6	117167.7	194401
46104.24	49013.7	18241.63	70763.01	13136.21	99399.22	147526.8	101583.5	118123.9	145056
46642.51	49971.67	19123.3	76204.4	12837.01	134084.3	130150.1	115026.2	182808.4	155108.1

LN423.007	LN423.007	LN423.007	LN423.007	LN423.007	LN423.007	LN423.007	LN423.007	LN423.007	LN423.007
202407.2	107778.6	57706.2	95563.28	98189.6	60407.86	47064.59	70752.93	108847.8	79608.63
147423.6	108983	80143.02	119870.4	153704.4	64760.96	83256.13	66877.83	87887.48	117958
121057.1	156229.1	51681.47	68133.65	92587.65	68447.52	69903.73	74194.96	147008.7	93012.07
88299.05	116150	59229.44	96681.66	87595.8	53669	82895.05	45691.06	100421.9	95836.85
124377.6	164543.6	50271.97	127994.2	76677.53	79032.57	68733.65	78411.77	101391.2	108524.5

LN423.007	LN423.007	LN423.007	LN423.007	LN423.007	LN423.007	LN423.007	LN423.007	LN423.007	LN423.007
64957.54	66652.17	64954.13	85375.32	32249.11	109567.9	162006.5	66442.34	25156.96	130399.6
51777.53	68984.76	76912.18	98257.71	37686.35	129810.7	104295.5	81523.65	17998.27	113292.8
52139.9	79991.46	116545.7	64324.48	31183.78	142852.4	117414.1	46840.7	15995.35	88607.69
103094.2	85614.74	68928.01	72674.27	34133.24	113187.9	117710	82761.44	15733.02	65245.77
72540.14	100099.5	68668.01	102325.7	32462.38	167896.8	86225.71	112541.7	20651.23	101799

LN423.007	LN423.007	LN423.007	LN423.007	LN423.007	LN423.007	LN423.007	LN423.007	LN423.007	LN423.007
100143.7	85299.5	64421.39	79906.32	53657.04	49149.83	49685.81	115463.1	134226.1	99944.73
101826	66724.09	79516.99	89486.11	72275.66	67296.42	54385.34	140700.7	117098.3	111063.2
86582	78147.66	58780.24	131246.5	54638.77	77057.86	40295.7	96983.18	110107.3	117033.8
113885	54624.38	96603.91	95401.65	69946.63	49130.7	63825.56	87850.7	166118.4	93863.41
67297.73	59772.49	58769.31	84415.64	51680.28	55819.34	33336.91	129137.9	77627.09	68745.81

LN423.007	LN423.007	LN423.007	LN423.007	LN423.007	LN423.007	LN423.007	LN423.007	LN423.007	LN423.007
62130.43	24366.75	43338.12	53537.94	56322.03	109429	28113.88	59838.48	69588.83	103074.1
118184.6	37893.56	45756.1	62550.91	51819.52	154994.9	46712.36	72164.59	57719.34	167274.8
88131.95	37700.86	56984.14	72300.63	43732.72	106561.5	39083.8	72932.21	79992.91	103139.5
98530.95	36724.89	29512.87	47684.3	71395.05	108030.7	35413.71	48218.93	66236.42	169608.1
95733.5	28550.31	43126.25	56838.8	72528.82	99428.48	33035.4	63594.21	57728.82	153661.6

LN423.007	LN423.007	LN423.007	LN423.007	LN423.007	LN423.007	LN423.007	LN423.007	LN423.007	LN423.007
30009.41	64033.49	80475.8	63838.18	128773.5	51140.62	55658.11	96548.84	54981.53	69090.81
26635.11	52121.39	83635.96	52857.69	88140.13	33545.58	52697.75	111405.6	57635.33	78627.4
27620.64	53934.7	58017.04	60100.98	88125.77	37778.61	53431.98	112685.6	39053.21	84083.55
32916.58	46340.06	61052.94	62669.54	108565.7	41399.07	50972.09	97217.4	61931.58	114668.9
30885.33	69272.86	57194.79	53808.75	96506.44	29647.77	70655.14	87132.82	39825.07	82763.55

LN423.007	LN423.007	LN423.007	LN423.007	LN423.007	LN423.007	LN423.007	LN423.007	LN423.007	LN423.007
65657.09	77117.91	103668.7	78897.73	58350.48	23901.94	65143.99	74978.92	127322.1	90945.63
47811.41	60317.74	105726.2	70655.54	63692.37	33790.09	70145.72	67876.49	93636.39	119852
63645.5	92396.14	109867.1	81102.57	61496.51	32092.94	58646.92	107207.7	83051.41	130240.5
48276.7	97304.41	106012.5	89594.39	45547.46	24301.05	64458.26	85671.24	98139.6	90538.08
66942.42	62725.06	82596.32	75289.99	87500.79	26834.45	50208.97	74115.78	106691.6	115389

LN423.007	LN423.007	LN423.007	LN423.007	LN423.007	LN423.007	LN423.007	LN423.007	LN423.007	LN423.007
127268	23288.16	61452.93	31646.26	75476.25	56352.28	76850.98	41227.22	35828.67	86046.19
101364.1	21725.75	61352.78	41056.52	131789.4	53427.64	82426.17	44684.84	69020.7	69400.69
82458.56	29762.19	69527.91	36685.76	88950.4	73004.96	94742.67	46737.73	58460.23	55885.71
111717.9	22496.45	49274.47	42085.79	109021.5	56988.22	63726.66	36666.56	54108.21	69586.97
95156.61	29402.69	54194.78	31994.31	93860.62	52291.52	71976.12	64307.3	50899.26	67460.66

LN423.007	LN423.007	LN423.007	LN423.007	LN423.007	LN423.188	LN423.223	LN423.224	LN423.223	LN423.223
89690.87	103194.3	60973.06	110678.2	51148.24	26153.41	27255.73	27850.22	34571.22	37137.24
53154.23	93860.08	54270.57	72627.8	57726.29	28789.3	30598.04	34758.12	28075.15	40142.66
51240.53	88453.31	51320.9	85917.75	67980.23	25159.96	24558.87	30348.43	30851.14	35221.39
48845.08	83960.68	55475.43	93601.01	56730.18	24986.31	28702.99	31982.03	32412.08	40231.35
53542.3	70464.65	77588.02	86357.09	69049.16	24679.57	27548.07	29232.94	31693.42	40607.13

LN423.223	LN423.223	LN423.223	LN423.797	LN423.835	LN424.832	LN426.850	LN426.850	LN426.850	LN426.922
29493.87	34107.54	34794.51	20453.15	18967	19655.99	36796.57	22539.65	24635.85	59643.06
30984.29	33756.25	33638.24	17664.51	16896.44	18147.52	39099.68	23878.86	24773.33	60422.57
27139.27	34353	32697.52	17034.05	18144.87	14536.32	37939.2	20156.49	21696.32	55823.78
28429.4	34441.07	35731.92	18220.42	14024.98	18169.85	34464.09	25767.5	20896.77	58709.07
29081.84	35169.8	39436.53	19342.95	20702.56	22961.24	34986.16	21346.45	20675.08	52724.93

LN426.923	LN426.982	LN427.215	LN429.037	LN429.037	LN429.037	LN429.037	LN429.037	LN429.194	LN430.197
67663.37	53324.35	9518.078	53094.12	39858.62	41795.59	30027.87	47409.56	111809	21774.18
66766.63	54448.34	8683.007	51834.79	46183.82	41567.71	30977.63	47132.55	121437.1	22208.45
63459.71	56468.4	11352.33	54361.44	43386.49	37646.43	29119.3	42737.38	125025.4	19644.5
69997.65	53472.37	11231.82	51627.95	38131.72	40407.51	29270.55	49735.62	117387.8	21150.08
65669.03	52786.26	13363.45	49748.78	44250.56	39171.73	31722.79	41607.78	99510.11	17480.52

LN430.861	LN430.995	LN430.994	LN430.995	LN430.995	LN430.995	LN430.995	LN430.995	LN430.995	LN430.995
29461.75	27534.74	52735.62	22384.94	26166.86	31738.92	43515.56	34127.13	44356.05	27954.41
31320.89	32702.15	51004.78	18966.22	25121.77	28909.26	39121.86	34285.67	39438.65	28012.94
33144.88	32320.78	57834.79	20456.19	24758.46	27948.33	41486.43	32323.77	40294.4	27234.86
26562.71	31760.43	56797.8	17847.76	22309.98	28710.96	40085.9	34183.67	39835.77	25871.95
28861.2	31928.45	55436.7	17832.85	23295.69	27099.82	39466.93	31463.6	39477.76	27750.5

LN430.994	LN430.995	LN430.995	LN430.995	LN430.995	LN430.994	LN430.995	LN430.995	LN430.995	LN430.995
48713.96	17340.24	29447.31	23072.38	19891.06	59191.74	49470.48	28674.56	42835.21	37844.2
45380.5	17085.57	25801.71	21247.43	20101.55	59973.3	50692.46	31117.36	45333.12	35797.03
47842.82	17436.7	29550.07	19583.51	20517.69	52886.39	49339.72	28257.31	43350.42	36495.43
49341.6	17029.19	26634.82	19798.93	18631.35	60458.84	51575.58	28927.39	38362.95	35313.28
47626.28	17402.53	25429.46	22950.03	19387	53040.84	51329.16	26676.45	41243.89	37038.14

LN430.995	LN430.994	LN430.994	LN430.995	LN430.995	LN430.995	LN430.995	LN430.995	LN430.995	LN430.995
67078.67	53648.7	47430.73	14584.35	20327.91	32591.55	32463.2	57202.02	29049.69	27258.21
57140.8	46973.28	45145.29	12583.2	19421.87	30581.16	30014.6	58797.57	29537.07	22056.21
64639.18	48277.44	42046.76	14194.38	22548.98	31468.57	32023.19	60080.42	27385.48	24729.73
58989.08	48473.31	51552.06	14157.08	20213.26	33994.16	32050.45	58029.21	25810.37	25871.35
59550.65	50602.26	49858.08	13010	18638.06	30136.8	31923.61	57532.89	27629.2	26789.48

LN430.995	LN430.995	LN430.995	LN430.995	LN430.995	LN430.995	LN430.994	LN430.995	LN430.995	LN430.995
48676.42	56367.89	19628.84	19789.25	21906	34767.52	39297.36	30196.91	34938.93	22205.97
53496.01	51496.31	17867.27	19674.27	22554.69	32181.61	41937.12	32554.18	31467.91	22147.61
52536.18	49373.16	18371.14	22605.69	21883.59	31647.09	40635.5	32383.18	33925.88	22345.51
50246	52639.85	17916.43	20299.24	22307.31	30351.96	41912.92	30002.98	34105.75	19934.21
49435.69	57298.22	17822.88	23444.03	22318.99	32564.69	41472.31	30739.52	28323.79	21682.49

LN430.994	LN430.995	LN430.995	LN430.995	LN430.995	LN430.995	LN430.995	LN430.995	LN430.995	LN430.995
55032.4	27512.24	29796.67	55108.77	31261.02	36418.69	18153.12	26079.33	32373.3	42201.44
59784.83	29498.78	27711.94	59100.33	27457.26	36304.71	16249.48	25253.94	32114.46	44184.13
60470.8	27447.43	27294.87	54588.84	30879.7	40016.08	16410.13	26355.54	31222.17	47589.23
57540.1	29299.94	28605.67	59350.68	28869.69	35382.23	16479.4	23397.98	31133.57	40426.79
60747.56	28552.84	28301.8	53663.14	28909.3	32448.64	16334.24	23168.11	30317.07	43210.54

LN430.996	LN430.995	LN430.995	LN430.995	LN430.995	LN430.995	LN430.994	LN430.995	LN430.995	LN430.995
15821.99	16017.96	44562.88	31452.73	35186.39	58358	87091.16	34907.49	42454.8	26663.46
15763.85	14732.51	39020.73	26613.9	36667.19	57808.89	84030.3	31381.62	38197.97	26487.84
14796.45	15889.91	42429.81	30081.31	35125.34	55109.42	85705.19	35705.35	38826.8	27731.64
15808.33	14796.43	46498.35	31049.38	35948.48	55718	84775.07	37265.15	40724.33	25292.33
13606.09	15034.41	40470.64	32066.75	36218.61	58833.55	84494.8	33872.29	42167.88	26043.54

LN430.995	LN430.995	LN430.995	LN430.995	LN430.995	LN430.995	LN430.995	LN430.995	LN430.995	LN430.995
49266.46	30381.57	26951.12	49978.72	28991.96	22424.9	19187.81	55175.12	31738.42	39281.19
47735.25	30528.1	25599.31	46260.76	29390.41	20455.42	18231.41	55003.6	32723.81	38613.69
47925.1	28013.55	24412.91	47130.78	28705.07	21920.21	20304.75	55386.37	35799	42607.29
44390.59	24538.73	25750.93	50246.49	29874.23	21264.88	15303.43	51798.09	30977.44	36167.27
45460.32	30764.07	26392.13	45411.07	26586.93	22555.61	18411.97	57689.65	33554.8	36882.15

LN430.995	LN430.995	LN430.995	LN430.995	LN430.995	LN430.995	LN430.995	LN430.995	LN430.995	LN430.995
21735.64	24277.28	23181.53	54802.31	23910.9	21068.93	35390.1	37158.9	45170.74	20518.4
22299.76	23739.33	21656.89	54654.72	20988.21	24529.3	34162.45	29315.09	41218.64	20383.21
19497.99	23688.18	24496.74	55157.88	22433.94	23010.02	34332.18	33951.85	42614.36	20561.99
22220.27	23631.2	21770.61	51644.33	22486.58	22549.56	41324.19	29112.43	42458.6	19618.68
20035.99	22580.87	22576.66	53429.55	20155.49	22282.99	34960.79	33422.55	41143.81	18306.37

LN430.995	LN430.996	LN430.995	LN430.995	LN430.995	LN431.168	LN431.168	LN431.168	LN431.169	LN431.168
24248.52	13009.05	52002.63	32457.34	50443.91	22449.91	16346.9	24989.05	25808.05	23285.01
26039.3	13314.53	48842.36	28695.06	55853.5	22344.74	17583.64	26729.13	24279.12	28837.77
24272.3	13348.75	48804.89	32482.32	49076.02	22510.25	19354.02	23777.24	24259.1	24732.34
24509.03	13656.87	56866.97	29313.22	53692.32	22765.71	16964.15	27082.52	23041.49	23295.76
25210.16	12948.53	48948.6	29823.34	51964.02	18772.41	15891.77	23143.75	26069.46	24398.84

LN431.168	LN431.194	LN432.464	LN432.991	LN432.991	LN432.991	LN432.992	LN432.992	LN432.991	LN432.991
24891.4	48156.14	33641.46	42904.66	56671.67	53140.81	46674.73	53366.85	52013.66	53159.16
23776.81	62272.78	30352.64	49888.03	77299.24	54372.08	50292.43	57614.02	63302.11	37973.38
25553.47	46725.45	34897.94	34716.81	55724.4	50719.19	49095.16	68805.47	66502.19	66624.04
26587.64	51534.38	28720.91	48385.94	43088.37	36770.51	42811.38	46702.58	51778.3	67091.18
25812.47	52259.01	24967.04	41397.6	44698.97	60455.85	45245.95	52934.59	58574.92	42750.64

LN432.991	LN432.992	LN432.991	LN432.992	LN432.992	LN432.992	LN432.991	LN432.991	LN432.991	LN432.991
71476.31	26649.66	59373.78	35413.16	35907.36	21999.98	75403.71	13739.65	50379.68	48490.65
68402.87	24278.29	102685.4	23881.02	43642.98	18057.06	71488.91	12713.6	45290.05	56753.31
110720.1	26100.14	55341.76	36987.46	41611.65	23563.45	46546.3	14803.14	48964.4	42001.22
77104.89	31661.76	59657.36	28330.95	43090.25	28921.72	78805.33	11808.77	42398.03	49811.66
90261.59	41886.93	75210.49	40180.01	47942.22	23213.3	75608.45	15412.72	39012.82	59646.35

LN432.991	LN432.992	LN432.991	LN432.991	LN432.992	LN432.992	LN432.992	LN432.991	LN432.992	LN432.992
70905.91	43654.3	71271.04	53238.42	40094.74	43223.65	27639.11	48664.48	29997.15	33998.39
70849.42	39730.67	78721.12	58493.79	39390.43	39868.52	26439.9	46259.18	36657.59	23275.13
62354.34	46610.22	69552.3	60610.71	39352.95	43533.84	29020.54	50240.93	37130.77	36914.87
51131.71	46370.28	72445.98	50866.79	31220.86	39369.89	30141.58	54399.7	26093.9	38245.28
72097.32	59178.8	55189.74	53877.21	48948.22	66747.41	31444.36	51389.55	35321.72	34718.29

LN432.991	LN432.992	LN432.991	LN432.992	LN432.992	LN432.991	LN432.991	LN432.992	LN432.991	LN432.992
48779.29	32849.28	57933.83	29258.54	45718.62	58728.32	36919.02	37384.79	50208.05	31176.8
59966.59	39486.74	64197.52	30898.39	46643.77	55321.64	38962.24	30483.69	54169.41	43091.62
49170.58	31215.74	65011.89	30014.21	46942.37	53126.6	38084.41	38021.24	39747.88	32566.52
56874.01	31110.08	70186.44	29465.27	37135.22	46951.76	42940.68	44206.38	47919.4	29801.04
43749.92	38712.86	54535.51	32878.74	42695.87	72005.85	35910.2	37147.42	64048.16	36053.13

LN432.992	LN432.992	LN432.992	LN432.991	LN432.992	LN432.992	LN432.992	LN432.991	LN432.991	LN432.991
40602.52	36256.95	26948.88	73105.72	39745.27	20511.79	36281.52	32329.79	51042.77	52657.45
44013.72	36518.45	25944.37	68469.22	40216.5	17370.82	29435.33	35447.79	67034.07	50641.31
29784.7	38889.4	47626.5	83148.33	45324.43	21531.66	26234.79	31857.54	50539.1	61171.79
42819.57	36802.72	38337.11	61191.24	35594.4	13840.9	23725.95	38526.38	63089.19	47320.45
48590.14	43181.47	23126.58	65874.04	54933.75	22101.77	41121.01	29264.58	48236.04	48741.51

LN432.992	LN432.992	LN432.992	LN432.992	LN432.992	LN432.992	LN432.992	LN432.992	LN432.992	LN432.992
26167.96	34625.82	33327.18	21359.32	32076.15	51630.24	19947.07	31878.84	19473.5	38291.67
29237.31	27527.57	34846.87	16673.53	27979.07	48894.25	18643.15	34695.76	15528.3	38222.58
31371.32	34710.58	32919.16	20861.93	32156.33	55304.98	19534.01	35114.92	23301.28	43340.57
25114.15	32363.63	35057.07	18367.71	27281.39	40178.34	18060.55	30170.26	18896.17	37014.87
24253.65	31191.19	30781.32	17078.15	34410.11	53552.25	15535.09	34064.91	32948.86	37888.9

LN432.992	LN432.991	LN432.992	LN432.991	LN432.992	LN432.992	LN432.991	LN432.992	LN432.991	LN432.992
40333.49	36092.04	38749.86	51688.3	28236.72	34783.6	47838.42	29636.88	38452.88	34917.02
55515.11	41291.66	35413.82	48118.46	22455.77	36366.65	43784.41	33203.41	46843.41	42649.63
43713.11	43099.06	35477.9	54006.15	23276.19	45570.53	48537.91	23387.49	39828.96	46960.63
39643.83	34110.63	31169.7	66704.72	23488.94	47953.28	46410.26	36821.5	37812.14	43732.02
50077.53	37327.23	42547.04	55804.37	24447.75	30221.07	53145.82	26727.67	40142.05	46823.12

LN432.992	LN432.991	LN432.992	LN432.992	LN432.992	LN432.992	LN432.992	LN432.992	LN432.992	LN432.992
36629.84	52842.19	26940.67	42381.72	26183.14	17045.39	35627.58	19666.82	45155.86	20378.07
37792.62	68058.09	23638.82	45994.24	45753.61	19946.84	43232.1	27958.64	50967.93	19894.19
47214.34	53729.97	35694.49	56935.85	38515.03	19552.08	39227.74	26578.72	49267.06	29305.81
34367.05	48585.86	24832.7	42332.46	28611.26	14713.31	27787.49	24140.86	39350.73	27928.28
33461.37	40763.77	18273.44	59127.09	27475.25	14143.55	53625.48	19931.42	36845.45	16522.84

LN432.992	LN432.992	LN432.992	LN432.992	LN432.992	LN432.993	LN432.993	LN432.993	LN432.993	LN432.993
19541.63	27750.49	49490.29	20984.6	34169.26	18543	48129.88	30037.62	44890.96	36027.33
17408.93	33170.15	50003.99	18881.07	31120.18	16419.16	51467.44	30947.58	48326.27	35625.39
21242.45	35968.41	43904.45	29889.94	42179.27	18281.39	50448.13	31493.19	46688.02	39330.8
17121.49	33284.97	64458.67	13538.72	31164.54	16135.36	46673.58	29818.93	45368.44	35500.45
20231.27	36190.82	43015.34	19704.17	31572.44	16314.04	51299.06	28293.37	43468.39	37062.86

LN432.993	LN432.993	LN432.993	LN432.993	LN433.010	LN433.010	LN433.047	LN433.047	LN433.047	LN433.190
20818.87	42060.89	27445.86	24114.34	64623.42	73506.5	69339.78	63484.14	78217.74	15472.82
22805.78	36368.35	26864.91	22355.46	68895.11	74448.58	77904.66	66008.33	81350.33	15341.09
22252.96	39463.77	25036.51	24134.76	74325.53	78500.59	69430.28	67494.19	85870.25	13069.73
20867.23	40096.29	25955.11	25495.11	70053.28	66223.23	76944.87	68560.78	83960.16	16076.02
24781.34	36633.86	24650.96	22034.13	67334.02	73705.14	72561.5	68035.72	86967	16879.13

LN433.207	LN433.207	LN433.207	LN433.207	LN433.207	LN433.207	LN433.207	LN433.207	LN433.207	LN433.208
21525.39	22879.31	32621.42	31815.6	25944.42	18462.79	20185.37	22135.66	27642.34	22107.33
23699.5	22851.59	26786.85	30822.03	36964.08	27049.57	21781.88	20866.46	27075.46	25763.94
22007.41	24030.33	23060.84	29778.42	39501.08	30181.44	22419.19	23115.03	30087.64	21936.68
22530.35	21877.28	26315.3	28627.07	39458.19	24178.74	23415.55	20235.54	28032.65	25264.16
25021.37	20056.94	24386.08	28402.29	39091.27	27271.83	21582.65	21902.62	27402.12	22925.93

LN433.208	LN433.208	LN433.243	LN434.804	LN435.006	LN435.006	LN435.007	LN435.006	LN435.007	LN435.007
23015.03	30087.31	26444.54	33845.74	39570.99	62472.98	63629.49	72368.89	71501.25	69590.04
24342.06	31899.38	29384.14	22881.64	41738.82	62607.85	58170.69	73522.33	69413.3	68481.19
26136.65	33348.04	27270.63	32112.87	45293.81	88315.79	71276.71	101412	62080.19	69679.04
22945.55	30619.32	25955.27	32796.11	43749.8	66451.57	57674.19	76469.5	67982.29	69027.38
21935.11	32566.13	27075.87	32836.3	44243.85	81872	84902.19	110315.8	66082.16	102776.6

LN435.007	LN435.007	LN435.007	LN435.007	LN435.007	LN435.007	LN435.007	LN435.007	LN435.007	LN435.007
44215.57	61552.5	42319.56	13352.13	44855.05	34566.22	33858.43	45460.71	32724.82	42342.6
42085.64	59161.79	40743.21	12957.8	46513.6	38560.05	35379.65	41725.94	32322.01	42798.09
52504.28	81820.48	48288.15	15882.61	41993.24	32659.76	32556.01	46017.04	21530.39	37764.87
46714.11	57491.45	43275.98	14553.75	50268.35	36377.25	38252.03	42411.98	34180.02	46044.51
54186.84	66342.41	47896.38	15554.41	40632.05	46684.56	42387.38	39928.08	34683.03	50748.99

LN435.007	LN435.007	LN435.007	LN435.007	LN435.007	LN435.007	LN435.007	LN435.007	LN435.007	LN435.007
30774.84	45866.74	43328.24	22143.22	51162.75	29300.75	62534.14	46237.96	33177.26	40600.34
32895.09	48676.57	46635.04	22041.06	54678.06	31234.25	63995.83	44036.28	35015.96	38279.9
45170.01	58835.94	54118.15	27414.92	65174.39	45722.98	51853.11	59636.62	51925.22	41602.07
28935.18	42553.11	46565.88	22937.55	47191.07	28012.64	59629.48	43469.77	34055.75	39625.66
44731.68	46673.91	49039.31	24284.95	47338.95	34685.94	44803.21	47785.29	30903.11	45927.79

LN435.007	LN435.007	LN435.007	LN435.007	LN435.007	LN435.007	LN435.007	LN435.007	LN435.007	LN435.008
48741.06	29938.52	26295.86	19306.14	23108.43	34515.83	21979.4	35442.56	51658.37	17528.25
46605.85	30612.05	24751.02	22018.2	22704.74	36526.32	20246.26	33510.17	51673.59	18318.49
56723.67	26340.01	43265.29	22364.67	26382.54	26422.18	35745.85	31455.26	93912.62	16604.85
47466.13	30431.45	32247.03	23587.78	26273.95	32156.44	21353.21	34407.59	53235.31	21009.81
50300.2	38479.1	28543.2	20651	23689.84	35956.05	25212.21	58129.56	57782.47	17759.06

LN435.007	LN435.007	LN435.007	LN435.007	LN435.007	LN435.007	LN435.007	LN435.007	LN435.007	LN435.007
53987.9	54243.43	31322.21	54243.86	35490.83	19698.88	35807.47	31722.83	22068.41	62649.63
54422.96	57556.86	30244.75	52637.31	36217.49	21134.52	37718.07	36131.76	22166.01	64603.09
37651.17	61534.99	43941.06	57051.38	31141.37	14827.52	50033.78	40741.84	21172.4	57220.77
48768.48	55655.53	34755.93	51470.94	42099.45	19390.16	31231.82	33400.54	23853.23	61101.11
59812.42	56343.64	29761.01	38966.66	26425.85	22077.03	39800.33	31748.04	17773.61	43771.14

LN435.007	LN435.007	LN435.007	LN435.007	LN435.007	LN435.007	LN435.007	LN435.008	LN435.007	LN435.007
37378.06	25890.71	54294.35	27033.43	19801.17	53021.18	35563.31	18620.65	28807.81	40195.21
35929.92	27875.37	49522.71	27476.07	21139.29	46542.5	34255.94	17326.16	28634.61	43256.47
52651.26	36714.72	55358.63	34872.68	18983.72	49233.93	37599.13	18519.73	28153.56	46332.11
35195.24	26215.61	53478.87	28741.15	21452.44	49376.28	36900.12	15868.06	26352.62	39746.01
39591.49	22485.34	61107.17	28533.98	24255.68	69498.14	38028.53	21781.7	27848.46	56524.88

LN435.007	LN435.007	LN435.007	LN435.007	LN435.007	LN435.007	LN435.007	LN435.007	LN435.007	LN435.007
49987.53	30582.56	45912.71	42365.37	34011.81	43839.08	39098.46	39095.1	30001.52	27749.8
52434.31	33310.54	46379.87	43113.4	33951.27	43172.97	42739.72	35938.58	29723.61	32143.12
56919.8	31156.76	42805.56	57002.72	25888	40551.35	60597.91	45233.14	29114.68	27286.36
48778.95	33165.17	49319.18	38189.6	34615.74	41670.71	42471.63	38559.27	30061.45	27139.64
45390.6	52132.71	53349.65	53976.32	33477.7	45476.89	70806.99	39036.93	37293.4	32202.6

LN435.007	LN435.007	LN435.007	LN435.007	LN435.007	LN435.007	LN435.007	LN435.007	LN435.007	LN435.007
48853.77	32195.58	16978.58	27725.28	18806.69	34190.28	37293.25	57748.98	33721.82	37940.01
50823.4	30380.76	17142.75	32224.55	18855.27	31436.45	37559.02	46047.28	33401.53	39128.25
46921.2	29196.86	12671.05	30271.45	13284.51	29861.72	39130.78	58355.13	30463.85	29129.36
52820.99	28651.05	19626.13	29859.63	17353.29	31810.16	35554.01	47601.38	34543.53	40695.97
81296.23	25015.93	13021.76	33790.31	13283.24	31933.37	34543.29	48114.98	31282.97	38800.09

LN435.007	LN435.008	LN435.007	LN435.008	LN435.008	LN435.008	LN435.008	LN435.007	LN435.186	LN435.186
40196.86	22101.19	33650.18	19602.63	21152.86	54579.03	47200.43	42054.71	29217.58	38080.31
37481.7	23255.31	31664.77	23738.21	19999.83	58955.21	45925.76	40952.11	30454.84	43577.79
24855.15	21099.03	32114.83	14831.83	18710.01	54131.9	48559.77	45044.64	30026.26	39258.97
37082.76	22005.14	35352.78	20953.45	19022.43	58726.3	50925.1	41986.36	29064.66	45216.45
47432.05	14787.48	30341.85	25180.19	18118.59	54458.71	46288.62	40515.56	30664.2	39322.88

LN435.186	LN435.186	LN435.186	LN435.186	LN435.186	LN435.186	LN435.186	LN435.186	LN435.186	LN435.186
52095.1	29452.01	29145.75	34094.26	58566.18	48014.73	35988.07	44852.37	29189.51	38288.67
55879.84	30794.38	30714.31	34525.5	57642.38	43952.41	32524.13	45362.48	32910.54	34374.35
55111.28	31858.48	34186.94	32371.32	59433.18	43128.76	37534.15	50073.21	34594.81	36722.84
60480.18	34268.95	31047.99	33810.5	57059.61	40998.43	29969.95	49482.66	33182.69	44717.16
57232.39	34689.51	33073.49	36808.14	60512.34	42126.95	38403.9	55207.76	28808.62	41640.78

LN435.186	LN436.059	LN436.229	LN436.805	LN436.818	LN437.004	LN437.004	LN437.005	LN437.004	LN437.005
21759.55	8175.076	35888.11	15352.95	18028.56	29304.46	58887.83	21471.23	37433.38	29127.83
25244.18	9335.816	33423.78	14424.44	17132.7	20366.37	46062.49	36851.97	41529.75	33243.92
26674.94	14738.35	28844.17	11017.15	16951.14	32538.88	61327.42	20976.93	36596.91	27039.78
24028.85	18337.78	22114.93	15191.58	15358.29	28321.22	56366.13	21302.34	38520.23	27081.89
25499.2	21420.02	26813.31	15359.87	16707.74	32896.23	55938.48	21150.39	38920.45	29088.08

LN437.004	LN437.004	LN437.005	LN437.004	LN437.004	LN437.005	LN437.004	LN437.004	LN437.004	LN437.004
28509.59	59666.71	44527.49	58358.99	55445.62	30222.59	51295.27	60042.02	42673.44	53202.65
33064.27	63968.22	43885.58	55801.91	56094.64	26967.06	33438.57	58657.26	54165.69	53438.38
28950.09	51237.84	39073.95	56188.47	58127.23	29718.85	58785.06	60488.92	37276.4	49708.32
26533.82	55131.91	40528.86	52987.51	51876.95	31409.38	49090.76	56466.02	35737.01	51040.07
27329.33	52099.9	38606.49	56371.46	51836.87	32445.11	46627.82	56325.57	36919.75	48681.09

LN437.004	LN437.004	LN437.005	LN437.004	LN437.003	LN437.004	LN437.004	LN437.004	LN437.003	LN437.004
35414.87	35246.77	22238.83	43692.06	78952.33	27791.24	30554.66	15537.21	60201.58	36764.91
27198.83	29207.26	18446.97	30070.84	77611.68	25009.39	25271.02	24082.69	48148.12	20399.59
33752.68	37504.34	19068.17	39470.52	72219.64	31109.78	27129.2	15052.43	52433.21	34581.72
33037.25	34270.59	21601.07	38028.18	74611.19	29246.95	30862.13	13704.51	53366.96	33277.16
34231.99	32716.91	17887.55	37997.19	71469.26	28761.49	27609.65	15726.77	56145.31	34381.65

LN437.004	LN437.004	LN437.004	LN437.004	LN437.004	LN437.004	LN437.004	LN437.005	LN437.004	LN437.004
27012.15	50926.24	38484.66	44829.3	29649.18	51486.96	60649.04	40166.49	27104.75	27424.66
25720.43	45599.34	39923.17	42829.89	20782.22	56657.76	57180.64	24034.01	24350.05	34076.42
24901.01	46081.35	43176.92	40798.31	31060.67	47811.75	56461.04	37000.02	26470.59	30744.41
26119.91	52108.88	37907.6	39502.82	30560.8	49565.43	54830.18	36291.43	25052.56	27221.69
26753.22	46739.49	41497.24	40767.64	31233.29	44550.72	52345.27	38108.47	25137.08	29130.01

LN437.004	LN437.004	LN437.004	LN437.004	LN437.004	LN437.005	LN437.005	LN437.004	LN437.005	LN437.005
74415.6	51653.27	36976.23	37084.13	38798.25	45057.11	28035.59	46302.46	14279.59	22456.48
55446.97	53859.54	31518.2	25580.5	40865.14	28412.65	32980.4	54105.11	14953.12	23417.12
68023.55	44959.65	36254.32	35647.9	40334.7	39667.4	29123.18	48256.07	13315.7	22950.31
75098.02	45646.66	33332.33	36128.46	41865.44	41039.32	30817.37	48576.81	15744.46	23366.84
76732.88	48920.54	35300.06	31260.68	39503.13	40334.87	29467.6	45299.23	14475.84	22991.13

LN437.004	LN437.004	LN437.005	LN437.004	LN437.005	LN437.004	LN437.004	LN437.004	LN437.004	LN437.004
77378.35	37970.77	22093.95	26818	19613.64	40164.49	46747.07	41110.49	41064.64	39149.41
63994.45	31223.49	18440.24	23941.11	20034.13	57567.86	59390.76	39107.36	27549.16	41245.13
68524.92	37765.92	22868.19	27882.73	17161.9	40366.2	49839.89	46706.2	37517.92	38321.06
67601.14	38802.3	23204.34	26963.56	16644.45	39721.3	44243.34	42716.08	40964.77	39034.2
73380.93	44277.2	21295.86	31049.77	16310.77	36629.57	39918.24	45633.71	40446.05	37131.28

LN437.005	LN437.004	LN437.004	LN437.005	LN437.005	LN437.004	LN437.004	LN437.005	LN437.003	LN437.004
30850.29	53378.35	37040.53	16793.02	31291.98	54111.98	51467.14	24545.61	84445.62	25621.88
30899.32	41991.01	38542.54	14121	28508.12	39927.14	39620.44	22678.76	47629.06	24895.97
28550.6	52527.99	35243.73	15342.99	29973.8	53289.48	50456.66	26328.1	78632.66	23370.99
29425.55	49743.49	36115.38	16917.81	29198.24	55682.82	53244.05	26666.7	81337.69	23045.1
29637.61	53489.42	38417.36	15916.17	29205.49	53440.76	51580.97	24902.36	72447.85	21709.1

LN437.004	LN437.004	LN437.004	LN437.005	LN437.004	LN437.004	LN437.004	LN437.004	LN437.004	LN437.004
19709.8	68532.25	37851.08	20872.82	21953.4	45549.62	72814.87	43659.34	55440.04	30410.84
29571.3	64050.49	36714.25	18530.88	20264.89	55638.97	65683.27	53607.57	48079.4	31491.48
18483.73	62188.32	38184.35	19095.76	23565.18	40850.47	71315.36	40457.89	55110.48	29495.58
19471.44	57760.27	36377.04	18895.65	24626.79	43630.06	66034.46	43650.08	52225.3	26531.97
20379.43	58211.53	34787.82	18927.59	22241.6	44767.86	66550.83	40565.4	50572.05	25891.71

LN437.004	LN437.004	LN437.004	LN437.004	LN437.005	LN437.004	LN437.005	LN437.004	LN437.004	LN437.004
27367.81	73993.69	51664.16	42070.03	18772.76	46222.04	30603.78	52179.9	51347.96	40029.11
18740.11	68415.62	48114.86	49303.84	12355.48	43661.37	29935.07	39985.75	38840.07	25097.2
26384.03	69722.57	55968.16	39777.25	17931	49019.17	33556.31	52314.98	50192.79	37001.52
24522.93	63041.96	49595.44	45709.75	17077.44	46832.38	34839.18	51611.83	49208.61	37569.36
23330.5	69153.38	44219.47	42256.87	16919.62	47405.06	29956.6	56766.71	57754.3	39867.53

LN437.022	LN437.022	LN437.022	LN437.022	LN437.022	LN437.022	LN437.022	LN437.021	LN437.022	LN437.022
54811.87	78439.28	77903.26	76253.44	61288.37	44079.04	29046.44	101353.9	42129.22	65869.67
55525.31	77908.98	84402.55	75606.6	61787.91	42421.96	31042.72	98732.46	44915.11	61967.61
54642.34	78689.18	76643.6	77826.58	69209.21	43020.21	28911.53	97825.95	40566.76	65584.05
57041.26	80096.93	78596.49	79941.88	61518.37	42099.47	30811.83	99409.99	40817.33	62527.35
61548.89	76841.51	80682.84	75391.88	59030.42	43109.8	30027.15	98431.3	45280.09	62997.66

LN437.021	LN437.022	LN437.022	LN437.022	LN437.201	LN437.202	LN437.202	LN437.202	LN437.202	LN437.202
56292.73	69896.48	32703.64	51349.82	42893.34	35543.66	26389.82	35976.95	40377.96	25269.15
58789.07	67127.95	27756.78	55547.99	40029.82	38727.01	26742.07	40854.48	40938.08	25930.68
53090.48	69226.48	29410.96	53111.77	43758.29	38577.52	26276.91	37810.24	40922.54	26526.94
50120.22	71291.76	28597.02	53096.94	41796.35	41597.95	25604.12	38017.36	41306.8	28303.78
63840.39	68587.16	27225.11	51487.06	39138.28	35026.41	25651.4	37892.53	41186.74	22873.41

LN437.202	LN437.202	LN437.202	LN437.202	LN437.202	LN437.202	LN437.202	LN437.813	LN438.785	LN438.856
22169.68	24855.48	46946.22	31302.76	38393.02	46202.93	46241.63	12063.75	20404.74	14541.15
22326.14	27640.25	41076.68	32278.16	38152.86	52250.11	41303.14	13599.56	11663.21	15845.75
22198.09	28259.37	42235.78	34348.16	37734.74	47175	41525.12	14900.63	15181.02	16120.86
23586.12	25959.16	46379.8	30417.11	41455.1	44480.5	43512.49	14389.27	19369.3	15873.91
21325.87	25694.47	44118.48	33049.89	37719.57	45873.07	40000.24	16882.09	19878.08	13937.48

LN438.856	LN438.856	LN438.856	LN438.856	LN438.856	LN438.856	LN438.856	LN438.856	LN438.856	LN438.856
24731.18	13659.11	21252.07	16135.93	14968.08	20364.19	15290.02	22669.54	22330.74	17400.84
22954.55	13926.52	17875.96	17425.17	16202.73	21527.35	17999.8	21399.08	22968.02	18108.7
25639.34	14930.36	17666.04	16971.16	13915.58	21058.63	15304.79	24306.1	23378.75	18123.45
24900.27	13011.26	18415.37	14655.3	17016.83	18385.5	17779.39	22429.62	23294.06	18485.92
23915.2	12233.04	18045.15	16508.89	16167.6	17748.31	14878.53	21235.23	23562.4	14612.77

LN438.856	LN439.000	LN439.001	LN439.001	LN439.001	LN439.001	LN439.001	LN439.001	LN439.001	LN439.001
22295.41	69332.1	33470.7	34752.73	25770.17	23301.58	39362.09	32176.08	43307.58	42826.34
19470.98	61771.65	42220.07	37710.25	31792.45	24814.94	44031.33	32853.54	48349.7	44690.99
20022.22	69784.89	37431.83	33818.13	28896.6	24423.76	37209.97	33099.55	39810.01	40458.88
18124.26	63836.96	32515.52	29765.76	28240.25	23871.93	35939.84	32277.51	38983.87	45800.98
19400.31	70973.25	33475.15	31281.58	26373.64	23453.12	38563.65	30626.96	39843.77	43479.73

LN439.000	LN439.001	LN439.001	LN439.001	LN439.001	LN439.001	LN439.001	LN439.001	LN439.001	LN439.001
39293.41	22998.25	27004.79	25975.93	31307.86	26974.83	21520.14	42913.84	18910.38	27340.73
42617.88	25519.67	30085.04	27316.12	35733.55	26910.69	23033.93	43387.27	20385.98	27758.56
38232.62	23903.05	22865.75	25451.6	33063.54	23683.94	20621.25	44917.51	19967.73	25526.8
35968.98	21990.46	24251.17	24076.97	32519.49	28397.52	21389.3	41017.44	18423.76	26158.91
39520.51	24196.28	26997.87	26057.93	30704.37	27053.76	23566.72	40396.63	19056.68	24049.31

LN439.001	LN439.001	LN439.001	LN439.001	LN439.001	LN439.001	LN439.001	LN439.001	LN439.001	LN439.001
17695.14	23212.69	15681.31	18911.52	18777.47	43294.26	22625.34	30791.83	22240.3	24529.83
19880.23	24349.14	15443.65	21054.72	17729.32	44955.65	23034.5	32536.21	25369.97	27360.27
16912.39	25127.84	12503.12	19091.99	16579.61	45390.15	23737.65	34810.84	24544.58	27306.07
18077.34	24351.69	14073.36	19766	16800.11	42463.62	26148.37	29815.06	22380.96	24578.18
16181.08	20910.53	15813.38	19288.77	16435.84	42565.69	22184.24	31945.22	19216.76	23849.43

LN439.001	LN439.001	LN439.001	LN439.001	LN439.001	LN439.001	LN439.001	LN439.001	LN439.000	LN439.001
25845.3	25559.04	37052.18	33572.21	24784.19	50134.7	24975.11	31403.18	37126.51	38916.08
27030.22	25611.34	39396.79	31819	30113.52	50298.39	31274.79	31633.44	39410.58	39703.57
26917.34	24730.57	38764.09	30911.53	27954.9	53745.27	27977.77	29329.7	39867.46	36050.97
25081.86	29117.07	34420.66	33127.33	29808.79	52192.78	26956.46	28365.26	34891.89	36904.9
24622.09	29140.09	31637.3	29235.61	25291.64	54646.16	27723.57	30964.4	37713.81	35303.48

LN439.001	LN439.001	LN439.001	LN439.001	LN439.001	LN439.001	LN439.001	LN439.001	LN439.001	LN439.001
49493.66	30898.4	24922.57	25256.94	36022.33	14172.37	35998.79	35232.42	44496.5	23325.62
48307.42	35703.63	30621.44	20721.17	37937.6	16803.66	40952.16	33732.11	47974.7	26999.01
42603.89	32542	25787.63	23595.35	35977.4	12526	36807.53	33117.06	44613.94	24626.41
47561.84	36075.4	23916.77	21325.33	33765.65	14753.1	38764.2	32903.67	43788.51	24427.06
48604.76	34067.58	26444.81	24343.95	33544.94	12159.25	34841.44	32953.77	45819.42	22720.28

LN439.001	LN439.000	LN439.001	LN439.001	LN439.001	LN439.001	LN439.000	LN439.000	LN439.001	LN439.001
34056	71697.35	23882.59	32930.34	22777	32137.04	35131.06	43749.21	39771.04	53385.26
35948.08	72537.49	26554.64	34718.1	22036.85	36793.15	42116.2	41927.8	40638.09	54619.01
33374.16	71167.26	25870.03	30189.11	20203.62	32967.04	36165.08	39382.74	42873.8	48798.61
37054.91	69772.49	24991.9	27444.34	19056.4	28704.24	38401.45	40183.75	40300.39	49163.43
33344.74	73231.83	22433.2	28837.51	20727.83	31335.78	37232.95	40435.14	35311.29	51878.59

LN439.001	LN439.001	LN439.001	LN439.001	LN439.001	LN439.001	LN439.001	LN439.001	LN439.001	LN439.000
39933.45	22609.7	17750.13	24676.36	33488.92	29895.2	11924.53	46217.63	34569.25	16622.54
44207.89	24968.83	20492.82	27232.22	36996.26	28659.93	15199.46	51742.51	38736.06	15114.08
40188.96	18717.64	19475.59	24332.68	33501.88	29820.74	13609.82	45535.79	37445.61	13217.75
42133.35	22901.26	16883.63	23566.55	33207.39	27760.13	13930.35	51269.63	38506.32	15327.58
38978.98	21879.74	18630	24715.91	31825.16	28600.56	13359.71	42915.82	33362.26	17196.49

LN439.001	LN439.001	LN439.001	LN439.001	LN439.001	LN439.001	LN439.001	LN439.001	LN439.001	LN439.001
57062.31	22721.49	32485.65	37993.59	19048.21	34388.46	16232.04	34712.82	39752.9	28992.71
57284.67	23171.06	32128.82	41841.08	25503.49	38064.52	17572.08	29504.02	42977.49	31988.73
49390.99	22217.95	29797.19	36108.27	18432.27	32141.98	18013.25	34945.84	37927.83	29328.98
52573.39	23122.64	32856.55	35032.56	20984.45	36296.53	18036.73	34274.98	37647.83	26622.94
54175.39	23697.69	29900.3	40274.07	20128.03	32323.87	13772.4	31959.09	36295.88	29102.43

LN439.001	LN439.001	LN439.001	LN439.001	LN439.001	LN439.001	LN439.001	LN439.019	LN439.019	LN439.019
17169.62	20570.41	19802.97	20545.59	25200.44	35130.57	46750.28	38293.49	31687.29	39980.58
24714.08	26099.67	19492.03	22313.2	24790.56	38421.22	55567.75	34715.88	38458.83	36513.68
23893.1	22732.44	20972.26	21104.47	23050.75	36503.01	52214.99	35435.32	33996.87	39059.18
24024.75	21955.98	19429.7	20047.93	23182.85	37083.71	50765.54	35286.52	38002.18	39405.67
23129.1	21951.99	20344.65	17744.96	24453.53	30095.19	50117.98	35742.16	31666.26	35308.98

LN439.019	LN439.019	LN439.019	LN439.019	LN439.02_	LN439.02_	LN439.019	LN439.019	LN439.019	LN439.039
55426.49	56712.06	36329.1	40081.1	47606.99	48122.35	64084.24	65388.5	49189.43	18072.77
54978.94	56869.37	28363.9	45030.74	50443.1	51441	64672.21	66201.11	46989.43	46436.02
48017.04	56655.45	28312.3	41784.21	46470.22	48694.11	55439.1	69611.62	50186.4	42430.66
53002.05	54395.55	31835.28	39815.98	44153.8	46973.27	63401.69	62035.28	48574.99	40578.06
51014.58	47322.12	28144.62	42765.07	43475.47	46792.15	59352.52	60565.12	46001.47	41072.42

LN439.039	LN439.040	LN439.04_	LN439.039	LN439.807	LN440.815	LN440.997	LN440.998	LN440.998	LN440.998
14261.01	17594.94	51213.75	55582.24	29668.99	13378.82	93291.24	67670.82	17448.6	69439.44
33030.05	34795.96	51854.24	47369.11	23919.51	11300.22	114174.3	67710.06	27556.07	72253.77
32228.93	35912.56	55553.65	54016.96	32582	11334.15	103741.6	58039.2	23185.92	55952.52
35047.77	37262.84	53292.82	49750.64	33680.62	13503.79	84808.54	83709.32	20684.65	86733.95
32097.2	33859.04	56430.83	56508.5	32259.78	13423.7	104028.7	69427.31	19290.37	91269.66

LN440.998	LN440.998	LN440.998	LN440.998	LN440.998	LN440.997	LN440.998	LN440.998	LN440.998	LN440.998
64241.72	65279.95	21443.05	60433.07	65967.74	93802.81	57744.09	100033	78849.23	59652.18
61656.57	48573.85	24833.11	48877.37	91464.99	102239.1	43852.74	85474.43	93668.65	38383.06
58036.08	58771.88	21102.4	73652.52	65326.38	119334.1	48045.91	108943.8	83184.09	49604.31
80507.53	65920.76	32604.08	57764.21	83207.94	118896.3	65020.95	86402.42	73687.36	52741.52
62841.67	51609.73	34067.35	56431.08	73169.74	142726.5	54036.71	87813.09	104026.1	56373.46

LN440.998	LN440.997	LN440.998	LN440.998	LN440.998	LN440.997	LN440.998	LN440.998	LN440.998	LN440.997
87015.4	70869.82	61613.34	110122.4	60429.03	96887.54	75581.52	26787.67	33684.02	122834.4
101906.2	81210.37	64473.64	84507.41	120386.5	109549.6	71836.7	35644.76	35817.66	91134.15
77894.34	72742.35	85762.56	56736.95	133977.4	91967.01	71954.19	50218.5	23661.96	88826.04
90542.23	80155.1	93478	76408.03	100086.4	109601.2	97673.18	47764.9	44419.44	90141.26
92831.27	97204.02	86994.33	58222.31	84281.41	144933.5	75662.31	40987.24	21927.58	90214

LN440.998	LN440.998	LN440.998	LN440.998	LN440.998	LN440.998	LN440.998	LN440.997	LN440.998	LN440.997
35136.54	77446.37	54412.78	55231.72	53510.17	68433.37	77782.45	103621.9	95859.43	165281.5
27998.79	42375.02	43979.84	62912.44	36539.18	72686.67	67001.44	136127	70131.59	136637.2
24749.64	40831.49	63797.8	54950.01	43588.33	74489.45	84056.5	134976.5	68035.27	132306.1
33108.02	46114.13	56368.75	41664.33	49912.45	60010.92	77600.25	128568.7	99832.79	144780.5
26149.63	65038.15	57660.74	71243.73	42057.99	86879.41	64095.64	130539.6	68884.25	107717.8

LN440.998	LN440.998	LN440.998	LN440.998	LN440.997	LN440.998	LN440.998	LN440.998	LN440.998	LN440.998
65987.17	53585.75	90595.05	31138.65	122490.2	76754.95	47482.52	71434.89	95578.86	53296.92
48401.12	62762.21	83717.05	43035.18	116855.7	49130.49	54382.09	82961.6	79878.83	68777.11
54842.44	70589.47	95592.04	49013.47	112557	72822.18	52064.1	46342.73	92597.75	58698.27
37587.9	62038.08	136414.9	57152.93	116477	49307.25	47508.2	78108.51	137385.1	70483.69
73462.78	70081.58	85383.16	49268.43	124371.1	64514.68	67180.73	39728.24	71831.95	48470.73

LN440.998	LN440.997	LN440.998	LN440.998	LN440.998	LN440.998	LN440.998	LN440.998	LN440.998	LN440.998
111302.9	90177.8	107426.3	28431.71	60891.78	59844.2	102524.1	83674.32	97764.31	31108.57
102745.3	118919.4	116828.6	29649.08	90219.72	56886.04	100836.8	91472.64	95529.28	34170.88
97270.05	91557.87	81714.84	28914.51	75681.01	66652.01	91723.05	111495.9	102037.2	34143.47
79712.32	120977.2	112783.1	23001.69	88527.54	56554.51	124646.2	153626.2	93314.21	32877.63
129304.5	125899.4	78908.07	32057.55	82894.33	87912.29	107544.6	146066.6	75109.61	52252.17

LN440.998	LN440.998	LN440.998	LN440.998	LN440.997	LN440.997	LN440.998	LN440.998	LN440.998	LN440.998
31355.2	80879.6	89555.71	79723.81	176181.8	85862.6	58324.75	52066.07	56647.9	52374.13
32844.24	104250.9	57873.23	75471.82	111642.7	94961.08	71058.87	42857.78	81201.16	64048.17
32351.45	94104.2	91313.45	79263.84	123067.9	119847.6	64511.33	52380.65	71854.95	80120.52
32271.04	71408.61	66437.1	108319	123465.1	107952.6	56195.36	47111.43	60213.93	67104.04
33697.18	86411.89	103427.2	75261.35	110960.2	78434.99	46436.68	50226.06	46865.06	46666.21

LN440.998	LN440.997	LN440.998	LN440.998	LN440.998	LN440.998	LN440.997	LN440.998	LN440.998	LN440.998
43061.14	110887.4	97388.03	38556.39	31260.73	34881.64	110058.4	149335.7	99258.57	39482.56
47690.3	93354.51	67735.61	25341.57	25079.87	29475.08	94475.5	95679.71	118183.7	46470.37
54578.79	190045.5	61950.36	23765.43	24427.31	31328.97	113166.8	108002.7	87325.51	49214.34
67205.6	100774	87479.86	27134.5	25179.4	20007.98	68316.74	89786.83	80542.92	42450.25
50385.14	86469.65	112299.8	26408.21	43710.15	30462.48	91603.56	92926.15	69854.06	39387.79

LN440.998	LN440.998	LN440.998	LN441.807	LN443.150	LN443.247	LN444.356	LN444.429	LN444.785	LN445.030
65129.35	70859.6	67047.79	20097.53	16897.29	164511.8	31370.3	36203.29	16602.37	45199.44
73544.25	87342.13	44586.32	17485.47	16668.29	185370	11980.62	38785.94	14588.17	46939.2
64212.33	74381.03	39573.27	17214.33	15537.01	280195.7	35327.17	38605.02	17726.29	45152.5
46548.23	73529.95	50168.59	22766	15501.23	346690.4	10545.87	34288.67	14797.47	44857.41
74469	75756.75	54076.17	18897.68	16392.18	380798.8	28299.03	31953.35	20443.08	44258.72

LN445.03_	LN445.030	LN445.030	LN445.030	LN445.030	LN445.239	LN445.243	LN446.088	LN446.160	LN446.834
48445.66	46937.69	38467.28	53156.59	64581.65	31654.08	30401.58	5434.102	37322.48	34631.17
50102.97	48196.73	40347.38	48667.29	63373.54	26893.55	30566.83	6173.154	44388.46	33177.21
48762.85	46893.59	38972.3	48719.46	61661.77	25181.82	37564.26	9678.09	47063.76	38273.26
48355.72	43862.52	35415.75	49676.46	57900.34	19795.22	40400.29	12012.46	42699.08	45797.66
43674.72	43798.79	37383.9	51080.74	60397.91	20578.18	46144.58	13213.32	44212.46	33933.49

LN446.905	LN446.905	LN446.905	LN446.905	LN446.905	LN446.905	LN446.905	LN446.905	LN446.905	LN446.905
123005.1	59606.05	47402.52	119336.1	198474.4	122712.4	121307.1	82028.38	103133.5	52380.65
147407	57960.32	49874.28	119273.8	141275.7	130806.1	135405.3	59620.17	141192.4	58485.95
151020.6	55427.26	45280.67	118225.9	122896.6	133512.4	139175	64369.66	116921.8	60676.58
100671.4	58727.92	44589.28	164668.4	165338.4	134982.2	117598.9	75768	113162.9	49948.88
104251.5	44824.57	45323.98	119253.5	107686.7	149561.3	140317.3	95135.36	116889.1	57670.94

LN446.905	LN446.905	LN446.905	LN446.905	LN446.905	LN446.905	LN446.905	LN446.905	LN446.905	LN446.905
167399.9	141081.9	138578.9	68547.94	134739.4	91776.82	134622.6	136525.5	128206.7	148150.2
113123	169046.6	146860	70539.23	121263.1	88753.27	94097.48	110484	120635.5	168883.9
117012.2	167755.3	141139.1	66779.32	114799.8	93441.54	116870.2	109496.2	131212	130820.2
123867	133134	99766.65	59371.66	113327.3	116926.1	118630.3	106587	153265.2	129285.9
112889.6	220819.2	102461.2	69490.05	115879.8	84781.04	106960.5	104089.3	125039.9	196385.5

LN446.905	LN446.905	LN446.905	LN446.905	LN446.905	LN446.905	LN446.905	LN446.905	LN446.905	LN446.905
159845.3	84841.38	175475.1	139578.1	118573.6	119542.2	122611.2	143065.9	107192.8	122098
159438	98357.48	134414.7	115605.4	92221.52	134027.6	132214.5	133065.3	110725.3	103013.2
161213	78499.58	107591.2	106603.8	120412.2	126094.3	123006.2	130269	91910.84	101271.3
159894.2	96936.98	108798.5	122231.4	107028.4	129561.4	186723.1	145387	113672.3	94385.72
117224.1	89070.42	93262.7	108096.8	108890.3	116721.4	112998	122878	98570.48	102012.9

LN446.905	LN446.905	LN446.905	LN446.905	LN446.905	LN446.905	LN446.905	LN446.905	LN446.905	LN446.905
153887.5	152511.1	118752.8	106731.6	120321.8	138175.7	114524.4	126595	147182.8	157416.8
191695.1	103193.7	118284.6	98300.95	173322.9	98598.44	134591	107722.9	123006.2	133405.1
132323.2	113352.6	113876.9	100896.5	124220.4	133843.1	119968.6	88620.48	113235.2	127737
143283.5	111275.8	120482.7	98017.46	128168.1	83654.92	107411	127926.2	105596.2	122981.3
176470.3	102142.9	122263.4	106535.1	154847.6	118518.9	114469.5	88717.12	123369.7	85239.62

LN446.905	LN446.905	LN446.905	LN446.905	LN446.905	LN446.906	LN446.905	LN446.905	LN446.906	LN446.905
151354.9	101255.9	138466.1	121814.9	126644.8	47229.3	71669.85	125796.4	54476.53	126959.6
116945.1	134401.5	99066.69	115541.1	126052.4	49199	68355.65	139082.4	36092.87	120764.1
117912.8	130085.2	86559.02	122315.5	115964.4	45985.01	66936.19	152665.7	32111.37	115569.7
111466.2	149491.9	88216.99	136900.3	124278.5	38818.26	67540.97	150142.5	46007.7	108829.6
115618.5	122824	122697.3	111719	139295.6	46196.64	66795.48	137314.2	34871.12	106359.6

LN446.905	LN446.905	LN446.905	LN446.905	LN446.905	LN446.905	LN446.905	LN446.905	LN446.905	LN446.905
143080	78287.78	117644.3	58552.21	145678.5	33997.92	34782.13	109342.4	47672.01	131057.5
152425.6	94796.32	103597	63821.28	150065	29229.22	29740.54	107271.5	49127.69	140365.2
153815.4	98839.19	98092.97	60372.04	144537.4	31563.14	28823.18	108471.9	46213.66	141395.4
122483.3	96607.35	106527.9	57192.37	141570.7	37791.48	32704.57	100710.2	47591.13	129590.1
133626.4	85890.14	96433.34	83668.29	143233.5	27754.91	27677.31	70390.9	56360.81	137752.2

LN446.905	LN446.905	LN446.906	LN447.148	LN447.148	LN447.163	LN448.441	LN449.166	LN449.165	LN449.165
29797.19	29682.73	60423.22	23037.31	33279.6	13650.45	123580.1	26615.48	42915.66	53364.97
29849.69	29729.99	61020.62	22801.79	27153.58	18453.79	126474.4	29812.03	46814.51	49600.74
29174.64	28947.47	63711.81	22984.4	31088.45	15528.52	114110.9	35782.12	39717.24	46671.09
28068.77	28109.85	58721.15	23582.64	31086.84	9268.853	110644.4	29161.72	42930.46	47299.33
26178.56	26008.39	54781.91	22408.17	27981.23	16691.73	100844.4	34532.66	36247.45	46350.83

LN449.202	LN449.202	LN449.202	LN449.202	LN449.791	LN450.314	LN450.314	LN450.315	LN450.315	LN450.315
34356.74	31527.71	46012.75	42967.23	31908.61	19756.37	19882.99	24738.13	22680.42	27672.5
34580.06	38082.24	43600.5	47368.48	24691.06	23505.24	15089.33	28365.81	23547.14	26322.13
33961.74	32349.79	41697.53	40047.24	17598	21986.83	16211.42	27072.69	21009.69	25593.83
30775.3	37158.31	43966.91	41004.56	22284.44	22814.48	19335.88	29744	20998.62	27629.34
34032.31	31486.99	36306.26	41790.98	22654.62	20187.57	17133.32	27160.65	20257.19	28881.7

LN450.315	LN450.839	LN451.001	LN451.791	LN451.841	LN452.838	LN452.922	LN452.922	LN452.922	LN452.922
25981.9	53420.91	50546.23	24636.54	23226.36	23364.29	487828.2	270785.9	276752.5	288592.9
21148.69	48837	51244.12	20918.6	26074.24	19675.34	312144.5	213031.4	262298.5	352535.3
24184.45	51489.72	46929.61	23890.97	27417.77	19281.63	225188.7	247561.2	280605.2	305028.1
24546.91	71870.97	46842.52	26818.19	25845.05	25741.01	300705.8	248225.2	279595.3	310196.1
22079.76	75292.66	50720.72	34419.55	24198.66	24878.85	251241.5	235747.8	271660.6	296210.8

LN452.922	LN452.922	LN452.922	LN452.922	LN452.922	LN452.922	LN452.922	LN452.922	LN452.922	LN452.922
255462.6	178673.7	200526.3	167688.7	152405.3	202890.4	183580.9	114736.7	156894.5	217680.7
251209.9	188940	196602.2	177067.6	143975.6	211535.2	184357.5	100833.7	143984.3	205689.4
249361.2	183951.7	196199.4	167113.4	141515	204910.2	182576.1	107712.2	142958.3	221507
250670.3	183950.8	205596.6	178734	141947.8	204473.5	187449.4	107145.9	149943	194433.5
239563.8	165371.3	195992.6	174700.9	145867.8	195361	173493	101584.9	143289.6	205850.4

LN452.922	LN452.922	LN452.922	LN452.922	LN452.922	LN452.922	LN452.922	LN452.922	LN452.922	LN452.922
165824.3	142580.4	119055.1	139233.5	139250.1	103626.6	217747.9	103526.4	85257.8	163216.3
169300.7	134076	125099.1	149564.6	143044.4	106511.7	229922.4	100634.9	89696.96	178040
159834	139358.5	121810.5	139255.4	140723	104454.2	212220.4	102058.8	84990	131225.5
164084	138574.1	114374.2	127466.9	146826	88899.76	216739.1	94458.74	83670.33	134057.8
162835.9	131292.7	108743.3	136169.2	135685.1	98320.14	209576.9	100788.3	86674.87	121048.1

LN452.923	LN452.922	LN452.922	LN452.922	LN452.922	LN452.922	LN452.923	LN452.922	LN452.922	LN452.923
92429.68	140124.9	326551.9	212333.3	195093.6	276429.2	112310.7	87154.1	298541.2	217706.7
87867.16	145967.1	192940.1	190331.8	299243.1	211653.8	80380.13	134699.3	293994	205588.7
73623.82	153078.1	185381.4	267405.2	304597.7	210181	73433.95	83545.68	356305.8	221018.7
93203.95	129326.5	171186.3	275484.1	271453.5	236807	75556.01	88794.42	278022	223854.5
81211.19	132912.8	230175.3	330181	264270	238749.6	69581.54	71970.82	420585.2	199018.4

LN452.922	LN452.922	LN452.922	LN452.922	LN452.922	LN452.922	LN452.923	LN452.922	LN452.922	LN452.922
415752.4	231303.8	183895.5	356350.5	181807.3	241195	199023.4	221003.2	215159.7	265187.8
355413.9	237858.2	173799.1	344738.3	189530.5	252727.4	218181.1	223509	202337.5	254515.7
341391.4	238210	143964.6	333878.5	263549	233476.7	212496	221590.1	212882	265491.5
328787.5	219789.7	178580.7	341702.9	189110.2	239881.3	183148.1	202571.3	198411.1	256328.5
366946.9	291768.4	174277.7	324323	180574.7	211550.8	182316.6	219441.4	190330	263044.1

LN452.923	LN452.922	LN452.922	LN452.922	LN452.923	LN452.922	LN452.923	LN452.923	LN452.923	LN452.923
95899.34	215978.3	343639.8	206549	214577.3	180712	215350.9	118188	169406.7	244137.3
98562.63	221267	380046.5	209979.6	217764	165075.7	217778.9	116763.8	169106.9	239827.2
80235.54	210692.9	356459.6	201106.8	223226.8	152422.4	205368.2	107455.8	152650	262825.7
89076.33	199284	332536.4	211556.9	240147.2	161799	219136.7	104787.7	155733.6	270814.6
90154.62	186208.4	335141.9	201144.1	209275.9	161977.3	195911.3	112382.2	164641.8	265021.9

LN452.923	LN452.923	LN452.922	LN452.923	LN453.178	LN453.234	LN453.785	LN454.458	LN454.793	LN454.995
165573.4	332150.3	187858.8	109886.6	80457.83	78248.03	92046.15	169935.5	35750.94	72663.41
165447.8	329387.7	186404.7	113505.8	84183.13	71894.19	93404.69	176513.7	28997.18	74266.71
170573.8	278671.8	190002.8	114924.3	96636.84	67284.44	90265.19	171394.3	35933.35	73823.7
180922.1	301854.8	181847.7	114345.3	72370.47	82856.51	116449.8	160384.9	37942.25	72960.73
184530.1	281816.4	178055.4	111072.7	78071.66	63952.04	157215.9	143513.3	38274.36	74940.69

LN454.994	LN454.995	LN454.995	LN454.995	LN454.996	LN454.995	LN454.995	LN454.996	LN454.995	LN454.995
77265.97	26724.73	43003.86	85764.6	77736.76	37402.94	65972.23	31467.64	44982.44	30780.82
81904.46	27603.6	42650.38	91078.52	73725.35	34806.53	64832.48	33418.6	43055.48	27782.12
73863.21	27923.42	43116.08	85454.31	72683.02	40467.61	65475.02	32464.42	45153.1	34686.69
85011.3	27619.49	45009.62	85195.56	66832.36	33551.67	64106.32	29837.56	42796.02	32779.24
86045.03	25993.76	43465.43	86450.38	75340.38	36598.35	66968.09	28699.66	44529.78	30400.35

LN454.995	LN454.995	LN454.995	LN454.995	LN454.995	LN454.995	LN454.995	LN454.995	LN454.995	LN455.012
25993.93	35528.75	52755.39	60914.18	18452.35	83028.4	24409.3	22020.14	99494.38	82656.88
24776.71	35482.71	55190.06	60780.25	16933.5	97224.54	23733.75	26550.38	96547.32	86088.59
23948.47	35079.9	51552.66	61925.77	17578.44	82081.9	21967.62	23645.51	99365.14	80498.91
25481.64	38620.74	50405.74	61665.1	16714.31	78575.11	23964.86	23807.72	92742.23	78848.31
24534.89	31695.52	49702.23	64858.31	15960.92	86392.35	24131.54	24336.35	93407.28	85567.93

LN455.013	LN455.013	LN455.013	LN455.013	LN455.013	LN455.013	LN455.013	LN455.013	LN455.013	LN455.014
78244	49991.47	72526.72	61703.43	46842.58	58535.19	76815.51	64439.13	61026.65	52824.08
132407.1	52008.41	94085.6	86242.19	72314.2	50228.66	111468.1	82081.93	61039.41	60312.33
84307.74	48341.97	75156.42	58093.06	48727.03	71377.2	74323.96	65173.08	63481.03	53987.65
78441.55	56526.53	74009.11	58749.57	51063.12	63865.92	77943.39	65758.97	70159.66	55619.78
80094.63	38615.6	69719.55	48845.19	53169.7	58148.46	114674.7	73946.58	72707.9	53198.75

LN455.014	LN455.013	LN455.013	LN455.013	LN455.013	LN455.013	LN455.013	LN455.014	LN455.013	LN455.013
60573.23	51253.11	37646.96	45857.44	70810.46	113435.5	79825.11	73104.06	44166.9	75337.67
74625.05	41644.75	41892.79	57526.76	62834.57	91956.95	92459.16	76574.47	100999.8	96111.65
61764.99	51577.84	41094.95	49963.49	75401.26	109174.7	77342.86	65662.18	55849.29	78356.27
57839.13	50343.96	37679.51	54767.85	78852.76	116800.4	79853.12	67151.47	56897.88	81956.27
67389.85	57666.72	26570.09	38468.67	65002.45	102597.7	70378.6	70017.84	50697.15	73836.27

LN455.014	LN455.013	LN455.013	LN455.014	LN455.013	LN455.013	LN455.014	LN455.013	LN455.013	LN455.013
34983.21	111848.6	74746.62	50336.74	61744.55	53746.88	25996.89	81846.26	77452.54	52254.82
47233.91	111528	92967.73	44205.13	58559.32	53338	31238.85	84693.62	78098.44	44722.25
39610.14	107726.2	81972.17	47880.93	68761.18	49463.1	24973.24	91617.8	77147.12	52528.13
38761.17	99849.49	86522.61	42723.33	74813.54	45129.15	25736.99	89405.15	85986.54	52435.13
42199.48	106449.4	91121.22	33953.42	77238.15	53262.18	20674.67	65115.87	114443.6	46316.56

LN455.013	LN455.013	LN455.013	LN455.013	LN455.013	LN455.013	LN455.013	LN455.014	LN455.013	LN455.014
45641.26	64236.77	99168.45	52109.36	64977.62	70815.63	47736.33	43679.61	38568.46	53694.38
60425.65	53607.93	76080.7	63078.68	104460.4	58249.23	56763.74	59886.48	44848.47	45847.69
41904.57	60822.17	88640.94	46138	70449.1	75775.22	49394.33	50184.22	43683.6	56497.09
43524.23	65547.9	89371.69	45063.96	65191.23	63702.03	48349.84	47340.02	42679.88	47379.24
43103.48	54435.45	108786.6	38899.35	94384.53	83817.26	43966.77	66782.47	44196.6	49939.91

LN455.013	LN455.014	LN455.013	LN455.013	LN455.014	LN455.013	LN455.013	LN455.014	LN455.014	LN455.013
106890.5	52049.35	105996.5	18309.59	21744.5	34073.9	50555.24	49451.35	41177.8	80619.47
105250.8	61659.3	121955.8	18318.7	21150.8	31048.82	44174.55	47656.33	39136.36	108739.2
100291.6	54351.87	128938.6	18786.04	21130.44	27883.37	48392.06	42162.98	39973.45	75528.55
100905.7	56856.75	116913.9	21882.15	24112.2	34135.95	47611.17	43178.47	40773.79	73347.18
111453.8	56920.23	96379.68	13102.21	29194.37	37480.61	49612.76	47207.4	33989.32	67245.88

LN455.013	LN455.013	LN455.013	LN455.013	LN455.013	LN455.013	LN455.013	LN455.014	LN455.013	LN455.013
57838.63	102321.7	80136.35	64720.93	82082.47	75972.37	55769.18	36081.29	85002.63	76298.71
57968.88	144061.9	93692.03	82229.06	107597.8	72781.45	41919.21	30790.28	78673.76	66974.68
60035	111927.3	79257.7	64934.26	81557.88	78283.42	59565.57	32268.16	76537.49	79456.3
61680.76	125105	74302.46	70710.78	83116.25	80047.87	56773.11	34285.71	72498.82	82257.54
62888.73	93220.58	77477.82	71553.3	79163.93	68211.45	58818.92	43133.86	45503.62	85080.94

LN455.014	LN455.013	LN455.013	LN455.014	LN455.014	LN455.013	LN455.014	LN455.014	LN455.013	LN455.014
23212.82	49210.75	80907.61	34490.39	59233.43	75368.48	53256.99	21957.95	71926.79	29024.18
26567.06	53801.85	71802.48	34492.03	62577.24	82481.37	53014.23	18106.58	99773.99	33127.37
23390.66	58381.54	70139.31	33723.1	59500.77	81076.33	56458.37	21345.27	80092.37	28303.01
19388.98	58029.09	70675.11	35907.19	61466.81	71993.21	48982.47	23715.96	76328.51	31799
25953.43	40200.8	78920.34	32231.46	63032.67	119800.1	46795.28	22842.39	77665.75	29621.48

LN455.013	LN455.014	LN455.013	LN455.013	LN455.013	LN455.013	LN455.014	LN455.013	LN455.014	LN455.014
47796.7	28265.68	122268.6	42378.07	56065.06	50369.28	29198.83	47521.27	59270.96	47841.65
45444.12	33470.93	130638.2	48930.08	50384.94	47418.05	21365.75	48410.45	55488.04	38404.16
41794.54	27807.6	106189.3	38087.34	53316.32	55381.2	27664.05	48253.93	60663.76	47427.18
44307.1	23777.84	113433.9	34589.3	45649.1	49723.23	28029.71	48914.6	63319.23	47874.14
50611.21	31640.26	133516.5	50319.77	58247.97	42564.07	28619.85	48266.65	77698.79	49702.11

LN455.014	LN455.013	LN455.013	LN455.013	LN455.250	LN455.786	LN456.788	LN456.860	LN456.861	LN456.861
22151.98	45633.68	99819.43	128349.2	42013.2	31949.94	96020.25	46572.74	56804.41	17606.84
20111.78	47294.28	113796.7	126078	39130.92	30361.74	78712.24	49367.03	63208.68	15744.63
22362.04	43117.95	101862.5	133451.8	42422.26	32518.35	107037.3	53269.13	67087.99	13409.98
18867.06	48097.1	105495.7	134893.8	42223.33	39996.03	102261.7	47950.55	59626.86	17547.91
18857.88	46065.25	129057.9	141266.7	36399.04	32853.3	145126.8	51699.91	62529.96	15492.22

LN456.974	LN456.974	LN457.791	LN458.789	LN458.989	LN458.989	LN458.989	LN458.988	LN458.988	LN458.988
39142.9	51479.18	13369.17	29316.56	21234.75	37040.75	30829.73	36168.61	49173.49	60550.86
36374.39	49991.17	9552.23	33221.35	19672.38	50103.34	35515.19	36303.97	48649.4	69500.19
36958.68	47560.2	9873.134	34389.83	11937.33	39891.71	33295.26	47439.85	50660.73	66011.5
36104.44	46293.96	10995.92	36792.31	13879.05	36337.55	30003.08	33512.26	49776.84	65212.94
36416.86	45561.97	14861.19	39716.94	12079.45	34400.07	33261.76	36764.64	39896.53	56094.98

LN458.988	LN458.988	LN458.989	LN458.988	LN458.989	LN458.989	LN458.989	LN458.988	LN458.988	LN458.989
53526.63	39547.61	18219.92	45437.03	39423.77	35689.78	19022.34	58133.29	55571.53	38375.75
49554.13	35642.51	22752.6	40817.14	46553.49	30792.86	23803.24	83594.25	59335.78	43147.47
44956.43	38616.51	13869.88	49897.27	43642.34	32790.14	14514.32	82721.25	48982.65	48254.54
52173.17	39380.01	18049.82	46336.19	36806.7	31897.82	19188.92	56224.57	52438.78	39414.26
46842.6	44138.71	14204.69	32805.75	41915.42	30489.88	18602.68	42833.07	60011.59	44204.8

LN458.988	LN458.989	LN458.989	LN458.988	LN458.988	LN458.988	LN458.988	LN458.988	LN458.988	LN458.988
52176.41	27457.84	20676.76	47828.46	39992.13	46467.43	58943.84	88812.98	47764.76	56776.37
49103.88	50065.29	19173.89	56856.27	65021.68	48972.86	66156.29	72260.72	67441.39	49114.86
75650.59	36067.06	32641.7	43532.11	42897.32	35789.67	56695.31	85393.07	53447.58	53786.87
49863.63	32068.77	19300.29	45281.67	39941.13	51969.7	58435.73	95438.03	45656.58	51551.87
52915.26	20302.75	22154.22	43876.66	52713.9	35936.73	50150.92	95895.49	58887.32	68501.8

LN458.989	LN458.988	LN458.988	LN458.988	LN458.988	LN458.988	LN458.989	LN458.989	LN458.989	LN458.989
31072.33	49104.11	61594.11	31938.57	49685.76	62009.91	38267.6	27167.48	18042.23	30262.66
45646.12	67122.69	55493.82	22904.7	77394.46	56592.74	35630.93	27981.38	17740.4	26580.77
40040.6	59275.76	79713.98	32636.84	34823.56	76538.49	41863.77	27874.38	18660.67	30784.56
30084.19	47563.04	69624.36	30671.49	49497.73	59150.39	35343.39	26595.26	19578.25	31100.39
30551.16	83105.58	59119.52	26361.51	57508.41	55625.43	41373.51	30501.07	18946.81	29681.81

LN458.988	LN458.989	LN458.988	LN458.988	LN458.988	LN458.988	LN458.989	LN458.989	LN458.989	LN458.989
50600.54	18977.83	60552.76	85458.67	34791.04	56955.15	28818.9	42312.66	35069.58	50222.31
42934.22	26181.27	50481.41	76767.74	42475.46	59790.98	26180.49	38687.98	42314.13	62070.31
53200.56	19618.76	47016.81	101464.6	29631.43	45458.25	25449.52	40773.29	30085.62	43126.02
47480.98	21125.7	58027.72	90146.07	34227.13	53582.72	30606.18	46462.19	30132.39	52884.86
33407.11	15730.85	60500.32	68349.7	34746.77	61645.31	26277.03	49083.29	28096.47	45150.21

LN458.988	LN458.988	LN458.988	LN458.989	LN458.989	LN458.988	LN458.988	LN458.989	LN458.989	LN458.989
79131.03	32374.02	62303.04	33986.96	29783.89	78814.85	55974.67	32024.6	37796.48	33579.59
126700.5	38480.64	45509.78	35955.04	30758.3	65508.16	77166.07	37032.3	38401.86	26106.53
87623.23	32967.61	94877.74	38412.45	31570.63	62083.55	50297.71	40905.45	38770.17	31829.77
69901.93	31444.72	56148.13	29907.68	26851.99	79872.69	51691.01	29582.14	38003.69	29393.13
125718.7	38437.8	60583.67	26669.99	33510.77	82211.21	54146.02	39035.44	37813.34	35645.97

LN458.989	LN458.989	LN458.988	LN458.989	LN458.989	LN458.988	LN458.989	LN458.989	LN458.989	LN458.989
28032.62	35073.71	48256.46	31076.98	27060.87	32561.72	33519.48	16380.72	40426.71	41753.82
36301.34	49906.45	57364.65	29068.94	25186.34	30135.25	37699.84	16144.38	43641.67	47196.38
27898.66	42379.97	52225.51	31110.18	33224.11	37061.99	30070.89	23923.07	25923.44	36193.18
32679.88	33947.59	53978.09	29775.31	25929.17	35973	37347.86	16054.79	41817.53	42528.87
25977.25	43891.38	34790.83	29924.23	30180.24	32491.69	37674.24	19209.23	30082.68	45315.36

LN458.988	LN458.989	LN458.988	LN458.989	LN458.989	LN458.989	LN458.989	LN458.988	LN458.988	LN458.989
43394.8	35045.74	44268.79	26408.47	53619.38	45688.63	18555.6	49645.87	42478.12	19066.4
44838.79	31921.98	53085.89	26742.64	36006.11	44961.01	16317.48	52606.88	50378.02	18841.25
40599.47	34853.1	41589.77	27338.51	39409.2	40279.75	25680.83	54442.14	56611.44	21059.53
41754.66	27280.1	41418.15	30103.78	53646.4	35559.19	16353.92	41169.93	45754.38	21061.06
45646.86	26131.86	38646.28	35232.02	39802.98	40451.85	15909.5	38277.56	49781.5	20999.94

LN458.988	LN458.988	LN458.989	LN458.988	LN458.989	LN458.989	LN458.989	LN458.988	LN458.989	LN458.988
59159.76	54276.91	33279.33	44888.28	40317.53	17757.21	22394.38	65266.3	35127.91	62770.68
66821.15	46505.77	39446.57	49546.58	44604.2	32906.3	21283.61	75091.62	37990.31	61828.12
57115.57	38178.52	36392.18	58581.67	48593.15	22674.05	21290.89	75437.68	42024.35	68167.48
58332.86	59478.87	37566.32	43502.08	47249.35	18384.86	21565.04	65063.51	30653.41	70908.39
85549.23	48866.4	34269.05	51528.81	40256.83	18233.89	17447.68	71562.79	33129.18	64210.68

LN459.026	LN459.121	LN459.195	LN459.223	LN459.223	LN460.198	LN460.786	LN460.985	LN460.985	LN462.803
35728.47	13828.09	101346.6	16255.53	17472.75	18557.19	12063.5	38030.67	42034.86	46451.49
37385.67	17482.87	104319.7	16835.21	17673.99	17389.46	11077.98	36073.6	41967.06	40095.51
37253.59	17142.43	112503.7	18145.52	18064.27	19614.94	12064.76	36877.57	42580.95	44108.92
36398.25	14618.95	92379.95	15917.27	18353.69	16766.24	12823.69	39792.83	40326.23	41199.02
39482.02	13379.49	84839.45	15003.14	17113.54	17520.83	13259.7	37742.76	40416.62	54582.05

LN462.804	LN462.805	LN462.982	LN462.982	LN462.982	LN462.982	LN462.982	LN462.982	LN462.982	LN462.982
22075.53	14211.38	45118.07	41034.6	54872.01	49558.66	65467.78	36969.27	54579.82	44507.76
23377.26	11080.89	40620.1	39551.49	55727.58	47191.69	71080.93	36587.98	52837.13	40715.25
23499.09	12062.24	44719.08	41245.81	53856.55	47484.34	58090.99	33808.51	52895.59	41353.89
22845.6	14604.19	50106.46	40105.59	49467.37	50990.46	63539.37	38557.79	56369.44	41443.94
24910.63	14814.73	46895.86	37917.76	52287.85	44546.69	63294.04	35940.62	54222.16	42200.65

LN462.983	LN462.983	LN462.983	LN462.983	LN462.983	LN462.983	LN462.983	LN462.983	LN463.039	LN463.039
28290.9	34877.76	39159.32	33368.68	27501.55	36484.53	50031.03	36269.24	51481.34	55740.03
30273.66	31627.86	33956.92	29384.12	29574.33	35443.83	48988.6	37295.37	53421.65	55382.59
29867.6	30733.06	38053.54	26158.04	27447.58	34734.72	50627.03	36443.03	47970.21	52727.75
30211.79	30633.65	34469.33	27647.01	26811.29	34996.06	49784.51	35395.46	53860.85	55796.81
26883.25	30948.36	38268.77	29658.29	28415.66	33526.38	45554.6	36884.97	56196.54	60191.42

LN463.038	LN463.180	LN463.180	LN463.255	LN464.413	LN464.801	LN464.820	LN464.997	LN464.997	LN464.997
57162.28	52001.84	36575.03	27286.15	72077.71	32680.02	35190.6	83118.62	28381.77	37165.26
53720.54	53754.14	29846.2	29558.93	80377.04	36169.84	18316.62	82968.71	25289.47	42855.47
54842.74	52339.05	30991	21114.66	72085.85	37613.77	21250.74	80510.13	26797.01	35526.42
60759.79	52796.79	27622.88	24969.13	64175.85	26936.82	42417.05	75926.77	27263.36	37925.55
58272.58	52006.6	31681.75	25983.95	58502.66	54396.53	33697.11	77271.5	28713.4	37928.8

LN464.997	LN464.997	LN464.997	LN464.998	LN464.998	LN464.998	LN464.998	LN464.998	LN464.998	LN464.998
42233.8	42974.38	41148.69	47405.66	21201.75	85842.71	30338.32	65489.58	32812.67	47778.56
44066.73	41852.3	42520.36	45591.06	18876.1	57152	45349.01	56550.85	41463.6	39879.62
45225.26	40330.11	36985.22	35850.11	17359.8	69325.4	49604.17	65182.51	37125.02	39468.65
41726.54	43067.25	40141.5	47638.98	19190.72	63316.25	37426.3	55429.82	37292.54	33463.97
44832.95	39261.36	42479.04	42510.13	17616.49	57135.42	44008.44	54245.91	38195.61	40377.76

LN464.999	LN464.999	LN464.998	LN464.998	LN464.998	LN464.999	LN464.998	LN464.998	LN464.998	LN464.998
14494.43	18818.1	43848.36	40640.59	41113.72	16740.28	77969.35	40650	36367.07	30189.78
19853.73	23552.53	48525.69	32774.51	34015.99	14290.69	61181.86	41153.89	53769.64	28272.69
15581.25	23955.73	51954.21	46046.6	35916.97	15841.34	61165.65	45301.36	45122.28	27132.14
15540.46	20496.94	53072.36	38982.43	31937.66	15387.62	63290.61	46412.88	48924.46	27569.63
16096.25	19964.31	47366.48	37164.14	34757.5	13352.08	63899.82	44584.12	51276.4	29660.74

LN464.998	LN464.998	LN464.998	LN464.998	LN464.998	LN464.998	LN464.998	LN464.998	LN464.998	LN464.998
51004.75	24040.31	27379.24	48287.27	30372.1	25784.4	44158.99	41989.62	61772.97	36111.94
52269.81	27830.32	28806.65	54966.57	40658.3	33580.05	44249.11	47112.76	55946.91	49205.6
72565.44	24840.97	24286.74	56374.36	46708.87	29507.89	49217.22	42275.47	51131.46	89030.7
51313.06	30255.85	29814.57	54360.53	41415.06	34867.12	45380.5	46704.26	53378.4	45466.03
60786.48	31375.68	30468.26	51828.29	35968.95	32683.33	42034.64	43310.81	52009.9	44665.86

LN464.998	LN464.998	LN464.998	LN464.998	LN464.998	LN464.998	LN464.998	LN464.998	LN464.998	LN464.998
55304.91	36955.81	31076.17	58508.1	26363.75	19780.27	27349.64	19436.23	40536.04	41612.64
42108.69	43583.49	32919.31	54876.21	41373.72	17806.76	28326.01	18830.66	29549.31	30043.35
56701.76	51179.49	33575.49	51218.73	40687.83	18266.74	43710.82	20799.64	29423.36	30125.86
40538.84	44957.77	30667.55	60900.75	39349.14	18678.43	28646.65	17350.85	26737.61	27850.45
42661.75	46038.77	36834.37	63566.17	36735.08	18547.61	26897.94	17847.8	25177.33	29986.23

LN464.998	LN464.998	LN464.998	LN464.998	LN464.998	LN464.998	LN464.998	LN464.998	LN464.998	LN464.998
32942.08	28726.2	84284.68	61857.31	30250.56	16374.61	30209.11	52333.84	41949.3	56655.71
31515.58	24784.43	53191.07	68400.07	26598.1	12224.72	29538.78	43949.31	29853.31	53449.87
42168.56	28134.15	74430.51	64645.37	32856.37	12982.03	32410.51	42520.06	29635.4	56620.55
29285.47	23089.02	54282.87	72566.1	28924.35	14296.25	27826.19	42308.94	30026.82	52289.49
31948.03	25575.08	62386.29	65750.53	25801.46	14432.2	31713.24	45754.71	29148.9	54017.03

LN464.998	LN464.998	LN464.998	LN464.998	LN464.998	LN464.998	LN464.998	LN464.998	LN464.998	LN464.998
21856.42	19540.37	65870.41	53961.71	35173.61	33059.89	36648.44	27706.6	39446.73	48334.4
29469.09	32017.69	59382.22	52007.4	30986.09	30489.82	23228.99	25526.22	75234.71	37626.63
23883.36	19200.13	51544.23	50533.08	27519.81	25874.16	12389.77	20910.09	70988.87	42399.52
29087.13	31029.38	59683.31	52812.52	28717.71	27960.11	21103.26	24154.2	67711.72	36864.78
26863.53	27441.23	57767.83	57997.77	31702.04	28477.05	18808.78	23804.33	72348.85	38134.08

LN464.998	LN464.998	LN464.998	LN464.998	LN464.998	LN464.998	LN464.998	LN464.998	LN464.998	LN464.998
25560.3	31858.37	46082.29	30568.03	33618.3	50998.44	22555.91	19648.88	72676.81	61828.64
23555.52	28064.86	31300.76	27243.22	40751.49	30884.2	21345.95	20416.2	56337.14	35689.13
32291.03	27831.43	55768.02	34245.7	46154.19	30595.72	22192.33	22165.66	52120.3	25709.77
24753.87	31641.37	31107.52	30144.03	42314.73	35034.19	22898.78	21961.15	55762.07	36290.84
28977.71	29364.76	36498.03	25772.64	40645.73	34437.98	21880.28	20117.69	61445.95	32190.53

LN464.999	LN464.998	LN464.998	LN464.998	LN464.998	LN464.999	LN464.998	LN464.998	LN464.998	LN464.998
22132.99	55383.59	44676.93	57996.39	36661.93	13984.58	50448.07	13465.34	36259.96	34789.57
24080.56	58075.93	39447.93	61690.18	33326.8	17375.83	52419.92	16264.79	35725.74	25492.5
20875.39	38569.99	37551.04	58438.7	30952.44	17742.63	62683.82	13426.08	37315.6	22791.67
20130.62	63640.64	37823.47	56715.27	36702.38	18959.61	55290.14	16221.28	38120.76	28193.13
16410.88	57945.91	32914.19	61121.63	40011.97	17652.9	57740.59	13388.9	37683.45	26164.54

LN464.998	LN464.998	LN464.998	LN465.016	LN465.035	LN465.034	LN465.055	LN465.055	LN465.232	LN465.786
62590.64	67272.88	41711.37	32182.89	66945.82	64866.5	13987.02	18276.94	94117.38	24459.65
59033.68	37892.84	42024.87	38073.92	67852.53	60914.65	19157.33	15158.09	80647.75	20906.59
48521.36	53736.29	43741.68	32338.86	68436.07	62215.14	11824.54	14810.69	59215	21247.07
60190.81	38324	47587.42	28665.83	72686.3	64880.08	14571.23	15955.79	73587.82	25428.73
58745.35	33943.77	43941.86	36057.55	69561.38	60974.09	16768.35	12430.72	75401.96	20280.6

LN465.821	LN466.234	LN466.414	LN466.816	LN466.976	LN466.976	LN466.976	LN466.976	LN466.976	LN467.012
24647.15	17292.47	34095.97	242758.7	49412.37	41121.47	43625.71	62386.49	76575.95	50761.98
27879.75	13959.26	37424.67	209814.8	54752.52	43563.83	45827.68	54956.6	70955.03	47037.74
26228.35	16930.85	32456.01	235016.5	49799.66	39537.11	47552.68	58642.8	70477.35	47092.65
23065.48	16360.7	26689.56	309696.9	54139.64	43731.5	45712.05	63477.33	69004.24	48985.03
31996.72	14518.34	28205.45	335079.9	51814.94	38441.56	46636.39	58647.2	72051.58	46482.63

LN467.012	LN467.013	LN467.013	LN467.013	LN467.013	LN467.014	LN467.013	LN467.013	LN467.013	LN467.013
45527.38	26134.01	30815.3	19840.92	18982.94	16057.22	48041.55	42889.06	43496.05	39575.81
46252.03	24782.05	35529.15	18570.57	18506.18	17204.86	51895.8	42273.63	43301.31	40310.52
43162.59	26349.54	34263.78	18644.94	20941.53	16797.69	44352.03	44471.7	45348.82	38328.83
42867.48	27094.07	34894.69	18960.88	19060.8	16471.19	48525.91	48453.33	44923.33	37030.96
56341.31	24258.25	32734.24	19417.27	20712.25	15582.05	47892.91	45198.73	51449.01	43399.22

LN467.013	LN467.013	LN467.013	LN467.013	LN467.013	LN467.013	LN467.013	LN467.013	LN467.013	LN467.013
23192.78	52275.28	51536.36	29152.15	40455.19	37409.76	43564.3	23983.26	23487.93	35910.57
27331.12	50690.7	52392.27	31704.96	42878.3	34476.97	48357.41	22218.8	25719.48	40913.44
25385.2	52079.14	47618.38	32149.36	40307.4	34372.93	46394.12	23438.23	25901.7	35639.44
23121.44	52779.15	44873.85	30855.97	41336.83	33396.67	46955.94	22524.56	26661.85	35789.54
25786.95	58620.31	49926.51	35610.26	43777.55	35725.92	50087.77	22267.17	27541.45	35883.8

LN467.013	LN467.013	LN467.013	LN467.013	LN467.013	LN467.013	LN467.013	LN467.013	LN467.014	LN467.013
23594.17	30267.96	49432.29	23837	55022.56	54088.47	38419.53	59534.56	21876.07	38237.57
22177.44	28171.19	45144.87	22436.5	53459.48	59860.42	37658.19	56953.26	19793.11	39600.06
22133.55	29874.2	46500.93	24909.29	53962.49	55784.56	43909.18	59647.88	21074.99	41137.43
22324.4	32536.25	41475.2	24939	51292.3	55934.77	39063	57440.2	19217.91	40604.26
22467.59	33305.08	57164.03	23647.2	58791.43	64466.06	45079.27	61831.4	20559.76	44389.13

LN467.013	LN467.013	LN467.013	LN467.013	LN467.013	LN467.013	LN467.013	LN467.013	LN467.013	LN467.013
33996.35	34544.67	47822.04	50135.52	25477.43	32546.1	40283.11	28010.95	59991.5	45520.98
36759.81	36633.07	48631.64	45998.21	24219.04	37997.16	38558.46	27620.78	59295.88	45238.75
36178.17	35572.12	49344.84	45938.85	23308.59	35125.43	35036.7	26991.66	61011.19	45459.04
35945.54	35124.64	49792.72	46878.07	24585.42	36636.53	39255.67	27102.59	56842.38	45217.72
37078.09	35672.89	51556.16	53937.21	26344.82	40368.07	36300.68	26837.01	65335.68	44617.87

LN467.013	LN467.013	LN467.013	LN467.013	LN467.013	LN467.013	LN467.013	LN467.013	LN467.013	LN467.013
56376.94	62356.22	39389.78	33269.2	50250.97	36171.18	48859.1	33872.45	22011.66	22785.54
53079.63	64665.38	35181.94	30711.51	47551.37	40586.99	52266.74	31006.21	25626.14	20311.72
51692.81	66738.56	34261.78	31572.29	47384.98	36912	56499.15	33146.96	23031.11	21766.86
51306.26	70568.59	34435.73	29717.42	41677.77	35170.13	48351.17	30686.12	22433.37	23572.23
53503.85	67551.23	40359.41	29942.94	54688.41	36085.82	45547.1	33442.84	24830.56	22333.93

LN467.013	LN467.013	LN467.013	LN467.013	LN467.013	LN467.013	LN467.013	LN467.013	LN467.013	LN467.013
38339.7	40327.84	33217.18	27566.16	62633.93	85359.12	14194.53	24825.9	56890.06	32834.7
37836.27	39142.85	32474.9	31621.12	68256.07	81539.7	15484.77	26718.59	58160.09	30412.78
36134.86	43052.87	32459.25	33189.43	67414.5	84518.27	15189.77	24308.28	58816.32	29597.13
34846.41	41781.97	32012.18	29312.39	60608.07	88798.21	14823.9	25772.27	59486.04	27779.99
41024.35	41648.6	35771.34	31895.22	62760.46	92507.17	13710.75	28392.17	64432.01	30311.06

LN467.013	LN467.013	LN467.013	LN467.013	LN467.014	LN467.013	LN467.013	LN467.013	LN467.013	LN467.014
62177.96	37141.6	45893.05	39852.54	21379.33	57591.93	38818.23	38260.84	46517.08	17774.29
65425.41	36930.09	41680.7	40849.21	21746.82	56685.57	39038.97	38496.73	46029.38	20003.49
63330.08	40935.77	40939.99	39520.08	19970.24	52881.86	41862.54	36940.73	50545.54	18161.38
65268.62	34142.47	42323.07	43518.84	20410.41	49095.25	37116.08	37399.5	47642.58	16581.51
70032.04	41718.8	36905.79	43045.63	20039.09	54228.04	45110.72	41740.56	42218.85	16725.1

LN467.013	LN467.013	LN467.013	LN467.013	LN467.014	LN467.013	LN467.013	LN467.013	LN467.013	LN467.013
43039.79	21864.27	34857.38	36985.35	21148.63	18710.56	27251.59	22220.99	56649.61	21841.33
39164.3	20558.11	30034.53	34833.84	20647.16	18001.89	25369.65	23218.87	56787.55	25514.21
40349.86	20588.33	30628.73	35347.53	21093.59	19861.84	26051.57	21934.75	58654.16	22012.09
41169.78	18689.61	28664.87	37724.54	21571.25	17584.59	25252.91	21775.35	59670.42	22480.36
48445.89	21697.59	31279.32	42871.06	19412.17	20483.16	27183.69	24775.82	64288.52	23996.38

LN467.014	LN467.014	LN467.013	LN467.013	LN467.013	LN467.013	LN467.013	LN467.013	LN467.013	LN467.032
20502.76	16396.49	40333.65	55590.95	48899.59	27699.37	38721.95	57717	26097.34	78577.3
21189.94	16345.38	38285.96	60691.24	46223.02	24736.54	35550.69	64563.14	25869.03	66846.07
18162.8	18564.68	38769.23	57637.13	46928.44	24675.02	36205.76	60681.44	25061.48	32748.19
19236.66	16215.36	41614.16	63008.11	49516.74	27321.8	32421.7	65609.13	24923.26	29805.05
19192.56	13728.68	40613.79	60497.61	48624.98	25685.16	39208.94	60352.9	25923.04	75442.84

LN467.032	LN467.033	LN467.033	LN467.033	LN467.033	LN467.033	LN467.033	LN467.249	LN467.249	LN467.249
62168.59	59215.3	57758.44	54557.56	57529.97	73757.69	64365.96	24417	23746.08	29810.05
60736.51	55933.42	58662.71	61799.31	56716.39	73549.09	60272.67	22489.24	23096.55	25672.68
61130.22	60872.68	55804.07	54895.29	52336.45	71745.18	58925.9	23010.87	21868.97	27058
26947.19	54819.99	57591.02	52067.42	55935.49	67980.07	56470.72	26001.94	20842.18	26794.06
68439.55	56439.45	62545.49	57439.34	50864.1	69083.63	49878.52	22376.96	20859.77	24275.9

LN467.249	LN467.800	LN467.819	LN468.816	LN468.992	LN468.992	LN468.992	LN468.992	LN468.992	LN468.992
29885.79	27964.8	28002.91	79587.87	23009.75	37735.74	30965.14	34149.5	23098.13	28937.36
26697.36	24615.66	24274.48	84932.43	23445.1	35883.86	29637.88	34423.75	22337.6	28716.81
24972.82	26526.33	26871.12	65548.08	23947.07	38861.04	29826.37	34534.25	23541.09	32083.57
27431.63	32785.13	30616.21	57223.65	23574.91	40083.04	35185.68	36778.17	20719.2	30526.01
28767.17	28891.61	36187.02	75096.32	23170.78	38123.92	32391.56	34011.65	20090.44	28949.55

LN468.992	LN468.992	LN468.991	LN468.992	LN468.992	LN468.992	LN468.992	LN468.992	LN468.991	LN468.992
27326.19	36006.53	57550.83	44059.31	27408.42	34423.68	48837.2	57351.81	81477.54	25491.74
28062.45	33738.47	61424.81	46541.15	32449.79	34719	58042.58	60767.98	81939.2	25890.17
27665.75	34527.53	62165.17	44376.37	29374.77	34077.72	56600.24	56032.72	71087.29	26632.91
25182.03	34346.41	61927.48	46263.33	27896.1	34180.48	50422.17	61628.11	83493.94	26309.44
26308.19	31294.77	64558.84	41545.01	26619.87	33206.6	53298.04	59291.48	79978.19	28219.2

LN468.992	LN468.992	LN468.992	LN468.992	LN468.992	LN468.991	LN468.992	LN468.991	LN468.992	LN468.992
33916.62	46834.32	41889.41	19674.56	53684.14	61372.73	21652.57	65775.57	33274.56	42590.71
35089.79	49730.99	43111.16	15810.27	51309.9	58640.84	23254.47	61598.55	33924.2	41725.46
32108.32	45851.18	43239.57	15250.13	57073.63	62913.13	21893.53	68437.97	31983.33	39491.32
33513.15	46452.63	43691.73	17597.94	51444.62	57730.75	21560.8	64384.21	38456.72	44317.84
31873.76	47288.99	41529.56	18010.58	57045.21	57177.13	20289.2	68386.39	34219.28	39848.69

LN468.992	LN468.992	LN468.992	LN468.992	LN468.992	LN468.992	LN468.992	LN468.992	LN468.992	LN468.992
32336.38	18915.46	80379.95	20538.08	26097.96	52870.53	37957.23	31035.44	33971.14	29002.95
28870.37	19778.05	77015.56	21406	29599.16	57052.23	33492.54	28559.24	34367.94	25698.87
31815.66	17682.48	80219.82	21133.14	28577.08	55549.47	39330.76	35208.23	35632.76	27569
33217.79	18257.16	76392.43	18344.57	28324.75	63385.96	36359.99	34816.73	33337.19	26650.18
32268.16	17234.47	75314.02	19681.75	26205.21	53850.71	38971.58	31792.36	32195.93	26734.16

LN468.992	LN468.992	LN468.991	LN468.992	LN468.992	LN468.992	LN468.992	LN468.992	LN468.992	LN468.992
34650.41	32172.19	54371.3	34330.35	58066.23	58157.6	42870.04	34563.49	25999.44	65361.44
33347.8	32443.48	57755.08	39106.53	52416.43	61046.5	42533.19	34274.78	26555.49	66936.92
34782.41	29889.35	59921.22	35967.77	54842.72	57781.99	37880.52	35168.32	24682.11	65723.35
33117.69	31982.8	58204.41	36180.57	56335.77	58833.91	44199.03	35528.6	25330.81	64504.29
35566.62	30668.37	57384.92	36027.2	53694.57	60471.86	40624.43	32841.39	24190.49	61791.19

LN468.992	LN468.992	LN468.992	LN468.992	LN468.992	LN468.992	LN468.992	LN468.992	LN468.992	LN468.992
23831.04	43967.76	40929.75	26295.37	65651.94	44517.56	42503.6	38794.7	36225.23	28793.2
29731.75	46252.02	40780.25	25086.85	65814.81	45639.97	38845.22	36438.8	34232.68	30870.63
31227.38	46235.69	42004.32	26343.67	63317.56	45300.2	40754.43	38110.67	37763.63	35247.6
27022.9	48481.29	42564.92	27413.36	65020.85	43058.94	40775.7	37628.64	35071.42	33822.89
26210.52	45772	40182.78	25885	65475.63	42354.44	36864.71	38694.83	33813.54	34130.2

LN468.992	LN468.992	LN468.992	LN468.992	LN468.992	LN468.992	LN468.992	LN468.992	LN468.992	LN468.991
33293.18	25977.25	93328.86	48079.13	36215.33	33186.19	19523.82	17321.92	48500.06	68561.52
32709.49	27197.9	92502.83	48405.42	40863.22	31999.26	20069.28	17685.64	45798.48	67413.89
34888.46	28562.81	97081.98	45682.87	37654.54	32527.5	18978.37	16532.54	48770.74	70664.82
29946.12	26934.73	92984.56	47896.59	38594.54	33902.09	19138.37	17031.79	47756.06	70564.11
33335.04	27855.44	90989.9	46853.75	36435.95	28834.47	19632.82	16265.69	49160.09	65563.71

LN468.991	LN468.991	LN468.992	LN468.992	LN468.992	LN468.992	LN468.992	LN468.992	LN468.992	LN468.992
66967.05	37420.11	17694.05	48007.58	34744.97	30773.34	31062.85	19748.99	27306.44	40405.05
64419.43	40981.21	15668.43	50923.13	34459.76	31938.4	27144.23	20047.87	26526.4	40131.52
70238.77	39656.52	18544.94	48141.05	33022.74	28549.38	26278.72	19440.35	25271.13	37112.22
66944.72	38730.43	17706.74	49673.46	32392.7	31217.85	31435.7	18960.8	25951.47	41641.49
70041.22	40204.73	18080.69	49495.13	34827.71	31925.9	30009.11	19389.27	26447.66	37420.95

LN468.992	LN468.992	LN468.992	LN468.992	LN468.992	LN468.992	LN468.992	LN468.992	LN468.993	LN468.993
36654.37	47212.7	56307.38	65641.88	40957.94	48311.3	53292.2	19326.55	38280.95	27776.69
36908.84	47721.9	56077.08	67878.58	37464.84	49033.11	61529.22	19639.41	37097.56	29489.83
36685.83	49294.85	53879.24	72729.43	40437.02	49484.11	58568.35	18250.07	43328.81	27757.7
38261.2	45524.7	54530.73	60507.1	41388.3	52877.13	56369.03	19030.93	40654.68	29842.01
36269.23	46167.59	54423.55	61885.75	38721.6	48050.37	54124.17	19733.59	41638.04	25612.22

LN468.992	LN468.993	LN468.993	LN469.010	LN469.010	LN469.028	LN469.150	LN469.781	LN470.153	LN470.764
23330	37676.13	25531.3	36383.7	36231.18	44904.33	90963.42	48212.73	16198.99	28583.2
24747.72	35322	23992.04	35890.92	40584.9	49930.73	87477.59	75737.19	16808.1	28611.39
24647.11	38542.42	25348.2	38868.68	40049.53	54645.2	104631.3	53743.56	15674.19	28207
22721.39	38061.9	22459.02	38905.09	33756.58	44518.24	77787.96	62694	14199.82	31928.85
25075.66	35809.8	25058.23	42896.7	36125.19	47764.13	82726.08	52595.96	13374.02	36095.77

LN470.812	LN470.837	LN471.150	LN471.181	LN471.294	LN471.311	LN471.763	LN471.836	LN472.759	LN472.833
33267.18	33808.82	29905.01	29502.13	10828.03	8741.41	21428.11	23451.97	102746.3	265176.3
33231.18	31853.31	27288.4	30517.79	10155.16	8386.628	19613.98	18837.29	88898.56	245989.2
34339.88	41900.91	28942.1	28009.73	10378.85	8851.148	21504.36	20821.75	101941.1	337374.6
37443.35	32923.82	25345.81	27068.23	12352.76	9516.608	22148.09	23244.08	122936.2	343443
42642.93	39034.1	23478.71	27758.2	11946.34	9560.189	21313.28	23397.98	139788.6	377862.9

LN472.916	LN472.916	LN472.916	LN472.916	LN472.916	LN472.916	LN472.916	LN472.916	LN472.916	LN472.916
19545.83	21495.31	26281.23	15311.57	22583.1	21106.18	21660.39	22377.67	32634.87	28185.45
20718.8	23858.82	27518.27	17559.3	25444.97	20746.86	24205.06	24099.1	32974.35	29161.49
19082.08	20162.14	26546.12	15191.69	26097.32	18608.99	26676.77	22677.92	32560.2	27814.71
17481.91	21479.79	24825.17	13598.71	26192.54	18977.16	22170.47	22571.33	33010.68	27260.15
13025.35	19937.48	21841.78	13623.83	27088.21	19035.19	23438.11	19955.58	27478.48	26682.07

LN472.916	LN472.916	LN473.003	LN473.004	LN473.004	LN473.004	LN473.004	LN473.004	LN473.004	LN473.004
27984.09	17310.73	82583.32	103142.8	46578.32	39285.82	106491.1	20487.9	134842.4	114225.3
30072.69	19556.05	85490.6	86727.24	60278.79	63717.98	85008	17238.17	105141.7	111961.5
26377.45	15637.74	122507.9	87813.24	58231.88	67995.05	93570.81	37235.19	68150.93	104750.4
26582.49	17861.01	78846.72	86494.53	44470.86	43047.34	79381.69	18410.26	93161.5	182514.9
26099.39	17327.69	72877.74	78977.03	84339.82	34617.39	118808.4	16050.06	84237.81	129626.9

LN473.004	LN473.004	LN473.004	LN473.004	LN473.004	LN473.004	LN473.004	LN473.004	LN473.004	LN473.004
70879.12	84101.93	40756.12	65044.25	102237.5	30212.39	40535.97	85653.98	69541.4	78032.96
91988.45	111182.4	60080.64	73128.55	68415.07	44063.63	52074.77	74101.25	53044.96	70427.13
62890.54	97274.78	38170.04	41318.06	79029.98	35229.64	41680.8	84336.71	51760.87	70397.41
92483.91	68237.98	58125.77	81371.16	89554.23	44430.61	51757.08	79582.82	56867.6	88330.41
64397.71	112327.2	47259.06	48486.46	109543.7	30350.63	53799	52304.83	52212.27	76714.09

LN473.003	LN473.004	LN473.004	LN473.004	LN473.004	LN473.004	LN473.004	LN473.004	LN473.004	LN473.004
133756.9	49382.22	43693.09	116714.9	138064.9	25571.48	85682.82	89109.03	34125.77	58384.08
137734.8	58675.02	56993.42	127199.9	120566	31891.29	118970.1	61290.15	47272.19	68622.8
129193.8	54911.91	34931.61	83952.05	129855.1	29926.36	78851.13	100175.1	31505.99	92936.89
174479.6	47531.52	76930.8	185574.3	80623.82	36793.94	118199.4	67238.55	34068.27	77045.03
148195.8	48798.1	36793.57	76165.01	126522	32305.21	106281.6	111747	33948.03	68450.51

LN473.003	LN473.004	LN473.004	LN473.004	LN473.004	LN473.004	LN473.004	LN473.004	LN473.004	LN473.004
126423.2	69592.5	35281.76	104061.9	76435.3	81141.04	70055.29	49900.58	97718.57	100491.3
122732.7	108269.2	35543.01	144126.5	126057.5	67111.59	147904.4	59287.08	101119.1	78153.26
153291.3	80258.14	37331.82	91151.9	87494.42	86865.25	84659.16	38214.77	88536.74	76313.28
141405.9	96747.83	35333.87	113456.2	107688.6	86492.79	82005.11	65219.48	83540.08	109275.2
116572.5	80571.93	43771.48	123863.9	80045.51	70639.17	101562.2	45690.93	107735	70199.57

LN473.004	LN473.004	LN473.004	LN473.003	LN473.004	LN473.004	LN473.004	LN473.004	LN473.004	LN473.004
28162.68	80473.52	62214.49	144404.2	48013.51	32493.9	92736.48	47855.54	69804.35	45967.75
30432.18	84995.75	67641.43	116312.5	57919.84	45548.55	49089.67	40240.48	99569.04	57646.85
33337.39	106112.4	55729.41	188511.7	71109.34	24166.44	58283.43	61448.62	110019.5	62184.74
33054.49	91458.61	47007.28	123121.5	65588.95	22434.42	44215.31	40395.63	92905.47	76175.44
22869.74	88629.17	49759.98	121098.6	73590.96	21834.46	44034.52	62699.6	101307.6	42961.05

LN473.004	LN473.004	LN473.004	LN473.004	LN473.004	LN473.004	LN473.004	LN473.004	LN473.004	LN473.004
143067.8	57930.88	112125.1	103882.6	59638.18	27079.81	77700.64	64621.39	74851.1	64057.84
100680.2	57954.66	132435	107550.5	55119.21	36517.29	57128.42	54074.7	72077.3	64242.76
123445.2	81404.74	84134.19	84614.12	60749.61	31057.21	81585.44	66721.98	59141.9	90155.78
114782.5	66104.8	58267.68	87235.44	46197.4	36229.59	62757.24	90235.28	62939.18	68566.64
158473.3	74976.51	72235.43	55852.58	56984.53	31839.15	67361.95	61258.72	74361.64	72903.37

LN473.004	LN473.004	LN473.004	LN473.004	LN473.004	LN473.004	LN473.004	LN473.004	LN473.004	LN473.004
61659.47	29563.79	70843.83	68797.57	49812.08	69215.32	105827.3	71829.93	92297.91	72969.4
36989.32	30206.91	70467.96	61834.91	59082.17	83366.27	110276.1	51073.98	93409.3	51830.29
38119.79	31269.95	63600.59	62127.77	43295.26	48670.5	105986	53711.52	96088.38	48818.95
63312.69	27862.74	75454.97	55053.68	60810.73	61783.4	91244.96	50623.25	103137.5	49361.5
52399.02	29237.57	64990.04	56223.19	55498.65	76407.74	106775.3	68839.77	74757.3	48120.67

LN473.004	LN473.004	LN473.004	LN473.004	LN473.004	LN473.004	LN473.004	LN473.004	LN473.004	LN473.003
44261.75	80288.08	75156.86	30559.3	67378.01	87373.09	64051.6	52808.17	47223.24	138267.8
64787.3	77751.3	66254.55	30804.41	70208.13	94919.53	61711.81	86228.06	53797.58	114349.8
51052.26	105807.6	84661.55	25941.09	84633.98	113878.3	55216.57	46912.11	57141.26	136582.8
46218.3	78563.05	65026.62	43894.61	69745.53	70113.81	84074.49	58035.13	50159.69	94582.43
47200.64	88654.56	68387.53	31897.38	67906.96	69634.59	56382.06	80451.37	57394.65	129514

LN473.004	LN473.004	LN473.004	LN473.003	LN473.004	LN473.004	LN473.004	LN473.004	LN473.270	LN473.282
37633.65	209071.3	88153.07	123380.6	75819.99	52562	61624.24	33586.25	12189.25	103591.2
23318.94	102481.8	114702	128630.5	67873.65	53710.65	71682.99	21940.37	13768.43	86091.69
21522.64	118871.6	95478.92	120234.1	70027.11	57145.81	85584.33	29644.3	13005.6	81836.34
22752.56	109397.3	85515.13	134604.9	69416.97	61809.05	80336.99	28649.91	12052.98	77479.34
19100.75	103307	83453.95	118001.4	69332.33	54585.12	59631.87	19562.6	11055.53	87778.52

LN473.282	LN473.762	LN473.835	LN474.192	LN474.286	LN474.286	LN474.759	LN474.833	LN474.982	LN474.982
52316	27969.3	27136.15	52854.47	28702.7	17537.39	64505.07	121910.6	40255.38	45164.97
42159.83	26849.49	32147.98	56348.75	29803.12	19268.27	63115.15	126809.9	40077.38	46573.07
61204.98	25636.82	24472.54	50216.93	25601.84	21709.73	76468.19	108191.4	39603.19	44025.5
59759.85	31732.73	32989.72	53434.96	24496.92	14727.31	79118.77	151821.8	38586.54	42117.62
49219.03	35234.23	37724.18	48395.09	25350.85	15988.54	91498.07	87913.08	40748.2	41730.94

LN474.982	LN474.982	LN474.983	LN474.983	LN474.983	LN474.983	LN474.983	LN474.983	LN475.039	LN475.039
32003.25	50663.49	47764.62	44560.24	57733.11	34194.1	35056.18	38724.86	32674.49	55308.45
30426.29	48802.03	45353.2	49215.45	56401.02	36363.53	31107.28	35875.2	37381	48487.39
30119.18	49669.2	43707.76	48228.74	55717.69	31399.45	31805.5	37529.02	33030.24	46647.74
31756.27	44247.84	41902.09	45665.48	54466.88	31628.44	32003.16	36821.77	35828.52	46774.65
31472.72	52170.32	41331.07	43657.36	51466.85	31156.24	28279.07	35528.08	32991.34	41503.27

LN475.195	LN475.217	LN475.217	LN475.217	LN475.217	LN475.761	LN475.834	LN476.759	LN476.775	LN476.830
14642.62	23280.71	23759.89	27760.47	22167.64	15201.25	14073.54	20533.97	143762.7	127766.7
14385.38	21829.03	27785.06	24621.59	23070.08	13503.72	17818.91	21161.6	133721.1	57284.54
15416.79	21230.14	23240.77	26687.35	21839.65	15029.58	11874.99	19628.61	140605.1	125636.3
13976.21	23956.87	23814.38	26412.17	21595.44	12346.94	12829.47	18503.05	168195.8	164764.3
14869.49	23341.15	23852.2	27038.34	21844.77	14857.85	13990.85	21615.86	194977.4	81351.76

LN477.035	LN477.035	LN477.035	LN477.035	LN477.035	LN477.197	LN477.197	LN477.778	LN478.776	LN478.865
39251.61	45844	45018.1	29988.96	31072.82	44898.95	62307.03	18250.45	46347.53	29436.11
36501.63	39958.73	51268.65	32828.71	32774.2	44314.58	65021.34	13822.75	41099.69	28374.51
37116.97	46162.71	47370.28	28079.69	34736.44	47342.8	59894.52	14215.8	45679.3	27245.53
41222.28	46925.62	51981.24	35584.64	36357.69	52629.29	61008.99	19272.24	44417.01	27091.37
38691.84	47625.9	50860.22	29517.39	32973.04	53243.69	62484.03	22522.6	63875.78	26267.34

LN478.865	LN478.865	LN478.866	LN478.865	LN478.865	LN478.865	LN478.866	LN478.865	LN478.866	LN478.866
26497.02	21824.9	29506.88	33191.98	22387.16	30866.21	31948.78	28571.65	26189.12	22083.92
28688.27	18042.94	31613.75	33050.08	21395.14	25625.14	35402.26	25584.9	27683.81	22490.17
26483.38	20512.76	25676.47	35655.78	20429.54	25862.91	33287.88	28184.93	25411.24	20216.41
29152.67	19397.58	27502.52	33618.45	22697.35	25259.39	29743.11	29589.38	23706.45	23765.74
27179.35	19020.91	28696.86	33586.85	19866.07	28256.91	35598.83	25242.61	23765.97	20183.6

LN478.866	LN478.865	LN478.866	LN478.865	LN478.865	LN478.865	LN478.865	LN478.866	LN478.866	LN478.865
29312.9	32970.87	25297.11	35802.76	30279.09	26267.22	38259.32	22857.55	34359.21	19677.28
25924.83	29997.05	25241.22	40667.79	29976.64	25399.86	36913.49	21921.63	36846.44	21831.04
26827.11	30539.5	27060.87	34065.29	28389.67	23942.63	34857.94	20919.66	31522.6	20114.68
26827.51	31406.89	24854.51	34074.17	26890.75	25562.08	32166.57	21410.67	36712.39	20342.41
26200.39	28518.83	26330.35	36695.77	28386.14	26235.26	34195.31	20866.12	33057.03	22675.29

LN478.865	LN478.866	LN478.866	LN478.866	LN479.177	LN479.194	LN479.212	LN479.212	LN479.213	LN479.213
39420.79	30981.15	35374.29	28488.87	13654.95	15258.45	38462.61	24483.05	31048.33	22887.79
35217.72	31877.59	39383.63	26633.74	12769.87	11550.54	41831.19	23142.15	28533.57	25310.48
37800.05	29941.01	41828.01	27399.67	14063.44	14390.88	38366.26	21407.26	27201.54	22902.6
34172.22	30014.17	39159.72	31118.79	12889.97	12993.47	39269.56	23117.98	27665.09	23477.12
34857.79	32243.83	36699.17	28297.23	11126.74	11800.25	40047.58	19862.99	28745	21674.54

LN479.224	LN479.249	LN479.249	LN479.249	LN479.249	LN479.249	LN479.249	LN479.249	LN479.249	LN479.797
39216.16	18641	14865.52	28872.16	25209.38	19669.76	25444.28	27621.07	23440.48	20226.69
48941.3	14969.88	15991.91	31206.7	26674.43	19736.08	27040.9	24199.85	19489.27	18227.3
65957.76	16243.25	14368.82	30123.99	25828.66	20337.68	23696.18	23619.34	21349.74	21966.44
74444.87	15535.92	15444.29	31210.67	23533.42	21743.39	25910.72	24048.56	22961.37	23578.74
84068.16	17064.34	13860.37	28758.7	22256.67	20677.57	21719.72	22646.41	22524	29453.82

LN480.793	LN481.209	LN482.212	LN482.788	LN482.988	LN482.988	LN482.988	LN482.988	LN482.989	LN483.007
304956.9	169263.6	27386.53	1619009	51619.34	58261.92	67900.13	33977.19	57648.94	47848.46
247548.1	168772.3	26591.95	1301794	49584.7	58333.01	60338.65	34866.12	68343.3	49650.79
283995.6	153174.4	28547.61	1409867	52205.65	54670.95	61232.34	31733.93	65392.38	51524.66
347741.7	141490.3	23291.35	1778856	53575.2	57056.22	61704.73	35710.84	67620.47	46165.54
417647.5	162438.4	25168.67	2224002	54080.46	58325.28	66831.35	32942.34	64523.13	49534.08

LN483.007	LN483.007	LN483.007	LN483.007	LN483.007	LN483.008	LN483.008	LN483.007	LN483.007	LN483.008
39080.39	29448.61	23686.46	22997.43	24589.39	21301.75	34942.88	38215.38	39275.81	36370.59
37707.6	28730.08	21545.34	27909.29	22640.02	21070.94	33332.15	36179.38	44182.67	42362.8
40750.76	29860.71	20072.05	28384.74	22761.24	19866.31	34041.16	30434.29	40180.95	40995.54
38692	26269.57	22561.3	28081.78	25644.87	19988.34	34070.65	33289.19	39988.22	36745.4
38159.44	29055.1	21354.47	29116.91	25130.85	19858.95	32217.84	32923.55	38233.84	37557.01

LN483.008	LN483.008	LN483.008	LN483.008	LN483.008	LN483.008	LN483.008	LN483.008	LN483.008	LN483.008
20764.12	24175.38	30589.89	35100.41	38273.58	40771.1	62207.59	53198.8	45081.87	46744.58
21013.27	20126.13	29496.52	33523.18	39691.89	42997.61	60934.16	50873.02	47135.53	50253.7
22394.55	20813.26	28578.94	34876.05	39904.21	42240.65	65638.71	54295.34	47121.35	46371.32
23997.04	19752.9	30358.1	34560.8	38307.6	39537.07	63015.29	52985.9	45103.1	46280.44
22910.73	20371.34	29812.91	31412.23	37277.85	42631	60121.71	55592.7	42664.79	45622.98

LN483.008	LN483.008	LN483.008	LN483.008	LN483.008	LN483.008	LN483.007	LN483.008	LN483.008	LN483.008
23349.69	28276.57	42862.11	15968.84	60499.42	33501.55	61371.45	26347.86	22423.9	32255.75
24766.78	24551.87	42870.97	12773.55	54108.24	32765.66	68111.56	29567.07	22454.29	32388.96
26768.38	24901.88	42348.63	14074.95	62361.37	34411.78	60375.55	32546.97	22725.02	32355.07
24413.05	27091.46	42285.4	13858.73	59361.19	31380.84	56312.79	29237.51	20042.28	34102.62
24605.01	23899.01	39966.76	13756.64	64826.6	30301.61	64004.37	30389.03	23494.15	31047.13

LN483.008	LN483.008	LN483.008	LN483.008	LN483.008	LN483.008	LN483.008	LN483.008	LN483.008	LN483.008
35155.3	24032.25	51308.1	39659.61	26238.68	21893.09	30017.78	48770.4	15138.38	47037.68
39505.36	25415.99	55740.93	39579.45	24908.17	22495.95	32255.68	44542.61	18766.7	42881.17
32725.54	25441.38	53923.09	39889.42	25301.6	22585.86	31091.23	43757.65	15745.17	45478.84
34459.47	26193.79	57488.13	40435.38	27425.36	20363	30335.83	46240.73	15644.79	50593.24
34486.26	26256.22	63468.39	39366.84	26622.77	22856.37	31614.98	45661.81	14458.88	48475.81

LN483.008	LN483.008	LN483.008	LN483.008	LN483.008	LN483.008	LN483.008	LN483.008	LN483.008	LN483.008
23590.24	34694.54	38528.97	56842.84	33931.06	44638.34	82295.03	31804.32	40304.37	42448.53
20678.89	34819.42	34111.36	55866.39	34185.28	44893.59	84236.44	35618.02	40680.78	39166.14
20195.5	30474.36	39740.41	52941.83	32364.97	46531.11	84552.28	37070.24	40452.41	40298.24
18658.01	32563.71	38161.01	58662.74	31431.63	48165.69	79168.87	33081.57	41111.04	41487.31
20085.24	32318.32	38589.38	58018.08	34574.67	48444.04	80539.9	36522.14	40734.27	42494.73

LN483.008	LN483.008	LN483.008	LN483.008	LN483.008	LN483.008	LN483.008	LN483.009	LN483.008	LN483.008
34467.61	25423.35	29682.92	25311.86	44165.47	34344.8	21389.01	20829.33	55031.59	32415.96
35826.57	26632.21	28362.59	26843.33	47071.2	33693.02	23533.1	20128.22	53031.25	37756.62
41669.55	26333.08	33518.13	24928.98	40408.26	33499.37	23775.83	19795.19	52514.25	35889.49
35506.68	26608.66	31366.2	24207.73	42222.93	33917.53	22263.32	21798.79	54622.66	39512.61
34850.16	24121.67	31434.84	28871.04	47228.81	33496.86	21262.23	20011.6	50329.92	37407.96

LN483.009	LN483.008	LN483.008	LN483.007	LN483.008	LN483.008	LN483.008	LN483.008	LN483.008	LN483.008
27450.95	31221.58	33237.25	44463.09	55244.61	46291.77	23057.26	35536.25	27427.39	45604.29
26781.72	35578.46	30479.68	47502.13	58712.83	45999.55	23057.98	31137.03	28891.58	43281.37
28164.29	34651.02	31695.72	45772.99	61246.19	48461.12	22883.64	37830.7	30822.94	41288.98
27142.12	36469.26	32482.17	50255.08	59235.5	43386.29	22617.1	33140.9	27229.32	44687.45
26845.01	35169.2	29531.2	41599.85	52746.42	43615.14	23216.7	28074.02	27133.44	36611.02

LN483.008	LN483.008	LN483.007	LN483.007	LN483.007	LN483.008	LN483.007	LN483.008	LN483.008	LN483.008
45653.45	64552.13	46769.98	63058.68	56181.12	72617.85	62056.47	17194.7	32452.15	27820.99
43137.32	57960.37	48282.17	65062.43	58980.5	66436.79	53095.32	17309.76	35581.23	24710.32
45927.26	62615.98	47995.4	66272.49	54780.82	64404.28	60186.79	16531.53	35226.94	24390.7
43208.23	59922.76	42416.77	62906.38	58035.91	66434.74	58789.41	16265.51	33987.66	26054.17
48004.44	57115.75	41969.56	64926.33	54252	69376.84	62171.98	14995.64	35062.15	24114.62

LN483.008	LN483.009	LN483.008	LN483.008	LN483.008	LN483.008	LN483.008	LN483.008	LN484.788	LN485.003
52913.49	19347.91	29687.97	33505.25	23327.35	49003.76	50771.29	29265.15	752828.9	33955.84
58740.78	19256.12	34011.99	32696.55	23830.91	47845.45	48927.02	37999.78	629103.7	35264.1
47646.58	18884	32470.78	35650.77	21910.07	47476.2	51198.81	31080.24	695456.1	33366.52
50096.96	19828.18	30909.57	32311.51	22984.54	47779.86	50906.8	35073.27	807307.4	37446.27
48637.83	19015.97	29908.78	34278.01	21766.68	44861.82	49376.79	30399.53	1011569	36221.37

LN485.004	LN485.004	LN485.004	LN485.004	LN485.005	LN485.004	LN485.004	LN485.004	LN485.005	LN485.004
61998.61	44465.24	34756.56	48441.06	17384	22600.67	35592.43	22826.5	28163.28	15246.32
63792.82	40333.29	33034.63	46595.37	20493.1	28708.38	36908.73	24068.51	29171.45	11005.68
64644.91	35041.89	32981.02	50003.31	18947.45	26175.76	39372.17	25809.68	28334.84	11424.96
61408.12	40216.51	28758.41	47660.96	21205.54	24900.64	38301.15	22676.96	28903.27	10126.63
64645.45	37440.91	30041.55	48216.82	20139.24	27122.72	36639.41	24069.65	31372.96	12722.75

LN485.005	LN485.004	LN485.004	LN485.004	LN485.004	LN485.004	LN485.005	LN485.005	LN485.004	LN485.004
19332.63	30206.04	31886.07	56241.34	80605.17	34859.33	35350.14	18676.92	41630.64	57107.42
20647.13	30599.63	35757.1	53912.26	87573.19	34200.09	39634.15	18884.77	37767.84	56572.99
19431.64	28632.44	36277.79	55664.35	74299.47	37227.57	36146.76	18023.83	39481.93	47760.1
21624.76	31880.89	35052.83	51694.47	76831.88	37965.05	35658.73	19539.36	37776.58	61056.14
20625.89	24692.27	33163.74	58223.91	84481.09	36591.98	30134.38	19839.37	36922.3	59805.19

LN485.004	LN485.004	LN485.004	LN485.004	LN485.004	LN485.005	LN485.004	LN485.004	LN485.004	LN485.004
36300.96	71045.33	42110.07	44300.94	38976.88	28424.81	39770.77	39455.27	33415.5	47089.68
34028.9	67857.86	44284.33	40694.01	38146.21	30679.26	37594.64	38735.12	30457.79	49256.19
32372.02	71713.99	42585.33	39090.72	44435.1	32481.26	36718.68	39767.28	34690.06	45987.39
33237.58	71823.43	36312.71	37064.73	39173.49	33415.42	36938.95	38494.07	29614.29	38934.67
36956.38	65587.76	43186.95	42525.64	39680.67	30205.49	35803.82	37877.24	32894.28	45516.04

LN485.004	LN485.004	LN485.004	LN485.004	LN485.004	LN485.004	LN485.004	LN485.005	LN485.004	LN485.005
39477.46	39042.17	58138.43	35691.16	63613.04	56795.94	32757.89	17674.19	37275.76	30705.11
35348.4	41611.7	60523.41	39229.26	55736.5	56880.42	28507.11	17747.55	35464.69	31862.31
34048.3	46752.43	58449.92	34706.8	54439.37	56158.05	26968.53	17730.98	37797.02	32372.66
40545.34	40247.3	60961.74	34403.91	64063.91	57008.98	31145.62	18351.81	34365.8	28815.6
40304.01	38777.84	56278.92	33518.14	62395.68	57064.19	33492.77	15876.75	36456.19	29680.75

LN485.005	LN485.005	LN485.004	LN485.004	LN485.004	LN485.004	LN485.004	LN485.005	LN485.005	LN485.004
23818.17	38974.9	52693.89	44136.29	64564.89	40571.74	44217.69	25865.05	19768.77	27669.56
24209.51	39716.89	52295.74	49833.58	66716.38	41015.54	41790.1	29711.56	19635.43	25951.89
22404.15	40180.96	52135.28	49393.46	66851.21	42548.9	40763.89	27589.67	17711.01	30123.06
24188.4	37141.9	54078.67	50606.4	67399.4	32470.38	46901.7	27134	19749.46	28651.17
21444.95	38062.26	52540.02	50045.4	68005.92	38701.45	42860.22	28463.58	18241.23	29283.55

LN485.004	LN485.004	LN485.004	LN485.005	LN485.004	LN485.004	LN485.004	LN485.005	LN485.004	LN485.005
29604.56	49030.76	53721.46	24021.51	30160.28	33614.3	51796.58	16436.77	54308.39	28930.66
31646.1	46508.7	55952.05	26790.73	29547.24	37794.11	49236.3	17332.91	57226.69	29286.44
29942.85	45909.6	56983.38	23357.06	30167.92	37887.69	58830.06	16727.42	59052.01	33281.06
33051.97	39960.53	52905.77	25011.58	28378.89	37002.81	52291.84	16503.86	53511.82	32081.73
25205.16	39970.04	52441.76	24500.43	29558.64	33262.45	52079.65	16991.63	51112.11	34443.83

LN485.004	LN485.005	LN485.004	LN485.004	LN485.004	LN485.004	LN485.004	LN485.004	LN485.004	LN485.004
64769.02	16948.14	49310.42	52332.99	35983.62	41219.16	26468.42	71594.14	48149.9	51897.09
58895.48	15564.01	45576.89	50022.44	34402.9	41080.82	27547.41	73643.93	48884.45	48585.28
63636.8	16478.99	48148.75	51243.19	37236.01	41159.92	24500.88	63369.74	48433.15	54379.59
61607.1	16903.53	48321.04	49704.28	33758.47	43673.47	27889.1	68621.66	47565.04	51010.66
61029.02	16344.57	48034.81	46747.58	37205.18	40149.8	26351.6	67777.05	50519.01	50703.98

LN485.004	LN485.004	LN485.004	LN485.004	LN485.004	LN485.004	LN485.004	LN485.004	LN485.004	LN485.004
58322.33	48172.41	30797.67	63889.13	63943.53	52069.07	37154.46	28752.56	34071.63	60403.32
62259.2	43611.74	30068.93	60078.11	68445.56	52141.41	35700.97	30667.78	31985.78	61848.09
67330.37	45612.63	31521.88	64405.13	68739.17	57222.64	33646.85	27777.29	34750.19	65548.05
62534.05	48299.63	29483.54	59264.47	66708.04	48540.4	33406.03	24899.06	29928	69342.12
66316.01	44606.3	26798.29	60796.03	66308.11	51266.21	35036.82	28071.66	31167.52	61743.24

LN485.004	LN485.004	LN485.005	LN485.283	LN485.283	LN486.786	LN487.002	LN487.001	LN487.002	LN487.002
54077.97	83037.11	25286.92	51905.63	30434.58	265301.2	14427.47	130576.8	74884.27	29937.42
52714.97	87361.75	27081.19	42547.19	26940.3	254090.5	9768.98	136942.3	83220.32	34388.22
47042.76	70546.49	27096.11	48142.17	33823.73	278965.1	13685.89	126612.5	83571.16	29850.58
43727.03	78871.02	25077.18	37346.89	19773.97	321003	13439.38	126955.5	73767.15	30584.9
43320.58	71807.83	27959.44	55012.35	24267.58	398622.3	13291.46	123945.2	78878.43	26926.24

LN487.002	LN487.002	LN487.001	LN487.002	LN487.001	LN487.002	LN487.002	LN487.001	LN487.002	LN487.001
39324.31	35192.04	107406	17661.94	80617.42	52618.24	38807.56	104686.9	47799.21	41920.63
44016.23	33569.47	109313.1	19177.72	75899.48	47152.43	36192.29	103139.6	51066.92	35359.12
45624.97	30394.05	105939.4	15875.47	85607.14	46906.95	35745.27	109369.7	45800	33139.45
44386.63	32709.49	105726.1	15677.18	72849.29	49207.93	34745.82	117599.5	50615.84	38667.51
43273.91	32423.43	109164.7	16637	79391.1	43728.5	35614.06	102355	51412.96	33307.95

LN487.002	LN487.002	LN487.001	LN487.002	LN487.002	LN487.002	LN487.002	LN487.002	LN487.002	LN487.019
90665.56	38976.8	47589.61	31630.54	28400.83	50918.64	48202.55	42795.13	82616.63	81797.1
95039.91	36362.67	45469.05	34420.7	29686.19	46476.63	47620.54	42587.07	81392.81	70133.32
87546.33	40348.21	51438.54	29660.69	28021.37	41992.25	44930.86	41704.29	74406.55	73029.83
92342.58	39286.01	42360.62	33060.39	26589.11	47428.86	47253.49	43485.9	82592.31	67476.45
88391.61	30443.72	49166.92	27593.16	27618.25	45009.92	44247.31	44506.52	63109.51	64013.32

LN487.020	LN487.02_	LN487.02_	LN487.020	LN487.02_	LN487.02_	LN487.020	LN487.020	LN487.020	LN487.020
79470.04	45930.08	18675.74	85637.73	67639.34	67424.56	28961.42	96602.6	108281.2	52097.08
41146.3	59657.69	11053.85	70095.49	68891.33	67260.71	33803.19	55430.3	83695.72	39182.16
37551.61	56045.55	14960.8	73303.38	62566.65	77020.03	21342.01	64700.66	73604.08	54593.61
48007.55	82770.76	14534.67	65160.26	88259.93	81279.3	22773.21	83752.38	72182	52399.77
41478.97	37378.4	13172.47	51493.71	74991.84	62946.94	26228.48	57392.32	87164.79	47717.36

LN487.019	LN487.020	LN487.02_	LN487.020	LN487.019	LN487.020	LN487.020	LN487.02_	LN487.019	LN487.020
51329.4	69221.04	111136.5	69329.03	83303.55	38540.51	67697.17	102593.9	58124.29	42627.29
71948.81	76361.68	78313.44	48386.36	100381.5	39677.35	56952.58	76996.88	58498.09	50734.22
42359.62	87995.91	75229.2	57468.75	80654.98	47570.64	84991.26	96299.36	51162.1	54025.16
43765.49	83643.63	77249.07	48971.7	116764.3	56147.65	65204.49	91587.19	60382.27	48368.73
52942.17	91115.94	55744.16	40731.59	103959.5	30106.54	68307.55	79076.01	59458.39	36999.63

LN487.020	LN487.019	LN487.02_	LN487.020	LN487.019	LN487.020	LN487.020	LN487.020	LN487.020	LN487.020
24006.17	107882.3	82460.74	60313.02	70758.2	53289.77	56117.41	72272.8	25803.01	38105.08
47042.22	86514.47	83707.02	54349.24	70741.86	51543.34	42102.06	182451.6	19129.05	50887.08
35977.6	140835.2	72144.37	95854.71	84101.22	42345.82	55286.88	81654.4	21833.21	46887.07
37732.93	109023.2	74625.14	76688.39	96643.19	54334.34	63390.78	87031	21522.28	43699.07
32858.75	112071.1	89120.53	74515.85	75000.96	43076.4	49829.47	130942	22688.3	37856.09

LN487.02_	LN487.020	LN487.019	LN487.020	LN487.02_	LN487.02_	LN487.02_	LN487.020	LN487.019	LN487.019
60795.44	27931.18	62530.79	50425.35	56297.77	78506.53	81754.65	71652.56	104396.9	121783.1
97729.05	25776.21	65375.73	61478.73	85207.53	87852.12	88714.04	109139.2	76427.59	70591.76
60529.66	25627.42	82590.8	51376.01	49664.93	83093.3	104530.4	53061.64	81015.83	121589.7
50961.44	17621.82	98896.2	65567	43607.87	85909.3	79789.8	67383.7	61891.42	58257.55
66225.32	23280.33	76062.05	60547.96	46588.09	92045.75	113247.2	74781.76	74396.84	65578.33

LN487.020	LN487.02_	LN487.020	LN487.020	LN487.020	LN487.020	LN487.020	LN487.020	LN487.020	LN487.020
19690.22	59150.78	21481.6	41333.38	39666.28	97291.29	44191.24	33599.84	35964.33	57311.75
25905.73	65820.49	23595.46	41067.31	56806.44	79304.51	53468.55	43745.76	60083.46	62248.28
23402.55	62833.78	24564.26	39894.03	45386.53	87378.25	60265.04	30394.09	69965.41	39880.55
23201.42	85163.36	20243.39	46668.84	44590.63	87197.64	61137.32	31572.4	30462.87	37734.03
20150.91	79675.85	19056.06	43725.07	51010.19	77511.07	45913.07	36132.68	51785.06	40962.51

LN487.020	LN487.020	LN487.020	LN487.020	LN487.02_	LN487.02_	LN487.020	LN487.020	LN487.019	LN487.020
54036.22	40334.4	26292.06	46488.87	109220.1	60698.47	61835.7	31628.84	102226.3	35192.33
57702.68	49095.3	18640.41	44322.82	94027.36	93358.98	49475.49	27053.25	94107.21	55714.16
44052.77	41284.51	33417.66	42531.58	77574.78	71626.6	52002.96	19096.78	87256.11	35271.3
38948.83	36580.71	25763.5	47812.72	93880.84	63562.77	58855.35	24685.24	116613.6	40676.02
51575.1	40193.19	27803.63	41429.17	70346.36	101253.3	50466.57	19737.1	100124.4	81654.65

LN487.02_	LN487.020	LN487.020	LN487.020	LN487.020	LN487.020	LN487.019	LN487.020	LN487.019	LN487.020
81230.27	39711.85	71431.79	33581.01	24568.38	41472.55	97245.35	25154.82	99534.93	67928.49
70894.93	65638.98	80217.07	25628.48	32192.7	73992.84	113782.5	34286.15	81180.14	59100.68
65742.39	57971.08	84069.35	30309.16	27344.08	46656.86	137833.1	26541.99	93206.73	56647.35
120207.1	55808.81	76152.3	26421.36	21940.8	41749.89	112314.7	27307.86	94403.96	59932.2
75883.15	76758.72	69769.22	35200.21	38992.21	51998.35	119843.6	27386.27	108230.9	66037.54

LN487.020	LN487.019	LN487.020	LN487.020	LN487.020	LN487.020	LN487.020	LN487.020	LN487.153	LN487.226
25425.73	75448.99	41315.92	31269.62	52070.86	33711.97	57584.02	37048.82	123587	154546.5
37037.03	98628.74	49629.96	47103.75	50657.26	48089.21	43020.49	43119	43199.76	158767.4
30373.08	98814.01	33940.88	40499.67	44818.8	53124.32	39335.83	65434.65	28027.9	170634.8
24350.68	115943.2	36947.51	33238.24	47882.44	38814.37	47141.12	41311.95	93107.76	121992
23007.36	135969.9	37023.64	38231.23	53587.65	42553.82	42180.92	42319.85	98462.16	141409

LN488.229	LN488.784	LN489.195	LN489.195	LN489.195	LN489.195	LN489.195	LN489.195	LN489.253	LN489.278
28285.65	109933.2	25270.17	18102.95	17561.85	28507.43	27831.61	24070.98	61217.25	15163.99
29827.18	83790.39	25922.6	16854.32	18095.06	28669.68	27586.11	22586.31	73288.95	14447.92
27341.24	86919.81	27724.46	17668.23	17462.81	26567.58	24813.27	23496.47	108108.9	10661.15
26170.92	84465.74	26947.33	16464.49	15894.36	26473.29	32294.66	25754.05	135070	14299.63
20995.32	109420.6	26989.74	16898.59	17982.21	27868.28	25730.46	25998.65	142387.9	8396.899

LN489.784	LN490.763	LN490.783	LN490.995	LN490.995	LN490.995	LN490.994	LN490.994	LN490.995	LN490.995
46531.52	37352.15	43579.85	44071.24	18007	40712.43	60248.69	80701.15	59098.97	75544.9
46768.33	35035.81	43659.41	58104.54	21171.67	41707.73	69831.93	84666.33	61495.13	52148.83
48012.41	37694.65	45582.68	53378.03	19431.84	41334.99	69309.6	81391.83	67016.8	64632.45
46290.69	41367.65	43239.19	42164.49	16174.13	62229.88	36596.43	80431.57	68697.66	76746.87
43731.7	47802.17	50048.91	39357.06	16622.87	37290.26	72424.32	84418.03	70254.14	66583.95

LN490.995	LN490.995	LN490.995	LN490.995	LN490.995	LN490.995	LN490.995	LN490.995	LN490.995	LN490.995
49737.65	57056.8	44204.67	45656.59	34987.44	23731.37	30468.32	51483.17	47887.9	44339.02
53915.17	70654.72	40262.65	49297.12	35407.81	27494.92	22864.16	50360.99	57187.04	45659.93
51562.43	69071.16	52142.59	33687.96	29914.72	27372.38	31248.97	62525.43	49790.05	35474.88
53340.82	50410.82	43783.97	45449.64	45277.72	18572.69	26084.42	66570.08	54210.88	44228.07
49887.34	57798.88	41498.96	46729.21	34712.84	27021.25	34659.96	73668.13	63459.61	47523.78

LN490.994	LN490.995	LN490.995	LN490.994	LN490.994	LN490.995	LN490.995	LN490.995	LN490.995	LN490.995
77051.24	70271.09	37541.45	69091.59	90512.44	37251.49	89249.4	31471.73	59092.72	66504.82
74672.82	96964.79	46279.57	51643.6	128642	69329.43	64682.49	57760.51	49469.43	109963.4
112492.5	71017.19	29140.54	63270.83	109584.5	37190.74	84126.95	52391.19	82594.73	118074.4
81463.81	55735.73	36410.53	69289.98	85169.02	46125.08	88087.27	39742.52	59791.64	61797.09
88940.97	77752.59	31179.19	59916.59	74447.8	55185.34	70622.2	39644.33	62498.02	65416.97

LN490.995	LN490.995	LN490.995	LN490.994	LN490.995	LN490.995	LN490.995	LN490.995	LN490.994	LN490.995
38919.68	47783.72	61080.09	57101.04	36157.99	66545.24	22611.01	70896.83	105753	63659.48
46104.66	69475.72	74108.09	58102.53	36039.51	89456.77	21312.83	101816.4	95076.14	52406.86
38060.69	49225.94	60315.36	63219.99	32372.53	51595.81	22337.71	67588.57	105571.9	53272.83
33319.68	63337.99	75307.61	53610.66	38030.38	94232.63	23818.67	105242.4	82478.5	57067.98
34527.51	63764.23	55957.17	68461.3	41683.27	69164.55	32894.55	128864.3	151079.4	70778.99

LN490.995	LN490.995	LN490.995	LN490.995	LN490.995	LN490.995	LN490.995	LN490.995	LN490.995	LN490.995
50139.5	35071.14	36584.13	53403.68	88098.19	24723.4	40220.36	27120.77	23944.75	33958.06
61904.1	33789.62	40950.92	71908.45	104328.8	21530.82	49170.52	33812.02	21328.84	47340.18
40720.43	33759.16	61954.14	54440.84	96297.72	19506.24	56721.86	24214.54	23159.03	35850.18
58467.93	33440.88	48945.37	64594.77	64987.66	25119.22	32467.99	37514.68	19722.43	36501.87
52360.49	33840.42	47680.46	51749.94	82944.28	23270.54	45294.35	24101.6	23831.58	47054.88

LN490.995	LN490.995	LN490.995	LN490.995	LN490.995	LN490.995	LN490.995	LN490.995	LN490.995	LN490.995
47131.82	58297.05	57869.88	82659.8	37082.36	52938.72	16950.22	25613.34	71596.75	39511.09
53002.09	68969.21	65348.54	75166.7	37612.64	54089.31	23649.65	23992.06	74550.58	33033.37
42876.91	66852.79	65771.04	74106.3	48933.66	40197.06	18407.48	19875.11	109216.1	36837.99
68253.78	57829.56	78169.3	76869.88	47922.92	79243.29	14768.86	20552.31	79981.61	32848.84
44593.35	60046.58	52579.42	70190.69	52801.89	38011.34	21096.89	20551.78	65625.34	45294.95

LN490.995	LN490.994	LN490.995	LN490.995	LN490.995	LN490.995	LN490.995	LN490.995	LN490.995	LN490.995
56439.15	82022.99	53141.52	40819.18	34029.04	40806.55	74612.52	40127.09	23726.87	42919.29
56131.34	81769.16	51812.22	42667.02	39864.12	49061.17	71550.6	51328	30610.52	42960.31
75122.28	69537.44	66989.82	51489.37	46631.07	50537.67	68975.87	51043.98	19151.69	51994.87
51707.56	84571.61	53589.67	42399.45	33263.58	39167.26	64841.24	53896.49	25677.29	32525.82
63342.34	89311.68	40185.15	35792.84	47194.73	37228.99	61114.98	42286.72	20359.92	44844.79

LN490.995	LN490.994	LN490.995	LN490.994	LN490.995	LN490.994	LN490.995	LN490.995	LN490.995	LN490.994
40616.85	80410.92	40582.25	97085.39	49709.87	51152.95	38117.73	48795.45	36453.02	88752.11
35984.39	81327.23	38193.43	74692.27	54385.75	55047.36	38174.37	58794.16	29990.8	91505.59
38608.14	80190.03	47649.02	116115.7	64498.35	60938.55	44457.52	41675.85	35066.73	100807.3
47938.73	106257.8	33598.53	102804.6	46573.44	56253.41	49276.55	48380.26	31961.69	67190.49
40394.26	80475.25	56791.15	80281.03	53268.17	77143.97	43414.53	48415.19	45615.62	147500.5

LN490.994	LN490.995	LN490.995	LN491.032	LN491.032	LN491.211	LN491.211	LN491.212	LN491.250	LN491.249
92631.44	53607.64	25475.96	75662.47	71249.2	19316.32	34586.66	26219.8	31824.76	29891.56
122219.3	72701.1	27535.75	79594.72	66720.17	21510.64	33917.56	28806.36	30293.09	33326.21
89790.06	54553.76	24909.51	75503.25	78814.3	21076.49	30892.72	23800.43	33135.84	31362.16
95738.84	65676.78	24225.19	78286.18	75374.97	21676.87	35110.3	25357.56	37261.16	30774.22
78398.2	53343.07	25784.74	78882.31	69578.49	18581.3	29794.83	26581.33	37660.25	30492.09

LN491.249	LN491.359	LN491.358	LN491.359	LN491.359	LN491.36_	LN491.359	LN491.358	LN491.359	LN491.358
24519.2	193253.5	113007.7	117405.1	142444.8	163613.2	157701.7	178192.8	203103.1	305993.6
25608.06	214764	100566	164767.8	132221.7	202014.1	230837.2	159848.8	212433.5	202278.4
22346.04	233081.5	115339.2	170117.4	125107.5	167606.8	237804.1	160122.2	192542.5	176332.5
18395.17	241891.6	113225.3	168319.8	131438.8	163434.3	236102.6	162857.2	211606.8	188593.6
21948.82	216180.7	97799.6	185413.4	133691.7	160230.7	238260.4	156067.3	205572.5	186596

LN491.359	LN491.359	LN491.359	LN491.359	LN491.359	LN491.784	LN492.361	LN492.362	LN492.362	LN492.795
63924.63	79681.87	119402.8	118806.4	180709.7	24364.49	71163.6	50073.97	56727.99	14586.75
69982.34	83361.12	129799.7	128256.9	184363.7	22974.05	71559.62	51777.57	52057.32	13197.59
60980.47	82615.9	117692.2	123326.2	176174.6	27022.81	65647.23	47996.17	55411.31	12016.31
69579.75	91589.53	121162.9	126453.1	175777.9	17641.4	67630.62	52509.26	51381.64	14994.9
62038.74	83253.54	121791.1	120646.6	178998.3	22231.66	67592.66	52075.2	52764.86	13194.02

LN492.821	LN493.010	LN493.189	LN493.189	LN493.189	LN493.190	LN493.189	LN493.189	LN493.189	LN493.189
19030.41	36641.71	28401.8	37762.87	28119.2	25313.43	24801.43	26696.49	35472.89	37889.87
14170.75	40594.47	26451.34	35537.73	24026.52	25881.89	25896.41	24043.55	38234.41	40827.55
14233.33	39870.55	27374.03	34827.51	29056.9	26930.68	27734.53	24141.13	32883.24	41099.59
19619.85	43209.23	27227.24	33988.75	27753.68	27916.21	24871.04	23609.86	34398.55	42314.19
18199.08	39722.6	29540.57	33580.59	30069.16	26051.34	26265.35	23097.9	37017.96	38610.31

LN493.189	LN493.190	LN493.228	LN493.228	LN493.228	LN493.228	LN493.228	LN493.228	LN493.228	LN493.819
42756.13	35888.56	44812.41	46064.12	35497.2	41537.01	31442.91	33061.39	24859.01	16778.47
41923.64	36199.43	45849.86	46957.5	32349.69	35972.36	32912.57	31902.85	25961.71	14691.84
46177.2	34455.71	41053.66	47963.02	34879.35	38291.24	32048.51	37156.23	26997.41	17164.6
40757.5	35194.17	46784.76	43441.96	33659.54	39300.22	31923.3	36093.07	25131.74	13946.29
48316.85	33903.06	45840.7	43653.98	39976.86	41785.54	34549.25	34954.62	29893.85	14584.79

LN494.79_	LN494.910	LN494.988	LN494.988	LN494.988	LN494.988	LN494.988	LN494.988	LN494.988	LN494.988
98395.55	38706.09	29137.38	39333.91	24046.71	35741.41	29690.86	45959.56	32014.22	41313.69
88220.35	38788.9	28383.02	36295.88	23714.21	35968.4	31470.95	46624.17	33819.23	42550.51
78244.74	38796.08	30747.54	35854.62	23077.64	35976.43	30322.48	45386.17	34059.71	41911.46
99239.27	36919.33	32198.07	37473.26	24300.37	36727.7	30277.11	45123.07	33042.37	44941.88
130058.1	35186.81	30870.64	38307.15	23693.35	31686.67	31828.4	44978.18	35508.2	45947.31

LN494.988	LN494.988	LN494.988	LN494.988	LN494.988	LN494.988	LN494.988	LN494.988	LN494.988	LN494.988
51263.34	23518.39	44953.66	44770.22	43834.3	33632.12	38738.58	37386.93	43177.45	29230.42
52918.55	19063.88	45970.57	43501.76	43786.06	34198.89	39204.48	34761.71	43885.53	25835.85
54068.96	21073.82	40728.71	44838.7	43229.76	35283.41	39133.11	37743.42	46081.59	25702.25
49824.46	28125.69	43583.87	41665.55	45166.81	36191.26	41706.9	39696.45	45299.94	27366.89
45225.9	24816.04	43689.99	49355.53	46141.72	33687.86	41460.6	40489.82	44049.83	25347.88

LN494.988	LN494.988	LN495.045	LN495.045	LN495.045	LN495.045	LN495.045	LN495.045	LN495.045	LN495.244
61209.73	32474.82	54537.49	59930.7	46310.58	45148.38	42966.62	54692.75	52728.1	33388.19
75643.3	32313.14	51722.4	58974.89	50142.45	49537.02	43320.15	52764.55	57026.82	30035.13
59805.6	32767.61	52554.47	62207.25	51770.84	46441.48	44245.31	53809.36	58004.53	36677.09
68062.27	32012.27	57922.82	64496.68	47582.82	43233.43	42667.87	43511.86	57210.54	32442.25
63118.68	30332.38	54617.31	19322.62	17186.84	45193.95	19369.16	51842.3	24599.38	31062.69

LN495.244	LN495.244	LN495.791	LN496.79_	LN497.181	LN498.185	LN498.783	LN499.001	LN499.002	LN499.002
19514.34	30922.11	14669.16	24110.97	145463.2	24695.67	36684.56	37304.89	36133.32	22953.65
20298.98	28881.06	12217.78	17420.15	125751.8	26847.42	36764.36	39308.94	37677.24	24755.81
20042.72	30697.24	13647.28	17277.51	131542.8	21882.59	50249.62	25756.69	33386.38	23171.92
18876.87	32974.21	18854.17	25611.04	119492.4	18217.81	43163.98	38865.19	34618.27	23805.38
23335.79	27412.53	24176.99	25738.32	97180.82	19648.91	57930.13	40247.61	34506.38	27529.99

LN499.002	LN499.002	LN499.002	LN499.002	LN499.002	LN499.002	LN499.002	LN499.002	LN499.002	LN499.002
30303.88	35082.06	26259.53	25415.01	17761.02	21394.84	26193.94	33834.19	32273.78	38270.94
36925.35	36135.78	29704.75	23754.15	19700.66	22107.31	28217.46	32833.11	33794.17	39472.23
31998.93	33143.96	27148.16	24214.43	19168.27	22484.34	27011.62	31558.46	34267.84	38386.26
32670.93	36528.89	27049.42	20460.59	19209.83	18677.31	26303.48	31929.06	33201.45	38746.18
28500.73	34451.88	28051.71	21577.48	17027.78	20602.57	27264.29	31200.99	32199.06	35948.63

LN499.001	LN499.002	LN499.002	LN499.002	LN499.001	LN499.002	LN499.002	LN499.002	LN499.002	LN499.002
40674.09	30207.74	39290.79	41297.21	28247.61	29634	24906.66	21680.77	25826.66	22886.33
41382.17	27676.86	40004.43	43306.6	27787.9	34312.86	23537.54	19117.14	29840.13	20685.1
37941.05	31443.61	39542.24	39386.96	27673.12	28521.14	25871.7	19395.01	30235.23	20560.57
40410.84	30891.24	36192.31	44647.78	30585.84	30919.15	24601.93	22130.26	28158.42	17512.85
44821.34	26524.58	38346.89	40095.91	27907.61	29218.86	24639.2	19521.84	27048.36	20509.16

LN499.002	LN499.002	LN499.002	LN499.002	LN499.002	LN499.002	LN499.002	LN499.002	LN499.002	LN499.002
33478.07	37871.64	17978.11	15748.77	19752.52	22555.12	30473.67	21594.26	39450.83	14332.99
34777.09	39915.09	21575.25	15066.4	19042.27	22260.63	33908.58	23187.73	36215.1	13243.47
32085.49	36058.9	17089.7	14922.36	19612.77	18725.34	30223.73	20992.16	41311.29	13557.79
32971.43	39033.24	19948.69	17617.32	22686.63	19226.07	33183.71	23355.84	38338.46	12948.03
33792.9	39851.69	20283.61	17142.76	19012.72	19090.65	33921.2	22477.43	38633.68	11894.65

LN499.002	LN499.002	LN499.002	LN499.002	LN499.002	LN499.002	LN499.002	LN499.002	LN499.002	LN499.002
41816.56	27751.84	35850.74	25364.42	23528.92	38612.41	27635.39	33708.7	30217.43	21828.45
39785.47	25058.29	38176.69	27999.32	23633.17	38607.54	24293.96	36486.17	26802.22	22277.41
39315.86	22901.73	33176.88	25774.51	23758.11	33924.52	26319.77	34650.33	27572.44	20747.33
40080.7	23888.59	34232.12	27116.58	20666.51	36348.75	26363.01	33267.88	30579.95	22981.77
40328.73	23483.72	35940.89	25342.39	23023.62	35607.58	26583.68	34927.23	27921.96	23714.59

LN499.002	LN499.002	LN499.002	LN499.002	LN499.002	LN499.002	LN499.002	LN499.002	LN499.001	LN499.002
33656.79	34629.11	22101.35	33405.4	16979.98	25850.61	50243.71	16011.13	37688.25	38000.26
33450.52	36012.78	26297.47	32517.04	14758.42	25232.65	53800.73	17451.33	33296.97	41412.56
33638.87	37041.53	22204.25	34360.43	15958.77	26147.35	48610.42	14936.21	37497.48	36158.87
31456.05	37221.9	23635.15	35859.47	15806.43	25562.69	52120.14	15214.15	38377.47	36786.38
30767.39	34994.38	20937.12	36293.72	16112.23	24173.16	51377.82	13328.64	36639.46	37923.95

LN499.002	LN499.001	LN499.002	LN499.002	LN499.002	LN499.002	LN499.001	LN499.002	LN499.001	LN499.001
47081.56	44175.72	30645.03	19187.14	18651.27	51390.17	41858.83	32738.33	36734.44	37909.9
49940.38	44285.08	33132.74	20286.83	21033.39	56872.86	44856.53	29245.39	37736.1	37531.18
48143.45	44889.8	29293.72	17947.79	17605.32	46799.77	40003.74	34532.81	34440.82	38792.26
46690.15	43707.98	28806.34	18423.22	19740.46	52838.51	43955.36	33562.63	33357.38	40037.84
50258.25	44485.75	32795.3	18911.84	17140.03	53252.34	45669.32	33255.22	34457.86	37509.36

LN499.002	LN499.002	LN499.002	LN499.002	LN499.002	LN499.002	LN499.002	LN499.002	LN499.002	LN499.002
46955.28	25774.69	44422.24	50656.9	17758.47	58251.47	19322.07	18854.94	18714.75	39908.57
44854.5	25806.47	43664.15	53879.28	17954.05	57455.07	22006.42	20516.58	17428.36	39595.16
45037.73	26110.43	41636.82	56634.64	19392.8	50558.43	19534.05	19668.25	15533.9	43405.04
48853.77	27152.99	42989.62	58648.72	19060.08	55130.31	21505.14	20958.22	15966.61	40555.14
41126.56	26762.56	41450.82	52451.27	19447.88	56238.25	20225.36	20451.05	18544.58	40421.53

LN499.002	LN499.002	LN499.001	LN499.002	LN499.002	LN499.002	LN499.002	LN499.002	LN499.002	LN499.002
31922.46	30095.24	27172.86	20344.96	23091.08	49671.51	33564.68	16405.3	17406.9	51187.31
34026.57	29414.84	31572.35	19959.08	21168.84	52411.39	28148.4	15600.64	20651.78	48489.98
33665.53	31597.4	28047.21	22752.57	21478.47	46221	29957.28	16534.9	16358.72	47155.4
33753.52	29650.95	28187.2	20661.61	19813.96	51899.26	30275.72	15347.13	19088.28	48327.95
36032.35	27815.7	27801.38	18729.55	20491.3	45908.87	30515.51	14638.93	17319.95	44370.15

LN499.002	LN499.039	LN499.039	LN499.182	LN499.778	LN500.451	LN500.782	LN500.829	LN500.999	LN501.779
17346.84	47274.92	56294.75	32115.69	88295.15	27832.7	27404.74	22825.05	55522.6	35451.51
17379.39	44087.48	58966.08	32539.29	65916.54	27299.58	22920.24	20399.95	58316.07	25288.13
16095.92	44286.23	59674.8	29248.97	71785.59	27844.09	25666.37	21502.85	58535.75	30614.19
17369.49	48213.3	60304.87	28856.51	96014.36	26527.49	29478.47	24817.86	58185.71	35309.34
14936.38	46550.32	28635.06	27692.62	103507.6	22972.28	37652.34	29044.55	59588.59	47056.63

LN502.223	LN502.994	LN502.995	LN502.995	LN502.995	LN502.994	LN502.995	LN502.995	LN502.995	LN502.995
6068.079	28098.44	18083.33	34554.82	14501.58	41791.86	22295.95	15784.21	21239	24756.36
3952.366	26089.78	18485.12	32263.59	16544.95	45370.69	22625.63	18959.49	22541	26839.93
7808.098	29222.56	17608.39	35138.15	13412.7	43260.86	26035.56	16177.99	24107.46	27164.38
10299.48	27753.31	17908.96	34489.97	15753.42	43057.98	19865.5	14907.71	21469.22	26195.81
10204.8	32586.94	16360.27	34160.73	12983.51	45834.25	22327.49	16684.9	20103.01	26094.19

LN502.995	LN502.995	LN502.995	LN502.995	LN502.995	LN502.995	LN502.995	LN502.995	LN502.995	LN502.995
20752.54	24526.37	26817.49	35577.34	28274.8	27328.27	41300.86	42043.84	16472.23	23180.89
22973.65	25262.57	28920.26	32638.42	34514.21	26682.73	42880.89	39883.48	21923.35	21678.63
19788.33	25957.25	30446.98	36815.34	34735.18	26315.59	41386.52	43219.67	19251.01	24416.29
18750.74	27814.27	29139.56	31264.84	33170.25	25411.97	40299.8	41745.94	18919.4	23279.27
19356.01	25258.78	26171.86	33976.44	33816.22	26774.39	39661.09	41209.62	17193.26	21498.28

LN502.995	LN502.995	LN502.994	LN502.995	LN502.995	LN502.995	LN502.995	LN502.995	LN502.995	LN502.995
57451.87	29424.59	50814.19	38343.97	18122.03	18391.42	20301.58	28431.7	16482.11	20466.39
57127.85	28969.75	52402.23	40337.05	18180.89	19640.74	21206.47	27495.97	16866.24	20590.4
56525.83	29378.39	54318.93	43866.75	18903.15	18973.62	18924.62	30026.04	19057.37	21416.92
54435.81	27652.56	51106.6	40201.52	17400.56	15887.03	18675.99	26541.58	18123.86	20171.54
52283.59	28191.21	55290.13	32632.99	20421.68	17180.85	17625.36	27788.51	16777.43	20668

LN502.995	LN502.995	LN502.994	LN502.994	LN502.995	LN502.995	LN502.995	LN502.995	LN502.995	LN502.994
20191.49	34301.09	57084.97	44415.21	49257.08	54492.82	32895.18	51812.38	19770.54	64418.78
19604.89	36437.48	58404.32	50766.29	46430.87	55265.78	35573.21	51189.69	20822.31	62010.89
23450.63	35373.83	62883.48	51965.46	50057.18	54376.79	39395.49	51459.88	23054.57	67479.6
17272.25	34681.79	58728.12	52012.44	47032.63	53987.04	35808.51	48342.7	21227.62	60211.23
19498.4	36717.2	55940.02	48773.88	48692.51	53832.46	33826.37	44953.39	21432.61	65701.32

LN502.995	LN502.995	LN502.995	LN502.995	LN502.994	LN502.995	LN502.995	LN502.995	LN502.995	LN502.995
43619.48	27798.62	42092.86	22900.09	64781.79	27377.46	32888.9	49506.19	35133.1	50749.55
41609.17	30230.58	34715.68	23656.46	65402.58	27621.51	28477.02	47562.18	33054.51	48935.56
42910.02	28274.84	40934.51	24282.47	70438.59	28795.99	31309.84	51232.91	37462.37	51297.5
40891.17	32110.38	39730.13	23225.72	63810.32	24392.46	29389.11	51088.5	34672.12	54800.97
41339.71	31193.71	40202.22	24730.25	62155.59	27312.56	29867.68	48547.77	33650.72	50348.43

LN502.995	LN502.995	LN502.995	LN502.995	LN502.995	LN502.995	LN502.994	LN502.995	LN502.995	LN502.995
25122.42	32301.35	30385.08	21240.89	20876.76	17114.17	38880.6	30461.7	23923.56	23865.61
29301.89	31739.66	32606.13	24616.99	23050.5	17716.31	42355.04	31722.95	26643.79	20674.07
26367.8	32627.03	32954.52	25550.83	21482.53	16078.4	42106.84	31756.82	27237.07	22052.64
31235.76	31181.22	30937.71	22952.1	24204.22	16933.34	37279.68	31970.01	25105.67	24245.55
29669.22	30029.79	31691.06	21859.74	23039.71	16041.38	40253.53	29211.79	24919.6	21796.37

LN502.995	LN502.994	LN502.995	LN502.995	LN502.995	LN502.995	LN502.995	LN502.995	LN502.995	LN502.995
24427.48	51236.09	25206.13	38986.08	26633.65	25405.1	33341.23	21376.51	34171.96	45044.33
20251.22	49204.48	25004.81	38591.66	23792.92	24737.69	33758.36	20513.76	35057.67	47879.03
21979.39	50300.74	23509.9	40807.17	24551.74	27343.49	32119.38	22730.2	35098.78	48492.77
20744.29	55362.12	22414.29	42066.38	24568.88	24657.03	33498.77	21127.32	34255.19	47992.84
21332.76	49776.14	23671.65	42948.25	26589.66	23690.82	30950.23	20926.29	33081.48	44154.02

LN502.994	LN502.995	LN502.995	LN502.995	LN502.995	LN502.995	LN502.995	LN502.995	LN502.995	LN502.995
46027.68	50163.94	47063.99	14383.06	20063.5	51924.97	27897.68	40694.92	21480.11	24359.05
46795.55	47839.15	44113.07	13730.28	21675.51	55020.17	27258.36	42899.07	21580.87	21617.95
51845.4	54788.46	45903.98	12100.18	20232.09	52999.86	28293.53	45673.17	22183.24	22422.42
45572.65	52663.27	43685.55	11984.61	19659.41	48859.05	23756.97	40495.54	22143.4	21356.99
47376.03	45526.99	43229.71	12769.28	19561.09	50389.61	28078.86	42463.53	23982.6	22059.52

LN502.995	LN502.995	LN502.995	LN502.994	LN502.994	LN502.995	LN502.995	LN503.053	LN503.053	LN503.053
49742.75	34458.49	31092.55	98339.54	38827.12	18975.51	34550.32	22682.63	17875.99	29182.18
47619.53	32310.8	32527.88	99486.45	38580.22	17259.03	32907.92	21612.07	16111.75	30232.43
53299.91	33666.35	34683.79	98216.39	38432.74	19283.47	35105.21	22281.77	14213.97	27037.17
50290	35979.76	30733.2	96661.33	38180.26	15109.43	32280.63	24580.03	15223.51	28792.7
50606.04	29470.51	33412.36	97997.93	33615.95	15406.38	32615.16	20837.57	15169.44	23081.38

LN503.053	LN503.775	LN505.010	LN505.011	LN505.01_	LN505.011	LN505.010	LN505.010	LN505.010	LN505.010
21055.73	12596.66	14956.09	53292.4	78745.35	63801.58	69591.73	58098.26	84824.36	62827.66
18879.14	8584.398	16308.2	47360	141823.7	59135.35	84666.12	63975.97	106331.9	84975.84
23377.89	11314.39	15206.31	51372.87	146328.7	46374.92	80256.14	64191.93	120615.3	90393.81
20866.09	16393.37	23688.59	65176.8	110384.1	50990.04	82408.59	52331.89	84805	55506.49
24762.52	14894.6	22064.9	75677.66	92108.52	60597.01	81948.59	82108.17	109047.3	67561.86

LN505.010	LN505.011	LN505.010	LN505.010	LN505.010	LN505.011	LN505.010	LN505.011	LN505.010	LN505.011
53073.61	29275.49	91455.84	53742.82	107961.3	45591.33	67163.86	80106.87	103537.4	44694.51
54462.36	36123.26	88340.98	76137.29	131030.9	66838.21	76878.27	65736.67	95823.48	33984.21
52063.49	44168.19	63352.45	63789.64	89253.49	74310.69	53886.68	67757.87	67949.77	51599.15
63256.2	24802.07	93497.01	98279.07	163391	58571.38	57597.14	83559.96	80343.44	30681.95
62313.91	29255.92	83513.61	68856.54	114871.9	49611.9	71136.87	83838.56	80151.55	36817.43

LN505.010	LN505.010	LN505.011	LN505.010	LN505.011	LN505.010	LN505.011	LN505.011	LN505.010	LN505.010
85459.01	130367.7	47840.18	99527.29	35028.81	119074.8	74021.54	43995.73	76095.69	48990.81
113058.8	68076.89	45355.88	96023.11	43468.69	79173.02	68913.62	53389.7	65439.52	47736.74
106989.7	88658.14	44497.41	73862.05	38617.95	135260.3	80935.84	61064.39	65782.86	51982.02
81741.88	79826.14	59988.57	111409.5	34150.04	74419.32	77026.44	77428.37	88577.41	60196.41
80675.1	91030.56	42369.2	98242.71	27747.33	123317.6	130128.6	46077.48	73134.75	57523.95

LN505.011	LN505.010	LN505.010	LN505.010	LN505.011	LN505.010	LN505.010	LN505.011	LN505.011	LN505.011
53278.43	86094	72730.92	86557.71	47725.81	100880.2	61375.7	25943.94	61178.83	29475.29
67204.55	81323.27	69242.34	68081.05	65319.9	112839.7	73503.74	36424.6	63363.71	40393.17
50095.99	75861.12	82256.46	80520.87	45007.94	90496.5	70406.89	29431.16	51905.06	51562.23
37742.49	87209.87	75906.87	76142.77	42388.1	127982.4	82240.8	30898.72	62968.66	35547.65
47176.51	132263.3	69029.44	68227.71	34094.22	116450	71119.66	22573.19	73254.6	45943.44

LN505.010	LN505.011	LN505.011	LN505.011	LN505.011	LN505.011	LN505.011	LN505.011	LN505.011	LN505.011
98892.71	23174.51	29253.57	50073.51	67152.29	78291.39	72324.03	30854.31	60545.77	60027.94
78144.73	22062.88	23154.47	50004.22	57730.98	45532.43	64138.44	24715.99	47634.52	43390.65
128468.4	25619.91	28008.25	54808.86	71737.21	65646.5	46475.64	26569.46	73365.02	62140.69
88353.86	19006.9	29232.44	59790.14	77941.56	51698.12	50759.47	26973.5	53334.42	57172.5
86432.02	23504.04	26751.1	49183.7	70707.23	56610.81	70771.4	27398.56	52960.42	43971.77

LN505.011	LN505.010	LN505.010	LN505.011	LN505.011	LN505.010	LN505.010	LN505.010	LN505.010	LN505.011
47121.76	46550.88	104319.6	47483.87	57885.66	89463.6	86285.09	139489.3	68562.56	59176.17
53127.3	63661.83	70041.03	49420.58	44092.61	100171.3	65423.79	143846.8	65620.32	58747.98
45474.16	50611.88	100852.1	58022.07	63476.37	106108.7	88134.24	135507.1	53619.6	57562.68
47649.52	53459.45	78400.65	40089.04	54652.69	76280.98	91796.66	99349.65	72688.77	56704.17
77450.63	48277.96	75845.89	51855.73	47714.2	69283.98	91516.26	182067.6	80814.39	83937.94

LN505.010	LN505.011	LN505.010	LN505.011	LN505.010	LN505.011	LN505.010	LN505.010	LN505.011	LN505.010
85944.88	65584.96	57448.33	33830.52	59037.8	72502.97	106165.6	93715.73	48014.86	69043.51
107503.5	54133.71	68000.36	28815.76	87499.21	68609.32	101043.7	95104.67	64822.8	46181.42
87853.15	51902.44	77093.7	34426.87	52437.01	49623.69	135737	104826.9	72581.88	82724.46
76811.1	71454.3	50860.92	34991.05	57918.09	47712.89	119694.9	77962.02	50040.33	53421.09
99962.04	57585.24	57749.03	29749.74	84622.04	57563.16	124045.8	81368.81	48370.58	54680.08

LN505.011	LN505.011	LN505.010	LN505.010	LN505.011	LN505.010	LN505.010	LN505.010	LN505.011	LN505.010
34976.6	52211.87	84513.82	79035.09	50550.2	95333.43	141575.2	44303.86	28782.55	130811.8
35378.89	49137.87	110224.7	90411.18	49833.86	91574.9	125588.9	60513.23	34000.12	112924.3
32175.38	64293.19	104984	87787.04	41053.61	126985.7	128383.8	49425.08	29339.67	131211.7
26885.21	61683.87	86929.34	108413.3	44907.72	107178.5	158235.3	54077.12	58462.74	140618.6
29899.57	54478.11	81071.96	81736.67	53616.82	114105	122185.8	44326.21	31125.2	100417.4

LN505.010	LN505.010	LN505.011	LN505.011	LN505.010	LN505.010	LN505.011	LN505.010	LN505.011	LN505.189
84767.56	169799.8	63481.53	33692.16	116799.4	71600.43	27639.68	81807.73	66821.46	33053.71
94801.74	144053.9	69636.02	26923.04	82027.27	84034.94	28074.58	86580.76	61908.22	31152.98
76341.1	137299.2	61655.21	27536.19	86098.86	98686.86	35131.5	71035.59	90196.11	33216.71
56623.81	103383.8	56250.72	33692.28	59383.81	61653.16	31478.03	46945.53	103560.9	34295.61
88149.43	162689.2	49462.27	34302.35	65771.98	74436.37	31307.46	66480.48	66959.13	35089.83

LN506.243	LN506.772	LN507.478	LN507.798	LN508.804	LN509.184	LN509.185	LN509.185	LN509.184	LN509.184
9327.305	12371.9	37312.72	15055.3	24713.11	25778.25	21097.5	25819.89	26486.81	34649.8
9311.693	10078.86	39518.86	14403.09	20526.64	26965.99	20368.13	27998.4	24672.39	38755.72
15825.66	12218.75	29883.31	14176.03	37781.54	24132.55	19648.43	24722.33	24909.38	39452.13
19953.39	11782.19	36548.22	13698.51	25732.04	23193.98	20708.71	25779.31	22265.38	37107.64
21270.55	15542.13	36964.69	8881.387	27215.11	21957.19	18405.92	25235.81	22416.51	35602.04

LN509.184	LN509.184	LN509.184	LN509.184	LN509.184	LN509.184	LN509.184	LN509.184	LN509.185	LN509.184
33499.88	22267.29	24461.84	41120.31	27933.35	27826.96	47136.67	24576.83	19516.18	33865.96
37999.77	23967.75	26652.89	42274.41	30224.42	30698.77	45566.12	22738.19	19339.68	35362.22
37426.43	24261.93	30119.03	36288.17	31311.83	29550.65	44592.89	17889.53	15751.4	37262.55
36262	23933.73	27187.29	45320.93	23188.79	29468.31	43875.52	22044.72	18882.47	35793.59
38887.23	20582.06	22087.3	41850.01	28954.33	24700.29	45354.12	21562.12	19528.14	36011.22

LN509.185	LN509.184	LN509.184	LN509.185	LN509.184	LN509.184	LN509.185	LN509.184	LN509.185	LN509.184
17571.76	22384.98	24141.37	30667.61	39718.72	45569.58	22465.18	24938.43	33847.23	39695.2
23329.52	18646.06	25871.08	33730.63	40481.72	45954.77	22930.84	21084.89	28642.11	41485.62
16457.41	24331.93	28556.89	34094.81	37080.33	48759.63	25347.11	28000.99	28959.62	37780.89
19682.49	21199.97	26062.26	30181.23	40816.09	48056.31	24039.36	25775.8	30068.38	42850.79
19461.99	23178.98	26269.22	31519.8	42489.9	45347.1	20611.86	24282.48	32192.45	41006.99

LN509.411	LN509.797	LN510.881	LN510.881	LN510.881	LN510.881	LN510.881	LN510.881	LN510.881	LN510.881
33970.4	15163.93	40579.19	67775.57	47063.4	67675.14	46352.62	48533.2	60963.31	34704.21
34736.15	10793.23	61131.24	109255.7	57155.16	60275.56	68674.3	45906.97	79853.82	28612.7
31057.84	10833.84	69070.9	54087.88	43785.66	65247.16	47569.38	40025.07	56594.95	29719.56
25407.89	13611	45799.9	67225.29	42994.78	62389.8	54298.62	51834.96	68105.11	33630.85
28595.14	15373.06	54767.39	68934.67	45350.81	61393.71	50625.42	46104.32	60001.67	32652.98

LN510.881	LN510.881	LN510.881	LN510.881	LN510.881	LN510.881	LN510.881	LN510.881	LN510.881	LN510.881
43864.27	59784.17	48500.39	36326.98	64152.66	32649.19	63662.63	24817.66	58981.06	33811.54
75115	74763	61705.81	41757.92	64313.48	33162.24	64286.61	20648	45108.68	42162.17
50323.89	100116.1	63725.54	37746.99	87991.92	33293.25	66555.53	24456.74	47461.24	43341.42
50547.58	58062.95	48703.5	36765.88	66190.94	28933.3	57781.8	20807.6	58430.72	32684.8
49163.04	57433.66	47748	31670.66	59015.94	29257.63	60026.26	19642.65	58977.65	28875.64

LN510.881	LN510.881	LN510.881	LN510.881	LN510.881	LN510.881	LN510.881	LN510.881	LN510.881	LN510.881
67865.87	27302.93	59187.49	59452.81	64593.71	57016.9	44455.77	44585.74	33269.7	77473.62
82242.09	43484.3	77122.24	56955.26	79652.31	48935.74	38776.24	47779.91	37063.07	68631.24
92524.23	26379.14	97039.38	77577.51	71252.49	59344.21	56124.21	48841.29	34937.11	74406.92
64180.91	31713.67	53936.71	60066.1	63630.47	59947.56	43078.68	45227.38	35443.39	79892.35
66298.37	31582	49822.54	59840.59	56503.48	49921.64	41777.12	38198.37	34248.35	76421.58

LN510.881	LN510.881	LN510.881	LN510.881	LN510.881	LN510.881	LN510.881	LN510.881	LN510.882	LN510.881
44018.93	54946.75	43687.54	62178.68	73367.13	47473.04	39478.83	32077.59	44595.11	38065.14
50245.72	46314.34	53058.54	67916.08	88445.6	63609.81	53462	37848.94	36300.27	39305.81
41018.01	46223.77	52243.96	57679.43	69866.56	36320.23	42027.37	45313.09	44847.28	40091.2
43924.11	50782.46	39471.52	57620.27	71236.78	43934.11	42507.71	32213.74	39817.92	40969.1
40216.14	50470.59	45713.32	65989.72	65963.57	42627.98	38936.44	28644.8	40528.73	38734.49

LN510.881	LN510.881	LN510.881	LN510.882	LN510.882	LN510.881	LN510.881	LN510.881	LN510.881	LN510.881
42094.54	78005.42	52284.98	62848.93	41672.44	52502.9	63327.09	52165.94	53883.59	23909.91
33784.88	67537.67	53907.73	49688.12	42371.54	61539.6	81654.35	46626.25	54318.85	25684.48
48975.83	72014.02	42922.44	57505.56	48969.56	80136.2	56844.42	59033.9	52514.81	25532.56
43881.91	76854.12	53104.27	64300.28	38770.4	49224.9	64948.08	57458.52	56327.06	23296.62
42504.01	72086.45	54217.8	59253.52	38238.59	52997.74	58194.28	45620.26	57793.04	26895.09

LN510.881	LN510.882	LN511.137	LN511.275	LN511.275	LN511.275	LN511.275	LN511.275	LN511.275	LN511.275
34778.52	33034.97	13984.33	24388.01	22839.03	18080.91	33928.92	24047.77	37172.81	26671.11
35936.45	31769.66	15014.03	22584.56	20707.81	20105.71	35630.61	25576.33	39447.67	32179.71
38264.94	32148.19	16654.99	21474.01	20910.12	18463.91	32523.21	26595.28	41675.56	33366.88
40201.1	31981.16	14614.25	25836.62	24294.79	19493.34	33466.5	27402.75	41705.68	34502.33
33392.74	29577.13	16079.33	21335.67	20001.27	15807.11	34407.75	25862.06	38080.87	27738.91

LN511.275	LN511.275	LN511.275	LN511.275	LN511.275	LN511.275	LN511.275	LN511.792	LN512.166	LN512.417
28750.01	32430	40982.77	21820.18	43762.5	34180.37	28427.87	82617.55	11049.65	16600.36
28985.18	33164.58	38697.47	18491.9	40879.78	30905.43	27479.58	71534.86	9911.36	22289.55
27042.63	32209.06	41556.35	20848.89	47355.49	31731.16	28266.93	86155.99	10020.68	19135.28
33974.04	35003.92	42876.8	23822.85	44334.39	32795.9	32095.03	76311.52	13099.66	19913.42
27837.17	30817.72	39975.62	17642.96	40082.9	30194.23	28754.03	131527.7	16459.77	16296.11

LN512.797	LN513.144	LN513.144	LN513.144	LN513.144	LN514.148	LN514.822	LN514.994	LN515.013	LN515.033
19556.17	25500.86	19335.9	29399.53	38230.48	21850.11	19301.04	49067.17	47183.55	81343.5
17872.75	27589.98	17813.89	31884.54	35171.21	20351.96	13314.36	48296.67	46946.34	82249.47
15618.27	27460.34	17993.66	33818.86	40412.13	18503.36	19576.64	50217.91	50854.92	82190.43
23236.42	28278.81	19937.81	34484.78	37314.95	21191.67	16705.2	54021.14	55420.96	77747.61
22127.89	28875.18	20044.16	30797.02	35175.21	22802.86	19573.17	52486.99	48182.83	77829.3

LN515.033	LN515.033	LN515.033	LN515.033	LN515.033	LN515.050	LN515.051	LN515.051	LN515.051	LN515.050
42981.69	52370.62	71343.7	41496.3	39524.43	47021.98	45215.05	22839.52	58518.36	45519.96
47824.16	48546.06	68231.22	36907.28	38351.14	47234.64	47799.85	19083.08	55820.43	46728.84
38628.31	39430.11	67799.23	35566.45	39048.27	43750.18	51588.85	21150.33	60788.88	48795.32
39568.77	50396.77	68634.23	34467.04	37856.68	45464.54	47670.69	20383.86	56995.82	47510.92
41904.87	46692.96	64507.47	34753.04	34887.43	40709.59	52314.66	21047.79	60575.2	47617.45

LN515.051	LN515.051	LN516.429	LN517.03_	LN517.030	LN517.029	LN517.029	LN517.153	LN517.779	LN517.831
28170.22	35939.62	87201.25	40255.01	51982.26	40278.65	68334.13	19269.94	24659.78	23064.56
25561.05	36723.71	89792.74	45211.61	51641.7	42534.74	69850.95	20602.72	23979.92	17354.41
24451.16	34755.99	81269.84	40018.14	47671.7	42912.15	67486.22	23339.31	21072.71	20656.12
25413.7	38785.34	78437.66	46794.44	50184.35	42464.64	78086.03	17392.47	23922.33	23443.13
22115.42	34207.98	78218.72	47087.27	48739.57	45598.62	68684.29	18127.83	31524.27	23189.36

LN518.827	LN519.025	LN519.025	LN519.025	LN519.025	LN519.026	LN519.025	LN519.026	LN519.025	LN519.025
18944.92	72130.11	32327.97	49484.26	20660.86	26149.72	23406.09	18936.39	26734.92	34145.85
19789.62	73440.93	33658.71	45558.88	20867.54	26531.96	24621.86	19058.19	26930.82	35045.47
18242.31	74471.02	35362.74	48189.49	16550.04	25720.55	22808.93	19184.14	27553.97	33594.77
17355.74	73668.9	31491.33	46587.1	19437.61	24677.41	23934.65	17161.72	26962.67	37089.25
21570.69	77175.17	44869.67	45637.57	18228.93	22385.38	23918.36	17372.54	26261.24	32781.99

LN519.026	LN519.025	LN519.026	LN519.026	LN519.026	LN519.025	LN519.025	LN519.026	LN519.025	LN519.025
22090.4	34173.3	25818.46	18555.69	24193	39203.95	40400.18	20690.75	52731.11	45553.11
22511.34	33861.15	28003.29	18721.89	26346.27	33457.25	40389.39	19472.62	52383.47	45657.45
21797.1	29500	28012.46	18005.95	26094.34	33014.72	35559.87	20984.72	50889.52	48404.92
22712.25	35221.4	29291.71	16310.62	24133.9	34437.26	44158.7	19996.06	54637.49	41606.54
21433.95	39143.73	24909.89	17030.78	26000.77	38896.99	39005.3	21031.28	51534.16	44957.21

LN519.025	LN519.025	LN519.025	LN519.025	LN519.025	LN519.025	LN519.026	LN519.025	LN519.025	LN519.025
34862.01	65345.75	23324.55	51352.67	49385.09	41957.15	33063.36	43107.99	35397.07	68382.76
32526.1	64889.46	18797.04	47373.04	49695.09	38698.91	29094.66	46288.96	33777.95	69515.6
34122.49	65650.59	23506.95	45494.81	47419.21	33887.78	31511.99	50242.99	31847.48	62472.6
33661.38	64837.28	19742.87	48000.52	46725.71	34395.52	29639.61	40961.39	36451.21	61740.38
32187.57	66123.51	18709.7	49454.12	50797.62	38933.84	32056.23	48096.55	33945.4	62562.68

LN519.025	LN519.025	LN519.025	LN519.026	LN519.025	LN519.025	LN519.026	LN519.025	LN519.026	LN519.026
53397.71	42074.49	43965.36	31755.88	35363.88	48971.2	23884.99	70250.77	19454.78	24069.03
52301.74	41254.91	44471.69	31203.03	32120.58	45477.72	23726.18	73291.57	20780.52	23090.84
53867.5	37472.96	45803.69	30470.34	34993.43	41104.59	26001.12	76878.13	19670.97	22090.17
52302.43	40563.89	42879.08	32206.37	30128.87	45710.74	24006.98	64380.94	18955.36	25446.86
50384.93	39074.24	45965.68	31128.04	34648.82	43736.5	23345.83	74840.79	20755.71	21839.49

LN519.025	LN519.025	LN519.026	LN519.026	LN519.026	LN519.026	LN519.026	LN519.026	LN519.026	LN519.026
61228.04	45320.26	34672.54	13034.78	16830.94	14152.34	22345.68	24584.19	15443.48	29383.25
66504.61	48206.42	31999.24	14742.87	17503.81	14219.38	22996.12	24313.14	16219.68	29834.84
63692.73	52115.14	31425.15	13446.74	19018.97	14240.87	22472.62	24580.45	15283.88	28030.01
66719.56	47119.81	33457.06	15095.26	18698.89	13411.32	22662.14	24434.24	15956.41	29021.01
55696.9	52216.99	33066.23	14113.52	18039.5	13234.81	24392.08	24091.4	14720.45	32823.81

LN519.025	LN519.026	LN519.025	LN519.025	LN519.025	LN519.025	LN519.025	LN519.025	LN519.025	LN519.025
39519.7	45175.6	37256.06	35573.02	57358.9	49762.77	32626.17	31014.95	45871.41	52161.6
42848.72	45689.75	33790.76	35614	61822.47	46584.05	32069.58	37618.61	43504.73	54050.86
44106.97	48111.42	35903.55	36625.09	60712	50318.55	32638.19	33122.01	46116.32	52854.69
44928.25	42958.01	32265.1	35628.1	61634.77	52791.56	29617.08	31909.12	44013.28	61192.76
39319.9	43957.14	36069	33661.88	60813.42	49235.73	33625.05	33243.19	39764.18	50344.39

LN519.025	LN519.024	LN519.025	LN519.025	LN519.025	LN519.026	LN519.026	LN519.026	LN519.025	LN519.025
63418.25	44032.33	44856.61	26022.07	34617.73	29060.97	40736.23	19376.55	42702.92	60697.4
65722.69	44967.12	40998.14	28932.78	32508.36	28605.28	35026.17	18111.01	41994.39	54584.07
64488.68	46916.3	42761.95	27178.15	31036.03	26909.86	37046.8	17936.54	41464.94	59709
60272.99	48770.65	44617.77	28268.49	32088.14	28845.3	37302.54	18437.42	44530.22	58938.74
60615.59	42631.46	42599.98	26431.31	32478.73	26144.63	39836.97	17211.55	36502.69	61968.93

LN519.026	LN519.026	LN519.025	LN519.025	LN519.025	LN519.026	LN519.025	LN519.025	LN519.026	LN519.025
32678.43	33062.04	36277.58	39553.63	17997.77	34173.38	82786.18	53516.88	23149.29	69678.91
28967.92	30194.99	44072.64	36102.23	15970.42	35335.09	93077.22	52449.35	23463.07	66592.89
31825.9	34786.02	40054.74	39021.27	17785.76	32049.28	85017.49	52801.17	23830.04	64435.52
30180.83	35614.35	39291.06	41348.19	15787.61	37157.79	85232.15	56597.39	23382.08	71130.08
29612.38	35031.71	38057.17	37427.23	17852.04	34744.45	80192.43	53046.25	24980.37	61420.07

LN519.025	LN519.025	LN519.026	LN519.252	LN519.778	LN520.385	LN521.165	LN521.773	LN522.195	LN522.372
63762.7	50479.35	20718.54	20031.04	17416.86	10313.15	61205.89	45529.3	16520.15	61225.73
66256.91	47644.44	19100.61	26326.47	17731.05	5798.957	57545.63	44111.7	12255.25	68853.54
63734.14	51389.31	20353.43	26140.06	17350.2	5675.859	70201.27	61019.51	13704.89	67553.3
67299.12	47271.81	22131.99	21297.3	19090.72	6796.263	60149.66	76638.03	14245.74	62195.13
64888.81	45257.22	19922.83	26455.94	21996.8	4919.701	62505.76	56174.33	15062.8	14747.95

LN522.445	LN522.780	LN523.001	LN523.000	LN523.001	LN523.001	LN523.001	LN523.001	LN523.001	LN523.001
85059.38	14637.39	24209.88	96905.56	81394.19	65589.97	38453.25	56538.44	47056.42	51408.02
89661.74	15866.37	17511.66	62116.23	59340.34	69646.88	64379.99	88672.68	52872.86	58000.56
86820.83	17841.97	20230.84	74551.79	62072.33	53724.87	61734.87	76463.25	53629.29	57497.2
77744.76	22067.02	23881.44	72466.21	52258.68	60110.39	51405.01	67888.42	44197.06	45412.45
74735.31	17601.02	19105.79	82552.12	56540.05	54705.14	56139.36	73538.4	49864.32	61242.14

LN523.001	LN523.001	LN523.001	LN523.001	LN523.001	LN523.001	LN523.001	LN523.001	LN523.001	LN523.001
50497.64	42004.12	54594.95	100204.5	44528.18	60896.6	46333.44	70726.79	78117.24	53781.03
92410.07	48583.96	64086.54	58215.06	43930.22	30198.39	82915.66	88968.61	53190.09	56064.53
58775.13	33922.92	51484.1	63764.84	45620.32	34889.42	54034.82	76435.73	62840.77	54295.87
55607.27	43172.69	73525.48	79743.48	63711.84	36818.53	57909.7	73618.44	52938.78	88352.44
67271.5	36732.14	48825.17	60545.45	45627.4	42650.14	58443.38	65779.02	61403.26	54091.3

LN523.001	LN523.001	LN523.001	LN523.001	LN523.001	LN523.001	LN523.001	LN523.001	LN523.001	LN523.001
38587.8	50292.54	94296.4	40865.08	126419.6	57318.43	73415.18	23735.03	48070.04	110458.5
40521.01	54519.81	85907.33	38762.25	70500.49	32536.37	71715.33	25867.39	36231.07	82308.24
39092.14	61595.71	78449.31	35925.77	61967.54	44665.01	74045.69	26796.26	41209.44	89221.57
40219.14	53784.5	113979.7	32404.31	72865.46	47777.5	110750	32295.12	49913.95	73268.97
41531.07	58718.56	75513.26	40038.4	64112.71	43107.28	67963.11	30731.6	41359.71	83438.3

LN523.001	LN523.001	LN523.001	LN523.001	LN523.001	LN523.001	LN523.001	LN523.001	LN523.001	LN523.001
103021.2	59531.41	34654.67	53604.46	37310.17	60579.68	19465.29	90687.98	57741.03	64767.8
94727.43	40117.11	39836.21	67837.98	39049.96	44039.07	26254.08	96866.11	49411.96	69399.22
88949.57	40069.88	38069.51	76863.05	45183.82	43118.3	25980.38	76062.2	43844.8	75889.56
104173.1	60073.45	43485.26	77474.66	36761.98	49501.04	17422.22	67238.68	46686.95	86461.29
83825.42	44053.22	41460.41	71489.38	50380.99	50661.52	21453.68	82040.93	42530.26	78728.65

LN523.001	LN523.001	LN523.001	LN523.001	LN523.001	LN523.001	LN523.001	LN523.001	LN523.001	LN523.001
42268.62	61323.6	65978.81	51981.96	60330.18	59909.79	28319.13	56488.73	53258.74	63652.5
48837.42	67922.71	72162.81	76431.28	77641.27	49354.49	24965.35	101143.9	43358.22	99583.05
31833.94	53549.05	70003.19	51014.01	52334.9	53014.44	25253.4	60070.93	57063.55	76851.02
39316.47	50888.64	59990.82	40386.6	74699.79	32353.75	33552.91	61820.93	50001.36	80675.48
38099.5	57583.15	56414.88	64344.78	57442.22	49423.91	26638.69	56529.73	59952.58	77912.26

LN523.001	LN523.001	LN523.001	LN523.001	LN523.001	LN523.001	LN523.001	LN523.001	LN523.001	LN523.001
118400.5	45607.91	30504.28	38805.04	38226.13	40124.4	98671.67	40613.87	32800.86	63085.57
98361.38	33606.47	18091.02	61734.16	27793.59	49717.13	150627.3	27870.21	32172.3	80355.47
87561.07	46888.33	26686.59	41744.93	42452.79	52715.1	103074	28813.34	29940.02	59580.12
132915.1	41906.58	24928.55	41574.93	38568.4	60958.37	103420.2	37139.35	39652.81	85080.86
69815.75	39329.55	26195.51	45620.83	37375.52	51257.53	106783.6	29199.74	31234.72	59241.51

LN523.001	LN523.001	LN523.001	LN523.001	LN523.000	LN523.001	LN523.001	LN523.001	LN523.001	LN523.001
45627.12	44540.57	25651.87	47669.3	100065.4	32623.27	71516.47	102844.6	51613.5	75352.89
46487.07	47747.22	23840.44	30977.32	97289.59	30430.42	73638.83	106639.6	48612.26	112249.5
44962.13	38576.27	23010.57	34696.09	101244.2	35726.02	75158.07	95558.12	49947.12	87553.13
50281.47	40110.75	23514.07	38976.29	90027.4	35067.64	72460.41	101367.4	50670.83	93611.1
37564.47	43872.92	20811.97	34198.03	110740.3	35162	77878.52	91397.03	45914.52	89787.18

LN523.001	LN523.001	LN523.001	LN523.001	LN523.001	LN523.001	LN523.001	LN523.001	LN523.001	LN523.001
55869.41	131555.8	74345.85	84402.88	57630.77	31574.64	64833.46	22330.96	69925.17	46191.11
59165.03	103385.1	68443.15	110076	73832.6	31900.23	41530.41	22198.81	67190	39314.06
75185.11	89690.09	60945.13	95474.86	70584.61	27023.86	53257.44	23704.63	71954.95	42269.3
80674.94	79957.66	65498.23	85904.5	84033.51	38259.11	65828.32	21061.06	65650.98	35512.19
69206.45	95927.1	67440.09	88181.23	72434.25	28640.25	50287.13	19420.13	69417.07	43869.16

LN523.001	LN523.001	LN523.001	LN523.001	LN523.001	LN523.001	LN523.001	LN523.019	LN523.019	LN523.02_
41471.44	37632.51	73062.05	42875.65	34541.19	23919.67	25322.5	66567.63	60004.12	69506.54
42394.88	39893.7	46796.01	50912.14	39344.28	24826.04	27107.34	75813.44	62372.94	76146.75
42503.21	42678.05	50431.52	55258.49	45708.07	23761.15	26472.37	79601.28	59976.53	70806.29
34661.93	39265.04	77706.93	52763.87	38271.13	25755.17	26945.81	77538.14	68781.7	76502.89
43213.55	46371.26	52180.7	54464.49	44564.22	24069.87	25759.23	80864.86	71226.59	73677.98

LN523.020	LN523.040	LN523.040	LN523.275	LN523.776	LN524.776	LN524.848	LN524.849	LN524.849	LN524.848
64451.87	79730.1	82461.76	27786.23	26744.04	96630.92	25945.89	30256.94	23942.21	45219.68
62581.85	78436.47	78803.59	29102.59	22518.47	77270.93	29131.16	27108.98	27931.92	54824.22
71261.85	81171.79	79133.81	28259.84	23253.6	82993.99	24345.94	27976.55	22199.99	54539.7
66351.58	79972.57	78264.92	29519.43	19378.48	106416.8	21308.88	25447.22	20860.94	57159.03
69996.43	79600.19	80720.55	29491.07	22818.13	121519.3	22269.28	24931.01	21748.44	54318.62

LN524.848	LN524.849	LN524.849	LN524.849	LN524.849	LN524.849	LN525.036	LN525.036	LN525.162	LN525.162
22912.67	23425.51	22122.25	22277.88	20203.06	19991.44	34865.82	28285.23	28690.07	18006
27551.75	24642.38	16728.69	19268.76	19161.94	19668.62	20717	20097.81	27800.44	17030.7
21172.03	21377.42	19414.92	21306.25	14916.81	21397.87	36334.4	26634.88	26380.35	20906.68
23192.47	18728.87	20428.41	19925.62	18886.69	18982.74	37968.77	30449	26776.62	18637.49
23668.65	16734.17	16688.29	21820.44	17455.76	18562.8	35934.18	28175.03	23467.43	17499.63

LN525.162	LN525.162	LN525.162	LN525.777	LN526.776	LN526.976	LN526.975	LN526.975	LN526.976	LN526.976
31340.81	28166.95	23128.09	14339.24	33607.9	42232.73	52244.23	45227.03	32084.18	51852.31
35918.67	26989.56	22549.46	13895.75	38194.68	40911.63	50726.29	46131.08	29911.58	48994.12
37021.43	31604.1	22043.84	13283.2	40014.28	36950.36	50736.08	46961.18	29760.17	53469.36
34672.83	25777.39	23044.94	13470.57	54081	43649.03	51008.27	44204.84	28129.16	55349.17
33749.77	26892.54	21611.1	17649.79	59792.42	40300.37	47475.95	44676.67	29888.95	48300.72

LN526.976	LN527.033	LN527.051	LN527.052	LN527.052	LN527.182	LN528.774	LN529.156	LN529.157	LN529.156
36841.07	35838.85	15788.98	20174.46	20053.24	66674.26	18321.71	28371.29	29754.38	22688.11
43307.75	33497.95	15741.32	22562.55	19627.23	67818.73	14403.43	27570.59	26512.19	22871.14
41401.63	36063.71	14832.84	22384.45	16870.8	75344.64	18558.22	28035.49	28446.92	23334.53
40110.35	36067.36	16217.72	21566.49	17556.05	71531.19	17424.5	33839.86	26621.61	23704.65
41421.46	36230.64	14351.68	20816.58	22958.51	60438.45	21143.95	33641.92	25009.53	23186.89

LN529.157	LN529.156	LN529.280	LN531.287	LN532.401	LN532.789	LN532.808	LN533.004	LN533.090	LN533.09_
26532.58	28476.38	276607.8	15313.68	64645.77	17707.98	15214.14	23267.99	22475.42	29773.89
32762.68	41397.67	272238.3	17109.04	69248.78	18836.19	13514.19	23023.05	21973.17	31516.67
31153.48	30680.39	304147	21039.13	70198.94	15341.77	10971.9	23356.58	20513.54	24841.01
26825.08	29237.91	285633.9	19604.2	64644.68	17649.81	15801.94	22583.03	19134.01	26173.25
29150.71	28380.48	297871.3	15108.53	55136.07	19757.49	16062.14	24097.34	19919.43	26488.31

LN533.090	LN533.143	LN533.219	LN533.455	LN533.773	LN534.402	LN534.782	LN534.803	LN535.002	LN535.783
35355.61	8609.857	32463.01	22748.66	34206.91	22770.46	107149.9	136901.3	47524.51	13790.59
26339.32	9927.898	31838.81	27083.44	25202.17	21319.26	90111.47	119147.8	47538.44	18058.18
34289.99	9174.184	31964.08	23949.5	21385.57	19891.38	148916.7	112794.8	47534.9	13965.49
33574	10724.98	27986.09	17491.39	35853.76	18416.54	169215.5	116896	48776.45	15228.55
29947.82	13893.05	30803.98	22017.43	43186.87	16021.1	126105.7	176084	49143.86	20548.82

LN535.806	LN536.784	LN536.804	LN537.016	LN537.017	LN537.016	LN537.017	LN537.016	LN537.017	LN537.016
17882.73	34066.41	28546.74	48151.79	49424.14	59954.87	16435.75	68072.41	37757.22	106971
15818.01	36156.62	43273.21	48430.52	52196.41	63408.73	14408.63	79195.32	46750.98	74106.36
15833.6	33135.96	32620.26	54277.37	57013.71	72201.43	12625.5	80716.88	47851.21	72046.1
18425.53	32255.61	26746.48	50824.14	54598.88	65536.4	13491.41	74522.34	49975.71	66932.67
16953.76	36019.53	40712.78	42864.18	42280.35	94237.16	20376.99	72369.76	51918.07	91093.17

LN537.016	LN537.016	LN537.017	LN537.016	LN537.017	LN537.017	LN537.016	LN537.016	LN537.016	LN537.017
102032.2	56861.11	70005.14	65220.2	64037.86	32352.06	74463.73	81112.74	103211.2	74069.98
97578.16	63601.9	66364.97	85395.51	63222.43	41309.16	71376.7	65684.05	106248.2	65543.82
97484.77	59697.81	60086.97	77957.37	67551.24	40214.92	59883.56	69027.51	108380.4	58828.68
97715.23	58233.25	61640.29	81557.36	60849.09	39919.78	59451.19	68422.62	109940.5	65101.35
89954.31	60964.91	64332.07	95241.11	57815.67	33734.03	70050.74	85932.26	120799.3	55331.29

LN537.017	LN537.016	LN537.016	LN537.016	LN537.016	LN537.017	LN537.016	LN537.017	LN537.017	LN537.017
32421.17	56317.88	94762.36	65000.53	65028.48	33132.4	108685.3	67797.18	45060.67	48718.58
44400.47	74820.22	93569.11	66960.02	70776.08	38193.05	68837.84	66421.18	33047.81	48323.76
44501.6	81330.36	83489.83	63478.62	74321.74	38996.06	65482.84	64532.54	33123.61	45745.21
44334.13	77806.59	89841.21	66970.91	69919.92	41341.99	59715.7	54533.55	32184.15	39624.01
42130.29	55069.82	103414.8	66929.95	68633.12	43953.96	61696.34	60072.75	35766.3	35254.99

LN537.016	LN537.017	LN537.016	LN537.017	LN537.017	LN537.016	LN537.017	LN537.017	LN537.017	LN537.017
75513.4	32560.96	96759.98	69841.06	57963.03	72985.04	49895.77	67224.82	43592.64	73603.99
70627.41	35286.29	92917	65176.97	34043.05	69775.23	51211.39	60993.95	40780.8	52614.83
75222.05	35973.31	93499.23	64067.44	34111.44	76059.5	46937.58	62597.13	39115.04	55443.82
68320.21	37835.9	86950.98	57124.18	33621.71	73602.13	49830.04	59576.82	45521.78	49304.19
85381.48	42396.61	82582.18	90835.57	32079.38	94363.18	52600.47	76878.5	47444.78	53089.66

LN537.017	LN537.017	LN537.017	LN537.017	LN537.017	LN537.017	LN537.016	LN537.017	LN537.017	LN537.017
40532.81	58916.73	45741.3	31941.21	27135.29	44189.84	60964.6	38254.13	48054.31	22548.7
50659.15	50527.19	50508.93	46426.24	30229.66	39836.68	46425.99	28635.38	40558.12	23462.03
52856.24	60389.92	57041.7	45930.38	30847.25	42960.08	54099.81	28403.08	44529.81	23028.08
50388.27	53895.75	56670.48	49568.85	30311.59	41316.36	53386.68	29576.91	40755.97	23989.01
55630.65	70393.48	57544	65785.9	34399.07	32042.39	51359	35289	40304.28	33951.05

LN537.017	LN537.017	LN537.017	LN537.017	LN537.017	LN537.017	LN537.017	LN537.017	LN537.017	LN537.017
29383.93	42370.03	30073.99	39605.54	34978.28	47030.72	31008.37	28912.15	38371.35	30711.3
42807.04	38966.54	25344.51	42116.49	38812.31	39463.45	33143.42	28731.85	22535.07	27744.49
43497.06	34101.57	27180.34	46874.07	38713.18	38183.62	30292.76	32649.5	22546.63	23740.46
41635.49	37245.11	29034.43	44021.27	39254.43	40498.85	30083.22	36863.36	20639.58	27119.83
43167.13	50291.33	42215.71	29326.83	33327.42	55064.18	44299.2	38569.02	23413.9	20902.18

LN537.016	LN537.017	LN537.017	LN537.017	LN537.017	LN537.017	LN537.017	LN537.017	LN537.017	LN537.017
72377.29	29689.96	23060.56	66756.44	32093.46	42111.44	44849.87	25617.9	55382.11	31882.29
52402.68	24045.66	18917.81	48984.92	28975.79	26618.01	41659.45	25141.2	48246.09	34527.29
50413.74	27273.61	25683.7	45412.41	25920.08	28578.92	40800.24	28424.1	48157.67	37102.91
49589.44	24501.27	21909.47	49616.54	26369.12	30752.55	38676.99	22112.21	56251.77	37437.29
64692.42	21414.59	13272.5	41556.83	34571.19	37678.35	37546.31	27741.25	40130.09	43309.62

LN537.017	LN537.017	LN537.016	LN537.017	LN537.016	LN537.017	LN537.017	LN537.017	LN537.017	LN537.017
21595.03	42042.83	84802.98	19657.63	53759.53	47218.58	41748.72	28497.55	25058.62	48084.66
19579.11	45126.24	74188.14	24491.35	52888.05	29614.08	34819.37	35781.91	20589.84	35568.33
17977.81	48251.94	74870.95	24384.7	50149.92	30816.53	31813.16	34540.03	18537.29	43251
14744.9	46951.91	75472.35	23706.79	51648.55	32229.88	35248.62	35229.05	16176.34	39770.09
22616.2	53436.28	71447.73	23753.02	59740.23	31231.84	32643.62	35348.33	24694.88	46450.92

LN537.017	LN537.017	LN537.017	LN537.017	LN537.017	LN537.018	LN537.018	LN537.018	LN537.018	LN537.018
88288.41	37492.72	47881.5	18112.51	52150.99	29466.79	34560.84	42057.89	34314.89	61625.99
67656.26	37727.8	45782.6	18483.31	42373.38	28141.73	35650.12	41407.29	33496.22	58483.53
64936.68	38474.64	48599.73	18542.26	42972.72	28984.77	37050.88	45192.65	35178.75	59314.09
62387.92	42031.15	44680.8	16773.35	43156.21	26843.08	36034.48	39002.47	34132.83	67263.61
51262.45	30752.54	71036.21	16439.38	67188.8	27416.42	38116.55	42966.73	34540.4	54483.58

LN537.018	LN537.137	LN537.216	LN537.216	LN537.216	LN537.216	LN537.254	LN537.254	LN537.255	LN537.254
40685.3	52118.21	26388.89	23301.3	25509.39	27285.32	21563.44	18161.52	20728.47	22384.69
42369	49359.79	28421.4	22012.28	27770.18	27101.18	21474.33	21943.63	21003.77	22228.27
46512.16	56394.69	25342.11	21464.78	26794.46	28316.91	20115.56	20586.8	19306.59	22182.68
41836.26	45331.39	22074.49	21124.58	28999.02	28105.92	24584.48	18238.33	22341.73	18951.08
38408.07	42886.21	23856.42	19906.47	27048.15	24857.13	20082.74	18900.4	20547.56	23224.95

LN537.254	LN537.255	LN537.254	LN537.255	LN537.254	LN537.254	LN537.255	LN537.255	LN538.780	LN538.800
21508.71	17214.21	33147.44	21019.53	28313.42	22670.05	32385.11	31928.17	16829.35	15055.65
20505.98	18231.33	32839.85	21451.22	27601.81	23432.63	31643.24	31869.29	16886.47	14936.44
21423.15	16804.3	34829.54	22519.35	31802.47	24994.84	35581.1	31331.66	16234.14	15563.18
21535.82	20027.27	34870.87	21498.69	30318.56	24534.01	32340.67	31814.06	15839.54	16531.97
18782.57	18189.27	32520.22	23210.75	25897.73	24305.48	32126.59	32807.57	17584.9	14705.97

LN539.033	LN539.033	LN539.033	LN539.034	LN539.033	LN539.033	LN539.139	LN539.168	LN539.177	LN539.178
26291.11	24577.23	39084.73	41183	27281.28	24670.16	20839.12	23987.71	31176.3	29430.44
36210.24	24212.67	47427.28	52168.75	26331.76	34300.82	18315.26	24947.73	30681.55	27668.17
34869.91	26829.53	48324.82	50077.5	29064.37	30260.52	22808.03	16607.48	31129.38	28938.95
33212.56	26065.31	46525.15	51928.8	32877.26	28030.18	18449.06	29531.23	28439.37	24499.42
29561	27605.35	46200.23	57864.68	30709.44	29227.46	23025.84	25507.01	24922.04	26854.19

LN539.178	LN539.178	LN539.178	LN539.178	LN539.178	LN539.786	LN539.787	LN539.787	LN539.787	LN540.747
33815.56	27107.3	23064.11	22524.97	27314.2	30955.86	27889.89	24714.87	43626.98	30950.06
32226.47	25864.34	24050.75	24956.62	27748.93	29848.08	32191.97	22915.7	49763.61	25441.19
30206.57	25832.48	22328.41	24574.74	22636.98	34409.03	27083.69	25486.15	43714	29499.86
31614.97	23419.7	21198.99	19995.46	29072.83	29257.71	27052.88	24131.88	48775.11	31798.32
31652.59	25424.11	21055.28	23591.08	26090.48	24997.44	27166.9	18141.36	41040.86	38461.7

LN540.820	LN541.030	LN541.029	LN541.030	LN541.030	LN541.030	LN542.179	LN542.747	LN542.821	LN542.988
47595.63	59765.24	58191.31	65767.89	59835.28	116230	29655.21	16642.16	24755.54	40593.26
49803.49	59877.5	56179.62	64056.56	61530.59	108027.1	31493.98	20600.49	29067.46	45253.84
50336.75	49497.28	62375.37	55799.4	57877.08	115463.3	34721.31	22078.05	24908.66	43892.12
57542.35	54057.65	52516.27	61301.97	56592.1	113025.6	24346.06	20790.18	25183.6	41727.19
39904.18	44453.46	56168.13	51959.95	56514.02	101714	25714.58	20765.2	25808.18	43635.59

LN542.988	LN542.988	LN542.988	LN543.046	LN543.181	LN543.466	LN543.781	LN544.745	LN544.764	LN545.276
39960.53	44109.86	31803.92	38946.72	12882.15	13042.72	15786.81	14679.68	9287.687	67682.94
38474.3	43748.06	32121.23	42374.63	14049.16	13824.03	18589.79	9595.939	9347.663	80790.73
40306	44761.74	31537.37	37477.26	13990.92	17791.41	13922.88	8746.366	7629.822	93720.94
41003.53	44789.9	31018.87	36530.23	12654.91	12251.55	17091.71	8777.748	9560.24	73407.47
38303.98	41973.28	31422.38	33132.52	13941.31	14929.51	16302.95	11841.83	14386.9	66661.56

LN547.057	LN547.057	LN547.057	LN547.058	LN548.780	LN549.102	LN549.102	LN549.102	LN549.102	LN549.102
54725.18	33223.76	41400.5	37957.27	40691.62	36216.16	23921.37	26618.49	28712.85	30039.3
52963.96	31616.25	43420.74	35962.31	41499.64	35090.03	26193.24	30470.54	26420.7	34044.02
49295.75	30971.43	44758.81	33893.23	50111.43	29610.29	24403.8	25534.24	26075.33	30012.61
48923.5	32390.82	41349.42	33535.12	58059.3	30633.23	27207.17	26923.64	26918.51	31040.94
55544.56	33040.69	46331.09	36631.45	69511.36	31077.69	23470.39	24778.14	26544.41	28512.11

LN549.102	LN549.103	LN549.103	LN549.103	LN549.197	LN549.254	LN549.254	LN549.525	LN550.200	LN550.529
28888.01	23884.52	29163.69	24079.16	105153.7	22069.95	22237.55	57075.73	17092.12	13031.71
32343.68	27033.07	26726.19	25031.8	124523.2	27095.38	21546.26	46957.43	16801.65	13379.22
29775.22	22332.39	28833.41	27534.99	107363.5	21516.78	21900.24	65593.81	17265.88	14483.41
30959.86	24087.71	24365.9	19865.9	109168.8	23871.82	20449.09	60777.98	15815.86	13028.96
30402.88	25457.42	28822.17	24862.8	80421.22	20831.61	19925.57	59267.96	18697.91	15980.82

LN550.776	LN551.032	LN551.231	LN551.231	LN551.231	LN551.231	LN551.231	LN551.270	LN551.270	LN551.270
277529.1	54424.69	36869.6	28946.62	37035.34	21263.18	26354.58	20974.11	16106	21624.86
262933.1	53575.95	34724.44	26851.87	36721.74	20659.88	23571.27	21917.44	12487.27	21644.3
295737.2	56231.37	37633.72	24033.61	34416.71	20340.76	27936.04	19282.57	13456.89	20866.99
339089.6	54862.57	35029	23862.19	37088.17	21191.67	25858.25	21249.24	13458.48	23863.42
415566.4	56075.7	35718.76	27781.33	36499.89	23037.06	23319.94	23257.36	13156.56	23911.07

LN551.270	LN551.270	LN551.778	LN552.723	LN552.776	LN553.029	LN553.029	LN553.049	LN553.049	LN553.049
22773.7	22505.56	47070.12	18323.54	150473.7	33833.98	35485.75	43553	38442.06	37822.82
24720.24	22768.71	47809.3	15316.34	128545.8	33062.31	43848.85	46038.16	38009.15	42147.15
22670.73	21013.64	52053.92	19613.24	150498.3	32637.41	37009.69	40385.97	38258.9	40713
25700.27	21704.01	50465.48	25313.07	169274.8	34460.8	39453.08	42104.27	37438.86	35179.16
22448.23	24746.85	62245.53	31105.12	200192.4	34205.75	37014.83	43943.95	23527.2	39456.75

LN553.048	LN553.049	LN553.049	LN553.050	LN553.194	LN553.194	LN553.194	LN553.195	LN553.195	LN553.495
36684.83	48182.88	33405.43	35931.47	19542.58	23348.19	26916.03	23864.45	27221.72	52532.23
32083.83	51785.14	36649.89	31205.81	24415.36	19434.92	28254.67	23819.27	25278.79	54128.54
36114.63	48214.54	34119.88	34684.85	23254.46	19919.41	26519.67	20002.32	22866.41	43216.73
38566.08	43379.75	33275.35	33758.63	26031.58	22093.7	27710.8	26224.9	23260.58	24828.87
21557.88	47184.42	33239.85	30064.77	23973.42	23727.17	27454.92	26002.59	28183.67	30671.1

LN553.778	LN554.498	LN554.718	LN554.752	LN554.773	LN554.989	LN555.006	LN555.008	LN555.008	LN555.008
23819.26	17587.15	91490.07	56600.94	63517.36	67151.07	71806.6	46130.38	36076.75	24572.94
22900.55	17454.95	86091.78	67419.55	68485.54	65503.06	124748.1	54091.64	47264.02	19944.51
22653.47	15510.75	82729.29	66079.32	56850.08	64463.02	69342.99	54196.15	30898.68	22132.54
26052.65	13494.38	112022.9	62855.07	54599.98	60336.18	67553.03	44326.33	42723.37	27649.57
31357	14022.27	144191.9	62165.19	80585.11	60136.9	69371.54	42249.97	38273.59	24850.73

LN555.008	LN555.007	LN555.008	LN555.008	LN555.008	LN555.007	LN555.008	LN555.008	LN555.008	LN555.008
34248.73	58494.01	53057.79	36058.44	39342.39	63332.83	43541.23	23748.14	35243.09	38412.72
29910.26	61773.62	61170.58	53160.93	49583.84	58836.46	48972.35	22306.17	43655.97	39310.39
45109.92	72000.1	59608.6	29056.28	37842.4	48793.39	41724.3	20573.92	40659.15	46065.74
37836.73	56504.4	60232.97	33671.2	34897.56	69912.87	42051.48	21647.51	35269.08	41727.66
35194.68	54107.66	54406.79	34464.13	35441.59	68376.43	39789.52	24897.67	31770.46	37456.54

LN555.008	LN555.008	LN555.008	LN555.008	LN555.007	LN555.007	LN555.008	LN555.008	LN555.008	LN555.008
36279.03	63515.08	85528.71	57661.17	61379.94	51741.53	58491.66	53460.92	33159.59	57377.52
34393.45	82529.71	69195.26	82193.02	64986.36	60594.15	66265.76	46378.58	39465.9	56861.73
52738.67	67198.02	70386.13	57096.91	100650.4	72976.56	68638.51	53455.77	55258.6	51629.5
40115.92	64743.64	81283.51	56386.11	70886.28	50861.76	57549.88	49173.64	33008.45	53979.21
34438.39	59679.51	74795.72	51823.83	69827.27	56123.8	63408.19	46885.6	37935.7	52478.27

LN555.008	LN555.008	LN555.008	LN555.007	LN555.008	LN555.008	LN555.008	LN555.008	LN555.008	LN555.008
42510.22	27784.04	42861.3	87961.9	51173.7	29746.03	27450.82	16528.34	54304.97	29286.77
43713.91	28623.08	53483.67	77125.25	40783.58	32638.69	39354.65	17463.44	41128.68	33123.96
65286.22	34070.96	71462.23	116555.9	52880.85	26006.04	43987.2	22446.41	70698.39	27504.53
41380.95	30064.35	45848.29	85642.41	52637.69	30716.49	29211.69	17149.18	54908.8	29593.68
37102.38	27067.15	46465.36	83501.11	54503.18	31515.44	29365.26	16177.61	54454.02	23459.92

LN555.008	LN555.008	LN555.008	LN555.008	LN555.008	LN555.008	LN555.008	LN555.008	LN555.007	LN555.007
18300.9	46361.64	38010.46	23244.08	38492.54	48734.55	65590.88	52581.33	62947.47	32331.13
23715.61	56969.03	33310.7	19125.87	54354.73	44989.88	67877.65	39603.66	70215.59	38928.18
19347.43	68687.75	29807.12	18333.43	72110.03	55597.71	59535.95	60916.37	61707.68	43824.59
21122.09	52562.48	41872.05	20258.72	37850.85	51491.8	67591.28	56105.85	59166.37	37486.87
20738.06	50602.55	40951.42	21983.57	34491.49	47781.57	62912.37	57570.26	60296.11	36120.09

LN555.008	LN555.007	LN555.008	LN555.007	LN555.007	LN555.008	LN555.008	LN555.007	LN555.008	LN555.008
38001.68	56259.84	71439.89	94596.21	73132.92	32901.31	43378.88	47993.95	40201.53	33207.47
53159.43	57977.89	66002.14	95186.29	74685.4	58306.77	42357.13	76627.48	46267.35	62529.65
49614.11	68748.21	70912.18	91208.37	68063.02	52993.71	71088.5	59215.03	35235.5	45447.81
40889.99	55679	68886.75	84901.94	78438.68	34733.47	41355.77	52842.12	38745.74	36656.72
34001.11	58148.55	64568.16	93514.73	69619.67	35109.22	45357.67	50173.96	33722.79	36179.21

LN555.008	LN555.007	LN555.008	LN555.008	LN555.008	LN555.008	LN555.008	LN555.007	LN555.008	LN555.008
45902.98	58902.24	33234.41	47550	16386.82	50499.81	19124.25	93192.01	56876.62	32726.84
56497.54	74751.27	32809.15	73683.71	17957.96	45462.33	24267.41	82813.32	46784.16	36309.59
48370.37	71201.9	43311.85	57553.21	16930.1	52089.85	11409.62	85332.23	67717.7	35889.03
45986.07	58578.6	34202.76	42608.87	19106.6	50908.19	20689.57	87141.43	57354.24	31606.63
43827.7	53282.04	31046.5	46527.09	18288.07	55342.5	20307.47	95642.41	53236.57	31303.53

LN555.008	LN555.008	LN555.008	LN555.008	LN555.008	LN555.007	LN555.007	LN555.007	LN555.008	LN555.008
30212.64	53977.71	49392.55	57152.5	43930.63	70364.29	69623.65	50468.74	24107.13	25136.67
40757.29	52867.76	38542.72	51163.47	35586.85	62681.87	60925.13	81298.58	51801.32	21422.97
40618.78	59992.05	53772.57	53356.98	45766.52	92777.21	61394.9	89534.48	28336.51	26348.92
31867.06	60879.54	50281.63	53361.3	42606.68	71079.81	63683.31	55061.51	28514.58	30195.01
27884.14	54559.64	54726.69	61398.94	39279.06	77400.15	63069.11	51031.87	26278.72	29268.49

LN555.008	LN555.007	LN555.007	LN555.008	LN555.008	LN555.007	LN555.008	LN555.008	LN555.027	LN555.213
23015.72	32020.5	82879.11	34673.18	52602.87	69772.07	34776.18	26521.23	74315.96	95336.05
17999.54	27683.58	112218.3	33328.57	53303.68	80899.16	42894.63	27680.82	67393.91	96352.61
24534.52	32127.62	94085.12	26834.09	54671.84	65951.04	36790.31	48788.5	64422.23	85162.2
18632.8	29462.42	80634.33	32675.71	51872.21	70750.8	34504.9	28477.91	67776.22	94989.05
18452.74	29430.49	81716.48	35902.12	52364.7	67074.78	33613.09	29996.13	63384.87	76225.05

LN555.266	LN555.265	LN555.265	LN555.265	LN555.265	LN555.265	LN555.265	LN555.265	LN555.265	LN555.265
17932.74	24376.71	27979.05	27210.45	37512.18	34515.84	24268.02	39484.24	49215.76	27700.18
22887.96	24913.89	28049.54	34050.46	32622.49	39578.2	26686.78	42529.49	49564.62	27512.61
23242.17	20947.65	33171.27	32865.68	33287.04	35423.53	23727.82	44182.22	56836.78	24481.29
21718.01	21139.54	34062.09	34786.18	38500.68	39246.62	24517.04	43555.23	54808.95	25657.09
24962.45	20076.42	28764.69	29677.33	32040.02	35484.29	22436.76	45068.41	50811.31	23545.46

LN555.265	LN555.265	LN555.755	LN555.775	LN556.718	LN556.752	LN556.772	LN556.881	LN557.187	LN557.187
26533.18	31604.22	13704.5	12583.97	31144.5	25111.29	22746.95	26876.69	16471.88	20955.95
27891.48	36245.63	12648.32	11559.3	24714.54	26564.16	24451.56	29408.83	16232.2	22618.8
21850.03	35488.81	14425.54	13670.28	30892.14	28553.96	24712.23	23317.26	14077.94	22883.98
28803.06	30788.17	18071.68	16109.48	34387.97	25801.19	26404	20723.15	12482.76	20667.76
24012.51	32996.34	19116.27	17461.71	44498.83	35492.26	20884.38	22613.13	15816.88	20865.35

LN557.187	LN557.188	LN557.188	LN557.188	LN557.240	LN557.772	LN558.714	LN558.750	LN558.770	LN558.982
29365.56	26627.12	20921.85	39458.31	41462.12	13729.2	13596.12	14608.32	12499.02	30811.12
28137.41	29179.27	20544.18	41044.2	42538.7	13934.62	11993.39	14712.56	9915.029	28833.06
28749.97	29237.46	21213.75	36102.06	68611.21	13915.04	11063.52	12997.12	9924.847	29937.95
27027.07	27183.88	23070.33	36670.75	86297.84	14210.32	11536.49	14246.99	12979.34	28242.96
26519.59	27555.48	19003.16	36759.97	94123.81	14563.55	17073.86	21641.36	13180.24	29748.53

LN558.982	LN558.983	LN559.215	LN560.997	LN560.997	LN560.997	LN560.997	LN560.997	LN560.997	LN560.998
34356.01	38941.33	7541.797	40194.54	34398.98	39823.38	25598.08	34771.14	37661.67	40893.29
34557.78	36720.74	8109.453	38593.75	30821.01	33138.87	26728.38	32663.12	44297.74	41624.52
31548.29	38152.59	10507.91	35454.35	35527.08	35241	28323	31698.34	37749.78	39683.07
33751.33	38863.87	12183.03	37435.58	31787.96	39871.13	25372.38	32205.13	42010.31	42774.78
33644.63	38555.78	12812.14	35516.85	32094.4	33476.73	27813.59	33956.41	41878.69	36776.11

LN560.998	LN560.998	LN560.998	LN560.998	LN560.998	LN560.998	LN560.998	LN560.998	LN560.998	LN560.998
35972.75	38689.38	29757.38	34359.95	32663.09	42514.47	42723.87	42886.63	45924.47	31769.09
38352.3	39750.71	33417.45	37594.17	35713.14	44276.23	42573	42761.1	44139.36	30847.86
39874.37	35459.89	27424.07	33925.73	33516.62	41803.89	42410.76	42220.89	47114.62	31229.05
36085.01	43128.95	36359.16	37645.64	34670.71	41908.99	39779.64	42381.38	48909	32624.91
37558.82	35157.03	32777.07	33534.31	30335.36	45893.72	38505.44	39500.23	48876.67	27684.15

LN560.998	LN560.998	LN560.998	LN560.998	LN560.998	LN560.998	LN561.037	LN561.037	LN561.037	LN561.036
27468.21	29049.71	38892.5	28992.83	45552.46	26721.99	48644.47	77690.5	73075.53	39143.23
27228.02	30019.39	36837.36	27921.37	42629.52	25289.84	48514.74	74068.91	79788.36	39952.32
30772.65	27294.76	31952.1	30304.14	45044.54	26812.78	50968.63	78184.66	75690.29	46204.26
28185.38	31063.47	40576.71	26611.7	45965.16	27074.84	50974.28	77286.51	73057.43	38992.72
30418.07	32525.63	35912.93	28578.82	44695.36	30098.52	47457.73	73368.75	74853.08	43059.54

LN561.037	LN562.777	LN563.119	LN563.231	LN563.231	LN563.232	LN563.231	LN563.230	LN563.232	LN563.232
58598.03	12966.16	22746.07	24435.38	25760.05	23158.61	25201.83	24809.28	22452.12	18565.84
58138.47	9026.32	22050.43	25056.55	24445.04	22398.97	21745.46	23979.36	24568.78	19339.98
56135.72	10997.48	20171.17	25659.64	25808.9	23104.62	20989.34	25579.62	22838.31	18266.88
57140.99	11725.64	17428.11	25441.87	22933.97	26939.29	21529.55	25660.57	24350.44	18627.6
53916.62	15695.88	19759.91	23200.25	25601.01	21216.75	23416.25	27961.68	23560.3	21128.68

LN563.231	LN563.232	LN563.232	LN564.344	LN564.345	LN565.114	LN565.114	LN565.169	LN565.211	LN565.211
24562.08	24246.01	26335.4	2142122	19736.52	23018.52	22351.84	65328.27	32243.2	25149.36
24893	21897.95	29462.53	2286623	22620.42	24659.71	23606.36	67066.03	35014.3	24256.9
20451.69	25565.98	28823.82	2224036	20190.27	18089.67	20447.84	65116.96	31486.67	22781.9
25244.74	26384.23	26866.27	2077560	16250.84	21809.59	23307.53	65491.05	36066.14	20349.44
22372.19	26215.31	31424.35	2158740	19361.96	23976.06	21194.56	58102.98	26939.21	23626.25

LN565.211	LN565.211	LN565.210	LN565.211	LN565.211	LN565.211	LN565.211	LN565.211	LN565.247	LN565.248
25739.77	25780.11	27454.42	27788.51	39725.67	29370.19	21815.29	20650.92	44147.4	26549.11
24701.74	26047.71	24633.9	24984.09	37159.49	28201.53	25055.84	24243.51	45940.61	30564.15
27703.29	26011.02	26513.65	26694.22	44513.61	30015.62	20988.34	20586.42	41109.96	28701.9
25704.36	27162.36	27582.14	27296.97	37662.41	27703.06	25157.52	20571.72	42691.7	28972.71
24641.62	25639.42	26819.56	23510.11	39886.95	24689.92	21633.6	20335.95	42737.16	26876.09

LN565.248	LN566.988	LN566.988	LN566.988	LN566.989	LN566.989	LN566.989	LN566.989	LN566.989	LN567.048
22960.91	48181.41	56840.88	41524.81	40601.71	41119.18	56475.39	42352.15	54262.81	33221.44
21741.76	53211.44	55961.3	38063.42	33085.53	40677.47	54057.13	45238.84	51676.69	40608.7
20095.81	20136.61	50620.32	42601.78	33659.87	39098.08	53758.64	38516.67	46818.12	34563.79
20421.6	52344.29	53882.37	40061.52	35284.48	40597.36	53738.85	45529	53936.76	34920.07
22158.89	51626.05	52555.52	40383.25	34349.9	38203.9	52084.39	45025.27	50456.09	32862.2

LN567.170	LN567.227	LN567.227	LN567.227	LN567.227	LN567.227	LN567.227	LN567.227	LN567.227	LN567.227
23787.16	26158.3	38223.29	40068.8	16533.07	16552.1	18834.53	28886.95	23618.03	13936.54
22170.2	23439.11	34270.29	43037.85	18077.6	19662.72	18684.85	32991.52	23727.42	11637.08
19791.88	25069.48	34054.9	38971.77	16981.25	17937.03	21148.21	28889.83	23744.02	12448.92
19492.08	27290.28	40799.45	39941.97	22553.2	21046.61	25229.59	29899.96	25264.88	11828.8
20594.22	23662.66	35837	39654.89	18116.87	17385.03	17966.57	28502.89	22430.42	11664.5

LN567.227	LN567.227	LN567.227	LN567.302	LN567.301	LN567.301	LN567.301	LN567.301	LN567.302	LN567.301
33941.4	35920.75	29177.25	22282.54	23371.27	26274.49	27446.56	14431.51	17342.94	26603.6
32523.62	36428.22	29644.87	23799.71	22284.27	27304.28	26575.16	17672.07	20199.49	27906.35
32772.25	34781.61	30112.94	22283.55	21903.15	26839.1	30332.58	16822.22	19632.91	25993.6
41432.68	38087.34	32856.88	22749.09	26469.67	24796.86	28053.25	17372.48	20688.32	27169.3
35069.72	28132.79	29596.65	23947.4	23782.17	23362.65	26349.34	14968.57	18124.85	27527.71

LN567.302	LN567.301	LN567.301	LN567.302	LN567.536	LN567.727	LN567.765	LN568.725	LN569.022	LN569.045
22534.44	26001.53	34200.73	18480.96	19642.53	46883.52	14053.36	17521.66	52504.53	70335.89
24912.24	23946.7	31927.54	17720.98	17741.71	37809.04	15103.93	10059.98	53719.23	76021.16
24910.86	20875.06	33312.72	18617.34	18245.93	40909.89	16643.59	14482.38	61096.41	68109.57
25056.29	20821.34	35276.49	19657.07	15826.43	45932.08	11944.4	16548.65	51082.93	72839.39
22567.81	21734.41	33330.95	19376.56	13468.69	51737.27	11917.96	23709.76	53426.98	66737.09

LN569.045	LN569.045	LN569.045	LN569.206	LN569.207	LN569.207	LN569.207	LN569.207	LN569.727	LN571.001
72676.88	61874.39	90803.1	25282.09	24710.44	25643.5	27143.96	25217.54	19817.52	23731.48
69597.06	59681.89	81339.65	22738.59	23296.44	25493.54	27838.45	27852.86	21059.43	23480.44
68163.6	58401.44	91232.99	21514.78	28293.32	20826.03	26547.33	20957.2	17816.73	18821.65
69865.49	65736.66	93468.94	21382.54	22492.73	23209.44	23091.71	25388.6	20332.47	20985.55
67480.59	59201.75	95868.2	23277.77	22118.71	23530.27	29035.07	22794.26	23750.49	19940.94

LN571.001	LN571.001	LN571.001	LN571.001	LN571.001	LN571.001	LN571.001	LN571.001	LN571.000	LN571.001
29993.8	28866.47	36097.62	32237.31	35650.9	31295.46	31331.51	40884.15	35312.13	26360.91
27780.09	26867.29	37963.68	31679.96	38282.05	32583.21	31601.59	39187.8	34232.89	27440.68
25134.8	22551.45	32055.31	26095.45	33495.72	30313.8	28059.58	33020.58	28249.13	31897.05
26758.33	30057.4	37060.61	30392.03	35441.83	30461.39	29705.89	37481.96	36541.48	25710.02
26477.15	27999.73	37287.29	32702.59	40120.8	33705.96	31484.56	39434.54	31684.14	27315.06

LN571.000	LN571.001	LN571.020	LN571.020	LN571.021	LN571.040	LN571.040	LN571.041	LN571.040	LN571.040
24005.2	35941	39485.36	42562.49	23963.71	36179.27	29990.85	33871.58	34374.19	42752.33
24362.09	35793.79	38551.27	45490.32	24123.74	37789.13	27838.05	34878.72	33865.26	45169.27
20011.93	35040.05	36206.98	43980.07	23218.17	31667.79	26720.45	34873.29	39528.96	43734.65
24401.89	38821.38	35695.13	38634.56	23782.8	34934.62	28404.15	33708.36	34729.02	42743.29
22272.76	39288.14	37609.03	43999.93	23884.77	32136.56	26467.83	32476.81	39440.22	40683.21

LN571.040	LN571.497	LN571.497	LN571.498	LN572.500	LN573.493	LN574.266	LN574.497	LN574.995	LN574.994
35713.91	30235.38	658229.5	31352.65	233557.9	183909	14896.92	63918.53	33480.3	26740.89
35593.41	25501.82	610248.1	32881.63	221184.4	184669.7	15500.26	63436.37	38886.13	30686.86
36532.84	28036.98	638869.3	27458.57	236458.4	176325.2	13157.46	64655.15	41488.58	33965.52
35641.2	25656.76	647031.8	32033.62	142451.8	147976.5	10469.34	57564.8	41765.83	30486.54
40120.8	30215.09	630890.4	33549.41	231567.9	177511.9	10950.33	58380.89	40998.56	31460.03

LN574.995	LN574.995	LN574.995	LN574.994	LN574.996	LN577.399	LN579.066	LN579.066	LN579.130	LN579.130
36065.57	24823.93	30985.54	27461.45	29725.15	17487.88	38976.17	22319.73	31237.89	21033.97
37271.44	25536.64	44513.04	33133.46	30208.36	22382.3	42429.85	23570.25	31486.46	22051.49
37707.15	27219.64	43836.84	25556.38	29455.89	19759.55	36869.09	22758.5	33512.09	19462.11
37012.56	28254.25	44299.63	27825.22	27316.99	13260.12	35053.41	22968.61	31890.29	20877.91
40083.4	25623.19	42424.05	27273.57	28975.62	14356.68	36109.02	19826.61	30756.08	19658.29

LN579.130	LN579.131	LN579.226	LN579.226	LN579.227	LN579.226	LN579.301	LN579.301	LN579.536	LN579.780
24201.84	30946.91	27533.86	27754.86	25662.65	32247.49	39956.62	23892.45	16861	10228.16
25751.78	29155.36	23710.14	29801.86	24693.63	31690.67	41165.81	26751.56	17372.9	9841.28
25770.56	26193.44	23307.58	26951.77	25939.75	29858.77	38003.27	23728.78	19594.75	6614.124
26791.61	28517.62	24504.75	27776.09	23211.73	32963.97	40538.4	24809.05	18428.35	11693.78
25558.46	27994.76	26911.06	29410.01	28465.63	30432.5	40883.08	22146.17	16594.01	12228.06

LN580.339	LN580.567	LN580.985	LN580.985	LN580.986	LN580.984	LN581.004	LN581.004	LN581.004	LN581.140
69218.36	7820.872	44466.96	53334.18	49755.88	62274.37	49146.77	50471.87	33453.25	30083.36
69010.28	8927.119	42507.53	52535.21	50446.19	69120.96	28548.71	33915.53	23549.73	44274.59
64346.87	14084.43	44672.09	58687.88	48540.62	64855.9	43405.76	55622.92	35004.37	66028.41
59124.83	17520.39	43399.5	56812.53	44901.11	62659.55	46938.42	49812.92	35158	72600.26
56560.25	19272.04	45969.04	60486.3	50897.77	75887.17	48374.96	48191.04	34095.74	74711.6

LN581.526	LN581.526	LN581.526	LN581.526	LN581.527	LN581.526	LN581.526	LN581.526	LN581.526	LN581.526
2353188	65375.85	65159.33	76693.97	33298.79	65052.31	47921.63	51422.47	67783.11	55630.27
2324095	73964.97	76901.23	55999.41	44983.94	61108.24	60849.71	55244.57	50216.75	66893.23
2289660	95039.34	63791.41	65153.92	35483.73	71329.67	55773.56	51339.24	47468.29	65311.61
2202380	90752.87	63177.49	62373.35	32018.42	68037.09	62512.56	44999.63	50033.27	62932.23
2269575	94795.3	73167.17	59611.39	34360.1	66024.98	54622.7	47342.29	49655.73	64360.91

LN581.526	LN581.526	LN581.526	LN581.527	LN581.526	LN581.526	LN581.527	LN581.526	LN581.527	LN581.526
43166.42	33801.88	50873.26	68740.21	47789.37	37036.88	59452.86	54293.73	53169.37	65785.75
43921.47	42544.92	73233.59	72770.92	44821.31	35331.95	49797.15	79231.93	45015.81	50888.64
50985.46	41029.47	67354.6	69026.06	37098.4	38623.75	47782.08	68007.25	46426.09	46946.14
43351.11	44359.34	65964	68937.38	36978.92	37667.24	43228.98	56211.8	42122.76	49131.2
43258.15	38707.82	65052.68	64084.57	35117.29	33375.24	46845.09	58231.22	43920.51	48787.78

LN581.526	LN581.527	LN581.527	LN581.526	LN581.527	LN581.526	LN581.527	LN581.526	LN581.526	LN581.526
55308.3	54783.67	47613.59	69248.11	49744.19	42953.85	48272.79	52593.94	31883.23	31992.97
38439.68	38898.54	41027.37	62155.24	44683.7	48937.15	40753.14	27746.33	44820.51	37732.4
37337.44	44495.37	42889.25	61849.98	38940.59	41163.68	43595.69	31393.77	43893.64	35876.78
37748.27	42520.29	42338	64821.45	39008.92	41224.53	40601.49	28957.92	46629.12	36531.24
37870.26	39197.09	34628.88	64412.6	38654.87	44772.09	37981.34	25189.14	42879.35	34738.7

LN581.526	LN581.526	LN581.526	LN581.527	LN581.526	LN581.527	LN581.526	LN581.527	LN581.527	LN581.527
36094.09	77301.02	33992.87	45005.54	44453.27	51987.47	64839.55	29102.04	52449.2	44176.62
41823.82	60702.64	43102.76	32320.85	47473.48	53351.61	47273.02	42203.34	56838.19	48405.67
36980.81	59949.08	40733.38	28033.52	46691.88	49442.68	49952.3	45345.97	53929.94	47686.69
40321.59	58865.74	39493.72	30759.6	43672.11	49018.65	44920.81	40578.48	51367.63	46091.5
38517.6	59284.62	36344.61	24933.4	41201.92	46043.32	39326.05	43913.27	51159.47	46732.71

LN581.527	LN581.527	LN581.527	LN581.526	LN581.528	LN582.529	LN583.062	LN583.062	LN583.062	LN583.062
55037.98	48759.12	55516.68	39377.2	66636.36	874838.4	25798.17	21613.18	24389.93	29198.02
52865.63	44977.37	43412	43952.96	70309.91	822199.3	25236.34	20967.47	27562.96	30203.98
48740.34	43398.23	42575.41	44399.28	73497.49	832063.1	23937.07	19969.22	29728.74	26156.79
47797.29	47151.76	45274.04	41737.62	63106.89	847062.8	22574.67	16921.1	31136.14	30696.64
50940.01	39964.48	43443.29	37833.51	66263.49	817937.7	23175.81	17900	24470.68	23674

LN583.062	LN583.296	LN583.532	LN584.416	LN584.535	LN585.056	LN585.055	LN585.056	LN585.056	LN585.312
31920.61	27907.04	177446.8	56853.92	14903.2	21289.15	15607.82	17939.08	20566.01	32528.5
30680.55	24600.53	175809.3	60114.62	13317.36	18260.05	17725.39	18082.76	21473.2	36034.26
32743.51	24690.84	165072.6	56870.61	15247.76	22917.42	17579	18329.86	23303.24	36476.98
32598.88	27381.48	164119.7	51333.69	15285.43	18955.16	15448.35	17923.59	20431.37	33033.85
28592.56	25925.48	169534.8	51775.34	14124.86	19538.3	15883.49	17719.05	24649.14	33667.23

LN585.312	LN585.312	LN585.329	LN586.691	LN586.747	LN587.013	LN588.691	LN588.749	LN588.897	LN588.897
25475.55	28446.14	56278.5	28172.33	22686.18	51324.69	20101.7	20743.01	30713.31	61423.7
17590.16	25600.6	56189.09	27237.25	28385.86	53591.38	22309.83	22073.9	24092.94	63150.67
23147.17	29162.34	89866.3	28464.51	25120.84	55393.62	21214.71	21079.2	28432.08	74697.44
20652.59	27082.67	112038.9	32183.09	25844.55	51286.71	23821.78	23070.19	24738.08	59669.14
22396.43	27976.87	120060	40522.05	33740.44	54602.4	31984.92	23191.65	24655.41	55783.51

LN588.897	LN588.897	LN588.897	LN588.897	LN588.897	LN588.897	LN588.897	LN588.897	LN588.897	LN588.897
20784.65	60062.67	45047.51	58569.59	22570.04	69648.99	50332.04	64198.93	46473.34	43003.5
14401.46	52895.89	57595.02	57008.63	26662.61	65689.79	57974.21	65454.9	48500.61	55995.91
18102.57	51851.02	66118.82	73627.71	28815.01	72451.68	56145.59	79266.17	60017.95	63439.68
20883.56	50969.97	48660.76	53486.81	25510.71	59170.83	53411.99	63118.45	48245.61	45834.6
17920.54	47537.46	47877.95	53236.65	22301.26	64323	55226.59	61295.79	48566.94	43381.56

LN588.897	LN588.897	LN588.897	LN588.897	LN588.897	LN588.897	LN588.897	LN588.897	LN588.897	LN588.897
59229.14	46852.26	51377.53	38084.5	66025.1	77953.56	42255.46	46921.72	45872.08	57881.19
44404.94	43206.32	34907.82	44998.65	63889.05	74011.37	43696.74	50491.34	53455.09	59032.22
47112.3	53782.48	45525.08	40505.44	63138.5	55539.46	36501.08	45252.53	54517.54	70246.29
47629.7	41460.76	32586.74	42178.12	56730.54	70504.42	43346.8	45036.41	54300.99	57749.16
41990.06	43023.14	31019.35	38493.74	62972.15	76056.11	41945.99	42899.7	47595.73	54546.69

LN588.897	LN588.897	LN588.897	LN588.897	LN588.898	LN588.898	LN588.897	LN588.897	LN588.897	LN588.897
71326.2	54331.64	51910.26	20037.96	31885.34	44190.97	48272.07	77492.74	27985.64	37386.83
66713.8	48270.53	50033.49	24194.55	28302.38	43152.19	42606.46	78229.86	24930.36	34790.1
55790.1	51494.91	48062.41	27088.31	27334.24	46081.73	47461.17	72190.97	28311.15	37814.43
56903.46	57337.2	46450.42	22796.69	29620.89	43502.37	44530.5	73512.06	26138.45	31782.59
53939.04	48615.8	39049.23	24424.55	27000.61	49294.9	40233.61	69613.54	26427.72	33584.25

LN588.897	LN588.898	LN588.897	LN588.897	LN588.897	LN588.898	LN588.897	LN588.898	LN588.897	LN588.897
46908.19	52393.88	60147.76	73057.94	36663.05	23099.26	32349.92	50301.96	76420.02	23565.63
33998.39	34680.89	47649.32	64121.82	40401.51	22303.05	31553.55	36186.08	67089.5	24441.5
46006.11	32576.61	57905.94	76198.39	33051.9	32037.28	31041.08	37746.57	62235.31	27472.69
42330.85	30681.76	46689.49	66860.3	41925.53	20700.72	29882.89	36342.56	71010.91	22595.6
34766.06	31904.45	46780.63	70476.24	36488.81	17242.82	25248.97	36049.66	71703.36	21762.92

LN588.898	LN588.897	LN588.897	LN588.897	LN588.898	LN588.898	LN588.898	LN588.898	LN588.992	LN588.992
61831.47	30579.64	22210.84	47914.35	40893.44	56291.86	34406.67	22456.85	36744.56	28631.98
66163.39	28256.04	20106.07	41155.95	36603.31	51644.03	28908.72	19794.2	36307.95	30055.27
62716.86	30128.8	22875.52	44107.53	36914.14	50325.59	30368.98	21206.65	32790.68	29238.45
58062.6	23052.99	19155.33	44040.18	39634.2	50462.64	32275.77	15781.19	36436.55	28716.99
55416.68	25887.1	18691.77	40592.21	38429.37	46857.16	27105.81	20390.99	35618.6	25311.77

LN588.992	LN588.992	LN589.010	LN589.029	LN589.029	LN589.153	LN589.517	LN590.155	LN590.155	LN590.433
40065.96	24436.16	31673.8	37281.73	25288.15	30309.87	40040.83	17574.88	24898.72	48653.79
40844.91	27618.59	33554.56	36076.58	28066.13	37974.94	35680.71	16787.31	23375.09	50435.08
38989.19	26134.43	27347.7	37280.31	21458.1	35223.09	30158.36	16242.91	26151.29	49513.1
42065.93	27359.73	32673.98	31723.08	24535.84	26846.58	29464.34	17074.02	25316.72	46236.09
35690.74	24439.03	30572.35	35847.33	22424.91	30523.71	28318.94	15340.32	24238.7	43686.17

LN590.987	LN590.987	LN590.987	LN590.987	LN590.987	LN590.987	LN590.987	LN590.987	LN590.987	LN590.987
23179.55	21523.41	24349.34	26607.11	40372.09	30728.12	27755.95	32269.79	31213.8	29488.44
27050.66	24546.27	27200.27	26818.15	35231.83	29930.7	27599.71	33603.02	36581.86	30478.6
25591.38	23163.53	25520.95	25956.81	35088.66	31597.82	28139.04	34619.88	32304.83	28141.24
26424.89	20950.32	25809.35	27497.81	37316.46	30834.96	26986.75	34036.31	32903.07	30127.94
25125.86	22623.04	23070.64	28103.45	41686.95	30632.49	28506.74	35545.01	34128.21	28226.43

LN590.987	LN590.987	LN590.987	LN590.988	LN590.988	LN590.988	LN590.988	LN590.988	LN590.988	LN591.263
30399.32	31944.27	21257.68	20600.43	28313.64	34596.21	27321.47	33849.4	21086.65	20855.69
29023.63	31603.58	23946.88	18046.5	29420.97	35199.11	27329.41	35905.7	21024.52	20914.99
31053.24	32745.31	23442.25	24132.12	28184.89	34947	29065.62	31670.27	18370.59	20288.89
27366.93	28596.86	22864.14	23271.97	27196.13	34868.72	31946.14	35165.14	18422.77	20772.78
30688.33	32855.76	22897.67	19502.44	28470.7	34167.55	30799.63	37839.94	20640.94	19898.19

LN591.263	LN591.263	LN591.263	LN591.263	LN591.572	LN592.576	LN592.764	LN593.004	LN593.004	LN593.004
20303.6	23441.41	23198.72	17914.84	63300.64	21780.87	33411.51	36850.96	34782.98	33509.12
19342.96	20409.41	18102.22	18409.71	57527.42	17523.88	33590.54	33422.04	32048.35	36742.51
17827.01	22161.16	22095.68	19764.08	51858.3	22349.76	30781.47	35357.3	32465.63	35759.51
20181.04	21955.02	19496.5	17962.91	51916.02	18570.27	29348	38749.79	35557.89	39439.36
18496.32	20251.98	21084.69	17850.08	41654.42	18704.23	30585.71	31437.2	34201.06	35718.39

LN593.004	LN593.003	LN593.004	LN593.004	LN593.022	LN593.023	LN593.023	LN593.023	LN593.023	LN593.023
35649.03	22484.11	39246.31	28042.58	30529.93	25587.53	35229.8	39250.07	43589.08	27644.23
34563.07	25089.76	37276.9	32844.95	31652.57	25016.3	35820.82	39476.83	46140.05	25040.83
35195.19	26431.09	37640.25	28212.96	31017.09	24606.85	35164.92	41815.22	42815.21	29456.62
38515.95	25661.76	38011.09	29309.29	34836.74	28041.67	38872.66	39989.69	47660.68	29032.95
37755.25	23535.08	33453.67	30541.95	29096.67	25363.27	34069.9	37094.28	45309.04	28136.71

LN593.123	LN594.766	LN595.000	LN595.001	LN595.001	LN595.020	LN595.039	LN595.040	LN595.040	LN595.040
14327.82	11782.26	43752.45	43492.7	44360.14	37544.79	26702.59	40038.62	25869.84	37528.08
15288	11276.46	37175.5	43221.98	46797.54	37183.68	32102.32	39588.53	27995.93	40589.31
17893.59	10188.54	38572.45	42544.94	46480.8	35978.56	30637.52	42994.98	26866.04	36452.65
20256.27	12852.82	41398.47	44770.94	49417.12	36135.73	30448.23	43063.22	27337.16	36807.93
18839.89	14691.25	43515.4	43843.58	44411.82	38657.71	25889.38	40612.73	28228.46	37355.1

LN595.041	LN595.119	LN595.170	LN595.425	LN596.770	LN597.055	LN597.056	LN597.056	LN597.056	LN597.312
42075.66	9916.473	34692.63	19912.68	24746.43	58026.9	43232.84	67283.97	60542.31	24138.93
39378.39	10268.25	28914.24	21118.45	22222.98	56325.41	36750.81	70071.19	59237.85	22463.88
37115.23	11171.4	36765.6	23074.78	23443.67	51661.19	47099.79	71243.44	47662.64	22518.99
42380.92	9190.437	28686.98	14841.65	29935.71	54180.04	41221.82	74609.05	54109.76	24650.11
42027.55	10159.77	31052.27	14663.38	28924.43	54853.91	45643.91	60780.16	50636.26	29224.3

LN597.311	LN598.516	LN598.770	LN599.073	LN599.519	LN599.529	LN599.716	LN600.389	LN600.522	LN600.532
20931.37	562594.7	13229.66	30119.15	193971	701719	40327.04	37065.08	29828.34	260077.4
20551.73	564310.2	8480.011	31077.25	199792.7	697212.8	36785.35	41571.94	31001.49	272199.5
18484.24	514886.5	12318.79	29359.83	181301.2	734140.1	39178.91	37426.08	24918.83	280702.5
20554.04	526430.9	14392.36	33015.37	185995.9	738972.6	46770.6	35068.19	28423.64	301658.8
19032.9	505702.5	16526.19	28267.79	192810.7	769602.8	59166.89	30455.89	16808.11	214789.1

LN600.723	LN600.992	LN600.991	LN600.990	LN601.010	LN601.010	LN601.010	LN601.009	LN601.010	LN601.010
19243.23	71154.91	68750.39	62141.82	63278.5	74484.67	62894.53	64790.77	71467.01	55620.37
19136.95	76947.89	69404.23	57529.35	72994.86	70846.05	33892.55	65959.48	38661.75	51821.37
18889.67	72199.61	75725.87	63120.53	64346.66	72755.54	58523.94	64827.37	64638.15	49170.13
26021.68	74650.25	69991.63	62135.37	65579.73	72445.52	61946.48	64670.54	65456.94	50616.98
34544.22	76332.61	67983.71	62227.44	54060.11	71509.12	64917.79	66429.28	68356.08	51463.3

LN601.010	LN601.172	LN601.171	LN601.172	LN601.171	LN601.307	LN601.307	LN601.307	LN601.306	LN601.306
52461.73	25182.31	25191.01	27513	23583.97	33890.79	26516.85	27436.56	19728.39	26131.4
55697.65	26729.01	28309.84	23376.42	24168.82	35106.33	28472.07	29286.64	21006.93	29060.7
56500.83	25341.3	27298.27	26197.44	24104.93	34818.36	22795.32	29432.73	20981.39	26156.71
49408.25	22602.93	27598.79	26568.84	22429.45	35917.7	30053.98	27637.44	18042.31	23702.43
45801	30113.66	27269.51	26125.08	23655	36979.03	22791.36	28947.21	22826.02	24246.66

LN601.443	LN601.525	LN601.719	LN602.529	LN602.719	LN602.769	LN602.791	LN602.988	LN602.988	LN602.988
37747.93	209628.4	22572.53	73196.3	78059.42	28887.7	23560.86	32751.26	47632.04	33045.95
38222.27	198910.4	22906.27	78660.46	66572.14	30746.46	33460.84	29677.95	48648.35	31982.75
41465.14	190548.8	24223.49	78395.3	76017.27	30087.55	29270.87	33704.06	36601.83	31336.16
26728.55	204318.2	30483.74	49210.39	86775.62	33244.62	26304.24	32128.51	40587.74	33208.04
33200.58	211857.4	34505.57	81087.29	126219.1	35773.93	33064.22	32673.3	43283.96	27969.08

LN603.007	LN603.007	LN603.007	LN603.007	LN603.007	LN603.007	LN603.046	LN603.476	LN603.722	LN604.479
44516.85	35954.45	32784.39	32722.56	50912.96	29339.39	31220.49	140678.7	20017.1	51062.01
38829.14	31719.44	32168.74	36103.31	47578.87	27942.14	31232.24	138069.6	12173.97	53976.8
35751.31	31945.46	35489.62	39592.85	39976.3	30796.65	33399.42	137002.9	18207.38	40327.07
38041.26	35471.69	31431.45	41313.17	47412.59	28074.06	28985.78	154589.9	20625.23	42964.74
35815.62	32197.26	32059.75	35319.83	42853.46	29168.4	31022.56	160242.8	22093.15	56884.29

LN604.719	LN605.125	LN605.165	LN605.165	LN605.164	LN605.164	LN605.164	LN605.473	LN606.476	LN606.716
36305.97	35169.6	24680.73	21528.2	54755.08	48508.74	49577.95	40130.26	7638.904	17433.22
37115.66	31857.48	25314.21	20503.61	52172.09	45031.69	52924.85	43845.8	11964.05	14210.83
39111.84	31451.03	30756.04	21743.05	52102.46	47751.24	51433.34	43362.25	9171.535	17306.16
40986.19	31752.53	22206.86	20808.51	57459.35	47822.71	51688.27	32946.08	12454.67	19517.27
53245.57	28522.84	25261.75	18887.69	52625.88	47116.19	50648.85	47576.85	10974.8	21179.14

LN607.002	LN607.001	LN607.001	LN607.020	LN607.020	LN607.021	LN607.020	LN607.021	LN607.020	LN607.100
27146.45	42767.3	34586.71	41492.71	27092.61	38041.49	27827.17	31830.4	26223.09	27030.44
27782.65	45179.07	39135.13	31765.46	27343.76	41009.21	28236.71	29227.65	25499.33	25316.55
28620.2	44160.93	31805.65	42138.78	25995.77	36880.39	26017.95	31048.86	26725.16	25405.47
29979.37	46538.55	33969.85	42000.73	23557.09	43151.23	28908.95	35929.22	29430.33	24437.72
30150.88	44568.64	30831.55	40283.58	24681.41	42394.72	29089.94	31758.47	26284.77	24255.74

LN607.100	LN607.100	LN607.100	LN607.100	LN607.100	LN607.100	LN607.156	LN607.257	LN607.258	LN607.258
17323.07	27806.56	36486.51	19009.59	24266.99	36795.28	19174.46	19351.81	19706.23	21375.51
21150.51	31971.25	33431.96	18806.06	23416.8	35703.85	21814.46	18088.9	18301.91	19809.47
21841.06	29091.99	34661.19	21044.76	22985.58	33448.52	18265.46	19485.97	21257.5	19926.39
21842.37	25432.34	43693.63	19828.34	23350.54	33986.76	15827.61	17678.27	18394.3	17990.03
19623.84	29569.63	33404.31	20352.25	26310.71	33088.25	15922.83	18427.36	18243.86	20300.15

LN607.258	LN607.258	LN607.258	LN607.258	LN607.258	LN607.258	LN607.258	LN607.720	LN608.737	LN609.017
20534.44	28874.1	19603.37	15144.81	31975.57	26587.74	31742.23	24273.97	13404.37	27147.65
19769.92	28232.12	21922.38	19192.46	30868.28	29181.43	29258.71	25378.83	15781.3	28887.1
18440.14	28283.48	21400.67	16984.73	32987	27034.57	30576.47	21162.31	15207.29	29493.22
20452.23	31236.32	22678.34	18816.54	29631.33	28666.96	34078.73	26691.39	17375.89	27966.15
18541.97	27656.41	24236.81	16230.32	30227.8	23486.33	28825.38	27015.61	17972.46	27518.61

LN609.017	LN609.018	LN609.018	LN609.018	LN609.018	LN609.510	LN609.557	LN609.558	LN609.557	LN609.558
23216.33	27760.12	26141.82	31214.96	26885.46	15387.52	1949130	58272.52	45833	67106.85
27006.98	28087.97	26884.47	33364.22	28852.39	20881.26	1995284	38372.36	46545.71	70408.98
25216.84	23878.62	24603.44	28617.38	33444.05	20600.03	1996583	52488.23	59830.71	97357.14
28983.06	24232.43	24148.92	26685.47	31601.52	20035.56	1974235	60743.79	47648.89	58046.66
23688.96	22853.84	24509.32	30499.38	29579.52	16388.46	2023633	53615.01	40628	65843.2

LN609.558	LN609.558	LN609.558	LN609.558	LN609.558	LN609.558	LN609.558	LN609.557	LN609.558	LN609.558
32207.46	55513.68	57384.48	57344.22	36885.91	51669.41	44150.73	33338.38	93400.27	38247.88
35599.34	57627.04	56257.4	52212.99	41456.14	64583.84	42215.22	38482.44	99774.89	38529.73
28315.97	60448.69	61387.14	54908.6	40058.39	47553.83	43205.86	30134.29	95197.63	34621.33
27078.88	64779.38	46181.46	60090.89	33765.79	47959.05	47117.61	38555.87	81483.38	37019.87
46956.24	50226	56832.38	49099.74	38594.45	40827.92	39351.59	35427.31	92566.06	42397.63

LN609.558	LN609.558	LN609.558	LN609.558	LN609.558	LN609.558	LN609.558	LN609.558	LN609.558	LN609.558
40932.14	37523.31	81437.19	37210.26	30201.38	37379.72	30192.26	43201.41	38261.57	34401.55
41509.76	38432.33	87458.29	38898.65	30264.77	39932.26	32453.38	41315.9	39611.5	35254.32
38903.55	34491.33	86540.54	38335.36	30720.31	38667.49	36219.22	57492.55	43704.01	33313.32
46860.26	51529.93	78029.51	74137.95	39607.51	52029.33	37856.05	51790.89	30856.56	31637.26
33630.3	33283.17	79813.54	55473.44	37523.93	46403.7	31339.01	32161.41	30369.5	33272.76

LN609.558	LN609.558	LN609.558	LN609.557	LN609.557	LN609.558	LN609.558	LN609.558	LN609.557	LN609.558
38235.2	42039.56	50314.86	32652.69	62223.57	43590.54	35277.36	45340.39	43882.53	55538.87
40119.22	43137.98	47635.65	32337.46	62610.68	40591.23	38647.26	46647.14	40069.44	58712.65
34011.97	40426.73	43492.65	33391.27	66608.37	38554.94	37730.21	46876.69	41678.7	55776.78
30859.87	48671.93	61869.95	44634.54	63176.59	36331.32	45570.41	35099.79	44578.96	53836.96
34877.33	36708.71	41918.75	33665	69836.68	41926.18	36038.31	44264.45	39313.08	42544.26

LN609.558	LN609.558	LN609.558	LN609.720	LN610.167	LN610.561	LN611.012	LN611.014	LN611.014	LN611.014
30636.29	41116.57	39516.17	16718.56	14518.18	867128.5	21854.24	32879.25	37218.12	24526.85
35306.46	39851.83	39929.65	12991.57	15438.06	824397.5	24063.75	34694.24	33810.14	20948.91
33315.09	39864.3	40695.17	16334.75	15517.59	858974	21173.81	34750.99	34878.74	24837.65
34758.17	39588.58	40078.14	13832.43	17553.17	871751.7	24386.71	33356.99	38382.45	23633.87
34662.12	45047.08	39740.74	18156.88	14903.22	863129.9	20556.55	32708.41	38624.4	26885.59

LN611.014	LN611.014	LN611.014	LN611.013	LN611.014	LN611.033	LN611.033	LN611.033	LN611.033	LN611.291
32564.24	25205.74	24384.94	28732.8	28551.13	31251.27	25607.75	35392.95	23760.06	14774.37
29936.65	26296.65	24903.81	29810.42	25049.25	28399.23	23292.38	34761.24	22046.17	15156.89
29712.41	27094.97	27037.86	27496.95	28022.33	27837.4	24617.68	37049.42	23534.64	10500.12
28994.75	27981.07	25458.66	33287.65	27112.55	29720.65	27173.06	37502.64	27201.22	14028.87
34431.18	23418.63	29609.59	30574.37	28006.6	30036.05	23457.21	38360.78	25029.07	13817.91

LN611.398	LN611.564	LN611.717	LN612.567	LN612.746	LN613.012	LN613.031	LN613.031	LN613.031	LN613.505
15203.92	174398.9	29965.96	21163.12	83753.52	33992.21	40529.78	28092.13	32349.52	108114
12695.88	176043.2	24433.36	20504.86	77759.67	35448.87	36873.35	30678.61	33270.66	107382
14704.6	179624.6	23655.22	17891.61	82992.35	32607.83	38275.36	28254.7	37329.72	63604.36
11240.81	183040.8	25397.84	17923.95	101390.3	36557.22	36909.47	27278.57	35259.66	124909.2
12941.72	192352.9	44221.22	17668.26	107652.5	34346.42	40053.13	33440.5	38863.43	77454.91

LN613.717	LN613.750	LN614.508	LN614.747	LN615.047	LN615.046	LN615.047	LN615.046	LN615.046	LN615.047
18984.1	19072.14	37758.27	37793.69	36414.01	36733.89	36777.19	29178.28	20563.73	31625.59
17458.77	16485.25	32938.41	32711.34	40868.68	38896.51	36713.01	32455.96	21513.87	32267.7
14070.76	15583.05	31278.89	29536.26	35261.78	41573.99	34936.86	33621.84	21932.39	34857.94
20898.22	19023.79	32943.48	36088.37	35977.47	38707.13	37713.25	32289.81	24165.9	35241.51
15608.02	25880.04	28858.73	50918.01	39757.41	39809.83	31218.37	28130.19	23614.17	32816.39

LN615.045	LN615.047	LN615.047	LN615.047	LN615.047	LN615.047	LN615.047	LN615.046	LN615.047	LN615.048
29091.96	42884.19	36992.39	37414.22	29580.96	31767.04	42819.47	48625.38	46189.36	38630.98
32444.09	40799.56	36656.59	35326.64	30871.98	32016.66	41268.17	49199.15	44488.91	39494.12
33172.4	41902.13	35608.32	38946.56	29549.07	32071.08	43487.38	45876.3	50806.64	34120.05
34016.69	43764.43	39095.92	42140.75	27116.37	30115.92	41992.8	48561.12	48046.18	39522.96
33360.43	42247.25	34169.56	40286.01	30629.91	33212.57	38073.42	45161.43	46512.99	41353.89

LN615.711	LN616.768	LN617.004	LN617.004	LN617.004	LN617.004	LN617.003	LN617.004	LN617.003	LN617.003
17756.76	27022.91	25570.84	33790.64	45538.65	43521.29	43159.86	45074.46	36052.72	40711.37
17931.37	21387.66	22493.3	36836.04	43660.45	45720.87	44864.25	48659.44	41329.47	40438.65
18003.42	28961.3	23110.6	40613.54	48080.65	43539.83	41402.05	45203.64	41666.27	47129.85
13448.92	30617.43	25174.51	34747.96	43245.22	44631.57	41715.19	45923.28	36498.46	47674.4
15651.53	36300.92	25255.41	36571.61	43203.8	41270.19	42818.56	47667.3	35543.17	42365.93

LN617.003	LN617.004	LN617.003	LN617.024	LN617.024	LN617.024	LN617.024	LN617.024	LN617.025	LN617.184
45149.11	37540.39	54404.34	32809.01	52479.81	43864.54	42138.18	52166.84	44717.82	53239.19
46272.3	40959.47	43168.62	30715.73	53755.52	44002.25	40999.57	46586.44	44195.34	57979.85
43037.14	32815.05	51806.53	31222.06	50470.49	49043.14	40269.92	51137.38	46057.01	50652.04
43240.46	40901.01	50845.15	32497.88	51100.12	45768.17	44745.04	50116.46	46579.8	49814.62
43064.1	32050.25	50151.68	31728.09	46776.78	50247.9	41605.01	48969.3	45106.89	55838.64

LN617.767	LN618.690	LN618.763	LN619.020	LN619.765	LN620.689	LN620.764	LN621.037	LN621.037	LN621.036
15870.75	36614.97	175780.2	71133.07	32314.46	25899.84	84759.55	25324.89	32247.3	22812.41
18730.43	33311.06	143614	72471.23	31051.06	26491.16	59119.26	26123.85	34273.15	25148.14
15944.51	38447.55	183265.7	74767.14	29216.58	29624.16	79271.17	23674.76	34251.39	24043.39
15004.98	46750.88	225150.4	78127.32	34057.55	33874.61	82249.63	27350.04	31287.83	24786.69
20987.81	63783.75	217760.8	72951.76	39791.05	39696.88	80210.95	29136.04	34221.4	24953.78

LN621.037	LN621.115	LN621.306	LN621.583	LN621.766	LN622.707	LN622.761	LN622.993	LN622.993	LN622.994
28783.09	29680.7	15473.17	16051.73	13292.66	84074.46	31549.63	29913.93	35153.12	30677.99
27913.92	34623.89	18020.91	17588.24	10306.76	74348.05	30320.06	33246.3	34250.44	31597.79
24813.68	38543.71	25210.11	15388.8	13431.23	78695.5	29010.98	29494.51	38069.58	32663.28
26139.1	33531.22	29905.42	12699.15	12759.88	101297.5	33632.26	29219.61	34674.29	34115.3
28121.77	30811.83	33520.15	16068.98	14891.49	124174.1	39725.25	29948.74	32694.29	33426.26

LN622.993	LN622.993	LN622.993	LN622.993	LN622.993	LN622.993	LN623.128	LN623.201	LN623.253	LN623.253
23584.74	26520.88	48455.05	42050.63	54632.57	28882.97	21261.21	32109.61	18708.78	21331.97
24074.87	30853.51	48909.26	47257.03	53648.63	33186.48	18504.96	36820.99	20047.93	21302.42
19663.51	28880.76	45601.39	38826.47	54925.59	29363.13	20219.64	33562.86	19311.94	22543.63
22226.48	25655.32	48229.57	42945.33	52785.04	28046.95	22414.41	25894.76	19414.37	22797.79
22909.8	25407.6	49223.99	40455.29	50821.31	28383.07	25240.53	39101.66	18538.79	21654.6

LN623.252	LN623.253	LN623.253	LN623.254	LN623.710	LN624.706	LN625.009	LN625.01_	LN625.010	LN626.547
28292.24	32225.75	21106.72	29822.84	12267.3	26736.61	26561.84	35463.71	23809.17	574531.7
26204.96	29510.36	18748.92	30040.57	11436.48	24274.7	26909.26	39255.31	24125.89	568089.4
26152.51	30475.42	18577.62	27761.31	15792.56	25068.74	24568.69	40678.44	24011.2	536736.6
27081.27	33060.45	21192.01	32179.69	17343.43	32588.98	25530.84	36142.72	25196.64	583685
25207.59	29865.12	17421.26	26969.41	22811.11	37511.28	33487.16	37858.6	19451.42	575737.5

LN626.723	LN627.006	LN627.006	LN627.006	LN627.006	LN627.008	LN627.026	LN627.046	LN627.047	LN627.047
126012.4	30352.48	22277.53	33236.25	35192.96	28366.09	19536.19	23771.24	33348.15	28351.01
104866.5	31256.74	23360.13	33764.46	34917.83	27770.56	16693.01	24394.4	32833.98	30342.36
110685.4	30319.39	21294.15	33182.28	35227.95	28755.29	20522.23	23584.52	31168.57	25104.39
127629.9	31730.78	20952.99	32922.26	32978.28	30105.51	18936.17	23024.63	32077.69	25633.29
173360.4	33386.31	22544.11	34720.68	35096.28	31135.34	18981.66	25227.43	35257.88	29447.56

LN627.047	LN627.047	LN627.106	LN627.106	LN627.106	LN627.106	LN627.106	LN627.106	LN627.551	LN627.560
28617.27	33377.61	31523.35	35365.38	30798.57	30567.97	32853.54	22050.46	233337.6	207644.6
29236.6	32196.16	37583	30828.89	31857.43	31275.74	34767.01	24198.18	226052.7	219946.2
29318.47	32529.25	29478.75	35329.18	36568.97	34040.99	34490.16	27066.1	228887.3	255339.4
28698.49	35322.39	33098.76	34940.37	33840.26	29442.4	35087.12	24674.43	212816.4	179906.3
28127.77	30149	30421.13	33015.47	33875.13	29027.09	34919.9	23273.82	145938.4	169502

LN628.554	LN628.563	LN628.719	LN629.024	LN629.023	LN629.023	LN629.042	LN629.042	LN629.043	LN629.557
40237.27	80534.57	555804.8	34186.67	41071.15	31591.93	33394.74	37026.78	37564.39	58880.96
43099.2	79608.32	412171.2	35486.15	44679.38	29496.32	33314.25	38914.53	40013.35	55655.17
42552.12	90991.64	477411.6	38106.21	43808	26204.76	32563.77	39016.1	42893.57	34227.18
38657.49	55016.8	579089.3	34791.88	43208.19	28699.73	33683.68	36946.28	36568.95	39619.56
35293.2	67044.96	758816.3	35594.9	40002.56	30345.02	30806.09	42085.2	36507	62719.12

LN630.495	LN630.561	LN630.719	LN631.021	LN631.020	LN631.021	LN631.021	LN631.020	LN631.020	LN631.040
48306.02	17406.29	310107.7	40117.52	40133.72	58064.92	28204.05	51144.39	38695.76	43342.3
56799.86	14209.19	261373.4	40067.7	37468.44	60363.98	29042.83	53644.65	42164.62	38444.7
61800.39	20907.15	290030.3	40157.67	41024.74	60247.51	32057	54294.68	37386.34	42393.89
63956.86	15528.18	352626.3	43800.2	39589.07	63550.61	28472.27	52919.17	39158.27	38757.88
59807.38	12385.4	445169.8	37174.72	41773.51	55430.87	30263.22	55026.37	40072.81	48684.03

LN631.040	LN631.040	LN631.040	LN631.040	LN631.040	LN631.040	LN631.334	LN631.499	LN631.507	LN632.332
34774.65	34411.04	36988.54	42657.89	31497.53	35290.34	17360.14	13574.2	462710.9	43668.47
31766.07	31131.33	36371.36	47517.94	33380.84	29806.62	19971.89	15562.39	469701.5	46237.79
33462.63	35038.2	38856.93	45349	33401.04	32914.8	30962.49	16301.06	335955.6	39033.22
35316.63	32040.93	35947.22	50162.68	36948.56	36725.56	37683.77	14704.29	367488.8	40408.58
32969.81	35086.99	34020.87	49139.1	31757.37	31555.11	40204.05	14385.93	370106.7	40902.99

LN632.510	LN632.717	LN633.157	LN633.504	LN633.619	LN634.507	LN634.623	LN634.716	LN634.994	LN634.994
112587.1	141339.2	45630.62	128945.6	67149.62	51235.17	24938.73	57319.31	22572.34	19417.72
101201	116571.2	44259.64	155843.6	63090.93	54793.31	28416	49373.11	21054.75	18140.08
104691.3	126317.6	43376.31	122418.8	63909.62	42662.28	21881.53	52573.73	22865.32	20593.05
108429.9	150966.6	36605.82	177331.2	44600.72	47245.3	21245.78	59614.27	22093.16	18221.03
206087.9	206538.2	31808.19	105058.6	62595.87	58310.59	23534.37	78152.9	18903.77	17136.52

LN635.131	LN635.131	LN635.291	LN635.716	LN636.277	LN636.714	LN637.129	LN637.129	LN637.129	LN637.128
18034	19071.93	23193.53	25813.14	29673.07	33594.55	33109.22	32311.04	40878.98	65029.01
22390.54	18269.41	19523.43	24831.92	31378.85	31411.03	34590.88	36123.52	44027.51	67238.01
21502.39	19173.7	22027.66	21487.86	30075.17	35434.66	35128.44	38246	42529.9	66916.57
20638.67	17206.17	19231.78	26902.73	27361.58	33690.62	37971.53	36572.04	45721.75	60940.32
18949.62	18502.23	20174.88	32442.6	23704.56	43682.09	30953.95	38226.16	40600.58	62610.32

LN637.129	LN637.128	LN637.130	LN637.541	LN637.589	LN638.592	LN638.709	LN639.027	LN639.026	LN639.026
50082.11	60568.86	48626.18	11625.22	588687.1	222004.3	17358.93	26611.28	27756.84	36235.81
49051.1	63192.95	54265.39	12981.3	581636.7	233811.3	15145.73	27914.65	26496.35	41884.31
49541.48	59879.84	47815.63	12789.71	397326.5	226723.8	18098.63	25360.99	23833.74	37504.68
45478.12	66611.26	49811.05	13395.45	434837.8	131146.9	18107.21	28506.43	22088.21	41819.57
50786.02	63236.87	55093.13	9252.926	461999.5	130395.2	24514.21	25979.23	21516.2	38120.83

LN639.028	LN639.027	LN639.595	LN641.536	LN642.539	LN643.059	LN643.543	LN644.711	LN645.056	LN645.056
26468.17	34003.9	56736.78	429851.1	158511.4	27579.27	33405.17	15853.1	40989.71	37165.14
25693.17	36114.58	54634.14	470216.9	166226.1	32385.3	40202.93	11145.24	39273.45	35497.44
28281.8	32275.11	30497.46	493300.6	158346.9	32666.76	23427.88	14539.55	36251.01	43501.2
27070.33	33421.47	49472.86	543212.6	109802.4	35520.99	26366.78	17408.3	35767.99	36245.86
23229.32	34234.15	58730.76	267233.7	163164.1	35592.1	36753.67	22561.81	40025.22	35969.9

LN645.056	LN645.056	LN645.056	LN645.057	LN645.056	LN645.709	LN646.712	LN646.994	LN647.129	LN647.711
33092.57	36569.72	33852.23	34521.63	31708.91	32769.96	14017.22	15028.61	15797.82	23502.1
31713.29	34371.72	36987.69	39062.47	33864.25	26054.42	11054.85	13668.43	17103.2	16897.13
36969.24	36632.82	32735.62	36873.47	38151.94	28471.69	12720.27	13747.77	16252.39	19143.52
35672.95	36871.42	38174.43	40634.09	37448.34	35207.05	12535.31	14511.36	21924.79	22650.39
37464.95	36054.7	36338.03	35806.95	36214.52	44219.83	16966.02	15488.34	22723.71	26918.03

LN648.325	LN649.010	LN649.010	LN649.010	LN649.010	LN649.010	LN649.011	LN649.514	LN650.517	LN651.028
7053.434	38929.97	36065.45	35572.61	25198.1	44264.35	37912.01	627196.3	226186.1	31862.49
8041.614	34066.77	39724.31	39053.25	26315.08	47360.64	37079.4	597494.4	214454.7	22375.4
12667.63	37525.64	40563.87	35128.54	32619.18	45646.57	29589.11	605561.9	217519.9	26121.85
15749.38	36872.8	34795.27	40044.33	29959.9	45429.84	31419.97	604650.4	136370.1	29076.33
17323.93	44332.55	38554.86	33162.72	32107.88	40920.47	35584.27	597866.8	229033.6	27809.09

LN651.029	LN651.029	LN651.029	LN651.028	LN651.488	LN651.520	LN652.404	LN654.579	LN654.679	LN655.159
25696.24	36756.33	47780.78	47216.42	24171.84	38446.6	24435.82	163893.7	44316.3	91535.64
31249.01	36116.21	41369.41	50000.39	24227.5	24043.78	27788.73	167999.3	33572.7	123986.3
25299.07	32770.02	42913.88	44130.88	24130.64	41150.46	25094.81	184632	41864.31	177995.2
27796	38568.13	46175.28	46260.12	21245.34	30893.97	20014.79	182595.3	47909.25	194992.2
24676.45	29652.63	46061.47	46768.54	19621.47	30198.18	22892.63	120096.8	52895.98	199550

LN655.582	LN655.678	LN656.678	LN656.714	LN657.075	LN657.075	LN657.075	LN657.227	LN657.505	LN658.239
60070.08	8468.225	40419.91	31770.48	25574.14	37798.69	31037.26	22221.68	47333.28	44099.31
68167.48	6242.978	38065.67	27866.64	29310.49	39093.76	29799.24	24514.75	46992.13	45112.35
66323.57	11477.06	25765.14	38948.17	26906.08	41531.39	31152.04	23662.12	49434.17	44338.44
45673.15	10239.93	34063.69	50534.83	28574.25	41537.5	31429.26	19143.48	41009.87	38314.7
38586.87	13605.35	58342.19	51630.08	27258.7	39677.5	33249.95	19491.78	40852.46	34556.46

LN658.421	LN658.526	LN658.676	LN659.074	LN659.074	LN659.074	LN659.074	LN659.074	LN659.074	LN659.074
28370.12	158882.5	15484.21	25323.62	39181.73	46578.76	40557.8	38657.97	40094.58	34818.53
25842.2	184948.2	14504.2	29353.13	34806.02	43601.15	41464.42	39778.24	42832.26	39480.45
26551.5	192846.1	15029.48	26982.14	32319.36	47647.45	38245.12	42149.72	42047.09	35458.98
22006.42	124663.2	18304.3	26863.52	34994.76	44649.06	41557.36	44115.64	42621.93	36702.05
19313.6	191984.4	21993.96	29966.63	33778.05	49882.62	43039.15	40482.2	44443.94	35699.28

LN659.530	LN659.538	LN659.957	LN660.542	LN660.709	LN661.068	LN661.069	LN661.069	LN661.069	LN661.069
67070.76	498980.6	17007.92	226819	15763.52	41178.7	84025.12	56430.28	33902.28	39725.03
73734.37	559368.9	21539.33	210505.3	21593.37	42220.47	84677.79	52426.6	35013.15	38095.52
46326.75	384188.5	20399.98	233900.1	17444.69	41543.58	83592.81	58584.72	34305.23	40189.94
41896.7	412331.1	15633.57	182435	18879.56	43086.87	81004.2	56355.62	35123.01	34613.23
75797.16	448170.5	13487.16	216832.6	20658.41	38858.89	77100.28	54739.56	35629.12	37533.36

LN661.068	LN661.069	LN661.536	LN662.539	LN663.413	LN663.631	LN664.309	LN665.004	LN665.004	LN665.004
30704.11	46297.55	135020.1	68919.52	17682.75	20546.4	46351.26	28506.05	31034.36	40853.44
31125.46	45950.81	183796.3	61819.69	21261.7	23531.96	48469.47	33657.67	28086.07	39353.79
28924.44	42961.47	214400.9	63669.14	19463.29	23402.95	47022.01	37454.23	33726.69	42865.94
28212.49	41549.05	154040.5	68718.78	15482.95	25069.98	31397.99	35872.09	33162.45	44144.32
31205.88	44236.99	136089.4	54162.05	15303.52	17271.12	39695.61	33861.41	33074.92	39689.7

LN665.003	LN665.042	LN665.043	LN665.044	LN665.044	LN665.609	LN666.997	LN666.999	LN666.999	LN667.000
36527.21	37255.87	37865.34	37743.51	27745.67	16451.7	50296.12	41251.65	36726.59	25867.88
33460.72	42393.6	39175.97	35837.15	32629.47	14624.6	48087.06	37777.55	36402.84	22760.34
31375.07	40905.85	35440.2	36155.92	35160.05	14532.47	50735.6	39175.03	33236.92	23928.12
31996.85	40976.67	38401.67	42167.45	31837.8	15631.86	45814.09	44079.42	37916.82	25604.21
34568.23	41227.56	35412.64	31501.09	33366.88	11152.64	51281.15	33696.3	34825.6	23815.64

LN666.999	LN667.000	LN666.999	LN667.460	LN667.652	LN667.704	LN668.268	LN668.376	LN668.710	LN669.017
38661.62	33797.81	33523.06	14706.83	25331.28	25236.51	19499.98	22488.47	13460.59	34143.77
35793.06	38677.36	30905.21	14647.89	22894.16	24205.99	23237.08	22675.28	11589.03	33112.43
37085.37	34843.62	33836.85	17060.03	19965.32	20480.33	21864.99	21757.83	13615.89	33935.42
39415.87	37849.01	34941.3	13486.63	25682.8	25534.35	16318.7	19417.79	17911.8	38427.78
35039.96	38531.72	34478.72	11549.14	36656.31	28662.31	16543.16	18933.19	20148.52	32291.75

LN669.017	LN669.017	LN669.017	LN669.017	LN669.018	LN669.018	LN669.018	LN669.018	LN669.018	LN669.018
39437.67	37254.99	36525.18	40529.96	40630.14	32048.64	33130.91	43902.89	29849.8	26010.67
37765.84	39420.42	39914.7	43098.14	39483.64	33488.3	31944.06	38079.73	35665	29420.05
39348.21	37489.16	43382.31	45735.22	46532.24	35215.16	32978.9	44332.55	39533.69	30377.16
43377.56	34518.68	39493.7	42892.89	41050.58	34588.3	32276.53	43496.28	37137.79	26655.58
37938.99	38835.59	37139.35	41199.14	39819.21	32868.83	31805.05	43504.31	39563.84	24297.23

LN669.430	LN669.567	LN669.708	LN670.571	LN670.707	LN671.268	LN671.574	LN671.709	LN672.271	LN672.707
26581.99	544505.7	17542.43	234132.4	56534.18	70553.98	48181.6	12025.56	25844.89	38145.54
29186.04	661620.6	14185.96	174493	54907.98	79702.03	48540.56	12469.48	27220.85	38193.42
30281.64	372130.1	14048.65	184022.8	54120.55	77142.16	37306.92	12345.66	24952.58	32656.5
19492.88	444168.9	23745.46	195532.1	73833.17	76136.13	33984.73	15202.76	23828.17	43088.81
22189.04	388434.9	22469.01	219782.7	86076.14	72617.64	53483.15	17189.37	24177.36	49868.96

LN673.265	LN673.526	LN673.527	LN673.711	LN674.705	LN675.666	LN675.703	LN676.670	LN677.545	LN678.308
24982.75	17025.11	13447.79	10310.57	20585.65	90981.72	20057.15	32356.53	603398.2	19707.97
27310.54	17241.23	15599.94	7864.054	15890.23	50546.38	22704.89	33224.81	569340.2	19387.99
22453.77	15979.47	12190.48	10432.2	18410.28	87162.22	18405.61	31337.1	569609.2	16272.26
21943.87	19121.62	17081.79	11830	17865.68	77185.29	17814.74	31766.15	357695.7	16887.63
21827.14	18196.62	16150.39	13318.96	22761.1	52262.2	18124.29	33779.63	651904.9	17257.42

LN678.548	LN679.021	LN679.021	LN679.021	LN679.022	LN679.020	LN679.385	LN679.551	LN679.705	LN680.734
250805	28251.16	25292.05	44844.19	30457.97	39488.21	19897.74	59752.67	14376.11	30680.98
233114.3	35197.54	25520.96	46505.35	28198.78	46397.94	21930.24	55492.48	9834.114	28926.05
239314.9	31003.27	26729.77	46230.97	26942.62	45241.16	21051.49	44869.39	12842.68	30374.03
238399.3	33385.24	25826.17	45494.89	36435.52	45500.66	15076.38	31853.21	15610.66	37245.46
257021	32890.69	25872.96	43667.86	29508.89	42936.96	16820.14	36940.66	18192.65	39756.68

LN681.016	LN681.016	LN681.016	LN681.016	LN681.016	LN681.016	LN681.296	LN682.735	LN683.034	LN683.034
37159.14	32491.5	35053.11	36931.68	48196.87	29411.78	896707.1	18014.96	30797.08	32196.01
44258.5	33814.76	31872.39	37635.37	48211.49	34165.41	920404.4	14074.03	38243.78	31093.69
37584	32322.47	38833.88	37664.47	43849.51	29827.32	928329.8	16530.92	29760.41	40672.68
35267.3	34339.94	41298.53	44293.35	49735.55	23299	920088.6	17181.21	32390.79	32869.5
38398.57	34089.8	38929.82	34638.12	44130.56	26848.97	855381.8	18326.17	27429.82	30618.55

LN683.034	LN683.035	LN683.035	LN683.035	LN683.035	LN683.035	LN683.035	LN684.305	LN685.172	LN685.258
39811.53	32255.36	40060.01	45743.1	40121.8	24374.82	29743.07	12138.75	14379.19	155827.7
34681.76	40271.51	40618.6	39381.2	40993.87	29771.16	33759.79	13512.6	17100.14	158256.6
38308.81	35229.14	39277.03	49210.28	40978.92	30132.55	31373.73	11613.92	12965.9	155441.4
40221.06	32480.67	35537.14	47594.73	36407.76	29316.24	28255.61	12898.04	13467.3	134287.7
38578.17	37498.25	36799.76	50900.94	45758.93	26048.27	34863.78	12329.77	9994.081	129183.9

LN686.269	LN686.558	LN686.678	LN686.751	LN687.005	LN687.006	LN687.005	LN687.005	LN687.006	LN687.006
154928.2	233050.9	17375.73	23180.27	39172.38	36752.78	26140.79	39970.27	29960.38	43413.9
149481.6	248736.1	10931.2	27250.39	38701.15	43551.52	30534.29	33151.11	24792.28	46405.91
166425.2	186983	15293.94	29087.31	40622.33	33966.18	31335.68	44454.74	24388.9	47764.16
155323.7	200200.2	18371.23	29176.49	41667.55	35500.41	29707.66	36800.4	28969.19	45458.74
139781.7	209064.1	25488.86	35581.53	30692.54	37640.94	32776.85	38674.78	29879.11	42848.63

LN687.005	LN687.561	LN688.677	LN688.753	LN689.682	LN691.189	LN692.340	LN693.343	LN694.711	LN695.032
27839.53	90317.25	15165.44	14260.58	12913.92	18477.45	59092.96	13903.91	21739.03	33209.52
22557.84	96360.91	10381.37	12214.85	9879.456	19554.13	62288.34	15567.91	20611.75	32473.36
28268.94	73597.99	11732.01	17086.06	10146.13	14570.37	58433.49	15792.8	25419.68	37679.53
27917.87	70237.03	17506.8	14564.62	12541.14	19625.85	30348.44	12004.04	26470.12	36767.77
27716.96	75324.39	18605.27	14947.92	12326.36	12642.36	50439.99	11820.63	36318.73	36900.29

LN695.033	LN695.034	LN695.034	LN695.034	LN695.504	LN696.298	LN696.506	LN696.706	LN697.301	LN697.599
37023.25	40261.3	43643.17	38486.85	38789.09	87569.61	12315.79	110692.7	25609.22	17138.23
30257.85	37574.15	42062.12	38273.15	27671	97343.64	11868.11	98300.63	24898.26	15187.41
29628.65	34962.81	36798.79	35485.36	34813.87	95665.66	10172.45	109942.3	23973.07	12987.3
27076.31	33136.53	38003.78	34718.9	37697.68	85592.27	13064.51	121801.5	17627.11	16509.17
31102.69	35488.91	39079.1	34554.42	38622.98	78274.13	14486.92	160017.1	17497.35	18079.99

LN698.287	LN698.706	LN699.290	LN699.322	LN700.649	LN700.703	LN701.024	LN701.024	LN701.024	LN701.023
79921.95	80196.32	28524.06	17492.75	58096.74	48282.41	42731.86	47401.87	35757.82	41837.39
89930.58	81885.21	29275.06	20509.79	54910.41	47382.82	30782.66	45277.57	37385.82	35157.76
85717.53	91390.9	26258.68	31940.21	58826.11	46717.51	30888.72	25856.76	33450.02	36551.95
79436.66	97618.46	30832.53	39840.1	63994.22	47221.37	30588.6	24586.46	42097.93	38298.44
77564.68	125318.8	27100.64	43823.73	89276.47	62059.53	30424.01	29863.49	37137.93	37543.84

LN701.023	LN701.023	LN701.024	LN701.024	LN701.023	LN701.023	LN701.024	LN701.024	LN701.024	LN701.556
57457.4	37982.62	30232.64	26449.36	27264.48	34813.61	39741.85	22325.36	28581.19	49762.94
44706.72	35828.15	37662.05	27244.84	36284.7	43123.02	36990.86	24453.45	25282.07	55246.33
50352.5	33667.94	34509.85	28777.19	29182.83	35579.82	31950.65	24760.52	22619.93	49150.11
49818.73	35042.84	39195.6	23413.8	31995.73	37988.29	37119.86	25573.57	26714.71	52865.28
54921.44	31326.64	35144.74	24279.46	30103.84	39861.34	36543.01	20736.13	29122.41	52067.67

LN702.650	LN702.702	LN705.576	LN705.676	LN706.339	LN706.580	LN707.583	LN707.656	LN709.524	LN713.288
21791.78	19592.65	165846.2	22948.11	47150.99	77243.99	10755.91	17028.11	21494.03	468064
20497.47	18223.73	166952.2	23786.54	45430.84	46510.98	7917.132	16610.69	23686.09	479031.7
21538.5	17954.94	179133.9	19388.76	33501.09	74364.13	10201.17	16502.28	17264.1	456036.8
25992.43	21512.98	123018	21098.65	40023.61	50561.48	11445.66	14731.88	19470.21	417712.3
30672.85	26051.13	187190.1	19511.22	36380.57	55904.74	11837.81	14338.6	21387.86	382344.2

LN713.649	LN714.301	LN714.655	LN715.040	LN715.041	LN715.041	LN715.041	LN715.040	LN715.040	LN715.040
40029.6	338567.9	85085.27	26899.18	38666.34	29142.08	33913.86	41469.37	38586.9	35517.79
47414.33	323371.5	98561.54	27469.69	39761.56	31877.14	32980.64	34644.53	38347.14	39495.54
48462.33	340650.2	90989.6	30543.42	40766.84	32586.02	33363.99	31072.26	32771.91	41728.26
41959.98	309915.5	49670.41	25205.92	44706.41	32194.8	39065.51	36783.62	39418.06	36793.69
42535.02	294323.6	79505.21	30379.76	48904.68	34255.17	35113.98	33844.32	31235.83	36828.73

LN715.040	LN715.040	LN715.041	LN715.040	LN715.041	LN715.040	LN715.041	LN715.661	LN716.665	LN717.713
22979.02	34647.02	34283.75	24559.14	33345.85	37723.38	36471.76	65988.53	15358.06	106365.8
16840.4	27666.34	35144.92	20137.94	27092.36	37858.64	35731.07	68961.08	20678.06	120030.3
16551.66	29746.71	38224.79	23142	27480.34	33993.44	32539.24	69706.6	19030.94	97202.36
16855.36	25427.23	33867.65	23782.44	36664.26	36250.46	36858.49	46701.28	18875.41	105052.8
22090.15	32766.87	32220.41	23318.05	28925.65	38481.15	34943.92	58728.52	15077.5	104483.2

LN718.717	LN719.012	LN719.012	LN719.013	LN719.014	LN719.013	LN719.013	LN719.149	LN719.476	LN720.372
45007.59	33914.45	26426.8	31146.8	42469.28	23396.87	26003.28	20147.29	23560.54	48856.18
46616.53	37102.36	26209.1	34295.41	39686.27	23775.91	25869.18	25946.81	26961.05	44950.14
46974.77	35718.5	24206.56	28880.67	38429.04	22747.54	26070.02	43196.06	22053.79	36975.68
33367.39	33440.33	25366.64	33256.4	43425.74	24202.91	23942.24	44918.07	20910.38	34273.39
43162.27	36554.07	30981.3	29025.6	38173.35	24482.72	31672.48	47967.98	20169.8	30109.1

LN722.664	LN723.147	LN723.331	LN723.535	LN724.330	LN724.538	LN724.662	LN724.703	LN725.493	LN727.944
11643.86	17310.29	19293.32	48817.66	199998.1	19472.95	16826.1	17970.55	34285.04	27079.44
9503.235	26195.58	22395.4	45265.96	206076.8	22351.73	14755.12	14555.32	32251.8	29144.35
11350.82	35659.89	11541.67	47344.22	201237.8	22157.57	15189.65	14100.72	26113.79	25109.94
12416.21	40320.68	15069.12	39792.7	171333.2	25639.55	18542.39	18294.7	28195.88	24522.73
11056.25	41091.53	12886.81	49530.13	164925.3	28569.6	21058.39	21193.64	25795.4	18726.96

LN729.178	LN729.678	LN730.683	LN731.400	LN731.689	LN731.729	LN733.030	LN733.029	LN733.030	LN733.029
133642.7	18671.42	36014.97	13437.4	28266.52	11823.7	39127.21	27827.79	32645.39	28511.02
185241.4	19037.43	35811.41	12595.65	24039.51	13657.47	32350.9	28945.53	33439.18	30961.75
266947.9	16352.25	43586.44	16526.62	33983.65	13424.3	42161.07	26387.65	34080.79	33169.96
301589.7	15505.5	37739.29	13219.8	26279.73	10303.73	39917.11	29832.13	29985.96	29738.08
304591.5	18530.96	37087.47	13209.01	27947.55	11377.4	34304.21	25589.99	31370.7	31590.75

LN733.031	LN733.030	LN733.030	LN734.371	LN735.375	LN735.448	LN737.418	LN737.556	LN738.695	LN741.320
32231.38	43580.31	46723.33	71639.96	27038.85	21528.55	15468.8	16655.92	18297.94	914331.7
32605.88	37568.63	44479.42	70533.97	26695.42	21538.65	20008.47	18897.27	18537.19	887532.4
35606.85	42504.82	48946.04	69216.38	20950.07	18774.1	18744.92	25234.38	18426.53	878007
38346.61	39220.96	51744.85	64448.23	18104.25	16639.9	13657.16	21534.11	23225.26	813755.3
35212.72	41700.01	50881.37	57768.46	21125.98	15382.62	15070.32	20217.99	23864.43	758506.2

LN742.332	LN742.699	LN743.336	LN743.692	LN744.330	LN744.698	LN745.333	LN745.704	LN745.780	LN745.780
460482.6	8371.315	170329.9	226774.3	186679.5	1988744	56609.69	11136099	83225.43	32615.4
481258.2	5392.067	163024	239878.1	189646.6	2093445	59287.89	11253451	77108.66	40591.38
490543.2	9138.589	165691.3	195489	129321.6	2381275	55962.76	11781534	80207.78	42755.07
446489.2	12760.72	103144.2	194775.2	179534.3	1550629	34186.91	8624469	77441.91	39647.9
287000.9	14159.76	103980.3	257222.6	169882.5	2215843	35024.8	12309617	74483.41	33747.84

LN746.710	LN747.374	LN747.715	LN747.724	LN748.403	LN748.651	LN748.719	LN748.724	LN749.284	LN749.704
19235435	16984.27	15150255	29427.37	38009.43	33583.22	5425642	31845.12	39011.86	23834.35
19235435	14948.01	15150255	27911.74	41594.39	41900.93	4926097	39795.51	38554.77	20059.69
19235435	10451.31	15150255	30091.27	29262.06	45003.33	5641608	42498.24	41516.53	21850.78
19235435	12167.46	15150255	27095.47	45093.31	44902.47	5759407	41072.85	46082.04	22311.25
19235435	13295.57	15150255	27494.64	29781.47	54708.33	5818306	50566.52	36427.17	21375.05

LN749.722	LN750.288	LN750.651	LN750.725	LN751.020	LN751.022	LN751.362	LN751.728	LN752.361	LN752.648
1204624	14307.21	18110.64	198481.5	38592.38	27966.28	26987.54	19656.95	361688.1	11221.65
1142841	14759.4	14523.43	195688.5	33686.5	34212.79	24430.41	22177.4	364238.3	12405.14
1183818	15928.46	18478.26	221034.7	37038.51	26471.87	22543.64	20212.82	376173.1	15369.19
923773.2	13009.36	33355.45	233720.4	45953.7	31546.31	20020.92	17383.04	329433	14271.25
1144825	12896.14	42018.75	149241.2	35917.14	32531.21	20718.1	16742.89	303430	23614.49

LN753.364	LN754.364	LN759.760	LN759.796	LN760.764	LN762.402	LN764.285	LN764.695	LN765.697	LN766.694
111576.4	26723.74	89133.77	25661.14	38919.4	88572.01	57793.06	60638.05	10387.86	45148.18
120014.2	28614.87	89749.46	25304.84	52674.05	89233.06	61355.16	51025.14	9876.2	37849.71
80866.73	27049.1	96863.36	27071.64	50328.34	91854.14	57041.23	54602.25	13193.09	45499.32
111685.9	16697.64	105273.4	25916.87	50013.7	53610.04	55415.7	68708.98	16947.93	53621.27
73070.91	24884.01	101109.7	24261.4	47507.62	82546.84	50462.02	80469.52	16850.08	58490.71

LN768.639	LN768.692	LN769.351	LN770.355	LN770.364	LN771.367	LN772.355	LN773.654	LN773.777	LN773.811
20976.98	20610.75	1234772	425026.1	527800.7	193785	18027.17	37758.42	13644.03	44182.33
19924.01	20004.41	1190048	414680.7	541157.9	200221.3	19606.16	29542.2	16099.99	48255.67
24004.99	18250.75	1114719	406170.9	545388.6	201411.3	16275.63	38447.5	19515.33	47212.54
34928.98	22648.25	699166.1	374870	548805	187223.6	13428.71	42644.08	18580.85	49207.17
40146.78	27017.22	1006832	356023.2	491213.3	118942.1	14752.86	56167.68	15672.39	47609.1

LN774.650	LN776.435	LN776.650	LN777.762	LN778.648	LN778.768	LN779.394	LN779.773	LN780.393	LN780.645
192541.1	29341.26	147164.8	49844.4	77437.29	81193.09	32883.64	68179.63	501577.8	44484.7
116441.8	35108.13	113939.4	48808.86	64836.44	88259.65	25050.7	66220.66	539140.6	35527.26
157166.7	25410.51	134247	55159.4	69365.55	95915.01	19461.17	71225.32	528239.1	40553.09
132337.8	25739.41	144951.4	40102.28	79576.75	54902.05	25243.97	66826.6	498879.4	44629.75
137725.2	26634.96	215778.7	47938.78	118295.2	89950.7	20968.79	43997.14	458541.5	59366.63

LN780.777	LN781.396	LN782.396	LN782.644	LN787.463	LN787.827	LN787.827	LN787.826	LN787.827	LN787.828
21108.58	182001.8	44905.1	8795.597	15835.8	32654.74	30974.48	39163.23	141809.1	18575.3
19854.83	190423.9	36794.28	12611.99	18535.69	33399.94	32200.59	45927.33	127251.2	21244.44
23057.19	201493.9	48997.04	13008.91	14900.84	38597.54	32818.6	43503.86	139173.8	20352.93
15399.99	186630.1	29658.21	11135.76	12771.55	35434.14	28851.54	42047.11	94641.88	15875.57
14193.28	166310.5	37503.44	12033.25	11617.94	40920.42	29686.66	38510.65	172766.3	18798.64

LN789.771	LN790.434	LN790.776	LN791.437	LN791.751	LN792.317	LN792.689	LN793.169	LN793.480	LN797.166
38814.03	92727.12	20673.91	32416.65	28337.66	124261.7	8635.098	18526.88	16777.89	28562.92
42947.22	89869.28	20977.12	31886.09	28102.33	124600.3	10878.87	23699.85	21097.25	43880.33
42896.79	90895.13	20632.11	35047.47	24827.77	118612.3	13998.92	37892.27	16389.68	58619.98
39163.72	92894.95	16023.8	22110.42	25077.96	115163.6	13348.91	43855.04	16173.19	67094.7
33220.87	85116.07	19191.79	18670.73	24456.37	106861.6	16647.05	46448.84	14230.97	66326.17

LN797.383	LN797.516	LN798.386	LN798.395	LN799.387	LN799.398	LN800.386	LN800.393	LN800.609	LN801.807
1179716	23164.25	420814.5	495454.4	116522.9	202403.5	19807.62	205496.2	17852.55	110509.1
1215106	19725.26	420129.7	522530.7	125515.1	214302.6	19475.89	201681.3	13842.46	92339.45
1123563	22241.1	434848.6	498053.7	116958.9	216350.2	26280.32	202952.7	15027.19	116429
1100561	21036.86	416457.4	474738.7	110011.3	197244.1	17687.88	182421.6	19430.51	102050.7
1002458	20798.56	391535.5	467579.1	101481.5	196677.3	15261.96	181537.2	22180.89	94405.73

LN801.842	LN801.842	LN802.656	LN802.810	LN803.197	LN803.663	LN803.786	LN804.466	LN804.578	LN804.668
58518.93	30033.11	56411.74	52742.3	129986	302362	18949.52	21283.9	37796.99	599018.6
64786.65	33482.76	50511.03	59159.4	172282.6	207669.1	14351.34	17262.91	31033.34	610935.5
59945.41	32513.33	36502.04	53713.54	248768.1	209124.4	19512.69	15220.18	30167.85	454585.9
66360.81	34902.23	47942.94	56176.4	276086.2	229358.7	16177.07	17175.76	34622.76	734474.9
63713	30486.39	30849.85	58524.9	281771.7	242204.4	9924.873	13397.9	33847.32	470884.5

LN805.675	LN806.670	LN807.424	LN807.670	LN808.424	LN808.704	LN809.707	LN810.276	LN810.428	LN810.712
464988.5	230481.2	26989.53	157988.7	655315.4	80574.1	187147.1	24076.9	65894.73	106917.2
440358.6	163303.3	22825	145466.6	692432.9	61125.71	194371.7	23129.17	72755.13	149357.8
492658.9	209400	19067.72	123453.6	679896.5	76545.33	204824.6	19725.14	42679.36	166855.1
391140	194568.3	18760.03	116368.8	622984.2	61133.51	164862.4	24379.62	65902.63	121230.5
331291.5	196696.4	20348.77	121188.4	618438.7	49676.37	207449.3	25047.25	39972.28	127236

LN811.679	LN811.715	LN812.685	LN813.691	LN814.697	LN814.698	LN815.703	LN815.823	LN815.859	LN816.638
39141.28	29316.81	368868.9	1982382	3931435	16139.19	2579802	10075.18	63539.5	29503.92
41153.52	43775.89	369664.4	2150209	3733484	9376.581	2831483	11303.81	58479.6	26266.16
36641.93	35915.67	379973.4	2299649	4044229	11914.36	1997757	12442.93	64020.14	28729.44
38451.46	42238.53	422889.2	1833777	3305389	12419.91	3196295	11435.65	63098.35	35335.72
34492.16	40187.85	269112.1	1831073	2651746	12301.58	3685146	9522.338	60161.66	39159.95

LN816.706	LN817.709	LN818.465	LN818.488	LN818.639	LN818.713	LN819.469	LN820.188	LN820.349	LN821.498
1024549	244763.5	78176.18	177270.1	20136.75	30957.84	30556.37	14638.22	178991.8	22866.53
1005723	239847	84553.11	187167.2	18004.16	29013.99	30360.23	21114.87	187337.2	23371.77
1111214	267033.2	78921.48	175011.2	20499.56	26387.91	31152.3	27165.36	188574.7	19340.1
870827.9	211778	49728.13	179485.6	24113.07	36678.3	31189.29	32996.19	178115.5	23864.04
1106740	195264.9	47933.54	180895.4	34873.64	26385.43	18293.35	33910.09	111315.9	24059.76

LN825.414	LN826.426	LN826.666	LN827.419	LN827.430	LN828.418	LN828.424	LN828.517	LN829.427	LN829.670
1074009	412727.8	18507.5	101082.1	167838.4	17998.62	157707	363721.8	55985.66	30274.51
1068699	440657.5	15643.23	113918.6	167423.2	19091.41	162625	367117.5	52298.86	38608.99
1002053	435390.6	13262.25	104306.9	161656.9	17677.29	169527.4	338128.2	38815.82	31850.58
941523.2	307750.7	14770.18	98457.83	152632.5	17078.19	103759.6	350857.6	50297.8	34180.28
897584.3	304928.8	26369.21	65242.92	145669.7	17458.86	152278.4	340309	32145.33	35314.5

LN829.874	LN830.678	LN830.878	LN831.687	LN831.819	LN832.694	LN832.822	LN833.697	LN834.801	LN836.455
134841.4	159727.2	73110.88	257385.8	35011.22	204421.8	13883.57	53722.06	12425.02	666606.5
178389.6	157170.2	62990.71	253791.2	34391.95	198350.4	14363.56	56260.12	9452.936	694918.4
142877.3	119593.4	68000.42	210803.9	30609.11	137299.1	16647.86	55026.99	9134.273	677210.4
139276.3	102945.3	82504.61	177239.3	35327.2	152451.7	17564.7	61018.72	13889.09	619164.2
136315.2	157918.4	67886.64	273422.6	32172.95	188612.9	13230.17	49239.04	7984.53	601697.6

LN837.458	LN838.308	LN838.46_	LN839.310	LN842.638	LN842.698	LN843.704	LN843.854	LN843.890	LN843.890
266778.6	55834.29	72669.57	23776.26	61260.11	36512.24	21525.69	77114.34	29317.04	76256.21
279717.4	59292.97	76523.43	23471.13	56400.76	37437.03	22486.49	70623.47	33265.85	74411.72
275294.3	62261.84	73287.66	20875.28	59647.93	30210.92	22290.1	80018.88	29046.5	67644.58
262679.6	63937.71	72855.25	21800.55	70719.67	34444.53	22543.72	81205.97	33382.39	79174.17
152517.5	43855.09	45135.12	17742.47	96188.34	39656.21	22166.35	76340.26	33369.84	74642.24

LN844.638	LN844.676	LN844.858	LN844.894	LN845.507	LN845.683	LN846.496	LN846.636	LN846.756	LN847.501
50020.59	76063.39	41684.15	22175.05	162342	64635.25	38355.4	30138.89	10984.49	16559.02
48846.63	75770.24	40622.35	22549.32	165057.8	59516.65	38566.19	28542.59	11749.42	19704.76
53970.52	65803.84	40856.78	22917.83	148331	59506.53	40383.2	34638.03	11817.17	17330.19
59849.87	72393.72	43731.09	25574.51	147655.5	60062.24	40399.4	42484.03	14193.86	14857.98
84076.16	66546.48	42251.07	20656.97	146972.3	54376.09	55898.01	46194.58	16102.55	14205.63

LN848.380	LN849.383	LN850.383	LN853.445	LN854.458	LN855.450	LN855.461	LN856.456	LN857.459	LN857.718
235698	90104.67	19623.23	763487.2	276243.7	83955.74	121439.6	120628.9	34645.33	16677.69
226456.7	89542.58	19798.5	766966.8	287402.4	89163.8	126934.6	122469	41842.68	19789.75
235962.1	91830.39	13706.3	763501.6	301417.2	64035.26	86684.99	80454.52	34296.88	19940.66
223179.2	86556.25	17860.31	522195.5	199386.5	53347.91	82984.04	107697.3	34265.38	25176.92
137271.7	79048.68	12533.29	460917.7	290113.4	49105.12	118258.1	119833.7	33172.63	27865.27

LN857.906	LN858.422	LN858.724	LN858.909	LN859.691	LN860.697	LN861.702	LN862.701	LN864.487	LN865.490
68141.98	15369.24	44483.13	31288.4	88128.07	185903.6	142856.7	51014.64	599819.4	261565.8
68127.47	12296.85	39412.16	30865.83	53816.02	172751.7	88927.03	50467.45	612334.9	270606.9
66599.46	15466.86	47446.01	34045.65	92524.14	177121.4	111288.7	44285.61	345909.5	257822.1
69066.18	12482.98	50175.25	36249.98	90053.35	194010.6	163365.1	49944.02	565586.9	163851.7
70520.03	11790.34	48650.21	39656.24	74434.96	143640.1	127019.4	45245.78	533565.5	228862.7

LN866.339	LN866.491	LN866.663	LN867.187	LN867.341	LN871.185	LN871.650	LN871.921	LN872.656	LN872.925
88311.59	69840.2	38963.39	18109.54	42603.3	30000.78	78395.98	75191.41	121830.8	50317.09
90790.28	73649.2	33182.86	21211.15	43926.53	39939.47	92024.36	89570.86	140239.8	43208.59
90865.26	73136.12	38922.56	33497.67	44201.39	53178.39	76533.06	87416.92	162382.6	53881.73
93239.35	49321	38372.83	40495.42	47034.07	66883.05	70798.66	92060.7	223944.7	59205.24
94486.77	59859.33	33737.85	43370.63	32635.42	66523.98	64249.72	92580.7	146035.6	58444

LN873.662	LN874.528	LN874.657	LN875.661	LN875.845	LN876.411	LN876.667	LN877.216	LN877.415	LN877.673
162316.5	30307.92	114690.1	235289.3	32243.96	242990.3	336939	118281.3	98650.67	268858.4
129718.9	33960.39	92053.17	238697.6	35190.84	254374.8	335016.4	160403.1	102587.4	271746.2
125273.3	24353.47	121957.6	254622.6	32305.69	254029.1	372559.2	224351.7	102413.9	309200.9
136949.7	17590.44	106087.9	209448.1	34134.77	168969	294831.5	260461.3	66688.27	309458
127702.6	25537.08	82047.68	206275.9	34849.51	159672.2	383665.5	264244.1	86376.74	238109.7

LN878.416	LN878.678	LN880.672	LN881.477	LN881.679	LN882.480	LN882.489	LN882.684	LN883.482	LN883.492
23082.64	63553.36	91589.87	508996.6	688931.9	216888.7	159107.1	1286283	67226.24	72850.3
26470.17	68049.72	134520.1	541182.9	474430.3	217601.9	162477.7	1287749	47715.06	83209.89
17555.19	105731.4	102381	538658.6	532325.2	140686	171088.2	1415224	59945.23	69375.94
21303.75	84234.19	90910.62	363794.3	580433.5	202906.9	107109.7	1211813	52104.71	50071.44
19772.57	96117.53	105271.3	343593.6	790961.7	203905	120676	1066885	48008.77	40407.63

LN883.690	LN884.488	LN884.693	LN885.490	LN885.696	LN885.901	LN885.937	LN885.937	LN886.905	LN886.941
1068076	64230.06	273203.3	21029.06	64499.99	69307.69	25682.63	46798.52	30908.54	19382.76
1052485	70977.53	364117.1	17091.91	85579.79	45228.61	25809.57	41488.08	29669.86	15131.19
1234846	64686.59	333895.1	16176.66	70872.97	45954.93	26085.39	46229.12	31985.55	17973.01
842978.6	62922.14	353220	14120.94	80345.58	50056.54	30341.77	46146.79	31604.75	16649.92
891861.3	45487.08	306461.1	12910.93	60976.22	45642.2	30527.81	50031.25	31613.63	19092.63

LN886.942	LN891.635	LN892.518	LN892.640	LN893.644	LN894.370	LN894.523	LN894.645	LN895.373	LN895.645
27210.45	61397.86	452018.8	138104.1	123798.6	124113	61754.83	54440.05	58002.35	23079.36
24016.83	73909.06	470142.2	137061.4	120252	128967.2	64262.3	48926.78	58212.58	27269.37
26056.95	96453.62	442368.1	150409.7	130476.6	145017.8	39186.87	58625.35	55539.49	27131.97
25529.71	93108.61	407879	122878.3	92591.21	109687.7	41231.72	51289.83	62178.74	22371.36
27119.25	58254.62	392406.3	115535.6	106189.5	101950	36825.23	49735.72	61716.95	24100.4

LN896.373	LN896.504	LN898.663	LN899.674	LN900.679	LN904.443	LN905.446	LN906.447	LN909.508	LN910.511
16326.08	159559.3	54617.72	55159.41	38737.31	234798.4	104828.5	24249.47	290025.3	130199.2
13923.05	169080.3	49305.63	75119.14	35935.52	233326.6	105299.8	21186.25	289804.5	135168
13536.5	159825.4	49197.09	46986.14	60876.89	218838.3	105508	14334.05	200775.1	73932.05
16474.44	151439	32824.49	83678.51	35455.12	154661.5	74178.53	19481.91	175318.5	73974.24
11894.56	152732.2	43553.44	44746.74	37636.86	140199.9	70567.9	19739.72	264851.5	71238.2

LN910.520	LN911.513	LN911.524	LN913.968	LN914.972	LN920.549	LN921.553	LN922.401	LN922.553	LN924.405
78345.24	35862.8	39419.6	84633.89	40592.05	290178.1	132810.8	112352.2	34346.2	14629.29
82120.53	31070.85	36233	63241.38	45558.21	295395.3	136936	118874.1	39569.28	16028.19
82374.96	20845.86	32762.58	46618.72	46632.95	308978.3	130598.2	129900	41323.86	15173.71
55895.81	28301.26	25338.68	70771.61	46765.13	192542.2	71693.73	86580.64	26481.25	13120.36
82350.81	24139.43	25919.36	74356.76	53577.52	188758.4	122507.2	93568.52	31244.36	15403.39

LN927.673	LN927.985	LN927.985	LN928.676	LN929.360	LN929.683	LN930.363	LN930.684	LN932.474	LN934.478
31383.45	13098	32159.1	86050.93	39675.54	43467.04	25151.25	28203.56	180841.9	17125.47
34295.32	12592.43	31261.37	64337.49	35851.03	68816.83	29824.69	32568.29	198858.2	16548.77
33662.01	14315.92	29510.26	54481.16	37263.01	65082.16	23438.84	28859.9	139230.6	13905.62
34265.16	13108.53	28385.85	67580.46	36248.95	77114.63	23922.14	34212.01	125495.4	12441.42
39838.43	15519.6	31978.27	62478.17	36736.57	50300.1	21068.45	28053.5	117714.1	14643.15

LN937.54_	LN938.543	LN938.552	LN939.638	LN940.644	LN941.206	LN941.648	LN942.000	LN942.645	LN943.649
129863.3	55530.31	30447.33	34367.04	70683.55	17517.15	81402.51	27188.51	41729.74	151732
128340.9	64924.34	27941.65	36293.43	71464.41	23021.37	46002.04	25876.65	37551.65	148468
127062.3	58733.3	27387.65	29244.33	79551.7	33126.36	64490.74	23154.57	38975.48	162184.3
79818.56	33961.6	20219.29	41724.34	86153.84	42498.64	51199.49	24641.97	50176.23	130836.4
73712.61	42520.75	27437.65	41166.48	72066.28	43885.68	77874.64	27821.53	45894.34	163992

LN944.655	LN945.203	LN945.660	LN946.664	LN948.580	LN948.660	LN949.665	LN950.432	LN950.672	LN951.235
230698.1	29580.88	180507.2	61385.61	181338.8	29115.96	240552	96164.07	412758.3	88795.19
234631.4	43865.23	123610	41037.39	184086.3	32796.34	241605.8	101915.3	421528.6	117205.6
200657	62123.56	161229.3	41984.14	171796.8	27245.04	197461.2	60153.03	287806.1	170401.6
223188.4	67822.16	175215.8	56793.09	153429	25574.21	225199.9	67058.18	326238.8	195820.8
201737.5	70206.65	143647.9	52063.76	150058	33457.48	197231.4	93854.63	452104.5	203533.3

LN951.435	LN951.677	LN952.682	LN956.015	LN959.623	LN960.506	LN960.629	LN961.509	LN961.633	LN962.635
50330.94	364600.3	72242.87	54366.11	34803.9	124439.1	57743.62	57631.95	67865.77	26997.14
49400.33	361576.6	93295.74	48063.72	36109.38	120925.7	87120.08	57599.08	62409.85	28091.19
36849.27	300601.2	102518.3	55182.21	39883.53	125321.8	67497.66	34850.3	77795.95	29976.39
54410.57	275192.2	100929.5	55271.33	36764.69	80055.43	64626.83	35466.43	62850.98	27575.96
41608.89	297033.1	81524.12	59036.23	42418.2	85148.33	80334.4	46093.73	66807.16	24187.27

LN965.571	LN966.574	LN966.652	LN967.660	LN969.995	LN970.999	LN976.612	LN978.464	LN979.467	LN988.537
48871.04	22612.87	17218.26	32412.27	36268.23	28988.38	104410.5	63301.58	33374.3	70100.85
47847.87	21453.25	15698.92	36726.9	36195.56	28994.66	107196.4	69355.25	26242.04	62594.6
44643.86	19902.33	21109.48	34813.61	42962	30485.3	90456.29	54678.24	33736.82	67019.24
36573.06	10105.88	13563	33282.35	45606.85	37601.23	86549.72	72060.15	34184.79	49435.56
32895.61	10577.24	14886.79	30940.24	44001.83	31685.32	83700.73	55305.47	30723.1	43784.85

LN989.540	LN998.026	LN998.063	LN999.030	LN1000.00	LN1004.64	LN1005.62	LN1006.49	LN1006.62	LN1007.62
31032.45	25953.03	25609.84	16857.3	24552.4	49916.34	28789.12	26793.39	26487.21	16980.6
29778.01	31277.02	30592.1	20667.01	23366.84	53871.19	29853.94	29475.19	22684.06	17580.75
19932.63	25398.44	25541.36	17823.59	26357.87	46858.65	31813.98	30090.92	23015.76	13224.26
23821.43	28059.19	28178.79	22404.47	25424.99	48360.26	28872.42	31734.7	25713.11	13494
20937.73	30514.77	27905.78	26871.39	24663.04	40605.4	27837.23	32786.16	22072.68	11536.89

LN1008.63	LN1011.63	LN1012.04	LN1012.64	LN1013.04	LN1013.64	LN1015.22	LN1016.56	LN1017.65	LN1018.65
25598.05	35440.25	36705.17	71570.87	22419.64	96196.55	18184.35	26538.26	39456.79	102560.5
25656.75	35169.42	38992.71	74810.98	30107.07	60104.15	21556.19	27188.1	51007.84	87331.7
29105.73	54742.31	37968.92	89323.98	29603.36	62071.19	33217.01	22626.55	39334.09	89512.74
26166.86	45978.16	40041.73	86403.43	27664.1	55371.47	41436.1	19311.14	36663.75	91266.91
27673.15	47044.15	38596.77	101452.4	25225.86	68209.57	45579.33	15314.33	54016.43	90557.09

LN1019.22	LN1019.66	LN1020.66	LN1025.25	LN1028.03	LN1028.61	LN1029.62	LN1032.67	LN1040.07	LN1041.08
27114.5	73817.69	21860.32	135197.1	11570.42	29696.8	25126.79	25755.85	23202.92	10563.23
37634.03	79772.28	22635.03	182799.7	10983.78	28310.48	20415.32	26139.1	22776.83	8850.73
56635.08	78335.37	22786.6	264635.6	13818.35	27957.42	23988.24	21768.21	20941.43	14722.73
67046.97	81130.96	24812.9	306709.1	14580.82	27589.69	21181.06	21577.01	23261.01	13126.54
65678.05	74718.91	20729.37	316830	10778.17	25685.65	20075.48	17310.11	26526.25	15280.41

LN1042.05	LN1043.05	LN1054.09	LN1055.09	LN1070.08	LN1080.63	LN1081.63	LN1082.12	LN1084.1_	LN1086.64
23642.57	20968	28021.52	18522.54	12211.9	31037.64	21888.79	13901.8	13349.9	20269.94
19945.16	20282.61	30775.1	18759.25	13684.24	29628.36	25612.84	11360.95	10533.29	23133.72
18217.24	18671.85	30530.62	16250.9	13282.33	26303.51	25727.36	13979.77	17769.6	27076.9
20601.97	20388.94	33267.49	22602.1	11594.92	29039.81	25818.53	13227.88	12907.12	23276.31
26129.26	25309.33	33520.71	22290.27	13146.13	24656.03	22636.19	16787.27	15559.35	19906.49

LN1087.65	LN1093.24	LN1096.13	LN1097.14	LN1099.27	LN1167.26	LN1173.29	LN1247.31	LN1493.42	LN1515.40
19617.89	50523.9	18467.23	12687.92	45653.07	18824.22	27967.93	19047.22	99709.83	65889.57
23092.27	71110.83	13377.46	16805.89	60843.83	23079.86	34925.43	22373.18	102141.8	58154.79
25917.3	100102.8	21100.76	16116.94	85553.94	37727.95	55414.99	36326.63	122110.3	82432.42
23313.4	117371	17316.63	14518.69	102271.9	43602.12	66441.86	43427.34	127922.3	79563.5
34693.19	122837.8	20850.27	17199.66	108287.2	47203.61	70414.56	47769.7	126139.5	84863.28

LN1583.39	LN1584.39	LN1586.4053_6.59
16524.08	34581.07	15202.35
15815.59	33571.39	17388.5
18322.82	43325.49	16142.36
22887.05	38952.72	21492.18
23683.57	41694.29	23085.28
